# Epigenomic integrative analysis pinpoint master regulator transcription factors associated with tumorigenesis in squamous cell carcinoma of oral tongue

**DOI:** 10.1590/1678-4685-GMB-2022-0358

**Published:** 2023-06-19

**Authors:** Larissa Miyuki Okano, Lívia Maria Maciel da Fonseca, Isabela Dias Erthal, Tathiane Maistro Malta

**Affiliations:** 1 Universidade de São Paulo, Faculdade de Medicina de Ribeirão Preto, Ribeirão Preto, SP, Brazil.; 2 Universidade de São Paulo, Faculdade de Ciências Farmacêuticas de Ribeirão Preto, Ribeirão Preto, SP, Brazil.

**Keywords:** Squamous cell carcinoma of oral tongue, head and neck cancer, master regulator, epigenetic

## Abstract

Head and Neck Cancer (HNC) is a heterogeneous group of cancers, which includes cancers arising in the oral cavity, nasopharynx, oropharynx, hypopharynx, and larynx. Epidemiological studies have revealed that several factors such as tobacco and alcohol use, exposure to environmental pollutants, viral infection, and genetic factors are risk factors for developing HNC. The squamous cell carcinoma of oral tongue (SCCOT), which is significantly more aggressive than the other forms of oral squamous cell carcinoma, presents a propensity for rapid local invasion and spread, and a high recurrence rate. Dysregulation in the epigenetic machinery of cancer cells might help uncover the mechanisms of SCOOT tumorigenesis. Here, we used DNA methylation changes to identify cancer-specific enhancers that were enriched for specific transcription factor binding sites (TFBS), and potential master regulator transcription factors (MRTF) associated with SCCOT. We identified the activation of MRTFs associated with increased invasiveness, metastasis, epithelial-to-mesenchymal transition, poor prognosis, and stemness. On the other hand, we found the downregulation of MRTFs associated with tumor suppression. The identified MRTFs should be further investigated to clarify their role in oral cancer tumorigenesis and for their potential use as biological markers.

Head and Neck Cancer (HNC) is a heterogeneous group of cancers, which includes cancers arising in the oral cavity, nasopharynx, oropharynx, hypopharynx, and larynx. Epidemiological studies have revealed that several factors such as cigarette (tobacco) use, alcohol consumption, exposure to environmental pollutants, and infection through viral agents such as the human papillomavirus (HPV) and the Epstein-Barr virus are risk factors for developing head and neck cancer. Additionally, genetic factors such as Fanconi anemia and genetic diseases, characterized by impaired DNA repair, increase the risk of first developing cancer in the oral cavity by 500-700 times (Johnson *et al.*, 2020). Oral Squamous Cell Carcinoma (OSCC) is one of the most common head and neck neoplasms, is a complex disease that arises in the oral cavity and oropharynx, is associated with tumor relapse and metastasis following traditional treatment, remaining a major clinical challenge in oral cancer management (Fang *et al.*, 2017; Bugshan and Farooq, 2020). One of the most common is Squamous Cell Carcinoma of the Oral Tongue (SCCOT), which is significantly more aggressive than the other forms of OSCC, with a propensity for rapid local invasion and spread, and a high recurrence rate (Franceschi *et al.*, 1993; Wang *et al.*, 2009). 

Some factors have been identified to increase the susceptibility to the development of head and neck cancer, such as genetic abnormalities that impact cell proliferation, characteristics of cell differentiation, changes in cell cycle, angiogenesis, and tumor metabolism. Furthermore, dysregulation in epigenetic machinery such as DNA methylation, histone modification, and non-coding RNAs have a direct influence on genetic activities (Hier *et al.*, 2021). 

Epigenetics is a term used to refer to changes that occur in the genome by altering gene expression without changes in the DNA sequence. One of the mechanisms that can lead to these changes is DNA methylation, which is defined by the addition of a methyl group (CH_3_) to a cytosine nucleotide and is responsible for modulating important cellular processes such as self-renewal and cellular dedifferentiation (Ehrlich and Lacey, 2013). DNA methylation can change the accessibility of transcription factors to regions containing gene promoters and also to distal regulatory regions such as enhancers, it can also remodel and organize the genome into genomic regions of active and inactive transcription. 

This epigenetic alteration can lead to dysregulation of pathways linked to cell differentiation, cell cycle control, apoptosis, angiogenesis, and metastasis; therefore DNA methylation and expression analysis have great potential to provide epigenetic markers for diagnosis, prognosis, evaluation risk assessment, and disease monitoring in various types of cancer (Zavridou *et al.*, 2020). In head and neck tumors, several genes were shown to be epigenetically regulated by DNA methylation and can be studied as therapeutic targets (Hier *et al.*, 2021). 

Here, we used DNA methylation changes to identify cancer-specific enhancers that were enriched for specific transcription factor binding sites (TFBS) and potential master regulator transcription factors (MRTF) associated with SCCOT.

The head and neck squamous cell carcinomas (HNSCC) data used were taken from The Cancer Genome Atlas (TCGA), named Genomic Data Commons Data Portal (GDC Data Portal), a reference program in cancer genomics that characterized about 20,000 primary cancers and corresponding normal samples, encompassing 33 different tumor types. We selected 147 HNSC tumor samples from the tongue region and 20 samples from adjacent regions. Of these tumor samples, 74 are HPV-negative, 11 are HPV-positive and 62 patients did not have this clinical data, of samples from adjacent regions 18 are HPV-negative, and 2 are HPV-positive (Table S1). The tongue region was chosen because it is one of the most frequent types of HNSCC, which comprises a heterogeneous group of cancers.

Level 3 gene expression data (RNAseq) and DNA methylation data (Illumina 450K) of TCGA-HNSCC were downloaded using the R/Bioconductor package TCGAbiolinks (Colaprico *et al.*, 2016; Silva *et al.*, 2016). Additionally, probes assessing DNA methylation levels present in distal regions of the genome were identified based on the genomic location provided by GENCODE. A probe is a single-stranded sequence of DNA used to assess the methylation level of a CpG site.

Data visualization and statistical analysis were performed using R software packages (https://www.r-project.org). Samples from the tongue and the adjacent regions were compared using the R/Bioconductor package ELMER (Silva *et al.*, 2019), using the unsupervised mode, to perform both hypomethylation and hypermethylation analyses. 

Elmer is an “R” based tool that uses DNA methylation levels to identify enhancer elements in the genome and correlates them with the expression of nearby genes in order to identify transcriptional targets and elucidate epigenetic regulatory mechanisms, it allows the inference of transcription factor (TF) binding motifs and the integration of transcription factor gene expression in order to identify which TFs regulate the biological process explored. 

DNA methylation can be used to identify functional changes at transcriptional enhancers and other cis-regulatory modules (CRMs) in tumors and other primary disease tissues. ELMER package reconstructs gene regulatory networks (GRNs) by combining methylation and gene expression data derived from the same set of samples, it uses methylation changes at CRMs as the central hub of these networks, using correlation analysis to associate them with both upstream master regulator (MR) transcription factors and downstream target genes. This analysis process can be briefly described in five steps: Identification of distal enhancer probes; Identification of distal probes that show significantly different DNA methylation levels between the analyzed groups; Identification of potential target genes for differentially methylated probes (probe-gene pairs) - search of nearby genes (10 upstream and 10 downstream) with a negative correlation between DNA methylation and gene expression; Identification of enriched motifs for probes belonging to probe-gene pairs - only the motifs that presented an odds ratio (95% CI) greater than 1.1 and that had a higher incidence in probes were used; and Identification of regulatory TFs whose expression is associated with DNA methylation in the previously identified motifs. 

In order not to have a large sampling difference between tumor and non-tumor (adjacent regions), we randomly selected 40% or tumor samples (59 out of 147) from the tongue region and 20 samples of adjacent tissue.

We applied the default probe filter “distal”, which uses 158.803 probes that are >2 kbp from any transcription start site as annotated by GENCODE. ELMER was used with the following parameters: get.diff.meth(sig.diff) = 0.3, get.diff.meth(p_value) = 0.001, get.diff.meth (minSubgroupFrac) = 0.4, get.pair(Pe) = 0.001, get.pair(raw.pvalue) = 0.05, get.pair(filter.probes) = FALSE, get.pair(diff.dir)=both, get.pair(permu.size) = 100, get.pair(minSubgroupFrac) = 0.4, get.enriched.motif(lower.OR) = 1.1, get.enriched.motif(min.incidence) = 10, and pathway analysis was performed using Reactome software (Gillespie *et al.*, 2022). 

Aberrant DNA methylation is a hallmark of the development of cancer and can affect gene expression, causing dysregulation of important cell mechanisms and leading to tumorigenesis; it could also affect the treatment response and resistance. Most studies focus on the CpG islands regions, once regions of focal hypermethylation in tumors were located primarily at CpG islands (Agrawal *et al.*, 2011; Bergman and Cedar, 2013). However there are studies demonstrating that methylations of distal regulatory sites are closely related to gene expression profiles of transformed cells and bear great relevance to further understanding of the specific regulatory networks, and provide critical information about gene expression control in tumorous and health cells (Aran *et al.*, 2013; Lin *et al.*, 2016; Pan *et al.*, 2020).

To investigate the alterations in the epigenome and its transcriptional implications in SCCOT, we compared DNA methylation data of tumor samples from the tongue to non-tumor samples from adjacent tumors from TCGA and identified 14,659 hypomethylated probes **(** Table S2A). Next, we searched for nearby genes with a negative correlation between DNA methylation and gene expression to identify putative transcriptional targets. We identified 696 probe-gene pairs with significant negative correlation (Figure 1a). A pathway analysis of these genes suggested activation of cell cycle and DNA replication (Figure 1b).

**Figure 1:**
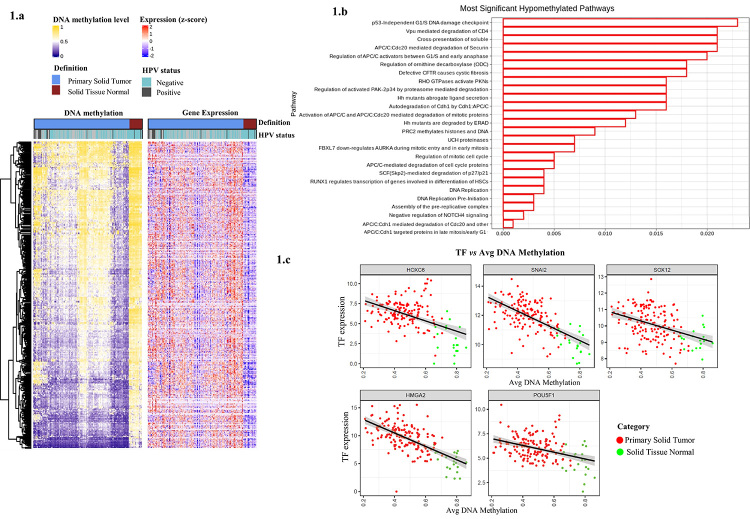
Hypomethylation analysis panel. **a.** Heatmap of hypomethylation analysis of tumor vs. non-tumor samples. **b.** Pathway analysis performed on Reactome showing the most significant hypomethylated pathways. **c.** Scatter plot of expression of the respective TFs (HMGA2, POU5F1, HOXC6, SNAI2, and SOX12) vs DNA methylation.. Source: Author.

Within the regulatory differentially methylated regions, we identified the enrichment of 69 distal-binding motifs for specific transcription factors. For each enriched motif, the pipeline computes the expression of each gene with the average DNA methylation of all distal probes using probe-gene pairs to identify possible TFs associated with these motifs (Table S2B). 

We identified 69 TFs that could potentially bind to these enriched motifs, TFs with TGAGTCA (Fos/Jun family) being the most represented. By computing the anticorrelation of TF gene expression and the DNA methylation levels within their binding sites, we identified HOXC6, SNAI2, SOX12, HMGA2, and POU5F1 candidate MRTFs with increasing expression in SCCOT compared to non-tumor sample (Table 1, Figure 1c).

**Table 1 -:** Hypomethylated TF’s related to HNSCC: biological function and influence in cancer development.

TF	Biological Function	Oncogene or tumor suppressive		Ref
		Cancer	HNSCC	
HMGA2	High cell proliferation, low apoptosis, tumor growth, promote angiogenesis, invasion, drug resistance and metastasis	Oncogene	Oncogene	(Mansoori *et al.*, 2021)
HOXC6	Associated with chemotherapeutic resistance, lymph node metastasis, induce apoptosis and cell growth	Oncogene	Oncogene	(Moon *et al.*, 2012; Zhang *et al.*, 2018)
POU5F1	tumor size, TNM stage, tumor differentiation, tumor invasion depth, lymph node metastasis, distant metastasis, lymphovascular invasion, vascular invasion, tumor number, and tumor recurrence	Oncogene	Oncogene	(He *et al.*, 2020)
SNAI2	lymph node metastasis, reduced overall survival, stem and/or progenitor cell biology, cellular differentiation, vascular remodeling and DNA damage repair	Oncogene	Oncogene	(Wang *et al.*, 2012; Steinbichler *et al.*, 2020)
SOX12	Tumor invasion, tumor differentiation, lymph node metastasis, distant metastasis	Oncogene	Oncogene	(Seok *et al.*, 2021; Su *et al.*, 2022)

Homeobox C6 (HOXC6) genes belong to the homeoprotein family of transcription factors. The overexpression has been found in several cancer types and OSCC, suggesting that HOX genes are implicated in the development of oral dysplasia and oral SCC and the acquisition of metastatic phenotypes (Zhang *et al.*, 2018).

SNAI2 is a transcription factor formerly known as Slug. It is involved in several biological processes and has been implicated in epithelial-mesenchymal transition (EMT) during embryonic development and tumor progression (Zhou *et al.*, 2019; Li Z *et al.*, 2022).

The SOX family of transcription factors is characterized by the presence of a DNA-binding high mobility group (HMG) domain. When SOX12 is highly expressed in OSCC, it is positively correlated with OSCC pathological grade, T stage, and N stage. In esophageal cancer (ESCC) overexpression of SOX12 indicated shorter overall survival (OS) time and disease-free survival (DFS) (Li *et al.*, 2021).

HMGA2 belongs to the family of small high mobility group proteins containing AT-hook DNA-binding domains. Is aberrantly regulated in several human tumors including tumors within the HNSCC type (Meyer *et al.*, 2007; Sterenczak *et al.*, 2014; Loeschke *et al.*, 2016; Zhao *et al.*, 2016; Binabaj *et al.*, 2019). The overexpression is associated with increased invasiveness, metastasis, epithelial-mesenchymal transition and stemness, and poor prognosis (Fang *et al.*, 2017; Li Z *et al.*, 2022).

POU Class 5 Homeobox 1 (POU5F1), also known as OCT-4, is a transcription factor of the POU family. Elevated POU5F1 was associated with tumorigenesis; poor overall survival, disease free survival, and recurrence free survival (RFS); and DSS in several cancers (Reers *et al.*, 2014; Koo *et al.*, 2015; Miyoshi *et al.*, 2018; He *et al.*, 2020; Sun *et al.*, 2021).

To investigate the regulatory factors silenced in SCCOT we analyzed genomic enhancer regions with gain of DNA methylation and associated downregulation of gene expression in tumor samples of the tongue compared to adjacent regions. We identified 6,956 hypermethylated probes and 331 probe-gene pairs with negative correlation (Table S3A, Figure 2a). A pathway analysis of these genes revealed inhibition of genes associated with transcription activity and development in tumor samples (Figure 2b). 

**Figure 2 -:**
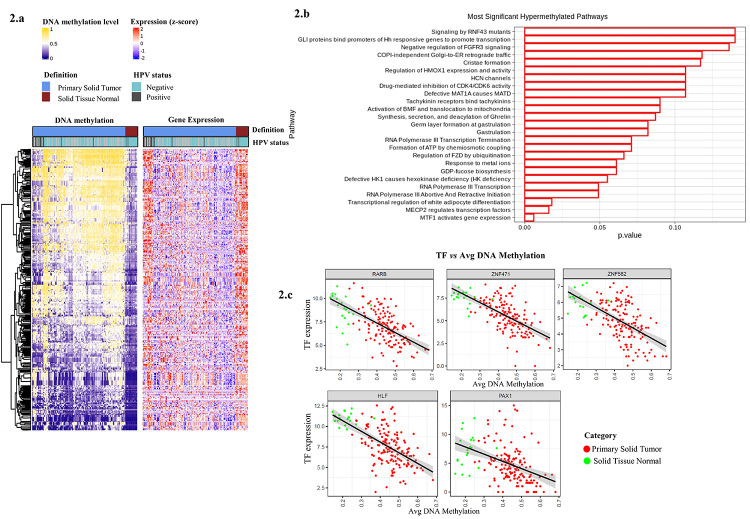
Hypermethylation analysis panel. **a.** Heatmap of hypermethylation analysis of tumor vs. non-tumor samples. **b.** Pathway analysis performed on Reactome showing the most significant hypermethylated pathways. **c.** Scatter plot of expression of the respective TFs (HLF, PAX1, RARB, ZNF471, and ZNF582) vs DNA methylation. Source: Author.

Next, we identified the enrichment of 76 TF binding motifs in regions with gain of DNA methylation in tumor samples, ABT4, KLF2 and E2F4 being the motifs most represented (Table S3B). Finally, we identified the downregulation of TF in tumors from the tongue, some of them associated with tumor suppression: HLF, PAX1, RARB, ZNF471, and ZNF582 (Figure 2c, Table 2). 

**Table 2 -:** Hypermethylated TF’s related to HNSCC: biological function and influence in cancer development.

TF	Biological Function	Oncogene or tumor suppressive		Ref
		Cancer	HNSCC	
HLF	Poorer progression-free survivals, cell growth, promotes anaerobic metabolism, distance metastasis and early relapse and progression	oncogene / tumor suppressive	tumor suppressive	(Chen *et al.*, 2020; Liu B *et al.*, 2021; Li *et al.*, 2022)
PAX1	Poor prognosis, apoptosis resistance, tumor cell proliferation and migration, repression of terminal differentiation, and finally progression of oral carcinogenesis	tumor suppressive	tumor suppressive	(Huang *et al.*, 2010; Huang *et al.*, 2014)
RARB	involved in suppressing cell growth and tumorigenicity, regulating gene expression and its retinoid-mediated antiproliferative, differentiative, immuno-modulatory, and apoptotic-inducing properties	tumor suppressive	tumor suppressive	(Giannini *et al.*, 1997; Bebek *et al.*, 2012)
ZNF471	poorer survival, suppressed cell proliferation, migration, and invasion	tumor suppressive	tumor suppressive	(Bhat *et al.*, 2016; Wang *et al.*, 2020)
ZNF582	DNA damage response, proliferation, cell cycle control, and neoplastic transformation	tumor suppressive	tumor suppressive	(Liu B *et al.*, 2019; Camuzi *et al.*, 2021)

HLF encodes a member of the proline and acidic-rich (PAR) protein family, a subset of the bZIP transcription factors. In Glioma, the overexpression could inhibit proliferation and invasion, in Triple Negative Breast Cancer promotes the ferroptosis resistance in TNBC cells via GGT1 and ultimately facilitates the malignant tumor progression (Liu Q *et al.*, 2021; Li H *et al.*, 2022).

PAX1 is a member of the paired box (PAX) transcription factor family. Inactivation of PAX1 gene by greater promoter methylation and/or somatic mutation may lead to apoptosis resistance, tumor cell proliferation and migration, repression of terminal differentiation, and progression of oral carcinogenesis (Cheng *et al.*, 2016).

Retinoic Acid Receptor Beta (RARB), is a member of the thyroid-steroid hormone receptor superfamily of nuclear transcriptional regulators. Changing in RARB expression is associated with cellular sensitivity to retinoid in numerous cancer cells, including HNSCC cells, betel quid related hypermethylation of RARB will increase the tumorigenesis and poor treatment outcome of oral cancer (Lai *et al.*, 2014).

ZNF471 e ZNF582 are members of the zinc-finger family. They are involved in all the principal pathways of cancer progression from carcinogenesis to metastasis formation, playing a key role as recruiters of chromatin modifiers or as structural proteins that regulate cancer cell migration and invasion (Huang *et al.*, 2012; Tao *et al.*, 2020).

Several studies have shown the relevance of studying distal regulatory regions of the gene code, and how epigenetic modulations can affect the overall gene expression (Stadler *et al.*, 2011; Aran *et al.*, 2013; Yao *et al.*, 2015; Lin *et al.*, 2016). Within the hypomethylation analysis, we observed that the candidates master regulators TFs in SCOOT are associated with increased invasiveness; metastasis; epithelial - mesenchymal transition; poor prognosis, overall survival, and recurrence-free survival; and stemness (Zhang *et al.*, 2018; Li *et al.*, 2021; Shahoumi, 2021; Li Z *et al.*, 2022). The majority of these genes, in healthy cells, are associated with cell differentiation during embryonic development (Moon *et al.*, 2012; Zhou *et al.*, 2019; He *et al.*, 2020). On the other hand, in the hypermethylation analysis, we noted that these candidates master regulators TFs, in oral cancers, are associated with tumor suppression and regulation, and their downregulation or inactivation can correlate with tumorigenesis and poor prognosis (Urrutia, 2003; Lai *et al.*, 2014; Cheng *et al.*, 2016). Besides, the ZNF582 and PAX1 are potential biomarkers for differentiating moderate dysplasia or worse (MODY+) oral lesions (Cheng *et al.*, 2018).

These results combined with the literature review indicate that ELMER package is an important and relevant tool to deduce regulatory element landscapes and gene regulatory networks from cancer methylomes (Yao *et al.*, 2015; Silva *et al.*, 2019). Moreover, the understanding of the epigenetic modulations, and how they can provoke global alterations during tumor evolution, is vital in order to characterize clinically heterogeneous malignancies, further understand their physiology, and improve diagnosis and treatment. Since epigenetic alteration of enhancer sites, such as methylation, is related to gene expression profiles of mutated cells, the identified MRTFs can further be used as biological markers for oral cancers, however, to completely understand their mechanisms and relevance in the overall development of the disease, further studies are required. 

## Supplementary material

The following online material is available for this article:

Table S1 - Identification of tumor samples from the tongue region and non-tumor samples from GDC Data Portal.

Table S2: S2A - Hypomethylated probes identified from the correlation between tumor samples from the tongue region and non-tumor samples. S2B: Transcription factors binding motifs and TF lists associated with DNA hypomethylation in SCCOT.

Table S3: S3A *-*
Hypermethylated probes identified from the correlation between tumor samples from the tongue region and non-tumor samples. S3B: Transcription factors binding motifs and TF lists associated with DNA hypermethylation in SCCOT.
