# Supplementary material for: Epigenomic integrative analysis pinpoint master regulator transcription factors associated with tumorigenesis in squamous cell carcinoma of oral tongue
Source: Genet Mol Biol. 2023 Jun 19;46(2):e20220358. doi: 10.1590/1678-4685-GMB-2022-0358 (PMC10280803; doi:10.1590/1678-4685-GMB-2022-0358)
Supplement: Table S1 - [file 1415-4757-GMB-46-2-e20220358-s1.pdf]

Genetics and Molecular Biology
https://doi.org/ 10.1590/1678-4685-GMB-2022-0358

Supplementary material to "Epigenomic integrative analysis pinpoint master regulator transcription factors associated with tumorigenesis in squamous cell carcinoma of oral tongue"

Table S1 – Identification and HPV status of tumor samples from the tongue region and non-tumor samples from GDC Data Portal.

Sample ID	Tissue type	HPV status	Sample ID	Tissue type	HPV status
TCGA-CQ-A4CA-01A	Primary solid Tumor	-	TCGA-MT-A51X-01A	Primary solid Tumor	-
TCGA-D6-A4Z9-01A	Primary solid Tumor	-	TCGA-IQ-A6SH-01A	Primary solid Tumor	-
TCGA-F7-A50J-01A	Primary solid Tumor	-	TCGA-CV-7438-01A	Primary solid Tumor	Negative
TCGA-CN-4736-01A	Primary solid Tumor	Negative	TCGA-CQ-5329-01A	Primary solid Tumor	Negative
TCGA-CQ-5330-01A	Primary solid Tumor	Negative	TCGA-CV-5971-01A	Primary solid Tumor	Positive
TCGA-CR-7391-01A	Primary solid Tumor	Negative	TCGA-D6-8569-01A	Primary solid Tumor	-
TCGA-BB-4224-01A	Primary solid Tumor	Negative	TCGA-CV-7180-01A	Primary solid Tumor	Negative

Sample ID	Tissue type	HPV status	Sample ID	Tissue type	HPV status
TCGA-CR-6493-01A	Primary solid Tumor	Negative	TCGA-CR-7401-01A	Primary solid Tumor	Negative
TCGA-CN-4737-01A	Primary solid Tumor	Negative	TCGA-CN-A642-01A	Primary solid Tumor	-
TCGA-F7-A61W-01A	Primary solid Tumor	-	TCGA-WA-A7H4-01A	Primary solid Tumor	-
TCGA-BA-A6DB-01A	Primary solid Tumor	-	TCGA-CV-7103-01A	Primary solid Tumor	Negative
TCGA-CV-7238-01A	Primary solid Tumor	Negative	TCGA-HD-8314-01A	Primary solid Tumor	-
TCGA-CR-6472-01A	Primary solid Tumor	Positive	TCGA-HD-7831-01A	Primary solid Tumor	Negative
TCGA-HD-A6HZ-01A	Primary solid Tumor	-	TCGA-CN-6019-01A	Primary solid Tumor	Negative
TCGA-CN-6024-01A	Primary solid Tumor	Negative	TCGA-DQ-5631-01A	Primary solid Tumor	Negative
TCGA-KU-A6H8-01A	Primary solid Tumor	-	TCGA-D6-6825-01A	Primary solid Tumor	Negative
TCGA-CV-5439-01A	Primary solid Tumor	Negative	TCGA-CV-5970-01A	Primary solid Tumor	Negative
TCGA-D6-6823-01A	Primary solid Tumor	Negative	TCGA-MZ-A7D7-01A	Primary solid Tumor	-
TCGA-CQ-6224-01A	Primary solid Tumor	Negative	TCGA-DQ-5625-01A	Primary solid Tumor	Negative
TCGA-CN-4742-01A	Primary solid Tumor	Negative	TCGA-CV-6941-01A	Primary solid Tumor	Negative

Sample ID	Tissue type	HPV status	Sample ID	Tissue type	HPV status
TCGA-CV-5979-01A	Primary solid Tumor	Negative	TCGA-CQ-A4CH-01A	Primary solid Tumor	-
TCGA-CN-5367-01A	Primary solid Tumor	Negative	TCGA-CV-6956-01A	Primary solid Tumor	Negative
TCGA-D6-A4ZB-01A	Primary solid Tumor	-	TCGA-CN-6998-01A	Primary solid Tumor	Negative
TCGA-CV-A6JU-01A	Primary solid Tumor	-	TCGA-CV-6954-01A	Primary solid Tumor	Negative
TCGA-CV-6436-01A	Primary solid Tumor	Negative	TCGA-CR-7393-01A	Primary solid Tumor	Negative
TCGA-D6-6515-01A	Primary solid Tumor	Negative	TCGA-UP-A6WW-01A	Primary solid Tumor	-
TCGA-CN-6996-01A	Primary solid Tumor	-	TCGA-CV-7446-01A	Primary solid Tumor	-
TCGA-QK-AA3K-01A	Primary solid Tumor	-	TCGA-BA-6873-01A	Primary solid Tumor	Negative
TCGA-CV-6951-01A	Primary solid Tumor	Negative	TCGA-CQ-5325-01A	Primary solid Tumor	Negative
TCGA-CQ-6229-01A	Primary solid Tumor	Negative	TCGA-CV-7104-01A	Primary solid Tumor	Negative
TCGA-F7-A61V-01A	Primary solid Tumor	-	TCGA-CN-5370-01A	Primary solid Tumor	Negative
TCGA-CQ-6218-01A	Primary solid Tumor	Negative	TCGA-C9-A480-01A	Primary solid Tumor	-
TCGA-CR-6488-01A	Primary solid Tumor	Negative	TCGA-CN-A6UY-01A	Primary solid Tumor	-

Sample ID	Tissue type	HPV status	Sample ID	Tissue type	HPV status
TCGA-CQ-6221-01A	Primary solid Tumor	Negative	TCGA-DQ-7591-01A	Primary solid Tumor	Positive
TCGA-CQ-A4CE-01A	Primary solid Tumor	-	TCGA-CR-7372-01A	Primary solid Tumor	Negative
TCGA-CR-7394-01A	Primary solid Tumor	Negative	TCGA-CV-6433-01A	Primary solid Tumor	Positive
TCGA-CV-6952-01A	Primary solid Tumor	Negative	TCGA-HD-8635-01A	Primary solid Tumor	-
TCGA-C9-A47Z-01A	Primary solid Tumor	-	TCGA-IQ-A6SG-01A	Primary solid Tumor	-
TCGA-MT-A67A-01A	Primary solid Tumor	-	TCGA-CN-A498-01A	Primary solid Tumor	-
TCGA-CV-6934-01A	Primary solid Tumor	Negative	TCGA-F7-A61S-01A	Primary solid Tumor	-
TCGA-BB-A6UO-01A	Primary solid Tumor	-	TCGA-CV-A45R-01A	Primary solid Tumor	-
TCGA-HD-8634-01A	Primary solid Tumor	-	TCGA-CN-6016-01A	Primary solid Tumor	Negative
TCGA-BB-4228-01A	Primary solid Tumor	Positive	TCGA-CQ-5333-01A	Primary solid Tumor	-
TCGA-DQ-5624-01A	Primary solid Tumor	Negative	TCGA-CV-A6K0-01B	Primary solid Tumor	-
TCGA-CV-6939-01A	Primary solid Tumor	Positive	TCGA-CR-5250-01A	Primary solid Tumor	Positive
TCGA-DQ-5630-01A	Primary solid Tumor	Negative	TCGA-CV-A6JO-01B	Primary solid Tumor	-

Sample ID	Tissue type	HPV status	Sample ID	Tissue type	HPV status
TCGA-CR-6477-01A	Primary solid Tumor	Negative	TCGA-CV-5976-01A	Primary solid Tumor	Negative
TCGA-QK-A652-01A	Primary solid Tumor	-	TCGA-CV-7255-01A	Primary solid Tumor	Negative
TCGA-BA-4074-01A	Primary solid Tumor	Negative	TCGA-H7-A6C4-01A	Primary solid Tumor	-
TCGA-CV-6961-01A	Primary solid Tumor	Positive	TCGA-CV-7236-01A	Primary solid Tumor	Negative
TCGA-CR-7397-01A	Primary solid Tumor	Negative	TCGA-CQ-6222-01A	Primary solid Tumor	-
TCGA-CV-6950-01A	Primary solid Tumor	Negative	TCGA-CN-A6V6-01A	Primary solid Tumor	-
TCGA-CV-6945-01A	Primary solid Tumor	Negative	TCGA-P3-A5QE-01A	Primary solid Tumor	-
TCGA-CQ-6219-01A	Primary solid Tumor	-	TCGA-CR-7382-01A	Primary solid Tumor	Negative
TCGA-CV-A45T-01A	Primary solid Tumor	-	TCGA-CV-7406-01A	Primary solid Tumor	Positive
TCGA-CV-6933-01A	Primary solid Tumor	Negative	TCGA-4P-AA8J-01A	Primary solid Tumor	-
TCGA-UF-A7JS-01A	Primary solid Tumor	-	TCGA-CX-7085-01A	Primary solid Tumor	Negative
TCGA-IQ-A61J-01A	Primary solid Tumor	-	TCGA-DQ-7592-01A	Primary solid Tumor	Negative
TCGA-CN-4725-01A	Primary solid Tumor	Negative	TCGA-CV-6003-01A	Primary solid Tumor	Negative

Sample ID	Tissue type	HPV status	Sample ID	Tissue type	HPV status
TCGA-CV-6959-01A	Primary solid Tumor	Negative	TCGA-BA-A6DE-01A	Primary solid Tumor	-
TCGA-CN-4733-01A	Primary solid Tumor	Negative	TCGA-IQ-A61E-01A	Primary solid Tumor	-
TCGA-F7-A50G-01A	Primary solid Tumor	-	TCGA-HD-8224-01A	Primary solid Tumor	-
TCGA-CV-A6JT-01A	Primary solid Tumor	-	TCGA-CQ-5327-01A	Primary solid Tumor	-
TCGA-BA-4075-01A	Primary solid Tumor	-	TCGA-CV-5973-01A	Primary solid Tumor	Negative
TCGA-CV-5977-01A	Primary solid Tumor	Negative	TCGA-CR-7390-01A	Primary solid Tumor	Negative
TCGA-BA-4077-01B	Primary solid Tumor	Positive	TCGA-IQ-A61H-01A	Primary solid Tumor	-
TCGA-T2-A6WZ-01A	Primary solid Tumor	-	TCGA-BA-6871-01A	Primary solid Tumor	Negative
TCGA-F7-A620-01A	Primary solid Tumor	-	TCGA-CQ-7065-01A	Primary solid Tumor	Negative
TCGA-CV-A465-01A	Primary solid Tumor	-	TCGA-CV-6441-01A	Primary solid Tumor	Negative
TCGA-CR-7392-01A	Primary solid Tumor	Negative	TCGA-P3-A5QA-01A	Primary solid Tumor	-
TCGA-CV-A45P-01A	Primary solid Tumor	-	TCGA-BB-4225-01A	Primary solid Tumor	Positive
TCGA-CN-6017-01A	Primary solid Tumor	Negative	TCGA-CV-6939-11A	Solid Tissue Normal	Positive

Sample ID	Tissue type	HPV status	Sample ID	Tissue type	HPV status
TCGA-CV-6943-01A	Primary solid Tumor	Negative	TCGA-CV-7103-11A	Solid Tissue Normal	Negative
TCGA-BA-A6DG-01A	Primary solid Tumor	-	TCGA-CV-6933-11A	Solid Tissue Normal	Negative
TCGA-BA-7269-01A	Primary solid Tumor	Negative	TCGA-CV-7101-11A	Solid Tissue Normal	Negative
TCGA-CQ-7072-01A	Primary solid Tumor	-	TCGA-CV-7250-11A	Solid Tissue Normal	Negative
TCGA-CV-7178-11A	Solid Tissue Normal	Negative			
TCGA-CV-6961-11A	Solid Tissue Normal	Positive			
TCGA-CV-6935-11A	Solid Tissue Normal	Negative			
TCGA-CV-7238-11A	Solid Tissue Normal	Negative			
TCGA-CV-7235-11A	Solid Tissue Normal	Negative			
TCGA-CV-6938-11A	Solid Tissue Normal	Negative			
TCGA-CV-6956-11A	Solid Tissue Normal	Negative			
TCGA-CV-6943-11A	Solid Tissue Normal	Negative			
TCGA-CV-6934-11A	Solid Tissue Normal	Negative			

Sample ID	Tissue type	HPV status	Sample ID	Tissue type	HPV status
TCGA-CV-6955-11A	Solid Tissue Normal	Negative			
TCGA-CV-6962-11A	Solid Tissue Normal	Negative			
TCGA-CV-7245-11A	Solid Tissue Normal	Negative			
TCGA-CV-6959-11A	Solid Tissue Normal	Negative			
TCGA-CV-7255-11A	Solid Tissue Normal	Negative			
TCGA-CV-6936-11A	Solid Tissue Normal	Negative			

Source: Author.
