# Supplementary material for: Epigenomic integrative analysis pinpoint master regulator transcription factors associated with tumorigenesis in squamous cell carcinoma of oral tongue
Source: Genet Mol Biol. 2023 Jun 19;46(2):e20220358. doi: 10.1590/1678-4685-GMB-2022-0358 (PMC10280803; doi:10.1590/1678-4685-GMB-2022-0358)
Supplement: Table S2: S2A - [file 1415-4757-GMB-46-2-e20220358-s2.pdf]

Genetics and Molecular Biology

https://doi.org/ 10.1590/1678-4685-GMB-2022-0358

Supplementary material to "Epigenomic integrative analysis pinpoint master regulator transcription factors associated with tumorigenesis in squamous cell carcinoma of oral tongue"

Table S2A - Hypomethylated probes identified from the correlation between tumor samples from the tongue region and non-tumor samples.

motif	Nº of probes	% of probes	lower OR	upper OR	OR	p.value	FDR	TF family	TF subfamily	TF.family.member	TF.subfamily.member
FOSB_HUMAN.H11MO.0.A	53	0.30994152	29.864.787	5.878.759	4,21E+06	0,00000001	3,05E-06	Fos-related factors {1.1.2}	Fos factors{1.1.2.1}	ATF3;FOS;FOSB;FOSL1;FOSL2;JDP2	FOSB;FOSL1;FOSL2;FOS
JUN_HUMAN.H11MO.0.A	53	0.30994152	29.827.737	5.871.455	4,21E+06	0,00000001	3,05E-06	Jun-related factors {1.1.1}	Jun factors{1.1.1.1}	ATF2;ATF7;BACH1;BACH2;CREB5;JUN;JUNB;JUND;NFE2;NFE2L1;NFE2L2;NFE2L3	JUNB;JUND;JUN
FOSL2_HUMAN.H11MO.0.A	51	0.29824561	27.932.074	5.539.291	3,96E+06	0,00000017	3,23E-05	Fos-related factors {1.1.2}	Fos factors{1.1.2.1}	ATF3;FOS;FOSB;FOSL1;FOSL2;JDP2	FOSB;FOSL1;FOSL2;FOS
JUNB_HUMAN.H11MO.0.A	53	0.30994152	27.678.753	5.448.045	3,91E+06	0,00000012	3,04E-05	Jun-related factors {1.1.1}	Jun factors{1.1.1.1}	ATF2;ATF7;BACH1;BACH2;CREB5;JUN;JUNB;JUND;NFE2;NFE2L1;NFE2L2;NFE2L3	JUNB;JUND;JUN
FOS_HUMAN.H11MO.0.A	48	0.28070175	25.168.290	5.056.576	3,59E+06	0,00001371	2,11E-03	Fos-related factors {1.1.2}	Fos factors{1.1.2.1}	ATF3;FOS;FOSB;FOSL1;FOSL2;JDP2	FOSB;FOSL1;FOSL2;FOS
JUND_HUMAN.H11MO.0.A	49	0.28654971	24.702.273	4.939.838	3,52E+06	0,00001852	2,38E-03	Jun-related factors {1.1.1}	Jun factors{1.1.1.1}	ATF2;ATF7;BACH1;BACH2;CREB5;JUN;JUNB;JUND;NFE2;NFE2L1;NFE2L2;NFE2L3	JUNB;JUND;JUN
FOSL1_HUMAN.H11MO.0.A	47	0.27485380	23.943.468	4.832.928	3,43E+06	0,00008548	9,41E-03	Fos-related factors {1.1.2}	Fos factors{1.1.2.1}	ATF3;FOS;FOSB;FOSL1;FOSL2;JDP2	FOSB;FOSL1;FOSL2;FOS
BATF_HUMAN.H11MO.1.A	29	0.16959064	21.620.086	5.021.980	3,35E+06	0,17942190	8,14E+00	B-ATF-related factors {1.1.4}	B-ATF{1.1.4.0.1}	BATF;BATF2;BATF3	BATF
HXD9_HUMAN.H11MO.0.D	14	0.08187135	19.712.349	6.393.622	3,69E+06	63,53484000	1,32E+03	HOX-related factors {3.1.1}	HOX9-13{3.1.1.8}	CDX1;CDX2;CDX4;EVX1;EVX2;GBX1;GBX2;GSX1;GSX2;HDX;HMBOX1;HNF1A;HNF1B;HOXA1;HOXA10;HOXA11;HOXA13;HOXA2;HOXA3;HOXA4;HOXA5;HOXA6;HOXA7;HOXA9;HOXB1;HOXB13;HOXB2;HOXB3;HOXB4;HOXB5;HOXB6;HOXB7;HOXB8;HOXB9;HOXC10;HOXC11;HOXC12;HOXC13;HOXC4;HOXC5;HOXC6;HOXC8;HOXC9;HOXD1;HOXD10;HOXD11;HOXD12;HOXD13;HOXD3;HOXD4;HOXD8;HOXD9;MEOX1;MEOX2;MNX1;PDX1;POU1F1;POU2F1;POU2F2;POU2F3;POU3F1;POU3F2;POU3F3;POU3F4;POU4F1;POU4F2;POU4F3;POU5F1;POU5F2;POU6F1;POU6F2	HOXA10;HOXA11;HOXA13;HOXA9;HOXB13;HOXC10;HOXC11;HOXC12;HOXC13;HOXC9;HOXD10;HOXD11;HOXD12;HOXD13;HOXD9
NF2L2_HUMAN.H11MO.0.A	35	0.20467836	17.239.323	3.762.900	2,58E+06	5,78908300	1,94E+02	Jun-related factors {1.1.1}	NF-E2-like factors{1.1.1.2}	ATF2;ATF7;BACH1;BACH2;CREB5;JUN;JUNB;JUND;NFE2;NFE2L1;NFE2L2;NFE2L3	BACH1;BACH2;NFE2L1;NFE2L2;NFE2

motif	Nº of probes	% of probes	lower OR	upper OR	OR	p.value	FDR	TF family	TF subfamily	TF.family.member	TF.subfamily.member
PO6F1_HUMAN.H11MO.0.D	20	0.11695906	15.872.204	4.282.159	2,67E+06	185,89920000	3,12E+03	POU domain factors{3.1.10}	POU6 (Brn-5-like factors){3.1.10.6}	CDX1;CDX2;CDX4;EVX1;EVX2;GBX1;GBX2;GSX1;GSX2;HDX;HMBOX1;HNF1A;HNF1B;HOXA1;HOXA10;HOXA11;HOXA13;HOXA2;HOXA3;HOXA4;HOXA5;HOXA6;HOXA7;HOXA9;HOXB1;HOXB13;HOXB2;HOXB3;HOXB4;HOXB5;HOXB6;HOXB7;HOXB8;HOXB9;HOXC10;HOXC11;HOXC12;HOXC13;HOXC4;HOXC5;HOXC6;HOXC8;HOXC9;HOXD1;HOXD10;HOXD11;HOXD12;HOXD13;HOXD3;HOXD4;HOXD8;HOXD9;MEOX1;MEOX2;MNX1;PDX1;POU1F1;POU2F1;POU2F2;POU2F3;POU3F1;POU3F2;POU3F3;POU3F4;POU4F1;POU4F2;POU4F3;POU5F1;POU5F1B;POU5F2;POU6F1;POU6F2	POU6F1;POU6F2
TWST1_HUMAN.H11MO.0.A	27	0.15789474	15.776.214	3.758.936	2,48E+06	72,98327000	1,48E+03	Tal-related factors{1.2.3}	Twist-like factors{1.2.3.2}	ATOH1;ATOH7;ATOH8;BHLHA15;BHLHA9;BHLHE22;BHLHE23;FERD3L;FIGLA;HAND1;HAND2;LYL1;MESP1;MESP2;MSC;MSGN1;NEUROD1;NEUROD2;NEUROD4;NEUROD6;NEUROG1;NEUROG2;NEUROG3;NHLH1;NHLH2;OLIG1;OLIG2;OLIG3;PTF1A;SCX;TAL1;TAL2;TCF15;TCF21;TCF23;TWIST1;TWIST2	FIGLA;HAND1;PTF1A;TWIST1
RX_HUMAN.H11MO.0.D	17	0.09941520	15.452.897	4.509.408	2,72E+06	415,84760000	4,86E+03	Paired-related HD factors{3.1.3}	RAX{3.1.3.22}	ALX1;ALX3;ALX4;ARGFX;ARX;CRX;DMBX1;DPRX;DRGX;DUX4;DUXA;ESX1;GSC;GSC2;HESX1;ISX;LEUTX;MIXL1;NOBOX;OTP;OTX1;OTX2;PHOX2A;PHOX2B;PITX1;PITX2;PITX3;PROP1;PRRX1;PRRX2;RAX;RAX2;RHOXF1;RHOXF2;SEBOX;SHOX;SHOX2;TPRX1;UNCX;VSX1;VSX2	RAX2;RAX
RUNX1_HUMAN.H11MO.0.A	38	0.22222222	15.252.002	3.249.590	2,25E+06	46,54854000	1,12E+03	Runt-related factors{6.4.1}	Runx1 (PEBP2alphaB, CBF-alpha2, AML-1){6.4.1.0.2}	RUNX1;RUNX2;RUNX3	RUNX1
ZNF8_HUMAN.H11MO.0.C	22	0.12865497	15.200.740	3.931.177	2,50E+06	291,31630000	3,87E+03	Factors with multiple dispersed zinc fingers{2.3.4}	unclassified{2.3.4.0}	BCL11A;E4F1;MECOM;HIC1;HIC2;HINFP;IKZF1;INSM1;MAZ;PATZ1;PRDM4;REST;RREB1;SALL4;VEZF1;ZBTB17;ZBTB4;HIVEP1;HIVEP2;ZNF134;ZNF219;ZNF335;ZNF341;ZNF382;ZNF418;ZNF423;ZNF467;ZNF770;ZNF784;ZNF8	E4F1;PRDM4;REST;RREB1;ZBTB17;ZBTB4;ZNF335;ZNF341;ZNF467;ZNF770;ZNF784;ZNF8
AIRE_HUMAN.H11MO.0.C	17	0.09941520	15.191.592	4.432.497	2,68E+06	499,18820000	5,58E+03	AIRE{5.3.1}	AIRE{5.3.1.0.1}	AIRE	AIRE
BACH2_HUMAN.H11MO.0.A	38	0.22222222	15.058.561	3.208.375	2,22E+06	53,13401000	1,20E+03	Jun-related factors{1.1.1}	NF-E2-like factors{1.1.1.2}	ATF2;ATF7;BACH1;BACH2;CREB5;JUN;JUNB;JUND;NFE2;NFE2L1;NFE2L2;NFE2L3	BACH1;BACH2;NFE2L1;NFE2L2;NFE2
HXC11_HUMAN.H11MO.0.D	22	0.12865497	14.834.924	3.836.523	2,44E+06	368,00660000	4,65E+03	HOX-related factors{3.1.1}	HOX9-13{3.1.1.8}	CDX1;CDX2;CDX4;EVX1;EVX2;GBX1;GBX2;GSX1;GSX2;HDX;HMBOX1;HNF1A;HNF1B;HOXA1;HOXA10;HOXA11;HOXA13;HOXA2;HOXA3;HOXA4;HOXA5;HOXA6;HOXA7;HOXA9;HOXB1;HOXB13;HOXB2;HOXB3;HOXB4;HOXB5;HOXB6;HOXB7;HOXB8;HOXB9;HOXC10;HOXC11;HOXC12;HOXC13;HOXC4;HOXC5;HOXC6;HOXC8;HOXC9;HOXD1;HOXD10;HOXD11;HOXD12;HOXD13;HOXD3;HOXD4;HOXD8;HOXD9;MEOX1;MEOX2;MNX1;PDX1;POU1F1;POU2F1;POU2F2;POU2F3;POU3F1;POU3F2;POU3F3;POU3F4;POU4F1;POU4F2;POU4F3;POU5F1;POU5F2;POU6F1;POU6F2	HOXA10;HOXA11;HOXA13;HOXA9;HOXB13;HOXC10;HOXC11;HOXC12;HOXC13;HOXC9;HOXD10;HOXD11;HOXD12;HOXD13;HOXD9
FOXF1_HUMAN.H11MO.0.D	37	0.21637427	14.771.602	3.171.312	2,19E+06	81,53663000	1,58E+03	Forkhead box (FOX) factors{3.3.1}	FOXF{3.3.1.6}	FOXA1;FOXA2;FOXA3;FOXB1;FOXB2;FOXC1;FOXC2;FOXD1;FOXD2;FOXD3;FOXD4;FOXD4L1;FOXD4L3;FOXD4L4;FOXD4L5;FOXD4L6;FOXE1;FOXE3;FOXF1;FOXF2;FOXG1;FOXH1;FOXI1;FOXI2;FOXI3;FOXJ1;FOXJ2;FOXJ3;FOXK1;FOXK2;FOXL1;FOXL2;FOXM1;FOXN1;FOXN2;FOXN3;FOXN4;FOXO1;FOXO3;FOXO4;FOXO6;FOXP1;FOXP2;FOXP3;FOXP4;FOXQ1;FOXR1;FOXR2;FOXS1	FOXF1;FOXF2

motif	Nº of probes	% of probes	lower OR	upper OR	OR	p.value	FDR	TF family	TF subfamily	TF.family.member	TF.subfamily.member
OLIG1_HUMAN.H11MO.0.D	19	0.11111111	13.933.175	3.849.608	2,38E+06	1138,74100000	9,65E+03	Tal-related factors{1.2.3}	Neurogenin / Atonal-like factors{1.2.3.4}	ATOH1;ATOH7;ATOH8;BHLHA15;BHLHA9;BHLHE22;BHLHE23;FERD3L;FIGLA;HAND1;HAND2;LYL1;MESP1;MESP2;MSC;MSGN1;NEUROD1;NEUROD2;NEUROD4;NEUROD6;NEUROG1;NEUROG2;NEUROG3;NHLH1;NHLH2;OLIG1;OLIG2;OLIG3;PTF1A;SCX;TAL1;TAL2;TCF15;TCF21;TCF23;TWIST1;TWIST2	ATOH1;BHLHA15;BHLHE22;BHLHE23;NEUROD1;NEUROD2;NEUROG2;OLIG1;OLIG2;OLIG3
HNF1B_HUMAN.H11MO.1.A	18	0.10526316	13.929.772	3.949.816	2,41E+06	1390,77100000	1,09E+04	POU domain factors{3.1.10}	HNF1-like factors{3.1.10.7}	CDX1;CDX2;CDX4;EVX1;EVX2;GBX1;GBX2;GSX1;GSX2;HDX;HMBOX1;HNF1A;HNF1B;HOXA1;HOXA10;HOXA11;HOXA13;HOXA2;HOXA3;HOXA4;HOXA5;HOXA6;HOXA7;HOXA9;HOXB1;HOXB13;HOXB2;HOXB3;HOXB4;HOXB5;HOXB6;HOXB7;HOXB8;HOXB9;HOXC10;HOXC11;HOXC12;HOXC13;HOXC4;HOXC5;HOXC6;HOXC8;HOXC9;HOXD1;HOXD10;HOXD11;HOXD12;HOXD13;HOXD3;HOXD4;HOXD8;HOXD9;MEOX1;MEOX2;MNX1;PDX1;POU1F1;POU2F1;POU2F2;POU2F3;POU3F1;POU3F2;POU3F3;POU3F4;POU4F1;POU4F2;POU4F3;POU5F1;POU5F1B;POU5F2;POU6F1;POU6F2	HMBOX1;HNF1A;HNF1B
PRRX1_HUMAN.H11MO.0.D	17	0.09941520	13.821.132	4.032.358	2,44E+06	1840,67800000	1,33E+04	Paired-related HD factors{3.1.3}	PRRX{3.1.3.21}	ALX1;ALX3;ALX4;ARGFX;ARX;CRX;DMBX1;DPRX;DRGX;DUX4;DUXA;ESX1;GSC;GSC2;HESX1;ISX;LEUTX;MIXL1;NOBOX;OTP;OTX1;OTX2;PHOX2A;PHOX2B;PITX1;PITX2;PITX3;PROP1;PRRX1;PRRX2;RAX;RAX2;RHOXF1;RHOXF2;SEBOX;SHOX;SHOX2;TPRX1;UNCX;VSX1;VSX2	PRRX1;PRRX2
RUNX3_HUMAN.H11MO.0.A	33	0.19298246	13.690.717	3.043.300	2,07E+06	575,97600000	5,84E+03	Runt-related factors{6.4.1}	Runx3 (PEBP2alphaC, CBF-alpha3, AML-2){6.4.1.0.3}	RUNX1;RUNX2;RUNX3	RUNX3
ZN136_HUMAN.H11MO.0.C	20	0.11695906	13.661.047	3.684.908	2,30E+06	1173,65300000	9,77E+03	More than 3 adjacent zinc finger factors{2.3.3}	ZNF763-like factors{2.3.3.33}	BCL6;BCL6B;CTCF;CTCFL;FEZF1;FEZF2;GFI1;GFI1B;GLI1;GLI2;GLI3;GLI4;GLIS1;GLIS2;GLIS3;HKR1;MTF1;MYNN;MZF1;OSR2;OVOL1;OVOL2;PLAG1;PLAGL1;PLAGL2;PRDM1;PRDM14;PRDM6;SCRT1;SCRT2;SNAI1;SNAI2;SNAI3;WT1;YY1;YY2;ZBTB12;ZBTB14;ZBTB18;ZBTB20;ZBTB26;ZBTB42;ZBTB45;ZBTB47;ZBTB48;ZBTB49;ZBTB6;ZBTB7A;ZBTB7B;ZBTB7C;ZFP14;ZFP2;ZFP28;ZFP30;ZFP37;ZFP42;ZFP64;ZFP69;ZFP69B;ZFP82;ZFP91;ZFX;ZIC1;ZIC2;ZIC3;ZIC4;ZIC5;ZIK1;ZIM3;ZKSCAN1;ZKSCAN2;ZKSCAN3;ZKSCAN4;ZNF121;ZNF124;ZNF133;ZNF136;ZNF138;ZNF14;ZNF140;ZNF143;ZNF146;ZNF148;ZNF155;ZNF157;ZNF160;ZNF169;ZNF175;ZNF177;ZNF18;ZNF180;ZNF181;ZNF2;ZNF20;ZNF212;ZNF213;ZNF214;ZNF221;ZNF222;ZNF223;ZNF224;ZNF225;ZNF226;ZNF227;ZNF229;ZNF230;ZNF232;ZNF233;ZNF234;ZNF235;ZNF24;ZNF25;ZNF250;ZNF257;ZNF26;ZNF260;ZNF263;ZNF264;ZNF268;ZNF274;ZNF276;ZNF28;ZNF280A;ZNF280B;ZNF280C;ZNF280D;ZNF281;ZNF282;ZNF283;ZNF284;ZNF285;ZNF286A;ZNF286B;ZNF3;ZNF30;ZNF300;ZNF302;ZNF317;ZNF32;ZNF320;ZNF322;ZNF324;ZNF324B;ZNF329;ZNF331;ZNF333;ZNF33A;ZNF33B;ZNF343;ZNF345;ZNF347;ZNF350;ZNF354A;ZNF354B;ZNF362;ZNF366;ZNF383;ZNF384;ZNF394;ZNF397;ZNF398;ZNF404;ZNF41;ZNF410;ZNF419;ZNF420;ZNF431;ZNF432;ZNF436;ZNF439;ZNF44;ZNF440;ZNF442;ZNF443;ZNF446;ZNF449;ZNF45;ZNF460;ZNF468;ZNF479;ZNF484;ZNF490;ZNF500;ZNF502;ZNF524;ZNF525;ZNF528;ZNF543;ZNF544;ZNF546;ZNF547;ZNF548;ZNF549;ZNF554;ZNF555;ZNF557;ZNF558;ZNF559;ZNF561;ZNF562;ZNF563;ZNF564;ZNF566;ZNF567;ZNF568;ZNF5	ZNF136;ZNF490;ZNF563

motif	Nº of probes	% of probes	lower OR	upper OR	OR	p.value	FDR	TF family	TF subfamily	TF.family.member	TF.subfamily.member
										7;ZNF570;ZNF571;ZNF572;ZNF577;ZNF581;ZNF582;ZNF583;ZNF585A;ZNF586;ZNF589;ZNF595;ZNF599;ZNF600;ZNF605;ZNF607;ZNF611;ZNF613;ZNF614;ZNF615;ZNF616;ZNF619;ZNF620;ZNF621;ZNF625;ZNF627;ZNF649;ZNF652;ZNF653;ZNF665;ZNF667;ZNF669;ZNF670;ZNF672;ZNF679;ZNF680;ZNF683;ZNF689;ZNF692;ZNF701;ZNF705D;ZNF705E;ZNF705G;ZNF708;ZNF709;ZNF71;ZNF710;ZNF713;ZNF721;ZNF727;ZNF729;ZNF736;ZNF75A;ZNF75D;ZNF76;ZNF763;ZNF764;ZNF765;ZNF768;ZNF77;ZNF771;ZNF773;ZNF774;ZNF776;ZNF777;ZNF780A;ZNF780B;ZNF782;ZNF785;ZNF799;ZNF805;ZNF808;ZNF81;ZNF813;ZNF816;ZNF823;ZNF829;ZNF836;ZNF841;ZNF844;ZNF845;ZNF846;ZNF85;ZNF853;ZNF860;ZNF878;ZNF891;ZNF99;ZSCAN16;ZSCAN2;ZSCAN22;ZSCAN23;ZSCAN29;ZSCAN31;ZSCAN32;ZSCAN4;ZSCAN5A;ZSCAN5B;ZSCAN5C;ZSCAN9;ZXDA;ZXDB;ZXDC	
NFIL3_HUMAN.H11MO.0.D	18	0.10526316	13.496.602	3.826.852	2,34E+06	1760,88800000	1,29E+04	C/EBP-related{1.1.8}	PAR factors{1.1.8.2}	CEBPA;CEBPB;CEBPD;CEBPE;CEBPG;DBP;DDIT3;HLF;NFIL3;TEF	DBP;HLF;NFIL3;TEF
FOXA2_HUMAN.H11MO.0.A	29	0.16959064	13.472.216	3.127.485	2,09E+06	706,32370000	6,75E+03	Forkhead box (FOX) factors {3.3.1}	FOXA{3.3.1.1}	FOXA1;FOXA2;FOXA3;FOXB1;FOXB2;FOXC1;FOXC2;FOXD1;FOXD2;FOXD3;FOXD4;FOXD4L1;FOXD4L3;FOXD4L4;FOXD4L5;FOXD4L6;FOXE1;FOXE3;FOXF1;FOXF2;FOXG1;FOXH1;FOXI1;FOXI2;FOXI3;FOXJ1;FOXJ2;FOXJ3;FOXK1;FOXK2;FOXL1;FOXL2;FOXM1;FOXN1;FOXN2;FOXN3;FOXN4;FOXO1;FOXO3;FOXO4;FOXO6;FOXP1;FOXP2;FOXP3;FOXP4;FOXQ1;FOXR1;FOXR2;FOXS1	FOXA1;FOXA2;FOXA3
ZIM3_HUMAN.H11MO.0.C	33	0.19298246	13.181.506	2.929.966	1,99E+06	768,69160000	7,14E+03	More than 3 adjacent zinc finger factors{2.3.3}	unclassified{2.3.3.0}	BCL6B;BCL6;CTCFL;CTCF;FEZF1;GFI1B;GFI1;GLI1;GLI2;GLI3;GLIS1;GLIS2;GLIS3;MTF1;MYNN;MZF1;OSR2;OVOL1;OVOL2;ZNF146;PLAG1;PLAGL1;PRDM14;PRDM1;PRDM6;SCRT1;SCRT2;SNAI1;SNAI2;YY1;YY2;WT1;ZNF324;ZNF354A;ZBTB14;ZBTB18;ZBTB48;ZBTB49;ZBTB7A;ZBTB7B;ZBTB6;ZFP64;ZFP28;ZFP42;ZFP82;ZFX;ZIC1;ZIC2;ZIC3;ZIC4;ZIM3;ZKSCAN1;ZKSCAN3;ZNF121;ZNF136;ZNF140;ZNF143;ZNF148;ZNF214;ZNF232;ZNF250;ZNF257;ZNF260;ZNF263;ZNF264;ZNF274;ZNF281;ZNF282;ZNF317;ZNF320;ZNF322;ZNF329;ZNF331;ZNF333;ZNF350;ZNF384;ZNF394;ZNF410;ZNF436;ZNF449;ZNF490;ZNF502;ZNF524;ZNF528;ZNF547;ZNF549;ZNF554;ZNF563;ZNF582;ZNF586;ZNF589;ZNF652;ZNF667;ZNF680;ZNF708;ZNF713;ZNF768;ZNF816;ZNF18;ZNF41;ZNF76;ZNF85;ZSCAN16;ZSCAN22;ZSCAN31;ZSCAN4	MYNN;MZF1;OSR2;PRDM14;PRDM6;WT1;ZBTB14;ZBTB48;ZBTB49;ZFP64;ZFP28;ZIM3;ZNF121;ZNF250;ZNF257;ZNF263;ZNF274;ZNF317;ZNF320;ZNF329;ZNF331;ZNF394;ZNF449;ZNF502;ZNF528;ZNF547;ZNF549;ZNF554;ZNF586;ZNF589;ZNF667;ZNF680;ZNF708;ZNF713;ZNF768;ZNF18;ZNF85;ZSCAN16;ZSCAN22
NFE2_HUMAN.H11MO.0.A	38	0.22222222	13.032.850	2.776.504	1,92E+06	828,12680000	7,60E+03	Jun-related factors{1.1.1}	NF-E2-like factors{1.1.1.2}	ATF2;ATF7;BACH1;BACH2;CREB5;JUN;JUNB;JUND;NFE2;NFE2L1;NFE2L2;NFE2L3	BACH1;BACH2;NFE2L1;NFE2L2;NFE2
GATA1_HUMAN.H11MO.1.A	22	0.12865497	13.016.836	3.366.019	2,14E+06	2513,70900000	1,74E+04	GATA-type zinc fingers{2.2.1}	Two zinc-finger GATA factors{2.2.1.1}	GATA1;GATA2;GATA3;GATA4;GATA5;GATA6;GATAD2A;GATAD2B;TRPS1;ZGLP1	GATA1;GATA2;GATA3;GATA4;GATA5;GATA6

motif	Nº of probes	% of probes	lower OR	upper OR	OR	p.value	FDR	TF family	TF subfamily	TF.family.member	TF.subfamily.member
Z354A_HUMAN.H11MO.0.C	32	0.18713450	12.988.570	2.915.898	1,97E+06	1043,87600000	8,94E+03	More than 3 adjacent zinc finger factors{2.3.3}	ZNF354A-like factors{2.3.3.64}	BCL6;BCL6B;CTCF;CTCFL;FEZF1;FEZF2;GFI1;GFI1B;GLI1;GLI2;GLI3;GLI4;GLIS1;GLIS2;GLIS3;HKR1;MTF1;MYNN;MZF1;OSR2;OVOL1;OVOL2;PLAG1;PLAGL1;PLAGL2;PRDM1;PRDM14;PRDM6;SCRT1;SCRT2;SNAI1;SNAI2;SNAI3;WT1;YY1;YY2;ZBTB12;ZBTB14;ZBTB18;ZBTB20;ZBTB26;ZBTB42;ZBTB45;ZBTB47;ZBTB48;ZBTB49;ZBTB6;ZBTB7A;ZBTB7B;ZBTB7C;ZFP14;ZFP2;ZFP28;ZFP30;ZFP37;ZFP42;ZFP64;ZFP69;ZFP69B;ZFP82;ZFP91;ZFX;ZIC1;ZIC2;ZIC3;ZIC4;ZIC5;ZIK1;ZIM3;ZKSCAN1;ZKSCAN2;ZKSCAN3;ZKSCAN4;ZNF121;ZNF124;ZNF133;ZNF136;ZNF138;ZNF14;ZNF140;ZNF143;ZNF146;ZNF148;ZNF155;ZNF157;ZNF160;ZNF169;ZNF175;ZNF177;ZNF18;ZNF180;ZNF181;ZNF2;ZNF20;ZNF212;ZNF213;ZNF214;ZNF221;ZNF222;ZNF223;ZNF224;ZNF225;ZNF226;ZNF227;ZNF229;ZNF230;ZNF232;ZNF233;ZNF234;ZNF235;ZNF24;ZNF25;ZNF250;ZNF257;ZNF26;ZNF260;ZNF263;ZNF264;ZNF268;ZNF274;ZNF276;ZNF28;ZNF280A;ZNF280B;ZNF280C;ZNF280D;ZNF281;ZNF282;ZNF283;ZNF284;ZNF285;ZNF286A;ZNF286B;ZNF3;ZNF30;ZNF300;ZNF302;ZNF317;ZNF32;ZNF320;ZNF322;ZNF324;ZNF324B;ZNF329;ZNF331;ZNF333;ZNF33A;ZNF33B;ZNF343;ZNF345;ZNF347;ZNF350;ZNF354A;ZNF354B;ZNF362;ZNF366;ZNF383;ZNF384;ZNF394;ZNF397;ZNF398;ZNF404;ZNF41;ZNF410;ZNF419;ZNF420;ZNF431;ZNF432;ZNF436;ZNF439;ZNF44;ZNF440;ZNF442;ZNF443;ZNF446;ZNF449;ZNF45;ZNF460;ZNF468;ZNF479;ZNF484;ZNF490;ZNF500;ZNF502;ZNF524;ZNF525;ZNF528;ZNF543;ZNF544;ZNF546;ZNF547;ZNF548;ZNF549;ZNF554;ZNF555;ZNF557;ZNF558;ZNF559;ZNF561;ZNF562;ZNF563;ZNF564;ZNF566;ZNF567;ZNF568;ZNF57;ZNF570;ZNF571;ZNF572;ZNF577;ZNF581;ZNF582;ZNF583;ZNF585A;ZNF586;ZNF589;ZNF595;ZNF599;ZNF600;ZNF605;ZNF607;ZNF611;ZNF613;ZNF614;ZNF615;ZNF616;ZNF619;ZNF620;ZNF621;ZNF625;ZNF627;ZNF649;ZNF652;ZNF653;ZNF665;ZNF667;ZNF669;ZNF670;ZNF672;ZNF679;ZNF680;ZNF683;ZNF689;ZNF692;ZNF701;ZNF705D;ZNF705E;ZNF705G;ZNF708;ZNF709;ZNF71;ZNF710;ZNF713;ZNF721;ZNF727;ZNF729;ZNF736;ZNF75A;ZNF75D;ZNF76;ZNF763;ZNF764;ZNF765;ZNF768;ZNF77;ZNF771;ZNF773;ZNF774;ZNF776;ZNF777;ZNF780A;ZNF780B;ZNF782;ZNF785;ZNF799;ZNF805;ZNF808;ZNF81;ZNF813;ZNF816;ZNF823;ZNF829;ZNF836;ZNF841;ZNF844;ZNF845;ZNF846;ZNF85;ZNF853;ZNF860;ZNF878;ZNF891;ZNF99;ZSCAN16;ZSCAN2;ZSCAN22;ZSCAN23;ZSCAN29;ZSCAN31;ZSCAN32;ZSCAN4;ZSCAN5A;ZSCAN5B;ZSCAN5C;ZSCAN9;ZXDA;ZXDB;ZXDC	ZNF354A
ZFP28_HUMAN.H11MO.0.C	44	0.25730994	12.817.839	2.627.098	1,85E+06	733,51680000	6,90E+03	More than 3 adjacent zinc finger factors{2.3.3}	unclassified{2.3.3.0}	BCL6B;BCL6;CTCFL;CTCF;FEZF1;GFI1B;GFI1;GLI1;GLI2;GLI3;GLIS1;GLIS2;GLIS3;MTF1;MYNN;MZF1;OSR2;OVOL1;OVOL2;ZNF146;PLAG1;PLAGL1;PRDM14;PRDM1;PRDM6;SCRT1;SCRT2;SNAI1;SNAI2;YY1;YY2;WT1;ZNF324;ZNF354A;ZBTB14;ZBTB18;ZBTB48;ZBTB49;ZBTB7A;ZBTB7B;ZBTB6;ZFP64;ZFP28;ZFP42;ZFP82;ZFX;ZIC1;ZIC2;ZIC3;ZIC4;ZIM3;ZKSCAN1;ZKSCAN3;ZNF121;ZNF136;ZNF140;ZNF143;ZNF148;ZNF214;ZNF232;ZNF250;ZNF257;ZNF260;ZNF263;ZNF264;ZNF274;ZNF281;ZNF282;ZNF317;ZNF320;ZNF322;ZNF329;ZNF331;ZNF333;ZN	MYNN;MZF1;OSR2;PRDM14;PRDM6;WT1;ZBTB14;ZBTB48;ZBTB49;ZFP64;ZFP28;ZIM3;ZNF121;ZNF250;ZNF257;ZNF263;ZNF274;ZNF317;ZNF320;ZNF329;ZNF331;ZNF394;ZNF449;ZNF502;ZNF528;ZNF547;ZNF549;ZNF554;ZNF586;ZNF589;ZNF667;ZNF680;ZNF708;ZNF713;ZNF768;ZNF18;ZNF85;ZSCAN16;ZSCAN22

motif	Nº of probes	% of probes	lower OR	upper OR	OR	p.value	FDR	TF family	TF subfamily	TF.family.member	TF.subfamily.member
										F350;ZNF384;ZNF394;ZNF410;ZNF436;ZNF449;ZNF490;ZNF502;ZNF524;ZNF528;ZNF547;ZNF549;ZNF554;ZNF563;ZNF582;ZNF586;ZNF589;ZNF652;ZNF667;ZNF680;ZNF708;ZNF713;ZNF768;ZNF816;ZNF18;ZNF41;ZNF76;ZNF85;ZSCAN16;ZSCAN22;ZSCAN31;ZSCAN4	
HXD12_HUMAN.H11MO.0.D	22	0.12865497	12.815.025	3.313.763	2,11E+06	2713,74900000	1,85E+04	HOX-related factors{3.1.1}	HOX9-13{3.1.1.8}	CDX1;CDX2;CDX4;EVX1;EVX2;GBX1;GBX2;GSX1;GSX2;HDX;HMBOX1;HNF1A;HNF1B;HOXA1;HOXA10;HOXA11;HOXA13;HOXA2;HOXA3;HOXA4;HOXA5;HOXA6;HOXA7;HOXA9;HOXB1;HOXB13;HOXB2;HOXB3;HOXB4;HOXB5;HOXB6;HOXB7;HOXB8;HOXB9;HOXC10;HOXC11;HOXC12;HOXC13;HOXC4;HOXC5;HOXC6;HOXC8;HOXC9;HOXD1;HOXD10;HOXD11;HOXD12;HOXD13;HOXD3;HOXD4;HOXD8;HOXD9;MEOX1;MEOX2;MNX1;PDX1;POU1F1;POU2F1;POU2F2;POU2F3;POU3F1;POU3F2;POU3F3;POU3F4;POU4F1;POU4F2;POU4F3;POU5F1;POU5F2;POU6F1;POU6F2	HOXA10;HOXA11;HOXA13;HOXA9;HOXB13;HOXC10;HOXC11;HOXC12;HOXC13;HOXC9;HOXD10;HOXD11;HOXD12;HOXD13;HOXD9
PO2F3_HUMAN.H11MO.0.D	18	0.10526316	12.700.581	3.600.719	2,20E+06	4216,71900000	2,73E+04	POU domain factors{3.1.10}	POU2 (Oct-1/2-like factors){3.1.10.2}	CDX1;CDX2;CDX4;EVX1;EVX2;GBX1;GBX2;GSX1;GSX2;HDX;HMBOX1;HNF1A;HNF1B;HOXA1;HOXA10;HOXA11;HOXA13;HOXA2;HOXA3;HOXA4;HOXA5;HOXA6;HOXA7;HOXA9;HOXB1;HOXB13;HOXB2;HOXB3;HOXB4;HOXB5;HOXB6;HOXB7;HOXB8;HOXB9;HOXC10;HOXC11;HOXC12;HOXC13;HOXC4;HOXC5;HOXC6;HOXC8;HOXC9;HOXD1;HOXD10;HOXD11;HOXD12;HOXD13;HOXD3;HOXD4;HOXD8;HOXD9;MEOX1;MEOX2;MNX1;PDX1;POU1F1;POU2F1;POU2F2;POU2F3;POU3F1;POU3F2;POU3F3;POU3F4;POU4F1;POU4F2;POU4F3;POU5F1;POU5F1B;POU5F2;POU6F1;POU6F2	POU2F1;POU2F2;POU2F3
PAX2_HUMAN.H11MO.0.D	20	0.11695906	12.561.525	3.388.281	2,12E+06	3134,25700000	2,12E+04	Paired domain only{3.2.2}	PAX-2-like factors (partial homeobox){3.2.2.2}	PAX1;PAX2;PAX5;PAX8;PAX9	PAX2;PAX5;PAX8
STAT2_HUMAN.H11MO.0.A	48	0.28070175	12.457.861	2.501.807	1,78E+06	1314,95200000	1,05E+04	STAT factors{6.2.1}	STAT2{6.2.1.0.2}	STAT1;STAT2;STAT3;STAT4;STAT5A;STAT5B;STAT6	STAT2
HXC12_HUMAN.H11MO.0.D	21	0.12280702	12.192.056	3.217.173	2,03E+06	4485,49100000	2,88E+04	HOX-related factors{3.1.1}	HOX9-13{3.1.1.8}	CDX1;CDX2;CDX4;EVX1;EVX2;GBX1;GBX2;GSX1;GSX2;HDX;HMBOX1;HNF1A;HNF1B;HOXA1;HOXA10;HOXA11;HOXA13;HOXA2;HOXA3;HOXA4;HOXA5;HOXA6;HOXA7;HOXA9;HOXB1;HOXB13;HOXB2;HOXB3;HOXB4;HOXB5;HOXB6;HOXB7;HOXB8;HOXB9;HOXC10;HOXC11;HOXC12;HOXC13;HOXC4;HOXC5;HOXC6;HOXC8;HOXC9;HOXD1;HOXD10;HOXD11;HOXD12;HOXD13;HOXD3;HOXD4;HOXD8;HOXD9;MEOX1;MEOX2;MNX1;PDX1;POU1F1;POU2F1;POU2F2;POU2F3;POU3F1;POU3F2;POU3F3;POU3F4;POU4F1;POU4F2;POU4F3;POU5F1;POU5F2;POU6F1;POU6F2	HOXA10;HOXA11;HOXA13;HOXA9;HOXB13;HOXC10;HOXC11;HOXC12;HOXC13;HOXC9;HOXD10;HOXD11;HOXD12;HOXD13;HOXD9
ETV6_HUMAN.H11MO.0.D	42	0.24561404	12.125.875	2.514.318	1,76E+06	2133,95500000	1,51E+04	Ets-related factors{3.5.2}	ETV6-like factors{3.5.2.6}	EHF;ELF1;ELF2;ELF3;ELF4;ELF5;ELK1;ELK3;ELK4;ERF;ERG;ETS1;ETS2;ETV1;ETV2;ETV3;ETV3L;ETV4;ETV5;ETV6;ETV7;FEV;FLI1;GABPA;SPDEF;SPI1;SPIB;SPIC	ETV6;ETV7
HLF_HUMAN.H11MO.0.C	17	0.09941520	12.103.804	3.530.567	2,13E+06	6595,08400000	4,04E+04	C/EBP-related{1.1.8}	PAR factors{1.1.8.2}	CEBPA;CEBPB;CEBPD;CEBPE;CEBPG;DBP;DDIT3;HLF;NFIL3;TEF	DBP;HLF;NFIL3;TEF

motif	Nº of probes	% of probes	lower OR	upper OR	OR	p.value	FDR	TF family	TF subfamily	TF.family.member	TF.subfamily.member
GATA4_HUMAN.H11MO.0.A	19	0.11111111	11.859.797	3.275.402	2,03E+06	7652,00700000	4,50E+04	GATA-type zinc fingers{2.2.1}	Two zinc-finger GATA factors{2.2.1.1}	GATA1;GATA2;GATA3;GATA4;GATA5;GATA6;GATAD2A;GATAD2B;TRPS1;ZGLP1	GATA1;GATA2;GATA3;GATA4;GATA5;GATA6
ZN260_HUMAN.H11MO.0.C	19	0.11111111	11.831.456	3.267.567	2,02E+06	7732,45000000	4,52E+04	More than 3 adjacent zinc finger factors{2.3.3}	ZNF146-like factors{2.3.3.55}	BCL6;BCL6B;CTCF;CTCFL;FEZF1;FEZF2;GFI1;GFI1B;GLI1;GLI2;GLI3;GLI4;GLIS1;GLIS2;GLIS3;HKR1;MTF1;MYNN;MZF1;OSR2;OVOL1;OVOL2;PLAG1;PLAGL1;PLAGL2;PRDM1;PRDM14;PRDM6;SCRT1;SCRT2;SNAI1;SNAI2;SNAI3;WT1;YY1;YY2;ZBTB12;ZBTB14;ZBTB18;ZBTB20;ZBTB26;ZBTB42;ZBTB45;ZBTB47;ZBTB48;ZBTB49;ZBTB6;ZBTB7A;ZBTB7B;ZBTB7C;ZFP14;ZFP2;ZFP28;ZFP30;ZFP37;ZFP42;ZFP64;ZFP69;ZFP69B;ZFP82;ZFP91;ZFX;ZIC1;ZIC2;ZIC3;ZIC4;ZIC5;ZIK1;ZIM3;ZKSCAN1;ZKSCAN2;ZKSCAN3;ZKSCAN4;ZNF121;ZNF124;ZNF133;ZNF136;ZNF138;ZNF14;ZNF140;ZNF143;ZNF146;ZNF148;ZNF155;ZNF157;ZNF160;ZNF169;ZNF175;ZNF177;ZNF18;ZNF180;ZNF181;ZNF2;ZNF20;ZNF212;ZNF213;ZNF214;ZNF221;ZNF222;ZNF223;ZNF224;ZNF225;ZNF226;ZNF227;ZNF229;ZNF230;ZNF232;ZNF233;ZNF234;ZNF235;ZNF24;ZNF25;ZNF250;ZNF257;ZNF26;ZNF260;ZNF263;ZNF264;ZNF268;ZNF274;ZNF276;ZNF28;ZNF280A;ZNF280B;ZNF280C;ZNF280D;ZNF281;ZNF282;ZNF283;ZNF284;ZNF285;ZNF286A;ZNF286B;ZNF3;ZNF30;ZNF300;ZNF302;ZNF317;ZNF32;ZNF320;ZNF322;ZNF324;ZNF324B;ZNF329;ZNF331;ZNF333;ZNF33A;ZNF33B;ZNF343;ZNF345;ZNF347;ZNF350;ZNF354A;ZNF354B;ZNF362;ZNF366;ZNF383;ZNF384;ZNF394;ZNF397;ZNF398;ZNF404;ZNF41;ZNF410;ZNF419;ZNF420;ZNF431;ZNF432;ZNF436;ZNF439;ZNF44;ZNF440;ZNF442;ZNF443;ZNF446;ZNF449;ZNF45;ZNF460;ZNF468;ZNF479;ZNF484;ZNF490;ZNF500;ZNF502;ZNF524;ZNF525;ZNF528;ZNF543;ZNF544;ZNF546;ZNF547;ZNF548;ZNF549;ZNF554;ZNF555;ZNF557;ZNF558;ZNF559;ZNF561;ZNF562;ZNF563;ZNF564;ZNF566;ZNF567;ZNF568;ZNF57;ZNF570;ZNF571;ZNF572;ZNF577;ZNF581;ZNF582;ZNF583;ZNF585A;ZNF586;ZNF589;ZNF595;ZNF599;ZNF600;ZNF605;ZNF607;ZNF611;ZNF613;ZNF614;ZNF615;ZNF616;ZNF619;ZNF620;ZNF621;ZNF625;ZNF627;ZNF649;ZNF652;ZNF653;ZNF665;ZNF667;ZNF669;ZNF670;ZNF672;ZNF679;ZNF680;ZNF683;ZNF689;ZNF692;ZNF701;ZNF705D;ZNF705E;ZNF705G;ZNF708;ZNF709;ZNF71;ZNF710;ZNF713;ZNF721;ZNF727;ZNF729;ZNF736;ZNF75A;ZNF75D;ZNF76;ZNF763;ZNF764;ZNF765;ZNF768;ZNF77;ZNF771;ZNF773;ZNF774;ZNF776;ZNF777;ZNF780A;ZNF780B;ZNF782;ZNF785;ZNF799;ZNF805;ZNF808;ZNF81;ZNF813;ZNF816;ZNF823;ZNF829;ZNF836;ZNF841;ZNF844;ZNF845;ZNF846;ZNF85;ZNF853;ZNF860;ZNF878;ZNF891;ZNF99;ZSCAN16;ZSCAN2;ZSCAN22;ZSCAN23;ZSCAN29;ZSCAN31;ZSCAN32;ZSCAN4;ZSCAN5A;ZSCAN5B;ZSCAN5C;ZSCAN9;ZXDA;ZXDB;ZXDC	ZNF146;ZNF260
FOXG1_HUMAN.H11MO.0.D	44	0.25730994	11.688.653	2.395.280	1,69E+06	4150,78700000	2,71E+04	Forkhead box (FOX) factors{3.3.1}	FOXG{3.3.1.7}	FOXA1;FOXA2;FOXA3;FOXB1;FOXB2;FOXC1;FOXC2;FOXD1;FOXD2;FOXD3;FOXD4;FOXD4L1;FOXD4L3;FOXD4L4;FOXD4L5;FOXD4L6;FOXE1;FOXE3;FOXF1;FOXF2;FOXG1;FOXH1;FOXI1;FOXI2;FOXI3;FOXJ1;FOXJ2;FOXJ3;FOXK1;FOXK2;FOXL1;FOXL2;FOXM1;FOXN1;FOXN2;FOXN3;FOXN4;FOXO1;FOXO3;FOXO4;F	FOXG1

motif	Nº of probes	% of probes	lower OR	upper OR	OR	p.value	FDR	TF family	TF subfamily	TF.family.member	TF.subfamily.member
										OXO6;FOXP1;FOXP2;FOXP3;FOXP4;FOXQ1;FOXR1;FOXR2;FOXS1	
NFAC2_HUMAN.H11MO.0.B	20	0.11695906	11.668.835	3.146.742	1,97E+06	7057,55600000	4,27E+04	NFAT-related factors {6.1.3}	NFATc2 (NFATp, NFAT1){6.1.3.0.2}	NFAT5;NFATC1;NFATC2;NFATC3;NFATC4	NFATC2
FOXL1_HUMAN.H11MO.0.D	38	0.22222222	11.581.884	2.467.204	1,71E+06	5940,43500000	3,73E+04	Forkhead box (FOX) factors {3.3.1}	FOXL{3.3.1.12}	FOXA1;FOXA2;FOXA3;FOXB1;FOXB2;FOXC1;FOXC2;FOXD1;FOXD2;FOXD3;FOXD4;FOXD4L1;FOXD4L3;FOXD4L4;FOXD4L5;FOXD4L6;FOXE1;FOXE3;FOXF1;FOXF2;FOXG1;FOXH1;FOXI1;FOXI2;FOXI3;FOXJ1;FOXJ2;FOXJ3;FOXK1;FOXK2;FOXL1;FOXL2;FOXM1;FOXN1;FOXN2;FOXN3;FOXN4;FOXO1;FOXO3;FOXO4;FOXO6;FOXP1;FOXP2;FOXP3;FOXP4;FOXQ1;FOXR1;FOXR2;FOXS1	FOXL1
CEBPA_HUMAN.H11MO.0.A	16	0.09356725	11.578.376	3.485.106	2,08E+06	9957,94300000	5,52E+04	C/EBP-related{1.1.8}	C/EBP{1.1.8.1}	CEBPA;CEBPB;CEBPD;CEBPE;CEBPG;DBP;DDIT3;HLF;NFIL3;TEF	CEBPA;CEBPB;CEBPD;CEBPE;CEBPG;DDIT3
MNX1_HUMAN.H11MO.0.D	24	0.14035088	11.576.543	2.886.477	1,87E+06	7090,63900000	4,27E+04	HOX-related factors {3.1.1}	MNX{3.1.1.13}	CDX1;CDX2;CDX4;EVX1;EVX2;GBX1;GBX2;GSX1;GSX2;HDX;HMBOX1;HNF1A;HNF1B;HOXA1;HOXA10;HOXA11;HOXA13;HOXA2;HOXA3;HOXA4;HOXA5;HOXA6;HOXA7;HOXA9;HOXB1;HOXB13;HOXB2;HOXB3;HOXB4;HOXB5;HOXB6;HOXB7;HOXB8;HOXB9;HOXC10;HOXC11;HOXC12;HOXC13;HOXC4;HOXC5;HOXC6;HOXC8;HOXC9;HOXD1;HOXD10;HOXD11;HOXD12;HOXD13;HOXD3;HOXD4;HOXD8;HOXD9;MEOX1;MEOX2;MNX1;PDX1;POU1F1;POU2F1;POU2F2;POU2F3;POU3F1;POU3F2;POU3F3;POU3F4;POU4F1;POU4F2;POU4F3;POU5F1;POU5F2;POU6F1;POU6F2	MNX1
NKX23_HUMAN.H11MO.0.D	18	0.10526316	11.448.423	3.245.033	1,98E+06	10987,90000000	6,00E+04	NK-related factors {3.1.2}	NK-4{3.1.2.17}	BARHL1;BARHL2;BARX1;BARX2;BSX;DBX1;DBX2;DLX1;DLX2;DLX3;DLX4;DLX5;DLX6;EMX1;EMX2;EN1;EN2;HHEX;HLX;HMX1;HMX2;HMX3;LBX1;LBX2;MSX1;MSX2;NANOG;NKX1-1;NKX1-2;NKX2-1;NKX2-2;NKX2-3;NKX2-4;NKX2-5;NKX2-6;NKX2-8;NKX3-1;NKX3-2;NKX6-1;NKX6-2;NKX6-3;NOTO;TLX1;TLX2;TLX3;VAX1;VAX2;VENTX	NKX2-3;NKX2-5

motif	Nº of probes	% of probes	lower OR	upper OR	OR	p.value	FDR	TF family	TF subfamily	TF.family.member	TF.subfamily.member
OZF_HUMAN.H11MO.0.C	19	0.11111111	11.431.895	3.157.212	1,95E+06	9293,33700000	5,27E+04	More than 3 adjacent zinc finger factors{2.3.3}	ZNF146-like factors{2.3.3.55}	BCL6;BCL6B;CTCF;CTCFL;FEZF1;FEZF2;GFI1;GFI1B;GLI1;GLI2;GLI3;GLI4;GLIS1;GLIS2;GLIS3;HKR1;MTF1;MYNN;MZF1;OSR2;OVOL1;OVOL2;PLAG1;PLAGL1;PLAGL2;PRDM1;PRDM14;PRDM6;SCRT1;SCRT2;SNAI1;SNAI2;SNAI3;WT1;YY1;YY2;ZBTB12;ZBTB14;ZBTB18;ZBTB20;ZBTB26;ZBTB42;ZBTB45;ZBTB47;ZBTB48;ZBTB49;ZBTB6;ZBTB7A;ZBTB7B;ZBTB7C;ZFP14;ZFP2;ZFP28;ZFP30;ZFP37;ZFP42;ZFP64;ZFP69;ZFP69B;ZFP82;ZFP91;ZFX;ZIC1;ZIC2;ZIC3;ZIC4;ZIC5;ZIK1;ZIM3;ZKSCAN1;ZKSCAN2;ZKSCAN3;ZKSCAN4;ZNF121;ZNF124;ZNF133;ZNF136;ZNF138;ZNF14;ZNF140;ZNF143;ZNF146;ZNF148;ZNF155;ZNF157;ZNF160;ZNF169;ZNF175;ZNF177;ZNF18;ZNF180;ZNF181;ZNF2;ZNF20;ZNF212;ZNF213;ZNF214;ZNF221;ZNF222;ZNF223;ZNF224;ZNF225;ZNF226;ZNF227;ZNF229;ZNF230;ZNF232;ZNF233;ZNF234;ZNF235;ZNF24;ZNF25;ZNF250;ZNF257;ZNF26;ZNF260;ZNF263;ZNF264;ZNF268;ZNF274;ZNF276;ZNF28;ZNF280A;ZNF280B;ZNF280C;ZNF280D;ZNF281;ZNF282;ZNF283;ZNF284;ZNF285;ZNF286A;ZNF286B;ZNF3;ZNF30;ZNF300;ZNF302;ZNF317;ZNF32;ZNF320;ZNF322;ZNF324;ZNF324B;ZNF329;ZNF331;ZNF333;ZNF33A;ZNF33B;ZNF343;ZNF345;ZNF347;ZNF350;ZNF354A;ZNF354B;ZNF362;ZNF366;ZNF383;ZNF384;ZNF394;ZNF397;ZNF398;ZNF404;ZNF41;ZNF410;ZNF419;ZNF420;ZNF431;ZNF432;ZNF436;ZNF439;ZNF44;ZNF440;ZNF442;ZNF443;ZNF446;ZNF449;ZNF45;ZNF460;ZNF468;ZNF479;ZNF484;ZNF490;ZNF500;ZNF502;ZNF524;ZNF525;ZNF528;ZNF543;ZNF544;ZNF546;ZNF547;ZNF548;ZNF549;ZNF554;ZNF555;ZNF557;ZNF558;ZNF559;ZNF561;ZNF562;ZNF563;ZNF564;ZNF566;ZNF567;ZNF568;ZNF57;ZNF570;ZNF571;ZNF572;ZNF577;ZNF581;ZNF582;ZNF583;ZNF585A;ZNF586;ZNF589;ZNF595;ZNF599;ZNF600;ZNF605;ZNF607;ZNF611;ZNF613;ZNF614;ZNF615;ZNF616;ZNF619;ZNF620;ZNF621;ZNF625;ZNF627;ZNF649;ZNF652;ZNF653;ZNF665;ZNF667;ZNF669;ZNF670;ZNF672;ZNF679;ZNF680;ZNF683;ZNF689;ZNF692;ZNF701;ZNF705D;ZNF705E;ZNF705G;ZNF708;ZNF709;ZNF71;ZNF710;ZNF713;ZNF721;ZNF727;ZNF729;ZNF736;ZNF75A;ZNF75D;ZNF76;ZNF763;ZNF764;ZNF765;ZNF768;ZNF77;ZNF771;ZNF773;ZNF774;ZNF776;ZNF777;ZNF780A;ZNF780B;ZNF782;ZNF785;ZNF799;ZNF805;ZNF808;ZNF81;ZNF813;ZNF816;ZNF823;ZNF829;ZNF836;ZNF841;ZNF844;ZNF845;ZNF846;ZNF85;ZNF853;ZNF860;ZNF878;ZNF891;ZNF99;ZSCAN16;ZSCAN2;ZSCAN22;ZSCAN23;ZSCAN29;ZSCAN31;ZSCAN32;ZSCAN4;ZSCAN5A;ZSCAN5B;ZSCAN5C;ZSCAN9;ZXDA;ZXDB;ZXDC	ZNF146;ZNF260
IRF1_HUMAN.H11MO.0.A	44	0.25730994	11.405.759	2.337.323	1,65E+06	6145,79100000	3,82E+04	Interferon-regulatory factors{3.5.3}	IRF-1{3.5.3.0.1}	IRF1;IRF2;IRF3;IRF4;IRF5;IRF6;IRF7;IRF8;IRF9	IRF1
DBP_HUMAN.H11MO.0.B	16	0.09356725	11.350.821	3.416.393	2,04E+06	11049,48000000	6,00E+04	C/EBP-related{1.1.8}	PAR factors{1.1.8.2}	CEBPA;CEBPB;CEBPD;CEBPE;CEBPG;DBP;DDIT3;HLF;NFIL3;TEF	DBP;HLF;NFIL3;TEF
IRF2_HUMAN.H11MO.0.A	39	0.22807018	11.321.291	2.394.124	1,66E+06	7240,36200000	4,33E+04	Interferon-regulatory factors{3.5.3}	IRF-2{3.5.3.0.2}	IRF1;IRF2;IRF3;IRF4;IRF5;IRF6;IRF7;IRF8;IRF9	IRF2
IRX3_HUMAN.H11MO.0.D	18	0.10526316	11.299.716	3.202.890	1,96E+06	11657,18000000	6,29E+04	TALE-type homeo domain factors{3.1.4}	IRX (Iroquois){3.1.4.1}	IRX1;IRX2;IRX3;IRX4;IRX5;IRX6;MEIS1;MEIS2;MEIS3;MKX;PBX1;PBX2;PBX3;PKNOX1;PKNOX2;TGIF1;TGIF2;TGIF2LX;TGIF2LY	IRX2;IRX3

motif	Nº of probes	% of probes	lower OR	upper OR	OR	p.value	FDR	TF family	TF subfamily	TF.family.member	TF.subfamily.member
NFAC1_HUMAN.H11MO.1.B	23	0.13450292	11.299.011	2.866.833	1,84E+06	9641,92200000	5,43E+04	NFAT-related factors{6.1.3}	NFATc1{6.1.3.0.1}	NFAT5;NFATC1;NFATC2;NFATC3;NFATC4	NFATC1
FOXJ3_HUMAN.H11MO.1.B	36	0.21052632	11.257.054	2.435.969	1,68E+06	10024,01000000	5,52E+04	Forkhead box (FOX) factors {3.3.1}	FOXJ{3.3.1.10}	FOXA1;FOXA2;FOXA3;FOXB1;FOXB2;FOXC1;FOXC2;FOXD1;FOXD2;FOXD3;FOXD4;FOXD4L1;FOXD4L3;FOXD4L4;FOXD4L5;FOXD4L6;FOXE1;FOXE3;FOXF1;FOXF2;FOXG1;FOXH1;FOXI1;FOXI2;FOXI3;FOXJ1;FOXJ2;FOXJ3;FOXK1;FOXK2;FOXL1;FOXL2;FOXM1;FOXN1;FOXN2;FOXN3;FOXN4;FOXO1;FOXO3;FOXO4;FOXO6;FOXP1;FOXP2;FOXP3;FOXP4;FOXQ1;FOXR1;FOXR2;FOXS1	FOXJ2;FOXJ3
FOXQ1_HUMAN.H11MO.0.C	22	0.12865497	11.020.757	2.849.186	1,81E+06	13004,78000000	6,77E+04	Forkhead box (FOX) factors {3.3.1}	FOXQ{3.3.1.17}	FOXA1;FOXA2;FOXA3;FOXB1;FOXB2;FOXC1;FOXC2;FOXD1;FOXD2;FOXD3;FOXD4;FOXD4L1;FOXD4L3;FOXD4L4;FOXD4L5;FOXD4L6;FOXE1;FOXE3;FOXF1;FOXF2;FOXG1;FOXH1;FOXI1;FOXI2;FOXI3;FOXJ1;FOXJ2;FOXJ3;FOXK1;FOXK2;FOXL1;FOXL2;FOXM1;FOXN1;FOXN3;FOXN4;FOXO1;FOXO3;FOXO4;FOXO6;FOXP1;FOXP2;FOXP3;FOXP4;FOXQ1;FOXR1;FOXR2;FOXS1	FOXQ1
NKX28_HUMAN.H11MO.0.C	13	0.07602339	10.996.336	3.721.252	2,11E+06	14287,35000000	7,28E+04	NK-related factors {3.1.2}	NK-2.2{3.1.2.15}	BARHL1;BARHL2;BARX1;BARX2;BSX;DBX1;DBX2;DLX1;DLX2;DLX3;DLX4;DLX5;DLX6;EMX1;EMX2;EN1;EN2;HHEX;HLX;HMX1;HMX2;HMX3;LBX1;LBX2;MSX1;MSX2;NANOG;NKX1-1;NKX1-2;NKX2-1;NKX2-2;NKX2-3;NKX2-4;NKX2-5;NKX2-6;NKX2-8;NKX3-1;NKX3-2;NKX6-1;NKX6-2;NKX6-3;NOTO;TLX1;TLX2;TLX3;VAX1;VAX2;VENTX	NKX2-2;NKX2-8
HXA1_HUMAN.H11MO.0.C	17	0.09941520	10.992.667	3.205.886	1,94E+06	15863,35000000	7,79E+04	HOX-related factors {3.1.1}	HOX1{3.1.1.1}	CDX1;CDX2;CDX4;EVX1;EVX2;GBX1;GBX2;GSX1;GSX2;HDX;HMBOX1;HNF1A;HNF1B;HOXA1;HOXA10;HOXA11;HOXA13;HOXA2;HOXA3;HOXA4;HOXA5;HOXA6;HOXA7;HOXA9;HOXB1;HOXB13;HOXB2;HOXB3;HOXB4;HOXB5;HOXB6;HOXB7;HOXB8;HOXB9;HOXC10;HOXC11;HOXC12;HOXC13;HOXC4;HOXC5;HOXC6;HOXC8;HOXC9;HOXD1;HOXD10;HOXD11;HOXD12;HOXD13;HOXD3;HOXD4;HOXD8;HOXD9;MEOX1;MEOX2;MNX1;PDX1;POU1F1;POU2F1;POU2F2;POU2F3;POU3F1;POU3F2;POU3F3;POU3F4;POU4F1;POU4F2;POU4F3;POU5F1;POU5F2;POU6F1;POU6F2	HOXA1;HOXB1
IRF9_HUMAN.H11MO.0.C	23	0.13450292	10.950.017	2.778.255	1,78E+06	15467,57000000	7,64E+04	Interferon-regulatory factors{3.5.3}	IRF-9 (ISGF-3gamma){3.5.3.0.9}	IRF1;IRF2;IRF3;IRF4;IRF5;IRF6;IRF7;IRF8;IRF9	IRF9
HOMEZ_HUMAN.H11MO.0.D	19	0.11111111	10.949.525	3.024.027	1,87E+06	16277,87000000	7,85E+04	HD-ZF factors{3.1.8}	ZHX{3.1.8.5}	ADNP;ADNP2;HOMEZ;NANOGNB;TSHZ1;TSHZ2;TSHZ3;ZEB1;ZEB2;ZFHX2;ZFHX3;ZFHX4;ZHX1;ZHX2;ZHX3	HOMEZ
IRF3_HUMAN.H11MO.0.B	56	0.32748538	10.882.326	2.118.681	1,53E+06	12057,72000000	6,46E+04	Interferon-regulatory factors{3.5.3}	IRF-3{3.5.3.0.3}	IRF1;IRF2;IRF3;IRF4;IRF5;IRF6;IRF7;IRF8;IRF9	IRF3
GATA2_HUMAN.H11MO.1.A	20	0.11695906	10.862.680	2.929.239	1,83E+06	14447,80000000	7,28E+04	GATA-type zinc fingers{2.2.1}	Two zinc-finger GATA factors{2.2.1.1}	GATA1;GATA2;GATA3;GATA4;GATA5;GATA6;GATAD2A;GATAD2B;TRPS1;ZGLP1	GATA1;GATA2;GATA3;GATA4;GATA5;GATA6
HXD11_HUMAN.H11MO.0.D	21	0.12280702	10.854.232	2.863.489	1,81E+06	16596,33000000	7,85E+04	HOX-related factors {3.1.1}	HOX9-13{3.1.1.8}	CDX1;CDX2;CDX4;EVX1;EVX2;GBX1;GBX2;GSX1;GSX2;HDX;HMBOX1;HNF1A;HNF1B;HOXA1;HOXA10;HOXA11;HOXA13;HOXA2;HOXA3;HOXA4;HOXA5;HOXA6;HOXA7;HOXA9;HOXB1;HOXB13;HOXB2;HOXB3;HOXB4;HOXB5;HOXB6;HOXB7;HOXB8;HOXB9;HOXC10;HOXC11;HOXC12;HOXC13;HOXC4;HOXC5;HOXC6;HOXC8;HOXC9;HOXD1;HOXD10;HOXD11;HOXD12;HOXD13;HOXD3;HOXD4;HOXD8;HOXD9;MEOX1;MEOX2;MNX1;PDX1;POU1F1;PO	HOXA10;HOXA11;HOXA13;HOXA9;HOXB13;HOXC10;HOXC11;HOXC12;HOXC13;HOXC9;HOXD10;HOXD11;HOXD12;HOXD13;HOXD9

motif	Nº of probes	% of probes	lower OR	upper OR	OR	p.value	FDR	TF family	TF subfamily	TF.family.member	TF.subfamily.member
										U2F1;POU2F2;POU2F3;POU3F1;POU3F2;POU3F3;POU3F4;POU4F1;POU4F2;POU4F3;POU5F1;POU5F2;POU6F1;POU6F2	
ELF3_HUMAN.H11MO.0.A	36	0.21052632	10.804.576	2.337.815	1,61E+06	15399,28000000	7,64E+04	Ets-related factors{3.5.2}	EHF-like factors{3.5.2.4}	EHF;ELF1;ELF2;ELF3;ELF4;ELF5;ELK1;ELK3;ELK4;ERF;ERG;ETS1;ETS2;ETV1;ETV2;ETV3;ETV3L;ETV4;ETV5;ETV6;ETV7;FEV;FLI1;GABPA;SPDEF;SPI1;SPIB;SPIC	EHF;ELF3;ELF5
FOXA1_HUMAN.H11MO.0.A	26	0.15204678	10.745.532	2.596.003	1,70E+06	18054,71000000	8,49E+04	Forkhead box (FOX) factors{3.3.1}	FOXA{3.3.1.1}	FOXA1;FOXA2;FOXA3;FOXB1;FOXB2;FOXC1;FOXC2;FOXD1;FOXD2;FOXD3;FOXD4;FOXD4L1;FOXD4L3;FOXD4L4;FOXD4L5;FOXD4L6;FOXE1;FOXE3;FOXF1;FOXF2;FOXG1;FOXH1;FOXI1;FOXI2;FOXI3;FOXJ1;FOXJ2;FOXJ3;FOXK1;FOXK2;FOXL1;FOXL2;FOXM1;FOXN1;FOXN2;FOXN3;FOXN4;FOXO1;FOXO3;FOXO4;FOXO6;FOXP1;FOXP2;FOXP3;FOXP4;FOXQ1;FOXR1;FOXR2;FOXS1	FOXA1;FOXA2;FOXA3
NKX32_HUMAN.H11MO.0.C	16	0.09356725	10.715.194	3.224.774	1,92E+06	21265,75000000	9,62E+04	NK-related factors{3.1.2}	NK-3{3.1.2.16}	BARHL1;BARHL2;BARX1;BARX2;BSX;DBX1;DBX2;DLX1;DLX2;DLX3;DLX4;DLX5;DLX6;EMX1;EMX2;EN1;EN2;HHEX;HLX;HMX1;HMX2;HMX3;LBX1;LBX2;MSX1;MSX2;NANOG;NKX1-1;NKX1-2;NKX2-1;NKX2-2;NKX2-3;NKX2-4;NKX2-5;NKX2-6;NKX2-8;NKX3-1;NKX3-2;NKX6-1;NKX6-2;NKX6-3;NOTO;TLX1;TLX2;TLX3;VAX1;VAX2;VENTX	NKX3-1;NKX3-2
BHE22_HUMAN.H11MO.0.D	15	0.08771930	10.548.971	3.287.118	1,93E+06	27846,52000000	1,14E+05	Tal-related factors{1.2.3}	Neurogenin / Atonal-like factors{1.2.3.4}	ATOH1;ATOH7;ATOH8;BHLHA15;BHLHA9;BHLHE22;BHLHE23;FERD3L;FIGLA;HAND1;HAND2;LYL1;MESP1;MESP2;MSC;MSGN1;NEUROD1;NEUROD2;NEUROD4;NEUROD6;NEUROG1;NEUROG2;NEUROG3;NHLH1;NHLH2;OLIG1;OLIG2;OLIG3;PTF1A;SCX;TAL1;TAL2;TCF15;TCF21;TCF23;TWIST1;TWIST2	ATOH1;BHLHA15;BHLHE22;BHLHE23;NEUROD1;NEUROD2;NEUROG2;OLIG1;OLIG2;OLIG3
BATF_HUMAN.H11MO.0.A	17	0.09941520	10.543.199	3.074.928	1,86E+06	19509,96000000	9,01E+04	B-ATF-related factors{1.1.4}	B-ATF{1.1.4.0.1}	BATF;BATF2;BATF3	BATF
NKX61_HUMAN.H11MO.0.B	17	0.09941520	10.518.659	3.067.764	1,85E+06	19769,61000000	9,07E+04	NK-related factors{3.1.2}	NK-6{3.1.2.19}	BARHL1;BARHL2;BARX1;BARX2;BSX;DBX1;DBX2;DLX1;DLX2;DLX3;DLX4;DLX5;DLX6;EMX1;EMX2;EN1;EN2;HHEX;HLX;HMX1;HMX2;HMX3;LBX1;LBX2;MSX1;MSX2;NANOG;NKX1-1;NKX1-2;NKX2-1;NKX2-2;NKX2-3;NKX2-4;NKX2-5;NKX2-6;NKX2-8;NKX3-1;NKX3-2;NKX6-1;NKX6-2;NKX6-3;NOTO;TLX1;TLX2;TLX3;VAX1;VAX2;VENTX	NKX6-1;NKX6-2
EOMES_HUMAN.H11MO.0.D	24	0.14035088	10.498.190	2.617.350	1,69E+06	21137,37000000	9,62E+04	TBrain-related factors{6.5.2}	TBR-2 (EOMES){6.5.2.0.2}	EOMES;TBR1;TBX21	EOMES
SRY_HUMAN.H11MO.0.B	26	0.15204678	10.483.145	2.532.672	1,66E+06	27058,79000000	1,12E+05	SOX-related factors{4.1.1}	Group A{4.1.1.1}	BBX;CIC;HBP1;SOX1;SOX10;SOX11;SOX12;SOX13;SOX14;SOX15;SOX17;SOX18;SOX2;SOX21;SOX3;SOX30;SOX4;SOX5;SOX6;SOX7;SOX8;SOX9;SRY	SRY
PIT1_HUMAN.H11MO.0.C	18	0.10526316	10.480.366	2.970.492	1,82E+06	23321,59000000	1,02E+05	POU domain factors{3.1.10}	POU1 (Pit-1-like factors){3.1.10.1}	CDX1;CDX2;CDX4;EVX1;EVX2;GBX1;GBX2;GSX1;GSX2;HDX;HMBOX1;HNF1A;HNF1B;HOXA1;HOXA10;HOXA11;HOXA13;HOXA2;HOXA3;HOXA4;HOXA5;HOXA6;HOXA7;HOXA9;HOXB1;HOXB13;HOXB2;HOXB3;HOXB4;HOXB5;HOXB6;HOXB7;HOXB8;HOXB9;HOXC10;HOXC11;HOXC12;HOXC13;HOXC4;HOXC5;HOXC6;HOXC8;HOXC9;HOXD1;HOXD10;HOXD11;HOXD12;HOXD13;HOXD3;HOXD4;HOXD8;HOXD9;MEOX1;MEOX2;MNX1;PDX1;POU1F1;POU2F1;POU2F2;POU2F3;POU3F1;POU3F2;POU3	POU1F1

motif	Nº of probes	% of probes	lower OR	upper OR	OR	p.value	FDR	TF family	TF subfamily	TF.family.member	TF.subfamily.member
										F3;POU3F4;POU4F1;POU4F2;POU4F3;POU5F1;POU5F1B;POU5F2;POU6F1;POU6F2	
GCR_HUMAN.H11MO.1.A	29	0.16959064	10.435.392	2.421.853	1,61E+06	28027,39000000	1,14E+05	Steroid hormone receptors (NR3){2.1.1}	GR-like receptors (NR3C){2.1.1.1}	AR;ESR1;ESR2;ESRRA;ESRRB;ESRRG;NR3C1;NR3C2;PGR	AR;NR3C1;NR3C2;PGR
SOX2_HUMAN.H11MO.1.A	21	0.12280702	10.423.946	2.749.906	1,73E+06	27272,80000000	1,12E+05	SOX-related factors {4.1.1}	Group B{4.1.1.2}	BBX;CIC;HBP1;SOX1;SOX10;SOX11;SOX12;SOX13;SOX14;SOX15;SOX17;SOX18;SOX2;SOX21;SOX3;SOX30;SOX4;SOX5;SOX6;SOX7;SOX8;SOX9;SRY	SOX1;SOX21;SOX2;SOX3
PO3F4_HUMAN.H11MO.0.D	19	0.11111111	10.416.626	2.876.707	1,78E+06	28459,49000000	1,15E+05	POU domain factors{3.1.10}	POU3 (Oct-6-like factors){3.1.10.3}	CDX1;CDX2;CDX4;EVX1;EVX2;GBX1;GBX2;GSX1;GSX2;HDX;HMBOX1;HNF1A;HNF1B;HOXA1;HOXA10;HOXA11;HOXA13;HOXA2;HOXA3;HOXA4;HOXA5;HOXA6;HOXA7;HOXA9;HOXB1;HOXB13;HOXB2;HOXB3;HOXB4;HOXB5;HOXB6;HOXB7;HOXB8;HOXB9;HOXC10;HOXC11;HOXC12;HOXC13;HOXC4;HOXC5;HOXC6;HOXC8;HOXC9;HOXD1;HOXD10;HOXD11;HOXD12;HOXD13;HOXD3;HOXD4;HOXD8;HOXD9;MEOX1;MEOX2;MNX1;PDX1;POU1F1;POU2F1;POU2F2;POU2F3;POU3F1;POU3F2;POU3F3;POU3F4;POU4F1;POU4F2;POU4F3;POU5F1;POU5F1B;POU5F2;POU6F1;POU6F2	POU3F1;POU3F2;POU3F3;POU3F4
RUNX2_HUMAN.H11MO.0.A	34	0.19883041	10.354.781	2.279.731	1,56E+06	25741,69000000	1,09E+05	Runt-related factors {6.4.1}	Runx2 (PEBP2alphaA, CBF-alpha1){6.4.1.0.1}	RUNX1;RUNX2;RUNX3	RUNX2
TEF_HUMAN.H11MO.0.D	19	0.11111111	10.338.521	2.855.132	1,77E+06	29045,68000000	1,17E+05	C/EBP-related{1.1.8}	PAR factors{1.1.8.2}	CEBPA;CEBPB;CEBPD;CEBPE;CEBPG;DBP;DDIT3;HLF;NFIL3;TEF	DBP;HLF;NFIL3;TEF
HXA11_HUMAN.H11MO.0.D	17	0.09941520	10.274.652	2.996.493	1,81E+06	30140,53000000	1,21E+05	HOX-related factors {3.1.1}	HOX9-13{3.1.1.8}	CDX1;CDX2;CDX4;EVX1;EVX2;GBX1;GBX2;GSX1;GSX2;HDX;HMBOX1;HNF1A;HNF1B;HOXA1;HOXA10;HOXA11;HOXA13;HOXA2;HOXA3;HOXA4;HOXA5;HOXA6;HOXA7;HOXA9;HOXB1;HOXB13;HOXB2;HOXB3;HOXB4;HOXB5;HOXB6;HOXB7;HOXB8;HOXB9;HOXC10;HOXC11;HOXC12;HOXC13;HOXC4;HOXC5;HOXC6;HOXC8;HOXC9;HOXD1;HOXD10;HOXD11;HOXD12;HOXD13;HOXD3;HOXD4;HOXD8;HOXD9;MEOX1;MEOX2;MNX1;PDX1;POU1F1;POU2F1;POU2F2;POU2F3;POU3F1;POU3F2;POU3F3;POU3F4;POU4F1;POU4F2;POU4F3;POU5F1;POU5F2;POU6F1;POU6F2	HOXA10;HOXA11;HOXA13;HOXA9;HOXB13;HOXC10;HOXC11;HOXC12;HOXC13;HOXC9;HOXD10;HOXD11;HOXD12;HOXD13;HOXD9
FOXP3_HUMAN.H11MO.0.D	16	0.09356725	10.213.760	3.073.823	1,83E+06	26034,64000000	1,10E+05	Forkhead box (FOX) factors {3.3.1}	FOXP{3.3.1.16}	FOXA1;FOXA2;FOXA3;FOXB1;FOXB2;FOXC1;FOXC2;FOXD1;FOXD2;FOXD3;FOXD4;FOXD4L1;FOXD4L3;FOXD4L4;FOXD4L5;FOXD4L6;FOXE1;FOXE3;FOXF1;FOXF2;FOXG1;FOXH1;FOXI1;FOXI2;FOXI3;FOXJ1;FOXJ2;FOXJ3;FOXK1;FOXK2;FOXL1;FOXL2;FOXM1;FOXN1;FOXN2;FOXN3;FOXN4;FOXO1;FOXO3;FOXO4;FOXO6;FOXP1;FOXP2;FOXP3;FOXP4;FOXQ1;FOXR1;FOXR2;FOXS1	FOXP1;FOXP2;FOXP3
ARI3A_HUMAN.H11MO.0.D	27	0.15789474	10.143.351	2.415.811	1,59E+06	33083,76000000	1,30E+05	ARID-related factors {3.7.1}	ARID3{3.7.1.3}	ARID2;ARID3A;ARID5A;ARID5B;KDM5B	ARID3A
FOXO3_HUMAN.H11MO.0.B	22	0.12865497	10.008.483	2.587.332	1,65E+06	35621,96000000	1,39E+05	Forkhead box (FOX) factors {3.3.1}	FOXO{3.3.1.15}	FOXA1;FOXA2;FOXA3;FOXB1;FOXB2;FOXC1;FOXC2;FOXD1;FOXD2;FOXD3;FOXD4;FOXD4L1;FOXD4L3;FOXD4L4;FOXD4L5;FOXD4L6;FOXE1;FOXE3;FOXF1;FOXF2;FOXG1;FOXH1;FOXI1;FOXI2;FOXI3;FOXJ1;FOXJ2;FOXJ3;FOXK1;FOXK2;FOXL1;FOXL2;FOXM1;FOXN1;FOXN2;FOXN3;FOXN4;FOXO1;FOXO3;FOXO4;F	FOXO1;FOXO3;FOXO4;FOXO6

motif	Nº of probes	% of probes	lower OR	upper OR	OR	p.value	FDR	TF family	TF subfamily	TF.family.member	TF.subfamily.member
										OXO6;FOXP1;FOXP2;FOXP3;FOXP4;FOXQ1;FOXR1;FOXR2;FOXS1	
GATA5_HUMAN.H11MO.0.D	14	0.08187135	10.003.451	3.240.290	1,87E+06	39601,43000000	1,50E+05	GATA-type zinc fingers{2.2.1}	Two zinc-finger GATA factors{2.2.1.1}	GATA1;GATA2;GATA3;GATA4;GATA5;GATA6;GATAD2A;GATAD2B;TRPS1;ZGLP1	GATA1;GATA2;GATA3;GATA4;GATA5;GATA6
FOXP1_HUMAN.H11MO.0.A	28	0.16374269	0.9998231	2.349.778	1,56E+06	38021,22000000	1,45E+05	Forkhead box (FOX) factors {3.3.1}	FOXP{3.3.1.16}	FOXA1;FOXA2;FOXA3;FOXB1;FOXB2;FOXC1;FOXC2;FOXD1;FOXD2;FOXD3;FOXD4;FOXD4L1;FOXD4L3;FOXD4L4;FOXD4L5;FOXD4L6;FOXE1;FOXE3;FOXF1;FOXF2;FOXG1;FOXH1;FOXI1;FOXI2;FOXI3;FOXJ1;FOXJ2;FOXJ3;FOXK1;FOXK2;FOXL1;FOXL2;FOXM1;FOXN1;FOXN2;FOXN3;FOXN4;FOXO1;FOXO3;FOXO4;FOXO6;FOXP1;FOXP2;FOXP3;FOXP4;FOXQ1;FOXR1;FOXR2;FOXS1	FOXP1;FOXP2;FOXP3
FOXP2_HUMAN.H11MO.0.C	21	0.12280702	0.9951963	2.625.315	1,66E+06	43674,41000000	1,60E+05	Forkhead box (FOX) factors {3.3.1}	FOXP{3.3.1.16}	FOXA1;FOXA2;FOXA3;FOXB1;FOXB2;FOXC1;FOXC2;FOXD1;FOXD2;FOXD3;FOXD4;FOXD4L1;FOXD4L3;FOXD4L4;FOXD4L5;FOXD4L6;FOXE1;FOXE3;FOXF1;FOXF2;FOXG1;FOXH1;FOXI1;FOXI2;FOXI3;FOXJ1;FOXJ2;FOXJ3;FOXK1;FOXK2;FOXL1;FOXL2;FOXM1;FOXN1;FOXN2;FOXN3;FOXN4;FOXO1;FOXO3;FOXO4;FOXO6;FOXP1;FOXP2;FOXP3;FOXP4;FOXQ1;FOXR1;FOXR2;FOXS1	FOXP1;FOXP2;FOXP3
ZN140_HUMAN.H11MO.0.C	21	0.12280702	0.9857794	2.600.623	1,64E+06	44646,72000000	1,61E+05	More than 3 adjacent zinc finger factors{2.3.3}	ZNF302-like factors{2.3.3.44}	BCL6;BCL6B;CTCF;CTCFL;FEZF1;FEZF2;GFI1;GFI1B;GLI1;GLI2;GLI3;GLI4;GLIS1;GLIS2;GLIS3;HKR1;MTF1;MYNN;MZF1;OSR2;OVOL1;OVOL2;PLAG1;PLAGL1;PLAGL2;PRDM1;PRDM14;PRDM6;SCRT1;SCRT2;SNAI1;SNAI2;SNAI3;WT1;YY1;YY2;ZBTB12;ZBTB14;ZBTB18;ZBTB20;ZBTB26;ZBTB42;ZBTB45;ZBTB47;ZBTB48;ZBTB49;ZBTB6;ZBTB7A;ZBTB7B;ZBTB7C;ZFP14;ZFP2;ZFP28;ZFP30;ZFP37;ZFP42;ZFP64;ZFP69;ZFP69B;ZFP82;ZFP91;ZFX;ZIC1;ZIC2;ZIC3;ZIC4;ZIC5;ZIK1;ZIM3;ZKSCAN1;ZKSCAN2;ZKSCAN3;ZKSCAN4;ZNF121;ZNF124;ZNF133;ZNF136;ZNF138;ZNF14;ZNF140;ZNF143;ZNF146;ZNF148;ZNF155;ZNF157;ZNF160;ZNF169;ZNF175;ZNF177;ZNF18;ZNF180;ZNF181;ZNF2;ZNF20;ZNF212;ZNF213;ZNF214;ZNF221;ZNF222;ZNF223;ZNF224;ZNF225;ZNF226;ZNF227;ZNF229;ZNF230;ZNF232;ZNF233;ZNF234;ZNF235;ZNF24;ZNF25;ZNF250;ZNF257;ZNF26;ZNF260;ZNF263;ZNF264;ZNF268;ZNF274;ZNF276;ZNF28;ZNF280A;ZNF280B;ZNF280C;ZNF280D;ZNF281;ZNF282;ZNF283;ZNF284;ZNF285;ZNF286A;ZNF286B;ZNF3;ZNF30;ZNF300;ZNF302;ZNF317;ZNF32;ZNF320;ZNF322;ZNF324;ZNF324B;ZNF329;ZNF331;ZNF333;ZNF33A;ZNF33B;ZNF343;ZNF345;ZNF347;ZNF350;ZNF354A;ZNF354B;ZNF362;ZNF366;ZNF383;ZNF384;ZNF394;ZNF397;ZNF398;ZNF404;ZNF41;ZNF410;ZNF419;ZNF420;ZNF431;ZNF432;ZNF436;ZNF439;ZNF44;ZNF440;ZNF442;ZNF443;ZNF446;ZNF449;ZNF45;ZNF460;ZNF468;ZNF479;ZNF484;ZNF490;ZNF500;ZNF502;ZNF524;ZNF525;ZNF528;ZNF543;ZNF544;ZNF546;ZNF547;ZNF548;ZNF549;ZNF554;ZNF555;ZNF557;ZNF558;ZNF559;ZNF561;ZNF562;ZNF563;ZNF564;ZNF566;ZNF567;ZNF568;ZNF57;ZNF570;ZNF571;ZNF572;ZNF577;ZNF581;ZNF582;ZNF583;ZNF585A;ZNF586;ZNF589;ZNF595;ZNF599;ZNF600;ZNF605;ZNF607;ZNF611;ZNF613;ZNF614;ZNF615;ZNF616;ZNF619;ZNF620;ZNF621;ZNF625;ZNF627;ZNF649;ZNF652;ZNF6	ZNF140

motif	Nº of probes	% of probes	lower OR	upper OR	OR	p.value	FDR	TF family	TF subfamily	TF.family.member	TF.subfamily.member
										53;ZNF665;ZNF667;ZNF669;ZNF670;ZNF672;ZNF679;ZNF680;ZNF683;ZNF689;ZNF692;ZNF701;ZNF705D;ZNF705E;ZNF705G;ZNF708;ZNF709;ZNF71;ZNF710;ZNF713;ZNF721;ZNF727;ZNF729;ZNF736;ZNF75A;ZNF75D;ZNF76;ZNF763;ZNF764;ZNF765;ZNF768;ZNF77;ZNF771;ZNF773;ZNF774;ZNF776;ZNF777;ZNF780A;ZNF780B;ZNF782;ZNF785;ZNF799;ZNF805;ZNF808;ZNF81;ZNF813;ZNF816;ZNF823;ZNF829;ZNF836;ZNF841;ZNF844;ZNF845;ZNF846;ZNF85;ZNF853;ZNF860;ZNF878;ZNF891;ZNF99;ZSCAN16;ZSCAN2;ZSCAN22;ZSCAN23;ZSCAN29;ZSCAN31;ZSCAN32;ZSCAN4;ZSCAN5A;ZSCAN5B;ZSCAN5C;ZSCAN9;ZXDA;ZXDB;ZXDC	
DUXA_HUMAN.H11MO.0.D	12	0.07017544	0.9806625	3.486.449	1,94E+06	39401,88000000	1,50E+05	Paired-related HD factors{3.1.3}	DUX{3.1.3.7}	ALX1;ALX3;ALX4;ARGFX;ARX;CRX;DMBX1;DPRX;DRGX;DUX4;DUXA;ESX1;GSC;GSC2;HESX1;ISX;LEUTX;MIXL1;NOBOX;OTP;OTX1;OTX2;PHOX2A;PHOX2B;PITX1;PITX2;PITX3;PROP1;PRRX1;PRRX2;RAX;RAX2;RHOXF1;RHOXF2;SEBOX;SHOX;SHOX2;TPRX1;UNCX;VSX1;VSX2	DUX4;DUXA
MEF2B_HUMAN.H11MO.0.A	25	0.14619883	0.9775388	2.397.805	1,56E+06	52848,02000000	1,83E+05	Regulators of differentiation {5.1.1}	MEF-2{5.1.1.1}	MEF2A;MEF2B;MEF2C;MEF2D	MEF2A;MEF2B;MEF2C;MEF2D
ETS1_HUMAN.H11MO.0.A	52	0.30409357	0.9766731	1.928.284	1,38E+06	59616,65000000	1,96E+05	Ets-related factors {3.5.2}	Ets-like factors{3.5.2.1}	EHF;ELF1;ELF2;ELF3;ELF4;ELF5;ELK1;ELK3;ELK4;ERF;ERG;ETS1;ETS2;ETV1;ETV2;ETV3;ETV3L;ETV4;ETV5;ETV6;ETV7;FEV;FLI1;GABPA;SPDEF;SPI1;SPIB;SPIC	ERG;ETS1;ETS2;ETV2;ETV3;FEV;FLI1;GABPA
CEBPD_HUMAN.H11MO.0.C	15	0.08771930	0.9763870	3.042.264	1,79E+06	36368,88000000	1,40E+05	C/EBP-related{1.1.8}	C/EBP{1.1.8.1}	CEBPA;CEBPB;CEBPD;CEBPE;CEBPG;DBP;DDIT3;HLF;NFIL3;TEF	CEBPA;CEBPB;CEBPD;CEBPE;CEBPG;DDIT3
FOXJ2_HUMAN.H11MO.0.C	28	0.16374269	0.9741936	2.289.471	1,52E+06	53016,75000000	1,83E+05	Forkhead box (FOX) factors {3.3.1}	FOXJ{3.3.1.10}	FOXA1;FOXA2;FOXA3;FOXB1;FOXB2;FOXC1;FOXC2;FOXD1;FOXD2;FOXD3;FOXD4;FOXD4L1;FOXD4L3;FOXD4L4;FOXD4L5;FOXD4L6;FOXE1;FOXE3;FOXF1;FOXF2;FOXG1;FOXH1;FOXI1;FOXI2;FOXI3;FOXJ1;FOXJ2;FOXJ3;FOXK1;FOXK2;FOXL1;FOXL2;FOXM1;FOXN1;FOXN2;FOXN3;FOXN4;FOXO1;FOXO3;FOXO4;FOXO6;FOXP1;FOXP2;FOXP3;FOXP4;FOXQ1;FOXR1;FOXR2;FOXS1	FOXJ2;FOXJ3
BARH1_HUMAN.H11MO.0.D	15	0.08771930	0.9739060	3.034.503	1,78E+06	36798,64000000	1,41E+05	NK-related factors {3.1.2}	BARHL{3.1.2.1}	BARHL1;BARHL2;BARX1;BARX2;BSX;DBX1;DBX2;DLX1;DLX2;DLX3;DLX4;DLX5;DLX6;EMX1;EMX2;EN1;EN2;HHEX;HLX;HMX1;HMX2;HMX3;LBX1;LBX2;MSX1;MSX2;NANOG;NKX1-1;NKX1-2;NKX2-1;NKX2-2;NKX2-3;NKX2-4;NKX2-5;NKX2-6;NKX2-8;NKX3-1;NKX3-2;NKX6-1;NKX6-2;NKX6-3;NOTO;TLX1;TLX2;TLX3;VAX1;VAX2;VENTX	BARHL1;BARHL2
MEOX2_HUMAN.H11MO.0.D	16	0.09356725	0.9717765	2.924.304	1,74E+06	43274,73000000	1,60E+05	HOX-related factors {3.1.1}	MEOX{3.1.1.14}	CDX1;CDX2;CDX4;EVX1;EVX2;GBX1;GBX2;GSX1;GSX2;HDX;HMBOX1;HNF1A;HNF1B;HOXA1;HOXA10;HOXA11;HOXA13;HOXA2;HOXA3;HOXA4;HOXA5;HOXA6;HOXA7;HOXA9;HOXB1;HOXB13;HOXB2;HOXB3;HOXB4;HOXB5;HOXB6;HOXB7;HOXB8;HOXB9;HOXC10;HO	MEOX1;MEOX2

motif	Nº of probes	% of probes	lower OR	upper OR	OR	p.value	FDR	TF family	TF subfamily	TF.family.member	TF.subfamily.member
										XC11;HOXC12;HOXC13;HOXC4;HOXC5;HOXC6;HOXC8;HOXC9;HOXD1;HOXD10;HOXD11;HOXD12;HOXD13;HOXD3;HOXD4;HOXD8;HOXD9;MEOX1;MEOX2;MNX1;PDX1;POU1F1;POU2F1;POU2F2;POU2F3;POU3F1;POU3F2;POU3F3;POU3F4;POU4F1;POU4F2;POU4F3;POU5F1;POU5F2;POU6F1;POU6F2	
CEBPG_HUMAN.H11MO.0.B	16	0.09356725	0.9713512	2.923.021	1,74E+06	43338,16000000	1,60E+05	C/EBP-related{1.1.8}	C/EBP{1.1.8.1}	CEBPA;CEBPB;CEBPD;CEBPE;CEBPG;DBP;DDIT3;HLF;NFIL3;TEF	CEBPA;CEBPB;CEBPD;CEBPE;CEBPG;DDIT3
PO5F1_HUMAN.H11MO.0.A	18	0.10526316	0.9655371	2.736.679	1,67E+06	43833,55000000	1,60E+05	POU domain factors{3.1.10}	POU5 (Oct-3/4-like factors){3.1.10.5}	CDX1;CDX2;CDX4;EVX1;EVX2;GBX1;GBX2;GSX1;GSX2;HDX;HMBOX1;HNF1A;HNF1B;HOXA1;HOXA10;HOXA11;HOXA13;HOXA2;HOXA3;HOXA4;HOXA5;HOXA6;HOXA7;HOXA9;HOXB1;HOXB13;HOXB2;HOXB3;HOXB4;HOXB5;HOXB6;HOXB7;HOXB8;HOXB9;HOXC10;HOXC11;HOXC12;HOXC13;HOXC4;HOXC5;HOXC6;HOXC8;HOXC9;HOXD1;HOXD10;HOXD11;HOXD12;HOXD13;HOXD3;HOXD4;HOXD8;HOXD9;MEOX1;MEOX2;MNX1;PDX1;POU1F1;POU2F1;POU2F2;POU2F3;POU3F1;POU3F2;POU3F3;POU3F4;POU4F1;POU4F2;POU4F3;POU5F1;POU5F1B;POU5F2;POU6F1;POU6F2	POU5F1B;POU5F1
PO3F3_HUMAN.H11MO.0.D	24	0.14035088	0.9637734	2.402.592	1,55E+06	49995,01000000	1,77E+05	POU domain factors{3.1.10}	POU3 (Oct-6-like factors){3.1.10.3}	CDX1;CDX2;CDX4;EVX1;EVX2;GBX1;GBX2;GSX1;GSX2;HDX;HMBOX1;HNF1A;HNF1B;HOXA1;HOXA10;HOXA11;HOXA13;HOXA2;HOXA3;HOXA4;HOXA5;HOXA6;HOXA7;HOXA9;HOXB1;HOXB13;HOXB2;HOXB3;HOXB4;HOXB5;HOXB6;HOXB7;HOXB8;HOXB9;HOXC10;HOXC11;HOXC12;HOXC13;HOXC4;HOXC5;HOXC6;HOXC8;HOXC9;HOXD1;HOXD10;HOXD11;HOXD12;HOXD13;HOXD3;HOXD4;HOXD8;HOXD9;MEOX1;MEOX2;MNX1;PDX1;POU1F1;POU2F1;POU2F2;POU2F3;POU3F1;POU3F2;POU3F3;POU3F4;POU4F1;POU4F2;POU4F3;POU5F1;POU5F1B;POU5F2;POU6F1;POU6F2	POU3F1;POU3F2;POU3F3;POU3F4
OLIG2_HUMAN.H11MO.0.B	39	0.22807018	0.9624329	2.035.254	1,41E+06	67707,38000000	2,10E+05	Tal-related factors{1.2.3}	Neurogenin / Atonal-like factors{1.2.3.4}	ATOH1;ATOH7;ATOH8;BHLHA15;BHLHA9;BHLHE22;BHLHE23;FERD3L;FIGLA;HAND1;HAND2;LYL1;MESP1;MESP2;MSC;MSGN1;NEUROD1;NEUROD2;NEUROD4;NEUROD6;NEUROG1;NEUROG2;NEUROG3;NHLH1;NHLH2;OLIG1;OLIG2;OLIG3;PTF1A;SCX;TAL1;TAL2;TCF15;TCF21;TCF23;TWIST1;TWIST2	ATOH1;BHLHA15;BHLHE22;BHLHE23;NEUROD1;NEUROD2;NEUROG2;OLIG1;OLIG2;OLIG3
BATF3_HUMAN.H11MO.0.B	18	0.10526316	0.9620338	2.726.689	1,67E+06	44488,29000000	1,61E+05	B-ATF-related factors{1.1.4}	B-ATF-3{1.1.4.0.3}	BATF;BATF2;BATF3	BATF3
SOX7_HUMAN.H11MO.0.D	14	0.08187135	0.9587900	3.105.639	1,80E+06	44723,21000000	1,61E+05	SOX-related factors{4.1.1}	Group F{4.1.1.6}	BBX;CIC;HBP1;SOX1;SOX10;SOX11;SOX12;SOX13;SOX14;SOX15;SOX17;SOX18;SOX2;SOX21;SOX3;SOX30;SOX4;SOX5;SOX6;SOX7;SOX8;SOX9;SRY	SOX17;SOX18;SOX7
ZN713_HUMAN.H11MO.0.D	31	0.18128655	0.9580219	2.173.080	1,46E+06	68703,99000000	2,11E+05	More than 3 adjacent zinc finger factors{2.3.3}	unclassified{2.3.3.0}	BCL6B;BCL6;CTCFL;CTCF;FEZF1;GFI1B;GFI1;GLI1;GLI2;GLI3;GLIS1;GLIS2;GLIS3;MTF1;MYNN;MZF1;OSR2;OVOL1;OVOL2;ZNF146;PLAG1;PLAGL1;PRDM14;PRDM1;PRDM6;SCRT1;SCRT2;SNAI1;SNAI2;YY1;YY2;WT1;ZNF324;ZNF354A;ZBTB14;ZBTB18;ZBTB48;ZBTB49;ZBTB7A;ZBTB7B;ZBTB6;ZFP64;ZFP28;ZFP42;ZFP82;ZFX;ZIC1;ZIC2;ZIC3;ZIC4;ZIM3;ZKSCAN1;ZKSCAN3;ZNF121;ZNF136;ZNF140;ZNF143;ZNF148;ZNF214;ZNF232;ZNF250;ZNF257;ZNF260;ZNF263;ZNF264;ZNF274;ZNF281;ZNF282;ZNF317;ZNF320;ZNF322;ZNF329;ZNF331;ZNF333;ZNF350;ZNF384;ZNF394;ZNF410;ZNF436;ZNF449;ZNF490;ZNF502;ZNF524;ZNF528;ZNF547;ZNF549;ZNF554;ZNF563;ZNF582;ZNF586;ZNF589;ZNF652;ZNF667;ZNF680;ZNF708;ZNF713;ZNF76	MYNN;MZF1;OSR2;PRDM14;PRDM6;WT1;ZBTB14;ZBTB48;ZBTB49;ZFP64;ZFP28;ZIM3;ZNF121;ZNF250;ZNF257;ZNF263;ZNF274;ZNF317;ZNF320;ZNF329;ZNF331;ZNF394;ZNF449;ZNF502;ZNF528;ZNF547;ZNF549;ZNF554;ZNF586;ZNF589;ZNF667;ZNF680;ZNF708;ZNF713;ZNF768;ZNF18;ZNF85;ZSCAN16;ZSCAN22

motif	Nº of probes	% of probes	lower OR	upper OR	OR	p.value	FDR	TF family	TF subfamily	TF.family.member	TF.subfamily.member
										8;ZNF816;ZNF18;ZNF41;ZNF76;ZNF85;ZSCAN16;ZSCAN22;ZSCAN31;ZSCAN4	
EHF_HUMAN.H11MO.0.B	33	0.19298246	0.9572456	2.127.378	1,45E+06	61931,56000000	2,01E+05	Ets-related factors{3.5.2}	EHF-like factors{3.5.2.4}	EHF;ELF1;ELF2;ELF3;ELF4;ELF5;ELK1;ELK3;ELK4;ERF;ERG;ETS1;ETS2;ETV1;ETV2;ETV3;ETV3L;ETV4;ETV5;ETV6;ETV7;FEV;FLI1;GABPA;SPDEF;SPI1;SPIB;SPIC	EHF;ELF3;ELF5
FOXO4_HUMAN.H11MO.0.C	20	0.11695906	0.9546078	2.574.111	1,61E+06	58430,31000000	1,95E+05	Forkhead box (FOX) factors{3.3.1}	FOXO{3.3.1.15}	FOXA1;FOXA2;FOXA3;FOXB1;FOXB2;FOXC1;FOXC2;FOXD1;FOXD2;FOXD3;FOXD4;FOXD4L1;FOXD4L3;FOXD4L4;FOXD4L5;FOXD4L6;FOXE1;FOXE3;FOXF1;FOXF2;FOXG1;FOXH1;FOXI1;FOXI2;FOXI3;FOXJ1;FOXJ2;FOXJ3;FOXK1;FOXK2;FOXL1;FOXL2;FOXM1;FOXN1;FOXN2;FOXN3;FOXN4;FOXO1;FOXO3;FOXO4;FOXO6;FOXP1;FOXP2;FOXP3;FOXP4;FOXQ1;FOXR1;FOXR2;FOXS1	FOXO1;FOXO3;FOXO4;FOXO6
CREB5_HUMAN.H11MO.0.D	20	0.11695906	0.9537668	2.571.774	1,61E+06	58550,65000000	1,95E+05	Jun-related factors{1.1.1}	ATF-2-like factors{1.1.1.3}	ATF2;ATF7;BACH1;BACH2;CREB5;JUN;JUNB;JUND;NFE2;NFE2L1;NFE2L2;NFE2L3	ATF2;ATF7;CREB5
ZFHX3_HUMAN.H11MO.0.D	16	0.09356725	0.9503304	2.859.791	1,71E+06	46980,80000000	1,67E+05	HD-ZF factors{3.1.8}	ZFHX{3.1.8.4}	ADNP;ADNP2;HOMEZ;NANOGNB;TSHZ1;TSHZ2;TSHZ3;ZEB1;ZEB2;ZFHX2;ZFHX3;ZFHX4;ZHX1;ZHX2;ZHX3	ZFHX3
CEBPB_HUMAN.H11MO.0.A	13	0.07602339	0.9488113	3.210.261	1,82E+06	55351,81000000	1,90E+05	C/EBP-related{1.1.8}	C/EBP{1.1.8.1}	CEBPA;CEBPB;CEBPD;CEBPE;CEBPG;DBP;DDIT3;HLF;NFIL3;TEF	CEBPA;CEBPB;CEBPD;CEBPE;CEBPG;DDIT3
ZN394_HUMAN.H11MO.0.C	30	0.17543860	0.9456436	2.169.011	1,45E+06	66373,10000000	2,07E+05	More than 3 adjacent zinc finger factors{2.3.3}	unclassified{2.3.3.0}	BCL6B;BCL6;CTCFL;CTCF;FEZF1;GFI1B;GFI1;GLI1;GLI2;GLI3;GLIS1;GLIS2;GLIS3;MTF1;MYNN;MZF1;OSR2;OVOL1;OVOL2;ZNF146;PLAG1;PLAGL1;PRDM14;PRDM1;PRDM6;SCRT1;SCRT2;SNAI1;SNAI2;YY1;YY2;WT1;ZNF324;ZNF354A;ZBTB14;ZBTB18;ZBTB48;ZBTB49;ZBTB7A;ZBTB7B;ZBTB6;ZFP64;ZFP28;ZFP42;ZFP82;ZFX;ZIC1;ZIC2;ZIC3;ZIC4;ZIM3;ZKSCAN1;ZKSCAN3;ZNF121;ZNF136;ZNF140;ZNF143;ZNF148;ZNF214;ZNF232;ZNF250;ZNF257;ZNF260;ZNF263;ZNF264;ZNF274;ZNF281;ZNF282;ZNF317;ZNF320;ZNF322;ZNF329;ZNF331;ZNF333;ZNF350;ZNF384;ZNF394;ZNF410;ZNF436;ZNF449;ZNF490;ZNF502;ZNF524;ZNF528;ZNF547;ZNF549;ZNF554;ZNF563;ZNF582;ZNF586;ZNF589;ZNF652;ZNF667;ZNF680;ZNF708;ZNF713;ZNF768;ZNF816;ZNF18;ZNF41;ZNF76;ZNF85;ZSCAN16;ZSCAN22;ZSCAN31;ZSCAN4	MYNN;MZF1;OSR2;PRDM14;PRDM6;WT1;ZBTB14;ZBTB48;ZBTB49;ZFP64;ZFP28;ZIM3;ZNF121;ZNF250;ZNF257;ZNF263;ZNF274;ZNF317;ZNF320;ZNF329;ZNF331;ZNF394;ZNF449;ZNF502;ZNF528;ZNF547;ZNF549;ZNF554;ZNF586;ZNF589;ZNF667;ZNF680;ZNF708;ZNF713;ZNF768;ZNF18;ZNF85;ZSCAN16;ZSCAN22
DDIT3_HUMAN.H11MO.0.D	17	0.09941520	0.9410651	2.744.465	1,66E+06	55801,67000000	1,90E+05	C/EBP-related{1.1.8}	C/EBP{1.1.8.1}	CEBPA;CEBPB;CEBPD;CEBPE;CEBPG;DBP;DDIT3;HLF;NFIL3;TEF	CEBPA;CEBPB;CEBPD;CEBPE;CEBPG;DDIT3
PO4F1_HUMAN.H11MO.0.D	18	0.10526316	0.9349046	2.649.708	1,62E+06	65049,38000000	2,06E+05	POU domain factors{3.1.10}	POU4 (Brn-3-like factors){3.1.10.4}	CDX1;CDX2;CDX4;EVX1;EVX2;GBX1;GBX2;GSX1;GSX2;HDX;HMBOX1;HNF1A;HNF1B;HOXA1;HOXA10;HOXA11;HOXA13;HOXA2;HOXA3;HOXA4;HOXA5;HOXA6;HOXA7;HOXA9;HOXB1;HOXB13;HOXB2;HOXB3;HOXB4;HOXB5;HOXB6;HOXB7;HOXB8;HOXB9;HOXC10;HOXC11;HOXC12;HOXC13;HOXC4;HOXC5;HOXC6;HOXC8;HOXC9;HOXD1;HOXD10;HOXD11;HOXD12;HOXD13;HOXD3;HOXD4;HOXD8;HOXD9;MEOX1;MEOX2;MNX1;PDX1;POU1F1;POU2F1;POU2F2;POU2F3;POU3F1;POU3F2;POU3	POU4F1;POU4F2;POU4F3

motif	Nº of probes	% of probes	lower OR	upper OR	OR	p.value	FDR	TF family	TF subfamily	TF.family.member	TF.subfamily.member
										F3;POU3F4;POU4F1;POU4F2;POU4F3;POU5F1;POU5F1B;POU5F2;POU6F1;POU6F2	
CDX1_HUMAN.H11MO.0.C	15	0.08771930	0.9346005	2.911.945	1,71E+06	57801,02000000	1,95E+05	HOX-related factors{3.1.1}	CDX (Caudal type homeobox){3.1.1.9}	CDX1;CDX2;CDX4;EVX1;EVX2;GBX1;GBX2;GSX1;GSX2;HDX;HMBOX1;HNF1A;HNF1B;HOXA1;HOXA10;HOXA11;HOXA13;HOXA2;HOXA3;HOXA4;HOXA5;HOXA6;HOXA7;HOXA9;HOXB1;HOXB13;HOXB2;HOXB3;HOXB4;HOXB5;HOXB6;HOXB7;HOXB8;HOXB9;HOXC10;HOXC11;HOXC12;HOXC13;HOXC4;HOXC5;HOXC6;HOXC8;HOXC9;HOXD1;HOXD10;HOXD11;HOXD12;HOXD13;HOXD3;HOXD4;HOXD8;HOXD9;MEOX1;MEOX2;MNX1;PDX1;POU1F1;POU2F1;POU2F2;POU2F3;POU3F1;POU3F2;POU3F3;POU3F4;POU4F1;POU4F2;POU4F3;POU5F1;POU5F2;POU6F1;POU6F2	CDX1;CDX2
OLIG3_HUMAN.H11MO.0.D	13	0.07602339	0.9317216	3.152.360	1,79E+06	57740,67000000	1,95E+05	Tal-related factors{1.2.3}	Neurogenin / Atonal-like factors{1.2.3.4}	ATOH1;ATOH7;ATOH8;BHLHA15;BHLHA9;BHLHE22;BHLHE23;FERD3L;FIGLA;HAND1;HAND2;LYL1;MESP1;MESP2;MSC;MSGN1;NEUROD1;NEUROD2;NEUROD4;NEUROD6;NEUROG1;NEUROG2;NEUROG3;NHLH1;NHLH2;OLIG1;OLIG2;OLIG3;PTF1A;SCX;TAL1;TAL2;TCF15;TCF21;TCF23;TWIST1;TWIST2	ATOH1;BHLHA15;BHLHE22;BHLHE23;NEUROD1;NEUROD2;NEUROG2;OLIG1;OLIG2;OLIG3
MAFB_HUMAN.H11MO.0.B	25	0.14619883	0.9308422	2.282.990	1,49E+06	77018,63000000	2,29E+05	Maf-related factors{1.1.3}	Large Maf factors{1.1.3.1}	MAF;MAFA;MAFB;MAFF;MAFG;MAFK;NRL	MAFA;MAFB;MAF;NRL
TF2LX_HUMAN.H11MO.0.D	20	0.11695906	0.9273853	2.500.365	1,56E+06	63671,89000000	2,05E+05	TALE-type homeo domain factors{3.1.4}	TGIF{3.1.4.6}	IRX1;IRX2;IRX3;IRX4;IRX5;IRX6;MEIS1;MEIS2;MEIS3;MKX;PBX1;PBX2;PBX3;PKNOX1;PKNOX2;TGIF1;TGIF2;TGIF2LX;TGIF2LY	TGIF2LX;TGIF1;TGIF2
TEAD3_HUMAN.H11MO.0.D	17	0.09941520	0.9266219	2.702.179	1,63E+06	58858,24000000	1,95E+05	TEF-1-related factors{3.6.1}	TEF-5 (TEAD-3, TEAD5){3.6.1.0.4}	TEAD1;TEAD2;TEAD3;TEAD4	TEAD3
ZNF41_HUMAN.H11MO.1.C	24	0.14035088	0.9260213	2.308.675	1,49E+06	72176,64000000	2,18E+05	More than 3 adjacent zinc finger factors{2.3.3}	ZFN81-like factors{2.3.3.68}	BCL6;BCL6B;CTCF;CTCFL;FEZF1;FEZF2;GFI1;GFI1B;GLI1;GLI2;GLI3;GLI4;GLIS1;GLIS2;GLIS3;HKR1;MTF1;MYNN;MZF1;OSR2;OVOL1;OVOL2;PLAG1;PLAGL1;PLAGL2;PRDM1;PRDM14;PRDM6;SCRT1;SCRT2;SNAI1;SNAI2;SNAI3;WT1;YY1;YY2;ZBTB12;ZBTB14;ZBTB18;ZBTB20;ZBTB26;ZBTB42;ZBTB45;ZBTB47;ZBTB48;ZBTB49;ZBTB6;ZBTB7A;ZBTB7B;ZBTB7C;ZFP14;ZFP2;ZFP28;ZFP30;ZFP37;ZFP42;ZFP64;ZFP69;ZFP69B;ZFP82;ZFP91;ZFX;ZIC1;ZIC2;ZIC3;ZIC4;ZIC5;ZIK1;ZIM3;ZKSCAN1;ZKSCAN2;ZKSCAN3;ZKSCAN4;ZNF121;ZNF124;ZNF133;ZNF136;ZNF138;ZNF14;ZNF140;ZNF143;ZNF146;ZNF148;ZNF155;ZNF157;ZNF160;ZNF169;ZNF175;ZNF177;ZNF18;ZNF180;ZNF181;ZNF2;ZNF20;ZNF212;ZNF213;ZNF214;ZNF221;ZNF222;ZNF223;ZNF224;ZNF225;ZNF226;ZNF227;ZNF229;ZNF230;ZNF232;ZNF233;ZNF234;ZNF235;ZNF24;ZNF25;ZNF250;ZNF257;ZNF26;ZNF260;ZNF263;ZNF264;ZNF268;ZNF274;ZNF276;ZNF28;ZNF280A;ZNF280B;ZNF280C;ZNF280D;ZNF281;ZNF282;ZNF283;ZNF284;ZNF285;ZNF286A;ZNF286B;ZNF3;ZNF30;ZNF300;ZNF302;ZNF317;ZNF32;ZNF320;ZNF322;ZNF324;ZNF324B;ZNF329;ZNF331;ZNF333;ZNF33A;ZNF33B;ZNF343;ZNF345;ZNF347;ZNF350;ZNF354A;ZNF354B;ZNF362;ZNF366;ZNF383;ZNF384;ZNF394;ZNF397;ZNF398;ZNF404;ZNF41;ZNF410;ZNF419;ZNF420;ZNF431;ZNF432;ZNF436;ZNF439;ZNF44;ZNF440	ZNF41

motif	Nº of probes	% of probes	lower OR	upper OR	OR	p.value	FDR	TF family	TF subfamily	TF.family.member	TF.subfamily.member
										;ZNF442;ZNF443;ZNF446;ZNF449;ZNF45;ZNF460;ZNF468;ZNF479;ZNF484;ZNF490;ZNF500;ZNF502;ZNF524;ZNF525;ZNF528;ZNF543;ZNF544;ZNF546;ZNF547;ZNF548;ZNF549;ZNF554;ZNF555;ZNF557;ZNF558;ZNF559;ZNF561;ZNF562;ZNF563;ZNF564;ZNF566;ZNF567;ZNF568;ZNF57;ZNF570;ZNF571;ZNF572;ZNF577;ZNF581;ZNF582;ZNF583;ZNF585A;ZNF586;ZNF589;ZNF595;ZNF599;ZNF600;ZNF605;ZNF607;ZNF611;ZNF613;ZNF614;ZNF615;ZNF616;ZNF619;ZNF620;ZNF621;ZNF625;ZNF627;ZNF649;ZNF652;ZNF653;ZNF665;ZNF667;ZNF669;ZNF670;ZNF672;ZNF679;ZNF680;ZNF683;ZNF689;ZNF692;ZNF701;ZNF705D;ZNF705E;ZNF705G;ZNF708;ZNF709;ZNF71;ZNF710;ZNF713;ZNF721;ZNF727;ZNF729;ZNF736;ZNF75A;ZNF75D;ZNF76;ZNF763;ZNF764;ZNF765;ZNF768;ZNF77;ZNF771;ZNF773;ZNF774;ZNF776;ZNF777;ZNF780A;ZNF780B;ZNF782;ZNF785;ZNF799;ZNF805;ZNF808;ZNF81;ZNF813;ZNF816;ZNF823;ZNF829;ZNF836;ZNF841;ZNF844;ZNF845;ZNF846;ZNF85;ZNF853;ZNF860;ZNF878;ZNF891;ZNF99;ZSCAN16;ZSCAN2;ZSCAN22;ZSCAN23;ZSCAN29;ZSCAN31;ZSCAN32;ZSCAN4;ZSCAN5A;ZSCAN5B;ZSCAN5C;ZSCAN9;ZXDA;ZXDB;ZXDC	
BARX1_HUMAN.H11MO.0.D	16	0.09356725	0.9214732	2.772.884	1,65E+06	70293,78000000	2,15E+05	NK-related factors {3.1.2}	BARX{3.1.2.2}	BARHL1;BARHL2;BARX1;BARX2;BSX;DBX1;DBX2;DLX1;DLX2;DLX3;DLX4;DLX5;DLX6;EMX1;EMX2;EN1;EN2;HHEX;HLX;HMX1;HMX2;HMX3;LBX1;LBX2;MSX1;MSX2;NANOG;NKX1-1;NKX1-2;NKX2-1;NKX2-2;NKX2-3;NKX2-4;NKX2-5;NKX2-6;NKX2-8;NKX3-1;NKX3-2;NKX6-1;NKX6-2;NKX6-3;NOTO;TLX1;TLX2;TLX3;VAX1;VAX2;VENTX	BARX1;BARX2
PROP1_HUMAN.H11MO.0.D	14	0.08187135	0.9202833	2.980.559	1,72E+06	72751,90000000	2,19E+05	Paired-related HD factors{3.1.3}	PROP{3.1.3.20}	ALX1;ALX3;ALX4;ARGFX;ARX;CRX;DMBX1;DPRX;DRGX;DUX4;DUXA;ESX1;GSC;GSC2;HESX1;ISX;LEUTX;MIXL1;NOBOX;OTP;OTX1;OTX2;PHOX2A;PHOX2B;PITX1;PITX2;PITX3;PROP1;PRRX1;PRRX2;RAX;RAX2;RHOXF1;RHOXF2;SEBOX;SHOX;SHOX2;TPRX1;UNCX;VSX1;VSX2	PROP1
CPEB1_HUMAN.H11MO.0.D	37	0.21637427	0.9197760	1.974.129	1,36E+06	101569,50000000	2,76E+05	*NA*	*NA*	CPEB1	CPEB1
HMX1_HUMAN.H11MO.0.D	16	0.09356725	0.9167239	2.758.534	1,64E+06	71128,61000000	2,16E+05	NK-related factors {3.1.2}	NK-5/HMX{3.1.2.18}	BARHL1;BARHL2;BARX1;BARX2;BSX;DBX1;DBX2;DLX1;DLX2;DLX3;DLX4;DLX5;DLX6;EMX1;EMX2;EN1;EN2;HHEX;HLX;HMX1;HMX2;HMX3;LBX1;LBX2;MSX1;MSX2;NANOG;NKX1-1;NKX1-2;NKX2-1;NKX2-2;NKX2-3;NKX2-4;NKX2-5;NKX2-6;NKX2-8;NKX3-1;NKX3-2;NKX6-1;NKX6-2;NKX6-3;NOTO;TLX1;TLX2;TLX3;VAX1;VAX2;VENTX	HMX1;HMX2;HMX3
SOX9_HUMAN.H11MO.1.B	15	0.08771930	0.9163197	2.854.996	1,68E+06	61493,78000000	2,01E+05	SOX-related factors {4.1.1}	Group E{4.1.1.5}	BBX;CIC;HBP1;SOX1;SOX10;SOX11;SOX12;SOX13;SOX14;SOX15;SOX17;SOX18;SOX2;SOX21;SOX3;SOX30;SOX4;SOX5;SOX6;SOX7;SOX8;SOX9;SRY	SOX10;SOX8;SOX9

motif	Nº of probes	% of probes	lower OR	upper OR	OR	p.value	FDR	TF family	TF subfamily	TF.family.member	TF.subfamily.member
PRDM6_HUMAN.H11MO.0.C	36	0.21052632	0.9056900	1.959.674	1,35E+06	121483,20000000	3,15E+05	More than 3 adjacent zinc finger factors{2.3.3}	unclassified{2.3.3.0}	BCL6B;BCL6;CTCFL;CTCF;FEZF1;GFI1B;GFI1;GLI1;GLI2;GLI3;GLIS1;GLIS2;GLIS3;MTF1;MYNN;MZF1;OSR2;OVOL1;OVOL2;ZNF146;PLAG1;PLAGL1;PRDM14;PRDM1;PRDM6;SCRT1;SCRT2;SNAI1;SNAI2;YY1;YY2;WT1;ZNF324;ZNF354A;ZBTB14;ZBTB18;ZBTB48;ZBTB49;ZBTB7A;ZBTB7B;ZBTB6;ZFP64;ZFP28;ZFP42;ZFP82;ZFX;ZIC1;ZIC2;ZIC3;ZIC4;ZIM3;ZKSCAN1;ZKSCAN3;ZNF121;ZNF136;ZNF140;ZNF143;ZNF148;ZNF214;ZNF232;ZNF250;ZNF257;ZNF260;ZNF263;ZNF264;ZNF274;ZNF281;ZNF282;ZNF317;ZNF320;ZNF322;ZNF329;ZNF331;ZNF333;ZNF350;ZNF384;ZNF394;ZNF410;ZNF436;ZNF449;ZNF490;ZNF502;ZNF524;ZNF528;ZNF547;ZNF549;ZNF554;ZNF563;ZNF582;ZNF586;ZNF589;ZNF652;ZNF667;ZNF680;ZNF708;ZNF713;ZNF768;ZNF816;ZNF18;ZNF41;ZNF76;ZNF85;ZSCAN16;ZSCAN22;ZSCAN31;ZSCAN4	MYNN;MZF1;OSR2;PRDM14;PRDM6;WT1;ZBTB14;ZBTB48;ZBTB49;ZFP64;ZFP28;ZIM3;ZNF121;ZNF250;ZNF257;ZNF263;ZNF274;ZNF317;ZNF320;ZNF329;ZNF331;ZNF394;ZNF449;ZNF502;ZNF528;ZNF547;ZNF549;ZNF554;ZNF586;ZNF589;ZNF667;ZNF680;ZNF708;ZNF713;ZNF768;ZNF18;ZNF85;ZSCAN16;ZSCAN22
PO2F1_HUMAN.H11MO.0.C	17	0.09941520	0.9044719	2.637.402	1,59E+06	84213,50000000	2,40E+05	POU domain factors{3.1.10}	POU2 (Oct-1/2-like factors){3.1.10.2}	CDX1;CDX2;CDX4;EVX1;EVX2;GBX1;GBX2;GSX1;GSX2;HDX;HMBOX1;HNF1A;HNF1B;HOXA1;HOXA10;HOXA11;HOXA13;HOXA2;HOXA3;HOXA4;HOXA5;HOXA6;HOXA7;HOXA9;HOXB1;HOXB13;HOXB2;HOXB3;HOXB4;HOXB5;HOXB6;HOXB7;HOXB8;HOXB9;HOXC10;HOXC11;HOXC12;HOXC13;HOXC4;HOXC5;HOXC6;HOXC8;HOXC9;HOXD1;HOXD10;HOXD11;HOXD12;HOXD13;HOXD3;HOXD4;HOXD8;HOXD9;MEOX1;MEOX2;MNX1;PDX1;POU1F1;POU2F1;POU2F2;POU2F3;POU3F1;POU3F2;POU3F3;POU3F4;POU4F1;POU4F2;POU4F3;POU5F1;POU5F1B;POU5F2;POU6F1;POU6F2	POU2F1;POU2F2;POU2F3
ETV4_HUMAN.H11MO.0.B	44	0.25730994	0.9040081	1.852.428	1,30E+06	132668,50000000	3,34E+05	Ets-related factors{3.5.2}	Elk-like factors{3.5.2.2}	EHF;ELF1;ELF2;ELF3;ELF4;ELF5;ELK1;ELK3;ELK4;ERF;ERG;ETS1;ETS2;ETV1;ETV2;ETV3;ETV3L;ETV4;ETV5;ETV6;ETV7;FEV;FLI1;GABPA;SPDEF;SPI1;SPIB;SPIC	ELK1;ELK3;ELK4;ETV1;ETV4;ETV5
ETV2_HUMAN.H11MO.0.B	47	0.27485380	0.9030316	1.821.819	1,29E+06	143458,40000000	3,55E+05	Ets-related factors{3.5.2}	Ets-like factors{3.5.2.1}	EHF;ELF1;ELF2;ELF3;ELF4;ELF5;ELK1;ELK3;ELK4;ERF;ERG;ETS1;ETS2;ETV1;ETV2;ETV3;ETV3L;ETV4;ETV5;ETV6;ETV7;FEV;FLI1;GABPA;SPDEF;SPI1;SPIB;SPIC	ERG;ETS1;ETS2;ETV2;ETV3;FEV;FLI1;GABPA
BACH1_HUMAN.H11MO.0.A	49	0.28654971	0.9005905	1.800.291	1,28E+06	150650,70000000	3,66E+05	Jun-related factors{1.1.1}	NF-E2-like factors{1.1.1.2}	ATF2;ATF7;BACH1;BACH2;CREB5;JUN;JUNB;JUND;NFE2;NFE2L1;NFE2L2;NFE2L3	BACH1;BACH2;NFE2L1;NFE2L2;NFE2
GATA6_HUMAN.H11MO.0.A	15	0.08771930	0.9003029	2.805.082	1,65E+06	65545,74000000	2,06E+05	GATA-type zinc fingers{2.2.1}	Two zinc-finger GATA factors{2.2.1.1}	GATA1;GATA2;GATA3;GATA4;GATA5;GATA6;GATAD2A;GATAD2B;TRPS1;ZGLP1	GATA1;GATA2;GATA3;GATA4;GATA5;GATA6
TAL1_HUMAN.H11MO.0.A	19	0.11111111	0.8979283	2.479.520	1,53E+06	81532,86000000	2,37E+05	Tal-related factors{1.2.3}	Tal / HEN-like factors{1.2.3.1}	ATOH1;ATOH7;ATOH8;BHLHA15;BHLHA9;BHLHE22;BHLHE23;FERD3L;FIGLA;HAND1;HAND2;LYL1;MESP1;MESP2;MSC;MSGN1;NEUROD1;NEUROD2;NEUROD4;NEUROD6;NEUROG1;NEUROG2;NEUROG3;NHLH1;NHLH2;OLIG1;OLIG2;OLIG3;PTF1A;SCX;TAL1;TAL2;TCF15;TCF21;TCF23;TWIST1;TWIST2	NHLH1;LYL1;TAL1
SOX1_HUMAN.H11MO.0.D	13	0.07602339	0.8978846	3.037.612	1,72E+06	64523,99000000	2,06E+05	SOX-related factors{4.1.1}	Group B{4.1.1.2}	BBX;CIC;HBP1;SOX1;SOX10;SOX11;SOX12;SOX13;SOX14;SOX15;SOX17;SOX18;SOX2;SOX21;SOX3;SOX30;SOX4;SOX5;SOX6;SOX7;SOX8;SOX9;SRY	SOX1;SOX21;SOX2;SOX3
PHX2B_HUMAN.H11MO.0.D	13	0.07602339	0.8954990	3.029.522	1,72E+06	65114,63000000	2,06E+05	Paired-related HD factors{3.1.3}	PHOX{3.1.3.18}	ALX1;ALX3;ALX4;ARGFX;ARX;CRX;DMBX1;DPRX;DRGX;DUX4;DUXA;ESX1;GSC;GSC2;HESX1;ISX;LEUTX;MIXL1;NOBOX;OTP;OTX1;OTX2;PHOX2A;PHOX2B;PITX1;PITX2;PITX3;PROP1;PRRX1;PRRX2;RAX;RAX2;RHOXF1;RHOXF2;SEBOX;SHOX;SHOX2;TPRX1;UNCX;VSX1;VSX2	PHOX2A;PHOX2B

motif	Nº of probes	% of probes	lower OR	upper OR	OR	p.value	FDR	TF family	TF subfamily	TF.family.member	TF.subfamily.member
ERG_HUMAN.H11MO.0.A	54	0.31578947	0.8897049	1.743.835	1,25E+07	0.16852320	0.3901844	Ets-related factors {3.5.2}	Ets-like factors{3.5.2.1}	EHF;ELF1;ELF2;ELF3;ELF4;ELF5;ELK1;ELK3;ELK4;ERF;ERG;ETS1;ETS2;ETV1;ETV2;ETV3;ETV3L;ETV4;ETV5;ETV6;ETV7;FEV;FLI1;GABPA;SPDEF;SPI1;SPIB;SPIC	ERG;ETS1;ETS2;ETV2;ETV3;FEV;FLI1;GABPA
LMX1B_HUMAN.H11MO.0.D	19	0.11111111	0.8883775	2.453.104	1,52E+07	0.10975835	0.2888181	HD-LIM factors{3.1.5}	Lmx{3.1.5.6}	ISL1;ISL2;LHX1;LHX2;LHX3;LHX4;LHX5;LHX6;LHX8;LHX9;LMX1A;LMX1B	LMX1A;LMX1B
SOX21_HUMAN.H11MO.0.D	13	0.07602339	0.8874779	3.002.870	1,70E+07	0.06721849	0.2089736	SOX-related factors {4.1.1}	Group B{4.1.1.2}	BBX;CIC;HBP1;SOX1;SOX10;SOX11;SOX12;SOX13;SOX14;SOX15;SOX17;SOX18;SOX2;SOX21;SOX3;SOX30;SOX4;SOX5;SOX6;SOX7;SOX8;SOX9;SRY	SOX1;SOX21;SOX2;SOX3
HXC10_HUMAN.H11MO.0.D	25	0.14619883	0.8865228	2.174.353	1,42E+07	0.10895479	0.2876854	HOX-related factors {3.1.1}	HOX9-13{3.1.1.8}	CDX1;CDX2;CDX4;EVX1;EVX2;GBX1;GBX2;GSX1;GSX2;HDX;HMBOX1;HNF1A;HNF1B;HOXA1;HOXA10;HOXA11;HOXA13;HOXA2;HOXA3;HOXA4;HOXA5;HOXA6;HOXA7;HOXA9;HOXB1;HOXB13;HOXB2;HOXB3;HOXB4;HOXB5;HOXB6;HOXB7;HOXB8;HOXB9;HOXC10;HOXC11;HOXC12;HOXC13;HOXC4;HOXC5;HOXC6;HOXC8;HOXC9;HOXD1;HOXD10;HOXD11;HOXD12;HOXD13;HOXD3;HOXD4;HOXD8;HOXD9;MEOX1;MEOX2;MNX1;PDX1;POU1F1;POU2F1;POU2F2;POU2F3;POU3F1;POU3F2;POU3F3;POU3F4;POU4F1;POU4F2;POU4F3;POU5F1;POU5F2;POU6F1;POU6F2	HOXA10;HOXA11;HOXA13;HOXA9;HOXB13;HOXC10;HOXC11;HOXC12;HOXC13;HOXC9;HOXD10;HOXD11;HOXD12;HOXD13;HOXD9
ZBT49_HUMAN.H11MO.0.D	11	0.06432749	0.8851127	3.329.714	1,81E+07	0.06364818	0.2045459	More than 3 adjacent zinc finger factors{2.3.3}	unclassified{2.3.3.0}	BCL6B;BCL6;CTCFL;CTCF;FEZF1;GFI1B;GFI1;GLI1;GLI2;GLI3;GLIS1;GLIS2;GLIS3;MTF1;MYNN;MZF1;OSR2;OVOL1;OVOL2;ZNF146;PLAG1;PLAGL1;PRDM14;PRDM1;PRDM6;SCRT1;SCRT2;SNAI1;SNAI2;YY1;YY2;WT1;ZNF324;ZNF354A;ZBTB14;ZBTB18;ZBTB48;ZBTB49;ZBTB7A;ZBTB7B;ZBTB6;ZFP64;ZFP28;ZFP42;ZFP82;ZFX;ZIC1;ZIC2;ZIC3;ZIC4;ZIM3;ZKSCAN1;ZKSCAN3;ZNF121;ZNF136;ZNF140;ZNF143;ZNF148;ZNF214;ZNF232;ZNF250;ZNF257;ZNF260;ZNF263;ZNF264;ZNF274;ZNF281;ZNF282;ZNF317;ZNF320;ZNF322;ZNF329;ZNF331;ZNF333;ZNF350;ZNF384;ZNF394;ZNF410;ZNF436;ZNF449;ZNF490;ZNF502;ZNF524;ZNF528;ZNF547;ZNF549;ZNF554;ZNF563;ZNF582;ZNF586;ZNF589;ZNF652;ZNF667;ZNF680;ZNF708;ZNF713;ZNF768;ZNF816;ZNF18;ZNF41;ZNF76;ZNF85;ZSCAN16;ZSCAN22;ZSCAN31;ZSCAN4	MYNN;MZF1;OSR2;PRDM14;PRDM6;WT1;ZBTB14;ZBTB48;ZBTB49;ZFP64;ZFP28;ZIM3;ZNF121;ZNF250;ZNF257;ZNF263;ZNF274;ZNF317;ZNF320;ZNF329;ZNF331;ZNF394;ZNF449;ZNF502;ZNF528;ZNF547;ZNF549;ZNF554;ZNF586;ZNF589;ZNF667;ZNF680;ZNF708;ZNF713;ZNF768;ZNF18;ZNF85;ZSCAN16;ZSCAN22
FOXJ3_HUMAN.H11MO.0.A	28	0.16374269	0.8835680	2.076.532	1,38E+07	0.13082077	0.3306978	Forkhead box (FOX) factors {3.3.1}	FOXJ{3.3.1.10}	FOXA1;FOXA2;FOXA3;FOXB1;FOXB2;FOXC1;FOXC2;FOXD1;FOXD2;FOXD3;FOXD4;FOXD4L1;FOXD4L3;FOXD4L4;FOXD4L5;FOXD4L6;FOXE1;FOXE3;FOXF1;FOXF2;FOXG1;FOXH1;FOXI1;FOXI2;FOXI3;FOXJ1;FOXJ2;FOXJ3;FOXK1;FOXK2;FOXL1;FOXL2;FOXM1;FOXN1;FOXN2;FOXN3;FOXN4;FOXO1;FOXO3;FOXO4;FOXO6;FOXP1;FOXP2;FOXP3;FOXP4;FOXQ1;FOXR1;FOXR2;FOXS1	FOXJ2;FOXJ3
FOXD1_HUMAN.H11MO.0.D	16	0.09356725	0.8824821	2.655.380	1,58E+07	0.07959392	0.2351223	Forkhead box (FOX) factors {3.3.1}	FOXD{3.3.1.4}	FOXA1;FOXA2;FOXA3;FOXB1;FOXB2;FOXC1;FOXC2;FOXD1;FOXD2;FOXD3;FOXD4;FOXD4L1;FOXD4L3;FOXD4L4;FOXD4L5;FOXD4L6;FOXE1;FOXE3;FOXF1;FOXF2;FOXG1;FOXH1;FOXI1;FOXI2;FOXI3;FOXJ1;FOXJ2;FOXJ3;FOXK1;FOXK2;FOXL1;FOXL2;FOXM1;FOXN1;FOXN2;FOXN3;FOXN4;FOXO1;FOXO3;FOXO4;FOXO6;FOXP1;FOXP2;FOXP3;FOXP4;FOXQ1;FOXR1;FOXR2;FOXS1	FOXD1;FOXD2;FOXD3
TBR1_HUMAN.H11MO.0.D	19	0.11111111	0.8792039	2.427.719	1,50E+07	0.11143831	0.2912506	TBrain-related factors {6.5.2}	TBR-1{6.5.2.0.1}	EOMES;TBR1;TBX21	TBR1

motif	Nº of probes	% of probes	lower OR	upper OR	OR	p.value	FDR	TF family	TF subfamily	TF.family.member	TF.subfamily.member
NKX62_HUMAN.H11MO.0.D	14	0.08187135	0.8770522	2.840.661	1,64E+07	0.08128290	0.2372145	NK-related factors {3.1.2}	NK-6{3.1.2.19}	BARHL1;BARHL2;BARX1;BARX2;BSX;DBX1;DBX2;DLX1;DLX2;DLX3;DLX4;DLX5;DLX6;EMX1;EMX2;EN1;EN2;HHEX;HLX;HMX1;HMX2;HMX3;LBX1;LBX2;MSX1;MSX2;NANOG;NKX1-1;NKX1-2;NKX2-1;NKX2-2;NKX2-3;NKX2-4;NKX2-5;NKX2-6;NKX2-8;NKX3-1;NKX3-2;NKX6-1;NKX6-2;NKX6-3;NOTO;TLX1;TLX2;TLX3;VAX1;VAX2;VENTX	NKX6-1;NKX6-2
ZKSC1_HUMAN.H11MO.0.B	31	0.18128655	0.8743900	1.983.248	1,34E+07	0.15352996	0.3722377	More than 3 adjacent zinc finger factors{2.3.3}	ZNF24-like factors{2.3.3.10}	BCL6;BCL6B;CTCF;CTCFL;FEZF1;FEZF2;GFI1;GFI1B;GLI1;GLI2;GLI3;GLI4;GLIS1;GLIS2;GLIS3;HKR1;MTF1;MYNN;MZF1;OSR2;OVOL1;OVOL2;PLAG1;PLAGL1;PLAGL2;PRDM1;PRDM14;PRDM6;SCRT1;SCRT2;SNAI1;SNAI2;SNAI3;WT1;YY1;YY2;ZBTB12;ZBTB14;ZBTB18;ZBTB20;ZBTB26;ZBTB42;ZBTB45;ZBTB47;ZBTB48;ZBTB49;ZBTB6;ZBTB7A;ZBTB7B;ZBTB7C;ZFP14;ZFP2;ZFP28;ZFP30;ZFP37;ZFP42;ZFP64;ZFP69;ZFP69B;ZFP82;ZFP91;ZFX;ZIC1;ZIC2;ZIC3;ZIC4;ZIC5;ZIK1;ZIM3;ZKSCAN1;ZKSCAN2;ZKSCAN3;ZKSCAN4;ZNF121;ZNF124;ZNF133;ZNF136;ZNF138;ZNF14;ZNF140;ZNF143;ZNF146;ZNF148;ZNF155;ZNF157;ZNF160;ZNF169;ZNF175;ZNF177;ZNF18;ZNF180;ZNF181;ZNF2;ZNF20;ZNF212;ZNF213;ZNF214;ZNF221;ZNF222;ZNF223;ZNF224;ZNF225;ZNF226;ZNF227;ZNF229;ZNF230;ZNF232;ZNF233;ZNF234;ZNF235;ZNF24;ZNF25;ZNF250;ZNF257;ZNF26;ZNF260;ZNF263;ZNF264;ZNF268;ZNF274;ZNF276;ZNF28;ZNF280A;ZNF280B;ZNF280C;ZNF280D;ZNF281;ZNF282;ZNF283;ZNF284;ZNF285;ZNF286A;ZNF286B;ZNF3;ZNF30;ZNF300;ZNF302;ZNF317;ZNF32;ZNF320;ZNF322;ZNF324;ZNF324B;ZNF329;ZNF331;ZNF333;ZNF33A;ZNF33B;ZNF343;ZNF345;ZNF347;ZNF350;ZNF354A;ZNF354B;ZNF362;ZNF366;ZNF383;ZNF384;ZNF394;ZNF397;ZNF398;ZNF404;ZNF41;ZNF410;ZNF419;ZNF420;ZNF431;ZNF432;ZNF436;ZNF439;ZNF44;ZNF440;ZNF442;ZNF443;ZNF446;ZNF449;ZNF45;ZNF460;ZNF468;ZNF479;ZNF484;ZNF490;ZNF500;ZNF502;ZNF524;ZNF525;ZNF528;ZNF543;ZNF544;ZNF546;ZNF547;ZNF548;ZNF549;ZNF554;ZNF555;ZNF557;ZNF558;ZNF559;ZNF561;ZNF562;ZNF563;ZNF564;ZNF566;ZNF567;ZNF568;ZNF57;ZNF570;ZNF571;ZNF572;ZNF577;ZNF581;ZNF582;ZNF583;ZNF585A;ZNF586;ZNF589;ZNF595;ZNF599;ZNF600;ZNF605;ZNF607;ZNF611;ZNF613;ZNF614;ZNF615;ZNF616;ZNF619;ZNF620;ZNF621;ZNF625;ZNF627;ZNF649;ZNF652;ZNF653;ZNF665;ZNF667;ZNF669;ZNF670;ZNF672;ZNF679;ZNF680;ZNF683;ZNF689;ZNF692;ZNF701;ZNF705D;ZNF705E;ZNF705G;ZNF708;ZNF709;ZNF71;ZNF710;ZNF713;ZNF721;ZNF727;ZNF729;ZNF736;ZNF75A;ZNF75D;ZNF76;ZNF763;ZNF764;ZNF765;ZNF768;ZNF77;ZNF771;ZNF773;ZNF774;ZNF776;ZNF777;ZNF780A;ZNF780B;ZNF782;ZNF785;ZNF799;ZNF805;ZNF808;ZNF81;ZNF813;ZNF816;ZNF823;ZNF829;ZNF836;ZNF841;ZNF844;ZNF845;ZNF846;ZNF85;ZNF853;ZNF860;ZNF878;ZNF891;ZNF99;ZSCAN16;ZSCAN2;ZSCAN22;ZSCAN23;ZSCAN29;ZSCAN31;ZSCAN32;ZSCAN4;ZSCAN5A;ZSCAN5B;ZSCAN5C;ZSCAN9;ZXDA;ZXDB;ZXDC	ZKSCAN1;ZNF232;ZSCAN31
SPIB_HUMAN.H11MO.0.A	33	0.19298246	0.8730605	1.940.147	1,32E+07	0.16666399	0.3884681	Ets-related factors {3.5.2}	Spi-like factors{3.5.2.5}	EHF;ELF1;ELF2;ELF3;ELF4;ELF5;ELK1;ELK3;ELK4;ERF;ERG;ETS1;ETS2;ETV1;ETV2;ETV3;	SPI1;SPIB;SPIC

motif	Nº of probes	% of probes	lower OR	upper OR	OR	p.value	FDR	TF family	TF subfamily	TF.family.member	TF.subfamily.member
										ETV3L;ETV4;ETV5;ETV6;ETV7;FEV;FLI1;GABPA;SPDEF;SPI1;SPIB;SPIC	
HXD8_HUMAN.H11MO.0.D	14	0.08187135	0.8725028	2.825.911	1,63E+07	0.08254946	0.2383732	HOX-related factors{3.1.1}	HOX8{3.1.1.7}	CDX1;CDX2;CDX4;EVX1;EVX2;GBX1;GBX2;GSX1;GSX2;HDX;HMBOX1;HNF1A;HNF1B;HOXA1;HOXA10;HOXA11;HOXA13;HOXA2;HOXA3;HOXA4;HOXA5;HOXA6;HOXA7;HOXA9;HOXB1;HOXB13;HOXB2;HOXB3;HOXB4;HOXB5;HOXB6;HOXB7;HOXB8;HOXB9;HOXC10;HOXC11;HOXC12;HOXC13;HOXC4;HOXC5;HOXC6;HOXC8;HOXC9;HOXD1;HOXD10;HOXD11;HOXD12;HOXD13;HOXD3;HOXD4;HOXD8;HOXD9;MEOX1;MEOX2;MNX1;PDX1;POU1F1;POU2F1;POU2F2;POU2F3;POU3F1;POU3F2;POU3F3;POU3F4;POU4F1;POU4F2;POU4F3;POU5F1;POU5F2;POU6F1;POU6F2	HOXB8;HOXC8;HOXD8
IRF8_HUMAN.H11MO.0.B	32	0.18713450	0.8708749	1.954.642	1,32E+07	0.16093358	0.3824694	Interferon-regulatory factors{3.5.3}	IRF-8 (ICSBP1){3.5.3.0.8}	IRF1;IRF2;IRF3;IRF4;IRF5;IRF6;IRF7;IRF8;IRF9	IRF8
CLOCK_HUMAN.H11MO.0.C	41	0.23976608	0.8700268	1.815.189	1,27E+07	0.18058515	0.4107633	PAS domain factors{1.2.5}	Arnt-like factors{1.2.5.2}	AHR;AHRR;ARNT;ARNT2;ARNTL;ARNTL2;CLOCK;EPAS1;HIF1A;HIF3A;NCOA1;NCOA2;NCOA3;NPAS1;NPAS2;NPAS3;NPAS4;SIM1;SIM2;SOHLH1;SOHLH2;TCFL5	ARNT2;ARNT;ARNTL;CLOCK
NFAC3_HUMAN.H11MO.0.B	17	0.09941520	0.8697044	2.536.015	1,53E+07	0.09319348	0.2612806	NFAT-related factors{6.1.3}	NFATc3 (NFATx){6.1.3.0.3}	NFAT5;NFATC1;NFATC2;NFATC3;NFATC4	NFATC3
ZBED1_HUMAN.H11MO.0.D	21	0.12280702	0.8648874	2.281.406	1,44E+07	0.13639819	0.3405957	BED zinc finger factors{2.3.5}	ZBED1 (ALTE, DREF, TRAMP){2.3.5.0.1}	ZBED1;ZBED2;ZBED3;ZBED4;ZBED5;ZBED6	ZBED1
TEAD4_HUMAN.H11MO.0.A	20	0.11695906	0.8637232	2.328.792	1,45E+07	0.12597105	0.3237456	TEF-1-related factors{3.6.1}	TEF-3 (TEAD-4, TCF-13L1){3.6.1.0.2}	TEAD1;TEAD2;TEAD3;TEAD4	TEAD4
HMX2_HUMAN.H11MO.0.D	12	0.07017544	0.8634598	3.069.601	1,71E+07	0.08338796	0.2393109	NK-related factors{3.1.2}	NK-5/HMX{3.1.2.18}	BARHL1;BARHL2;BARX1;BARX2;BSX;DBX1;DBX2;DLX1;DLX2;DLX3;DLX4;DLX5;DLX6;EMX1;EMX2;EN1;EN2;HHEX;HLX;HMX1;HMX2;HMX3;LBX1;LBX2;MSX1;MSX2;NANOG;NKX1-1;NKX1-2;NKX2-1;NKX2-2;NKX2-3;NKX2-4;NKX2-5;NKX2-6;NKX2-8;NKX3-1;NKX3-2;NKX6-1;NKX6-2;NKX6-3;NOTO;TLX1;TLX2;TLX3;VAX1;VAX2;VENTX	HMX1;HMX2;HMX3
MSX1_HUMAN.H11MO.0.D	14	0.08187135	0.8608559	2.788.141	1,61E+07	0.08614665	0.2432933	NK-related factors{3.1.2}	MSX{3.1.2.11}	BARHL1;BARHL2;BARX1;BARX2;BSX;DBX1;DBX2;DLX1;DLX2;DLX3;DLX4;DLX5;DLX6;EMX1;EMX2;EN1;EN2;HHEX;HLX;HMX1;HMX2;HMX3;LBX1;LBX2;MSX1;MSX2;NANOG;NKX1-1;NKX1-2;NKX2-1;NKX2-2;NKX2-3;NKX2-4;NKX2-5;NKX2-6;NKX2-8;NKX3-1;NKX3-2;NKX6-1;NKX6-2;NKX6-3;NOTO;TLX1;TLX2;TLX3;VAX1;VAX2;VENTX	MSX1;MSX2
TCF7_HUMAN.H11MO.0.A	18	0.10526316	0.8586296	2.433.451	1,49E+07	0.10692374	0.2842697	TCF-7-related factors{4.1.3}	TCF-7 (TCF-1) [1]{4.1.3.0.1}	LEF1;TAF1;TCF7;TCF7L1;TCF7L2	TCF7
DLX3_HUMAN.H11MO.0.C	13	0.07602339	0.8574526	2.900.856	1,65E+07	0.10115427	0.2757397	NK-related factors{3.1.2}	DLX{3.1.2.5}	BARHL1;BARHL2;BARX1;BARX2;BSX;DBX1;DBX2;DLX1;DLX2;DLX3;DLX4;DLX5;DLX6;EMX1;EMX2;EN1;EN2;HHEX;HLX;HMX1;HMX2;HMX3;LBX1;LBX2;MSX1;MSX2;NANOG;NKX1-1;NKX1-2;NKX2-1;NKX2-2;NKX2-3;NKX2-4;NKX2-5;NKX2-6;NKX2-8;NKX3-1;NKX3-2;NKX6-1;NKX6-2;NKX6-3;NOTO;TLX1;TLX2;TLX3;VAX1;VAX2;VENTX	DLX1;DLX2;DLX3;DLX4;DLX5;DLX6
PITX3_HUMAN.H11MO.0.D	12	0.07017544	0.8550028	3.039.437	1,69E+07	0.08567818	0.2428598	Paired-related HD factors{3.1.3}	PITX{3.1.3.19}	ALX1;ALX3;ALX4;ARGFX;ARX;CRX;DMBX1;DPRX;DRGX;DUX4;DUXA;ESX1;GSC;GSC2;HESX1;ISX;LEUTX;MIXL1;NOBOX;OTP;OTX1;OTX2;PHOX2A;PHOX2B;PITX1;PITX2;PITX3;P	PITX1;PITX2;PITX3

motif	Nº of probes	% of probes	lower OR	upper OR	OR	p.value	FDR	TF family	TF subfamily	TF.family.member	TF.subfamily.member
										ROP1;PRRX1;PRRX2;RAX;RAX2;RHOXF1;RHOXF2;SEBOX;SHOX;SHOX2;TPRX1;UNCX;VSX1;VSX2	
ELF2_HUMAN.H11MO.0.C	48	0.28070175	0.8545678	1.715.904	1,22E+07	0.24610329	0.4803687	Ets-related factors{3.5.2}	Elf-1-like factors{3.5.2.3}	EHF;ELF1;ELF2;ELF3;ELF4;ELF5;ELK1;ELK3;ELK4;ERF;ERG;ETS1;ETS2;ETV1;ETV2;ETV3;ETV3L;ETV4;ETV5;ETV6;ETV7;FEV;FLI1;GABPA;SPDEF;SPI1;SPIB;SPIC	ELF1;ELF2
NR1I3_HUMAN.H11MO.1.D	13	0.07602339	0.8509300	2.878.772	1,63E+07	0.10269562	0.2768473	Thyroid hormone receptor-related factors (NR1){2.1.2}	Vitamin D receptor (NR1I){2.1.2.4}	NR1D1;NR1D2;NR1H2;NR1H3;NR1H4;NR1I2;NR1I3;PPARA;PPARD;PPARG;RARA;RARB;RARG;RORA;RORB;RORC;THRA;THRB;VDR	NR1I2;NR1I3;VDR
NR1I2_HUMAN.H11MO.1.D	13	0.07602339	0.8509300	2.878.772	1,63E+07	0.10269562	0.2768473	Thyroid hormone receptor-related factors (NR1){2.1.2}	Vitamin D receptor (NR1I){2.1.2.4}	NR1D1;NR1D2;NR1H2;NR1H3;NR1H4;NR1I2;NR1I3;PPARA;PPARD;PPARG;RARA;RARB;RARG;RORA;RORB;RORC;THRA;THRB;VDR	NR1I2;NR1I3;VDR
NKX31_HUMAN.H11MO.0.C	15	0.08771930	0.8506184	2.649.991	1,56E+07	0.10144118	0.2757397	NK-related factors{3.1.2}	NK-3{3.1.2.16}	BARHL1;BARHL2;BARX1;BARX2;BSX;DBX1;DBX2;DLX1;DLX2;DLX3;DLX4;DLX5;DLX6;EMX1;EMX2;EN1;EN2;HHEX;HLX;HMX1;HMX2;HMX3;LBX1;LBX2;MSX1;MSX2;NANOG;NKX1-1;NKX1-2;NKX2-1;NKX2-2;NKX2-3;NKX2-4;NKX2-5;NKX2-6;NKX2-8;NKX3-1;NKX3-2;NKX6-1;NKX6-2;NKX6-3;NOTO;TLX1;TLX2;TLX3;VAX1;VAX2;VENTX	NKX3-1;NKX3-2
STAT1_HUMAN.H11MO.1.A	40	0.23391813	0.8482798	1.781.436	1,24E+07	0.24812576	0.4816176	STAT factors{6.2.1}	STAT1{6.2.1.0.1}	STAT1;STAT2;STAT3;STAT4;STAT5A;STAT5B;STAT6	STAT1
CRX_HUMAN.H11MO.0.B	13	0.07602339	0.8479183	2.868.574	1,63E+07	0.10346473	0.2779488	Paired-related HD factors{3.1.3}	OTX{3.1.3.17}	ALX1;ALX3;ALX4;ARGFX;ARX;CRX;DMBX1;DPRX;DRGX;DUX4;DUXA;ESX1;GSC;GSC2;HESX1;ISX;LEUTX;MIXL1;NOBOX;OTP;OTX1;OTX2;PHOX2A;PHOX2B;PITX1;PITX2;PITX3;PROP1;PRRX1;PRRX2;RAX;RAX2;RHOXF1;RHOXF2;SEBOX;SHOX;SHOX2;TPRX1;UNCX;VSX1;VSX2	CRX;OTX1;OTX2
SPI1_HUMAN.H11MO.0.A	35	0.20467836	0.8474513	1.849.062	1,27E+07	0.21982847	0.4482521	Ets-related factors{3.5.2}	Spi-like factors{3.5.2.5}	EHF;ELF1;ELF2;ELF3;ELF4;ELF5;ELK1;ELK3;ELK4;ERF;ERG;ETS1;ETS2;ETV1;ETV2;ETV3;ETV3L;ETV4;ETV5;ETV6;ETV7;FEV;FLI1;GABPA;SPDEF;SPI1;SPIB;SPIC	SPI1;SPIB;SPIC
BC11A_HUMAN.H11MO.0.A	40	0.23391813	0.8462584	1.777.189	1,24E+07	0.24861711	0.4816176	Factors with multiple dispersed zinc fingers{2.3.4}	BCL11{2.3.4.15}	BCL11A;BCL11B;BNC1;BNC2;E4F1;HIC1;HIC2;HINFP;HIVEP1;HIVEP2;HIVEP3;IKZF1;IKZF2;IKZF3;IKZF4;IKZF5;INSM1;INSM2;MAZ;MECOM;PATZ1;PRDM16;PRDM4;REST;RLF;RREB1;SALL1;SALL2;SALL3;SALL4;VEZF1;ZBTB1;ZBTB17;ZBTB2;ZBTB25;ZBTB4;ZFAT;ZNF134;ZNF211;ZNF217;ZNF219;ZNF248;ZNF256;ZNF292;ZNF296;ZNF319;ZNF334;ZNF335;ZNF341;ZNF37A;ZNF382;ZNF417;ZNF418;ZNF423;ZNF467;ZNF510;ZNF512;ZNF512B;ZNF516;ZNF518A;ZNF518B;ZNF521;ZNF526;ZNF532;ZNF536;ZNF552;ZNF574;ZNF587;ZNF587B;ZNF592;ZNF639;ZNF654;ZNF658;ZNF671;ZNF687;ZNF711;ZNF717;ZNF770;ZNF772;ZNF784;ZNF786;ZNF792;ZNF8;ZNF814	BCL11A
HXC8_HUMAN.H11MO.0.D	11	0.06432749	0.8412025	3.164.617	1,72E+07	0.10514406	0.2805054	HOX-related factors{3.1.1}	HOX8{3.1.1.7}	CDX1;CDX2;CDX4;EVX1;EVX2;GBX1;GBX2;GSX1;GSX2;HDX;HMBOX1;HNF1A;HNF1B;HOXA1;HOXA10;HOXA11;HOXA13;HOXA2;HOXA3;HOXA4;HOXA5;HOXA6;HOXA7;HOXA9;HOXB1;HOXB13;HOXB2;HOXB3;HOXB4;HOXB5;HOXB6;HOXB7;HOXB8;HOXB9;HOXC10;HOXC11;HOXC12;HOXC13;HOXC4;HOXC5;HOXC6;HOXC8;HOXC9;HOXD1;HOXD10;HOXD11;HOXD12;HOXD13;HOXD3;HOXD4;HOXD8;HOXD9;MEOX1;MEOX2;MNX1;PDX1;POU1F1;POU2F1;POU2F2;POU2F3;POU3F1;POU3F2;POU3F3;POU3F4;POU4F1;POU4F2;POU4F3;POU5F1;POU5F2;POU6F1;POU6F2	HOXB8;HOXC8;HOXD8

motif	Nº of probes	% of probes	lower OR	upper OR	OR	p.value	FDR	TF family	TF subfamily	TF.family.member	TF.subfamily.member
ZNF41_HUMAN.H11MO.0.C	28	0.16374269	0.8388565	1.971.254	1,31E+07	0.20987183	0.4413491	More than 3 adjacent zinc finger factors{2.3.3}	ZFN81-like factors{2.3.3.68}	BCL6;BCL6B;CTCF;CTCFL;FEZF1;FEZF2;GFI1;GFI1B;GLI1;GLI2;GLI3;GLI4;GLIS1;GLIS2;GLIS3;HKR1;MTF1;MYNN;MZF1;OSR2;OVOL1;OVOL2;PLAG1;PLAGL1;PLAGL2;PRDM1;PRDM14;PRDM6;SCRT1;SCRT2;SNAI1;SNAI2;SNAI3;WT1;YY1;YY2;ZBTB12;ZBTB14;ZBTB18;ZBTB20;ZBTB26;ZBTB42;ZBTB45;ZBTB47;ZBTB48;ZBTB49;ZBTB6;ZBTB7A;ZBTB7B;ZBTB7C;ZFP14;ZFP2;ZFP28;ZFP30;ZFP37;ZFP42;ZFP64;ZFP69;ZFP69B;ZFP82;ZFP91;ZFX;ZIC1;ZIC2;ZIC3;ZIC4;ZIC5;ZIK1;ZIM3;ZKSCAN1;ZKSCAN2;ZKSCAN3;ZKSCAN4;ZNF121;ZNF124;ZNF133;ZNF136;ZNF138;ZNF14;ZNF140;ZNF143;ZNF146;ZNF148;ZNF155;ZNF157;ZNF160;ZNF169;ZNF175;ZNF177;ZNF18;ZNF180;ZNF181;ZNF2;ZNF20;ZNF212;ZNF213;ZNF214;ZNF221;ZNF222;ZNF223;ZNF224;ZNF225;ZNF226;ZNF227;ZNF229;ZNF230;ZNF232;ZNF233;ZNF234;ZNF235;ZNF24;ZNF25;ZNF250;ZNF257;ZNF26;ZNF260;ZNF263;ZNF264;ZNF268;ZNF274;ZNF276;ZNF28;ZNF280A;ZNF280B;ZNF280C;ZNF280D;ZNF281;ZNF282;ZNF283;ZNF284;ZNF285;ZNF286A;ZNF286B;ZNF3;ZNF30;ZNF300;ZNF302;ZNF317;ZNF32;ZNF320;ZNF322;ZNF324;ZNF324B;ZNF329;ZNF331;ZNF333;ZNF33A;ZNF33B;ZNF343;ZNF345;ZNF347;ZNF350;ZNF354A;ZNF354B;ZNF362;ZNF366;ZNF383;ZNF384;ZNF394;ZNF397;ZNF398;ZNF404;ZNF41;ZNF410;ZNF419;ZNF420;ZNF431;ZNF432;ZNF436;ZNF439;ZNF44;ZNF440;ZNF442;ZNF443;ZNF446;ZNF449;ZNF45;ZNF460;ZNF468;ZNF479;ZNF484;ZNF490;ZNF500;ZNF502;ZNF524;ZNF525;ZNF528;ZNF543;ZNF544;ZNF546;ZNF547;ZNF548;ZNF549;ZNF554;ZNF555;ZNF557;ZNF558;ZNF559;ZNF561;ZNF562;ZNF563;ZNF564;ZNF566;ZNF567;ZNF568;ZNF57;ZNF570;ZNF571;ZNF572;ZNF577;ZNF581;ZNF582;ZNF583;ZNF585A;ZNF586;ZNF589;ZNF595;ZNF599;ZNF600;ZNF605;ZNF607;ZNF611;ZNF613;ZNF614;ZNF615;ZNF616;ZNF619;ZNF620;ZNF621;ZNF625;ZNF627;ZNF649;ZNF652;ZNF653;ZNF665;ZNF667;ZNF669;ZNF670;ZNF672;ZNF679;ZNF680;ZNF683;ZNF689;ZNF692;ZNF701;ZNF705D;ZNF705E;ZNF705G;ZNF708;ZNF709;ZNF71;ZNF710;ZNF713;ZNF721;ZNF727;ZNF729;ZNF736;ZNF75A;ZNF75D;ZNF76;ZNF763;ZNF764;ZNF765;ZNF768;ZNF77;ZNF771;ZNF773;ZNF774;ZNF776;ZNF777;ZNF780A;ZNF780B;ZNF782;ZNF785;ZNF799;ZNF805;ZNF808;ZNF81;ZNF813;ZNF816;ZNF823;ZNF829;ZNF836;ZNF841;ZNF844;ZNF845;ZNF846;ZNF85;ZNF853;ZNF860;ZNF878;ZNF891;ZNF99;ZSCAN16;ZSCAN2;ZSCAN22;ZSCAN23;ZSCAN29;ZSCAN31;ZSCAN32;ZSCAN4;ZSCAN5A;ZSCAN5B;ZSCAN5C;ZSCAN9;ZXDA;ZXDB;ZXDC	ZNF41

motif	Nº of probes	% of probes	lower OR	upper OR	OR	p.value	FDR	TF family	TF subfamily	TF.family.member	TF.subfamily.member
BCL6_HUMAN.H11MO.0.A	21	0.12280702	0.8333169	2.198.027	1,39E+07	0.18265890	0.4129912	More than 3 adjacent zinc finger factors{2.3.3}	BCL6 factors{2.3.3.22}	BCL6;BCL6B;CTCF;CTCFL;FEZF1;FEZF2;GFI1;GFI1B;GLI1;GLI2;GLI3;GLI4;GLIS1;GLIS2;GLIS3;HKR1;MTF1;MYNN;MZF1;OSR2;OVOL1;OVOL2;PLAG1;PLAGL1;PLAGL2;PRDM1;PRDM14;PRDM6;SCRT1;SCRT2;SNAI1;SNAI2;SNAI3;WT1;YY1;YY2;ZBTB12;ZBTB14;ZBTB18;ZBTB20;ZBTB26;ZBTB42;ZBTB45;ZBTB47;ZBTB48;ZBTB49;ZBTB6;ZBTB7A;ZBTB7B;ZBTB7C;ZFP14;ZFP2;ZFP28;ZFP30;ZFP37;ZFP42;ZFP64;ZFP69;ZFP69B;ZFP82;ZFP91;ZFX;ZIC1;ZIC2;ZIC3;ZIC4;ZIC5;ZIK1;ZIM3;ZKSCAN1;ZKSCAN2;ZKSCAN3;ZKSCAN4;ZNF121;ZNF124;ZNF133;ZNF136;ZNF138;ZNF14;ZNF140;ZNF143;ZNF146;ZNF148;ZNF155;ZNF157;ZNF160;ZNF169;ZNF175;ZNF177;ZNF18;ZNF180;ZNF181;ZNF2;ZNF20;ZNF212;ZNF213;ZNF214;ZNF221;ZNF222;ZNF223;ZNF224;ZNF225;ZNF226;ZNF227;ZNF229;ZNF230;ZNF232;ZNF233;ZNF234;ZNF235;ZNF24;ZNF25;ZNF250;ZNF257;ZNF26;ZNF260;ZNF263;ZNF264;ZNF268;ZNF274;ZNF276;ZNF28;ZNF280A;ZNF280B;ZNF280C;ZNF280D;ZNF281;ZNF282;ZNF283;ZNF284;ZNF285;ZNF286A;ZNF286B;ZNF3;ZNF30;ZNF300;ZNF302;ZNF317;ZNF32;ZNF320;ZNF322;ZNF324;ZNF324B;ZNF329;ZNF331;ZNF333;ZNF33A;ZNF33B;ZNF343;ZNF345;ZNF347;ZNF350;ZNF354A;ZNF354B;ZNF362;ZNF366;ZNF383;ZNF384;ZNF394;ZNF397;ZNF398;ZNF404;ZNF41;ZNF410;ZNF419;ZNF420;ZNF431;ZNF432;ZNF436;ZNF439;ZNF44;ZNF440;ZNF442;ZNF443;ZNF446;ZNF449;ZNF45;ZNF460;ZNF468;ZNF479;ZNF484;ZNF490;ZNF500;ZNF502;ZNF524;ZNF525;ZNF528;ZNF543;ZNF544;ZNF546;ZNF547;ZNF548;ZNF549;ZNF554;ZNF555;ZNF557;ZNF558;ZNF559;ZNF561;ZNF562;ZNF563;ZNF564;ZNF566;ZNF567;ZNF568;ZNF57;ZNF570;ZNF571;ZNF572;ZNF577;ZNF581;ZNF582;ZNF583;ZNF585A;ZNF586;ZNF589;ZNF595;ZNF599;ZNF600;ZNF605;ZNF607;ZNF611;ZNF613;ZNF614;ZNF615;ZNF616;ZNF619;ZNF620;ZNF621;ZNF625;ZNF627;ZNF649;ZNF652;ZNF653;ZNF665;ZNF667;ZNF669;ZNF670;ZNF672;ZNF679;ZNF680;ZNF683;ZNF689;ZNF692;ZNF701;ZNF705D;ZNF705E;ZNF705G;ZNF708;ZNF709;ZNF71;ZNF710;ZNF713;ZNF721;ZNF727;ZNF729;ZNF736;ZNF75A;ZNF75D;ZNF76;ZNF763;ZNF764;ZNF765;ZNF768;ZNF77;ZNF771;ZNF773;ZNF774;ZNF776;ZNF777;ZNF780A;ZNF780B;ZNF782;ZNF785;ZNF799;ZNF805;ZNF808;ZNF81;ZNF813;ZNF816;ZNF823;ZNF829;ZNF836;ZNF841;ZNF844;ZNF845;ZNF846;ZNF85;ZNF853;ZNF860;ZNF878;ZNF891;ZNF99;ZSCAN16;ZSCAN2;ZSCAN22;ZSCAN23;ZSCAN29;ZSCAN31;ZSCAN32;ZSCAN4;ZSCAN5A;ZSCAN5B;ZSCAN5C;ZSCAN9;ZXDA;ZXDB;ZXDC	BCL6B;BCL6
MEF2A_HUMAN.H11MO.0.A	23	0.13450292	0.8268835	2.097.398	1,35E+07	0.20721809	0.4401244	Regulators of differentiation {5.1.1}	MEF-2{5.1.1.1}	MEF2A;MEF2B;MEF2C;MEF2D	MEF2A;MEF2B;MEF2C;MEF2D
IRF5_HUMAN.H11MO.0.D	28	0.16374269	0.8265720	1.942.468	1,29E+07	0.21404080	0.4436168	Interferon-regulatory factors{3.5.3}	IRF-5{3.5.3.0.5}	IRF1;IRF2;IRF3;IRF4;IRF5;IRF6;IRF7;IRF8;IRF9	IRF5
MAFF_HUMAN.H11MO.1.B	27	0.15789474	0.8262839	1.967.846	1,30E+07	0.20579693	0.4401244	Maf-related factors {1.1.3}	Small Maf factors{1.1.3.2}	MAF;MAFA;MAFB;MAFF;MAFG;MAFK;NRL	MAFF;MAFG;MAFK
ETV1_HUMAN.H11MO.0.A	48	0.28070175	0.8256800	1.657.920	1,18E+07	0.33104261	0.5800769	Ets-related factors {3.5.2}	Elk-like factors{3.5.2.2}	EHF;ELF1;ELF2;ELF3;ELF4;ELF5;ELK1;ELK3;ELK4;ERF;ERG;ETS1;ETS2;ETV1;ETV2;ETV3;	ELK1;ELK3;ELK4;ETV1;ETV4;ETV5

motif	Nº of probes	% of probes	lower OR	upper OR	OR	p.value	FDR	TF family	TF subfamily	TF.family.member	TF.subfamily.member
										ETV3L;ETV4;ETV5;ETV6;ETV7;FEV;FLI1;GABPA;SPDEF;SPI1;SPIB;SPIC	
ZSC16_HUMAN.H11MO.0.D	17	0.09941520	0.8236836	2.401.724	1,45E+07	0.13650334	0.3405957	More than 3 adjacent zinc finger factors{2.3.3}	unclassified{2.3.3.0}	BCL6B;BCL6;CTCFL;CTCF;FEZF1;GFI1B;GFI1;GLI1;GLI2;GLI3;GLIS1;GLIS2;GLIS3;MTF1;MYNN;MZF1;OSR2;OVOL1;OVOL2;ZNF146;PLAG1;PLAGL1;PRDM14;PRDM1;PRDM6;SCRT1;SCRT2;SNAI1;SNAI2;YY1;YY2;WT1;ZNF324;ZNF354A;ZBTB14;ZBTB18;ZBTB48;ZBTB49;ZBTB7A;ZBTB7B;ZBTB6;ZFP64;ZFP28;ZFP42;ZFP82;ZFX;ZIC1;ZIC2;ZIC3;ZIC4;ZIM3;ZKSCAN1;ZKSCAN3;ZNF121;ZNF136;ZNF140;ZNF143;ZNF148;ZNF214;ZNF232;ZNF250;ZNF257;ZNF260;ZNF263;ZNF264;ZNF274;ZNF281;ZNF282;ZNF317;ZNF320;ZNF322;ZNF329;ZNF331;ZNF333;ZNF350;ZNF384;ZNF394;ZNF410;ZNF436;ZNF449;ZNF490;ZNF502;ZNF524;ZNF528;ZNF547;ZNF549;ZNF554;ZNF563;ZNF582;ZNF586;ZNF589;ZNF652;ZNF667;ZNF680;ZNF708;ZNF713;ZNF768;ZNF816;ZNF18;ZNF41;ZNF76;ZNF85;ZSCAN16;ZSCAN22;ZSCAN31;ZSCAN4	MYNN;MZF1;OSR2;PRDM14;PRDM6;WT1;ZBTB14;ZBTB48;ZBTB49;ZFP64;ZFP28;ZIM3;ZNF121;ZNF250;ZNF257;ZNF263;ZNF274;ZNF317;ZNF320;ZNF329;ZNF331;ZNF394;ZNF449;ZNF502;ZNF528;ZNF547;ZNF549;ZNF554;ZNF586;ZNF589;ZNF667;ZNF680;ZNF708;ZNF713;ZNF768;ZNF18;ZNF85;ZSCAN16;ZSCAN22
TBX19_HUMAN.H11MO.0.D	14	0.08187135	0.8225563	2.663.976	1,54E+07	0.12732314	0.3239807	Brachyury-related factors {6.5.1}	TBX19{6.5.1.0.2}	T;TBX19	TBX19
PRDM1_HUMAN.H11MO.0.A	28	0.16374269	0.8214793	1.930.393	1,28E+07	0.25800751	0.4895321	More than 3 adjacent zinc finger factors{2.3.3}	PRDM1-like factors{2.3.3.12}	BCL6;BCL6B;CTCF;CTCFL;FEZF1;FEZF2;GFI1;GFI1B;GLI1;GLI2;GLI3;GLI4;GLIS1;GLIS2;GLIS3;HKR1;MTF1;MYNN;MZF1;OSR2;OVOL1;OVOL2;PLAG1;PLAGL1;PLAGL2;PRDM1;PRDM14;PRDM6;SCRT1;SCRT2;SNAI1;SNAI2;SNAI3;WT1;YY1;YY2;ZBTB12;ZBTB14;ZBTB18;ZBTB20;ZBTB26;ZBTB42;ZBTB45;ZBTB47;ZBTB48;ZBTB49;ZBTB6;ZBTB7A;ZBTB7B;ZBTB7C;ZFP14;ZFP2;ZFP28;ZFP30;ZFP37;ZFP42;ZFP64;ZFP69;ZFP69B;ZFP82;ZFP91;ZFX;ZIC1;ZIC2;ZIC3;ZIC4;ZIC5;ZIK1;ZIM3;ZKSCAN1;ZKSCAN2;ZKSCAN3;ZKSCAN4;ZNF121;ZNF124;ZNF133;ZNF136;ZNF138;ZNF14;ZNF140;ZNF143;ZNF146;ZNF148;ZNF155;ZNF157;ZNF160;ZNF169;ZNF175;ZNF177;ZNF18;ZNF180;ZNF181;ZNF2;ZNF20;ZNF212;ZNF213;ZNF214;ZNF221;ZNF222;ZNF223;ZNF224;ZNF225;ZNF226;ZNF227;ZNF229;ZNF230;ZNF232;ZNF233;ZNF234;ZNF235;ZNF24;ZNF25;ZNF250;ZNF257;ZNF26;ZNF260;ZNF263;ZNF264;ZNF268;ZNF274;ZNF276;ZNF28;ZNF280A;ZNF280B;ZNF280C;ZNF280D;ZNF281;ZNF282;ZNF283;ZNF284;ZNF285;ZNF286A;ZNF286B;ZNF3;ZNF30;ZNF300;ZNF302;ZNF317;ZNF32;ZNF320;ZNF322;ZNF324;ZNF324B;ZNF329;ZNF331;ZNF333;ZNF33A;ZNF33B;ZNF343;ZNF345;ZNF347;ZNF350;ZNF354A;ZNF354B;ZNF362;ZNF366;ZNF383;ZNF384;ZNF394;ZNF397;ZNF398;ZNF404;ZNF41;ZNF410;ZNF419;ZNF420;ZNF431;ZNF432;ZNF436;ZNF439;ZNF44;ZNF440;ZNF442;ZNF443;ZNF446;ZNF449;ZNF45;ZNF460;ZNF468;ZNF479;ZNF484;ZNF490;ZNF500;ZNF502;ZNF524;ZNF525;ZNF528;ZNF543;ZNF544;ZNF546;ZNF547;ZNF548;ZNF549;ZNF554;ZNF555;ZNF557;ZNF558;ZNF559;ZNF561;ZNF562;ZNF563;ZNF564;ZNF566;ZNF567;ZNF568;ZNF57;ZNF570;ZNF571;ZNF572;ZNF577;ZNF581;ZNF582;ZNF583;ZNF585A;ZNF586;ZNF589;ZNF595;ZNF599;ZNF600;ZNF605;ZNF607;ZNF611;ZNF613;ZNF614;ZNF615;ZNF616;ZNF619;ZNF620;ZNF621;ZNF625;ZNF627;ZNF649;ZNF652;ZNF653;ZNF665;ZNF667;ZNF669;ZNF670;ZNF672;ZNF679;ZNF680;ZNF683;ZNF689;ZNF692;ZNF70	PRDM1

motif	Nº of probes	% of probes	lower OR	upper OR	OR	p.value	FDR	TF family	TF subfamily	TF.family.member	TF.subfamily.member
										1;ZNF705D;ZNF705E;ZNF705G;ZNF708;ZNF709;ZNF71;ZNF710;ZNF713;ZNF721;ZNF727;ZNF729;ZNF736;ZNF75A;ZNF75D;ZNF76;ZNF763;ZNF764;ZNF765;ZNF768;ZNF77;ZNF771;ZNF773;ZNF774;ZNF776;ZNF777;ZNF780A;ZNF780B;ZNF782;ZNF785;ZNF799;ZNF805;ZNF808;ZNF81;ZNF813;ZNF816;ZNF823;ZNF829;ZNF836;ZNF841;ZNF844;ZNF845;ZNF846;ZNF85;ZNF853;ZNF860;ZNF878;ZNF891;ZNF99;ZSCAN16;ZSCAN2;ZSCAN22;ZSCAN23;ZSCAN29;ZSCAN31;ZSCAN32;ZSCAN4;ZSCAN5A;ZSCAN5B;ZSCAN5C;ZSCAN9;ZXDA;ZXDB;ZXDC	
FLI1_HUMAN.H11MO.1.A	48	0.28070175	0.8173260	1.641.128	1,17E+07	0.37735630	0.6225095	Ets-related factors{3.5.2}	Ets-like factors{3.5.2.1}	EHF;ELF1;ELF2;ELF3;ELF4;ELF5;ELK1;ELK3;ELK4;ERF;ERG;ETS1;ETS2;ETV1;ETV2;ETV3;ETV3L;ETV4;ETV5;ETV6;ETV7;FEV;FLI1;GABPA;SPDEF;SPI1;SPIB;SPIC	ERG;ETS1;ETS2;ETV2;ETV3;FEV;FLI1;GABPA
IRF7_HUMAN.H11MO.0.C	21	0.12280702	0.8157780	2.151.668	1,36E+07	0.18815418	0.4204837	Interferon-regulatory factors{3.5.3}	IRF-7{3.5.3.0.7}	IRF1;IRF2;IRF3;IRF4;IRF5;IRF6;IRF7;IRF8;IRF9	IRF7
FOXB1_HUMAN.H11MO.0.D	18	0.10526316	0.8156212	2.311.381	1,41E+07	0.19313893	0.4279026	Forkhead box (FOX) factors{3.3.1}	FOXB{3.3.1.2}	FOXA1;FOXA2;FOXA3;FOXB1;FOXB2;FOXC1;FOXC2;FOXD1;FOXD2;FOXD3;FOXD4;FOXD4L1;FOXD4L3;FOXD4L4;FOXD4L5;FOXD4L6;FOXE1;FOXE3;FOXF1;FOXF2;FOXG1;FOXH1;FOXI1;FOXI2;FOXI3;FOXJ1;FOXJ2;FOXJ3;FOXK1;FOXK2;FOXL1;FOXL2;FOXM1;FOXN1;FOXN2;FOXN3;FOXN4;FOXO1;FOXO3;FOXO4;FOXO6;FOXP1;FOXP2;FOXP3;FOXP4;FOXQ1;FOXR1;FOXR2;FOXS1	FOXB1
ANDR_HUMAN.H11MO.0.A	26	0.15204678	0.8145976	1.967.760	1,29E+07	0.24141331	0.4726051	Steroid hormone receptors (NR3){2.1.1}	GR-like receptors (NR3C){2.1.1.1}	AR;ESR1;ESR2;ESRRA;ESRRB;ESRRG;NR3C1;NR3C2;PGR	AR;NR3C1;NR3C2;PGR
BARH2_HUMAN.H11MO.0.D	13	0.07602339	0.8101391	2.740.635	1,56E+07	0.15634884	0.3755294	NK-related factors{3.1.2}	BARHL{3.1.2.1}	BARHL1;BARHL2;BARX1;BARX2;BSX;DBX1;DBX2;DLX1;DLX2;DLX3;DLX4;DLX5;DLX6;EMX1;EMX2;EN1;EN2;HHEX;HLX;HMX1;HMX2;HMX3;LBX1;LBX2;MSX1;MSX2;NANOG;NKX1-1;NKX1-2;NKX2-1;NKX2-2;NKX2-3;NKX2-4;NKX2-5;NKX2-6;NKX2-8;NKX3-1;NKX3-2;NKX6-1;NKX6-2;NKX6-3;NOTO;TLX1;TLX2;TLX3;VAX1;VAX2;VENTX	BARHL1;BARHL2
ZN418_HUMAN.H11MO.0.C	23	0.13450292	0.8094881	2.053.390	1,32E+07	0.21297198	0.4436168	Factors with multiple dispersed zinc fingers{2.3.4}	ZNF417-like factors{2.3.4.1}	BCL11A;BCL11B;BNC1;BNC2;E4F1;HIC1;HIC2;HINFP;HIVEP1;HIVEP2;HIVEP3;IKZF1;IKZF2;IKZF3;IKZF4;IKZF5;INSM1;INSM2;MAZ;MECOM;PATZ1;PRDM16;PRDM4;REST;RLF;RREB1;SALL1;SALL2;SALL3;SALL4;VEZF1;ZBTB1;ZBTB17;ZBTB2;ZBTB25;ZBTB4;ZFAT;ZNF134;ZNF211;ZNF217;ZNF219;ZNF248;ZNF256;ZNF292;ZNF296;ZNF319;ZNF334;ZNF335;ZNF341;ZNF37A;ZNF382;ZNF417;ZNF418;ZNF423;ZNF467;ZNF510;ZNF512;ZNF512B;ZNF516;ZNF518A;ZNF518B;ZNF521;ZNF526;ZNF532;ZNF536;ZNF552;ZNF574;ZNF587;ZNF587B;ZNF592;ZNF639;	ZNF418

motif	Nº of probes	% of probes	lower OR	upper OR	OR	p.value	FDR	TF family	TF subfamily	TF.family.member	TF.subfamily.member
										ZNF654;ZNF658;ZNF671;ZNF687;ZNF711;ZNF717;ZNF770;ZNF772;ZNF784;ZNF786;ZNF792;ZNF8;ZNF814	
GATA1_HUMAN.H11MO.0.A	16	0.09356725	0.8091445	2.434.543	1,45E+07	0.16396965	0.3867189	GATA-type zinc fingers{2.2.1}	Two zinc-finger GATA factors{2.2.1.1}	GATA1;GATA2;GATA3;GATA4;GATA5;GATA6;GATAD2A;GATAD2B;TRPS1;ZGLP1	GATA1;GATA2;GATA3;GATA4;GATA5;GATA6
FOXD2_HUMAN.H11MO.0.D	19	0.11111111	0.8056814	2.224.605	1,38E+07	0.21010938	0.4413491	Forkhead box (FOX) factors {3.3.1}	FOXD{3.3.1.4}	FOXA1;FOXA2;FOXA3;FOXB1;FOXB2;FOXC1;FOXC2;FOXD1;FOXD2;FOXD3;FOXD4;FOXD4L1;FOXD4L3;FOXD4L4;FOXD4L5;FOXD4L6;FOXE1;FOXE3;FOXF1;FOXF2;FOXG1;FOXH1;FOXI1;FOXI2;FOXI3;FOXJ1;FOXJ2;FOXJ3;FOXK1;FOXK2;FOXL1;FOXL2;FOXM1;FOXN1;FOXN2;FOXN3;FOXN4;FOXO1;FOXO3;FOXO4;FOXO6;FOXP1;FOXP2;FOXP3;FOXP4;FOXQ1;FOXR1;FOXR2;FOXS1	FOXD1;FOXD2;FOXD3
FOXO6_HUMAN.H11MO.0.D	17	0.09941520	0.8041913	2.344.675	1,42E+07	0.18114077	0.4107633	Forkhead box (FOX) factors {3.3.1}	FOXO{3.3.1.15}	FOXA1;FOXA2;FOXA3;FOXB1;FOXB2;FOXC1;FOXC2;FOXD1;FOXD2;FOXD3;FOXD4;FOXD4L1;FOXD4L3;FOXD4L4;FOXD4L5;FOXD4L6;FOXE1;FOXE3;FOXF1;FOXF2;FOXG1;FOXH1;FOXI1;FOXI2;FOXI3;FOXJ1;FOXJ2;FOXJ3;FOXK1;FOXK2;FOXL1;FOXL2;FOXM1;FOXN1;FOXN2;FOXN3;FOXN4;FOXO1;FOXO3;FOXO4;FOXO6;FOXP1;FOXP2;FOXP3;FOXP4;FOXQ1;FOXR1;FOXR2;FOXS1	FOXO1;FOXO3;FOXO4;FOXO6
RORG_HUMAN.H11MO.0.C	18	0.10526316	0.8041428	2.278.862	1,39E+07	0.19612146	0.4308499	Thyroid hormone receptor-related factors (NR1){2.1.2}	ROR (NR1F){2.1.2.6}	NR1D1;NR1D2;NR1H2;NR1H3;NR1H4;NR1I2;NR1I3;PPARA;PPARD;PPARG;RARA;RARB;RARG;RORA;RORB;RORC;THRA;THRB;VDR	RORA;RORC
ANDR_HUMAN.H11MO.2.A	24	0.14035088	0.8018374	1.998.765	1,29E+07	0.22699720	0.4546178	Steroid hormone receptors (NR3){2.1.1}	GR-like receptors (NR3C){2.1.1.1}	AR;ESR1;ESR2;ESRRA;ESRRB;ESRRG;NR3C1;NR3C2;PGR	AR;NR3C1;NR3C2;PGR
NR1D1_HUMAN.H11MO.1.D	13	0.07602339	0.8009336	2.709.266	1,54E+07	0.15847099	0.3794445	Thyroid hormone receptor-related factors (NR1){2.1.2}	Rev-ErbA (NR1D){2.1.2.3}	NR1D1;NR1D2;NR1H2;NR1H3;NR1H4;NR1I2;NR1I3;PPARA;PPARD;PPARG;RARA;RARB;RARG;RORA;RORB;RORC;THRA;THRB;VDR	NR1D1
P53_HUMAN.H11MO.0.A	21	0.12280702	0.8008705	2.112.338	1,33E+07	0.23829274	0.4686829	p53-related factors {6.3.1}	p53{6.3.1.0.1}	TP53;TP63;TP73	TP53
RFX4_HUMAN.H11MO.0.D	16	0.09356725	0.7990863	2.404.242	1,43E+07	0.16772074	0.3894960	RFX-related factors {3.3.3}	RFX4{3.3.3.0.4}	RFX1;RFX2;RFX3;RFX4;RFX5;RFX6;RFX7;RFX8	RFX4

motif	Nº of probes	% of probes	lower OR	upper OR	OR	p.value	FDR	TF family	TF subfamily	TF.family.member	TF.subfamily.member
ZN333_HUMAN.H11MO.0.D	12	0.07017544	0.7990519	2.840.077	1,58E+07	0.13777198	0.3415505	More than 3 adjacent zinc finger factors{2.3.3}	ZNF177-like factors{2.3.3.40}	BCL6;BCL6B;CTCF;CTCFL;FEZF1;FEZF2;GFI1;GFI1B;GLI1;GLI2;GLI3;GLI4;GLIS1;GLIS2;GLIS3;HKR1;MTF1;MYNN;MZF1;OSR2;OVOL1;OVOL2;PLAG1;PLAGL1;PLAGL2;PRDM1;PRDM14;PRDM6;SCRT1;SCRT2;SNAI1;SNAI2;SNAI3;WT1;YY1;YY2;ZBTB12;ZBTB14;ZBTB18;ZBTB20;ZBTB26;ZBTB42;ZBTB45;ZBTB47;ZBTB48;ZBTB49;ZBTB6;ZBTB7A;ZBTB7B;ZBTB7C;ZFP14;ZFP2;ZFP28;ZFP30;ZFP37;ZFP42;ZFP64;ZFP69;ZFP69B;ZFP82;ZFP91;ZFX;ZIC1;ZIC2;ZIC3;ZIC4;ZIC5;ZIK1;ZIM3;ZKSCAN1;ZKSCAN2;ZKSCAN3;ZKSCAN4;ZNF121;ZNF124;ZNF133;ZNF136;ZNF138;ZNF14;ZNF140;ZNF143;ZNF146;ZNF148;ZNF155;ZNF157;ZNF160;ZNF169;ZNF175;ZNF177;ZNF18;ZNF180;ZNF181;ZNF2;ZNF20;ZNF212;ZNF213;ZNF214;ZNF221;ZNF222;ZNF223;ZNF224;ZNF225;ZNF226;ZNF227;ZNF229;ZNF230;ZNF232;ZNF233;ZNF234;ZNF235;ZNF24;ZNF25;ZNF250;ZNF257;ZNF26;ZNF260;ZNF263;ZNF264;ZNF268;ZNF274;ZNF276;ZNF28;ZNF280A;ZNF280B;ZNF280C;ZNF280D;ZNF281;ZNF282;ZNF283;ZNF284;ZNF285;ZNF286A;ZNF286B;ZNF3;ZNF30;ZNF300;ZNF302;ZNF317;ZNF32;ZNF320;ZNF322;ZNF324;ZNF324B;ZNF329;ZNF331;ZNF333;ZNF33A;ZNF33B;ZNF343;ZNF345;ZNF347;ZNF350;ZNF354A;ZNF354B;ZNF362;ZNF366;ZNF383;ZNF384;ZNF394;ZNF397;ZNF398;ZNF404;ZNF41;ZNF410;ZNF419;ZNF420;ZNF431;ZNF432;ZNF436;ZNF439;ZNF44;ZNF440;ZNF442;ZNF443;ZNF446;ZNF449;ZNF45;ZNF460;ZNF468;ZNF479;ZNF484;ZNF490;ZNF500;ZNF502;ZNF524;ZNF525;ZNF528;ZNF543;ZNF544;ZNF546;ZNF547;ZNF548;ZNF549;ZNF554;ZNF555;ZNF557;ZNF558;ZNF559;ZNF561;ZNF562;ZNF563;ZNF564;ZNF566;ZNF567;ZNF568;ZNF57;ZNF570;ZNF571;ZNF572;ZNF577;ZNF581;ZNF582;ZNF583;ZNF585A;ZNF586;ZNF589;ZNF595;ZNF599;ZNF600;ZNF605;ZNF607;ZNF611;ZNF613;ZNF614;ZNF615;ZNF616;ZNF619;ZNF620;ZNF621;ZNF625;ZNF627;ZNF649;ZNF652;ZNF653;ZNF665;ZNF667;ZNF669;ZNF670;ZNF672;ZNF679;ZNF680;ZNF683;ZNF689;ZNF692;ZNF701;ZNF705D;ZNF705E;ZNF705G;ZNF708;ZNF709;ZNF71;ZNF710;ZNF713;ZNF721;ZNF727;ZNF729;ZNF736;ZNF75A;ZNF75D;ZNF76;ZNF763;ZNF764;ZNF765;ZNF768;ZNF77;ZNF771;ZNF773;ZNF774;ZNF776;ZNF777;ZNF780A;ZNF780B;ZNF782;ZNF785;ZNF799;ZNF805;ZNF808;ZNF81;ZNF813;ZNF816;ZNF823;ZNF829;ZNF836;ZNF841;ZNF844;ZNF845;ZNF846;ZNF85;ZNF853;ZNF860;ZNF878;ZNF891;ZNF99;ZSCAN16;ZSCAN2;ZSCAN22;ZSCAN23;ZSCAN29;ZSCAN31;ZSCAN32;ZSCAN4;ZSCAN5A;ZSCAN5B;ZSCAN5C;ZSCAN9;ZXDA;ZXDB;ZXDC	ZNF333
STA5B_HUMAN.H11MO.0.A	18	0.10526316	0.7943160	2.250.981	1,38E+07	0.19959950	0.4347209	STAT factors{6.2.1}	STAT5B{6.2.1.0.6}	STAT1;STAT2;STAT3;STAT4;STAT5A;STAT5B;STAT6	STAT5B
RELB_HUMAN.H11MO.0.C	21	0.12280702	0.7909108	2.086.186	1,32E+07	0.24151287	0.4726051	NF-kappaB-related factors{6.1.1}	NF-kappaB p65 subunit-like factors{6.1.1.2}	NFKB1;NFKB2;REL;RELA;RELB	RELB;REL;RELA
SOX18_HUMAN.H11MO.0.D	15	0.08771930	0.7853654	2.446.574	1,44E+07	0.20295409	0.4401244	SOX-related factors{4.1.1}	Group F{4.1.1.6}	BBX;CIC;HBP1;SOX1;SOX10;SOX11;SOX12;SOX13;SOX14;SOX15;SOX17;SOX18;SOX2;SOX21;SOX3;SOX30;SOX4;SOX5;SOX6;SOX7;SOX8;SOX9;SRY	SOX17;SOX18;SOX7

motif	Nº of probes	% of probes	lower OR	upper OR	OR	p.value	FDR	TF family	TF subfamily	TF.family.member	TF.subfamily.member
GABPA_HUMAN.H11MO.0.A	49	0.28654971	0.7847794	1.568.660	1,12E+07	0.54358458	0.7563887	Ets-related factors{3.5.2}	Ets-like factors{3.5.2.1}	EHF;ELF1;ELF2;ELF3;ELF4;ELF5;ELK1;ELK3;ELK4;ERF;ERG;ETS1;ETS2;ETV1;ETV2;ETV3;ETV3L;ETV4;ETV5;ETV6;ETV7;FEV;FLI1;GABPA;SPDEF;SPI1;SPIB;SPIC	ERG;ETS1;ETS2;ETV2;ETV3;FEV;FLI1;GABPA
ONEC2_HUMAN.H11MO.0.D	18	0.10526316	0.7831935	2.219.498	1,36E+07	0.20459667	0.4401244	HD-CUT factors{3.1.9}	ONECUT{3.1.9.1}	CUX1;CUX2;ONECUT1;ONECUT2;ONECUT3;SATB1;SATB2	ONECUT1;ONECUT2;ONECUT3
NR1D1_HUMAN.H11MO.0.B	34	0.19883041	0.7831676	1.724.082	1,18E+07	0.41901695	0.6661074	Thyroid hormone receptor-related factors (NR1){2.1.2}	Rev-ErbA (NR1D){2.1.2.3}	NR1D1;NR1D2;NR1H2;NR1H3;NR1H4;NR1I2;NR1I3;PPARA;PPARD;PPARG;RARA;RARB;RARG;RORA;RORB;RORC;THRA;THRB;VDR	NR1D1
CREB1_HUMAN.H11MO.0.A	23	0.13450292	0.7795198	1.977.308	1,27E+07	0.27047365	0.5000844	CREB-related factors{1.1.7}	CREB-like factors{1.1.7.1}	ATF1;ATF6;ATF6B;CREB1;CREB3;CREB3L1;CREB3L2;CREB3L3;CREB3L4;CREBL2;CREBZF;CREM	ATF1;CREB1;CREM
ZN143_HUMAN.H11MO.0.A	29	0.16959064	0.7794235	1.808.822	1,21E+07	0.38343741	0.6250111	More than 3 adjacent zinc finger factors{2.3.3}	ZNF76-like factors{2.3.3.28}	BCL6;BCL6B;CTCF;CTCFL;FEZF1;FEZF2;GFI1;GFI1B;GLI1;GLI2;GLI3;GLI4;GLIS1;GLIS2;GLIS3;HKR1;MTF1;MYNN;MZF1;OSR2;OVOL1;OVOL2;PLAG1;PLAGL1;PLAGL2;PRDM1;PRDM14;PRDM6;SCRT1;SCRT2;SNAI1;SNAI2;SNAI3;WT1;YY1;YY2;ZBTB12;ZBTB14;ZBTB18;ZBTB20;ZBTB26;ZBTB42;ZBTB45;ZBTB47;ZBTB48;ZBTB49;ZBTB6;ZBTB7A;ZBTB7B;ZBTB7C;ZFP14;ZFP2;ZFP28;ZFP30;ZFP37;ZFP42;ZFP64;ZFP69;ZFP69B;ZFP82;ZFP91;ZFX;ZIC1;ZIC2;ZIC3;ZIC4;ZIC5;ZIK1;ZIM3;ZKSCAN1;ZKSCAN2;ZKSCAN3;ZKSCAN4;ZNF121;ZNF124;ZNF133;ZNF136;ZNF138;ZNF14;ZNF140;ZNF143;ZNF146;ZNF148;ZNF155;ZNF157;ZNF160;ZNF169;ZNF175;ZNF177;ZNF18;ZNF180;ZNF181;ZNF2;ZNF20;ZNF212;ZNF213;ZNF214;ZNF221;ZNF222;ZNF223;ZNF224;ZNF225;ZNF226;ZNF227;ZNF229;ZNF230;ZNF232;ZNF233;ZNF234;ZNF235;ZNF24;ZNF25;ZNF250;ZNF257;ZNF26;ZNF260;ZNF263;ZNF264;ZNF268;ZNF274;ZNF276;ZNF28;ZNF280A;ZNF280B;ZNF280C;ZNF280D;ZNF281;ZNF282;ZNF283;ZNF284;ZNF285;ZNF286A;ZNF286B;ZNF3;ZNF30;ZNF300;ZNF302;ZNF317;ZNF32;ZNF320;ZNF322;ZNF324;ZNF324B;ZNF329;ZNF331;ZNF333;ZNF33A;ZNF33B;ZNF343;ZNF345;ZNF347;ZNF350;ZNF354A;ZNF354B;ZNF362;ZNF366;ZNF383;ZNF384;ZNF394;ZNF397;ZNF398;ZNF404;ZNF41;ZNF410;ZNF419;ZNF420;ZNF431;ZNF432;ZNF436;ZNF439;ZNF44;ZNF440;ZNF442;ZNF443;ZNF446;ZNF449;ZNF45;ZNF460;ZNF468;ZNF479;ZNF484;ZNF490;ZNF500;ZNF502;ZNF524;ZNF525;ZNF528;ZNF543;ZNF544;ZNF546;ZNF547;ZNF548;ZNF549;ZNF554;ZNF555;ZNF557;ZNF558;ZNF559;ZNF561;ZNF562;ZNF563;ZNF564;ZNF566;ZNF567;ZNF568;ZNF57;ZNF570;ZNF571;ZNF572;ZNF577;ZNF581;ZNF582;ZNF583;ZNF585A;ZNF586;ZNF589;ZNF595;ZNF599;ZNF600;ZNF605;ZNF607;ZNF611;ZNF613;ZNF614;ZNF615;ZNF616;ZNF619;ZNF620;ZNF621;ZNF625;ZNF627;ZNF649;ZNF652;ZNF653;ZNF665;ZNF667;ZNF669;ZNF670;ZNF672;ZNF679;ZNF680;ZNF683;ZNF689;ZNF692;ZNF701;ZNF705D;ZNF705E;ZNF705G;ZNF708;ZNF709;ZNF71;ZNF710;ZNF713;ZNF721;ZNF727;ZNF729;ZNF736;ZNF75A;ZNF75D;ZNF76;ZNF763;ZNF764;ZNF765;ZNF768;ZNF77;ZNF771;ZNF773;ZNF774;ZNF776;ZNF777;ZNF780A;ZNF780B;ZNF782;ZNF785;ZNF799;ZNF805;ZNF808;ZNF81;ZNF813;ZNF816;ZNF823;ZNF829;ZNF836;ZNF841;ZNF844;ZNF845;ZNF846;ZNF85;ZNF853;ZNF860;ZNF878;ZNF891;ZNF99;ZSCAN16;ZSCAN2;ZSCAN22;ZSCAN23;ZSCAN29;ZSCAN31;Z	ZNF143;ZNF76

motif	Nº of probes	% of probes	lower OR	upper OR	OR	p.value	FDR	TF family	TF subfamily	TF.family.member	TF.subfamily.member
										SCAN32;ZSCAN4;ZSCAN5A;ZSCAN5B;ZSCAN5C;ZSCAN9;ZXDA;ZXDB;ZXDC	
HXD3_HUMAN.H11MO.0.D	12	0.07017544	0.7792745	2.769.670	1,54E+07	0.14507800	0.3570559	HOX-related factors{3.1.1}	HOX3{3.1.1.3}	CDX1;CDX2;CDX4;EVX1;EVX2;GBX1;GBX2;GSX1;GSX2;HDX;HMBOX1;HNF1A;HNF1B;HOXA1;HOXA10;HOXA11;HOXA13;HOXA2;HOXA3;HOXA4;HOXA5;HOXA6;HOXA7;HOXA9;HOXB1;HOXB13;HOXB2;HOXB3;HOXB4;HOXB5;HOXB6;HOXB7;HOXB8;HOXB9;HOXC10;HOXC11;HOXC12;HOXC13;HOXC4;HOXC5;HOXC6;HOXC8;HOXC9;HOXD1;HOXD10;HOXD11;HOXD12;HOXD13;HOXD3;HOXD4;HOXD8;HOXD9;MEOX1;MEOX2;MNX1;PDX1;POU1F1;POU2F1;POU2F2;POU2F3;POU3F1;POU3F2;POU3F3;POU3F4;POU4F1;POU4F2;POU4F3;POU5F1;POU5F2;POU6F1;POU6F2	HOXB3;HOXD3
DMRT1_HUMAN.H11MO.0.D	14	0.08187135	0.7784228	2.520.983	1,46E+07	0.18572587	0.4162635	DMRT{2.5.1}	DMRT1{2.5.1.0.1}	DMRT1;DMRT2;DMRT3;DMRTA1;DMRTA2;DMRTB1;DMRTC2	DMRT1
PO4F3_HUMAN.H11MO.0.D	16	0.09356725	0.7729560	2.325.651	1,39E+07	0.22511473	0.4531683	POU domain factors{3.1.10}	POU4 (Brn-3-like factors){3.1.10.4}	CDX1;CDX2;CDX4;EVX1;EVX2;GBX1;GBX2;GSX1;GSX2;HDX;HMBOX1;HNF1A;HNF1B;HOXA1;HOXA10;HOXA11;HOXA13;HOXA2;HOXA3;HOXA4;HOXA5;HOXA6;HOXA7;HOXA9;HOXB1;HOXB13;HOXB2;HOXB3;HOXB4;HOXB5;HOXB6;HOXB7;HOXB8;HOXB9;HOXC10;HOXC11;HOXC12;HOXC13;HOXC4;HOXC5;HOXC6;HOXC8;HOXC9;HOXD1;HOXD10;HOXD11;HOXD12;HOXD13;HOXD3;HOXD4;HOXD8;HOXD9;MEOX1;MEOX2;MNX1;PDX1;POU1F1;POU2F1;POU2F2;POU2F3;POU3F1;POU3F2;POU3F3;POU3F4;POU4F1;POU4F2;POU4F3;POU5F1;POU5F1B;POU5F2;POU6F1;POU6F2	POU4F1;POU4F2;POU4F3
NR2E3_HUMAN.H11MO.0.C	15	0.08771930	0.7703915	2.399.810	1,41E+07	0.20786636	0.4402884	RXR-related receptors (NR2){2.1.3}	Tailless-like receptors (NR2E){2.1.3.3}	HNF4A;HNF4G;NR2C1;NR2C2;NR2E1;NR2E3;NR2F1;NR2F2;NR2F6;RXRA;RXRB;RXRG	NR2E1;NR2E3
VAX2_HUMAN.H11MO.0.D	11	0.06432749	0.7691766	2.892.968	1,57E+07	0.17521029	0.4032452	NK-related factors{3.1.2}	VAX{3.1.2.22}	BARHL1;BARHL2;BARX1;BARX2;BSX;DBX1;DBX2;DLX1;DLX2;DLX3;DLX4;DLX5;DLX6;EMX1;EMX2;EN1;EN2;HHEX;HLX;HMX1;HMX2;HMX3;LBX1;LBX2;MSX1;MSX2;NANOG;NKX1-1;NKX1-2;NKX2-1;NKX2-2;NKX2-3;NKX2-4;NKX2-5;NKX2-6;NKX2-8;NKX3-1;NKX3-2;NKX6-1;NKX6-2;NKX6-	VAX1;VAX2

motif	Nº of probes	% of probes	lower OR	upper OR	OR	p.value	FDR	TF family	TF subfamily	TF.family.member	TF.subfamily.member
										3;NOTO;TLX1;TLX2;TLX3;VAX1;VAX2;VENTX	
HME2_HUMAN.H11MO.0.D	12	0.07017544	0.7684905	2.731.245	1,52E+07	0.14995525	0.3658718	NK-related factors {3.1.2}	EN (Engrailed-like factors){3.1.2.7}	BARHL1;BARHL2;BARX1;BARX2;BSX;DBX1;DBX2;DLX1;DLX2;DLX3;DLX4;DLX5;DLX6;EMX1;EMX2;EN1;EN2;HHEX;HLX;HMX1;HMX2;HMX3;LBX1;LBX2;MSX1;MSX2;NANOG;NKX1-1;NKX1-2;NKX2-1;NKX2-2;NKX2-3;NKX2-4;NKX2-5;NKX2-6;NKX2-8;NKX3-1;NKX3-2;NKX6-1;NKX6-2;NKX6-3;NOTO;TLX1;TLX2;TLX3;VAX1;VAX2;VENTX	EN1;EN2
ZNF85_HUMAN.H11MO.0.C	15	0.08771930	0.7636398	2.378.881	1,40E+07	0.21065692	0.4413491	More than 3 adjacent zinc finger factors{2.3.3}	unclassified{2.3.3.0}	BCL6B;BCL6;CTCFL;CTCF;FEZF1;GFI1B;GFI1;GLI1;GLI2;GLI3;GLIS1;GLIS2;GLIS3;MTF1;MYNN;MZF1;OSR2;OVOL1;OVOL2;ZNF146;PLAG1;PLAGL1;PRDM14;PRDM1;PRDM6;SCRT1;SCRT2;SNAI1;SNAI2;YY1;YY2;WT1;ZNF324;ZNF354A;ZBTB14;ZBTB18;ZBTB48;ZBTB49;ZBTB7A;ZBTB7B;ZBTB6;ZFP64;ZFP28;ZFP42;ZFP82;ZFX;ZIC1;ZIC2;ZIC3;ZIC4;ZIM3;ZKSCAN1;ZKSCAN3;ZNF121;ZNF136;ZNF140;ZNF143;ZNF148;ZNF214;ZNF232;ZNF250;ZNF257;ZNF260;ZNF263;ZNF264;ZNF274;ZNF281;ZNF282;ZNF317;ZNF320;ZNF322;ZNF329;ZNF331;ZNF333;ZNF350;ZNF384;ZNF394;ZNF410;ZNF436;ZNF449;ZNF490;ZNF502;ZNF524;ZNF528;ZNF547;ZNF549;ZNF554;ZNF563;ZNF582;ZNF586;ZNF589;ZNF652;ZNF667;ZNF680;ZNF708;ZNF713;ZNF768;ZNF816;ZNF18;ZNF41;ZNF76;ZNF85;ZSCAN16;ZSCAN22;ZSCAN31;ZSCAN4	MYNN;MZF1;OSR2;PRDM14;PRDM6;WT1;ZBTB14;ZBTB48;ZBTB49;ZFP64;ZFP28;ZIM3;ZNF121;ZNF250;ZNF257;ZNF263;ZNF274;ZNF317;ZNF320;ZNF329;ZNF331;ZNF394;ZNF449;ZNF502;ZNF528;ZNF547;ZNF549;ZNF554;ZNF586;ZNF589;ZNF667;ZNF680;ZNF708;ZNF713;ZNF768;ZNF18;ZNF85;ZSCAN16;ZSCAN22
ELF1_HUMAN.H11MO.0.A	40	0.23391813	0.7612024	1.598.484	1,11E+07	0.57617664	0.7845295	Ets-related factors {3.5.2}	Elf-1-like factors{3.5.2.3}	EHF;ELF1;ELF2;ELF3;ELF4;ELF5;ELK1;ELK3;ELK4;ERF;ERG;ETS1;ETS2;ETV1;ETV2;ETV3;ETV3L;ETV4;ETV5;ETV6;ETV7;FEV;FLI1;GABPA;SPDEF;SPI1;SPIB;SPIC	ELF1;ELF2
PAX4_HUMAN.H11MO.0.D	12	0.07017544	0.7607588	2.703.756	1,50E+07	0.20408859	0.4401244	Paired plus homeo domain{3.2.1}	PAX-4/6{3.2.1.2}	PAX3;PAX4;PAX6;PAX7	PAX4;PAX6
ALX3_HUMAN.H11MO.0.D	12	0.07017544	0.7593455	2.698.682	1,50E+07	0.20445144	0.4401244	Paired-related HD factors{3.1.3}	ALX{3.1.3.1}	ALX1;ALX3;ALX4;ARGFX;ARX;CRX;DMBX1;DPRX;DRGX;DUX4;DUXA;ESX1;GSC;GSC2;HESX1;ISX;LEUTX;MIXL1;NOBOX;OTP;OTX1;OTX2;PHOX2A;PHOX2B;PITX1;PITX2;PITX3;PROP1;PRRX1;PRRX2;RAX;RAX2;RHOXF1;RHOXF2;SEBOX;SHOX;SHOX2;TPRX1;UNCX;VSX1;VSX2	ALX1;ALX3;ALX4
PBX1_HUMAN.H11MO.1.C	9	0.05263158	0.7585403	3.291.652	1,69E+07	0.12370678	0.3189897	TALE-type homeo domain factors{3.1.4}	PBX{3.1.4.4}	IRX2;IRX3;MEIS1;MEIS2;MEIS3;PBX1;PBX2;PBX3;PKNOX1;TGIF2LX;TGIF1;TGIF2	PBX1;PBX2;PBX3
FLI1_HUMAN.H11MO.0.A	63	0.36842105	0.7571896	1.446.702	1,05E+07	0.75001263	0.8847658	Ets-related factors {3.5.2}	Ets-like factors{3.5.2.1}	EHF;ELF1;ELF2;ELF3;ELF4;ELF5;ELK1;ELK3;ELK4;ERF;ERG;ETS1;ETS2;ETV1;ETV2;ETV3;ETV3L;ETV4;ETV5;ETV6;ETV7;FEV;FLI1;GABPA;SPDEF;SPI1;SPIB;SPIC	ERG;ETS1;ETS2;ETV2;ETV3;FEV;FLI1;GABPA
TFE3_HUMAN.H11MO.0.B	27	0.15789474	0.7570618	1.802.912	1,19E+07	0.43398425	0.6718913	bHLH-ZIP factors{1.2.6}	TFE3-like factors{1.2.6.1}	MAX;MITF;MLX;MLXIP;MLXIPL;MNT;MXD1;MXD3;MXD4;MXI1;MYC;MYCL;MYCN;REPIN1;SREBF1;SREBF2;TFAP4;TFE3;TFEB;TFEC;USF1;USF2	MITF;TFE3;TFEB

motif	Nº of probes	% of probes	lower OR	upper OR	OR	p.value	FDR	TF family	TF subfamily	TF.family.member	TF.subfamily.member
ZN232_HUMAN.H11MO.0.D	12	0.07017544	0.7558989	2.686.755	1,49E+07	0.20539134	0.4401244	More than 3 adjacent zinc finger factors{2.3.3}	ZNF24-like factors{2.3.3.10}	BCL6;BCL6B;CTCF;CTCFL;FEZF1;FEZF2;GFI1;GFI1B;GLI1;GLI2;GLI3;GLI4;GLIS1;GLIS2;GLIS3;HKR1;MTF1;MYNN;MZF1;OSR2;OVOL1;OVOL2;PLAG1;PLAGL1;PLAGL2;PRDM1;PRDM14;PRDM6;SCRT1;SCRT2;SNAI1;SNAI2;SNAI3;WT1;YY1;YY2;ZBTB12;ZBTB14;ZBTB18;ZBTB20;ZBTB26;ZBTB42;ZBTB45;ZBTB47;ZBTB48;ZBTB49;ZBTB6;ZBTB7A;ZBTB7B;ZBTB7C;ZFP14;ZFP2;ZFP28;ZFP30;ZFP37;ZFP42;ZFP64;ZFP69;ZFP69B;ZFP82;ZFP91;ZFX;ZIC1;ZIC2;ZIC3;ZIC4;ZIC5;ZIK1;ZIM3;ZKSCAN1;ZKSCAN2;ZKSCAN3;ZKSCAN4;ZNF121;ZNF124;ZNF133;ZNF136;ZNF138;ZNF14;ZNF140;ZNF143;ZNF146;ZNF148;ZNF155;ZNF157;ZNF160;ZNF169;ZNF175;ZNF177;ZNF18;ZNF180;ZNF181;ZNF2;ZNF20;ZNF212;ZNF213;ZNF214;ZNF221;ZNF222;ZNF223;ZNF224;ZNF225;ZNF226;ZNF227;ZNF229;ZNF230;ZNF232;ZNF233;ZNF234;ZNF235;ZNF24;ZNF25;ZNF250;ZNF257;ZNF26;ZNF260;ZNF263;ZNF264;ZNF268;ZNF274;ZNF276;ZNF28;ZNF280A;ZNF280B;ZNF280C;ZNF280D;ZNF281;ZNF282;ZNF283;ZNF284;ZNF285;ZNF286A;ZNF286B;ZNF3;ZNF30;ZNF300;ZNF302;ZNF317;ZNF32;ZNF320;ZNF322;ZNF324;ZNF324B;ZNF329;ZNF331;ZNF333;ZNF33A;ZNF33B;ZNF343;ZNF345;ZNF347;ZNF350;ZNF354A;ZNF354B;ZNF362;ZNF366;ZNF383;ZNF384;ZNF394;ZNF397;ZNF398;ZNF404;ZNF41;ZNF410;ZNF419;ZNF420;ZNF431;ZNF432;ZNF436;ZNF439;ZNF44;ZNF440;ZNF442;ZNF443;ZNF446;ZNF449;ZNF45;ZNF460;ZNF468;ZNF479;ZNF484;ZNF490;ZNF500;ZNF502;ZNF524;ZNF525;ZNF528;ZNF543;ZNF544;ZNF546;ZNF547;ZNF548;ZNF549;ZNF554;ZNF555;ZNF557;ZNF558;ZNF559;ZNF561;ZNF562;ZNF563;ZNF564;ZNF566;ZNF567;ZNF568;ZNF57;ZNF570;ZNF571;ZNF572;ZNF577;ZNF581;ZNF582;ZNF583;ZNF585A;ZNF586;ZNF589;ZNF595;ZNF599;ZNF600;ZNF605;ZNF607;ZNF611;ZNF613;ZNF614;ZNF615;ZNF616;ZNF619;ZNF620;ZNF621;ZNF625;ZNF627;ZNF649;ZNF652;ZNF653;ZNF665;ZNF667;ZNF669;ZNF670;ZNF672;ZNF679;ZNF680;ZNF683;ZNF689;ZNF692;ZNF701;ZNF705D;ZNF705E;ZNF705G;ZNF708;ZNF709;ZNF71;ZNF710;ZNF713;ZNF721;ZNF727;ZNF729;ZNF736;ZNF75A;ZNF75D;ZNF76;ZNF763;ZNF764;ZNF765;ZNF768;ZNF77;ZNF771;ZNF773;ZNF774;ZNF776;ZNF777;ZNF780A;ZNF780B;ZNF782;ZNF785;ZNF799;ZNF805;ZNF808;ZNF81;ZNF813;ZNF816;ZNF823;ZNF829;ZNF836;ZNF841;ZNF844;ZNF845;ZNF846;ZNF85;ZNF853;ZNF860;ZNF878;ZNF891;ZNF99;ZSCAN16;ZSCAN2;ZSCAN22;ZSCAN23;ZSCAN29;ZSCAN31;ZSCAN32;ZSCAN4;ZSCAN5A;ZSCAN5B;ZSCAN5C;ZSCAN9;ZXDA;ZXDB;ZXDC	ZKSCAN1;ZNF232;ZSCAN31
BPTF_HUMAN.H11MO.0.D	26	0.15204678	0.7552388	1.824.308	1,20E+07	0.42539929	0.6679895	NA	NA	BPTF	BPTF
IKZF1_HUMAN.H11MO.0.C	28	0.16374269	0.7546370	1.773.335	1,18E+07	0.44315405	0.6757909	Factors with multiple dispersed zinc fingers{2.3.4}	Ikaros{2.3.4.4}	BCL11A;BCL11B;BNC1;BNC2;E4F1;HIC1;HIC2;HINFP;HIVEP1;HIVEP2;HIVEP3;IKZF1;IKZF2;IKZF3;IKZF4;IKZF5;INSM1;INSM2;MAZ;MECOM;PATZ1;PRDM16;PRDM4;REST;RLF;RREB1;SALL1;SALL2;SALL3;SALL4;VEZF1;ZBTB1;ZBTB17;ZBTB2;ZBTB25;ZBTB4;ZFAT;ZNF134;ZNF211;ZNF217;ZNF219;ZNF248;ZNF256;ZNF292;ZNF296;ZNF319;ZNF334;ZNF335;ZNF341;ZNF37A;ZNF382;ZNF417;ZNF418;ZNF423;ZNF467;ZNF510;ZNF512;ZNF512B;ZNF516;ZNF518A;Z	IKZF1

motif	Nº of probes	% of probes	lower OR	upper OR	OR	p.value	FDR	TF family	TF subfamily	TF.family.member	TF.subfamily.member
										NF518B;ZNF521;ZNF526;ZNF532;ZNF536;ZNF552;ZNF574;ZNF587;ZNF587B;ZNF592;ZNF639;ZNF654;ZNF658;ZNF671;ZNF687;ZNF711;ZNF717;ZNF770;ZNF772;ZNF784;ZNF786;ZNF792;ZNF8;ZNF814	
STAT3_HUMAN.H11MO.0.A	20	0.11695906	0.7545793	2.034.387	1,27E+07	0.29543086	0.5334361	STAT factors{6.2.1}	STAT3{6.2.1.0.3}	STAT1;STAT2;STAT3;STAT4;STAT5A;STAT5B;STAT6	STAT3
BCL6B_HUMAN.H11MO.0.D	18	0.10526316	0.7541789	2.137.105	1,31E+07	0.26617258	0.4968985	More than 3 adjacent zinc finger factors{2.3.3}	BCL6 factors{2.3.3.22}	BCL6;BCL6B;CTCF;CTCFL;FEZF1;FEZF2;GFI1;GFI1B;GLI1;GLI2;GLI3;GLI4;GLIS1;GLIS2;GLIS3;HKR1;MTF1;MYNN;MZF1;OSR2;OVOL1;OVOL2;PLAG1;PLAGL1;PLAGL2;PRDM1;PRDM14;PRDM6;SCRT1;SCRT2;SNAI1;SNAI2;SNAI3;WT1;YY1;YY2;ZBTB12;ZBTB14;ZBTB18;ZBTB20;ZBTB26;ZBTB42;ZBTB45;ZBTB47;ZBTB48;ZBTB49;ZBTB6;ZBTB7A;ZBTB7B;ZBTB7C;ZFP14;ZFP2;ZFP28;ZFP30;ZFP37;ZFP42;ZFP64;ZFP69;ZFP69B;ZFP82;ZFP91;ZFX;ZIC1;ZIC2;ZIC3;ZIC4;ZIC5;ZIK1;ZIM3;ZKSCAN1;ZKSCAN2;ZKSCAN3;ZKSCAN4;ZNF121;ZNF124;ZNF133;ZNF136;ZNF138;ZNF14;ZNF140;ZNF143;ZNF146;ZNF148;ZNF155;ZNF157;ZNF160;ZNF169;ZNF175;ZNF177;ZNF18;ZNF180;ZNF181;ZNF2;ZNF20;ZNF212;ZNF213;ZNF214;ZNF221;ZNF222;ZNF223;ZNF224;ZNF225;ZNF226;ZNF227;ZNF229;ZNF230;ZNF232;ZNF233;ZNF234;ZNF235;ZNF24;ZNF25;ZNF250;ZNF257;ZNF26;ZNF260;ZNF263;ZNF264;ZNF268;ZNF274;ZNF276;ZNF28;ZNF280A;ZNF280B;ZNF280C;ZNF280D;ZNF281;ZNF282;ZNF283;ZNF284;ZNF285;ZNF286A;ZNF286B;ZNF3;ZNF30;ZNF300;ZNF302;ZNF317;ZNF32;ZNF320;ZNF322;ZNF324;ZNF324B;ZNF329;ZNF331;ZNF333;ZNF33A;ZNF33B;ZNF343;ZNF345;ZNF347;ZNF350;ZNF354A;ZNF354B;ZNF362;ZNF366;ZNF383;ZNF384;ZNF394;ZNF397;ZNF398;ZNF404;ZNF41;ZNF410;ZNF419;ZNF420;ZNF431;ZNF432;ZNF436;ZNF439;ZNF44;ZNF440;ZNF442;ZNF443;ZNF446;ZNF449;ZNF45;ZNF460;ZNF468;ZNF479;ZNF484;ZNF490;ZNF500;ZNF502;ZNF524;ZNF525;ZNF528;ZNF543;ZNF544;ZNF546;ZNF547;ZNF548;ZNF549;ZNF554;ZNF555;ZNF557;ZNF558;ZNF559;ZNF561;ZNF562;ZNF563;ZNF564;ZNF566;ZNF567;ZNF568;ZNF57;ZNF570;ZNF571;ZNF572;ZNF577;ZNF581;ZNF582;ZNF583;ZNF585A;ZNF586;ZNF589;ZNF595;ZNF599;ZNF600;ZNF605;ZNF607;ZNF611;ZNF613;ZNF614;ZNF615;ZNF616;ZNF619;ZNF620;ZNF621;ZNF625;ZNF627;ZNF649;ZNF652;ZNF653;ZNF665;ZNF667;ZNF669;ZNF670;ZNF672;ZNF679;ZNF680;ZNF683;ZNF689;ZNF692;ZNF701;ZNF705D;ZNF705E;ZNF705G;ZNF708;ZNF709;ZNF71;ZNF710;ZNF713;ZNF721;ZNF727;ZNF729;ZNF736;ZNF75A;ZNF75D;ZNF76;ZNF763;ZNF764;ZNF765;ZNF768;ZNF77;ZNF771;ZNF773;ZNF774;ZNF776;ZNF777;ZNF780A;ZNF780B;ZNF782;ZNF785;ZNF799;ZNF805;ZNF808;ZNF81;ZNF813;ZNF816;ZNF823;ZNF829;ZNF836;ZNF841;ZNF844;ZNF845;ZNF846;ZNF85;ZNF853;ZNF860;ZNF878;ZNF891;ZNF99;ZSCAN16;ZSCAN2;ZSCAN22;ZSCAN23;ZSCAN29;ZSCAN31;ZSCAN32;ZSCAN4;ZSCAN5A;ZSCAN5B;ZSCAN5C;ZSCAN9;ZXDA;ZXDB;ZXDC	BCL6B;BCL6

motif	Nº of probes	% of probes	lower OR	upper OR	OR	p.value	FDR	TF family	TF subfamily	TF.family.member	TF.subfamily.member
HXA10_HUMAN.H11MO.0.C	14	0.08187135	0.7537375	2.440.865	1,41E+07	0.19676472	0.4309818	HOX-related factors{3.1.1}	HOX9-13{3.1.1.8}	CDX1;CDX2;CDX4;EVX1;EVX2;GBX1;GBX2;GSX1;GSX2;HDX;HMBOX1;HNF1A;HNF1B;HOXA1;HOXA10;HOXA11;HOXA13;HOXA2;HOXA3;HOXA4;HOXA5;HOXA6;HOXA7;HOXA9;HOXB1;HOXB13;HOXB2;HOXB3;HOXB4;HOXB5;HOXB6;HOXB7;HOXB8;HOXB9;HOXC10;HOXC11;HOXC12;HOXC13;HOXC4;HOXC5;HOXC6;HOXC8;HOXC9;HOXD1;HOXD10;HOXD11;HOXD12;HOXD13;HOXD3;HOXD4;HOXD8;HOXD9;MEOX1;MEOX2;MNX1;PDX1;POU1F1;POU2F1;POU2F2;POU2F3;POU3F1;POU3F2;POU3F3;POU3F4;POU4F1;POU4F2;POU4F3;POU5F1;POU5F2;POU6F1;POU6F2	HOXA10;HOXA11;HOXA13;HOXA9;HOXB13;HOXC10;HOXC11;HOXC12;HOXC13;HOXC9;HOXD10;HOXD11;HOXD12;HOXD13;HOXD9
ELK1_HUMAN.H11MO.0.B	32	0.18713450	0.7537147	1.691.583	1,14E+07	0.47376683	0.6984211	Ets-related factors{3.5.2}	Elk-like factors{3.5.2.2}	EHF;ELF1;ELF2;ELF3;ELF4;ELF5;ELK1;ELK3;ELK4;ERF;ERG;ETS1;ETS2;ETV1;ETV2;ETV3;ETV3L;ETV4;ETV5;ETV6;ETV7;FEV;FLI1;GABPA;SPDEF;SPI1;SPIB;SPIC	ELK1;ELK3;ELK4;ETV1;ETV4;ETV5
FOXM1_HUMAN.H11MO.0.A	21	0.12280702	0.7530246	1.986.006	1,25E+07	0.30974457	0.5528080	Forkhead box (FOX) factors{3.3.1}	FOXM{3.3.1.13}	FOXA1;FOXA2;FOXA3;FOXB1;FOXB2;FOXC1;FOXC2;FOXD1;FOXD2;FOXD3;FOXD4;FOXD4L1;FOXD4L3;FOXD4L4;FOXD4L5;FOXD4L6;FOXE1;FOXE3;FOXF1;FOXF2;FOXG1;FOXH1;FOXI1;FOXI2;FOXI3;FOXJ1;FOXJ2;FOXJ3;FOXK1;FOXK2;FOXL1;FOXL2;FOXM1;FOXN1;FOXN2;FOXN3;FOXN4;FOXO1;FOXO3;FOXO4;FOXO6;FOXP1;FOXP2;FOXP3;FOXP4;FOXQ1;FOXR1;FOXR2;FOXS1	FOXM1
P5F1B_HUMAN.H11MO.0.D	15	0.08771930	0.7526472	2.344.515	1,38E+07	0.21606491	0.4442848	POU domain factors{3.1.10}	POU5 (Oct-3/4-like factors){3.1.10.5}	HMBOX1;HNF1A;HNF1B;POU5F1B;POU1F1;POU2F1;POU2F2;POU2F3;POU3F1;POU3F2;POU3F3;POU3F4;POU4F1;POU4F2;POU4F3;POU5F1;POU6F1;POU6F2	POU5F1B;POU5F1
ELF5_HUMAN.H11MO.0.A	40	0.23391813	0.7515226	1.578.128	1,10E+07	0.57866306	0.7845295	Ets-related factors{3.5.2}	EHF-like factors{3.5.2.4}	EHF;ELF1;ELF2;ELF3;ELF4;ELF5;ELK1;ELK3;ELK4;ERF;ERG;ETS1;ETS2;ETV1;ETV2;ETV3;ETV3L;ETV4;ETV5;ETV6;ETV7;FEV;FLI1;GABPA;SPDEF;SPI1;SPIB;SPIC	EHF;ELF3;ELF5
VSX2_HUMAN.H11MO.0.D	11	0.06432749	0.7505128	2.822.796	1,53E+07	0.18093472	0.4107633	Paired-related HD factors{3.1.3}	VSX{3.1.3.28}	ALX1;ALX3;ALX4;ARGFX;ARX;CRX;DMBX1;DPRX;DRGX;DUX4;DUXA;ESX1;GSC;GSC2;HESX1;ISX;LEUTX;MIXL1;NOBOX;OTP;OTX1;OTX2;PHOX2A;PHOX2B;PITX1;PITX2;PITX3;PROP1;PRRX1;PRRX2;RAX;RAX2;RHOXF1;RHOXF2;SEBOX;SHOX;SHOX2;TPRX1;UNCX;VSX1;VSX2	VSX1;VSX2
MEIS2_HUMAN.H11MO.0.B	17	0.09941520	0.7472799	2.178.754	1,32E+07	0.25388171	0.4881367	TALE-type homeo domain factors{3.1.4}	MEIS{3.1.4.2}	IRX1;IRX2;IRX3;IRX4;IRX5;IRX6;MEIS1;MEIS2;MEIS3;MKX;PBX1;PBX2;PBX3;PKNOX1;PKNOX2;TGIF1;TGIF2;TGIF2LX;TGIF2LY	MEIS1;MEIS2;MEIS3
HNF1A_HUMAN.H11MO.0.C	13	0.07602339	0.7472445	2.527.732	1,44E+07	0.23251425	0.4644261	POU domain factors{3.1.10}	HNF1-like factors{3.1.10.7}	CDX1;CDX2;CDX4;EVX1;EVX2;GBX1;GBX2;GSX1;GSX2;HDX;HMBOX1;HNF1A;HNF1B;HOXA1;HOXA10;HOXA11;HOXA13;HOXA2;HOXA3;HOXA4;HOXA5;HOXA6;HOXA7;HOXA9;HOXB1;HOXB13;HOXB2;HOXB3;HOXB4;HOXB5;HOXB6;HOXB7;HOXB8;HOXB9;HOXC10;HOXC11;HOXC12;HOXC13;HOXC4;HOXC5;HOXC6;HOXC8;HOXC9;HOXD1;HOXD10;HOXD11;HOXD12;HOXD13;HOXD3;HOXD4;HOXD8;HOXD9;MEOX1;MEOX2;MNX1;PDX1;POU1F1;POU2F1;POU2F2;POU2F3;POU3F1;POU3F2;POU3F3;POU3F4;POU4F1;POU4F2;POU4F3;POU5F1;POU5F1B;POU5F2;POU6F1;POU6F2	HMBOX1;HNF1A;HNF1B
TF65_HUMAN.H11MO.0.A	17	0.09941520	0.7442996	2.170.064	1,31E+07	0.31424173	0.5572862	NF-kappaB-related factors{6.1.1}	NF-kappaB p65 subunit-like factors{6.1.1.2}	NFKB1;NFKB2;REL;RELA;RELB	RELB;REL;RELA

motif	Nº of probes	% of probes	lower OR	upper OR	OR	p.value	FDR	TF family	TF subfamily	TF.family.member	TF.subfamily.member
MAFG_HUMAN.H11MO.0.A	17	0.09941520	0.7436580	2.168.193	1,31E+07	0.31442220	0.5572862	Maf-related factors {1.1.3}	Small Maf factors{1.1.3.2}	MAF;MAFA;MAFB;MAFF;MAFG;MAFK;NRL	MAFF;MAFG;MAFK
MEF2C_HUMAN.H11MO.0.A	21	0.12280702	0.7430449	1.959.750	1,24E+07	0.37408708	0.6225095	Regulators of differentiation {5.1.1}	MEF-2{5.1.1.1}	MEF2A;MEF2B;MEF2C;MEF2D	MEF2A;MEF2B;MEF2C;MEF2D
LHX3_HUMAN.H11MO.0.C	12	0.07017544	0.7428568	2.640.285	1,47E+07	0.20965547	0.4413491	HD-LIM factors{3.1.5}	Lhx-3-like factors{3.1.5.4}	ISL1;ISL2;LHX1;LHX2;LHX3;LHX4;LHX5;LHX6;LHX8;LHX9;LMX1A;LMX1B	LHX3;LHX4
ZFP82_HUMAN.H11MO.0.C	25	0.14619883	0.7423127	1.820.539	1,19E+07	0.41998036	0.6662651	More than 3 adjacent zinc finger factors{2.3.3}	ZFP30-like factors{2.3.3.63}	BCL6;BCL6B;CTCF;CTCFL;FEZF1;FEZF2;GFI1;GFI1B;GLI1;GLI2;GLI3;GLI4;GLIS1;GLIS2;GLIS3;HKR1;MTF1;MYNN;MZF1;OSR2;OVOL1;OVOL2;PLAG1;PLAGL1;PLAGL2;PRDM1;PRDM14;PRDM6;SCRT1;SCRT2;SNAI1;SNAI2;SNAI3;WT1;YY1;YY2;ZBTB12;ZBTB14;ZBTB18;ZBTB20;ZBTB26;ZBTB42;ZBTB45;ZBTB47;ZBTB48;ZBTB49;ZBTB6;ZBTB7A;ZBTB7B;ZBTB7C;ZFP14;ZFP2;ZFP28;ZFP30;ZFP37;ZFP42;ZFP64;ZFP69;ZFP69B;ZFP82;ZFP91;ZFX;ZIC1;ZIC2;ZIC3;ZIC4;ZIC5;ZIK1;ZIM3;ZKSCAN1;ZKSCAN2;ZKSCAN3;ZKSCAN4;ZNF121;ZNF124;ZNF133;ZNF136;ZNF138;ZNF14;ZNF140;ZNF143;ZNF146;ZNF148;ZNF155;ZNF157;ZNF160;ZNF169;ZNF175;ZNF177;ZNF18;ZNF180;ZNF181;ZNF2;ZNF20;ZNF212;ZNF213;ZNF214;ZNF221;ZNF222;ZNF223;ZNF224;ZNF225;ZNF226;ZNF227;ZNF229;ZNF230;ZNF232;ZNF233;ZNF234;ZNF235;ZNF24;ZNF25;ZNF250;ZNF257;ZNF26;ZNF260;ZNF263;ZNF264;ZNF268;ZNF274;ZNF276;ZNF28;ZNF280A;ZNF280B;ZNF280C;ZNF280D;ZNF281;ZNF282;ZNF283;ZNF284;ZNF285;ZNF286A;ZNF286B;ZNF3;ZNF30;ZNF300;ZNF302;ZNF317;ZNF32;ZNF320;ZNF322;ZNF324;ZNF324B;ZNF329;ZNF331;ZNF333;ZNF33A;ZNF33B;ZNF343;ZNF345;ZNF347;ZNF350;ZNF354A;ZNF354B;ZNF362;ZNF366;ZNF383;ZNF384;ZNF394;ZNF397;ZNF398;ZNF404;ZNF41;ZNF410;ZNF419;ZNF420;ZNF431;ZNF432;ZNF436;ZNF439;ZNF44;ZNF440;ZNF442;ZNF443;ZNF446;ZNF449;ZNF45;ZNF460;ZNF468;ZNF479;ZNF484;ZNF490;ZNF500;ZNF502;ZNF524;ZNF525;ZNF528;ZNF543;ZNF544;ZNF546;ZNF547;ZNF548;ZNF549;ZNF554;ZNF555;ZNF557;ZNF558;ZNF559;ZNF561;ZNF562;ZNF563;ZNF564;ZNF566;ZNF567;ZNF568;ZNF57;ZNF570;ZNF571;ZNF572;ZNF577;ZNF581;ZNF582;ZNF583;ZNF585A;ZNF586;ZNF589;ZNF595;ZNF599;ZNF600;ZNF605;ZNF607;ZNF611;ZNF613;ZNF614;ZNF615;ZNF616;ZNF619;ZNF620;ZNF621;ZNF625;ZNF627;ZNF649;ZNF652;ZNF653;ZNF665;ZNF667;ZNF669;ZNF670;ZNF672;ZNF679;ZNF680;ZNF683;ZNF689;ZNF692;ZNF701;ZNF705D;ZNF705E;ZNF705G;ZNF708;ZNF709;ZNF71;ZNF710;ZNF713;ZNF721;ZNF727;ZNF729;ZNF736;ZNF75A;ZNF75D;ZNF76;ZNF763;ZNF764;ZNF765;ZNF768;ZNF77;ZNF771;ZNF773;ZNF774;ZNF776;ZNF777;ZNF780A;ZNF780B;ZNF782;ZNF785;ZNF799;ZNF805;ZNF808;ZNF81;ZNF813;ZNF816;ZNF823;ZNF829;ZNF836;ZNF841;ZNF844;ZNF845;ZNF846;ZNF85;ZNF853;ZNF860;ZNF878;ZNF891;ZNF99;ZSCAN16;ZSCAN2;ZSCAN22;ZSCAN23;ZSCAN29;ZSCAN31;ZSCAN32;ZSCAN4;ZSCAN5A;ZSCAN5B;ZSCAN5C;ZSCAN9;ZXDA;ZXDB;ZXDC	ZFP82

motif	Nº of probes	% of probes	lower OR	upper OR	OR	p.value	FDR	TF family	TF subfamily	TF.family.member	TF.subfamily.member
MZF1_HUMAN.H11MO.0.B	27	0.15789474	0.7420074	1.767.095	1,17E+07	0.4393205	0.6751669	More than 3 adjacent zinc finger factors{2.3.3}	unclassified{2.3.3.0}	BCL6B;BCL6;CTCFL;CTCF;FEZF1;GFI1B;GFI1;GLI1;GLI2;GLI3;GLIS1;GLIS2;GLIS3;MTF1;MYNN;MZF1;OSR2;OVOL1;OVOL2;ZNF146;PLAG1;PLAGL1;PRDM14;PRDM1;PRDM6;SCRT1;SCRT2;SNAI1;SNAI2;YY1;YY2;WT1;ZNF324;ZNF354A;ZBTB14;ZBTB18;ZBTB48;ZBTB49;ZBTB7A;ZBTB7B;ZBTB6;ZFP64;ZFP28;ZFP42;ZFP82;ZFX;ZIC1;ZIC2;ZIC3;ZIC4;ZIM3;ZKSCAN1;ZKSCAN3;ZNF121;ZNF136;ZNF140;ZNF143;ZNF148;ZNF214;ZNF232;ZNF250;ZNF257;ZNF260;ZNF263;ZNF264;ZNF274;ZNF281;ZNF282;ZNF317;ZNF320;ZNF322;ZNF329;ZNF331;ZNF333;ZNF350;ZNF384;ZNF394;ZNF410;ZNF436;ZNF449;ZNF490;ZNF502;ZNF524;ZNF528;ZNF547;ZNF549;ZNF554;ZNF563;ZNF582;ZNF586;ZNF589;ZNF652;ZNF667;ZNF680;ZNF708;ZNF713;ZNF768;ZNF816;ZNF18;ZNF41;ZNF76;ZNF85;ZSCAN16;ZSCAN22;ZSCAN31;ZSCAN4	MYNN;MZF1;OSR2;PRDM14;PRDM6;WT1;ZBTB14;ZBTB48;ZBTB49;ZFP64;ZFP28;ZIM3;ZNF121;ZNF250;ZNF257;ZNF263;ZNF274;ZNF317;ZNF320;ZNF329;ZNF331;ZNF394;ZNF449;ZNF502;ZNF528;ZNF547;ZNF549;ZNF554;ZNF586;ZNF589;ZNF667;ZNF680;ZNF708;ZNF713;ZNF768;ZNF18;ZNF85;ZSCAN16;ZSCAN22
ATF1_HUMAN.H11MO.0.B	26	0.15204678	0.7407597	1.789.375	1,17E+07	0.4305493	0.6712382	CREB-related factors{1.1.7}	CREB-like factors{1.1.7.1}	ATF1;ATF6;ATF6B;CREB1;CREB3;CREB3L1;CREB3L2;CREB3L3;CREB3L4;CREBL2;CREBZF;CREM	ATF1;CREB1;CREM
FOXF2_HUMAN.H11MO.0.D	19	0.11111111	0.7400297	2.043.190	1,26E+07	0.3474473	0.5984976	Forkhead box (FOX) factors{3.3.1}	FOXF{3.3.1.6}	FOXA1;FOXA2;FOXA3;FOXB1;FOXB2;FOXC1;FOXC2;FOXD1;FOXD2;FOXD3;FOXD4;FOXD4L1;FOXD4L3;FOXD4L4;FOXD4L5;FOXD4L6;FOXE1;FOXE3;FOXF1;FOXF2;FOXG1;FOXH1;FOXI1;FOXI2;FOXI3;FOXJ1;FOXJ2;FOXJ3;FOXK1;FOXK2;FOXL1;FOXL2;FOXM1;FOXN1;FOXN2;FOXN3;FOXN4;FOXO1;FOXO3;FOXO4;FOXO6;FOXP1;FOXP2;FOXP3;FOXP4;FOXQ1;FOXR1;FOXR2;FOXS1	FOXF1;FOXF2
MAF_HUMAN.H11MO.0.A	21	0.12280702	0.7395404	1.950.537	1,23E+07	0.3753174	0.6225095	Maf-related factors{1.1.3}	Large Maf factors{1.1.3.1}	MAF;MAFA;MAFB;MAFF;MAFG;MAFK;NRL	MAFA;MAFB;MAF;NRL
CDX2_HUMAN.H11MO.0.A	13	0.07602339	0.7374684	2.494.468	1,42E+07	0.2359051	0.4686829	HOX-related factors{3.1.1}	CDX (Caudal type homeobox){3.1.1.9}	CDX1;CDX2;CDX4;EVX1;EVX2;GBX1;GBX2;GSX1;GSX2;HDX;HMBOX1;HNF1A;HNF1B;HOXA1;HOXA10;HOXA11;HOXA13;HOXA2;HOXA3;HOXA4;HOXA5;HOXA6;HOXA7;HOXA9;HOXB1;HOXB13;HOXB2;HOXB3;HOXB4;HOXB5;HOXB6;HOXB7;HOXB8;HOXB9;HOXC10;HOXC11;HOXC12;HOXC13;HOXC4;HOXC5;HOXC6;HOXC8;HOXC9;HOXD1;HOXD10;HOXD11;HOXD12;HOXD13;HOXD3;HOXD4;HOXD8;HOXD9;MEOX1;MEOX2;MNX1;PDX1;POU1F1;POU2F1;POU2F2;POU2F3;POU3F1;POU3F2;POU3F3;POU3F4;POU4F1;POU4F2;POU4F3;POU5F1;POU5F2;POU6F1;POU6F2	CDX1;CDX2
TF7L1_HUMAN.H11MO.0.B	14	0.08187135	0.7374221	2.387.978	1,38E+07	0.2584171	0.4895321	TCF-7-related factors{4.1.3}	TCF-7L1 (TCF-3) [1]{4.1.3.0.2}	LEF1;TAF1;TCF7;TCF7L1;TCF7L2	TCF7L1
TBX2_HUMAN.H11MO.0.D	26	0.15204678	0.7364036	1.778.740	1,17E+07	0.4985741	0.7144993	TBX2-related factors{6.5.4}	TBX2{6.5.4.0.1}	TBX2;TBX3;TBX4;TBX5	TBX2
ETV5_HUMAN.H11MO.0.C	46	0.26900585	0.7352100	1.490.556	1,05E+07	0.7276473	0.8770496	Ets-related factors{3.5.2}	Elk-like factors{3.5.2.2}	EHF;ELF1;ELF2;ELF3;ELF4;ELF5;ELK1;ELK3;ELK4;ERF;ERG;ETS1;ETS2;ETV1;ETV2;ETV3;ETV3L;ETV4;ETV5;ETV6;ETV7;FEV;FLI1;GABPA;SPDEF;SPI1;SPIB;SPIC	ELK1;ELK3;ELK4;ETV1;ETV4;ETV5
ZN134_HUMAN.H11MO.0.C	22	0.12865497	0.7330658	1.894.532	1,21E+07	0.3907489	0.6285525	Factors with multiple dispersed zinc fingers{2.3.4}	ZNF134-like factors{2.3.4.24}	BCL11A;BCL11B;BNC1;BNC2;E4F1;HIC1;HIC2;HINFP;HIVEP1;HIVEP2;HIVEP3;IKZF1;IKZF2;IKZF3;IKZF4;IKZF5;INSM1;INSM2;MAZ;MECOM;PATZ1;PRDM16;PRDM4;REST;RLF;RREB1;SALL1;SALL2;SALL3;SALL4;VEZF1;ZBTB1;ZBTB17;ZBTB2;ZBTB25;ZBTB4;ZFAT;ZNF134;ZNF211;ZNF217;ZNF219;ZNF248;ZNF256;ZNF292;ZNF296;ZNF319;ZNF334;ZNF335;ZNF341;ZNF37A;ZNF382;ZNF417;ZNF418;ZNF423;ZNF467	ZNF134

motif	Nº of probes	% of probes	lower OR	upper OR	OR	p.value	FDR	TF family	TF subfamily	TF.family.member	TF.subfamily.member
										;ZNF510;ZNF512;ZNF512B;ZNF516;ZNF518A;ZNF518B;ZNF521;ZNF526;ZNF532;ZNF536;ZNF552;ZNF574;ZNF587;ZNF587B;ZNF592;ZNF639;ZNF654;ZNF658;ZNF671;ZNF687;ZNF711;ZNF717;ZNF770;ZNF772;ZNF784;ZNF786;ZNF792;ZNF8;ZNF814	
LBX2_HUMAN.H11MO.0.D	9	0.05263158	0.7327921	3.179.920	1,63E+07	0.1904633	0.4238693	NK-related factors{3.1.2}	LBX{3.1.2.10}	BARHL1;BARHL2;BARX1;BARX2;BSX;DBX1;DBX2;DLX1;DLX2;DLX3;DLX4;DLX5;DLX6;EMX1;EMX2;EN1;EN2;HHEX;HLX;HMX1;HMX2;HMX3;LBX1;LBX2;MSX1;MSX2;NANOG;NKX1-1;NKX1-2;NKX2-1;NKX2-2;NKX2-3;NKX2-4;NKX2-5;NKX2-6;NKX2-8;NKX3-1;NKX3-2;NKX6-1;NKX6-2;NKX6-3;NOTO;TLX1;TLX2;TLX3;VAX1;VAX2;VENTX	LBX2
MYNN_HUMAN.H11MO.0.D	13	0.07602339	0.7327785	2.478.567	1,41E+07	0.2378032	0.4686829	More than 3 adjacent zinc finger factors{2.3.3}	unclassified{2.3.3.0}	BCL6B;BCL6;CTCFL;CTCF;FEZF1;GFI1B;GFI1;GLI1;GLI2;GLI3;GLIS1;GLIS2;GLIS3;MTF1;MYNN;MZF1;OSR2;OVOL1;OVOL2;ZNF146;PLAG1;PLAGL1;PRDM14;PRDM1;PRDM6;SCRT1;SCRT2;SNAI1;SNAI2;YY1;YY2;WT1;ZNF324;ZNF354A;ZBTB14;ZBTB18;ZBTB48;ZBTB49;ZBTB7A;ZBTB7B;ZBTB6;ZFP64;ZFP28;ZFP42;ZFP82;ZFX;ZIC1;ZIC2;ZIC3;ZIC4;ZIM3;ZKSCAN1;ZKSCAN3;ZNF121;ZNF136;ZNF140;ZNF143;ZNF148;ZNF214;ZNF232;ZNF250;ZNF257;ZNF260;ZNF263;ZNF264;ZNF274;ZNF281;ZNF282;ZNF317;ZNF320;ZNF322;ZNF329;ZNF331;ZNF333;ZNF350;ZNF384;ZNF394;ZNF410;ZNF436;ZNF449;ZNF490;ZNF502;ZNF524;ZNF528;ZNF547;ZNF549;ZNF554;ZNF563;ZNF582;ZNF586;ZNF589;ZNF652;ZNF667;ZNF680;ZNF708;ZNF713;ZNF768;ZNF816;ZNF18;ZNF41;ZNF76;ZNF85;ZSCAN16;ZSCAN22;ZSCAN31;ZSCAN4	MYNN;MZF1;OSR2;PRDM14;PRDM6;WT1;ZBTB14;ZBTB48;ZBTB49;ZFP64;ZFP28;ZIM3;ZNF121;ZNF250;ZNF257;ZNF263;ZNF274;ZNF317;ZNF320;ZNF329;ZNF331;ZNF394;ZNF449;ZNF502;ZNF528;ZNF547;ZNF549;ZNF554;ZNF586;ZNF589;ZNF667;ZNF680;ZNF708;ZNF713;ZNF768;ZNF18;ZNF85;ZSCAN16;ZSCAN22
FOXO1_HUMAN.H11MO.0.A	24	0.14035088	0.7319101	1.824.350	1,18E+07	0.4813647	0.7038799	Forkhead box (FOX) factors{3.3.1}	FOXO{3.3.1.15}	FOXA1;FOXA2;FOXA3;FOXB1;FOXB2;FOXC1;FOXC2;FOXD1;FOXD2;FOXD3;FOXD4;FOXD4L1;FOXD4L3;FOXD4L4;FOXD4L5;FOXD4L6;FOXE1;FOXE3;FOXF1;FOXF2;FOXG1;FOXH1;FOXI1;FOXI2;FOXI3;FOXJ1;FOXJ2;FOXJ3;FOXK1;FOXK2;FOXL1;FOXL2;FOXM1;FOXN1;FOXN2;FOXN3;FOXN4;FOXO1;FOXO3;FOXO4;FOXO6;FOXP1;FOXP2;FOXP3;FOXP4;FOXQ1;FOXR1;FOXR2;FOXS1	FOXO1;FOXO3;FOXO4;FOXO6
SOX4_HUMAN.H11MO.0.B	24	0.14035088	0.7291037	1.817.384	1,17E+07	0.4820345	0.7038799	SOX-related factors{4.1.1}	Group C{4.1.1.3}	BBX;CIC;HBP1;SOX1;SOX10;SOX11;SOX12;SOX13;SOX14;SOX15;SOX17;SOX18;SOX2;SOX21;SOX3;SOX30;SOX4;SOX5;SOX6;SOX7;SOX8;SOX9;SRY	SOX11;SOX4
TEAD1_HUMAN.H11MO.0.A	19	0.11111111	0.7285976	2.011.635	1,24E+07	0.3521579	0.6006941	TEF-1-related factors{3.6.1}	TEF-1 (TEAD-1, TCF-13){3.6.1.0.1}	TEAD1;TEAD2;TEAD3;TEAD4	TEAD1
SRF_HUMAN.H11MO.0.A	11	0.06432749	0.7276294	2.736.639	1,49E+07	0.1907687	0.4238693	Responders to external signals (SRF/RLM1){5.1.2}	SRF{5.1.2.0.1}	SRF	SRF
ESR2_HUMAN.H11MO.1.A	17	0.09941520	0.7265518	2.118.335	1,28E+07	0.3209473	0.5649551	Steroid hormone receptors (NR3){2.1.1}	ER-like receptors (NR3A&B){2.1.1.2}	AR;ESR1;ESR2;ESRRA;ESRRB;ESRRG;NR3C1;NR3C2;PGR	ESRRA;ESRRB;ESRRG;ESR1;ESR2
GCR_HUMAN.H11MO.0.A	21	0.12280702	0.7256135	1.913.676	1,21E+07	0.3819499	0.6241319	Steroid hormone receptors (NR3){2.1.1}	GR-like receptors (NR3C){2.1.1.1}	AR;ESR1;ESR2;ESRRA;ESRRB;ESRRG;NR3C1;NR3C2;PGR	AR;NR3C1;NR3C2;PGR
MYBA_HUMAN.H11MO.0.D	24	0.14035088	0.7253382	1.807.995	1,17E+07	0.4831274	0.7041422	Myb/SANT domain factors{3.5.1}	Myb-like factors{3.5.1.1}	CDC5L;DMTF1;MYB;MYBL1;MYBL2;SMARCA1;SMARCA5;SNAPC4;TRERF1;ZNF541	CDC5L;MYBL1;MYBL2;MYB

motif	Nº of probes	% of probes	lower OR	upper OR	OR	p.value	FDR	TF family	TF subfamily	TF.family.member	TF.subfamily.member
HAND1_HUMAN.H11MO.0.D	28	0.16374269	0.7245604	1.702.621	1,13E+07	0.5191454	0.7365048	Tal-related factors{1.2.3}	Twist-like factors{1.2.3.2}	ATOH1;ATOH7;ATOH8;BHLHA15;BHLHA9;BHLHE22;BHLHE23;FERD3L;FIGLA;HAND1;HAND2;LYL1;MESP1;MESP2;MSC;MSGN1;NEUROD1;NEUROD2;NEUROD4;NEUROD6;NEUROG1;NEUROG2;NEUROG3;NHLH1;NHLH2;OLIG1;OLIG2;OLIG3;PTF1A;SCX;TAL1;TAL2;TCF15;TCF21;TCF23;TWIST1;TWIST2	FIGLA;HAND1;PTF1A;TWIST1
GATA3_HUMAN.H11MO.0.A	18	0.10526316	0.7240080	2.051.477	1,25E+07	0.3389535	0.5899169	GATA-type zinc fingers{2.2.1}	Two zinc-finger GATA factors{2.2.1.1}	GATA1;GATA2;GATA3;GATA4;GATA5;GATA6;GATAD2A;GATAD2B;TRPS1;ZGLP1	GATA1;GATA2;GATA3;GATA4;GATA5;GATA6
GATA2_HUMAN.H11MO.0.A	15	0.08771930	0.7225540	2.250.801	1,32E+07	0.2856842	0.5218212	GATA-type zinc fingers{2.2.1}	Two zinc-finger GATA factors{2.2.1.1}	GATA1;GATA2;GATA3;GATA4;GATA5;GATA6;GATAD2A;GATAD2B;TRPS1;ZGLP1	GATA1;GATA2;GATA3;GATA4;GATA5;GATA6
HXC9_HUMAN.H11MO.0.C	12	0.07017544	0.7211474	2.562.936	1,43E+07	0.2194241	0.4482521	HOX-related factors{3.1.1}	HOX9-13{3.1.1.8}	CDX1;CDX2;CDX4;EVX1;EVX2;GBX1;GBX2;GSX1;GSX2;HDX;HMBOX1;HNF1A;HNF1B;HOXA1;HOXA10;HOXA11;HOXA13;HOXA2;HOXA3;HOXA4;HOXA5;HOXA6;HOXA7;HOXA9;HOXB1;HOXB13;HOXB2;HOXB3;HOXB4;HOXB5;HOXB6;HOXB7;HOXB8;HOXB9;HOXC10;HOXC11;HOXC12;HOXC13;HOXC4;HOXC5;HOXC6;HOXC8;HOXC9;HOXD1;HOXD10;HOXD11;HOXD12;HOXD13;HOXD3;HOXD4;HOXD8;HOXD9;MEOX1;MEOX2;MNX1;PDX1;POU1F1;POU2F1;POU2F2;POU2F3;POU3F1;POU3F2;POU3F3;POU3F4;POU4F1;POU4F2;POU4F3;POU5F1;POU5F2;POU6F1;POU6F2	HOXA10;HOXA11;HOXA13;HOXA9;HOXB13;HOXC10;HOXC11;HOXC12;HOXC13;HOXC9;HOXD10;HOXD11;HOXD12;HOXD13;HOXD9
ZN708_HUMAN.H11MO.1.D	34	0.19883041	0.7208330	1.586.825	1,08E+07	0.6941380	0.8678943	More than 3 adjacent zinc finger factors{2.3.3}	unclassified{2.3.3.0}	BCL6B;BCL6;CTCFL;CTCF;FEZF1;GFI1B;GFI1;GLI1;GLI2;GLI3;GLIS1;GLIS2;GLIS3;MTF1;MYNN;MZF1;OSR2;OVOL1;OVOL2;ZNF146;PLAG1;PLAGL1;PRDM14;PRDM1;PRDM6;SCRT1;SCRT2;SNAI1;SNAI2;YY1;YY2;WT1;ZNF324;ZNF354A;ZBTB14;ZBTB18;ZBTB48;ZBTB49;ZBTB7A;ZBTB7B;ZBTB6;ZFP64;ZFP28;ZFP42;ZFP82;ZFX;ZIC1;ZIC2;ZIC3;ZIC4;ZIM3;ZKSCAN1;ZKSCAN3;ZNF121;ZNF136;ZNF140;ZNF143;ZNF148;ZNF214;ZNF232;ZNF250;ZNF257;ZNF260;ZNF263;ZNF264;ZNF274;ZNF281;ZNF282;ZNF317;ZNF320;ZNF322;ZNF329;ZNF331;ZNF333;ZNF350;ZNF384;ZNF394;ZNF410;ZNF436;ZNF449;ZNF490;ZNF502;ZNF524;ZNF528;ZNF547;ZNF549;ZNF554;ZNF563;ZNF582;ZNF586;ZNF589;ZNF652;ZNF667;ZNF680;ZNF708;ZNF713;ZNF768;ZNF816;ZNF18;ZNF41;ZNF76;ZNF85;ZSCAN16;ZSCAN22;ZSCAN31;ZSCAN4	MYNN;MZF1;OSR2;PRDM14;PRDM6;WT1;ZBTB14;ZBTB48;ZBTB49;ZFP64;ZFP28;ZIM3;ZNF121;ZNF250;ZNF257;ZNF263;ZNF274;ZNF317;ZNF320;ZNF329;ZNF331;ZNF394;ZNF449;ZNF502;ZNF528;ZNF547;ZNF549;ZNF554;ZNF586;ZNF589;ZNF667;ZNF680;ZNF708;ZNF713;ZNF768;ZNF18;ZNF85;ZSCAN16;ZSCAN22
MAFK_HUMAN.H11MO.1.A	27	0.15789474	0.7207829	1.716.477	1,13E+07	0.5831317	0.7848089	Maf-related factors{1.1.3}	Small Maf factors{1.1.3.2}	MAF;MAFA;MAFB;MAFF;MAFG;MAFK;NRL	MAFF;MAFG;MAFK
HXD13_HUMAN.H11MO.0.D	15	0.08771930	0.7205267	2.244.490	1,32E+07	0.2867337	0.5218212	HOX-related factors{3.1.1}	HOX9-13{3.1.1.8}	CDX1;CDX2;CDX4;EVX1;EVX2;GBX1;GBX2;GSX1;GSX2;HDX;HMBOX1;HNF1A;HNF1B;HOXA1;HOXA10;HOXA11;HOXA13;HOXA2;HOXA3;HOXA4;HOXA5;HOXA6;HOXA7;HOXA9;HOXB1;HOXB13;HOXB2;HOXB3;HOXB4;HOXB5;HOXB6;HOXB7;HOXB8;HOXB9;HOXC10;HOXC11;HOXC12;HOXC13;HOXC4;HOXC5;HOXC6;HOXC8;HOXC9;HOXD1;HOXD10;HOXD11;HOXD12;HOXD13;HOXD3;HOXD4;HOXD8;HOXD9;MEOX1;MEOX2;MNX1;PDX1;POU1F1;POU2F1;POU2F2;POU2F3;POU3F1;POU3F2;POU3F3;POU3F4;POU4F1;POU4F2;POU4F3;POU5F1;POU5F2;POU6F1;POU6F2	HOXA10;HOXA11;HOXA13;HOXA9;HOXB13;HOXC10;HOXC11;HOXC12;HOXC13;HOXC9;HOXD10;HOXD11;HOXD12;HOXD13;HOXD9
NR4A3_HUMAN.H11MO.0.D	19	0.11111111	0.7205148	1.989.128	1,23E+07	0.3565536	0.6068495	NGFI-B-related receptors (NR4){2.1.4}	NOR1 (NR4A3){2.1.4.0.3}	NR4A1;NR4A2;NR4A3	NR4A3

motif	Nº of probes	% of probes	lower OR	upper OR	OR	p.value	FDR	TF family	TF subfamily	TF.family.member	TF.subfamily.member
E4F1_HUMAN.H11MO.0.D	28	0.16374269	0.7183412	1.688.004	1,12E+07	0.5905518	0.7863825	Factors with multiple dispersed zinc fingers{2.3.4}	unclassified{2.3.4.0}	BCL11A;E4F1;MECOM;HIC1;HIC2;HINFP;IKZF1;INSM1;MAZ;PATZ1;PRDM4;REST;RREB1;SALL4;VEZF1;ZBTB17;ZBTB4;HIVEP1;HIVEP2;ZNF134;ZNF219;ZNF335;ZNF341;ZNF382;ZNF418;ZNF423;ZNF467;ZNF770;ZNF784;ZNF8	E4F1;PRDM4;REST;RREB1;ZBTB17;ZBTB4;ZNF335;ZNF341;ZNF467;ZNF770;ZNF784;ZNF8
BRAC_HUMAN.H11MO.1.B	20	0.11695906	0.7163463	1.931.145	1,21E+07	0.4404244	0.6751669	Brachyury-related factors{6.5.1}	T (Brachyury){6.5.1.0.1}	T;TBX19	T
BMAL1_HUMAN.H11MO.0.A	38	0.22222222	0.7161889	1.525.365	1,06E+07	0.7791240	0.8989759	PAS domain factors{1.2.5}	Arnt-like factors{1.2.5.2}	AHR;AHRR;ARNT;ARNT2;ARNTL;ARNTL2;CLOCK;EPAS1;HIF1A;HIF3A;NCOA1;NCOA2;NCOA3;NPAS1;NPAS2;NPAS3;NPAS4;SIM1;SIM2;SOHLH1;SOHLH2;TCFL5	ARNT2;ARNT;ARNTL;CLOCK
NR1H4_HUMAN.H11MO.1.B	19	0.11111111	0.7141520	1.971.735	1,22E+07	0.4274978	0.6699203	Thyroid hormone receptor-related factors (NR1){2.1.2}	LXR (NR1H){2.1.2.7}	NR1D1;NR1D2;NR1H2;NR1H3;NR1H4;NR1I2;NR1I3;PPARA;PPARD;PPARG;RARA;RARB;RARG;RORA;RORB;RORC;THRA;THRB;VDR	NR1H2;NR1H3;NR1H4
SOX13_HUMAN.H11MO.0.D	12	0.07017544	0.7136802	2.536.231	1,41E+07	0.2236270	0.4525366	SOX-related factors{4.1.1}	Group D{4.1.1.4}	BBX;CIC;HBP1;SOX1;SOX10;SOX11;SOX12;SOX13;SOX14;SOX15;SOX17;SOX18;SOX2;SOX21;SOX3;SOX30;SOX4;SOX5;SOX6;SOX7;SOX8;SOX9;SRY	SOX13;SOX5
NR2E1_HUMAN.H11MO.0.D	12	0.07017544	0.7120821	2.530.587	1,41E+07	0.2245786	0.4531683	RXR-related receptors (NR2){2.1.3}	Tailless-like receptors (NR2E){2.1.3.3}	HNF4A;HNF4G;NR2C1;NR2C2;NR2E1;NR2E3;NR2F1;NR2F2;NR2F6;RXRA;RXRB;RXRG	NR2E1;NR2E3
ZNF85_HUMAN.H11MO.1.C	13	0.07602339	0.7082247	2.395.605	1,36E+07	0.3183390	0.5616461	More than 3 adjacent zinc finger factors{2.3.3}	unclassified{2.3.3.0}	BCL6B;BCL6;CTCFL;CTCF;FEZF1;GFI1B;GFI1;GLI1;GLI2;GLI3;GLIS1;GLIS2;GLIS3;MTF1;MYNN;MZF1;OSR2;OVOL1;OVOL2;ZNF146;PLAG1;PLAGL1;PRDM14;PRDM1;PRDM6;SCRT1;SCRT2;SNAI1;SNAI2;YY1;YY2;WT1;ZNF324;ZNF354A;ZBTB14;ZBTB18;ZBTB48;ZBTB49;ZBTB7A;ZBTB7B;ZBTB6;ZFP64;ZFP28;ZFP42;ZFP82;ZFX;ZIC1;ZIC2;ZIC3;ZIC4;ZIM3;ZKSCAN1;ZKSCAN3;ZNF121;ZNF136;ZNF140;ZNF143;ZNF148;ZNF214;ZNF232;ZNF250;ZNF257;ZNF260;ZNF263;ZNF264;ZNF274;ZNF281;ZNF282;ZNF317;ZNF320;ZNF322;ZNF329;ZNF331;ZNF333;ZNF350;ZNF384;ZNF394;ZNF410;ZNF436;ZNF449;ZNF490;ZNF502;ZNF524;ZNF528;ZNF547;ZNF549;ZNF554;ZNF563;ZNF582;ZNF586;ZNF589;ZNF652;ZNF667;ZNF680;ZNF708;ZNF713;ZNF768;ZNF816;ZNF18;ZNF41;ZNF76;ZNF85;ZSCAN16;ZSCAN22;ZSCAN31;ZSCAN4	MYNN;MZF1;OSR2;PRDM14;PRDM6;WT1;ZBTB14;ZBTB48;ZBTB49;ZFP64;ZFP28;ZIM3;ZNF121;ZNF250;ZNF257;ZNF263;ZNF274;ZNF317;ZNF320;ZNF329;ZNF331;ZNF394;ZNF449;ZNF502;ZNF528;ZNF547;ZNF549;ZNF554;ZNF586;ZNF589;ZNF667;ZNF680;ZNF708;ZNF713;ZNF768;ZNF18;ZNF85;ZSCAN16;ZSCAN22
PEBB_HUMAN.H11MO.0.C	25	0.14619883	0.7054096	1.729.950	1,13E+07	0.5717467	0.7815899	*NA*	*NA*	CBFB	CBFB
ATF2_HUMAN.H11MO.0.B	21	0.12280702	0.7050049	1.859.261	1,17E+07	0.4580407	0.6863927	Jun-related factors{1.1.1}	ATF-2-like factors{1.1.1.3}	ATF2;ATF7;BACH1;BACH2;CREB5;JUN;JUNB;JUND;NFE2;NFE2L1;NFE2L2;NFE2L3	ATF2;ATF7;CREB5
ATF7_HUMAN.H11MO.0.D	20	0.11695906	0.7041994	1.898.354	1,19E+07	0.4453409	0.6762321	Jun-related factors{1.1.1}	ATF-2-like factors{1.1.1.3}	ATF2;ATF7;BACH1;BACH2;CREB5;JUN;JUNB;JUND;NFE2;NFE2L1;NFE2L2;NFE2L3	ATF2;ATF7;CREB5
ATF2_HUMAN.H11MO.2.C	20	0.11695906	0.7020299	1.892.521	1,18E+07	0.4464360	0.6762321	Jun-related factors{1.1.1}	ATF-2-like factors{1.1.1.3}	ATF2;ATF7;BACH1;BACH2;CREB5;JUN;JUNB;JUND;NFE2;NFE2L1;NFE2L2;NFE2L3	ATF2;ATF7;CREB5
SOX11_HUMAN.H11MO.0.D	11	0.06432749	0.6989370	2.628.767	1,43E+07	0.2674737	0.4970064	SOX-related factors{4.1.1}	Group C{4.1.1.3}	BBX;CIC;HBP1;SOX1;SOX10;SOX11;SOX12;SOX13;SOX14;SOX15;SOX17;SOX18;SOX2;SOX21;SOX3;SOX30;SOX4;SOX5;SOX6;SOX7;SOX8;SOX9;SRY	SOX11;SOX4
PHX2A_HUMAN.H11MO.0.D	11	0.06432749	0.6988138	2.628.303	1,43E+07	0.2675197	0.4970064	Paired-related HD factors{3.1.3}	PHOX{3.1.3.18}	ALX1;ALX3;ALX4;ARGFX;ARX;CRX;DMBX1;DPRX;DRGX;DUX4;DUXA;ESX1;GSC;GSC2;HESX1;ISX;LEUTX;MIXL1;NOBOX;OTP;OTX1;OTX2;PHOX2A;PHOX2B;PITX1;PITX2;PITX3;PROP1;PRRX1;PRRX2;RAX;RAX2;RHOXF1;RHOXF2;SEBOX;SHOX;SHOX2;TPRX1;UNCX;VSX1;VSX2	PHOX2A;PHOX2B

motif	Nº of probes	% of probes	lower OR	upper OR	OR	p.value	FDR	TF family	TF subfamily	TF.family.member	TF.subfamily.member
SIX2_HUMAN.H11MO.0.A	15	0.08771930	0.6981207	2.174.555	1,28E+07	0.3657849	0.6160028	HD-SINE factors{3.1.6}	SIX1-like factors{3.1.6.1}	SIX1;SIX2;SIX3;SIX4;SIX5;SIX6	SIX1;SIX2
TEAD2_HUMAN.H11MO.0.D	19	0.11111111	0.6966670	1.923.300	1,19E+07	0.4351493	0.6723448	TEF-1-related factors{3.6.1}	TEF-4 (TEAD-2){3.6.1.0.3}	TEAD1;TEAD2;TEAD3;TEAD4	TEAD2
ETS2_HUMAN.H11MO.0.B	43	0.25146199	0.6963301	1.434.970	1,01E+07	0.9298285	0.9820517	Ets-related factors{3.5.2}	Ets-like factors{3.5.2.1}	EHF;ELF1;ELF2;ELF3;ELF4;ELF5;ELK1;ELK3;ELK4;ERF;ERG;ETS1;ETS2;ETV1;ETV2;ETV3;ETV3L;ETV4;ETV5;ETV6;ETV7;FEV;FLI1;GABPA;SPDEF;SPI1;SPIB;SPIC	ERG;ETS1;ETS2;ETV2;ETV3;FEV;FLI1;GABPA
HXD4_HUMAN.H11MO.0.D	8	0.04678363	0.6953903	3.310.499	1,64E+07	0.1667469	0.3884681	HOX-related factors{3.1.1}	HOX4{3.1.1.4}	CDX1;CDX2;CDX4;EVX1;EVX2;GBX1;GBX2;GSX1;GSX2;HDX;HMBOX1;HNF1A;HNF1B;HOXA1;HOXA10;HOXA11;HOXA13;HOXA2;HOXA3;HOXA4;HOXA5;HOXA6;HOXA7;HOXA9;HOXB1;HOXB13;HOXB2;HOXB3;HOXB4;HOXB5;HOXB6;HOXB7;HOXB8;HOXB9;HOXC10;HOXC11;HOXC12;HOXC13;HOXC4;HOXC5;HOXC6;HOXC8;HOXC9;HOXD1;HOXD10;HOXD11;HOXD12;HOXD13;HOXD3;HOXD4;HOXD8;HOXD9;MEOX1;MEOX2;MNX1;PDX1;POU1F1;POU2F1;POU2F2;POU2F3;POU3F1;POU3F2;POU3F3;POU3F4;POU4F1;POU4F2;POU4F3;POU5F1;POU5F2;POU6F1;POU6F2	HOXB4;HOXD4
FOXA3_HUMAN.H11MO.0.B	16	0.09356725	0.6947483	2.090.041	1,25E+07	0.3865538	0.6285525	Forkhead box (FOX) factors{3.3.1}	FOXA{3.3.1.1}	FOXA1;FOXA2;FOXA3;FOXB1;FOXB2;FOXC1;FOXC2;FOXD1;FOXD2;FOXD3;FOXD4;FOXD4L1;FOXD4L3;FOXD4L4;FOXD4L5;FOXD4L6;FOXE1;FOXE3;FOXF1;FOXF2;FOXG1;FOXH1;FOXI1;FOXI2;FOXI3;FOXJ1;FOXJ2;FOXJ3;FOXK1;FOXK2;FOXL1;FOXL2;FOXM1;FOXN1;FOXN2;FOXN3;FOXN4;FOXO1;FOXO3;FOXO4;FOXO6;FOXP1;FOXP2;FOXP3;FOXP4;FOXQ1;FOXR1;FOXR2;FOXS1	FOXA1;FOXA2;FOXA3
SOX2_HUMAN.H11MO.0.A	25	0.14619883	0.6925647	1.698.496	1,11E+07	0.6528086	0.8360721	SOX-related factors{4.1.1}	Group B{4.1.1.2}	BBX;CIC;HBP1;SOX1;SOX10;SOX11;SOX12;SOX13;SOX14;SOX15;SOX17;SOX18;SOX2;SOX21;SOX3;SOX30;SOX4;SOX5;SOX6;SOX7;SOX8;SOX9;SRY	SOX1;SOX21;SOX2;SOX3
ZN394_HUMAN.H11MO.1.D	16	0.09356725	0.6919500	2.081.547	1,24E+07	0.3879267	0.6285525	More than 3 adjacent zinc finger factors{2.3.3}	unclassified{2.3.3.0}	BCL6B;BCL6;CTCFL;CTCF;FEZF1;GFI1B;GFI1;GLI1;GLI2;GLI3;GLIS1;GLIS2;GLIS3;MTF1;MYNN;MZF1;OSR2;OVOL1;OVOL2;ZNF146;PLAG1;PLAGL1;PRDM14;PRDM1;PRDM6;SCRT1;SCRT2;SNAI1;SNAI2;YY1;YY2;WT1;ZNF324;ZNF354A;ZBTB14;ZBTB18;ZBTB48;ZBTB49;ZBTB7A;ZBTB7B;ZBTB6;ZFP64;ZFP28;ZFP42;ZFP82;ZFX;ZIC1;ZIC2;ZIC3;ZIC4;ZIM3;ZKSCAN1;ZKSCAN3;ZNF121;ZNF136;ZNF140;ZNF143;ZNF148;ZNF214;ZNF232;ZNF250;ZNF257;ZNF260;ZNF263;ZNF264;ZNF274;ZNF281;ZNF282;ZNF317;ZNF320;ZNF322;ZNF329;ZNF331;ZNF333;ZNF350;ZNF384;ZNF394;ZNF410;ZNF436;ZNF449;ZNF490;ZNF502;ZNF524;ZNF528;ZNF547;ZNF549;ZNF554;ZNF563;ZNF582;ZNF586;ZNF589;ZNF652;ZNF667;ZNF680;ZNF708;ZNF713;ZNF768;ZNF816;ZNF18;ZNF41;ZNF76;ZNF85;ZSCAN16;ZSCAN22;ZSCAN31;ZSCAN4	MYNN;MZF1;OSR2;PRDM14;PRDM6;WT1;ZBTB14;ZBTB48;ZBTB49;ZFP64;ZFP28;ZIM3;ZNF121;ZNF250;ZNF257;ZNF263;ZNF274;ZNF317;ZNF320;ZNF329;ZNF331;ZNF394;ZNF449;ZNF502;ZNF528;ZNF547;ZNF549;ZNF554;ZNF586;ZNF589;ZNF667;ZNF680;ZNF708;ZNF713;ZNF768;ZNF18;ZNF85;ZSCAN16;ZSCAN22
SRBP1_HUMAN.H11MO.0.A	32	0.18713450	0.6897040	1.547.847	1,05E+07	0.8420493	0.9450073	bHLH-ZIP factors{1.2.6}	SREBP factors{1.2.6.3}	MAX;MITF;MLX;MLXIP;MLXIPL;MNT;MXD1;MXD3;MXD4;MXI1;MYC;MYCL;MYCN;REPIN1;SREBF1;SREBF2;TFAP4;TFE3;TFEB;TFEC;USF1;USF2	SREBF1;SREBF2
USF1_HUMAN.H11MO.0.A	30	0.17543860	0.6889844	1.580.118	1,06E+07	0.7585644	0.8879081	bHLH-ZIP factors{1.2.6}	USF factors{1.2.6.2}	MAX;MITF;MLX;MLXIP;MLXIPL;MNT;MXD1;MXD3;MXD4;MXI1;MYC;MYCL;MYCN;REPIN1;SREBF1;SREBF2;TFAP4;TFE3;TFEB;TFEC;USF1;USF2	USF1;USF2

motif	Nº of probes	% of probes	lower OR	upper OR	OR	p.value	FDR	TF family	TF subfamily	TF.family.member	TF.subfamily.member
PO3F2_HUMAN.H11MO.0.A	13	0.07602339	0.6886278	2.329.155	1,32E+07	0.3256241	0.5718819	POU domain factors{3.1.10}	POU3 (Oct-6-like factors){3.1.10.3}	CDX1;CDX2;CDX4;EVX1;EVX2;GBX1;GBX2;GSX1;GSX2;HDX;HMBOX1;HNF1A;HNF1B;HOXA1;HOXA10;HOXA11;HOXA13;HOXA2;HOXA3;HOXA4;HOXA5;HOXA6;HOXA7;HOXA9;HOXB1;HOXB13;HOXB2;HOXB3;HOXB4;HOXB5;HOXB6;HOXB7;HOXB8;HOXB9;HOXC10;HOXC11;HOXC12;HOXC13;HOXC4;HOXC5;HOXC6;HOXC8;HOXC9;HOXD1;HOXD10;HOXD11;HOXD12;HOXD13;HOXD3;HOXD4;HOXD8;HOXD9;MEOX1;MEOX2;MNX1;PDX1;POU1F1;POU2F1;POU2F2;POU2F3;POU3F1;POU3F2;POU3F3;POU3F4;POU4F1;POU4F2;POU4F3;POU5F1;POU5F1B;POU5F2;POU6F1;POU6F2	POU3F1;POU3F2;POU3F3;POU3F4
PPARD_HUMAN.H11MO.0.D	21	0.12280702	0.6877662	1.813.854	1,14E+07	0.5393025	0.7546320	Thyroid hormone receptor-related factors (NR1){2.1.2}	PPAR (NR1C){2.1.2.5}	NR1D1;NR1D2;NR1H2;NR1H3;NR1H4;NR1I2;NR1I3;PPARA;PPARD;PPARG;RARA;RARB;RARG;RORA;RORB;RORC;THRA;THRB;VDR	PPARA;PPARD;PPARG
HXB3_HUMAN.H11MO.0.D	12	0.07017544	0.6876134	2.443.651	1,36E+07	0.3004841	0.5412926	HOX-related factors {3.1.1}	HOX3{3.1.1.3}	CDX1;CDX2;CDX4;EVX1;EVX2;GBX1;GBX2;GSX1;GSX2;HDX;HMBOX1;HNF1A;HNF1B;HOXA1;HOXA10;HOXA11;HOXA13;HOXA2;HOXA3;HOXA4;HOXA5;HOXA6;HOXA7;HOXA9;HOXB1;HOXB13;HOXB2;HOXB3;HOXB4;HOXB5;HOXB6;HOXB7;HOXB8;HOXB9;HOXC10;HOXC11;HOXC12;HOXC13;HOXC4;HOXC5;HOXC6;HOXC8;HOXC9;HOXD1;HOXD10;HOXD11;HOXD12;HOXD13;HOXD3;HOXD4;HOXD8;HOXD9;MEOX1;MEOX2;MNX1;PDX1;POU1F1;POU2F1;POU2F2;POU2F3;POU3F1;POU3F2;POU3F3;POU3F4;POU4F1;POU4F2;POU4F3;POU5F1;POU5F2;POU6F1;POU6F2	HOXB3;HOXD3
NR1H3_HUMAN.H11MO.0.B	22	0.12865497	0.6864188	1.774.009	1,13E+07	0.5509592	0.7612716	Thyroid hormone receptor-related factors (NR1){2.1.2}	LXR (NR1H){2.1.2.7}	NR1D1;NR1D2;NR1H2;NR1H3;NR1H4;NR1I2;NR1I3;PPARA;PPARD;PPARG;RARA;RARB;RARG;RORA;RORB;RORC;THRA;THRB;VDR	NR1H2;NR1H3;NR1H4
DRGX_HUMAN.H11MO.0.D	11	0.06432749	0.6861297	2.580.402	1,40E+07	0.2729101	0.5033821	Paired-related HD factors{3.1.3}	DRGX{3.1.3.6}	ALX1;ALX3;ALX4;ARGFX;ARX;CRX;DMBX1;DPRX;DRGX;DUX4;DUXA;ESX1;GSC;GSC2;HESX1;ISX;LEUTX;MIXL1;NOBOX;OTP;OTX1;OTX2;PHOX2A;PHOX2B;PITX1;PITX2;PITX3;PROP1;PRRX1;PRRX2;RAX;RAX2;RHOXF1;RHOXF2;SEBOX;SHOX;SHOX2;TPRX1;UNCX;VSX1;VSX2	DRGX

motif	Nº of probes	% of probes	lower OR	upper OR	OR	p.value	FDR	TF family	TF subfamily	TF.family.member	TF.subfamily.member
ZN490_HUMAN.H11MO.0.C	16	0.09356725	0.6857478	2.062.994	1,23E+07	0.3913167	0.6285525	More than 3 adjacent zinc finger factors{2.3.3}	ZNF763-like factors{2.3.3.33}	BCL6;BCL6B;CTCF;CTCFL;FEZF1;FEZF2;GFI1;GFI1B;GLI1;GLI2;GLI3;GLI4;GLIS1;GLIS2;GLIS3;HKR1;MTF1;MYNN;MZF1;OSR2;OVOL1;OVOL2;PLAG1;PLAGL1;PLAGL2;PRDM1;PRDM14;PRDM6;SCRT1;SCRT2;SNAI1;SNAI2;SNAI3;WT1;YY1;YY2;ZBTB12;ZBTB14;ZBTB18;ZBTB20;ZBTB26;ZBTB42;ZBTB45;ZBTB47;ZBTB48;ZBTB49;ZBTB6;ZBTB7A;ZBTB7B;ZBTB7C;ZFP14;ZFP2;ZFP28;ZFP30;ZFP37;ZFP42;ZFP64;ZFP69;ZFP69B;ZFP82;ZFP91;ZFX;ZIC1;ZIC2;ZIC3;ZIC4;ZIC5;ZIK1;ZIM3;ZKSCAN1;ZKSCAN2;ZKSCAN3;ZKSCAN4;ZNF121;ZNF124;ZNF133;ZNF136;ZNF138;ZNF14;ZNF140;ZNF143;ZNF146;ZNF148;ZNF155;ZNF157;ZNF160;ZNF169;ZNF175;ZNF177;ZNF18;ZNF180;ZNF181;ZNF2;ZNF20;ZNF212;ZNF213;ZNF214;ZNF221;ZNF222;ZNF223;ZNF224;ZNF225;ZNF226;ZNF227;ZNF229;ZNF230;ZNF232;ZNF233;ZNF234;ZNF235;ZNF24;ZNF25;ZNF250;ZNF257;ZNF26;ZNF260;ZNF263;ZNF264;ZNF268;ZNF274;ZNF276;ZNF28;ZNF280A;ZNF280B;ZNF280C;ZNF280D;ZNF281;ZNF282;ZNF283;ZNF284;ZNF285;ZNF286A;ZNF286B;ZNF3;ZNF30;ZNF300;ZNF302;ZNF317;ZNF32;ZNF320;ZNF322;ZNF324;ZNF324B;ZNF329;ZNF331;ZNF333;ZNF33A;ZNF33B;ZNF343;ZNF345;ZNF347;ZNF350;ZNF354A;ZNF354B;ZNF362;ZNF366;ZNF383;ZNF384;ZNF394;ZNF397;ZNF398;ZNF404;ZNF41;ZNF410;ZNF419;ZNF420;ZNF431;ZNF432;ZNF436;ZNF439;ZNF44;ZNF440;ZNF442;ZNF443;ZNF446;ZNF449;ZNF45;ZNF460;ZNF468;ZNF479;ZNF484;ZNF490;ZNF500;ZNF502;ZNF524;ZNF525;ZNF528;ZNF543;ZNF544;ZNF546;ZNF547;ZNF548;ZNF549;ZNF554;ZNF555;ZNF557;ZNF558;ZNF559;ZNF561;ZNF562;ZNF563;ZNF564;ZNF566;ZNF567;ZNF568;ZNF57;ZNF570;ZNF571;ZNF572;ZNF577;ZNF581;ZNF582;ZNF583;ZNF585A;ZNF586;ZNF589;ZNF595;ZNF599;ZNF600;ZNF605;ZNF607;ZNF611;ZNF613;ZNF614;ZNF615;ZNF616;ZNF619;ZNF620;ZNF621;ZNF625;ZNF627;ZNF649;ZNF652;ZNF653;ZNF665;ZNF667;ZNF669;ZNF670;ZNF672;ZNF679;ZNF680;ZNF683;ZNF689;ZNF692;ZNF701;ZNF705D;ZNF705E;ZNF705G;ZNF708;ZNF709;ZNF71;ZNF710;ZNF713;ZNF721;ZNF727;ZNF729;ZNF736;ZNF75A;ZNF75D;ZNF76;ZNF763;ZNF764;ZNF765;ZNF768;ZNF77;ZNF771;ZNF773;ZNF774;ZNF776;ZNF777;ZNF780A;ZNF780B;ZNF782;ZNF785;ZNF799;ZNF805;ZNF808;ZNF81;ZNF813;ZNF816;ZNF823;ZNF829;ZNF836;ZNF841;ZNF844;ZNF845;ZNF846;ZNF85;ZNF853;ZNF860;ZNF878;ZNF891;ZNF99;ZSCAN16;ZSCAN2;ZSCAN22;ZSCAN23;ZSCAN29;ZSCAN31;ZSCAN32;ZSCAN4;ZSCAN5A;ZSCAN5B;ZSCAN5C;ZSCAN9;ZXDA;ZXDB;ZXDC	ZNF136;ZNF490;ZNF563
THA11_HUMAN.H11MO.0.B	24	0.14035088	0.6856901	1.709.112	1,10E+07	0.6473102	0.8345756	THAP-related factors{2.9.1}	THAP11 (HRIHFB2206){2.9.1.0.11}	THAP1;THAP10;THAP11;THAP12;THAP2;THAP3;THAP4;THAP5;THAP6;THAP7;THAP8;THAP9	THAP11
TAL1_HUMAN.H11MO.1.A	34	0.19883041	0.6854022	1.508.786	1,03E+07	0.8469785	0.9461359	Tal-related factors{1.2.3}	Tal / HEN-like factors{1.2.3.1}	ATOH1;ATOH7;ATOH8;BHLHA15;BHLHA9;BHLHE22;BHLHE23;FERD3L;FIGLA;HAND1;HAND2;LYL1;MESP1;MESP2;MSC;MSGN1;NEUROD1;NEUROD2;NEUROD4;NEUROD6;NEUROG1;NEUROG2;NEUROG3;NHLH1;NHLH2;OLIG1;OLIG2;OLIG3;PTF1A;SCX;TAL1;TAL2;TCF15;TCF21;TCF23;TWIST1;TWIST2	NHLH1;LYL1;TAL1

motif	Nº of probes	% of probes	lower OR	upper OR	OR	p.value	FDR	TF family	TF subfamily	TF.family.member	TF.subfamily.member
BHE40_HUMAN.H11MO.0.A	39	0.22807018	0.6847307	1.447.778	1,01E+07	10000000,00000000	1,00E+07	Hairy-related factors{1.2.4}	Hairy-like factors{1.2.4.1}	BHLHE40;BHLHE41;HELT;HES1;HES2;HES3;HES4;HES5;HES6;HES7;HEY1;HEY2;HEYL	BHLHE40;BHLHE41;HES1;HES5;HES7;HEY1;HEY2
SOX9_HUMAN.H11MO.0.B	15	0.08771930	0.6839864	2.130.496	1,25E+07	0.3726770	0.6225095	SOX-related factors{4.1.1}	Group E{4.1.1.5}	BBX;CIC;HBP1;SOX1;SOX10;SOX11;SOX12;SOX13;SOX14;SOX15;SOX17;SOX18;SOX2;SOX21;SOX3;SOX30;SOX4;SOX5;SOX6;SOX7;SOX8;SOX9;SRY	SOX10;SOX8;SOX9
ZN250_HUMAN.H11MO.0.C	14	0.08187135	0.6837408	2.213.985	1,28E+07	0.3514143	0.6006941	More than 3 adjacent zinc finger factors{2.3.3}	unclassified{2.3.3.0}	BCL6B;BCL6;CTCFL;CTCF;FEZF1;GFI1B;GFI1;GLI1;GLI2;GLI3;GLIS1;GLIS2;GLIS3;MTF1;MYNN;MZF1;OSR2;OVOL1;OVOL2;ZNF146;PLAG1;PLAGL1;PRDM14;PRDM1;PRDM6;SCRT1;SCRT2;SNAI1;SNAI2;YY1;YY2;WT1;ZNF324;ZNF354A;ZBTB14;ZBTB18;ZBTB48;ZBTB49;ZBTB7A;ZBTB7B;ZBTB6;ZFP64;ZFP28;ZFP42;ZFP82;ZFX;ZIC1;ZIC2;ZIC3;ZIC4;ZIM3;ZKSCAN1;ZKSCAN3;ZNF121;ZNF136;ZNF140;ZNF143;ZNF148;ZNF214;ZNF232;ZNF250;ZNF257;ZNF260;ZNF263;ZNF264;ZNF274;ZNF281;ZNF282;ZNF317;ZNF320;ZNF322;ZNF329;ZNF331;ZNF333;ZNF350;ZNF384;ZNF394;ZNF410;ZNF436;ZNF449;ZNF490;ZNF502;ZNF524;ZNF528;ZNF547;ZNF549;ZNF554;ZNF563;ZNF582;ZNF586;ZNF589;ZNF652;ZNF667;ZNF680;ZNF708;ZNF713;ZNF768;ZNF816;ZNF18;ZNF41;ZNF76;ZNF85;ZSCAN16;ZSCAN22;ZSCAN31;ZSCAN4	MYNN;MZF1;OSR2;PRDM14;PRDM6;WT1;ZBTB14;ZBTB48;ZBTB49;ZFP64;ZFP28;ZIM3;ZNF121;ZNF250;ZNF257;ZNF263;ZNF274;ZNF317;ZNF320;ZNF329;ZNF331;ZNF394;ZNF449;ZNF502;ZNF528;ZNF547;ZNF549;ZNF554;ZNF586;ZNF589;ZNF667;ZNF680;ZNF708;ZNF713;ZNF768;ZNF18;ZNF85;ZSCAN16;ZSCAN22
NFAC1_HUMAN.H11MO.0.B	26	0.15204678	0.6824331	1.648.351	1,08E+07	0.7421515	0.8816623	NFAT-related factors{6.1.3}	NFATc1{6.1.3.0.1}	NFAT5;NFATC1;NFATC2;NFATC3;NFATC4	NFATC1
SPDEF_HUMAN.H11MO.0.D	20	0.11695906	0.6803355	1.834.018	1,15E+07	0.5306912	0.7493826	Ets-related factors{3.5.2}	SPDEF-like factors{3.5.2.7}	EHF;ELF1;ELF2;ELF3;ELF4;ELF5;ELK1;ELK3;ELK4;ERF;ERG;ETS1;ETS2;ETV1;ETV2;ETV3;ETV3L;ETV4;ETV5;ETV6;ETV7;FEV;FLI1;GABPA;SPDEF;SPI1;SPIB;SPIC	SPDEF
FOXC1_HUMAN.H11MO.0.C	15	0.08771930	0.6802595	2.118.878	1,25E+07	0.3748874	0.6225095	Forkhead box (FOX) factors{3.3.1}	FOXC{3.3.1.3}	FOXA1;FOXA2;FOXA3;FOXB1;FOXB2;FOXC1;FOXC2;FOXD1;FOXD2;FOXD3;FOXD4;FOXD4L1;FOXD4L3;FOXD4L4;FOXD4L5;FOXD4L6;FOXE1;FOXE3;FOXF1;FOXF2;FOXG1;FOXH1;FOXI1;FOXI2;FOXI3;FOXJ1;FOXJ2;FOXJ3;FOXK1;FOXK2;FOXL1;FOXL2;FOXM1;FOXN1;FOXN2;FOXN3;FOXN4;FOXO1;FOXO3;FOXO4;FOXO6;FOXP1;FOXP2;FOXP3;FOXP4;FOXQ1;FOXR1;FOXR2;FOXS1	FOXC1;FOXC2
FOXC2_HUMAN.H11MO.0.D	15	0.08771930	0.6789994	2.114.949	1,24E+07	0.3756727	0.6225095	Forkhead box (FOX) factors{3.3.1}	FOXC{3.3.1.3}	FOXA1;FOXA2;FOXA3;FOXB1;FOXB2;FOXC1;FOXC2;FOXD1;FOXD2;FOXD3;FOXD4;FOXD4L1;FOXD4L3;FOXD4L4;FOXD4L5;FOXD4L6;FOXE1;FOXE3;FOXF1;FOXF2;FOXG1;FOXH1;FOXI1;FOXI2;FOXI3;FOXJ1;FOXJ2;FOXJ3;FOXK1;FOXK2;FOXL1;FOXL2;FOXM1;FOXN1;FOXN2;FOXN3;FOXN4;FOXO1;FOXO3;FOXO4;FOXO6;FOXP1;FOXP2;FOXP3;FOXP4;FOXQ1;FOXR1;FOXR2;FOXS1	FOXC1;FOXC2
PAX3_HUMAN.H11MO.0.D	9	0.05263158	0.6747137	2.927.516	1,50E+07	0.2138120	0.4436168	Paired plus homeo domain{3.2.1}	PAX-3/7{3.2.1.1}	PAX3;PAX4;PAX6;PAX7	PAX3;PAX7
MITF_HUMAN.H11MO.0.A	29	0.16959064	0.6747110	1.565.687	1,04E+07	0.8360054	0.9395920	bHLH-ZIP factors{1.2.6}	TFE3-like factors{1.2.6.1}	MAX;MITF;MLX;MLXIP;MLXIPL;MNT;MXD1;MXD3;MXD4;MXI1;MYC;MYCL;MYCN;REPIN1;SREBF1;SREBF2;TFAP4;TFE3;TFEB;TFEC;USF1;USF2	MITF;TFE3;TFEB
RREB1_HUMAN.H11MO.0.D	47	0.27485380	0.6740081	1.359.708	0.9643578	0.8654123	0.9464296	Factors with multiple dispersed zinc fingers{2.3.4}	unclassified{2.3.4.0}	BCL11A;E4F1;MECOM;HIC1;HIC2;HINFP;IKZF1;INSM1;MAZ;PATZ1;PRDM4;REST;RREB1;SALL4;VEZF1;ZBTB17;ZBTB4;HIVEP1;HIVEP2;ZNF134;ZNF219;ZNF335;ZNF341;ZNF382;ZNF418;ZNF423;ZNF467;ZNF770;ZNF784;ZNF8	E4F1;PRDM4;REST;RREB1;ZBTB17;ZBTB4;ZNF335;ZNF341;ZNF467;ZNF770;ZNF784;ZNF8
NRL_HUMAN.H11MO.0.D	22	0.12865497	0.6739833	1.741.849	1,11E+07	0.6344472	0.8207362	Maf-related factors{1.1.3}	Large Maf factors{1.1.3.1}	MAF;MAFA;MAFB;MAFF;MAFG;MAFK;NRL	MAFA;MAFB;MAF;NRL

motif	Nº of probes	% of probes	lower OR	upper OR	OR	p.value	FDR	TF family	TF subfamily	TF.family.member	TF.subfamily.member
HXD10_HUMAN.H11MO.0.D	13	0.07602339	0.6703032	2.266.991	1,29E+07	0.3359264	0.5859711	HOX-related factors{3.1.1}	HOX9-13{3.1.1.8}	CDX1;CDX2;CDX4;EVX1;EVX2;GBX1;GBX2;GSX1;GSX2;HDX;HMBOX1;HNF1A;HNF1B;HOXA1;HOXA10;HOXA11;HOXA13;HOXA2;HOXA3;HOXA4;HOXA5;HOXA6;HOXA7;HOXA9;HOXB1;HOXB13;HOXB2;HOXB3;HOXB4;HOXB5;HOXB6;HOXB7;HOXB8;HOXB9;HOXC10;HOXC11;HOXC12;HOXC13;HOXC4;HOXC5;HOXC6;HOXC8;HOXC9;HOXD1;HOXD10;HOXD11;HOXD12;HOXD13;HOXD3;HOXD4;HOXD8;HOXD9;MEOX1;MEOX2;MNX1;PDX1;POU1F1;POU2F1;POU2F2;POU2F3;POU3F1;POU3F2;POU3F3;POU3F4;POU4F1;POU4F2;POU4F3;POU5F1;POU5F2;POU6F1;POU6F2	HOXA10;HOXA11;HOXA13;HOXA9;HOXB13;HOXC10;HOXC11;HOXC12;HOXC13;HOXC9;HOXD10;HOXD11;HOXD12;HOXD13;HOXD9
FUBP1_HUMAN.H11MO.0.D	23	0.13450292	0.6684015	1.695.298	1,09E+07	0.7280308	0.8770496	NA	NA	FUBP1	FUBP1
PO3F1_HUMAN.H11MO.0.C	12	0.07017544	0.6640119	2.359.662	1,31E+07	0.3949926	0.6318243	POU domain factors{3.1.10}	POU3 (Oct-6-like factors){3.1.10.3}	CDX1;CDX2;CDX4;EVX1;EVX2;GBX1;GBX2;GSX1;GSX2;HDX;HMBOX1;HNF1A;HNF1B;HOXA1;HOXA10;HOXA11;HOXA13;HOXA2;HOXA3;HOXA4;HOXA5;HOXA6;HOXA7;HOXA9;HOXB1;HOXB13;HOXB2;HOXB3;HOXB4;HOXB5;HOXB6;HOXB7;HOXB8;HOXB9;HOXC10;HOXC11;HOXC12;HOXC13;HOXC4;HOXC5;HOXC6;HOXC8;HOXC9;HOXD1;HOXD10;HOXD11;HOXD12;HOXD13;HOXD3;HOXD4;HOXD8;HOXD9;MEOX1;MEOX2;MNX1;PDX1;POU1F1;POU2F1;POU2F2;POU2F3;POU3F1;POU3F2;POU3F3;POU3F4;POU4F1;POU4F2;POU4F3;POU5F1;POU5F1B;POU5F2;POU6F1;POU6F2	POU3F1;POU3F2;POU3F3;POU3F4
ZN350_HUMAN.H11MO.1.D	34	0.19883041	0.6632158	1.459.934	0.9964005	10000000,00000000	1,00E+07	More than 3 adjacent zinc finger factors{2.3.3}	ZNF350-like factors{2.3.3.30}	BCL6;BCL6B;CTCF;CTCFL;FEZF1;FEZF2;GFI1;GFI1B;GLI1;GLI2;GLI3;GLI4;GLIS1;GLIS2;GLIS3;HKR1;MTF1;MYNN;MZF1;OSR2;OVOL1;OVOL2;PLAG1;PLAGL1;PLAGL2;PRDM1;PRDM14;PRDM6;SCRT1;SCRT2;SNAI1;SNAI2;SNAI3;WT1;YY1;YY2;ZBTB12;ZBTB14;ZBTB18;ZBTB20;ZBTB26;ZBTB42;ZBTB45;ZBTB47;ZBTB48;ZBTB49;ZBTB6;ZBTB7A;ZBTB7B;ZBTB7C;ZFP14;ZFP2;ZFP28;ZFP30;ZFP37;ZFP42;ZFP64;ZFP69;ZFP69B;ZFP82;ZFP91;ZFX;ZIC1;ZIC2;ZIC3;ZIC4;ZIC5;ZIK1;ZIM3;ZKSCAN1;ZKSCAN2;ZKSCAN3;ZKSCAN4;ZNF121;ZNF124;ZNF133;ZNF136;ZNF138;ZNF14;ZNF140;ZNF143;ZNF146;ZNF148;ZNF155;ZNF157;ZNF160;ZNF169;ZNF175;ZNF177;ZNF18;ZNF180;ZNF181;ZNF2;ZNF20;ZNF212;ZNF213;ZNF214;ZNF221;ZNF222;ZNF223;ZNF224;ZNF225;ZNF226;ZNF227;ZNF229;ZNF230;ZNF232;ZNF233;ZNF234;ZNF235;ZNF24;ZNF25;ZNF250;ZNF257;ZNF26;ZNF260;ZNF263;ZNF264;ZNF268;ZNF274;ZNF276;ZNF28;ZNF280A;ZNF280B;ZNF280C;ZNF280D;ZNF281;ZNF282;ZNF283;ZNF284;ZNF285;ZNF286A;ZNF286B;ZNF3;ZNF30;ZNF300;ZNF302;ZNF317;ZNF32;ZNF320;ZNF322;ZNF324;ZNF324B;ZNF329;ZNF331;ZNF333;ZNF33A;ZNF33B;ZNF343;ZNF345;ZNF347;ZNF350;ZNF354A;ZNF354B;ZNF362;ZNF366;ZNF383;ZNF384;ZNF394;ZNF397;ZNF398;ZNF404;ZNF41;ZNF410;ZNF419;ZNF420;ZNF431;ZNF432;ZNF436;ZNF439;ZNF44;ZNF440;ZNF442;ZNF443;ZNF446;ZNF449;ZNF45;ZNF460;ZNF468;ZNF479;ZNF484;ZNF490;ZNF500;ZNF502;ZNF524;ZNF525;ZNF528;ZNF543;ZNF544;ZNF546;ZNF547;ZNF548;ZNF549;ZNF554;ZNF555;ZNF557;ZNF558;ZNF559;ZNF561;ZNF562;ZNF563;ZNF564;ZNF566;ZNF567;ZNF568;ZNF57;ZNF570;ZNF571;ZNF572;ZNF577;ZNF581;ZNF582;ZNF583;ZNF585A;ZNF586;ZNF589;ZNF595;ZNF599;ZNF600;ZNF605;ZNF607;ZNF611;ZN	ZNF350

motif	Nº of probes	% of probes	lower OR	upper OR	OR	p.value	FDR	TF family	TF subfamily	TF.family.member	TF.subfamily.member
										F613;ZNF614;ZNF615;ZNF616;ZNF619;ZNF620;ZNF621;ZNF625;ZNF627;ZNF649;ZNF652;ZNF653;ZNF665;ZNF667;ZNF669;ZNF670;ZNF672;ZNF679;ZNF680;ZNF683;ZNF689;ZNF692;ZNF701;ZNF705D;ZNF705E;ZNF705G;ZNF708;ZNF709;ZNF71;ZNF710;ZNF713;ZNF721;ZNF727;ZNF729;ZNF736;ZNF75A;ZNF75D;ZNF76;ZNF763;ZNF764;ZNF765;ZNF768;ZNF77;ZNF771;ZNF773;ZNF774;ZNF776;ZNF777;ZNF780A;ZNF780B;ZNF782;ZNF785;ZNF799;ZNF805;ZNF808;ZNF81;ZNF813;ZNF816;ZNF823;ZNF829;ZNF836;ZNF841;ZNF844;ZNF845;ZNF846;ZNF85;ZNF853;ZNF860;ZNF878;ZNF891;ZNF99;ZSCAN16;ZSCAN2;ZSCAN22;ZSCAN23;ZSCAN29;ZSCAN31;ZSCAN32;ZSCAN4;ZSCAN5A;ZSCAN5B;ZSCAN5C;ZSCAN9;ZXDA;ZXDB;ZXDC	
NFIC_HUMAN.H11MO.1.A	20	0.11695906	0.6630429	1.787.368	1,12E+07	0.6188817	0.8131233	Nuclear factor 1{7.1.2}	NF-1C (NF-IC){7.1.2.0.3}	NFIA;NFIB;NFIC	NFIC
BARX2_HUMAN.H11MO.0.D	10	0.05847953	0.6599674	2.650.444	1,40E+07	0.2569510	0.4895321	NK-related factors{3.1.2}	BARX{3.1.2.2}	BARHL1;BARHL2;BARX1;BARX2;BSX;DBX1;DBX2;DLX1;DLX2;DLX3;DLX4;DLX5;DLX6;EMX1;EMX2;EN1;EN2;HHEX;HLX;HMX1;HMX2;HMX3;LBX1;LBX2;MSX1;MSX2;NANOG;NKX1-1;NKX1-2;NKX2-1;NKX2-2;NKX2-3;NKX2-4;NKX2-5;NKX2-6;NKX2-8;NKX3-1;NKX3-2;NKX6-1;NKX6-2;NKX6-3;NOTO;TLX1;TLX2;TLX3;VAX1;VAX2;VENTX	BARX1;BARX2
HNF1B_HUMAN.H11MO.0.A	12	0.07017544	0.6599475	2.345.186	1,30E+07	0.3962555	0.6325320	POU domain factors{3.1.10}	HNF1-like factors{3.1.10.7}	CDX1;CDX2;CDX4;EVX1;EVX2;GBX1;GBX2;GSX1;GSX2;HDX;HMBOX1;HNF1A;HNF1B;HOXA1;HOXA10;HOXA11;HOXA13;HOXA2;HOXA3;HOXA4;HOXA5;HOXA6;HOXA7;HOXA9;HOXB1;HOXB13;HOXB2;HOXB3;HOXB4;HOXB5;HOXB6;HOXB7;HOXB8;HOXB9;HOXC10;HOXC11;HOXC12;HOXC13;HOXC4;HOXC5;HOXC6;HOXC8;HOXC9;HOXD1;HOXD10;HOXD11;HOXD12;HOXD13;HOXD3;HOXD4;HOXD8;HOXD9;MEOX1;MEOX2;MNX1;PDX1;POU1F1;POU2F1;POU2F2;POU2F3;POU3F1;POU3F2;POU3F3;POU3F4;POU4F1;POU4F2;POU4F3;POU5F1;POU5F1B;POU5F2;POU6F1;POU6F2	HMBOX1;HNF1A;HNF1B
MXI1_HUMAN.H11MO.1.A	46	0.26900585	0.6593964	1.336.807	0.9460986	0.7985427	0.9091139	bHLH-ZIP factors{1.2.6}	Mad-like factors{1.2.6.7}	MAX;MITF;MLX;MLXIP;MLXIPL;MNT;MXD1;MXD3;MXD4;MXI1;MYC;MYCL;MYCN;REPIN1;SREBF1;SREBF2;TFAP4;TFE3;TFEB;TFEC;USF1;USF2	MXI1
IRF4_HUMAN.H11MO.0.A	25	0.14619883	0.6592401	1.616.648	1,05E+07	0.8251736	0.9328575	Interferon-regulatory factors{3.5.3}	IRF-4 (LSIRF, NF-EM5, MUM1, Pip){3.5.3.0.4}	IRF1;IRF2;IRF3;IRF4;IRF5;IRF6;IRF7;IRF8;IRF9	IRF4
HSF4_HUMAN.H11MO.0.D	16	0.09356725	0.6588935	1.982.081	1,18E+07	0.4820295	0.7038799	HSF factors{3.4.1}	HSF4 (HSTF4){3.4.1.0.3}	HSF1;HSF2;HSF4;HSF5;HSFX1;HSFY1;HSFY1;HSFY2	HSF4

motif	Nº of probes	% of probes	lower OR	upper OR	OR	p.value	FDR	TF family	TF subfamily	TF.family.member	TF.subfamily.member
PO2F2_HUMAN.H11MO.0.A	14	0.08187135	0.6586213	2.132.591	1,23E+07	0.4435152	0.6757909	POU domain factors{3.1.10}	POU2 (Oct-1/2-like factors){3.1.10.2}	CDX1;CDX2;CDX4;EVX1;EVX2;GBX1;GBX2;GSX1;GSX2;HDX;HMBOX1;HNF1A;HNF1B;HOXA1;HOXA10;HOXA11;HOXA13;HOXA2;HOXA3;HOXA4;HOXA5;HOXA6;HOXA7;HOXA9;HOXB1;HOXB13;HOXB2;HOXB3;HOXB4;HOXB5;HOXB6;HOXB7;HOXB8;HOXB9;HOXC10;HOXC11;HOXC12;HOXC13;HOXC4;HOXC5;HOXC6;HOXC8;HOXC9;HOXD1;HOXD10;HOXD11;HOXD12;HOXD13;HOXD3;HOXD4;HOXD8;HOXD9;MEOX1;MEOX2;MNX1;PDX1;POU1F1;POU2F1;POU2F2;POU2F3;POU3F1;POU3F2;POU3F3;POU3F4;POU4F1;POU4F2;POU4F3;POU5F1;POU5F1B;POU5F2;POU6F1;POU6F2	POU2F1;POU2F2;POU2F3
MGAP_HUMAN.H11MO.0.D	14	0.08187135	0.6586213	2.132.591	1,23E+07	0.4435152	0.6757909	bHLH-ZIP factors{1.2.6}; TBX6-related factors{6.5.5}	Mad-like factors{1.2.6.7}; MGA (MAD5){6.5.5.0.2}	MGA;TBX6	MGA
LEF1_HUMAN.H11MO.0.A	19	0.11111111	0.6579737	1.816.456	1,12E+07	0.6097731	0.8044238	TCF-7-related factors{4.1.3}	LEF-1 (TCF-1alpha) [1]{4.1.3.0.4}	LEF1;TAF1;TCF7;TCF7L1;TCF7L2	LEF1
JDP2_HUMAN.H11MO.0.D	17	0.09941520	0.6545507	1.908.172	1,15E+07	0.5864179	0.7863825	Fos-related factors{1.1.2}	ATF-3-like factors{1.1.2.2}	ATF3;FOS;FOSB;FOSL1;FOSL2;JDP2	ATF3;JDP2
MYC_HUMAN.H11MO.0.A	38	0.22222222	0.6508754	1.386.165	0.9599453	0.9274669	0.9809013	bHLH-ZIP factors{1.2.6}	Myc / Max factors{1.2.6.5}	MAX;MITF;MLXIPL;MLX;MXI1;MYCN;MYC;SREBF1;SREBF2;TFAP4;TFE3;TFEB;USF1;USF2	MAX;MYCN;MYC
FOXK1_HUMAN.H11MO.0.A	20	0.11695906	0.6496875	1.751.325	1,09E+07	0.7108074	0.8746379	Forkhead box (FOX) factors{3.3.1}	FOXK{3.3.1.11}	FOXA1;FOXA2;FOXA3;FOXB1;FOXB2;FOXC1;FOXC2;FOXD1;FOXD2;FOXD3;FOXD4;FOXD4L1;FOXD4L3;FOXD4L4;FOXD4L5;FOXD4L6;FOXE1;FOXE3;FOXF1;FOXF2;FOXG1;FOXH1;FOXI1;FOXI2;FOXI3;FOXJ1;FOXJ2;FOXJ3;FOXK1;FOXK2;FOXL1;FOXL2;FOXM1;FOXN1;FOXN2;FOXN3;FOXN4;FOXO1;FOXO3;FOXO4;FOXO6;FOXP1;FOXP2;FOXP3;FOXP4;FOXQ1;FOXR1;FOXR2;FOXS1	FOXK1
HMX3_HUMAN.H11MO.0.D	9	0.05263158	0.6481933	28.122.146	1,44E+07	0.304472806	0.54592682	NK-related factors{3.1.2}	NK-5/HMX{3.1.2.18}	BARHL1;BARHL2;BARX1;BARX2;BSX;DBX1;DBX2;DLX1;DLX2;DLX3;DLX4;DLX5;DLX6;EMX1;EMX2;EN1;EN2;HHEX;HLX;HMX1;HMX2;HMX3;LBX1;LBX2;MSX1;MSX2;NANOG;NKX1-1;NKX1-2;NKX2-1;NKX2-2;NKX2-3;NKX2-4;NKX2-5;NKX2-6;NKX2-8;NKX3-1;NKX3-2;NKX6-1;NKX6-2;NKX6-3;NOTO;TLX1;TLX2;TLX3;VAX1;VAX2;VENTX	HMX1;HMX2;HMX3
HXC6_HUMAN.H11MO.0.D	14	0.08187135	0.6475583	20.967.518	1,21E+07	0.449010686	0.67661442	HOX-related factors{3.1.1}	HOX6-7{3.1.1.6}	CDX1;CDX2;CDX4;EVX1;EVX2;GBX1;GBX2;GSX1;GSX2;HDX;HMBOX1;HNF1A;HNF1B;HOXA1;HOXA10;HOXA11;HOXA13;HOXA2;HOXA3;HOXA4;HOXA5;HOXA6;HOXA7;HOXA9;HOXB1;HOXB13;HOXB2;HOXB3;HOXB4;HOXB5;HOXB6;HOXB7;HOXB8;HOXB9;HOXC10;HOXC11;HOXC12;HOXC13;HOXC4;HOXC5;HOXC6;HOXC8;HOXC9;HOXD1;HOXD10;HOXD11;HOXD12;HOXD13;HOXD3;HOXD4;HOXD8;HOXD9;MEOX1;MEOX2;MNX1;PDX1;POU1F1;POU2F1;POU2F2;POU2F3;POU3F1;POU3F2;POU3F3;POU3F4;POU4F1;POU4F2;POU4F3;POU5F1;POU5F2;POU6F1;POU6F2	HOXA7;HOXB6;HOXB7;HOXC6
NANOG_HUMAN.H11MO.0.A	14	0.08187135	0.6470103	20.949.766	1,21E+07	0.449321122	0.67661442	NK-related factors{3.1.2}	NANOG{3.1.2.12}	BARHL1;BARHL2;BARX1;BARX2;BSX;DBX1;DBX2;DLX1;DLX2;DLX3;DLX4;DLX5;DLX6;EMX1;EMX2;EN1;EN2;HHEX;HLX;HMX1;HMX2;HMX3;LBX1;LBX2;MSX1;MSX2;NANOG;NKX1-1;NKX1-2;NKX2-1;NKX2-2;NKX2-3;NKX2-4;NKX2-5;NKX2-6;NKX2-8;NKX3-1;NKX3-2;NKX6-1;NKX6-2;NKX6-	NANOG

motif	Nº of probes	% of probes	lower OR	upper OR	OR	p.value	FDR	TF family	TF subfamily	TF.family.member	TF.subfamily.member
										3;NOTO;TLX1;TLX2;TLX3;VAX1;VAX2;VENTX	
XBP1_HUMAN.H11MO.0.D	52	0.30409357	0.6446017	12.725.414	0.9111451	0.624086458	0.81312329	XBP-1-related factors{1.1.5}	XBP-1{1.1.5.0.1}	XBP1	XBP1
STAT6_HUMAN.H11MO.0.B	17	0.09941520	0.6439061	18.772.000	1,13E+07	0.590057451	0.78638246	STAT factors{6.2.1}	STAT6{6.2.1.0.7}	STAT1;STAT2;STAT3;STAT4;STAT5A;STAT5B;STAT6	STAT6
MYOD1_HUMAN.H11MO.0.A	43	0.25146199	0.6424301	13.237.801	0.9301713	0.729320591	0.87723272	MyoD / ASC-related factors{1.2.2}	Myogenic transcription factors{1.2.2.1}	ASCL1;ASCL2;ASCL3;ASCL4;ASCL5;MYF5;MYF6;MYOD1;MYOG	MYF6;MYOD1;MYOG
ELK4_HUMAN.H11MO.0.A	35	0.20467836	0.6399322	13.961.566	0.9566531	0.925381575	0.98004010	Ets-related factors{3.5.2}	Elk-like factors{3.5.2.2}	EHF;ELF1;ELF2;ELF3;ELF4;ELF5;ELK1;ELK3;ELK4;ERF;ERG;ETS1;ETS2;ETV1;ETV2;ETV3;ETV3L;ETV4;ETV5;ETV6;ETV7;FEV;FLI1;GABPA;SPDEF;SPI1;SPIB;SPIC	ELK1;ELK3;ELK4;ETV1;ETV4;ETV5
VDR_HUMAN.H11MO.1.A	22	0.12865497	0.6393910	16.523.284	1,05E+07	0.815652875	0.92616843	Thyroid hormone receptor-related factors (NR1){2.1.2}	Vitamin D receptor (NR1I){2.1.2.4}	NR1D1;NR1D2;NR1H2;NR1H3;NR1H4;NR1I2;NR1I3;PPARA;PPARD;PPARG;RARA;RARB;RARG;RORA;RORB;RORC;THRA;THRB;VDR	NR1I2;NR1I3;VDR
FOXD3_HUMAN.H11MO.0.D	20	0.11695906	0.6392284	17.230.838	1,08E+07	0.713570379	0.87463785	Forkhead box (FOX) factors{3.3.1}	FOXD{3.3.1.4}	FOXA1;FOXA2;FOXA3;FOXB1;FOXB2;FOXC1;FOXC2;FOXD1;FOXD2;FOXD3;FOXD4;FOXD4L1;FOXD4L3;FOXD4L4;FOXD4L5;FOXD4L6;FOXE1;FOXE3;FOXF1;FOXF2;FOXG1;FOXH1;FOXI1;FOXI2;FOXI3;FOXJ1;FOXJ2;FOXJ3;FOXK1;FOXK2;FOXL1;FOXL2;FOXM1;FOXN1;FOXN2;FOXN3;FOXN4;FOXO1;FOXO3;FOXO4;FOXO6;FOXP1;FOXP2;FOXP3;FOXP4;FOXQ1;FOXR1;FOXR2;FOXS1	FOXD1;FOXD2;FOXD3
PO4F2_HUMAN.H11MO.0.D	11	0.06432749	0.6372493	23.964.505	1,30E+07	0.377865678	0.62250948	POU domain factors{3.1.10}	POU4 (Brn-3-like factors){3.1.10.4}	CDX1;CDX2;CDX4;EVX1;EVX2;GBX1;GBX2;GSX1;GSX2;HDX;HMBOX1;HNF1A;HNF1B;HOXA1;HOXA10;HOXA11;HOXA13;HOXA2;HOXA3;HOXA4;HOXA5;HOXA6;HOXA7;HOXA9;HOXB1;HOXB13;HOXB2;HOXB3;HOXB4;HOXB5;HOXB6;HOXB7;HOXB8;HOXB9;HOXC10;HOXC11;HOXC12;HOXC13;HOXC4;HOXC5;HOXC6;HOXC8;HOXC9;HOXD1;HOXD10;HOXD11;HOXD12;HOXD13;HOXD3;HOXD4;HOXD8;HOXD9;MEOX1;MEOX2;MNX1;PDX1;POU1F1;POU2F1;POU2F2;POU2F3;POU3F1;POU3F2;POU3F3;POU3F4;POU4F1;POU4F2;POU4F3;POU5F1;POU5F1B;POU5F2;POU6F1;POU6F2	POU4F1;POU4F2;POU4F3
RFX1_HUMAN.H11MO.1.B	20	0.11695906	0.6372134	17.176.414	1,07E+07	0.714244824	0.87463785	RFX-related factors{3.3.3}	RFX1 (EF-C){3.3.3.0.1}	RFX1;RFX2;RFX3;RFX4;RFX5;RFX6;RFX7;RFX8	RFX1
SIX1_HUMAN.H11MO.0.A	13	0.07602339	0.6365952	21.530.614	1,22E+07	0.432525848	0.67123815	HD-SINE factors{3.1.6}	SIX1-like factors{3.1.6.1}	SIX1;SIX2;SIX3;SIX4;SIX5;SIX6	SIX1;SIX2
BHE23_HUMAN.H11MO.0.D	16	0.09356725	0.6356137	19.121.978	1,14E+07	0.578984818	0.78452952	Tal-related factors{1.2.3}	Neurogenin / Atonal-like factors{1.2.3.4}	ATOH1;ATOH7;ATOH8;BHLHA15;BHLHA9;BHLHE22;BHLHE23;FERD3L;FIGLA;HAND1;HAND2;LYL1;MESP1;MESP2;MSC;MSGN1;NEUROD1;NEUROD2;NEUROD4;NEUROD6;NEUROG1;NEUROG2;NEUROG3;NHLH1;NHLH2;OLIG1;OLIG2;OLIG3;PTF1A;SCX;TAL1;TAL2;TCF15;TCF21;TCF23;TWIST1;TWIST2	ATOH1;BHLHA15;BHLHE22;BHLHE23;NEUROD1;NEUROD2;NEUROG2;OLIG1;OLIG2;OLIG3
MEF2D_HUMAN.H11MO.0.A	18	0.10526316	0.6350134	17.991.636	1,10E+07	0.696813244	0.86932526	Regulators of differentiation{5.1.1}	MEF-2{5.1.1.1}	MEF2A;MEF2B;MEF2C;MEF2D	MEF2A;MEF2B;MEF2C;MEF2D

motif	Nº of probes	% of probes	lower OR	upper OR	OR	p.value	FDR	TF family	TF subfamily	TF.family.member	TF.subfamily.member
ZN547_HUMAN.H11MO.0.C	20	0.11695906	0.6349318	17.115.550	1,07E+07	0.715054122	0.87463785	More than 3 adjacent zinc finger factors{2.3.3}	unclassified{2.3.3.0}	BCL6B;BCL6;CTCFL;CTCF;FEZF1;GFI1B;GFI1;GLI1;GLI2;GLI3;GLIS1;GLIS2;GLIS3;MTF1;MYNN;MZF1;OSR2;OVOL1;OVOL2;ZNF146;PLAG1;PLAGL1;PRDM14;PRDM1;PRDM6;SCRT1;SCRT2;SNAI1;SNAI2;YY1;YY2;WT1;ZNF324;ZNF354A;ZBTB14;ZBTB18;ZBTB48;ZBTB49;ZBTB7A;ZBTB7B;ZBTB6;ZFP64;ZFP28;ZFP42;ZFP82;ZFX;ZIC1;ZIC2;ZIC3;ZIC4;ZIM3;ZKSCAN1;ZKSCAN3;ZNF121;ZNF136;ZNF140;ZNF143;ZNF148;ZNF214;ZNF232;ZNF250;ZNF257;ZNF260;ZNF263;ZNF264;ZNF274;ZNF281;ZNF282;ZNF317;ZNF320;ZNF322;ZNF329;ZNF331;ZNF333;ZNF350;ZNF384;ZNF394;ZNF410;ZNF436;ZNF449;ZNF490;ZNF502;ZNF524;ZNF528;ZNF547;ZNF549;ZNF554;ZNF563;ZNF582;ZNF586;ZNF589;ZNF652;ZNF667;ZNF680;ZNF708;ZNF713;ZNF768;ZNF816;ZNF18;ZNF41;ZNF76;ZNF85;ZSCAN16;ZSCAN22;ZSCAN31;ZSCAN4	MYNN;MZF1;OSR2;PRDM14;PRDM6;WT1;ZBTB14;ZBTB48;ZBTB49;ZFP64;ZFP28;ZIM3;ZNF121;ZNF250;ZNF257;ZNF263;ZNF274;ZNF317;ZNF320;ZNF329;ZNF331;ZNF394;ZNF449;ZNF502;ZNF528;ZNF547;ZNF549;ZNF554;ZNF586;ZNF589;ZNF667;ZNF680;ZNF708;ZNF713;ZNF768;ZNF18;ZNF85;ZSCAN16;ZSCAN22
RXRB_HUMAN.H11MO.0.C	25	0.14619883	0.6342651	15.554.925	1,01E+07	0.913549514	0.97058566	RXR-related receptors (NR2){2.1.3}	Retinoid X receptors (NR2B){2.1.3.1}	HNF4A;HNF4G;NR2C1;NR2C2;NR2E1;NR2E3;NR2F1;NR2F2;NR2F6;RXRA;RXRB;RXRG	RXRA;RXRB;RXRG
ALX4_HUMAN.H11MO.0.D	11	0.06432749	0.6316147	23.751.818	1,29E+07	0.380957999	0.62413190	Paired-related HD factors{3.1.3}	ALX{3.1.3.1}	ALX1;ALX3;ALX4;ARGFX;ARX;CRX;DMBX1;DPRX;DRGX;DUX4;DUXA;ESX1;GSC;GSC2;HESX1;ISX;LEUTX;MIXL1;NOBOX;OTP;OTX1;OTX2;PHOX2A;PHOX2B;PITX1;PITX2;PITX3;PROP1;PRRX1;PRRX2;RAX;RAX2;RHOXF1;RHOXF2;SEBOX;SHOX;SHOX2;TPRX1;UNCX;VSX1;VSX2	ALX1;ALX3;ALX4
ZN784_HUMAN.H11MO.0.D	22	0.12865497	0.6305586	16.295.607	1,04E+07	0.817391031	0.92677718	Factors with multiple dispersed zinc fingers{2.3.4}	unclassified{2.3.4.0}	BCL11A;E4F1;MECOM;HIC1;HIC2;HINFP;IKZF1;INSM1;MAZ;PATZ1;PRDM4;REST;RREB1;SALL4;VEZF1;ZBTB17;ZBTB4;HIVEP1;HIVEP2;ZNF134;ZNF219;ZNF335;ZNF341;ZNF382;ZNF418;ZNF423;ZNF467;ZNF770;ZNF784;ZNF8	E4F1;PRDM4;REST;RREB1;ZBTB17;ZBTB4;ZNF335;ZNF341;ZNF467;ZNF770;ZNF784;ZNF8
DLX5_HUMAN.H11MO.0.D	11	0.06432749	0.6296901	23.680.041	1,29E+07	0.382088531	0.62413190	NK-related factors{3.1.2}	DLX{3.1.2.5}	BARHL1;BARHL2;BARX1;BARX2;BSX;DBX1;DBX2;DLX1;DLX2;DLX3;DLX4;DLX5;DLX6;EMX1;EMX2;EN1;EN2;HHEX;HLX;HMX1;HMX2;HMX3;LBX1;LBX2;MSX1;MSX2;NANOG;NKX1-1;NKX1-2;NKX2-1;NKX2-2;NKX2-3;NKX2-4;NKX2-5;NKX2-6;NKX2-8;NKX3-1;NKX3-2;NKX6-1;NKX6-2;NKX6-3;NOTO;TLX1;TLX2;TLX3;VAX1;VAX2;VENTX	DLX1;DLX2;DLX3;DLX4;DLX5;DLX6
ZN382_HUMAN.H11MO.0.C	16	0.09356725	0.6294130	18.933.851	1,13E+07	0.581976360	0.78480889	Factors with multiple dispersed zinc fingers{2.3.4}	ZNF37A-like factors{2.3.4.25}	BCL11A;BCL11B;BNC1;BNC2;E4F1;HIC1;HIC2;HINFP;HIVEP1;HIVEP2;HIVEP3;IKZF1;IKZF2;IKZF3;IKZF4;IKZF5;INSM1;INSM2;MAZ;MECOM;PATZ1;PRDM16;PRDM4;REST;RLF;RREB1;SALL1;SALL2;SALL3;SALL4;VEZF1;ZBTB1;ZBTB17;ZBTB2;ZBTB25;ZBTB4;ZFAT;ZNF134;ZNF211;ZNF217;ZNF219;ZNF248;ZNF256;ZNF292;ZNF296;ZNF319;ZNF334;ZNF335;ZNF341;ZNF37A;ZNF382;ZNF417;ZNF418;ZNF423;ZNF467;ZNF510;ZNF512;ZNF512B;ZNF516;ZNF518A;ZNF518B;ZNF521;ZNF526;ZNF532;ZNF536;ZNF552;ZNF574;ZNF587;ZNF587B;ZNF592;ZNF639;ZNF654;ZNF658;ZNF671;ZNF687;ZNF711;ZNF717;ZNF770;ZNF772;ZNF784;ZNF786;ZNF792;ZNF8;ZNF814	ZNF382
HLTF_HUMAN.H11MO.0.D	18	0.10526316	0.6281618	17.797.645	1,09E+07	0.698744042	0.87032578	NA	NA	HLTF	HLTF
HXB2_HUMAN.H11MO.0.D	14	0.08187135	0.6273982	20.313.896	1,17E+07	0.548550366	0.75963080	HOX-related factors{3.1.1}	HOX2{3.1.1.2}	CDX1;CDX2;CDX4;EVX1;EVX2;GBX1;GBX2;GSX1;GSX2;HDX;HMBOX1;HNF1A;HNF1B;HOXA1;HOXA10;HOXA11;HOXA13;HOXA2;HOXA3;HOXA4;HOXA5;HOXA6;HOXA7;HOXA9;HOXB1;HOXB13;HOXB2;HOXB3;HOXB4;HOXB	HOXA2;HOXB2

motif	Nº of probes	% of probes	lower OR	upper OR	OR	p.value	FDR	TF family	TF subfamily	TF.family.member	TF.subfamily.member
										5;HOXB6;HOXB7;HOXB8;HOXB9;HOXC10;HOXC11;HOXC12;HOXC13;HOXC4;HOXC5;HOXC6;HOXC8;HOXC9;HOXD1;HOXD10;HOXD11;HOXD12;HOXD13;HOXD3;HOXD4;HOXD8;HOXD9;MEOX1;MEOX2;MNX1;PDX1;POU1F1;POU2F1;POU2F2;POU2F3;POU3F1;POU3F2;POU3F3;POU3F4;POU4F1;POU4F2;POU4F3;POU5F1;POU5F2;POU6F1;POU6F2	
SCRT1_HUMAN.H11MO.0.D	14	0.08187135	0.6268078	20.294.851	1,17E+07	0.548786454	0.75963080	More than 3 adjacent zinc finger factors{2.3.3}	Snail-like factors{2.3.3.2}	BCL6;BCL6B;CTCF;CTCFL;FEZF1;FEZF2;GFI1;GFI1B;GLI1;GLI2;GLI3;GLI4;GLIS1;GLIS2;GLIS3;HKR1;MTF1;MYNN;MZF1;OSR2;OVOL1;OVOL2;PLAG1;PLAGL1;PLAGL2;PRDM1;PRDM14;PRDM6;SCRT1;SCRT2;SNAI1;SNAI2;SNAI3;WT1;YY1;YY2;ZBTB12;ZBTB14;ZBTB18;ZBTB20;ZBTB26;ZBTB42;ZBTB45;ZBTB47;ZBTB48;ZBTB49;ZBTB6;ZBTB7A;ZBTB7B;ZBTB7C;ZFP14;ZFP2;ZFP28;ZFP30;ZFP37;ZFP42;ZFP64;ZFP69;ZFP69B;ZFP82;ZFP91;ZFX;ZIC1;ZIC2;ZIC3;ZIC4;ZIC5;ZIK1;ZIM3;ZKSCAN1;ZKSCAN2;ZKSCAN3;ZKSCAN4;ZNF121;ZNF124;ZNF133;ZNF136;ZNF138;ZNF14;ZNF140;ZNF143;ZNF146;ZNF148;ZNF155;ZNF157;ZNF160;ZNF169;ZNF175;ZNF177;ZNF18;ZNF180;ZNF181;ZNF2;ZNF20;ZNF212;ZNF213;ZNF214;ZNF221;ZNF222;ZNF223;ZNF224;ZNF225;ZNF226;ZNF227;ZNF229;ZNF230;ZNF232;ZNF233;ZNF234;ZNF235;ZNF24;ZNF25;ZNF250;ZNF257;ZNF26;ZNF260;ZNF263;ZNF264;ZNF268;ZNF274;ZNF276;ZNF28;ZNF280A;ZNF280B;ZNF280C;ZNF280D;ZNF281;ZNF282;ZNF283;ZNF284;ZNF285;ZNF286A;ZNF286B;ZNF3;ZNF30;ZNF300;ZNF302;ZNF317;ZNF32;ZNF320;ZNF322;ZNF324;ZNF324B;ZNF329;ZNF331;ZNF333;ZNF33A;ZNF33B;ZNF343;ZNF345;ZNF347;ZNF350;ZNF354A;ZNF354B;ZNF362;ZNF366;ZNF383;ZNF384;ZNF394;ZNF397;ZNF398;ZNF404;ZNF41;ZNF410;ZNF419;ZNF420;ZNF431;ZNF432;ZNF436;ZNF439;ZNF44;ZNF440;ZNF442;ZNF443;ZNF446;ZNF449;ZNF45;ZNF460;ZNF468;ZNF479;ZNF484;ZNF490;ZNF500;ZNF502;ZNF524;ZNF525;ZNF528;ZNF543;ZNF544;ZNF546;ZNF547;ZNF548;ZNF549;ZNF554;ZNF555;ZNF557;ZNF558;ZNF559;ZNF561;ZNF562;ZNF563;ZNF564;ZNF566;ZNF567;ZNF568;ZNF57;ZNF570;ZNF571;ZNF572;ZNF577;ZNF581;ZNF582;ZNF583;ZNF585A;ZNF586;ZNF589;ZNF595;ZNF599;ZNF600;ZNF605;ZNF607;ZNF611;ZNF613;ZNF614;ZNF615;ZNF616;ZNF619;ZNF620;ZNF621;ZNF625;ZNF627;ZNF649;ZNF652;ZNF653;ZNF665;ZNF667;ZNF669;ZNF670;ZNF672;ZNF679;ZNF680;ZNF683;ZNF689;ZNF692;ZNF701;ZNF705D;ZNF705E;ZNF705G;ZNF708;ZNF709;ZNF71;ZNF710;ZNF713;ZNF721;ZNF727;ZNF729;ZNF736;ZNF75A;ZNF75D;ZNF76;ZNF763;ZNF764;ZNF765;ZNF768;ZNF77;ZNF771;ZNF773;ZNF774;ZNF776;ZNF777;ZNF780A;ZNF780B;ZNF782;ZNF785;ZNF799;ZNF805;ZNF808;ZNF81;ZNF813;ZNF816;ZNF823;ZNF829;ZNF836;ZNF841;ZNF844;ZNF845;ZNF846;ZNF85;ZNF853;ZNF860;ZNF878;ZNF891;ZNF99;ZSCAN16;ZSCAN2;ZSCAN22;ZSCAN23;ZSCAN29;ZSCAN31;ZSCAN32;ZSCAN4;ZSCAN5A;ZSCAN5B;ZSCAN5C;ZSCAN9;ZXDA;ZXDB;ZXDC	SCRT1;SCRT2;SNAI1;SNAI2
FEV_HUMAN.H11MO.0.B	43	0.25146199	0.6218445	12.814.548	0.9004176	0.606069738	0.80150904	Ets-related factors{3.5.2}	Ets-like factors{3.5.2.1}	EHF;ELF1;ELF2;ELF3;ELF4;ELF5;ELK1;ELK3;ELK4;ERF;ERG;ETS1;ETS2;ETV1;ETV2;ETV3;ETV3L;ETV4;ETV5;ETV6;ETV7;FEV;FLI1;GABPA;SPDEF;SPI1;SPIB;SPIC	ERG;ETS1;ETS2;ETV2;ETV3;FEV;FLI1;GABPA

motif	Nº of probes	% of probes	lower OR	upper OR	OR	p.value	FDR	TF family	TF subfamily	TF.family.member	TF.subfamily.member
P53_HUMAN.H11MO.1.A	22	0.12865497	0.6190995	15.999.659	1,02E+07	0.908434240	0.96740718	p53-related factors{6.3.1}	p53{6.3.1.0.1}	TP53;TP63;TP73	TP53
NFAC4_HUMAN.H11MO.0.C	13	0.07602339	0.6186949	20.924.737	1,19E+07	0.532270353	0.74951747	NFAT-related factors{6.1.3}	NFATc4 (NFAT3){6.1.3.0.4}	NFAT5;NFATC1;NFATC2;NFATC3;NFATC4	NFATC4
HSF1_HUMAN.H11MO.1.A	15	0.08771930	0.6181925	19.254.952	1,13E+07	0.667828562	0.84911706	HSF factors{3.4.1}	HSF1 (HSTF1){3.4.1.0.1}	HSF1;HSF2;HSF4;HSF5;HSFX1;HSFY1;HSFY1;HSFY2	HSF1
CXXC1_HUMAN.H11MO.0.D	36	0.21052632	0.6173105	13.355.008	0.9185092	0.714253201	0.87463785	CpG-binding proteins{2.6.1}	CpG-binding protein (CXXC1, CFP1, CGBP, PCCX1, PHF18){2.6.1.0.1}	CXXC1;DNMT1;KDM2A;KMT2A;KMT2B;MBD1;TET1	CXXC1
TF7L2_HUMAN.H11MO.0.A	13	0.07602339	0.6172785	20.876.851	1,19E+07	0.532878750	0.74951747	TCF-7-related factors{4.1.3}	TCF-7L2 (TCF-4) [1]{4.1.3.0.3}	LEF1;TAF1;TCF7;TCF7L1;TCF7L2	TCF7L2
MAF_HUMAN.H11MO.1.B	17	0.09941520	0.6159435	17.955.585	1,09E+07	0.692394588	0.86789430	Maf-related factors{1.1.3}	Large Maf factors{1.1.3.1}	MAF;MAFA;MAFB;MAFF;MAFG;MAFK;NRL	MAFA;MAFB;MAF;NRL
ATF4_HUMAN.H11MO.0.A	11	0.06432749	0.6145893	23.111.393	1,26E+07	0.486556932	0.70450934	ATF-4-related factors{1.1.6}	ATF-4{1.1.6.0.1}	ATF4;ATF5	ATF4
SOX3_HUMAN.H11MO.0.B	18	0.10526316	0.6137471	17.389.266	1,06E+07	0.797832676	0.90911388	SOX-related factors{4.1.1}	Group B{4.1.1.2}	BBX;CIC;HBP1;SOX1;SOX10;SOX11;SOX12;SOX13;SOX14;SOX15;SOX17;SOX18;SOX2;SOX21;SOX3;SOX30;SOX4;SOX5;SOX6;SOX7;SOX8;SOX9;SRY	SOX1;SOX21;SOX2;SOX3
ZN502_HUMAN.H11MO.0.C	13	0.07602339	0.6133757	20.745.828	1,18E+07	0.534675235	0.74951747	More than 3 adjacent zinc finger factors{2.3.3}	unclassified{2.3.3.0}	BCL6B;BCL6;CTCFL;CTCF;FEZF1;GFI1B;GFI1;GLI1;GLI2;GLI3;GLIS1;GLIS2;GLIS3;MTF1;MYNN;MZF1;OSR2;OVOL1;OVOL2;ZNF146;PLAG1;PLAGL1;PRDM14;PRDM1;PRDM6;SCRT1;SCRT2;SNAI1;SNAI2;YY1;YY2;WT1;ZNF324;ZNF354A;ZBTB14;ZBTB18;ZBTB48;ZBTB49;ZBTB7A;ZBTB7B;ZBTB6;ZFP64;ZFP28;ZFP42;ZFP82;ZFX;ZIC1;ZIC2;ZIC3;ZIC4;ZIM3;ZKSCAN1;ZKSCAN3;ZNF121;ZNF136;ZNF140;ZNF143;ZNF148;ZNF214;ZNF232;ZNF250;ZNF257;ZNF260;ZNF263;ZNF264;ZNF274;ZNF281;ZNF282;ZNF317;ZNF320;ZNF322;ZNF329;ZNF331;ZNF333;ZNF350;ZNF384;ZNF394;ZNF410;ZNF436;ZNF449;ZNF490;ZNF502;ZNF524;ZNF528;ZNF547;ZNF549;ZNF554;ZNF563;ZNF582;ZNF586;ZNF589;ZNF652;ZNF667;ZNF680;ZNF708;ZNF713;ZNF768;ZNF816;ZNF18;ZNF41;ZNF76;ZNF85;ZSCAN16;ZSCAN22;ZSCAN31;ZSCAN4	MYNN;MZF1;OSR2;PRDM14;PRDM6;WT1;ZBTB14;ZBTB48;ZBTB49;ZFP64;ZFP28;ZIM3;ZNF121;ZNF250;ZNF257;ZNF263;ZNF274;ZNF317;ZNF320;ZNF329;ZNF331;ZNF394;ZNF449;ZNF502;ZNF528;ZNF547;ZNF549;ZNF554;ZNF586;ZNF589;ZNF667;ZNF680;ZNF708;ZNF713;ZNF768;ZNF18;ZNF85;ZSCAN16;ZSCAN22
HXC13_HUMAN.H11MO.0.D	11	0.06432749	0.6109449	22.974.592	1,25E+07	0.487780045	0.70450934	HOX-related factors{3.1.1}	HOX9-13{3.1.1.8}	CDX1;CDX2;CDX4;EVX1;EVX2;GBX1;GBX2;GSX1;GSX2;HDX;HMBOX1;HNF1A;HNF1B;HOXA1;HOXA10;HOXA11;HOXA13;HOXA2;HOXA3;HOXA4;HOXA5;HOXA6;HOXA7;HOXA9;HOXB1;HOXB13;HOXB2;HOXB3;HOXB4;HOXB5;HOXB6;HOXB7;HOXB8;HOXB9;HOXC10;HOXC11;HOXC12;HOXC13;HOXC4;HOXC5;HOXC6;HOXC8;HOXC9;HOXD1;HOXD10;HOXD11;HOXD12;HOXD13;HOXD3;HOXD4;HOXD8;HOXD9;MEOX1;MEOX2;MNX1;PDX1;POU1F1;POU2F1;POU2F2;POU2F3;POU3F1;POU3F2;POU3F3;POU3F4;POU4F1;POU4F2;POU4F3;POU5F1;POU5F2;POU6F1;POU6F2	HOXA10;HOXA11;HOXA13;HOXA9;HOXB13;HOXC10;HOXC11;HOXC12;HOXC13;HOXC9;HOXD10;HOXD11;HOXD12;HOXD13;HOXD9
TFEB_HUMAN.H11MO.0.C	26	0.15204678	0.6103849	14.742.703	0.9660609	1000000000,00000000	1,00E+08	bHLH-ZIP factors{1.2.6}	TFE3-like factors{1.2.6.1}	MAX;MITF;MLX;MLXIP;MLXIPL;MNT;MXD1;MXD3;MXD4;MXI1;MYC;MYCL;MYCN;REPIN1;SREBF1;SREBF2;TFAP4;TFE3;TFEB;TFEC;USF1;USF2	MITF;TFE3;TFEB

motif	Nº of probes	% of probes	lower OR	upper OR	OR	p.value	FDR	TF family	TF subfamily	TF.family.member	TF.subfamily.member
HXA2_HUMAN.H11MO.0.D	11	0.06432749	0.6102780	22.949.567	1,25E+07	0.488018588	0.70450934	HOX-related factors{3.1.1}	HOX2{3.1.1.2}	CDX1;CDX2;CDX4;EVX1;EVX2;GBX1;GBX2;GSX1;GSX2;HDX;HMBOX1;HNF1A;HNF1B;HOXA1;HOXA10;HOXA11;HOXA13;HOXA2;HOXA3;HOXA4;HOXA5;HOXA6;HOXA7;HOXA9;HOXB1;HOXB13;HOXB2;HOXB3;HOXB4;HOXB5;HOXB6;HOXB7;HOXB8;HOXB9;HOXC10;HOXC11;HOXC12;HOXC13;HOXC4;HOXC5;HOXC6;HOXC8;HOXC9;HOXD1;HOXD10;HOXD11;HOXD12;HOXD13;HOXD3;HOXD4;HOXD8;HOXD9;MEOX1;MEOX2;MNX1;PDX1;POU1F1;POU2F1;POU2F2;POU2F3;POU3F1;POU3F2;POU3F3;POU3F4;POU4F1;POU4F2;POU4F3;POU5F1;POU5F2;POU6F1;POU6F2	HOXA2;HOXB2
ZNF76_HUMAN.H11MO.0.C	38	0.22222222	0.6093218	12.977.086	0.8986818	0.592802724	0.78801879	More than 3 adjacent zinc finger factors{2.3.3}	ZNF76-like factors{2.3.3.28}	BCL6;BCL6B;CTCF;CTCFL;FEZF1;FEZF2;GFI1;GFI1B;GLI1;GLI2;GLI3;GLI4;GLIS1;GLIS2;GLIS3;HKR1;MTF1;MYNN;MZF1;OSR2;OVOL1;OVOL2;PLAG1;PLAGL1;PLAGL2;PRDM1;PRDM14;PRDM6;SCRT1;SCRT2;SNAI1;SNAI2;SNAI3;WT1;YY1;YY2;ZBTB12;ZBTB14;ZBTB18;ZBTB20;ZBTB26;ZBTB42;ZBTB45;ZBTB47;ZBTB48;ZBTB49;ZBTB6;ZBTB7A;ZBTB7B;ZBTB7C;ZFP14;ZFP2;ZFP28;ZFP30;ZFP37;ZFP42;ZFP64;ZFP69;ZFP69B;ZFP82;ZFP91;ZFX;ZIC1;ZIC2;ZIC3;ZIC4;ZIC5;ZIK1;ZIM3;ZKSCAN1;ZKSCAN2;ZKSCAN3;ZKSCAN4;ZNF121;ZNF124;ZNF133;ZNF136;ZNF138;ZNF14;ZNF140;ZNF143;ZNF146;ZNF148;ZNF155;ZNF157;ZNF160;ZNF169;ZNF175;ZNF177;ZNF18;ZNF180;ZNF181;ZNF2;ZNF20;ZNF212;ZNF213;ZNF214;ZNF221;ZNF222;ZNF223;ZNF224;ZNF225;ZNF226;ZNF227;ZNF229;ZNF230;ZNF232;ZNF233;ZNF234;ZNF235;ZNF24;ZNF25;ZNF250;ZNF257;ZNF26;ZNF260;ZNF263;ZNF264;ZNF268;ZNF274;ZNF276;ZNF28;ZNF280A;ZNF280B;ZNF280C;ZNF280D;ZNF281;ZNF282;ZNF283;ZNF284;ZNF285;ZNF286A;ZNF286B;ZNF3;ZNF30;ZNF300;ZNF302;ZNF317;ZNF32;ZNF320;ZNF322;ZNF324;ZNF324B;ZNF329;ZNF331;ZNF333;ZNF33A;ZNF33B;ZNF343;ZNF345;ZNF347;ZNF350;ZNF354A;ZNF354B;ZNF362;ZNF366;ZNF383;ZNF384;ZNF394;ZNF397;ZNF398;ZNF404;ZNF41;ZNF410;ZNF419;ZNF420;ZNF431;ZNF432;ZNF436;ZNF439;ZNF44;ZNF440;ZNF442;ZNF443;ZNF446;ZNF449;ZNF45;ZNF460;ZNF468;ZNF479;ZNF484;ZNF490;ZNF500;ZNF502;ZNF524;ZNF525;ZNF528;ZNF543;ZNF544;ZNF546;ZNF547;ZNF548;ZNF549;ZNF554;ZNF555;ZNF557;ZNF558;ZNF559;ZNF561;ZNF562;ZNF563;ZNF564;ZNF566;ZNF567;ZNF568;ZNF57;ZNF570;ZNF571;ZNF572;ZNF577;ZNF581;ZNF582;ZNF583;ZNF585A;ZNF586;ZNF589;ZNF595;ZNF599;ZNF600;ZNF605;ZNF607;ZNF611;ZNF613;ZNF614;ZNF615;ZNF616;ZNF619;ZNF620;ZNF621;ZNF625;ZNF627;ZNF649;ZNF652;ZNF653;ZNF665;ZNF667;ZNF669;ZNF670;ZNF672;ZNF679;ZNF680;ZNF683;ZNF689;ZNF692;ZNF701;ZNF705D;ZNF705E;ZNF705G;ZNF708;ZNF709;ZNF71;ZNF710;ZNF713;ZNF721;ZNF727;ZNF729;ZNF736;ZNF75A;ZNF75D;ZNF76;ZNF763;ZNF764;ZNF765;ZNF768;ZNF77;ZNF771;ZNF773;ZNF774;ZNF776;ZNF777;ZNF780A;ZNF780B;ZNF782;ZNF785;ZNF799;ZNF805;ZNF808;ZNF81;ZNF813;ZNF816;ZNF823;ZNF829;ZNF836;ZNF841;ZNF844;ZNF845;ZNF846;ZNF85;ZNF853;ZNF860;ZNF878;ZNF891;ZNF99;ZSCAN16;ZSCAN2;ZSCAN22;ZSCAN23;ZSCAN29;ZSCAN31;Z	ZNF143;ZNF76

motif	Nº of probes	% of probes	lower OR	upper OR	OR	p.value	FDR	TF family	TF subfamily	TF.family.member	TF.subfamily.member
										SCAN32;ZSCAN4;ZSCAN5A;ZSCAN5B;ZSCAN5C;ZSCAN9;ZXDA;ZXDB;ZXDC	
PRGR_HUMAN.H11MO.0.A	16	0.09356725	0.6081156	18.292.547	1,09E+07	0.683495518	0.86439904	Steroid hormone receptors (NR3){2.1.1}	GR-like receptors (NR3C){2.1.1.1}	AR;ESR1;ESR2;ESRRA;ESRRB;ESRRG;NR3C1;NR3C2;PGR	AR;NR3C1;NR3C2;PGR
ZBTB4_HUMAN.H11MO.1.D	26	0.15204678	0.6067980	14.656.369	0.9603820	0.916562512	0.97203535	Factors with multiple dispersed zinc fingers{2.3.4}	unclassified{2.3.4.0}	BCL11A;E4F1;MECOM;HIC1;HIC2;HINFP;IKZF1;INSM1;MAZ;PATZ1;PRDM4;REST;RREB1;SALL4;VEZF1;ZBTB17;ZBTB4;HIVEP1;HIVEP2;ZNF134;ZNF219;ZNF335;ZNF341;ZNF382;ZNF418;ZNF423;ZNF467;ZNF770;ZNF784;ZNF8	E4F1;PRDM4;REST;RREB1;ZBTB17;ZBTB4;ZNF335;ZNF341;ZNF467;ZNF770;ZNF784;ZNF8
STA5A_HUMAN.H11MO.0.A	15	0.08771930	0.6061118	18.877.719	1,11E+07	0.671187200	0.85112719	STAT factors{6.2.1}	STAT5A{6.2.1.0.5}	STAT1;STAT2;STAT3;STAT4;STAT5A;STAT5B;STAT6	STAT5A
ZN134_HUMAN.H11MO.1.C	18	0.10526316	0.6059132	17.167.168	1,05E+07	0.799454226	0.90911388	Factors with multiple dispersed zinc fingers{2.3.4}	ZNF134-like factors{2.3.4.24}	BCL11A;BCL11B;BNC1;BNC2;E4F1;HIC1;HIC2;HINFP;HIVEP1;HIVEP2;HIVEP3;IKZF1;IKZF2;IKZF3;IKZF4;IKZF5;INSM1;INSM2;MAZ;MECOM;PATZ1;PRDM16;PRDM4;REST;RLF;RREB1;SALL1;SALL2;SALL3;SALL4;VEZF1;ZBTB1;ZBTB17;ZBTB2;ZBTB25;ZBTB4;ZFAT;ZNF134;ZNF211;ZNF217;ZNF219;ZNF248;ZNF256;ZNF292;ZNF296;ZNF319;ZNF334;ZNF335;ZNF341;ZNF37A;ZNF382;ZNF417;ZNF418;ZNF423;ZNF467;ZNF510;ZNF512;ZNF512B;ZNF516;ZNF518A;ZNF518B;ZNF521;ZNF526;ZNF532;ZNF536;ZNF552;ZNF574;ZNF587;ZNF587B;ZNF592;ZNF639;ZNF654;ZNF658;ZNF671;ZNF687;ZNF711;ZNF717;ZNF770;ZNF772;ZNF784;ZNF786;ZNF792;ZNF8;ZNF814	ZNF134
ERR1_HUMAN.H11MO.0.A	19	0.11111111	0.6047087	16.693.663	1,03E+07	0.901651605	0.96284403	Steroid hormone receptors (NR3){2.1.1}	ER-like receptors (NR3A&B){2.1.1.2}	AR;ESR1;ESR2;ESRRA;ESRRB;ESRRG;NR3C1;NR3C2;PGR	ESRRA;ESRRB;ESRRG;ESR1;ESR2
PO5F1_HUMAN.H11MO.1.A	14	0.08187135	0.6000437	19.428.505	1,12E+07	0.658973851	0.84256856	POU domain factors{3.1.10}	POU5 (Oct-3/4-like factors){3.1.10.5}	CDX1;CDX2;CDX4;EVX1;EVX2;GBX1;GBX2;GSX1;GSX2;HDX;HMBOX1;HNF1A;HNF1B;HOXA1;HOXA10;HOXA11;HOXA13;HOXA2;HOXA3;HOXA4;HOXA5;HOXA6;HOXA7;HOXA9;HOXB1;HOXB13;HOXB2;HOXB3;HOXB4;HOXB5;HOXB6;HOXB7;HOXB8;HOXB9;HOXC10;HOXC11;HOXC12;HOXC13;HOXC4;HOXC5;HOXC6;HOXC8;HOXC9;HOXD1;HOXD10;HOXD11;HOXD12;HOXD13;HOXD3;HOXD4;HOXD8;HOXD9;MEOX1;MEOX2;MNX1;PDX1;POU1F1;POU2F1;POU2F2;POU2F3;POU3F1;POU3F2;POU3	POU5F1B;POU5F1

motif	Nº of probes	% of probes	lower OR	upper OR	OR	p.value	FDR	TF family	TF subfamily	TF.family.member	TF.subfamily.member
										F3;POU3F4;POU4F1;POU4F2;POU4F3;POU5F1;POU5F1B;POU5F2;POU6F1;POU6F2	
CEBPE_HUMAN.H11MO.0.A	10	0.05847953	0.5999371	24.090.008	1,28E+07	0.462865359	0.69026923	C/EBP-related{1.1.8}	C/EBP{1.1.8.1}	CEBPA;CEBPB;CEBPD;CEBPE;CEBPG;DBP;DDIT3;HLF;NFIL3;TEF	CEBPA;CEBPB;CEBPD;CEBPE;CEBPG;DDIT3
ASCL2_HUMAN.H11MO.0.D	38	0.22222222	0.5991970	12.761.203	0.8837468	0.534194836	0.74951747	MyoD / ASC-related factors{1.2.2}	Achaete-Scute-like factors{1.2.2.2}	ASCL1;ASCL2;ASCL3;ASCL4;ASCL5;MYF5;MYF6;MYOD1;MYOG	ASCL1;ASCL2
PAX6_HUMAN.H11MO.0.C	13	0.07602339	0.5983647	20.237.248	1,15E+07	0.643900724	0.83157028	Paired plus homeo domain{3.2.1}	PAX-4/6{3.2.1.2}	PAX3;PAX4;PAX6;PAX7	PAX4;PAX6
SMCA1_HUMAN.H11MO.0.C	16	0.09356725	0.5971249	17.961.381	1,07E+07	0.786325721	0.89949129	Myb/SANT domain factors{3.5.1}	SMARCA-like factors{3.5.1.5}	CDC5L;MYBL1;MYBL2;MYB;SMARCA1;SMARCA5	SMARCA1;SMARCA5
SPIC_HUMAN.H11MO.0.D	42	0.24561404	0.5968741	12.373.135	0.8671372	0.491889527	0.70623245	Ets-related factors{3.5.2}	Spi-like factors{3.5.2.5}	EHF;ELF1;ELF2;ELF3;ELF4;ELF5;ELK1;ELK3;ELK4;ERF;ERG;ETS1;ETS2;ETV1;ETV2;ETV3;ETV3L;ETV4;ETV5;ETV6;ETV7;FEV;FLI1;GABPA;SPDEF;SPI1;SPIB;SPIC	SPI1;SPIB;SPIC
ESR1_HUMAN.H11MO.1.A	26	0.15204678	0.5928622	14.319.545	0.9383233	0.835209872	0.93959204	Steroid hormone receptors (NR3){2.1.1}	ER-like receptors (NR3A&B){2.1.1.2}	AR;ESR1;ESR2;ESRRA;ESRRB;ESRRG;NR3C1;NR3C2;PGR	ESRRA;ESRRB;ESRRG;ESR1;ESR2
HXB4_HUMAN.H11MO.0.B	9	0.05263158	0.5917972	25.672.586	1,32E+07	0.432004924	0.67123815	HOX-related factors{3.1.1}	HOX4{3.1.1.4}	CDX1;CDX2;CDX4;EVX1;EVX2;GBX1;GBX2;GSX1;GSX2;HDX;HMBOX1;HNF1A;HNF1B;HOXA1;HOXA10;HOXA11;HOXA13;HOXA2;HOXA3;HOXA4;HOXA5;HOXA6;HOXA7;HOXA9;HOXB1;HOXB13;HOXB2;HOXB3;HOXB4;HOXB5;HOXB6;HOXB7;HOXB8;HOXB9;HOXC10;HOXC11;HOXC12;HOXC13;HOXC4;HOXC5;HOXC6;HOXC8;HOXC9;HOXD1;HOXD10;HOXD11;HOXD12;HOXD13;HOXD3;HOXD4;HOXD8;HOXD9;MEOX1;MEOX2;MNX1;PDX1;POU1F1;POU2F1;POU2F2;POU2F3;POU3F1;POU3F2;POU3F3;POU3F4;POU4F1;POU4F2;POU4F3;POU5F1;POU5F2;POU6F1;POU6F2	HOXB4;HOXD4
SMCA5_HUMAN.H11MO.0.C	17	0.09941520	0.5899173	17.196.550	1,04E+07	0.896350980	0.96124022	Myb/SANT domain factors{3.5.1}	SMARCA-like factors{3.5.1.5}	CDC5L;MYBL1;MYBL2;MYB;SMARCA1;SMARCA5	SMARCA1;SMARCA5
NR1I3_HUMAN.H11MO.0.C	14	0.08187135	0.5892816	19.080.495	1,10E+07	0.663075142	0.84500981	Thyroid hormone receptor-related factors (NR1){2.1.2}	Vitamin D receptor (NR1I){2.1.2.4}	NR1D1;NR1D2;NR1H2;NR1H3;NR1H4;NR1I2;NR1I3;PPARA;PPARD;PPARG;RARA;RARB;RARG;RORA;RORB;RORC;THRA;THRB;VDR	NR1I2;NR1I3;VDR
RARG_HUMAN.H11MO.0.B	28	0.16374269	0.5891332	13.842.606	0.9181054	0.763068156	0.88790811	Thyroid hormone receptor-related factors (NR1){2.1.2}	Retinoic acid receptors (NR1B){2.1.2.1}	NR1D1;NR1D2;NR1H2;NR1H3;NR1H4;NR1I2;NR1I3;PPARA;PPARD;PPARG;RARA;RARB;RARG;RORA;RORB;RORC;THRA;THRB;VDR	RARA;RARB;RARG
USF2_HUMAN.H11MO.0.A	41	0.23976608	0.5883788	12.273.731	0.8578003	0.437475389	0.67458705	bHLH-ZIP factors{1.2.6}	USF factors{1.2.6.2}	MAX;MITF;MLX;MLXIP;MLXIPL;MNT;MXD1;MXD3;MXD4;MXI1;MYC;MYCL;MYCN;REPIN1;SREBF1;SREBF2;TFAP4;TFE3;TFEB;TFEC;USF1;USF2	USF1;USF2
ZN708_HUMAN.H11MO.0.C	24	0.14035088	0.5875090	14.643.196	0.9464988	0.913936693	0.97058566	More than 3 adjacent zinc finger factors{2.3.3}	unclassified{2.3.3.0}	BCL6B;BCL6;CTCFL;CTCF;FEZF1;GFI1B;GFI1;GLI1;GLI2;GLI3;GLIS1;GLIS2;GLIS3;MTF1;MYNN;MZF1;OSR2;OVOL1;OVOL2;ZNF146;PLAG1;PLAGL1;PRDM14;PRDM1;PRDM6;SCRT1;SCRT2;SNAI1;SNAI2;YY1;YY2;WT1;ZNF324;ZNF354A;ZBTB14;ZBTB18;ZBTB48;ZBTB49;ZBTB7A;ZBTB7B;ZBTB6;ZFP64;ZFP28;ZFP42;ZFP82;ZFX;ZIC1;ZIC2;ZIC3;ZIC4;ZIM3;ZKSCAN1;ZKSCAN3;ZNF121;ZNF136;ZNF140;ZNF143;ZNF148;ZNF214;ZNF232;ZNF250;ZNF257;ZNF260;ZNF263;ZNF264;ZNF274;ZNF281;ZNF282;ZNF317;ZNF320;ZNF322;ZNF329;ZNF331;ZNF333;ZN	MYNN;MZF1;OSR2;PRDM14;PRDM6;WT1;ZBTB14;ZBTB48;ZBTB49;ZFP64;ZFP28;ZIM3;ZNF121;ZNF250;ZNF257;ZNF263;ZNF274;ZNF317;ZNF320;ZNF329;ZNF331;ZNF394;ZNF449;ZNF502;ZNF528;ZNF547;ZNF549;ZNF554;ZNF586;ZNF589;ZNF667;ZNF680;ZNF708;ZNF713;ZNF768;ZNF18;ZNF85;ZSCAN16;ZSCAN22

motif	Nº of probes	% of probes	lower OR	upper OR	OR	p.value	FDR	TF family	TF subfamily	TF.family.member	TF.subfamily.member
										F350;ZNF384;ZNF394;ZNF410;ZNF436;ZNF449;ZNF490;ZNF502;ZNF524;ZNF528;ZNF547;ZNF549;ZNF554;ZNF563;ZNF582;ZNF586;ZNF589;ZNF652;ZNF667;ZNF680;ZNF708;ZNF713;ZNF768;ZNF816;ZNF18;ZNF41;ZNF76;ZNF85;ZSCAN16;ZSCAN22;ZSCAN31;ZSCAN4	
DLX2_HUMAN.H11MO.0.D	10	0.05847953	0.5866297	23.555.593	1,25E+07	0.468985080	0.69670038	NK-related factors{3.1.2}	DLX{3.1.2.5}	BARHL1;BARHL2;BARX1;BARX2;BSX;DBX1;DBX2;DLX1;DLX2;DLX3;DLX4;DLX5;DLX6;EMX1;EMX2;EN1;EN2;HHEX;HLX;HMX1;HMX2;HMX3;LBX1;LBX2;MSX1;MSX2;NANOG;NKX1-1;NKX1-2;NKX2-1;NKX2-2;NKX2-3;NKX2-4;NKX2-5;NKX2-6;NKX2-8;NKX3-1;NKX3-2;NKX6-1;NKX6-2;NKX6-3;NOTO;TLX1;TLX2;TLX3;VAX1;VAX2;VENTX	DLX1;DLX2;DLX3;DLX4;DLX5;DLX6
GMEB2_HUMAN.H11MO.0.D	20	0.11695906	0.5853639	15.779.471	0.9859978	1000000000,00000000	1,00E+08	GMEB{5.3.3}	GMEB{5.3.3.1}	GMEB1;GMEB2	GMEB2
ZBTB4_HUMAN.H11MO.0.D	30	0.17543860	0.5827059	13.362.478	0.8957441	0.628236339	0.81543808	Factors with multiple dispersed zinc fingers{2.3.4}	unclassified{2.3.4.0}	BCL11A;E4F1;MECOM;HIC1;HIC2;HINFP;IKZF1;INSM1;MAZ;PATZ1;PRDM4;REST;RREB1;SALL4;VEZF1;ZBTB17;ZBTB4;HIVEP1;HIVEP2;ZNF134;ZNF219;ZNF335;ZNF341;ZNF382;ZNF418;ZNF423;ZNF467;ZNF770;ZNF784;ZNF8	E4F1;PRDM4;REST;RREB1;ZBTB17;ZBTB4;ZNF335;ZNF341;ZNF467;ZNF770;ZNF784;ZNF8
DLX4_HUMAN.H11MO.0.D	11	0.06432749	0.5825923	21.906.289	1,19E+07	0.502128344	0.71825780	NK-related factors{3.1.2}	DLX{3.1.2.5}	BARHL1;BARHL2;BARX1;BARX2;BSX;DBX1;DBX2;DLX1;DLX2;DLX3;DLX4;DLX5;DLX6;EMX1;EMX2;EN1;EN2;HHEX;HLX;HMX1;HMX2;HMX3;LBX1;LBX2;MSX1;MSX2;NANOG;NKX1-1;NKX1-2;NKX2-1;NKX2-2;NKX2-3;NKX2-4;NKX2-5;NKX2-6;NKX2-8;NKX3-1;NKX3-2;NKX6-1;NKX6-2;NKX6-3;NOTO;TLX1;TLX2;TLX3;VAX1;VAX2;VENTX	DLX1;DLX2;DLX3;DLX4;DLX5;DLX6
ZN335_HUMAN.H11MO.1.A	17	0.09941520	0.5804208	16.919.221	1,02E+07	0.897260951	0.96124022	Factors with multiple dispersed zinc fingers{2.3.4}	unclassified{2.3.4.0}	BCL11A;E4F1;MECOM;HIC1;HIC2;HINFP;IKZF1;INSM1;MAZ;PATZ1;PRDM4;REST;RREB1;SALL4;VEZF1;ZBTB17;ZBTB4;HIVEP1;HIVEP2;ZNF134;ZNF219;ZNF335;ZNF341;ZNF382;ZNF418;ZNF423;ZNF467;ZNF770;ZNF784;ZNF8	E4F1;PRDM4;REST;RREB1;ZBTB17;ZBTB4;ZNF335;ZNF341;ZNF467;ZNF770;ZNF784;ZNF8
HTF4_HUMAN.H11MO.0.A	42	0.24561404	0.5795022	12.013.220	0.8419095	0.349317654	0.59849758	E2A-related factors{1.2.1}	HTF-4 (TCF-12, HEB){1.2.1.0.3}	TCF12;TCF4;TCF3	TCF12
PO6F2_HUMAN.H11MO.0.D	10	0.05847953	0.5792061	23.256.741	1,23E+07	0.473198089	0.69842109	POU domain factors{3.1.10}	POU6 (Brn-5-like factors){3.1.10.6}	CDX1;CDX2;CDX4;EVX1;EVX2;GBX1;GBX2;GSX1;GSX2;HDX;HMBOX1;HNF1A;HNF1B;HOXA1;HOXA10;HOXA11;HOXA13;HOXA2;HOXA3;HOXA4;HOXA5;HOXA6;HOXA7;HOXA9;HOXB1;HOXB13;HOXB2;HOXB3;HOXB4;HOXB5;HOXB6;HOXB7;HOXB8;HOXB9;HOXC10;HOXC11;HOXC12;HOXC13;HOXC4;HOXC5;HOXC6;HOXC8;HOXC9;HOXD1;HOXD10;HOXD11;HOXD12;HOXD13;HOXD3;HOXD4;HOXD8;HOXD9;MEOX1;MEOX2;MNX1;PDX1;POU1F1;POU2F1;POU2F2;POU2F3;POU3F1;POU3F2;POU3F3;POU3F4;POU4F1;POU4F2;POU4F3;POU5F1;POU5F1B;POU5F2;POU6F1;POU6F2	POU6F1;POU6F2

motif	Nº of probes	% of probes	lower OR	upper OR	OR	p.value	FDR	TF family	TF subfamily	TF.family.member	TF.subfamily.member
SNAI2_HUMAN.H11MO.0.A	33	0.19298246	0.5784953	12.853.973	0.8736912	0.575963402	0.78452952	More than 3 adjacent zinc finger factors{2.3.3}	Snail-like factors{2.3.3.2}	BCL6;BCL6B;CTCF;CTCFL;FEZF1;FEZF2;GFI1;GFI1B;GLI1;GLI2;GLI3;GLI4;GLIS1;GLIS2;GLIS3;HKR1;MTF1;MYNN;MZF1;OSR2;OVOL1;OVOL2;PLAG1;PLAGL1;PLAGL2;PRDM1;PRDM14;PRDM6;SCRT1;SCRT2;SNAI1;SNAI2;SNAI3;WT1;YY1;YY2;ZBTB12;ZBTB14;ZBTB18;ZBTB20;ZBTB26;ZBTB42;ZBTB45;ZBTB47;ZBTB48;ZBTB49;ZBTB6;ZBTB7A;ZBTB7B;ZBTB7C;ZFP14;ZFP2;ZFP28;ZFP30;ZFP37;ZFP42;ZFP64;ZFP69;ZFP69B;ZFP82;ZFP91;ZFX;ZIC1;ZIC2;ZIC3;ZIC4;ZIC5;ZIK1;ZIM3;ZKSCAN1;ZKSCAN2;ZKSCAN3;ZKSCAN4;ZNF121;ZNF124;ZNF133;ZNF136;ZNF138;ZNF14;ZNF140;ZNF143;ZNF146;ZNF148;ZNF155;ZNF157;ZNF160;ZNF169;ZNF175;ZNF177;ZNF18;ZNF180;ZNF181;ZNF2;ZNF20;ZNF212;ZNF213;ZNF214;ZNF221;ZNF222;ZNF223;ZNF224;ZNF225;ZNF226;ZNF227;ZNF229;ZNF230;ZNF232;ZNF233;ZNF234;ZNF235;ZNF24;ZNF25;ZNF250;ZNF257;ZNF26;ZNF260;ZNF263;ZNF264;ZNF268;ZNF274;ZNF276;ZNF28;ZNF280A;ZNF280B;ZNF280C;ZNF280D;ZNF281;ZNF282;ZNF283;ZNF284;ZNF285;ZNF286A;ZNF286B;ZNF3;ZNF30;ZNF300;ZNF302;ZNF317;ZNF32;ZNF320;ZNF322;ZNF324;ZNF324B;ZNF329;ZNF331;ZNF333;ZNF33A;ZNF33B;ZNF343;ZNF345;ZNF347;ZNF350;ZNF354A;ZNF354B;ZNF362;ZNF366;ZNF383;ZNF384;ZNF394;ZNF397;ZNF398;ZNF404;ZNF41;ZNF410;ZNF419;ZNF420;ZNF431;ZNF432;ZNF436;ZNF439;ZNF44;ZNF440;ZNF442;ZNF443;ZNF446;ZNF449;ZNF45;ZNF460;ZNF468;ZNF479;ZNF484;ZNF490;ZNF500;ZNF502;ZNF524;ZNF525;ZNF528;ZNF543;ZNF544;ZNF546;ZNF547;ZNF548;ZNF549;ZNF554;ZNF555;ZNF557;ZNF558;ZNF559;ZNF561;ZNF562;ZNF563;ZNF564;ZNF566;ZNF567;ZNF568;ZNF57;ZNF570;ZNF571;ZNF572;ZNF577;ZNF581;ZNF582;ZNF583;ZNF585A;ZNF586;ZNF589;ZNF595;ZNF599;ZNF600;ZNF605;ZNF607;ZNF611;ZNF613;ZNF614;ZNF615;ZNF616;ZNF619;ZNF620;ZNF621;ZNF625;ZNF627;ZNF649;ZNF652;ZNF653;ZNF665;ZNF667;ZNF669;ZNF670;ZNF672;ZNF679;ZNF680;ZNF683;ZNF689;ZNF692;ZNF701;ZNF705D;ZNF705E;ZNF705G;ZNF708;ZNF709;ZNF71;ZNF710;ZNF713;ZNF721;ZNF727;ZNF729;ZNF736;ZNF75A;ZNF75D;ZNF76;ZNF763;ZNF764;ZNF765;ZNF768;ZNF77;ZNF771;ZNF773;ZNF774;ZNF776;ZNF777;ZNF780A;ZNF780B;ZNF782;ZNF785;ZNF799;ZNF805;ZNF808;ZNF81;ZNF813;ZNF816;ZNF823;ZNF829;ZNF836;ZNF841;ZNF844;ZNF845;ZNF846;ZNF85;ZNF853;ZNF860;ZNF878;ZNF891;ZNF99;ZSCAN16;ZSCAN2;ZSCAN22;ZSCAN23;ZSCAN29;ZSCAN31;ZSCAN32;ZSCAN4;ZSCAN5A;ZSCAN5B;ZSCAN5C;ZSCAN9;ZXDA;ZXDB;ZXDC	SCRT1;SCRT2;SNAI1;SNAI2
ALX1_HUMAN.H11MO.0.B	10	0.05847953	0.5783274	23.221.437	1,23E+07	0.473735773	0.69842109	Paired-related HD factors{3.1.3}	ALX{3.1.3.1}	ALX1;ALX3;ALX4;ARGFX;ARX;CRX;DMBX1;DPRX;DRGX;DUX4;DUXA;ESX1;GSC;GSC2;HESX1;ISX;LEUTX;MIXL1;NOBOX;OTP;OTX1;OTX2;PHOX2A;PHOX2B;PITX1;PITX2;PITX3;PROP1;PRRX1;PRRX2;RAX;RAX2;RHOXF1;RHOXF2;SEBOX;SHOX;SHOX2;TPRX1;UNCX;VSX1;VSX2	ALX1;ALX3;ALX4
CREB3_HUMAN.H11MO.0.D	26	0.15204678	0.5781019	13.962.681	0.9149267	0.756677897	0.88790811	CREB-related factors{1.1.7}	CREB-3-like factors{1.1.7.2}	ATF1;ATF6;ATF6B;CREB1;CREB3;CREB3L1;CREB3L2;CREB3L3;CREB3L4;CREBL2;CREBZF;CREM	CREB3L1;CREB3L2;CREB3

motif	Nº of probes	% of probes	lower OR	upper OR	OR	p.value	FDR	TF family	TF subfamily	TF.family.member	TF.subfamily.member
HEY1_HUMAN.H11MO.0.D	42	0.24561404	0.5767299	11.955.856	0.8378832	0.348916150	0.59849758	Hairy-related factors{1.2.4}	Hairy-like factors{1.2.4.1}	BHLHE40;BHLHE41;HELT;HES1;HES2;HES3;HES4;HES5;HES6;HES7;HEY1;HEY2;HEYL	BHLHE40;BHLHE41;HES1;HES5;HES7;HEY1;HEY2
SOX10_HUMAN.H11MO.1.A	26	0.15204678	0.5765094	13.924.200	0.9124042	0.756717262	0.88790811	SOX-related factors{4.1.1}	Group E{4.1.1.5}	BBX;CIC;HBP1;SOX1;SOX10;SOX11;SOX12;SOX13;SOX14;SOX15;SOX17;SOX18;SOX2;SOX21;SOX3;SOX30;SOX4;SOX5;SOX6;SOX7;SOX8;SOX9;SRY	SOX10;SOX8;SOX9
PITX2_HUMAN.H11MO.0.D	15	0.08771930	0.5758346	17.934.878	1,05E+07	0.782572659	0.89897594	Paired-related HD factors{3.1.3}	PITX{3.1.3.19}	ALX1;ALX3;ALX4;ARGFX;ARX;CRX;DMBX1;DPRX;DRGX;DUX4;DUXA;ESX1;GSC;GSC2;HESX1;ISX;LEUTX;MIXL1;NOBOX;OTP;OTX1;OTX2;PHOX2A;PHOX2B;PITX1;PITX2;PITX3;PROP1;PRRX1;PRRX2;RAX;RAX2;RHOXF1;RHOXF2;SEBOX;SHOX;SHOX2;TPRX1;UNCX;VSX1;VSX2	PITX1;PITX2;PITX3
RXRG_HUMAN.H11MO.0.B	13	0.07602339	0.5749591	19.445.006	1,10E+07	0.652733666	0.83607212	RXR-related receptors (NR2){2.1.3}	Retinoid X receptors (NR2B){2.1.3.1}	HNF4A;HNF4G;NR2C1;NR2C2;NR2E1;NR2E3;NR2F1;NR2F2;NR2F6;RXRA;RXRB;RXRG	RXRA;RXRB;RXRG
MLX_HUMAN.H11MO.0.D	72	0.42105263	0.5748386	10.814.763	0.7901223	0.145415769	0.35705592	bHLH-ZIP factors{1.2.6}	Mondo-like factors{1.2.6.6}	MAX;MITF;MLX;MLXIP;MLXIPL;MNT;MXD1;MXD3;MXD4;MXI1;MYC;MYCL;MYCN;REPIN1;SREBF1;SREBF2;TFAP4;TFE3;TFEB;TFEC;USF1;USF2	MLXIPL;MLX
ATF6A_HUMAN.H11MO.0.B	42	0.24561404	0.5747898	11.915.707	0.8350654	0.348862181	0.59849758	CREB-related factors{1.1.7}	ATF-6 factors{1.1.7.3}	ATF1;ATF6;ATF6B;CREB1;CREB3;CREB3L1;CREB3L2;CREB3L3;CREB3L4;CREBL2;CREBZF;CREM	ATF6
LHX4_HUMAN.H11MO.0.D	9	0.05263158	0.5742852	24.911.189	1,28E+07	0.440478509	0.67516686	HD-LIM factors{3.1.5}	Lhx-3-like factors{3.1.5.4}	ISL1;ISL2;LHX1;LHX2;LHX3;LHX4;LHX5;LHX6;LHX8;LHX9;LMX1A;LMX1B	LHX3;LHX4
VSX1_HUMAN.H11MO.0.D	10	0.05847953	0.5739648	23.046.490	1,22E+07	0.476524603	0.69981042	Paired-related HD factors{3.1.3}	VSX{3.1.3.28}	ALX1;ALX3;ALX4;ARGFX;ARX;CRX;DMBX1;DPRX;DRGX;DUX4;DUXA;ESX1;GSC;GSC2;HESX1;ISX;LEUTX;MIXL1;NOBOX;OTP;OTX1;OTX2;PHOX2A;PHOX2B;PITX1;PITX2;PITX3;PROP1;PRRX1;PRRX2;RAX;RAX2;RHOXF1;RHOXF2;SEBOX;SHOX;SHOX2;TPRX1;UNCX;VSX1;VSX2	VSX1;VSX2
TBX21_HUMAN.H11MO.0.A	33	0.19298246	0.5721695	12.713.444	0.8641375	0.515613035	0.73346430	TBrain-related factors{6.5.2}	TBX21 (T-bet){6.5.2.0.3}	EOMES;TBR1;TBX21	TBX21
LMX1A_HUMAN.H11MO.0.D	15	0.08771930	0.5718370	17.809.023	1,05E+07	0.783845747	0.89897594	HD-LIM factors{3.1.5}	Lmx{3.1.5.6}	ISL1;ISL2;LHX1;LHX2;LHX3;LHX4;LHX5;LHX6;LHX8;LHX9;LMX1A;LMX1B	LMX1A;LMX1B

motif	Nº of probes	% of probes	lower OR	upper OR	OR	p.value	FDR	TF family	TF subfamily	TF.family.member	TF.subfamily.member
TYY1_HUMAN.H11MO.0.A	17	0.09941520	0.5714808	16.658.817	1,01E+07	0.898499519	0.96124022	More than 3 adjacent zinc finger factors{2.3.3}	YY1-like factors{2.3.3.9}	BCL6;BCL6B;CTCF;CTCFL;FEZF1;FEZF2;GFI1;GFI1B;GLI1;GLI2;GLI3;GLI4;GLIS1;GLIS2;GLIS3;HKR1;MTF1;MYNN;MZF1;OSR2;OVOL1;OVOL2;PLAG1;PLAGL1;PLAGL2;PRDM1;PRDM14;PRDM6;SCRT1;SCRT2;SNAI1;SNAI2;SNAI3;WT1;YY1;YY2;ZBTB12;ZBTB14;ZBTB18;ZBTB20;ZBTB26;ZBTB42;ZBTB45;ZBTB47;ZBTB48;ZBTB49;ZBTB6;ZBTB7A;ZBTB7B;ZBTB7C;ZFP14;ZFP2;ZFP28;ZFP30;ZFP37;ZFP42;ZFP64;ZFP69;ZFP69B;ZFP82;ZFP91;ZFX;ZIC1;ZIC2;ZIC3;ZIC4;ZIC5;ZIK1;ZIM3;ZKSCAN1;ZKSCAN2;ZKSCAN3;ZKSCAN4;ZNF121;ZNF124;ZNF133;ZNF136;ZNF138;ZNF14;ZNF140;ZNF143;ZNF146;ZNF148;ZNF155;ZNF157;ZNF160;ZNF169;ZNF175;ZNF177;ZNF18;ZNF180;ZNF181;ZNF2;ZNF20;ZNF212;ZNF213;ZNF214;ZNF221;ZNF222;ZNF223;ZNF224;ZNF225;ZNF226;ZNF227;ZNF229;ZNF230;ZNF232;ZNF233;ZNF234;ZNF235;ZNF24;ZNF25;ZNF250;ZNF257;ZNF26;ZNF260;ZNF263;ZNF264;ZNF268;ZNF274;ZNF276;ZNF28;ZNF280A;ZNF280B;ZNF280C;ZNF280D;ZNF281;ZNF282;ZNF283;ZNF284;ZNF285;ZNF286A;ZNF286B;ZNF3;ZNF30;ZNF300;ZNF302;ZNF317;ZNF32;ZNF320;ZNF322;ZNF324;ZNF324B;ZNF329;ZNF331;ZNF333;ZNF33A;ZNF33B;ZNF343;ZNF345;ZNF347;ZNF350;ZNF354A;ZNF354B;ZNF362;ZNF366;ZNF383;ZNF384;ZNF394;ZNF397;ZNF398;ZNF404;ZNF41;ZNF410;ZNF419;ZNF420;ZNF431;ZNF432;ZNF436;ZNF439;ZNF44;ZNF440;ZNF442;ZNF443;ZNF446;ZNF449;ZNF45;ZNF460;ZNF468;ZNF479;ZNF484;ZNF490;ZNF500;ZNF502;ZNF524;ZNF525;ZNF528;ZNF543;ZNF544;ZNF546;ZNF547;ZNF548;ZNF549;ZNF554;ZNF555;ZNF557;ZNF558;ZNF559;ZNF561;ZNF562;ZNF563;ZNF564;ZNF566;ZNF567;ZNF568;ZNF57;ZNF570;ZNF571;ZNF572;ZNF577;ZNF581;ZNF582;ZNF583;ZNF585A;ZNF586;ZNF589;ZNF595;ZNF599;ZNF600;ZNF605;ZNF607;ZNF611;ZNF613;ZNF614;ZNF615;ZNF616;ZNF619;ZNF620;ZNF621;ZNF625;ZNF627;ZNF649;ZNF652;ZNF653;ZNF665;ZNF667;ZNF669;ZNF670;ZNF672;ZNF679;ZNF680;ZNF683;ZNF689;ZNF692;ZNF701;ZNF705D;ZNF705E;ZNF705G;ZNF708;ZNF709;ZNF71;ZNF710;ZNF713;ZNF721;ZNF727;ZNF729;ZNF736;ZNF75A;ZNF75D;ZNF76;ZNF763;ZNF764;ZNF765;ZNF768;ZNF77;ZNF771;ZNF773;ZNF774;ZNF776;ZNF777;ZNF780A;ZNF780B;ZNF782;ZNF785;ZNF799;ZNF805;ZNF808;ZNF81;ZNF813;ZNF816;ZNF823;ZNF829;ZNF836;ZNF841;ZNF844;ZNF845;ZNF846;ZNF85;ZNF853;ZNF860;ZNF878;ZNF891;ZNF99;ZSCAN16;ZSCAN2;ZSCAN22;ZSCAN23;ZSCAN29;ZSCAN31;ZSCAN32;ZSCAN4;ZSCAN5A;ZSCAN5B;ZSCAN5C;ZSCAN9;ZXDA;ZXDB;ZXDC	YY1;YY2;ZFP42
MYB_HUMAN.H11MO.0.A	15	0.08771930	0.5701233	17.755.591	1,04E+07	0.890093787	0.95980743	Myb/SANT domain factors{3.5.1}	Myb-like factors{3.5.1.1}	CDC5L;DMTF1;MYB;MYBL1;MYBL2;SMARCA1;SMARCA5;SNAPC4;TRERF1;ZNF541	CDC5L;MYBL1;MYBL2;MYB
SMAD4_HUMAN.H11MO.0.B	28	0.16374269	0.5700665	13.394.318	0.8883610	0.619791912	0.81312329	SMAD factors{7.1.1}	Co-activating Smads (Co-Smad){7.1.1.2}	SMAD1;SMAD2;SMAD3;SMAD4;SMAD5;SMAD9	SMAD4

motif	Nº of probes	% of probes	lower OR	upper OR	OR	p.value	FDR	TF family	TF subfamily	TF.family.member	TF.subfamily.member
OVOL1_HUMAN.H11MO.0.C	13	0.07602339	0.5692380	19.250.705	1,09E+07	0.763091852	0.88790811	More than 3 adjacent zinc finger factors{2.3.3}	OVOL-factors{2.3.3.17}	BCL6;BCL6B;CTCF;CTCFL;FEZF1;FEZF2;GFI1;GFI1B;GLI1;GLI2;GLI3;GLI4;GLIS1;GLIS2;GLIS3;HKR1;MTF1;MYNN;MZF1;OSR2;OVOL1;OVOL2;PLAG1;PLAGL1;PLAGL2;PRDM1;PRDM14;PRDM6;SCRT1;SCRT2;SNAI1;SNAI2;SNAI3;WT1;YY1;YY2;ZBTB12;ZBTB14;ZBTB18;ZBTB20;ZBTB26;ZBTB42;ZBTB45;ZBTB47;ZBTB48;ZBTB49;ZBTB6;ZBTB7A;ZBTB7B;ZBTB7C;ZFP14;ZFP2;ZFP28;ZFP30;ZFP37;ZFP42;ZFP64;ZFP69;ZFP69B;ZFP82;ZFP91;ZFX;ZIC1;ZIC2;ZIC3;ZIC4;ZIC5;ZIK1;ZIM3;ZKSCAN1;ZKSCAN2;ZKSCAN3;ZKSCAN4;ZNF121;ZNF124;ZNF133;ZNF136;ZNF138;ZNF14;ZNF140;ZNF143;ZNF146;ZNF148;ZNF155;ZNF157;ZNF160;ZNF169;ZNF175;ZNF177;ZNF18;ZNF180;ZNF181;ZNF2;ZNF20;ZNF212;ZNF213;ZNF214;ZNF221;ZNF222;ZNF223;ZNF224;ZNF225;ZNF226;ZNF227;ZNF229;ZNF230;ZNF232;ZNF233;ZNF234;ZNF235;ZNF24;ZNF25;ZNF250;ZNF257;ZNF26;ZNF260;ZNF263;ZNF264;ZNF268;ZNF274;ZNF276;ZNF28;ZNF280A;ZNF280B;ZNF280C;ZNF280D;ZNF281;ZNF282;ZNF283;ZNF284;ZNF285;ZNF286A;ZNF286B;ZNF3;ZNF30;ZNF300;ZNF302;ZNF317;ZNF32;ZNF320;ZNF322;ZNF324;ZNF324B;ZNF329;ZNF331;ZNF333;ZNF33A;ZNF33B;ZNF343;ZNF345;ZNF347;ZNF350;ZNF354A;ZNF354B;ZNF362;ZNF366;ZNF383;ZNF384;ZNF394;ZNF397;ZNF398;ZNF404;ZNF41;ZNF410;ZNF419;ZNF420;ZNF431;ZNF432;ZNF436;ZNF439;ZNF44;ZNF440;ZNF442;ZNF443;ZNF446;ZNF449;ZNF45;ZNF460;ZNF468;ZNF479;ZNF484;ZNF490;ZNF500;ZNF502;ZNF524;ZNF525;ZNF528;ZNF543;ZNF544;ZNF546;ZNF547;ZNF548;ZNF549;ZNF554;ZNF555;ZNF557;ZNF558;ZNF559;ZNF561;ZNF562;ZNF563;ZNF564;ZNF566;ZNF567;ZNF568;ZNF57;ZNF570;ZNF571;ZNF572;ZNF577;ZNF581;ZNF582;ZNF583;ZNF585A;ZNF586;ZNF589;ZNF595;ZNF599;ZNF600;ZNF605;ZNF607;ZNF611;ZNF613;ZNF614;ZNF615;ZNF616;ZNF619;ZNF620;ZNF621;ZNF625;ZNF627;ZNF649;ZNF652;ZNF653;ZNF665;ZNF667;ZNF669;ZNF670;ZNF672;ZNF679;ZNF680;ZNF683;ZNF689;ZNF692;ZNF701;ZNF705D;ZNF705E;ZNF705G;ZNF708;ZNF709;ZNF71;ZNF710;ZNF713;ZNF721;ZNF727;ZNF729;ZNF736;ZNF75A;ZNF75D;ZNF76;ZNF763;ZNF764;ZNF765;ZNF768;ZNF77;ZNF771;ZNF773;ZNF774;ZNF776;ZNF777;ZNF780A;ZNF780B;ZNF782;ZNF785;ZNF799;ZNF805;ZNF808;ZNF81;ZNF813;ZNF816;ZNF823;ZNF829;ZNF836;ZNF841;ZNF844;ZNF845;ZNF846;ZNF85;ZNF853;ZNF860;ZNF878;ZNF891;ZNF99;ZSCAN16;ZSCAN2;ZSCAN22;ZSCAN23;ZSCAN29;ZSCAN31;ZSCAN32;ZSCAN4;ZSCAN5A;ZSCAN5B;ZSCAN5C;ZSCAN9;ZXDA;ZXDB;ZXDC	OVOL1;OVOL2
NKX22_HUMAN.H11MO.0.D	29	0.16959064	0.5688214	13.198.833	0.8802049	0.624343692	0.81312329	NK-related factors{3.1.2}	NK-2.2{3.1.2.15}	BARHL1;BARHL2;BARX1;BARX2;BSX;DBX1;DBX2;DLX1;DLX2;DLX3;DLX4;DLX5;DLX6;EMX1;EMX2;EN1;EN2;HHEX;HLX;HMX1;HMX2;HMX3;LBX1;LBX2;MSX1;MSX2;NANOG;NKX1-1;NKX1-2;NKX2-1;NKX2-2;NKX2-3;NKX2-4;NKX2-5;NKX2-6;NKX2-8;NKX3-1;NKX3-2;NKX6-1;NKX6-2;NKX6-3;NOTO;TLX1;TLX2;TLX3;VAX1;VAX2;VENTX	NKX2-2;NKX2-8
TFE2_HUMAN.H11MO.0.A	42	0.24561404	0.5682458	11.779.856	0.8255610	0.308273806	0.55145964	E2A-related factors{1.2.1}	E2A (TCF-3, ITF-1){1.2.1.0.1}	TCF12;TCF4;TCF3	TCF3

motif	Nº of probes	% of probes	lower OR	upper OR	OR	p.value	FDR	TF family	TF subfamily	TF.family.member	TF.subfamily.member
LYL1_HUMAN.H11MO.0.A	35	0.20467836	0.5674138	12.379.195	0.8482252	0.416101316	0.66283908	Tal-related factors{1.2.3}	Tal / HEN-like factors{1.2.3.1}	ATOH1;ATOH7;ATOH8;BHLHA15;BHLHA9;BHLHE22;BHLHE23;FERD3L;FIGLA;HAND1;HAND2;LYL1;MESP1;MESP2;MSC;MSGN1;NEUROD1;NEUROD2;NEUROD4;NEUROD6;NEUROG1;NEUROG2;NEUROG3;NHLH1;NHLH2;OLIG1;OLIG2;OLIG3;PTF1A;SCX;TAL1;TAL2;TCF15;TCF21;TCF23;TWIST1;TWIST2	NHLH1;LYL1;TAL1
MAFK_HUMAN.H11MO.0.A	13	0.07602339	0.5670864	19.177.779	1,09E+07	0.763531878	0.88790811	Maf-related factors{1.1.3}	Small Maf factors{1.1.3.2}	MAF;MAFA;MAFB;MAFF;MAFG;MAFK;NRL	MAFF;MAFG;MAFK
SUH_HUMAN.H11MO.0.A	34	0.19883041	0.5650491	12.437.976	0.8489079	0.464099432	0.69077348	CSL-related factors{6.1.4}	M{6.1.4.1}	RBPJ;RBPJL	RBPJ
STF1_HUMAN.H11MO.0.B	24	0.14035088	0.5646989	14.074.151	0.9097078	0.749504146	0.88476575	FTZ-F1-related receptors (NR5){2.1.5}	FTZ-F1 (SF-1) (NR5A1){2.1.5.0.1}	NR5A1;NR5A2	NR5A1
TBX3_HUMAN.H11MO.0.C	29	0.16959064	0.5639712	13.085.886	0.8726988	0.558668141	0.76916632	TBX2-related factors{6.5.4}	TBX3{6.5.4.0.2}	TBX2;TBX3;TBX4;TBX5	TBX3
THA_HUMAN.H11MO.0.C	24	0.14035088	0.5625096	14.019.566	0.9061781	0.749570398	0.88476575	Thyroid hormone receptor-related factors (NR1){2.1.2}	Thyroid hormone receptors (NR1A){2.1.2.2}	NR1D1;NR1D2;NR1H2;NR1H3;NR1H4;NR1I2;NR1I3;PPARA;PPARD;PPARG;RARA;RARB;RARG;RORA;RORB;RORC;THRA;THRB;VDR	THRA;THRB
THB_HUMAN.H11MO.0.C	29	0.16959064	0.5622472	13.046.520	0.8700307	0.558666378	0.76916632	Thyroid hormone receptor-related factors (NR1){2.1.2}	Thyroid hormone receptors (NR1A){2.1.2.2}	NR1D1;NR1D2;NR1H2;NR1H3;NR1H4;NR1I2;NR1I3;PPARA;PPARD;PPARG;RARA;RARB;RARG;RORA;RORB;RORC;THRA;THRB;VDR	THRA;THRB
SCRT2_HUMAN.H11MO.0.D	13	0.07602339	0.5619048	19.002.467	1,08E+07	0.764748211	0.88798324	More than 3 adjacent zinc finger factors{2.3}	Snail-like factors{2.3.3.2}	BCL6;BCL6B;CTCF;CTCFL;FEZF1;FEZF2;GFI1;GFI1B;GLI1;GLI2;GLI3;GLI4;GLIS1;GLIS2;GLIS3;HKR1;MTF1;MYNN;MZF1;OSR2;OVOL1;OVOL2;PLAG1;PLAGL1;PLAGL2;PRDM1;PRDM14;PRDM6;SCRT1;SCRT2;SNAI1;SNAI2;SNAI3;WT1;YY1;YY2;ZBTB12;ZBTB14;ZBTB18;ZBTB20;ZBTB26;ZBTB42;ZBTB45;ZBTB47;ZBTB48;ZBTB49;ZBTB6;ZBTB7A;ZBTB7B;ZBTB7C;ZFP14;ZFP2;ZFP28;ZFP30;ZFP37;ZFP42;ZFP64;ZFP69;ZFP69B;ZFP82;ZFP91;ZFX;ZIC1;ZIC2;ZIC3;ZIC4;ZIC5;ZIK1;ZIM3;ZKSCAN1;ZKSCAN2;ZKSCAN3;ZKSCAN4;ZNF121;ZNF124;ZNF133;ZNF136;ZNF138;ZNF14;ZNF140;ZNF143;ZNF146;ZNF148;ZNF155;ZNF157;ZNF160;ZNF169;ZNF175;ZNF177;ZNF18;ZNF180;ZNF181;ZNF2;ZNF20;ZNF212;ZNF213;ZNF214;ZNF221;ZNF222;ZNF223;ZNF224;ZNF225;ZNF226;ZNF227;ZNF229;ZNF230;ZNF232;ZNF233;ZNF234;ZNF235;ZNF24;ZNF25;ZNF250;ZNF257;ZNF26;ZNF260;ZNF263;ZNF264;ZNF268;ZNF274;ZNF276;ZNF28;ZNF280A;ZNF280B;ZNF280C;ZNF280D;ZNF281;ZNF282;ZNF283;ZNF284;ZNF285;ZNF286A;ZNF286B;ZNF3;ZNF30;ZNF300;ZNF302;ZNF317;ZNF32;ZNF320;ZNF322;ZNF324;ZNF324B;ZNF329;ZNF331;ZNF333;ZNF33A;ZNF33B;ZNF343;ZNF345;ZNF347;ZNF350;ZNF354A;ZNF354B;ZNF362;ZNF366;ZNF383;ZNF384;ZNF394;ZNF397;ZNF398;ZNF404;ZNF41;ZNF410;ZNF419;ZNF420;ZNF431;ZNF432;ZNF436;ZNF439;ZNF44;ZNF440;ZNF442;ZNF443;ZNF446;ZNF449;ZNF45;ZNF460;ZNF468;ZNF479;ZNF484;ZNF490;ZNF500;ZNF502;ZNF524;ZNF525;ZNF528;ZNF543;ZNF544;ZNF546;ZNF547;ZNF548;ZNF549;ZNF554;ZNF555;ZNF557;ZNF558;ZNF559;ZNF561;ZNF562;ZNF563;ZNF564;ZNF566;ZNF567;ZNF568;ZNF57;ZNF570;ZNF571;ZNF572;ZNF577;ZNF581;ZNF582;ZNF583;ZNF585A;ZNF586;ZNF589;ZNF59	SCRT1;SCRT2;SNAI1;SNAI2

motif	Nº of probes	% of probes	lower OR	upper OR	OR	p.value	FDR	TF family	TF subfamily	TF.family.member	TF.subfamily.member
										5;ZNF599;ZNF600;ZNF605;ZNF607;ZNF611;ZNF613;ZNF614;ZNF615;ZNF616;ZNF619;ZNF620;ZNF621;ZNF625;ZNF627;ZNF649;ZNF652;ZNF653;ZNF665;ZNF667;ZNF669;ZNF670;ZNF672;ZNF679;ZNF680;ZNF683;ZNF689;ZNF692;ZNF701;ZNF705D;ZNF705E;ZNF705G;ZNF708;ZNF709;ZNF71;ZNF710;ZNF713;ZNF721;ZNF727;ZNF729;ZNF736;ZNF75A;ZNF75D;ZNF76;ZNF763;ZNF764;ZNF765;ZNF768;ZNF77;ZNF771;ZNF773;ZNF774;ZNF776;ZNF777;ZNF780A;ZNF780B;ZNF782;ZNF785;ZNF799;ZNF805;ZNF808;ZNF81;ZNF813;ZNF816;ZNF823;ZNF829;ZNF836;ZNF841;ZNF844;ZNF845;ZNF846;ZNF85;ZNF853;ZNF860;ZNF878;ZNF891;ZNF99;ZSCAN16;ZSCAN2;ZSCAN22;ZSCAN23;ZSCAN29;ZSCAN31;ZSCAN32;ZSCAN4;ZSCAN5A;ZSCAN5B;ZSCAN5C;ZSCAN9;ZXDA;ZXDB;ZXDC	
ATF2_HUMAN.H11MO.1.B	12	0.07017544	0.5592564	19.870.028	1,11E+07	0.752990472	0.88634451	Jun-related factors{1.1.1}	ATF-2-like factors{1.1.1.3}	ATF2;ATF7;BACH1;BACH2;CREB5;JUN;JUNB;JUND;NFE2;NFE2L1;NFE2L2;NFE2L3	ATF2;ATF7;CREB5
ZN436_HUMAN.H11MO.0.C	35	0.20467836	0.5581367	12.176.772	0.8343564	0.368156828	0.61840722	More than 3 adjacent zinc finger factors{2.3.3}	ZNF180-like factors{2.3.3.58}	BCL6;BCL6B;CTCF;CTCFL;FEZF1;FEZF2;GFI1;GFI1B;GLI1;GLI2;GLI3;GLI4;GLIS1;GLIS2;GLIS3;HKR1;MTF1;MYNN;MZF1;OSR2;OVOL1;OVOL2;PLAG1;PLAGL1;PLAGL2;PRDM1;PRDM14;PRDM6;SCRT1;SCRT2;SNAI1;SNAI2;SNAI3;WT1;YY1;YY2;ZBTB12;ZBTB14;ZBTB18;ZBTB20;ZBTB26;ZBTB42;ZBTB45;ZBTB47;ZBTB48;ZBTB49;ZBTB6;ZBTB7A;ZBTB7B;ZBTB7C;ZFP14;ZFP2;ZFP28;ZFP30;ZFP37;ZFP42;ZFP64;ZFP69;ZFP69B;ZFP82;ZFP91;ZFX;ZIC1;ZIC2;ZIC3;ZIC4;ZIC5;ZIK1;ZIM3;ZKSCAN1;ZKSCAN2;ZKSCAN3;ZKSCAN4;ZNF121;ZNF124;ZNF133;ZNF136;ZNF138;ZNF14;ZNF140;ZNF143;ZNF146;ZNF148;ZNF155;ZNF157;ZNF160;ZNF169;ZNF175;ZNF177;ZNF18;ZNF180;ZNF181;ZNF2;ZNF20;ZNF212;ZNF213;ZNF214;ZNF221;ZNF222;ZNF223;ZNF224;ZNF225;ZNF226;ZNF227;ZNF229;ZNF230;ZNF232;ZNF233;ZNF234;ZNF235;ZNF24;ZNF25;ZNF250;ZNF257;ZNF26;ZNF260;ZNF263;ZNF264;ZNF268;ZNF274;ZNF276;ZNF28;ZNF280A;ZNF280B;ZNF280C;ZNF280D;ZNF281;ZNF282;ZNF283;ZNF284;ZNF285;ZNF286A;ZNF286B;ZNF3;ZNF30;ZNF300;ZNF302;ZNF317;ZNF32;ZNF320;ZNF322;ZNF324;ZNF324B;ZNF329;ZNF331;ZNF333;ZNF33A;ZNF33B;ZNF343;ZNF345;ZNF347;ZNF350;ZNF354A;ZNF354B;ZNF362;ZNF366;ZNF383;ZNF384;ZNF394;ZNF397;ZNF398;ZNF404;ZNF41;ZNF410;ZNF419;ZNF420;ZNF431;ZNF432;ZNF436;ZNF439;ZNF44;ZNF440;ZNF442;ZNF443;ZNF446;ZNF449;ZNF45;ZNF460;ZNF468;ZNF479;ZNF484;ZNF490;ZNF500;ZNF502;ZNF524;ZNF525;ZNF528;ZNF543;ZNF544;ZNF546;ZNF547;ZNF548;ZNF549;ZNF554;ZNF555;ZNF557;ZNF558;ZNF559;ZNF561;ZNF562;ZNF563;ZNF564;ZNF566;ZNF567;ZNF568;ZNF57;ZNF570;ZNF571;ZNF572;ZNF577;ZNF581;ZNF582;ZNF583;ZNF585A;ZNF586;ZNF589;ZNF59	ZNF436

motif	Nº of probes	% of probes	lower OR	upper OR	OR	p.value	FDR	TF family	TF subfamily	TF.family.member	TF.subfamily.member
										5;ZNF599;ZNF600;ZNF605;ZNF607;ZNF611;ZNF613;ZNF614;ZNF615;ZNF616;ZNF619;ZNF620;ZNF621;ZNF625;ZNF627;ZNF649;ZNF652;ZNF653;ZNF665;ZNF667;ZNF669;ZNF670;ZNF672;ZNF679;ZNF680;ZNF683;ZNF689;ZNF692;ZNF701;ZNF705D;ZNF705E;ZNF705G;ZNF708;ZNF709;ZNF71;ZNF710;ZNF713;ZNF721;ZNF727;ZNF729;ZNF736;ZNF75A;ZNF75D;ZNF76;ZNF763;ZNF764;ZNF765;ZNF768;ZNF77;ZNF771;ZNF773;ZNF774;ZNF776;ZNF777;ZNF780A;ZNF780B;ZNF782;ZNF785;ZNF799;ZNF805;ZNF808;ZNF81;ZNF813;ZNF816;ZNF823;ZNF829;ZNF836;ZNF841;ZNF844;ZNF845;ZNF846;ZNF85;ZNF853;ZNF860;ZNF878;ZNF891;ZNF99;ZSCAN16;ZSCAN2;ZSCAN22;ZSCAN23;ZSCAN29;ZSCAN31;ZSCAN32;ZSCAN4;ZSCAN5A;ZSCAN5B;ZSCAN5C;ZSCAN9;ZXDA;ZXDB;ZXDC	
ASCL1_HUMAN.H11MO.0.A	41	0.23976608	0.5580383	11.640.871	0.8135759	0.268216702	0.49710355	MyoD / ASC-related factors {1.2.2}	Achaete-Scute-like factors{1.2.2.2}	ASCL1;ASCL2;ASCL3;ASCL4;ASCL5;MYF5;MYF6;MYOD1;MYOG	ASCL1;ASCL2
KAISO_HUMAN.H11MO.2.A	19	0.11111111	0.5524164	15.248.090	0.9431370	0.905408447	0.96551855	Other factors with up to three adjacent zinc fingers{2.3.2}	Factors with 2-3 adjacent zinc fingersand a BTB/POZ domain{2.3.2.1}	AEBP2;OSR1;OVOL3;ZBTB22;ZBTB32;ZBTB33;ZBTB34;ZBTB37;ZBTB43;ZBTB46;ZBTB5;ZBTB8B;ZFPM2;ZNF174;ZNF396;ZNF414;ZNF446;ZNF487;ZNF511;ZNF580;ZNF705A;ZNF740;ZSCAN1	ZBTB33
ZN418_HUMAN.H11MO.1.D	13	0.07602339	0.5516030	18.653.782	1,06E+07	0.767835730	0.88901808	Factors with multiple dispersed zinc fingers {2.3.4}	ZNF417-like factors{2.3.4.1}	BCL11A;BCL11B;BNC1;BNC2;E4F1;HIC1;HIC2;HINFP;HIVEP1;HIVEP2;HIVEP3;IKZF1;IKZF2;IKZF3;IKZF4;IKZF5;INSM1;INSM2;MAZ;MECOM;PATZ1;PRDM16;PRDM4;REST;RLF;RREB1;SALL1;SALL2;SALL3;SALL4;VEZF1;ZBTB1;ZBTB17;ZBTB2;ZBTB25;ZBTB4;ZFAT;ZNF134;ZNF211;ZNF217;ZNF219;ZNF248;ZNF256;ZNF292;ZNF296;ZNF319;ZNF334;ZNF335;ZNF341;ZNF37A;ZNF382;ZNF417;ZNF418;ZNF423;ZNF467;ZNF510;ZNF512;ZNF512B;ZNF516;ZNF518A;ZNF518B;ZNF521;ZNF526;ZNF532;ZNF536;ZNF552;ZNF574;ZNF587;ZNF587B;ZNF592;ZNF639;ZNF654;ZNF658;ZNF671;ZNF687;ZNF711;ZNF717;ZNF770;ZNF772;ZNF784;ZNF786;ZNF792;ZNF8;ZNF814	ZNF418

motif	Nº of probes	% of probes	lower OR	upper OR	OR	p.value	FDR	TF family	TF subfamily	TF.family.member	TF.subfamily.member
ZSCA4_HUMAN.H11MO.0.D	25	0.14619883	0.5514566	13.521.913	0.8802886	0.605543654	0.80150904	More than 3 adjacent zinc finger factors{2.3.3}	ZSCAN5-like zinc finger factors{2.3.3.5}	BCL6;BCL6B;CTCF;CTCFL;FEZF1;FEZF2;GFI1;GFI1B;GLI1;GLI2;GLI3;GLI4;GLIS1;GLIS2;GLIS3;HKR1;MTF1;MYNN;MZF1;OSR2;OVOL1;OVOL2;PLAG1;PLAGL1;PLAGL2;PRDM1;PRDM14;PRDM6;SCRT1;SCRT2;SNAI1;SNAI2;SNAI3;WT1;YY1;YY2;ZBTB12;ZBTB14;ZBTB18;ZBTB20;ZBTB26;ZBTB42;ZBTB45;ZBTB47;ZBTB48;ZBTB49;ZBTB6;ZBTB7A;ZBTB7B;ZBTB7C;ZFP14;ZFP2;ZFP28;ZFP30;ZFP37;ZFP42;ZFP64;ZFP69;ZFP69B;ZFP82;ZFP91;ZFX;ZIC1;ZIC2;ZIC3;ZIC4;ZIC5;ZIK1;ZIM3;ZKSCAN1;ZKSCAN2;ZKSCAN3;ZKSCAN4;ZNF121;ZNF124;ZNF133;ZNF136;ZNF138;ZNF14;ZNF140;ZNF143;ZNF146;ZNF148;ZNF155;ZNF157;ZNF160;ZNF169;ZNF175;ZNF177;ZNF18;ZNF180;ZNF181;ZNF2;ZNF20;ZNF212;ZNF213;ZNF214;ZNF221;ZNF222;ZNF223;ZNF224;ZNF225;ZNF226;ZNF227;ZNF229;ZNF230;ZNF232;ZNF233;ZNF234;ZNF235;ZNF24;ZNF25;ZNF250;ZNF257;ZNF26;ZNF260;ZNF263;ZNF264;ZNF268;ZNF274;ZNF276;ZNF28;ZNF280A;ZNF280B;ZNF280C;ZNF280D;ZNF281;ZNF282;ZNF283;ZNF284;ZNF285;ZNF286A;ZNF286B;ZNF3;ZNF30;ZNF300;ZNF302;ZNF317;ZNF32;ZNF320;ZNF322;ZNF324;ZNF324B;ZNF329;ZNF331;ZNF333;ZNF33A;ZNF33B;ZNF343;ZNF345;ZNF347;ZNF350;ZNF354A;ZNF354B;ZNF362;ZNF366;ZNF383;ZNF384;ZNF394;ZNF397;ZNF398;ZNF404;ZNF41;ZNF410;ZNF419;ZNF420;ZNF431;ZNF432;ZNF436;ZNF439;ZNF44;ZNF440;ZNF442;ZNF443;ZNF446;ZNF449;ZNF45;ZNF460;ZNF468;ZNF479;ZNF484;ZNF490;ZNF500;ZNF502;ZNF524;ZNF525;ZNF528;ZNF543;ZNF544;ZNF546;ZNF547;ZNF548;ZNF549;ZNF554;ZNF555;ZNF557;ZNF558;ZNF559;ZNF561;ZNF562;ZNF563;ZNF564;ZNF566;ZNF567;ZNF568;ZNF57;ZNF570;ZNF571;ZNF572;ZNF577;ZNF581;ZNF582;ZNF583;ZNF585A;ZNF586;ZNF589;ZNF595;ZNF599;ZNF600;ZNF605;ZNF607;ZNF611;ZNF613;ZNF614;ZNF615;ZNF616;ZNF619;ZNF620;ZNF621;ZNF625;ZNF627;ZNF649;ZNF652;ZNF653;ZNF665;ZNF667;ZNF669;ZNF670;ZNF672;ZNF679;ZNF680;ZNF683;ZNF689;ZNF692;ZNF701;ZNF705D;ZNF705E;ZNF705G;ZNF708;ZNF709;ZNF71;ZNF710;ZNF713;ZNF721;ZNF727;ZNF729;ZNF736;ZNF75A;ZNF75D;ZNF76;ZNF763;ZNF764;ZNF765;ZNF768;ZNF77;ZNF771;ZNF773;ZNF774;ZNF776;ZNF777;ZNF780A;ZNF780B;ZNF782;ZNF785;ZNF799;ZNF805;ZNF808;ZNF81;ZNF813;ZNF816;ZNF823;ZNF829;ZNF836;ZNF841;ZNF844;ZNF845;ZNF846;ZNF85;ZNF853;ZNF860;ZNF878;ZNF891;ZNF99;ZSCAN16;ZSCAN2;ZSCAN22;ZSCAN23;ZSCAN29;ZSCAN31;ZSCAN32;ZSCAN4;ZSCAN5A;ZSCAN5B;ZSCAN5C;ZSCAN9;ZXDA;ZXDB;ZXDC	ZSCAN4

motif	Nº of probes	% of probes	lower OR	upper OR	OR	p.value	FDR	TF family	TF subfamily	TF.family.member	TF.subfamily.member
ZIC2_HUMAN.H11MO.0.D	40	0.23391813	0.5513155	11.576.374	0.8067308	0.264952126	0.49582060	More than 3 adjacent zinc finger factors{2.3.3}	GLI-like factors{2.3.3.1}	BCL6;BCL6B;CTCF;CTCFL;FEZF1;FEZF2;GFI1;GFI1B;GLI1;GLI2;GLI3;GLI4;GLIS1;GLIS2;GLIS3;HKR1;MTF1;MYNN;MZF1;OSR2;OVOL1;OVOL2;PLAG1;PLAGL1;PLAGL2;PRDM1;PRDM14;PRDM6;SCRT1;SCRT2;SNAI1;SNAI2;SNAI3;WT1;YY1;YY2;ZBTB12;ZBTB14;ZBTB18;ZBTB20;ZBTB26;ZBTB42;ZBTB45;ZBTB47;ZBTB48;ZBTB49;ZBTB6;ZBTB7A;ZBTB7B;ZBTB7C;ZFP14;ZFP2;ZFP28;ZFP30;ZFP37;ZFP42;ZFP64;ZFP69;ZFP69B;ZFP82;ZFP91;ZFX;ZIC1;ZIC2;ZIC3;ZIC4;ZIC5;ZIK1;ZIM3;ZKSCAN1;ZKSCAN2;ZKSCAN3;ZKSCAN4;ZNF121;ZNF124;ZNF133;ZNF136;ZNF138;ZNF14;ZNF140;ZNF143;ZNF146;ZNF148;ZNF155;ZNF157;ZNF160;ZNF169;ZNF175;ZNF177;ZNF18;ZNF180;ZNF181;ZNF2;ZNF20;ZNF212;ZNF213;ZNF214;ZNF221;ZNF222;ZNF223;ZNF224;ZNF225;ZNF226;ZNF227;ZNF229;ZNF230;ZNF232;ZNF233;ZNF234;ZNF235;ZNF24;ZNF25;ZNF250;ZNF257;ZNF26;ZNF260;ZNF263;ZNF264;ZNF268;ZNF274;ZNF276;ZNF28;ZNF280A;ZNF280B;ZNF280C;ZNF280D;ZNF281;ZNF282;ZNF283;ZNF284;ZNF285;ZNF286A;ZNF286B;ZNF3;ZNF30;ZNF300;ZNF302;ZNF317;ZNF32;ZNF320;ZNF322;ZNF324;ZNF324B;ZNF329;ZNF331;ZNF333;ZNF33A;ZNF33B;ZNF343;ZNF345;ZNF347;ZNF350;ZNF354A;ZNF354B;ZNF362;ZNF366;ZNF383;ZNF384;ZNF394;ZNF397;ZNF398;ZNF404;ZNF41;ZNF410;ZNF419;ZNF420;ZNF431;ZNF432;ZNF436;ZNF439;ZNF44;ZNF440;ZNF442;ZNF443;ZNF446;ZNF449;ZNF45;ZNF460;ZNF468;ZNF479;ZNF484;ZNF490;ZNF500;ZNF502;ZNF524;ZNF525;ZNF528;ZNF543;ZNF544;ZNF546;ZNF547;ZNF548;ZNF549;ZNF554;ZNF555;ZNF557;ZNF558;ZNF559;ZNF561;ZNF562;ZNF563;ZNF564;ZNF566;ZNF567;ZNF568;ZNF57;ZNF570;ZNF571;ZNF572;ZNF577;ZNF581;ZNF582;ZNF583;ZNF585A;ZNF586;ZNF589;ZNF595;ZNF599;ZNF600;ZNF605;ZNF607;ZNF611;ZNF613;ZNF614;ZNF615;ZNF616;ZNF619;ZNF620;ZNF621;ZNF625;ZNF627;ZNF649;ZNF652;ZNF653;ZNF665;ZNF667;ZNF669;ZNF670;ZNF672;ZNF679;ZNF680;ZNF683;ZNF689;ZNF692;ZNF701;ZNF705D;ZNF705E;ZNF705G;ZNF708;ZNF709;ZNF71;ZNF710;ZNF713;ZNF721;ZNF727;ZNF729;ZNF736;ZNF75A;ZNF75D;ZNF76;ZNF763;ZNF764;ZNF765;ZNF768;ZNF77;ZNF771;ZNF773;ZNF774;ZNF776;ZNF777;ZNF780A;ZNF780B;ZNF782;ZNF785;ZNF799;ZNF805;ZNF808;ZNF81;ZNF813;ZNF816;ZNF823;ZNF829;ZNF836;ZNF841;ZNF844;ZNF845;ZNF846;ZNF85;ZNF853;ZNF860;ZNF878;ZNF891;ZNF99;ZSCAN16;ZSCAN2;ZSCAN22;ZSCAN23;ZSCAN29;ZSCAN31;ZSCAN32;ZSCAN4;ZSCAN5A;ZSCAN5B;ZSCAN5C;ZSCAN9;ZXDA;ZXDB;ZXDC	GLI1;GLI2;GLI3;GLIS1;GLIS2;GLIS3;ZIC1;ZIC2;ZIC3;ZIC4
RFX3_HUMAN.H11MO.0.B	13	0.07602339	0.5512820	18.642.999	1,06E+07	0.767945576	0.88901808	RFX-related factors{3.3.3}	RFX3{3.3.3.0.3}	RFX1;RFX2;RFX3;RFX4;RFX5;RFX6;RFX7;RFX8	RFX3

motif	Nº of probes	% of probes	lower OR	upper OR	OR	p.value	FDR	TF family	TF subfamily	TF.family.member	TF.subfamily.member
ZN274_HUMAN.H11MO.0.A	15	0.08771930	0.5511177	17.163.699	1,01E+07	0.892505830	0.96106424	More than 3 adjacent zinc finger factors{2.3.3}	unclassified{2.3.3.0}	BCL6;BCL6B;CTCF;CTCFL;FEZF1;FEZF2;GFI1;GFI1B;GLI1;GLI2;GLI3;GLI4;GLIS1;GLIS2;GLIS3;HKR1;MTF1;MYNN;MZF1;OSR2;OVOL1;OVOL2;PLAG1;PLAGL1;PLAGL2;PRDM1;PRDM14;PRDM6;SCRT1;SCRT2;SNAI1;SNAI2;SNAI3;WT1;YY1;YY2;ZBTB12;ZBTB14;ZBTB18;ZBTB20;ZBTB26;ZBTB42;ZBTB45;ZBTB47;ZBTB48;ZBTB49;ZBTB6;ZBTB7A;ZBTB7B;ZBTB7C;ZFP14;ZFP2;ZFP28;ZFP30;ZFP37;ZFP42;ZFP64;ZFP69;ZFP69B;ZFP82;ZFP91;ZFX;ZIC1;ZIC2;ZIC3;ZIC4;ZIC5;ZIK1;ZIM3;ZKSCAN1;ZKSCAN2;ZKSCAN3;ZKSCAN4;ZNF121;ZNF124;ZNF133;ZNF136;ZNF138;ZNF14;ZNF140;ZNF143;ZNF146;ZNF148;ZNF155;ZNF157;ZNF160;ZNF169;ZNF175;ZNF177;ZNF18;ZNF180;ZNF181;ZNF2;ZNF20;ZNF212;ZNF213;ZNF214;ZNF221;ZNF222;ZNF223;ZNF224;ZNF225;ZNF226;ZNF227;ZNF229;ZNF230;ZNF232;ZNF233;ZNF234;ZNF235;ZNF24;ZNF25;ZNF250;ZNF257;ZNF26;ZNF260;ZNF263;ZNF264;ZNF268;ZNF274;ZNF276;ZNF28;ZNF280A;ZNF280B;ZNF280C;ZNF280D;ZNF281;ZNF282;ZNF283;ZNF284;ZNF285;ZNF286A;ZNF286B;ZNF3;ZNF30;ZNF300;ZNF302;ZNF317;ZNF32;ZNF320;ZNF322;ZNF324;ZNF324B;ZNF329;ZNF331;ZNF333;ZNF33A;ZNF33B;ZNF343;ZNF345;ZNF347;ZNF350;ZNF354A;ZNF354B;ZNF362;ZNF366;ZNF383;ZNF384;ZNF394;ZNF397;ZNF398;ZNF404;ZNF41;ZNF410;ZNF419;ZNF420;ZNF431;ZNF432;ZNF436;ZNF439;ZNF44;ZNF440;ZNF442;ZNF443;ZNF446;ZNF449;ZNF45;ZNF460;ZNF468;ZNF479;ZNF484;ZNF490;ZNF500;ZNF502;ZNF524;ZNF525;ZNF528;ZNF543;ZNF544;ZNF546;ZNF547;ZNF548;ZNF549;ZNF554;ZNF555;ZNF557;ZNF558;ZNF559;ZNF561;ZNF562;ZNF563;ZNF564;ZNF566;ZNF567;ZNF568;ZNF57;ZNF570;ZNF571;ZNF572;ZNF577;ZNF581;ZNF582;ZNF583;ZNF585A;ZNF586;ZNF589;ZNF595;ZNF599;ZNF600;ZNF605;ZNF607;ZNF611;ZNF613;ZNF614;ZNF615;ZNF616;ZNF619;ZNF620;ZNF621;ZNF625;ZNF627;ZNF649;ZNF652;ZNF653;ZNF665;ZNF667;ZNF669;ZNF670;ZNF672;ZNF679;ZNF680;ZNF683;ZNF689;ZNF692;ZNF701;ZNF705D;ZNF705E;ZNF705G;ZNF708;ZNF709;ZNF71;ZNF710;ZNF713;ZNF721;ZNF727;ZNF729;ZNF736;ZNF75A;ZNF75D;ZNF76;ZNF763;ZNF764;ZNF765;ZNF768;ZNF77;ZNF771;ZNF773;ZNF774;ZNF776;ZNF777;ZNF780A;ZNF780B;ZNF782;ZNF785;ZNF799;ZNF805;ZNF808;ZNF81;ZNF813;ZNF816;ZNF823;ZNF829;ZNF836;ZNF841;ZNF844;ZNF845;ZNF846;ZNF85;ZNF853;ZNF860;ZNF878;ZNF891;ZNF99;ZSCAN16;ZSCAN2;ZSCAN22;ZSCAN23;ZSCAN29;ZSCAN31;ZSCAN32;ZSCAN4;ZSCAN5A;ZSCAN5B;ZSCAN5C;ZSCAN9;ZXDA;ZXDB;ZXDC	MYNN;MZF1;OSR2;PRDM14;PRDM6;WT1;ZBTB14;ZBTB48;ZBTB49;ZFP64;ZFP28;ZIM3;ZNF121;ZNF250;ZNF257;ZNF263;ZNF274;ZNF317;ZNF320;ZNF329;ZNF331;ZNF394;ZNF449;ZNF502;ZNF528;ZNF547;ZNF549;ZNF554;ZNF586;ZNF589;ZNF667;ZNF680;ZNF708;ZNF713;ZNF768;ZNF18;ZNF85;ZSCAN16;ZSCAN22
MYOD1_HUMAN.H11MO.1.A	34	0.19883041	0.5510909	12.130.638	0.8279354	0.363851990	0.61519711	MyoD / ASC-related factors{1.2.2}	Myogenic transcription factors{1.2.2.1}	ASCL1;ASCL2;ASCL3;ASCL4;ASCL5;MYF5;MYF6;MYOD1;MYOG	MYF6;MYOD1;MYOG
CDC5L_HUMAN.H11MO.0.D	11	0.06432749	0.5500646	20.682.264	1,12E+07	0.623586224	0.81312329	Myb/SANT domain factors{3.5.1}	Myb-like factors{3.5.1.1}	CDC5L;DMTF1;MYB;MYBL1;MYBL2;SMARCA1;SMARCA5;SNAPC4;TRERF1;ZNF541	CDC5L;MYBL1;MYBL2;MYB
LHX6_HUMAN.H11MO.0.D	9	0.05263158	0.5475555	23.749.417	1,22E+07	0.570157278	0.78158992	HD-LIM factors{3.1.5}	Lhx-6-like factors{3.1.5.5}	ISL1;ISL2;LHX1;LHX2;LHX3;LHX4;LHX5;LHX6;LHX8;LHX9;LMX1A;LMX1B	LHX6;LHX8
MAFA_HUMAN.H11MO.0.D	33	0.19298246	0.5472389	12.159.574	0.8264814	0.359519627	0.60920798	Maf-related factors{1.1.3}	Large Maf factors{1.1.3.1}	MAF;MAFA;MAFB;MAFF;MAFG;MAFK;NRL	MAFA;MAFB;MAF;NRL

motif	Nº of probes	% of probes	lower OR	upper OR	OR	p.value	FDR	TF family	TF subfamily	TF.family.member	TF.subfamily.member
MAX_HUMAN.H11MO.0.A	41	0.23976608	0.5469029	11.408.819	0.7973400	0.234387434	0.46695791	bHLH-ZIP factors{1.2.6}	Myc / Max factors{1.2.6.5}	MAX;MITF;MLX;MLXIP;MLXIPL;MNT;MXD1;MXD3;MXD4;MXI1;MYC;MYCL;MYCN;REPIN1;SREBF1;SREBF2;TFAP4;TFE3;TFEB;TFEC;USF1;USF2	MAX;MYCN;MYC
NFKB2_HUMAN.H11MO.0.B	21	0.12280702	0.5460230	14.399.639	0.9082834	0.821695174	0.93028925	NF-kappaB-related factors{6.1.1}	NF-kappaB p50 subunit-like factors{6.1.1.1}	NFKB1;NFKB2;REL;RELA;RELB	NFKB1;NFKB2
ESR1_HUMAN.H11MO.0.A	26	0.15204678	0.5453348	13.170.861	0.8630862	0.543650431	0.75638870	Steroid hormone receptors (NR3){2.1.1}	ER-like receptors (NR3A&B){2.1.1.2}	AR;ESR1;ESR2;ESRRA;ESRRB;ESRRG;NR3C1;NR3C2;PGR	ESRRA;ESRRB;ESRRG;ESR1;ESR2
NR0B1_HUMAN.H11MO.0.D	44	0.25730994	0.5451109	11.168.967	0.7868051	0.184133510	0.41432007	DAX-related receptors (NR0){2.1.7}	DAX1 (NR0B1){2.1.7.0.1}	NR0B1	NR0B1
SMAD2_HUMAN.H11MO.0.A	30	0.17543860	0.5448098	12.493.585	0.8374827	0.445950453	0.67623208	SMAD factors{7.1.1}	Regulatory Smads (R-Smad){7.1.1.1}	SMAD1;SMAD2;SMAD3;SMAD4	SMAD1;SMAD2;SMAD3
MYCN_HUMAN.H11MO.0.A	42	0.24561404	0.5424277	11.244.299	0.7880522	0.206721024	0.44012438	bHLH-ZIP factors{1.2.6}	Myc / Max factors{1.2.6.5}	MAX;MITF;MLX;MLXIP;MLXIPL;MNT;MXD1;MXD3;MXD4;MXI1;MYC;MYCL;MYCN;REPIN1;SREBF1;SREBF2;TFAP4;TFE3;TFEB;TFEC;USF1;USF2	MAX;MYCN;MYC
RORA_HUMAN.H11MO.0.C	21	0.12280702	0.5386816	14.204.539	0.8959988	0.736972780	0.88009011	Thyroid hormone receptor-related factors (NR1){2.1.2}	ROR (NR1F){2.1.2.6}	NR1D1;NR1D2;NR1H2;NR1H3;NR1H4;NR1I2;NR1I3;PPARA;PPARD;PPARG;RARA;RARB;RARG;RORA;RORB;RORC;THRA;THRB;VDR	RORA;RORC
ZN667_HUMAN.H11MO.0.C	22	0.12865497	0.5381721	13.906.655	0.8849549	0.662556573	0.84500981	More than 3 adjacent zinc finger factors{2.3.3}	unclassified{2.3.3.0}	BCL6B;BCL6;CTCFL;CTCF;FEZF1;GFI1B;GFI1;GLI1;GLI2;GLI3;GLIS1;GLIS2;GLIS3;MTF1;MYNN;MZF1;OSR2;OVOL1;OVOL2;ZNF146;PLAG1;PLAGL1;PRDM14;PRDM1;PRDM6;SCRT1;SCRT2;SNAI1;SNAI2;YY1;YY2;WT1;ZNF324;ZNF354A;ZBTB14;ZBTB18;ZBTB48;ZBTB49;ZBTB7A;ZBTB7B;ZBTB6;ZFP64;ZFP28;ZFP42;ZFP82;ZFX;ZIC1;ZIC2;ZIC3;ZIC4;ZIM3;ZKSCAN1;ZKSCAN3;ZNF121;ZNF136;ZNF140;ZNF143;ZNF148;ZNF214;ZNF232;ZNF250;ZNF257;ZNF260;ZNF263;ZNF264;ZNF274;ZNF281;ZNF282;ZNF317;ZNF320;ZNF322;ZNF329;ZNF331;ZNF333;ZNF350;ZNF384;ZNF394;ZNF410;ZNF436;ZNF449;ZNF490;ZNF502;ZNF524;ZNF528;ZNF547;ZNF549;ZNF554;ZNF563;ZNF582;ZNF586;ZNF589;ZNF652;ZNF667;ZNF680;ZNF708;ZNF713;ZNF768;ZNF816;ZNF18;ZNF41;ZNF76;ZNF85;ZSCAN16;ZSCAN22;ZSCAN31;ZSCAN4	MYNN;MZF1;OSR2;PRDM14;PRDM6;WT1;ZBTB14;ZBTB48;ZBTB49;ZFP64;ZFP28;ZIM3;ZNF121;ZNF250;ZNF257;ZNF263;ZNF274;ZNF317;ZNF320;ZNF329;ZNF331;ZNF394;ZNF449;ZNF502;ZNF528;ZNF547;ZNF549;ZNF554;ZNF586;ZNF589;ZNF667;ZNF680;ZNF708;ZNF713;ZNF768;ZNF18;ZNF85;ZSCAN16;ZSCAN22
PRGR_HUMAN.H11MO.1.A	23	0.13450292	0.5374631	13.630.138	0.8744193	0.668500717	0.84911706	Steroid hormone receptors (NR3){2.1.1}	GR-like receptors (NR3C){2.1.1.1}	AR;ESR1;ESR2;ESRRA;ESRRB;ESRRG;NR3C1;NR3C2;PGR	AR;NR3C1;NR3C2;PGR
ESR2_HUMAN.H11MO.0.A	33	0.19298246	0.5374027	11.940.247	0.8116228	0.315567541	0.55803343	Steroid hormone receptors (NR3){2.1.1}	ER-like receptors (NR3A&B){2.1.1.2}	AR;ESR1;ESR2;ESRRA;ESRRB;ESRRG;NR3C1;NR3C2;PGR	ESRRA;ESRRB;ESRRG;ESR1;ESR2
PDX1_HUMAN.H11MO.0.A	8	0.04678363	0.5333042	25.379.149	1,26E+07	0.541383136	0.75617101	HOX-related factors{3.1.1}	PDX{3.1.1.15}	CDX1;CDX2;CDX4;EVX1;EVX2;GBX1;GBX2;GSX1;GSX2;HDX;HMBOX1;HNF1A;HNF1B;HOXA1;HOXA10;HOXA11;HOXA13;HOXA2;HOXA3;HOXA4;HOXA5;HOXA6;HOXA7;HOXA9;HOXB1;HOXB13;HOXB2;HOXB3;HOXB4;HOXB5;HOXB6;HOXB7;HOXB8;HOXB9;HOXC10;HOXC11;HOXC12;HOXC13;HOXC4;HOXC5;HOXC6;HOXC8;HOXC9;HOXD1;HOXD10;HOXD11;HOXD12;HOXD13;HOXD3;HOXD4;HOXD8;HOXD9;MEOX1;MEOX2;MNX1;PDX1;POU1F1;POU2F1;POU2F2;POU2F3;POU3F1;POU3F2;POU3F3;POU3F4;POU4F1;POU4F2;POU4F3;POU5F1;POU5F2;POU6F1;POU6F2	PDX1

motif	Nº of probes	% of probes	lower OR	upper OR	OR	p.value	FDR	TF family	TF subfamily	TF.family.member	TF.subfamily.member
ZN257_HUMAN.H11MO.0.C	20	0.11695906	0.5332079	14.372.756	0.8980898	0.732080038	0.87781292	More than 3 adjacent zinc finger factors{2.3.3}	unclassified{2.3.3.0}	BCL6B;BCL6;CTCFL;CTCF;FEZF1;GFI1B;GFI1;GLI1;GLI2;GLI3;GLIS1;GLIS2;GLIS3;MTF1;MYNN;MZF1;OSR2;OVOL1;OVOL2;ZNF146;PLAG1;PLAGL1;PRDM14;PRDM1;PRDM6;SCRT1;SCRT2;SNAI1;SNAI2;YY1;YY2;WT1;ZNF324;ZNF354A;ZBTB14;ZBTB18;ZBTB48;ZBTB49;ZBTB7A;ZBTB7B;ZBTB6;ZFP64;ZFP28;ZFP42;ZFP82;ZFX;ZIC1;ZIC2;ZIC3;ZIC4;ZIM3;ZKSCAN1;ZKSCAN3;ZNF121;ZNF136;ZNF140;ZNF143;ZNF148;ZNF214;ZNF232;ZNF250;ZNF257;ZNF260;ZNF263;ZNF264;ZNF274;ZNF281;ZNF282;ZNF317;ZNF320;ZNF322;ZNF329;ZNF331;ZNF333;ZNF350;ZNF384;ZNF394;ZNF410;ZNF436;ZNF449;ZNF490;ZNF502;ZNF524;ZNF528;ZNF547;ZNF549;ZNF554;ZNF563;ZNF582;ZNF586;ZNF589;ZNF652;ZNF667;ZNF680;ZNF708;ZNF713;ZNF768;ZNF816;ZNF18;ZNF41;ZNF76;ZNF85;ZSCAN16;ZSCAN22;ZSCAN31;ZSCAN4	MYNN;MZF1;OSR2;PRDM14;PRDM6;WT1;ZBTB14;ZBTB48;ZBTB49;ZFP64;ZFP28;ZIM3;ZNF121;ZNF250;ZNF257;ZNF263;ZNF274;ZNF317;ZNF320;ZNF329;ZNF331;ZNF394;ZNF449;ZNF502;ZNF528;ZNF547;ZNF549;ZNF554;ZNF586;ZNF589;ZNF667;ZNF680;ZNF708;ZNF713;ZNF768;ZNF18;ZNF85;ZSCAN16;ZSCAN22
PTF1A_HUMAN.H11MO.0.B	42	0.24561404	0.5330206	11.049.135	0.7743734	0.154984552	0.37341590	Tal-related factors{1.2.3}	Twist-like factors{1.2.3.2}	ATOH1;ATOH7;ATOH8;BHLHA15;BHLHA9;BHLHE22;BHLHE23;FERD3L;FIGLA;HAND1;HAND2;LYL1;MESP1;MESP2;MSC;MSGN1;NEUROD1;NEUROD2;NEUROD4;NEUROD6;NEUROG1;NEUROG2;NEUROG3;NHLH1;NHLH2;OLIG1;OLIG2;OLIG3;PTF1A;SCX;TAL1;TAL2;TCF15;TCF21;TCF23;TWIST1;TWIST2	FIGLA;HAND1;PTF1A;TWIST1
FOXH1_HUMAN.H11MO.0.A	13	0.07602339	0.5325851	18.010.258	1,02E+07	0.884048358	0.95980743	Forkhead box (FOX) factors{3.3.1}	FOXH{3.3.1.8}	FOXA1;FOXA2;FOXA3;FOXB1;FOXB2;FOXC1;FOXC2;FOXD1;FOXD2;FOXD3;FOXD4;FOXD4L1;FOXD4L3;FOXD4L4;FOXD4L5;FOXD4L6;FOXE1;FOXE3;FOXF1;FOXF2;FOXG1;FOXH1;FOXI1;FOXI2;FOXI3;FOXJ1;FOXJ2;FOXJ3;FOXK1;FOXK2;FOXL1;FOXL2;FOXM1;FOXN1;FOXN2;FOXN3;FOXN4;FOXO1;FOXO3;FOXO4;FOXO6;FOXP1;FOXP2;FOXP3;FOXP4;FOXQ1;FOXR1;FOXR2;FOXS1	FOXH1
Z324A_HUMAN.H11MO.0.C	29	0.16959064	0.5318089	12.339.659	0.8228915	0.388175820	0.62855249	More than 3 adjacent zinc finger factors{2.3.3}	ZNF324 factors{2.3.3.36}	BCL6;BCL6B;CTCF;CTCFL;FEZF1;FEZF2;GFI1;GFI1B;GLI1;GLI2;GLI3;GLI4;GLIS1;GLIS2;GLIS3;HKR1;MTF1;MYNN;MZF1;OSR2;OVOL1;OVOL2;PLAG1;PLAGL1;PLAGL2;PRDM1;PRDM14;PRDM6;SCRT1;SCRT2;SNAI1;SNAI2;SNAI3;WT1;YY1;YY2;ZBTB12;ZBTB14;ZBTB18;ZBTB20;ZBTB26;ZBTB42;ZBTB45;ZBTB47;ZBTB48;ZBTB49;ZBTB6;ZBTB7A;ZBTB7B;ZBTB7C;ZFP14;ZFP2;ZFP28;ZFP30;ZFP37;ZFP42;ZFP64;ZFP69;ZFP69B;ZFP82;ZFP91;ZFX;ZIC1;ZIC2;ZIC3;ZIC4;ZIC5;ZIK1;ZIM3;ZKSCAN1;ZKSCAN2;ZKSCAN3;ZKSCAN4;ZNF121;ZNF124;ZNF133;ZNF136;ZNF138;ZNF14;ZNF140;ZNF143;ZNF146;ZNF148;ZNF155;ZNF157;ZNF160;ZNF169;ZNF175;ZNF177;ZNF18;ZNF180;ZNF181;ZNF2;ZNF20;ZNF212;ZNF213;ZNF214;ZNF221;ZNF222;ZNF223;ZNF224;ZNF225;ZNF226;ZNF227;ZNF229;ZNF230;ZNF232;ZNF233;ZNF234;ZNF235;ZNF24;ZNF25;ZNF250;ZNF257;ZNF26;ZNF260;ZNF263;ZNF264;ZNF268;ZNF274;ZNF276;ZNF28;ZNF280A;ZNF280B;ZNF280C;ZNF280D;ZNF281;ZNF282;ZNF283;ZNF284;ZNF285;ZNF286A;ZNF286B;ZNF3;ZNF30;ZNF300;ZNF302;ZNF317;ZNF32;ZNF320;ZNF322;ZNF324;ZNF324B;ZNF329;ZNF331;ZNF333;ZNF33A;ZNF33B;ZNF343;ZNF345;ZNF347;ZNF350;ZNF354A;ZNF354B;ZNF362;ZNF366;ZNF383;ZNF384;ZNF394;ZNF397;ZNF398;ZNF404;ZNF41;ZNF410;ZNF419;ZNF420;ZNF431;ZNF432;ZNF436;ZNF439;ZNF44;ZNF440;ZNF442;ZNF443;ZNF446;ZNF449;ZNF45;ZNF460;ZNF468;ZNF479;ZNF484;ZNF490;ZNF500;ZNF502;ZNF524;ZNF525;ZNF528;ZNF543;ZNF54	ZNF324

motif	Nº of probes	% of probes	lower OR	upper OR	OR	p.value	FDR	TF family	TF subfamily	TF.family.member	TF.subfamily.member
										4;ZNF546;ZNF547;ZNF548;ZNF549;ZNF554;ZNF555;ZNF557;ZNF558;ZNF559;ZNF561;ZNF562;ZNF563;ZNF564;ZNF566;ZNF567;ZNF568;ZNF57;ZNF570;ZNF571;ZNF572;ZNF577;ZNF581;ZNF582;ZNF583;ZNF585A;ZNF586;ZNF589;ZNF595;ZNF599;ZNF600;ZNF605;ZNF607;ZNF611;ZNF613;ZNF614;ZNF615;ZNF616;ZNF619;ZNF620;ZNF621;ZNF625;ZNF627;ZNF649;ZNF652;ZNF653;ZNF665;ZNF667;ZNF669;ZNF670;ZNF672;ZNF679;ZNF680;ZNF683;ZNF689;ZNF692;ZNF701;ZNF705D;ZNF705E;ZNF705G;ZNF708;ZNF709;ZNF71;ZNF710;ZNF713;ZNF721;ZNF727;ZNF729;ZNF736;ZNF75A;ZNF75D;ZNF76;ZNF763;ZNF764;ZNF765;ZNF768;ZNF77;ZNF771;ZNF773;ZNF774;ZNF776;ZNF777;ZNF780A;ZNF780B;ZNF782;ZNF785;ZNF799;ZNF805;ZNF808;ZNF81;ZNF813;ZNF816;ZNF823;ZNF829;ZNF836;ZNF841;ZNF844;ZNF845;ZNF846;ZNF85;ZNF853;ZNF860;ZNF878;ZNF891;ZNF99;ZSCAN16;ZSCAN2;ZSCAN22;ZSCAN23;ZSCAN29;ZSCAN31;ZSCAN32;ZSCAN4;ZSCAN5A;ZSCAN5B;ZSCAN5C;ZSCAN9;ZXDA;ZXDB;ZXDC	
PRDM4_HUMAN.H11MO.0.D	14	0.08187135	0.5313589	17.201.901	0.9947006	1000000000,00000000	1,00E+08	Factors with multiple dispersed zinc fingers {2.3.4}	unclassified{2.3.4.0}	BCL11A;E4F1;MECOM;HIC1;HIC2;HINFP;IKZF1;INSM1;MAZ;PATZ1;PRDM4;REST;RREB1;SALL4;VEZF1;ZBTB17;ZBTB4;HIVEP1;HIVEP2;ZNF134;ZNF219;ZNF335;ZNF341;ZNF382;ZNF418;ZNF423;ZNF467;ZNF770;ZNF784;ZNF8	E4F1;PRDM4;REST;RREB1;ZBTB17;ZBTB4;ZNF335;ZNF341;ZNF467;ZNF770;ZNF784;ZNF8
MEIS3_HUMAN.H11MO.0.D	14	0.08187135	0.5300484	17.159.788	0.9922647	1000000000,00000000	1,00E+08	TALE-type homeo domain factors{3.1.4}	MEIS{3.1.4.2}	IRX1;IRX2;IRX3;IRX4;IRX5;IRX6;MEIS1;MEIS2;MEIS3;MKX;PBX1;PBX2;PBX3;PKNOX1;PKNOX2;TGIF1;TGIF2;TGIF2LX;TGIF2LY	MEIS1;MEIS2;MEIS3
ISX_HUMAN.H11MO.0.D	9	0.05263158	0.5286138	22.928.961	1,18E+07	578633,20000000	0.7845295158	Paired-related HD factors{3.1.3}	ISX{3.1.3.12}	ALX1;ALX3;ALX4;ARGFX;ARX;CRX;DMBX1;DPRX;DRGX;DUX4;DUXA;ESX1;GSC;GSC2;HESX1;ISX;LEUTX;MIXL1;NOBOX;OTP;OTX1;OTX2;PHOX2A;PHOX2B;PITX1;PITX2;PITX3;PROP1;PRRX1;PRRX2;RAX;RAX2;RHOXF1;RHOXF2;SEBOX;SHOX;SHOX2;TPRX1;UNCX;VSX1;VSX2	ISX
RFX5_HUMAN.H11MO.0.A	25	0.14619883	0.5278876	12.944.829	0.8426845	475520,20000000	0.6996680473	RFX-related factors {3.3.3}	RFX5{3.3.3.0.5}	RFX1;RFX2;RFX3;RFX4;RFX5;RFX6;RFX7;RFX8	RFX5
MCR_HUMAN.H11MO.0.D	13	0.07602339	0.5259080	17.784.633	1,01E+07	885221,40000000	0.9598074264	Steroid hormone receptors (NR3){2.1.1}	GR-like receptors (NR3C){2.1.1.1}	AR;ESR1;ESR2;ESRRA;ESRRB;ESRRG;NR3C1;NR3C2;PGR	AR;NR3C1;NR3C2;PGR
DUX4_HUMAN.H11MO.0.A	8	0.04678363	0.5256817	25.013.613	1,24E+07	544482,10000000	0.7563887045	Paired-related HD factors{3.1.3}	DUX{3.1.3.7}	ALX1;ALX3;ALX4;ARGFX;ARX;CRX;DMBX1;DPRX;DRGX;DUX4;DUXA;ESX1;GSC;GSC2;HESX1;ISX;LEUTX;MIXL1;NOBOX;OTP;OTX1;OTX2;PHOX2A;PHOX2B;PITX1;PITX2;PITX3;PROP1;PRRX1;PRRX2;RAX;RAX2;RHOXF1;RHOXF2;SEBOX;SHOX;SHOX2;TPRX1;UNCX;VSX1;VSX2	DUX4;DUXA
NR6A1_HUMAN.H11MO.0.B	12	0.07017544	0.5251630	18.658.345	1,04E+07	878507,50000000	0.9553304856	GCNF-related receptors (NR6){2.1.6}	GCNF (NR6A1){2.1.6.0.1}	NR6A1	NR6A1
HBP1_HUMAN.H11MO.0.D	7	0.04093567	0.5247633	27.980.773	1,33E+07	381703,00000000	0.6241319018	SOX-related factors {4.1.1}	Further Sox-related factors{4.1.1.9}	BBX;CIC;HBP1;SOX1;SOX10;SOX11;SOX12;SOX13;SOX14;SOX15;SOX17;SOX18;SOX2;SOX21;SOX3;SOX30;SOX4;SOX5;SOX6;SOX7;SOX8;SOX9;SRY	HBP1
ELK3_HUMAN.H11MO.0.D	26	0.15204678	0.5234246	12.641.718	0.8283930	423896,60000000	0.6675501646	Ets-related factors {3.5.2}	Elk-like factors{3.5.2.2}	EHF;ELF1;ELF2;ELF3;ELF4;ELF5;ELK1;ELK3;ELK4;ERF;ERG;ETS1;ETS2;ETV1;ETV2;ETV3;	ELK1;ELK3;ELK4;ETV1;ETV4;ETV5

motif	Nº of probes	% of probes	lower OR	upper OR	OR	p.value	FDR	TF family	TF subfamily	TF.family.member	TF.subfamily.member
										ETV3L;ETV4;ETV5;ETV6;ETV7;FEV;FLI1;GABPA;SPDEF;SPI1;SPIB;SPIC	
HXA9_HUMAN.H11MO.0.B	10	0.05847953	0.5222662	20.969.136	1,11E+07	730928,20000000	0.8777969811	HOX-related factors{3.1.1}	HOX9-13{3.1.1.8}	CDX1;CDX2;CDX4;EVX1;EVX2;GBX1;GBX2;GSX1;GSX2;HDX;HMBOX1;HNF1A;HNF1B;HOXA1;HOXA10;HOXA11;HOXA13;HOXA2;HOXA3;HOXA4;HOXA5;HOXA6;HOXA7;HOXA9;HOXB1;HOXB13;HOXB2;HOXB3;HOXB4;HOXB5;HOXB6;HOXB7;HOXB8;HOXB9;HOXC10;HOXC11;HOXC12;HOXC13;HOXC4;HOXC5;HOXC6;HOXC8;HOXC9;HOXD1;HOXD10;HOXD11;HOXD12;HOXD13;HOXD3;HOXD4;HOXD8;HOXD9;MEOX1;MEOX2;MNX1;PDX1;POU1F1;POU2F1;POU2F2;POU2F3;POU3F1;POU3F2;POU3F3;POU3F4;POU4F1;POU4F2;POU4F3;POU5F1;POU5F2;POU6F1;POU6F2	HOXA10;HOXA11;HOXA13;HOXA9;HOXB13;HOXC10;HOXC11;HOXC12;HOXC13;HOXC9;HOXD10;HOXD11;HOXD12;HOXD13;HOXD9
MAFG_HUMAN.H11MO.1.A	20	0.11695906	0.5215159	14.056.628	0.8783447	651111,10000000	0.8360721166	Maf-related factors{1.1.3}	Small Maf factors{1.1.3.2}	MAF;MAFA;MAFB;MAFF;MAFG;MAFK;NRL	MAFF;MAFG;MAFK
NANOG_HUMAN.H11MO.1.B	11	0.06432749	0.5211398	19.594.004	1,07E+07	749340,50000000	0.8847657508	NK-related factors{3.1.2}	NANOG{3.1.2.12}	BARHL1;BARHL2;BARX1;BARX2;BSX;DBX1;DBX2;DLX1;DLX2;DLX3;DLX4;DLX5;DLX6;EMX1;EMX2;EN1;EN2;HHEX;HLX;HMX1;HMX2;HMX3;LBX1;LBX2;MSX1;MSX2;NANOG;NKX1-1;NKX1-2;NKX2-1;NKX2-2;NKX2-3;NKX2-4;NKX2-5;NKX2-6;NKX2-8;NKX3-1;NKX3-2;NKX6-1;NKX6-2;NKX6-3;NOTO;TLX1;TLX2;TLX3;VAX1;VAX2;VENTX	NANOG
ZBTB6_HUMAN.H11MO.0.C	35	0.20467836	0.5203631	11.351.848	0.7778700	214781,80000000	0.4439591877	More than 3 adjacent zinc finger factors{2.3.3}	ZBTB6-like factors{2.3.3.11}	BCL6;BCL6B;CTCF;CTCFL;FEZF1;FEZF2;GFI1;GFI1B;GLI1;GLI2;GLI3;GLI4;GLIS1;GLIS2;GLIS3;HKR1;MTF1;MYNN;MZF1;OSR2;OVOL1;OVOL2;PLAG1;PLAGL1;PLAGL2;PRDM1;PRDM14;PRDM6;SCRT1;SCRT2;SNAI1;SNAI2;SNAI3;WT1;YY1;YY2;ZBTB12;ZBTB14;ZBTB18;ZBTB20;ZBTB26;ZBTB42;ZBTB45;ZBTB47;ZBTB48;ZBTB49;ZBTB6;ZBTB7A;ZBTB7B;ZBTB7C;ZFP14;ZFP2;ZFP28;ZFP30;ZFP37;ZFP42;ZFP64;ZFP69;ZFP69B;ZFP82;ZFP91;ZFX;ZIC1;ZIC2;ZIC3;ZIC4;ZIC5;ZIK1;ZIM3;ZKSCAN1;ZKSCAN2;ZKSCAN3;ZKSCAN4;ZNF121;ZNF124;ZNF133;ZNF136;ZNF138;ZNF14;ZNF140;ZNF143;ZNF146;ZNF148;ZNF155;ZNF157;ZNF160;ZNF169;ZNF175;ZNF177;ZNF18;ZNF180;ZNF181;ZNF2;ZNF20;ZNF212;ZNF213;ZNF214;ZNF221;ZNF222;ZNF223;ZNF224;ZNF225;ZNF226;ZNF227;ZNF229;ZNF230;ZNF232;ZNF233;ZNF234;ZNF235;ZNF24;ZNF25;ZNF250;ZNF257;ZNF26;ZNF260;ZNF263;ZNF264;ZNF268;ZNF274;ZNF276;ZNF28;ZNF280A;ZNF280B;ZNF280C;ZNF280D;ZNF281;ZNF282;ZNF283;ZNF284;ZNF285;ZNF286A;ZNF286B;ZNF3;ZNF30;ZNF300;ZNF302;ZNF317;ZNF32;ZNF320;ZNF322;ZNF324;ZNF324B;ZNF329;ZNF331;ZNF333;ZNF33A;ZNF33B;ZNF343;ZNF345;ZNF347;ZNF350;ZNF354A;ZNF354B;ZNF362;ZNF366;ZNF383;ZNF384;ZNF394;ZNF397;ZNF398;ZNF404;ZNF41;ZNF410;ZNF419;ZNF420;ZNF431;ZNF432;ZNF436;ZNF439;ZNF44;ZNF440;ZNF442;ZNF443;ZNF446;ZNF449;ZNF45;ZNF460;ZNF468;ZNF479;ZNF484;ZNF490;ZNF500;ZNF502;ZNF524;ZNF525;ZNF528;ZNF543;ZNF544;ZNF546;ZNF547;ZNF548;ZNF549;ZNF554;ZNF555;ZNF557;ZNF558;ZNF559;ZNF561;ZNF562;ZNF563;ZNF564;ZNF566;ZNF567;ZNF568;ZNF57;ZNF570;ZNF571;ZNF572;ZNF577;ZNF581;ZNF582;ZNF583;ZNF585A;ZNF586;ZNF589;ZNF595;ZNF599;ZNF600;ZNF605;ZNF607;ZNF611;ZN	ZBTB6

motif	Nº of probes	% of probes	lower OR	upper OR	OR	p.value	FDR	TF family	TF subfamily	TF.family.member	TF.subfamily.member
										F613;ZNF614;ZNF615;ZNF616;ZNF619;ZNF620;ZNF621;ZNF625;ZNF627;ZNF649;ZNF652;ZNF653;ZNF665;ZNF667;ZNF669;ZNF670;ZNF672;ZNF679;ZNF680;ZNF683;ZNF689;ZNF692;ZNF701;ZNF705D;ZNF705E;ZNF705G;ZNF708;ZNF709;ZNF71;ZNF710;ZNF713;ZNF721;ZNF727;ZNF729;ZNF736;ZNF75A;ZNF75D;ZNF76;ZNF763;ZNF764;ZNF765;ZNF768;ZNF77;ZNF771;ZNF773;ZNF774;ZNF776;ZNF777;ZNF780A;ZNF780B;ZNF782;ZNF785;ZNF799;ZNF805;ZNF808;ZNF81;ZNF813;ZNF816;ZNF823;ZNF829;ZNF836;ZNF841;ZNF844;ZNF845;ZNF846;ZNF85;ZNF853;ZNF860;ZNF878;ZNF891;ZNF99;ZSCAN16;ZSCAN2;ZSCAN22;ZSCAN23;ZSCAN29;ZSCAN31;ZSCAN32;ZSCAN4;ZSCAN5A;ZSCAN5B;ZSCAN5C;ZSCAN9;ZXDA;ZXDB;ZXDC	
BRAC_HUMAN.H11MO.0.A	14	0.08187135	0.5195739	16.821.190	0.9726761	1000000,00000000	1,00E+10	Brachyury-related factors{6.5.1}	T (Brachyury){6.5.1.0.1}	T;TBX19	T
PKNX1_HUMAN.H11MO.0.B	20	0.11695906	0.5193608	13.998.677	0.8747225	651067,40000000	0.8360721166	TALE-type homeo domain factors{3.1.4}	PKNOX{3.1.4.5}	IRX1;IRX2;IRX3;IRX4;IRX5;IRX6;MEIS1;MEIS2;MEIS3;MKX;PBX1;PBX2;PBX3;PKNOX1;PKNOX2;TGIF1;TGIF2;TGIF2LX;TGIF2LY	PKNOX1
HNF4A_HUMAN.H11MO.0.A	14	0.08187135	0.5191042	16.806.015	0.9717981	1000000,00000000	1,00E+10	RXR-related receptors (NR2){2.1.3}	HNF-4 (NR2A){2.1.3.2}	HNF4A;HNF4G;NR2C1;NR2C2;NR2E1;NR2E3;NR2F1;NR2F2;NR2F6;RXRA;RXRB;RXRG	HNF4A;HNF4G
ATF3_HUMAN.H11MO.0.A	23	0.13450292	0.5185974	13.151.872	0.8437298	526167,30000000	0.7443577118	Fos-related factors{1.1.2}	ATF-3-like factors{1.1.2.2}	ATF3;FOS;FOSB;FOSL1;FOSL2;JDP2	ATF3;JDP2
TBX1_HUMAN.H11MO.0.D	72	0.42105263	0.5185917	0.9756333	0.7127785	31885,14000000	0.1260689547	TBX1-related factors{6.5.3}	TBX1{6.5.3.0.1}	TBX1;TBX10;TBX15;TBX18;TBX20;TBX22	TBX1

motif	Nº of probes	% of probes	lower OR	upper OR	OR	p.value	FDR	TF family	TF subfamily	TF.family.member	TF.subfamily.member
ZN384_HUMAN.H11MO.0.C	11	0.06432749	0.5184310	19.490.855	1,06E+07	750501,70000000	0.8847657508	More than 3 adjacent zinc finger factors{2.3.3}	ZNF362-like factors{2.3.3.37}	BCL6;BCL6B;CTCF;CTCFL;FEZF1;FEZF2;GFI1;GFI1B;GLI1;GLI2;GLI3;GLI4;GLIS1;GLIS2;GLIS3;HKR1;MTF1;MYNN;MZF1;OSR2;OVOL1;OVOL2;PLAG1;PLAGL1;PLAGL2;PRDM1;PRDM14;PRDM6;SCRT1;SCRT2;SNAI1;SNAI2;SNAI3;WT1;YY1;YY2;ZBTB12;ZBTB14;ZBTB18;ZBTB20;ZBTB26;ZBTB42;ZBTB45;ZBTB47;ZBTB48;ZBTB49;ZBTB6;ZBTB7A;ZBTB7B;ZBTB7C;ZFP14;ZFP2;ZFP28;ZFP30;ZFP37;ZFP42;ZFP64;ZFP69;ZFP69B;ZFP82;ZFP91;ZFX;ZIC1;ZIC2;ZIC3;ZIC4;ZIC5;ZIK1;ZIM3;ZKSCAN1;ZKSCAN2;ZKSCAN3;ZKSCAN4;ZNF121;ZNF124;ZNF133;ZNF136;ZNF138;ZNF14;ZNF140;ZNF143;ZNF146;ZNF148;ZNF155;ZNF157;ZNF160;ZNF169;ZNF175;ZNF177;ZNF18;ZNF180;ZNF181;ZNF2;ZNF20;ZNF212;ZNF213;ZNF214;ZNF221;ZNF222;ZNF223;ZNF224;ZNF225;ZNF226;ZNF227;ZNF229;ZNF230;ZNF232;ZNF233;ZNF234;ZNF235;ZNF24;ZNF25;ZNF250;ZNF257;ZNF26;ZNF260;ZNF263;ZNF264;ZNF268;ZNF274;ZNF276;ZNF28;ZNF280A;ZNF280B;ZNF280C;ZNF280D;ZNF281;ZNF282;ZNF283;ZNF284;ZNF285;ZNF286A;ZNF286B;ZNF3;ZNF30;ZNF300;ZNF302;ZNF317;ZNF32;ZNF320;ZNF322;ZNF324;ZNF324B;ZNF329;ZNF331;ZNF333;ZNF33A;ZNF33B;ZNF343;ZNF345;ZNF347;ZNF350;ZNF354A;ZNF354B;ZNF362;ZNF366;ZNF383;ZNF384;ZNF394;ZNF397;ZNF398;ZNF404;ZNF41;ZNF410;ZNF419;ZNF420;ZNF431;ZNF432;ZNF436;ZNF439;ZNF44;ZNF440;ZNF442;ZNF443;ZNF446;ZNF449;ZNF45;ZNF460;ZNF468;ZNF479;ZNF484;ZNF490;ZNF500;ZNF502;ZNF524;ZNF525;ZNF528;ZNF543;ZNF544;ZNF546;ZNF547;ZNF548;ZNF549;ZNF554;ZNF555;ZNF557;ZNF558;ZNF559;ZNF561;ZNF562;ZNF563;ZNF564;ZNF566;ZNF567;ZNF568;ZNF57;ZNF570;ZNF571;ZNF572;ZNF577;ZNF581;ZNF582;ZNF583;ZNF585A;ZNF586;ZNF589;ZNF595;ZNF599;ZNF600;ZNF605;ZNF607;ZNF611;ZNF613;ZNF614;ZNF615;ZNF616;ZNF619;ZNF620;ZNF621;ZNF625;ZNF627;ZNF649;ZNF652;ZNF653;ZNF665;ZNF667;ZNF669;ZNF670;ZNF672;ZNF679;ZNF680;ZNF683;ZNF689;ZNF692;ZNF701;ZNF705D;ZNF705E;ZNF705G;ZNF708;ZNF709;ZNF71;ZNF710;ZNF713;ZNF721;ZNF727;ZNF729;ZNF736;ZNF75A;ZNF75D;ZNF76;ZNF763;ZNF764;ZNF765;ZNF768;ZNF77;ZNF771;ZNF773;ZNF774;ZNF776;ZNF777;ZNF780A;ZNF780B;ZNF782;ZNF785;ZNF799;ZNF805;ZNF808;ZNF81;ZNF813;ZNF816;ZNF823;ZNF829;ZNF836;ZNF841;ZNF844;ZNF845;ZNF846;ZNF85;ZNF853;ZNF860;ZNF878;ZNF891;ZNF99;ZSCAN16;ZSCAN2;ZSCAN22;ZSCAN23;ZSCAN29;ZSCAN31;ZSCAN32;ZSCAN4;ZSCAN5A;ZSCAN5B;ZSCAN5C;ZSCAN9;ZXDA;ZXDB;ZXDC	ZNF384
OSR2_HUMAN.H11MO.0.C	41	0.23976608	0.5168315	10.780.189	0.7534713	130609,70000000	0.3306977504	More than 3 adjacent zinc finger factors{2.3.3}	unclassified{2.3.3.0}	BCL6B;BCL6;CTCFL;CTCF;FEZF1;GFI1B;GFI1;GLI1;GLI2;GLI3;GLIS1;GLIS2;GLIS3;MTF1;MYNN;MZF1;OSR2;OVOL1;OVOL2;ZNF146;PLAG1;PLAGL1;PRDM14;PRDM1;PRDM6;SCRT1;SCRT2;SNAI1;SNAI2;YY1;YY2;WT1;ZNF324;ZNF354A;ZBTB14;ZBTB18;ZBTB48;ZBTB49;ZBTB7A;ZBTB7B;ZBTB6;ZFP64;ZFP28;ZFP42;ZFP82;ZFX;ZIC1;ZIC2;ZIC3;ZIC4;ZIM3;ZKSCAN1;ZKSCAN3;ZNF121;ZNF136;ZNF140;ZNF143;ZNF148;ZNF214;ZNF232;ZNF250;ZNF257;ZNF260;ZNF263;ZNF264;ZNF274;ZNF281;ZNF282;ZNF317;ZNF320;ZNF322;ZNF329;ZNF331;ZNF333;ZN	MYNN;MZF1;OSR2;PRDM14;PRDM6;WT1;ZBTB14;ZBTB48;ZBTB49;ZFP64;ZFP28;ZIM3;ZNF121;ZNF250;ZNF257;ZNF263;ZNF274;ZNF317;ZNF320;ZNF329;ZNF331;ZNF394;ZNF449;ZNF502;ZNF528;ZNF547;ZNF549;ZNF554;ZNF586;ZNF589;ZNF667;ZNF680;ZNF708;ZNF713;ZNF768;ZNF18;ZNF85;ZSCAN16;ZSCAN22

motif	Nº of probes	% of probes	lower OR	upper OR	OR	p.value	FDR	TF family	TF subfamily	TF.family.member	TF.subfamily.member
										F350;ZNF384;ZNF394;ZNF410;ZNF436;ZNF449;ZNF490;ZNF502;ZNF524;ZNF528;ZNF547;ZNF549;ZNF554;ZNF563;ZNF582;ZNF586;ZNF589;ZNF652;ZNF667;ZNF680;ZNF708;ZNF713;ZNF768;ZNF816;ZNF18;ZNF41;ZNF76;ZNF85;ZSCAN16;ZSCAN22;ZSCAN31;ZSCAN4	
PURA_HUMAN.H11MO.0.D	66	0.38596491	0.5163332	0.9804180	0.7137070	31854,51000000	0.1260689547	PUR{0.0.5}	Pur-alpha (PURA, PUR1){0.0.5.0.1}	PURA;PURB;PURG	PURA
HES5_HUMAN.H11MO.0.D	41	0.23976608	0.5152090	10.746.847	0.7511278	111825,20000000	0.2912743247	Hairy-related factors{1.2.4}	Hairy-like factors{1.2.4.1}	BHLHE40;BHLHE41;HELT;HES1;HES2;HES3;HES4;HES5;HES6;HES7;HEY1;HEY2;HEYL	BHLHE40;BHLHE41;HES1;HES5;HES7;HEY1;HEY2
ANDR_HUMAN.H11MO.1.A	18	0.10526316	0.5130033	14.534.390	0.8890416	721356,50000000	0.8766208224	Steroid hormone receptors (NR3){2.1.1}	GR-like receptors (NR3C){2.1.1.1}	AR;ESR1;ESR2;ESRRA;ESRRB;ESRRG;NR3C1;NR3C2;PGR	AR;NR3C1;NR3C2;PGR
ZNF18_HUMAN.H11MO.0.C	21	0.12280702	0.5120502	13.502.428	0.8517759	582324,90000000	0.7848088889	More than 3 adjacent zinc finger factors{2.3.3}	unclassified{2.3.3.0}	BCL6B;BCL6;CTCFL;CTCF;FEZF1;GFI1B;GFI1;GLI1;GLI2;GLI3;GLIS1;GLIS2;GLIS3;MTF1;MYNN;MZF1;OSR2;OVOL1;OVOL2;ZNF146;PLAG1;PLAGL1;PRDM14;PRDM1;PRDM6;SCRT1;SCRT2;SNAI1;SNAI2;YY1;YY2;WT1;ZNF324;ZNF354A;ZBTB14;ZBTB18;ZBTB48;ZBTB49;ZBTB7A;ZBTB7B;ZBTB6;ZFP64;ZFP28;ZFP42;ZFP82;ZFX;ZIC1;ZIC2;ZIC3;ZIC4;ZIM3;ZKSCAN1;ZKSCAN3;ZNF121;ZNF136;ZNF140;ZNF143;ZNF148;ZNF214;ZNF232;ZNF250;ZNF257;ZNF260;ZNF263;ZNF264;ZNF274;ZNF281;ZNF282;ZNF317;ZNF320;ZNF322;ZNF329;ZNF331;ZNF333;ZNF350;ZNF384;ZNF394;ZNF410;ZNF436;ZNF449;ZNF490;ZNF502;ZNF524;ZNF528;ZNF547;ZNF549;ZNF554;ZNF563;ZNF582;ZNF586;ZNF589;ZNF652;ZNF667;ZNF680;ZNF708;ZNF713;ZNF768;ZNF816;ZNF18;ZNF41;ZNF76;ZNF85;ZSCAN16;ZSCAN22;ZSCAN31;ZSCAN4	MYNN;MZF1;OSR2;PRDM14;PRDM6;WT1;ZBTB14;ZBTB48;ZBTB49;ZFP64;ZFP28;ZIM3;ZNF121;ZNF250;ZNF257;ZNF263;ZNF274;ZNF317;ZNF320;ZNF329;ZNF331;ZNF394;ZNF449;ZNF502;ZNF528;ZNF547;ZNF549;ZNF554;ZNF586;ZNF589;ZNF667;ZNF680;ZNF708;ZNF713;ZNF768;ZNF18;ZNF85;ZSCAN16;ZSCAN22
ZN768_HUMAN.H11MO.0.C	33	0.19298246	0.5115605	11.366.171	0.7725971	207119,20000000	0.4401243789	More than 3 adjacent zinc finger factors{2.3.3}	unclassified{2.3.3.0}	BCL6B;BCL6;CTCFL;CTCF;FEZF1;GFI1B;GFI1;GLI1;GLI2;GLI3;GLIS1;GLIS2;GLIS3;MTF1;MYNN;MZF1;OSR2;OVOL1;OVOL2;ZNF146;PLAG1;PLAGL1;PRDM14;PRDM1;PRDM6;SCRT1;SCRT2;SNAI1;SNAI2;YY1;YY2;WT1;ZNF324;ZNF354A;ZBTB14;ZBTB18;ZBTB48;ZBTB49;ZBTB7A;ZBTB7B;ZBTB6;ZFP64;ZFP28;ZFP42;ZFP82;ZFX;ZIC1;ZIC2;ZIC3;ZIC4;ZIM3;ZKSCAN1;ZKSCAN3;ZNF121;ZNF136;ZNF140;ZNF143;ZNF148;ZNF214;ZNF232;ZNF250;ZNF257;ZNF260;ZNF263;ZNF264;ZNF274;ZNF281;ZNF282;ZNF317;ZNF320;ZNF322;ZNF329;ZNF331;ZNF333;ZNF350;ZNF384;ZNF394;ZNF410;ZNF436;ZNF449;ZNF490;ZNF502;ZNF524;ZNF528;ZNF547;ZNF549;ZNF554;ZNF563;ZNF582;ZNF586;ZNF589;ZNF652;ZNF667;ZNF680;ZNF708;ZNF713;ZNF768;ZNF816;ZNF18;ZNF41;ZNF76;ZNF85;ZSCAN16;ZSCAN22;ZSCAN31;ZSCAN4	MYNN;MZF1;OSR2;PRDM14;PRDM6;WT1;ZBTB14;ZBTB48;ZBTB49;ZFP64;ZFP28;ZIM3;ZNF121;ZNF250;ZNF257;ZNF263;ZNF274;ZNF317;ZNF320;ZNF329;ZNF331;ZNF394;ZNF449;ZNF502;ZNF528;ZNF547;ZNF549;ZNF554;ZNF586;ZNF589;ZNF667;ZNF680;ZNF708;ZNF713;ZNF768;ZNF18;ZNF85;ZSCAN16;ZSCAN22

motif	Nº of probes	% of probes	lower OR	upper OR	OR	p.value	FDR	TF family	TF subfamily	TF.family.member	TF.subfamily.member
ZN563_HUMAN.H11MO.1.C	42	0.24561404	0.5109219	10.591.237	0.7422946	96697,92000000	0.2672189818	More than 3 adjacent zinc finger factors{2.3.3}	ZNF763-like factors{2.3.3.33}	BCL6;BCL6B;CTCF;CTCFL;FEZF1;FEZF2;GFI1;GFI1B;GLI1;GLI2;GLI3;GLI4;GLIS1;GLIS2;GLIS3;HKR1;MTF1;MYNN;MZF1;OSR2;OVOL1;OVOL2;PLAG1;PLAGL1;PLAGL2;PRDM1;PRDM14;PRDM6;SCRT1;SCRT2;SNAI1;SNAI2;SNAI3;WT1;YY1;YY2;ZBTB12;ZBTB14;ZBTB18;ZBTB20;ZBTB26;ZBTB42;ZBTB45;ZBTB47;ZBTB48;ZBTB49;ZBTB6;ZBTB7A;ZBTB7B;ZBTB7C;ZFP14;ZFP2;ZFP28;ZFP30;ZFP37;ZFP42;ZFP64;ZFP69;ZFP69B;ZFP82;ZFP91;ZFX;ZIC1;ZIC2;ZIC3;ZIC4;ZIC5;ZIK1;ZIM3;ZKSCAN1;ZKSCAN2;ZKSCAN3;ZKSCAN4;ZNF121;ZNF124;ZNF133;ZNF136;ZNF138;ZNF14;ZNF140;ZNF143;ZNF146;ZNF148;ZNF155;ZNF157;ZNF160;ZNF169;ZNF175;ZNF177;ZNF18;ZNF180;ZNF181;ZNF2;ZNF20;ZNF212;ZNF213;ZNF214;ZNF221;ZNF222;ZNF223;ZNF224;ZNF225;ZNF226;ZNF227;ZNF229;ZNF230;ZNF232;ZNF233;ZNF234;ZNF235;ZNF24;ZNF25;ZNF250;ZNF257;ZNF26;ZNF260;ZNF263;ZNF264;ZNF268;ZNF274;ZNF276;ZNF28;ZNF280A;ZNF280B;ZNF280C;ZNF280D;ZNF281;ZNF282;ZNF283;ZNF284;ZNF285;ZNF286A;ZNF286B;ZNF3;ZNF30;ZNF300;ZNF302;ZNF317;ZNF32;ZNF320;ZNF322;ZNF324;ZNF324B;ZNF329;ZNF331;ZNF333;ZNF33A;ZNF33B;ZNF343;ZNF345;ZNF347;ZNF350;ZNF354A;ZNF354B;ZNF362;ZNF366;ZNF383;ZNF384;ZNF394;ZNF397;ZNF398;ZNF404;ZNF41;ZNF410;ZNF419;ZNF420;ZNF431;ZNF432;ZNF436;ZNF439;ZNF44;ZNF440;ZNF442;ZNF443;ZNF446;ZNF449;ZNF45;ZNF460;ZNF468;ZNF479;ZNF484;ZNF490;ZNF500;ZNF502;ZNF524;ZNF525;ZNF528;ZNF543;ZNF544;ZNF546;ZNF547;ZNF548;ZNF549;ZNF554;ZNF555;ZNF557;ZNF558;ZNF559;ZNF561;ZNF562;ZNF563;ZNF564;ZNF566;ZNF567;ZNF568;ZNF57;ZNF570;ZNF571;ZNF572;ZNF577;ZNF581;ZNF582;ZNF583;ZNF585A;ZNF586;ZNF589;ZNF595;ZNF599;ZNF600;ZNF605;ZNF607;ZNF611;ZNF613;ZNF614;ZNF615;ZNF616;ZNF619;ZNF620;ZNF621;ZNF625;ZNF627;ZNF649;ZNF652;ZNF653;ZNF665;ZNF667;ZNF669;ZNF670;ZNF672;ZNF679;ZNF680;ZNF683;ZNF689;ZNF692;ZNF701;ZNF705D;ZNF705E;ZNF705G;ZNF708;ZNF709;ZNF71;ZNF710;ZNF713;ZNF721;ZNF727;ZNF729;ZNF736;ZNF75A;ZNF75D;ZNF76;ZNF763;ZNF764;ZNF765;ZNF768;ZNF77;ZNF771;ZNF773;ZNF774;ZNF776;ZNF777;ZNF780A;ZNF780B;ZNF782;ZNF785;ZNF799;ZNF805;ZNF808;ZNF81;ZNF813;ZNF816;ZNF823;ZNF829;ZNF836;ZNF841;ZNF844;ZNF845;ZNF846;ZNF85;ZNF853;ZNF860;ZNF878;ZNF891;ZNF99;ZSCAN16;ZSCAN2;ZSCAN22;ZSCAN23;ZSCAN29;ZSCAN31;ZSCAN32;ZSCAN4;ZSCAN5A;ZSCAN5B;ZSCAN5C;ZSCAN9;ZXDA;ZXDB;ZXDC	ZNF136;ZNF490;ZNF563
RARG_HUMAN.H11MO.1.B	22	0.12865497	0.5096151	13.168.758	0.8379841	519660,90000000	0.7365047620	Thyroid hormone receptor-related factors (NR1){2.1.2}	Retinoic acid receptors (NR1B){2.1.2.1}	NR1D1;NR1D2;NR1H2;NR1H3;NR1H4;NR1I2;NR1I3;PPARA;PPARD;PPARG;RARA;RARB;RARG;RORA;RORB;RORC;THRA;THRB;VDR	RARA;RARB;RARG
TGIF2_HUMAN.H11MO.0.D	16	0.09356725	0.5092563	15.316.937	0.9134821	898903,00000000	0.9612402184	TALE-type homeo domain factors{3.1.4}	TGIF{3.1.4.6}	IRX1;IRX2;IRX3;IRX4;IRX5;IRX6;MEIS1;MEIS2;MEIS3;MKX;PBX1;PBX2;PBX3;PKNOX1;PKNOX2;TGIF1;TGIF2;TGIF2LX;TGIF2LY	TGIF2LX;TGIF1;TGIF2
ARI5B_HUMAN.H11MO.0.C	10	0.05847953	0.5088860	20.429.210	1,08E+07	735544,10000000	0.8800901085	ARID-related factors{3.7.1}	ARID5{3.7.1.5}	ARID2;ARID3A;ARID5A;ARID5B;KDM5B	ARID5B

motif	Nº of probes	% of probes	lower OR	upper OR	OR	p.value	FDR	TF family	TF subfamily	TF.family.member	TF.subfamily.member
NR4A1_HUMAN.H11MO.0.A	13	0.07602339	0.5063812	17.123.845	0.9724463	1000000,00000000	1,00E+10	NGFI-B-related receptors (NR4){2.1.4}	NGFI-B (NR4A1){2.1.4.0.1}	NR4A1;NR4A2;NR4A3	NR4A1
GFI1B_HUMAN.H11MO.0.A	14	0.08187135	0.5050976	16.351.473	0.9455603	1000000,00000000	1,00E+10	More than 3 adjacent zinc finger factors{2.3}	GFI1 factors{2.3.3.21}	BCL6;BCL6B;CTCF;CTCFL;FEZF1;FEZF2;GFI1;GFI1B;GLI1;GLI2;GLI3;GLI4;GLIS1;GLIS2;GLIS3;HKR1;MTF1;MYNN;MZF1;OSR2;OVOL1;OVOL2;PLAG1;PLAGL1;PLAGL2;PRDM1;PRDM14;PRDM6;SCRT1;SCRT2;SNAI1;SNAI2;SNAI3;WT1;YY1;YY2;ZBTB12;ZBTB14;ZBTB18;ZBTB20;ZBTB26;ZBTB42;ZBTB45;ZBTB47;ZBTB48;ZBTB49;ZBTB6;ZBTB7A;ZBTB7B;ZBTB7C;ZFP14;ZFP2;ZFP28;ZFP30;ZFP37;ZFP42;ZFP64;ZFP69;ZFP69B;ZFP82;ZFP91;ZFX;ZIC1;ZIC2;ZIC3;ZIC4;ZIC5;ZIK1;ZIM3;ZKSCAN1;ZKSCAN2;ZKSCAN3;ZKSCAN4;ZNF121;ZNF124;ZNF133;ZNF136;ZNF138;ZNF14;ZNF140;ZNF143;ZNF146;ZNF148;ZNF155;ZNF157;ZNF160;ZNF169;ZNF175;ZNF177;ZNF18;ZNF180;ZNF181;ZNF2;ZNF20;ZNF212;ZNF213;ZNF214;ZNF221;ZNF222;ZNF223;ZNF224;ZNF225;ZNF226;ZNF227;ZNF229;ZNF230;ZNF232;ZNF233;ZNF234;ZNF235;ZNF24;ZNF25;ZNF250;ZNF257;ZNF26;ZNF260;ZNF263;ZNF264;ZNF268;ZNF274;ZNF276;ZNF28;ZNF280A;ZNF280B;ZNF280C;ZNF280D;ZNF281;ZNF282;ZNF283;ZNF284;ZNF285;ZNF286A;ZNF286B;ZNF3;ZNF30;ZNF300;ZNF302;ZNF317;ZNF32;ZNF320;ZNF322;ZNF324;ZNF324B;ZNF329;ZNF331;ZNF333;ZNF33A;ZNF33B;ZNF343;ZNF345;ZNF347;ZNF350;ZNF354A;ZNF354B;ZNF362;ZNF366;ZNF383;ZNF384;ZNF394;ZNF397;ZNF398;ZNF404;ZNF41;ZNF410;ZNF419;ZNF420;ZNF431;ZNF432;ZNF436;ZNF439;ZNF44;ZNF440;ZNF442;ZNF443;ZNF446;ZNF449;ZNF45;ZNF460;ZNF468;ZNF479;ZNF484;ZNF490;ZNF500;ZNF502;ZNF524;ZNF525;ZNF528;ZNF543;ZNF544;ZNF546;ZNF547;ZNF548;ZNF549;ZNF554;ZNF555;ZNF557;ZNF558;ZNF559;ZNF561;ZNF562;ZNF563;ZNF564;ZNF566;ZNF567;ZNF568;ZNF57;ZNF570;ZNF571;ZNF572;ZNF577;ZNF581;ZNF582;ZNF583;ZNF585A;ZNF586;ZNF589;ZNF595;ZNF599;ZNF600;ZNF605;ZNF607;ZNF611;ZNF613;ZNF614;ZNF615;ZNF616;ZNF619;ZNF620;ZNF621;ZNF625;ZNF627;ZNF649;ZNF652;ZNF653;ZNF665;ZNF667;ZNF669;ZNF670;ZNF672;ZNF679;ZNF680;ZNF683;ZNF689;ZNF692;ZNF701;ZNF705D;ZNF705E;ZNF705G;ZNF708;ZNF709;ZNF71;ZNF710;ZNF713;ZNF721;ZNF727;ZNF729;ZNF736;ZNF75A;ZNF75D;ZNF76;ZNF763;ZNF764;ZNF765;ZNF768;ZNF77;ZNF771;ZNF773;ZNF774;ZNF776;ZNF777;ZNF780A;ZNF780B;ZNF782;ZNF785;ZNF799;ZNF805;ZNF808;ZNF81;ZNF813;ZNF816;ZNF823;ZNF829;ZNF836;ZNF841;ZNF844;ZNF845;ZNF846;ZNF85;ZNF853;ZNF860;ZNF878;ZNF891;ZNF99;ZSCAN16;ZSCAN2;ZSCAN22;ZSCAN23;ZSCAN29;ZSCAN31;ZSCAN32;ZSCAN4;ZSCAN5A;ZSCAN5B;ZSCAN5C;ZSCAN9;ZXDA;ZXDB;ZXDC	GFI1B;GFI1
E2F3_HUMAN.H11MO.0.A	35	0.20467836	0.5047619	11.011.701	0.7545525	159291,50000000	0.3802283444	E2F-related factors{3.3.2}	E2F{3.3.2.1}	E2F1;E2F2;E2F3;E2F4;E2F5;E2F6;E2F7;E2F8;TFDP1;TFDP2	E2F1;E2F2;E2F3;E2F4;E2F5;E2F6;E2F7;E2F8

motif	Nº of probes	% of probes	lower OR	upper OR	OR	p.value	FDR	TF family	TF subfamily	TF.family.member	TF.subfamily.member
GBX2_HUMAN.H11MO.0.D	9	0.05263158	0.5046761	21.889.872	1,12E+07	715831,30000000	0.8746378511	HOX-related factors{3.1.1}	GBX (Gastrulation brain homeobox){3.1.1.11}	CDX1;CDX2;CDX4;EVX1;EVX2;GBX1;GBX2;GSX1;GSX2;HDX;HMBOX1;HNF1A;HNF1B;HOXA1;HOXA10;HOXA11;HOXA13;HOXA2;HOXA3;HOXA4;HOXA5;HOXA6;HOXA7;HOXA9;HOXB1;HOXB13;HOXB2;HOXB3;HOXB4;HOXB5;HOXB6;HOXB7;HOXB8;HOXB9;HOXC10;HOXC11;HOXC12;HOXC13;HOXC4;HOXC5;HOXC6;HOXC8;HOXC9;HOXD1;HOXD10;HOXD11;HOXD12;HOXD13;HOXD3;HOXD4;HOXD8;HOXD9;MEOX1;MEOX2;MNX1;PDX1;POU1F1;POU2F1;POU2F2;POU2F3;POU3F1;POU3F2;POU3F3;POU3F4;POU4F1;POU4F2;POU4F3;POU5F1;POU5F2;POU6F1;POU6F2	GBX1;GBX2
CEBPZ_HUMAN.H11MO.0.D	10	0.05847953	0.5044881	20.253.833	1,07E+07	737403,60000000	0.8800901085	*NA*	*NA*	CEBPZ	CEBPZ
MESP1_HUMAN.H11MO.0.D	31	0.18128655	0.5029670	11.405.420	0.7683339	199079,80000000	0.4347209456	Tal-related factors{1.2.3}	Mesp-like factors{1.2.3.3}	ATOH1;ATOH7;ATOH8;BHLHA15;BHLHA9;BHLHE22;BHLHE23;FERD3L;FIGLA;HAND1;HAND2;LYL1;MESP1;MESP2;MSC;MSGN1;NEUROD1;NEUROD2;NEUROD4;NEUROD6;NEUROG1;NEUROG2;NEUROG3;NHLH1;NHLH2;OLIG1;OLIG2;OLIG3;PTF1A;SCX;TAL1;TAL2;TCF15;TCF21;TCF23;TWIST1;TWIST2	MESP1
DLX1_HUMAN.H11MO.0.D	11	0.06432749	0.5023253	18.885.291	1,03E+07	874434,40000000	0.9529396584	NK-related factors{3.1.2}	DLX{3.1.2.5}	BARHL1;BARHL2;BARX1;BARX2;BSX;DBX1;DBX2;DLX1;DLX2;DLX3;DLX4;DLX5;DLX6;EMX1;EMX2;EN1;EN2;HHEX;HLX;HMX1;HMX2;HMX3;LBX1;LBX2;MSX1;MSX2;NANOG;NKX1-1;NKX1-2;NKX2-1;NKX2-2;NKX2-3;NKX2-4;NKX2-5;NKX2-6;NKX2-8;NKX3-1;NKX3-2;NKX6-1;NKX6-2;NKX6-3;NOTO;TLX1;TLX2;TLX3;VAX1;VAX2;VENTX	DLX1;DLX2;DLX3;DLX4;DLX5;DLX6
CUX1_HUMAN.H11MO.0.C	9	0.05263158	0.5013167	21.743.865	1,12E+07	716924,30000000	0.8746378511	HD-CUT factors{3.1.9}	CUX{3.1.9.2}	CUX1;CUX2;ONECUT1;ONECUT2;ONECUT3;SATB1;SATB2	CUX1;CUX2
SOX5_HUMAN.H11MO.0.C	9	0.05263158	0.5012311	21.740.145	1,12E+07	716953,50000000	0.8746378511	SOX-related factors{4.1.1}	Group D{4.1.1.4}	BBX;CIC;HBP1;SOX1;SOX10;SOX11;SOX12;SOX13;SOX14;SOX15;SOX17;SOX18;SOX2;SOX21;SOX3;SOX30;SOX4;SOX5;SOX6;SOX7;SOX8;SOX9;SRY	SOX13;SOX5
KLF6_HUMAN.H11MO.0.A	83	0.48538012	0.5010212	0.9350982	0.6847373	16020,82000000	0.0781775497	Three-zinc finger Krüppel-related factors{2.3.1}	Krüppel-like factors{2.3.1.2}	EGR1;EGR2;EGR3;EGR4;KLF1;KLF10;KLF11;KLF12;KLF13;KLF14;KLF15;KLF16;KLF17;KLF2;KLF3;KLF4;KLF5;KLF6;KLF7;KLF8;KLF9;SP1;SP2;SP3;SP4;SP5;SP6;SP7;SP8;SP9	KLF12;KLF13;KLF14;KLF15;KLF16;KLF1;KLF3;KLF4;KLF5;KLF6;KLF8;KLF9

motif	Nº of probes	% of probes	lower OR	upper OR	OR	p.value	FDR	TF family	TF subfamily	TF.family.member	TF.subfamily.member
ZN816_HUMAN.H11MO.1.C	21	0.12280702	0.5004778	13.196.620	0.8324550	512712,80000000	0.7306867825	More than 3 adjacent zinc finger factors{2.3.3}	ZNF816A-like factors{2.3.3.73}	BCL6;BCL6B;CTCF;CTCFL;FEZF1;FEZF2;GFI1;GFI1B;GLI1;GLI2;GLI3;GLI4;GLIS1;GLIS2;GLIS3;HKR1;MTF1;MYNN;MZF1;OSR2;OVOL1;OVOL2;PLAG1;PLAGL1;PLAGL2;PRDM1;PRDM14;PRDM6;SCRT1;SCRT2;SNAI1;SNAI2;SNAI3;WT1;YY1;YY2;ZBTB12;ZBTB14;ZBTB18;ZBTB20;ZBTB26;ZBTB42;ZBTB45;ZBTB47;ZBTB48;ZBTB49;ZBTB6;ZBTB7A;ZBTB7B;ZBTB7C;ZFP14;ZFP2;ZFP28;ZFP30;ZFP37;ZFP42;ZFP64;ZFP69;ZFP69B;ZFP82;ZFP91;ZFX;ZIC1;ZIC2;ZIC3;ZIC4;ZIC5;ZIK1;ZIM3;ZKSCAN1;ZKSCAN2;ZKSCAN3;ZKSCAN4;ZNF121;ZNF124;ZNF133;ZNF136;ZNF138;ZNF14;ZNF140;ZNF143;ZNF146;ZNF148;ZNF155;ZNF157;ZNF160;ZNF169;ZNF175;ZNF177;ZNF18;ZNF180;ZNF181;ZNF2;ZNF20;ZNF212;ZNF213;ZNF214;ZNF221;ZNF222;ZNF223;ZNF224;ZNF225;ZNF226;ZNF227;ZNF229;ZNF230;ZNF232;ZNF233;ZNF234;ZNF235;ZNF24;ZNF25;ZNF250;ZNF257;ZNF26;ZNF260;ZNF263;ZNF264;ZNF268;ZNF274;ZNF276;ZNF28;ZNF280A;ZNF280B;ZNF280C;ZNF280D;ZNF281;ZNF282;ZNF283;ZNF284;ZNF285;ZNF286A;ZNF286B;ZNF3;ZNF30;ZNF300;ZNF302;ZNF317;ZNF32;ZNF320;ZNF322;ZNF324;ZNF324B;ZNF329;ZNF331;ZNF333;ZNF33A;ZNF33B;ZNF343;ZNF345;ZNF347;ZNF350;ZNF354A;ZNF354B;ZNF362;ZNF366;ZNF383;ZNF384;ZNF394;ZNF397;ZNF398;ZNF404;ZNF41;ZNF410;ZNF419;ZNF420;ZNF431;ZNF432;ZNF436;ZNF439;ZNF44;ZNF440;ZNF442;ZNF443;ZNF446;ZNF449;ZNF45;ZNF460;ZNF468;ZNF479;ZNF484;ZNF490;ZNF500;ZNF502;ZNF524;ZNF525;ZNF528;ZNF543;ZNF544;ZNF546;ZNF547;ZNF548;ZNF549;ZNF554;ZNF555;ZNF557;ZNF558;ZNF559;ZNF561;ZNF562;ZNF563;ZNF564;ZNF566;ZNF567;ZNF568;ZNF57;ZNF570;ZNF571;ZNF572;ZNF577;ZNF581;ZNF582;ZNF583;ZNF585A;ZNF586;ZNF589;ZNF595;ZNF599;ZNF600;ZNF605;ZNF607;ZNF611;ZNF613;ZNF614;ZNF615;ZNF616;ZNF619;ZNF620;ZNF621;ZNF625;ZNF627;ZNF649;ZNF652;ZNF653;ZNF665;ZNF667;ZNF669;ZNF670;ZNF672;ZNF679;ZNF680;ZNF683;ZNF689;ZNF692;ZNF701;ZNF705D;ZNF705E;ZNF705G;ZNF708;ZNF709;ZNF71;ZNF710;ZNF713;ZNF721;ZNF727;ZNF729;ZNF736;ZNF75A;ZNF75D;ZNF76;ZNF763;ZNF764;ZNF765;ZNF768;ZNF77;ZNF771;ZNF773;ZNF774;ZNF776;ZNF777;ZNF780A;ZNF780B;ZNF782;ZNF785;ZNF799;ZNF805;ZNF808;ZNF81;ZNF813;ZNF816;ZNF823;ZNF829;ZNF836;ZNF841;ZNF844;ZNF845;ZNF846;ZNF85;ZNF853;ZNF860;ZNF878;ZNF891;ZNF99;ZSCAN16;ZSCAN2;ZSCAN22;ZSCAN23;ZSCAN29;ZSCAN31;ZSCAN32;ZSCAN4;ZSCAN5A;ZSCAN5B;ZSCAN5C;ZSCAN9;ZXDA;ZXDB;ZXDC	ZNF816
ERR3_HUMAN.H11MO.0.B	11	0.06432749	0.4989912	18.759.051	1,02E+07	875073,00000000	0.9529396584	Steroid hormone receptors (NR3){2.1.1}	ER-like receptors (NR3A&B){2.1.1.2}	AR;ESR1;ESR2;ESRRA;ESRRB;ESRRG;NR3C1;NR3C2;PGR	ESRRA;ESRRB;ESRRG;ESR1;ESR2
VEZF1_HUMAN.H11MO.0.C	73	0.42690058	0.4988502	0.9373204	0.6851035	14263,16000000	0.0728055818	Factors with multiple dispersed zinc fingers{2.3.4}	MAZ-like factors{2.3.4.8}	BCL11A;BCL11B;BNC1;BNC2;E4F1;HIC1;HIC2;HINFP;HIVEP1;HIVEP2;HIVEP3;IKZF1;IKZF2;IKZF3;IKZF4;IKZF5;INSM1;INSM2;MAZ;MECOM;PATZ1;PRDM16;PRDM4;REST;RLF;RREB1;SALL1;SALL2;SALL3;SALL4;VEZF1;ZBTB1;ZBTB17;ZBTB2;ZBTB25;ZBTB4;ZFAT;ZNF134;ZNF211;ZNF217;ZNF219;ZNF248;ZNF256;ZNF292;ZNF296;ZNF319;ZNF334;ZNF335;ZNF341;ZN	MAZ;PATZ1;VEZF1

motif	Nº of probes	% of probes	lower OR	upper OR	OR	p.value	FDR	TF family	TF subfamily	TF.family.member	TF.subfamily.member
										F37A;ZNF382;ZNF417;ZNF418;ZNF423;ZNF467;ZNF510;ZNF512;ZNF512B;ZNF516;ZNF518A;ZNF518B;ZNF521;ZNF526;ZNF532;ZNF536;ZNF552;ZNF574;ZNF587;ZNF587B;ZNF592;ZNF639;ZNF654;ZNF658;ZNF671;ZNF687;ZNF711;ZNF717;ZNF770;ZNF772;ZNF784;ZNF786;ZNF792;ZNF8;ZNF814	
HSF1_HUMAN.H11MO.0.A	15	0.08771930	0.4988058	15.532.347	0.9129071	896029,90000000	0.9612402184	HSF factors{3.4.1}	HSF1 (HSTF1){3.4.1.0.1}	HSF1;HSF2;HSF4;HSF5;HSFX1;HSFY1;HSFY1;HSFY2	HSF1
LHX2_HUMAN.H11MO.0.A	9	0.05263158	0.4965721	21.537.355	1,11E+07	718637,70000000	0.8753075332	HD-LIM factors{3.1.5}	Lhx-2-like factors{3.1.5.3}	ISL1;ISL2;LHX1;LHX2;LHX3;LHX4;LHX5;LHX6;LHX8;LHX9;LMX1A;LMX1B	LHX2;LHX9
ZN219_HUMAN.H11MO.0.D	46	0.26900585	0.4962736	10.060.318	0.7120100	52407,34000000	0.1830046458	Factors with multiple dispersed zinc fingers{2.3.4}	ZNF219-like factors{2.3.4.2}	BCL11A;BCL11B;BNC1;BNC2;E4F1;HIC1;HIC2;HINFP;HIVEP1;HIVEP2;HIVEP3;IKZF1;IKZF2;IKZF3;IKZF4;IKZF5;INSM1;INSM2;MAZ;MECOM;PATZ1;PRDM16;PRDM4;REST;RLF;RREB1;SALL1;SALL2;SALL3;SALL4;VEZF1;ZBTB1;ZBTB17;ZBTB2;ZBTB25;ZBTB4;ZFAT;ZNF134;ZNF211;ZNF217;ZNF219;ZNF248;ZNF256;ZNF292;ZNF296;ZNF319;ZNF334;ZNF335;ZNF341;ZNF37A;ZNF382;ZNF417;ZNF418;ZNF423;ZNF467;ZNF510;ZNF512;ZNF512B;ZNF516;ZNF518A;ZNF518B;ZNF521;ZNF526;ZNF532;ZNF536;ZNF552;ZNF574;ZNF587;ZNF587B;ZNF592;ZNF639;ZNF654;ZNF658;ZNF671;ZNF687;ZNF711;ZNF717;ZNF770;ZNF772;ZNF784;ZNF786;ZNF792;ZNF8;ZNF814	ZNF219
CR3L1_HUMAN.H11MO.0.D	52	0.30409357	0.4956285	0.9784823	0.7006128	33493,44000000	0.1310834448	CREB-related factors{1.1.7}	CREB-3-like factors{1.1.7.2}	ATF1;ATF6;ATF6B;CREB1;CREB3;CREB3L1;CREB3L2;CREB3L3;CREB3L4;CREBL2;CREBZF;CREM	CREB3L1;CREB3L2;CREB3
NR2C1_HUMAN.H11MO.0.C	20	0.11695906	0.4955286	13.355.986	0.8346147	505046,60000000	0.7210942868	RXR-related receptors (NR2){2.1.3}	Testicular receptors (NR2C){2.1.3.4}	HNF4A;HNF4G;NR2C1;NR2C2;NR2E1;NR2E3;NR2F1;NR2F2;NR2F6;RXRA;RXRB;RXRG	NR2C1;NR2C2
HXB6_HUMAN.H11MO.0.D	8	0.04678363	0.4954846	23.574.655	1,17E+07	562464,80000000	0.7730131813	HOX-related factors{3.1.1}	HOX6-7{3.1.1.6}	CDX1;CDX2;CDX4;EVX1;EVX2;GBX1;GBX2;GSX1;GSX2;HDX;HMBOX1;HNF1A;HNF1B;HOXA1;HOXA10;HOXA11;HOXA13;HOXA2;HOXA3;HOXA4;HOXA5;HOXA6;HOXA7;HOXA9;HOXB1;HOXB13;HOXB2;HOXB3;HOXB4;HOXB5;HOXB6;HOXB7;HOXB8;HOXB9;HOXC10;HOXC11;HOXC12;HOXC13;HOXC4;HOXC5;HOXC6;HOXC8;HOXC9;HOXD1;HOXD10;HOXD11;HOXD12;HOXD13;HOXD3;HOXD4;HOXD8;HOXD9;MEOX1;MEOX2;MNX1;PDX1;POU1F1;POU2F1;POU2F2;POU2F3;POU3F1;POU3F2;POU3F3;POU3F4;POU4F1;POU4F2;POU4F3;POU5F1;POU5F2;POU6F1;POU6F2	HOXA7;HOXB6;HOXB7;HOXC6
PATZ1_HUMAN.H11MO.1.C	45	0.26315789	0.4945496	10.078.353	0.7116624	51478,70000000	0.1812332257	Factors with multiple dispersed zinc fingers{2.3.4}	MAZ-like factors{2.3.4.8}	BCL11A;BCL11B;BNC1;BNC2;E4F1;HIC1;HIC2;HINFP;HIVEP1;HIVEP2;HIVEP3;IKZF1;IKZF2;IKZF3;IKZF4;IKZF5;INSM1;INSM2;MAZ;MECOM;PATZ1;PRDM16;PRDM4;REST;RLF;RREB1;SALL1;SALL2;SALL3;SALL4;VEZF1;ZBTB1;ZBTB17;ZBTB2;ZBTB25;ZBTB4;ZFAT;ZNF134;ZNF211;ZNF217;ZNF219;ZNF248;ZNF256;ZNF292;ZNF296;ZNF319;ZNF334;ZNF335;ZNF341;ZNF37A;ZNF382;ZNF417;ZNF418;ZNF423;ZNF467;ZNF510;ZNF512;ZNF512B;ZNF516;ZNF518A;ZNF518B;ZNF521;ZNF526;ZNF532;ZNF536;ZNF552;ZNF574;ZNF587;ZNF587B;ZNF592;ZNF639;ZNF654;ZNF658;ZNF671;ZNF687;ZNF711;ZNF717;ZNF770;ZNF772;ZNF784;ZNF786;ZNF792;ZNF8;ZNF814	MAZ;PATZ1;VEZF1

motif	Nº of probes	% of probes	lower OR	upper OR	OR	p.value	FDR	TF family	TF subfamily	TF.family.member	TF.subfamily.member
ZN554_HUMAN.H11MO.1.D	34	0.19883041	0.4933150	10.857.895	0.7410998	133082,50000000	0.3342235031	More than 3 adjacent zinc finger factors{2.3.3}	unclassified{2.3.3.0}	BCL6B;BCL6;CTCFL;CTCF;FEZF1;GFI1B;GFI1;GLI1;GLI2;GLI3;GLIS1;GLIS2;GLIS3;MTF1;MYNN;MZF1;OSR2;OVOL1;OVOL2;ZNF146;PLAG1;PLAGL1;PRDM14;PRDM1;PRDM6;SCRT1;SCRT2;SNAI1;SNAI2;YY1;YY2;WT1;ZNF324;ZNF354A;ZBTB14;ZBTB18;ZBTB48;ZBTB49;ZBTB7A;ZBTB7B;ZBTB6;ZFP64;ZFP28;ZFP42;ZFP82;ZFX;ZIC1;ZIC2;ZIC3;ZIC4;ZIM3;ZKSCAN1;ZKSCAN3;ZNF121;ZNF136;ZNF140;ZNF143;ZNF148;ZNF214;ZNF232;ZNF250;ZNF257;ZNF260;ZNF263;ZNF264;ZNF274;ZNF281;ZNF282;ZNF317;ZNF320;ZNF322;ZNF329;ZNF331;ZNF333;ZNF350;ZNF384;ZNF394;ZNF410;ZNF436;ZNF449;ZNF490;ZNF502;ZNF524;ZNF528;ZNF547;ZNF549;ZNF554;ZNF563;ZNF582;ZNF586;ZNF589;ZNF652;ZNF667;ZNF680;ZNF708;ZNF713;ZNF768;ZNF816;ZNF18;ZNF41;ZNF76;ZNF85;ZSCAN16;ZSCAN22;ZSCAN31;ZSCAN4	MYNN;MZF1;OSR2;PRDM14;PRDM6;WT1;ZBTB14;ZBTB48;ZBTB49;ZFP64;ZFP28;ZIM3;ZNF121;ZNF250;ZNF257;ZNF263;ZNF274;ZNF317;ZNF320;ZNF329;ZNF331;ZNF394;ZNF449;ZNF502;ZNF528;ZNF547;ZNF549;ZNF554;ZNF586;ZNF589;ZNF667;ZNF680;ZNF708;ZNF713;ZNF768;ZNF18;ZNF85;ZSCAN16;ZSCAN22
ZN449_HUMAN.H11MO.0.C	24	0.14035088	0.4928209	12.282.016	0.7939341	359161,40000000	0.6092079825	More than 3 adjacent zinc finger factors{2.3.3}	unclassified{2.3.3.0}	BCL6B;BCL6;CTCFL;CTCF;FEZF1;GFI1B;GFI1;GLI1;GLI2;GLI3;GLIS1;GLIS2;GLIS3;MTF1;MYNN;MZF1;OSR2;OVOL1;OVOL2;ZNF146;PLAG1;PLAGL1;PRDM14;PRDM1;PRDM6;SCRT1;SCRT2;SNAI1;SNAI2;YY1;YY2;WT1;ZNF324;ZNF354A;ZBTB14;ZBTB18;ZBTB48;ZBTB49;ZBTB7A;ZBTB7B;ZBTB6;ZFP64;ZFP28;ZFP42;ZFP82;ZFX;ZIC1;ZIC2;ZIC3;ZIC4;ZIM3;ZKSCAN1;ZKSCAN3;ZNF121;ZNF136;ZNF140;ZNF143;ZNF148;ZNF214;ZNF232;ZNF250;ZNF257;ZNF260;ZNF263;ZNF264;ZNF274;ZNF281;ZNF282;ZNF317;ZNF320;ZNF322;ZNF329;ZNF331;ZNF333;ZNF350;ZNF384;ZNF394;ZNF410;ZNF436;ZNF449;ZNF490;ZNF502;ZNF524;ZNF528;ZNF547;ZNF549;ZNF554;ZNF563;ZNF582;ZNF586;ZNF589;ZNF652;ZNF667;ZNF680;ZNF708;ZNF713;ZNF768;ZNF816;ZNF18;ZNF41;ZNF76;ZNF85;ZSCAN16;ZSCAN22;ZSCAN31;ZSCAN4	MYNN;MZF1;OSR2;PRDM14;PRDM6;WT1;ZBTB14;ZBTB48;ZBTB49;ZFP64;ZFP28;ZIM3;ZNF121;ZNF250;ZNF257;ZNF263;ZNF274;ZNF317;ZNF320;ZNF329;ZNF331;ZNF394;ZNF449;ZNF502;ZNF528;ZNF547;ZNF549;ZNF554;ZNF586;ZNF589;ZNF667;ZNF680;ZNF708;ZNF713;ZNF768;ZNF18;ZNF85;ZSCAN16;ZSCAN22
DLX6_HUMAN.H11MO.0.D	10	0.05847953	0.4924749	19.771.437	1,05E+07	867065,40000000	0.9468943764	NK-related factors{3.1.2}	DLX{3.1.2.5}	BARHL1;BARHL2;BARX1;BARX2;BSX;DBX1;DBX2;DLX1;DLX2;DLX3;DLX4;DLX5;DLX6;EMX1;EMX2;EN1;EN2;HHEX;HLX;HMX1;HMX2;HMX3;LBX1;LBX2;MSX1;MSX2;NANOG;NKX1-1;NKX1-2;NKX2-1;NKX2-2;NKX2-3;NKX2-4;NKX2-5;NKX2-6;NKX2-8;NKX3-1;NKX3-2;NKX6-1;NKX6-2;NKX6-3;NOTO;TLX1;TLX2;TLX3;VAX1;VAX2;VENTX	DLX1;DLX2;DLX3;DLX4;DLX5;DLX6
ATOH1_HUMAN.H11MO.0.B	26	0.15204678	0.4909279	11.856.035	0.7769406	280283,80000000	0.5132987807	Tal-related factors{1.2.3}	Neurogenin / Atonal-like factors{1.2.3.4}	ATOH1;ATOH7;ATOH8;BHLHA15;BHLHA9;BHLHE22;BHLHE23;FERD3L;FIGLA;HAND1;HAND2;LYL1;MESP1;MESP2;MSC;MSGN1;NEUROD1;NEUROD2;NEUROD4;NEUROD6;NEUROG1;NEUROG2;NEUROG3;NHLH1;NHLH2;OLIG1;OLIG2;OLIG3;PTF1A;SCX;TAL1;TAL2;TCF15;TCF21;TCF23;TWIST1;TWIST2	ATOH1;BHLHA15;BHLHE22;BHLHE23;NEUROD1;NEUROD2;NEUROG2;OLIG1;OLIG2;OLIG3
ZN554_HUMAN.H11MO.0.C	30	0.17543860	0.4896692	11.228.799	0.7527383	166774,20000000	0.3884681142	More than 3 adjacent zinc finger factors{2.3.3}	unclassified{2.3.3.0}	BCL6B;BCL6;CTCFL;CTCF;FEZF1;GFI1B;GFI1;GLI1;GLI2;GLI3;GLIS1;GLIS2;GLIS3;MTF1;MYNN;MZF1;OSR2;OVOL1;OVOL2;ZNF146;PLAG1;PLAGL1;PRDM14;PRDM1;PRDM6;SCRT1;SCRT2;SNAI1;SNAI2;YY1;YY2;WT1;ZNF324;ZNF354A;ZBTB14;ZBTB18;ZBTB48;ZBTB49;ZBTB7A;ZBTB7B;ZBTB6;ZFP64;ZFP28;ZFP42;ZFP82;ZFX;ZIC1;ZIC2;ZIC3;ZIC4;ZIM3;ZKSCAN1;ZKSCAN3;ZNF121;ZNF136;ZNF140;ZNF143;ZNF148;ZNF214;ZNF232;ZNF250;ZNF257;ZNF260;ZNF263;ZNF264;ZNF274;ZNF281;ZNF282;ZNF317;ZNF320;ZNF322;ZNF329;ZNF331;ZNF333;ZNF350;ZNF384;ZNF394;ZNF410;ZNF436;ZNF449;ZNF490;ZNF502;ZNF524;ZNF528;ZNF547;ZNF5	MYNN;MZF1;OSR2;PRDM14;PRDM6;WT1;ZBTB14;ZBTB48;ZBTB49;ZFP64;ZFP28;ZIM3;ZNF121;ZNF250;ZNF257;ZNF263;ZNF274;ZNF317;ZNF320;ZNF329;ZNF331;ZNF394;ZNF449;ZNF502;ZNF528;ZNF547;ZNF549;ZNF554;ZNF586;ZNF589;ZNF667;ZNF680;ZNF708;ZNF713;ZNF768;ZNF18;ZNF85;ZSCAN16;ZSCAN22

motif	Nº of probes	% of probes	lower OR	upper OR	OR	p.value	FDR	TF family	TF subfamily	TF.family.member	TF.subfamily.member
										49;ZNF554;ZNF563;ZNF582;ZNF586;ZNF589;ZNF652;ZNF667;ZNF680;ZNF708;ZNF713;ZNF768;ZNF816;ZNF18;ZNF41;ZNF76;ZNF85;ZSCAN16;ZSCAN22;ZSCAN31;ZSCAN4	
WT1_HUMAN.H11MO.1.B	45	0.26315789	0.4894517	0.9974143	0.7043050	43002,34000000	0.1598742469	More than 3 adjacent zinc finger factors{2.3.3}	unclassified{2.3.3.0}	BCL6B;BCL6;CTCFL;CTCF;FEZF1;GFI1B;GFI1;GLI1;GLI2;GLI3;GLIS1;GLIS2;GLIS3;MTF1;MYNN;MZF1;OSR2;OVOL1;OVOL2;ZNF146;PLAG1;PLAGL1;PRDM14;PRDM1;PRDM6;SCRT1;SCRT2;SNAI1;SNAI2;YY1;YY2;WT1;ZNF324;ZNF354A;ZBTB14;ZBTB18;ZBTB48;ZBTB49;ZBTB7A;ZBTB7B;ZBTB6;ZFP64;ZFP28;ZFP42;ZFP82;ZFX;ZIC1;ZIC2;ZIC3;ZIC4;ZIM3;ZKSCAN1;ZKSCAN3;ZNF121;ZNF136;ZNF140;ZNF143;ZNF148;ZNF214;ZNF232;ZNF250;ZNF257;ZNF260;ZNF263;ZNF264;ZNF274;ZNF281;ZNF282;ZNF317;ZNF320;ZNF322;ZNF329;ZNF331;ZNF333;ZNF350;ZNF384;ZNF394;ZNF410;ZNF436;ZNF449;ZNF490;ZNF502;ZNF524;ZNF528;ZNF547;ZNF549;ZNF554;ZNF563;ZNF582;ZNF586;ZNF589;ZNF652;ZNF667;ZNF680;ZNF708;ZNF713;ZNF768;ZNF816;ZNF18;ZNF41;ZNF76;ZNF85;ZSCAN16;ZSCAN22;ZSCAN31;ZSCAN4	MYNN;MZF1;OSR2;PRDM14;PRDM6;WT1;ZBTB14;ZBTB48;ZBTB49;ZFP64;ZFP28;ZIM3;ZNF121;ZNF250;ZNF257;ZNF263;ZNF274;ZNF317;ZNF320;ZNF329;ZNF331;ZNF394;ZNF449;ZNF502;ZNF528;ZNF547;ZNF549;ZNF554;ZNF586;ZNF589;ZNF667;ZNF680;ZNF708;ZNF713;ZNF768;ZNF18;ZNF85;ZSCAN16;ZSCAN22
ZN317_HUMAN.H11MO.0.C	11	0.06432749	0.4892600	18.393.575	1,00E+07	1000000,00000000	1,00E+10	More than 3 adjacent zinc finger factors{2.3.3}	unclassified{2.3.3.0}	BCL6B;BCL6;CTCFL;CTCF;FEZF1;GFI1B;GFI1;GLI1;GLI2;GLI3;GLIS1;GLIS2;GLIS3;MTF1;MYNN;MZF1;OSR2;OVOL1;OVOL2;ZNF146;PLAG1;PLAGL1;PRDM14;PRDM1;PRDM6;SCRT1;SCRT2;SNAI1;SNAI2;YY1;YY2;WT1;ZNF324;ZNF354A;ZBTB14;ZBTB18;ZBTB48;ZBTB49;ZBTB7A;ZBTB7B;ZBTB6;ZFP64;ZFP28;ZFP42;ZFP82;ZFX;ZIC1;ZIC2;ZIC3;ZIC4;ZIM3;ZKSCAN1;ZKSCAN3;ZNF121;ZNF136;ZNF140;ZNF143;ZNF148;ZNF214;ZNF232;ZNF250;ZNF257;ZNF260;ZNF263;ZNF264;ZNF274;ZNF281;ZNF282;ZNF317;ZNF320;ZNF322;ZNF329;ZNF331;ZNF333;ZNF350;ZNF384;ZNF394;ZNF410;ZNF436;ZNF449;ZNF490;ZNF502;ZNF524;ZNF528;ZNF547;ZNF549;ZNF554;ZNF563;ZNF582;ZNF586;ZNF589;ZNF652;ZNF667;ZNF680;ZNF708;ZNF713;ZNF768;ZNF816;ZNF18;ZNF41;ZNF76;ZNF85;ZSCAN16;ZSCAN22;ZSCAN31;ZSCAN4	MYNN;MZF1;OSR2;PRDM14;PRDM6;WT1;ZBTB14;ZBTB48;ZBTB49;ZFP64;ZFP28;ZIM3;ZNF121;ZNF250;ZNF257;ZNF263;ZNF274;ZNF317;ZNF320;ZNF329;ZNF331;ZNF394;ZNF449;ZNF502;ZNF528;ZNF547;ZNF549;ZNF554;ZNF586;ZNF589;ZNF667;ZNF680;ZNF708;ZNF713;ZNF768;ZNF18;ZNF85;ZSCAN16;ZSCAN22
MEIS1_HUMAN.H11MO.0.A	9	0.05263158	0.4885143	21.188.182	1,09E+07	721989,90000000	0.8766208224	TALE-type homeo domain factors{3.1.4}	MEIS{3.1.4.2}	IRX1;IRX2;IRX3;IRX4;IRX5;IRX6;MEIS1;MEIS2;MEIS3;MKX;PBX1;PBX2;PBX3;PKNOX1;PKNOX2;TGIF1;TGIF2;TGIF2LX;TGIF2LY	MEIS1;MEIS2;MEIS3
TLX1_HUMAN.H11MO.0.D	16	0.09356725	0.4878896	14.674.486	0.8751540	708857,50000000	0.8746378511	NK-related factors{3.1.2}	TLX{3.1.2.21}	BARHL1;BARHL2;BARX1;BARX2;BSX;DBX1;DBX2;DLX1;DLX2;DLX3;DLX4;DLX5;DLX6;EMX1;EMX2;EN1;EN2;HHEX;HLX;HMX1;HMX2;HMX3;LBX1;LBX2;MSX1;MSX2;NANOG;NKX1-1;NKX1-2;NKX2-1;NKX2-2;NKX2-3;NKX2-4;NKX2-5;NKX2-6;NKX2-8;NKX3-1;NKX3-2;NKX6-1;NKX6-2;NKX6-3;NOTO;TLX1;TLX2;TLX3;VAX1;VAX2;VENTX	TLX1
COT1_HUMAN.H11MO.0.C	40	0.23391813	0.4872396	10.230.818	0.7129398	65875,11000000	0.2064622393	RXR-related receptors (NR2){2.1.3}	COUP-like receptors (NR2F){2.1.3.5}	HNF4A;HNF4G;NR2C1;NR2C2;NR2E1;NR2E3;NR2F1;NR2F2;NR2F6;RXRA;RXRB;RXRG	NR2F1;NR2F2;NR2F6

motif	Nº of probes	% of probes	lower OR	upper OR	OR	p.value	FDR	TF family	TF subfamily	TF.family.member	TF.subfamily.member
E2F8_HUMAN.H11MO.0.D	13	0.07602339	0.4868763	16.463.431	0.9349883	1000000,00000000	1,00E+10	E2F-related factors{3.3.2}	E2F{3.3.2.1}	E2F1;E2F2;E2F3;E2F4;E2F5;E2F6;E2F7;E2F8;TFDP1;TFDP2	E2F1;E2F2;E2F3;E2F4;E2F5;E2F6;E2F7;E2F8
ITF2_HUMAN.H11MO.0.C	39	0.22807018	0.4850560	10.254.620	0.7124870	64414,14000000	0.2055702335	E2A-related factors{1.2.1}	SEF2 (E2-2, TCF-4, ITF-2){1.2.1.0.2}	TCF12;TCF4;TCF3	TCF4
KLF5_HUMAN.H11MO.0.A	59	0.34502924	0.4849768	0.9357553	0.6766326	16565,39000000	0.0785016722	Three-zinc finger Krüppel-related factors{2.3.1}	Krüppel-like factors{2.3.1.2}	EGR1;EGR2;EGR3;EGR4;KLF1;KLF10;KLF11;KLF12;KLF13;KLF14;KLF15;KLF16;KLF17;KLF2;KLF3;KLF4;KLF5;KLF6;KLF7;KLF8;KLF9;SP1;SP2;SP3;SP4;SP5;SP6;SP7;SP8;SP9	KLF12;KLF13;KLF14;KLF15;KLF16;KLF1;KLF3;KLF4;KLF5;KLF6;KLF8;KLF9
NKX21_HUMAN.H11MO.0.A	17	0.09941520	0.4847335	14.128.177	0.8538109	630645,60000000	0.8171895349	NK-related factors{3.1.2}	NK-2.1{3.1.2.14}	BARHL1;BARHL2;BARX1;BARX2;BSX;DBX1;DBX2;DLX1;DLX2;DLX3;DLX4;DLX5;DLX6;EMX1;EMX2;EN1;EN2;HHEX;HLX;HMX1;HMX2;HMX3;LBX1;LBX2;MSX1;MSX2;NANOG;NKX1-1;NKX1-2;NKX2-1;NKX2-2;NKX2-3;NKX2-4;NKX2-5;NKX2-6;NKX2-8;NKX3-1;NKX3-2;NKX6-1;NKX6-2;NKX6-3;NOTO;TLX1;TLX2;TLX3;VAX1;VAX2;VENTX	NKX2-1
KLF8_HUMAN.H11MO.0.C	32	0.18713450	0.4846650	10.876.415	0.7360645	126803,70000000	0.3239806755	Three-zinc finger Krüppel-related factors{2.3.1}	Krüppel-like factors{2.3.1.2}	EGR1;EGR2;EGR3;EGR4;KLF1;KLF10;KLF11;KLF12;KLF13;KLF14;KLF15;KLF16;KLF17;KLF2;KLF3;KLF4;KLF5;KLF6;KLF7;KLF8;KLF9;SP1;SP2;SP3;SP4;SP5;SP6;SP7;SP8;SP9	KLF12;KLF13;KLF14;KLF15;KLF16;KLF1;KLF3;KLF4;KLF5;KLF6;KLF8;KLF9
ID4_HUMAN.H11MO.0.D	27	0.15789474	0.4843392	11.532.635	0.7604339	211897,60000000	0.4427452981	HLH domain only{1.2.8}	Id4{1.2.8.0.4}	ID4	ID4
EGR3_HUMAN.H11MO.0.D	25	0.14619883	0.4837544	11.861.937	0.7722043	274251,90000000	0.5034606818	Three-zinc finger Krüppel-related factors{2.3.1}	EGR factors{2.3.1.3}	EGR1;EGR2;EGR3;EGR4;KLF1;KLF10;KLF11;KLF12;KLF13;KLF14;KLF15;KLF16;KLF17;KLF2;KLF3;KLF4;KLF5;KLF6;KLF7;KLF8;KLF9;SP1;SP2;SP3;SP4;SP5;SP6;SP7;SP8;SP9	EGR1;EGR2;EGR3;EGR4
RARG_HUMAN.H11MO.2.D	24	0.14035088	0.4836637	12.053.860	0.7791729	311245,70000000	0.5542042813	Thyroid hormone receptor-related factors (NR1){2.1.2}	Retinoic acid receptors (NR1B){2.1.2.1}	NR1D1;NR1D2;NR1H2;NR1H3;NR1H4;NR1I2;NR1I3;PPARA;PPARD;PPARG;RARA;RARB;RARG;RORA;RORB;RORC;THRA;THRB;VDR	RARA;RARB;RARG
KLF15_HUMAN.H11MO.0.A	81	0.47368421	0.4833364	0.9026629	0.6609819	8274,93800000	0.0476117714	Three-zinc finger Krüppel-related factors{2.3.1}	Krüppel-like factors{2.3.1.2}	EGR1;EGR2;EGR3;EGR4;KLF1;KLF10;KLF11;KLF12;KLF13;KLF14;KLF15;KLF16;KLF17;KLF2;KLF3;KLF4;KLF5;KLF6;KLF7;KLF8;KLF9;SP1;SP2;SP3;SP4;SP5;SP6;SP7;SP8;SP9	KLF12;KLF13;KLF14;KLF15;KLF16;KLF1;KLF3;KLF4;KLF5;KLF6;KLF8;KLF9
NR2F6_HUMAN.H11MO.0.D	13	0.07602339	0.4822059	16.305.740	0.9260356	889327,50000000	0.9598074264	RXR-related receptors (NR2){2.1.3}	COUP-like receptors (NR2F){2.1.3.5}	HNF4A;HNF4G;NR2C1;NR2C2;NR2E1;NR2E3;NR2F1;NR2F2;NR2F6;RXRA;RXRB;RXRG	NR2F1;NR2F2;NR2F6

motif	Nº of probes	% of probes	lower OR	upper OR	OR	p.value	FDR	TF family	TF subfamily	TF.family.member	TF.subfamily.member
FEZF1_HUMAN.H11MO.0.C	13	0.07602339	0.4817078	16.288.949	0.9250823	889322,90000000	0.9598074264	More than 3 adjacent zinc finger factors{2.3.3}	FEZF factors{2.3.3.20}	BCL6;BCL6B;CTCF;CTCFL;FEZF1;FEZF2;GFI1;GFI1B;GLI1;GLI2;GLI3;GLI4;GLIS1;GLIS2;GLIS3;HKR1;MTF1;MYNN;MZF1;OSR2;OVOL1;OVOL2;PLAG1;PLAGL1;PLAGL2;PRDM1;PRDM14;PRDM6;SCRT1;SCRT2;SNAI1;SNAI2;SNAI3;WT1;YY1;YY2;ZBTB12;ZBTB14;ZBTB18;ZBTB20;ZBTB26;ZBTB42;ZBTB45;ZBTB47;ZBTB48;ZBTB49;ZBTB6;ZBTB7A;ZBTB7B;ZBTB7C;ZFP14;ZFP2;ZFP28;ZFP30;ZFP37;ZFP42;ZFP64;ZFP69;ZFP69B;ZFP82;ZFP91;ZFX;ZIC1;ZIC2;ZIC3;ZIC4;ZIC5;ZIK1;ZIM3;ZKSCAN1;ZKSCAN2;ZKSCAN3;ZKSCAN4;ZNF121;ZNF124;ZNF133;ZNF136;ZNF138;ZNF14;ZNF140;ZNF143;ZNF146;ZNF148;ZNF155;ZNF157;ZNF160;ZNF169;ZNF175;ZNF177;ZNF18;ZNF180;ZNF181;ZNF2;ZNF20;ZNF212;ZNF213;ZNF214;ZNF221;ZNF222;ZNF223;ZNF224;ZNF225;ZNF226;ZNF227;ZNF229;ZNF230;ZNF232;ZNF233;ZNF234;ZNF235;ZNF24;ZNF25;ZNF250;ZNF257;ZNF26;ZNF260;ZNF263;ZNF264;ZNF268;ZNF274;ZNF276;ZNF28;ZNF280A;ZNF280B;ZNF280C;ZNF280D;ZNF281;ZNF282;ZNF283;ZNF284;ZNF285;ZNF286A;ZNF286B;ZNF3;ZNF30;ZNF300;ZNF302;ZNF317;ZNF32;ZNF320;ZNF322;ZNF324;ZNF324B;ZNF329;ZNF331;ZNF333;ZNF33A;ZNF33B;ZNF343;ZNF345;ZNF347;ZNF350;ZNF354A;ZNF354B;ZNF362;ZNF366;ZNF383;ZNF384;ZNF394;ZNF397;ZNF398;ZNF404;ZNF41;ZNF410;ZNF419;ZNF420;ZNF431;ZNF432;ZNF436;ZNF439;ZNF44;ZNF440;ZNF442;ZNF443;ZNF446;ZNF449;ZNF45;ZNF460;ZNF468;ZNF479;ZNF484;ZNF490;ZNF500;ZNF502;ZNF524;ZNF525;ZNF528;ZNF543;ZNF544;ZNF546;ZNF547;ZNF548;ZNF549;ZNF554;ZNF555;ZNF557;ZNF558;ZNF559;ZNF561;ZNF562;ZNF563;ZNF564;ZNF566;ZNF567;ZNF568;ZNF57;ZNF570;ZNF571;ZNF572;ZNF577;ZNF581;ZNF582;ZNF583;ZNF585A;ZNF586;ZNF589;ZNF595;ZNF599;ZNF600;ZNF605;ZNF607;ZNF611;ZNF613;ZNF614;ZNF615;ZNF616;ZNF619;ZNF620;ZNF621;ZNF625;ZNF627;ZNF649;ZNF652;ZNF653;ZNF665;ZNF667;ZNF669;ZNF670;ZNF672;ZNF679;ZNF680;ZNF683;ZNF689;ZNF692;ZNF701;ZNF705D;ZNF705E;ZNF705G;ZNF708;ZNF709;ZNF71;ZNF710;ZNF713;ZNF721;ZNF727;ZNF729;ZNF736;ZNF75A;ZNF75D;ZNF76;ZNF763;ZNF764;ZNF765;ZNF768;ZNF77;ZNF771;ZNF773;ZNF774;ZNF776;ZNF777;ZNF780A;ZNF780B;ZNF782;ZNF785;ZNF799;ZNF805;ZNF808;ZNF81;ZNF813;ZNF816;ZNF823;ZNF829;ZNF836;ZNF841;ZNF844;ZNF845;ZNF846;ZNF85;ZNF853;ZNF860;ZNF878;ZNF891;ZNF99;ZSCAN16;ZSCAN2;ZSCAN22;ZSCAN23;ZSCAN29;ZSCAN31;ZSCAN32;ZSCAN4;ZSCAN5A;ZSCAN5B;ZSCAN5C;ZSCAN9;ZXDA;ZXDB;ZXDC	FEZF1
CR3L2_HUMAN.H11MO.0.D	31	0.18128655	0.4801191	10.888.346	0.7334686	123634,10000000	0.3189897281	CREB-related factors{1.1.7}	CREB-3-like factors{1.1.7.2}	ATF1;ATF6;ATF6B;CREB1;CREB3;CREB3L1;CREB3L2;CREB3L3;CREB3L4;CREBL2;CREBZF;CREM	CREB3L1;CREB3L2;CREB3

motif	Nº of probes	% of probes	lower OR	upper OR	OR	p.value	FDR	TF family	TF subfamily	TF.family.member	TF.subfamily.member
GFI1_HUMAN.H11MO.0.C	13	0.07602339	0.4793767	16.210.454	0.9206259	889321,30000000	0.9598074264	More than 3 adjacent zinc finger factors{2.3.3}	GFI1 factors{2.3.3.21}	BCL6;BCL6B;CTCF;CTCFL;FEZF1;FEZF2;GFI1;GFI1B;GLI1;GLI2;GLI3;GLI4;GLIS1;GLIS2;GLIS3;HKR1;MTF1;MYNN;MZF1;OSR2;OVOL1;OVOL2;PLAG1;PLAGL1;PLAGL2;PRDM1;PRDM14;PRDM6;SCRT1;SCRT2;SNAI1;SNAI2;SNAI3;WT1;YY1;YY2;ZBTB12;ZBTB14;ZBTB18;ZBTB20;ZBTB26;ZBTB42;ZBTB45;ZBTB47;ZBTB48;ZBTB49;ZBTB6;ZBTB7A;ZBTB7B;ZBTB7C;ZFP14;ZFP2;ZFP28;ZFP30;ZFP37;ZFP42;ZFP64;ZFP69;ZFP69B;ZFP82;ZFP91;ZFX;ZIC1;ZIC2;ZIC3;ZIC4;ZIC5;ZIK1;ZIM3;ZKSCAN1;ZKSCAN2;ZKSCAN3;ZKSCAN4;ZNF121;ZNF124;ZNF133;ZNF136;ZNF138;ZNF14;ZNF140;ZNF143;ZNF146;ZNF148;ZNF155;ZNF157;ZNF160;ZNF169;ZNF175;ZNF177;ZNF18;ZNF180;ZNF181;ZNF2;ZNF20;ZNF212;ZNF213;ZNF214;ZNF221;ZNF222;ZNF223;ZNF224;ZNF225;ZNF226;ZNF227;ZNF229;ZNF230;ZNF232;ZNF233;ZNF234;ZNF235;ZNF24;ZNF25;ZNF250;ZNF257;ZNF26;ZNF260;ZNF263;ZNF264;ZNF268;ZNF274;ZNF276;ZNF28;ZNF280A;ZNF280B;ZNF280C;ZNF280D;ZNF281;ZNF282;ZNF283;ZNF284;ZNF285;ZNF286A;ZNF286B;ZNF3;ZNF30;ZNF300;ZNF302;ZNF317;ZNF32;ZNF320;ZNF322;ZNF324;ZNF324B;ZNF329;ZNF331;ZNF333;ZNF33A;ZNF33B;ZNF343;ZNF345;ZNF347;ZNF350;ZNF354A;ZNF354B;ZNF362;ZNF366;ZNF383;ZNF384;ZNF394;ZNF397;ZNF398;ZNF404;ZNF41;ZNF410;ZNF419;ZNF420;ZNF431;ZNF432;ZNF436;ZNF439;ZNF44;ZNF440;ZNF442;ZNF443;ZNF446;ZNF449;ZNF45;ZNF460;ZNF468;ZNF479;ZNF484;ZNF490;ZNF500;ZNF502;ZNF524;ZNF525;ZNF528;ZNF543;ZNF544;ZNF546;ZNF547;ZNF548;ZNF549;ZNF554;ZNF555;ZNF557;ZNF558;ZNF559;ZNF561;ZNF562;ZNF563;ZNF564;ZNF566;ZNF567;ZNF568;ZNF57;ZNF570;ZNF571;ZNF572;ZNF577;ZNF581;ZNF582;ZNF583;ZNF585A;ZNF586;ZNF589;ZNF595;ZNF599;ZNF600;ZNF605;ZNF607;ZNF611;ZNF613;ZNF614;ZNF615;ZNF616;ZNF619;ZNF620;ZNF621;ZNF625;ZNF627;ZNF649;ZNF652;ZNF653;ZNF665;ZNF667;ZNF669;ZNF670;ZNF672;ZNF679;ZNF680;ZNF683;ZNF689;ZNF692;ZNF701;ZNF705D;ZNF705E;ZNF705G;ZNF708;ZNF709;ZNF71;ZNF710;ZNF713;ZNF721;ZNF727;ZNF729;ZNF736;ZNF75A;ZNF75D;ZNF76;ZNF763;ZNF764;ZNF765;ZNF768;ZNF77;ZNF771;ZNF773;ZNF774;ZNF776;ZNF777;ZNF780A;ZNF780B;ZNF782;ZNF785;ZNF799;ZNF805;ZNF808;ZNF81;ZNF813;ZNF816;ZNF823;ZNF829;ZNF836;ZNF841;ZNF844;ZNF845;ZNF846;ZNF85;ZNF853;ZNF860;ZNF878;ZNF891;ZNF99;ZSCAN16;ZSCAN2;ZSCAN22;ZSCAN23;ZSCAN29;ZSCAN31;ZSCAN32;ZSCAN4;ZSCAN5A;ZSCAN5B;ZSCAN5C;ZSCAN9;ZXDA;ZXDB;ZXDC	GFI1B;GFI1
ZN423_HUMAN.H11MO.0.D	22	0.12865497	0.4791312	12.381.361	0.7878833	344653,00000000	0.5958014910	Factors with multiple dispersed zinc fingers{2.3.4}	ZNF423-like factors{2.3.4.12}	BCL11A;BCL11B;BNC1;BNC2;E4F1;HIC1;HIC2;HINFP;HIVEP1;HIVEP2;HIVEP3;IKZF1;IKZF2;IKZF3;IKZF4;IKZF5;INSM1;INSM2;MAZ;MECOM;PATZ1;PRDM16;PRDM4;REST;RLF;RREB1;SALL1;SALL2;SALL3;SALL4;VEZF1;ZBTB1;ZBTB17;ZBTB2;ZBTB25;ZBTB4;ZFAT;ZNF134;ZNF211;ZNF217;ZNF219;ZNF248;ZNF256;ZNF292;ZNF296;ZNF319;ZNF334;ZNF335;ZNF341;ZNF37A;ZNF382;ZNF417;ZNF418;ZNF423;ZNF467;ZNF510;ZNF512;ZNF512B;ZNF516;ZNF518A;ZNF518B;ZNF521;ZNF526;ZNF532;ZNF536;ZNF552;ZNF574;ZNF587;ZNF587B;ZNF592;ZNF639;	ZNF423

motif	Nº of probes	% of probes	lower OR	upper OR	OR	p.value	FDR	TF family	TF subfamily	TF.family.member	TF.subfamily.member
										ZNF654;ZNF658;ZNF671;ZNF687;ZNF711;ZNF717;ZNF770;ZNF772;ZNF784;ZNF786;ZNF792;ZNF8;ZNF814	
UNC4_HUMAN.H11MO.0.D	9	0.05263158	0.4785892	20.756.860	1,07E+07	858936,60000000	0.9464295858	Paired-related HD factors{3.1.3}	UNCX{3.1.3.27}	ALX1;ALX3;ALX4;ARGFX;ARX;CRX;DMBX1;DPRX;DRGX;DUX4;DUXA;ESX1;GSC;GSC2;HESX1;ISX;LEUTX;MIXL1;NOBOX;OTP;OTX1;OTX2;PHOX2A;PHOX2B;PITX1;PITX2;PITX3;PROP1;PRRX1;PRRX2;RAX;RAX2;RHOXF1;RHOXF2;SEBOX;SHOX;SHOX2;TPRX1;UNCX;VSX1;VSX2	UNCX
PPARG_HUMAN.H11MO.0.A	21	0.12280702	0.4785687	12.619.363	0.7960574	390192,40000000	0.6285524880	Thyroid hormone receptor-related factors (NR1){2.1.2}	PPAR (NR1C){2.1.2.5}	NR1D1;NR1D2;NR1H2;NR1H3;NR1H4;NR1I2;NR1I3;PPARA;PPARD;PPARG;RARA;RARB;RARG;RORA;RORB;RORC;THRA;THRB;VDR	PPARA;PPARD;PPARG
ZN652_HUMAN.H11MO.0.D	8	0.04678363	0.4767065	22.682.467	1,12E+07	700520,60000000	0.8711313094	More than 3 adjacent zinc finger factors{2.3.3}	ZNF652-like factors{2.3.3.29}	BCL6;BCL6B;CTCF;CTCFL;FEZF1;FEZF2;GFI1;GFI1B;GLI1;GLI2;GLI3;GLI4;GLIS1;GLIS2;GLIS3;HKR1;MTF1;MYNN;MZF1;OSR2;OVOL1;OVOL2;PLAG1;PLAGL1;PLAGL2;PRDM1;PRDM14;PRDM6;SCRT1;SCRT2;SNAI1;SNAI2;SNAI3;WT1;YY1;YY2;ZBTB12;ZBTB14;ZBTB18;ZBTB20;ZBTB26;ZBTB42;ZBTB45;ZBTB47;ZBTB48;ZBTB49;ZBTB6;ZBTB7A;ZBTB7B;ZBTB7C;ZFP14;ZFP2;ZFP28;ZFP30;ZFP37;ZFP42;ZFP64;ZFP69;ZFP69B;ZFP82;ZFP91;ZFX;ZIC1;ZIC2;ZIC3;ZIC4;ZIC5;ZIK1;ZIM3;ZKSCAN1;ZKSCAN2;ZKSCAN3;ZKSCAN4;ZNF121;ZNF124;ZNF133;ZNF136;ZNF138;ZNF14;ZNF140;ZNF143;ZNF146;ZNF148;ZNF155;ZNF157;ZNF160;ZNF169;ZNF175;ZNF177;ZNF18;ZNF180;ZNF181;ZNF2;ZNF20;ZNF212;ZNF213;ZNF214;ZNF221;ZNF222;ZNF223;ZNF224;ZNF225;ZNF226;ZNF227;ZNF229;ZNF230;ZNF232;ZNF233;ZNF234;ZNF235;ZNF24;ZNF25;ZNF250;ZNF257;ZNF26;ZNF260;ZNF263;ZNF264;ZNF268;ZNF274;ZNF276;ZNF28;ZNF280A;ZNF280B;ZNF280C;ZNF280D;ZNF281;ZNF282;ZNF283;ZNF284;ZNF285;ZNF286A;ZNF286B;ZNF3;ZNF30;ZNF300;ZNF302;ZNF317;ZNF32;ZNF320;ZNF322;ZNF324;ZNF324B;ZNF329;ZNF331;ZNF333;ZNF33A;ZNF33B;ZNF343;ZNF345;ZNF347;ZNF350;ZNF354A;ZNF354B;ZNF362;ZNF366;ZNF383;ZNF384;ZNF394;ZNF397;ZNF398;ZNF404;ZNF41;ZNF410;ZNF419;ZNF420;ZNF431;ZNF432;ZNF436;ZNF439;ZNF44;ZNF440;ZNF442;ZNF443;ZNF446;ZNF449;ZNF45;ZNF460;ZNF468;ZNF479;ZNF484;ZNF490;ZNF500;ZNF502;ZNF524;ZNF525;ZNF528;ZNF543;ZNF544;ZNF546;ZNF547;ZNF548;ZNF549;ZNF554;ZNF555;ZNF557;ZNF558;ZNF559;ZNF561;ZNF562;ZNF563;ZNF564;ZNF566;ZNF567;ZNF568;ZNF57;ZNF570;ZNF571;ZNF572;ZNF577;ZNF581;ZNF582;ZNF583;ZNF585A;ZNF586;ZNF589;ZNF595;ZNF599;ZNF600;ZNF605;ZNF607;ZNF611;ZNF613;ZNF614;ZNF615;ZNF616;ZNF619;ZNF620;ZNF621;ZNF625;ZNF627;ZNF649;ZNF652;ZNF653;ZNF665;ZNF667;ZNF669;ZNF670;ZNF672;ZNF679;ZNF680;ZNF683;ZNF689;ZNF692;ZNF701;ZNF705D;ZNF705E;ZNF705G;ZNF708;ZNF709;ZNF71;ZNF710;ZNF713;ZNF721;ZNF727;ZNF729;ZNF736;ZNF75A;ZNF75D;ZNF76;ZNF763;ZNF764;ZNF765;ZNF768;ZNF77;ZNF771;ZNF773;ZNF774;ZNF776;ZNF777;ZNF780A;ZNF780B;ZNF782;ZNF785;ZNF799;ZNF805;ZNF808;ZNF81	ZNF652

motif	Nº of probes	% of probes	lower OR	upper OR	OR	p.value	FDR	TF family	TF subfamily	TF.family.member	TF.subfamily.member
										;ZNF813;ZNF816;ZNF823;ZNF829;ZNF836;ZNF841;ZNF844;ZNF845;ZNF846;ZNF85;ZNF853;ZNF860;ZNF878;ZNF891;ZNF99;ZSCAN16;ZSCAN2;ZSCAN22;ZSCAN23;ZSCAN29;ZSCAN31;ZSCAN32;ZSCAN4;ZSCAN5A;ZSCAN5B;ZSCAN5C;ZSCAN9;ZXDA;ZXDB;ZXDC	
ETV3_HUMAN.H11MO.0.D	11	0.06432749	0.4763251	17.907.194	0.9738510	1000000,00000000	1,00E+10	Ets-related factors{3.5.2}	Ets-like factors{3.5.2.1}	EHF;ELF1;ELF2;ELF3;ELF4;ELF5;ELK1;ELK3;ELK4;ERF;ERG;ETS1;ETS2;ETV1;ETV2;ETV3;ETV3L;ETV4;ETV5;ETV6;ETV7;FEV;FLI1;GABPA;SPDEF;SPI1;SPIB;SPIC	ERG;ETS1;ETS2;ETV2;ETV3;FEV;FLI1;GABPA
P73_HUMAN.H11MO.1.A	18	0.10526316	0.4759414	13.482.655	0.8248136	488861,90000000	0.7045093404	p53-related factors{6.3.1}	p73{6.3.1.0.3}	TP53;TP63;TP73	TP73
VAX1_HUMAN.H11MO.0.D	9	0.05263158	0.4756996	20.631.466	1,06E+07	859359,10000000	0.9464295858	NK-related factors{3.1.2}	VAX{3.1.2.22}	BARHL1;BARHL2;BARX1;BARX2;BSX;DBX1;DBX2;DLX1;DLX2;DLX3;DLX4;DLX5;DLX6;EMX1;EMX2;EN1;EN2;HHEX;HLX;HMX1;HMX2;HMX3;LBX1;LBX2;MSX1;MSX2;NANOG;NKX1-1;NKX1-2;NKX2-1;NKX2-2;NKX2-3;NKX2-4;NKX2-5;NKX2-6;NKX2-8;NKX3-1;NKX3-2;NKX6-1;NKX6-2;NKX6-3;NOTO;TLX1;TLX2;TLX3;VAX1;VAX2;VENTX	VAX1;VAX2
ZEB1_HUMAN.H11MO.0.A	27	0.15789474	0.4753588	11.318.195	0.7463272	181039,00000000	0.4107633264	HD-ZF factors{3.1.8}	ZEB{3.1.8.3}	ADNP;ADNP2;HOMEZ;NANOGNB;TSHZ1;TSHZ2;TSHZ3;ZEB1;ZEB2;ZFHX2;ZFHX3;ZFHX4;ZHX1;ZHX2;ZHX3	ZEB1
MYOG_HUMAN.H11MO.0.B	33	0.19298246	0.4750989	10.555.790	0.7175083	92942,45000000	0.2612806213	MyoD / ASC-related factors{1.2.2}	Myogenic transcription factors{1.2.2.1}	ASCL1;ASCL2;ASCL3;ASCL4;ASCL5;MYF5;MYF6;MYOD1;MYOG	MYF6;MYOD1;MYOG
TFAP4_HUMAN.H11MO.0.A	23	0.13450292	0.4729179	11.993.059	0.7694030	261495,60000000	0.4905428709	bHLH-ZIP factors{1.2.6}	AP-4{1.2.6.4}	MAX;MITF;MLX;MLXIP;MLXIPL;MNT;MXD1;MXD3;MXD4;MXI1;MYC;MYCL;MYCN;REPIN1;SREBF1;SREBF2;TFAP4;TFE3;TFEB;TFEC;USF1;USF2	TFAP4
TBX20_HUMAN.H11MO.0.D	19	0.11111111	0.4727283	13.048.350	0.8071036	432346,80000000	0.6712381522	TBX1-related factors{6.5.3}	TBX20{6.5.3.0.5}	TBX1;TBX10;TBX15;TBX18;TBX20;TBX22	TBX20
RARA_HUMAN.H11MO.1.A	31	0.18128655	0.4724676	10.714.237	0.7217556	104400,00000000	0.2794874639	Thyroid hormone receptor-related factors (NR1){2.1.2}	Retinoic acid receptors (NR1B){2.1.2.1}	NR1D1;NR1D2;NR1H2;NR1H3;NR1H4;NR1I2;NR1I3;PPARA;PPARD;PPARG;RARA;RARB;RARG;RORA;RORB;RORC;THRA;THRB;VDR	RARA;RARB;RARG
OTX1_HUMAN.H11MO.0.D	9	0.05263158	0.4723038	20.483.295	1,05E+07	859910,50000000	0.9464295858	Paired-related HD factors{3.1.3}	OTX{3.1.3.17}	ALX1;ALX3;ALX4;ARGFX;ARX;CRX;DMBX1;DPRX;DRGX;DUX4;DUXA;ESX1;GSC;GSC2;HESX1;ISX;LEUTX;MIXL1;NOBOX;OTP;OTX1;OTX2;PHOX2A;PHOX2B;PITX1;PITX2;PITX3;PROP1;PRRX1;PRRX2;RAX;RAX2;RHOXF1;RHOXF2;SEBOX;SHOX;SHOX2;TPRX1;UNCX;VSX1;VSX2	CRX;OTX1;OTX2

motif	Nº of probes	% of probes	lower OR	upper OR	OR	p.value	FDR	TF family	TF subfamily	TF.family.member	TF.subfamily.member
PTF1A_HUMAN.H11MO.1.B	34	0.19883041	0.4701647	10.348.627	0.7063511	80470,49000000	0.2368043780	Tal-related factors{1.2.3}	Twist-like factors{1.2.3.2}	ATOH1;ATOH7;ATOH8;BHLHA15;BHLHA9;BHLHE22;BHLHE23;FERD3L;FIGLA;HAND1;HAND2;LYL1;MESP1;MESP2;MSC;MSGN1;NEUROD1;NEUROD2;NEUROD4;NEUROD6;NEUROG1;NEUROG2;NEUROG3;NHLH1;NHLH2;OLIG1;OLIG2;OLIG3;PTF1A;SCX;TAL1;TAL2;TCF15;TCF21;TCF23;TWIST1;TWIST2	FIGLA;HAND1;PTF1A;TWIST1
ZN586_HUMAN.H11MO.0.C	19	0.11111111	0.4687021	12.936.587	0.8001786	432691,80000000	0.6712381522	More than 3 adjacent zinc finger factors{2.3.3}	unclassified{2.3.3.0}	BCL6B;BCL6;CTCFL;CTCF;FEZF1;GFI1B;GFI1;GLI1;GLI2;GLI3;GLIS1;GLIS2;GLIS3;MTF1;MYNN;MZF1;OSR2;OVOL1;OVOL2;ZNF146;PLAG1;PLAGL1;PRDM14;PRDM1;PRDM6;SCRT1;SCRT2;SNAI1;SNAI2;YY1;YY2;WT1;ZNF324;ZNF354A;ZBTB14;ZBTB18;ZBTB48;ZBTB49;ZBTB7A;ZBTB7B;ZBTB6;ZFP64;ZFP28;ZFP42;ZFP82;ZFX;ZIC1;ZIC2;ZIC3;ZIC4;ZIM3;ZKSCAN1;ZKSCAN3;ZNF121;ZNF136;ZNF140;ZNF143;ZNF148;ZNF214;ZNF232;ZNF250;ZNF257;ZNF260;ZNF263;ZNF264;ZNF274;ZNF281;ZNF282;ZNF317;ZNF320;ZNF322;ZNF329;ZNF331;ZNF333;ZNF350;ZNF384;ZNF394;ZNF410;ZNF436;ZNF449;ZNF490;ZNF502;ZNF524;ZNF528;ZNF547;ZNF549;ZNF554;ZNF563;ZNF582;ZNF586;ZNF589;ZNF652;ZNF667;ZNF680;ZNF708;ZNF713;ZNF768;ZNF816;ZNF18;ZNF41;ZNF76;ZNF85;ZSCAN16;ZSCAN22;ZSCAN31;ZSCAN4	MYNN;MZF1;OSR2;PRDM14;PRDM6;WT1;ZBTB14;ZBTB48;ZBTB49;ZFP64;ZFP28;ZIM3;ZNF121;ZNF250;ZNF257;ZNF263;ZNF274;ZNF317;ZNF320;ZNF329;ZNF331;ZNF394;ZNF449;ZNF502;ZNF528;ZNF547;ZNF549;ZNF554;ZNF586;ZNF589;ZNF667;ZNF680;ZNF708;ZNF713;ZNF768;ZNF18;ZNF85;ZSCAN16;ZSCAN22
ZN410_HUMAN.H11MO.0.D	8	0.04678363	0.4671572	22.228.341	1,10E+07	704842,30000000	0.8746378511	More than 3 adjacent zinc finger factors{2.3.3}	MTF1-like factors{2.3.3.24}	BCL6;BCL6B;CTCF;CTCFL;FEZF1;FEZF2;GFI1;GFI1B;GLI1;GLI2;GLI3;GLI4;GLIS1;GLIS2;GLIS3;HKR1;MTF1;MYNN;MZF1;OSR2;OVOL1;OVOL2;PLAG1;PLAGL1;PLAGL2;PRDM1;PRDM14;PRDM6;SCRT1;SCRT2;SNAI1;SNAI2;SNAI3;WT1;YY1;YY2;ZBTB12;ZBTB14;ZBTB18;ZBTB20;ZBTB26;ZBTB42;ZBTB45;ZBTB47;ZBTB48;ZBTB49;ZBTB6;ZBTB7A;ZBTB7B;ZBTB7C;ZFP14;ZFP2;ZFP28;ZFP30;ZFP37;ZFP42;ZFP64;ZFP69;ZFP69B;ZFP82;ZFP91;ZFX;ZIC1;ZIC2;ZIC3;ZIC4;ZIC5;ZIK1;ZIM3;ZKSCAN1;ZKSCAN2;ZKSCAN3;ZKSCAN4;ZNF121;ZNF124;ZNF133;ZNF136;ZNF138;ZNF14;ZNF140;ZNF143;ZNF146;ZNF148;ZNF155;ZNF157;ZNF160;ZNF169;ZNF175;ZNF177;ZNF18;ZNF180;ZNF181;ZNF2;ZNF20;ZNF212;ZNF213;ZNF214;ZNF221;ZNF222;ZNF223;ZNF224;ZNF225;ZNF226;ZNF227;ZNF229;ZNF230;ZNF232;ZNF233;ZNF234;ZNF235;ZNF24;ZNF25;ZNF250;ZNF257;ZNF26;ZNF260;ZNF263;ZNF264;ZNF268;ZNF274;ZNF276;ZNF28;ZNF280A;ZNF280B;ZNF280C;ZNF280D;ZNF281;ZNF282;ZNF283;ZNF284;ZNF285;ZNF286A;ZNF286B;ZNF3;ZNF30;ZNF300;ZNF302;ZNF317;ZNF32;ZNF320;ZNF322;ZNF324;ZNF324B;ZNF329;ZNF331;ZNF333;ZNF33A;ZNF33B;ZNF343;ZNF345;ZNF347;ZNF350;ZNF354A;ZNF354B;ZNF362;ZNF366;ZNF383;ZNF384;ZNF394;ZNF397;ZNF398;ZNF404;ZNF41;ZNF410;ZNF419;ZNF420;ZNF431;ZNF432;ZNF436;ZNF439;ZNF44;ZNF440;ZNF442;ZNF443;ZNF446;ZNF449;ZNF45;ZNF460;ZNF468;ZNF479;ZNF484;ZNF490;ZNF500;ZNF502;ZNF524;ZNF525;ZNF528;ZNF543;ZNF544;ZNF546;ZNF547;ZNF548;ZNF549;ZNF554;ZNF555;ZNF557;ZNF558;ZNF559;ZNF561;ZNF562;ZNF563;ZNF564;ZNF566;ZNF567;ZNF568;ZNF57;ZNF570;ZNF571;ZNF572;ZNF577;ZNF581;ZNF582;ZNF583;ZNF585A;ZNF586;ZNF589;ZNF595;ZNF599;ZNF600;ZNF605;ZNF607;ZNF611;ZNF613;ZNF614;ZNF615;ZNF616;ZNF619;ZNF620;ZNF621;ZNF625;ZNF627;ZNF649;ZNF652;ZNF653;ZNF665;ZNF667;ZNF669;ZNF670;ZNF672;Z	MTF1;ZNF410

motif	Nº of probes	% of probes	lower OR	upper OR	OR	p.value	FDR	TF family	TF subfamily	TF.family.member	TF.subfamily.member
										NF679;ZNF680;ZNF683;ZNF689;ZNF692;ZNF701;ZNF705D;ZNF705E;ZNF705G;ZNF708;ZNF709;ZNF71;ZNF710;ZNF713;ZNF721;ZNF727;ZNF729;ZNF736;ZNF75A;ZNF75D;ZNF76;ZNF763;ZNF764;ZNF765;ZNF768;ZNF77;ZNF771;ZNF773;ZNF774;ZNF776;ZNF777;ZNF780A;ZNF780B;ZNF782;ZNF785;ZNF799;ZNF805;ZNF808;ZNF81;ZNF813;ZNF816;ZNF823;ZNF829;ZNF836;ZNF841;ZNF844;ZNF845;ZNF846;ZNF85;ZNF853;ZNF860;ZNF878;ZNF891;ZNF99;ZSCAN16;ZSCAN2;ZSCAN22;ZSCAN23;ZSCAN29;ZSCAN31;ZSCAN32;ZSCAN4;ZSCAN5A;ZSCAN5B;ZSCAN5C;ZSCAN9;ZXDA;ZXDB;ZXDC	
ARNT2_HUMAN.H11MO.0.D	56	0.32748538	0.4667825	0.9087097	0.6545904	8515,19900000	0.0486312476	PAS domain factors{1.2.5}	Arnt-like factors{1.2.5.2}	AHR;AHRR;ARNT;ARNT2;ARNTL;ARNTL2;CLOCK;EPAS1;HIF1A;HIF3A;NCOA1;NCOA2;NCOA3;NPAS1;NPAS2;NPAS3;NPAS4;SIM1;SIM2;SOHLH1;SOHLH2;TCFL5	ARNT2;ARNT;ARNTL;CLOCK
PBX1_HUMAN.H11MO.0.A	16	0.09356725	0.4663863	14.027.465	0.8365570	623605,60000000	0.8131232880	TALE-type homeo domain factors{3.1.4}	PBX{3.1.4.4}	IRX2;IRX3;MEIS1;MEIS2;MEIS3;PBX1;PBX2;PBX3;PKNOX1;TGIF2LX;TGIF1;TGIF2	PBX1;PBX2;PBX3
PAX1_HUMAN.H11MO.0.D	14	0.08187135	0.4653060	15.062.486	0.8710462	791349,30000000	0.9038967801	Paired domain only{3.2.2}	PAX-1/9 (no homeo remnant){3.2.2.1}	PAX1;PAX2;PAX5;PAX8;PAX9	PAX1
NR1H3_HUMAN.H11MO.1.B	16	0.09356725	0.4652470	13.993.224	0.8345126	623886,20000000	0.8131232880	Thyroid hormone receptor-related factors (NR1){2.1.2}	LXR (NR1H){2.1.2.7}	NR1D1;NR1D2;NR1H2;NR1H3;NR1H4;NR1I2;NR1I3;PPARA;PPARD;PPARG;RARA;RARB;RARG;RORA;RORB;RORC;THRA;THRB;VDR	NR1H2;NR1H3;NR1H4
RARA_HUMAN.H11MO.2.A	18	0.10526316	0.4646636	13.163.957	0.8052784	490171,50000000	0.7050787317	Thyroid hormone receptor-related factors (NR1){2.1.2}	Retinoic acid receptors (NR1B){2.1.2.1}	NR1D1;NR1D2;NR1H2;NR1H3;NR1H4;NR1I2;NR1I3;PPARA;PPARD;PPARG;RARA;RARB;RARG;RORA;RORB;RORC;THRA;THRB;VDR	RARA;RARB;RARG

motif	Nº of probes	% of probes	lower OR	upper OR	OR	p.value	FDR	TF family	TF subfamily	TF.family.member	TF.subfamily.member
ZN816_HUMAN.H11MO.0.C	27	0.15789474	0.4638544	11.044.313	0.7282623	154179,40000000	0.3726405807	More than 3 adjacent zinc finger factors{2.3.3}	ZNF816A-like factors{2.3.3.73}	BCL6;BCL6B;CTCF;CTCFL;FEZF1;FEZF2;GFI1;GFI1B;GLI1;GLI2;GLI3;GLI4;GLIS1;GLIS2;GLIS3;HKR1;MTF1;MYNN;MZF1;OSR2;OVOL1;OVOL2;PLAG1;PLAGL1;PLAGL2;PRDM1;PRDM14;PRDM6;SCRT1;SCRT2;SNAI1;SNAI2;SNAI3;WT1;YY1;YY2;ZBTB12;ZBTB14;ZBTB18;ZBTB20;ZBTB26;ZBTB42;ZBTB45;ZBTB47;ZBTB48;ZBTB49;ZBTB6;ZBTB7A;ZBTB7B;ZBTB7C;ZFP14;ZFP2;ZFP28;ZFP30;ZFP37;ZFP42;ZFP64;ZFP69;ZFP69B;ZFP82;ZFP91;ZFX;ZIC1;ZIC2;ZIC3;ZIC4;ZIC5;ZIK1;ZIM3;ZKSCAN1;ZKSCAN2;ZKSCAN3;ZKSCAN4;ZNF121;ZNF124;ZNF133;ZNF136;ZNF138;ZNF14;ZNF140;ZNF143;ZNF146;ZNF148;ZNF155;ZNF157;ZNF160;ZNF169;ZNF175;ZNF177;ZNF18;ZNF180;ZNF181;ZNF2;ZNF20;ZNF212;ZNF213;ZNF214;ZNF221;ZNF222;ZNF223;ZNF224;ZNF225;ZNF226;ZNF227;ZNF229;ZNF230;ZNF232;ZNF233;ZNF234;ZNF235;ZNF24;ZNF25;ZNF250;ZNF257;ZNF26;ZNF260;ZNF263;ZNF264;ZNF268;ZNF274;ZNF276;ZNF28;ZNF280A;ZNF280B;ZNF280C;ZNF280D;ZNF281;ZNF282;ZNF283;ZNF284;ZNF285;ZNF286A;ZNF286B;ZNF3;ZNF30;ZNF300;ZNF302;ZNF317;ZNF32;ZNF320;ZNF322;ZNF324;ZNF324B;ZNF329;ZNF331;ZNF333;ZNF33A;ZNF33B;ZNF343;ZNF345;ZNF347;ZNF350;ZNF354A;ZNF354B;ZNF362;ZNF366;ZNF383;ZNF384;ZNF394;ZNF397;ZNF398;ZNF404;ZNF41;ZNF410;ZNF419;ZNF420;ZNF431;ZNF432;ZNF436;ZNF439;ZNF44;ZNF440;ZNF442;ZNF443;ZNF446;ZNF449;ZNF45;ZNF460;ZNF468;ZNF479;ZNF484;ZNF490;ZNF500;ZNF502;ZNF524;ZNF525;ZNF528;ZNF543;ZNF544;ZNF546;ZNF547;ZNF548;ZNF549;ZNF554;ZNF555;ZNF557;ZNF558;ZNF559;ZNF561;ZNF562;ZNF563;ZNF564;ZNF566;ZNF567;ZNF568;ZNF57;ZNF570;ZNF571;ZNF572;ZNF577;ZNF581;ZNF582;ZNF583;ZNF585A;ZNF586;ZNF589;ZNF595;ZNF599;ZNF600;ZNF605;ZNF607;ZNF611;ZNF613;ZNF614;ZNF615;ZNF616;ZNF619;ZNF620;ZNF621;ZNF625;ZNF627;ZNF649;ZNF652;ZNF653;ZNF665;ZNF667;ZNF669;ZNF670;ZNF672;ZNF679;ZNF680;ZNF683;ZNF689;ZNF692;ZNF701;ZNF705D;ZNF705E;ZNF705G;ZNF708;ZNF709;ZNF71;ZNF710;ZNF713;ZNF721;ZNF727;ZNF729;ZNF736;ZNF75A;ZNF75D;ZNF76;ZNF763;ZNF764;ZNF765;ZNF768;ZNF77;ZNF771;ZNF773;ZNF774;ZNF776;ZNF777;ZNF780A;ZNF780B;ZNF782;ZNF785;ZNF799;ZNF805;ZNF808;ZNF81;ZNF813;ZNF816;ZNF823;ZNF829;ZNF836;ZNF841;ZNF844;ZNF845;ZNF846;ZNF85;ZNF853;ZNF860;ZNF878;ZNF891;ZNF99;ZSCAN16;ZSCAN2;ZSCAN22;ZSCAN23;ZSCAN29;ZSCAN31;ZSCAN32;ZSCAN4;ZSCAN5A;ZSCAN5B;ZSCAN5C;ZSCAN9;ZXDA;ZXDB;ZXDC	ZNF816
GRHL1_HUMAN.H11MO.0.D	13	0.07602339	0.4632710	15.664.959	0.8896138	784053,50000000	0.8989759399	Grainyhead-related factors{6.7.1}	GRH-like proteins{6.7.1.1}	GRHL1;GRHL2;GRHL3	GRHL1;GRHL2
PITX1_HUMAN.H11MO.0.D	9	0.05263158	0.4618318	20.029.215	1,03E+07	862003,30000000	0.9464295858	Paired-related HD factors{3.1.3}	PITX{3.1.3.19}	ALX1;ALX3;ALX4;ARGFX;ARX;CRX;DMBX1;DPRX;DRGX;DUX4;DUXA;ESX1;GSC;GSC2;HESX1;ISX;LEUTX;MIXL1;NOBOX;OTP;OTX1;OTX2;PHOX2A;PHOX2B;PITX1;PITX2;PITX3;PROP1;PRRX1;PRRX2;RAX;RAX2;RHOXF1;RHOXF2;SEBOX;SHOX;SHOX2;TPRX1;UNCX;VSX1;VSX2	PITX1;PITX2;PITX3

motif	Nº of probes	% of probes	lower OR	upper OR	OR	p.value	FDR	TF family	TF subfamily	TF.family.member	TF.subfamily.member
NKX25_HUMAN.H11MO.0.B	24	0.14035088	0.4610637	11.489.930	0.7427492	195667,00000000	0.4308499091	NK-related factors{3.1.2}	NK-4{3.1.2.17}	BARHL1;BARHL2;BARX1;BARX2;BSX;DBX1;DBX2;DLX1;DLX2;DLX3;DLX4;DLX5;DLX6;EMX1;EMX2;EN1;EN2;HHEX;HLX;HMX1;HMX2;HMX3;LBX1;LBX2;MSX1;MSX2;NANOG;NKX1-1;NKX1-2;NKX2-1;NKX2-2;NKX2-3;NKX2-4;NKX2-5;NKX2-6;NKX2-8;NKX3-1;NKX3-2;NKX6-1;NKX6-2;NKX6-3;NOTO;TLX1;TLX2;TLX3;VAX1;VAX2;VENTX	NKX2-3;NKX2-5
ZN121_HUMAN.H11MO.0.C	30	0.17543860	0.4608626	10.567.725	0.7084262	101533,00000000	0.2757397274	More than 3 adjacent zinc finger factors{2.3.3}	unclassified{2.3.3.0}	BCL6B;BCL6;CTCFL;CTCF;FEZF1;GFI1B;GFI1;GLI1;GLI2;GLI3;GLIS1;GLIS2;GLIS3;MTF1;MYNN;MZF1;OSR2;OVOL1;OVOL2;ZNF146;PLAG1;PLAGL1;PRDM14;PRDM1;PRDM6;SCRT1;SCRT2;SNAI1;SNAI2;YY1;YY2;WT1;ZNF324;ZNF354A;ZBTB14;ZBTB18;ZBTB48;ZBTB49;ZBTB7A;ZBTB7B;ZBTB6;ZFP64;ZFP28;ZFP42;ZFP82;ZFX;ZIC1;ZIC2;ZIC3;ZIC4;ZIM3;ZKSCAN1;ZKSCAN3;ZNF121;ZNF136;ZNF140;ZNF143;ZNF148;ZNF214;ZNF232;ZNF250;ZNF257;ZNF260;ZNF263;ZNF264;ZNF274;ZNF281;ZNF282;ZNF317;ZNF320;ZNF322;ZNF329;ZNF331;ZNF333;ZNF350;ZNF384;ZNF394;ZNF410;ZNF436;ZNF449;ZNF490;ZNF502;ZNF524;ZNF528;ZNF547;ZNF549;ZNF554;ZNF563;ZNF582;ZNF586;ZNF589;ZNF652;ZNF667;ZNF680;ZNF708;ZNF713;ZNF768;ZNF816;ZNF18;ZNF41;ZNF76;ZNF85;ZSCAN16;ZSCAN22;ZSCAN31;ZSCAN4	MYNN;MZF1;OSR2;PRDM14;PRDM6;WT1;ZBTB14;ZBTB48;ZBTB49;ZFP64;ZFP28;ZIM3;ZNF121;ZNF250;ZNF257;ZNF263;ZNF274;ZNF317;ZNF320;ZNF329;ZNF331;ZNF394;ZNF449;ZNF502;ZNF528;ZNF547;ZNF549;ZNF554;ZNF586;ZNF589;ZNF667;ZNF680;ZNF708;ZNF713;ZNF768;ZNF18;ZNF85;ZSCAN16;ZSCAN22
ZN263_HUMAN.H11MO.1.A	40	0.23391813	0.4601513	0.9661831	0.6732956	31377,15000000	0.1253460221	More than 3 adjacent zinc finger factors{2.3.3}	unclassified{2.3.3.0}	BCL6B;BCL6;CTCFL;CTCF;FEZF1;GFI1B;GFI1;GLI1;GLI2;GLI3;GLIS1;GLIS2;GLIS3;MTF1;MYNN;MZF1;OSR2;OVOL1;OVOL2;ZNF146;PLAG1;PLAGL1;PRDM14;PRDM1;PRDM6;SCRT1;SCRT2;SNAI1;SNAI2;YY1;YY2;WT1;ZNF324;ZNF354A;ZBTB14;ZBTB18;ZBTB48;ZBTB49;ZBTB7A;ZBTB7B;ZBTB6;ZFP64;ZFP28;ZFP42;ZFP82;ZFX;ZIC1;ZIC2;ZIC3;ZIC4;ZIM3;ZKSCAN1;ZKSCAN3;ZNF121;ZNF136;ZNF140;ZNF143;ZNF148;ZNF214;ZNF232;ZNF250;ZNF257;ZNF260;ZNF263;ZNF264;ZNF274;ZNF281;ZNF282;ZNF317;ZNF320;ZNF322;ZNF329;ZNF331;ZNF333;ZNF350;ZNF384;ZNF394;ZNF410;ZNF436;ZNF449;ZNF490;ZNF502;ZNF524;ZNF528;ZNF547;ZNF549;ZNF554;ZNF563;ZNF582;ZNF586;ZNF589;ZNF652;ZNF667;ZNF680;ZNF708;ZNF713;ZNF768;ZNF816;ZNF18;ZNF41;ZNF76;ZNF85;ZSCAN16;ZSCAN22;ZSCAN31;ZSCAN4	MYNN;MZF1;OSR2;PRDM14;PRDM6;WT1;ZBTB14;ZBTB48;ZBTB49;ZFP64;ZFP28;ZIM3;ZNF121;ZNF250;ZNF257;ZNF263;ZNF274;ZNF317;ZNF320;ZNF329;ZNF331;ZNF394;ZNF449;ZNF502;ZNF528;ZNF547;ZNF549;ZNF554;ZNF586;ZNF589;ZNF667;ZNF680;ZNF708;ZNF713;ZNF768;ZNF18;ZNF85;ZSCAN16;ZSCAN22
GSX1_HUMAN.H11MO.0.D	9	0.05263158	0.4596196	19.932.885	1,02E+07	862515,70000000	0.9464295858	HOX-related factors{3.1.1}	GSX{3.1.1.12}	CDX1;CDX2;CDX4;EVX1;EVX2;GBX1;GBX2;GSX1;GSX2;HDX;HMBOX1;HNF1A;HNF1B;HOXA1;HOXA10;HOXA11;HOXA13;HOXA2;HOXA3;HOXA4;HOXA5;HOXA6;HOXA7;HOXA9;HOXB1;HOXB13;HOXB2;HOXB3;HOXB4;HOXB5;HOXB6;HOXB7;HOXB8;HOXB9;HOXC10;HOXC11;HOXC12;HOXC13;HOXC4;HOXC5;HOXC6;HOXC8;HOXC9;HOXD1;HOXD10;HOXD11;HOXD12;HOXD13;HOXD3;HOXD4;HOXD8;HOXD9;MEOX1;MEOX2;MNX1;PDX1;POU1F1;POU2F1;POU2F2;POU2F3;POU3F1;POU3F2;POU3F3;POU3F4;POU4F1;POU4F2;POU4F3;POU5F1;POU5F2;POU6F1;POU6F2	GSX1;GSX2
ONEC3_HUMAN.H11MO.0.D	10	0.05847953	0.4581570	18.392.185	0.9740984	1000000,00000000	1,00E+10	HD-CUT factors{3.1.9}	ONECUT{3.1.9.1}	CUX1;CUX2;ONECUT1;ONECUT2;ONECUT3;SATB1;SATB2	ONECUT1;ONECUT2;ONECUT3
RXRA_HUMAN.H11MO.0.A	38	0.22222222	0.4581530	0.9756538	0.6756803	35927,02000000	0.1391946465	RXR-related receptors (NR2){2.1.3}	Retinoid X receptors (NR2B){2.1.3.1}	HNF4A;HNF4G;NR2C1;NR2C2;NR2E1;NR2E3;NR2F1;NR2F2;NR2F6;RXRA;RXRB;RXRG	RXRA;RXRB;RXRG

motif	Nº of probes	% of probes	lower OR	upper OR	OR	p.value	FDR	TF family	TF subfamily	TF.family.member	TF.subfamily.member
HXB13_HUMAN.H11MO.0.A	9	0.05263158	0.4574197	19.836.182	1,02E+07	863067,90000000	0.9464295858	HOX-related factors{3.1.1}	HOX9-13{3.1.1.8}	CDX1;CDX2;CDX4;EVX1;EVX2;GBX1;GBX2;GSX1;GSX2;HDX;HMBOX1;HNF1A;HNF1B;HOXA1;HOXA10;HOXA11;HOXA13;HOXA2;HOXA3;HOXA4;HOXA5;HOXA6;HOXA7;HOXA9;HOXB1;HOXB13;HOXB2;HOXB3;HOXB4;HOXB5;HOXB6;HOXB7;HOXB8;HOXB9;HOXC10;HOXC11;HOXC12;HOXC13;HOXC4;HOXC5;HOXC6;HOXC8;HOXC9;HOXD1;HOXD10;HOXD11;HOXD12;HOXD13;HOXD3;HOXD4;HOXD8;HOXD9;MEOX1;MEOX2;MNX1;PDX1;POU1F1;POU2F1;POU2F2;POU2F3;POU3F1;POU3F2;POU3F3;POU3F4;POU4F1;POU4F2;POU4F3;POU5F1;POU5F2;POU6F1;POU6F2	HOXA10;HOXA11;HOXA13;HOXA9;HOXB13;HOXC10;HOXC11;HOXC12;HOXC13;HOXC9;HOXD10;HOXD11;HOXD12;HOXD13;HOXD9
HESX1_HUMAN.H11MO.0.D	19	0.11111111	0.4565499	12.601.129	0.7794516	374200,80000000	0.6225094823	Paired-related HD factors{3.1.3}	HESX{3.1.3.10}	ALX1;ALX3;ALX4;ARGFX;ARX;CRX;DMBX1;DPRX;DRGX;DUX4;DUXA;ESX1;GSC;GSC2;HESX1;ISX;LEUTX;MIXL1;NOBOX;OTP;OTX1;OTX2;PHOX2A;PHOX2B;PITX1;PITX2;PITX3;PROP1;PRRX1;PRRX2;RAX;RAX2;RHOXF1;RHOXF2;SEBOX;SHOX;SHOX2;TPRX1;UNCX;VSX1;VSX2	HESX1
THA_HUMAN.H11MO.1.D	18	0.10526316	0.4559044	12.916.020	0.7900977	424253,70000000	0.6675501646	Thyroid hormone receptor-related factors (NR1){2.1.2}	Thyroid hormone receptors (NR1A){2.1.2.2}	NR1D1;NR1D2;NR1H2;NR1H3;NR1H4;NR1I2;NR1I3;PPARA;PPARD;PPARG;RARA;RARB;RARG;RORA;RORB;RORC;THRA;THRB;VDR	THRA;THRB
HNF4G_HUMAN.H11MO.0.B	14	0.08187135	0.4545367	14.713.744	0.8508764	694540,60000000	0.8678942956	RXR-related receptors (NR2){2.1.3}	HNF-4 (NR2A){2.1.3.2}	HNF4A;HNF4G;NR2C1;NR2C2;NR2E1;NR2E3;NR2F1;NR2F2;NR2F6;RXRA;RXRB;RXRG	HNF4A;HNF4G
ZN549_HUMAN.H11MO.0.C	13	0.07602339	0.4531472	15.322.692	0.8702377	784656,60000000	0.8989759399	More than 3 adjacent zinc finger factors{2.3.3}	unclassified{2.3.3.0}	BCL6B;BCL6;CTCFL;CTCF;FEZF1;GFI1B;GFI1;GLI1;GLI2;GLI3;GLIS1;GLIS2;GLIS3;MTF1;MYNN;MZF1;OSR2;OVOL1;OVOL2;ZNF146;PLAG1;PLAGL1;PRDM14;PRDM1;PRDM6;SCRT1;SCRT2;SNAI1;SNAI2;YY1;YY2;WT1;ZNF324;ZNF354A;ZBTB14;ZBTB18;ZBTB48;ZBTB49;ZBTB7A;ZBTB7B;ZBTB6;ZFP64;ZFP28;ZFP42;ZFP82;ZFX;ZIC1;ZIC2;ZIC3;ZIC4;ZIM3;ZKSCAN1;ZKSCAN3;ZNF121;ZNF136;ZNF140;ZNF143;ZNF148;ZNF214;ZNF232;ZNF250;ZNF257;ZNF260;ZNF263;ZNF264;ZNF274;ZNF281;ZNF282;ZNF317;ZNF320;ZNF322;ZNF329;ZNF331;ZNF333;ZNF350;ZNF384;ZNF394;ZNF410;ZNF436;ZNF449;ZNF490;ZNF502;ZNF524;ZNF528;ZNF547;ZNF549;ZNF554;ZNF563;ZNF582;ZNF586;ZNF589;ZNF652;ZNF667;ZNF680;ZNF708;ZNF713;ZNF768;ZNF816;ZNF18;ZNF41;ZNF76;ZNF85;ZSCAN16;ZSCAN22;ZSCAN31;ZSCAN4	MYNN;MZF1;OSR2;PRDM14;PRDM6;WT1;ZBTB14;ZBTB48;ZBTB49;ZFP64;ZFP28;ZIM3;ZNF121;ZNF250;ZNF257;ZNF263;ZNF274;ZNF317;ZNF320;ZNF329;ZNF331;ZNF394;ZNF449;ZNF502;ZNF528;ZNF547;ZNF549;ZNF554;ZNF586;ZNF589;ZNF667;ZNF680;ZNF708;ZNF713;ZNF768;ZNF18;ZNF85;ZSCAN16;ZSCAN22

motif	Nº of probes	% of probes	lower OR	upper OR	OR	p.value	FDR	TF family	TF subfamily	TF.family.member	TF.subfamily.member
ZN282_HUMAN.H11MO.0.D	13	0.07602339	0.4527497	15.309.218	0.8694719	784709,20000000	0.8989759399	More than 3 adjacent zinc finger factors{2.3.3}	ZNF282-like factors{2.3.3.38}	BCL6;BCL6B;CTCF;CTCFL;FEZF1;FEZF2;GFI1;GFI1B;GLI1;GLI2;GLI3;GLI4;GLIS1;GLIS2;GLIS3;HKR1;MTF1;MYNN;MZF1;OSR2;OVOL1;OVOL2;PLAG1;PLAGL1;PLAGL2;PRDM1;PRDM14;PRDM6;SCRT1;SCRT2;SNAI1;SNAI2;SNAI3;WT1;YY1;YY2;ZBTB12;ZBTB14;ZBTB18;ZBTB20;ZBTB26;ZBTB42;ZBTB45;ZBTB47;ZBTB48;ZBTB49;ZBTB6;ZBTB7A;ZBTB7B;ZBTB7C;ZFP14;ZFP2;ZFP28;ZFP30;ZFP37;ZFP42;ZFP64;ZFP69;ZFP69B;ZFP82;ZFP91;ZFX;ZIC1;ZIC2;ZIC3;ZIC4;ZIC5;ZIK1;ZIM3;ZKSCAN1;ZKSCAN2;ZKSCAN3;ZKSCAN4;ZNF121;ZNF124;ZNF133;ZNF136;ZNF138;ZNF14;ZNF140;ZNF143;ZNF146;ZNF148;ZNF155;ZNF157;ZNF160;ZNF169;ZNF175;ZNF177;ZNF18;ZNF180;ZNF181;ZNF2;ZNF20;ZNF212;ZNF213;ZNF214;ZNF221;ZNF222;ZNF223;ZNF224;ZNF225;ZNF226;ZNF227;ZNF229;ZNF230;ZNF232;ZNF233;ZNF234;ZNF235;ZNF24;ZNF25;ZNF250;ZNF257;ZNF26;ZNF260;ZNF263;ZNF264;ZNF268;ZNF274;ZNF276;ZNF28;ZNF280A;ZNF280B;ZNF280C;ZNF280D;ZNF281;ZNF282;ZNF283;ZNF284;ZNF285;ZNF286A;ZNF286B;ZNF3;ZNF30;ZNF300;ZNF302;ZNF317;ZNF32;ZNF320;ZNF322;ZNF324;ZNF324B;ZNF329;ZNF331;ZNF333;ZNF33A;ZNF33B;ZNF343;ZNF345;ZNF347;ZNF350;ZNF354A;ZNF354B;ZNF362;ZNF366;ZNF383;ZNF384;ZNF394;ZNF397;ZNF398;ZNF404;ZNF41;ZNF410;ZNF419;ZNF420;ZNF431;ZNF432;ZNF436;ZNF439;ZNF44;ZNF440;ZNF442;ZNF443;ZNF446;ZNF449;ZNF45;ZNF460;ZNF468;ZNF479;ZNF484;ZNF490;ZNF500;ZNF502;ZNF524;ZNF525;ZNF528;ZNF543;ZNF544;ZNF546;ZNF547;ZNF548;ZNF549;ZNF554;ZNF555;ZNF557;ZNF558;ZNF559;ZNF561;ZNF562;ZNF563;ZNF564;ZNF566;ZNF567;ZNF568;ZNF57;ZNF570;ZNF571;ZNF572;ZNF577;ZNF581;ZNF582;ZNF583;ZNF585A;ZNF586;ZNF589;ZNF595;ZNF599;ZNF600;ZNF605;ZNF607;ZNF611;ZNF613;ZNF614;ZNF615;ZNF616;ZNF619;ZNF620;ZNF621;ZNF625;ZNF627;ZNF649;ZNF652;ZNF653;ZNF665;ZNF667;ZNF669;ZNF670;ZNF672;ZNF679;ZNF680;ZNF683;ZNF689;ZNF692;ZNF701;ZNF705D;ZNF705E;ZNF705G;ZNF708;ZNF709;ZNF71;ZNF710;ZNF713;ZNF721;ZNF727;ZNF729;ZNF736;ZNF75A;ZNF75D;ZNF76;ZNF763;ZNF764;ZNF765;ZNF768;ZNF77;ZNF771;ZNF773;ZNF774;ZNF776;ZNF777;ZNF780A;ZNF780B;ZNF782;ZNF785;ZNF799;ZNF805;ZNF808;ZNF81;ZNF813;ZNF816;ZNF823;ZNF829;ZNF836;ZNF841;ZNF844;ZNF845;ZNF846;ZNF85;ZNF853;ZNF860;ZNF878;ZNF891;ZNF99;ZSCAN16;ZSCAN2;ZSCAN22;ZSCAN23;ZSCAN29;ZSCAN31;ZSCAN32;ZSCAN4;ZSCAN5A;ZSCAN5B;ZSCAN5C;ZSCAN9;ZXDA;ZXDB;ZXDC	ZNF282
ZN341_HUMAN.H11MO.0.C	72	0.42105263	0.4511396	0.8486766	0.6200467	2070,11700000	0.0147783356	Factors with multiple dispersed zinc fingers{2.3.4}	unclassified{2.3.4.0}	BCL11A;E4F1;MECOM;HIC1;HIC2;HINFP;IKZF1;INSM1;MAZ;PATZ1;PRDM4;REST;RREB1;SALL4;VEZF1;ZBTB17;ZBTB4;HIVEP1;HIVEP2;ZNF134;ZNF219;ZNF335;ZNF341;ZNF382;ZNF418;ZNF423;ZNF467;ZNF770;ZNF784;ZNF8	E4F1;PRDM4;REST;RREB1;ZBTB17;ZBTB4;ZNF335;ZNF341;ZNF467;ZNF770;ZNF784;ZNF8
MXI1_HUMAN.H11MO.0.A	52	0.30409357	0.4510227	0.8903577	0.6375033	6304,22700000	0.0388844708	bHLH-ZIP factors{1.2.6}	Mad-like factors{1.2.6.7}	MAX;MITF;MLX;MLXIP;MLXIPL;MNT;MXD1;MXD3;MXD4;MXI1;MYC;MYCL;MYCN;REPIN1;SREBF1;SREBF2;TFAP4;TFE3;TFEB;TFEC;USF1;USF2	MXI1
MLXPL_HUMAN.H11MO.0.D	33	0.19298246	0.4503878	10.007.451	0.6802299	44985,16000000	0.1613188714	bHLH-ZIP factors{1.2.6}	Mondo-like factors{1.2.6.6}	MAX;MITF;MLX;MLXIP;MLXIPL;MNT;MXD1;MXD3;MXD4;MXI1;MYC;MYCL;MYCN;REPIN	MLXIPL;MLX

motif	Nº of probes	% of probes	lower OR	upper OR	OR	p.value	FDR	TF family	TF subfamily	TF.family.member	TF.subfamily.member
										1;SREBF1;SREBF2;TFAP4;TFE3;TFEB;TFEC;USF1;USF2	
PAX5_HUMAN.H11MO.0.A	44	0.25730994	0.4498506	0.9216500	0.6492538	12646,51000000	0.0663296404	Paired domain only{3.2.2}	PAX-2-like factors (partial homeobox){3.2.2.2}	PAX1;PAX2;PAX5;PAX8;PAX9	PAX2;PAX5;PAX8
HAND1_HUMAN.H11MO.1.D	29	0.16959064	0.4489327	10.416.463	0.6946845	82167,56000000	0.2381623553	Tal-related factors{1.2.3}	Twist-like factors{1.2.3.2}	ATOH1;ATOH7;ATOH8;BHLHA15;BHLHA9;BHLHE22;BHLHE23;FERD3L;FIGLA;HAND1;HAND2;LYL1;MESP1;MESP2;MSC;MSGN1;NEUROD1;NEUROD2;NEUROD4;NEUROD6;NEUROG1;NEUROG2;NEUROG3;NHLH1;NHLH2;OLIG1;OLIG2;OLIG3;PTF1A;SCX;TAL1;TAL2;TCF15;TCF21;TCF23;TWIST1;TWIST2	FIGLA;HAND1;PTF1A;TWIST1
SMAD1_HUMAN.H11MO.0.D	30	0.17543860	0.4473775	10.258.671	0.6877179	71001,29000000	0.2159061384	SMAD factors{7.1.1}	Regulatory Smads (R-Smad){7.1.1.1}	SMAD1;SMAD2;SMAD3;SMAD4;SMAD5;SMAD9	SMAD1;SMAD2;SMAD3
PATZ1_HUMAN.H11MO.0.C	83	0.48538012	0.4433499	0.8274837	0.6059449	1243,47500000	0.0101991377	Factors with multiple dispersed zinc fingers{2.3.4}	MAZ-like factors{2.3.4.8}	BCL11A;BCL11B;BNC1;BNC2;E4F1;HIC1;HIC2;HINFP;HIVEP1;HIVEP2;HIVEP3;IKZF1;IKZF2;IKZF3;IKZF4;IKZF5;INSM1;INSM2;MAZ;MECOM;PATZ1;PRDM16;PRDM4;REST;RLF;RREB1;SALL1;SALL2;SALL3;SALL4;VEZF1;ZBTB1;ZBTB17;ZBTB2;ZBTB25;ZBTB4;ZFAT;ZNF134;ZNF211;ZNF217;ZNF219;ZNF248;ZNF256;ZNF292;ZNF296;ZNF319;ZNF334;ZNF335;ZNF341;ZNF37A;ZNF382;ZNF417;ZNF418;ZNF423;ZNF467;ZNF510;ZNF512;ZNF512B;ZNF516;ZNF518A;ZNF518B;ZNF521;ZNF526;ZNF532;ZNF536;ZNF552;ZNF574;ZNF587;ZNF587B;ZNF592;ZNF639;ZNF654;ZNF658;ZNF671;ZNF687;ZNF711;ZNF717;ZNF770;ZNF772;ZNF784;ZNF786;ZNF792;ZNF8;ZNF814	MAZ;PATZ1;VEZF1
HMBX1_HUMAN.H11MO.0.D	8	0.04678363	0.4431746	21.085.993	1,04E+07	852569,50000000	0.9464295858	POU domain factors{3.1.10}	HNF1-like factors{3.1.10.7}	CDX1;CDX2;CDX4;EVX1;EVX2;GBX1;GBX2;GSX1;GSX2;HDX;HMBOX1;HNF1A;HNF1B;HOXA1;HOXA10;HOXA11;HOXA13;HOXA2;HOXA3;HOXA4;HOXA5;HOXA6;HOXA7;HOXA9;HOXB1;HOXB13;HOXB2;HOXB3;HOXB4;HOXB5;HOXB6;HOXB7;HOXB8;HOXB9;HOXC10;HOXC11;HOXC12;HOXC13;HOXC4;HOXC5;HOXC6;HOXC8;HOXC9;HOXD1;HOXD10;HOXD11;HOXD12;HOXD13;HOXD3;HOXD4;HOXD8;HOXD9;MEOX1;MEOX2;MNX1;PDX1;POU1F1;POU2F1;POU2F2;POU2F3;POU3F1;POU3F2;POU3F3;POU3F4;POU4F1;POU4F2;POU4F3;POU5F1;POU5F1B;POU5F2;POU6F1;POU6F2	HMBOX1;HNF1A;HNF1B
HIC1_HUMAN.H11MO.0.C	18	0.10526316	0.4431593	12.554.244	0.7679900	365926,40000000	0.6160027892	Factors with multiple dispersed zinc fingers{2.3.4}	Hypermethylated in Cancer proteins{2.3.4.17}	BCL11A;BCL11B;BNC1;BNC2;E4F1;HIC1;HIC2;HINFP;HIVEP1;HIVEP2;HIVEP3;IKZF1;IKZF2;IKZF3;IKZF4;IKZF5;INSM1;INSM2;MAZ;MECOM;PATZ1;PRDM16;PRDM4;REST;RLF;RREB1;SALL1;SALL2;SALL3;SALL4;VEZF1;ZBTB1;ZBTB17;ZBTB2;ZBTB25;ZBTB4;ZFAT;ZNF134;ZNF211;ZNF217;ZNF219;ZNF248;ZNF256;ZNF292;ZNF296;ZNF319;ZNF334;ZNF335;ZNF341;ZNF37A;ZNF382;ZNF417;ZNF418;ZNF423;ZNF467;ZNF510;ZNF512;ZNF512B;ZNF516;ZNF518A;ZNF518B;ZNF521;ZNF526;ZNF532;ZNF536;ZNF552;ZNF574;ZNF587;ZNF587B;ZNF592;ZNF639;ZNF654;ZNF658;ZNF671;ZNF687;ZNF711;ZNF717;ZNF770;ZNF772;ZNF784;ZNF786;ZNF792;ZNF8;ZNF814	HIC1;HIC2
SRBP2_HUMAN.H11MO.0.B	43	0.25146199	0.4413423	0.9093942	0.6390174	9902,55100000	0.0552036756	bHLH-ZIP factors{1.2.6}	SREBP factors{1.2.6.3}	MAX;MITF;MLX;MLXIP;MLXIPL;MNT;MXD1;MXD3;MXD4;MXI1;MYC;MYCL;MYCN;REPIN1;SREBF1;SREBF2;TFAP4;TFE3;TFEB;TFEC;USF1;USF2	SREBF1;SREBF2

motif	Nº of probes	% of probes	lower OR	upper OR	OR	p.value	FDR	TF family	TF subfamily	TF.family.member	TF.subfamily.member
RFX2_HUMAN.H11MO.1.A	16	0.09356725	0.4411541	13.267.913	0.7913221	471247,50000000	0.6984210886	RFX-related factors{3.3.3}	RFX2{3.3.3.0.2}	RFX1;RFX2;RFX3;RFX4;RFX5;RFX6;RFX7;RFX8	RFX2
MAFF_HUMAN.H11MO.0.B	13	0.07602339	0.4394930	14.859.207	0.8439056	685928,50000000	0.8643990384	Maf-related factors{1.1.3}	Small Maf factors{1.1.3.2}	MAF;MAFA;MAFB;MAFF;MAFG;MAFK;NRL	MAFF;MAFG;MAFK
EGR2_HUMAN.H11MO.0.A	67	0.39181287	0.4392950	0.8326465	0.6065720	1277,13200000	0.0103649348	Three-zinc finger Krüppel-related factors{2.3.1}	EGR factors{2.3.1.3}	EGR1;EGR2;EGR3;EGR4;KLF1;KLF10;KLF11;KLF12;KLF13;KLF14;KLF15;KLF16;KLF17;KLF2;KLF3;KLF4;KLF5;KLF6;KLF7;KLF8;KLF9;SP1;SP2;SP3;SP4;SP5;SP6;SP7;SP8;SP9	EGR1;EGR2;EGR3;EGR4
ZN770_HUMAN.H11MO.1.C	47	0.27485380	0.4387603	0.8850708	0.6277686	5660,05000000	0.0360652784	Factors with multiple dispersed zinc fingers{2.3.4}	unclassified{2.3.4.0}	BCL11A;E4F1;MECOM;HIC1;HIC2;HINFP;IKZF1;INSM1;MAZ;PATZ1;PRDM4;REST;RREB1;SALL4;VEZF1;ZBTB17;ZBTB4;HIVEP1;HIVEP2;ZNF134;ZNF219;ZNF335;ZNF341;ZNF382;ZNF418;ZNF423;ZNF467;ZNF770;ZNF784;ZNF8	E4F1;PRDM4;REST;RREB1;ZBTB17;ZBTB4;ZNF335;ZNF341;ZNF467;ZNF770;ZNF784;ZNF8
MIXL1_HUMAN.H11MO.0.D	8	0.04678363	0.4387495	20.874.438	1,03E+07	853621,20000000	0.9464295858	Paired-related HD factors{3.1.3}	MIX{3.1.3.14}	ALX1;ALX3;ALX4;ARGFX;ARX;CRX;DMBX1;DPRX;DRGX;DUX4;DUXA;ESX1;GSC;GSC2;HESX1;ISX;LEUTX;MIXL1;NOBOX;OTP;OTX1;OTX2;PHOX2A;PHOX2B;PITX1;PITX2;PITX3;PROP1;PRRX1;PRRX2;RAX;RAX2;RHOXF1;RHOXF2;SEBOX;SHOX;SHOX2;TPRX1;UNCX;VSX1;VSX2	MIXL1
NDF2_HUMAN.H11MO.0.B	23	0.13450292	0.4383975	11.118.087	0.7132830	161222,50000000	0.3824693825	Tal-related factors{1.2.3}	Neurogenin / Atonal-like factors{1.2.3.4}	ATOH1;ATOH7;ATOH8;BHLHA15;BHLHA9;BHLHE22;BHLHE23;FERD3L;FIGLA;HAND1;HAND2;LYL1;MESP1;MESP2;MSC;MSGN1;NEUROD1;NEUROD2;NEUROD4;NEUROD6;NEUROG1;NEUROG2;NEUROG3;NHLH1;NHLH2;OLIG1;OLIG2;OLIG3;PTF1A;SCX;TAL1;TAL2;TCF15;TCF21;TCF23;TWIST1;TWIST2	ATOH1;BHLHA15;BHLHE22;BHLHE23;NEUROD1;NEUROD2;NEUROG2;OLIG1;OLIG2;OLIG3
REL_HUMAN.H11MO.0.B	13	0.07602339	0.4383820	14.822.430	0.8418169	686095,60000000	0.8643990384	NF-kappaB-related factors{6.1.1}	NF-kappaB p65 subunit-like factors{6.1.1.2}	NFKB1;NFKB2;RELB;REL;RELA	RELB;REL;RELA
ZN680_HUMAN.H11MO.0.C	13	0.07602339	0.43813154	14.813.967	0.8413362	686137,80000000	8,64E+05	More than 3 adjacent zinc finger factors{2.3.3}	unclassified{2.3.3.0}	BCL6B;BCL6;CTCFL;CTCF;FEZF1;GFI1B;GFI1;GLI1;GLI2;GLI3;GLIS1;GLIS2;GLIS3;MTF1;MYNN;MZF1;OSR2;OVOL1;OVOL2;ZNF146;PLAG1;PLAGL1;PRDM14;PRDM1;PRDM6;SCRT1;SCRT2;SNAI1;SNAI2;YY1;YY2;WT1;ZNF324;ZNF354A;ZBTB14;ZBTB18;ZBTB48;ZBTB49;ZBTB7A;ZBTB7B;ZBTB6;ZFP64;ZFP28;ZFP42;ZFP82;ZFX;ZIC1;ZIC2;ZIC3;ZIC4;ZIM3;ZKSCAN1;ZKSCAN3;ZNF121;ZNF136;ZNF140;ZNF143;ZNF148;ZNF214;ZNF232;ZNF250;ZNF257;ZNF260;ZNF263;ZNF264;ZNF274;ZNF281;ZNF282;ZNF317;ZNF320;ZNF322;ZNF329;ZNF331;ZNF333;ZNF350;ZNF384;ZNF394;ZNF410;ZNF436;ZNF449;ZNF490;ZNF502;ZNF524;ZNF528;ZNF547;ZNF549;ZNF554;ZNF563;ZNF582;ZNF586;ZNF589;ZNF652;ZNF667;ZNF680;ZNF708;ZNF713;ZNF768;ZNF816;ZNF18;ZNF41;ZNF76;ZNF85;ZSCAN16;ZSCAN22;ZSCAN31;ZSCAN4	MYNN;MZF1;OSR2;PRDM14;PRDM6;WT1;ZBTB14;ZBTB48;ZBTB49;ZFP64;ZFP28;ZIM3;ZNF121;ZNF250;ZNF257;ZNF263;ZNF274;ZNF317;ZNF320;ZNF329;ZNF331;ZNF394;ZNF449;ZNF502;ZNF528;ZNF547;ZNF549;ZNF554;ZNF586;ZNF589;ZNF667;ZNF680;ZNF708;ZNF713;ZNF768;ZNF18;ZNF85;ZSCAN16;ZSCAN22
NR5A2_HUMAN.H11MO.0.B	15	0.08771930	0.43739555	13.620.870	0.8006038	459938,40000000	6,87E+05	FTZ-F1-related receptors (NR5){2.1.5}	LRH-1 (NR5A2){2.1.5.0.2}	NR5A1;NR5A2	NR5A2
P63_HUMAN.H11MO.1.A	19	0.11111111	0.43688445	12.058.654	0.7459268	274258,70000000	5,03E+05	p53-related factors{6.3.1}	p63{6.3.1.0.2}	TP53;TP63;TP73	TP63
EPAS1_HUMAN.H11MO.0.B	34	0.19883041	0.43534822	0.9582797	0.6540908	25699,39000000	1,09E+05	PAS domain factors{1.2.5}	Ahr-like factors{1.2.5.1}	AHR;AHRR;ARNT;ARNT2;ARNTL;ARNTL2;CLOCK;EPAS1;HIF1A;HIF3A;NCOA1;NCOA2;NCOA3;NPAS1;NPAS2;NPAS3;NPAS4;SIM1;SIM2;SOHLH1;SOHLH2;TCFL5	AHR;EPAS1;HIF1A

motif	Nº of probes	% of probes	lower OR	upper OR	OR	p.value	FDR	TF family	TF subfamily	TF.family.member	TF.subfamily.member
ZN467_HUMAN.H11MO.0.C	69	0.40350877	0.43500396	0.8217524	0.5994198	950,35980000	8,23E+03	Factors with multiple dispersed zinc fingers{2.3.4}	unclassified{2.3.4.0}	BCL11A;E4F1;MECOM;HIC1;HIC2;HINFP;IKZF1;INSM1;MAZ;PATZ1;PRDM4;REST;RREB1;SALL4;VEZF1;ZBTB17;ZBTB4;HIVEP1;HIVEP2;ZNF134;ZNF219;ZNF335;ZNF341;ZNF382;ZNF418;ZNF423;ZNF467;ZNF770;ZNF784;ZNF8	E4F1;PRDM4;REST;RREB1;ZBTB17;ZBTB4;ZNF335;ZNF341;ZNF467;ZNF770;ZNF784;ZNF8
HXB8_HUMAN.H11MO.0.C	7	0.04093567	0.43485261	23.181.305	1,10E+07	688167,40000000	8,66E+05	HOX-related factors{3.1.1}	HOX8{3.1.1.7}	CDX1;CDX2;CDX4;EVX1;EVX2;GBX1;GBX2;GSX1;GSX2;HDX;HMBOX1;HNF1A;HNF1B;HOXA1;HOXA10;HOXA11;HOXA13;HOXA2;HOXA3;HOXA4;HOXA5;HOXA6;HOXA7;HOXA9;HOXB1;HOXB13;HOXB2;HOXB3;HOXB4;HOXB5;HOXB6;HOXB7;HOXB8;HOXB9;HOXC10;HOXC11;HOXC12;HOXC13;HOXC4;HOXC5;HOXC6;HOXC8;HOXC9;HOXD1;HOXD10;HOXD11;HOXD12;HOXD13;HOXD3;HOXD4;HOXD8;HOXD9;MEOX1;MEOX2;MNX1;PDX1;POU1F1;POU2F1;POU2F2;POU2F3;POU3F1;POU3F2;POU3F3;POU3F4;POU4F1;POU4F2;POU4F3;POU5F1;POU5F2;POU6F1;POU6F2	HOXB8;HOXC8;HOXD8
SOX17_HUMAN.H11MO.0.C	10	0.05847953	0.43259440	17.364.359	0.9197132	1000000,00000000	1,00E+06	SOX-related factors{4.1.1}	Group F{4.1.1.6}	BBX;CIC;HBP1;SOX1;SOX10;SOX11;SOX12;SOX13;SOX14;SOX15;SOX17;SOX18;SOX2;SOX21;SOX3;SOX30;SOX4;SOX5;SOX6;SOX7;SOX8;SOX9;SRY	SOX17;SOX18;SOX7
COT1_HUMAN.H11MO.1.C	28	0.16374269	0.43165471	10.141.430	0.6726522	54470,28000000	1,87E+05	RXR-related receptors (NR2){2.1.3}	COUP-like receptors (NR2F){2.1.3.5}	HNF4A;HNF4G;NR2C1;NR2C2;NR2E1;NR2E3;NR2F1;NR2F2;NR2F6;RXRA;RXRB;RXRG	NR2F1;NR2F2;NR2F6
ARNT_HUMAN.H11MO.0.B	35	0.20467836	0.43078581	0.9398158	0.6439684	21782,49000000	9,76E+04	PAS domain factors{1.2.5}	Arnt-like factors{1.2.5.2}	AHR;AHRR;ARNT;ARNT2;ARNTL;ARNTL2;CLOCK;EPAS1;HIF1A;HIF3A;NCOA1;NCOA2;NCOA3;NPAS1;NPAS2;NPAS3;NPAS4;SIM1;SIM2;SOHLH1;SOHLH2;TCFL5	ARNT2;ARNT;ARNTL;CLOCK
NDF1_HUMAN.H11MO.0.A	21	0.12280702	0.43030530	11.346.556	0.7157824	177859,20000000	4,08E+05	Tal-related factors{1.2.3}	Neurogenin / Atonal-like factors{1.2.3.4}	ATOH1;ATOH7;ATOH8;BHLHA15;BHLHA9;BHLHE22;BHLHE23;FERD3L;FIGLA;HAND1;HAND2;LYL1;MESP1;MESP2;MSC;MSGN1;NEUROD1;NEUROD2;NEUROD4;NEUROD6;NEUROG1;NEUROG2;NEUROG3;NHLH1;NHLH2;OLIG1;OLIG2;OLIG3;PTF1A;SCX;TAL1;TAL2;TCF15;TCF21;TCF23;TWIST1;TWIST2	ATOH1;BHLHA15;BHLHE22;BHLHE23;NEUROD1;NEUROD2;NEUROG2;OLIG1;OLIG2;OLIG3
ZN322_HUMAN.H11MO.0.B	37	0.21637427	0.42881939	0.9202517	0.6351768	12497,49000000	6,60E+04	More than 3 adjacent zinc finger factors{2.3.3}	ZNF322-like factors{2.3.3.52}	BCL6;BCL6B;CTCF;CTCFL;FEZF1;FEZF2;GFI1;GFI1B;GLI1;GLI2;GLI3;GLI4;GLIS1;GLIS2;GLIS3;HKR1;MTF1;MYNN;MZF1;OSR2;OVOL1;OVOL2;PLAG1;PLAGL1;PLAGL2;PRDM1;PRDM14;PRDM6;SCRT1;SCRT2;SNAI1;SNAI2;SNAI3;WT1;YY1;YY2;ZBTB12;ZBTB14;ZBTB18;ZBTB20;ZBTB26;ZBTB42;ZBTB45;ZBTB47;ZBTB48;ZBTB49;ZBTB6;ZBTB7A;ZBTB7B;ZBTB7C;ZFP14;ZFP2;ZFP28;ZFP30;ZFP37;ZFP42;ZFP64;ZFP69;ZFP69B;ZFP82;ZFP91;ZFX;ZIC1;ZIC2;ZIC3;ZIC4;ZIC5;ZIK1;ZIM3;ZKSCAN1;ZKSCAN2;ZKSCAN3;ZKSCAN4;ZNF121;ZNF124;ZNF133;ZNF136;ZNF138;ZNF14;ZNF140;ZNF143;ZNF146;ZNF148;ZNF155;ZNF157;ZNF160;ZNF169;ZNF175;ZNF177;ZNF18;ZNF180;ZNF181;ZNF2;ZNF20;ZNF212;ZNF213;ZNF214;ZNF221;ZNF222;ZNF223;ZNF224;ZNF225;ZNF226;ZNF227;ZNF229;ZNF230;ZNF232;ZNF233;ZNF234;ZNF235;ZNF24;ZNF25;ZNF250;ZNF257;ZNF26;ZNF260;ZNF263;ZNF264;ZNF268;ZNF274;ZNF276;ZNF28;ZNF280A;ZNF280B;ZNF280C;ZNF280D;ZNF281;ZNF282;ZNF283;ZNF284;ZNF285;ZNF286A;ZNF286B;ZNF3;ZNF30;ZNF300;ZNF302;ZNF317;ZNF32;ZNF320;ZNF322;ZNF324;ZNF324B;ZNF329;ZNF331;ZNF333;ZNF33A;ZNF33B;ZNF343;ZNF345;ZNF347;ZNF350;ZNF354A;ZNF354B;ZNF362;ZNF366;ZNF383;ZNF384;ZNF394;ZNF397;ZNF398;ZNF404;ZNF41;ZNF410;ZNF419;ZNF420;Z	ZNF322

motif	Nº of probes	% of probes	lower OR	upper OR	OR	p.value	FDR	TF family	TF subfamily	TF.family.member	TF.subfamily.member
										NF431;ZNF432;ZNF436;ZNF439;ZNF44;ZNF440;ZNF442;ZNF443;ZNF446;ZNF449;ZNF45;ZNF460;ZNF468;ZNF479;ZNF484;ZNF490;ZNF500;ZNF502;ZNF524;ZNF525;ZNF528;ZNF543;ZNF544;ZNF546;ZNF547;ZNF548;ZNF549;ZNF554;ZNF555;ZNF557;ZNF558;ZNF559;ZNF561;ZNF562;ZNF563;ZNF564;ZNF566;ZNF567;ZNF568;ZNF57;ZNF570;ZNF571;ZNF572;ZNF577;ZNF581;ZNF582;ZNF583;ZNF585A;ZNF586;ZNF589;ZNF595;ZNF599;ZNF600;ZNF605;ZNF607;ZNF611;ZNF613;ZNF614;ZNF615;ZNF616;ZNF619;ZNF620;ZNF621;ZNF625;ZNF627;ZNF649;ZNF652;ZNF653;ZNF665;ZNF667;ZNF669;ZNF670;ZNF672;ZNF679;ZNF680;ZNF683;ZNF689;ZNF692;ZNF701;ZNF705D;ZNF705E;ZNF705G;ZNF708;ZNF709;ZNF71;ZNF710;ZNF713;ZNF721;ZNF727;ZNF729;ZNF736;ZNF75A;ZNF75D;ZNF76;ZNF763;ZNF764;ZNF765;ZNF768;ZNF77;ZNF771;ZNF773;ZNF774;ZNF776;ZNF777;ZNF780A;ZNF780B;ZNF782;ZNF785;ZNF799;ZNF805;ZNF808;ZNF81;ZNF813;ZNF816;ZNF823;ZNF829;ZNF836;ZNF841;ZNF844;ZNF845;ZNF846;ZNF85;ZNF853;ZNF860;ZNF878;ZNF891;ZNF99;ZSCAN16;ZSCAN2;ZSCAN22;ZSCAN23;ZSCAN29;ZSCAN31;ZSCAN32;ZSCAN4;ZSCAN5A;ZSCAN5B;ZSCAN5C;ZSCAN9;ZXDA;ZXDB;ZXDC	
MAZ_HUMAN.H11MO.0.A	75	0.43859649	0.42855732	0.8036347	0.5878332	630,25340000	6,23E+03	Factors with multiple dispersed zinc fingers{2.3.4}	MAZ-like factors{2.3.4.8}	BCL11A;BCL11B;BNC1;BNC2;E4F1;HIC1;HIC2;HINFP;HIVEP1;HIVEP2;HIVEP3;IKZF1;IKZF2;IKZF3;IKZF4;IKZF5;INSM1;INSM2;MAZ;MECOM;PATZ1;PRDM16;PRDM4;REST;RLF;RREB1;SALL1;SALL2;SALL3;SALL4;VEZF1;ZBTB1;ZBTB17;ZBTB2;ZBTB25;ZBTB4;ZFAT;ZNF134;ZNF211;ZNF217;ZNF219;ZNF248;ZNF256;ZNF292;ZNF296;ZNF319;ZNF334;ZNF335;ZNF341;ZNF37A;ZNF382;ZNF417;ZNF418;ZNF423;ZNF467;ZNF510;ZNF512;ZNF512B;ZNF516;ZNF518A;ZNF518B;ZNF521;ZNF526;ZNF532;ZNF536;ZNF552;ZNF574;ZNF587;ZNF587B;ZNF592;ZNF639;ZNF654;ZNF658;ZNF671;ZNF687;ZNF711;ZNF717;ZNF770;ZNF772;ZNF784;ZNF786;ZNF792;ZNF8;ZNF814	MAZ;PATZ1;VEZF1
E2F7_HUMAN.H11MO.0.B	56	0.32748538	0.42855478	0.8342355	0.6009502	1555,75700000	1,18E+04	E2F-related factors{3.3.2}	E2F{3.3.2.1}	E2F1;E2F2;E2F3;E2F4;E2F5;E2F6;E2F7;E2F8;TFDP1;TFDP2	E2F1;E2F2;E2F3;E2F4;E2F5;E2F6;E2F7;E2F8
ZSC22_HUMAN.H11MO.0.C	58	0.33918129	0.42848982	0.8290788	0.5987841	1573,28300000	1,18E+04	More than 3 adjacent zinc finger factors{2.3.3}	unclassified{2.3.3.0}	BCL6B;BCL6;CTCFL;CTCF;FEZF1;GFI1B;GFI1;GLI1;GLI2;GLI3;GLIS1;GLIS2;GLIS3;MTF1;MYNN;MZF1;OSR2;OVOL1;OVOL2;ZNF146;PLAG1;PLAGL1;PRDM14;PRDM1;PRDM6;SCRT1;SCRT2;SNAI1;SNAI2;YY1;YY2;WT1;ZNF324;ZNF354A;ZBTB14;ZBTB18;ZBTB48;ZBTB49;ZBTB7A;ZBTB7B;ZBTB6;ZFP64;ZFP28;ZFP42;ZFP82;ZFX;ZIC1;ZIC2;ZIC3;ZIC4;ZIM3;ZKSCAN1;ZKSCAN3;ZNF121;ZNF136;ZNF140;ZNF143;ZNF148;ZNF214;ZNF232;ZNF250;ZNF257;ZNF260;ZNF263;ZNF264;ZNF274;ZNF281;ZNF282;ZNF317;ZNF320;ZNF322;ZNF329;ZNF331;ZNF333;ZNF350;ZNF384;ZNF394;ZNF410;ZNF436;ZNF449;ZNF490;ZNF502;ZNF524;ZNF528;ZNF547;ZNF549;ZNF554;ZNF563;ZNF582;ZNF586;ZNF589;ZNF652;ZNF667;ZNF680;ZNF708;ZNF713;ZNF768;ZNF816;ZNF18;ZNF41;ZNF76;ZNF85;ZSCAN16;ZSCAN22;ZSCAN31;ZSCAN4	MYNN;MZF1;OSR2;PRDM14;PRDM6;WT1;ZBTB14;ZBTB48;ZBTB49;ZFP64;ZFP28;ZIM3;ZNF121;ZNF250;ZNF257;ZNF263;ZNF274;ZNF317;ZNF320;ZNF329;ZNF331;ZNF394;ZNF449;ZNF502;ZNF528;ZNF547;ZNF549;ZNF554;ZNF586;ZNF589;ZNF667;ZNF680;ZNF708;ZNF713;ZNF768;ZNF18;ZNF85;ZSCAN16;ZSCAN22

motif	Nº of probes	% of probes	lower OR	upper OR	OR	p.value	FDR	TF family	TF subfamily	TF.family.member	TF.subfamily.member
KAISO_HUMAN.H11MO.1.A	24	0.14035088	0.42796603	10.664.944	0.6894091	97948,77000000	2,70E+05	Other factors with up to three adjacent zinc fingers{2.3.2}	Factors with 2-3 adjacent zinc fingersand a BTB/POZ domain{2.3.2.1}	AEBP2;OSR1;OVOL3;ZBTB22;ZBTB32;ZBTB33;ZBTB34;ZBTB37;ZBTB43;ZBTB46;ZBTB5;ZBTB8B;ZFPM2;ZNF174;ZNF396;ZNF414;ZNF446;ZNF487;ZNF511;ZNF580;ZNF705A;ZNF740;ZSCAN1	ZBTB33
ZFP42_HUMAN.H11MO.0.A	13	0.07602339	0.42729551	14.447.369	0.8205166	594583,40000000	7,89E+05	More than 3 adjacent zinc finger factors{2.3.3}	YY1-like factors{2.3.3.9}	BCL6;BCL6B;CTCF;CTCFL;FEZF1;FEZF2;GFI1;GFI1B;GLI1;GLI2;GLI3;GLI4;GLIS1;GLIS2;GLIS3;HKR1;MTF1;MYNN;MZF1;OSR2;OVOL1;OVOL2;PLAG1;PLAGL1;PLAGL2;PRDM1;PRDM14;PRDM6;SCRT1;SCRT2;SNAI1;SNAI2;SNAI3;WT1;YY1;YY2;ZBTB12;ZBTB14;ZBTB18;ZBTB20;ZBTB26;ZBTB42;ZBTB45;ZBTB47;ZBTB48;ZBTB49;ZBTB6;ZBTB7A;ZBTB7B;ZBTB7C;ZFP14;ZFP2;ZFP28;ZFP30;ZFP37;ZFP42;ZFP64;ZFP69;ZFP69B;ZFP82;ZFP91;ZFX;ZIC1;ZIC2;ZIC3;ZIC4;ZIC5;ZIK1;ZIM3;ZKSCAN1;ZKSCAN2;ZKSCAN3;ZKSCAN4;ZNF121;ZNF124;ZNF133;ZNF136;ZNF138;ZNF14;ZNF140;ZNF143;ZNF146;ZNF148;ZNF155;ZNF157;ZNF160;ZNF169;ZNF175;ZNF177;ZNF18;ZNF180;ZNF181;ZNF2;ZNF20;ZNF212;ZNF213;ZNF214;ZNF221;ZNF222;ZNF223;ZNF224;ZNF225;ZNF226;ZNF227;ZNF229;ZNF230;ZNF232;ZNF233;ZNF234;ZNF235;ZNF24;ZNF25;ZNF250;ZNF257;ZNF26;ZNF260;ZNF263;ZNF264;ZNF268;ZNF274;ZNF276;ZNF28;ZNF280A;ZNF280B;ZNF280C;ZNF280D;ZNF281;ZNF282;ZNF283;ZNF284;ZNF285;ZNF286A;ZNF286B;ZNF3;ZNF30;ZNF300;ZNF302;ZNF317;ZNF32;ZNF320;ZNF322;ZNF324;ZNF324B;ZNF329;ZNF331;ZNF333;ZNF33A;ZNF33B;ZNF343;ZNF345;ZNF347;ZNF350;ZNF354A;ZNF354B;ZNF362;ZNF366;ZNF383;ZNF384;ZNF394;ZNF397;ZNF398;ZNF404;ZNF41;ZNF410;ZNF419;ZNF420;ZNF431;ZNF432;ZNF436;ZNF439;ZNF44;ZNF440;ZNF442;ZNF443;ZNF446;ZNF449;ZNF45;ZNF460;ZNF468;ZNF479;ZNF484;ZNF490;ZNF500;ZNF502;ZNF524;ZNF525;ZNF528;ZNF543;ZNF544;ZNF546;ZNF547;ZNF548;ZNF549;ZNF554;ZNF555;ZNF557;ZNF558;ZNF559;ZNF561;ZNF562;ZNF563;ZNF564;ZNF566;ZNF567;ZNF568;ZNF57;ZNF570;ZNF571;ZNF572;ZNF577;ZNF581;ZNF582;ZNF583;ZNF585A;ZNF586;ZNF589;ZNF595;ZNF599;ZNF600;ZNF605;ZNF607;ZNF611;ZNF613;ZNF614;ZNF615;ZNF616;ZNF619;ZNF620;ZNF621;ZNF625;ZNF627;ZNF649;ZNF652;ZNF653;ZNF665;ZNF667;ZNF669;ZNF670;ZNF672;ZNF679;ZNF680;ZNF683;ZNF689;ZNF692;ZNF701;ZNF705D;ZNF705E;ZNF705G;ZNF708;ZNF709;ZNF71;ZNF710;ZNF713;ZNF721;ZNF727;ZNF729;ZNF736;ZNF75A;ZNF75D;ZNF76;ZNF763;ZNF764;ZNF765;ZNF768;ZNF77;ZNF771;ZNF773;ZNF774;ZNF776;ZNF777;ZNF780A;ZNF780B;ZNF782;ZNF785;ZNF799;ZNF805;ZNF808;ZNF81;ZNF813;ZNF816;ZNF823;ZNF829;ZNF836;ZNF841;ZNF844;ZNF845;ZNF846;ZNF85;ZNF853;ZNF860;ZNF878;ZNF891;ZNF99;ZSCAN16;ZSCAN2;ZSCAN22;ZSCAN23;ZSCAN29;ZSCAN31;ZSCAN32;ZSCAN4;ZSCAN5A;ZSCAN5B;ZSCAN5C;ZSCAN9;ZXDA;ZXDB;ZXDC	YY1;YY2;ZFP42
RARB_HUMAN.H11MO.0.D	21	0.12280702	0.42427770	11.187.155	0.7057236	149192,70000000	3,65E+05	Thyroid hormone receptor-related factors (NR1){2.1.2}	Retinoic acid receptors (NR1B){2.1.2.1}	NR1D1;NR1D2;NR1H2;NR1H3;NR1H4;NR1I2;NR1I3;PPARA;PPARD;PPARG;RARA;RARB;RARG;RORA;RORB;RORC;THRA;THRB;VDR	RARA;RARB;RARG
SP2_HUMAN.H11MO.1.B	60	0.35087719	0.42405798	0.8159960	0.5907185	942,31290000	8,23E+03	Three-zinc finger Krüppel-	Sp1-like factors{2.3.1.1}	EGR1;EGR2;EGR3;EGR4;KLF1;KLF10;KLF11;KLF12;KLF13;KLF14;KLF15;KLF16;KLF17;KLF2	SP1;SP2;SP3;SP4

motif	Nº of probes	% of probes	lower OR	upper OR	OR	p.value	FDR	TF family	TF subfamily	TF.family.member	TF.subfamily.member
								related factors {2.3.1}		;KLF3;KLF4;KLF5;KLF6;KLF7;KLF8;KLF9;SP1;SP2;SP3;SP4;SP5;SP6;SP7;SP8;SP9	
OVOL2_HUMAN.H11MO.0.D	11	0.06432749	0.42348261	15.919.191	0.8657343	769457,30000000	8,89E+05	More than 3 adjacent zinc finger factors{2.3.3}	OVOL-factors{2.3.3.17}	BCL6;BCL6B;CTCF;CTCFL;FEZF1;FEZF2;GFI1;GFI1B;GLI1;GLI2;GLI3;GLI4;GLIS1;GLIS2;GLIS3;HKR1;MTF1;MYNN;MZF1;OSR2;OVOL1;OVOL2;PLAG1;PLAGL1;PLAGL2;PRDM1;PRDM14;PRDM6;SCRT1;SCRT2;SNAI1;SNAI2;SNAI3;WT1;YY1;YY2;ZBTB12;ZBTB14;ZBTB18;ZBTB20;ZBTB26;ZBTB42;ZBTB45;ZBTB47;ZBTB48;ZBTB49;ZBTB6;ZBTB7A;ZBTB7B;ZBTB7C;ZFP14;ZFP2;ZFP28;ZFP30;ZFP37;ZFP42;ZFP64;ZFP69;ZFP69B;ZFP82;ZFP91;ZFX;ZIC1;ZIC2;ZIC3;ZIC4;ZIC5;ZIK1;ZIM3;ZKSCAN1;ZKSCAN2;ZKSCAN3;ZKSCAN4;ZNF121;ZNF124;ZNF133;ZNF136;ZNF138;ZNF14;ZNF140;ZNF143;ZNF146;ZNF148;ZNF155;ZNF157;ZNF160;ZNF169;ZNF175;ZNF177;ZNF18;ZNF180;ZNF181;ZNF2;ZNF20;ZNF212;ZNF213;ZNF214;ZNF221;ZNF222;ZNF223;ZNF224;ZNF225;ZNF226;ZNF227;ZNF229;ZNF230;ZNF232;ZNF233;ZNF234;ZNF235;ZNF24;ZNF25;ZNF250;ZNF257;ZNF26;ZNF260;ZNF263;ZNF264;ZNF268;ZNF274;ZNF276;ZNF28;ZNF280A;ZNF280B;ZNF280C;ZNF280D;ZNF281;ZNF282;ZNF283;ZNF284;ZNF285;ZNF286A;ZNF286B;ZNF3;ZNF30;ZNF300;ZNF302;ZNF317;ZNF32;ZNF320;ZNF322;ZNF324;ZNF324B;ZNF329;ZNF331;ZNF333;ZNF33A;ZNF33B;ZNF343;ZNF345;ZNF347;ZNF350;ZNF354A;ZNF354B;ZNF362;ZNF366;ZNF383;ZNF384;ZNF394;ZNF397;ZNF398;ZNF404;ZNF41;ZNF410;ZNF419;ZNF420;ZNF431;ZNF432;ZNF436;ZNF439;ZNF44;ZNF440;ZNF442;ZNF443;ZNF446;ZNF449;ZNF45;ZNF460;ZNF468;ZNF479;ZNF484;ZNF490;ZNF500;ZNF502;ZNF524;ZNF525;ZNF528;ZNF543;ZNF544;ZNF546;ZNF547;ZNF548;ZNF549;ZNF554;ZNF555;ZNF557;ZNF558;ZNF559;ZNF561;ZNF562;ZNF563;ZNF564;ZNF566;ZNF567;ZNF568;ZNF57;ZNF570;ZNF571;ZNF572;ZNF577;ZNF581;ZNF582;ZNF583;ZNF585A;ZNF586;ZNF589;ZNF595;ZNF599;ZNF600;ZNF605;ZNF607;ZNF611;ZNF613;ZNF614;ZNF615;ZNF616;ZNF619;ZNF620;ZNF621;ZNF625;ZNF627;ZNF649;ZNF652;ZNF653;ZNF665;ZNF667;ZNF669;ZNF670;ZNF672;ZNF679;ZNF680;ZNF683;ZNF689;ZNF692;ZNF701;ZNF705D;ZNF705E;ZNF705G;ZNF708;ZNF709;ZNF71;ZNF710;ZNF713;ZNF721;ZNF727;ZNF729;ZNF736;ZNF75A;ZNF75D;ZNF76;ZNF763;ZNF764;ZNF765;ZNF768;ZNF77;ZNF771;ZNF773;ZNF774;ZNF776;ZNF777;ZNF780A;ZNF780B;ZNF782;ZNF785;ZNF799;ZNF805;ZNF808;ZNF81;ZNF813;ZNF816;ZNF823;ZNF829;ZNF836;ZNF841;ZNF844;ZNF845;ZNF846;ZNF85;ZNF853;ZNF860;ZNF878;ZNF891;ZNF99;ZSCAN16;ZSCAN2;ZSCAN22;ZSCAN23;ZSCAN29;ZSCAN31;ZSCAN32;ZSCAN4;ZSCAN5A;ZSCAN5B;ZSCAN5C;ZSCAN9;ZXDA;ZXDB;ZXDC	OVOL1;OVOL2
MEIS1_HUMAN.H11MO.1.B	19	0.11111111	0.42333279	11.683.477	0.7227140	196145,70000000	4,31E+05	TALE-type homeo domain factors{3.1.4}	MEIS{3.1.4.2}	IRX1;IRX2;IRX3;IRX4;IRX5;IRX6;MEIS1;MEIS2;MEIS3;MKX;PBX1;PBX2;PBX3;PKNOX1;PKNOX2;TGIF1;TGIF2;TGIF2LX;TGIF2LY	MEIS1;MEIS2;MEIS3
P63_HUMAN.H11MO.0.A	17	0.09941520	0.42181354	12.294.229	0.7430362	303620,40000000	5,46E+05	p53-related factors {6.3.1}	p63{6.3.1.0.2}	TP53;TP63;TP73	TP63

motif	Nº of probes	% of probes	lower OR	upper OR	OR	p.value	FDR	TF family	TF subfamily	TF.family.member	TF.subfamily.member
BHA15_HUMAN.H11MO.0.B	32	0.18713450	0.42147579	0.9457854	0.6401022	23693,41000000	1,03E+05	Tal-related factors{1.2.3}	Neurogenin / Atonal-like factors{1.2.3.4}	ATOH1;ATOH7;ATOH8;BHLHA15;BHLHA9;BHLHE22;BHLHE23;FERD3L;FIGLA;HAND1;HAND2;LYL1;MESP1;MESP2;MSC;MSGN1;NEUROD1;NEUROD2;NEUROD4;NEUROD6;NEUROG1;NEUROG2;NEUROG3;NHLH1;NHLH2;OLIG1;OLIG2;OLIG3;PTF1A;SCX;TAL1;TAL2;TCF15;TCF21;TCF23;TWIST1;TWIST2	ATOH1;BHLHA15;BHLHE22;BHLHE23;NEUROD1;NEUROD2;NEUROG2;OLIG1;OLIG2;OLIG3
SOX8_HUMAN.H11MO.0.D	8	0.04678363	0.42113780	20.035.600	0.9924276	1000000,00000000	1,00E+06	SOX-related factors{4.1.1}	Group E{4.1.1.5}	BBX;CIC;HBP1;SOX1;SOX10;SOX11;SOX12;SOX13;SOX14;SOX15;SOX17;SOX18;SOX2;SOX21;SOX3;SOX30;SOX4;SOX5;SOX6;SOX7;SOX8;SOX9;SRY	SOX10;SOX8;SOX9
MAZ_HUMAN.H11MO.1.A	56	0.32748538	0.42046839	0.8184929	0.5896035	1179,04400000	9,77E+03	Factors with multiple dispersed zinc fingers{2.3.4}	MAZ-like factors{2.3.4.8}	BCL11A;BCL11B;BNC1;BNC2;E4F1;HIC1;HIC2;HINFP;HIVEP1;HIVEP2;HIVEP3;IKZF1;IKZF2;IKZF3;IKZF4;IKZF5;INSM1;INSM2;MAZ;MECOM;PATZ1;PRDM16;PRDM4;REST;RLF;RREB1;SALL1;SALL2;SALL3;SALL4;VEZF1;ZBTB1;ZBTB17;ZBTB2;ZBTB25;ZBTB4;ZFAT;ZNF134;ZNF211;ZNF217;ZNF219;ZNF248;ZNF256;ZNF292;ZNF296;ZNF319;ZNF334;ZNF335;ZNF341;ZNF37A;ZNF382;ZNF417;ZNF418;ZNF423;ZNF467;ZNF510;ZNF512;ZNF512B;ZNF516;ZNF518A;ZNF518B;ZNF521;ZNF526;ZNF532;ZNF536;ZNF552;ZNF574;ZNF587;ZNF587B;ZNF592;ZNF639;ZNF654;ZNF658;ZNF671;ZNF687;ZNF711;ZNF717;ZNF770;ZNF772;ZNF784;ZNF786;ZNF792;ZNF8;ZNF814	MAZ;PATZ1;VEZF1
GLIS1_HUMAN.H11MO.0.D	22	0.12865497	0.42044917	10.863.897	0.6913439	108422,50000000	2,87E+05	More than 3 adjacent zinc finger factors{2.3.3}	GLI-like factors{2.3.3.1}	BCL6;BCL6B;CTCF;CTCFL;FEZF1;FEZF2;GFI1;GFI1B;GLI1;GLI2;GLI3;GLI4;GLIS1;GLIS2;GLIS3;HKR1;MTF1;MYNN;MZF1;OSR2;OVOL1;OVOL2;PLAG1;PLAGL1;PLAGL2;PRDM1;PRDM14;PRDM6;SCRT1;SCRT2;SNAI1;SNAI2;SNAI3;WT1;YY1;YY2;ZBTB12;ZBTB14;ZBTB18;ZBTB20;ZBTB26;ZBTB42;ZBTB45;ZBTB47;ZBTB48;ZBTB49;ZBTB6;ZBTB7A;ZBTB7B;ZBTB7C;ZFP14;ZFP2;ZFP28;ZFP30;ZFP37;ZFP42;ZFP64;ZFP69;ZFP69B;ZFP82;ZFP91;ZFX;ZIC1;ZIC2;ZIC3;ZIC4;ZIC5;ZIK1;ZIM3;ZKSCAN1;ZKSCAN2;ZKSCAN3;ZKSCAN4;ZNF121;ZNF124;ZNF133;ZNF136;ZNF138;ZNF14;ZNF140;ZNF143;ZNF146;ZNF148;ZNF155;ZNF157;ZNF160;ZNF169;ZNF175;ZNF177;ZNF18;ZNF180;ZNF181;ZNF2;ZNF20;ZNF212;ZNF213;ZNF214;ZNF221;ZNF222;ZNF223;ZNF224;ZNF225;ZNF226;ZNF227;ZNF229;ZNF230;ZNF232;ZNF233;ZNF234;ZNF235;ZNF24;ZNF25;ZNF250;ZNF257;ZNF26;ZNF260;ZNF263;ZNF264;ZNF268;ZNF274;ZNF276;ZNF28;ZNF280A;ZNF280B;ZNF280C;ZNF280D;ZNF281;ZNF282;ZNF283;ZNF284;ZNF285;ZNF286A;ZNF286B;ZNF3;ZNF30;ZNF300;ZNF302;ZNF317;ZNF32;ZNF320;ZNF322;ZNF324;ZNF324B;ZNF329;ZNF331;ZNF333;ZNF33A;ZNF33B;ZNF343;ZNF345;ZNF347;ZNF350;ZNF354A;ZNF354B;ZNF362;ZNF366;ZNF383;ZNF384;ZNF394;ZNF397;ZNF398;ZNF404;ZNF41;ZNF410;ZNF419;ZNF420;ZNF431;ZNF432;ZNF436;ZNF439;ZNF44;ZNF440;ZNF442;ZNF443;ZNF446;ZNF449;ZNF45;ZNF460;ZNF468;ZNF479;ZNF484;ZNF490;ZNF500;ZNF502;ZNF524;ZNF525;ZNF528;ZNF543;ZNF544;ZNF546;ZNF547;ZNF548;ZNF549;ZNF554;ZNF555;ZNF557;ZNF558;ZNF559;ZNF561;ZNF562;ZNF563;ZNF564;ZNF566;ZNF567;ZNF568;ZNF57;ZNF570;ZNF571;ZNF572;ZNF577;ZNF581;ZNF582;ZNF583;ZNF585A;ZNF586;ZNF589;ZNF595;ZNF599;ZNF600;ZNF605;ZNF607;ZNF611;ZNF613;ZNF614;ZNF615;ZNF616;ZNF619;ZNF620;ZNF621;ZNF625;ZNF627;ZNF649;ZNF652;ZNF6	GLI1;GLI2;GLI3;GLIS1;GLIS2;GLIS3;ZIC1;ZIC2;ZIC3;ZIC4

motif	Nº of probes	% of probes	lower OR	upper OR	OR	p.value	FDR	TF family	TF subfamily	TF.family.member	TF.subfamily.member
										53;ZNF665;ZNF667;ZNF669;ZNF670;ZNF672;ZNF679;ZNF680;ZNF683;ZNF689;ZNF692;ZNF701;ZNF705D;ZNF705E;ZNF705G;ZNF708;ZNF709;ZNF71;ZNF710;ZNF713;ZNF721;ZNF727;ZNF729;ZNF736;ZNF75A;ZNF75D;ZNF76;ZNF763;ZNF764;ZNF765;ZNF768;ZNF77;ZNF771;ZNF773;ZNF774;ZNF776;ZNF777;ZNF780A;ZNF780B;ZNF782;ZNF785;ZNF799;ZNF805;ZNF808;ZNF81;ZNF813;ZNF816;ZNF823;ZNF829;ZNF836;ZNF841;ZNF844;ZNF845;ZNF846;ZNF85;ZNF853;ZNF860;ZNF878;ZNF891;ZNF99;ZSCAN16;ZSCAN2;ZSCAN22;ZSCAN23;ZSCAN29;ZSCAN31;ZSCAN32;ZSCAN4;ZSCAN5A;ZSCAN5B;ZSCAN5C;ZSCAN9;ZXDA;ZXDB;ZXDC	
E2F6_HUMAN.H11MO.0.A	50	0.29239766	0.42029222	0.8363611	0.5967549	1796,53300000	1,31E+04	E2F-related factors{3.3.2}	E2F{3.3.2.1}	E2F1;E2F2;E2F3;E2F4;E2F5;E2F6;E2F7;E2F8;TFDP1;TFDP2	E2F1;E2F2;E2F3;E2F4;E2F5;E2F6;E2F7;E2F8
HES7_HUMAN.H11MO.0.D	26	0.15204678	0.41852652	10.107.298	0.6623675	60893,65000000	2,00E+05	Hairy-related factors{1.2.4}	Hairy-like factors{1.2.4.1}	BHLHE40;BHLHE41;HELT;HES1;HES2;HES3;HES4;HES5;HES6;HES7;HEY1;HEY2;HEYL	BHLHE40;BHLHE41;HES1;HES5;HES7;HEY1;HEY2
ZBT17_HUMAN.H11MO.0.A	63	0.36842105	0.41814608	0.7989245	0.5801233	543,23800000	5,72E+03	Factors with multiple dispersed zinc fingers{2.3.4}	unclassified{2.3.4.0}	BCL11A;E4F1;MECOM;HIC1;HIC2;HINFP;IKZF1;INSM1;MAZ;PATZ1;PRDM4;REST;RREB1;SALL4;VEZF1;ZBTB17;ZBTB4;HIVEP1;HIVEP2;ZNF134;ZNF219;ZNF335;ZNF341;ZNF382;ZNF418;ZNF423;ZNF467;ZNF770;ZNF784;ZNF8	E4F1;PRDM4;REST;RREB1;ZBTB17;ZBTB4;ZNF335;ZNF341;ZNF467;ZNF770;ZNF784;ZNF8
EGR4_HUMAN.H11MO.0.D	60	0.35087719	0.41792462	0.8041818	0.5821735	709,54380000	6,75E+03	Three-zinc finger Krüppel-related factors{2.3.1}	EGR factors{2.3.1.3}	EGR1;EGR2;EGR3;EGR4;KLF1;KLF10;KLF11;KLF12;KLF13;KLF14;KLF15;KLF16;KLF17;KLF2;KLF3;KLF4;KLF5;KLF6;KLF7;KLF8;KLF9;SP1;SP2;SP3;SP4;SP5;SP6;SP7;SP8;SP9	EGR1;EGR2;EGR3;EGR4
NRF1_HUMAN.H11MO.0.A	42	0.24561404	0.41576400	0.8618339	0.6040076	3797,51000000	2,52E+04	NRF{0.0.6}	NRF-1 (alpha-pal){0.0.6.0.1}	NRF1	NRF1
PPARA_HUMAN.H11MO.0.B	14	0.08187135	0.41566713	13.455.068	0.7781321	449009,60000000	6,77E+05	Thyroid hormone receptor-related factors (NR1){2.1.2}	PPAR (NR1C){2.1.2.5}	NR1D1;NR1D2;NR1H2;NR1H3;NR1H4;NR1I2;NR1I3;PPARA;PPARD;PPARG;RARA;RARB;RARG;RORA;RORB;RORC;THRA;THRB;VDR	PPARA;PPARD;PPARG
SP4_HUMAN.H11MO.0.A	72	0.42105263	0.41564606	0.7819901	0.5713260	277,76550000	3,76E+03	Three-zinc finger Krüppel-related factors{2.3.1}	Sp1-like factors{2.3.1.1}	EGR1;EGR2;EGR3;EGR4;KLF1;KLF10;KLF11;KLF12;KLF13;KLF14;KLF15;KLF16;KLF17;KLF2;KLF3;KLF4;KLF5;KLF6;KLF7;KLF8;KLF9;SP1;SP2;SP3;SP4;SP5;SP6;SP7;SP8;SP9	SP1;SP2;SP3;SP4
CREM_HUMAN.H11MO.0.C	16	0.09356725	0.41405657	12.452.374	0.7426867	293118,10000000	5,31E+05	CREB-related factors{1.1.7}	CREB-like factors{1.1.7.1}	ATF1;ATF6;ATF6B;CREB1;CREB3;CREB3L1;CREB3L2;CREB3L3;CREB3L4;CREBL2;CREBZF;CREM	ATF1;CREB1;CREM

motif	Nº of probes	% of probes	lower OR	upper OR	OR	p.value	FDR	TF family	TF subfamily	TF.family.member	TF.subfamily.member
ZN329_HUMAN.H11MO.0.C	17	0.09941520	0.41245981	12.021.575	0.7265494	257040,80000000	4,90E+05	More than 3 adjacent zinc finger factors{2.3.3}	unclassified{2.3.3.0}	BCL6B;BCL6;CTCFL;CTCF;FEZF1;GFI1B;GFI1;GLI1;GLI2;GLI3;GLIS1;GLIS2;GLIS3;MTF1;MYNN;MZF1;OSR2;OVOL1;OVOL2;ZNF146;PLAG1;PLAGL1;PRDM14;PRDM1;PRDM6;SCRT1;SCRT2;SNAI1;SNAI2;YY1;YY2;WT1;ZNF324;ZNF354A;ZBTB14;ZBTB18;ZBTB48;ZBTB49;ZBTB7A;ZBTB7B;ZBTB6;ZFP64;ZFP28;ZFP42;ZFP82;ZFX;ZIC1;ZIC2;ZIC3;ZIC4;ZIM3;ZKSCAN1;ZKSCAN3;ZNF121;ZNF136;ZNF140;ZNF143;ZNF148;ZNF214;ZNF232;ZNF250;ZNF257;ZNF260;ZNF263;ZNF264;ZNF274;ZNF281;ZNF282;ZNF317;ZNF320;ZNF322;ZNF329;ZNF331;ZNF333;ZNF350;ZNF384;ZNF394;ZNF410;ZNF436;ZNF449;ZNF490;ZNF502;ZNF524;ZNF528;ZNF547;ZNF549;ZNF554;ZNF563;ZNF582;ZNF586;ZNF589;ZNF652;ZNF667;ZNF680;ZNF708;ZNF713;ZNF768;ZNF816;ZNF18;ZNF41;ZNF76;ZNF85;ZSCAN16;ZSCAN22;ZSCAN31;ZSCAN4	MYNN;MZF1;OSR2;PRDM14;PRDM6;WT1;ZBTB14;ZBTB48;ZBTB49;ZFP64;ZFP28;ZIM3;ZNF121;ZNF250;ZNF257;ZNF263;ZNF274;ZNF317;ZNF320;ZNF329;ZNF331;ZNF394;ZNF449;ZNF502;ZNF528;ZNF547;ZNF549;ZNF554;ZNF586;ZNF589;ZNF667;ZNF680;ZNF708;ZNF713;ZNF768;ZNF18;ZNF85;ZSCAN16;ZSCAN22
ERR2_HUMAN.H11MO.0.A	12	0.07017544	0.41056968	14.584.247	0.8116386	583262,60000000	7,85E+05	Steroid hormone receptors (NR3){2.1.1}	ER-like receptors (NR3A&B){2.1.1.2}	AR;ESR1;ESR2;ESRRA;ESRRB;ESRRG;NR3C1;NR3C2;PGR	ESRRA;ESRRB;ESRRG;ESR1;ESR2
HIC2_HUMAN.H11MO.0.D	19	0.11111111	0.41049515	11.329.817	0.7008329	164518,60000000	3,87E+05	Factors with multiple dispersed zinc fingers{2.3.4}	Hypermethylated in Cancer proteins{2.3.4.17}	BCL11A;BCL11B;BNC1;BNC2;E4F1;HIC1;HIC2;HINFP;HIVEP1;HIVEP2;HIVEP3;IKZF1;IKZF2;IKZF3;IKZF4;IKZF5;INSM1;INSM2;MAZ;MECOM;PATZ1;PRDM16;PRDM4;REST;RLF;RREB1;SALL1;SALL2;SALL3;SALL4;VEZF1;ZBTB1;ZBTB17;ZBTB2;ZBTB25;ZBTB4;ZFAT;ZNF134;ZNF211;ZNF217;ZNF219;ZNF248;ZNF256;ZNF292;ZNF296;ZNF319;ZNF334;ZNF335;ZNF341;ZNF37A;ZNF382;ZNF417;ZNF418;ZNF423;ZNF467;ZNF510;ZNF512;ZNF512B;ZNF516;ZNF518A;ZNF518B;ZNF521;ZNF526;ZNF532;ZNF536;ZNF552;ZNF574;ZNF587;ZNF587B;ZNF592;ZNF639;ZNF654;ZNF658;ZNF671;ZNF687;ZNF711;ZNF717;ZNF770;ZNF772;ZNF784;ZNF786;ZNF792;ZNF8;ZNF814	HIC1;HIC2
SALL4_HUMAN.H11MO.0.B	48	0.28070175	0.40945073	0.8219757	0.5842406	1311,42100000	1,05E+04	Factors with multiple dispersed zinc fingers{2.3.4}	Sal-like factors{2.3.4.3}	BCL11A;BCL11B;BNC1;BNC2;E4F1;HIC1;HIC2;HINFP;HIVEP1;HIVEP2;HIVEP3;IKZF1;IKZF2;IKZF3;IKZF4;IKZF5;INSM1;INSM2;MAZ;MECOM;PATZ1;PRDM16;PRDM4;REST;RLF;RREB1;SALL1;SALL2;SALL3;SALL4;VEZF1;ZBTB1;ZBTB17;ZBTB2;ZBTB25;ZBTB4;ZFAT;ZNF134;ZNF211;ZNF217;ZNF219;ZNF248;ZNF256;ZNF292;ZNF296;ZNF319;ZNF334;ZNF335;ZNF341;ZNF37A;ZNF382;ZNF417;ZNF418;ZNF423;ZNF467;ZNF510;ZNF512;ZNF512B;ZNF516;ZNF518A;ZNF518B;ZNF521;ZNF526;ZNF532;ZNF536;ZNF552;ZNF574;ZNF587;ZNF587B;ZNF592;ZNF639;ZNF654;ZNF658;ZNF671;ZNF687;ZNF711;ZNF717;ZNF770;ZNF772;ZNF784;ZNF786;ZNF792;ZNF8;ZNF814	SALL4
SOX10_HUMAN.H11MO.0.B	9	0.05263158	0.40890501	17.730.955	0.9107344	1000000,00000000	1,00E+06	SOX-related factors{4.1.1}	Group E{4.1.1.5}	BBX;CIC;HBP1;SOX1;SOX10;SOX11;SOX12;SOX13;SOX14;SOX15;SOX17;SOX18;SOX2;SOX21;SOX3;SOX30;SOX4;SOX5;SOX6;SOX7;SOX8;SOX9;SRY	SOX10;SOX8;SOX9

motif	Nº of probes	% of probes	lower OR	upper OR	OR	p.value	FDR	TF family	TF subfamily	TF.family.member	TF.subfamily.member
ZN263_HUMAN.H11MO.0.A	66	0.38596491	0.40793995	0.7746214	0.5638882	223,46210000	3,21E+03	More than 3 adjacent zinc finger factors{2.3.3}	unclassified{2.3.3.0}	BCL6B;BCL6;CTCFL;CTCF;FEZF1;GFI1B;GFI1;GLI1;GLI2;GLI3;GLIS1;GLIS2;GLIS3;MTF1;MYNN;MZF1;OSR2;OVOL1;OVOL2;ZNF146;PLAG1;PLAGL1;PRDM14;PRDM1;PRDM6;SCRT1;SCRT2;SNAI1;SNAI2;YY1;YY2;WT1;ZNF324;ZNF354A;ZBTB14;ZBTB18;ZBTB48;ZBTB49;ZBTB7A;ZBTB7B;ZBTB6;ZFP64;ZFP28;ZFP42;ZFP82;ZFX;ZIC1;ZIC2;ZIC3;ZIC4;ZIM3;ZKSCAN1;ZKSCAN3;ZNF121;ZNF136;ZNF140;ZNF143;ZNF148;ZNF214;ZNF232;ZNF250;ZNF257;ZNF260;ZNF263;ZNF264;ZNF274;ZNF281;ZNF282;ZNF317;ZNF320;ZNF322;ZNF329;ZNF331;ZNF333;ZNF350;ZNF384;ZNF394;ZNF410;ZNF436;ZNF449;ZNF490;ZNF502;ZNF524;ZNF528;ZNF547;ZNF549;ZNF554;ZNF563;ZNF582;ZNF586;ZNF589;ZNF652;ZNF667;ZNF680;ZNF708;ZNF713;ZNF768;ZNF816;ZNF18;ZNF41;ZNF76;ZNF85;ZSCAN16;ZSCAN22;ZSCAN31;ZSCAN4	MYNN;MZF1;OSR2;PRDM14;PRDM6;WT1;ZBTB14;ZBTB48;ZBTB49;ZFP64;ZFP28;ZIM3;ZNF121;ZNF250;ZNF257;ZNF263;ZNF274;ZNF317;ZNF320;ZNF329;ZNF331;ZNF394;ZNF449;ZNF502;ZNF528;ZNF547;ZNF549;ZNF554;ZNF586;ZNF589;ZNF667;ZNF680;ZNF708;ZNF713;ZNF768;ZNF18;ZNF85;ZSCAN16;ZSCAN22
RFX2_HUMAN.H11MO.0.A	15	0.08771930	0.40727402	12.681.634	0.7453736	335105,80000000	5,86E+05	RFX-related factors{3.3.3}	RFX2{3.3.3.0.2}	RFX1;RFX2;RFX3;RFX4;RFX5;RFX6;RFX7;RFX8	RFX2
VEZF1_HUMAN.H11MO.1.C	45	0.26315789	0.40461575	0.8244963	0.5822096	1546,08900000	1,18E+04	Factors with multiple dispersed zinc fingers{2.3.4}	MAZ-like factors{2.3.4.8}	BCL11A;BCL11B;BNC1;BNC2;E4F1;HIC1;HIC2;HINFP;HIVEP1;HIVEP2;HIVEP3;IKZF1;IKZF2;IKZF3;IKZF4;IKZF5;INSM1;INSM2;MAZ;MECOM;PATZ1;PRDM16;PRDM4;REST;RLF;RREB1;SALL1;SALL2;SALL3;SALL4;VEZF1;ZBTB1;ZBTB17;ZBTB2;ZBTB25;ZBTB4;ZFAT;ZNF134;ZNF211;ZNF217;ZNF219;ZNF248;ZNF256;ZNF292;ZNF296;ZNF319;ZNF334;ZNF335;ZNF341;ZNF37A;ZNF382;ZNF417;ZNF418;ZNF423;ZNF467;ZNF510;ZNF512;ZNF512B;ZNF516;ZNF518A;ZNF518B;ZNF521;ZNF526;ZNF532;ZNF536;ZNF552;ZNF574;ZNF587;ZNF587B;ZNF592;ZNF639;ZNF654;ZNF658;ZNF671;ZNF687;ZNF711;ZNF717;ZNF770;ZNF772;ZNF784;ZNF786;ZNF792;ZNF8;ZNF814	MAZ;PATZ1;VEZF1
E2F1_HUMAN.H11MO.0.A	52	0.30409357	0.40380924	0.7971748	0.5707875	640,30640000	6,25E+03	E2F-related factors{3.3.2}	E2F{3.3.2.1}	E2F1;E2F2;E2F3;E2F4;E2F5;E2F6;E2F7;E2F8;TFDP1;TFDP2	E2F1;E2F2;E2F3;E2F4;E2F5;E2F6;E2F7;E2F8
REST_HUMAN.H11MO.0.A	28	0.16374269	0.40282035	0.9464154	0.6277318	24217,34000000	1,04E+05	Factors with multiple dispersed zinc fingers{2.3.4}	unclassified{2.3.4.0}	BCL11A;E4F1;MECOM;HIC1;HIC2;HINFP;IKZF1;INSM1;MAZ;PATZ1;PRDM4;REST;RREB1;SALL4;VEZF1;ZBTB17;ZBTB4;HIVEP1;HIVEP2;ZNF134;ZNF219;ZNF335;ZNF341;ZNF382;ZNF418;ZNF423;ZNF467;ZNF770;ZNF784;ZNF8	E4F1;PRDM4;REST;RREB1;ZBTB17;ZBTB4;ZNF335;ZNF341;ZNF467;ZNF770;ZNF784;ZNF8
HXB1_HUMAN.H11MO.0.D	8	0.04678363	0.40240373	19.143.354	0.9483029	1000000,00000000	1,00E+06	HOX-related factors{3.1.1}	HOX1{3.1.1.1}	CDX1;CDX2;CDX4;EVX1;EVX2;GBX1;GBX2;GSX1;GSX2;HDX;HMBOX1;HNF1A;HNF1B;HOXA1;HOXA10;HOXA11;HOXA13;HOXA2;HOXA3;HOXA4;HOXA5;HOXA6;HOXA7;HOXA9;HOXB1;HOXB13;HOXB2;HOXB3;HOXB4;HOXB5;HOXB6;HOXB7;HOXB8;HOXB9;HOXC10;HOXC11;HOXC12;HOXC13;HOXC4;HOXC5;HOXC6;HOXC8;HOXC9;HOXD1;HOXD10;HOXD11;HOXD12;HOXD13;HOXD3;HOXD4;HOXD8;HOXD9;MEOX1;MEOX2;MNX1;PDX1;POU1F1;POU2F1;POU2F2;POU2F3;POU3F1;POU3F2;POU3F3;POU3F4;POU4F1;POU4F2;POU4F3;POU5F1;POU5F2;POU6F1;POU6F2	HOXA1;HOXB1
SP4_HUMAN.H11MO.1.A	61	0.35672515	0.40216342	0.7719650	0.5594369	225,11940000	3,21E+03	Three-zinc finger Krüppel-related factors{2.3.1}	Sp1-like factors{2.3.1.1}	EGR1;EGR2;EGR3;EGR4;KLF1;KLF10;KLF11;KLF12;KLF13;KLF14;KLF15;KLF16;KLF17;KLF2;KLF3;KLF4;KLF5;KLF6;KLF7;KLF8;KLF9;SP1;SP2;SP3;SP4;SP5;SP6;SP7;SP8;SP9	SP1;SP2;SP3;SP4

motif	Nº of probes	% of probes	lower OR	upper OR	OR	p.value	FDR	TF family	TF subfamily	TF.family.member	TF.subfamily.member
ZN589_HUMAN.H11MO.0.D	16	0.09356725	0.40208801	12.092.147	0.7211948	247240,80000000	4,81E+05	More than 3 adjacent zinc finger factors{2.3.3}	unclassified{2.3.3.0}	BCL6B;BCL6;CTCFL;CTCF;FEZF1;GFI1B;GFI1;GLI1;GLI2;GLI3;GLIS1;GLIS2;GLIS3;MTF1;MYNN;MZF1;OSR2;OVOL1;OVOL2;ZNF146;PLAG1;PLAGL1;PRDM14;PRDM1;PRDM6;SCRT1;SCRT2;SNAI1;SNAI2;YY1;YY2;WT1;ZNF324;ZNF354A;ZBTB14;ZBTB18;ZBTB48;ZBTB49;ZBTB7A;ZBTB7B;ZBTB6;ZFP64;ZFP28;ZFP42;ZFP82;ZFX;ZIC1;ZIC2;ZIC3;ZIC4;ZIM3;ZKSCAN1;ZKSCAN3;ZNF121;ZNF136;ZNF140;ZNF143;ZNF148;ZNF214;ZNF232;ZNF250;ZNF257;ZNF260;ZNF263;ZNF264;ZNF274;ZNF281;ZNF282;ZNF317;ZNF320;ZNF322;ZNF329;ZNF331;ZNF333;ZNF350;ZNF384;ZNF394;ZNF410;ZNF436;ZNF449;ZNF490;ZNF502;ZNF524;ZNF528;ZNF547;ZNF549;ZNF554;ZNF563;ZNF582;ZNF586;ZNF589;ZNF652;ZNF667;ZNF680;ZNF708;ZNF713;ZNF768;ZNF816;ZNF18;ZNF41;ZNF76;ZNF85;ZSCAN16;ZSCAN22;ZSCAN31;ZSCAN4	MYNN;MZF1;OSR2;PRDM14;PRDM6;WT1;ZBTB14;ZBTB48;ZBTB49;ZFP64;ZFP28;ZIM3;ZNF121;ZNF250;ZNF257;ZNF263;ZNF274;ZNF317;ZNF320;ZNF329;ZNF331;ZNF394;ZNF449;ZNF502;ZNF528;ZNF547;ZNF549;ZNF554;ZNF586;ZNF589;ZNF667;ZNF680;ZNF708;ZNF713;ZNF768;ZNF18;ZNF85;ZSCAN16;ZSCAN22
COT2_HUMAN.H11MO.1.A	22	0.12865497	0.40189699	10.384.227	0.6608047	74117,05000000	2,22E+05	RXR-related receptors (NR2){2.1.3}	COUP-like receptors (NR2F){2.1.3.5}	HNF4A;HNF4G;NR2C1;NR2C2;NR2E1;NR2E3;NR2F1;NR2F2;NR2F6;RXRA;RXRB;RXRG	NR2F1;NR2F2;NR2F6
FIGLA_HUMAN.H11MO.0.D	17	0.09941520	0.40157532	11.704.067	0.7073531	216737,70000000	4,44E+05	Tal-related factors{1.2.3}	Twist-like factors{1.2.3.2}	ATOH1;ATOH7;ATOH8;BHLHA15;BHLHA9;BHLHE22;BHLHE23;FERD3L;FIGLA;HAND1;HAND2;LYL1;MESP1;MESP2;MSC;MSGN1;NEUROD1;NEUROD2;NEUROD4;NEUROD6;NEUROG1;NEUROG2;NEUROG3;NHLH1;NHLH2;OLIG1;OLIG2;OLIG3;PTF1A;SCX;TAL1;TAL2;TCF15;TCF21;TCF23;TWIST1;TWIST2	FIGLA;HAND1;PTF1A;TWIST1
NOTO_HUMAN.H11MO.0.D	7	0.04093567	0.40070218	21.358.826	1,01E+07	846841,90000000	9,46E+05	NK-related factors{3.1.2}	NOTO{3.1.2.20}	BARHL1;BARHL2;BARX1;BARX2;BSX;DBX1;DBX2;DLX1;DLX2;DLX3;DLX4;DLX5;DLX6;EMX1;EMX2;EN1;EN2;HHEX;HLX;HMX1;HMX2;HMX3;LBX1;LBX2;MSX1;MSX2;NANOG;NKX1-1;NKX1-2;NKX2-1;NKX2-2;NKX2-3;NKX2-4;NKX2-5;NKX2-6;NKX2-8;NKX3-1;NKX3-2;NKX6-1;NKX6-2;NKX6-3;NOTO;TLX1;TLX2;TLX3;VAX1;VAX2;VENTX	NOTO
MYBB_HUMAN.H11MO.0.D	8	0.04678363	0.40022285	19.040.363	0.9431649	1000000,00000000	1,00E+06	Myb/SANT domain factors{3.5.1}	Myb-like factors{3.5.1.1}	CDC5L;DMTF1;MYB;MYBL1;MYBL2;SMARCA1;SMARCA5;SNAPC4;TRERF1;ZNF541	CDC5L;MYBL1;MYBL2;MYB
ZEP2_HUMAN.H11MO.0.D	19	0.11111111	0.39935160	11.021.797	0.6817781	137630,80000000	3,42E+05	Factors with multiple dispersed zinc fingers{2.3.4}	HIV EP-factors{2.3.4.5}	BCL11A;BCL11B;BNC1;BNC2;E4F1;HIC1;HIC2;HINFP;HIVEP1;HIVEP2;HIVEP3;IKZF1;IKZF2;IKZF3;IKZF4;IKZF5;INSM1;INSM2;MAZ;MECOM;PATZ1;PRDM16;PRDM4;REST;RLF;RREB1;SALL1;SALL2;SALL3;SALL4;VEZF1;ZBTB1;ZBTB17;ZBTB2;ZBTB25;ZBTB4;ZFAT;ZNF134;ZNF211;ZNF217;ZNF219;ZNF248;ZNF256;ZNF292;ZNF296;ZNF319;ZNF334;ZNF335;ZNF341;ZNF37A;ZNF382;ZNF417;ZNF418;ZNF423;ZNF467;ZNF510;ZNF512;ZNF512B;ZNF516;ZNF518A;ZNF518B;ZNF521;ZNF526;ZNF532;ZNF536;ZNF552;ZNF574;ZNF587;ZNF587B;ZNF592;ZNF639;ZNF654;ZNF658;ZNF671;ZNF687;ZNF711;ZNF717;ZNF770;ZNF772;ZNF784;ZNF786;ZNF792;ZNF8;ZNF814	HIVEP1;HIVEP2
EGR2_HUMAN.H11MO.1.A	60	0.35087719	0.39902722	0.7679360	0.5559206	220,28500000	3,21E+03	Three-zinc finger Krüppel-related factors{2.3.1}	EGR factors{2.3.1.3}	EGR1;EGR2;EGR3;EGR4;KLF1;KLF10;KLF11;KLF12;KLF13;KLF14;KLF15;KLF16;KLF17;KLF2;KLF3;KLF4;KLF5;KLF6;KLF7;KLF8;KLF9;SP1;SP2;SP3;SP4;SP5;SP6;SP7;SP8;SP9	EGR1;EGR2;EGR3;EGR4
BRCA1_HUMAN.H11MO.0.D	10	0.05847953	0.39867574	16.001.725	0.8475408	761007,30000000	8,88E+05	*NA*	*NA*	BRCA1	BRCA1

motif	Nº of probes	% of probes	lower OR	upper OR	OR	p.value	FDR	TF family	TF subfamily	TF.family.member	TF.subfamily.member
KLF13_HUMAN.H11MO.0.D	38	0.22222222	0.39839533	0.8484582	0.5875942	3212,21300000	2,15E+04	Three-zinc finger Krüppel-related factors{2.3.1}	Krüppel-like factors{2.3.1.2}	EGR1;EGR2;EGR3;EGR4;KLF1;KLF10;KLF11;KLF12;KLF13;KLF14;KLF15;KLF16;KLF17;KLF2;KLF3;KLF4;KLF5;KLF6;KLF7;KLF8;KLF9;SP1;SP2;SP3;SP4;SP5;SP6;SP7;SP8;SP9	KLF12;KLF13;KLF14;KLF15;KLF16;KLF1;KLF3;KLF4;KLF5;KLF6;KLF8;KLF9
AP2A_HUMAN.H11MO.0.A	23	0.13450292	0.39766899	10.083.726	0.6469354	52456,58000000	1,83E+05	AP-2{1.3.1}	AP-2alpha{1.3.1.0.1}	TFAP2A;TFAP2B;TFAP2C;TFAP2D	TFAP2A
ZN740_HUMAN.H11MO.0.D	27	0.15789474	0.39645251	0.9439232	0.6224393	22962,98000000	1,01E+05	Other factors with up to three adjacent zinc fingers{2.3.2}	Other three adjacent zinc finger factors{2.3.2.4}	AEBP2;OSR1;OVOL3;ZBTB22;ZBTB32;ZBTB33;ZBTB34;ZBTB37;ZBTB43;ZBTB46;ZBTB5;ZBTB8B;ZFPM2;ZNF174;ZNF396;ZNF414;ZNF446;ZNF487;ZNF511;ZNF580;ZNF705A;ZNF740;ZSCAN1	ZNF740
CTCFL_HUMAN.H11MO.0.A	49	0.28654971	0.39592164	0.7912565	0.5635067	595,40230000	5,96E+03	More than 3 adjacent zinc finger factors{2.3.3}	CTCF-like factors{2.3.3.50}	BCL6;BCL6B;CTCF;CTCFL;FEZF1;FEZF2;GFI1;GFI1B;GLI1;GLI2;GLI3;GLI4;GLIS1;GLIS2;GLIS3;HKR1;MTF1;MYNN;MZF1;OSR2;OVOL1;OVOL2;PLAG1;PLAGL1;PLAGL2;PRDM1;PRDM14;PRDM6;SCRT1;SCRT2;SNAI1;SNAI2;SNAI3;WT1;YY1;YY2;ZBTB12;ZBTB14;ZBTB18;ZBTB20;ZBTB26;ZBTB42;ZBTB45;ZBTB47;ZBTB48;ZBTB49;ZBTB6;ZBTB7A;ZBTB7B;ZBTB7C;ZFP14;ZFP2;ZFP28;ZFP30;ZFP37;ZFP42;ZFP64;ZFP69;ZFP69B;ZFP82;ZFP91;ZFX;ZIC1;ZIC2;ZIC3;ZIC4;ZIC5;ZIK1;ZIM3;ZKSCAN1;ZKSCAN2;ZKSCAN3;ZKSCAN4;ZNF121;ZNF124;ZNF133;ZNF136;ZNF138;ZNF14;ZNF140;ZNF143;ZNF146;ZNF148;ZNF155;ZNF157;ZNF160;ZNF169;ZNF175;ZNF177;ZNF18;ZNF180;ZNF181;ZNF2;ZNF20;ZNF212;ZNF213;ZNF214;ZNF221;ZNF222;ZNF223;ZNF224;ZNF225;ZNF226;ZNF227;ZNF229;ZNF230;ZNF232;ZNF233;ZNF234;ZNF235;ZNF24;ZNF25;ZNF250;ZNF257;ZNF26;ZNF260;ZNF263;ZNF264;ZNF268;ZNF274;ZNF276;ZNF28;ZNF280A;ZNF280B;ZNF280C;ZNF280D;ZNF281;ZNF282;ZNF283;ZNF284;ZNF285;ZNF286A;ZNF286B;ZNF3;ZNF30;ZNF300;ZNF302;ZNF317;ZNF32;ZNF320;ZNF322;ZNF324;ZNF324B;ZNF329;ZNF331;ZNF333;ZNF33A;ZNF33B;ZNF343;ZNF345;ZNF347;ZNF350;ZNF354A;ZNF354B;ZNF362;ZNF366;ZNF383;ZNF384;ZNF394;ZNF397;ZNF398;ZNF404;ZNF41;ZNF410;ZNF419;ZNF420;ZNF431;ZNF432;ZNF436;ZNF439;ZNF44;ZNF440;ZNF442;ZNF443;ZNF446;ZNF449;ZNF45;ZNF460;ZNF468;ZNF479;ZNF484;ZNF490;ZNF500;ZNF502;ZNF524;ZNF525;ZNF528;ZNF543;ZNF544;ZNF546;ZNF547;ZNF548;ZNF549;ZNF554;ZNF555;ZNF557;ZNF558;ZNF559;ZNF561;ZNF562;ZNF563;ZNF564;ZNF566;ZNF567;ZNF568;ZNF57;ZNF570;ZNF571;ZNF572;ZNF577;ZNF581;ZNF582;ZNF583;ZNF585A;ZNF586;ZNF589;ZNF595;ZNF599;ZNF600;ZNF605;ZNF607;ZNF611;ZNF613;ZNF614;ZNF615;ZNF616;ZNF619;ZNF620;ZNF621;ZNF625;ZNF627;ZNF649;ZNF652;ZNF653;ZNF665;ZNF667;ZNF669;ZNF670;ZNF672;ZNF679;ZNF680;ZNF683;ZNF689;ZNF692;ZNF701;ZNF705D;ZNF705E;ZNF705G;ZNF708;ZNF709;ZNF71;ZNF710;ZNF713;ZNF721;ZNF727;ZNF729;ZNF736;ZNF75A;ZNF75D;ZNF76;ZNF763;ZNF764;ZNF765;ZNF768;ZNF77;ZNF771;ZNF773;ZNF774;ZNF776;ZNF777;ZNF780A;ZNF780B;ZNF782;ZNF785;ZNF799;ZNF805;ZNF808;ZNF81;ZNF813;ZNF816;ZNF823;ZNF829;ZNF836;ZNF841;ZNF844;ZNF845;ZNF846;ZNF85;ZNF853;ZNF860;ZNF878;ZNF891;ZNF99;ZSCAN16;ZSCAN2;ZSCAN22;ZSCAN23;ZSCAN29;ZSCAN31;ZSCAN32;ZSCAN4;ZSCAN5A;ZSCAN5B;ZSCAN5C;ZSCAN9;ZXDA;ZXDB;ZXDC	CTCFL;CTCF

motif	Nº of probes	% of probes	lower OR	upper OR	OR	p.value	FDR	TF family	TF subfamily	TF.family.member	TF.subfamily.member
ZN582_HUMAN.H11MO.0.C	10	0.05847953	0.39574925	15.883.899	0.8413084	761394,00000000	8,88E+05	More than 3 adjacent zinc finger factors{2.3.3}	ZNF620-like factors{2.3.3.35}	BCL6;BCL6B;CTCF;CTCFL;FEZF1;FEZF2;GFI1;GFI1B;GLI1;GLI2;GLI3;GLI4;GLIS1;GLIS2;GLIS3;HKR1;MTF1;MYNN;MZF1;OSR2;OVOL1;OVOL2;PLAG1;PLAGL1;PLAGL2;PRDM1;PRDM14;PRDM6;SCRT1;SCRT2;SNAI1;SNAI2;SNAI3;WT1;YY1;YY2;ZBTB12;ZBTB14;ZBTB18;ZBTB20;ZBTB26;ZBTB42;ZBTB45;ZBTB47;ZBTB48;ZBTB49;ZBTB6;ZBTB7A;ZBTB7B;ZBTB7C;ZFP14;ZFP2;ZFP28;ZFP30;ZFP37;ZFP42;ZFP64;ZFP69;ZFP69B;ZFP82;ZFP91;ZFX;ZIC1;ZIC2;ZIC3;ZIC4;ZIC5;ZIK1;ZIM3;ZKSCAN1;ZKSCAN2;ZKSCAN3;ZKSCAN4;ZNF121;ZNF124;ZNF133;ZNF136;ZNF138;ZNF14;ZNF140;ZNF143;ZNF146;ZNF148;ZNF155;ZNF157;ZNF160;ZNF169;ZNF175;ZNF177;ZNF18;ZNF180;ZNF181;ZNF2;ZNF20;ZNF212;ZNF213;ZNF214;ZNF221;ZNF222;ZNF223;ZNF224;ZNF225;ZNF226;ZNF227;ZNF229;ZNF230;ZNF232;ZNF233;ZNF234;ZNF235;ZNF24;ZNF25;ZNF250;ZNF257;ZNF26;ZNF260;ZNF263;ZNF264;ZNF268;ZNF274;ZNF276;ZNF28;ZNF280A;ZNF280B;ZNF280C;ZNF280D;ZNF281;ZNF282;ZNF283;ZNF284;ZNF285;ZNF286A;ZNF286B;ZNF3;ZNF30;ZNF300;ZNF302;ZNF317;ZNF32;ZNF320;ZNF322;ZNF324;ZNF324B;ZNF329;ZNF331;ZNF333;ZNF33A;ZNF33B;ZNF343;ZNF345;ZNF347;ZNF350;ZNF354A;ZNF354B;ZNF362;ZNF366;ZNF383;ZNF384;ZNF394;ZNF397;ZNF398;ZNF404;ZNF41;ZNF410;ZNF419;ZNF420;ZNF431;ZNF432;ZNF436;ZNF439;ZNF44;ZNF440;ZNF442;ZNF443;ZNF446;ZNF449;ZNF45;ZNF460;ZNF468;ZNF479;ZNF484;ZNF490;ZNF500;ZNF502;ZNF524;ZNF525;ZNF528;ZNF543;ZNF544;ZNF546;ZNF547;ZNF548;ZNF549;ZNF554;ZNF555;ZNF557;ZNF558;ZNF559;ZNF561;ZNF562;ZNF563;ZNF564;ZNF566;ZNF567;ZNF568;ZNF57;ZNF570;ZNF571;ZNF572;ZNF577;ZNF581;ZNF582;ZNF583;ZNF585A;ZNF586;ZNF589;ZNF595;ZNF599;ZNF600;ZNF605;ZNF607;ZNF611;ZNF613;ZNF614;ZNF615;ZNF616;ZNF619;ZNF620;ZNF621;ZNF625;ZNF627;ZNF649;ZNF652;ZNF653;ZNF665;ZNF667;ZNF669;ZNF670;ZNF672;ZNF679;ZNF680;ZNF683;ZNF689;ZNF692;ZNF701;ZNF705D;ZNF705E;ZNF705G;ZNF708;ZNF709;ZNF71;ZNF710;ZNF713;ZNF721;ZNF727;ZNF729;ZNF736;ZNF75A;ZNF75D;ZNF76;ZNF763;ZNF764;ZNF765;ZNF768;ZNF77;ZNF771;ZNF773;ZNF774;ZNF776;ZNF777;ZNF780A;ZNF780B;ZNF782;ZNF785;ZNF799;ZNF805;ZNF808;ZNF81;ZNF813;ZNF816;ZNF823;ZNF829;ZNF836;ZNF841;ZNF844;ZNF845;ZNF846;ZNF85;ZNF853;ZNF860;ZNF878;ZNF891;ZNF99;ZSCAN16;ZSCAN2;ZSCAN22;ZSCAN23;ZSCAN29;ZSCAN31;ZSCAN32;ZSCAN4;ZSCAN5A;ZSCAN5B;ZSCAN5C;ZSCAN9;ZXDA;ZXDB;ZXDC	ZNF582
HES1_HUMAN.H11MO.0.D	38	0.22222222	0.39557765	0.8423781	0.5834098	2531,77600000	1,74E+04	Hairy-related factors{1.2.4}	Hairy-like factors{1.2.4.1}	BHLHE40;BHLHE41;HELT;HES1;HES2;HES3;HES4;HES5;HES6;HES7;HEY1;HEY2;HEYL	BHLHE40;BHLHE41;HES1;HES5;HES7;HEY1;HEY2
CUX2_HUMAN.H11MO.0.D	7	0.04093567	0.39527293	21.069.425	0.9991068	1000000,00000000	1,00E+06	HD-CUT factors{3.1.9}	CUX{3.1.9.2}	CUX1;CUX2;ONECUT1;ONECUT2;ONECUT3;SATB1;SATB2	CUX1;CUX2
KLF12_HUMAN.H11MO.0.C	55	0.32163743	0.39367781	0.7688800	0.5530936	217,51280000	3,21E+03	Three-zinc finger Krüppel-related factors{2.3.1}	Krüppel-like factors{2.3.1.2}	EGR1;EGR2;EGR3;EGR4;KLF1;KLF10;KLF11;KLF12;KLF13;KLF14;KLF15;KLF16;KLF17;KLF2;KLF3;KLF4;KLF5;KLF6;KLF7;KLF8;KLF9;SP1;SP2;SP3;SP4;SP5;SP6;SP7;SP8;SP9	KLF12;KLF13;KLF14;KLF15;KLF16;KLF1;KLF3;KLF4;KLF5;KLF6;KLF8;KLF9

motif	Nº of probes	% of probes	lower OR	upper OR	OR	p.value	FDR	TF family	TF subfamily	TF.family.member	TF.subfamily.member
LHX9_HUMAN.H11MO.0.D	8	0.04678363	0.39349407	18.720.419	0.9273132	1000000,00000000	1,00E+06	HD-LIM factors{3.1.5}	Lhx-2-like factors{3.1.5.3}	ISL1;ISL2;LHX1;LHX2;LHX3;LHX4;LHX5;LHX6;LHX8;LHX9;LMX1A;LMX1B	LHX2;LHX9
PAX7_HUMAN.H11MO.0.D	6	0.03508772	0.39214261	24.136.669	1,08E+07	826880,00000000	9,33E+05	Paired plus homeo domain{3.2.1}	PAX-3/7{3.2.1.1}	PAX3;PAX4;PAX6;PAX7	PAX3;PAX7
GLI1_HUMAN.H11MO.0.D	29	0.16959064	0.39151871	0.9083815	0.6058343	13281,56000000	6,87E+04	More than 3 adjacent zinc finger factors{2.3.3}	GLI-like factors{2.3.3.1}	BCL6;BCL6B;CTCF;CTCFL;FEZF1;FEZF2;GFI1;GFI1B;GLI1;GLI2;GLI3;GLI4;GLIS1;GLIS2;GLIS3;HKR1;MTF1;MYNN;MZF1;OSR2;OVOL1;OVOL2;PLAG1;PLAGL1;PLAGL2;PRDM1;PRDM14;PRDM6;SCRT1;SCRT2;SNAI1;SNAI2;SNAI3;WT1;YY1;YY2;ZBTB12;ZBTB14;ZBTB18;ZBTB20;ZBTB26;ZBTB42;ZBTB45;ZBTB47;ZBTB48;ZBTB49;ZBTB6;ZBTB7A;ZBTB7B;ZBTB7C;ZFP14;ZFP2;ZFP28;ZFP30;ZFP37;ZFP42;ZFP64;ZFP69;ZFP69B;ZFP82;ZFP91;ZFX;ZIC1;ZIC2;ZIC3;ZIC4;ZIC5;ZIK1;ZIM3;ZKSCAN1;ZKSCAN2;ZKSCAN3;ZKSCAN4;ZNF121;ZNF124;ZNF133;ZNF136;ZNF138;ZNF14;ZNF140;ZNF143;ZNF146;ZNF148;ZNF155;ZNF157;ZNF160;ZNF169;ZNF175;ZNF177;ZNF18;ZNF180;ZNF181;ZNF2;ZNF20;ZNF212;ZNF213;ZNF214;ZNF221;ZNF222;ZNF223;ZNF224;ZNF225;ZNF226;ZNF227;ZNF229;ZNF230;ZNF232;ZNF233;ZNF234;ZNF235;ZNF24;ZNF25;ZNF250;ZNF257;ZNF26;ZNF260;ZNF263;ZNF264;ZNF268;ZNF274;ZNF276;ZNF28;ZNF280A;ZNF280B;ZNF280C;ZNF280D;ZNF281;ZNF282;ZNF283;ZNF284;ZNF285;ZNF286A;ZNF286B;ZNF3;ZNF30;ZNF300;ZNF302;ZNF317;ZNF32;ZNF320;ZNF322;ZNF324;ZNF324B;ZNF329;ZNF331;ZNF333;ZNF33A;ZNF33B;ZNF343;ZNF345;ZNF347;ZNF350;ZNF354A;ZNF354B;ZNF362;ZNF366;ZNF383;ZNF384;ZNF394;ZNF397;ZNF398;ZNF404;ZNF41;ZNF410;ZNF419;ZNF420;ZNF431;ZNF432;ZNF436;ZNF439;ZNF44;ZNF440;ZNF442;ZNF443;ZNF446;ZNF449;ZNF45;ZNF460;ZNF468;ZNF479;ZNF484;ZNF490;ZNF500;ZNF502;ZNF524;ZNF525;ZNF528;ZNF543;ZNF544;ZNF546;ZNF547;ZNF548;ZNF549;ZNF554;ZNF555;ZNF557;ZNF558;ZNF559;ZNF561;ZNF562;ZNF563;ZNF564;ZNF566;ZNF567;ZNF568;ZNF57;ZNF570;ZNF571;ZNF572;ZNF577;ZNF581;ZNF582;ZNF583;ZNF585A;ZNF586;ZNF589;ZNF595;ZNF599;ZNF600;ZNF605;ZNF607;ZNF611;ZNF613;ZNF614;ZNF615;ZNF616;ZNF619;ZNF620;ZNF621;ZNF625;ZNF627;ZNF649;ZNF652;ZNF653;ZNF665;ZNF667;ZNF669;ZNF670;ZNF672;ZNF679;ZNF680;ZNF683;ZNF689;ZNF692;ZNF701;ZNF705D;ZNF705E;ZNF705G;ZNF708;ZNF709;ZNF71;ZNF710;ZNF713;ZNF721;ZNF727;ZNF729;ZNF736;ZNF75A;ZNF75D;ZNF76;ZNF763;ZNF764;ZNF765;ZNF768;ZNF77;ZNF771;ZNF773;ZNF774;ZNF776;ZNF777;ZNF780A;ZNF780B;ZNF782;ZNF785;ZNF799;ZNF805;ZNF808;ZNF81;ZNF813;ZNF816;ZNF823;ZNF829;ZNF836;ZNF841;ZNF844;ZNF845;ZNF846;ZNF85;ZNF853;ZNF860;ZNF878;ZNF891;ZNF99;ZSCAN16;ZSCAN2;ZSCAN22;ZSCAN23;ZSCAN29;ZSCAN31;ZSCAN32;ZSCAN4;ZSCAN5A;ZSCAN5B;ZSCAN5C;ZSCAN9;ZXDA;ZXDB;ZXDC	GLI1;GLI2;GLI3;GLIS1;GLIS2;GLIS3;ZIC1;ZIC2;ZIC3;ZIC4
KLF9_HUMAN.H11MO.0.C	55	0.32163743	0.39115559	0.7639525	0.5495489	211,61300000	3,21E+03	Three-zinc finger Krüppel-related factors{2.3.1}	Krüppel-like factors{2.3.1.2}	EGR1;EGR2;EGR3;EGR4;KLF1;KLF10;KLF11;KLF12;KLF13;KLF14;KLF15;KLF16;KLF17;KLF2;KLF3;KLF4;KLF5;KLF6;KLF7;KLF8;KLF9;SP1;SP2;SP3;SP4;SP5;SP6;SP7;SP8;SP9	KLF12;KLF13;KLF14;KLF15;KLF16;KLF1;KLF3;KLF4;KLF5;KLF6;KLF8;KLF9

motif	Nº of probes	% of probes	lower OR	upper OR	OR	p.value	FDR	TF family	TF subfamily	TF.family.member	TF.subfamily.member
ARX_HUMAN.H11MO.0.D	7	0.04093567	0.39089324	20.835.465	0.9880585	1000000,00000000	1,00E+06	Paired-related HD factors{3.1.3}	ARX{3.1.3.3}	ALX1;ALX3;ALX4;ARGFX;ARX;CRX;DMBX1;DPRX;DRGX;DUX4;DUXA;ESX1;GSC;GSC2;HESX1;ISX;LEUTX;MIXL1;NOBOX;OTP;OTX1;OTX2;PHOX2A;PHOX2B;PITX1;PITX2;PITX3;PROP1;PRRX1;PRRX2;RAX;RAX2;RHOXF1;RHOXF2;SEBOX;SHOX;SHOX2;TPRX1;UNCX;VSX1;VSX2	ARX
ZN528_HUMAN.H11MO.0.C	15	0.08771930	0.39067535	12.164.766	0.7150052	237390,90000000	4,69E+05	More than 3 adjacent zinc finger factors{2.3.3}	unclassified{2.3.3.0}	BCL6B;BCL6;CTCFL;CTCF;FEZF1;GFI1B;GFI1;GLI1;GLI2;GLI3;GLIS1;GLIS2;GLIS3;MTF1;MYNN;MZF1;OSR2;OVOL1;OVOL2;ZNF146;PLAG1;PLAGL1;PRDM14;PRDM1;PRDM6;SCRT1;SCRT2;SNAI1;SNAI2;YY1;YY2;WT1;ZNF324;ZNF354A;ZBTB14;ZBTB18;ZBTB48;ZBTB49;ZBTB7A;ZBTB7B;ZBTB6;ZFP64;ZFP28;ZFP42;ZFP82;ZFX;ZIC1;ZIC2;ZIC3;ZIC4;ZIM3;ZKSCAN1;ZKSCAN3;ZNF121;ZNF136;ZNF140;ZNF143;ZNF148;ZNF214;ZNF232;ZNF250;ZNF257;ZNF260;ZNF263;ZNF264;ZNF274;ZNF281;ZNF282;ZNF317;ZNF320;ZNF322;ZNF329;ZNF331;ZNF333;ZNF350;ZNF384;ZNF394;ZNF410;ZNF436;ZNF449;ZNF490;ZNF502;ZNF524;ZNF528;ZNF547;ZNF549;ZNF554;ZNF563;ZNF582;ZNF586;ZNF589;ZNF652;ZNF667;ZNF680;ZNF708;ZNF713;ZNF768;ZNF816;ZNF18;ZNF41;ZNF76;ZNF85;ZSCAN16;ZSCAN22;ZSCAN31;ZSCAN4	MYNN;MZF1;OSR2;PRDM14;PRDM6;WT1;ZBTB14;ZBTB48;ZBTB49;ZFP64;ZFP28;ZIM3;ZNF121;ZNF250;ZNF257;ZNF263;ZNF274;ZNF317;ZNF320;ZNF329;ZNF331;ZNF394;ZNF449;ZNF502;ZNF528;ZNF547;ZNF549;ZNF554;ZNF586;ZNF589;ZNF667;ZNF680;ZNF708;ZNF713;ZNF768;ZNF18;ZNF85;ZSCAN16;ZSCAN22
TWST1_HUMAN.H11MO.1.A	15	0.08771930	0.38741198	12.063.044	0.7090262	237449,80000000	4,69E+05	Tal-related factors{1.2.3}	Twist-like factors{1.2.3.2}	ATOH1;ATOH7;ATOH8;BHLHA15;BHLHA9;BHLHE22;BHLHE23;FERD3L;FIGLA;HAND1;HAND2;LYL1;MESP1;MESP2;MSC;MSGN1;NEUROD1;NEUROD2;NEUROD4;NEUROD6;NEUROG1;NEUROG2;NEUROG3;NHLH1;NHLH2;OLIG1;OLIG2;OLIG3;PTF1A;SCX;TAL1;TAL2;TCF15;TCF21;TCF23;TWIST1;TWIST2	FIGLA;HAND1;PTF1A;TWIST1
TBX4_HUMAN.H11MO.0.D	11	0.06432749	0.38640262	14.524.985	0.7899314	571569,80000000	7,82E+05	TBX2-related factors{6.5.4}	TBX4{6.5.4.0.3}	TBX2;TBX3;TBX4;TBX5	TBX4
EGR1_HUMAN.H11MO.0.A	60	0.35087719	0.38585381	0.7425308	0.5375164	86,26389000	1,59E+03	Three-zinc finger Krüppel-related factors{2.3.1}	EGR factors{2.3.1.3}	EGR1;EGR2;EGR3;EGR4;KLF1;KLF10;KLF11;KLF12;KLF13;KLF14;KLF15;KLF16;KLF17;KLF2;KLF3;KLF4;KLF5;KLF6;KLF7;KLF8;KLF9;SP1;SP2;SP3;SP4;SP5;SP6;SP7;SP8;SP9	EGR1;EGR2;EGR3;EGR4
PBX3_HUMAN.H11MO.0.A	19	0.11111111	0.38565872	10.644.229	0.6584311	93862,44000000	2,61E+05	TALE-type homeo domain factors{3.1.4}	PBX{3.1.4.4}	IRX2;IRX3;MEIS1;MEIS2;MEIS3;PBX1;PBX2;PBX3;PKNOX1;TGIF2LX;TGIF1;TGIF2	PBX1;PBX2;PBX3
AHR_HUMAN.H11MO.0.B	36	0.21052632	0.38544480	0.8337366	0.5734436	2279,58900000	1,60E+04	PAS domain factors{1.2.5}	Ahr-like factors{1.2.5.1}	AHR;AHRR;ARNT;ARNT2;ARNTL;ARNTL2;CLOCK;EPAS1;HIF1A;HIF3A;NCOA1;NCOA2;NCOA3;NPAS1;NPAS2;NPAS3;NPAS4;SIM1;SIM2;SOHLH1;SOHLH2;TCFL5	AHR;EPAS1;HIF1A

motif	Nº of probes	% of probes	lower OR	upper OR	OR	p.value	FDR	TF family	TF subfamily	TF.family.member	TF.subfamily.member
GLI2_HUMAN.H11MO.0.D	19	0.11111111	0.38503743	10.626.918	0.6573710	93921,06000000	2,61E+05	More than 3 adjacent zinc finger factors{2.3.3}	GLI-like factors{2.3.3.1}	BCL6;BCL6B;CTCF;CTCFL;FEZF1;FEZF2;GFI1;GFI1B;GLI1;GLI2;GLI3;GLI4;GLIS1;GLIS2;GLIS3;HKR1;MTF1;MYNN;MZF1;OSR2;OVOL1;OVOL2;PLAG1;PLAGL1;PLAGL2;PRDM1;PRDM14;PRDM6;SCRT1;SCRT2;SNAI1;SNAI2;SNAI3;WT1;YY1;YY2;ZBTB12;ZBTB14;ZBTB18;ZBTB20;ZBTB26;ZBTB42;ZBTB45;ZBTB47;ZBTB48;ZBTB49;ZBTB6;ZBTB7A;ZBTB7B;ZBTB7C;ZFP14;ZFP2;ZFP28;ZFP30;ZFP37;ZFP42;ZFP64;ZFP69;ZFP69B;ZFP82;ZFP91;ZFX;ZIC1;ZIC2;ZIC3;ZIC4;ZIC5;ZIK1;ZIM3;ZKSCAN1;ZKSCAN2;ZKSCAN3;ZKSCAN4;ZNF121;ZNF124;ZNF133;ZNF136;ZNF138;ZNF14;ZNF140;ZNF143;ZNF146;ZNF148;ZNF155;ZNF157;ZNF160;ZNF169;ZNF175;ZNF177;ZNF18;ZNF180;ZNF181;ZNF2;ZNF20;ZNF212;ZNF213;ZNF214;ZNF221;ZNF222;ZNF223;ZNF224;ZNF225;ZNF226;ZNF227;ZNF229;ZNF230;ZNF232;ZNF233;ZNF234;ZNF235;ZNF24;ZNF25;ZNF250;ZNF257;ZNF26;ZNF260;ZNF263;ZNF264;ZNF268;ZNF274;ZNF276;ZNF28;ZNF280A;ZNF280B;ZNF280C;ZNF280D;ZNF281;ZNF282;ZNF283;ZNF284;ZNF285;ZNF286A;ZNF286B;ZNF3;ZNF30;ZNF300;ZNF302;ZNF317;ZNF32;ZNF320;ZNF322;ZNF324;ZNF324B;ZNF329;ZNF331;ZNF333;ZNF33A;ZNF33B;ZNF343;ZNF345;ZNF347;ZNF350;ZNF354A;ZNF354B;ZNF362;ZNF366;ZNF383;ZNF384;ZNF394;ZNF397;ZNF398;ZNF404;ZNF41;ZNF410;ZNF419;ZNF420;ZNF431;ZNF432;ZNF436;ZNF439;ZNF44;ZNF440;ZNF442;ZNF443;ZNF446;ZNF449;ZNF45;ZNF460;ZNF468;ZNF479;ZNF484;ZNF490;ZNF500;ZNF502;ZNF524;ZNF525;ZNF528;ZNF543;ZNF544;ZNF546;ZNF547;ZNF548;ZNF549;ZNF554;ZNF555;ZNF557;ZNF558;ZNF559;ZNF561;ZNF562;ZNF563;ZNF564;ZNF566;ZNF567;ZNF568;ZNF57;ZNF570;ZNF571;ZNF572;ZNF577;ZNF581;ZNF582;ZNF583;ZNF585A;ZNF586;ZNF589;ZNF595;ZNF599;ZNF600;ZNF605;ZNF607;ZNF611;ZNF613;ZNF614;ZNF615;ZNF616;ZNF619;ZNF620;ZNF621;ZNF625;ZNF627;ZNF649;ZNF652;ZNF653;ZNF665;ZNF667;ZNF669;ZNF670;ZNF672;ZNF679;ZNF680;ZNF683;ZNF689;ZNF692;ZNF701;ZNF705D;ZNF705E;ZNF705G;ZNF708;ZNF709;ZNF71;ZNF710;ZNF713;ZNF721;ZNF727;ZNF729;ZNF736;ZNF75A;ZNF75D;ZNF76;ZNF763;ZNF764;ZNF765;ZNF768;ZNF77;ZNF771;ZNF773;ZNF774;ZNF776;ZNF777;ZNF780A;ZNF780B;ZNF782;ZNF785;ZNF799;ZNF805;ZNF808;ZNF81;ZNF813;ZNF816;ZNF823;ZNF829;ZNF836;ZNF841;ZNF844;ZNF845;ZNF846;ZNF85;ZNF853;ZNF860;ZNF878;ZNF891;ZNF99;ZSCAN16;ZSCAN2;ZSCAN22;ZSCAN23;ZSCAN29;ZSCAN31;ZSCAN32;ZSCAN4;ZSCAN5A;ZSCAN5B;ZSCAN5C;ZSCAN9;ZXDA;ZXDB;ZXDC	GLI1;GLI2;GLI3;GLIS1;GLIS2;GLIS3;ZIC1;ZIC2;ZIC3;ZIC4
TGIF1_HUMAN.H11MO.0.A	12	0.07017544	0.38437717	13.653.014	0.7598451	423630,90000000	6,68E+05	TALE-type homeo domain factors{3.1.4}	TGIF{3.1.4.6}	IRX1;IRX2;IRX3;IRX4;IRX5;IRX6;MEIS1;MEIS2;MEIS3;MKX;PBX1;PBX2;PBX3;PKNOX1;PKNOX2;TGIF1;TGIF2;TGIF2LX;TGIF2LY	TGIF2LX;TGIF1;TGIF2

motif	Nº of probes	% of probes	lower OR	upper OR	OR	p.value	FDR	TF family	TF subfamily	TF.family.member	TF.subfamily.member
ZN148_HUMAN.H11MO.0.D	58	0.33918129	0.38365211	0.7424373	0.5361958	86,52893000	1,59E+03	More than 3 adjacent zinc finger factors{2.3.3}	ZNF148-like factors{2.3.3.13}	BCL6;BCL6B;CTCF;CTCFL;FEZF1;FEZF2;GFI1;GFI1B;GLI1;GLI2;GLI3;GLI4;GLIS1;GLIS2;GLIS3;HKR1;MTF1;MYNN;MZF1;OSR2;OVOL1;OVOL2;PLAG1;PLAGL1;PLAGL2;PRDM1;PRDM14;PRDM6;SCRT1;SCRT2;SNAI1;SNAI2;SNAI3;WT1;YY1;YY2;ZBTB12;ZBTB14;ZBTB18;ZBTB20;ZBTB26;ZBTB42;ZBTB45;ZBTB47;ZBTB48;ZBTB49;ZBTB6;ZBTB7A;ZBTB7B;ZBTB7C;ZFP14;ZFP2;ZFP28;ZFP30;ZFP37;ZFP42;ZFP64;ZFP69;ZFP69B;ZFP82;ZFP91;ZFX;ZIC1;ZIC2;ZIC3;ZIC4;ZIC5;ZIK1;ZIM3;ZKSCAN1;ZKSCAN2;ZKSCAN3;ZKSCAN4;ZNF121;ZNF124;ZNF133;ZNF136;ZNF138;ZNF14;ZNF140;ZNF143;ZNF146;ZNF148;ZNF155;ZNF157;ZNF160;ZNF169;ZNF175;ZNF177;ZNF18;ZNF180;ZNF181;ZNF2;ZNF20;ZNF212;ZNF213;ZNF214;ZNF221;ZNF222;ZNF223;ZNF224;ZNF225;ZNF226;ZNF227;ZNF229;ZNF230;ZNF232;ZNF233;ZNF234;ZNF235;ZNF24;ZNF25;ZNF250;ZNF257;ZNF26;ZNF260;ZNF263;ZNF264;ZNF268;ZNF274;ZNF276;ZNF28;ZNF280A;ZNF280B;ZNF280C;ZNF280D;ZNF281;ZNF282;ZNF283;ZNF284;ZNF285;ZNF286A;ZNF286B;ZNF3;ZNF30;ZNF300;ZNF302;ZNF317;ZNF32;ZNF320;ZNF322;ZNF324;ZNF324B;ZNF329;ZNF331;ZNF333;ZNF33A;ZNF33B;ZNF343;ZNF345;ZNF347;ZNF350;ZNF354A;ZNF354B;ZNF362;ZNF366;ZNF383;ZNF384;ZNF394;ZNF397;ZNF398;ZNF404;ZNF41;ZNF410;ZNF419;ZNF420;ZNF431;ZNF432;ZNF436;ZNF439;ZNF44;ZNF440;ZNF442;ZNF443;ZNF446;ZNF449;ZNF45;ZNF460;ZNF468;ZNF479;ZNF484;ZNF490;ZNF500;ZNF502;ZNF524;ZNF525;ZNF528;ZNF543;ZNF544;ZNF546;ZNF547;ZNF548;ZNF549;ZNF554;ZNF555;ZNF557;ZNF558;ZNF559;ZNF561;ZNF562;ZNF563;ZNF564;ZNF566;ZNF567;ZNF568;ZNF57;ZNF570;ZNF571;ZNF572;ZNF577;ZNF581;ZNF582;ZNF583;ZNF585A;ZNF586;ZNF589;ZNF595;ZNF599;ZNF600;ZNF605;ZNF607;ZNF611;ZNF613;ZNF614;ZNF615;ZNF616;ZNF619;ZNF620;ZNF621;ZNF625;ZNF627;ZNF649;ZNF652;ZNF653;ZNF665;ZNF667;ZNF669;ZNF670;ZNF672;ZNF679;ZNF680;ZNF683;ZNF689;ZNF692;ZNF701;ZNF705D;ZNF705E;ZNF705G;ZNF708;ZNF709;ZNF71;ZNF710;ZNF713;ZNF721;ZNF727;ZNF729;ZNF736;ZNF75A;ZNF75D;ZNF76;ZNF763;ZNF764;ZNF765;ZNF768;ZNF77;ZNF771;ZNF773;ZNF774;ZNF776;ZNF777;ZNF780A;ZNF780B;ZNF782;ZNF785;ZNF799;ZNF805;ZNF808;ZNF81;ZNF813;ZNF816;ZNF823;ZNF829;ZNF836;ZNF841;ZNF844;ZNF845;ZNF846;ZNF85;ZNF853;ZNF860;ZNF878;ZNF891;ZNF99;ZSCAN16;ZSCAN2;ZSCAN22;ZSCAN23;ZSCAN29;ZSCAN31;ZSCAN32;ZSCAN4;ZSCAN5A;ZSCAN5B;ZSCAN5C;ZSCAN9;ZXDA;ZXDB;ZXDC	ZNF148;ZNF281
STAT1_HUMAN.H11MO.0.A	12	0.07017544	0.38161504	13.555.733	0.7543825	423904,70000000	6,68E+05	STAT factors{6.2.1}	STAT1{6.2.1.0.1}	STAT1;STAT2;STAT3;STAT4;STAT5A;STAT5B;STAT6	STAT1
TBX15_HUMAN.H11MO.0.D	68	0.39766082	0.38088381	0.7207254	0.5253977	38,83551000	9,66E+02	TBX1-related factors{6.5.3}	TBX15 (TBX14){6.5.3.0.3}	TBX1;TBX10;TBX15;TBX18;TBX20;TBX22	TBX15
TAF1_HUMAN.H11MO.0.A	48	0.28070175	0.38045871	0.7638454	0.5429138	245,85350000	3,45E+03	TCF-7-related factors{4.1.3}	TAF-1 (TAF-2A, TAF(II)250) [1]{4.1.3.0.5}	LEF1;TAF1;TCF7;TCF7L1;TCF7L2	TAF1
E2F2_HUMAN.H11MO.0.B	24	0.14035088	0.37912690	0.9448614	0.6107869	23978,54000000	1,04E+05	E2F-related factors{3.3.2}	E2F{3.3.2.1}	E2F1;E2F2;E2F3;E2F4;E2F5;E2F6;E2F7;E2F8;TFDP1;TFDP2	E2F1;E2F2;E2F3;E2F4;E2F5;E2F6;E2F7;E2F8

motif	Nº of probes	% of probes	lower OR	upper OR	OR	p.value	FDR	TF family	TF subfamily	TF.family.member	TF.subfamily.member
GSC_HUMAN.H11MO.0.D	7	0.04093567	0.37908492	20.205.467	0.9581983	1000000,00000000	1,00E+06	Paired-related HD factors{3.1.3}	GSC{3.1.3.9}	ALX1;ALX3;ALX4;ARGFX;ARX;CRX;DMBX1;DPRX;DRGX;DUX4;DUXA;ESX1;GSC;GSC2;HESX1;ISX;LEUTX;MIXL1;NOBOX;OTP;OTX1;OTX2;PHOX2A;PHOX2B;PITX1;PITX2;PITX3;PROP1;PRRX1;PRRX2;RAX;RAX2;RHOXF1;RHOXF2;SEBOX;SHOX;SHOX2;TPRX1;UNCX;VSX1;VSX2	GSC2;GSC
ZN281_HUMAN.H11MO.0.A	54	0.31578947	0.37899876	0.7427810	0.5335190	112,19410000	1,97E+03	More than 3 adjacent zinc finger factors{2.3.3}	ZNF148-like factors{2.3.3.13}	BCL6;BCL6B;CTCF;CTCFL;FEZF1;FEZF2;GFI1;GFI1B;GLI1;GLI2;GLI3;GLI4;GLIS1;GLIS2;GLIS3;HKR1;MTF1;MYNN;MZF1;OSR2;OVOL1;OVOL2;PLAG1;PLAGL1;PLAGL2;PRDM1;PRDM14;PRDM6;SCRT1;SCRT2;SNAI1;SNAI2;SNAI3;WT1;YY1;YY2;ZBTB12;ZBTB14;ZBTB18;ZBTB20;ZBTB26;ZBTB42;ZBTB45;ZBTB47;ZBTB48;ZBTB49;ZBTB6;ZBTB7A;ZBTB7B;ZBTB7C;ZFP14;ZFP2;ZFP28;ZFP30;ZFP37;ZFP42;ZFP64;ZFP69;ZFP69B;ZFP82;ZFP91;ZFX;ZIC1;ZIC2;ZIC3;ZIC4;ZIC5;ZIK1;ZIM3;ZKSCAN1;ZKSCAN2;ZKSCAN3;ZKSCAN4;ZNF121;ZNF124;ZNF133;ZNF136;ZNF138;ZNF14;ZNF140;ZNF143;ZNF146;ZNF148;ZNF155;ZNF157;ZNF160;ZNF169;ZNF175;ZNF177;ZNF18;ZNF180;ZNF181;ZNF2;ZNF20;ZNF212;ZNF213;ZNF214;ZNF221;ZNF222;ZNF223;ZNF224;ZNF225;ZNF226;ZNF227;ZNF229;ZNF230;ZNF232;ZNF233;ZNF234;ZNF235;ZNF24;ZNF25;ZNF250;ZNF257;ZNF26;ZNF260;ZNF263;ZNF264;ZNF268;ZNF274;ZNF276;ZNF28;ZNF280A;ZNF280B;ZNF280C;ZNF280D;ZNF281;ZNF282;ZNF283;ZNF284;ZNF285;ZNF286A;ZNF286B;ZNF3;ZNF30;ZNF300;ZNF302;ZNF317;ZNF32;ZNF320;ZNF322;ZNF324;ZNF324B;ZNF329;ZNF331;ZNF333;ZNF33A;ZNF33B;ZNF343;ZNF345;ZNF347;ZNF350;ZNF354A;ZNF354B;ZNF362;ZNF366;ZNF383;ZNF384;ZNF394;ZNF397;ZNF398;ZNF404;ZNF41;ZNF410;ZNF419;ZNF420;ZNF431;ZNF432;ZNF436;ZNF439;ZNF44;ZNF440;ZNF442;ZNF443;ZNF446;ZNF449;ZNF45;ZNF460;ZNF468;ZNF479;ZNF484;ZNF490;ZNF500;ZNF502;ZNF524;ZNF525;ZNF528;ZNF543;ZNF544;ZNF546;ZNF547;ZNF548;ZNF549;ZNF554;ZNF555;ZNF557;ZNF558;ZNF559;ZNF561;ZNF562;ZNF563;ZNF564;ZNF566;ZNF567;ZNF568;ZNF57;ZNF570;ZNF571;ZNF572;ZNF577;ZNF581;ZNF582;ZNF583;ZNF585A;ZNF586;ZNF589;ZNF595;ZNF599;ZNF600;ZNF605;ZNF607;ZNF611;ZNF613;ZNF614;ZNF615;ZNF616;ZNF619;ZNF620;ZNF621;ZNF625;ZNF627;ZNF649;ZNF652;ZNF653;ZNF665;ZNF667;ZNF669;ZNF670;ZNF672;ZNF679;ZNF680;ZNF683;ZNF689;ZNF692;ZNF701;ZNF705D;ZNF705E;ZNF705G;ZNF708;ZNF709;ZNF71;ZNF710;ZNF713;ZNF721;ZNF727;ZNF729;ZNF736;ZNF75A;ZNF75D;ZNF76;ZNF763;ZNF764;ZNF765;ZNF768;ZNF77;ZNF771;ZNF773;ZNF774;ZNF776;ZNF777;ZNF780A;ZNF780B;ZNF782;ZNF785;ZNF799;ZNF805;ZNF808;ZNF81;ZNF813;ZNF816;ZNF823;ZNF829;ZNF836;ZNF841;ZNF844;ZNF845;ZNF846;ZNF85;ZNF853;ZNF860;ZNF878;ZNF891;ZNF99;ZSCAN16;ZSCAN2;ZSCAN22;ZSCAN23;ZSCAN29;ZSCAN31;ZSCAN32;ZSCAN4;ZSCAN5A;ZSCAN5B;ZSCAN5C;ZSCAN9;ZXDA;ZXDB;ZXDC	ZNF148;ZNF281

motif	Nº of probes	% of probes	lower OR	upper OR	OR	p.value	FDR	TF family	TF subfamily	TF.family.member	TF.subfamily.member
MTF1_HUMAN.H11MO.0.C	24	0.14035088	0.37625352	0.9375804	0.6060841	19311,28000000	9,00E+04	More than 3 adjacent zinc finger factors{2.3.3}	MTF1-like factors{2.3.3.24}	BCL6;BCL6B;CTCF;CTCFL;FEZF1;FEZF2;GFI1;GFI1B;GLI1;GLI2;GLI3;GLI4;GLIS1;GLIS2;GLIS3;HKR1;MTF1;MYNN;MZF1;OSR2;OVOL1;OVOL2;PLAG1;PLAGL1;PLAGL2;PRDM1;PRDM14;PRDM6;SCRT1;SCRT2;SNAI1;SNAI2;SNAI3;WT1;YY1;YY2;ZBTB12;ZBTB14;ZBTB18;ZBTB20;ZBTB26;ZBTB42;ZBTB45;ZBTB47;ZBTB48;ZBTB49;ZBTB6;ZBTB7A;ZBTB7B;ZBTB7C;ZFP14;ZFP2;ZFP28;ZFP30;ZFP37;ZFP42;ZFP64;ZFP69;ZFP69B;ZFP82;ZFP91;ZFX;ZIC1;ZIC2;ZIC3;ZIC4;ZIC5;ZIK1;ZIM3;ZKSCAN1;ZKSCAN2;ZKSCAN3;ZKSCAN4;ZNF121;ZNF124;ZNF133;ZNF136;ZNF138;ZNF14;ZNF140;ZNF143;ZNF146;ZNF148;ZNF155;ZNF157;ZNF160;ZNF169;ZNF175;ZNF177;ZNF18;ZNF180;ZNF181;ZNF2;ZNF20;ZNF212;ZNF213;ZNF214;ZNF221;ZNF222;ZNF223;ZNF224;ZNF225;ZNF226;ZNF227;ZNF229;ZNF230;ZNF232;ZNF233;ZNF234;ZNF235;ZNF24;ZNF25;ZNF250;ZNF257;ZNF26;ZNF260;ZNF263;ZNF264;ZNF268;ZNF274;ZNF276;ZNF28;ZNF280A;ZNF280B;ZNF280C;ZNF280D;ZNF281;ZNF282;ZNF283;ZNF284;ZNF285;ZNF286A;ZNF286B;ZNF3;ZNF30;ZNF300;ZNF302;ZNF317;ZNF32;ZNF320;ZNF322;ZNF324;ZNF324B;ZNF329;ZNF331;ZNF333;ZNF33A;ZNF33B;ZNF343;ZNF345;ZNF347;ZNF350;ZNF354A;ZNF354B;ZNF362;ZNF366;ZNF383;ZNF384;ZNF394;ZNF397;ZNF398;ZNF404;ZNF41;ZNF410;ZNF419;ZNF420;ZNF431;ZNF432;ZNF436;ZNF439;ZNF44;ZNF440;ZNF442;ZNF443;ZNF446;ZNF449;ZNF45;ZNF460;ZNF468;ZNF479;ZNF484;ZNF490;ZNF500;ZNF502;ZNF524;ZNF525;ZNF528;ZNF543;ZNF544;ZNF546;ZNF547;ZNF548;ZNF549;ZNF554;ZNF555;ZNF557;ZNF558;ZNF559;ZNF561;ZNF562;ZNF563;ZNF564;ZNF566;ZNF567;ZNF568;ZNF57;ZNF570;ZNF571;ZNF572;ZNF577;ZNF581;ZNF582;ZNF583;ZNF585A;ZNF586;ZNF589;ZNF595;ZNF599;ZNF600;ZNF605;ZNF607;ZNF611;ZNF613;ZNF614;ZNF615;ZNF616;ZNF619;ZNF620;ZNF621;ZNF625;ZNF627;ZNF649;ZNF652;ZNF653;ZNF665;ZNF667;ZNF669;ZNF670;ZNF672;ZNF679;ZNF680;ZNF683;ZNF689;ZNF692;ZNF701;ZNF705D;ZNF705E;ZNF705G;ZNF708;ZNF709;ZNF71;ZNF710;ZNF713;ZNF721;ZNF727;ZNF729;ZNF736;ZNF75A;ZNF75D;ZNF76;ZNF763;ZNF764;ZNF765;ZNF768;ZNF77;ZNF771;ZNF773;ZNF774;ZNF776;ZNF777;ZNF780A;ZNF780B;ZNF782;ZNF785;ZNF799;ZNF805;ZNF808;ZNF81;ZNF813;ZNF816;ZNF823;ZNF829;ZNF836;ZNF841;ZNF844;ZNF845;ZNF846;ZNF85;ZNF853;ZNF860;ZNF878;ZNF891;ZNF99;ZSCAN16;ZSCAN2;ZSCAN22;ZSCAN23;ZSCAN29;ZSCAN31;ZSCAN32;ZSCAN4;ZSCAN5A;ZSCAN5B;ZSCAN5C;ZSCAN9;ZXDA;ZXDB;ZXDC	MTF1;ZNF410
GRHL2_HUMAN.H11MO.0.A	11	0.06432749	0.37618106	14.139.857	0.7690017	485507,70000000	7,05E+05	Grainyhead-related factors{6.7.1}	GRH-like proteins{6.7.1.1}	GRHL1;GRHL2;GRHL3	GRHL1;GRHL2

motif	Nº of probes	% of probes	lower OR	upper OR	OR	p.value	FDR	TF family	TF subfamily	TF.family.member	TF.subfamily.member
SNAI1_HUMAN.H11MO.0.C	25	0.14619883	0.37549228	0.9206355	0.5993561	16387,22000000	7,85E+04	More than 3 adjacent zinc finger factors{2.3.3}	Snail-like factors{2.3.3.2}	BCL6;BCL6B;CTCF;CTCFL;FEZF1;FEZF2;GFI1;GFI1B;GLI1;GLI2;GLI3;GLI4;GLIS1;GLIS2;GLIS3;HKR1;MTF1;MYNN;MZF1;OSR2;OVOL1;OVOL2;PLAG1;PLAGL1;PLAGL2;PRDM1;PRDM14;PRDM6;SCRT1;SCRT2;SNAI1;SNAI2;SNAI3;WT1;YY1;YY2;ZBTB12;ZBTB14;ZBTB18;ZBTB20;ZBTB26;ZBTB42;ZBTB45;ZBTB47;ZBTB48;ZBTB49;ZBTB6;ZBTB7A;ZBTB7B;ZBTB7C;ZFP14;ZFP2;ZFP28;ZFP30;ZFP37;ZFP42;ZFP64;ZFP69;ZFP69B;ZFP82;ZFP91;ZFX;ZIC1;ZIC2;ZIC3;ZIC4;ZIC5;ZIK1;ZIM3;ZKSCAN1;ZKSCAN2;ZKSCAN3;ZKSCAN4;ZNF121;ZNF124;ZNF133;ZNF136;ZNF138;ZNF14;ZNF140;ZNF143;ZNF146;ZNF148;ZNF155;ZNF157;ZNF160;ZNF169;ZNF175;ZNF177;ZNF18;ZNF180;ZNF181;ZNF2;ZNF20;ZNF212;ZNF213;ZNF214;ZNF221;ZNF222;ZNF223;ZNF224;ZNF225;ZNF226;ZNF227;ZNF229;ZNF230;ZNF232;ZNF233;ZNF234;ZNF235;ZNF24;ZNF25;ZNF250;ZNF257;ZNF26;ZNF260;ZNF263;ZNF264;ZNF268;ZNF274;ZNF276;ZNF28;ZNF280A;ZNF280B;ZNF280C;ZNF280D;ZNF281;ZNF282;ZNF283;ZNF284;ZNF285;ZNF286A;ZNF286B;ZNF3;ZNF30;ZNF300;ZNF302;ZNF317;ZNF32;ZNF320;ZNF322;ZNF324;ZNF324B;ZNF329;ZNF331;ZNF333;ZNF33A;ZNF33B;ZNF343;ZNF345;ZNF347;ZNF350;ZNF354A;ZNF354B;ZNF362;ZNF366;ZNF383;ZNF384;ZNF394;ZNF397;ZNF398;ZNF404;ZNF41;ZNF410;ZNF419;ZNF420;ZNF431;ZNF432;ZNF436;ZNF439;ZNF44;ZNF440;ZNF442;ZNF443;ZNF446;ZNF449;ZNF45;ZNF460;ZNF468;ZNF479;ZNF484;ZNF490;ZNF500;ZNF502;ZNF524;ZNF525;ZNF528;ZNF543;ZNF544;ZNF546;ZNF547;ZNF548;ZNF549;ZNF554;ZNF555;ZNF557;ZNF558;ZNF559;ZNF561;ZNF562;ZNF563;ZNF564;ZNF566;ZNF567;ZNF568;ZNF57;ZNF570;ZNF571;ZNF572;ZNF577;ZNF581;ZNF582;ZNF583;ZNF585A;ZNF586;ZNF589;ZNF595;ZNF599;ZNF600;ZNF605;ZNF607;ZNF611;ZNF613;ZNF614;ZNF615;ZNF616;ZNF619;ZNF620;ZNF621;ZNF625;ZNF627;ZNF649;ZNF652;ZNF653;ZNF665;ZNF667;ZNF669;ZNF670;ZNF672;ZNF679;ZNF680;ZNF683;ZNF689;ZNF692;ZNF701;ZNF705D;ZNF705E;ZNF705G;ZNF708;ZNF709;ZNF71;ZNF710;ZNF713;ZNF721;ZNF727;ZNF729;ZNF736;ZNF75A;ZNF75D;ZNF76;ZNF763;ZNF764;ZNF765;ZNF768;ZNF77;ZNF771;ZNF773;ZNF774;ZNF776;ZNF777;ZNF780A;ZNF780B;ZNF782;ZNF785;ZNF799;ZNF805;ZNF808;ZNF81;ZNF813;ZNF816;ZNF823;ZNF829;ZNF836;ZNF841;ZNF844;ZNF845;ZNF846;ZNF85;ZNF853;ZNF860;ZNF878;ZNF891;ZNF99;ZSCAN16;ZSCAN2;ZSCAN22;ZSCAN23;ZSCAN29;ZSCAN31;ZSCAN32;ZSCAN4;ZSCAN5A;ZSCAN5B;ZSCAN5C;ZSCAN9;ZXDA;ZXDB;ZXDC	SCRT1;SCRT2;SNAI1;SNAI2
EMX1_HUMAN.H11MO.0.D	7	0.04093567	0.37455151	19.964.493	0.9467330	1000000,00000000	1,00E+06	NK-related factors{3.1.2}	EMX{3.1.2.6}	BARHL1;BARHL2;BARX1;BARX2;BSX;DBX1;DBX2;DLX1;DLX2;DLX3;DLX4;DLX5;DLX6;EMX1;EMX2;EN1;EN2;HHEX;HLX;HMX1;HMX2;HMX3;LBX1;LBX2;MSX1;MSX2;NANOG;NKX1-1;NKX1-2;NKX2-1;NKX2-2;NKX2-3;NKX2-4;NKX2-5;NKX2-6;NKX2-8;NKX3-1;NKX3-2;NKX6-1;NKX6-2;NKX6-3;NOTO;TLX1;TLX2;TLX3;VAX1;VAX2;VENTX	EMX1;EMX2
E2F4_HUMAN.H11MO.0.A	57	0.33333333	0.37447387	0.7267981	0.5242367	60,70464000	1,30E+03	E2F-related factors{3.3.2}	E2F{3.3.2.1}	E2F1;E2F2;E2F3;E2F4;E2F5;E2F6;E2F7;E2F8;TFDP1;TFDP2	E2F1;E2F2;E2F3;E2F4;E2F5;E2F6;E2F7;E2F8

motif	Nº of probes	% of probes	lower OR	upper OR	OR	p.value	FDR	TF family	TF subfamily	TF.family.member	TF.subfamily.member
TFCP2_HUMAN.H11MO.0.D	25	0.14619883	0.37344625	0.9156399	0.5961037	16444,01000000	7,85E+04	CP2-related factors{6.7.2}	CP2 (LSF, SEF){6.7.2.0.1}	TFCP2;UBP1	TFCP2
NR4A2_HUMAN.H11MO.0.C	11	0.06432749	0.37247508	14.001.154	0.7614233	486196,40000000	7,05E+05	NGFI-B-related receptors (NR4){2.1.4}	NURR1 (NR4A2){2.1.4.0.2}	NR4A1;NR4A2;NR4A3	NR4A2
NR2C2_HUMAN.H11MO.0.B	19	0.11111111	0.37027082	10.218.636	0.6321246	62863,85000000	2,04E+05	RXR-related receptors (NR2){2.1.3}	Testicular receptors (NR2C){2.1.3.4}	HNF4A;HNF4G;NR2C1;NR2C2;NR2E1;NR2E3;NR2F1;NR2F2;NR2F6;RXRA;RXRB;RXRG	NR2C1;NR2C2
COT2_HUMAN.H11MO.0.A	15	0.08771930	0.37011845	11.524.459	0.6773674	164062,40000000	3,87E+05	RXR-related receptors (NR2){2.1.3}	COUP-like receptors (NR2F){2.1.3.5}	HNF4A;HNF4G;NR2C1;NR2C2;NR2E1;NR2E3;NR2F1;NR2F2;NR2F6;RXRA;RXRB;RXRG	NR2F1;NR2F2;NR2F6
PROX1_HUMAN.H11MO.0.D	44	0.25730994	0.36983838	0.7577276	0.5337920	215,64640000	3,21E+03	HD-PROS factors{3.1.7}	PROX-1{3.1.7.0.1}	PROX1;PROX2	PROX1
GCM1_HUMAN.H11MO.0.D	13	0.07602339	0.36863720	12.463.042	0.7078743	260310,00000000	4,91E+05	GCM factors{7.2.1}	GCMa (GCM1){7.2.1.0.1}	GCM1;GCM2	GCM1
ZN214_HUMAN.H11MO.0.C	8	0.04678363	0.36860559	17.532.630	0.8685340	864714,20000000	9,46E+05	More than 3 adjacent zinc finger factors{2.3.3}	ZNF214-like factors{2.3.3.56}	BCL6;BCL6B;CTCF;CTCFL;FEZF1;FEZF2;GFI1;GFI1B;GLI1;GLI2;GLI3;GLI4;GLIS1;GLIS2;GLIS3;HKR1;MTF1;MYNN;MZF1;OSR2;OVOL1;OVOL2;PLAG1;PLAGL1;PLAGL2;PRDM1;PRDM14;PRDM6;SCRT1;SCRT2;SNAI1;SNAI2;SNAI3;WT1;YY1;YY2;ZBTB12;ZBTB14;ZBTB18;ZBTB20;ZBTB26;ZBTB42;ZBTB45;ZBTB47;ZBTB48;ZBTB49;ZBTB6;ZBTB7A;ZBTB7B;ZBTB7C;ZFP14;ZFP2;ZFP28;ZFP30;ZFP37;ZFP42;ZFP64;ZFP69;ZFP69B;ZFP82;ZFP91;ZFX;ZIC1;ZIC2;ZIC3;ZIC4;ZIC5;ZIK1;ZIM3;ZKSCAN1;ZKSCAN2;ZKSCAN3;ZKSCAN4;ZNF121;ZNF124;ZNF133;ZNF136;ZNF138;ZNF14;ZNF140;ZNF143;ZNF146;ZNF148;ZNF155;ZNF157;ZNF160;ZNF169;ZNF175;ZNF177;ZNF18;ZNF180;ZNF181;ZNF2;ZNF20;ZNF212;ZNF213;ZNF214;ZNF221;ZNF222;ZNF223;ZNF224;ZNF225;ZNF226;ZNF227;ZNF229;ZNF230;ZNF232;ZNF233;ZNF234;ZNF235;ZNF24;ZNF25;ZNF250;ZNF257;ZNF26;ZNF260;ZNF263;ZNF264;ZNF268;ZNF274;ZNF276;ZNF28;ZNF280A;ZNF280B;ZNF280C;ZNF280D;ZNF281;ZNF282;ZNF283;ZNF284;ZNF285;ZNF286A;ZNF286B;ZNF3;ZNF30;ZNF300;ZNF302;ZNF317;ZNF32;ZNF320;ZNF322;ZNF324;ZNF324B;ZNF329;ZNF331;ZNF333;ZNF33A;ZNF33B;ZNF343;ZNF345;ZNF347;ZNF350;ZNF354A;ZNF354B;ZNF362;ZNF366;ZNF383;ZNF384;ZNF394;ZNF397;ZNF398;ZNF404;ZNF41;ZNF410;ZNF419;ZNF420;ZNF431;ZNF432;ZNF436;ZNF439;ZNF44;ZNF440;ZNF442;ZNF443;ZNF446;ZNF449;ZNF45;ZNF460;ZNF468;ZNF479;ZNF484;ZNF490;ZNF500;ZNF502;ZNF524;ZNF525;ZNF528;ZNF543;ZNF544;ZNF546;ZNF547;ZNF548;ZNF549;ZNF554;ZNF555;ZNF557;ZNF558;ZNF559;ZNF561;ZNF562;ZNF563;ZNF564;ZNF566;ZNF567;ZNF568;ZNF57;ZNF570;ZNF571;ZNF572;ZNF577;ZNF581;ZNF582;ZNF583;ZNF585A;ZNF586;ZNF589;ZNF595;ZNF599;ZNF600;ZNF605;ZNF607;ZNF611;ZNF613;ZNF614;ZNF615;ZNF616;ZNF619;ZNF620;ZNF621;ZNF625;ZNF627;ZNF649;ZNF652;ZNF653;ZNF665;ZNF667;ZNF669;ZNF670;ZNF672;ZNF679;ZNF680;ZNF683;ZNF689;ZNF692;ZNF701;ZNF705D;ZNF705E;ZNF705G;ZNF708;ZNF709;ZNF71;ZNF710;ZNF713;ZNF721;ZNF727;ZNF729;ZNF736;ZNF75A;ZNF75D;ZNF76;ZNF763;Z	ZNF214

motif	Nº of probes	% of probes	lower OR	upper OR	OR	p.value	FDR	TF family	TF subfamily	TF.family.member	TF.subfamily.member
										NF764;ZNF765;ZNF768;ZNF77;ZNF771;ZNF773;ZNF774;ZNF776;ZNF777;ZNF780A;ZNF780B;ZNF782;ZNF785;ZNF799;ZNF805;ZNF808;ZNF81;ZNF813;ZNF816;ZNF823;ZNF829;ZNF836;ZNF841;ZNF844;ZNF845;ZNF846;ZNF85;ZNF853;ZNF860;ZNF878;ZNF891;ZNF99;ZSCAN16;ZSCAN2;ZSCAN22;ZSCAN23;ZSCAN29;ZSCAN31;ZSCAN32;ZSCAN4;ZSCAN5A;ZSCAN5B;ZSCAN5C;ZSCAN9;ZXDA;ZXDB;ZXDC	
DMBX1_HUMAN.H11MO.0.D	8	0.04678363	0.36681503	17.447.407	0.8643143	864823,80000000	9,46E+05	Paired-related HD factors{3.1.3}	DMBX{3.1.3.4}	ALX1;ALX3;ALX4;ARGFX;ARX;CRX;DMBX1;DPRX;DRGX;DUX4;DUXA;ESX1;GSC;GSC2;HESX1;ISX;LEUTX;MIXL1;NOBOX;OTP;OTX1;OTX2;PHOX2A;PHOX2B;PITX1;PITX2;PITX3;PROP1;PRRX1;PRRX2;RAX;RAX2;RHOXF1;RHOXF2;SEBOX;SHOX;SHOX2;TPRX1;UNCX;VSX1;VSX2	DMBX1
CTCF_HUMAN.H11MO.0.A	39	0.22807018	0.36654532	0.7748888	0.5383751	412,78750000	4,86E+03	More than 3 adjacent zinc finger factors{2.3.3}	CTCF-like factors{2.3.3.50}	BCL6;BCL6B;CTCF;CTCFL;FEZF1;FEZF2;GFI1;GFI1B;GLI1;GLI2;GLI3;GLI4;GLIS1;GLIS2;GLIS3;HKR1;MTF1;MYNN;MZF1;OSR2;OVOL1;OVOL2;PLAG1;PLAGL1;PLAGL2;PRDM1;PRDM14;PRDM6;SCRT1;SCRT2;SNAI1;SNAI2;SNAI3;WT1;YY1;YY2;ZBTB12;ZBTB14;ZBTB18;ZBTB20;ZBTB26;ZBTB42;ZBTB45;ZBTB47;ZBTB48;ZBTB49;ZBTB6;ZBTB7A;ZBTB7B;ZBTB7C;ZFP14;ZFP2;ZFP28;ZFP30;ZFP37;ZFP42;ZFP64;ZFP69;ZFP69B;ZFP82;ZFP91;ZFX;ZIC1;ZIC2;ZIC3;ZIC4;ZIC5;ZIK1;ZIM3;ZKSCAN1;ZKSCAN2;ZKSCAN3;ZKSCAN4;ZNF121;ZNF124;ZNF133;ZNF136;ZNF138;ZNF14;ZNF140;ZNF143;ZNF146;ZNF148;ZNF155;ZNF157;ZNF160;ZNF169;ZNF175;ZNF177;ZNF18;ZNF180;ZNF181;ZNF2;ZNF20;ZNF212;ZNF213;ZNF214;ZNF221;ZNF222;ZNF223;ZNF224;ZNF225;ZNF226;ZNF227;ZNF229;ZNF230;ZNF232;ZNF233;ZNF234;ZNF235;ZNF24;ZNF25;ZNF250;ZNF257;ZNF26;ZNF260;ZNF263;ZNF264;ZNF268;ZNF274;ZNF276;ZNF28;ZNF280A;ZNF280B;ZNF280C;ZNF280D;ZNF281;ZNF282;ZNF283;ZNF284;ZNF285;ZNF286A;ZNF286B;ZNF3;ZNF30;ZNF300;ZNF302;ZNF317;ZNF32;ZNF320;ZNF322;ZNF324;ZNF324B;ZNF329;ZNF331;ZNF333;ZNF33A;ZNF33B;ZNF343;ZNF345;ZNF347;ZNF350;ZNF354A;ZNF354B;ZNF362;ZNF366;ZNF383;ZNF384;ZNF394;ZNF397;ZNF398;ZNF404;ZNF41;ZNF410;ZNF419;ZNF420;ZNF431;ZNF432;ZNF436;ZNF439;ZNF44;ZNF440;ZNF442;ZNF443;ZNF446;ZNF449;ZNF45;ZNF460;ZNF468;ZNF479;ZNF484;ZNF490;ZNF500;ZNF502;ZNF524;ZNF525;ZNF528;ZNF543;ZNF544;ZNF546;ZNF547;ZNF548;ZNF549;ZNF554;ZN	CTCFL;CTCF

motif	Nº of probes	% of probes	lower OR	upper OR	OR	p.value	FDR	TF family	TF subfamily	TF.family.member	TF.subfamily.member
										F555;ZNF557;ZNF558;ZNF559;ZNF561;ZNF562;ZNF563;ZNF564;ZNF566;ZNF567;ZNF568;ZNF57;ZNF570;ZNF571;ZNF572;ZNF577;ZNF581;ZNF582;ZNF583;ZNF585A;ZNF586;ZNF589;ZNF595;ZNF599;ZNF600;ZNF605;ZNF607;ZNF611;ZNF613;ZNF614;ZNF615;ZNF616;ZNF619;ZNF620;ZNF621;ZNF625;ZNF627;ZNF649;ZNF652;ZNF653;ZNF665;ZNF667;ZNF669;ZNF670;ZNF672;ZNF679;ZNF680;ZNF683;ZNF689;ZNF692;ZNF701;ZNF705D;ZNF705E;ZNF705G;ZNF708;ZNF709;ZNF71;ZNF710;ZNF713;ZNF721;ZNF727;ZNF729;ZNF736;ZNF75A;ZNF75D;ZNF76;ZNF763;ZNF764;ZNF765;ZNF768;ZNF77;ZNF771;ZNF773;ZNF774;ZNF776;ZNF777;ZNF780A;ZNF780B;ZNF782;ZNF785;ZNF799;ZNF805;ZNF808;ZNF81;ZNF813;ZNF816;ZNF823;ZNF829;ZNF836;ZNF841;ZNF844;ZNF845;ZNF846;ZNF85;ZNF853;ZNF860;ZNF878;ZNF891;ZNF99;ZSCAN16;ZSCAN2;ZSCAN22;ZSCAN23;ZSCAN29;ZSCAN31;ZSCAN32;ZSCAN4;ZSCAN5A;ZSCAN5B;ZSCAN5C;ZSCAN9;ZXDA;ZXDB;ZXDC	
NOBOX_HUMAN.H11MO.0.C	6	0.03508772	0.36625731	22.540.584	1,01E+07	835366,40000000	9,40E+05	Paired-related HD factors{3.1.3}	NOBOX{3.1.3.15}	ALX1;ALX3;ALX4;ARGFX;ARX;CRX;DMBX1;DPRX;DRGX;DUX4;DUXA;ESX1;GSC;GSC2;HESX1;ISX;LEUTX;MIXL1;NOBOX;OTP;OTX1;OTX2;PHOX2A;PHOX2B;PITX1;PITX2;PITX3;PROP1;PRRX1;PRRX2;RAX;RAX2;RHOXF1;RHOXF2;SEBOX;SHOX;SHOX2;TPRX1;UNCX;VSX1;VSX2	NOBOX
KLF16_HUMAN.H11MO.0.D	66	0.38596491	0.36497662	0.6929931	0.5044731	10,02198000	2,86E+02	Three-zinc finger Krüppel-related factors{2.3.1}	Krüppel-like factors{2.3.1.2}	EGR1;EGR2;EGR3;EGR4;KLF1;KLF10;KLF11;KLF12;KLF13;KLF14;KLF15;KLF16;KLF17;KLF2;KLF3;KLF4;KLF5;KLF6;KLF7;KLF8;KLF9;SP1;SP2;SP3;SP4;SP5;SP6;SP7;SP8;SP9	KLF12;KLF13;KLF14;KLF15;KLF16;KLF1;KLF3;KLF4;KLF5;KLF6;KLF8;KLF9
IRX2_HUMAN.H11MO.0.D	17	0.09941520	0.36486491	10.633.924	0.6426980	83662,60000000	2,39E+05	TALE-type homeo domain factors{3.1.4}	IRX (Iroquois){3.1.4.1}	IRX1;IRX2;IRX3;IRX4;IRX5;IRX6;MEIS1;MEIS2;MEIS3;MKX;PBX1;PBX2;PBX3;PKNOX1;PKNOX2;TGIF1;TGIF2;TGIF2LX;TGIF2LY	IRX2;IRX3
HNF6_HUMAN.H11MO.0.B	6	0.03508772	0.36323774	22.355.058	1,00E+07	1000000,00000000	1,00E+06	HD-CUT factors{3.1.9}	ONECUT{3.1.9.1}	CUX1;CUX2;ONECUT1;ONECUT2;ONECUT3;SATB1;SATB2	ONECUT1;ONECUT2;ONECUT3
WT1_HUMAN.H11MO.0.C	74	0.43274854	0.36249679	0.6803709	0.4974904	6,64119600	2,05E+02	More than 3 adjacent zinc finger factors{2.3}	unclassified{2.3.3.0}	BCL6B;BCL6;CTCFL;CTCF;FEZF1;GFI1B;GFI1;GLI1;GLI2;GLI3;GLIS1;GLIS2;GLIS3;MTF1;MYNN;MZF1;OSR2;OVOL1;OVOL2;ZNF146;PLAG1;PLAGL1;PRDM14;PRDM1;PRDM6;SCRT1;SCRT2;SNAI1;SNAI2;YY1;YY2;WT1;ZNF324;ZNF354A;ZBTB14;ZBTB18;ZBTB48;ZBTB49;ZBTB7A;ZBTB7B;ZBTB6;ZFP64;ZFP28;ZFP42;ZFP82;ZFX;ZIC1;ZIC2;ZIC3;ZIC4;ZIM3;ZKSCAN1;ZKSCAN3;ZNF121;ZNF136;ZNF140;ZNF143;ZNF148;ZNF214;ZNF232;ZNF250;ZNF257;ZNF260;ZNF263;ZNF264;ZNF274;ZNF281;ZNF282;ZNF317;ZNF320;ZNF322;ZNF329;ZNF331;ZNF333;ZNF350;ZNF384;ZNF394;ZNF410;ZNF436;ZNF449;ZNF490;ZNF502;ZNF524;ZNF528;ZNF547;ZNF549;ZNF554;ZNF563;ZNF582;ZNF586;ZNF589;ZNF652;ZNF667;ZNF680;ZNF708;ZNF713;ZNF768;ZNF816;ZNF18;ZNF41;ZNF76;ZNF85;ZSCAN16;ZSCAN22;ZSCAN31;ZSCAN4	MYNN;MZF1;OSR2;PRDM14;PRDM6;WT1;ZBTB14;ZBTB48;ZBTB49;ZFP64;ZFP28;ZIM3;ZNF121;ZNF250;ZNF257;ZNF263;ZNF274;ZNF317;ZNF320;ZNF329;ZNF331;ZNF394;ZNF449;ZNF502;ZNF528;ZNF547;ZNF549;ZNF554;ZNF586;ZNF589;ZNF667;ZNF680;ZNF708;ZNF713;ZNF768;ZNF18;ZNF85;ZSCAN16;ZSCAN22
E2F5_HUMAN.H11MO.0.B	13	0.07602339	0.36170935	12.228.460	0.6945619	261381,30000000	4,91E+05	E2F-related factors{3.3.2}	E2F{3.3.2.1}	E2F1;E2F2;E2F3;E2F4;E2F5;E2F6;E2F7;E2F8;TFDP1;TFDP2	E2F1;E2F2;E2F3;E2F4;E2F5;E2F6;E2F7;E2F8

motif	Nº of probes	% of probes	lower OR	upper OR	OR	p.value	FDR	TF family	TF subfamily	TF.family.member	TF.subfamily.member
KLF3_HUMAN.H11MO.0.B	68	0.39766082	0.36121007	0.6833962	0.4981897	6,39556800	2,05E+02	Three-zinc finger Krüppel-related factors{2.3.1}	Krüppel-like factors{2.3.1.2}	EGR1;EGR2;EGR3;EGR4;KLF1;KLF10;KLF11;KLF12;KLF13;KLF14;KLF15;KLF16;KLF17;KLF2;KLF3;KLF4;KLF5;KLF6;KLF7;KLF8;KLF9;SP1;SP2;SP3;SP4;SP5;SP6;SP7;SP8;SP9	KLF12;KLF13;KLF14;KLF15;KLF16;KLF1;KLF3;KLF4;KLF5;KLF6;KLF8;KLF9
OLIG2_HUMAN.H11MO.1.B	17	0.09941520	0.36045275	10.505.042	0.6349246	83805,38000000	2,39E+05	Tal-related factors{1.2.3}	Neurogenin / Atonal-like factors{1.2.3.4}	ATOH1;ATOH7;ATOH8;BHLHA15;BHLHA9;BHLHE22;BHLHE23;FERD3L;FIGLA;HAND1;HAND2;LYL1;MESP1;MESP2;MSC;MSGN1;NEUROD1;NEUROD2;NEUROD4;NEUROD6;NEUROG1;NEUROG2;NEUROG3;NHLH1;NHLH2;OLIG1;OLIG2;OLIG3;PTF1A;SCX;TAL1;TAL2;TCF15;TCF21;TCF23;TWIST1;TWIST2	ATOH1;BHLHA15;BHLHE22;BHLHE23;NEUROD1;NEUROD2;NEUROG2;OLIG1;OLIG2;OLIG3
RAX2_HUMAN.H11MO.0.D	7	0.04093567	0.35914878	19.141.600	0.9077754	1000000,00000000	1,00E+06	Paired-related HD factors{3.1.3}	RAX{3.1.3.22}	ALX1;ALX3;ALX4;ARGFX;ARX;CRX;DMBX1;DPRX;DRGX;DUX4;DUXA;ESX1;GSC;GSC2;HESX1;ISX;LEUTX;MIXL1;NOBOX;OTP;OTX1;OTX2;PHOX2A;PHOX2B;PITX1;PITX2;PITX3;PROP1;PRRX1;PRRX2;RAX;RAX2;RHOXF1;RHOXF2;SEBOX;SHOX;SHOX2;TPRX1;UNCX;VSX1;VSX2	RAX2;RAX
VDR_HUMAN.H11MO.0.A	18	0.10526316	0.35904250	10.170.101	0.6221708	58970,86000000	1,95E+05	Thyroid hormone receptor-related factors (NR1){2.1.2}	Vitamin D receptor (NR1I){2.1.2.4}	NR1D1;NR1D2;NR1H2;NR1H3;NR1H4;NR1I2;NR1I3;PPARA;PPARD;PPARG;RARA;RARB;RARG;RORA;RORB;RORC;THRA;THRB;VDR	NR1I2;NR1I3;VDR
KLF1_HUMAN.H11MO.0.A	55	0.32163743	0.35903248	0.7012175	0.5044266	21,77429000	6,00E+02	Three-zinc finger Krüppel-related factors{2.3.1}	Krüppel-like factors{2.3.1.2}	EGR1;EGR2;EGR3;EGR4;KLF1;KLF10;KLF11;KLF12;KLF13;KLF14;KLF15;KLF16;KLF17;KLF2;KLF3;KLF4;KLF5;KLF6;KLF7;KLF8;KLF9;SP1;SP2;SP3;SP4;SP5;SP6;SP7;SP8;SP9	KLF12;KLF13;KLF14;KLF15;KLF16;KLF1;KLF3;KLF4;KLF5;KLF6;KLF8;KLF9
HME1_HUMAN.H11MO.0.D	7	0.04093567	0.35882600	19.124.330	0.9069590	1000000,00000000	1,00E+06	NK-related factors{3.1.2}	EN (Engrailed-like factors){3.1.2.7}	BARHL1;BARHL2;BARX1;BARX2;BSX;DBX1;DBX2;DLX1;DLX2;DLX3;DLX4;DLX5;DLX6;EMX1;EMX2;EN1;EN2;HHEX;HLX;HMX1;HMX2;HMX3;LBX1;LBX2;MSX1;MSX2;NANOG;NKX1-1;NKX1-2;NKX2-1;NKX2-2;NKX2-3;NKX2-4;NKX2-5;NKX2-6;NKX2-8;NKX3-1;NKX3-2;NKX6-1;NKX6-2;NKX6-3;NOTO;TLX1;TLX2;TLX3;VAX1;VAX2;VENTX	EN1;EN2
PDX1_HUMAN.H11MO.1.A	7	0.04093567	0.35843939	19.103.662	0.9059812	1000000,00000000	1,00E+06	HOX-related factors{3.1.1}	PDX{3.1.1.15}	CDX1;CDX2;CDX4;EVX1;EVX2;GBX1;GBX2;GSX1;GSX2;HDX;HMBOX1;HNF1A;HNF1B;HOXA1;HOXA10;HOXA11;HOXA13;HOXA2;HOXA3;HOXA4;HOXA5;HOXA6;HOXA7;HOXA9;HOXB1;HOXB13;HOXB2;HOXB3;HOXB4;HOXB5;HOXB6;HOXB7;HOXB8;HOXB9;HOXC10;HOXC11;HOXC12;HOXC13;HOXC4;HOXC5;HOXC6;HOXC8;HOXC9;HOXD1;HOXD10;HOXD11;HOXD12;HOXD13;HOXD3;HOXD4;HOXD8;HOXD9;MEOX1;MEOX2;MNX1;PDX1;POU1F1;POU2F1;POU2F2;POU2F3;POU3F1;POU3F2;POU3F3;POU3F4;POU4F1;POU4F2;POU4F3;POU5F1;POU5F2;POU6F1;POU6F2	PDX1
RXRA_HUMAN.H11MO.1.A	16	0.09356725	0.35793885	10.764.113	0.6420121	96260,36000000	2,67E+05	RXR-related receptors (NR2){2.1.3}	Retinoid X receptors (NR2B){2.1.3.1}	HNF4A;HNF4G;NR2C1;NR2C2;NR2E1;NR2E3;NR2F1;NR2F2;NR2F6;RXRA;RXRB;RXRG	RXRA;RXRB;RXRG
P73_HUMAN.H11MO.0.A	13	0.07602339	0.35712023	12.073.995	0.6857706	216091,80000000	4,44E+05	p53-related factors{6.3.1}	p73{6.3.1.0.3}	TP53;TP63;TP73	TP73
BSH_HUMAN.H11MO.0.D	7	0.04093567	0.35632721	18.991.801	0.9006388	1000000,00000000	1,00E+06	NK-related factors{3.1.2}	BSX{3.1.2.3}	BARHL1;BARHL2;BARX1;BARX2;BSX;DBX1;DBX2;DLX1;DLX2;DLX3;DLX4;DLX5;DLX6;EMX1;EMX2;EN1;EN2;HHEX;HLX;HMX1;HMX2;HMX3;LBX1;LBX2;MSX1;MSX2;NANOG;NKX1-1;NKX1-2;NKX2-1;NKX2-2;NKX2-3;NKX2-4;NKX2-5;NKX2-6;NKX2-8;NKX3-1;NKX3-2;NKX6-1;NKX6-2;NKX6-	BSX

motif	Nº of probes	% of probes	lower OR	upper OR	OR	p.value	FDR	TF family	TF subfamily	TF.family.member	TF.subfamily.member
										3;NOTO;TLX1;TLX2;TLX3;VAX1;VAX2;VENTX	
STAT4_HUMAN.H11MO.0.A	11	0.06432749	0.35495746	13.342.764	0.7256390	342852,20000000	5,94E+05	STAT factors{6.2.1}	STAT4{6.2.1.0.4}	STAT1;STAT2;STAT3;STAT4;STAT5A;STAT5B;STAT6	STAT4
COE1_HUMAN.H11MO.0.A	19	0.11111111	0.35372647	0.9762463	0.6038996	41484,39000000	1,56E+05	Early B-Cell Factor-related factors{6.1.5}	EBF1 (COE1){6.1.5.0.1}	EBF1;EBF2;EBF3;EBF4	EBF1
ESX1_HUMAN.H11MO.0.D	7	0.04093567	0.35285921	18.805.666	0.8918673	1000000,00000000	1,00E+06	Paired-related HD factors{3.1.3}	ESX{3.1.3.8}	ALX1;ALX3;ALX4;ARGFX;ARX;CRX;DMBX1;DPRX;DRGX;DUX4;DUXA;ESX1;GSC;GSC2;HESX1;ISX;LEUTX;MIXL1;NOBOX;OTP;OTX1;OTX2;PHOX2A;PHOX2B;PITX1;PITX2;PITX3;PROP1;PRRX1;PRRX2;RAX;RAX2;RHOXF1;RHOXF2;SEBOX;SHOX;SHOX2;TPRX1;UNCX;VSX1;VSX2	ESX1
THB_HUMAN.H11MO.1.D	17	0.09941520	0.35234161	10.268.537	0.6206337	68252,85000000	2,10E+05	Thyroid hormone receptor-related factors (NR1){2.1.2}	Thyroid hormone receptors (NR1A){2.1.2.2}	NR1D1;NR1D2;NR1H2;NR1H3;NR1H4;NR1I2;NR1I3;PPARA;PPARD;PPARG;RARA;RARB;RARG;RORA;RORB;RORC;THRA;THRB;VDR	THRA;THRB
ZFX_HUMAN.H11MO.1.A	64	0.37426901	0.35161801	0.6704152	0.4872065	4,57552800	1,60E+02	More than 3 adjacent zinc finger factors{2.3.3}	ZFX/ZFY factors{2.3.3.65}	BCL6;BCL6B;CTCF;CTCFL;FEZF1;FEZF2;GFI1;GFI1B;GLI1;GLI2;GLI3;GLI4;GLIS1;GLIS2;GLIS3;HKR1;MTF1;MYNN;MZF1;OSR2;OVOL1;OVOL2;PLAG1;PLAGL1;PLAGL2;PRDM1;PRDM14;PRDM6;SCRT1;SCRT2;SNAI1;SNAI2;SNAI3;WT1;YY1;YY2;ZBTB12;ZBTB14;ZBTB18;ZBTB20;ZBTB26;ZBTB42;ZBTB45;ZBTB47;ZBTB48;ZBTB49;ZBTB6;ZBTB7A;ZBTB7B;ZBTB7C;ZFP14;ZFP2;ZFP28;ZFP30;ZFP37;ZFP42;ZFP64;ZFP69;ZFP69B;ZFP82;ZFP91;ZFX;ZIC1;ZIC2;ZIC3;ZIC4;ZIC5;ZIK1;ZIM3;ZKSCAN1;ZKSCAN2;ZKSCAN3;ZKSCAN4;ZNF121;ZNF124;ZNF133;ZNF136;ZNF138;ZNF14;ZNF140;ZNF143;ZNF146;ZNF148;ZNF155;ZNF157;ZNF160;ZNF169;ZNF175;ZNF177;ZNF18;ZNF180;ZNF181;ZNF2;ZNF20;ZNF212;ZNF213;ZNF214;ZNF221;ZNF222;ZNF223;ZNF224;ZNF225;ZNF226;ZNF227;ZNF229;ZNF230;ZNF232;ZNF233;ZNF234;ZNF235;ZNF24;ZNF25;ZNF250;ZNF257;ZNF26;ZNF260;ZNF263;ZNF264;ZNF268;ZNF274;ZNF276;ZNF28;ZNF280A;ZNF280B;ZNF280C;ZNF280D;ZNF281;ZNF282;ZNF283;ZNF284;ZNF285;ZNF286A;ZNF286B;ZNF3;ZNF30;ZNF300;ZNF302;ZNF317;ZNF32;ZNF320;ZNF322;ZNF324;ZNF324B;ZNF329;ZNF331;ZNF333;ZNF33A;ZNF33B;ZNF343;ZNF345;ZNF347;ZNF350;ZNF354A;ZNF354B;ZNF362;ZNF366;ZNF383;ZNF384;ZNF394;ZNF397;ZNF398;ZNF404;ZNF41;ZNF410;ZNF419;ZNF420;ZNF431;ZNF432;ZNF436;ZNF439;ZNF44;ZNF440;ZNF442;ZNF443;ZNF446;ZNF449;ZNF45;ZNF460;ZNF468;ZNF479;ZNF484;ZNF490;ZNF500;ZNF502;ZNF524;ZNF525;ZNF528;ZNF543;ZNF544;ZNF546;ZNF547;ZNF548;ZNF549;ZNF554;ZNF555;ZNF557;ZNF558;ZNF559;ZNF561;ZNF562;ZNF563;ZNF564;ZNF566;ZNF567;ZNF568;ZNF57;ZNF570;ZNF571;ZNF572;ZNF577;ZNF581;ZNF582;ZNF583;ZNF585A;ZNF586;ZNF589;ZNF595;ZNF599;ZNF600;ZNF605;ZNF607;ZNF611;ZNF613;ZNF614;ZNF615;ZNF616;ZNF619;ZNF620;ZNF621;ZNF625;ZNF627;ZNF649;ZNF652;ZNF653;ZNF665;ZNF667;ZNF669;ZNF670;ZNF672;ZNF679;ZNF680;ZNF683;ZNF689;ZNF692;ZNF701;ZNF705D;ZNF705E;ZNF705G;ZNF708;ZNF709;ZNF71;ZNF710;ZNF713;ZNF721;ZNF727;ZNF729;ZNF736;ZNF75A;ZNF75D;ZNF76;ZNF763;Z	ZFX

motif	Nº of probes	% of probes	lower OR	upper OR	OR	p.value	FDR	TF family	TF subfamily	TF.family.member	TF.subfamily.member
										NF764;ZNF765;ZNF768;ZNF77;ZNF771;ZNF773;ZNF774;ZNF776;ZNF777;ZNF780A;ZNF780B;ZNF782;ZNF785;ZNF799;ZNF805;ZNF808;ZNF81;ZNF813;ZNF816;ZNF823;ZNF829;ZNF836;ZNF841;ZNF844;ZNF845;ZNF846;ZNF85;ZNF853;ZNF860;ZNF878;ZNF891;ZNF99;ZSCAN16;ZSCAN2;ZSCAN22;ZSCAN23;ZSCAN29;ZSCAN31;ZSCAN32;ZSCAN4;ZSCAN5A;ZSCAN5B;ZSCAN5C;ZSCAN9;ZXDA;ZXDB;ZXDC	
PPARG_HUMAN.H11MO.1.A	11	0.06432749	0.34973374	13.145.921	0.7149989	342972,20000000	5,94E+05	Thyroid hormone receptor-related factors (NR1){2.1.2}	PPAR (NR1C){2.1.2.5}	NR1D1;NR1D2;NR1H2;NR1H3;NR1H4;NR1I2;NR1I3;PPARA;PPARD;PPARG;RARA;RARB;RARG;RORA;RORB;RORC;THRA;THRB;VDR	PPARA;PPARD;PPARG
HINFP_HUMAN.H11MO.0.C	20	0.11695906	0.34961404	0.9422238	0.5888126	22786,88000000	1,01E+05	Factors with multiple dispersed zinc fingers{2.3.4}	HINFP-like factors{2.3.4.21}	BCL11A;BCL11B;BNC1;BNC2;E4F1;HIC1;HIC2;HINFP;HIVEP1;HIVEP2;HIVEP3;IKZF1;IKZF2;IKZF3;IKZF4;IKZF5;INSM1;INSM2;MAZ;MECOM;PATZ1;PRDM16;PRDM4;REST;RLF;RREB1;SALL1;SALL2;SALL3;SALL4;VEZF1;ZBTB1;ZBTB17;ZBTB2;ZBTB25;ZBTB4;ZFAT;ZNF134;ZNF211;ZNF217;ZNF219;ZNF248;ZNF256;ZNF292;ZNF296;ZNF319;ZNF334;ZNF335;ZNF341;ZNF37A;ZNF382;ZNF417;ZNF418;ZNF423;ZNF467;ZNF510;ZNF512;ZNF512B;ZNF516;ZNF518A;ZNF518B;ZNF521;ZNF526;ZNF532;ZNF536;ZNF552;ZNF574;ZNF587;ZNF587B;ZNF592;ZNF639;ZNF654;ZNF658;ZNF671;ZNF687;ZNF711;ZNF717;ZNF770;ZNF772;ZNF784;ZNF786;ZNF792;ZNF8;ZNF814	HINFP
ZN320_HUMAN.H11MO.0.C	34	0.19883041	0.34848763	0.7669816	0.5235240	406,80090000	4,86E+03	More than 3 adjacent zinc finger factors{2.3}	unclassified{2.3.3.0}	BCL6B;BCL6;CTCFL;CTCF;FEZF1;GFI1B;GFI1;GLI1;GLI2;GLI3;GLIS1;GLIS2;GLIS3;MTF1;MYNN;MZF1;OSR2;OVOL1;OVOL2;ZNF146;PLAG1;PLAGL1;PRDM14;PRDM1;PRDM6;SCRT1;SCRT2;SNAI1;SNAI2;YY1;YY2;WT1;ZNF324;ZNF354A;ZBTB14;ZBTB18;ZBTB48;ZBTB49;ZBTB7A;ZBTB7B;ZBTB6;ZFP64;ZFP28;ZFP42;ZFP82;ZFX;ZIC1;ZIC2;ZIC3;ZIC4;ZIM3;ZKSCAN1;ZKSCAN3;ZNF121;ZNF136;ZNF140;ZNF143;ZNF148;ZNF214;ZNF232;ZNF250;ZNF257;ZNF260;ZNF263;ZNF264;ZNF274;ZNF281;ZNF282;ZNF317;ZNF320;ZNF322;ZNF329;ZNF331;ZNF333;ZNF350;ZNF384;ZNF394;ZNF410;ZNF436;ZNF449;ZNF490;ZNF502;ZNF524;ZNF528;ZNF547;ZNF549;ZNF554;ZNF563;ZNF582;ZNF586;ZNF589;ZNF652;ZNF667;ZNF680;ZNF708;ZNF713;ZNF768;ZNF816;ZNF18;ZNF41;ZNF76;ZNF85;ZSCAN16;ZSCAN22;ZSCAN31;ZSCAN4	MYNN;MZF1;OSR2;PRDM14;PRDM6;WT1;ZBTB14;ZBTB48;ZBTB49;ZFP64;ZFP28;ZIM3;ZNF121;ZNF250;ZNF257;ZNF263;ZNF274;ZNF317;ZNF320;ZNF329;ZNF331;ZNF394;ZNF449;ZNF502;ZNF528;ZNF547;ZNF549;ZNF554;ZNF586;ZNF589;ZNF667;ZNF680;ZNF708;ZNF713;ZNF768;ZNF18;ZNF85;ZSCAN16;ZSCAN22
GCM2_HUMAN.H11MO.0.D	20	0.11695906	0.34842936	0.9390279	0.5868167	22777,16000000	1,01E+05	GCM factors{7.2.1}	GCMb (GCM2){7.2.1.0.2}	GCM1;GCM2	GCM2

motif	Nº of probes	% of probes	lower OR	upper OR	OR	p.value	FDR	TF family	TF subfamily	TF.family.member	TF.subfamily.member
THAP1_HUMAN.H11MO.0.C	65	0.38011696	0.34798384	0.6620507	0.4815562	2,97964600	1,09E+02	THAP-related factors{2.9.1}	THAP1{2.9.1.0.1}	THAP1;THAP10;THAP11;THAP12;THAP2;THAP3;THAP4;THAP5;THAP6;THAP7;THAP8;THAP9	THAP1
KLF4_HUMAN.H11MO.0.A	37	0.21637427	0.34764316	0.7459778	0.5148987	203,67030000	3,21E+03	Three-zinc finger Krüppel-related factors{2.3.1}	Krüppel-like factors{2.3.1.2}	EGR1;EGR2;EGR3;EGR4;KLF1;KLF10;KLF11;KLF12;KLF13;KLF14;KLF15;KLF16;KLF17;KLF2;KLF3;KLF4;KLF5;KLF6;KLF7;KLF8;KLF9;SP1;SP2;SP3;SP4;SP5;SP6;SP7;SP8;SP9	KLF12;KLF13;KLF14;KLF15;KLF16;KLF1;KLF3;KLF4;KLF5;KLF6;KLF8;KLF9
GSC2_HUMAN.H11MO.0.D	14	0.08187135	0.34713718	11.235.604	0.6498028	127283,90000000	3,24E+05	Paired-related HD factors{3.1.3}	GSC{3.1.3.9}	ALX1;ALX3;ALX4;ARGFX;ARX;CRX;DMBX1;DPRX;DRGX;DUX4;DUXA;ESX1;GSC;GSC2;HESX1;ISX;LEUTX;MIXL1;NOBOX;OTP;OTX1;OTX2;PHOX2A;PHOX2B;PITX1;PITX2;PITX3;PROP1;PRRX1;PRRX2;RAX;RAX2;RHOXF1;RHOXF2;SEBOX;SHOX;SHOX2;TPRX1;UNCX;VSX1;VSX2	GSC2;GSC
ZN331_HUMAN.H11MO.0.C	24	0.14035088	0.34640965	0.8632222	0.5580379	5952,88500000	3,73E+04	More than 3 adjacent zinc finger factors{2.3.3}	unclassified{2.3.3.0}	BCL6B;BCL6;CTCFL;CTCF;FEZF1;GFI1B;GFI1;GLI1;GLI2;GLI3;GLIS1;GLIS2;GLIS3;MTF1;MYNN;MZF1;OSR2;OVOL1;OVOL2;ZNF146;PLAG1;PLAGL1;PRDM14;PRDM1;PRDM6;SCRT1;SCRT2;SNAI1;SNAI2;YY1;YY2;WT1;ZNF324;ZNF354A;ZBTB14;ZBTB18;ZBTB48;ZBTB49;ZBTB7A;ZBTB7B;ZBTB6;ZFP64;ZFP28;ZFP42;ZFP82;ZFX;ZIC1;ZIC2;ZIC3;ZIC4;ZIM3;ZKSCAN1;ZKSCAN3;ZNF121;ZNF136;ZNF140;ZNF143;ZNF148;ZNF214;ZNF232;ZNF250;ZNF257;ZNF260;ZNF263;ZNF264;ZNF274;ZNF281;ZNF282;ZNF317;ZNF320;ZNF322;ZNF329;ZNF331;ZNF333;ZNF350;ZNF384;ZNF394;ZNF410;ZNF436;ZNF449;ZNF490;ZNF502;ZNF524;ZNF528;ZNF547;ZNF549;ZNF554;ZNF563;ZNF582;ZNF586;ZNF589;ZNF652;ZNF667;ZNF680;ZNF708;ZNF713;ZNF768;ZNF816;ZNF18;ZNF41;ZNF76;ZNF85;ZSCAN16;ZSCAN22;ZSCAN31;ZSCAN4	MYNN;MZF1;OSR2;PRDM14;PRDM6;WT1;ZBTB14;ZBTB48;ZBTB49;ZFP64;ZFP28;ZIM3;ZNF121;ZNF250;ZNF257;ZNF263;ZNF274;ZNF317;ZNF320;ZNF329;ZNF331;ZNF394;ZNF449;ZNF502;ZNF528;ZNF547;ZNF549;ZNF554;ZNF586;ZNF589;ZNF667;ZNF680;ZNF708;ZNF713;ZNF768;ZNF18;ZNF85;ZSCAN16;ZSCAN22
ZKSC3_HUMAN.H11MO.0.D	10	0.05847953	0.34525180	13.857.436	0.7340000	394005,30000000	6,32E+05	More than 3 adjacent zinc finger factors{2.3.3}	ZKSCAN3-like factors{2.3.3.26}	BCL6;BCL6B;CTCF;CTCFL;FEZF1;FEZF2;GFI1;GFI1B;GLI1;GLI2;GLI3;GLI4;GLIS1;GLIS2;GLIS3;HKR1;MTF1;MYNN;MZF1;OSR2;OVOL1;OVOL2;PLAG1;PLAGL1;PLAGL2;PRDM1;PRDM14;PRDM6;SCRT1;SCRT2;SNAI1;SNAI2;SNAI3;WT1;YY1;YY2;ZBTB12;ZBTB14;ZBTB18;ZBTB20;ZBTB26;ZBTB42;ZBTB45;ZBTB47;ZBTB48;ZBTB49;ZBTB6;ZBTB7A;ZBTB7B;ZBTB7C;ZFP14;ZFP2;ZFP28;ZFP30;ZFP37;ZFP42;ZFP64;ZFP69;ZFP69B;ZFP82;ZFP91;ZFX;ZIC1;ZIC2;ZIC3;ZIC4;ZIC5;ZIK1;ZIM3;ZKSCAN1;ZKSCAN2;ZKSCAN3;ZKSCAN4;ZNF121;ZNF124;ZNF133;ZNF136;ZNF138;ZNF14;ZNF140;ZNF143;ZNF146;ZNF148;ZNF155;ZNF157;ZNF160;ZNF169;ZNF175;ZNF177;ZNF18;ZNF180;ZNF181;ZNF2;ZNF20;ZNF212;ZNF213;ZNF214;ZNF221;ZNF222;ZNF223;ZNF224;ZNF225;ZNF226;ZNF227;ZNF229;ZNF230;ZNF232;ZNF233;ZNF234;ZNF235;ZNF24;ZNF25;ZNF250;ZNF257;ZNF26;ZNF260;ZNF263;ZNF264;ZNF268;ZNF274;ZNF276;ZNF28;ZNF280A;ZNF280B;ZNF280C;ZNF280D;ZNF281;ZNF282;ZNF283;ZNF284;ZNF285;ZNF286A;ZNF286B;ZNF3;ZNF30;ZNF300;ZNF302;ZNF317;ZNF32;ZNF320;ZNF322;ZNF324;ZNF324B;ZNF329;ZNF331;ZNF333;ZNF33A;ZNF33B;ZNF343;ZNF345;ZNF347;ZNF350;ZNF354A;ZNF354B;ZNF362;ZNF366;ZNF383;ZNF384;ZNF394;ZNF397;ZNF398;ZNF404;ZNF41;ZNF410;ZNF419;ZNF420;ZNF431;ZNF432;ZNF436;ZNF439;ZNF44;ZNF440;ZNF442;ZNF443;ZNF446;ZNF449;ZNF45;ZNF460;ZNF468;ZNF479;ZNF484;ZNF490;ZNF500;ZNF502;ZNF524;ZNF525;ZNF528;ZNF543;ZNF544;ZNF546;ZNF547;ZNF548;ZNF549;ZNF554;ZN	ZKSCAN3

motif	Nº of probes	% of probes	lower OR	upper OR	OR	p.value	FDR	TF family	TF subfamily	TF.family.member	TF.subfamily.member
										F555;ZNF557;ZNF558;ZNF559;ZNF561;ZNF562;ZNF563;ZNF564;ZNF566;ZNF567;ZNF568;ZNF57;ZNF570;ZNF571;ZNF572;ZNF577;ZNF581;ZNF582;ZNF583;ZNF585A;ZNF586;ZNF589;ZNF595;ZNF599;ZNF600;ZNF605;ZNF607;ZNF611;ZNF613;ZNF614;ZNF615;ZNF616;ZNF619;ZNF620;ZNF621;ZNF625;ZNF627;ZNF649;ZNF652;ZNF653;ZNF665;ZNF667;ZNF669;ZNF670;ZNF672;ZNF679;ZNF680;ZNF683;ZNF689;ZNF692;ZNF701;ZNF705D;ZNF705E;ZNF705G;ZNF708;ZNF709;ZNF71;ZNF710;ZNF713;ZNF721;ZNF727;ZNF729;ZNF736;ZNF75A;ZNF75D;ZNF76;ZNF763;ZNF764;ZNF765;ZNF768;ZNF77;ZNF771;ZNF773;ZNF774;ZNF776;ZNF777;ZNF780A;ZNF780B;ZNF782;ZNF785;ZNF799;ZNF805;ZNF808;ZNF81;ZNF813;ZNF816;ZNF823;ZNF829;ZNF836;ZNF841;ZNF844;ZNF845;ZNF846;ZNF85;ZNF853;ZNF860;ZNF878;ZNF891;ZNF99;ZSCAN16;ZSCAN2;ZSCAN22;ZSCAN23;ZSCAN29;ZSCAN31;ZSCAN32;ZSCAN4;ZSCAN5A;ZSCAN5B;ZSCAN5C;ZSCAN9;ZXDA;ZXDB;ZXDC	
GLI3_HUMAN.H11MO.0.B	16	0.09356725	0.34507610	10.377.228	0.6189452	79007,72000000	2,34E+05	More than 3 adjacent zinc finger factors{2.3.3}	GLI-like factors{2.3.3.1}	BCL6;BCL6B;CTCF;CTCFL;FEZF1;FEZF2;GFI1;GFI1B;GLI1;GLI2;GLI3;GLI4;GLIS1;GLIS2;GLIS3;HKR1;MTF1;MYNN;MZF1;OSR2;OVOL1;OVOL2;PLAG1;PLAGL1;PLAGL2;PRDM1;PRDM14;PRDM6;SCRT1;SCRT2;SNAI1;SNAI2;SNAI3;WT1;YY1;YY2;ZBTB12;ZBTB14;ZBTB18;ZBTB20;ZBTB26;ZBTB42;ZBTB45;ZBTB47;ZBTB48;ZBTB49;ZBTB6;ZBTB7A;ZBTB7B;ZBTB7C;ZFP14;ZFP2;ZFP28;ZFP30;ZFP37;ZFP42;ZFP64;ZFP69;ZFP69B;ZFP82;ZFP91;ZFX;ZIC1;ZIC2;ZIC3;ZIC4;ZIC5;ZIK1;ZIM3;ZKSCAN1;ZKSCAN2;ZKSCAN3;ZKSCAN4;ZNF121;ZNF124;ZNF133;ZNF136;ZNF138;ZNF14;ZNF140;ZNF143;ZNF146;ZNF148;ZNF155;ZNF157;ZNF160;ZNF169;ZNF175;ZNF177;ZNF18;ZNF180;ZNF181;ZNF2;ZNF20;ZNF212;ZNF213;ZNF214;ZNF221;ZNF222;ZNF223;ZNF224;ZNF225;ZNF226;ZNF227;ZNF229;ZNF230;ZNF232;ZNF233;ZNF234;ZNF235;ZNF24;ZNF25;ZNF250;ZNF257;ZNF26;ZNF260;ZNF263;ZNF264;ZNF268;ZNF274;ZNF276;ZNF28;ZNF280A;ZNF280B;ZNF280C;ZNF280D;ZNF281;ZNF282;ZNF283;ZNF284;ZNF285;ZNF286A;ZNF286B;ZNF3;ZNF30;ZNF300;ZNF302;ZNF317;ZNF32;ZNF320;ZNF322;ZNF324;ZNF324B;ZNF329;ZNF331;ZNF333;ZNF33A;ZNF33B;ZNF343;ZNF345;ZNF347;ZNF350;ZNF354A;ZNF354B;ZNF362;ZNF366;ZNF383;ZNF384;ZNF394;ZNF397;ZNF398;ZNF404;ZNF41;ZNF410;ZNF419;ZNF420;ZNF431;ZNF432;ZNF436;ZNF439;ZNF44;ZNF440;ZNF442;ZNF443;ZNF446;ZNF449;ZNF45;ZNF460;ZNF468;ZNF479;ZNF484;ZNF490;ZNF500;ZNF502;ZNF524;ZNF525;ZNF528;ZNF543;ZNF544;ZNF546;ZNF547;ZNF548;ZNF549;ZNF554;ZNF555;ZNF557;ZNF558;ZNF559;ZNF561;ZNF562;ZNF563;ZNF564;ZNF566;ZNF567;ZNF568;ZNF57;ZNF570;ZNF571;ZNF572;ZNF577;ZNF581;ZNF582;ZNF583;ZNF585A;ZNF586;ZNF589;ZNF595;ZNF599;ZNF600;ZNF605;ZNF607;ZNF611;ZNF613;ZNF614;ZNF615;ZNF616;ZNF619;ZNF620;ZNF621;ZNF625;ZNF627;ZNF649;ZNF652;ZNF6	GLI1;GLI2;GLI3;GLIS1;GLIS2;GLIS3;ZIC1;ZIC2;ZIC3;ZIC4

motif	Nº of probes	% of probes	lower OR	upper OR	OR	p.value	FDR	TF family	TF subfamily	TF.family.member	TF.subfamily.member
										53;ZNF665;ZNF667;ZNF669;ZNF670;ZNF672;ZNF679;ZNF680;ZNF683;ZNF689;ZNF692;ZNF701;ZNF705D;ZNF705E;ZNF705G;ZNF708;ZNF709;ZNF71;ZNF710;ZNF713;ZNF721;ZNF727;ZNF729;ZNF736;ZNF75A;ZNF75D;ZNF76;ZNF763;ZNF764;ZNF765;ZNF768;ZNF77;ZNF771;ZNF773;ZNF774;ZNF776;ZNF777;ZNF780A;ZNF780B;ZNF782;ZNF785;ZNF799;ZNF805;ZNF808;ZNF81;ZNF813;ZNF816;ZNF823;ZNF829;ZNF836;ZNF841;ZNF844;ZNF845;ZNF846;ZNF85;ZNF853;ZNF860;ZNF878;ZNF891;ZNF99;ZSCAN16;ZSCAN2;ZSCAN22;ZSCAN23;ZSCAN29;ZSCAN31;ZSCAN32;ZSCAN4;ZSCAN5A;ZSCAN5B;ZSCAN5C;ZSCAN9;ZXDA;ZXDB;ZXDC	
TFDP1_HUMAN.H11MO.0.C	42	0.24561404	0.34430743	0.7137117	0.5001878	57,28355000	1,26E+03	E2F-related factors {3.3.2}	Dp-1{3.3.2.2}	E2F1;E2F2;E2F3;E2F4;E2F5;E2F6;E2F7;E2F8;TFDP1;TFDP2	TFDP1
MYF6_HUMAN.H11MO.0.C	19	0.11111111	0.34255551	0.9453773	0.5848206	26511,46000000	1,11E+05	MyoD / ASC-related factors {1.2.2}	Myogenic transcription factors{1.2.2.1}	ASCL1;ASCL2;ASCL3;ASCL4;ASCL5;MYF5;MYF6;MYOD1;MYOG	MYF6;MYOD1;MYOG
TBP_HUMAN.H11MO.0.A	8	0.04678363	0.34171386	16.255.983	0.8052604	740737,30000000	8,81E+05	TBP-related factors {8.1.1}	TBP{8.1.1.0.1}	TBP;TBPL1;TBPL2	TBP
INSM1_HUMAN.H11MO.0.C	19	0.11111111	0.34168287	0.9429658	0.5833299	26575,06000000	1,11E+05	Factors with multiple dispersed zinc fingers {2.3.4}	Insulinoma-associated proteins{2.3.4.16}	BCL11A;BCL11B;BNC1;BNC2;E4F1;HIC1;HIC2;HINFP;HIVEP1;HIVEP2;HIVEP3;IKZF1;IKZF2;IKZF3;IKZF4;IKZF5;INSM1;INSM2;MAZ;MECOM;PATZ1;PRDM16;PRDM4;REST;RLF;RREB1;SALL1;SALL2;SALL3;SALL4;VEZF1;ZBTB1;ZBTB17;ZBTB2;ZBTB25;ZBTB4;ZFAT;ZNF134;ZNF211;ZNF217;ZNF219;ZNF248;ZNF256;ZNF292;ZNF296;ZNF319;ZNF334;ZNF335;ZNF341;ZNF37A;ZNF382;ZNF417;ZNF418;ZNF423;ZNF467;ZNF510;ZNF512;ZNF512B;ZNF516;ZNF518A;ZNF518B;ZNF521;ZNF526;ZNF532;ZNF536;ZNF552;ZNF574;ZNF587;ZNF587B;ZNF592;ZNF639;ZNF654;ZNF658;ZNF671;ZNF687;ZNF711;ZNF717;ZNF770;ZNF772;ZNF784;ZNF786;ZNF792;ZNF8;ZNF814	INSM1
GSX2_HUMAN.H11MO.0.D	8	0.04678363	0.34137392	16.239.759	0.8044573	740822,50000000	8,81E+05	HOX-related factors {3.1.1}	GSX{3.1.1.12}	CDX1;CDX2;CDX4;EVX1;EVX2;GBX1;GBX2;GSX1;GSX2;HDX;HMBOX1;HNF1A;HNF1B;HOXA1;HOXA10;HOXA11;HOXA13;HOXA2;HOXA3;HOXA4;HOXA5;HOXA6;HOXA7;HOXA9;HOXB1;HOXB13;HOXB2;HOXB3;HOXB4;HOXB5;HOXB6;HOXB7;HOXB8;HOXB9;HOXC10;HOXC11;HOXC12;HOXC13;HOXC4;HOXC5;HOXC6;HOXC8;HOXC9;HOXD1;HOXD10;HOXD11;HOXD12;HOXD13;HOXD3;HOXD4;HOXD8;HOXD9;MEOX1;MEOX2;MNX1;PDX1;POU1F1;POU2F1;POU2F2;POU2F3;POU3F1;POU3F2;POU3F3;POU3F4;POU4F1;POU4F2;POU4F3;POU5F1;POU5F2;POU6F1;POU6F2	GSX1;GSX2
ZN335_HUMAN.H11MO.0.A	30	0.17543860	0.34061772	0.7809994	0.5235567	924,00010000	8,19E+03	Factors with multiple dispers	unclassified{2.3.4.0}	BCL11A;E4F1;MECOM;HIC1;HIC2;HINFP;IKZF1;INSM1;MAZ;PATZ1;PRDM4;REST;RREB1;SALL4;VEZF1;ZBTB17;ZBTB4;HIVEP1;HIVEP2;Z	E4F1;PRDM4;REST;RREB1;ZBTB17;ZBTB4;ZNF335;ZNF341;ZNF467;ZNF770;ZNF784;ZNF8

motif	Nº of probes	% of probes	lower OR	upper OR	OR	p.value	FDR	TF family	TF subfamily	TF.family.member	TF.subfamily.member
								ed zinc fingers {2.3.4}		NF134;ZNF219;ZNF335;ZNF341;ZNF382;ZNF418;ZNF423;ZNF467;ZNF770;ZNF784;ZNF8	
HIF1A_HUMAN.H11MO.0.C	25	0.14619883	0.34033061	0.8343887	0.5432361	3928,22400000	2,59E+04	PAS domain factors{1.2.5}	Ahr-like factors{1.2.5.1}	AHR;AHRR;ARNT;ARNT2;ARNTL;ARNTL2;CLOCK;EPAS1;HIF1A;HIF3A;NCOA1;NCOA2;NCOA3;NPAS1;NPAS2;NPAS3;NPAS4;SIM1;SIM2;SOHLH1;SOHLH2;TCFL5	AHR;EPAS1;HIF1A
NR1H2_HUMAN.H11MO.0.D	8	0.04678363	0.33805636	16.081.453	0.7966210	625510,80000000	8,13E+05	Thyroid hormone receptor-related factors (NR1){2.1.2}	LXR (NR1H){2.1.2.7}	NR1D1;NR1D2;NR1H2;NR1H3;NR1H4;NR1I2;NR1I3;PPARA;PPARD;PPARG;RARA;RARB;RARG;RORA;RORB;RORC;THRA;THRB;VDR	NR1H2;NR1H3;NR1H4
ZIC4_HUMAN.H11MO.0.D	31	0.18128655	0.33747492	0.7652327	0.5154931	572,46370000	5,84E+03	More than 3 adjacent zinc finger factors{2.3.3}	GLI-like factors{2.3.3.1}	BCL6;BCL6B;CTCF;CTCFL;FEZF1;FEZF2;GFI1;GFI1B;GLI1;GLI2;GLI3;GLI4;GLIS1;GLIS2;GLIS3;HKR1;MTF1;MYNN;MZF1;OSR2;OVOL1;OVOL2;PLAG1;PLAGL1;PLAGL2;PRDM1;PRDM14;PRDM6;SCRT1;SCRT2;SNAI1;SNAI2;SNAI3;WT1;YY1;YY2;ZBTB12;ZBTB14;ZBTB18;ZBTB20;ZBTB26;ZBTB42;ZBTB45;ZBTB47;ZBTB48;ZBTB49;ZBTB6;ZBTB7A;ZBTB7B;ZBTB7C;ZFP14;ZFP2;ZFP28;ZFP30;ZFP37;ZFP42;ZFP64;ZFP69;ZFP69B;ZFP82;ZFP91;ZFX;ZIC1;ZIC2;ZIC3;ZIC4;ZIC5;ZIK1;ZIM3;ZKSCAN1;ZKSCAN2;ZKSCAN3;ZKSCAN4;ZNF121;ZNF124;ZNF133;ZNF136;ZNF138;ZNF14;ZNF140;ZNF143;ZNF146;ZNF148;ZNF155;ZNF157;ZNF160;ZNF169;ZNF175;ZNF177;ZNF18;ZNF180;ZNF181;ZNF2;ZNF20;ZNF212;ZNF213;ZNF214;ZNF221;ZNF222;ZNF223;ZNF224;ZNF225;ZNF226;ZNF227;ZNF229;ZNF230;ZNF232;ZNF233;ZNF234;ZNF235;ZNF24;ZNF25;ZNF250;ZNF257;ZNF26;ZNF260;ZNF263;ZNF264;ZNF268;ZNF274;ZNF276;ZNF28;ZNF280A;ZNF280B;ZNF280C;ZNF280D;ZNF281;ZNF282;ZNF283;ZNF284;ZNF285;ZNF286A;ZNF286B;ZNF3;ZNF30;ZNF300;ZNF302;ZNF317;ZNF32;ZNF320;ZNF322;ZNF324;ZNF324B;ZNF329;ZNF331;ZNF333;ZNF33A;ZNF33B;ZNF343;ZNF345;ZNF347;ZNF350;ZNF354A;ZNF354B;ZNF362;ZNF366;ZNF383;ZNF384;ZNF394;ZNF397;ZNF398;ZNF404;ZNF41;ZNF410;ZNF419;ZNF420;ZNF431;ZNF432;ZNF436;ZNF439;ZNF44;ZNF440;ZNF442;ZNF443;ZNF446;ZNF449;ZNF45;ZNF460;ZNF468;ZNF479;ZNF484;ZNF490;ZNF500;ZNF502;ZNF524;ZNF525;ZNF528;ZNF543;ZNF544;ZNF546;ZNF547;ZNF548;ZNF549;ZNF554;ZNF555;ZNF557;ZNF558;ZNF559;ZNF561;ZNF562;ZNF563;ZNF564;ZNF566;ZNF567;ZNF568;ZNF57;ZNF570;ZNF571;ZNF572;ZNF577;ZNF581;ZNF582;ZNF583;ZNF585A;ZNF586;ZNF589;ZNF595;ZNF599;ZNF600;ZNF605;ZNF607;ZNF611;ZNF613;ZNF614;ZNF615;ZNF616;ZNF619;ZNF620;ZNF621;ZNF625;ZNF627;ZNF649;ZNF652;ZNF653;ZNF665;ZNF667;ZNF669;ZNF670;ZNF672;ZNF679;ZNF680;ZNF683;ZNF689;ZNF692;ZNF701;ZNF705D;ZNF705E;ZNF705G;ZNF708;ZNF709;ZNF71;ZNF710;ZNF713;ZNF721;ZNF727;ZNF729;ZNF736;ZNF75A;ZNF75D;ZNF76;ZNF763;ZNF764;ZNF765;ZNF768;ZNF77;ZNF771;ZNF773;ZNF774;ZNF776;ZNF777;ZNF780A;ZNF780B;ZNF782;ZNF785;ZNF799;ZNF805;ZNF808;ZNF81;ZNF813;ZNF816;ZNF823;ZNF829;ZNF836;ZNF841;ZNF844;ZNF845;ZNF846;ZNF85;ZNF853;ZNF860;ZNF878;ZNF891;ZNF99;ZSCAN16;ZSCAN2;ZSCAN22;ZSCAN23;ZSCAN29;ZSCAN31;ZSCAN32;ZSCAN4;ZSCAN5A;ZSCAN5B;ZSCAN5C;ZSCAN9;ZXDA;ZXDB;ZXDC	GLI1;GLI2;GLI3;GLIS1;GLIS2;GLIS3;ZIC1;ZIC2;ZIC3;ZIC4

motif	Nº of probes	% of probes	lower OR	upper OR	OR	p.value	FDR	TF family	TF subfamily	TF.family.member	TF.subfamily.member
NFYB_HUMAN.H11MO.0.A	7	0.04093567	0.33695627	17.957.474	0.8516481	857176,00000000	9,46E+05	Heteromeric CCAAT-binding factors{4.2.1}	NF-YB (CP1B, CBF-A){4.2.1.0.2}	NFYA;NFYB;NFYC	NFYB
E2F4_HUMAN.H11MO.1.A	32	0.18713450	0.33681134	0.7557468	0.5114595	349,14780000	4,49E+03	E2F-related factors{3.3.2}	E2F{3.3.2.1}	E2F1;E2F2;E2F3;E2F4;E2F5;E2F6;E2F7;E2F8;TFDP1;TFDP2	E2F1;E2F2;E2F3;E2F4;E2F5;E2F6;E2F7;E2F8
AP2C_HUMAN.H11MO.0.A	29	0.16959064	0.33625871	0.7801236	0.5202712	850,97590000	7,72E+03	AP-2{1.3.1}	AP-2gamma{1.3.1.0.3}	TFAP2A;TFAP2B;TFAP2C;TFAP2D	TFAP2C
KLF14_HUMAN.H11MO.0.D	37	0.21637427	0.33499385	0.7187742	0.4961258	81,98965000	1,58E+03	Three-zinc finger Krüppel-related factors{2.3.1}	Krüppel-like factors{2.3.1.2}	EGR1;EGR2;EGR3;EGR4;KLF1;KLF10;KLF11;KLF12;KLF13;KLF14;KLF15;KLF16;KLF17;KLF2;KLF3;KLF4;KLF5;KLF6;KLF7;KLF8;KLF9;SP1;SP2;SP3;SP4;SP5;SP6;SP7;SP8;SP9	KLF12;KLF13;KLF14;KLF15;KLF16;KLF1;KLF3;KLF4;KLF5;KLF6;KLF8;KLF9
OTX2_HUMAN.H11MO.0.A	7	0.04093567	0.33456149	17.829.787	0.8456120	857366,80000000	9,46E+05	Paired-related HD factors{3.1.3}	OTX{3.1.3.17}	ALX1;ALX3;ALX4;ARGFX;ARX;CRX;DMBX1;DPRX;DRGX;DUX4;DUXA;ESX1;GSC;GSC2;HESX1;ISX;LEUTX;MIXL1;NOBOX;OTP;OTX1;OTX2;PHOX2A;PHOX2B;PITX1;PITX2;PITX3;PROP1;PRRX1;PRRX2;RAX;RAX2;RHOXF1;RHOXF2;SEBOX;SHOX;SHOX2;TPRX1;UNCX;VSX1;VSX2	CRX;OTX1;OTX2
ZBT18_HUMAN.H11MO.0.C	18	0.10526316	0.33418019	0.9465791	0.5791052	24432,56000000	1,05E+05	More than 3 adjacent zinc finger factors{2.3.3}	ZNF238-like factors{2.3.3.16}	BCL6;BCL6B;CTCF;CTCFL;FEZF1;FEZF2;GFI1;GFI1B;GLI1;GLI2;GLI3;GLI4;GLIS1;GLIS2;GLIS3;HKR1;MTF1;MYNN;MZF1;OSR2;OVOL1;OVOL2;PLAG1;PLAGL1;PLAGL2;PRDM1;PRDM14;PRDM6;SCRT1;SCRT2;SNAI1;SNAI2;SNAI3;WT1;YY1;YY2;ZBTB12;ZBTB14;ZBTB18;ZBTB20;ZBTB26;ZBTB42;ZBTB45;ZBTB47;ZBTB48;ZBTB49;ZBTB6;ZBTB7A;ZBTB7B;ZBTB7C;ZFP14;ZFP2;ZFP28;ZFP30;ZFP37;ZFP42;ZFP64;ZFP69;ZFP69B;ZFP82;ZFP91;ZFX;ZIC1;ZIC2;ZIC3;ZIC4;ZIC5;ZIK1;ZIM3;ZKSCAN1;ZKSCAN2;ZKSCAN3;ZKSCAN4;ZNF121;ZNF124;ZNF133;ZNF136;ZNF138;ZNF14;ZNF140;ZNF143;ZNF146;ZNF148;ZNF155;ZNF157;ZNF160;ZNF169;ZNF175;ZNF177;ZNF18;ZNF180;ZNF181;ZNF2;ZNF20;ZNF212;ZNF213;ZNF214;ZNF221;ZNF222;ZNF223;ZNF224;ZNF225;ZNF226;ZNF227;ZNF229;ZNF230;ZNF232;ZNF233;ZNF234;ZNF235;ZNF24;ZNF25;ZNF250;ZNF257;ZNF26;ZNF260;ZNF263;ZNF264;ZNF268;ZNF274;ZNF276;ZNF28;ZNF280A;ZNF280B;ZNF280C;ZNF280D;ZNF281;ZNF282;ZNF283;ZNF284;ZNF285;ZNF286A;ZNF286B;ZNF3;ZNF30;ZNF300;ZNF302;ZNF317;ZNF32;ZNF320;ZNF322;ZNF324;ZNF324B;ZNF329;ZNF331;ZNF333;ZNF33A;ZNF33B;ZNF343;ZNF345;ZNF347;ZNF350;ZNF354A;ZNF354B;ZNF362;ZNF366;ZNF383;ZNF384;ZNF394;ZNF397;ZNF398;ZNF404;ZNF41;ZNF410;ZNF419;ZNF420;ZNF431;ZNF432;ZNF436;ZNF439;ZNF44;ZNF440;ZNF442;ZNF443;ZNF446;ZNF449;ZNF45;ZNF460;ZNF468;ZNF479;ZNF484;ZNF490;ZNF500;ZNF502;ZNF524;ZNF525;ZNF528;ZNF543;ZNF544;ZNF546;ZNF547;ZNF548;ZNF549;ZNF554;ZNF555;ZNF557;ZNF558;ZNF559;ZNF561;ZNF562;ZNF563;ZNF564;ZNF566;ZNF567;ZNF568;ZNF57;ZNF570;ZNF571;ZNF572;ZNF577;ZNF581;ZNF582;ZNF583;ZNF585A;ZNF586;ZNF589;ZNF595;ZNF599;ZNF600;ZNF605;ZNF607;ZNF611;ZNF613;ZNF614;ZNF615;ZNF616;ZNF619;ZNF620;ZNF621;ZNF625;ZNF627;ZNF649;ZNF652;ZNF653;ZNF665;ZNF667;ZNF669;ZNF670;ZNF672;ZNF679;ZNF680;ZNF683;ZNF689;ZNF692;ZNF701;ZNF705D;ZNF705E;ZNF705G;ZNF708;ZNF709;ZNF71;ZNF710;ZNF713;ZNF721;ZNF727;ZNF	ZBTB18

motif	Nº of probes	% of probes	lower OR	upper OR	OR	p.value	FDR	TF family	TF subfamily	TF.family.member	TF.subfamily.member
										729;ZNF736;ZNF75A;ZNF75D;ZNF76;ZNF763;ZNF764;ZNF765;ZNF768;ZNF77;ZNF771;ZNF773;ZNF774;ZNF776;ZNF777;ZNF780A;ZNF780B;ZNF782;ZNF785;ZNF799;ZNF805;ZNF808;ZNF81;ZNF813;ZNF816;ZNF823;ZNF829;ZNF836;ZNF841;ZNF844;ZNF845;ZNF846;ZNF85;ZNF853;ZNF860;ZNF878;ZNF891;ZNF99;ZSCAN16;ZSCAN2;ZSCAN22;ZSCAN23;ZSCAN29;ZSCAN31;ZSCAN32;ZSCAN4;ZSCAN5A;ZSCAN5B;ZSCAN5C;ZSCAN9;ZXDA;ZXDB;ZXDC	
NR1H4_HUMAN.H11MO.0.B	33	0.19298246	0.33409989	0.7421929	0.5045157	210,88680000	3,21E+03	Thyroid hormone receptor-related factors (NR1){2.1.2}	LXR (NR1H){2.1.2.7}	NR1D1;NR1D2;NR1H2;NR1H3;NR1H4;NR1I2;NR1I3;PPARA;PPARD;PPARG;RARA;RARB;RARG;RORA;RORB;RORC;THRA;THRB;VDR	NR1H2;NR1H3;NR1H4
ZIC3_HUMAN.H11MO.0.B	30	0.17543860	0.33353520	0.7647002	0.5126436	527,79510000	5,65E+03	More than 3 adjacent zinc finger factors{2.3.3}	GLI-like factors{2.3.3.1}	BCL6;BCL6B;CTCF;CTCFL;FEZF1;FEZF2;GFI1;GFI1B;GLI1;GLI2;GLI3;GLI4;GLIS1;GLIS2;GLIS3;HKR1;MTF1;MYNN;MZF1;OSR2;OVOL1;OVOL2;PLAG1;PLAGL1;PLAGL2;PRDM1;PRDM14;PRDM6;SCRT1;SCRT2;SNAI1;SNAI2;SNAI3;WT1;YY1;YY2;ZBTB12;ZBTB14;ZBTB18;ZBTB20;ZBTB26;ZBTB42;ZBTB45;ZBTB47;ZBTB48;ZBTB49;ZBTB6;ZBTB7A;ZBTB7B;ZBTB7C;ZFP14;ZFP2;ZFP28;ZFP30;ZFP37;ZFP42;ZFP64;ZFP69;ZFP69B;ZFP82;ZFP91;ZFX;ZIC1;ZIC2;ZIC3;ZIC4;ZIC5;ZIK1;ZIM3;ZKSCAN1;ZKSCAN2;ZKSCAN3;ZKSCAN4;ZNF121;ZNF124;ZNF133;ZNF136;ZNF138;ZNF14;ZNF140;ZNF143;ZNF146;ZNF148;ZNF155;ZNF157;ZNF160;ZNF169;ZNF175;ZNF177;ZNF18;ZNF180;ZNF181;ZNF2;ZNF20;ZNF212;ZNF213;ZNF214;ZNF221;ZNF222;ZNF223;ZNF224;ZNF225;ZNF226;ZNF227;ZNF229;ZNF230;ZNF232;ZNF233;ZNF234;ZNF235;ZNF24;ZNF25;ZNF250;ZNF257;ZNF26;ZNF260;ZNF263;ZNF264;ZNF268;ZNF274;ZNF276;ZNF28;ZNF280A;ZNF280B;ZNF280C;ZNF280D;ZNF281;ZNF282;ZNF283;ZNF284;ZNF285;ZNF286A;ZNF286B;ZNF3;ZNF30;ZNF300;ZNF302;ZNF317;ZNF32;ZNF320;ZNF322;ZNF324;ZNF324B;ZNF329;ZNF331;ZNF333;ZNF33A;ZNF33B;ZNF343;ZNF345;ZNF347;ZNF350;ZNF354A;ZNF354B;ZNF362;ZNF366;ZNF383;ZNF384;ZNF394;ZNF397;ZNF398;ZNF404;ZNF41;ZNF410;ZNF419;ZNF420;ZNF431;ZNF432;ZNF436;ZNF439;ZNF44;ZNF440;ZNF442;ZNF443;ZNF446;ZNF449;ZNF45;ZNF460;ZNF468;ZNF479;ZNF484;ZNF490;ZNF500;ZNF502;ZNF524;ZNF525;ZNF528;ZNF543;ZNF544;ZNF546;ZNF547;ZNF548;ZNF549;ZNF554;ZNF555;ZNF557;ZNF558;ZNF559;ZNF561;ZNF562;ZNF563;ZNF564;ZNF566;ZNF567;ZNF568;ZNF57;ZNF570;ZNF571;ZNF572;ZNF577;ZNF581;ZN	GLI1;GLI2;GLI3;GLIS1;GLIS2;GLIS3;ZIC1;ZIC2;ZIC3;ZIC4

motif	Nº of probes	% of probes	lower OR	upper OR	OR	p.value	FDR	TF family	TF subfamily	TF.family.member	TF.subfamily.member
										F582;ZNF583;ZNF585A;ZNF586;ZNF589;ZNF595;ZNF599;ZNF600;ZNF605;ZNF607;ZNF611;ZNF613;ZNF614;ZNF615;ZNF616;ZNF619;ZNF620;ZNF621;ZNF625;ZNF627;ZNF649;ZNF652;ZNF653;ZNF665;ZNF667;ZNF669;ZNF670;ZNF672;ZNF679;ZNF680;ZNF683;ZNF689;ZNF692;ZNF701;ZNF705D;ZNF705E;ZNF705G;ZNF708;ZNF709;ZNF71;ZNF710;ZNF713;ZNF721;ZNF727;ZNF729;ZNF736;ZNF75A;ZNF75D;ZNF76;ZNF763;ZNF764;ZNF765;ZNF768;ZNF77;ZNF771;ZNF773;ZNF774;ZNF776;ZNF777;ZNF780A;ZNF780B;ZNF782;ZNF785;ZNF799;ZNF805;ZNF808;ZNF81;ZNF813;ZNF816;ZNF823;ZNF829;ZNF836;ZNF841;ZNF844;ZNF845;ZNF846;ZNF85;ZNF853;ZNF860;ZNF878;ZNF891;ZNF99;ZSCAN16;ZSCAN2;ZSCAN22;ZSCAN23;ZSCAN29;ZSCAN31;ZSCAN32;ZSCAN4;ZSCAN5A;ZSCAN5B;ZSCAN5C;ZSCAN9;ZXDA;ZXDB;ZXDC	
NGN2_HUMAN.H11MO.0.D	20	0.11695906	0.33339514	0.8985038	0.5615314	14432,73000000	7,28E+04	Tal-related factors{1.2.3}	Neurogenin / Atonal-like factors{1.2.3.4}	ATOH1;ATOH7;ATOH8;BHLHA15;BHLHA9;BHLHE22;BHLHE23;FERD3L;FIGLA;HAND1;HAND2;LYL1;MESP1;MESP2;MSC;MSGN1;NEUROD1;NEUROD2;NEUROD4;NEUROD6;NEUROG1;NEUROG2;NEUROG3;NHLH1;NHLH2;OLIG1;OLIG2;OLIG3;PTF1A;SCX;TAL1;TAL2;TCF15;TCF21;TCF23;TWIST1;TWIST2	ATOH1;BHLHA15;BHLHE22;BHLHE23;NEUROD1;NEUROD2;NEUROG2;OLIG1;OLIG2;OLIG3
PLAG1_HUMAN.H11MO.0.D	35	0.20467836	0.32885611	0.7173812	0.4915831	97,42704000	1,75E+03	More than 3 adjacent zinc finger factors{2.3.3}	PLAG factors{2.3.3.25}	BCL6;BCL6B;CTCF;CTCFL;FEZF1;FEZF2;GFI1;GFI1B;GLI1;GLI2;GLI3;GLI4;GLIS1;GLIS2;GLIS3;HKR1;MTF1;MYNN;MZF1;OSR2;OVOL1;OVOL2;PLAG1;PLAGL1;PLAGL2;PRDM1;PRDM14;PRDM6;SCRT1;SCRT2;SNAI1;SNAI2;SNAI3;WT1;YY1;YY2;ZBTB12;ZBTB14;ZBTB18;ZBTB20;ZBTB26;ZBTB42;ZBTB45;ZBTB47;ZBTB48;ZBTB49;ZBTB6;ZBTB7A;ZBTB7B;ZBTB7C;ZFP14;ZFP2;ZFP28;ZFP30;ZFP37;ZFP42;ZFP64;ZFP69;ZFP69B;ZFP82;ZFP91;ZFX;ZIC1;ZIC2;ZIC3;ZIC4;ZIC5;ZIK1;ZIM3;ZKSCAN1;ZKSCAN2;ZKSCAN3;ZKSCAN4;ZNF121;ZNF124;ZNF133;ZNF136;ZNF138;ZNF14;ZNF140;ZNF143;ZNF146;ZNF148;ZNF155;ZNF157;ZNF160;ZNF169;ZNF175;ZNF177;ZNF18;ZNF180;ZNF181;ZNF2;ZNF20;ZNF212;ZNF213;ZNF214;ZNF221;ZNF222;ZNF223;ZNF224;ZNF225;ZNF226;ZNF227;ZNF229;ZNF230;ZNF232;ZNF233;ZNF234;ZNF235;ZNF24;ZNF25;ZNF250;ZNF257;ZNF26;ZNF260;ZNF263;ZNF264;ZNF268;ZNF274;ZNF276;ZNF28;ZNF280A;ZNF280B;ZNF280C;ZNF280D;ZNF281;ZNF282;ZNF283;ZNF284;ZNF285;ZNF286A;ZNF286B;ZNF3;ZNF30;ZNF300;ZNF302;ZNF317;ZNF32;ZNF320;ZNF322;ZNF324;ZNF324B;ZNF329;ZNF331;ZNF333;ZNF33A;ZNF33B;ZNF343;ZNF345;ZNF347;ZNF350;ZNF354A;ZNF354B;ZNF362;ZNF366;ZNF383;ZNF384;ZNF394;ZNF397;ZNF398;ZNF404;ZNF41;ZNF410;ZNF419;ZNF420;ZNF431;ZNF432;ZNF436;ZNF439;ZNF44;ZNF440;ZNF442;ZNF443;ZNF446;ZNF449;ZNF45;ZNF460;ZNF468;ZNF479;ZNF484;ZNF490;ZNF500;ZNF502;ZNF524;ZNF525;ZNF528;ZNF543;ZNF544;ZNF546;ZNF547;ZNF548;ZNF549;ZNF554;ZN	PLAG1;PLAGL1

motif	Nº of probes	% of probes	lower OR	upper OR	OR	p.value	FDR	TF family	TF subfamily	TF.family.member	TF.subfamily.member
										F555;ZNF557;ZNF558;ZNF559;ZNF561;ZNF562;ZNF563;ZNF564;ZNF566;ZNF567;ZNF568;ZNF57;ZNF570;ZNF571;ZNF572;ZNF577;ZNF581;ZNF582;ZNF583;ZNF585A;ZNF586;ZNF589;ZNF595;ZNF599;ZNF600;ZNF605;ZNF607;ZNF611;ZNF613;ZNF614;ZNF615;ZNF616;ZNF619;ZNF620;ZNF621;ZNF625;ZNF627;ZNF649;ZNF652;ZNF653;ZNF665;ZNF667;ZNF669;ZNF670;ZNF672;ZNF679;ZNF680;ZNF683;ZNF689;ZNF692;ZNF701;ZNF705D;ZNF705E;ZNF705G;ZNF708;ZNF709;ZNF71;ZNF710;ZNF713;ZNF721;ZNF727;ZNF729;ZNF736;ZNF75A;ZNF75D;ZNF76;ZNF763;ZNF764;ZNF765;ZNF768;ZNF77;ZNF771;ZNF773;ZNF774;ZNF776;ZNF777;ZNF780A;ZNF780B;ZNF782;ZNF785;ZNF799;ZNF805;ZNF808;ZNF81;ZNF813;ZNF816;ZNF823;ZNF829;ZNF836;ZNF841;ZNF844;ZNF845;ZNF846;ZNF85;ZNF853;ZNF860;ZNF878;ZNF891;ZNF99;ZSCAN16;ZSCAN2;ZSCAN22;ZSCAN23;ZSCAN29;ZSCAN31;ZSCAN32;ZSCAN4;ZSCAN5A;ZSCAN5B;ZSCAN5C;ZSCAN9;ZXDA;ZXDB;ZXDC	
CENPB_HUMAN.H11MO.0.D	15	0.08771930	0.32838065	10.224.221	0.6009819	58806,60000000	1,95E+05	*NA*	*NA*	CENPB	CENPB
SP3_HUMAN.H11MO.0.B	73	0.42690058	0.32644799	0.6134388	0.4483624	0,20029280	8,58E+00	Three-zinc finger Krüppel-related factors {2.3.1}	Sp1-like factors{2.3.1.1}	EGR1;EGR2;EGR3;EGR4;KLF1;KLF10;KLF11;KLF12;KLF13;KLF14;KLF15;KLF16;KLF17;KLF2;KLF3;KLF4;KLF5;KLF6;KLF7;KLF8;KLF9;SP1;SP2;SP3;SP4;SP5;SP6;SP7;SP8;SP9	SP1;SP2;SP3;SP4
HXA13_HUMAN.H11MO.0.C	7	0.04093567	0.32545946	17.343.480	0.8225316	858642,30000000	9,46E+05	HOX-related factors {3.1.1}	HOX9-13{3.1.1.8}	CDX1;CDX2;CDX4;EVX1;EVX2;GBX1;GBX2;GSX1;GSX2;HDX;HMBOX1;HNF1A;HNF1B;HOXA1;HOXA10;HOXA11;HOXA13;HOXA2;HOXA3;HOXA4;HOXA5;HOXA6;HOXA7;HOXA9;HOXB1;HOXB13;HOXB2;HOXB3;HOXB4;HOXB5;HOXB6;HOXB7;HOXB8;HOXB9;HOXC10;HOXC11;HOXC12;HOXC13;HOXC4;HOXC5;HOXC6;HOXC8;HOXC9;HOXD1;HOXD10;HOXD11;HOXD12;HOXD13;HOXD3;HOXD4;HOXD8;HOXD9;MEOX1;MEOX2;MNX1;PDX1;POU1F1;POU2F1;POU2F2;POU2F3;POU3F1;POU3F2;POU3F3;POU3F4;POU4F1;POU4F2;POU4F3;POU5F1;POU5F2;POU6F1;POU6F2	HOXA10;HOXA11;HOXA13;HOXA9;HOXB13;HOXC10;HOXC11;HOXC12;HOXC13;HOXC9;HOXD10;HOXD11;HOXD12;HOXD13;HOXD9
ZN341_HUMAN.H11MO.1.C	18	0.10526316	0.32518818	0.9211723	0.5635311	19378,62000000	9,00E+04	Factors with multiple dispersed zinc fingers {2.3.4}	unclassified{2.3.4.0}	BCL11A;E4F1;MECOM;HIC1;HIC2;HINFP;IKZF1;INSM1;MAZ;PATZ1;PRDM4;REST;RREB1;SALL4;VEZF1;ZBTB17;ZBTB4;HIVEP1;HIVEP2;ZNF134;ZNF219;ZNF335;ZNF341;ZNF382;ZNF418;ZNF423;ZNF467;ZNF770;ZNF784;ZNF8	E4F1;PRDM4;REST;RREB1;ZBTB17;ZBTB4;ZNF335;ZNF341;ZNF467;ZNF770;ZNF784;ZNF8
FOXI1_HUMAN.H11MO.0.B	7	0.04093567	0.32503040	17.320.662	0.8214495	725798,00000000	8,77E+05	Forkhead box (FOX) factors {3.3.1}	FOXI{3.3.1.9}	FOXA1;FOXA2;FOXA3;FOXB1;FOXB2;FOXC1;FOXC2;FOXD1;FOXD2;FOXD3;FOXD4;FOXD4L1;FOXD4L3;FOXD4L4;FOXD4L5;FOXD4L6;FOXE1;FOXE3;FOXF1;FOXF2;FOXG1;FOXH1;FOXI1;FOXI2;FOXI3;FOXJ1;FOXJ2;FOXJ3;FOXK1;FOXK2;FOXL1;FOXL2;FOXM1;FOXN1;FOXN2;FOXN3;FOXN4;FOXO1;FOXO3;FOXO4;FOXO6;FOXP1;FOXP2;FOXP3;FOXP4;FOXQ1;FOXR1;FOXR2;FOXS1	FOXI1
SP1_HUMAN.H11MO.1.A	63	0.36842105	0.32487972	0.6207204	0.4507274	0,38156210	1,55E+01	Three-zinc finger Krüppel-related factors {2.3.1}	Sp1-like factors{2.3.1.1}	EGR1;EGR2;EGR3;EGR4;KLF1;KLF10;KLF11;KLF12;KLF13;KLF14;KLF15;KLF16;KLF17;KLF2;KLF3;KLF4;KLF5;KLF6;KLF7;KLF8;KLF9;SP1;SP2;SP3;SP4;SP5;SP6;SP7;SP8;SP9	SP1;SP2;SP3;SP4

motif	Nº of probes	% of probes	lower OR	upper OR	OR	p.value	FDR	TF family	TF subfamily	TF.family.member	TF.subfamily.member
PBX2_HUMAN.H11MO.0.C	6	0.03508772	0.32390339	19.930.970	0.8958326	1000000,00000000	1,00E+06	TALE-type homeo domain factors{3.1.4}	PBX{3.1.4.4}	IRX2;IRX3;MEIS1;MEIS2;MEIS3;PBX1;PBX2;PBX3;PKNOX1;TGIF2LX;TGIF1;TGIF2	PBX1;PBX2;PBX3
VENTX_HUMAN.H11MO.0.D	7	0.04093567	0.32237391	17.179.329	0.8147475	725780,30000000	8,77E+05	NK-related factors{3.1.2}	VENTX{3.1.2.23}	BARHL1;BARHL2;BARX1;BARX2;BSX;DBX1;DBX2;DLX1;DLX2;DLX3;DLX4;DLX5;DLX6;EMX1;EMX2;EN1;EN2;HHEX;HLX;HMX1;HMX2;HMX3;LBX1;LBX2;MSX1;MSX2;NANOG;NKX1-1;NKX1-2;NKX2-1;NKX2-2;NKX2-3;NKX2-4;NKX2-5;NKX2-6;NKX2-8;NKX3-1;NKX3-2;NKX6-1;NKX6-2;NKX6-3;NOTO;TLX1;TLX2;TLX3;VAX1;VAX2;VENTX	VENTX
KAISO_HUMAN.H11MO.0.A	27	0.15789474	0.32108585	0.7644410	0.5040795	548,63790000	5,72E+03	Other factors with up to three adjacent zinc fingers{2.3.2}	Factors with 2-3 adjacent zinc fingersand a BTB/POZ domain{2.3.2.1}	AEBP2;OSR1;OVOL3;ZBTB22;ZBTB32;ZBTB33;ZBTB34;ZBTB37;ZBTB43;ZBTB46;ZBTB5;ZBTB8B;ZFPM2;ZNF174;ZNF396;ZNF414;ZNF446;ZNF487;ZNF511;ZNF580;ZNF705A;ZNF740;ZSCAN1	ZBTB33
ZIC1_HUMAN.H11MO.0.B	28	0.16374269	0.31925227	0.7500196	0.4974728	448,66720000	5,16E+03	More than 3 adjacent zinc finger factors{2.3.3}	GLI-like factors{2.3.3.1}	BCL6;BCL6B;CTCF;CTCFL;FEZF1;FEZF2;GFI1;GFI1B;GLI1;GLI2;GLI3;GLI4;GLIS1;GLIS2;GLIS3;HKR1;MTF1;MYNN;MZF1;OSR2;OVOL1;OVOL2;PLAG1;PLAGL1;PLAGL2;PRDM1;PRDM14;PRDM6;SCRT1;SCRT2;SNAI1;SNAI2;SNAI3;WT1;YY1;YY2;ZBTB12;ZBTB14;ZBTB18;ZBTB20;ZBTB26;ZBTB42;ZBTB45;ZBTB47;ZBTB48;ZBTB49;ZBTB6;ZBTB7A;ZBTB7B;ZBTB7C;ZFP14;ZFP2;ZFP28;ZFP30;ZFP37;ZFP42;ZFP64;ZFP69;ZFP69B;ZFP82;ZFP91;ZFX;ZIC1;ZIC2;ZIC3;ZIC4;ZIC5;ZIK1;ZIM3;ZKSCAN1;ZKSCAN2;ZKSCAN3;ZKSCAN4;ZNF121;ZNF124;ZNF133;ZNF136;ZNF138;ZNF14;ZNF140;ZNF143;ZNF146;ZNF148;ZNF155;ZNF157;ZNF160;ZNF169;ZNF175;ZNF177;ZNF18;ZNF180;ZNF181;ZNF2;ZNF20;ZNF212;ZNF213;ZNF214;ZNF221;ZNF222;ZNF223;ZNF224;ZNF225;ZNF226;ZNF227;ZNF229;ZNF230;ZNF232;ZNF233;ZNF234;ZNF235;ZNF24;ZNF25;ZNF250;ZNF257;ZNF26;ZNF260;ZNF263;ZNF264;ZNF268;ZNF274;ZNF276;ZNF28;ZNF280A;ZNF280B;ZNF280C;ZNF280D;ZNF281;ZNF282;ZNF283;ZNF284;ZNF285;ZNF286A;ZNF286B;ZNF3;ZNF30;ZNF300;ZNF302;ZNF317;ZNF32;ZNF320;ZNF322;ZNF324;ZNF324B;ZNF329;ZNF331;ZNF333;ZNF33A;ZNF33B;ZNF343;ZNF345;ZNF347;ZNF350;ZNF354A;ZNF354B;ZNF362;ZNF366;ZNF383;ZNF384;ZNF394;ZNF397;ZNF398;ZNF404;ZNF41;ZNF410;ZNF419;ZNF420;ZNF431;ZNF432;ZNF436;ZNF439;ZNF44;ZNF440;ZNF442;ZNF443;ZNF446;ZNF449;ZNF45;ZNF460;ZNF468;ZNF479;ZNF484;ZNF490;ZNF500;ZNF502;ZNF524;ZNF525;ZNF528;ZNF543;ZNF544;ZNF546;ZNF547;ZNF548;ZNF549;ZNF554;ZNF555;ZNF557;ZNF558;ZNF559;ZNF561;ZNF562;ZNF563;ZNF564;ZNF566;ZNF567;ZNF568;ZNF57;ZNF570;ZNF571;ZNF572;ZNF577;ZNF581;ZNF582;ZNF583;ZNF585A;ZNF586;ZNF589;ZNF595;ZNF599;ZNF600;ZNF605;ZNF607;ZNF611;ZNF613;ZNF614;ZNF615;ZNF616;ZNF619;ZNF620;ZNF621;ZNF625;ZNF627;ZNF649;ZNF652;ZNF653;ZNF665;ZNF667;ZNF669;ZNF670;ZNF672;ZNF679;ZNF680;ZNF683;ZNF689;ZNF692;ZNF701;ZNF705D;ZNF705E;ZNF705G;ZNF708;ZNF709;ZNF71;ZNF710;ZNF713;ZNF721;ZNF727;ZNF729;ZNF736;ZNF75A;ZNF75D;ZNF76;ZNF763;ZNF764;ZNF765;ZNF768;ZNF77;ZNF771;ZNF773;ZNF774;ZNF776;ZNF777;ZNF780A;ZNF780B;ZNF782;ZNF785;ZNF799;ZNF805;ZNF808;ZNF81	GLI1;GLI2;GLI3;GLIS1;GLIS2;GLIS3;ZIC1;ZIC2;ZIC3;ZIC4

motif	Nº of probes	% of probes	lower OR	upper OR	OR	p.value	FDR	TF family	TF subfamily	TF.family.member	TF.subfamily.member
										;ZNF813;ZNF816;ZNF823;ZNF829;ZNF836;ZNF841;ZNF844;ZNF845;ZNF846;ZNF85;ZNF853;ZNF860;ZNF878;ZNF891;ZNF99;ZSCAN16;ZSCAN2;ZSCAN22;ZSCAN23;ZSCAN29;ZSCAN31;ZSCAN32;ZSCAN4;ZSCAN5A;ZSCAN5B;ZSCAN5C;ZSCAN9;ZXDA;ZXDB;ZXDC	
ZF64A_HUMAN.H11MO.0.D	52	0.30409357	0.31889342	0.6295646	0.4507821	0,74280320	2,86E+01	More than 3 adjacent zinc finger factors{2.3.3}	unclassified{2.3.3.0}	BCL6B;BCL6;CTCFL;CTCF;FEZF1;GFI1B;GFI1;GLI1;GLI2;GLI3;GLIS1;GLIS2;GLIS3;MTF1;MYNN;MZF1;OSR2;OVOL1;OVOL2;ZNF146;PLAG1;PLAGL1;PRDM14;PRDM1;PRDM6;SCRT1;SCRT2;SNAI1;SNAI2;YY1;YY2;WT1;ZNF324;ZNF354A;ZBTB14;ZBTB18;ZBTB48;ZBTB49;ZBTB7A;ZBTB7B;ZBTB6;ZFP64;ZFP28;ZFP42;ZFP82;ZFX;ZIC1;ZIC2;ZIC3;ZIC4;ZIM3;ZKSCAN1;ZKSCAN3;ZNF121;ZNF136;ZNF140;ZNF143;ZNF148;ZNF214;ZNF232;ZNF250;ZNF257;ZNF260;ZNF263;ZNF264;ZNF274;ZNF281;ZNF282;ZNF317;ZNF320;ZNF322;ZNF329;ZNF331;ZNF333;ZNF350;ZNF384;ZNF394;ZNF410;ZNF436;ZNF449;ZNF490;ZNF502;ZNF524;ZNF528;ZNF547;ZNF549;ZNF554;ZNF563;ZNF582;ZNF586;ZNF589;ZNF652;ZNF667;ZNF680;ZNF708;ZNF713;ZNF768;ZNF816;ZNF18;ZNF41;ZNF76;ZNF85;ZSCAN16;ZSCAN22;ZSCAN31;ZSCAN4	MYNN;MZF1;OSR2;PRDM14;PRDM6;WT1;ZBTB14;ZBTB48;ZBTB49;ZFP64;ZFP28;ZIM3;ZNF121;ZNF250;ZNF257;ZNF263;ZNF274;ZNF317;ZNF320;ZNF329;ZNF331;ZNF394;ZNF449;ZNF502;ZNF528;ZNF547;ZNF549;ZNF554;ZNF586;ZNF589;ZNF667;ZNF680;ZNF708;ZNF713;ZNF768;ZNF18;ZNF85;ZSCAN16;ZSCAN22
HSF2_HUMAN.H11MO.0.A	9	0.05263158	0.31709407	13.748.713	0.7062064	377025,50000000	6,23E+05	HSF factors{3.4.1}	HSF2 (HSTF2){3.4.1.0.2}	HSF1;HSF2;HSF4;HSF5;HSFX1;HSFY1;HSFY1;HSFY2	HSF2

motif	Nº of probes	% of probes	lower OR	upper OR	OR	p.value	FDR	TF family	TF subfamily	TF.family.member	TF.subfamily.member
ZN563_HUMAN.H11MO.0.C	27	0.15789474	0.31641133	0.7532768	0.4967171	411,08990000	4,86E+03	More than 3 adjacent zinc finger factors{2.3.3}	ZNF763-like factors{2.3.3.33}	BCL6;BCL6B;CTCF;CTCFL;FEZF1;FEZF2;GFI1;GFI1B;GLI1;GLI2;GLI3;GLI4;GLIS1;GLIS2;GLIS3;HKR1;MTF1;MYNN;MZF1;OSR2;OVOL1;OVOL2;PLAG1;PLAGL1;PLAGL2;PRDM1;PRDM14;PRDM6;SCRT1;SCRT2;SNAI1;SNAI2;SNAI3;WT1;YY1;YY2;ZBTB12;ZBTB14;ZBTB18;ZBTB20;ZBTB26;ZBTB42;ZBTB45;ZBTB47;ZBTB48;ZBTB49;ZBTB6;ZBTB7A;ZBTB7B;ZBTB7C;ZFP14;ZFP2;ZFP28;ZFP30;ZFP37;ZFP42;ZFP64;ZFP69;ZFP69B;ZFP82;ZFP91;ZFX;ZIC1;ZIC2;ZIC3;ZIC4;ZIC5;ZIK1;ZIM3;ZKSCAN1;ZKSCAN2;ZKSCAN3;ZKSCAN4;ZNF121;ZNF124;ZNF133;ZNF136;ZNF138;ZNF14;ZNF140;ZNF143;ZNF146;ZNF148;ZNF155;ZNF157;ZNF160;ZNF169;ZNF175;ZNF177;ZNF18;ZNF180;ZNF181;ZNF2;ZNF20;ZNF212;ZNF213;ZNF214;ZNF221;ZNF222;ZNF223;ZNF224;ZNF225;ZNF226;ZNF227;ZNF229;ZNF230;ZNF232;ZNF233;ZNF234;ZNF235;ZNF24;ZNF25;ZNF250;ZNF257;ZNF26;ZNF260;ZNF263;ZNF264;ZNF268;ZNF274;ZNF276;ZNF28;ZNF280A;ZNF280B;ZNF280C;ZNF280D;ZNF281;ZNF282;ZNF283;ZNF284;ZNF285;ZNF286A;ZNF286B;ZNF3;ZNF30;ZNF300;ZNF302;ZNF317;ZNF32;ZNF320;ZNF322;ZNF324;ZNF324B;ZNF329;ZNF331;ZNF333;ZNF33A;ZNF33B;ZNF343;ZNF345;ZNF347;ZNF350;ZNF354A;ZNF354B;ZNF362;ZNF366;ZNF383;ZNF384;ZNF394;ZNF397;ZNF398;ZNF404;ZNF41;ZNF410;ZNF419;ZNF420;ZNF431;ZNF432;ZNF436;ZNF439;ZNF44;ZNF440;ZNF442;ZNF443;ZNF446;ZNF449;ZNF45;ZNF460;ZNF468;ZNF479;ZNF484;ZNF490;ZNF500;ZNF502;ZNF524;ZNF525;ZNF528;ZNF543;ZNF544;ZNF546;ZNF547;ZNF548;ZNF549;ZNF554;ZNF555;ZNF557;ZNF558;ZNF559;ZNF561;ZNF562;ZNF563;ZNF564;ZNF566;ZNF567;ZNF568;ZNF57;ZNF570;ZNF571;ZNF572;ZNF577;ZNF581;ZNF582;ZNF583;ZNF585A;ZNF586;ZNF589;ZNF595;ZNF599;ZNF600;ZNF605;ZNF607;ZNF611;ZNF613;ZNF614;ZNF615;ZNF616;ZNF619;ZNF620;ZNF621;ZNF625;ZNF627;ZNF649;ZNF652;ZNF653;ZNF665;ZNF667;ZNF669;ZNF670;ZNF672;ZNF679;ZNF680;ZNF683;ZNF689;ZNF692;ZNF701;ZNF705D;ZNF705E;ZNF705G;ZNF708;ZNF709;ZNF71;ZNF710;ZNF713;ZNF721;ZNF727;ZNF729;ZNF736;ZNF75A;ZNF75D;ZNF76;ZNF763;ZNF764;ZNF765;ZNF768;ZNF77;ZNF771;ZNF773;ZNF774;ZNF776;ZNF777;ZNF780A;ZNF780B;ZNF782;ZNF785;ZNF799;ZNF805;ZNF808;ZNF81;ZNF813;ZNF816;ZNF823;ZNF829;ZNF836;ZNF841;ZNF844;ZNF845;ZNF846;ZNF85;ZNF853;ZNF860;ZNF878;ZNF891;ZNF99;ZSCAN16;ZSCAN2;ZSCAN22;ZSCAN23;ZSCAN29;ZSCAN31;ZSCAN32;ZSCAN4;ZSCAN5A;ZSCAN5B;ZSCAN5C;ZSCAN9;ZXDA;ZXDB;ZXDC	ZNF136;ZNF490;ZNF563
MEOX1_HUMAN.H11MO.0.D	7	0.04093567	0.31530882	16.803.047	0.7969054	726455,90000000	8,77E+05	HOX-related factors{3.1.1}	MEOX{3.1.1.14}	CDX1;CDX2;CDX4;EVX1;EVX2;GBX1;GBX2;GSX1;GSX2;HDX;HMBOX1;HNF1A;HNF1B;HOXA1;HOXA10;HOXA11;HOXA13;HOXA2;HOXA3;HOXA4;HOXA5;HOXA6;HOXA7;HOXA9;HOXB1;HOXB13;HOXB2;HOXB3;HOXB4;HOXB5;HOXB6;HOXB7;HOXB8;HOXB9;HOXC10;HOXC11;HOXC12;HOXC13;HOXC4;HOXC5;HOXC6;HOXC8;HOXC9;HOXD1;HOXD10;HOXD11;HOXD12;HOXD13;HOXD3;HOXD4;HOXD8;HOXD9;MEOX1;MEOX2;MNX1;PDX1;POU1F1;POU2F1;POU2F2;POU2F3;POU3F1;POU3F2;POU3	MEOX1;MEOX2

motif	Nº of probes	% of probes	lower OR	upper OR	OR	p.value	FDR	TF family	TF subfamily	TF.family.member	TF.subfamily.member
										F3;POU3F4;POU4F1;POU4F2;POU4F3;POU5F1;POU5F2;POU6F1;POU6F2	
ZBT7A_HUMAN.H11MO.0.A	23	0.13450292	0.31384446	0.7957476	0.5105740	1496,12800000	1,15E+04	More than 3 adjacent zinc finger factors{2.3.3}	ZBTB7 factors{2.3.3.8}	BCL6;BCL6B;CTCF;CTCFL;FEZF1;FEZF2;GFI1;GFI1B;GLI1;GLI2;GLI3;GLI4;GLIS1;GLIS2;GLIS3;HKR1;MTF1;MYNN;MZF1;OSR2;OVOL1;OVOL2;PLAG1;PLAGL1;PLAGL2;PRDM1;PRDM14;PRDM6;SCRT1;SCRT2;SNAI1;SNAI2;SNAI3;WT1;YY1;YY2;ZBTB12;ZBTB14;ZBTB18;ZBTB20;ZBTB26;ZBTB42;ZBTB45;ZBTB47;ZBTB48;ZBTB49;ZBTB6;ZBTB7A;ZBTB7B;ZBTB7C;ZFP14;ZFP2;ZFP28;ZFP30;ZFP37;ZFP42;ZFP64;ZFP69;ZFP69B;ZFP82;ZFP91;ZFX;ZIC1;ZIC2;ZIC3;ZIC4;ZIC5;ZIK1;ZIM3;ZKSCAN1;ZKSCAN2;ZKSCAN3;ZKSCAN4;ZNF121;ZNF124;ZNF133;ZNF136;ZNF138;ZNF14;ZNF140;ZNF143;ZNF146;ZNF148;ZNF155;ZNF157;ZNF160;ZNF169;ZNF175;ZNF177;ZNF18;ZNF180;ZNF181;ZNF2;ZNF20;ZNF212;ZNF213;ZNF214;ZNF221;ZNF222;ZNF223;ZNF224;ZNF225;ZNF226;ZNF227;ZNF229;ZNF230;ZNF232;ZNF233;ZNF234;ZNF235;ZNF24;ZNF25;ZNF250;ZNF257;ZNF26;ZNF260;ZNF263;ZNF264;ZNF268;ZNF274;ZNF276;ZNF28;ZNF280A;ZNF280B;ZNF280C;ZNF280D;ZNF281;ZNF282;ZNF283;ZNF284;ZNF285;ZNF286A;ZNF286B;ZNF3;ZNF30;ZNF300;ZNF302;ZNF317;ZNF32;ZNF320;ZNF322;ZNF324;ZNF324B;ZNF329;ZNF331;ZNF333;ZNF33A;ZNF33B;ZNF343;ZNF345;ZNF347;ZNF350;ZNF354A;ZNF354B;ZNF362;ZNF366;ZNF383;ZNF384;ZNF394;ZNF397;ZNF398;ZNF404;ZNF41;ZNF410;ZNF419;ZNF420;ZNF431;ZNF432;ZNF436;ZNF439;ZNF44;ZNF440;ZNF442;ZNF443;ZNF446;ZNF449;ZNF45;ZNF460;ZNF468;ZNF479;ZNF484;ZNF490;ZNF500;ZNF502;ZNF524;ZNF525;ZNF528;ZNF543;ZNF544;ZNF546;ZNF547;ZNF548;ZNF549;ZNF554;ZNF555;ZNF557;ZNF558;ZNF559;ZNF561;ZNF562;ZNF563;ZNF564;ZNF566;ZNF567;ZNF568;ZNF57;ZNF570;ZNF571;ZNF572;ZNF577;ZNF581;ZNF582;ZNF583;ZNF585A;ZNF586;ZNF589;ZNF595;ZNF599;ZNF600;ZNF605;ZNF607;ZNF611;ZNF613;ZNF614;ZNF615;ZNF616;ZNF619;ZNF620;ZNF621;ZNF625;ZNF627;ZNF649;ZNF652;ZNF653;ZNF665;ZNF667;ZNF669;ZNF670;ZNF672;ZNF679;ZNF680;ZNF683;ZNF689;ZNF692;ZNF701;ZNF705D;ZNF705E;ZNF705G;ZNF708;ZNF709;ZNF71;ZNF710;ZNF713;ZNF721;ZNF727;ZNF729;ZNF736;ZNF75A;ZNF75D;ZNF76;ZNF763;ZNF764;ZNF765;ZNF768;ZNF77;ZNF771;ZNF773;ZNF774;ZNF776;ZNF777;ZNF780A;ZNF780B;ZNF782;ZNF785;ZNF799;ZNF805;ZNF808;ZNF81;ZNF813;ZNF816;ZNF823;ZNF829;ZNF836;ZNF841;ZNF844;ZNF845;ZNF846;ZNF85;ZNF853;ZNF860;ZNF878;ZNF891;ZNF99;ZSCAN16;ZSCAN2;ZSCAN22;ZSCAN23;ZSCAN29;ZSCAN31;ZSCAN32;ZSCAN4;ZSCAN5A;ZSCAN5B;ZSCAN5C;ZSCAN9;ZXDA;ZXDB;ZXDC	ZBTB7A;ZBTB7B
RARA_HUMAN.H11MO.0.A	19	0.11111111	0.31345280	0.8651436	0.5351807	8051,16200000	4,67E+04	Thyroid hormone receptor-related factors (NR1){2.1.2}	Retinoic acid receptors (NR1B){2.1.2.1}	NR1D1;NR1D2;NR1H2;NR1H3;NR1H4;NR1I2;NR1I3;PPARA;PPARD;PPARG;RARA;RARB;RARG;RORA;RORB;RORC;THRA;THRB;VDR	RARA;RARB;RARG

motif	Nº of probes	% of probes	lower OR	upper OR	OR	p.value	FDR	TF family	TF subfamily	TF.family.member	TF.subfamily.member
HEN1_HUMAN.H11MO.0.C	35	0.20467836	0.31289734	0.6826297	0.4677621	27,27680000	7,25E+02	Tal-related factors{1.2.3}	Tal / HEN-like factors{1.2.3.1}	ATOH1;ATOH7;ATOH8;BHLHA15;BHLHA9;BHLHE22;BHLHE23;FERD3L;FIGLA;HAND1;HAND2;LYL1;MESP1;MESP2;MSC;MSGN1;NEUROD1;NEUROD2;NEUROD4;NEUROD6;NEUROG1;NEUROG2;NEUROG3;NHLH1;NHLH2;OLIG1;OLIG2;OLIG3;PTF1A;SCX;TAL1;TAL2;TCF15;TCF21;TCF23;TWIST1;TWIST2	NHLH1;LYL1;TAL1
TBX5_HUMAN.H11MO.0.D	12	0.07017544	0.31224866	11.090.242	0.6172547	110564,50000000	2,90E+05	TBX2-related factors{6.5.4}	TBX5{6.5.4.0.4}	TBX2;TBX3;TBX4;TBX5	TBX5
ZSC31_HUMAN.H11MO.0.C	26	0.15204678	0.31159445	0.7524020	0.4930694	493,64890000	5,58E+03	More than 3 adjacent zinc finger factors{2.3.3}	ZNF24-like factors{2.3.3.10}	BCL6;BCL6B;CTCF;CTCFL;FEZF1;FEZF2;GFI1;GFI1B;GLI1;GLI2;GLI3;GLI4;GLIS1;GLIS2;GLIS3;HKR1;MTF1;MYNN;MZF1;OSR2;OVOL1;OVOL2;PLAG1;PLAGL1;PLAGL2;PRDM1;PRDM14;PRDM6;SCRT1;SCRT2;SNAI1;SNAI2;SNAI3;WT1;YY1;YY2;ZBTB12;ZBTB14;ZBTB18;ZBTB20;ZBTB26;ZBTB42;ZBTB45;ZBTB47;ZBTB48;ZBTB49;ZBTB6;ZBTB7A;ZBTB7B;ZBTB7C;ZFP14;ZFP2;ZFP28;ZFP30;ZFP37;ZFP42;ZFP64;ZFP69;ZFP69B;ZFP82;ZFP91;ZFX;ZIC1;ZIC2;ZIC3;ZIC4;ZIC5;ZIK1;ZIM3;ZKSCAN1;ZKSCAN2;ZKSCAN3;ZKSCAN4;ZNF121;ZNF124;ZNF133;ZNF136;ZNF138;ZNF14;ZNF140;ZNF143;ZNF146;ZNF148;ZNF155;ZNF157;ZNF160;ZNF169;ZNF175;ZNF177;ZNF18;ZNF180;ZNF181;ZNF2;ZNF20;ZNF212;ZNF213;ZNF214;ZNF221;ZNF222;ZNF223;ZNF224;ZNF225;ZNF226;ZNF227;ZNF229;ZNF230;ZNF232;ZNF233;ZNF234;ZNF235;ZNF24;ZNF25;ZNF250;ZNF257;ZNF26;ZNF260;ZNF263;ZNF264;ZNF268;ZNF274;ZNF276;ZNF28;ZNF280A;ZNF280B;ZNF280C;ZNF280D;ZNF281;ZNF282;ZNF283;ZNF284;ZNF285;ZNF286A;ZNF286B;ZNF3;ZNF30;ZNF300;ZNF302;ZNF317;ZNF32;ZNF320;ZNF322;ZNF324;ZNF324B;ZNF329;ZNF331;ZNF333;ZNF33A;ZNF33B;ZNF343;ZNF345;ZNF347;ZNF350;ZNF354A;ZNF354B;ZNF362;ZNF366;ZNF383;ZNF384;ZNF394;ZNF397;ZNF398;ZNF404;ZNF41;ZNF410;ZNF419;ZNF420;ZNF431;ZNF432;ZNF436;ZNF439;ZNF44;ZNF440;ZNF442;ZNF443;ZNF446;ZNF449;ZNF45;ZNF460;ZNF468;ZNF479;ZNF484;ZNF490;ZNF500;ZNF502;ZNF524;ZNF525;ZNF528;ZNF543;ZNF544;ZNF546;ZNF547;ZNF548;ZNF549;ZNF554;ZNF555;ZNF557;ZNF558;ZNF559;ZNF561;ZNF562;ZNF563;ZNF564;ZNF566;ZNF567;ZNF568;ZNF57;ZNF570;ZNF571;ZNF572;ZNF577;ZNF581;ZNF582;ZNF583;ZNF585A;ZNF586;ZNF589;ZNF595;ZNF599;ZNF600;ZNF605;ZNF607;ZNF611;ZNF613;ZNF614;ZNF615;ZNF616;ZNF619;ZNF620;ZNF621;ZNF625;ZNF627;ZNF649;ZNF652;ZNF653;ZNF665;ZNF667;ZNF669;ZNF670;ZNF672;ZNF679;ZNF680;ZNF683;ZNF689;ZNF692;ZNF701;ZNF705D;ZNF705E;ZNF705G;ZNF708;ZNF709;ZNF71;ZNF710;ZNF713;ZNF721;ZNF727;ZNF729;ZNF736;ZNF75A;ZNF75D;ZNF76;ZNF763;ZNF764;ZNF765;ZNF768;ZNF77;ZNF771;ZNF773;ZNF774;ZNF776;ZNF777;ZNF780A;ZNF780B;ZNF782;ZNF785;ZNF799;ZNF805;ZNF808;ZNF81;ZNF813;ZNF816;ZNF823;ZNF829;ZNF836;ZNF841;ZNF844;ZNF845;ZNF846;ZNF85;ZNF853;ZNF860;ZNF878;ZNF891;ZNF99;ZSCAN16;ZSCAN2;ZSCAN22;ZSCAN23;ZSCAN29;ZSCAN31;ZSCAN32;ZSCAN4;ZSCAN5A;ZSCAN5B;ZSCAN5C;ZSCAN9;ZXDA;ZXDB;ZXDC	ZKSCAN1;ZNF232;ZSCAN31

motif	Nº of probes	% of probes	lower OR	upper OR	OR	p.value	FDR	TF family	TF subfamily	TF.family.member	TF.subfamily.member
PAX8_HUMAN.H11MO.0.D	10	0.05847953	0.30787708	12.356.228	0.6545371	221023,60000000	4,48E+05	Paired domain only{3.2.2}	PAX-2-like factors (partial homeobox){3.2.2.2}	PAX1;PAX2;PAX5;PAX8;PAX9	PAX2;PAX5;PAX8
ZBT48_HUMAN.H11MO.0.C	14	0.08187135	0.30557901	0.9890075	0.5720013	43149,15000000	1,60E+05	More than 3 adjacent zinc finger factors{2.3.3}	unclassified{2.3.3.0}	BCL6B;BCL6;CTCFL;CTCF;FEZF1;GFI1B;GFI1;GLI1;GLI2;GLI3;GLIS1;GLIS2;GLIS3;MTF1;MYNN;MZF1;OSR2;OVOL1;OVOL2;ZNF146;PLAG1;PLAGL1;PRDM14;PRDM1;PRDM6;SCRT1;SCRT2;SNAI1;SNAI2;YY1;YY2;WT1;ZNF324;ZNF354A;ZBTB14;ZBTB18;ZBTB48;ZBTB49;ZBTB7A;ZBTB7B;ZBTB6;ZFP64;ZFP28;ZFP42;ZFP82;ZFX;ZIC1;ZIC2;ZIC3;ZIC4;ZIM3;ZKSCAN1;ZKSCAN3;ZNF121;ZNF136;ZNF140;ZNF143;ZNF148;ZNF214;ZNF232;ZNF250;ZNF257;ZNF260;ZNF263;ZNF264;ZNF274;ZNF281;ZNF282;ZNF317;ZNF320;ZNF322;ZNF329;ZNF331;ZNF333;ZNF350;ZNF384;ZNF394;ZNF410;ZNF436;ZNF449;ZNF490;ZNF502;ZNF524;ZNF528;ZNF547;ZNF549;ZNF554;ZNF563;ZNF582;ZNF586;ZNF589;ZNF652;ZNF667;ZNF680;ZNF708;ZNF713;ZNF768;ZNF816;ZNF18;ZNF41;ZNF76;ZNF85;ZSCAN16;ZSCAN22;ZSCAN31;ZSCAN4	MYNN;MZF1;OSR2;PRDM14;PRDM6;WT1;ZBTB14;ZBTB48;ZBTB49;ZFP64;ZFP28;ZIM3;ZNF121;ZNF250;ZNF257;ZNF263;ZNF274;ZNF317;ZNF320;ZNF329;ZNF331;ZNF394;ZNF449;ZNF502;ZNF528;ZNF547;ZNF549;ZNF554;ZNF586;ZNF589;ZNF667;ZNF680;ZNF708;ZNF713;ZNF768;ZNF18;ZNF85;ZSCAN16;ZSCAN22
SMAD3_HUMAN.H11MO.0.B	32	0.18713450	0.30523025	0.6849409	0.4635519	30,43397000	7,82E+02	SMAD factors{7.1.1}	Regulatory Smads (R-Smad){7.1.1.1}	SMAD1;SMAD2;SMAD3;SMAD4;SMAD5;SMAD9	SMAD1;SMAD2;SMAD3
ZFX_HUMAN.H11MO.0.A	31	0.18128655	0.30514599	0.6919793	0.4661615	51,95831000	1,20E+03	More than 3 adjacent zinc finger factors{2.3.3}	ZFX/ZFY factors{2.3.3.65}	BCL6;BCL6B;CTCF;CTCFL;FEZF1;FEZF2;GFI1;GFI1B;GLI1;GLI2;GLI3;GLI4;GLIS1;GLIS2;GLIS3;HKR1;MTF1;MYNN;MZF1;OSR2;OVOL1;OVOL2;PLAG1;PLAGL1;PLAGL2;PRDM1;PRDM14;PRDM6;SCRT1;SCRT2;SNAI1;SNAI2;SNAI3;WT1;YY1;YY2;ZBTB12;ZBTB14;ZBTB18;ZBTB20;ZBTB26;ZBTB42;ZBTB45;ZBTB47;ZBTB48;ZBTB49;ZBTB6;ZBTB7A;ZBTB7B;ZBTB7C;ZFP14;ZFP2;ZFP28;ZFP30;ZFP37;ZFP42;ZFP64;ZFP69;ZFP69B;ZFP82;ZFP91;ZFX;ZIC1;ZIC2;ZIC3;ZIC4;ZIC5;ZIK1;ZIM3;ZKSCAN1;ZKSCAN2;ZKSCAN3;ZKSCAN4;ZNF121;ZNF124;ZNF133;ZNF136;ZNF138;ZNF14;ZNF140;ZNF143;ZNF146;ZNF148;ZNF155;ZNF157;ZNF160;ZNF169;ZNF175;ZNF177;ZNF18;ZNF180;ZNF181;ZNF2;ZNF20;ZNF212;ZNF213;ZNF214;ZNF221;ZNF222;ZNF223;ZNF224;ZNF225;ZNF226;ZNF227;ZNF229;ZNF230;ZNF232;ZNF233;ZNF234;ZNF235;ZNF24;ZNF25;ZNF250;ZNF257;ZNF26;ZNF260;ZNF263;ZNF264;ZNF268;ZNF274;ZNF276;ZNF28;ZNF280A;ZNF280B;ZNF280C;ZNF280D;ZNF281;ZNF282;ZNF283;ZNF284;ZNF285;ZNF286A;ZNF286B;ZNF3;ZNF30;ZNF300;ZNF302;ZNF317;ZNF32;ZNF320;ZNF322;ZNF324;ZNF324B;ZNF329;ZNF331;ZNF333;ZNF33A;ZNF33B;ZNF343;ZNF345;ZNF347;ZNF350;ZNF354A;ZNF354B;ZNF362;ZNF366;ZNF383;ZNF384;ZNF394;ZNF397;ZNF398;ZNF404;ZNF41;ZNF410;ZNF419;ZNF420;ZNF431;ZNF432;ZNF436;ZNF439;ZNF44;ZNF440;ZNF442;ZNF443;ZNF446;ZNF449;ZNF45;ZNF460;ZNF468;ZNF479;ZNF484;ZNF490;ZNF500;ZNF502;ZNF524;ZNF525;ZNF528;ZNF543;ZNF544;ZNF546;ZNF547;ZNF548;ZNF549;ZNF554;ZNF555;ZNF557;ZNF558;ZNF559;ZNF561;ZNF562;ZNF563;ZNF564;ZNF566;ZNF567;ZNF568;ZNF57;ZNF570;ZNF571;ZNF572;ZNF577;ZNF581;ZNF582;ZNF583;ZNF585A;ZNF586;ZNF589;ZNF595;ZNF599;ZNF600;ZNF605;ZNF607;ZNF611;ZNF613;ZNF614;ZNF615;ZNF616;ZNF619;ZNF620;ZNF621;ZNF625;ZNF627;ZNF649;ZNF652;ZNF653;ZNF665;ZNF667;ZNF669;ZNF670;ZNF672;Z	ZFX

motif	Nº of probes	% of probes	lower OR	upper OR	OR	p.value	FDR	TF family	TF subfamily	TF.family.member	TF.subfamily.member
										NF679;ZNF680;ZNF683;ZNF689;ZNF692;ZNF701;ZNF705D;ZNF705E;ZNF705G;ZNF708;ZNF709;ZNF71;ZNF710;ZNF713;ZNF721;ZNF727;ZNF729;ZNF736;ZNF75A;ZNF75D;ZNF76;ZNF763;ZNF764;ZNF765;ZNF768;ZNF77;ZNF771;ZNF773;ZNF774;ZNF776;ZNF777;ZNF780A;ZNF780B;ZNF782;ZNF785;ZNF799;ZNF805;ZNF808;ZNF81;ZNF813;ZNF816;ZNF823;ZNF829;ZNF836;ZNF841;ZNF844;ZNF845;ZNF846;ZNF85;ZNF853;ZNF860;ZNF878;ZNF891;ZNF99;ZSCAN16;ZSCAN2;ZSCAN22;ZSCAN23;ZSCAN29;ZSCAN31;ZSCAN32;ZSCAN4;ZSCAN5A;ZSCAN5B;ZSCAN5C;ZSCAN9;ZXDA;ZXDB;ZXDC	
HMGA2_HUMAN.H11MO.0.D	6	0.03508772	0.30429693	18.724.852	0.8416104	847828,30000000	9,46E+05	HMGA factors{8.2.1}	HMGA2 (HMGI-C){8.2.1.0.2}	HMGA1;HMGA2	HMGA2
AP2D_HUMAN.H11MO.0.D	56	0.32748538	0.30207863	0.5880580	0.4236226	0,05459966	2,81E+00	AP-2{1.3.1}	AP-2delta{1.3.1.0.4}	TFAP2A;TFAP2B;TFAP2C;TFAP2D	TFAP2D
HXB7_HUMAN.H11MO.0.C	6	0.03508772	0.30182353	18.572.244	0.8347892	847963,50000000	9,46E+05	HOX-related factors{3.1.1}	HOX6-7{3.1.1.6}	CDX1;CDX2;CDX4;EVX1;EVX2;GBX1;GBX2;GSX1;GSX2;HDX;HMBOX1;HNF1A;HNF1B;HOXA1;HOXA10;HOXA11;HOXA13;HOXA2;HOXA3;HOXA4;HOXA5;HOXA6;HOXA7;HOXA9;HOXB1;HOXB13;HOXB2;HOXB3;HOXB4;HOXB5;HOXB6;HOXB7;HOXB8;HOXB9;HOXC10;HOXC11;HOXC12;HOXC13;HOXC4;HOXC5;HOXC6;HOXC8;HOXC9;HOXD1;HOXD10;HOXD11;HOXD12;HOXD13;HOXD3;HOXD4;HOXD8;HOXD9;MEOX1;MEOX2;MNX1;PDX1;POU1F1;POU2F1;POU2F2;POU2F3;POU3F1;POU3F2;POU3F3;POU3F4;POU4F1;POU4F2;POU4F3;POU5F1;POU5F2;POU6F1;POU6F2	HOXA7;HOXB6;HOXB7;HOXC6
SP1_HUMAN.H11MO.0.A	75	0.43859649	0.30123590	0.5648752	0.4131741	0,01045475	7,18E-01	Three-zinc finger Krüppel-related factors{2.3.1}	Sp1-like factors{2.3.1.1}	EGR1;EGR2;EGR3;EGR4;KLF1;KLF10;KLF11;KLF12;KLF13;KLF14;KLF15;KLF16;KLF17;KLF2;KLF3;KLF4;KLF5;KLF6;KLF7;KLF8;KLF9;SP1;SP2;SP3;SP4;SP5;SP6;SP7;SP8;SP9	SP1;SP2;SP3;SP4
RFX1_HUMAN.H11MO.0.B	35	0.20467836	0.30119075	0.6570256	0.4502288	9,96136800	2,86E+02	RFX-related factors{3.3.3}	RFX1 (EF-C){3.3.3.0.1}	RFX1;RFX2;RFX3;RFX4;RFX5;RFX6;RFX7;RFX8	RFX1

motif	Nº of probes	% of probes	lower OR	upper OR	OR	p.value	FDR	TF family	TF subfamily	TF.family.member	TF.subfamily.member
PLAL1_HUMAN.H11MO.0.D	25	0.14619883	0.30049509	0.7366677	0.4795796	333,85950000	4,36E+03	More than 3 adjacent zinc finger factors{2.3.3}	PLAG factors{2.3.3.25}	BCL6;BCL6B;CTCF;CTCFL;FEZF1;FEZF2;GFI1;GFI1B;GLI1;GLI2;GLI3;GLI4;GLIS1;GLIS2;GLIS3;HKR1;MTF1;MYNN;MZF1;OSR2;OVOL1;OVOL2;PLAG1;PLAGL1;PLAGL2;PRDM1;PRDM14;PRDM6;SCRT1;SCRT2;SNAI1;SNAI2;SNAI3;WT1;YY1;YY2;ZBTB12;ZBTB14;ZBTB18;ZBTB20;ZBTB26;ZBTB42;ZBTB45;ZBTB47;ZBTB48;ZBTB49;ZBTB6;ZBTB7A;ZBTB7B;ZBTB7C;ZFP14;ZFP2;ZFP28;ZFP30;ZFP37;ZFP42;ZFP64;ZFP69;ZFP69B;ZFP82;ZFP91;ZFX;ZIC1;ZIC2;ZIC3;ZIC4;ZIC5;ZIK1;ZIM3;ZKSCAN1;ZKSCAN2;ZKSCAN3;ZKSCAN4;ZNF121;ZNF124;ZNF133;ZNF136;ZNF138;ZNF14;ZNF140;ZNF143;ZNF146;ZNF148;ZNF155;ZNF157;ZNF160;ZNF169;ZNF175;ZNF177;ZNF18;ZNF180;ZNF181;ZNF2;ZNF20;ZNF212;ZNF213;ZNF214;ZNF221;ZNF222;ZNF223;ZNF224;ZNF225;ZNF226;ZNF227;ZNF229;ZNF230;ZNF232;ZNF233;ZNF234;ZNF235;ZNF24;ZNF25;ZNF250;ZNF257;ZNF26;ZNF260;ZNF263;ZNF264;ZNF268;ZNF274;ZNF276;ZNF28;ZNF280A;ZNF280B;ZNF280C;ZNF280D;ZNF281;ZNF282;ZNF283;ZNF284;ZNF285;ZNF286A;ZNF286B;ZNF3;ZNF30;ZNF300;ZNF302;ZNF317;ZNF32;ZNF320;ZNF322;ZNF324;ZNF324B;ZNF329;ZNF331;ZNF333;ZNF33A;ZNF33B;ZNF343;ZNF345;ZNF347;ZNF350;ZNF354A;ZNF354B;ZNF362;ZNF366;ZNF383;ZNF384;ZNF394;ZNF397;ZNF398;ZNF404;ZNF41;ZNF410;ZNF419;ZNF420;ZNF431;ZNF432;ZNF436;ZNF439;ZNF44;ZNF440;ZNF442;ZNF443;ZNF446;ZNF449;ZNF45;ZNF460;ZNF468;ZNF479;ZNF484;ZNF490;ZNF500;ZNF502;ZNF524;ZNF525;ZNF528;ZNF543;ZNF544;ZNF546;ZNF547;ZNF548;ZNF549;ZNF554;ZNF555;ZNF557;ZNF558;ZNF559;ZNF561;ZNF562;ZNF563;ZNF564;ZNF566;ZNF567;ZNF568;ZNF57;ZNF570;ZNF571;ZNF572;ZNF577;ZNF581;ZNF582;ZNF583;ZNF585A;ZNF586;ZNF589;ZNF595;ZNF599;ZNF600;ZNF605;ZNF607;ZNF611;ZNF613;ZNF614;ZNF615;ZNF616;ZNF619;ZNF620;ZNF621;ZNF625;ZNF627;ZNF649;ZNF652;ZNF653;ZNF665;ZNF667;ZNF669;ZNF670;ZNF672;ZNF679;ZNF680;ZNF683;ZNF689;ZNF692;ZNF701;ZNF705D;ZNF705E;ZNF705G;ZNF708;ZNF709;ZNF71;ZNF710;ZNF713;ZNF721;ZNF727;ZNF729;ZNF736;ZNF75A;ZNF75D;ZNF76;ZNF763;ZNF764;ZNF765;ZNF768;ZNF77;ZNF771;ZNF773;ZNF774;ZNF776;ZNF777;ZNF780A;ZNF780B;ZNF782;ZNF785;ZNF799;ZNF805;ZNF808;ZNF81;ZNF813;ZNF816;ZNF823;ZNF829;ZNF836;ZNF841;ZNF844;ZNF845;ZNF846;ZNF85;ZNF853;ZNF860;ZNF878;ZNF891;ZNF99;ZSCAN16;ZSCAN2;ZSCAN22;ZSCAN23;ZSCAN29;ZSCAN31;ZSCAN32;ZSCAN4;ZSCAN5A;ZSCAN5B;ZSCAN5C;ZSCAN9;ZXDA;ZXDB;ZXDC	PLAG1;PLAGL1

motif	Nº of probes	% of probes	lower OR	upper OR	OR	p.value	FDR	TF family	TF subfamily	TF.family.member	TF.subfamily.member
ZN264_HUMAN.H11MO.0.C	7	0.04093567	0.29351789	15.641.594	0.7418517	610360,50000000	8,04E+05	More than 3 adjacent zinc finger factors{2.3.3}	ZNF460-like factors{2.3.3.54}	BCL6;BCL6B;CTCF;CTCFL;FEZF1;FEZF2;GFI1;GFI1B;GLI1;GLI2;GLI3;GLI4;GLIS1;GLIS2;GLIS3;HKR1;MTF1;MYNN;MZF1;OSR2;OVOL1;OVOL2;PLAG1;PLAGL1;PLAGL2;PRDM1;PRDM14;PRDM6;SCRT1;SCRT2;SNAI1;SNAI2;SNAI3;WT1;YY1;YY2;ZBTB12;ZBTB14;ZBTB18;ZBTB20;ZBTB26;ZBTB42;ZBTB45;ZBTB47;ZBTB48;ZBTB49;ZBTB6;ZBTB7A;ZBTB7B;ZBTB7C;ZFP14;ZFP2;ZFP28;ZFP30;ZFP37;ZFP42;ZFP64;ZFP69;ZFP69B;ZFP82;ZFP91;ZFX;ZIC1;ZIC2;ZIC3;ZIC4;ZIC5;ZIK1;ZIM3;ZKSCAN1;ZKSCAN2;ZKSCAN3;ZKSCAN4;ZNF121;ZNF124;ZNF133;ZNF136;ZNF138;ZNF14;ZNF140;ZNF143;ZNF146;ZNF148;ZNF155;ZNF157;ZNF160;ZNF169;ZNF175;ZNF177;ZNF18;ZNF180;ZNF181;ZNF2;ZNF20;ZNF212;ZNF213;ZNF214;ZNF221;ZNF222;ZNF223;ZNF224;ZNF225;ZNF226;ZNF227;ZNF229;ZNF230;ZNF232;ZNF233;ZNF234;ZNF235;ZNF24;ZNF25;ZNF250;ZNF257;ZNF26;ZNF260;ZNF263;ZNF264;ZNF268;ZNF274;ZNF276;ZNF28;ZNF280A;ZNF280B;ZNF280C;ZNF280D;ZNF281;ZNF282;ZNF283;ZNF284;ZNF285;ZNF286A;ZNF286B;ZNF3;ZNF30;ZNF300;ZNF302;ZNF317;ZNF32;ZNF320;ZNF322;ZNF324;ZNF324B;ZNF329;ZNF331;ZNF333;ZNF33A;ZNF33B;ZNF343;ZNF345;ZNF347;ZNF350;ZNF354A;ZNF354B;ZNF362;ZNF366;ZNF383;ZNF384;ZNF394;ZNF397;ZNF398;ZNF404;ZNF41;ZNF410;ZNF419;ZNF420;ZNF431;ZNF432;ZNF436;ZNF439;ZNF44;ZNF440;ZNF442;ZNF443;ZNF446;ZNF449;ZNF45;ZNF460;ZNF468;ZNF479;ZNF484;ZNF490;ZNF500;ZNF502;ZNF524;ZNF525;ZNF528;ZNF543;ZNF544;ZNF546;ZNF547;ZNF548;ZNF549;ZNF554;ZNF555;ZNF557;ZNF558;ZNF559;ZNF561;ZNF562;ZNF563;ZNF564;ZNF566;ZNF567;ZNF568;ZNF57;ZNF570;ZNF571;ZNF572;ZNF577;ZNF581;ZNF582;ZNF583;ZNF585A;ZNF586;ZNF589;ZNF595;ZNF599;ZNF600;ZNF605;ZNF607;ZNF611;ZNF613;ZNF614;ZNF615;ZNF616;ZNF619;ZNF620;ZNF621;ZNF625;ZNF627;ZNF649;ZNF652;ZNF653;ZNF665;ZNF667;ZNF669;ZNF670;ZNF672;ZNF679;ZNF680;ZNF683;ZNF689;ZNF692;ZNF701;ZNF705D;ZNF705E;ZNF705G;ZNF708;ZNF709;ZNF71;ZNF710;ZNF713;ZNF721;ZNF727;ZNF729;ZNF736;ZNF75A;ZNF75D;ZNF76;ZNF763;ZNF764;ZNF765;ZNF768;ZNF77;ZNF771;ZNF773;ZNF774;ZNF776;ZNF777;ZNF780A;ZNF780B;ZNF782;ZNF785;ZNF799;ZNF805;ZNF808;ZNF81;ZNF813;ZNF816;ZNF823;ZNF829;ZNF836;ZNF841;ZNF844;ZNF845;ZNF846;ZNF85;ZNF853;ZNF860;ZNF878;ZNF891;ZNF99;ZSCAN16;ZSCAN2;ZSCAN22;ZSCAN23;ZSCAN29;ZSCAN31;ZSCAN32;ZSCAN4;ZSCAN5A;ZSCAN5B;ZSCAN5C;ZSCAN9;ZXDA;ZXDB;ZXDC	ZNF264
ZN770_HUMAN.H11MO.0.C	59	0.34502924	0.29283490	0.5650592	0.4085851	0,01116939	7,18E-01	Factors with multiple dispersed zinc fingers{2.3.4}	unclassified{2.3.4.0}	BCL11A;E4F1;MECOM;HIC1;HIC2;HINFP;IKZF1;INSM1;MAZ;PATZ1;PRDM4;REST;RREB1;SALL4;VEZF1;ZBTB17;ZBTB4;HIVEP1;HIVEP2;ZNF134;ZNF219;ZNF335;ZNF341;ZNF382;ZNF418;ZNF423;ZNF467;ZNF770;ZNF784;ZNF8	E4F1;PRDM4;REST;RREB1;ZBTB17;ZBTB4;ZNF335;ZNF341;ZNF467;ZNF770;ZNF784;ZNF8

motif	Nº of probes	% of probes	lower OR	upper OR	OR	p.value	FDR	TF family	TF subfamily	TF.family.member	TF.subfamily.member
ZBT7B_HUMAN.H11MO.0.D	21	0.12280702	0.29145730	0.7683888	0.4847655	911,05980000	8,17E+03	More than 3 adjacent zinc finger factors{2.3.3}	ZBTB7 factors{2.3.3.8}	BCL6;BCL6B;CTCF;CTCFL;FEZF1;FEZF2;GFI1;GFI1B;GLI1;GLI2;GLI3;GLI4;GLIS1;GLIS2;GLIS3;HKR1;MTF1;MYNN;MZF1;OSR2;OVOL1;OVOL2;PLAG1;PLAGL1;PLAGL2;PRDM1;PRDM14;PRDM6;SCRT1;SCRT2;SNAI1;SNAI2;SNAI3;WT1;YY1;YY2;ZBTB12;ZBTB14;ZBTB18;ZBTB20;ZBTB26;ZBTB42;ZBTB45;ZBTB47;ZBTB48;ZBTB49;ZBTB6;ZBTB7A;ZBTB7B;ZBTB7C;ZFP14;ZFP2;ZFP28;ZFP30;ZFP37;ZFP42;ZFP64;ZFP69;ZFP69B;ZFP82;ZFP91;ZFX;ZIC1;ZIC2;ZIC3;ZIC4;ZIC5;ZIK1;ZIM3;ZKSCAN1;ZKSCAN2;ZKSCAN3;ZKSCAN4;ZNF121;ZNF124;ZNF133;ZNF136;ZNF138;ZNF14;ZNF140;ZNF143;ZNF146;ZNF148;ZNF155;ZNF157;ZNF160;ZNF169;ZNF175;ZNF177;ZNF18;ZNF180;ZNF181;ZNF2;ZNF20;ZNF212;ZNF213;ZNF214;ZNF221;ZNF222;ZNF223;ZNF224;ZNF225;ZNF226;ZNF227;ZNF229;ZNF230;ZNF232;ZNF233;ZNF234;ZNF235;ZNF24;ZNF25;ZNF250;ZNF257;ZNF26;ZNF260;ZNF263;ZNF264;ZNF268;ZNF274;ZNF276;ZNF28;ZNF280A;ZNF280B;ZNF280C;ZNF280D;ZNF281;ZNF282;ZNF283;ZNF284;ZNF285;ZNF286A;ZNF286B;ZNF3;ZNF30;ZNF300;ZNF302;ZNF317;ZNF32;ZNF320;ZNF322;ZNF324;ZNF324B;ZNF329;ZNF331;ZNF333;ZNF33A;ZNF33B;ZNF343;ZNF345;ZNF347;ZNF350;ZNF354A;ZNF354B;ZNF362;ZNF366;ZNF383;ZNF384;ZNF394;ZNF397;ZNF398;ZNF404;ZNF41;ZNF410;ZNF419;ZNF420;ZNF431;ZNF432;ZNF436;ZNF439;ZNF44;ZNF440;ZNF442;ZNF443;ZNF446;ZNF449;ZNF45;ZNF460;ZNF468;ZNF479;ZNF484;ZNF490;ZNF500;ZNF502;ZNF524;ZNF525;ZNF528;ZNF543;ZNF544;ZNF546;ZNF547;ZNF548;ZNF549;ZNF554;ZNF555;ZNF557;ZNF558;ZNF559;ZNF561;ZNF562;ZNF563;ZNF564;ZNF566;ZNF567;ZNF568;ZNF57;ZNF570;ZNF571;ZNF572;ZNF577;ZNF581;ZNF582;ZNF583;ZNF585A;ZNF586;ZNF589;ZNF595;ZNF599;ZNF600;ZNF605;ZNF607;ZNF611;ZNF613;ZNF614;ZNF615;ZNF616;ZNF619;ZNF620;ZNF621;ZNF625;ZNF627;ZNF649;ZNF652;ZNF653;ZNF665;ZNF667;ZNF669;ZNF670;ZNF672;ZNF679;ZNF680;ZNF683;ZNF689;ZNF692;ZNF701;ZNF705D;ZNF705E;ZNF705G;ZNF708;ZNF709;ZNF71;ZNF710;ZNF713;ZNF721;ZNF727;ZNF729;ZNF736;ZNF75A;ZNF75D;ZNF76;ZNF763;ZNF764;ZNF765;ZNF768;ZNF77;ZNF771;ZNF773;ZNF774;ZNF776;ZNF777;ZNF780A;ZNF780B;ZNF782;ZNF785;ZNF799;ZNF805;ZNF808;ZNF81;ZNF813;ZNF816;ZNF823;ZNF829;ZNF836;ZNF841;ZNF844;ZNF845;ZNF846;ZNF85;ZNF853;ZNF860;ZNF878;ZNF891;ZNF99;ZSCAN16;ZSCAN2;ZSCAN22;ZSCAN23;ZSCAN29;ZSCAN31;ZSCAN32;ZSCAN4;ZSCAN5A;ZSCAN5B;ZSCAN5C;ZSCAN9;ZXDA;ZXDB;ZXDC	ZBTB7A;ZBTB7B
SP2_HUMAN.H11MO.0.A	82	0.47953216	0.28886973	0.5393161	0.3949087	0,00248217	1,91E-01	Three-zinc finger Krüppel-related factors{2.3.1}	Sp1-like factors{2.3.1.1}	EGR1;EGR2;EGR3;EGR4;KLF1;KLF10;KLF11;KLF12;KLF13;KLF14;KLF15;KLF16;KLF17;KLF2;KLF3;KLF4;KLF5;KLF6;KLF7;KLF8;KLF9;SP1;SP2;SP3;SP4;SP5;SP6;SP7;SP8;SP9	SP1;SP2;SP3;SP4
BHE41_HUMAN.H11MO.0.D	24	0.14035088	0.28607164	0.7128339	0.4607978	165,09740000	2,83E+03	Hairy-related factors{1.2.4}	Hairy-like factors{1.2.4.1}	BHLHE40;BHLHE41;HELT;HES1;HES2;HES3;HES4;HES5;HES6;HES7;HEY1;HEY2;HEYL	BHLHE40;BHLHE41;HES1;HES5;HES7;HEY1;HEY2
NFAT5_HUMAN.H11MO.0.D	16	0.09356725	0.28561931	0.8587966	0.5122281	7526,53200000	4,46E+04	NFAT-related factors{6.1.3}	NFAT5 (TonEBP){6.1.3.0.5}	NFAT5;NFATC1;NFATC2;NFATC3;NFATC4	NFAT5

motif	Nº of probes	% of probes	lower OR	upper OR	OR	p.value	FDR	TF family	TF subfamily	TF.family.member	TF.subfamily.member
EMX2_HUMAN.H11MO.0.D	6	0.03508772	0.28476586	17.521.513	0.7875424	709877,10000000	8,75E+05	NK-related factors{3.1.2}	EMX{3.1.2.6}	BARHL1;BARHL2;BARX1;BARX2;BSX;DBX1;DBX2;DLX1;DLX2;DLX3;DLX4;DLX5;DLX6;EMX1;EMX2;EN1;EN2;HHEX;HLX;HMX1;HMX2;HMX3;LBX1;LBX2;MSX1;MSX2;NANOG;NKX1-1;NKX1-2;NKX2-1;NKX2-2;NKX2-3;NKX2-4;NKX2-5;NKX2-6;NKX2-8;NKX3-1;NKX3-2;NKX6-1;NKX6-2;NKX6-3;NOTO;TLX1;TLX2;TLX3;VAX1;VAX2;VENTX	EMX1;EMX2
RFX5_HUMAN.H11MO.1.A	11	0.06432749	0.28152047	10.580.515	0.5754757	81532,54000000	2,37E+05	RFX-related factors{3.3.3}	RFX5{3.3.3.0.5}	RFX1;RFX2;RFX3;RFX4;RFX5;RFX6;RFX7;RFX8	RFX5
GLIS3_HUMAN.H11MO.0.D	21	0.12280702	0.27970367	0.7374051	0.4652294	510,82500000	5,63E+03	More than 3 adjacent zinc finger factors{2.3.3}	GLI-like factors{2.3.3.1}	BCL6;BCL6B;CTCF;CTCFL;FEZF1;FEZF2;GFI1;GFI1B;GLI1;GLI2;GLI3;GLI4;GLIS1;GLIS2;GLIS3;HKR1;MTF1;MYNN;MZF1;OSR2;OVOL1;OVOL2;PLAG1;PLAGL1;PLAGL2;PRDM1;PRDM14;PRDM6;SCRT1;SCRT2;SNAI1;SNAI2;SNAI3;WT1;YY1;YY2;ZBTB12;ZBTB14;ZBTB18;ZBTB20;ZBTB26;ZBTB42;ZBTB45;ZBTB47;ZBTB48;ZBTB49;ZBTB6;ZBTB7A;ZBTB7B;ZBTB7C;ZFP14;ZFP2;ZFP28;ZFP30;ZFP37;ZFP42;ZFP64;ZFP69;ZFP69B;ZFP82;ZFP91;ZFX;ZIC1;ZIC2;ZIC3;ZIC4;ZIC5;ZIK1;ZIM3;ZKSCAN1;ZKSCAN2;ZKSCAN3;ZKSCAN4;ZNF121;ZNF124;ZNF133;ZNF136;ZNF138;ZNF14;ZNF140;ZNF143;ZNF146;ZNF148;ZNF155;ZNF157;ZNF160;ZNF169;ZNF175;ZNF177;ZNF18;ZNF180;ZNF181;ZNF2;ZNF20;ZNF212;ZNF213;ZNF214;ZNF221;ZNF222;ZNF223;ZNF224;ZNF225;ZNF226;ZNF227;ZNF229;ZNF230;ZNF232;ZNF233;ZNF234;ZNF235;ZNF24;ZNF25;ZNF250;ZNF257;ZNF26;ZNF260;ZNF263;ZNF264;ZNF268;ZNF274;ZNF276;ZNF28;ZNF280A;ZNF280B;ZNF280C;ZNF280D;ZNF281;ZNF282;ZNF283;ZNF284;ZNF285;ZNF286A;ZNF286B;ZNF3;ZNF30;ZNF300;ZNF302;ZNF317;ZNF32;ZNF320;ZNF322;ZNF324;ZNF324B;ZNF329;ZNF331;ZNF333;ZNF33A;ZNF33B;ZNF343;ZNF345;ZNF347;ZNF350;ZNF354A;ZNF354B;ZNF362;ZNF366;ZNF383;ZNF384;ZNF394;ZNF397;ZNF398;ZNF404;ZNF41;ZNF410;ZNF419;ZNF420;ZNF431;ZNF432;ZNF436;ZNF439;ZNF44;ZNF440;ZNF442;ZNF443;ZNF446;ZNF449;ZNF45;ZNF460;ZNF468;ZNF479;ZNF484;ZNF490;ZNF500;ZNF502;ZNF524;ZNF525;ZNF528;ZNF543;ZNF544;ZNF546;ZNF547;ZNF548;ZNF549;ZNF554;ZNF555;ZNF557;ZNF558;ZNF559;ZNF561;ZNF562;ZNF563;ZNF564;ZNF566;ZNF567;ZNF568;ZNF57;ZNF570;ZNF571;ZNF572;ZNF577;ZNF581;ZNF582;ZNF583;ZNF585A;ZNF586;ZNF589;ZNF595;ZNF599;ZNF600;ZNF605;ZNF607;ZNF611;ZNF613;ZNF614;ZNF615;ZNF616;ZNF619;ZNF620;ZNF621;ZNF625;ZNF627;ZNF649;ZNF652;ZNF653;ZNF665;ZNF667;ZNF669;ZNF670;ZNF672;ZNF679;ZNF680;ZNF683;ZNF689;ZNF692;ZNF701;ZNF705D;ZNF705E;ZNF705G;ZNF708;ZNF709;ZNF71;ZNF710;ZNF713;ZNF721;ZNF727;ZNF729;ZNF736;ZNF75A;ZNF75D;ZNF76;ZNF763;ZNF764;ZNF765;ZNF768;ZNF77;ZNF771;ZNF773;ZNF774;ZNF776;ZNF777;ZNF780A;ZNF780B;ZNF782;ZNF785;ZNF799;ZNF805;ZNF808;ZNF81;ZNF813;ZNF816;ZNF823;ZNF829;ZNF836;ZNF841;ZNF844;ZNF845;ZNF846;ZNF85;ZNF853;ZNF860;ZNF878;ZNF891;ZNF99;ZSCAN16;ZSCAN2;ZSCAN22;ZSCAN23;ZSCAN29;ZSCAN31;ZSCAN32;ZSCAN4;ZSCAN5A;ZSCAN5B;ZSCAN5C;ZSCAN9;ZXDA;ZXDB;ZXDC	GLI1;GLI2;GLI3;GLIS1;GLIS2;GLIS3;ZIC1;ZIC2;ZIC3;ZIC4

motif	Nº of probes	% of probes	lower OR	upper OR	OR	p.value	FDR	TF family	TF subfamily	TF.family.member	TF.subfamily.member
ISL1_HUMAN.H11MO.0.A	6	0.03508772	0.27562763	16.958.695	0.7622531	711521,90000000	8,75E+05	HD-LIM factors{3.1.5}	ISL{3.1.5.1}	ISL1;ISL2;LHX1;LHX2;LHX3;LHX4;LHX5;LHX6;LHX8;LHX9;LMX1A;LMX1B	ISL1;ISL2
ZN524_HUMAN.H11MO.0.D	18	0.10526316	0.27351667	0.7746949	0.4739564	1457,09900000	1,13E+04	More than 3 adjacent zinc finger factors{2.3.3}	ZNF524-like factors{2.3.3.15}	BCL6;BCL6B;CTCF;CTCFL;FEZF1;FEZF2;GFI1;GFI1B;GLI1;GLI2;GLI3;GLI4;GLIS1;GLIS2;GLIS3;HKR1;MTF1;MYNN;MZF1;OSR2;OVOL1;OVOL2;PLAG1;PLAGL1;PLAGL2;PRDM1;PRDM14;PRDM6;SCRT1;SCRT2;SNAI1;SNAI2;SNAI3;WT1;YY1;YY2;ZBTB12;ZBTB14;ZBTB18;ZBTB20;ZBTB26;ZBTB42;ZBTB45;ZBTB47;ZBTB48;ZBTB49;ZBTB6;ZBTB7A;ZBTB7B;ZBTB7C;ZFP14;ZFP2;ZFP28;ZFP30;ZFP37;ZFP42;ZFP64;ZFP69;ZFP69B;ZFP82;ZFP91;ZFX;ZIC1;ZIC2;ZIC3;ZIC4;ZIC5;ZIK1;ZIM3;ZKSCAN1;ZKSCAN2;ZKSCAN3;ZKSCAN4;ZNF121;ZNF124;ZNF133;ZNF136;ZNF138;ZNF14;ZNF140;ZNF143;ZNF146;ZNF148;ZNF155;ZNF157;ZNF160;ZNF169;ZNF175;ZNF177;ZNF18;ZNF180;ZNF181;ZNF2;ZNF20;ZNF212;ZNF213;ZNF214;ZNF221;ZNF222;ZNF223;ZNF224;ZNF225;ZNF226;ZNF227;ZNF229;ZNF230;ZNF232;ZNF233;ZNF234;ZNF235;ZNF24;ZNF25;ZNF250;ZNF257;ZNF26;ZNF260;ZNF263;ZNF264;ZNF268;ZNF274;ZNF276;ZNF28;ZNF280A;ZNF280B;ZNF280C;ZNF280D;ZNF281;ZNF282;ZNF283;ZNF284;ZNF285;ZNF286A;ZNF286B;ZNF3;ZNF30;ZNF300;ZNF302;ZNF317;ZNF32;ZNF320;ZNF322;ZNF324;ZNF324B;ZNF329;ZNF331;ZNF333;ZNF33A;ZNF33B;ZNF343;ZNF345;ZNF347;ZNF350;ZNF354A;ZNF354B;ZNF362;ZNF366;ZNF383;ZNF384;ZNF394;ZNF397;ZNF398;ZNF404;ZNF41;ZNF410;ZNF419;ZNF420;ZNF431;ZNF432;ZNF436;ZNF439;ZNF44;ZNF440;ZNF442;ZNF443;ZNF446;ZNF449;ZNF45;ZNF460;ZNF468;ZNF479;ZNF484;ZNF490;ZNF500;ZNF502;ZNF524;ZNF525;ZNF528;ZNF543;ZNF544;ZNF546;ZNF547;ZNF548;ZNF549;ZNF554;ZNF555;ZNF557;ZNF558;ZNF559;ZNF561;ZNF562;ZNF563;ZNF564;ZNF566;ZNF567;ZNF568;ZNF57;ZNF570;ZNF571;ZNF572;ZNF577;ZNF581;ZNF582;ZNF583;ZNF585A;ZNF586;ZNF589;ZNF595;ZNF599;ZNF600;ZNF605;ZNF607;ZNF611;ZNF613;ZNF614;ZNF615;ZNF616;ZNF619;ZNF620;ZNF621;ZNF625;ZNF627;ZNF649;ZNF652;ZNF653;ZNF665;ZNF667;ZNF669;ZNF670;ZNF672;ZNF679;ZNF680;ZNF683;ZNF689;ZNF692;ZNF701;ZNF705D;ZNF705E;ZNF705G;ZNF708;ZNF709;ZNF71;ZNF710;ZNF713;ZNF721;ZNF727;ZNF729;ZNF736;ZNF75A;ZNF75D;ZNF76;ZNF763;ZNF764;ZNF765;ZNF768;ZNF77;ZNF771;ZNF773;ZNF774;ZNF776;ZNF777;ZNF780A;ZNF780B;ZNF782;ZNF785;ZNF799;ZNF805;ZNF808;ZNF81;ZNF813;ZNF816;ZNF823;ZNF829;ZNF836;ZNF841;ZNF844;ZNF845;ZNF846;ZNF85;ZNF853;ZNF860;ZNF878;ZNF891;ZNF99;ZSCAN16;ZSCAN2;ZSCAN22;ZSCAN23;ZSCAN29;ZSCAN31;ZSCAN32;ZSCAN4;ZSCAN5A;ZSCAN5B;ZSCAN5C;ZSCAN9;ZXDA;ZXDB;ZXDC	ZNF524
PBX3_HUMAN.H11MO.1.A	6	0.03508772	0.26987496	16.604.198	0.7463254	587574,20000000	7,86E+05	TALE-type homeo domain factors{3.1.4}	PBX{3.1.4.4}	IRX2;IRX3;MEIS1;MEIS2;MEIS3;PBX1;PBX2;PBX3;PKNOX1;TGIF2LX;TGIF1;TGIF2	PBX1;PBX2;PBX3
NFIC_HUMAN.H11MO.0.A	19	0.11111111	0.26613226	0.7343673	0.4543026	526,42810000	5,65E+03	Nuclear factor 1{7.1.2}	NF-1C (NF-IC){7.1.2.0.3}	NFIA;NFIB;NFIC	NFIC
NFIB_HUMAN.H11MO.0.D	17	0.09941520	0.26533915	0.7733131	0.4674215	1700,92900000	1,26E+04	Nuclear factor 1{7.1.2}	NF-1B (NF-IB){7.1.2.0.2}	NFIA;NFIB;NFIC	NFIB

motif	Nº of probes	% of probes	lower OR	upper OR	OR	p.value	FDR	TF family	TF subfamily	TF.family.member	TF.subfamily.member
GLIS2_HUMAN.H11MO.0.D	19	0.11111111	0.26371488	0.7277800	0.4502278	393,74470000	4,86E+03	More than 3 adjacent zinc finger factors{2.3.3}	GLI-like factors{2.3.3.1}	BCL6;BCL6B;CTCF;CTCFL;FEZF1;FEZF2;GFI1;GFI1B;GLI1;GLI2;GLI3;GLI4;GLIS1;GLIS2;GLIS3;HKR1;MTF1;MYNN;MZF1;OSR2;OVOL1;OVOL2;PLAG1;PLAGL1;PLAGL2;PRDM1;PRDM14;PRDM6;SCRT1;SCRT2;SNAI1;SNAI2;SNAI3;WT1;YY1;YY2;ZBTB12;ZBTB14;ZBTB18;ZBTB20;ZBTB26;ZBTB42;ZBTB45;ZBTB47;ZBTB48;ZBTB49;ZBTB6;ZBTB7A;ZBTB7B;ZBTB7C;ZFP14;ZFP2;ZFP28;ZFP30;ZFP37;ZFP42;ZFP64;ZFP69;ZFP69B;ZFP82;ZFP91;ZFX;ZIC1;ZIC2;ZIC3;ZIC4;ZIC5;ZIK1;ZIM3;ZKSCAN1;ZKSCAN2;ZKSCAN3;ZKSCAN4;ZNF121;ZNF124;ZNF133;ZNF136;ZNF138;ZNF14;ZNF140;ZNF143;ZNF146;ZNF148;ZNF155;ZNF157;ZNF160;ZNF169;ZNF175;ZNF177;ZNF18;ZNF180;ZNF181;ZNF2;ZNF20;ZNF212;ZNF213;ZNF214;ZNF221;ZNF222;ZNF223;ZNF224;ZNF225;ZNF226;ZNF227;ZNF229;ZNF230;ZNF232;ZNF233;ZNF234;ZNF235;ZNF24;ZNF25;ZNF250;ZNF257;ZNF26;ZNF260;ZNF263;ZNF264;ZNF268;ZNF274;ZNF276;ZNF28;ZNF280A;ZNF280B;ZNF280C;ZNF280D;ZNF281;ZNF282;ZNF283;ZNF284;ZNF285;ZNF286A;ZNF286B;ZNF3;ZNF30;ZNF300;ZNF302;ZNF317;ZNF32;ZNF320;ZNF322;ZNF324;ZNF324B;ZNF329;ZNF331;ZNF333;ZNF33A;ZNF33B;ZNF343;ZNF345;ZNF347;ZNF350;ZNF354A;ZNF354B;ZNF362;ZNF366;ZNF383;ZNF384;ZNF394;ZNF397;ZNF398;ZNF404;ZNF41;ZNF410;ZNF419;ZNF420;ZNF431;ZNF432;ZNF436;ZNF439;ZNF44;ZNF440;ZNF442;ZNF443;ZNF446;ZNF449;ZNF45;ZNF460;ZNF468;ZNF479;ZNF484;ZNF490;ZNF500;ZNF502;ZNF524;ZNF525;ZNF528;ZNF543;ZNF544;ZNF546;ZNF547;ZNF548;ZNF549;ZNF554;ZNF555;ZNF557;ZNF558;ZNF559;ZNF561;ZNF562;ZNF563;ZNF564;ZNF566;ZNF567;ZNF568;ZNF57;ZNF570;ZNF571;ZNF572;ZNF577;ZNF581;ZNF582;ZNF583;ZNF585A;ZNF586;ZNF589;ZNF595;ZNF599;ZNF600;ZNF605;ZNF607;ZNF611;ZNF613;ZNF614;ZNF615;ZNF616;ZNF619;ZNF620;ZNF621;ZNF625;ZNF627;ZNF649;ZNF652;ZNF653;ZNF665;ZNF667;ZNF669;ZNF670;ZNF672;ZNF679;ZNF680;ZNF683;ZNF689;ZNF692;ZNF701;ZNF705D;ZNF705E;ZNF705G;ZNF708;ZNF709;ZNF71;ZNF710;ZNF713;ZNF721;ZNF727;ZNF729;ZNF736;ZNF75A;ZNF75D;ZNF76;ZNF763;ZNF764;ZNF765;ZNF768;ZNF77;ZNF771;ZNF773;ZNF774;ZNF776;ZNF777;ZNF780A;ZNF780B;ZNF782;ZNF785;ZNF799;ZNF805;ZNF808;ZNF81;ZNF813;ZNF816;ZNF823;ZNF829;ZNF836;ZNF841;ZNF844;ZNF845;ZNF846;ZNF85;ZNF853;ZNF860;ZNF878;ZNF891;ZNF99;ZSCAN16;ZSCAN2;ZSCAN22;ZSCAN23;ZSCAN29;ZSCAN31;ZSCAN32;ZSCAN4;ZSCAN5A;ZSCAN5B;ZSCAN5C;ZSCAN9;ZXDA;ZXDB;ZXDC	GLI1;GLI2;GLI3;GLIS1;GLIS2;GLIS3;ZIC1;ZIC2;ZIC3;ZIC4
HEY2_HUMAN.H11MO.0.D	36	0.21052632	0.26255686	0.5679351	0.3906540	0,07970583	3,84E+00	Hairy-related factors{1.2.4}	Hairy-like factors{1.2.4.1}	BHLHE40;BHLHE41;HELT;HES1;HES2;HES3;HES4;HES5;HES6;HES7;HEY1;HEY2;HEYL	BHLHE40;BHLHE41;HES1;HES5;HES7;HEY1;HEY2
SHOX2_HUMAN.H11MO.0.D	6	0.03508772	0.26134551	16.079.050	0.7227328	588846,60000000	7,86E+05	Paired-related HD factors{3.1.3}	SHOX{3.1.3.25}	ALX1;ALX3;ALX4;ARGFX;ARX;CRX;DMBX1;DPRX;DRGX;DUX4;DUXA;ESX1;GSC;GSC2;HESX1;ISX;LEUTX;MIXL1;NOBOX;OTP;OTX1;OTX2;PHOX2A;PHOX2B;PITX1;PITX2;PITX3;PROP1;PRRX1;PRRX2;RAX;RAX2;RHOXF1;RHOXF2;SEBOX;SHOX;SHOX2;TPRX1;UNCX;VSX1;VSX2	SHOX2;SHOX

motif	Nº of probes	% of probes	lower OR	upper OR	OR	p.value	FDR	TF family	TF subfamily	TF.family.member	TF.subfamily.member
HSFY1_HUMAN.H11MO.0.D	6	0.03508772	0.25871160	15.917.185	0.7154614	589867,30000000	7,86E+05	HSF factors{3.4.1}	HSFY1 (HSFY2, HSF2L){3.4.1.0.6}	HSF1;HSF2;HSF4;HSFY1; HSFY2	HSFY1; HSFY2
ZEP1_HUMAN.H11MO.0.D	10	0.05847953	0.25778167	10.344.569	0.5479852	74621,73000000	2,23E+05	Factors with multiple dispersed zinc fingers{2.3.4}	HIV EP-factors{2.3.4.5}	BCL11A;BCL11B;BNC1;BNC2;E4F1;HIC1;HIC2;HINFP;HIVEP1;HIVEP2;HIVEP3;IKZF1;IKZF2;IKZF3;IKZF4;IKZF5;INSM1;INSM2;MAZ;MECOM;PATZ1;PRDM16;PRDM4;REST;RLF;RREB1;SALL1;SALL2;SALL3;SALL4;VEZF1;ZBTB1;ZBTB17;ZBTB2;ZBTB25;ZBTB4;ZFAT;ZNF134;ZNF211;ZNF217;ZNF219;ZNF248;ZNF256;ZNF292;ZNF296;ZNF319;ZNF334;ZNF335;ZNF341;ZNF37A;ZNF382;ZNF417;ZNF418;ZNF423;ZNF467;ZNF510;ZNF512;ZNF512B;ZNF516;ZNF518A;ZNF518B;ZNF521;ZNF526;ZNF532;ZNF536;ZNF552;ZNF574;ZNF587;ZNF587B;ZNF592;ZNF639;ZNF654;ZNF658;ZNF671;ZNF687;ZNF711;ZNF717;ZNF770;ZNF772;ZNF784;ZNF786;ZNF792;ZNF8;ZNF814	HIVEP1;HIVEP2
HXA5_HUMAN.H11MO.0.D	5	0.02923977	0.24584148	18.284.338	0.7674467	690408,80000000	8,67E+05	HOX-related factors{3.1.1}	HOX5{3.1.1.5}	CDX1;CDX2;CDX4;EVX1;EVX2;GBX1;GBX2;GSX1;GSX2;HDX;HMBOX1;HNF1A;HNF1B;HOXA1;HOXA10;HOXA11;HOXA13;HOXA2;HOXA3;HOXA4;HOXA5;HOXA6;HOXA7;HOXA9;HOXB1;HOXB13;HOXB2;HOXB3;HOXB4;HOXB5;HOXB6;HOXB7;HOXB8;HOXB9;HOXC10;HOXC11;HOXC12;HOXC13;HOXC4;HOXC5;HOXC6;HOXC8;HOXC9;HOXD1;HOXD10;HOXD11;HOXD12;HOXD13;HOXD3;HOXD4;HOXD8;HOXD9;MEOX1;MEOX2;MNX1;PDX1;POU1F1;POU2F1;POU2F2;POU2F3;POU3F1;POU3F2;POU3F3;POU3F4;POU4F1;POU4F2;POU4F3;POU5F1;POU5F2;POU6F1;POU6F2	HOXA5
AP2B_HUMAN.H11MO.0.B	35	0.20467836	0.24557947	0.5357048	0.3671009	0,01303176	7,73E-01	AP-2{1.3.1}	AP-2beta{1.3.1.0.2}	TFAP2A;TFAP2B;TFAP2C;TFAP2D	TFAP2B
RHXF1_HUMAN.H11MO.0.D	6	0.03508772	0.23695707	14.577.523	0.6552814	390705,70000000	6,29E+05	Paired-related HD factors{3.1.3}	RHOX{3.1.3.23}	ALX1;ALX3;ALX4;ARGFX;ARX;CRX;DMBX1;DPRX;DRGX;DUX4;DUXA;ESX1;GSC;GSC2;HESX1;ISX;LEUTX;MIXL1;NOBOX;OTP;OTX1;OTX2;PHOX2A;PHOX2B;PITX1;PITX2;PITX3;PROP1;PRRX1;PRRX2;RAX;RAX2;RHOXF1;RHOXF2;SEBOX;SHOX;SHOX2;TPRX1;UNCX;VSX1;VSX2	RHOXF1
MECP2_HUMAN.H11MO.0.C	31	0.18128655	0.23387212	0.5302710	0.3572359	0,01444963	7,96E-01	NA	NA	MECP2	MECP2
NR1I2_HUMAN.H11MO.0.C	7	0.04093567	0.23198402	12.358.608	0.5861794	220347,00000000	4,48E+05	Thyroid hormone receptor-related factors (NR1){2.1.2}	Vitamin D receptor (NR1I){2.1.2.4}	NR1D1;NR1D2;NR1H2;NR1H3;NR1H4;NR1I2;NR1I3;PPARA;PPARD;PPARG;RARA;RARB;RARG;RORA;RORB;RORC;THRA;THRB;VDR	NR1I2;NR1I3;VDR
MBD2_HUMAN.H11MO.0.B	37	0.21637427	0.23092851	0.4954818	0.3420172	0,00057481	5,54E-02	NA	NA	MBD2	MBD2

motif	Nº of probes	% of probes	lower OR	upper OR	OR	p.value	FDR	TF family	TF subfamily	TF.family.member	TF.subfamily.member
TYY2_HUMAN.H11MO.0.D	10	0.05847953	0.22685418	0.9102513	0.4822200	21334,22000000	9,62E+04	More than 3 adjacent zinc finger factors{2.3.3}	YY1-like factors{2.3.3.9}	BCL6;BCL6B;CTCF;CTCFL;FEZF1;FEZF2;GFI1;GFI1B;GLI1;GLI2;GLI3;GLI4;GLIS1;GLIS2;GLIS3;HKR1;MTF1;MYNN;MZF1;OSR2;OVOL1;OVOL2;PLAG1;PLAGL1;PLAGL2;PRDM1;PRDM14;PRDM6;SCRT1;SCRT2;SNAI1;SNAI2;SNAI3;WT1;YY1;YY2;ZBTB12;ZBTB14;ZBTB18;ZBTB20;ZBTB26;ZBTB42;ZBTB45;ZBTB47;ZBTB48;ZBTB49;ZBTB6;ZBTB7A;ZBTB7B;ZBTB7C;ZFP14;ZFP2;ZFP28;ZFP30;ZFP37;ZFP42;ZFP64;ZFP69;ZFP69B;ZFP82;ZFP91;ZFX;ZIC1;ZIC2;ZIC3;ZIC4;ZIC5;ZIK1;ZIM3;ZKSCAN1;ZKSCAN2;ZKSCAN3;ZKSCAN4;ZNF121;ZNF124;ZNF133;ZNF136;ZNF138;ZNF14;ZNF140;ZNF143;ZNF146;ZNF148;ZNF155;ZNF157;ZNF160;ZNF169;ZNF175;ZNF177;ZNF18;ZNF180;ZNF181;ZNF2;ZNF20;ZNF212;ZNF213;ZNF214;ZNF221;ZNF222;ZNF223;ZNF224;ZNF225;ZNF226;ZNF227;ZNF229;ZNF230;ZNF232;ZNF233;ZNF234;ZNF235;ZNF24;ZNF25;ZNF250;ZNF257;ZNF26;ZNF260;ZNF263;ZNF264;ZNF268;ZNF274;ZNF276;ZNF28;ZNF280A;ZNF280B;ZNF280C;ZNF280D;ZNF281;ZNF282;ZNF283;ZNF284;ZNF285;ZNF286A;ZNF286B;ZNF3;ZNF30;ZNF300;ZNF302;ZNF317;ZNF32;ZNF320;ZNF322;ZNF324;ZNF324B;ZNF329;ZNF331;ZNF333;ZNF33A;ZNF33B;ZNF343;ZNF345;ZNF347;ZNF350;ZNF354A;ZNF354B;ZNF362;ZNF366;ZNF383;ZNF384;ZNF394;ZNF397;ZNF398;ZNF404;ZNF41;ZNF410;ZNF419;ZNF420;ZNF431;ZNF432;ZNF436;ZNF439;ZNF44;ZNF440;ZNF442;ZNF443;ZNF446;ZNF449;ZNF45;ZNF460;ZNF468;ZNF479;ZNF484;ZNF490;ZNF500;ZNF502;ZNF524;ZNF525;ZNF528;ZNF543;ZNF544;ZNF546;ZNF547;ZNF548;ZNF549;ZNF554;ZNF555;ZNF557;ZNF558;ZNF559;ZNF561;ZNF562;ZNF563;ZNF564;ZNF566;ZNF567;ZNF568;ZNF57;ZNF570;ZNF571;ZNF572;ZNF577;ZNF581;ZNF582;ZNF583;ZNF585A;ZNF586;ZNF589;ZNF595;ZNF599;ZNF600;ZNF605;ZNF607;ZNF611;ZNF613;ZNF614;ZNF615;ZNF616;ZNF619;ZNF620;ZNF621;ZNF625;ZNF627;ZNF649;ZNF652;ZNF653;ZNF665;ZNF667;ZNF669;ZNF670;ZNF672;ZNF679;ZNF680;ZNF683;ZNF689;ZNF692;ZNF701;ZNF705D;ZNF705E;ZNF705G;ZNF708;ZNF709;ZNF71;ZNF710;ZNF713;ZNF721;ZNF727;ZNF729;ZNF736;ZNF75A;ZNF75D;ZNF76;ZNF763;ZNF764;ZNF765;ZNF768;ZNF77;ZNF771;ZNF773;ZNF774;ZNF776;ZNF777;ZNF780A;ZNF780B;ZNF782;ZNF785;ZNF799;ZNF805;ZNF808;ZNF81;ZNF813;ZNF816;ZNF823;ZNF829;ZNF836;ZNF841;ZNF844;ZNF845;ZNF846;ZNF85;ZNF853;ZNF860;ZNF878;ZNF891;ZNF99;ZSCAN16;ZSCAN2;ZSCAN22;ZSCAN23;ZSCAN29;ZSCAN31;ZSCAN32;ZSCAN4;ZSCAN5A;ZSCAN5B;ZSCAN5C;ZSCAN9;ZXDA;ZXDB;ZXDC	YY1;YY2;ZFP42
ZBT14_HUMAN.H11MO.0.C	31	0.18128655	0.21935159	0.4973019	0.3350270	0,00150060	1,29E-01	More than 3 adjacent zinc finger factors{2.3.3}	unclassified{2.3.3.0}	BCL6B;BCL6;CTCFL;CTCF;FEZF1;GFI1B;GFI1;GLI1;GLI2;GLI3;GLIS1;GLIS2;GLIS3;MTF1;MYNN;MZF1;OSR2;OVOL1;OVOL2;ZNF146;PLAG1;PLAGL1;PRDM14;PRDM1;PRDM6;SCRT1;SCRT2;SNAI1;SNAI2;YY1;YY2;WT1;ZNF324;ZNF354A;ZBTB14;ZBTB18;ZBTB48;ZBTB49;ZBTB7A;ZBTB7B;ZBTB6;ZFP64;ZFP28;ZFP42;ZFP82;ZFX;ZIC1;ZIC2;ZIC3;ZIC4;ZIM3;ZKSCAN1;ZKSCAN3;ZNF121;ZNF136;ZNF140;ZNF143;ZNF148;ZNF214;ZNF232;ZNF250;ZNF257;ZNF260;ZNF263;ZNF264;ZNF274;ZNF281;ZNF282;ZNF317;ZNF320;ZNF322;ZNF329;ZNF331;ZNF333;ZN	MYNN;MZF1;OSR2;PRDM14;PRDM6;WT1;ZBTB14;ZBTB48;ZBTB49;ZFP64;ZFP28;ZIM3;ZNF121;ZNF250;ZNF257;ZNF263;ZNF274;ZNF317;ZNF320;ZNF329;ZNF331;ZNF394;ZNF449;ZNF502;ZNF528;ZNF547;ZNF549;ZNF554;ZNF586;ZNF589;ZNF667;ZNF680;ZNF708;ZNF713;ZNF768;ZNF18;ZNF85;ZSCAN16;ZSCAN22

motif	Nº of probes	% of probes	lower OR	upper OR	OR	p.value	FDR	TF family	TF subfamily	TF.family.member	TF.subfamily.member
										F350;ZNF384;ZNF394;ZNF410;ZNF436;ZNF449;ZNF490;ZNF502;ZNF524;ZNF528;ZNF547;ZNF549;ZNF554;ZNF563;ZNF582;ZNF586;ZNF589;ZNF652;ZNF667;ZNF680;ZNF708;ZNF713;ZNF768;ZNF816;ZNF18;ZNF41;ZNF76;ZNF85;ZSCAN16;ZSCAN22;ZSCAN31;ZSCAN4	
NFIA_HUMAN.H11MO.0.C	10	0.05847953	0.21828286	0.8759087	0.4640262	12428,19000000	6,60E+04	Nuclear factor 1{7.1.2}	NF-1A (NF-IA){7.1.2.0.1}	NFIA;NFIB;NFIC	NFIA
EVI1_HUMAN.H11MO.0.B	6	0.03508772	0.21559682	13.262.590	0.5961920	250289,50000000	4,84E+05	Factors with multiple dispersed zinc fingers{2.3.4}	Evi-1-like factors{2.3.4.14}	BCL11A;BCL11B;BNC1;BNC2;E4F1;HIC1;HIC2;HINFP;HIVEP1;HIVEP2;HIVEP3;IKZF1;IKZF2;IKZF3;IKZF4;IKZF5;INSM1;INSM2;MAZ;MECOM;PATZ1;PRDM16;PRDM4;REST;RLF;RREB1;SALL1;SALL2;SALL3;SALL4;VEZF1;ZBTB1;ZBTB17;ZBTB2;ZBTB25;ZBTB4;ZFAT;ZNF134;ZNF211;ZNF217;ZNF219;ZNF248;ZNF256;ZNF292;ZNF296;ZNF319;ZNF334;ZNF335;ZNF341;ZNF37A;ZNF382;ZNF417;ZNF418;ZNF423;ZNF467;ZNF510;ZNF512;ZNF512B;ZNF516;ZNF518A;ZNF518B;ZNF521;ZNF526;ZNF532;ZNF536;ZNF552;ZNF574;ZNF587;ZNF587B;ZNF592;ZNF639;ZNF654;ZNF658;ZNF671;ZNF687;ZNF711;ZNF717;ZNF770;ZNF772;ZNF784;ZNF786;ZNF792;ZNF8;ZNF814	MECOM
PRD14_HUMAN.H11MO.0.A	6	0.03508772	0.21087469	12.971.406	0.5831269	251568,50000000	4,85E+05	More than 3 adjacent zinc finger factors{2.3.3}	unclassified{2.3.3.0}	BCL6B;BCL6;CTCFL;CTCF;FEZF1;GFI1B;GFI1;GLI1;GLI2;GLI3;GLIS1;GLIS2;GLIS3;MTF1;MYNN;MZF1;OSR2;OVOL1;OVOL2;ZNF146;PLAG1;PLAGL1;PRDM14;PRDM1;PRDM6;SCRT1;SCRT2;SNAI1;SNAI2;YY1;YY2;WT1;ZNF324;ZNF354A;ZBTB14;ZBTB18;ZBTB48;ZBTB49;ZBTB7A;ZBTB7B;ZBTB6;ZFP64;ZFP28;ZFP42;ZFP82;ZFX;ZIC1;ZIC2;ZIC3;ZIC4;ZIM3;ZKSCAN1;ZKSCAN3;ZNF121;ZNF136;ZNF140;ZNF143;ZNF148;ZNF214;ZNF232;ZNF250;ZNF257;ZNF260;ZNF263;ZNF264;ZNF274;ZNF281;ZNF282;ZNF317;ZNF320;ZNF322;ZNF329;ZNF331;ZNF333;ZNF350;ZNF384;ZNF394;ZNF410;ZNF436;ZNF449;ZNF490;ZNF502;ZNF524;ZNF528;ZNF547;ZNF549;ZNF554;ZNF563;ZNF582;ZNF586;ZNF589;ZNF652;ZNF667;ZNF680;ZNF708;ZNF713;ZNF768;ZNF816;ZNF18;ZNF41;ZNF76;ZNF85;ZSCAN16;ZSCAN22;ZSCAN31;ZSCAN4	MYNN;MZF1;OSR2;PRDM14;PRDM6;WT1;ZBTB14;ZBTB48;ZBTB49;ZFP64;ZFP28;ZIM3;ZNF121;ZNF250;ZNF257;ZNF263;ZNF274;ZNF317;ZNF320;ZNF329;ZNF331;ZNF394;ZNF449;ZNF502;ZNF528;ZNF547;ZNF549;ZNF554;ZNF586;ZNF589;ZNF667;ZNF680;ZNF708;ZNF713;ZNF768;ZNF18;ZNF85;ZSCAN16;ZSCAN22
ISL2_HUMAN.H11MO.0.D	5	0.02923977	0.21057803	15.660.796	0.6573816	454647,80000000	6,83E+05	HD-LIM factors{3.1.5}	ISL{3.1.5.1}	ISL1;ISL2;LHX1;LHX2;LHX3;LHX4;LHX5;LHX6;LHX8;LHX9;LMX1A;LMX1B	ISL1;ISL2
NFYC_HUMAN.H11MO.0.A	5	0.02923977	0.20241635	15.051.763	0.6318312	458485,40000000	6,86E+05	Heteromeric CCAAT-binding factors{4.2.1}	NF-YC{4.2.1.0.3}	NFYA;NFYB;NFYC	NFYC
SPZ1_HUMAN.H11MO.0.D	13	0.07602339	0.20029429	0.6770156	0.3845725	261,29950000	3,60E+03	NA	NA	SPZ1	SPZ1
GBX1_HUMAN.H11MO.0.D	5	0.02923977	0.18859613	14.022.576	0.5886720	287642,70000000	5,22E+05	HOX-related factors{3.1.1}	GBX (Gastrulation brain homeobox){3.1.1.11}	CDX1;CDX2;CDX4;EVX1;EVX2;GBX1;GBX2;GSX1;GSX2;HDX;HMBOX1;HNF1A;HNF1B;HOXA1;HOXA10;HOXA11;HOXA13;HOXA2;HOXA3;HOXA4;HOXA5;HOXA6;HOXA7;HOXA9;HOXB1;HOXB13;HOXB2;HOXB3;HOXB4;HOXB5;HOXB6;HOXB7;HOXB8;HOXB9;HOXC10;HOXC11;HOXC12;HOXC13;HOXC4;HOXC5;HOXC6;HOXC8;HOXC9;HOXD1;HOXD10;HOXD11;HOXD12;HOXD13;HOXD3;HOXD4;HOXD8;HOXD9;MEOX1;MEOX2;MNX1;PDX1;POU1F1;PO	GBX1;GBX2

motif	Nº of probes	% of probes	lower OR	upper OR	OR	p.value	FDR	TF family	TF subfamily	TF.family.member	TF.subfamily.member
										U2F1;POU2F2;POU2F3;POU3F1;POU3F2;POU3F3;POU3F4;POU4F1;POU4F2;POU4F3;POU5F1;POU5F2;POU6F1;POU6F2	
SHOX_HUMAN.H11MO.0.D	5	0.02923977	0.18790710	13.971.249	0.5865199	287644,60000000	5,22E+05	Paired-related HD factors{3.1.3}	SHOX{3.1.3.25}	ALX1;ALX3;ALX4;ARGFX;ARX;CRX;DMBX1;DPRX;DRGX;DUX4;DUXA;ESX1;GSC;GSC2;HESX1;ISX;LEUTX;MIXL1;NOBOX;OTP;OTX1;OTX2;PHOX2A;PHOX2B;PITX1;PITX2;PITX3;PROP1;PRRX1;PRRX2;RAX;RAX2;RHOXF1;RHOXF2;SEBOX;SHOX;SHOX2;TPRX1;UNCX;VSX1;VSX2	SHOX2;SHOX
PPARA_HUMAN.H11MO.1.B	8	0.04678363	0.18591193	0.8839830	0.4379963	15251,64000000	7,64E+04	Thyroid hormone receptor-related factors (NR1){2.1.2}	PPAR (NR1C){2.1.2.5}	NR1D1;NR1D2;NR1H2;NR1H3;NR1H4;NR1I2;NR1I3;PPARA;PPARD;PPARG;RARA;RARB;RARG;RORA;RORB;RORC;THRA;THRB;VDR	PPARA;PPARD;PPARG
NFYA_HUMAN.H11MO.0.A	4	0.02339181	0.14477483	14.037.479	0.5378040	257771,80000000	4,90E+05	Heteromeric CCAAT-binding factors{4.2.1}	NF-YA (CP1A, CBF-B){4.2.1.0.1}	NFYA;NFYB;NFYC	NFYA
DPRX_HUMAN.H11MO.0.D	4	0.02339181	0.13991455	13.564.219	0.5196884	259409,70000000	4,90E+05	Paired-related HD factors{3.1.3}	DPRX{3.1.3.5}	ALX1;ALX3;ALX4;ARGFX;ARX;CRX;DMBX1;DPRX;DRGX;DUX4;DUXA;ESX1;GSC;GSC2;HESX1;ISX;LEUTX;MIXL1;NOBOX;OTP;OTX1;OTX2;PHOX2A;PHOX2B;PITX1;PITX2;PITX3;PROP1;PRRX1;PRRX2;RAX;RAX2;RHOXF1;RHOXF2;SEBOX;SHOX;SHOX2;TPRX1;UNCX;VSX1;VSX2	DPRX
ZN350_HUMAN.H11MO.0.C	5	0.02923977	0.13351717	0.9925414	0.4167204	45888,32000000	1,64E+05	More than 3 adjacent zinc finger factors{2.3.3}	ZNF350-like factors{2.3.3.30}	BCL6;BCL6B;CTCF;CTCFL;FEZF1;FEZF2;GFI1;GFI1B;GLI1;GLI2;GLI3;GLI4;GLIS1;GLIS2;GLIS3;HKR1;MTF1;MYNN;MZF1;OSR2;OVOL1;OVOL2;PLAG1;PLAGL1;PLAGL2;PRDM1;PRDM14;PRDM6;SCRT1;SCRT2;SNAI1;SNAI2;SNAI3;WT1;YY1;YY2;ZBTB12;ZBTB14;ZBTB18;ZBTB20;ZBTB26;ZBTB42;ZBTB45;ZBTB47;ZBTB48;ZBTB49;ZBTB6;ZBTB7A;ZBTB7B;ZBTB7C;ZFP14;ZFP2;ZFP28;ZFP30;ZFP37;ZFP42;ZFP64;ZFP69;ZFP69B;ZFP82;ZFP91;ZFX;ZIC1;ZIC2;ZIC3;ZIC4;ZIC5;ZIK1;ZIM3;ZKSCAN1;ZKSCAN2;ZKSCAN3;ZKSCAN4;ZNF121;ZNF124;ZNF133;ZNF136;ZNF138;ZNF14;ZNF140;ZNF143;ZNF146;ZNF148;ZNF155;ZNF157;ZNF160;ZNF169;ZNF175;ZNF177;ZNF18;ZNF180;ZNF181;ZNF2;ZNF20;ZNF212;ZNF213;ZNF214;ZNF221;ZNF222;ZNF223;ZNF224;ZNF225;ZNF226;ZNF227;ZNF229;ZNF230;ZNF232;ZNF233;ZNF234;ZNF235;ZNF24;ZNF25;ZNF250;ZNF257;ZNF26;ZNF260;ZNF263;ZNF264;ZNF268;ZNF274;ZNF276;ZNF28;ZNF280A;ZNF280B;ZNF280C;ZNF280D;ZNF281;ZNF282;ZNF283;ZNF284;ZNF285;ZNF286A;ZNF286B;ZNF3;ZNF30;ZNF300;ZNF302;ZNF317;ZNF32;ZNF320;ZNF322;ZNF324;ZNF324B;ZNF329;ZNF331;ZNF333;ZNF33A;ZNF33B;ZNF343;ZNF345;ZNF347;ZNF350;ZNF354A;ZNF354B;ZNF362;ZNF366;ZNF383;ZNF384;ZNF394;ZNF397;ZNF398;ZNF404;ZNF41;ZNF410;ZNF419;ZNF420;ZNF431;ZNF432;ZNF436;ZNF439;ZNF44;ZNF440;ZNF442;ZNF443;ZNF446;ZNF449;ZNF45;ZNF460;ZNF468;ZNF479;ZNF484;ZNF490;ZNF500;ZNF502;ZNF524;ZNF525;ZNF528;ZNF543;ZNF544;ZNF546;ZNF547;ZNF548;ZNF549;ZNF554;ZNF555;ZNF557;ZNF558;ZNF559;ZNF561;ZNF562;ZNF563;ZNF564;ZNF566;ZNF567;ZNF568;ZNF57;ZNF570;ZNF571;ZNF572;ZNF577;ZNF581;ZNF582;ZNF583;ZNF585A;ZNF586;ZNF589;ZNF595;ZNF599;ZNF600;ZNF605;ZNF607;ZNF611;ZN	ZNF350

motif	Nº of probes	% of probes	lower OR	upper OR	OR	p.value	FDR	TF family	TF subfamily	TF.family.member	TF.subfamily.member
										F613;ZNF614;ZNF615;ZNF616;ZNF619;ZNF620;ZNF621;ZNF625;ZNF627;ZNF649;ZNF652;ZNF653;ZNF665;ZNF667;ZNF669;ZNF670;ZNF672;ZNF679;ZNF680;ZNF683;ZNF689;ZNF692;ZNF701;ZNF705D;ZNF705E;ZNF705G;ZNF708;ZNF709;ZNF71;ZNF710;ZNF713;ZNF721;ZNF727;ZNF729;ZNF736;ZNF75A;ZNF75D;ZNF76;ZNF763;ZNF764;ZNF765;ZNF768;ZNF77;ZNF771;ZNF773;ZNF774;ZNF776;ZNF777;ZNF780A;ZNF780B;ZNF782;ZNF785;ZNF799;ZNF805;ZNF808;ZNF81;ZNF813;ZNF816;ZNF823;ZNF829;ZNF836;ZNF841;ZNF844;ZNF845;ZNF846;ZNF85;ZNF853;ZNF860;ZNF878;ZNF891;ZNF99;ZSCAN16;ZSCAN2;ZSCAN22;ZSCAN23;ZSCAN29;ZSCAN31;ZSCAN32;ZSCAN4;ZSCAN5A;ZSCAN5B;ZSCAN5C;ZSCAN9;ZXDA;ZXDB;ZXDC	
LHX8_HUMAN.H11MO.0.D	3	0.01754386	0.09096546	13.258.735	0.4457003	227014,10000000	4,55E+05	HD-LIM factors{3.1.5}	Lhx-6-like factors{3.1.5.5}	ISL1;ISL2;LHX1;LHX2;LHX3;LHX4;LHX5;LHX6;LHX8;LHX9;LMX1A;LMX1B	LHX6;LHX8
HXA7_HUMAN.H11MO.0.D	2	0.01169591	0.04391388	13.423.293	0.3656706	184321,40000000	4,14E+05	HOX-related factors{3.1.1}	HOX6-7{3.1.1.6}	CDX1;CDX2;CDX4;EVX1;EVX2;GBX1;GBX2;GSX1;GSX2;HDX;HMBOX1;HNF1A;HNF1B;HOXA1;HOXA10;HOXA11;HOXA13;HOXA2;HOXA3;HOXA4;HOXA5;HOXA6;HOXA7;HOXA9;HOXB1;HOXB13;HOXB2;HOXB3;HOXB4;HOXB5;HOXB6;HOXB7;HOXB8;HOXB9;HOXC10;HOXC11;HOXC12;HOXC13;HOXC4;HOXC5;HOXC6;HOXC8;HOXC9;HOXD1;HOXD10;HOXD11;HOXD12;HOXD13;HOXD3;HOXD4;HOXD8;HOXD9;MEOX1;MEOX2;MNX1;PDX1;POU1F1;POU2F1;POU2F2;POU2F3;POU3F1;POU3F2;POU3F3;POU3F4;POU4F1;POU4F2;POU4F3;POU5F1;POU5F2;POU6F1;POU6F2	HOXA7;HOXB6;HOXB7;HOXC6
UBIP1_HUMAN.H11MO.0.D	0	0.00000000	0.00000000	Inf	0.0000000	1000000,00000000	1,00E+06	CP2-related factors{6.7.2}	UBP-1 (LBP-1, SEF){6.7.2.0.3}	TFCP2;UBP1	UBP1
SOX15_HUMAN.H11MO.0.D	0	0.00000000	0.00000000	Inf	0.0000000	1000000,00000000	1,00E+06	SOX-related factors{4.1.1}	Group G{4.1.1.7}	BBX;CIC;HBP1;SOX1;SOX10;SOX11;SOX12;SOX13;SOX14;SOX15;SOX17;SOX18;SOX2;SOX21;SOX3;SOX30;SOX4;SOX5;SOX6;SOX7;SOX8;SOX9;SRY	SOX15
PRRX2_HUMAN.H11MO.0.C	0	0.00000000	0.00000000	Inf	0.0000000	1000000,00000000	1,00E+06	Paired-related HD factors{3.1.3}	PRRX{3.1.3.21}	ALX1;ALX3;ALX4;ARGFX;ARX;CRX;DMBX1;DPRX;DRGX;DUX4;DUXA;ESX1;GSC;GSC2;HESX1;ISX;LEUTX;MIXL1;NOBOX;OTP;OTX1;OTX2;PHOX2A;PHOX2B;PITX1;PITX2;PITX3;PROP1;PRRX1;PRRX2;RAX;RAX2;RHOXF1;RHOXF2;SEBOX;SHOX;SHOX2;TPRX1;UNCX;VSX1;VSX2	PRRX1;PRRX2
NKX61_HUMAN.H11MO.1.B	0	0.00000000	0.00000000	Inf	0.0000000	1000000,00000000	1,00E+06	NK-related factors{3.1.2}	NK-6{3.1.2.19}	BARHL1;BARHL2;BARX1;BARX2;BSX;DBX1;DBX2;DLX1;DLX2;DLX3;DLX4;DLX5;DLX6;EMX1;EMX2;EN1;EN2;HHEX;HLX;HMX1;HMX2;HMX3;LBX1;LBX2;MSX1;MSX2;NANOG;NKX1-1;NKX1-2;NKX2-1;NKX2-2;NKX2-3;NKX2-4;NKX2-5;NKX2-6;NKX2-8;NKX3-1;NKX3-2;NKX6-1;NKX6-2;NKX6-3;NOTO;TLX1;TLX2;TLX3;VAX1;VAX2;VENTX	NKX6-1;NKX6-2

motif	Nº of probes	% of probes	lower OR	upper OR	OR	p.value	FDR	TF family	TF subfamily	TF.family.member	TF.subfamily.member
NFIA_HUMAN.H11MO.1.D	0	0.00000000	0.00000000	Inf	0.0000000	1000000,00000000	1,00E+06	Nuclear factor 1{7.1.2}	NF-1A (NF-IA){7.1.2.0.1}	NFIA;NFIB;NFIC	NFIA
NF2L1_HUMAN.H11MO.0.C	0	0.00000000	0.00000000	Inf	0.0000000	1000000,00000000	1,00E+06	Jun-related factors{1.1.1}	NF-E2-like factors{1.1.1.2}	ATF2;ATF7;BACH1;BACH2;CREB5;JUN;JUNB;JUND;NFE2;NFE2L1;NFE2L2;NFE2L3	BACH1;BACH2;NFE2L1;NFE2L2;NFE2
MSX2_HUMAN.H11MO.0.D	0	0.00000000	0.00000000	Inf	0.0000000	1000000,00000000	1,00E+06	NK-related factors{3.1.2}	MSX{3.1.2.11}	BARHL1;BARHL2;BARX1;BARX2;BSX;DBX1;DBX2;DLX1;DLX2;DLX3;DLX4;DLX5;DLX6;EMX1;EMX2;EN1;EN2;HHEX;HLX;HMX1;HMX2;HMX3;LBX1;LBX2;MSX1;MSX2;NANOG;NKX1-1;NKX1-2;NKX2-1;NKX2-2;NKX2-3;NKX2-4;NKX2-5;NKX2-6;NKX2-8;NKX3-1;NKX3-2;NKX6-1;NKX6-2;NKX6-3;NOTO;TLX1;TLX2;TLX3;VAX1;VAX2;VENTX	MSX1;MSX2
HMGA1_HUMAN.H11MO.0.D	0	0.00000000	0.00000000	Inf	0.0000000	1000000,00000000	1,00E+06	HMGA factors{8.2.1}	HMGA1 (HMGI(Y)){8.2.1.0.1}	HMGA1;HMGA2	HMGA1
ETV7_HUMAN.H11MO.0.D	0	0.00000000	0.00000000	Inf	0.0000000	1000000,00000000	1,00E+06	Ets-related factors{3.5.2}	ETV6-like factors{3.5.2.6}	EHF;ELF1;ELF2;ELF3;ELF4;ELF5;ELK1;ELK3;ELK4;ERF;ERG;ETS1;ETS2;ETV1;ETV2;ETV3;ETV3L;ETV4;ETV5;ETV6;ETV7;FEV;FLI1;GABPA;SPDEF;SPI1;SPIB;SPIC	ETV6;ETV7

Table S2B - Transcription factors binding motifs and TF lists associated with DNA hypomethylation in SCCOT

Probe	GeneID	Symbol	Distance	Sides	Raw.p	Pe
cg02577773	ENSG00000229647	MYOSLID	76502	R6	2,16E+03	0.000331491712707182
cg10149296	ENSG00000231948	HS1BP3-IT1	-3603	L2	2,83E+03	0.000184162062615101
cg00929345	ENSG00000160886	LY6K	159117	R7	6,05E+03	0.000147329650092081
cg01963702	ENSG00000251381	LINC00958	794391	R10	8,48E+03	0.000184162062615101
cg14176942	ENSG00000237548	TTLL11-IT1	-420435	L10	8,77E+03	0.000368324125230203
cg02452500	ENSG00000251381	LINC00958	-150831	L4	9,22E+03	0.000220994475138122
cg22729539	ENSG00000158023	WDR66	-77285	L5	1,79E+04	7,37E+09
cg02555944	ENSG00000160886	LY6K	137363	R6	2,70E+03	0.000368324125230203
cg04756252	ENSG00000243537	KC877373.1	288891	R5	2,78E+03	0.000184162062615101
cg07865418	ENSG00000133398	MED10	-32723	L1	5,16E+04	0.000257826887661142
cg25939350	ENSG00000153395	LPCAT1	187323	R7	5,16E+04	0.000552486187845304
cg21787176	ENSG00000244128	LINC01322	16127	R1	5,46E+04	0.000368324125230203
cg23939096	ENSG00000153395	LPCAT1	-31713	L2	7,10E+04	0.000699815837937385
cg18291443	ENSG00000178053	MLF1	1110816	R11	7,69E+04	0.000184162062615101
cg20390052	ENSG00000231298	MANCR	725748	R8	8,23E+04	7,37E+09
cg07189394	ENSG00000114686	MRPL3	-580687	L6	9,75E+04	3,68E+09
cg08646805	ENSG00000122641	INHBA	1368602	R5	1,14E+05	0.000220994475138122
cg21351392	ENSG00000026652	AGPAT4	0	L1	1,14E+05	0.000257826887661142
cg10308673	ENSG00000185627	PSMD13	-15991	L2	1,19E+05	7,37E+09
cg19409687	ENSG00000175550	DRAP1	3783	R1	1,24E+05	3,68E+09
cg02902102	ENSG00000105464	GRIN2D	0	L1	1,56E+05	7,37E+09
cg26571814	ENSG00000111700	SLCO1B3	373962	R4	1,83E+05	3,68E+09
cg03821121	ENSG00000255400	AC124276.2	-37299	L2	1,86E+05	7,37E+09
cg10371643	ENSG00000275216	AL161431.1	114144	R2	1,97E+05	0.000847145488029466
cg09127371	ENSG00000162302	RPS6KA4	-173819	L7	2,13E+04	3,68E+09
cg17238677	ENSG00000173207	CKS1B	210904	R8	2,30E+05	0.000957642725598527
cg09756014	ENSG00000141012	GALNS	-228987	L10	2,59E+05	7,37E+09
cg08646805	ENSG00000175600	SUGCT	0	L1	2,69E+05	0.000883977900552486
cg02261047	ENSG00000131148	EMC8	-128967	L7	2,69E+04	0.000147329650092081
cg19255307	ENSG00000149716	LTO1	-103624	L4	2,79E+05	0.000257826887661142
cg09791621	ENSG00000127586	CHTF18	-227984	L10	2,90E+05	0.000110497237569061
cg21802682	ENSG00000168040	FADD	335336	R10	2,90E+05	0.000147329650092081
cg27145347	ENSG00000171067	C11orf24	233144	R9	2,90E+05	0.000441988950276243
cg17222829	ENSG00000246889	AP000487.1	-188699	L4	3,17E+05	0.000257826887661142
cg25763127	ENSG00000229656	ITGB1-DT	-156232	L4	3,37E+05	0.000883977900552486
cg20391126	ENSG00000106348	IMPDH1	200898	R7	3,38E+05	0.000847145488029466
cg16618104	ENSG00000171310	CHST11	0	L1	3,65E+05	0.000147329650092081
cg19106326	ENSG00000169490	TM2D2	270975	R7	4,41E+05	3,68E+09
cg08823113	ENSG00000171421	MRPL36	135892	R3	4,59E+05	3,68E+09
cg06469726	ENSG00000168393	DTYMK	131693	R6	4,95E+05	7,37E+09

cg13491731	ENSG00000119650	IFT43	-43854	L2	5,33E+05	3,68E+09
cg25213547	ENSG00000133398	MED10	-208608	L4	5,33E+05	0.000626151012891344
cg07470207	ENSG00000132297	HHLA1	244985	R3	5,34E+05	3,68E+09
**Probe**	**GeneID**	**Symbol**	**Distance**	**Sides**	**Raw.p**	**Pe**
cg13185005	ENSG00000185627	PSMD13	-15965	L2	5,54E+05	7,37E+09
cg15717250	ENSG00000121578	B4GALT4	35092	R2	5,75E+05	0.000368324125230203
cg19409687	ENSG00000172757	CFL1	-53462	L8	5,75E+05	0.000736648250460405
cg24121967	ENSG00000149716	LTO1	331423	R8	6,44E+05	0.000294659300184162
cg05968369	ENSG00000116213	WRAP73	226883	R7	6,69E+05	0.000368324125230203
cg19761458	ENSG00000132432	SEC61G	53668	R1	6,69E+05	0.000810313075506446
cg16209064	ENSG00000250829	AC108865.1	45530	L1	7,15E+05	3,68E+09
cg26066724	ENSG00000258534	AL132712.1	-9380	L1	7,15E+05	0.000147329650092081
cg09371112	ENSG00000133816	MICAL2	0	L1	7,20E+05	3,68E+09
cg09989037	ENSG00000117411	B4GALT2	143671	R7	7,77E+05	0.000773480662983425
cg18293437	ENSG00000125877	ITPA	133968	R8	8,06E+05	3,68E+09
cg11838237	ENSG00000171421	MRPL36	45634	R1	8,37E+05	0.000110497237569061
cg13016851	ENSG00000146731	CCT6A	-163709	L8	9,01E+05	3,68E+09
cg15877233	ENSG00000122641	INHBA	1095077	R4	9,01E+05	0.000810313075506446
cg01397507	ENSG00000131626	PPFIA1	-208977	L5	9,71E+05	3,68E+09
cg04756252	ENSG00000104419	NDRG1	200065	R4	9,71E+05	0.000110497237569061
cg04590790	ENSG00000124279	FASTKD3	88580	R3	1,04E+06	0.000184162062615101
cg23287510	ENSG00000250571	GLI4	192386	R5	1,04E+06	0.000331491712707182
cg25609434	ENSG00000244675	AC108676.1	138802	R5	1,11E+05	0.000405156537753223
cg18014547	ENSG00000213729	AC098828.1	-10624	L2	1,14E+06	0.000368324125230203
cg01963702	ENSG00000254991	AC124276.1	-94434	L4	1,20E+05	3,68E+09
cg08291854	ENSG00000028310	BRD9	241606	R8	1,21E+05	0.000184162062615101
cg16861964	ENSG00000171310	CHST11	0	L1	1,21E+05	0.000368324125230203
cg04515533	ENSG00000182118	FAM89A	-119785	L3	1,21E+06	0.000662983425414365
cg08760493	ENSG00000198856	OSTC	-405062	L5	1,26E+06	0.000147329650092081
cg25132257	ENSG00000254968	AP003063.1	-423687	L5	1,28E+06	7,37E+09
cg08823113	ENSG00000145494	NDUFS6	138907	R4	1,30E+05	3,68E+09
cg19305545	ENSG00000116213	WRAP73	0	L1	1,30E+05	0.000589318600368324
cg23939096	ENSG00000171421	MRPL36	242707	R8	1,35E+06	0.000110497237569061
cg03676624	ENSG00000074201	CLNS1A	0	L1	1,40E+06	0.000220994475138122
cg04543510	ENSG00000113460	BRIX1	-102814	L3	1,40E+06	0.000478821362799263
cg08105590	ENSG00000278341	AC138028.6	-72753	L7	1,43E+06	7,37E+09
cg01397507	ENSG00000246889	AP000487.1	-195801	L4	1,47E+06	0.000552486187845304
cg21802682	ENSG00000149716	LTO1	-192408	L6	1,62E+06	0.000405156537753223
cg06829584	ENSG00000122861	PLAU	9450	L1	1,63E+06	0.000847145488029466
cg04515533	ENSG00000227934	AL732414.1	-137856	L4	1,83E+06	7,37E+09
cg04119977	ENSG00000124279	FASTKD3	32298	R3	1,88E+06	0.000184162062615101

cg00902153	ENSG00000090530	P3H2	1167799	R8	1,88E+05	0.000626151012891344	cg22578425	ENSG00000237870	AC073130.1	458754	R6	9,19E+06	0.000810313075506446
cg25025181	ENSG00000162849	KIF26B	-505775	L6	1,88E+05	0.000110497237569061	cg07450210	ENSG00000280734	LINC01232	258267	R7	9,60E+06	0.000294659300184162
cg20660802	ENSG00000249915	PDCD6	216693	R9	2,02E+06	0.000773480662983425	cg11691844	ENSG00000074266	EED	495190	R10	9,60E+06	0.000331491712707182
cg12892638	ENSG00000248599	AC018781.1	-164303	L9	2,21E+06	0.000699815837937385	cg18519834	ENSG00000101181	MTG2	454689	R8	9,61E+06	0.000147329650092081
cg10453337	ENSG00000124279	FASTKD3	357256	R4	2,25E+06	0.000184162062615101	cg15999077	ENSG00000049656	CLPTM1L	-62308	L5	9,61E+05	0.000478821362799263
cg11299980	ENSG00000125835	SNRPB	256783	R4	2,34E+06	0.000441988950276243	cg03191504	ENSG00000175874	CREG2	39583	R2	9,92E+06	0.000110497237569061
**Probe**	**GeneID**	**Symbol**	**Distance**	**Sides**	**Raw.p**	**Pe**	**Probe**	**GeneID**	**Symbol**	**Distance**	**Sides**	**Raw.p**	**Pe**
cg03857198	ENSG00000181467	RAP2B	-39187	L2	2,34E+06	3,68E+09	cg21545548	ENSG00000275713	HIST1H2BH	-42636	L5	1,02E+07	0.000810313075506446
cg17512922	ENSG00000116213	WRAP73	0	L1	2,34E+06	0.000847145488029466	cg08270682	ENSG00000119650	IFT43	-64013	L2	1,03E+07	0.000847145488029466
cg26571814	ENSG00000257046	AC011604.2	378934	R5	2,35E+06	0.000110497237569061	cg26873457	ENSG00000107937	GTPBP4	305168	R5	1,03E+07	0.000184162062615101
cg15735030	ENSG00000257046	AC011604.2	816020	R9	2,45E+06	0.000110497237569061	cg12324353	ENSG00000049656	CLPTM1L	48668	R2	1,10E+07	0.000736648250460405
cg17494199	ENSG00000233532	LINC00460	191882	R1	2,51E+06	0.000552486187845304	cg01203651	ENSG00000250829	AC108865.1	49865	L1	1,11E+05	0.000110497237569061
cg09371112	ENSG00000050165	DKK3	-191253	L6	2,51E+06	3,68E+09	cg25932290	ENSG00000140525	FANCI	-78759	L6	1,14E+07	0.000220994475138122
cg11020333	ENSG00000146535	GNA12	0	L1	2,51E+06	0.000147329650092081	cg04115702	ENSG00000141570	CBX8	401832	R6	1,18E+07	0.000110497237569061
cg24032710	ENSG00000258534	AL132712.1	-424010	L10	2,59E+06	0.000257826887661142	cg11590974	ENSG00000145592	RPL37	-307556	L7	1,18E+07	0.000184162062615101
cg02577773	ENSG00000225064	LINC01802	97297	R8	2,61E+06	0.000147329650092081	cg15898536	ENSG00000171421	MRPL36	45197	R1	1,18E+07	0.000810313075506446
cg24531955	ENSG00000134013	LOXL2	9	L1	2,70E+06	7,37E+09	cg01397507	ENSG00000085733	CTTN	-157705	L3	1,27E+07	0.000957642725598527
cg13794404	ENSG00000136930	PSMB7	298783	R4	2,90E+06	7,37E+09	cg02849956	ENSG00000074842	MYDGF	6557	R1	1,27E+07	0.000110497237569061
cg20390052	ENSG00000225269	LINC00705	730990	R9	2,93E+06	0.000184162062615101	cg19635401	ENSG00000153989	NUS1	-841180	L6	1,27E+07	7,37E+09
cg06829584	ENSG00000222047	C10orf55	10242	R1	3,11E+06	0.000294659300184162	cg07236958	ENSG00000104472	CHRAC1	898825	R6	1,35E+07	0.000294659300184162
cg03676624	ENSG00000178301	AQP11	0	R1	3,43E+06	0.000515653775322284	cg08326879	ENSG00000108010	GLRX3	-1160336	L7	1,35E+07	0.000662983425414365
cg05368910	ENSG00000231971	AL078590.2	-69337	L2	3,57E+06	7,37E+09	cg08646805	ENSG00000224116	INHBA-AS1	1395350	R6	1,40E+07	0.000773480662983425
cg17871403	ENSG00000124279	FASTKD3	32155	R3	3,59E+06	0.000184162062615101	cg27399125	ENSG00000218358	RAET1K	-52444	L6	1,54E+07	0.000626151012891344
cg23939096	ENSG00000049656	CLPTM1L	-210576	L8	3,60E+06	0.000257826887661142	cg04242070	ENSG00000171421	MRPL36	49397	R1	1,55E+07	0.000883977900552486
cg26129110	ENSG00000124279	FASTKD3	239287	R4	3,86E+06	0.000184162062615101	cg13185005	ENSG00000177963	RIC8A	-53836	L5	1,55E+07	0.000368324125230203
cg20558091	ENSG00000141577	CEP131	41093	R2	3,86E+06	0.000589318600368324	cg18291443	ENSG00000174891	RSRC1	645508	R6	1,55E+07	0.000294659300184162
cg02577773	ENSG00000118263	KLF7	0	L1	4,15E+06	0.000847145488029466	cg25446789	ENSG00000084731	KIF3C	339072	R7	1,55E+07	0.000515653775322284
cg20558091	ENSG00000167302	TEPSIN	79777	R4	4,15E+06	0.000147329650092081	cg03676624	ENSG00000149269	PAK1	-128005	L4	1,66E+07	0.000257826887661142
cg17222829	ENSG00000131626	PPFIA1	-201875	L5	4,15E+06	7,37E+09	cg11872672	ENSG00000146918	NCAPG2	909271	R10	1,66E+07	0.000294659300184162
cg01850245	ENSG00000278828	HIST1H3H	208747	R8	4,29E+06	0.000147329650092081	cg15877233	ENSG00000224116	INHBA-AS1	1121825	R5	1,69E+07	0.000994475138121547
cg17284116	ENSG00000121621	KIF18A	116271	R2	4,45E+05	0.000847145488029466	cg13760060	ENSG00000259015	AL442663.4	214724	R4	1,71E+07	0.000110497237569061
cg13299324	ENSG00000237609	AF064858.2	254509	R9	4,58E+06	0.000294659300184162	cg01886524	ENSG00000175792	RUVBL1	-353066	L6	1,78E+07	0.000478821362799263
cg15735030	ENSG00000111700	SLCO1B3	811048	R8	4,73E+06	0.000294659300184162	cg22881265	ENSG00000240682	ISY1	45124	R3	1,78E+07	0.000368324125230203
cg00576086	ENSG00000049656	CLPTM1L	48799	R2	4,78E+06	0.000368324125230203	cg11838237	ENSG00000145494	NDUFS6	48649	R2	1,78E+07	0.000110497237569061
cg03600432	ENSG00000110880	CORO1C	32660	R2	4,78E+06	0.000368324125230203	cg01715680	ENSG00000153485	TMEM251	-71941	L7	1,84E+07	7,37E+09
cg01425409	ENSG00000228952	LINC02041	-246674	L3	5,11E+06	0.000331491712707182	cg17208530	ENSG00000011260	UTP18	-434765	L7	1,84E+06	0.000589318600368324
cg06638383	ENSG00000186615	KTN1-AS1	-688355	L9	5,12E+06	0.000773480662983425	cg15148573	ENSG00000228393	LINC01004	1092194	R9	1,88E+07	0.000626151012891344
cg26685404	ENSG00000166851	PLK1	-255583	L7	5,13E+06	0.000220994475138122	cg02041108	ENSG00000145604	SKP2	205904	R5	1,90E+07	0.000478821362799263
cg02583620	ENSG00000129084	PSMA1	-433426	L8	5,50E+06	0.000220994475138122	cg27258696	ENSG00000109854	HTATIP2	-582029	L6	2,03E+07	0.000589318600368324
cg05521538	ENSG00000049656	CLPTM1L	58871	R2	5,50E+06	0.000368324125230203	cg13938959	ENSG00000188290	HES4	100157	R10	2,17E+06	0.000699815837937385
cg07972716	ENSG00000087586	AURKA	361733	R6	5,70E+06	0.000736648250460405	cg12185975	ENSG00000153310	FAM49B	-744320	L7	2,18E+07	0.000220994475138122
cg01810186	ENSG00000182197	EXT1	-934129	L9	6,33E+06	0.000883977900552486	cg01827426	ENSG00000171421	MRPL36	45830	R1	2,33E+07	0.000994475138121547
cg18305912	ENSG00000271134	AC012498.2	1120403	R10	6,44E+06	0.000257826887661142	cg08489623	ENSG00000146731	CCT6A	217156	R8	2,33E+07	0.000331491712707182
cg08421910	ENSG00000111863	ADTRP	0	L1	6,55E+06	7,37E+09	cg12163365	ENSG00000104472	CHRAC1	1057970	R8	2,49E+06	0.000441988950276243
cg24121967	ENSG00000162341	TPCN2	-159210	L4	6,78E+06	3,68E+09	cg05539265	ENSG00000175874	CREG2	-713474	L9	2,57E+07	0.000294659300184162
cg26767081	ENSG00000145014	TMEM44	15140	R2	6,79E+04	0.000478821362799263	cg08610901	ENSG00000242802	AP5Z1	1628481	R8	2,66E+07	0.000294659300184162
cg15890009	ENSG00000120647	CCDC77	170178	R4	7,28E+06	0.000257826887661142	cg02500075	ENSG00000121864	ZNF639	-206831	L9	2,66E+07	0.000515653775322284
cg11584519	ENSG00000173545	ZNF622	275759	R5	7,28E+06	7,37E+09	cg16337763	ENSG00000050165	DKK3	546443	R8	2,66E+07	0.000110497237569061
cg01987353	ENSG00000172156	CCL11	43045	R2	7,42E+06	0.000699815837937385	cg01963702	ENSG00000255400	AC124276.2	-78007	L3	2,70E+07	0.000699815837937385
cg11691844	ENSG00000171204	TMEM126B	-113021	L5	8,66E+06	3,68E+09	cg08983279	ENSG00000171241	SHCBP1	-8242	L3	2,84E+07	0.000847145488029466
cg03676624	ENSG00000219529	AP000580.1	210676	R7	8,73E+06	0.000147329650092081	cg13760060	ENSG00000119599	DCAF4	180461	R2	2,84E+07	0.000110497237569061
cg08913523	ENSG00000156831	NSMCE2	-270442	L3	8,97E+06	0.000147329650092081	cg25204172	ENSG00000126226	PCID2	57472	R6	2,84E+07	3,68E+09

cg08515891	ENSG00000279692	AC110285.7	-95297	L6	3,02E+07	7,37E+09	cg06237220	ENSG00000110104	CCDC86	127067	R7	7,58E+07	0.000810313075506446
cg16861964	ENSG00000235162	C12orf75	630535	R8	3,04E+07	0.000257826887661142	cg26647771	ENSG00000113387	SUB1	-164209	L3	7,58E+07	0.000920810313075506
cg17238677	ENSG00000160688	FLAD1	219589	R10	3,04E+07	0.000147329650092081	cg15810386	ENSG00000113360	DROSHA	38111	R1	7,82E+06	7,37E+09
cg18950108	ENSG00000213780	GTF2H4	-38287	L4	3,13E+07	0.000294659300184162	cg10800346	ENSG00000130177	CDC16	170864	R2	8,08E+07	7,37E+09
cg12056696	ENSG00000165501	LRR1	152223	R6	3,25E+07	0.000478821362799263	cg01425409	ENSG00000239093	RF01210	-272705	L4	8,23E+07	0.000184162062615101
cg23939096	ENSG00000145494	NDUFS6	245722	R9	3,25E+07	0.000220994475138122	cg07865418	ENSG00000272071	AC122710.2	368161	R9	8,51E+07	0.000147329650092081
**Probe**	**GeneID**	**Symbol**	**Distance**	**Sides**	**Raw.p**	**Pe**	**Probe**	**GeneID**	**Symbol**	**Distance**	**Sides**	**Raw.p**	**Pe**
cg26039867	ENSG00000229043	AC091729.3	-98314	L3	3,35E+07	7,37E+09	cg06638935	ENSG00000229043	AC091729.3	-95300	L3	8,59E+07	0.000368324125230203
cg09763439	ENSG00000168994	PXDC1	249466	R3	3,35E+07	0.000184162062615101	cg13587665	ENSG00000132394	EEFSEC	-26754	L1	8,61E+06	0.000405156537753223
cg01140416	ENSG00000229043	AC091729.3	-88725	L3	3,46E+07	7,37E+09	cg05644602	ENSG00000115350	POLE4	390431	R10	8,61E+07	0.000184162062615101
cg00522451	ENSG00000115008	IL1A	67442	R2	3,47E+07	0.000736648250460405	cg26767081	ENSG00000133657	ATP13A3	-74166	L5	8,61E+07	0.000515653775322284
cg16391242	ENSG00000146535	GNA12	-350756	L7	3,47E+07	0.000736648250460405	cg06436185	ENSG00000106615	RHEB	-225144	L2	8,89E+07	0.000220994475138122
cg27122888	ENSG00000162302	RPS6KA4	-239898	L9	3,47E+07	0.000405156537753223	cg12179884	ENSG00000109685	NSD2	-82068	L4	9,18E+07	0.000257826887661142
cg01850245	ENSG00000196747	HIST1H2AI	206882	R7	3,63E+07	0.000405156537753223	cg06609496	ENSG00000250584	LINC01511	189269	R6	9,22E+07	0.000552486187845304
cg15999077	ENSG00000250584	LINC01511	-27340	L3	3,66E+07	0.000184162062615101	cg11203211	ENSG00000185730	ZNF696	70250	R5	9,77E+07	0.000441988950276243
cg07470207	ENSG00000270137	AF230666.2	1009678	R9	3,67E+07	0.000405156537753223	cg03532013	ENSG00000105722	ERF	158736	R9	9,78E+07	0.000441988950276243
cg07524873	ENSG00000162129	CLPB	-159621	L8	3,71E+07	7,37E+09	cg04242070	ENSG00000145494	NDUFS6	52412	R2	9,78E+07	0.000331491712707182
cg24482747	ENSG00000139726	DENR	-60168	L3	3,71E+07	0.000589318600368324	cg15910070	ENSG00000137693	YAP1	299237	R7	9,78E+07	0.000552486187845304
cg16906187	ENSG00000215417	MIR17HG	-40574	L2	3,80E+07	0.000662983425414365	cg09032499	ENSG00000213780	GTF2H4	-111821	L9	1,04E+08	0.000589318600368324
cg15210851	ENSG00000181788	SIAH2	221772	R7	3,83E+07	0.000368324125230203	cg00119127	ENSG00000229043	AC091729.3	-218095	L4	1,04E+08	0.000478821362799263
cg13242871	ENSG00000124228	DDX27	557284	R8	3,96E+07	0.000552486187845304	cg10308673	ENSG00000177963	RIC8A	-53862	L5	1,04E+08	0.000957642725598527
cg13958583	ENSG00000164924	YWHAZ	154684	R6	3,96E+07	0.000220994475138122	cg26828017	ENSG00000140743	AC092338.1	0	L1	1,11E+08	0.000147329650092081
cg23725924	ENSG00000151657	KIN	29146	R1	4,22E+07	0.000699815837937385	cg05217983	ENSG00000196284	SUPT3H	-61176	L3	1,11E+08	0.000589318600368324
cg11020333	ENSG00000136295	TTYH3	-157418	L3	4,23E+07	0.000405156537753223	cg11075718	ENSG00000120533	ENY2	-343000	L6	1,11E+08	0.000662983425414365
cg16046375	ENSG00000229373	LINC00452	-192031	L4	4,29E+07	0.000441988950276243	cg24074153	ENSG00000257900	AL162632.1	-1714431	L9	1,12E+08	0.000331491712707182
cg12014181	ENSG00000107937	GTPBP4	304893	R5	4,51E+07	0.000478821362799263	cg13967811	ENSG00000246859	STARD4-AS1	-608874	L10	1,18E+08	3,68E+09
cg12808565	ENSG00000107937	GTPBP4	305113	R5	4,51E+07	0.000478821362799263	cg11203211	ENSG00000181638	ZFP41	27395	R1	1,18E+08	0.000257826887661142
cg03376308	ENSG00000229672	AL450322.2	295446	R2	4,63E+07	0.000147329650092081	cg17329859	ENSG00000141560	FN3KRP	-345179	L11	1,18E+08	0.000147329650092081
cg09883650	ENSG00000229720	AL109924.2	-61846	L2	4,71E+07	0.000220994475138122	cg24996491	ENSG00000146918	NCAPG2	888705	R9	1,18E+08	0.000810313075506446
cg26426186	ENSG00000169427	KCNK9	0	L1	4,81E+07	0.000441988950276243	cg21127079	ENSG00000150750	C11orf53	0	L1	1,22E+08	3,68E+09
cg11808936	ENSG00000170801	HTRA3	377276	R8	4,82E+07	0.000515653775322284	cg09220563	ENSG00000197045	GMFB	679347	R9	1,22E+08	0.000405156537753223
cg13171679	ENSG00000147536	GINS4	128101	R4	5,13E+07	0.000810313075506446	cg16233797	ENSG00000132031	MATN3	190321	R6	1,34E+08	0.000699815837937385
cg14279416	ENSG00000137074	APTX	183571	R6	5,14E+06	0.000184162062615101	cg03951877	ENSG00000145990	GFOD1	237810	R5	1,34E+08	0.000294659300184162
cg01974549	ENSG00000115368	WDR75	154904	R1	5,14E+07	0.000957642725598527	cg14928510	ENSG00000167634	NLRP7	0	L1	1,45E+08	0.000294659300184162
cg02657441	ENSG00000101391	CDK5RAP1	116209	R5	5,14E+07	7,37E+09	cg06576557	ENSG00000149609	C20orf144	421382	R10	1,46E+08	0.000847145488029466
cg26233084	ENSG00000173545	ZNF622	941368	R10	5,14E+07	0.000110497237569061	cg22776912	ENSG00000259518	LINC01583	750144	R10	1,48E+08	0.000405156537753223
cg23982574	ENSG00000061936	SFSWAP	21193	L1	5,49E+07	0.000810313075506446	cg07780377	ENSG00000118369	USP35	-13211	L2	1,52E+08	0.000957642725598527
cg22626525	ENSG00000158747	NBL1	-23950	L3	5,49E+07	3,68E+09	cg03252770	ENSG00000137462	TLR2	250866	R4	1,52E+08	3,68E+09
cg05136737	ENSG00000121644	DESI2	-52582	L1	5,86E+07	0.000368324125230203	cg01149929	ENSG00000173545	ZNF622	700790	R9	1,52E+08	0.000294659300184162
cg16104371	ENSG00000163950	SLBP	155931	R4	5,86E+07	0.000810313075506446	cg06923967	ENSG00000260714	AC133552.1	-76177	L7	1,52E+08	3,68E+09
cg26181731	ENSG00000170044	ZPLD1	-1879790	L10	5,96E+07	0.000294659300184162	cg15972148	ENSG00000272695	GAS6-DT	-248580	L8	1,61E+08	0.000147329650092081
cg04821520	ENSG00000255477	AC021713.1	994834	R5	6,21E+07	0.000257826887661142	cg02704181	ENSG00000173545	ZNF622	879058	R10	1,62E+08	0.000331491712707182
cg04097334	ENSG00000204262	COL5A2	296958	R4	6,25E+07	0.000957642725598527	cg11041239	ENSG00000066855	MTFR1	8230	R1	1,62E+08	0.000773480662983425
cg16750953	ENSG00000250584	LINC01511	71807	R3	6,30E+07	0.000368324125230203	cg23856611	ENSG00000228499	TMSB10P1	381155	R3	1,65E+08	0.000441988950276243
cg22943388	ENSG00000061936	SFSWAP	118994	R8	6,45E+07	0.000883977900552486	cg13780718	ENSG00000064601	CTSA	5013	R3	1,72E+08	0.000441988950276243
cg02500075	ENSG00000260743	AC007823.1	-203527	L8	6,78E+07	3,68E+09	cg17316862	ENSG00000134001	EIF2S1	-170707	L5	1,72E+08	0.000478821362799263
cg01203651	ENSG00000272218	AC108865.2	51448	R1	6,80E+07	0.000147329650092081	cg26039867	ENSG00000178381	ZFAND2A	-102822	L4	1,77E+08	0.000478821362799263
cg03222523	ENSG00000166961	MS4A15	-23370	L2	6,84E+07	0.000773480662983425	cg19044550	ENSG00000255329	H2AFZP4	-182646	L10	1,81E+08	0.000220994475138122
cg05006473	ENSG00000163950	SLBP	189975	R4	7,11E+07	0.000883977900552486	cg16287451	ENSG00000170619	COMMD5	129478	R7	1,83E+08	0.000810313075506446
cg12179884	ENSG00000013810	TACC3	-319099	L8	7,11E+07	0.000662983425414365	cg10120897	ENSG00000172530	BANP	-354405	L10	1,95E+08	0.000441988950276243
cg15898536	ENSG00000145494	NDUFS6	48212	R2	7,11E+07	0.000331491712707182	cg02524834	ENSG00000124641	MED20	-45887	L3	1,95E+08	0.000184162062615101
cg25667997	ENSG00000198169	ZNF251	187803	R6	7,57E+07	0.000294659300184162	cg08197072	ENSG00000103275	UBE2I	42798	R6	1,95E+08	0.000515653775322284

cg18052274	ENSG00000185238	PRMT3	671944	R8	1,95E+08	0.000515653775322284	cg07364729	ENSG00000126216	TUBGCP3	352711	R5	4,69E+08	0.000184162062615101
cg25616055	ENSG00000139318	DUSP6	102120	R3	1,95E+08	0.000515653775322284	cg20660802	ENSG00000250020	AC113430.1	3270	L1	4,83E+08	0.000478821362799263
cg04438395	ENSG00000277218	AL139123.1	-296919	L7	2,00E+08	7,37E+09	cg16456576	ENSG00000103248	MTHFSD	154529	R6	4,83E+07	0.000184162062615101
cg23224042	ENSG00000170619	COMMD5	129983	R8	2,14E+08	0.000883977900552486	cg26631454	ENSG00000126226	PCID2	-202638	L9	4,83E+08	0.000220994475138122
cg02452500	ENSG00000255558	AC013762.1	-5540	L1	2,31E+08	0.000147329650092081	cg07470207	ENSG00000223697	AF230666.1	1021616	R10	4,88E+08	0.000589318600368324
cg08294336	ENSG00000262094	AC139099.1	206045	R6	2,31E+08	0.000220994475138122	cg07986257	ENSG00000112273	HDGFL1	248548	R1	4,98E+08	0.000441988950276243
**Probe**	**GeneID**	**Symbol**	**Distance**	**Sides**	**Raw.p**	**Pe**	**Probe**	**GeneID**	**Symbol**	**Distance**	**Sides**	**Raw.p**	**Pe**
cg24373608	ENSG00000170044	ZPLD1	-1447311	L9	2,32E+08	0.000699815837937385	cg22112035	ENSG00000254604	AP002336.2	126057	R6	5,01E+08	0.000257826887661142
cg01827426	ENSG00000145494	NDUFS6	48845	R2	2,34E+08	0.000662983425414365	cg00594191	ENSG00000065665	SEC61A2	416531	R7	5,12E+08	0.000810313075506446
cg05409967	ENSG00000101654	RNMT	-63990	L2	2,34E+08	0.000478821362799263	cg21138935	ENSG00000147437	GNRH1	-176218	L5	5,23E+08	0.000662983425414365
cg12592179	ENSG00000129055	ANAPC13	275904	R8	2,34E+08	0.000736648250460405	cg15210851	ENSG00000214237	MINDY4B	351692	R10	5,28E+08	0.000184162062615101
cg15928188	ENSG00000164778	EN2	-18910	L2	2,40E+08	0.000110497237569061	cg02463513	ENSG00000152952	PLOD2	-2048	L1	5,44E+08	0.000994475138121547
cg01894985	ENSG00000206527	HACD2	-285278	L6	2,42E+08	0.000220994475138122	cg09461530	ENSG00000280239	AC011498.7	-118200	L10	5,73E+08	0.000368324125230203
cg03016496	ENSG00000122687	MRM2	579234	R7	2,49E+08	0.000736648250460405	cg04642300	ENSG00000183137	CEP57L1	127719	R4	5,77E+08	0.000994475138121547
cg09791621	ENSG00000276931	AC009041.4	-59709	L5	2,57E+08	0.000110497237569061	cg15148573	ENSG00000161057	PSMC2	-488723	L7	5,77E+08	0.000883977900552486
cg08913523	ENSG00000180938	ZNF572	-658173	L10	2,64E+08	0.000810313075506446	cg02463513	ENSG00000240032	LNCSRLR	-96516	L3	5,90E+08	0.000773480662983425
cg26283496	ENSG00000240032	LNCSRLR	-482153	L9	2,65E+08	0.000331491712707182	cg18131582	ENSG00000162704	ARPC5	-307387	L6	6,12E+08	0.000515653775322284
cg03611487	ENSG00000185238	PRMT3	671865	R8	2,65E+08	0.000699815837937385	cg17727795	ENSG00000249116	AC025183.1	-818556	L9	6,25E+08	0.000662983425414365
cg23024047	ENSG00000189229	AC069277.1	1026181	R4	2,66E+08	0.000184162062615101	cg16072535	ENSG00000273521	AL162274.1	1620598	R10	6,39E+08	0.000441988950276243
cg13372658	ENSG00000253892	AC022915.3	-212453	L4	2,69E+08	0.000368324125230203	cg25951217	ENSG00000014164	ZC3H3	47744	R4	6,49E+08	0.000957642725598527
cg21044933	ENSG00000100567	PSMA3	269407	R8	2,81E+08	0.000957642725598527	cg10243459	ENSG00000223872	AC006372.1	-287811	L7	6,65E+08	7,37E+09
cg21351392	ENSG00000272841	AL139393.2	-194112	L4	3,17E+08	0.000441988950276243	cg22112035	ENSG00000255143	AP000879.2	-6684	L2	6,80E+08	3,68E+09
cg06576557	ENSG00000101391	CDK5RAP1	117894	R5	3,18E+07	0.000184162062615101	cg09244312	ENSG00000237263	MAPK6P3	522409	R8	6,97E+08	0.000331491712707182
cg02688422	ENSG00000232615	AC026412.2	205012	R8	3,18E+07	0.000110497237569061	cg12091936	ENSG00000255329	H2AFZP4	-178131	L10	7,19E+07	0.000589318600368324
cg07206053	ENSG00000251138	AC090502.1	1754522	R13	3,31E+08	0.000478821362799263	cg15810386	ENSG00000082213	C5orf22	169901	R3	7,30E+08	0.000552486187845304
cg22255095	ENSG00000101181	MTG2	285752	R7	3,37E+08	0.000847145488029466	cg02757516	ENSG00000198546	ZNF511	200657	R10	7,30E+07	0.000184162062615101
cg14557064	ENSG00000145494	NDUFS6	48673	R2	3,38E+08	0.000957642725598527	cg03829734	ENSG00000173545	ZNF622	369366	R6	7,30E+08	0.000847145488029466
cg19761458	ENSG00000146648	EGFR	320435	R4	3,38E+08	0.000294659300184162	cg12844277	ENSG00000260046	AL162632.3	-1704201	L10	7,34E+08	0.000478821362799263
cg27009439	ENSG00000148773	MKI67	-1218192	L6	3,38E+08	0.000257826887661142	cg14541582	ENSG00000180104	EXOC3	-129422	L7	7,51E+08	0.000294659300184162
cg14651293	ENSG00000272853	AC069544.1	491516	R9	3,45E+08	0.000847145488029466	cg26927190	ENSG00000173918	C1QTNF1	143216	R7	7,51E+08	0.000220994475138122
cg18485530	ENSG00000139734	DIAPH3	692678	R4	3,58E+08	0.000220994475138122	cg20558091	ENSG00000260005	AC027601.1	80207	R5	7,92E+08	0.000994475138121547
cg05197133	ENSG00000101407	TTI1	-343320	L10	3,59E+08	0.000883977900552486	cg23441248	ENSG00000275580	AC022306.2	-504097	L9	7,99E+08	0.000589318600368324
cg07213771	ENSG00000112837	TBX18	68962	R1	3,69E+08	0.000184162062615101	cg01432552	ENSG00000153233	PTPRR	0	L1	8,16E+08	0.000331491712707182
cg26608486	ENSG00000172530	BANP	-338614	L10	3,80E+08	0.000626151012891344	cg11549874	ENSG00000100029	PES1	-199407	L7	8,20E+08	0.000626151012891344
cg01520402	ENSG00000175040	CHST2	48213	R2	3,81E+08	0.000662983425414365	cg19099433	ENSG00000171865	RNASEH1	-142455	L8	8,44E+08	0.000331491712707182
cg25139084	ENSG00000163877	SNIP1	492755	R10	3,81E+08	0.000368324125230203	cg20660802	ENSG00000153404	PLEKHG4B	37223	R1	8,44E+08	0.000699815837937385
cg01058360	ENSG00000106615	RHEB	-225164	L2	3,81E+08	0.000773480662983425	cg25762854	ENSG00000147586	MRPS28	782426	R8	8,44E+08	3,68E+09
cg11808936	ENSG00000184985	SORCS2	-149639	L3	3,81E+08	0.000662983425414365	cg04102854	ENSG00000158525	CPA5	0	L1	8,51E+07	0.000368324125230203
cg04001071	ENSG00000136159	NUDT15	998374	R9	3,81E+08	0.000405156537753223	cg04546573	ENSG00000231739	GAPDHP59	-918021	L9	8,52E+08	0.000220994475138122
cg14380939	ENSG00000221829	FANCG	96307	R10	3,81E+08	0.000957642725598527	cg26812486	ENSG00000260022	AL031716.1	5000	R1	9,13E+08	0.000294659300184162
cg03041510	ENSG00000091136	LAMB1	0	L1	4,04E+08	0.000847145488029466	cg16209064	ENSG00000272218	AC108865.2	47113	R1	9,18E+08	0.000294659300184162
cg04352006	ENSG00000101407	TTI1	293223	R3	4,04E+08	0.000994475138121547	cg16153549	ENSG00000171865	RNASEH1	48378	R5	9,20E+08	0.000331491712707182
cg16009120	ENSG00000166532	RIMKLB	147487	R10	4,04E+08	0.000626151012891344	cg23441248	ENSG00000156958	GALK2	-480482	L6	9,20E+08	0.000552486187845304
cg02412169	ENSG00000249326	AC025183.2	154701	R8	4,05E+07	0.000441988950276243	cg09853702	ENSG00000258116	PPIAP45	70165	R1	9,25E+08	0.000847145488029466
cg22112035	ENSG00000255329	H2AFZP4	122611	R5	4,13E+08	0.000478821362799263	cg01715680	ENSG00000170270	GON7	-51965	L6	9,47E+08	7,37E+09
cg00108282	ENSG00000164818	DNAAF5	-18934	L2	4,29E+08	0.000184162062615101	cg16960046	ENSG00000146648	EGFR	33317	L1	9,75E+08	0.000883977900552486
cg08515891	ENSG00000185298	CCDC137	174293	R10	4,29E+08	0.000847145488029466	cg00936309	ENSG00000232940	HCG25	75485	R10	9,79E+08	0.000920810313075506
cg04476531	ENSG00000273998	AL049794.1	1095907	R5	4,35E+08	0.000736648250460405	cg11419030	ENSG00000250020	AC113430.1	3188	L1	9,85E+08	0.000773480662983425
cg02995493	ENSG00000224570	AC097063.1	-267154	L4	4,44E+08	0.000515653775322284	cg10365900	ENSG00000254885	AP001547.1	334592	R2	1,01E+09	0.000626151012891344
cg02197005	ENSG00000272218	AC108865.2	-26473	L2	4,56E+07	0.000257826887661142	cg06120359	ENSG00000244791	AC087667.1	232014	R2	1,02E+09	0.000957642725598527
cg04214938	ENSG00000229867	STEAP3-AS1	505825	R7	4,61E+08	0.000368324125230203	cg11177858	ENSG00000156411	ATP5MPL	-4416	L2	1,03E+09	0.000957642725598527
cg08950105	ENSG00000269946	MIRLET7A1HG	306211	R6	4,63E+08	0.000110497237569061	cg16861964	ENSG00000257642	AC011313.1	700112	R9	1,05E+09	0.000257826887661142

cg18708402	ENSG00000201736	RNA5SP160	-350893	L8	1,09E+09	0.000699815837937385	cg08950105	ENSG00000230262	LINC02603	316513	R9	2,90E+09	0.000147329650092081
cg12051819	ENSG00000118655	DCLRE1B	-260929	L6	1,09E+09	0.000626151012891344	cg00594191	ENSG00000272507	RNU6-88P	403174	R6	2,92E+09	0.000257826887661142
cg16769791	ENSG00000253898	LINC01419	-856894	L5	1,11E+09	0.000441988950276243	cg01425409	ENSG00000224049	AC108681.1	100060	R3	2,97E+09	0.000478821362799263
cg12445832	ENSG00000237491	AL669831.5	-88854	L9	1,12E+08	0.000110497237569061	cg09763439	ENSG00000270504	AL391422.4	277963	R4	2,98E+09	0.000847145488029466
cg22338152	ENSG00000268713	AC005261.3	130577	R9	1,12E+09	0.000662983425414365	cg08364970	ENSG00000158545	ZC3H18	160643	R4	2,98E+09	0.000773480662983425
cg26207568	ENSG00000039139	DNAH5	-229120	L2	1,19E+09	0.000441988950276243	cg12117986	ENSG00000099246	RAB18	-145210	L2	2,98E+09	0.000441988950276243
**Probe**	**GeneID**	**Symbol**	**Distance**	**Sides**	**Raw.p**	**Pe**	**Probe**	**GeneID**	**Symbol**	**Distance**	**Sides**	**Raw.p**	**Pe**
cg11419030	ENSG00000153404	PLEKHG4B	37141	R1	1,19E+09	0.000736648250460405	cg22008551	ENSG00000176182	MYPOP	-47117	L3	3,15E+09	0.000478821362799263
cg07314530	ENSG00000164818	DNAAF5	564006	R13	1,23E+09	0.000626151012891344	cg14939821	ENSG00000235180	LINC00601	-727086	L6	3,36E+09	0.000736648250460405
cg26236329	ENSG00000166793	YPEL4	212233	R14	1,26E+09	0.000184162062615101	cg03554573	ENSG00000150750	C11orf53	0	L1	3,43E+09	0.000810313075506446
cg02069592	ENSG00000050130	JKAMP	752949	R9	1,30E+09	0.000736648250460405	cg05705018	ENSG00000254648	AP000911.2	673228	R9	3,48E+09	0.000478821362799263
cg06704631	ENSG00000214367	HAUS3	-64106	L3	1,30E+09	0.000257826887661142	cg13797205	ENSG00000224049	AC108681.1	122177	R4	3,48E+09	0.000552486187845304
cg06902828	ENSG00000155100	OTUD6B	-548464	L7	1,30E+09	0.000883977900552486	cg10937802	ENSG00000091490	SEL1L3	-311715	L5	3,51E+09	0.000920810313075506
cg23319289	ENSG00000242163	AL121769.1	-492490	L10	1,34E+09	0.000184162062615101	cg04829509	ENSG00000137869	CYP19A1	439409	R8	3,68E+08	0.000773480662983425
cg07633317	ENSG00000261751	AC007603.1	222016	R6	1,35E+09	3,68E+09	cg21531389	ENSG00000128203	ASPHD2	-165518	L9	3,70E+09	0.000478821362799263
cg18617860	ENSG00000125863	MKKS	179133	R5	1,37E+09	0.000626151012891344	cg06657721	ENSG00000112308	C6orf62	-307391	L9	3,70E+09	0.000847145488029466
cg03174644	ENSG00000110696	C11orf58	1168739	R10	1,37E+09	0.000441988950276243	cg08436756	ENSG00000172893	DHCR7	493178	R2	3,70E+09	0.000699815837937385
cg07543138	ENSG00000180530	NRIP1	0	L1	1,45E+09	0.000110497237569061	cg08426157	ENSG00000228675	AC006482.1	-1073891	L9	3,70E+08	0.000257826887661142
cg17473656	ENSG00000109536	FRG1	575078	R7	1,45E+09	0.000515653775322284	cg08515891	ENSG00000182446	NPLOC4	64443	R5	3,91E+09	0.000368324125230203
cg09020697	ENSG00000103248	MTHFSD	74822	R4	1,49E+09	0.000589318600368324	cg06884470	ENSG00000165934	CPSF2	-147434	L4	4,12E+08	0.000478821362799263
cg00019654	ENSG00000242163	AL121769.1	-348583	L7	1,52E+09	0.000184162062615101	cg03535253	ENSG00000100605	ITPK1	-123904	L9	4,23E+07	0.000294659300184162
cg07543138	ENSG00000235609	AF127577.4	-47315	L3	1,53E+09	7,37E+09	cg03821121	ENSG00000254486	LINC02547	-64955	L4	4,37E+09	0.000957642725598527
cg07895124	ENSG00000005194	CIAPIN1	-117743	L7	1,54E+09	0.000920810313075506	cg13938959	ENSG00000237491	AL669831.5	-88742	L9	4,45E+09	0.000405156537753223
cg02565878	ENSG00000273010	AL360270.3	210177	R7	1,61E+09	0.000220994475138122	cg06915667	ENSG00000277250	RF00017	119799	R3	4,57E+09	0.000368324125230203
cg18847904	ENSG00000232615	AC026412.2	-162728	L5	1,62E+09	0.000662983425414365	cg00650953	ENSG00000170279	C7orf33	787353	R8	4,61E+09	0.000405156537753223
cg24121967	ENSG00000265539	MIR3164	-238392	L6	1,63E+09	0.000368324125230203	cg06098693	ENSG00000255143	AP000879.2	-6553	L2	4,78E+09	0.000147329650092081
cg09729182	ENSG00000138496	PARP9	281841	R10	1,72E+09	0.000478821362799263	cg03857198	ENSG00000244268	AC117394.2	-48032	L3	5,10E+09	0.000883977900552486
cg11969493	ENSG00000124172	ATP5F1E	-576878	L10	1,72E+09	0.000589318600368324	cg02500075	ENSG00000114450	GNB4	-93938	L4	5,10E+09	0.000220994475138122
cg01759218	ENSG00000258301	VASH1-AS1	432537	R9	1,79E+09	0.000552486187845304	cg21787848	ENSG00000221611	RF00604	-338402	L10	5,34E+09	0.000331491712707182
cg16099687	ENSG00000111145	ELK3	-176356	L6	1,82E+09	0.000220994475138122	cg12445832	ENSG00000230092	AL669831.4	-81202	L8	5,37E+09	0.000184162062615101
cg02484673	ENSG00000276337	AC105429.1	-166736	L5	1,85E+09	0.000699815837937385	cg01397507	ENSG00000254495	AP000487.2	-235612	L7	5,61E+09	0.000368324125230203
cg07226484	ENSG00000271806	AL590822.2	99745	R2	1,85E+09	0.000147329650092081	cg04603130	ENSG00000099800	TIMM13	-122442	L7	5,67E+09	0.000920810313075506
cg02440396	ENSG00000154957	ZNF18	-465752	L8	1,87E+09	0.000368324125230203	cg19417168	ENSG00000142002	DPP9	143273	R8	5,67E+09	0.000773480662983425
cg05506581	ENSG00000132964	CDK8	-93125	L2	1,87E+09	0.000331491712707182	cg19509829	ENSG00000111229	ARPC3	93961	R5	5,82E+09	0.000736648250460405
cg10907912	ENSG00000081320	STK17B	0	L1	1,92E+09	0.000110497237569061	cg22171607	ENSG00000116288	PARK7	-91998	L4	6,63E+09	0.000920810313075506
cg04829509	ENSG00000259701	AC020891.3	510279	R10	1,94E+08	0.000331491712707182	cg19738281	ENSG00000266910	AC008507.1	-410595	L3	6,91E+08	0.000478821362799263
cg21814238	ENSG00000137478	FCHSD2	204314	R10	2,03E+09	0.000626151012891344	cg09835220	ENSG00000202337	RNU6-8	-1348970	L9	7,28E+09	0.000957642725598527
cg13455326	ENSG00000267387	AC020931.1	156365	R7	2,07E+09	0.000589318600368324	cg12904737	ENSG00000164778	EN2	-18989	L2	7,33E+09	0.000920810313075506
cg04001071	ENSG00000227848	SUCLA2-AS1	962037	R8	2,12E+09	0.000810313075506446	cg12512771	ENSG00000048052	HDAC9	396544	R6	7,35E+09	0.000736648250460405
cg13016851	ENSG00000129103	SUMF2	-147028	L7	2,15E+09	0.000847145488029466	cg26685404	ENSG00000166847	DCTN5	-268478	L9	7,35E+09	0.000515653775322284
cg25667997	ENSG00000160959	LRRC14	-7929	L4	2,15E+09	0.000847145488029466	cg14174502	ENSG00000215869	AC092506.1	92244	R1	7,37E+09	0.000220994475138122
cg25414370	ENSG00000273192	AL671710.1	-140345	L8	2,18E+09	0.000662983425414365	cg10599446	ENSG00000238077	NMNAT1P3	810877	R8	7,38E+09	0.000994475138121547
cg04214938	ENSG00000163064	EN1	103574	R1	2,20E+09	0.000920810313075506	cg25702651	ENSG00000180611	MB21D2	-39869	L1	7,74E+09	0.000810313075506446
cg13016851	ENSG00000223559	AC073136.1	60529	R1	2,22E+09	0.000294659300184162	cg17718728	ENSG00000165832	TRUB1	-1159953	L5	8,15E+09	0.000773480662983425
cg18561247	ENSG00000227598	Z94721.1	184573	R4	2,23E+09	7,37E+09	cg02939767	ENSG00000125826	RBCK1	-173162	L5	8,36E+09	0.000515653775322284
cg05255994	ENSG00000184432	COPB2	45005	R2	2,27E+09	0.000847145488029466	cg26873457	ENSG00000225140	AL358216.1	-52008	L4	8,67E+09	0.000552486187845304
cg22092126	ENSG00000115514	TXNDC9	-413858	L5	2,27E+09	0.000920810313075506	cg04854908	ENSG00000215244	LINC02649	-563751	L9	8,86E+09	0.000957642725598527
cg13431678	ENSG00000187713	TMEM203	-28921	L6	2,40E+09	0.000810313075506446	cg07543138	ENSG00000231201	AF127577.2	-68891	L4	9,93E+09	7,37E+09
cg24609819	ENSG00000204147	ASAH2B	-264121	L7	2,46E+08	0.000147329650092081	cg01549525	ENSG00000162783	IER5	-959761	L10	0.000102521204139582	0.000994475138121547
cg06098693	ENSG00000254604	AP002336.2	126188	R6	2,49E+09	0.000810313075506446	cg21031035	ENSG00000236053	LINC01067	-315666	L3	0.000103653108872098	0.000810313075506446
cg16209064	ENSG00000083857	FAT1	-118714	L2	2,53E+09	0.000920810313075506	cg03854025	ENSG00000248641	HMGA1P2	211450	R4	0.000107691688593679	0.000773480662983425
cg22927134	ENSG00000259287	AC010809.1	-192225	L6	2,77E+09	0.000552486187845304	cg10453337	ENSG00000124275	MTRR	349283	R3	0.000110567373338784	0.000626151012891344

cg14424376	ENSG00000213761	MT1P1	-337586	L10	0.000123614704417571	0.000589318600368324	cg06931356	ENSG00000181211	HECW1-IT1	-373104	L7	0.000353785316637978	0.000331491712707182
cg25888386	ENSG00000165806	CASP7	297032	R4	0.000128604654744713	0.000699815837937385	cg19486070	ENSG00000234977	AC074389.3	105387	R4	0.00035613978880898	0.000552486187845304
cg08197072	ENSG00000274751	AC120498.9	39465	R5	0.000130551700428106	0.000368324125230203	cg20219053	ENSG00000255014	ARL6IP1P3	-993262	L5	0.000365970206784768	0.000331491712707182
cg09756014	ENSG00000268218	AC137932.3	182144	R10	0.000131115840434574	0.000589318600368324	cg06098693	ENSG00000255539	AP002336.3	168692	R9	0.000449814470930703	0.000626151012891344
cg19044550	ENSG00000254495	AP000487.2	-103266	L6	0.000136230874739866	0.000699815837937385	cg19274612	ENSG00000215869	AC092506.1	-1857040	L9	0.000449978636456072	0.000920810313075506
cg01819142	ENSG00000253915	MAPRE1P1	-2426082	L9	0.000143727788210021	0.000220994475138122	cg17222829	ENSG00000255539	AP002336.3	-259978	L8	0.000461470814349652	0.000662983425414365
**Probe**	**GeneID**	**Symbol**	**Distance**	**Sides**	**Raw.p**	**Pe**	**Probe**	**GeneID**	**Symbol**	**Distance**	**Sides**	**Raw.p**	**Pe**
cg23533270	ENSG00000213513	IMPDH1P5	904540	R10	0.000144957588415458	0.000920810313075506	cg21531389	ENSG00000206028	Z99774.1	57157	R5	0.000468527775746429	0.000552486187845304
cg01203651	ENSG00000250971	AC110772.2	164840	R4	0.000146850829718519	0.000147329650092081	cg10200451	ENSG00000253720	AC022973.2	598818	R10	0.000472688687809902	0.000552486187845304
cg01203651	ENSG00000249742	AC110772.1	138386	R3	0.000152184860490486	0.000331491712707182	cg11154040	ENSG00000184160	ADRA2C	-24809	L1	0.000478888369433237	0.000883977900552486
cg06884470	ENSG00000100605	ITPK1	617332	R7	0.000153024865495966	0.000515653775322284	cg27640794	ENSG00000207171	RF00432	-163008	L4	0.000481174519695915	0.000994475138121547
cg04352006	ENSG00000276603	AL109614.1	-162721	L8	0.000154006517319066	0.000957642725598527	cg22795489	ENSG00000235264	RPL5P28	-98195	L4	0.000491722595799864	0.000220994475138122
cg01467592	ENSG00000232600	TONSL-AS1	13700	R3	0.000155448536353965	0.000184162062615101	cg03961010	ENSG00000238280	AC067751.1	44923	R1	0.000497382231053593	0.000257826887661142
cg09729182	ENSG00000114030	KPNA1	175819	R8	0.000156874741841032	0.000478821362799263	cg13760060	ENSG00000259079	AC005476.1	-1415130	L10	0.000518929391652815	0.000515653775322284
cg06755296	ENSG00000259478	AC024651.1	325812	R3	0.000161947405040972	0.000810313075506446	cg20670679	ENSG00000222012	AC005481.1	-150328	L3	0.000530774808905193	0.000957642725598527
cg25865108	ENSG00000277769	RBM22P4	-492106	L7	0.000167703043672953	0.000662983425414365	cg14928510	ENSG00000267149	AC011476.2	89520	R6	0.000602254368439091	0.000920810313075506
cg24833674	ENSG00000231605	LINC01363	230961	R7	0.00016946992007246	0.000294659300184162	cg16527108	ENSG00000179010	MRFAP1	528940	R7	0.000643324999836037	0.000920810313075506
cg21742001	ENSG00000274023	AL360169.2	-116938	L5	0.000171121133438628	0.000662983425414365	cg10599446	ENSG00000236182	AC009319.1	-365749	L3	0.000644331495352915	0.000736648250460405
cg14852736	ENSG00000279839	AL512383.1	116221	R2	0.000172347926910214	3,68E+09	cg20495476	ENSG00000128254	C22orf24	-164991	L7	0.000656539322328059	0.000662983425414365
cg05968369	ENSG00000227589	AL136528.1	255054	R9	0.000180841574555042	0.000883977900552486	cg01185573	ENSG00000214288	HMGB3P13	-1275497	L10	0.000674755811876291	0.000699815837937385
cg14380939	ENSG00000165280	VCP	78536	R9	0.000181788391368056	0.000552486187845304	cg26150981	ENSG00000212303	RNU6-1154P	-258236	L6	0.000712742713545486	0.000478821362799263
cg01715680	ENSG00000100605	ITPK1	-142710	L10	0.00019088372026597	0.000662983425414365	cg19255307	ENSG00000248844	AP003555.1	277187	R6	0.000715024609281881	0.000405156537753223
cg07068735	ENSG00000185088	RPS27L	-54650	L2	0.00019088372026597	0.000883977900552486	cg13996593	ENSG00000231534	FBXO36-IT1	232846	R5	0.000719019047482012	0.000184162062615101
cg04438395	ENSG00000277959	AL162274.2	1569282	R9	0.0001923668956915	0.000736648250460405	cg26236329	ENSG00000222998	RN7SKP259	18836	R2	0.000719019047482012	0.000147329650092081
cg20236089	ENSG00000008083	JARID2	-106888	L3	0.000195561776019318	0.000662983425414365	cg13332832	ENSG00000166917	MIR202HG	418919	R10	0.000726738728277778	0.000626151012891344
cg08069287	ENSG00000256568	AP002761.2	18904	R1	0.00019566138738648	0.000883977900552486	cg02444118	ENSG00000174418	RPL35P9	-276432	L7	0.000757029713119084	0.000147329650092081
cg09814633	ENSG00000215057	PKMP5	3126	L1	0.000196091987847198	0.000441988950276243	cg19904347	ENSG00000269148	AC092301.1	-106662	L7	0.000777969721538852	0.000478821362799263
cg23703079	ENSG00000257452	AC004551.1	329037	R2	0.000197065496169088	0.000699815837937385	cg22112035	ENSG00000255539	AP002336.3	168561	R9	0.000787656655664374	0.000847145488029466
cg10271186	ENSG00000162105	SHANK2	0	L1	0.000200402985711369	0.000662983425414365	cg00100440	ENSG00000257604	AC027288.2	1011471	R7	0.000810499925981451	0.000883977900552486
cg05939089	ENSG00000279636	AL132989.2	210849	R10	0.000205298483162315	0.000920810313075506	cg04762129	ENSG00000276404	MIR6835	102663	R4	0.000815267299332006	0.000957642725598527
cg07213771	ENSG00000228290	TBX18-AS1	69323	R2	0.000215355755941814	0.000220994475138122	cg00350503	ENSG00000267633	AC008686.1	269316	R3	0.000840560166136789	0.000883977900552486
cg22493372	ENSG00000232729	AC211433.1	471379	R8	0.000220079147233917	0.000184162062615101	cg18248586	ENSG00000243353	RPS29P19	292810	R4	0.000861752117886774	0.000920810313075506
cg16267322	ENSG00000100605	ITPK1	539182	R7	0.000226145385165521	0.000699815837937385	cg21368566	ENSG00000280744	LINC01173	-942504	L9	0.000871775871371535	0.000994475138121547
cg03612582	ENSG00000260690	CYCSP39	-961381	L9	0.000228548754705507	0.000626151012891344	cg00169897	ENSG00000261546	AC135782.3	80799	R2	0.000907744474201294	0.000405156537753223
cg18275228	ENSG00000213260	YWHAZP5	1105850	R5	0.000248751846672206	0.000589318600368324	cg00594191	ENSG00000251957	RNU6-1095P	210075	R4	0.00104302088067459	0.000515653775322284
cg11728664	ENSG00000172689	MS4A10	267168	R5	0.000254901971623395	0.000184162062615101	cg03329576	ENSG00000255710	AP003170.1	-494437	L9	0.00109115367003289	0.000810313075506446
cg27022558	ENSG00000168065	SLC22A11	182055	R6	0.000260659311762999	0.000957642725598527	cg20670679	ENSG00000233191	AC006372.2	-250435	L6	0.00112527393476913	0.000478821362799263
cg04922015	ENSG00000269826	AC092327.2	-117211	L4	0.000273529778642996	0.000810313075506446	cg06735285	ENSG00000276505	AP000892.2	-485816	L8	0.00135427410623377	0.000883977900552486
cg02006405	ENSG00000216360	AL136968.1	-236550	L6	0.000284002265243943	0.000810313075506446	cg06704631	ENSG00000063978	RNF4	155947	R5	0.00140344283591304	0.000810313075506446
cg07783480	ENSG00000214178	PPIAP23	-1547994	L9	0.000287922362973598	0.000920810313075506	cg00962147	ENSG00000260379	AC074052.1	1608225	R10	0.00141537896405366	0.000810313075506446
cg15160746	ENSG00000256902	IQSEC3P1	118522	R6	0.000293361703007076	0.000147329650092081	cg09813301	ENSG00000230428	AL049542.1	90220	R8	0.00144038617722156	0.000110497237569061
cg12186816	ENSG00000176623	RMDN1	-634234	L10	0.000301105992609205	0.000257826887661142	cg26039867	ENSG00000230444	TFAMP1	351006	R10	0.00144038617722156	0.000883977900552486
cg13587665	ENSG00000239608	RUVBL1-AS1	-356344	L4	0.00030249040697929	0.000515653775322284	cg00477086	ENSG00000222536	RNU2-39P	1163014	R5	0.00145642715096599	0.000994475138121547
cg08387270	ENSG00000232600	TONSL-AS1	14020	R3	0.000305351463883484	0.000847145488029466	cg01528542	ENSG00000258375	AC079363.1	470871	R6	0.00145642715096599	0.000883977900552486
cg11736739	ENSG00000234616	JRK	196952	R5	0.000308342317964836	0.000220994475138122	cg04233201	ENSG00000254194	AF279873.4	-70654	L2	0.00145642715096599	7,37E+09
cg26707845	ENSG00000251323	AP003086.1	-578502	L8	0.000316300182156774	0.000736648250460405	cg04322162	ENSG00000221502	MIR1245A	526900	R4	0.00145642715096599	0.000552486187845304
cg15210851	ENSG00000244265	SIAH2-AS1	242584	R8	0.000325584569795927	0.000368324125230203	cg20168964	ENSG00000256137	AC117373.1	-150620	L3	0.00145642715096599	0.000147329650092081
cg16209064	ENSG00000250971	AC110772.2	160505	R4	0.000328368904424021	0.000331491712707182	cg02450832	ENSG00000260737	LINC01227	292535	R3	0.0014610300034086	0.000184162062615101
cg16148956	ENSG00000271555	AC113139.1	-505069	L6	0.000331283844893242	0.000626151012891344	cg22304522	ENSG00000253656	AC090987.1	-246078	L3	0.00146149077082804	0.000294659300184162
cg09586743	ENSG00000254246	AC011377.1	1350102	R10	0.000346322820954852	0.000331491712707182	cg21177502	ENSG00000254025	BRIX1P1	1379582	R8	0.00150016324725953	0.000810313075506446
cg08609578	ENSG00000130023	ERMARD	323557	R7	0.000346608043744037	0.000920810313075506	cg11280082	ENSG00000238169	LINC01053	116314	R4	0.00150052304387745	0.000515653775322284
cg19958593	ENSG00000258912	LINC02316	-736312	L4	0.00035111389703912	0.000405156537753223	cg16153549	ENSG00000188765	TMSB4XP2	124496	R10	0.00154331208356285	0.000920810313075506

cg06098693	ENSG00000248844	AP003555.1	-90805	L5	0.00161006454167503	0.000957642725598527	cg11688378	ENSG00000237478	AL390023.1	918243	R5	0.0210992520232155	0.000184162062615101
cg16702469	ENSG00000237298	TTN-AS1	-66385	L4	0.00162363086374529	0.000994475138121547	cg16824643	ENSG00000266586	AC091027.2	206315	R1	0.0210992520232155	0.000368324125230203
cg00493617	ENSG00000236172	MIR7515HG	614074	R5	0.00172818394173935	0.000515653775322284	cg17136275	ENSG00000215802	RFKP1	111411	R5	0.0210992520232155	3,68E+09
cg05316785	ENSG00000234707	AC074351.1	221253	R6	0.00209696432021698	0.000552486187845304	cg18403193	ENSG00000226739	AL358972.1	-1587844	L9	0.0210992520232155	3,68E+09
cg03565720	ENSG00000270764	AC005070.1	370505	R10	0.00212135970504487	0.000847145488029466	cg18927604	ENSG00000257254	AC010202.1	-307558	L2	0.0210992520232155	3,68E+09
cg01397507	ENSG00000171671	SHANK2-AS3	268498	R6	0.00231509142120936	0.000883977900552486							
**Probe**	**GeneID**	**Symbol**	**Distance**	**Sides**	**Raw.p**	**Pe**	**Probe**	**GeneID**	**Symbol**	**Distance**	**Sides**	**Raw.p**	**Pe**
cg07000713	ENSG00000258316	KLF17P1	164728	R2	0.00244327075731631	0.000773480662983425	cg20067526	ENSG00000257254	AC010202.1	-891080	L7	0.0210992520232155	3,68E+09
cg03252770	ENSG00000248208	WDR45P1	225368	R3	0.00248608486265702	0.000589318600368324	cg20890989	ENSG00000231189	AC013448.1	789252	R9	0.0210992520232155	0.000883977900552486
cg13643764	ENSG00000248949	AC122719.1	-71597	L5	0.00256539148713538	0.000957642725598527	cg22927134	ENSG00000200008	RF00019	-334454	L9	0.0210992520232155	0.000736648250460405
cg17715141	ENSG00000248816	AC079226.2	1009356	R9	0.00287718603720921	7,37E+09	cg25432792	ENSG00000228999	AC096570.1	-792190	L5	0.0210992520232155	3,68E+09
cg00921309	ENSG00000253803	AC091163.3	-307818	L9	0.00287807644167594	0.000626151012891344	cg01990257	ENSG00000254344	LINC01288	794064	R6	0.02118104822389	0.000147329650092081
cg02314201	ENSG00000273980	AL592071.1	226871	R7	0.00293169629212861	0.000810313075506446	cg02638567	ENSG00000270694	AC211469.2	1394722	R8	0.02118104822389	3,68E+09
cg07142009	ENSG00000262089	AC040977.1	241644	R9	0.00300963223995606	0.000441988950276243	cg07764113	ENSG00000234306	AL583859.1	-276412	L4	0.02118104822389	3,68E+09
cg01549106	ENSG00000250576	AC012629.1	-11544	L1	0.00308675865923032	0.000626151012891344	cg08954776	ENSG00000248308	AC138938.1	901874	R6	0.02118104822389	3,68E+09
cg23154849	ENSG00000229670	PKP4P1	803496	R8	0.00316653379249996	0.000736648250460405	cg11075718	ENSG00000241385	AC023245.1	210289	R2	0.02118104822389	0.000589318600368324
cg17989763	ENSG00000279910	AL731532.1	422872	R4	0.00316778821516775	0.000957642725598527	cg13333233	ENSG00000234306	AL583859.1	26228	L1	0.02118104822389	3,68E+09
cg10309886	ENSG00000254419	AL139349.1	-121058	L8	0.00316881677380889	0.000441988950276243	cg20085588	ENSG00000225827	SFTPA3P	38860	R2	0.02118104822389	3,68E+09
cg13374432	ENSG00000259276	AC087286.1	-21824	L2	0.00317442485819893	0.000773480662983425	cg20393620	ENSG00000276703	RPS29P32	-77162	L4	0.02118104822389	7,37E+09
cg08270682	ENSG00000242488	AF107885.1	-309484	L8	0.00347612945709023	0.000920810313075506	cg22545168	ENSG00000251431	AC245884.7	-56090	L8	0.02118104822389	3,68E+09
cg06861395	ENSG00000248444	HNRNPA1P65	996996	R8	0.00525681240296044	0.000552486187845304	cg23397013	ENSG00000234306	AL583859.1	-162681	L3	0.02118104822389	3,68E+09
cg03481855	ENSG00000222501	RNU4-25P	-66435	L3	0.00547148239729757	0.000810313075506446	cg24580655	ENSG00000251686	OR10J8P	190311	R10	0.02118104822389	3,68E+09
cg25296103	ENSG00000234940	AC023128.1	177445	R7	0.00559620384805923	0.000110497237569061	cg00100440	ENSG00000257948	AC068993.2	315700	R2	0.0408295922183942	3,68E+09
cg07581775	ENSG00000254118	MTCYBP20	-79331	L5	0.00562182785370962	0.000773480662983425	cg00378313	ENSG00000225944	RIOK3P1	623349	R7	0.0408295922183942	3,68E+09
cg15259904	ENSG00000219095	DHFRP6	582112	R4	0.00563894131693046	0.000736648250460405	cg00650953	ENSG00000230190	AC005518.1	-130267	L3	0.0408295922183942	3,68E+09
cg19180555	ENSG00000240533	RN7SL69P	222568	R2	0.00563894131693046	0.000957642725598527	cg01882260	ENSG00000242178	MTND4P17	968059	R3	0.0408295922183942	3,68E+09
cg01285237	ENSG00000259282	SPATA8-AS1	-413059	L4	0.00564750727734767	0.000294659300184162	cg02415992	ENSG00000252927	RNA5SP404	-408192	L3	0.0408295922183942	3,68E+09
cg14380519	ENSG00000232851	ATP5MFP3	138468	R5	0.00564922120734989	7,37E+09	cg03602297	ENSG00000225943	AC016907.1	157829	R2	0.0408295922183942	3,68E+09
cg16072535	ENSG00000233319	PPIAP32	-227398	L4	0.00766658105680141	0.000920810313075506	cg03654504	ENSG00000234481	AC117945.1	-253735	L2	0.0408295922183942	3,68E+09
cg04425617	ENSG00000201229	SNORA63D	-140208	L9	0.0108886890915923	0.000662983425414365	cg06931356	ENSG00000201933	RF00019	-439447	L10	0.0408295922183942	3,68E+09
cg21141318	ENSG00000248484	AC113391.1	-402733	L10	0.0108886890915923	0.000257826887661142	cg07260541	ENSG00000260605	AC027688.1	-45172	L3	0.0408295922183942	3,68E+09
cg01514465	ENSG00000250272	TRMT112P1	-703789	L7	0.0109214228431017	0.000405156537753223	cg07260541	ENSG00000260620	AC027688.2	-27537	L2	0.0408295922183942	0.000515653775322284
cg02323699	ENSG00000254626	AP003100.1	-468855	L4	0.0109214228431017	0.000699815837937385	cg07855575	ENSG00000248908	LINC02239	-553201	L9	0.0408295922183942	3,68E+09
cg06333233	ENSG00000223826	LINC01870	128899	R3	0.0109214228431017	0.000147329650092081	cg08025405	ENSG00000232668	AC010967.3	-654326	L5	0.0408295922183942	3,68E+09
cg06740209	ENSG00000232409	AC019185.3	-683587	L8	0.0109214228431017	0.000847145488029466	cg09741912	ENSG00000255903	RPL12P46	526873	R7	0.0408295922183942	0.000736648250460405
cg08177332	ENSG00000232382	OR5K1	-152113	L8	0.0109214228431017	0.000515653775322284	cg09994435	ENSG00000279610	C20orf181	70009	R2	0.0408295922183942	0.000810313075506446
cg12056696	ENSG00000270359	AL358335.3	-1127141	L8	0.0109214228431017	3,68E+09	cg11041239	ENSG00000280725	LINC00251	-452291	L6	0.0408295922183942	3,68E+09
cg15506036	ENSG00000232409	AC019185.3	-621791	L6	0.0109214228431017	0.000847145488029466	cg11091914	ENSG00000253997	AC090142.3	404310	R3	0.0408295922183942	3,68E+09
cg16337763	ENSG00000280288	AC131935.2	308669	R3	0.0109214228431017	0.000552486187845304	cg11367259	ENSG00000251904	RNA5SP272	-560458	L4	0.0408295922183942	3,68E+09
cg02394701	ENSG00000270763	DDX43P2	-261095	L2	0.0109378034886543	0.000810313075506446	cg11969108	ENSG00000228806	AC092628.1	315690	R10	0.0408295922183942	3,68E+09
cg20000602	ENSG00000275967	MIR6880	87879	R2	0.0109378034886543	0.000184162062615101	cg13241663	ENSG00000265614	AC011193.2	-466286	L9	0.0408295922183942	3,68E+09
cg21141318	ENSG00000251446	AC113391.2	-389518	L9	0.0109410807180666	0.000331491712707182	cg16341266	ENSG00000230190	AC005518.1	112434	R1	0.0408295922183942	3,68E+09
cg22162757	ENSG00000224584	UBE2V1P13	87068	R4	0.0109410807180666	0.000994475138121547	cg16703220	ENSG00000223087	RNU6-602P	-233210	L4	0.0408295922183942	0.000331491712707182
cg25432792	ENSG00000223530	AC012506.1	7366	L1	0.0109410807180666	0.000552486187845304	cg17006281	ENSG00000228806	AC092628.1	308513	R10	0.0408295922183942	3,68E+09
cg08025405	ENSG00000233083	FTH1P6	-907703	L8	0.0109509146054654	0.000699815837937385	cg17952114	ENSG00000235181	AC245036.2	23455	R1	0.0408295922183942	3,68E+09
cg11518938	ENSG00000229724	OR2AQ1P	-372213	L9	0.0109509146054654	3,68E+09	cg18222913	ENSG00000276487	AC061709.2	-170903	L5	0.0408295922183942	3,68E+09
cg24580655	ENSG00000229724	OR2AQ1P	-379634	L9	0.0109509146054654	3,68E+09	cg18504196	ENSG00000242036	AC128713.1	-657044	L7	0.0408295922183942	3,68E+09
cg18646851	ENSG00000258540	AC107958.1	-714518	L8	0.0210615581060693	0.000957642725598527	cg18555142	ENSG00000228806	AC092628.1	-234330	L9	0.0408295922183942	3,68E+09
cg00320216	ENSG00000222774	RN7SKP121	-1173801	L9	0.0210992520232155	3,68E+09	cg18561261	ENSG00000128519	TAS2R16	-648721	L10	0.0408295922183942	3,68E+09
cg06601993	ENSG00000232968	EIF4EP4	847850	R8	0.0210992520232155	3,68E+09	cg19731286	ENSG00000225944	RIOK3P1	670302	R7	0.0408295922183942	3,68E+09
cg08530838	ENSG00000251741	RNU4ATAC13P	-1364200	L7	0.0210992520232155	3,68E+09	cg22378678	ENSG00000242036	AC128713.1	488345	R8	0.0408295922183942	3,68E+09
cg09102329	ENSG00000215418	PEX12P1	-799197	L7	0.0210992520232155	0.000257826887661142	cg24083274	ENSG00000128519	TAS2R16	657565	R7	0.0408295922183942	3,68E+09

cg25728328	ENSG00000260232	PWRN4	33851	L1	0.0408295922183942	0.000552486187845304	cg27589988	ENSG00000229242	AL358452.1	912032	R6	0.0408295922183942	3,68E+09
cg26738880	ENSG00000228806	AC092628.1	469076	R10	0.0408295922183942	3,68E+09	cg27616666	ENSG00000222376	RN7SKP152	-1155950	L8	0.0408295922183942	3,68E+09
cg27357229	ENSG00000222376	RN7SKP152	-78214	L2	0.0408295922183942	3,68E+09							
