# Supplementary material for: Epigenomic integrative analysis pinpoint master regulator transcription factors associated with tumorigenesis in squamous cell carcinoma of oral tongue
Source: Genet Mol Biol. 2023 Jun 19;46(2):e20220358. doi: 10.1590/1678-4685-GMB-2022-0358 (PMC10280803; doi:10.1590/1678-4685-GMB-2022-0358)
Supplement: Table S3: S3A - [file 1415-4757-GMB-46-2-e20220358-s3.pdf]

Genetics and Molecular Biology

https://doi.org/ 10.1590/1678-4685-GMB-2022-0358

Supplementary material to "Epigenomic integrative analysis pinpoint master regulator transcription factors associated with tumorigenesis in squamous cell carcinoma of oral tongue"

Table S3 - Hypermethylated probes identified from the correlation between tumor samples from the tongue region and non-tumor samples.

Motif	Nº of probes	% of probes	lower OR	upper OR	OR	p.value	FDR	TF family	TF subfamily	TF.family.member	TF.subfamily.member
ZBT14_HUMAN.H11MO.0.C	169	0.66274510	22.795.150	3,90E+06	2973201,00	2,51E-11	1,94E-08	More than 3 adjacent zinc finger factors{2.3.3}	Unclassified {2.3.3.0}	BCL6B;BCL6;CTCFL;CTCF;FEZF1;GFI1B;GFI1;GLI1;GLI2;GLI3;GLIS1;GLIS2;GLIS3;MTF1;MYNN;MZF1;OSR2;OVOL1;OVOL2;ZNF146;PLAG1;PLAGL1;PRDM14;PRDM1;PRDM6;SCRT1;SCRT2;SNAI1;SNAI2;YY1;YY2;WT1;ZNF324;ZNF354A;ZBTB14;ZBTB18;ZBTB48;ZBTB49;ZBTB7A;ZBTB7B;ZBTB6;ZFP64;ZFP28;ZFP42;ZFP82;ZFX;ZIC1;ZIC2;ZIC3;ZIC4;ZIM3;ZKSCAN1;ZKSCAN3;ZNF121;ZNF136;ZNF140;ZNF143;ZNF148;ZNF214;ZNF232;ZNF250;ZNF257;ZNF260;ZNF263;ZNF264;ZNF274;ZNF281;ZNF282;ZNF317;ZNF320;ZNF322;ZNF329;ZNF331;ZNF333;ZNF350;ZNF384;ZNF394;ZNF410;ZNF436;ZNF449;ZNF490;ZNF502;ZNF524;ZNF528;ZNF547;ZNF549;ZNF554;ZNF563;ZNF582;ZNF586;ZNF589;ZNF652;ZNF667;ZNF680;ZNF708;ZNF713;ZNF768;ZNF816;ZNF18;ZNF41;ZNF76;ZNF85;ZSCAN16;ZSCAN22;ZSCAN31;ZSCAN4	MYNN;MZF1;OSR2;PRDM14;PRDM6;WT1;ZBTB14;ZBTB48;ZBTB49;ZFP64;ZFP28;ZIM3;ZNF121;ZNF250;ZNF257;ZNF263;ZNF274;ZNF317;ZNF320;ZNF329;ZNF331;ZNF394;ZNF449;ZNF502;ZNF528;ZNF547;ZNF549;ZNF554;ZNF586;ZNF589;ZNF667;ZNF680;ZNF708;ZNF713;ZNF768;ZNF18;ZNF85;ZSCAN16;ZSCAN22
KLF12_HUMAN.H11MO.0.C	177	0.69411765	20.165.179	3,50E+06	2647082,00	1,11E-07	4,27E-05	Three-zinc finger Krüppel-related factors{2.3.1}	Krüppel-like factors {2.3.1.2}	EGR1;EGR2;EGR3;EGR4;KLF1;KLF10;KLF11;KLF12;KLF13;KLF14;KLF15;KLF16;KLF17;KLF2;KLF3;KLF4;KLF5;KLF6;KLF7;KLF8;KLF9;SP1;SP2;SP3;SP4;SP5;SP6;SP7;SP8;SP9	KLF12;KLF13;KLF14;KLF15;KLF16;KLF1;KLF3;KLF4;KLF5;KLF6;KLF8;KLF9
WT1_HUMAN.H11MO.0.C	203	0.79607843	18.686.139	3,52E+06	2545762,00	1,20E-04	1,33E-02	More than 3 adjacent zinc finger factors{2.3.3}	unclassified{2.3.3.0}	BCL6B;BCL6;CTCFL;CTCF;FEZF1;GFI1B;GFI1;GLI1;GLI2;GLI3;GLIS1;GLIS2;GLIS3;MTF1;MYNN;MZF1;OSR2;OVOL1;OVOL2;ZNF146;PLAG1;PLAGL1;PRDM14;PRDM1;PRDM6;SCRT1;SCRT2;SNAI1;SNAI2;YY1;YY2;WT1;ZNF324;ZNF354A;ZBTB14;ZBTB18;ZBTB48;ZBTB49;ZBTB7A;ZBTB7B;ZBTB6;ZFP64;ZFP28;ZFP42;ZFP82;ZFX;ZIC1;ZIC2;ZIC3;ZIC4;ZIM3;ZKSCAN1;ZKSCAN3;ZNF121;ZNF136;ZNF140;ZNF143;ZNF148;ZNF214;ZNF232;ZNF250;ZNF257;ZNF260;ZNF263;ZNF264;ZNF274;ZNF281;ZNF282;ZNF317;ZNF320;ZNF322;ZNF329;ZNF331;ZNF333;ZNF350;ZNF384;ZNF394;ZNF410;ZNF436;ZNF449;ZNF490;ZNF502;ZNF524;ZNF528;ZNF547;ZNF549;ZNF554;ZNF563;ZNF582;ZNF586;ZNF589;ZNF652;ZNF667;ZNF680;ZNF708;ZNF713;ZNF768;ZNF816;ZNF18;ZNF41;ZNF76;ZNF85;ZSCAN16;ZSCAN22;ZSCAN31;ZSCAN4	MYNN;MZF1;OSR2;PRDM14;PRDM6;WT1;ZBTB14;ZBTB48;ZBTB49;ZFP64;ZFP28;ZIM3;ZNF121;ZNF250;ZNF257;ZNF263;ZNF274;ZNF317;ZNF320;ZNF329;ZNF331;ZNF394;ZNF449;ZNF502;ZNF528;ZNF547;ZNF549;ZNF554;ZNF586;ZNF589;ZNF667;ZNF680;ZNF708;ZNF713;ZNF768;ZNF18;ZNF85;ZSCAN16;ZSCAN22
SP3_HUMAN.H11MO.0.B	206	0.80784314	18.449.177	3,53E+06	2530476,00	3,05E-04	2,29E-02	Three-zinc finger Krüppel-related factors{2.3.1}	Sp1-like factors{2.3.1.1}	EGR1;EGR2;EGR3;EGR4;KLF1;KLF10;KLF11;KLF12;KLF13;KLF14;KLF15;KLF16;KLF17;KLF2;KLF3;KLF4;KLF5;KLF6;KLF7;KLF8;KLF9;SP1;SP2;SP3;SP4;SP5;SP6;SP7;SP8;SP9	SP1;SP2;SP3;SP4
E2F4_HUMAN.H11MO.1.A	134	0.52549020	19.090.523	3,17E+06	2460327,00	1,23E-06	3,17E-04	E2F-related factors{3.3.2}	E2F{3.3.2.1}	E2F1;E2F2;E2F3;E2F4;E2F5;E2F6;E2F7;E2F8;TFDP1;TFDP2	E2F1;E2F2;E2F3;E2F4;E2F5;E2F6;E2F7;E2F8
MECP2_HUMAN.H11MO.0.C	153	0.60000000	18.707.770	3,14E+06	2419953,00	3,69E-06	7,11E-04	*NA*	*NA*	MECP2	MECP2

Motif	Nº of probes	% of probes	lower OR	upper OR	OR	p.value	FDR	TF family	TF subfamily	TF.family.member	TF.subfamily.member
MAZ_HUMAN.H11MO.1.A	169	0.66274510	18.242.787	3,12E+06	2379329,00	1,95E-05	3,01E-03	Factors with multiple dispersed zinc fingers {2.3.4}	MAZ-like factors{2.3.4.8}	BCL11A;BCL11B;BNC1;BNC2;E4F1;HIC1;HIC2;HINFP;HIVEP1;HIVEP2;HIVEP3;IKZF1;IKZF2;IKZF3;IKZF4;IKZF5;INSM1;INSM2;MAZ;MECOM;PATZ1;PRDM16;PRDM4;REST;RLF;RREB1;SALL1;SALL2;SALL3;SALL4;VEZF1;ZBTB1;ZBTB17;ZBTB2;ZBTB25;ZBTB4;ZFAT;ZNF134;ZNF211;ZNF217;ZNF219;ZNF248;ZNF256;ZNF292;ZNF296;ZNF319;ZNF334;ZNF335;ZNF341;ZNF37A;ZNF382;ZNF417;ZNF418;ZNF423;ZNF467;ZNF510;ZNF512;ZNF512B;ZNF516;ZNF518A;ZNF518B;ZNF521;ZNF526;ZNF532;ZNF536;ZNF552;ZNF574;ZNF587;ZNF587B;ZNF592;ZNF639;ZNF654;ZNF658;ZNF671;ZNF687;ZNF711;ZNF717;ZNF770;ZNF772;ZNF784;ZNF786;ZNF792;ZNF8;ZNF814	MAZ;PATZ1;VEZF1
E2F2_HUMAN.H11MO.0.B	99	0.38823529	18.259.296	3,07E+06	2374085,00	1,38E-04	1,33E-02	E2F-related factors{3.3.2}	E2F{3.3.2.1}	E2F1;E2F2;E2F3;E2F4;E2F5;E2F6;E2F7;E2F8;TFDP1;TFDP2	E2F1;E2F2;E2F3;E2F4;E2F5;E2F6;E2F7;E2F8
SP2_HUMAN.H11MO.1.B	174	0.68235294	17.929.590	3,10E+06	2347534,00	5,72E-05	7,36E-03	Three-zinc finger Krüppel-related factors{2.3.1}	Sp1-like factors{2.3.1.1}	EGR1;EGR2;EGR3;EGR4;KLF1;KLF10;KLF11;KLF12;KLF13;KLF14;KLF15;KLF16;KLF17;KLF2;KLF3;KLF4;KLF5;KLF6;KLF7;KLF8;KLF9;SP1;SP2;SP3;SP4;SP5;SP6;SP7;SP8;SP9	SP1;SP2;SP3;SP4
THAP1_HUMAN.H11MO.0.C	191	0.74901961	17.568.509	3,16E+06	2343606,00	5,60E-04	3,09E-02	THAP-related factors{2.9.1}	THAP1{2.9.1.0.1}	THAP1;THAP10;THAP11;THAP12;THAP2;THAP3;THAP4;THAP5;THAP6;THAP7;THAP8;THAP9	THAP1
E2F5_HUMAN.H11MO.0.B	55	0.21568627	16.891.868	3,14E+06	2321407,00	3,54E-01	7,37E+00	E2F-related factors{3.3.2}	E2F{3.3.2.1}	E2F1;E2F2;E2F3;E2F4;E2F5;E2F6;E2F7;E2F8;TFDP1;TFDP2	E2F1;E2F2;E2F3;E2F4;E2F5;E2F6;E2F7;E2F8
SP1_HUMAN.H11MO.1.A	191	0.74901961	17.284.277	3,11E+06	2305909,00	1,23E-03	6,32E-02	Three-zinc finger Krüppel-related factors{2.3.1}	Sp1-like factors{2.3.1.1}	EGR1;EGR2;EGR3;EGR4;KLF1;KLF10;KLF11;KLF12;KLF13;KLF14;KLF15;KLF16;KLF17;KLF2;KLF3;KLF4;KLF5;KLF6;KLF7;KLF8;KLF9;SP1;SP2;SP3;SP4;SP5;SP6;SP7;SP8;SP9	SP1;SP2;SP3;SP4
SP4_HUMAN.H11MO.1.A	177	0.69411765	17.438.128	3,03E+06	2289335,00	3,26E-04	2,29E-02	Three-zinc finger Krüppel-related factors{2.3.1}	Sp1-like factors{2.3.1.1}	EGR1;EGR2;EGR3;EGR4;KLF1;KLF10;KLF11;KLF12;KLF13;KLF14;KLF15;KLF16;KLF17;KLF2;KLF3;KLF4;KLF5;KLF6;KLF7;KLF8;KLF9;SP1;SP2;SP3;SP4;SP5;SP6;SP7;SP8;SP9	SP1;SP2;SP3;SP4
ZN148_HUMAN.H11MO.0.D	174	0.68235294	17.138.896	2,96E+06	2244053,00	5,22E-04	3,09E-02	More than 3 adjacent zinc finger factors{2.3.3}	ZNF148-like factors{2.3.3.13}	BCL6;BCL6B;CTCF;CTCFL;FEZF1;FEZF2;GFI1;GFI1B;GLI1;GLI2;GLI3;GLI4;GLIS1;GLIS2;GLIS3;HKR1;MTF1;MYNN;MZF1;OSR2;OVOL1;OVOL2;PLAG1;PLAGL1;PLAGL2;PRDM1;PRDM14;PRDM6;SCRT1;SCRT2;SNAI1;SNAI2;SNAI3;WT1;YY1;YY2;ZBTB12;ZBTB14;ZBTB18;ZBTB20;ZBTB26;ZBTB42;ZBTB45;ZBTB47;ZBTB48;ZBTB49;ZBTB6;ZBTB7A;ZBTB7B;ZBTB7C;ZFP14;ZFP2;ZFP28;ZFP30;ZFP37;ZFP42;ZFP64;ZFP69;ZFP69B;ZFP82;ZFP91;ZFX;ZIC1;ZIC2;ZIC3;ZIC4;ZIC5;ZIK1;ZIM3;ZKSCAN1;ZKSCAN2;ZKSCAN3;ZKSCAN4;ZNF121;ZNF124;ZNF133;ZNF136;ZNF138;ZNF14;ZNF140;ZNF143;ZNF146;ZNF148;ZNF155;ZNF157;ZNF160;ZNF169;ZNF175;ZNF177;ZNF18;ZNF180;ZNF181;ZNF2;ZNF20;ZNF212;ZNF213;ZNF214;ZNF221;ZNF222;ZNF223;ZNF224;ZNF225;ZNF226;ZNF227;ZNF229;ZNF230;ZNF232;ZNF233;ZNF234;ZNF235;ZNF24;ZNF25;ZNF250;ZNF257;ZNF26;ZNF260;ZNF263;ZNF264;ZNF268;ZNF274;ZNF276;ZNF28;ZNF280A;ZNF280B;ZNF280C;ZNF280D;ZNF281;ZNF282;ZNF283;ZNF284;ZNF285;ZNF286A;ZNF286B;ZNF3;ZNF30;ZNF300;ZNF302;ZNF317;ZNF32;ZNF320;ZNF322;ZNF324;ZNF324B;ZNF329;ZNF331;ZNF333;ZNF33A;ZNF33B;ZNF343;ZNF345;ZNF347;ZNF350;ZNF354A;ZNF354B;ZNF362;ZNF366;ZNF383;ZNF384;ZNF394;ZNF397;ZNF398;ZNF404;ZNF41;ZNF410;ZNF419;ZNF420;ZNF431;ZNF432;ZNF436;ZNF439;ZNF44;ZNF440;ZNF442;ZNF443;ZNF446;ZNF449;ZNF45;ZNF460;ZNF468;ZNF479;ZNF484;ZNF490;ZNF500;ZNF502;ZNF524;ZNF525;ZNF528;ZNF543;ZNF544;ZNF546;ZNF547;ZNF548;ZNF549;ZNF554;ZNF555;ZNF557;ZNF558;ZNF559;ZNF561;ZNF562;ZNF563;ZNF564;ZNF566;ZNF567;ZNF568;ZNF57;ZNF570;ZNF571;ZNF572;ZNF577;ZNF581;ZNF582;ZNF583;ZNF585A;ZNF586;ZNF589;ZNF595;ZNF599;ZNF600;ZNF605;ZNF607;ZNF611;ZNF613;ZNF614;ZNF615;ZNF616;ZNF619;ZNF620;ZNF621;ZNF625;ZNF627;ZNF649;ZNF652;ZNF653;ZNF665;ZNF667;ZNF669;ZNF670;ZNF672;ZNF679;ZNF680;ZNF683;ZNF689;ZNF692;ZNF701;ZNF705D;ZNF705E;ZNF705G;ZNF708;ZNF709;ZNF71;ZNF710;ZNF713;ZNF721;ZNF727;ZNF729;ZNF736;ZNF75A;ZNF75D;ZNF76;ZN	ZNF148;ZNF281

Motif	Nº of probes	% of probes	lower OR	upper OR	OR	p.value	FDR	TF family	TF subfamily	TF.family.member	TF.subfamily.member
										F763;ZNF764;ZNF765;ZNF768;ZNF77;ZNF771;ZNF773;ZNF774;ZNF776;ZNF777;ZNF780A;ZNF780B;ZNF782;ZNF785;ZNF799;ZNF805;ZNF808;ZNF81;ZNF813;ZNF816;ZNF823;ZNF829;ZNF836;ZNF841;ZNF844;ZNF845;ZNF846;ZNF85;ZNF853;ZNF860;ZNF878;ZNF891;ZNF99;ZSCAN16;ZSCAN2;ZSCAN22;ZSCAN23;ZSCAN29;ZSCAN31;ZSCAN32;ZSCAN4;ZSCAN5A;ZSCAN5B;ZSCAN5C;ZSCAN9;ZXDA;ZXDB;ZXDC	
KAISO_HUMAN.H11MO.0.A	116	0.45490196	17.375.831	2,89E+06	2243643,00	4,74E-04	3,05E-02	Other factors with up to three adjacent zinc fingers{2.3.2}	Factors with 2-3 adjacent zinc fingersand a BTB/POZ domain{2.3.2.1}	AEBP2;OSR1;OVOL3;ZBTB22;ZBTB32;ZBTB33;ZBTB34;ZBTB37;ZBTB43;ZBTB46;ZBTB5;ZBTB8B;ZFPM2;ZNF174;ZNF396;ZNF414;ZNF446;ZNF487;ZNF511;ZNF580;ZNF705A;ZNF740;ZSCAN1	ZBTB33
E2F1_HUMAN.H11MO.0.A	161	0.63137255	17.235.658	2,92E+06	2237241,00	2,44E-04	2,09E-02	E2F-related factors{3.3.2}	E2F{3.3.2.1}	E2F1;E2F2;E2F3;E2F4;E2F5;E2F6;E2F7;E2F8;TFDP1;TFDP2	E2F1;E2F2;E2F3;E2F4;E2F5;E2F6;E2F7;E2F8
SP2_HUMAN.H11MO.0.A	213	0.83529412	15.549.533	3,10E+06	2173698,00	9,60E-01	1,85E+01	Three-zinc finger Krüppel-related factors{2.3.1}	Sp1-like factors{2.3.1.1}	EGR1;EGR2;EGR3;EGR4;KLF1;KLF10;KLF11;KLF12;KLF13;KLF14;KLF15;KLF16;KLF17;KLF2;KLF3;KLF4;KLF5;KLF6;KLF7;KLF8;KLF9;SP1;SP2;SP3;SP4;SP5;SP6;SP7;SP8;SP9	SP1;SP2;SP3;SP4
E2F4_HUMAN.H11MO.0.A	172	0.67450980	16.620.792	2,86E+06	2172694,00	2,52E-03	1,20E-01	E2F-related factors{3.3.2}	E2F{3.3.2.1}	E2F1;E2F2;E2F3;E2F4;E2F5;E2F6;E2F7;E2F8;TFDP1;TFDP2	E2F1;E2F2;E2F3;E2F4;E2F5;E2F6;E2F7;E2F8
SP1_HUMAN.H11MO.0.A	205	0.80392157	15.845.329	3,02E+06	2168326,00	2,12E-01	5,40E+00	Three-zinc finger Krüppel-related factors{2.3.1}	Sp1-like factors{2.3.1.1}	EGR1;EGR2;EGR3;EGR4;KLF1;KLF10;KLF11;KLF12;KLF13;KLF14;KLF15;KLF16;KLF17;KLF2;KLF3;KLF4;KLF5;KLF6;KLF7;KLF8;KLF9;SP1;SP2;SP3;SP4;SP5;SP6;SP7;SP8;SP9	SP1;SP2;SP3;SP4
ZN467_HUMAN.H11MO.0.C	181	0.70980392	16.445.921	2,88E+06	2167237,00	5,50E-03	2,36E-01	Factors with multiple dispersed zinc fingers{2.3.4}	unclassified{2.3.4.0}	BCL11A;E4F1;MECOM;HIC1;HIC2;HINFP;IKZF1;INSM1;MAZ;PATZ1;PRDM4;REST;RREB1;SALL4;VEZF1;ZBTB17;ZBTB4;HIVEP1;HIVEP2;ZNF134;ZNF219;ZNF335;ZNF341;ZNF382;ZNF418;ZNF423;ZNF467;ZNF770;ZNF784;ZNF8	E4F1;PRDM4;REST;RREB1;ZBTB17;ZBTB4;ZNF335;ZNF341;ZNF467;ZNF770;ZNF784;ZNF8
ZN263_HUMAN.H11MO.0.A	180	0.70588235	16.354.002	2,86E+06	2152912,00	7,82E-03	3,17E-01	More than 3 adjacent zinc finger factors{2.3.3}	unclassified{2.3.3.0}	BCL6B;BCL6;CTCFL;CTCF;FEZF1;GFI1B;GFI1;GLI1;GLI2;GLI3;GLIS1;GLIS2;GLIS3;MTF1;MYNN;MZF1;OSR2;OVOL1;OVOL2;ZNF146;PLAG1;PLAGL1;PRDM14;PRDM1;PRDM6;SCRT1;SCRT2;SNAI1;SNAI2;YY1;YY2;WT1;ZNF324;ZNF354A;ZBTB14;ZBTB18;ZBTB48;ZBTB49;ZBTB7A;ZBTB7B;ZBTB6;ZFP64;ZFP28;ZFP42;ZFP82;ZFX;ZIC1;ZIC2;ZIC3;ZIC4;ZIM3;ZKSCAN1;ZKSCAN3;ZNF121;ZNF136;ZNF140;ZNF143;ZNF148;ZNF214;ZNF232;ZNF250;ZNF257;ZNF260;ZNF263;ZNF264;ZNF274;ZNF281;ZNF282;ZNF317;ZNF320;ZNF322;ZNF329;ZNF331;ZNF333;ZNF350;ZNF384;ZNF394;ZNF410;ZNF436;ZNF449;ZNF490;ZNF502;ZNF524;ZNF528;ZNF547;ZNF549;ZNF554;ZNF563;ZNF582;ZNF586;ZNF589;ZNF652;ZNF667;ZNF680;ZNF708;ZNF713;ZNF768;ZNF816;ZNF18;ZNF41;ZNF76;ZNF85;ZSCAN16;ZSCAN22;ZSCAN31;ZSCAN4	MYNN;MZF1;OSR2;PRDM14;PRDM6;WT1;ZBTB14;ZBTB48;ZBTB49;ZFP64;ZFP28;ZIM3;ZNF121;ZNF250;ZNF257;ZNF263;ZNF274;ZNF317;ZNF320;ZNF329;ZNF331;ZNF394;ZNF449;ZNF502;ZNF528;ZNF547;ZNF549;ZNF554;ZNF586;ZNF589;ZNF667;ZNF680;ZNF708;ZNF713;ZNF768;ZNF18;ZNF85;ZSCAN16;ZSCAN22
KLF6_HUMAN.H11MO.0.A	190	0.74509804	15.928.448	2,86E+06	2122042,00	5,05E-02	1,77E+00	Three-zinc finger Krüppel-related factors{2.3.1}	Krüppel-like factors{2.3.1.2}	EGR1;EGR2;EGR3;EGR4;KLF1;KLF10;KLF11;KLF12;KLF13;KLF14;KLF15;KLF16;KLF17;KLF2;KLF3;KLF4;KLF5;KLF6;KLF7;KLF8;KLF9;SP1;SP2;SP3;SP4;SP5;SP6;SP7;SP8;SP9	KLF12;KLF13;KLF14;KLF15;KLF16;KLF1;KLF3;KLF4;KLF5;KLF6;KLF8;KLF9

Motif	Nº of probes	% of probes	lower OR	upper OR	OR	p.value	FDR	TF family	TF subfamily	TF.family.member	TF.subfamily.member
KLF14_HUMAN.H11MO.0.D	138	0.54117647	16.440.711	2,74E+06	2119293,00	2,66E-03	1,20E-01	Three-zinc finger Krüppel-related factors{2.3.1}	Krüppel-like factors{2.3.1.2}	EGR1;EGR2;EGR3;EGR4;KLF1;KLF10;KLF11;KLF12;KLF13;KLF14;KLF15;KLF16;KLF17;KLF2;KLF3;KLF4;KLF5;KLF6;KLF7;KLF8;KLF9;SP1;SP2;SP3;SP4;SP5;SP6;SP7;SP8;SP9	KLF12;KLF13;KLF14;KLF15;KLF16;KLF1;KLF3;KLF4;KLF5;KLF6;KLF8;KLF9
KAISO_HUMAN.H11MO.1.A	85	0.33333333	16.071.196	2,76E+06	2111327,00	9,84E-02	2,92E+00	Other factors with up to three adjacent zinc fingers{2.3.2}	Factors with 2-3 adjacent zinc fingersand a BTB/POZ domain{2.3.2.1}	AEBP2;OSR1;OVOL3;ZBTB22;ZBTB32;ZBTB33;ZBTB34;ZBTB37;ZBTB43;ZBTB46;ZBTB5;ZBTB8B;ZFPM2;ZNF174;ZNF396;ZNF414;ZNF446;ZNF487;ZNF511;ZNF580;ZNF705A;ZNF740;ZSCAN1	ZBTB33
SP4_HUMAN.H11MO.0.A	185	0.72549020	15.684.071	2,77E+06	2076076,00	6,20E-02	2,01E+00	Three-zinc finger Krüppel-related factors{2.3.1}	Sp1-like factors{2.3.1.1}	EGR1;EGR2;EGR3;EGR4;KLF1;KLF10;KLF11;KLF12;KLF13;KLF14;KLF15;KLF16;KLF17;KLF2;KLF3;KLF4;KLF5;KLF6;KLF7;KLF8;KLF9;SP1;SP2;SP3;SP4;SP5;SP6;SP7;SP8;SP9	SP1;SP2;SP3;SP4
ZN281_HUMAN.H11MO.0.A	163	0.63921569	15.762.002	2,67E+06	2048008,00	2,34E-02	9,04E-01	More than 3 adjacent zinc finger factors{2.3.3}	ZNF148-like factors{2.3.3.13}	BCL6;BCL6B;CTCF;CTCFL;FEZF1;FEZF2;GFI1;GFI1B;GLI1;GLI2;GLI3;GLI4;GLIS1;GLIS2;GLIS3;HKR1;MTF1;MYNN;MZF1;OSR2;OVOL1;OVOL2;PLAG1;PLAGL1;PLAGL2;PRDM1;PRDM14;PRDM6;SCRT1;SCRT2;SNAI1;SNAI2;SNAI3;WT1;YY1;YY2;ZBTB12;ZBTB14;ZBTB18;ZBTB20;ZBTB26;ZBTB42;ZBTB45;ZBTB47;ZBTB48;ZBTB49;ZBTB6;ZBTB7A;ZBTB7B;ZBTB7C;ZFP14;ZFP2;ZFP28;ZFP30;ZFP37;ZFP42;ZFP64;ZFP69;ZFP69B;ZFP82;ZFP91;ZFX;ZIC1;ZIC2;ZIC3;ZIC4;ZIC5;ZIK1;ZIM3;ZKSCAN1;ZKSCAN2;ZKSCAN3;ZKSCAN4;ZNF121;ZNF124;ZNF133;ZNF136;ZNF138;ZNF14;ZNF140;ZNF143;ZNF146;ZNF148;ZNF155;ZNF157;ZNF160;ZNF169;ZNF175;ZNF177;ZNF18;ZNF180;ZNF181;ZNF2;ZNF20;ZNF212;ZNF213;ZNF214;ZNF221;ZNF222;ZNF223;ZNF224;ZNF225;ZNF226;ZNF227;ZNF229;ZNF230;ZNF232;ZNF233;ZNF234;ZNF235;ZNF24;ZNF25;ZNF250;ZNF257;ZNF26;ZNF260;ZNF263;ZNF264;ZNF268;ZNF274;ZNF276;ZNF28;ZNF280A;ZNF280B;ZNF280C;ZNF280D;ZNF281;ZNF282;ZNF283;ZNF284;ZNF285;ZNF286A;ZNF286B;ZNF3;ZNF30;ZNF300;ZNF302;ZNF317;ZNF32;ZNF320;ZNF322;ZNF324;ZNF324B;ZNF329;ZNF331;ZNF333;ZNF33A;ZNF33B;ZNF343;ZNF345;ZNF347;ZNF350;ZNF354A;ZNF354B;ZNF362;ZNF366;ZNF383;ZNF384;ZNF394;ZNF397;ZNF398;ZNF404;ZNF41;ZNF410;ZNF419;ZNF420;ZNF431;ZNF432;ZNF436;ZNF439;ZNF44;ZNF440;ZNF442;ZNF443;ZNF446;ZNF449;ZNF45;ZNF460;ZNF468;ZNF479;ZNF484;ZNF490;ZNF500;ZNF502;ZNF524;ZNF525;ZNF528;ZNF543;ZNF544;ZNF546;ZNF547;ZNF548;ZNF549;ZNF554;ZNF555;ZNF557;ZNF558;ZNF559;ZNF561;ZNF562;ZNF563;ZNF564;ZNF566;ZNF567;ZNF568;ZNF57;ZNF570;ZNF571;ZNF572;ZNF577;ZNF581;ZNF582;ZNF583;ZNF585A;ZNF586;ZNF589;ZNF595;ZNF599;ZNF600;ZNF605;ZNF607;ZNF611;ZNF613;ZNF614;ZNF615;ZNF616;ZNF619;ZNF620;ZNF621;ZNF625;ZNF627;ZNF649;ZNF652;ZNF653;ZNF665;ZNF667;ZNF669;ZNF670;ZNF672;ZNF679;ZNF680;ZNF683;ZNF689;ZNF692;ZNF701;ZNF705D;ZNF705E;ZNF705G;ZNF708;ZNF709;ZNF71;ZNF710;ZNF713;ZNF721;ZNF727;ZNF729;ZNF736;ZNF75A;ZNF75D;ZNF76;ZNF763;ZNF764;ZNF765;ZNF768;ZNF77;ZNF771;ZNF773;ZNF774;ZNF776;ZNF777;ZNF780A;ZNF780B;ZNF782;ZNF785;ZNF799;ZNF805;ZNF808;ZNF81;ZNF813;ZNF816;ZNF823;ZNF829;ZNF836;ZNF841;ZNF844;ZNF845;ZNF846;ZNF85;ZNF853;ZNF860;ZNF878;ZNF891;ZNF99;ZSCAN16;ZSCAN2;ZSCAN22;ZSCAN23;ZSCAN29;ZSCAN31;ZSCAN32;ZSCAN4;ZSCAN5A;ZSCAN5B;ZSCAN5C;ZSCAN9;ZXDA;ZXDB;ZXDC	ZNF148;ZNF281
PATZ1_HUMAN.H11MO.0.C	194	0.76078431	15.246.124	2,77E+06	2043100,00	3,25E-01	6,95E+00	Factors with multiple dispersed zinc fingers{2.3.4}	MAZ-like factors{2.3.4.8}	BCL11A;BCL11B;BNC1;BNC2;E4F1;HIC1;HIC2;HINFP;HIVEP1;HIVEP2;HIVEP3;IKZF1;IKZF2;IKZF3;IKZF4;IKZF5;INSM1;INSM2;MAZ;MECOM;PATZ1;PRDM16;PRDM4;REST;RLF;RREB1;SALL1;SALL2;SALL3;SALL4;VEZF1;ZBTB1;ZBTB17;ZBTB2;ZBTB25;ZBTB4;ZFAT;ZNF134;ZNF211;ZNF217;ZNF219;ZNF248;ZNF256;ZNF292;ZNF296;ZNF319;ZNF334;ZNF335;ZNF341;ZNF37A;ZNF382;ZNF417;ZNF418;ZNF423;ZNF467;ZNF510;ZNF512;ZNF512B;ZNF516;ZNF518A;ZNF518B;ZNF521;ZNF526;ZNF532;ZNF536;ZNF552;ZNF574;ZNF587;ZNF587B;ZNF592;ZNF639;ZNF654;ZNF658;ZNF671;ZNF687;ZNF711;ZNF717;ZNF770;ZNF772;ZNF784;ZNF786;ZNF792;ZNF8;ZNF814	MAZ;PATZ1;VEZF1

Motif	Nº of probes	% of probes	lower OR	upper OR	OR	p.value	FDR	TF family	TF subfamily	TF.family.member	TF.subfamily.member
KLF3_HUMAN.H11MO.0.B	186	0.72941176	15.348.943	2,72E+06	2034154,00	1,67E-01	4,48E+00	Three-zinc finger Krüppel-related factors{2.3.1}	Krüppel-like factors{2.3.1.2}	EGR1;EGR2;EGR3;EGR4;KLF1;KLF10;KLF11;KLF12;KLF13;KLF14;KLF15;KLF16;KLF17;KLF2;KLF3;KLF4;KLF5;KLF6;KLF7;KLF8;KLF9;SP1;SP2;SP3;SP4;SP5;SP6;SP7;SP8;SP9	KLF12;KLF13;KLF14;KLF15;KLF16;KLF1;KLF3;KLF4;KLF5;KLF6;KLF8;KLF9
MAZ_HUMAN.H11MO.0.A	186	0.72941176	15.304.043	2,71E+06	2028203,00	2,29E-01	5,52E+00	Factors with multiple dispersed zinc fingers{2.3.4}	MAZ-like factors{2.3.4.8}	BCL11A;BCL11B;BNC1;BNC2;E4F1;HIC1;HIC2;HINFP;HIVEP1;HIVEP2;HIVEP3;IKZF1;IKZF2;IKZF3;IKZF4;IKZF5;INSM1;INSM2;MAZ;MECOM;PATZ1;PRDM16;PRDM4;REST;RLF;RREB1;SALL1;SALL2;SALL3;SALL4;VEZF1;ZBTB1;ZBTB17;ZBTB2;ZBTB25;ZBTB4;ZFAT;ZNF134;ZNF211;ZNF217;ZNF219;ZNF248;ZNF256;ZNF292;ZNF296;ZNF319;ZNF334;ZNF335;ZNF341;ZNF37A;ZNF382;ZNF417;ZNF418;ZNF423;ZNF467;ZNF510;ZNF512;ZNF512B;ZNF516;ZNF518A;ZNF518B;ZNF521;ZNF526;ZNF532;ZNF536;ZNF552;ZNF574;ZNF587;ZNF587B;ZNF592;ZNF639;ZNF654;ZNF658;ZNF671;ZNF687;ZNF711;ZNF717;ZNF770;ZNF772;ZNF784;ZNF786;ZNF792;ZNF8;ZNF814	MAZ;PATZ1;VEZF1
MBD2_HUMAN.H11MO.0.B	158	0.61960784	15.566.046	2,63E+06	2017483,00	3,23E-02	1,19E+00	*NA*	*NA*	MBD2	MBD2
TBX1_HUMAN.H11MO.0.D	171	0.67058824	15.274.364	2,62E+06	1995108,00	1,10E-01	3,15E+00	TBX1-related factors{6.5.3}	TBX1{6.5.3.0.1}	TBX1;TBX10;TBX15;TBX18;TBX20;TBX22	TBX1
ZFX_HUMAN.H11MO.1.A	181	0.70980392	15.118.368	2,65E+06	1992311,00	2,66E-01	6,21E+00	More than 3 adjacent zinc finger factors{2.3.3}	ZFX/ZFY factors{2.3.3.65}	BCL6;BCL6B;CTCF;CTCFL;FEZF1;FEZF2;GFI1;GFI1B;GLI1;GLI2;GLI3;GLI4;GLIS1;GLIS2;GLIS3;HKR1;MTF1;MYNN;MZF1;OSR2;OVOL1;OVOL2;PLAG1;PLAGL1;PLAGL2;PRDM1;PRDM14;PRDM6;SCRT1;SCRT2;SNAI1;SNAI2;SNAI3;WT1;YY1;YY2;ZBTB12;ZBTB14;ZBTB18;ZBTB20;ZBTB26;ZBTB42;ZBTB45;ZBTB47;ZBTB48;ZBTB49;ZBTB6;ZBTB7A;ZBTB7B;ZBTB7C;ZFP14;ZFP2;ZFP28;ZFP30;ZFP37;ZFP42;ZFP64;ZFP69;ZFP69B;ZFP82;ZFP91;ZFX;ZIC1;ZIC2;ZIC3;ZIC4;ZIC5;ZIK1;ZIM3;ZKSCAN1;ZKSCAN2;ZKSCAN3;ZKSCAN4;ZNF121;ZNF124;ZNF133;ZNF136;ZNF138;ZNF14;ZNF140;ZNF143;ZNF146;ZNF148;ZNF155;ZNF157;ZNF160;ZNF169;ZNF175;ZNF177;ZNF18;ZNF180;ZNF181;ZNF2;ZNF20;ZNF212;ZNF213;ZNF214;ZNF221;ZNF222;ZNF223;ZNF224;ZNF225;ZNF226;ZNF227;ZNF229;ZNF230;ZNF232;ZNF233;ZNF234;ZNF235;ZNF24;ZNF25;ZNF250;ZNF257;ZNF26;ZNF260;ZNF263;ZNF264;ZNF268;ZNF274;ZNF276;ZNF28;ZNF280A;ZNF280B;ZNF280C;ZNF280D;ZNF281;ZNF282;ZNF283;ZNF284;ZNF285;ZNF286A;ZNF286B;ZNF3;ZNF30;ZNF300;ZNF302;ZNF317;ZNF32;ZNF320;ZNF322;ZNF324;ZNF324B;ZNF329;ZNF331;ZNF333;ZNF33A;ZNF33B;ZNF343;ZNF345;ZNF347;ZNF350;ZNF354A;ZNF354B;ZNF362;ZNF366;ZNF383;ZNF384;ZNF394;ZNF397;ZNF398;ZNF404;ZNF41;ZNF410;ZNF419;ZNF420;ZNF431;ZNF432;ZNF436;ZNF439;ZNF44;ZNF440;ZNF442;ZNF443;ZNF446;ZNF449;ZNF45;ZNF460;ZNF468;ZNF479;ZNF484;ZNF490;ZNF500;ZNF502;ZNF524;ZNF525;ZNF528;ZNF543;ZNF544;ZNF546;ZNF547;ZNF548;ZNF549;ZNF554;ZNF555;ZNF557;ZNF558;ZNF559;ZNF561;ZNF562;ZNF563;ZNF564;ZNF566;ZNF567;ZNF568;ZNF57;ZNF570;ZNF571;ZNF572;ZNF577;ZNF581;ZNF582;ZNF583;ZNF585A;ZNF586;ZNF589;ZNF595;ZNF599;ZNF600;ZNF605;ZNF607;ZNF611;ZNF613;ZNF614;ZNF615;ZNF616;ZNF619;ZNF620;ZNF621;ZNF625;ZNF627;ZNF649;ZNF652;ZNF653;ZNF665;ZNF667;ZNF669;ZNF670;ZNF672;ZNF679;ZNF680;ZNF683;ZNF689;ZNF692;ZNF701;ZNF705D;ZNF705E;ZNF705G;ZNF708;ZNF709;ZNF71;ZNF710;ZNF713;ZNF721;ZNF727;ZNF729;ZNF736;ZNF75A;ZNF75D;ZNF76;ZNF763;ZNF764;ZNF765;ZNF768;ZNF77;ZNF771;ZNF773;ZNF774;ZNF776;ZNF777;ZNF780A;ZNF780B;ZNF782;ZNF785;ZNF799;ZNF805;ZNF808;ZNF81;ZNF813;ZNF816;ZNF823;ZNF829;ZNF836;ZNF841;ZNF844;ZNF845;ZNF846;ZNF85;ZNF853;ZNF860;ZNF878;ZNF891;ZNF99;ZSCAN16;ZSCAN2;ZSCAN22;ZSCAN23;ZSCAN29;ZSCAN31;ZSCAN32;ZSCAN4;ZSCAN5A;ZSCAN5B;ZSCAN5C;ZSCAN9;ZXDA;ZXDB;ZXDC	ZFX

Motif	Nº of probes	% of probes	lower OR	upper OR	OR	p.value	FDR	TF family	TF subfamily	TF.family.member	TF.subfamily.member
VEZF1_HUMAN.H11MO.1.C	140	0.54901961	15.392.167	2,56E+06	1984598,00	6,28E-02	2,01E+00	Factors with multiple dispersed zinc fingers {2.3.4}	MAZ-like factors{2.3.4.8}	BCL11A;BCL11B;BNC1;BNC2;E4F1;HIC1;HIC2;HINFP;HIVEP1;HIVEP2;HIVEP3;IKZF1;IKZF2;IKZF3;IKZF4;IKZF5;INSM1;INSM2;MAZ;MECOM;PATZ1;PRDM16;PRDM4;REST;RLF;RREB1;SALL1;SALL2;SALL3;SALL4;VEZF1;ZBTB1;ZBTB17;ZBTB2;ZBTB25;ZBTB4;ZFAT;ZNF134;ZNF211;ZNF217;ZNF219;ZNF248;ZNF256;ZNF292;ZNF296;ZNF319;ZNF334;ZNF335;ZNF341;ZNF37A;ZNF382;ZNF417;ZNF418;ZNF423;ZNF467;ZNF510;ZNF512;ZNF512B;ZNF516;ZNF518A;ZNF518B;ZNF521;ZNF526;ZNF532;ZNF536;ZNF552;ZNF574;ZNF587;ZNF587B;ZNF592;ZNF639;ZNF654;ZNF658;ZNF671;ZNF687;ZNF711;ZNF717;ZNF770;ZNF772;ZNF784;ZNF786;ZNF792;ZNF8;ZNF814	MAZ;PATZ1;VEZF1
ZN219_HUMAN.H11MO.0.D	129	0.50588235	15.371.229	2,55E+06	1980920,00	6,53E-02	2,01E+00	Factors with multiple dispersed zinc fingers {2.3.4}	ZNF219-like factors{2.3.4.2}	BCL11A;BCL11B;BNC1;BNC2;E4F1;HIC1;HIC2;HINFP;HIVEP1;HIVEP2;HIVEP3;IKZF1;IKZF2;IKZF3;IKZF4;IKZF5;INSM1;INSM2;MAZ;MECOM;PATZ1;PRDM16;PRDM4;REST;RLF;RREB1;SALL1;SALL2;SALL3;SALL4;VEZF1;ZBTB1;ZBTB17;ZBTB2;ZBTB25;ZBTB4;ZFAT;ZNF134;ZNF211;ZNF217;ZNF219;ZNF248;ZNF256;ZNF292;ZNF296;ZNF319;ZNF334;ZNF335;ZNF341;ZNF37A;ZNF382;ZNF417;ZNF418;ZNF423;ZNF467;ZNF510;ZNF512;ZNF512B;ZNF516;ZNF518A;ZNF518B;ZNF521;ZNF526;ZNF532;ZNF536;ZNF552;ZNF574;ZNF587;ZNF587B;ZNF592;ZNF639;ZNF654;ZNF658;ZNF671;ZNF687;ZNF711;ZNF717;ZNF770;ZNF772;ZNF784;ZNF786;ZNF792;ZNF8;ZNF814	ZNF219
KLF1_HUMAN.H11MO.0.A	165	0.64705882	14.993.189	2,55E+06	1950406,00	2,17E-01	5,40E+00	Three-zinc finger Krüppel-related factors{2.3.1}	Krüppel-like factors{2.3.1.2}	EGR1;EGR2;EGR3;EGR4;KLF1;KLF10;KLF11;KLF12;KLF13;KLF14;KLF15;KLF16;KLF17;KLF2;KLF3;KLF4;KLF5;KLF6;KLF7;KLF8;KLF9;SP1;SP2;SP3;SP4;SP5;SP6;SP7;SP8;SP9	KLF12;KLF13;KLF14;KLF15;KLF16;KLF1;KLF3;KLF4;KLF5;KLF6;KLF8;KLF9
ZN784_HUMAN.H11MO.0.D	55	0.21568627	14.053.725	2,62E+06	1931322,00	5,58E+01	7,54E+02	Factors with multiple dispersed zinc fingers {2.3.4}	unclassified{2.3.4.0}	BCL11A;E4F1;MECOM;HIC1;HIC2;HINFP;IKZF1;INSM1;MAZ;PATZ1;PRDM4;REST;RREB1;SALL4;VEZF1;ZBTB17;ZBTB4;HIVEP1;HIVEP2;ZNF134;ZNF219;ZNF335;ZNF341;ZNF382;ZNF418;ZNF423;ZNF467;ZNF770;ZNF784;ZNF8	E4F1;PRDM4;REST;RREB1;ZBTB17;ZBTB4;ZNF335;ZNF341;ZNF467;ZNF770;ZNF784;ZNF8
TFDP1_HUMAN.H11MO.0.C	142	0.55686275	14.969.183	2,49E+06	1930577,00	1,68E-01	4,48E+00	E2F-related factors{3.3.2}	Dp-1{3.3.2.2}	E2F1;E2F2;E2F3;E2F4;E2F5;E2F6;E2F7;E2F8;TFDP1;TFDP2	TFDP1
KLF9_HUMAN.H11MO.0.C	159	0.62352941	14.804.091	2,50E+06	1919629,00	2,98E-01	6,76E+00	Three-zinc finger Krüppel-related factors{2.3.1}	Krüppel-like factors{2.3.1.2}	EGR1;EGR2;EGR3;EGR4;KLF1;KLF10;KLF11;KLF12;KLF13;KLF14;KLF15;KLF16;KLF17;KLF2;KLF3;KLF4;KLF5;KLF6;KLF7;KLF8;KLF9;SP1;SP2;SP3;SP4;SP5;SP6;SP7;SP8;SP9	KLF12;KLF13;KLF14;KLF15;KLF16;KLF1;KLF3;KLF4;KLF5;KLF6;KLF8;KLF9
KLF13_HUMAN.H11MO.0.D	123	0.48235294	14.862.429	2,47E+06	1916417,00	3,09E-01	6,81E+00	Three-zinc finger Krüppel-related factors{2.3.1}	Krüppel-like factors{2.3.1.2}	EGR1;EGR2;EGR3;EGR4;KLF1;KLF10;KLF11;KLF12;KLF13;KLF14;KLF15;KLF16;KLF17;KLF2;KLF3;KLF4;KLF5;KLF6;KLF7;KLF8;KLF9;SP1;SP2;SP3;SP4;SP5;SP6;SP7;SP8;SP9	KLF12;KLF13;KLF14;KLF15;KLF16;KLF1;KLF3;KLF4;KLF5;KLF6;KLF8;KLF9
EGR4_HUMAN.H11MO.0.D	163	0.63921569	14.685.107	2,49E+06	1908159,00	4,44E-01	9,00E+00	Three-zinc finger Krüppel-related factors{2.3.1}	EGR factors{2.3.1.3}	EGR1;EGR2;EGR3;EGR4;KLF1;KLF10;KLF11;KLF12;KLF13;KLF14;KLF15;KLF16;KLF17;KLF2;KLF3;KLF4;KLF5;KLF6;KLF7;KLF8;KLF9;SP1;SP2;SP3;SP4;SP5;SP6;SP7;SP8;SP9	EGR1;EGR2;EGR3;EGR4
VEZF1_HUMAN.H11MO.0.C	172	0.67450980	14.580.568	2,51E+06	1905907,00	8,27E-01	1,63E+01	Factors with multiple dispersed zinc fingers {2.3.4}	MAZ-like factors{2.3.4.8}	BCL11A;BCL11B;BNC1;BNC2;E4F1;HIC1;HIC2;HINFP;HIVEP1;HIVEP2;HIVEP3;IKZF1;IKZF2;IKZF3;IKZF4;IKZF5;INSM1;INSM2;MAZ;MECOM;PATZ1;PRDM16;PRDM4;REST;RLF;RREB1;SALL1;SALL2;SALL3;SALL4;VEZF1;ZBTB1;ZBTB17;ZBTB2;ZBTB25;ZBTB4;ZFAT;ZNF134;ZNF211;ZNF217;ZNF219;ZNF248;ZNF256;ZNF292;ZNF296;ZNF319;ZNF334;ZNF335;ZNF341;ZNF37A;ZNF382;ZNF417;ZNF418;ZNF423;ZNF467;ZNF510;ZNF512;ZNF512B;ZNF516;ZNF518A;ZNF518B;ZNF521;ZNF526;ZNF532;ZNF536;ZNF552;ZNF574;ZNF587;ZNF587B;ZNF592;ZNF639;ZNF654;ZNF658;ZNF671;ZNF687;ZNF711;ZNF717;ZNF770;ZNF772;ZNF784;ZNF786;ZNF792;ZNF8;ZNF814	MAZ;PATZ1;VEZF1
AP2D_HUMAN.H11MO.0.D	175	0.68627451	14.522.588	2,51E+06	1902945,00	1,10E+00	2,06E+01	AP-2{1.3.1}	AP-2delta{1.3.1.0.4}	TFAP2A;TFAP2B;TFAP2C;TFAP2D	TFAP2D
TBX15_HUMAN.H11MO.0.D	179	0.70196078	14.251.713	2,49E+06	1874308,00	2,59E+00	4,54E+01	TBX1-related factors{6.5.3}	TBX15 (TBX14){6.5.3.0.3}	TBX1;TBX10;TBX15;TBX18;TBX20;TBX22	TBX15
KLF15_HUMAN.H11MO.0.A	183	0.71764706	14.133.962	2,49E+06	1866602,00	4,26E+00	7,15E+01	Three-zinc finger Krüppel-related factors{2.3.1}	Krüppel-like factors{2.3.1.2}	EGR1;EGR2;EGR3;EGR4;KLF1;KLF10;KLF11;KLF12;KLF13;KLF14;KLF15;KLF16;KLF17;KLF2;KLF3;KLF4;KLF5;KLF6;KLF7;KLF8;KLF9;SP1;SP2;SP3;SP4;SP5;SP6;SP7;SP8;SP9	KLF12;KLF13;KLF14;KLF15;KLF16;KLF1;KLF3;KLF4;KLF5;KLF6;KLF8;KLF9

Motif	Nº of probes	% of probes	lower OR	upper OR	OR	p.value	FDR	TF family	TF subfamily	TF.family.member	TF.subfamily.member
EGR1_HUMAN.H11MO.0.A	166	0.65098039	14.248.644	2,43E+06	1854694,00	1,65E+00	3,03E+01	Three-zinc finger Krüppel-related factors{2.3.1}	EGR factors{2.3.1.3}	EGR1;EGR2;EGR3;EGR4;KLF1;KLF10;KLF11;KLF12;KLF13;KLF14;KLF15;KLF16;KLF17;KLF2;KLF3;KLF4;KLF5;KLF6;KLF7;KLF8;KLF9;SP1;SP2;SP3;SP4;SP5;SP6;SP7;SP8;SP9	EGR1;EGR2;EGR3;EGR4
ZSC22_HUMAN.H11MO.0.C	156	0.61176471	14.195.856	2,39E+06	1838231,00	2,05E+00	3,67E+01	More than 3 adjacent zinc finger factors{2.3.3}	unclassified{2.3.3.0}	BCL6B;BCL6;CTCFL;CTCF;FEZF1;GFI1B;GFI1;GLI1;GLI2;GLI3;GLIS1;GLIS2;GLIS3;MTF1;MYNN;MZF1;OSR2;OVOL1;OVOL2;ZNF146;PLAG1;PLAGL1;PRDM14;PRDM1;PRDM6;SCRT1;SCRT2;SNAI1;SNAI2;YY1;YY2;WT1;ZNF324;ZNF354A;ZBTB14;ZBTB18;ZBTB48;ZBTB49;ZBTB7A;ZBTB7B;ZBTB6;ZFP64;ZFP28;ZFP42;ZFP82;ZFX;ZIC1;ZIC2;ZIC3;ZIC4;ZIM3;ZKSCAN1;ZKSCAN3;ZNF121;ZNF136;ZNF140;ZNF143;ZNF148;ZNF214;ZNF232;ZNF250;ZNF257;ZNF260;ZNF263;ZNF264;ZNF274;ZNF281;ZNF282;ZNF317;ZNF320;ZNF322;ZNF329;ZNF331;ZNF333;ZNF350;ZNF384;ZNF394;ZNF410;ZNF436;ZNF449;ZNF490;ZNF502;ZNF524;ZNF528;ZNF547;ZNF549;ZNF554;ZNF563;ZNF582;ZNF586;ZNF589;ZNF652;ZNF667;ZNF680;ZNF708;ZNF713;ZNF768;ZNF816;ZNF18;ZNF41;ZNF76;ZNF85;ZSCAN16;ZSCAN22;ZSCAN31;ZSCAN4	MYNN;MZF1;OSR2;PRDM14;PRDM6;WT1;ZBTB14;ZBTB48;ZBTB49;ZFP64;ZFP28;ZIM3;ZNF121;ZNF250;ZNF257;ZNF263;ZNF274;ZNF317;ZNF320;ZNF329;ZNF331;ZNF394;ZNF449;ZNF502;ZNF528;ZNF547;ZNF549;ZNF554;ZNF586;ZNF589;ZNF667;ZNF680;ZNF708;ZNF713;ZNF768;ZNF18;ZNF85;ZSCAN16;ZSCAN22
KLF16_HUMAN.H11MO.0.D	177	0.69411765	13.873.471	2,41E+06	1821159,00	6,64E+00	1,04E+02	Three-zinc finger Krüppel-related factors{2.3.1}	Krüppel-like factors{2.3.1.2}	EGR1;EGR2;EGR3;EGR4;KLF1;KLF10;KLF11;KLF12;KLF13;KLF14;KLF15;KLF16;KLF17;KLF2;KLF3;KLF4;KLF5;KLF6;KLF7;KLF8;KLF9;SP1;SP2;SP3;SP4;SP5;SP6;SP7;SP8;SP9	KLF12;KLF13;KLF14;KLF15;KLF16;KLF1;KLF3;KLF4;KLF5;KLF6;KLF8;KLF9
EGR2_HUMAN.H11MO.0.A	168	0.65882353	13.949.108	2,38E+06	1818117,00	4,12E+00	7,07E+01	Three-zinc finger Krüppel-related factors{2.3.1}	EGR factors{2.3.1.3}	EGR1;EGR2;EGR3;EGR4;KLF1;KLF10;KLF11;KLF12;KLF13;KLF14;KLF15;KLF16;KLF17;KLF2;KLF3;KLF4;KLF5;KLF6;KLF7;KLF8;KLF9;SP1;SP2;SP3;SP4;SP5;SP6;SP7;SP8;SP9	EGR1;EGR2;EGR3;EGR4
ZN770_HUMAN.H11MO.0.C	178	0.69803922	13.645.942	2,37E+06	1792967,00	1,17E+01	1,73E+02	Factors with multiple dispersed zinc fingers{2.3.4}	unclassified{2.3.4.0}	BCL11A;E4F1;MECOM;HIC1;HIC2;HINFP;IKZF1;INSM1;MAZ;PATZ1;PRDM4;REST;RREB1;SALL4;VEZF1;ZBTB17;ZBTB4;HIVEP1;HIVEP2;ZNF134;ZNF219;ZNF335;ZNF341;ZNF382;ZNF418;ZNF423;ZNF467;ZNF770;ZNF784;ZNF8	E4F1;PRDM4;REST;RREB1;ZBTB17;ZBTB4;ZNF335;ZNF341;ZNF467;ZNF770;ZNF784;ZNF8
E2F7_HUMAN.H11MO.0.B	151	0.59215686	13.862.381	2,32E+06	1791764,00	4,92E+00	8,07E+01	E2F-related factors{3.3.2}	E2F{3.3.2.1}	E2F1;E2F2;E2F3;E2F4;E2F5;E2F6;E2F7;E2F8;TFDP1;TFDP2	E2F1;E2F2;E2F3;E2F4;E2F5;E2F6;E2F7;E2F8
PURA_HUMAN.H11MO.0.D	156	0.61176471	13.816.146	2,33E+06	1789147,00	5,35E+00	8,59E+01	PUR{0.0.5}	Pur-alpha (PURA, PUR1){0.0.5.0.1}	PURA;PURB;PURG	PURA
E2F6_HUMAN.H11MO.0.A	140	0.54901961	13.635.733	2,27E+06	1758027,00	7,32E+00	1,13E+02	E2F-related factors{3.3.2}	E2F{3.3.2.1}	E2F1;E2F2;E2F3;E2F4;E2F5;E2F6;E2F7;E2F8;TFDP1;TFDP2	E2F1;E2F2;E2F3;E2F4;E2F5;E2F6;E2F7;E2F8
FEV_HUMAN.H11MO.0.B	100	0.39215686	13.307.403	2,24E+06	1729208,00	2,97E+01	4,32E+02	Ets-related factors{3.5.2}	Ets-like factors{3.5.2.1}	EHF;ELF1;ELF2;ELF3;ELF4;ELF5;ELK1;ELK3;ELK4;ERF;ERG;ETS1;ETS2;ETV1;ETV2;ETV3;ETV3L;ETV4;ETV5;ETV6;ETV7;FEV;FLI1;GABPA;SPDEF;SPI1;SPIB;SPIC	ERG;ETS1;ETS2;ETV2;ETV3;FEV;FLI1;GABPA
ZEP2_HUMAN.H11MO.0.D	61	0.23921569	12.639.276	2,30E+06	1714968,00	4,86E+02	5,51E+03	Factors with multiple dispersed zinc fingers{2.3.4}	HIV EP-factors{2.3.4.5}	BCL11A;BCL11B;BNC1;BNC2;E4F1;HIC1;HIC2;HINFP;HIVEP1;HIVEP2;HIVEP3;IKZF1;IKZF2;IKZF3;IKZF4;IKZF5;INSM1;INSM2;MAZ;MECOM;PATZ1;PRDM16;PRDM4;REST;RLF;RREB1;SALL1;SALL2;SALL3;SALL4;VEZF1;ZBTB1;ZBTB17;ZBTB2;ZBTB25;ZBTB4;ZFAT;ZNF134;ZNF211;ZNF217;ZNF219;ZNF248;ZNF256;ZNF292;ZNF296;ZNF319;ZNF334;ZNF335;ZNF341;ZNF37A;ZNF382;ZNF417;ZNF418;ZNF423;ZNF467;ZNF510;ZNF512;ZNF512B;ZNF516;ZNF518A;ZNF518B;ZNF521;ZNF526;ZNF532;ZNF536;ZNF552;ZNF574;ZNF587;ZNF587B;ZNF592;ZNF639;ZNF654;ZNF658;ZNF671;ZNF687;ZNF711;ZNF717;ZNF770;ZNF772;ZNF784;ZNF786;ZNF792;ZNF8;ZNF814	HIVEP1;HIVEP2
EGR2_HUMAN.H11MO.1.A	159	0.62352941	13.136.575	2,22E+06	1703340,00	3,23E+01	4,61E+02	Three-zinc finger Krüppel-related factors{2.3.1}	EGR factors{2.3.1.3}	EGR1;EGR2;EGR3;EGR4;KLF1;KLF10;KLF11;KLF12;KLF13;KLF14;KLF15;KLF16;KLF17;KLF2;KLF3;KLF4;KLF5;KLF6;KLF7;KLF8;KLF9;SP1;SP2;SP3;SP4;SP5;SP6;SP7;SP8;SP9	EGR1;EGR2;EGR3;EGR4
ZFX_HUMAN.H11MO.0.A	114	0.44705882	13.175.344	2,19E+06	1702127,00	3,86E+01	5,42E+02	More than 3 adjacent zinc finger factors{2.3.3}	ZFX/ZFY factors{2.3.3.65}	BCL6;BCL6B;CTCF;CTCFL;FEZF1;FEZF2;GFI1;GFI1B;GLI1;GLI2;GLI3;GLI4;GLIS1;GLIS2;GLIS3;HKR1;MTF1;MYNN;MZF1;OSR2;OVOL1;OVOL2;PLAG1;PLAGL1;PLAGL2;PRDM1;PRDM14;PRDM6;SCRT1;SCRT2;SNAI1;SNAI2;SNAI3;WT1;YY1;YY2;ZBTB12;ZBTB14;ZBTB18;ZBTB20;ZBTB26;ZBTB42;ZBTB45;ZBTB47;ZBTB48;ZBTB49;ZBTB6;ZBTB7A;ZBTB7B;ZBTB7C;ZFP14;ZFP2;ZFP28;ZFP30;ZFP37;ZFP42;ZFP64;ZFP69;ZFP69B;ZFP82;ZFP91;ZFX;ZIC1;ZIC2;ZIC3;ZIC4;ZIC5;ZIK1;ZIM3;ZKSCAN1;ZKSCAN2;ZKSCAN3;ZKSCAN4;ZNF121;ZNF124;ZNF133;ZNF136;ZNF138;ZNF14;ZNF140;ZNF143;ZNF146;ZNF148;ZNF155;ZNF157;ZNF160;ZNF169;ZNF175;	ZFX

Motif	Nº of probes	% of probes	lower OR	upper OR	OR	p.value	FDR	TF family	TF subfamily	TF.family.member	TF.subfamily.member
										ZNF177;ZNF18;ZNF180;ZNF181;ZNF2;ZNF20;ZNF212;ZNF213;ZNF214;ZNF221;ZNF222;ZNF223;ZNF224;ZNF225;ZNF226;ZNF227;ZNF229;ZNF230;ZNF232;ZNF233;ZNF234;ZNF235;ZNF24;ZNF25;ZNF250;ZNF257;ZNF26;ZNF260;ZNF263;ZNF264;ZNF268;ZNF274;ZNF276;ZNF28;ZNF280A;ZNF280B;ZNF280C;ZNF280D;ZNF281;ZNF282;ZNF283;ZNF284;ZNF285;ZNF286A;ZNF286B;ZNF3;ZNF30;ZNF300;ZNF302;ZNF317;ZNF32;ZNF320;ZNF322;ZNF324;ZNF324B;ZNF329;ZNF331;ZNF333;ZNF33A;ZNF33B;ZNF343;ZNF345;ZNF347;ZNF350;ZNF354A;ZNF354B;ZNF362;ZNF366;ZNF383;ZNF384;ZNF394;ZNF397;ZNF398;ZNF404;ZNF41;ZNF410;ZNF419;ZNF420;ZNF431;ZNF432;ZNF436;ZNF439;ZNF44;ZNF440;ZNF442;ZNF443;ZNF446;ZNF449;ZNF45;ZNF460;ZNF468;ZNF479;ZNF484;ZNF490;ZNF500;ZNF502;ZNF524;ZNF525;ZNF528;ZNF543;ZNF544;ZNF546;ZNF547;ZNF548;ZNF549;ZNF554;ZNF555;ZNF557;ZNF558;ZNF559;ZNF561;ZNF562;ZNF563;ZNF564;ZNF566;ZNF567;ZNF568;ZNF57;ZNF570;ZNF571;ZNF572;ZNF577;ZNF581;ZNF582;ZNF583;ZNF585A;ZNF586;ZNF589;ZNF595;ZNF599;ZNF600;ZNF605;ZNF607;ZNF611;ZNF613;ZNF614;ZNF615;ZNF616;ZNF619;ZNF620;ZNF621;ZNF625;ZNF627;ZNF649;ZNF652;ZNF653;ZNF665;ZNF667;ZNF669;ZNF670;ZNF672;ZNF679;ZNF680;ZNF683;ZNF689;ZNF692;ZNF701;ZNF705D;ZNF705E;ZNF705G;ZNF708;ZNF709;ZNF71;ZNF710;ZNF713;ZNF721;ZNF727;ZNF729;ZNF736;ZNF75A;ZNF75D;ZNF76;ZNF763;ZNF764;ZNF765;ZNF768;ZNF77;ZNF771;ZNF773;ZNF774;ZNF776;ZNF777;ZNF780A;ZNF780B;ZNF782;ZNF785;ZNF799;ZNF805;ZNF808;ZNF81;ZNF813;ZNF816;ZNF823;ZNF829;ZNF836;ZNF841;ZNF844;ZNF845;ZNF846;ZNF85;ZNF853;ZNF860;ZNF878;ZNF891;ZNF99;ZSCAN16;ZSCAN2;ZSCAN22;ZSCAN23;ZSCAN29;ZSCAN31;ZSCAN32;ZSCAN4;ZSCAN5A;ZSCAN5B;ZSCAN5C;ZSCAN9;ZXDA;ZXDB;ZXDC	
AP2B_HUMAN.H11MO.0.B	138	0.54117647	13.052.143	2,17E+06	1682391,00	4,19E+01	5,77E+02	AP-2{1.3.1}	AP-2beta{1.3.1.0.2}	TFAP2A;TFAP2B;TFAP2C;TFAP2D	TFAP2B
OVOL2_HUMAN.H11MO.0.D	30	0.11764706	11.062.417	2,47E+06	1678992,00	1,12E+04	7,48E+04	More than 3 adjacent zinc finger factors{2.3.3}	OVOL-factors{2.3.3.17}	BCL6;BCL6B;CTCF;CTCFL;FEZF1;FEZF2;GFI1;GFI1B;GLI1;GLI2;GLI3;GLI4;GLIS1;GLIS2;GLIS3;HKR1;MTF1;MYNN;MZF1;OSR2;OVOL1;OVOL2;PLAG1;PLAGL1;PLAGL2;PRDM1;PRDM14;PRDM6;SCRT1;SCRT2;SNAI1;SNAI2;SNAI3;WT1;YY1;YY2;ZBTB12;ZBTB14;ZBTB18;ZBTB20;ZBTB26;ZBTB42;ZBTB45;ZBTB47;ZBTB48;ZBTB49;ZBTB6;ZBTB7A;ZBTB7B;ZBTB7C;ZFP14;ZFP2;ZFP28;ZFP30;ZFP37;ZFP42;ZFP64;ZFP69;ZFP69B;ZFP82;ZFP91;ZFX;ZIC1;ZIC2;ZIC3;ZIC4;ZIC5;ZIK1;ZIM3;ZKSCAN1;ZKSCAN2;ZKSCAN3;ZKSCAN4;ZNF121;ZNF124;ZNF133;ZNF136;ZNF138;ZNF14;ZNF140;ZNF143;ZNF146;ZNF148;ZNF155;ZNF157;ZNF160;ZNF169;ZNF175;ZNF177;ZNF18;ZNF180;ZNF181;ZNF2;ZNF20;ZNF212;ZNF213;ZNF214;ZNF221;ZNF222;ZNF223;ZNF224;ZNF225;ZNF226;ZNF227;ZNF229;ZNF230;ZNF232;ZNF233;ZNF234;ZNF235;ZNF24;ZNF25;ZNF250;ZNF257;ZNF26;ZNF260;ZNF263;ZNF264;ZNF268;ZNF274;ZNF276;ZNF28;ZNF280A;ZNF280B;ZNF280C;ZNF280D;ZNF281;ZNF282;ZNF283;ZNF284;ZNF285;ZNF286A;ZNF286B;ZNF3;ZNF30;ZNF300;ZNF302;ZNF317;ZNF32;ZNF320;ZNF322;ZNF324;ZNF324B;ZNF329;ZNF331;ZNF333;ZNF33A;ZNF33B;ZNF343;ZNF345;ZNF347;ZNF350;ZNF354A;ZNF354B;ZNF362;ZNF366;ZNF383;ZNF384;ZNF394;ZNF397;ZNF398;ZNF404;ZNF41;ZNF410;ZNF419;ZNF420;ZNF431;ZNF432;ZNF436;ZNF439;ZNF44;ZNF440;ZNF442;ZNF443;ZNF446;ZNF449;ZNF45;ZNF460;ZNF468;ZNF479;ZNF484;ZNF490;ZNF500;ZNF502;ZNF524;ZNF525;ZNF528;ZNF543;ZNF544;ZNF546;ZNF547;ZNF548;ZNF549;ZNF554;ZNF555;ZNF557;ZNF558;ZNF559;ZNF561;ZNF562;ZNF563;ZNF564;ZNF566;ZNF567;ZNF568;ZNF57;ZNF570;ZNF571;ZNF572;ZNF577;ZNF581;ZNF582;ZNF583;ZNF585A;ZNF586;ZNF589;ZNF595;ZNF599;ZNF600;ZNF605;ZNF607;ZNF611;ZNF613;ZNF614;ZNF615;ZNF616;ZNF619;ZNF620;ZNF621;ZNF625;ZNF627;ZNF649;ZNF652;ZNF653;ZNF665;ZNF667;ZNF669;ZNF670;ZNF672;Z	OVOL1;OVOL2

Motif	Nº of probes	% of probes	lower OR	upper OR	OR	p.value	FDR	TF family	TF subfamily	TF.family.member	TF.subfamily.member
										NF679;ZNF680;ZNF683;ZNF689;ZNF692;ZNF701;ZNF705D;ZNF705E;ZNF705G;ZNF708;ZNF709;ZNF71;ZNF710;ZNF713;ZNF721;ZNF727;ZNF729;ZNF736;ZNF75A;ZNF75D;ZNF76;ZNF763;ZNF764;ZNF765;ZNF768;ZNF77;ZNF771;ZNF773;ZNF774;ZNF776;ZNF777;ZNF780A;ZNF780B;ZNF782;ZNF785;ZNF799;ZNF805;ZNF808;ZNF81;ZNF813;ZNF816;ZNF823;ZNF829;ZNF836;ZNF841;ZNF844;ZNF845;ZNF846;ZNF85;ZNF853;ZNF860;ZNF878;ZNF891;ZNF99;ZSCAN16;ZSCAN2;ZSCAN22;ZSCAN23;ZSCAN29;ZSCAN31;ZSCAN32;ZSCAN4;ZSCAN5A;ZSCAN5B;ZSCAN5C;ZSCAN9;ZXDA;ZXDB;ZXDC	
SOX9_HUMAN.H11MO.0.B	29	0.11372549	10.932.156	2.467.654	1.670.792	1,41E+04	8,62E+04	SOX-related factors{4.1.1}	Group E{4.1.1.5}	BBX;CIC;HBP1;SOX1;SOX10;SOX11;SOX12;SOX13;SOX14;SOX15;SOX17;SOX18;SOX2;SOX21;SOX3;SOX30;SOX4;SOX5;SOX6;SOX7;SOX8;SOX9;SRY	SOX10;SOX8;SOX9
ZN341_HUMAN.H11MO.0.C	168	0.65882353	12.630.415	2.158.557	1.646.295	1,51E+02	1,94E+03	Factors with multiple dispersed zinc fingers {2.3.4}	unclassified{2.3.4.0}	BCL11A;E4F1;MECOM;HIC1;HIC2;HINFP;IKZF1;INSM1;MAZ;PATZ1;PRDM4;REST;RREB1;SALL4;VEZF1;ZBTB17;ZBTB4;HIVEP1;HIVEP2;ZNF134;ZNF219;ZNF335;ZNF341;ZNF382;ZNF418;ZNF423;ZNF467;ZNF770;ZNF784;ZNF8	E4F1;PRDM4;REST;RREB1;ZBTB17;ZBTB4;ZNF335;ZNF341;ZNF467;ZNF770;ZNF784;ZNF8
FOXP2_HUMAN.H11MO.0.C	31	0.12156863	10.855.150	2.391.223	1.636.695	1,36E+04	8,62E+04	Forkhead box (FOX) factors{3.3.1}	FOXP{3.3.1.16}	FOXA1;FOXA2;FOXA3;FOXB1;FOXB2;FOXC1;FOXC2;FOXD1;FOXD2;FOXD3;FOXD4;FOXD4L1;FOXD4L3;FOXD4L4;FOXD4L5;FOXD4L6;FOXE1;FOXE3;FOXF1;FOXF2;FOXG1;FOXH1;FOXI1;FOXI2;FOXI3;FOXJ1;FOXJ2;FOXJ3;FOXK1;FOXK2;FOXL1;FOXL2;FOXM1;FOXN1;FOXN2;FOXN3;FOXN4;FOXO1;FOXO3;FOXO4;FOXO6;FOXP1;FOXP2;FOXP3;FOXP4;FOXQ1;FOXR1;FOXR2;FOXS1	FOXP1;FOXP2;FOXP3
CTCFL_HUMAN.H11MO.0.A	137	0.53725490	12.637.696	2.101.986	1.628.869	1,27E+02	1,66E+03	More than 3 adjacent zinc finger factors{2.3.3}	CTCF-like factors{2.3.3.50}	BCL6;BCL6B;CTCF;CTCFL;FEZF1;FEZF2;GFI1;GFI1B;GLI1;GLI2;GLI3;GLI4;GLIS1;GLIS2;GLIS3;HKR1;MTF1;MYNN;MZF1;OSR2;OVOL1;OVOL2;PLAG1;PLAGL1;PLAGL2;PRDM1;PRDM14;PRDM6;SCRT1;SCRT2;SNAI1;SNAI2;SNAI3;WT1;YY1;YY2;ZBTB12;ZBTB14;ZBTB18;ZBTB20;ZBTB26;ZBTB42;ZBTB45;ZBTB47;ZBTB48;ZBTB49;ZBTB6;ZBTB7A;ZBTB7B;ZBTB7C;ZFP14;ZFP2;ZFP28;ZFP30;ZFP37;ZFP42;ZFP64;ZFP69;ZFP69B;ZFP82;ZFP91;ZFX;ZIC1;ZIC2;ZIC3;ZIC4;ZIC5;ZIK1;ZIM3;ZKSCAN1;ZKSCAN2;ZKSCAN3;ZKSCAN4;ZNF121;ZNF124;ZNF133;ZNF136;ZNF138;ZNF14;ZNF140;ZNF143;ZNF146;ZNF148;ZNF155;ZNF157;ZNF160;ZNF169;ZNF175;ZNF177;ZNF18;ZNF180;ZNF181;ZNF2;ZNF20;ZNF212;ZNF213;ZNF214;ZNF221;ZNF222;ZNF223;ZNF224;ZNF225;ZNF226;ZNF227;ZNF229;ZNF230;ZNF232;ZNF233;ZNF234;ZNF235;ZNF24;ZNF25;ZNF250;ZNF257;ZNF26;ZNF260;ZNF263;ZNF264;ZNF268;ZNF274;ZNF276;ZNF28;ZNF280A;ZNF280B;ZNF280C;ZNF280D;ZNF281;ZNF282;ZNF283;ZNF284;ZNF285;ZNF286A;ZNF286B;ZNF3;ZNF30;ZNF300;ZNF302;ZNF317;ZNF32;ZNF320;ZNF322;ZNF324;ZNF324B;ZNF329;ZNF331;ZNF333;ZNF33A;ZNF33B;ZNF343;ZNF345;ZNF347;ZNF350;ZNF354A;ZNF354B;ZNF362;ZNF366;ZNF383;ZNF384;ZNF394;ZNF397;ZNF398;ZNF404;ZNF41;ZNF410;ZNF419;ZNF420;ZNF431;ZNF432;ZNF436;ZNF439;ZNF44;ZNF440;ZNF442;ZNF443;ZNF446;ZNF449;ZNF45;ZNF460;ZNF468;ZNF479;ZNF484;ZNF490;ZNF500;ZNF502;ZNF524;ZNF525;ZNF528;ZNF543;ZNF544;ZNF546;ZNF547;ZNF548;ZNF549;ZNF554;ZNF555;ZNF557;ZNF558;ZNF559;ZNF561;ZNF562;ZNF563;ZNF564;ZNF566;ZNF567;ZNF568;ZNF57;ZNF570;ZNF571;ZNF572;ZNF577;ZNF581;ZNF582;ZNF583;ZNF585A;ZNF586;ZNF589;ZNF595;ZNF599;ZNF600;ZNF605;ZNF607;ZNF611;ZNF613;ZNF614;ZNF615;ZNF616;ZNF619;ZNF620;ZNF621;ZNF625;ZNF627;ZNF649;ZNF652;ZNF653;ZNF665;ZNF667;ZNF669;ZNF670;ZNF672;ZNF679;ZNF680;ZNF683;ZNF689;ZNF692;ZNF701;ZNF705D;ZNF705E;ZNF705G;ZNF708;ZNF709;ZNF71;ZNF710;ZNF713;ZNF721;ZNF727;ZNF729;ZNF736;ZNF75A;ZNF75D;ZNF76;ZNF763;ZNF764;ZNF765;ZNF768;ZNF77;ZNF771;ZNF773;ZNF774;ZNF776;ZNF777;ZNF780A;ZNF780B;ZNF782;ZNF785;ZNF799;ZNF805;ZNF808;ZNF81;ZNF813;ZNF816;ZNF823;ZNF829;ZNF836;ZNF841;ZNF844;ZNF845;ZNF846;ZNF85;ZNF853;ZNF860;ZNF878;ZNF891;ZNF99;ZSCAN16;ZSCAN2;ZSCAN22	CTCFL;CTCF

Motif	Nº of probes	% of probes	lower OR	upper OR	OR	p.value	FDR	TF family	TF subfamily	TF.family.member	TF.subfamily.member
										;ZSCAN23;ZSCAN29;ZSCAN31;ZSCAN32;ZSCAN4;ZSCAN5A;ZSCAN5B;ZSCAN5C;ZSCAN9;ZXDA;ZXDB;ZXDC	
PROX1_HUMAN.H11MO.0.D	131	0.51372549	12.631.012	2.098.291	1.627.623	1,12E+02	1,49E+03	HD-PROS factors{3.1.7}	PROX-1{3.1.7.0.1}	PROX1;PROX2	PROX1
ZF64A_HUMAN.H11MO.0.D	156	0.61176471	12.551.652	2.113.249	1.625.423	1,58E+02	2,00E+03	More than 3 adjacent zinc finger factors{2.3.3}	unclassified{2.3.3.0}	BCL6B;BCL6;CTCFL;CTCF;FEZF1;GFI1B;GFI1;GLI1;GLI2;GLI3;GLIS1;GLIS2;GLIS3;MTF1;MYNN;MZF1;OSR2;OVOL1;OVOL2;ZNF146;PLAG1;PLAGL1;PRDM14;PRDM1;PRDM6;SCRT1;SCRT2;SNAI1;SNAI2;YY1;YY2;WT1;ZNF324;ZNF354A;ZBTB14;ZBTB18;ZBTB48;ZBTB49;ZBTB7A;ZBTB7B;ZBTB6;ZFP64;ZFP28;ZFP42;ZFP82;ZFX;ZIC1;ZIC2;ZIC3;ZIC4;ZIM3;ZKSCAN1;ZKSCAN3;ZNF121;ZNF136;ZNF140;ZNF143;ZNF148;ZNF214;ZNF232;ZNF250;ZNF257;ZNF260;ZNF263;ZNF264;ZNF274;ZNF281;ZNF282;ZNF317;ZNF320;ZNF322;ZNF329;ZNF331;ZNF333;ZNF350;ZNF384;ZNF394;ZNF410;ZNF436;ZNF449;ZNF490;ZNF502;ZNF524;ZNF528;ZNF547;ZNF549;ZNF554;ZNF563;ZNF582;ZNF586;ZNF589;ZNF652;ZNF667;ZNF680;ZNF708;ZNF713;ZNF768;ZNF816;ZNF18;ZNF41;ZNF76;ZNF85;ZSCAN16;ZSCAN22;ZSCAN31;ZSCAN4	MYNN;MZF1;OSR2;PRDM14;PRDM6;WT1;ZBTB14;ZBTB48;ZBTB49;ZFP64;ZFP28;ZIM3;ZNF121;ZNF250;ZNF257;ZNF263;ZNF274;ZNF317;ZNF320;ZNF329;ZNF331;ZNF394;ZNF449;ZNF502;ZNF528;ZNF547;ZNF549;ZNF554;ZNF586;ZNF589;ZNF667;ZNF680;ZNF708;ZNF713;ZNF768;ZNF18;ZNF85;ZSCAN16;ZSCAN22
SOX10_HUMAN.H11MO.0.B	23	0.09019608	10.093.854	2.500.703	1.625.170	3,08E+04	1,59E+05	SOX-related factors{4.1.1}	Group E{4.1.1.5}	BBX;CIC;HBP1;SOX1;SOX10;SOX11;SOX12;SOX13;SOX14;SOX15;SOX17;SOX18;SOX2;SOX21;SOX3;SOX30;SOX4;SOX5;SOX6;SOX7;SOX8;SOX9;SRY	SOX10;SOX8;SOX9
PATZ1_HUMAN.H11MO.1.C	114	0.44705882	12.470.817	2.077.535	1.611.116	1,89E+02	2,35E+03	Factors with multiple dispersed zinc fingers {2.3.4}	MAZ-like factors{2.3.4.8}	BCL11A;BCL11B;BNC1;BNC2;E4F1;HIC1;HIC2;HINFP;HIVEP1;HIVEP2;HIVEP3;IKZF1;IKZF2;IKZF3;IKZF4;IKZF5;INSM1;INSM2;MAZ;MECOM;PATZ1;PRDM16;PRDM4;REST;RLF;RREB1;SALL1;SALL2;SALL3;SALL4;VEZF1;ZBTB1;ZBTB17;ZBTB2;ZBTB25;ZBTB4;ZFAT;ZNF134;ZNF211;ZNF217;ZNF219;ZNF248;ZNF256;ZNF292;ZNF296;ZNF319;ZNF334;ZNF335;ZNF341;ZNF37A;ZNF382;ZNF417;ZNF418;ZNF423;ZNF467;ZNF510;ZNF512;ZNF512B;ZNF516;ZNF518A;ZNF518B;ZNF521;ZNF526;ZNF532;ZNF536;ZNF552;ZNF574;ZNF587;ZNF587B;ZNF592;ZNF639;ZNF654;ZNF658;ZNF671;ZNF687;ZNF711;ZNF717;ZNF770;ZNF772;ZNF784;ZNF786;ZNF792;ZNF8;ZNF814	MAZ;PATZ1;VEZF1
NR1H4_HUMAN.H11MO.0.B	110	0.43137255	12.373.202	2.065.470	1.600.540	2,74E+02	3,25E+03	Thyroid hormone receptor-related factors (NR1){2.1.2}	LXR (NR1H){2.1.2.7}	NR1D1;NR1D2;NR1H2;NR1H3;NR1H4;NR1I2;NR1I3;PPARA;PPARD;PPARG;RARA;RARB;RARG;RORA;RORB;RORC;THRA;THRB;VDR	NR1H2;NR1H3;NR1H4
RELB_HUMAN.H11MO.0.C	37	0.14509804	10.934.129	2.269.319	1.595.093	1,39E+04	8,62E+04	NF-kappaB-related factors{6.1.1}	NF-kappaB p65 subunit-like factors{6.1.1.2}	NFKB1;NFKB2;REL;RELA;RELB	RELB;REL;RELA
ZBT17_HUMAN.H11MO.0.A	157	0.61568627	12.297.488	2.072.404	1.593.194	2,67E+02	3,25E+03	Factors with multiple dispersed zinc fingers {2.3.4}	unclassified{2.3.4.0}	BCL11A;E4F1;MECOM;HIC1;HIC2;HINFP;IKZF1;INSM1;MAZ;PATZ1;PRDM4;REST;RREB1;SALL4;VEZF1;ZBTB17;ZBTB4;HIVEP1;HIVEP2;ZNF134;ZNF219;ZNF335;ZNF341;ZNF382;ZNF418;ZNF423;ZNF467;ZNF770;ZNF784;ZNF8	E4F1;PRDM4;REST;RREB1;ZBTB17;ZBTB4;ZNF335;ZNF341;ZNF467;ZNF770;ZNF784;ZNF8
ZIC4_HUMAN.H11MO.0.D	103	0.40392157	12.159.306	2.039.812	1.577.535	4,68E+02	5,38E+03	More than 3 adjacent zinc finger factors{2.3.3}	GLI-like factors{2.3.3.1}	BCL6;BCL6B;CTCF;CTCFL;FEZF1;FEZF2;GFI1;GFI1B;GLI1;GLI2;GLI3;GLI4;GLIS1;GLIS2;GLIS3;HKR1;MTF1;MYNN;MZF1;OSR2;OVOL1;OVOL2;PLAG1;PLAGL1;PLAGL2;PRDM1;PRDM14;PRDM6;SCRT1;SCRT2;SNAI1;SNAI2;SNAI3;WT1;YY1;YY2;ZBTB12;ZBTB14;ZBTB18;ZBTB20;ZBTB26;ZBTB42;ZBTB45;ZBTB47;ZBTB48;ZBTB49;ZBTB6;ZBTB7A;ZBTB7B;ZBTB7C;ZFP14;ZFP2;ZFP28;ZFP30;ZFP37;ZFP42;ZFP64;ZFP69;ZFP69B;ZFP82;ZFP91;ZFX;ZIC1;ZIC2;ZIC3;ZIC4;ZIC5;ZIK1;ZIM3;ZKSCAN1;ZKSCAN2;ZKSCAN3;ZKSCAN4;ZNF121;ZNF124;ZNF133;ZNF136;ZNF138;ZNF14;ZNF140;ZNF143;ZNF146;ZNF148;ZNF155;ZNF157;ZNF160;ZNF169;ZNF175;ZNF177;ZNF18;ZNF180;ZNF181;ZNF2;ZNF20;ZNF212;ZNF213;ZNF214;ZNF221;ZNF222;ZNF223;ZNF224;ZNF225;ZNF226;ZNF227;ZNF229;ZNF230;ZNF232;ZNF233;ZNF234;ZNF235;ZNF24;ZNF25;ZNF250;ZNF257;ZNF26;ZNF260;ZNF263;ZNF264;ZNF268;ZNF274;ZNF276;ZNF28;ZNF280A;ZNF280B;ZNF280C;ZNF280D;ZNF281;ZNF282;ZNF283;ZNF284;ZNF285;ZNF286A;ZNF286B;ZNF3;ZNF30;ZNF300;ZNF302;ZNF317;ZNF32;ZNF320;ZNF322;ZNF324;ZNF324B;ZNF329;ZNF331;ZNF333;	GLI1;GLI2;GLI3;GLIS1;GLIS2;GLIS3;ZIC1;ZIC2;ZIC3;ZIC4

Motif	Nº of probes	% of probes	lower OR	upper OR	OR	p.value	FDR	TF family	TF subfamily	TF.family.member	TF.subfamily.member
										ZNF33A;ZNF33B;ZNF343;ZNF345;ZNF347;ZNF350;ZNF354A;ZNF354B;ZNF362;ZNF366;ZNF383;ZNF384;ZNF394;ZNF397;ZNF398;ZNF404;ZNF41;ZNF410;ZNF419;ZNF420;ZNF431;ZNF432;ZNF436;ZNF439;ZNF44;ZNF440;ZNF442;ZNF443;ZNF446;ZNF449;ZNF45;ZNF460;ZNF468;ZNF479;ZNF484;ZNF490;ZNF500;ZNF502;ZNF524;ZNF525;ZNF528;ZNF543;ZNF544;ZNF546;ZNF547;ZNF548;ZNF549;ZNF554;ZNF555;ZNF557;ZNF558;ZNF559;ZNF561;ZNF562;ZNF563;ZNF564;ZNF566;ZNF567;ZNF568;ZNF57;ZNF570;ZNF571;ZNF572;ZNF577;ZNF581;ZNF582;ZNF583;ZNF585A;ZNF586;ZNF589;ZNF595;ZNF599;ZNF600;ZNF605;ZNF607;ZNF611;ZNF613;ZNF614;ZNF615;ZNF616;ZNF619;ZNF620;ZNF621;ZNF625;ZNF627;ZNF649;ZNF652;ZNF653;ZNF665;ZNF667;ZNF669;ZNF670;ZNF672;ZNF679;ZNF680;ZNF683;ZNF689;ZNF692;ZNF701;ZNF705D;ZNF705E;ZNF705G;ZNF708;ZNF709;ZNF71;ZNF710;ZNF713;ZNF721;ZNF727;ZNF729;ZNF736;ZNF75A;ZNF75D;ZNF76;ZNF763;ZNF764;ZNF765;ZNF768;ZNF77;ZNF771;ZNF773;ZNF774;ZNF776;ZNF777;ZNF780A;ZNF780B;ZNF782;ZNF785;ZNF799;ZNF805;ZNF808;ZNF81;ZNF813;ZNF816;ZNF823;ZNF829;ZNF836;ZNF841;ZNF844;ZNF845;ZNF846;ZNF85;ZNF853;ZNF860;ZNF878;ZNF891;ZNF99;ZSCAN16;ZSCAN2;ZSCAN22;ZSCAN23;ZSCAN29;ZSCAN31;ZSCAN32;ZSCAN4;ZSCAN5A;ZSCAN5B;ZSCAN5C;ZSCAN9;ZXDA;ZXDB;ZXDC	
CXXC1_HUMAN.H11MO.0.D	80	0.31372549	11.927.530	2.063.426	1.574.578	1,19E+03	1,26E+04	CpG-binding proteins{2.6.1}	CpG-binding protein (CXXC1, CFP1, CGBP, PCCX1, PHF18){2.6.1.0.1}	CXXC1;DNMT1;KDM2A;KMT2A;KMT2B;MBD1;TET1	CXXC1
HEY2_HUMAN.H11MO.0.D	132	0.51764706	12.200.383	2.026.921	1.572.076	3,36E+02	3,93E+03	Hairy-related factors{1.2.4}	Hairy-like factors{1.2.4.1}	BHLHE40;BHLHE41;HELT;HES1;HES2;HES3;HES4;HES5;HES6;HES7;HEY1;HEY2;HEYL	BHLHE40;BHLHE41;HES1;HES5;HES7;HEY1;HEY2
NR0B1_HUMAN.H11MO.0.D	104	0.40784314	12.061.337	2.021.807	1.564.092	6,38E+02	6,83E+03	DAX-related receptors (NR0){2.1.7}	DAX1 (NR0B1){2.1.7.0.1}	NR0B1	NR0B1
ZN740_HUMAN.H11MO.0.D	81	0.31764706	11.718.038	2.023.480	1.545.320	1,73E+03	1,78E+04	Other factors with up to three adjacent zinc fingers{2.3.2}	Other three adjacent zinc finger factors{2.3.2.4}	AEBP2;OSR1;OVOL3;ZBTB22;ZBTB32;ZBTB33;ZBTB34;ZBTB37;ZBTB43;ZBTB46;ZBTB5;ZBTB8B;ZFPM2;ZNF174;ZNF396;ZNF414;ZNF446;ZNF487;ZNF511;ZNF580;ZNF705A;ZNF740;ZSCAN1	ZNF740
TAF1_HUMAN.H11MO.0.A	134	0.52549020	11.955.333	1.987.042	1.540.635	5,86E+02	6,36E+03	TCF-7-related factors{4.1.3}	TAF-1 (TAF-2A, TAF(II)250) [1]{4.1.3.0.5}	LEF1;TAF1;TCF7;TCF7L1;TCF7L2	TAF1
NDF2_HUMAN.H11MO.0.B	63	0.24705882	11.140.955	2.012.396	1.506.019	6,76E+03	5,60E+04	Tal-related factors{1.2.3}	Neurogenin / Atonal-like factors{1.2.3.4}	ATOH1;ATOH7;ATOH8;BHLHA15;BHLHA9;BHLHE22;BHLHE23;FERD3L;FIGLA;HAND1;HAND2;LYL1;MESP1;MESP2;MSC;MSGN1;NEUROD1;NEUROD2;NEUROD4;NEUROD6;NEUROG1;NEUROG2;NEUROG3;NHLH1;NHLH2;OLIG1;OLIG2;OLIG3;PTF1A;SCX;TAL1;TAL2;TCF15;TCF21;TCF23;TWIST1;TWIST2	ATOH1;BHLHA15;BHLHE22;BHLHE23;NEUROD1;NEUROD2;NEUROG2;OLIG1;OLIG2;OLIG3
NFKB2_HUMAN.H11MO.0.B	48	0.18823529	10.748.180	2.068.960	1.504.420	1,28E+04	8,16E+04	NF-kappaB-related factors{6.1.1}	NF-kappaB p50 subunit-like factors{6.1.1.1}	NFKB1;NFKB2;REL;RELA;RELB	NFKB1;NFKB2
PBX1_HUMAN.H11MO.1.C	12	0.04705882	0.7648924	2.675.438	1.502.149	1,54E+05	4,35E+05	TALE-type homeo domain factors{3.1.4}	PBX{3.1.4.4}	IRX2;IRX3;MEIS1;MEIS2;MEIS3;PBX1;PBX2;PBX3;PKNOX1;TGIF2LX;TGIF1;TGIF2	PBX1;PBX2;PBX3
GLIS2_HUMAN.H11MO.0.D	75	0.29411765	11.302.662	1.975.879	1.500.698	3,84E+03	3,74E+04	More than 3 adjacent zinc finger factors{2.3.3}	GLI-like factors{2.3.3.1}	BCL6;BCL6B;CTCF;CTCFL;FEZF1;FEZF2;GFI1;GFI1B;GLI1;GLI2;GLI3;GLI4;GLIS1;GLIS2;GLIS3;HKR1;MTF1;MYNN;MZF1;OSR2;OVOL1;OVOL2;PLAG1;PLAGL1;PLAGL2;PRDM1;PRDM14;PRDM6;SCRT1;SCRT2;SNAI1;SNAI2;SNAI3;WT1;YY1;YY2;ZBTB12;ZBTB14;ZBTB18;ZBTB20;ZBTB26;ZBTB42;ZBTB45;ZBTB47;ZBTB48;ZBTB49;ZBTB6;ZBTB7A;ZBTB7B;ZBTB7C;ZFP14;ZFP2;ZFP28;ZFP30;ZFP37;ZFP42;ZFP64;ZFP69;ZFP69B;ZFP82;ZFP91;ZFX;ZIC1;ZIC2;ZIC3;ZIC4;ZIC5;ZIK1;ZIM3;ZKSCAN1;ZKSCAN2;ZKSCAN3;ZKSCAN4;ZNF121;ZNF124;ZNF133;ZNF136;ZNF138;ZNF14;ZNF140;ZNF143;ZNF146;ZNF148;ZNF155;ZNF157;ZNF160;ZNF169;ZNF175;	GLI1;GLI2;GLI3;GLIS1;GLIS2;GLIS3;ZIC1;ZIC2;ZIC3;ZIC4

Motif	Nº of probes	% of probes	lower OR	upper OR	OR	p.value	FDR	TF family	TF subfamily	TF.family.member	TF.subfamily.member
										ZNF177;ZNF18;ZNF180;ZNF181;ZNF2;ZNF20;ZNF212;ZNF213;ZNF214;ZNF221;ZNF222;ZNF223;ZNF224;ZNF225;ZNF226;ZNF227;ZNF229;ZNF230;ZNF232;ZNF233;ZNF234;ZNF235;ZNF24;ZNF25;ZNF250;ZNF257;ZNF26;ZNF260;ZNF263;ZNF264;ZNF268;ZNF274;ZNF276;ZNF28;ZNF280A;ZNF280B;ZNF280C;ZNF280D;ZNF281;ZNF282;ZNF283;ZNF284;ZNF285;ZNF286A;ZNF286B;ZNF3;ZNF30;ZNF300;ZNF302;ZNF317;ZNF32;ZNF320;ZNF322;ZNF324;ZNF324B;ZNF329;ZNF331;ZNF333;ZNF33A;ZNF33B;ZNF343;ZNF345;ZNF347;ZNF350;ZNF354A;ZNF354B;ZNF362;ZNF366;ZNF383;ZNF384;ZNF394;ZNF397;ZNF398;ZNF404;ZNF41;ZNF410;ZNF419;ZNF420;ZNF431;ZNF432;ZNF436;ZNF439;ZNF44;ZNF440;ZNF442;ZNF443;ZNF446;ZNF449;ZNF45;ZNF460;ZNF468;ZNF479;ZNF484;ZNF490;ZNF500;ZNF502;ZNF524;ZNF525;ZNF528;ZNF543;ZNF544;ZNF546;ZNF547;ZNF548;ZNF549;ZNF554;ZNF555;ZNF557;ZNF558;ZNF559;ZNF561;ZNF562;ZNF563;ZNF564;ZNF566;ZNF567;ZNF568;ZNF57;ZNF570;ZNF571;ZNF572;ZNF577;ZNF581;ZNF582;ZNF583;ZNF585A;ZNF586;ZNF589;ZNF595;ZNF599;ZNF600;ZNF605;ZNF607;ZNF611;ZNF613;ZNF614;ZNF615;ZNF616;ZNF619;ZNF620;ZNF621;ZNF625;ZNF627;ZNF649;ZNF652;ZNF653;ZNF665;ZNF667;ZNF669;ZNF670;ZNF672;ZNF679;ZNF680;ZNF683;ZNF689;ZNF692;ZNF701;ZNF705D;ZNF705E;ZNF705G;ZNF708;ZNF709;ZNF71;ZNF710;ZNF713;ZNF721;ZNF727;ZNF729;ZNF736;ZNF75A;ZNF75D;ZNF76;ZNF763;ZNF764;ZNF765;ZNF768;ZNF77;ZNF771;ZNF773;ZNF774;ZNF776;ZNF777;ZNF780A;ZNF780B;ZNF782;ZNF785;ZNF799;ZNF805;ZNF808;ZNF81;ZNF813;ZNF816;ZNF823;ZNF829;ZNF836;ZNF841;ZNF844;ZNF845;ZNF846;ZNF85;ZNF853;ZNF860;ZNF878;ZNF891;ZNF99;ZSCAN16;ZSCAN2;ZSCAN22;ZSCAN23;ZSCAN29;ZSCAN31;ZSCAN32;ZSCAN4;ZSCAN5A;ZSCAN5B;ZSCAN5C;ZSCAN9;ZXDA;ZXDB;ZXDC	
FOXA3_HUMAN.H11MO.0.B	28	0.10980392	0.9673767	2.212.296	1.489.271	5,78E+04	2,32E+05	Forkhead box (FOX) factors{3.3.1}	FOXA{3.3.1.1}	FOXA1;FOXA2;FOXA3;FOXB1;FOXB2;FOXC1;FOXC2;FOXD1;FOXD2;FOXD3;FOXD4;FOXD4L1;FOXD4L3;FOXD4L4;FOXD4L5;FOXD4L6;FOXE1;FOXE3;FOXF1;FOXF2;FOXG1;FOXH1;FOXI1;FOXI2;FOXI3;FOXJ1;FOXJ2;FOXJ3;FOXK1;FOXK2;FOXL1;FOXL2;FOXM1;FOXN1;FOXN2;FOXN3;FOXN4;FOXO1;FOXO3;FOXO4;FOXO6;FOXP1;FOXP2;FOXP3;FOXP4;FOXQ1;FOXR1;FOXR2;FOXS1	FOXA1;FOXA2;FOXA3
FOXO3_HUMAN.H11MO.0.B	30	0.11764706	0.9794513	2.183.011	1.486.384	5,13E+04	2,15E+05	Forkhead box (FOX) factors{3.3.1}	FOXO{3.3.1.15}	FOXA1;FOXA2;FOXA3;FOXB1;FOXB2;FOXC1;FOXC2;FOXD1;FOXD2;FOXD3;FOXD4;FOXD4L1;FOXD4L3;FOXD4L4;FOXD4L5;FOXD4L6;FOXE1;FOXE3;FOXF1;FOXF2;FOXG1;FOXH1;FOXI1;FOXI2;FOXI3;FOXJ1;FOXJ2;FOXJ3;FOXK1;FOXK2;FOXL1;FOXL2;FOXM1;FOXN1;FOXN2;FOXN3;FOXN4;FOXO1;FOXO3;FOXO4;FOXO6;FOXP1;FOXP2;FOXP3;FOXP4;FOXQ1;FOXR1;FOXR2;FOXS1	FOXO1;FOXO3;FOXO4;FOXO6
NKX22_HUMAN.H11MO.0.D	65	0.25490196	10.945.350	1.964.534	1.474.454	8,12E+03	6,20E+04	NK-related factors{3.1.2}	NK-2.2{3.1.2.15}	BARHL1;BARHL2;BARX1;BARX2;BSX;DBX1;DBX2;DLX1;DLX2;DLX3;DLX4;DLX5;DLX6;EMX1;EMX2;EN1;EN2;HHEX;HLX;HMX1;HMX2;HMX3;LBX1;LBX2;MSX1;MSX2;NANOG;NKX1-1;NKX1-2;NKX2-1;NKX2-2;NKX2-3;NKX2-4;NKX2-5;NKX2-6;NKX2-8;NKX3-1;NKX3-2;NKX6-1;NKX6-2;NKX6-3;NOTO;TLX1;TLX2;TLX3;VAX1;VAX2;VENTX	NKX2-2;NKX2-8
SIX1_HUMAN.H11MO.0.A	23	0.09019608	0.9150075	2.266.389	1.473.134	9,16E+04	3,12E+05	HD-SINE factors{3.1.6}	SIX1-like factors{3.1.6.1}	SIX1;SIX2;SIX3;SIX4;SIX5;SIX6	SIX1;SIX2
HAND1_HUMAN.H11MO.1.D	77	0.30196078	11.109.002	1.933.550	1.471.434	5,58E+03	4,89E+04	Tal-related factors{1.2.3}	Twist-like factors{1.2.3.2}	ATOH1;ATOH7;ATOH8;BHLHA15;BHLHA9;BHLHE22;BHLHE23;FERD3L;FIGLA;HAND1;HAND2;LYL1;MESP1;MESP2;MSC;MSGN1;NEUROD1;NEUROD2;NEUROD4;NEUROD6;NEUROG1;NEUROG2;NEUROG3;NHLH1;NHLH2;OLIG1;OLIG2;OLIG3;PTF1A;SCX;TAL1;TAL2;TCF15;TCF21;TCF23;TWIST1;TWIST2	FIGLA;HAND1;PTF1A;TWIST1
FOXM1_HUMAN.H11MO.0.A	36	0.14117647	10.034.144	2.100.717	1.470.852	3,67E+04	1,75E+05	Forkhead box (FOX) factors{3.3.1}	FOXM{3.3.1.13}	FOXA1;FOXA2;FOXA3;FOXB1;FOXB2;FOXC1;FOXC2;FOXD1;FOXD2;FOXD3;FOXD4;FOXD4L1;FOXD4L3;FOXD4L4;FOXD4L5;FOXD4L6;FOXE1;FOXE3;FOXF1;FOXF2;FOXG1;FOXH1;FOXI1;FOXI2;FOXI3;FOXJ1;FOXJ2;FOXJ3;FOXK1;FOXK2;FOXL1;FOXL2;FOXM1;FOXN1;FOXN2;FOXN3;FOXN4	FOXM1

Motif	Nº of probes	% of probes	lower OR	upper OR	OR	p.value	FDR	TF family	TF subfamily	TF.family.member	TF.subfamily.member
										;FOXO1;FOXO3;FOXO4;FOXO6;FOXP1;FOXP2;FOXP3;FOXP4;FOXQ1;FOXR1;FOXR2;FOXS1	
FOXC2_HUMAN.H11MO.0.D	26	0.10196078	0.9379485	2.208.014	1.467.539	6,81E+04	2,60E+05	Forkhead box (FOX) factors{3.3.1}	FOXC{3.3.1.3}	FOXA1;FOXA2;FOXA3;FOXB1;FOXB2;FOXC1;FOXC2;FOXD1;FOXD2;FOXD3;FOXD4;FOXD4L1;FOXD4L3;FOXD4L4;FOXD4L5;FOXD4L6;FOXE1;FOXE3;FOXF1;FOXF2;FOXG1;FOXH1;FOXI1;FOXI2;FOXI3;FOXJ1;FOXJ2;FOXJ3;FOXK1;FOXK2;FOXL1;FOXL2;FOXM1;FOXN1;FOXN2;FOXN3;FOXN4;FOXO1;FOXO3;FOXO4;FOXO6;FOXP1;FOXP2;FOXP3;FOXP4;FOXQ1;FOXR1;FOXR2;FOXS1	FOXC1;FOXC2
BHA15_HUMAN.H11MO.0.B	88	0.34509804	11.182.286	1.908.404	1.465.074	4,42E+03	4,21E+04	Tal-related factors{1.2.3}	Neurogenin / Atonal-like factors{1.2.3.4}	ATOH1;ATOH7;ATOH8;BHLHA15;BHLHA9;BHLHE22;BHLHE23;FERD3L;FIGLA;HAND1;HAND2;LYL1;MESP1;MESP2;MSC;MSGN1;NEUROD1;NEUROD2;NEUROD4;NEUROD6;NEUROG1;NEUROG2;NEUROG3;NHLH1;NHLH2;OLIG1;OLIG2;OLIG3;PTF1A;SCX;TAL1;TAL2;TCF15;TCF21;TCF23;TWIST1;TWIST2	ATOH1;BHLHA15;BHLHE22;BHLHE23;NEUROD1;NEUROD2;NEUROG2;OLIG1;OLIG2;OLIG3
NKX25_HUMAN.H11MO.0.B	62	0.24313725	10.791.465	1.955.727	1.461.432	1,13E+04	7,52E+04	NK-related factors{3.1.2}	NK-4{3.1.2.17}	BARHL1;BARHL2;BARX1;BARX2;BSX;DBX1;DBX2;DLX1;DLX2;DLX3;DLX4;DLX5;DLX6;EMX1;EMX2;EN1;EN2;HHEX;HLX;HMX1;HMX2;HMX3;LBX1;LBX2;MSX1;MSX2;NANOG;NKX1-1;NKX1-2;NKX2-1;NKX2-2;NKX2-3;NKX2-4;NKX2-5;NKX2-6;NKX2-8;NKX3-1;NKX3-2;NKX6-1;NKX6-2;NKX6-3;NOTO;TLX1;TLX2;TLX3;VAX1;VAX2;VENTX	NKX2-3;NKX2-5
ZBT7A_HUMAN.H11MO.0.A	78	0.30588235	10.942.671	1.900.595	1.447.749	7,57E+03	5,90E+04	More than 3 adjacent zinc finger factors{2.3.3}	ZBTB7 factors{2.3.3.8}	BCL6;BCL6B;CTCF;CTCFL;FEZF1;FEZF2;GFI1;GFI1B;GLI1;GLI2;GLI3;GLI4;GLIS1;GLIS2;GLIS3;HKR1;MTF1;MYNN;MZF1;OSR2;OVOL1;OVOL2;PLAG1;PLAGL1;PLAGL2;PRDM1;PRDM14;PRDM6;SCRT1;SCRT2;SNAI1;SNAI2;SNAI3;WT1;YY1;YY2;ZBTB12;ZBTB14;ZBTB18;ZBTB20;ZBTB26;ZBTB42;ZBTB45;ZBTB47;ZBTB48;ZBTB49;ZBTB6;ZBTB7A;ZBTB7B;ZBTB7C;ZFP14;ZFP2;ZFP28;ZFP30;ZFP37;ZFP42;ZFP64;ZFP69;ZFP69B;ZFP82;ZFP91;ZFX;ZIC1;ZIC2;ZIC3;ZIC4;ZIC5;ZIK1;ZIM3;ZKSCAN1;ZKSCAN2;ZKSCAN3;ZKSCAN4;ZNF121;ZNF124;ZNF133;ZNF136;ZNF138;ZNF14;ZNF140;ZNF143;ZNF146;ZNF148;ZNF155;ZNF157;ZNF160;ZNF169;ZNF175;ZNF177;ZNF18;ZNF180;ZNF181;ZNF2;ZNF20;ZNF212;ZNF213;ZNF214;ZNF221;ZNF222;ZNF223;ZNF224;ZNF225;ZNF226;ZNF227;ZNF229;ZNF230;ZNF232;ZNF233;ZNF234;ZNF235;ZNF24;ZNF25;ZNF250;ZNF257;ZNF26;ZNF260;ZNF263;ZNF264;ZNF268;ZNF274;ZNF276;ZNF28;ZNF280A;ZNF280B;ZNF280C;ZNF280D;ZNF281;ZNF282;ZNF283;ZNF284;ZNF285;ZNF286A;ZNF286B;ZNF3;ZNF30;ZNF300;ZNF302;ZNF317;ZNF32;ZNF320;ZNF322;ZNF324;ZNF324B;ZNF329;ZNF331;ZNF333;ZNF33A;ZNF33B;ZNF343;ZNF345;ZNF347;ZNF350;ZNF354A;ZNF354B;ZNF362;ZNF366;ZNF383;ZNF384;ZNF394;ZNF397;ZNF398;ZNF404;ZNF41;ZNF410;ZNF419;ZNF420;ZNF431;ZNF432;ZNF436;ZNF439;ZNF44;ZNF440;ZNF442;ZNF443;ZNF446;ZNF449;ZNF45;ZNF460;ZNF468;ZNF479;ZNF484;ZNF490;ZNF500;ZNF502;ZNF524;ZNF525;ZNF528;ZNF543;ZNF544;ZNF546;ZNF547;ZNF548;ZNF549;ZNF554;ZNF555;ZNF557;ZNF558;ZNF559;ZNF561;ZNF562;ZNF563;ZNF564;ZNF566;ZNF567;ZNF568;ZNF57;ZNF570;ZNF571;ZNF572;ZNF577;ZNF581;ZNF582;ZNF583;ZNF585A;ZNF586;ZNF589;ZNF595;ZNF599;ZNF600;ZNF605;ZNF607;ZNF611;ZNF613;ZNF614;ZNF615;ZNF616;ZNF619;ZNF620;ZNF621;ZNF625;ZNF627;ZNF649;ZNF652;ZNF653;ZNF665;ZNF667;ZNF669;ZNF670;ZNF672;ZNF679;ZNF680;ZNF683;ZNF689;ZNF692;ZNF701;ZNF705D;ZNF705E;ZNF705G;ZNF708;ZNF709;ZNF71;ZNF710;ZNF713;ZNF721;ZNF727;ZNF729;ZNF736;ZNF75A;ZNF75D;ZNF76;ZNF763;ZNF764;ZNF765;ZNF768;ZNF77;ZNF771;ZNF773;ZNF774;ZNF776;ZNF777;ZNF780A;ZNF780B;ZNF782;ZNF785;ZNF799;ZNF805;ZNF808;ZNF81;ZNF813;ZNF816;ZNF823;ZNF829;ZNF836;ZNF841;ZNF844;ZNF845;ZNF846;ZNF85;ZNF853;ZNF860;ZNF878;ZNF891;ZNF99;ZSCAN16;ZSCAN2;ZSCAN22;ZSCAN23;ZSCAN29;ZSCAN31;ZSCAN32;ZSCAN4;ZSCAN5A;ZSCAN5B;ZSCAN5C;ZSCAN9;ZXDA;ZXDB;ZXDC	ZBTB7A;ZBTB7B

Motif	Nº of probes	% of probes	lower OR	upper OR	OR	p.value	FDR	TF family	TF subfamily	TF.family.member	TF.subfamily.member
PAX5_HUMAN.H11MO.0.A	111	0.43529412	11.171.311	1.863.811	1.444.578	4,56E+03	4,28E+04	Paired domain only{3.2.2}	PAX-2-like factors (partial homeobox){3.2.2.2}	PAX1;PAX2;PAX5;PAX8;PAX9	PAX2;PAX5;PAX8
SIX2_HUMAN.H11MO.0.A	25	0.09803922	0.9151144	2.188.634	1.444.543	8,48E+04	2,97E+05	HD-SINE factors{3.1.6}	SIX1-like factors{3.1.6.1}	SIX1;SIX2;SIX3;SIX4;SIX5;SIX6	SIX1;SIX2
GABPA_HUMAN.H11MO.0.A	87	0.34117647	10.983.950	1.877.348	1.440.318	6,83E+03	5,60E+04	Ets-related factors{3.5.2}	Ets-like factors{3.5.2.1}	EHF;ELF1;ELF2;ELF3;ELF4;ELF5;ELK1;ELK3;ELK4;ERF;ERG;ETS1;ETS2;ETV1;ETV2;ETV3;ETV3L;ETV4;ETV5;ETV6;ETV7;FEV;FLI1;GABPA;SPDEF;SPI1;SPIB;SPIC	ERG;ETS1;ETS2;ETV2;ETV3;FEV;FLI1;GABPA
PBX2_HUMAN.H11MO.0.C	14	0.05490196	0.7701978	2.450.710	1.431.075	1,93E+05	4,85E+05	TALE-type homeo domain factors{3.1.4}	PBX{3.1.4.4}	IRX2;IRX3;MEIS1;MEIS2;MEIS3;PBX1;PBX2;PBX3;PKNOX1;TGIF2LX;TGIF1;TGIF2	PBX1;PBX2;PBX3
NRF1_HUMAN.H11MO.0.A	111	0.43529412	11.059.000	1.845.086	1.430.075	5,68E+03	4,92E+04	NRF{0.0.6}	NRF-1 (alpha-pal){0.0.6.0.1}	NRF1	NRF1
NFIC_HUMAN.H11MO.0.A	72	0.28235294	10.727.487	1.888.546	1.429.881	1,18E+04	7,66E+04	Nuclear factor 1{7.1.2}	NF-1C (NF-IC){7.1.2.0.3}	NFIA;NFIB;NFIC	NFIC
ZN134_HUMAN.H11MO.0.C	38	0.14901961	0.9846111	2.026.286	1.429.683	4,45E+04	1,94E+05	Factors with multiple dispersed zinc fingers{2.3.4}	ZNF134-like factors{2.3.4.24}	BCL11A;BCL11B;BNC1;BNC2;E4F1;HIC1;HIC2;HINFP;HIVEP1;HIVEP2;HIVEP3;IKZF1;IKZF2;IKZF3;IKZF4;IKZF5;INSM1;INSM2;MAZ;MECOM;PATZ1;PRDM16;PRDM4;REST;RLF;RREB1;SALL1;SALL2;SALL3;SALL4;VEZF1;ZBTB1;ZBTB17;ZBTB2;ZBTB25;ZBTB4;ZFAT;ZNF134;ZNF211;ZNF217;ZNF219;ZNF248;ZNF256;ZNF292;ZNF296;ZNF319;ZNF334;ZNF335;ZNF341;ZNF37A;ZNF382;ZNF417;ZNF418;ZNF423;ZNF467;ZNF510;ZNF512;ZNF512B;ZNF516;ZNF518A;ZNF518B;ZNF521;ZNF526;ZNF532;ZNF536;ZNF552;ZNF574;ZNF587;ZNF587B;ZNF592;ZNF639;ZNF654;ZNF658;ZNF671;ZNF687;ZNF711;ZNF717;ZNF770;ZNF772;ZNF784;ZNF786;ZNF792;ZNF8;ZNF814	ZNF134
FOXP3_HUMAN.H11MO.0.D	19	0.07450980	0.8448074	2.284.132	1.429.266	1,60E+05	4,41E+05	Forkhead box (FOX) factors{3.3.1}	FOXP{3.3.1.16}	FOXA1;FOXA2;FOXA3;FOXB1;FOXB2;FOXC1;FOXC2;FOXD1;FOXD2;FOXD3;FOXD4;FOXD4L1;FOXD4L3;FOXD4L4;FOXD4L5;FOXD4L6;FOXE1;FOXE3;FOXF1;FOXF2;FOXG1;FOXH1;FOXI1;FOXI2;FOXI3;FOXJ1;FOXJ2;FOXJ3;FOXK1;FOXK2;FOXL1;FOXL2;FOXM1;FOXN1;FOXN2;FOXN3;FOXN4;FOXO1;FOXO3;FOXO4;FOXO6;FOXP1;FOXP2;FOXP3;FOXP4;FOXQ1;FOXR1;FOXR2;FOXS1	FOXP1;FOXP2;FOXP3
PLAG1_HUMAN.H11MO.0.D	109	0.42745098	11.020.461	1.840.799	1.426.115	5,53E+03	4,89E+04	More than 3 adjacent zinc finger factors{2.3.3}	PLAG factors{2.3.3.25}	BCL6;BCL6B;CTCF;CTCFL;FEZF1;FEZF2;GFI1;GFI1B;GLI1;GLI2;GLI3;GLI4;GLIS1;GLIS2;GLIS3;HKR1;MTF1;MYNN;MZF1;OSR2;OVOL1;OVOL2;PLAG1;PLAGL1;PLAGL2;PRDM1;PRDM14;PRDM6;SCRT1;SCRT2;SNAI1;SNAI2;SNAI3;WT1;YY1;YY2;ZBTB12;ZBTB14;ZBTB18;ZBTB20;ZBTB26;ZBTB42;ZBTB45;ZBTB47;ZBTB48;ZBTB49;ZBTB6;ZBTB7A;ZBTB7B;ZBTB7C;ZFP14;ZFP2;ZFP28;ZFP30;ZFP37;ZFP42;ZFP64;ZFP69;ZFP69B;ZFP82;ZFP91;ZFX;ZIC1;ZIC2;ZIC3;ZIC4;ZIC5;ZIK1;ZIM3;ZKSCAN1;ZKSCAN2;ZKSCAN3;ZKSCAN4;ZNF121;ZNF124;ZNF133;ZNF136;ZNF138;ZNF14;ZNF140;ZNF143;ZNF146;ZNF148;ZNF155;ZNF157;ZNF160;ZNF169;ZNF175;ZNF177;ZNF18;ZNF180;ZNF181;ZNF2;ZNF20;ZNF212;ZNF213;ZNF214;ZNF221;ZNF222;ZNF223;ZNF224;ZNF225;ZNF226;ZNF227;ZNF229;ZNF230;ZNF232;ZNF233;ZNF234;ZNF235;ZNF24;ZNF25;ZNF250;ZNF257;ZNF26;ZNF260;ZNF263;ZNF264;ZNF268;ZNF274;ZNF276;ZNF28;ZNF280A;ZNF280B;ZNF280C;ZNF280D;ZNF281;ZNF282;ZNF283;ZNF284;ZNF285;ZNF286A;ZNF286B;ZNF3;ZNF30;ZNF300;ZNF302;ZNF317;ZNF32;ZNF320;ZNF322;ZNF324;ZNF324B;ZNF329;ZNF331;ZNF333;ZNF33A;ZNF33B;ZNF343;ZNF345;ZNF347;ZNF350;ZNF354A;ZNF354B;ZNF362;ZNF366;ZNF383;ZNF384;ZNF394;ZNF397;ZNF398;ZNF404;ZNF41;ZNF410;ZNF419;ZNF420;ZNF431;ZNF432;ZNF436;ZNF439;ZNF44;ZNF440;ZNF442;ZNF443;ZNF446;ZNF449;ZNF45;ZNF460;ZNF468;ZNF479;ZNF484;ZNF490;ZNF500;ZNF502;ZNF524;ZNF525;ZNF528;ZNF543;ZNF544;ZNF546;ZNF547;ZNF548;ZNF549;ZNF554;ZNF555;ZNF557;ZNF558;ZNF559;ZNF561;ZNF562;ZNF563;ZNF564;ZNF566;ZN	PLAG1;PLAGL1

Motif	Nº of probes	% of probes	lower OR	upper OR	OR	p.value	FDR	TF family	TF subfamily	TF.family.member	TF.subfamily.member
										F567;ZNF568;ZNF57;ZNF570;ZNF571;ZNF572;ZNF577;ZNF581;ZNF582;ZNF583;ZNF585A;ZNF586;ZNF589;ZNF595;ZNF599;ZNF600;ZNF605;ZNF607;ZNF611;ZNF613;ZNF614;ZNF615;ZNF616;ZNF619;ZNF620;ZNF621;ZNF625;ZNF627;ZNF649;ZNF652;ZNF653;ZNF665;ZNF667;ZNF669;ZNF670;ZNF672;ZNF679;ZNF680;ZNF683;ZNF689;ZNF692;ZNF701;ZNF705D;ZNF705E;ZNF705G;ZNF708;ZNF709;ZNF71;ZNF710;ZNF713;ZNF721;ZNF727;ZNF729;ZNF736;ZNF75A;ZNF75D;ZNF76;ZNF763;ZNF764;ZNF765;ZNF768;ZNF77;ZNF771;ZNF773;ZNF774;ZNF776;ZNF777;ZNF780A;ZNF780B;ZNF782;ZNF785;ZNF799;ZNF805;ZNF808;ZNF81;ZNF813;ZNF816;ZNF823;ZNF829;ZNF836;ZNF841;ZNF844;ZNF845;ZNF846;ZNF85;ZNF853;ZNF860;ZNF878;ZNF891;ZNF99;ZSCAN16;ZSCAN2;ZSCAN22;ZSCAN23;ZSCAN29;ZSCAN31;ZSCAN32;ZSCAN4;ZSCAN5A;ZSCAN5B;ZSCAN5C;ZSCAN9;ZXDA;ZXDB;ZXDC	
ZN335_HUMAN.H11MO.0.A	93	0.36470588	10.823.388	1.834.721	1.412.623	8,60E+03	6,26E+04	Factors with multiple dispersed zinc fingers {2.3.4}	unclassified{2.3.4.0}	BCL11A;E4F1;MECOM;HIC1;HIC2;HINFP;IKZF1;INSM1;MAZ;PATZ1;PRDM4;REST;RREB1;SALL4;VEZF1;ZBTB17;ZBTB4;HIVEP1;HIVEP2;ZNF134;ZNF219;ZNF335;ZNF341;ZNF382;ZNF418;ZNF423;ZNF467;ZNF770;ZNF784;ZNF8	E4F1;PRDM4;REST;RREB1;ZBTB17;ZBTB4;ZNF335;ZNF341;ZNF467;ZNF770;ZNF784;ZNF8
NR4A3_HUMAN.H11MO.0.D	32	0.12549020	0.9426241	2.053.059	1.412.320	8,18E+04	2,89E+05	NGFI-B-related receptors (NR4){2.1.4}	NOR1 (NR4A3){2.1.4.0.3}	NR4A1;NR4A2;NR4A3	NR4A3
CENPB_HUMAN.H11MO.0.D	47	0.18431373	10.059.521	1.947.091	1.412.293	3,63E+04	1,75E+05	NA	NA	CENPB	CENPB
GLIS3_HUMAN.H11MO.0.D	76	0.29803922	10.638.890	1.855.595	1.410.839	1,40E+04	8,62E+04	More than 3 adjacent zinc finger factors{2.3.3}	GLI-like factors{2.3.3.1}	BCL6;BCL6B;CTCF;CTCFL;FEZF1;FEZF2;GFI1;GFI1B;GLI1;GLI2;GLI3;GLI4;GLIS1;GLIS2;GLIS3;HKR1;MTF1;MYNN;MZF1;OSR2;OVOL1;OVOL2;PLAG1;PLAGL1;PLAGL2;PRDM1;PRDM14;PRDM6;SCRT1;SCRT2;SNAI1;SNAI2;SNAI3;WT1;YY1;YY2;ZBTB12;ZBTB14;ZBTB18;ZBTB20;ZBTB26;ZBTB42;ZBTB45;ZBTB47;ZBTB48;ZBTB49;ZBTB6;ZBTB7A;ZBTB7B;ZBTB7C;ZFP14;ZFP2;ZFP28;ZFP30;ZFP37;ZFP42;ZFP64;ZFP69;ZFP69B;ZFP82;ZFP91;ZFX;ZIC1;ZIC2;ZIC3;ZIC4;ZIC5;ZIK1;ZIM3;ZKSCAN1;ZKSCAN2;ZKSCAN3;ZKSCAN4;ZNF121;ZNF124;ZNF133;ZNF136;ZNF138;ZNF14;ZNF140;ZNF143;ZNF146;ZNF148;ZNF155;ZNF157;ZNF160;ZNF169;ZNF175;ZNF177;ZNF18;ZNF180;ZNF181;ZNF2;ZNF20;ZNF212;ZNF213;ZNF214;ZNF221;ZNF222;ZNF223;ZNF224;ZNF225;ZNF226;ZNF227;ZNF229;ZNF230;ZNF232;ZNF233;ZNF234;ZNF235;ZNF24;ZNF25;ZNF250;ZNF257;ZNF26;ZNF260;ZNF263;ZNF264;ZNF268;ZNF274;ZNF276;ZNF28;ZNF280A;ZNF280B;ZNF280C;ZNF280D;ZNF281;ZNF282;ZNF283;ZNF284;ZNF285;ZNF286A;ZNF286B;ZNF3;ZNF30;ZNF300;ZNF302;ZNF317;ZNF32;ZNF320;ZNF322;ZNF324;ZNF324B;ZNF329;ZNF331;ZNF333;ZNF33A;ZNF33B;ZNF343;ZNF345;ZNF347;ZNF350;ZNF354A;ZNF354B;ZNF362;ZNF366;ZNF383;ZNF384;ZNF394;ZNF397;ZNF398;ZNF404;ZNF41;ZNF410;ZNF419;ZNF420;ZNF431;ZNF432;ZNF436;ZNF439;ZNF44;ZNF440;ZNF442;ZNF443;ZNF446;ZNF449;ZNF45;ZNF460;ZNF468;ZNF479;ZNF484;ZNF490;ZNF500;ZNF502;ZNF524;ZNF525;ZNF528;ZNF543;ZNF544;ZNF546;ZNF547;ZNF548;ZNF549;ZNF554;ZNF555;ZNF557;ZNF558;ZNF559;ZNF561;ZNF562;ZNF563;ZNF564;ZNF566;ZNF567;ZNF568;ZNF57;ZNF570;ZNF571;ZNF572;ZNF577;ZNF581;ZNF582;ZNF583;ZNF585A;ZNF586;ZNF589;ZNF595;ZNF599;ZNF600;ZNF605;ZNF607;ZNF611;ZNF613;ZNF614;ZNF615;ZNF616;ZNF619;ZNF620;ZNF621;ZNF625;ZNF627;ZNF649;ZNF652;ZNF653;ZNF665;ZNF667;ZNF669;ZNF670;ZNF672;ZNF679;ZNF680;ZNF683;ZNF689;ZNF692;ZNF701;ZNF705D;ZNF705E;ZNF705G;ZNF708;ZNF709;ZNF71;ZNF710;ZNF713;ZNF721;ZNF727;ZNF729;ZNF736;ZNF75A;ZNF75D;ZNF76;ZNF763;ZNF764;ZNF765;ZNF768;ZNF77;ZNF771;ZNF773;ZNF774;ZNF776;ZNF777;ZNF780A;ZNF780B;ZNF782;ZNF785;ZNF799;ZNF805;ZNF808;ZNF81;ZNF813;ZNF816;ZNF823;ZNF829;ZNF836;ZNF841;ZNF844;ZNF845;ZNF846;ZNF85;ZNF853;Z	GLI1;GLI2;GLI3;GLIS1;GLIS2;GLIS3;ZIC1;ZIC2;ZIC3;ZIC4

Motif	Nº of probes	% of probes	lower OR	upper OR	OR	p.value	FDR	TF family	TF subfamily	TF.family.member	TF.subfamily.member
										NF860;ZNF878;ZNF891;ZNF99;ZSCAN16;ZSCAN2;ZSCAN22;ZSCAN23;ZSCAN29;ZSCAN31;ZSCAN32;ZSCAN4;ZSCAN5A;ZSCAN5B;ZSCAN5C;ZSCAN9;ZXDA;ZXDB;ZXDC	
ZN341_HUMAN.H11MO.1.C	58	0.22745098	10.331.661	1.899.501	1.410.238	2,49E+04	1,37E+05	Factors with multiple dispersed zinc fingers{2.3.4}	unclassified{2.3.4.0}	BCL11A;E4F1;MECOM;HIC1;HIC2;HINFP;IKZF1;INSM1;MAZ;PATZ1;PRDM4;REST;RREB1;SALL4;VEZF1;ZBTB17;ZBTB4;HIVEP1;HIVEP2;ZNF134;ZNF219;ZNF335;ZNF341;ZNF382;ZNF418;ZNF423;ZNF467;ZNF770;ZNF784;ZNF8	E4F1;PRDM4;REST;RREB1;ZBTB17;ZBTB4;ZNF335;ZNF341;ZNF467;ZNF770;ZNF784;ZNF8
GLIS1_HUMAN.H11MO.0.D	59	0.23137255	10.347.328	1.895.183	1.409.449	2,59E+04	1,41E+05	More than 3 adjacent zinc finger factors{2.3.3}	GLI-like factors{2.3.3.1}	BCL6;BCL6B;CTCF;CTCFL;FEZF1;FEZF2;GFI1;GFI1B;GLI1;GLI2;GLI3;GLI4;GLIS1;GLIS2;GLIS3;HKR1;MTF1;MYNN;MZF1;OSR2;OVOL1;OVOL2;PLAG1;PLAGL1;PLAGL2;PRDM1;PRDM14;PRDM6;SCRT1;SCRT2;SNAI1;SNAI2;SNAI3;WT1;YY1;YY2;ZBTB12;ZBTB14;ZBTB18;ZBTB20;ZBTB26;ZBTB42;ZBTB45;ZBTB47;ZBTB48;ZBTB49;ZBTB6;ZBTB7A;ZBTB7B;ZBTB7C;ZFP14;ZFP2;ZFP28;ZFP30;ZFP37;ZFP42;ZFP64;ZFP69;ZFP69B;ZFP82;ZFP91;ZFX;ZIC1;ZIC2;ZIC3;ZIC4;ZIC5;ZIK1;ZIM3;ZKSCAN1;ZKSCAN2;ZKSCAN3;ZKSCAN4;ZNF121;ZNF124;ZNF133;ZNF136;ZNF138;ZNF14;ZNF140;ZNF143;ZNF146;ZNF148;ZNF155;ZNF157;ZNF160;ZNF169;ZNF175;ZNF177;ZNF18;ZNF180;ZNF181;ZNF2;ZNF20;ZNF212;ZNF213;ZNF214;ZNF221;ZNF222;ZNF223;ZNF224;ZNF225;ZNF226;ZNF227;ZNF229;ZNF230;ZNF232;ZNF233;ZNF234;ZNF235;ZNF24;ZNF25;ZNF250;ZNF257;ZNF26;ZNF260;ZNF263;ZNF264;ZNF268;ZNF274;ZNF276;ZNF28;ZNF280A;ZNF280B;ZNF280C;ZNF280D;ZNF281;ZNF282;ZNF283;ZNF284;ZNF285;ZNF286A;ZNF286B;ZNF3;ZNF30;ZNF300;ZNF302;ZNF317;ZNF32;ZNF320;ZNF322;ZNF324;ZNF324B;ZNF329;ZNF331;ZNF333;ZNF33A;ZNF33B;ZNF343;ZNF345;ZNF347;ZNF350;ZNF354A;ZNF354B;ZNF362;ZNF366;ZNF383;ZNF384;ZNF394;ZNF397;ZNF398;ZNF404;ZNF41;ZNF410;ZNF419;ZNF420;ZNF431;ZNF432;ZNF436;ZNF439;ZNF44;ZNF440;ZNF442;ZNF443;ZNF446;ZNF449;ZNF45;ZNF460;ZNF468;ZNF479;ZNF484;ZNF490;ZNF500;ZNF502;ZNF524;ZNF525;ZNF528;ZNF543;ZNF544;ZNF546;ZNF547;ZNF548;ZNF549;ZNF554;ZNF555;ZNF557;ZNF558;ZNF559;ZNF561;ZNF562;ZNF563;ZNF564;ZNF566;ZNF567;ZNF568;ZNF57;ZNF570;ZNF571;ZNF572;ZNF577;ZNF581;ZNF582;ZNF583;ZNF585A;ZNF586;ZNF589;ZNF595;ZNF599;ZNF600;ZNF605;ZNF607;ZNF611;ZNF613;ZNF614;ZNF615;ZNF616;ZNF619;ZNF620;ZNF621;ZNF625;ZNF627;ZNF649;ZNF652;ZNF653;ZNF665;ZNF667;ZNF669;ZNF670;ZNF672;ZNF679;ZNF680;ZNF683;ZNF689;ZNF692;ZNF701;ZNF705D;ZNF705E;ZNF705G;ZNF708;ZNF709;ZNF71;ZNF710;ZNF713;ZNF721;ZNF727;ZNF729;ZNF736;ZNF75A;ZNF75D;ZNF76;ZNF763;ZNF764;ZNF765;ZNF768;ZNF77;ZNF771;ZNF773;ZNF774;ZNF776;ZNF777;ZNF780A;ZNF780B;ZNF782;ZNF785;ZNF799;ZNF805;ZNF808;ZNF81;ZNF813;ZNF816;ZNF823;ZNF829;ZNF836;ZNF841;ZNF844;ZNF845;ZNF846;ZNF85;ZNF853;ZNF860;ZNF878;ZNF891;ZNF99;ZSCAN16;ZSCAN2;ZSCAN22;ZSCAN23;ZSCAN29;ZSCAN31;ZSCAN32;ZSCAN4;ZSCAN5A;ZSCAN5B;ZSCAN5C;ZSCAN9;ZXDA;ZXDB;ZXDC	GLI1;GLI2;GLI3;GLIS1;GLIS2;GLIS3;ZIC1;ZIC2;ZIC3;ZIC4
HESX1_HUMAN.H11MO.0.D	47	0.18431373	10.036.048	1.942.527	1.408.998	3,67E+04	1,75E+05	Paired-related HD factors{3.1.3}	HESX{3.1.3.10}	ALX1;ALX3;ALX4;ARGFX;ARX;CRX;DMBX1;DPRX;DRGX;DUX4;DUXA;ESX1;GSC;GSC2;HESX1;ISX;LEUTX;MIXL1;NOBOX;OTP;OTX1;OTX2;PHOX2A;PHOX2B;PITX1;PITX2;PITX3;PROP1;PRRX1;PRRX2;RAX;RAX2;RHOXF1;RHOXF2;SEBOX;SHOX;SHOX2;TPRX1;UNCX;VSX1;VSX2	HESX1
TF65_HUMAN.H11MO.0.A	27	0.10588235	0.9066291	2.102.650	1.406.695	1,00E+05	3,35E+05	NF-kappaB-related factors{6.1.1}	NF-kappaB p65 subunit-like factors{6.1.1.2}	NFKB1;NFKB2;REL;RELA;RELB	RELB;REL;RELA
ELK4_HUMAN.H11MO.0.A	70	0.27450980	10.523.098	1.861.980	1.406.558	1,73E+04	1,03E+05	Ets-related factors{3.5.2}	Elk-like factors{3.5.2.2}	EHF;ELF1;ELF2;ELF3;ELF4;ELF5;ELK1;ELK3;ELK4;ERF;ERG;ETS1;ETS2;ETV1;ETV2;ETV3;ETV3L;ETV4;ETV5;ETV6;ETV7;FEV;FLI1;GABPA;SPDEF;SPI1;SPIB;SPIC	ELK1;ELK3;ELK4;ETV1;ETV4;ETV5
HES1_HUMAN.H11MO.0.D	104	0.40784314	10.845.089	1.817.946	1.406.399	9,12E+03	6,51E+04	Hairy-related factors{1.2.4}	Hairy-like factors{1.2.4.1}	BHLHE40;BHLHE41;HELT;HES1;HES2;HES3;HES4;HES5;HES6;HES7;HEY1;HEY2;HEYL	BHLHE40;BHLHE41;HES1;HES5;HES7;HEY1;HEY2

Motif	Nº of probes	% of probes	lower OR	upper OR	OR	p.value	FDR	TF family	TF subfamily	TF.family.member	TF.subfamily.member
ZEP1_HUMAN.H11MO.0.D	35	0.13725490	0.9526731	2.012.954	1.403.573	7,69E+04	2,84E+05	Factors with multiple dispersed zinc fingers {2.3.4}	HIV EP-factors{2.3.4.5}	BCL11A;BCL11B;BNC1;BNC2;E4F1;HIC1;HIC2;HINFP;HIVEP1;HIVEP2;HIVEP3;IKZF1;IKZF2;IKZF3;IKZF4;IKZF5;INSM1;INSM2;MAZ;MECOM;PATZ1;PRDM16;PRDM4;REST;RLF;RREB1;SALL1;SALL2;SALL3;SALL4;VEZF1;ZBTB1;ZBTB17;ZBTB2;ZBTB25;ZBTB4;ZFAT;ZNF134;ZNF211;ZNF217;ZNF219;ZNF248;ZNF256;ZNF292;ZNF296;ZNF319;ZNF334;ZNF335;ZNF341;ZNF37A;ZNF382;ZNF417;ZNF418;ZNF423;ZNF467;ZNF510;ZNF512;ZNF512B;ZNF516;ZNF518A;ZNF518B;ZNF521;ZNF526;ZNF532;ZNF536;ZNF552;ZNF574;ZNF587;ZNF587B;ZNF592;ZNF639;ZNF654;ZNF658;ZNF671;ZNF687;ZNF711;ZNF717;ZNF770;ZNF772;ZNF784;ZNF786;ZNF792;ZNF8;ZNF814	HIVEP1;HIVEP2
E2F8_HUMAN.H11MO.0.D	28	0.10980392	0.9104976	2.082.159	1.401.679	1,06E+05	3,48E+05	E2F-related factors{3.3.2}	E2F{3.3.2.1}	E2F1;E2F2;E2F3;E2F4;E2F5;E2F6;E2F7;E2F8;TFDP1;TFDP2	E2F1;E2F2;E2F3;E2F4;E2F5;E2F6;E2F7;E2F8
KLF5_HUMAN.H11MO.0.A	133	0.52156863	10.866.306	1.805.582	1.400.240	7,88E+03	6,07E+04	Three-zinc finger Krüppel-related factors{2.3.1}	Krüppel-like factors{2.3.1.2}	EGR1;EGR2;EGR3;EGR4;KLF1;KLF10;KLF11;KLF12;KLF13;KLF14;KLF15;KLF16;KLF17;KLF2;KLF3;KLF4;KLF5;KLF6;KLF7;KLF8;KLF9;SP1;SP2;SP3;SP4;SP5;SP6;SP7;SP8;SP9	KLF12;KLF13;KLF14;KLF15;KLF16;KLF1;KLF3;KLF4;KLF5;KLF6;KLF8;KLF9
HINFP_HUMAN.H11MO.0.C	61	0.23921569	10.302.275	1.873.376	1.397.802	2,84E+04	1,50E+05	Factors with multiple dispersed zinc fingers {2.3.4}	HINFP-like factors{2.3.4.21}	BCL11A;BCL11B;BNC1;BNC2;E4F1;HIC1;HIC2;HINFP;HIVEP1;HIVEP2;HIVEP3;IKZF1;IKZF2;IKZF3;IKZF4;IKZF5;INSM1;INSM2;MAZ;MECOM;PATZ1;PRDM16;PRDM4;REST;RLF;RREB1;SALL1;SALL2;SALL3;SALL4;VEZF1;ZBTB1;ZBTB17;ZBTB2;ZBTB25;ZBTB4;ZFAT;ZNF134;ZNF211;ZNF217;ZNF219;ZNF248;ZNF256;ZNF292;ZNF296;ZNF319;ZNF334;ZNF335;ZNF341;ZNF37A;ZNF382;ZNF417;ZNF418;ZNF423;ZNF467;ZNF510;ZNF512;ZNF512B;ZNF516;ZNF518A;ZNF518B;ZNF521;ZNF526;ZNF532;ZNF536;ZNF552;ZNF574;ZNF587;ZNF587B;ZNF592;ZNF639;ZNF654;ZNF658;ZNF671;ZNF687;ZNF711;ZNF717;ZNF770;ZNF772;ZNF784;ZNF786;ZNF792;ZNF8;ZNF814	HINFP
TEAD3_HUMAN.H11MO.0.D	22	0.08627451	0.8579395	2.166.800	1.396.469	1,55E+05	4,35E+05	TEF-1-related factors{3.6.1}	TEF-5 (TEAD-3, TEAD5){3.6.1.0.4}	TEAD1;TEAD2;TEAD3;TEAD4	TEAD3
KLF4_HUMAN.H11MO.0.A	109	0.42745098	10.758.616	1.796.916	1.392.123	1,02E+04	7,16E+04	Three-zinc finger Krüppel-related factors{2.3.1}	Krüppel-like factors{2.3.1.2}	EGR1;EGR2;EGR3;EGR4;KLF1;KLF10;KLF11;KLF12;KLF13;KLF14;KLF15;KLF16;KLF17;KLF2;KLF3;KLF4;KLF5;KLF6;KLF7;KLF8;KLF9;SP1;SP2;SP3;SP4;SP5;SP6;SP7;SP8;SP9	KLF12;KLF13;KLF14;KLF15;KLF16;KLF1;KLF3;KLF4;KLF5;KLF6;KLF8;KLF9
ETV1_HUMAN.H11MO.0.A	80	0.31372549	10.455.973	1.808.800	1.380.306	2,01E+04	1,18E+05	Ets-related factors{3.5.2}	Elk-like factors{3.5.2.2}	EHF;ELF1;ELF2;ELF3;ELF4;ELF5;ELK1;ELK3;ELK4;ERF;ERG;ETS1;ETS2;ETV1;ETV2;ETV3;ETV3L;ETV4;ETV5;ETV6;ETV7;FEV;FLI1;GABPA;SPDEF;SPI1;SPIB;SPIC	ELK1;ELK3;ELK4;ETV1;ETV4;ETV5
HEN1_HUMAN.H11MO.0.C	110	0.43137255	10.659.573	1.779.331	1.378.800	1,27E+04	8,14E+04	Tal-related factors{1.2.3}	Tal / HEN-like factors{1.2.3.1}	ATOH1;ATOH7;ATOH8;BHLHA15;BHLHA9;BHLHE22;BHLHE23;FERD3L;FIGLA;HAND1;HAND2;LYL1;MESP1;MESP2;MSC;MSGN1;NEUROD1;NEUROD2;NEUROD4;NEUROD6;NEUROG1;NEUROG2;NEUROG3;NHLH1;NHLH2;OLIG1;OLIG2;OLIG3;PTF1A;SCX;TAL1;TAL2;TCF15;TCF21;TCF23;TWIST1;TWIST2	NHLH1;LYL1;TAL1
NOTO_HUMAN.H11MO.0.D	14	0.05490196	0.7418770	2.360.510	1.378.475	2,61E+05	5,66E+05	NK-related factors{3.1.2}	NOTO{3.1.2.20}	BARHL1;BARHL2;BARX1;BARX2;BSX;DBX1;DBX2;DLX1;DLX2;DLX3;DLX4;DLX5;DLX6;EMX1;EMX2;EN1;EN2;HHEX;HLX;HMX1;HMX2;HMX3;LBX1;LBX2;MSX1;MSX2;NANOG;NKX1-1;NKX1-2;NKX2-1;NKX2-2;NKX2-3;NKX2-4;NKX2-5;NKX2-6;NKX2-8;NKX3-1;NKX3-2;NKX6-1;NKX6-2;NKX6-3;NOTO;TLX1;TLX2;TLX3;VAX1;VAX2;VENTX	NOTO
ZN524_HUMAN.H11MO.0.D	65	0.25490196	10.230.875	1.836.150	1.378.179	2,79E+04	1,49E+05	More than 3 adjacent zinc finger factors{2.3.3}	ZNF524-like factors{2.3.3.15}	BCL6;BCL6B;CTCF;CTCFL;FEZF1;FEZF2;GFI1;GFI1B;GLI1;GLI2;GLI3;GLI4;GLIS1;GLIS2;GLIS3;HKR1;MTF1;MYNN;MZF1;OSR2;OVOL1;OVOL2;PLAG1;PLAGL1;PLAGL2;PRDM1;PRDM14;PRDM6;SCRT1;SCRT2;SNAI1;SNAI2;SNAI3;WT1;YY1;YY2;ZBTB12;ZBTB14;ZBTB18;ZBTB20;ZBTB26;ZBTB42;ZBTB45;ZBTB47;ZBTB48;ZBTB49;ZBTB6;ZBTB7A;ZBTB7B;ZBTB7C;ZFP14;ZFP2;ZFP28;ZFP30;ZFP37;ZFP42;ZFP64;ZFP69;ZFP69B;ZFP82;ZFP91;ZFX;ZIC1;ZIC2;ZIC3;ZIC4;ZIC5;ZIK1;ZIM3;ZKSCAN1;ZKSCAN2;ZKSCAN3;ZKSCAN4;ZNF121;ZNF124;ZNF133;ZNF136;ZNF138;ZNF14;ZNF140;ZNF143;ZNF146;ZNF148;ZNF155;ZNF157;ZNF160;ZNF169;ZNF175;ZNF177;ZNF18;ZNF180;ZNF181;ZNF2;ZNF20;ZNF212;ZNF213;ZNF214;ZNF221;ZNF222;ZNF223;ZNF224;ZNF225;ZNF226;	ZNF524

Motif	Nº of probes	% of probes	lower OR	upper OR	OR	p.value	FDR	TF family	TF subfamily	TF.family.member	TF.subfamily.member
										ZNF227;ZNF229;ZNF230;ZNF232;ZNF233;ZNF234;ZNF235;ZNF24;ZNF25;ZNF250;ZNF257;ZNF26;ZNF260;ZNF263;ZNF264;ZNF268;ZNF274;ZNF276;ZNF28;ZNF280A;ZNF280B;ZNF280C;ZNF280D;ZNF281;ZNF282;ZNF283;ZNF284;ZNF285;ZNF286A;ZNF286B;ZNF3;ZNF30;ZNF300;ZNF302;ZNF317;ZNF32;ZNF320;ZNF322;ZNF324;ZNF324B;ZNF329;ZNF331;ZNF333;ZNF33A;ZNF33B;ZNF343;ZNF345;ZNF347;ZNF350;ZNF354A;ZNF354B;ZNF362;ZNF366;ZNF383;ZNF384;ZNF394;ZNF397;ZNF398;ZNF404;ZNF41;ZNF410;ZNF419;ZNF420;ZNF431;ZNF432;ZNF436;ZNF439;ZNF44;ZNF440;ZNF442;ZNF443;ZNF446;ZNF449;ZNF45;ZNF460;ZNF468;ZNF479;ZNF484;ZNF490;ZNF500;ZNF502;ZNF524;ZNF525;ZNF528;ZNF543;ZNF544;ZNF546;ZNF547;ZNF548;ZNF549;ZNF554;ZNF555;ZNF557;ZNF558;ZNF559;ZNF561;ZNF562;ZNF563;ZNF564;ZNF566;ZNF567;ZNF568;ZNF57;ZNF570;ZNF571;ZNF572;ZNF577;ZNF581;ZNF582;ZNF583;ZNF585A;ZNF586;ZNF589;ZNF595;ZNF599;ZNF600;ZNF605;ZNF607;ZNF611;ZNF613;ZNF614;ZNF615;ZNF616;ZNF619;ZNF620;ZNF621;ZNF625;ZNF627;ZNF649;ZNF652;ZNF653;ZNF665;ZNF667;ZNF669;ZNF670;ZNF672;ZNF679;ZNF680;ZNF683;ZNF689;ZNF692;ZNF701;ZNF705D;ZNF705E;ZNF705G;ZNF708;ZNF709;ZNF71;ZNF710;ZNF713;ZNF721;ZNF727;ZNF729;ZNF736;ZNF75A;ZNF75D;ZNF76;ZNF763;ZNF764;ZNF765;ZNF768;ZNF77;ZNF771;ZNF773;ZNF774;ZNF776;ZNF777;ZNF780A;ZNF780B;ZNF782;ZNF785;ZNF799;ZNF805;ZNF808;ZNF81;ZNF813;ZNF816;ZNF823;ZNF829;ZNF836;ZNF841;ZNF844;ZNF845;ZNF846;ZNF85;ZNF853;ZNF860;ZNF878;ZNF891;ZNF99;ZSCAN16;ZSCAN2;ZSCAN22;ZSCAN23;ZSCAN29;ZSCAN31;ZSCAN32;ZSCAN4;ZSCAN5A;ZSCAN5B;ZSCAN5C;ZSCAN9;ZXDA;ZXDB;ZXDC	
MYF6_HUMAN.H11MO.0.C	58	0.22745098	10.091.396	1.855.263	1.377.427	3,92E+04	1,82E+05	MyoD / ASC-related factors{1.2.2}	Myogenic transcription factors{1.2.2.1}	ASCL1;ASCL2;ASCL3;ASCL4;ASCL5;MYF5;MYF6;MYOD1;MYOG	MYF6;MYOD1;MYOG
RFX4_HUMAN.H11MO.0.D	23	0.09019608	0.8551885	2.118.237	1.376.805	1,66E+05	4,50E+05	RFX-related factors{3.3.3}	RFX4{3.3.3.0.4}	RFX1;RFX2;RFX3;RFX4;RFX5;RFX6;RFX7;RFX8	RFX4
FOXD1_HUMAN.H11MO.0.D	21	0.08235294	0.8357016	2.155.251	1.376.457	1,51E+05	4,33E+05	Forkhead box (FOX) factors{3.3.1}	FOXD{3.3.1.4}	FOXA1;FOXA2;FOXA3;FOXB1;FOXB2;FOXC1;FOXC2;FOXD1;FOXD2;FOXD3;FOXD4;FOXD4L1;FOXD4L3;FOXD4L4;FOXD4L5;FOXD4L6;FOXE1;FOXE3;FOXF1;FOXF2;FOXG1;FOXH1;FOXI1;FOXI2;FOXI3;FOXJ1;FOXJ2;FOXJ3;FOXK1;FOXK2;FOXL1;FOXL2;FOXM1;FOXN1;FOXN2;FOXN3;FOXN4;FOXO1;FOXO3;FOXO4;FOXO6;FOXP1;FOXP2;FOXP3;FOXP4;FOXQ1;FOXR1;FOXR2;FOXS1	FOXD1;FOXD2;FOXD3
PBX3_HUMAN.H11MO.1.A	16	0.06274510	0.7722802	2.279.717	1.373.974	2,30E+05	5,40E+05	TALE-type homeo domain factors{3.1.4}	PBX{3.1.4.4}	IRX2;IRX3;MEIS1;MEIS2;MEIS3;PBX1;PBX2;PBX3;PKNOX1;TGIF2LX;TGIF1;TGIF2	PBX1;PBX2;PBX3
BARX1_HUMAN.H11MO.0.D	20	0.07843137	0.8170386	2.155.264	1.363.063	1,82E+05	4,67E+05	NK-related factors{3.1.2}	BARX{3.1.2.2}	BARHL1;BARHL2;BARX1;BARX2;BSX;DBX1;DBX2;DLX1;DLX2;DLX3;DLX4;DLX5;DLX6;EMX1;EMX2;EN1;EN2;HHEX;HLX;HMX1;HMX2;HMX3;LBX1;LBX2;MSX1;MSX2;NANOG;NKX1-1;NKX1-2;NKX2-1;NKX2-2;NKX2-3;NKX2-4;NKX2-5;NKX2-6;NKX2-8;NKX3-1;NKX3-2;NKX6-1;NKX6-2;NKX6-3;NOTO;TLX1;TLX2;TLX3;VAX1;VAX2;VENTX	BARX1;BARX2
ZIC1_HUMAN.H11MO.0.B	89	0.34901961	10.405.379	1.773.240	1.362.154	2,14E+04	1,23E+05	More than 3 adjacent zinc finger factors{2.3.3}	GLI-like factors{2.3.3.1}	BCL6;BCL6B;CTCF;CTCFL;FEZF1;FEZF2;GFI1;GFI1B;GLI1;GLI2;GLI3;GLI4;GLIS1;GLIS2;GLIS3;HKR1;MTF1;MYNN;MZF1;OSR2;OVOL1;OVOL2;PLAG1;PLAGL1;PLAGL2;PRDM1;PRDM14;PRDM6;SCRT1;SCRT2;SNAI1;SNAI2;SNAI3;WT1;YY1;YY2;ZBTB12;ZBTB14;ZBTB18;ZBTB20;ZBTB26;ZBTB42;ZBTB45;ZBTB47;ZBTB48;ZBTB49;ZBTB6;ZBTB7A;ZBTB7B;ZBTB7C;ZFP14;ZFP2;ZFP28;ZFP30;ZFP37;ZFP42;ZFP64;ZFP69;ZFP69B;ZFP82;ZFP91;ZFX;ZIC1;ZIC2;ZIC3;ZIC4;ZIC5;ZIK1;ZIM3;ZKSCAN1;ZKSCAN2;ZKSCAN3;ZKSCAN4;ZNF121;ZNF124;ZNF133;ZNF136;ZNF138;ZNF14;ZNF140;ZNF143;ZNF146;ZNF148;ZNF155;ZNF157;ZNF160;ZNF169;ZNF175;ZNF177;ZNF18;ZNF180;ZNF181;ZNF2;ZNF20;ZNF212;ZNF213;ZNF214;ZNF221;ZNF222;ZNF223;ZNF224;ZNF225;ZNF226;ZNF227;ZNF229;ZNF230;ZNF232;ZNF233;ZNF234;ZNF235;Z	GLI1;GLI2;GLI3;GLIS1;GLIS2;GLIS3;ZIC1;ZIC2;ZIC3;ZIC4

Motif	Nº of probes	% of probes	lower OR	upper OR	OR	p.value	FDR	TF family	TF subfamily	TF.family.member	TF.subfamily.member
										NF24;ZNF25;ZNF250;ZNF257;ZNF26;ZNF260;ZNF263;ZNF264;ZNF268;ZNF274;ZNF276;ZNF28;ZNF280A;ZNF280B;ZNF280C;ZNF280D;ZNF281;ZNF282;ZNF283;ZNF284;ZNF285;ZNF286A;ZNF286B;ZNF3;ZNF30;ZNF300;ZNF302;ZNF317;ZNF32;ZNF320;ZNF322;ZNF324;ZNF324B;ZNF329;ZNF331;ZNF333;ZNF33A;ZNF33B;ZNF343;ZNF345;ZNF347;ZNF350;ZNF354A;ZNF354B;ZNF362;ZNF366;ZNF383;ZNF384;ZNF394;ZNF397;ZNF398;ZNF404;ZNF41;ZNF410;ZNF419;ZNF420;ZNF431;ZNF432;ZNF436;ZNF439;ZNF44;ZNF440;ZNF442;ZNF443;ZNF446;ZNF449;ZNF45;ZNF460;ZNF468;ZNF479;ZNF484;ZNF490;ZNF500;ZNF502;ZNF524;ZNF525;ZNF528;ZNF543;ZNF544;ZNF546;ZNF547;ZNF548;ZNF549;ZNF554;ZNF555;ZNF557;ZNF558;ZNF559;ZNF561;ZNF562;ZNF563;ZNF564;ZNF566;ZNF567;ZNF568;ZNF57;ZNF570;ZNF571;ZNF572;ZNF577;ZNF581;ZNF582;ZNF583;ZNF585A;ZNF586;ZNF589;ZNF595;ZNF599;ZNF600;ZNF605;ZNF607;ZNF611;ZNF613;ZNF614;ZNF615;ZNF616;ZNF619;ZNF620;ZNF621;ZNF625;ZNF627;ZNF649;ZNF652;ZNF653;ZNF665;ZNF667;ZNF669;ZNF670;ZNF672;ZNF679;ZNF680;ZNF683;ZNF689;ZNF692;ZNF701;ZNF705D;ZNF705E;ZNF705G;ZNF708;ZNF709;ZNF71;ZNF710;ZNF713;ZNF721;ZNF727;ZNF729;ZNF736;ZNF75A;ZNF75D;ZNF76;ZNF763;ZNF764;ZNF765;ZNF768;ZNF77;ZNF771;ZNF773;ZNF774;ZNF776;ZNF777;ZNF780A;ZNF780B;ZNF782;ZNF785;ZNF799;ZNF805;ZNF808;ZNF81;ZNF813;ZNF816;ZNF823;ZNF829;ZNF836;ZNF841;ZNF844;ZNF845;ZNF846;ZNF85;ZNF853;ZNF860;ZNF878;ZNF891;ZNF99;ZSCAN16;ZSCAN2;ZSCAN22;ZSCAN23;ZSCAN29;ZSCAN31;ZSCAN32;ZSCAN4;ZSCAN5A;ZSCAN5B;ZSCAN5C;ZSCAN9;ZXDA;ZXDB;ZXDC	
NR2C2_HUMAN.H11MO.0.B	54	0.21176471	0.9864120	1.843.569	1.358.582	5,18E+04	2,16E+05	RXR-related receptors (NR2){2.1.3}	Testicular receptors (NR2C){2.1.3.4}	HNF4A;HNF4G;NR2C1;NR2C2;NR2E1;NR2E3;NR2F1;NR2F2;NR2F6;RXRA;RXRB;RXRG	NR2C1;NR2C2
SPIC_HUMAN.H11MO.0.D	86	0.33725490	10.326.821	1.767.847	1.355.305	2,42E+04	1,35E+05	Ets-related factors{3.5.2}	Spi-like factors{3.5.2.5}	EHF;ELF1;ELF2;ELF3;ELF4;ELF5;ELK1;ELK3;ELK4;ERF;ERG;ETS1;ETS2;ETV1;ETV2;ETV3;ETV3L;ETV4;ETV5;ETV6;ETV7;FEV;FLI1;GABPA;SPDEF;SPI1;SPIB;SPIC	SPI1;SPIB;SPIC
SOX8_HUMAN.H11MO.0.D	16	0.06274510	0.7608529	2.246.016	1.353.672	2,35E+05	5,47E+05	SOX-related factors{4.1.1}	Group E{4.1.1.5}	BBX;CIC;HBP1;SOX1;SOX10;SOX11;SOX12;SOX13;SOX14;SOX15;SOX17;SOX18;SOX2;SOX21;SOX3;SOX30;SOX4;SOX5;SOX6;SOX7;SOX8;SOX9;SRY	SOX10;SOX8;SOX9
ELF2_HUMAN.H11MO.0.C	77	0.30196078	10.206.243	1.776.383	1.351.843	3,36E+04	1,71E+05	Ets-related factors{3.5.2}	Elf-1-like factors{3.5.2.3}	EHF;ELF1;ELF2;ELF3;ELF4;ELF5;ELK1;ELK3;ELK4;ERF;ERG;ETS1;ETS2;ETV1;ETV2;ETV3;ETV3L;ETV4;ETV5;ETV6;ETV7;FEV;FLI1;GABPA;SPDEF;SPI1;SPIB;SPIC	ELF1;ELF2
FOXF2_HUMAN.H11MO.0.D	30	0.11764706	0.8881953	1.979.481	1.347.872	1,25E+05	3,87E+05	Forkhead box (FOX) factors{3.3.1}	FOXF{3.3.1.6}	FOXA1;FOXA2;FOXA3;FOXB1;FOXB2;FOXC1;FOXC2;FOXD1;FOXD2;FOXD3;FOXD4;FOXD4L1;FOXD4L3;FOXD4L4;FOXD4L5;FOXD4L6;FOXE1;FOXE3;FOXF1;FOXF2;FOXG1;FOXH1;FOXI1;FOXI2;FOXI3;FOXJ1;FOXJ2;FOXJ3;FOXK1;FOXK2;FOXL1;FOXL2;FOXM1;FOXN1;FOXN2;FOXN3;FOXN4;FOXO1;FOXO3;FOXO4;FOXO6;FOXP1;FOXP2;FOXP3;FOXP4;FOXQ1;FOXR1;FOXR2;FOXS1	FOXF1;FOXF2
SOX2_HUMAN.H11MO.0.A	44	0.17254902	0.9496300	1.871.381	1.346.439	8,00E+04	2,85E+05	SOX-related factors{4.1.1}	Group B{4.1.1.2}	BBX;CIC;HBP1;SOX1;SOX10;SOX11;SOX12;SOX13;SOX14;SOX15;SOX17;SOX18;SOX2;SOX21;SOX3;SOX30;SOX4;SOX5;SOX6;SOX7;SOX8;SOX9;SRY	SOX1;SOX21;SOX2;SOX3
RREB1_HUMAN.H11MO.0.D	88	0.34509804	10.233.295	1.746.456	1.340.729	3,06E+04	1,59E+05	Factors with multiple dispersed zinc fingers{2.3.4}	unclassified{2.3.4.0}	BCL11A;E4F1;MECOM;HIC1;HIC2;HINFP;IKZF1;INSM1;MAZ;PATZ1;PRDM4;REST;RREB1;SALL4;VEZF1;ZBTB17;ZBTB4;HIVEP1;HIVEP2;ZNF134;ZNF219;ZNF335;ZNF341;ZNF382;ZNF418;ZNF423;ZNF467;ZNF770;ZNF784;ZNF8	E4F1;PRDM4;REST;RREB1;ZBTB17;ZBTB4;ZNF335;ZNF341;ZNF467;ZNF770;ZNF784;ZNF8
E2F3_HUMAN.H11MO.0.A	80	0.31372549	10.153.079	1.756.462	1.340.309	3,66E+04	1,75E+05	E2F-related factors{3.3.2}	E2F{3.3.2.1}	E2F1;E2F2;E2F3;E2F4;E2F5;E2F6;E2F7;E2F8;TFDP1;TFDP2	E2F1;E2F2;E2F3;E2F4;E2F5;E2F6;E2F7;E2F8
ZBTB4_HUMAN.H11MO.1.D	51	0.20000000	0.9648111	1.828.473	1.338.997	7,01E+04	2,66E+05	Factors with multiple dispersed zinc fingers{2.3.4}	unclassified{2.3.4.0}	BCL11A;E4F1;MECOM;HIC1;HIC2;HINFP;IKZF1;INSM1;MAZ;PATZ1;PRDM4;REST;RREB1;SALL4;VEZF1;ZBTB17;ZBTB4;HIVEP1;HIVEP2;ZNF134;ZNF219;ZNF335;ZNF341;ZNF382;ZNF418;ZNF423;ZNF467;ZNF770;ZNF784;ZNF8	E4F1;PRDM4;REST;RREB1;ZBTB17;ZBTB4;ZNF335;ZNF341;ZNF467;ZNF770;ZNF784;ZNF8
ASCL2_HUMAN.H11MO.0.D	77	0.30196078	10.101.936	1.758.223	1.338.026	3,45E+04	1,71E+05	MyoD / ASC-related factors{1.2.2}	Achaete-Scute-like factors{1.2.2.2}	ASCL1;ASCL2;ASCL3;ASCL4;ASCL5;MYF5;MYF6;MYOD1;MYOG	ASCL1;ASCL2

Motif	Nº of probes	% of probes	lower OR	upper OR	OR	p.value	FDR	TF family	TF subfamily	TF.family.member	TF.subfamily.member
REST_HUMAN.H11MO.0.A	75	0.29411765	10.060.589	1.758.677	1.335.757	3,91E+04	1,82E+05	Factors with multiple dispersed zinc fingers {2.3.4}	unclassified{2.3.4.0}	BCL11A;E4F1;MECOM;HIC1;HIC2;HINFP;IKZF1;INSM1;MAZ;PATZ1;PRDM4;REST;RREB1;SALL4;VEZF1;ZBTB17;ZBTB4;HIVEP1;HIVEP2;ZNF134;ZNF219;ZNF335;ZNF341;ZNF382;ZNF418;ZNF423;ZNF467;ZNF770;ZNF784;ZNF8	E4F1;PRDM4;REST;RREB1;ZBTB17;ZBTB4;ZNF335;ZNF341;ZNF467;ZNF770;ZNF784;ZNF8
ESR2_HUMAN.H11MO.0.A	72	0.28235294	10.018.821	1.763.747	1.335.357	4,32E+04	1,92E+05	Steroid hormone receptors (NR3){2.1.1}	ER-like receptors (NR3A&B){2.1.1.2}	AR;ESR1;ESR2;ESRRA;ESRRB;ESRRG;NR3C1;NR3C2;PGR	ESRRA;ESRRB;ESRRG;ESR1;ESR2
SOX10_HUMAN.H11MO.1.A	53	0.20784314	0.9670013	1.815.402	1.335.116	6,29E+04	2,49E+05	SOX-related factors{4.1.1}	Group E{4.1.1.5}	BBX;CIC;HBP1;SOX1;SOX10;SOX11;SOX12;SOX13;SOX14;SOX15;SOX17;SOX18;SOX2;SOX21;SOX3;SOX30;SOX4;SOX5;SOX6;SOX7;SOX8;SOX9;SRY	SOX10;SOX8;SOX9
GCM2_HUMAN.H11MO.0.D	59	0.23137255	0.9790980	1.793.224	1.333.642	6,24E+04	2,48E+05	GCM factors{7.2.1}	GCMb (GCM2){7.2.1.0.2}	GCM1;GCM2	GCM2
MEOX1_HUMAN.H11MO.0.D	17	0.06666667	0.7635117	2.182.642	1.333.596	2,51E+05	5,65E+05	HOX-related factors{3.1.1}	MEOX{3.1.1.14}	CDX1;CDX2;CDX4;EVX1;EVX2;GBX1;GBX2;GSX1;GSX2;HDX;HMBOX1;HNF1A;HNF1B;HOXA1;HOXA10;HOXA11;HOXA13;HOXA2;HOXA3;HOXA4;HOXA5;HOXA6;HOXA7;HOXA9;HOXB1;HOXB13;HOXB2;HOXB3;HOXB4;HOXB5;HOXB6;HOXB7;HOXB8;HOXB9;HOXC10;HOXC11;HOXC12;HOXC13;HOXC4;HOXC5;HOXC6;HOXC8;HOXC9;HOXD1;HOXD10;HOXD11;HOXD12;HOXD13;HOXD3;HOXD4;HOXD8;HOXD9;MEOX1;MEOX2;MNX1;PDX1;POU1F1;POU2F1;POU2F2;POU2F3;POU3F1;POU3F2;POU3F3;POU3F4;POU4F1;POU4F2;POU4F3;POU5F1;POU5F2;POU6F1;POU6F2	MEOX1;MEOX2
ZN816_HUMAN.H11MO.0.C	65	0.25490196	0.9863967	1.770.357	1.328.746	5,20E+04	2,16E+05	More than 3 adjacent zinc finger factors{2.3.3}	ZNF816A-like factors{2.3.3.73}	BCL6;BCL6B;CTCF;CTCFL;FEZF1;FEZF2;GFI1;GFI1B;GLI1;GLI2;GLI3;GLI4;GLIS1;GLIS2;GLIS3;HKR1;MTF1;MYNN;MZF1;OSR2;OVOL1;OVOL2;PLAG1;PLAGL1;PLAGL2;PRDM1;PRDM14;PRDM6;SCRT1;SCRT2;SNAI1;SNAI2;SNAI3;WT1;YY1;YY2;ZBTB12;ZBTB14;ZBTB18;ZBTB20;ZBTB26;ZBTB42;ZBTB45;ZBTB47;ZBTB48;ZBTB49;ZBTB6;ZBTB7A;ZBTB7B;ZBTB7C;ZFP14;ZFP2;ZFP28;ZFP30;ZFP37;ZFP42;ZFP64;ZFP69;ZFP69B;ZFP82;ZFP91;ZFX;ZIC1;ZIC2;ZIC3;ZIC4;ZIC5;ZIK1;ZIM3;ZKSCAN1;ZKSCAN2;ZKSCAN3;ZKSCAN4;ZNF121;ZNF124;ZNF133;ZNF136;ZNF138;ZNF14;ZNF140;ZNF143;ZNF146;ZNF148;ZNF155;ZNF157;ZNF160;ZNF169;ZNF175;ZNF177;ZNF18;ZNF180;ZNF181;ZNF2;ZNF20;ZNF212;ZNF213;ZNF214;ZNF221;ZNF222;ZNF223;ZNF224;ZNF225;ZNF226;ZNF227;ZNF229;ZNF230;ZNF232;ZNF233;ZNF234;ZNF235;ZNF24;ZNF25;ZNF250;ZNF257;ZNF26;ZNF260;ZNF263;ZNF264;ZNF268;ZNF274;ZNF276;ZNF28;ZNF280A;ZNF280B;ZNF280C;ZNF280D;ZNF281;ZNF282;ZNF283;ZNF284;ZNF285;ZNF286A;ZNF286B;ZNF3;ZNF30;ZNF300;ZNF302;ZNF317;ZNF32;ZNF320;ZNF322;ZNF324;ZNF324B;ZNF329;ZNF331;ZNF333;ZNF33A;ZNF33B;ZNF343;ZNF345;ZNF347;ZNF350;ZNF354A;ZNF354B;ZNF362;ZNF366;ZNF383;ZNF384;ZNF394;ZNF397;ZNF398;ZNF404;ZNF41;ZNF410;ZNF419;ZNF420;ZNF431;ZNF432;ZNF436;ZNF439;ZNF44;ZNF440;ZNF442;ZNF443;ZNF446;ZNF449;ZNF45;ZNF460;ZNF468;ZNF479;ZNF484;ZNF490;ZNF500;ZNF502;ZNF524;ZNF525;ZNF528;ZNF543;ZNF544;ZNF546;ZNF547;ZNF548;ZNF549;ZNF554;ZNF555;ZNF557;ZNF558;ZNF559;ZNF561;ZNF562;ZNF563;ZNF564;ZNF566;ZNF567;ZNF568;ZNF57;ZNF570;ZNF571;ZNF572;ZNF577;ZNF581;ZNF582;ZNF583;ZNF585A;ZNF586;ZNF589;ZNF595;ZNF599;ZNF600;ZNF605;ZNF607;ZNF611;ZNF613;ZNF614;ZNF615;ZNF616;ZNF619;ZNF620;ZNF621;ZNF625;ZNF627;ZNF649;ZNF652;ZNF653;ZNF665;ZNF667;ZNF669;ZNF670;ZNF672;ZNF679;ZNF680;ZNF683;ZNF689;ZNF692;ZNF701;ZNF705D;ZNF705E;ZNF705G;ZNF708;ZNF709;ZNF71;ZNF710;ZNF713;ZNF721;ZNF727;ZNF729;ZNF736;ZNF75A;ZNF75D;ZNF76;ZNF763;ZNF764;ZNF765;ZNF768;ZNF77;ZNF771;ZNF773;ZNF774;ZNF776;ZNF777;ZNF780A;ZNF780B;ZNF782;ZNF785;ZNF799;ZNF805;ZNF808;ZNF81;ZNF813;ZNF816;ZNF823;ZNF829;ZNF836;ZNF841;ZNF844;ZNF845;ZNF846;ZNF85;ZNF853;ZNF860;ZNF878;ZNF891;ZNF99;ZSCAN16;ZSCAN2;ZSCAN22;ZSCAN23;ZSCAN29;ZSCAN31;ZSCAN32;ZSCAN4;ZSCAN5A;ZSCAN5B;ZSCAN5C;ZSCAN9;ZXDA;ZXDB;ZXDC	ZNF816

Motif	Nº of probes	% of probes	lower OR	upper OR	OR	p.value	FDR	TF family	TF subfamily	TF.family.member	TF.subfamily.member
RFX1_HUMAN.H11MO.0.B	110	0.43137255	10.260.199	1.712.655	1.327.130	2,67E+04	1,44E+05	RFX-related factors{3.3.3}	RFX1 (EF-C){3.3.3.0.1}	RFX1;RFX2;RFX3;RFX4;RFX5;RFX6;RFX7;RFX8	RFX1
ZN563_HUMAN.H11MO.0.C	85	0.33333333	10.083.755	1.728.950	1.324.597	4,13E+04	1,87E+05	More than 3 adjacent zinc finger factors{2.3.3}	ZNF763-like factors{2.3.3.33}	BCL6;BCL6B;CTCF;CTCFL;FEZF1;FEZF2;GFI1;GFI1B;GLI1;GLI2;GLI3;GLI4;GLIS1;GLIS2;GLIS3;HKR1;MTF1;MYNN;MZF1;OSR2;OVOL1;OVOL2;PLAG1;PLAGL1;PLAGL2;PRDM1;PRDM14;PRDM6;SCRT1;SCRT2;SNAI1;SNAI2;SNAI3;WT1;YY1;YY2;ZBTB12;ZBTB14;ZBTB18;ZBTB20;ZBTB26;ZBTB42;ZBTB45;ZBTB47;ZBTB48;ZBTB49;ZBTB6;ZBTB7A;ZBTB7B;ZBTB7C;ZFP14;ZFP2;ZFP28;ZFP30;ZFP37;ZFP42;ZFP64;ZFP69;ZFP69B;ZFP82;ZFP91;ZFX;ZIC1;ZIC2;ZIC3;ZIC4;ZIC5;ZIK1;ZIM3;ZKSCAN1;ZKSCAN2;ZKSCAN3;ZKSCAN4;ZNF121;ZNF124;ZNF133;ZNF136;ZNF138;ZNF14;ZNF140;ZNF143;ZNF146;ZNF148;ZNF155;ZNF157;ZNF160;ZNF169;ZNF175;ZNF177;ZNF18;ZNF180;ZNF181;ZNF2;ZNF20;ZNF212;ZNF213;ZNF214;ZNF221;ZNF222;ZNF223;ZNF224;ZNF225;ZNF226;ZNF227;ZNF229;ZNF230;ZNF232;ZNF233;ZNF234;ZNF235;ZNF24;ZNF25;ZNF250;ZNF257;ZNF26;ZNF260;ZNF263;ZNF264;ZNF268;ZNF274;ZNF276;ZNF28;ZNF280A;ZNF280B;ZNF280C;ZNF280D;ZNF281;ZNF282;ZNF283;ZNF284;ZNF285;ZNF286A;ZNF286B;ZNF3;ZNF30;ZNF300;ZNF302;ZNF317;ZNF32;ZNF320;ZNF322;ZNF324;ZNF324B;ZNF329;ZNF331;ZNF333;ZNF33A;ZNF33B;ZNF343;ZNF345;ZNF347;ZNF350;ZNF354A;ZNF354B;ZNF362;ZNF366;ZNF383;ZNF384;ZNF394;ZNF397;ZNF398;ZNF404;ZNF41;ZNF410;ZNF419;ZNF420;ZNF431;ZNF432;ZNF436;ZNF439;ZNF44;ZNF440;ZNF442;ZNF443;ZNF446;ZNF449;ZNF45;ZNF460;ZNF468;ZNF479;ZNF484;ZNF490;ZNF500;ZNF502;ZNF524;ZNF525;ZNF528;ZNF543;ZNF544;ZNF546;ZNF547;ZNF548;ZNF549;ZNF554;ZNF555;ZNF557;ZNF558;ZNF559;ZNF561;ZNF562;ZNF563;ZNF564;ZNF566;ZNF567;ZNF568;ZNF57;ZNF570;ZNF571;ZNF572;ZNF577;ZNF581;ZNF582;ZNF583;ZNF585A;ZNF586;ZNF589;ZNF595;ZNF599;ZNF600;ZNF605;ZNF607;ZNF611;ZNF613;ZNF614;ZNF615;ZNF616;ZNF619;ZNF620;ZNF621;ZNF625;ZNF627;ZNF649;ZNF652;ZNF653;ZNF665;ZNF667;ZNF669;ZNF670;ZNF672;ZNF679;ZNF680;ZNF683;ZNF689;ZNF692;ZNF701;ZNF705D;ZNF705E;ZNF705G;ZNF708;ZNF709;ZNF71;ZNF710;ZNF713;ZNF721;ZNF727;ZNF729;ZNF736;ZNF75A;ZNF75D;ZNF76;ZNF763;ZNF764;ZNF765;ZNF768;ZNF77;ZNF771;ZNF773;ZNF774;ZNF776;ZNF777;ZNF780A;ZNF780B;ZNF782;ZNF785;ZNF799;ZNF805;ZNF808;ZNF81;ZNF813;ZNF816;ZNF823;ZNF829;ZNF836;ZNF841;ZNF844;ZNF845;ZNF846;ZNF85;ZNF853;ZNF860;ZNF878;ZNF891;ZNF99;ZSCAN16;ZSCAN2;ZSCAN22;ZSCAN23;ZSCAN29;ZSCAN31;ZSCAN32;ZSCAN4;ZSCAN5A;ZSCAN5B;ZSCAN5C;ZSCAN9;ZXDA;ZXDB;ZXDC	ZNF136;ZNF490;ZNF563
FOXJ2_HUMAN.H11MO.0.C	37	0.14509804	0.9022130	1.872.174	1.316.078	1,38E+05	4,13E+05	Forkhead box (FOX) factors{3.3.1}	FOXJ{3.3.1.10}	FOXA1;FOXA2;FOXA3;FOXB1;FOXB2;FOXC1;FOXC2;FOXD1;FOXD2;FOXD3;FOXD4;FOXD4L1;FOXD4L3;FOXD4L4;FOXD4L5;FOXD4L6;FOXE1;FOXE3;FOXF1;FOXF2;FOXG1;FOXH1;FOXI1;FOXI2;FOXI3;FOXJ1;FOXJ2;FOXJ3;FOXK1;FOXK2;FOXL1;FOXL2;FOXM1;FOXN1;FOXN2;FOXN3;FOXN4;FOXO1;FOXO3;FOXO4;FOXO6;FOXP1;FOXP2;FOXP3;FOXP4;FOXQ1;FOXR1;FOXR2;FOXS1	FOXJ2;FOXJ3
SPZ1_HUMAN.H11MO.0.D	56	0.21960784	0.9594520	1.778.027	1.315.304	7,07E+04	2,67E+05	NA	NA	SPZ1	SPZ1
FOXA1_HUMAN.H11MO.0.A	31	0.12156863	0.8707769	1.917.796	1.312.761	1,64E+05	4,48E+05	Forkhead box (FOX) factors{3.3.1}	FOXA{3.3.1.1}	FOXA1;FOXA2;FOXA3;FOXB1;FOXB2;FOXC1;FOXC2;FOXD1;FOXD2;FOXD3;FOXD4;FOXD4L1;FOXD4L3;FOXD4L4;FOXD4L5;FOXD4L6;FOXE1;FOXE3;FOXF1;FOXF2;FOXG1;FOXH1;FOXI1;FOXI2;FOXI3;FOXJ1;FOXJ2;FOXJ3;FOXK1;FOXK2;FOXL1;FOXL2;FOXM1;FOXN1;FOXN2;FOXN3;FOXN4;FOXO1;FOXO3;FOXO4;FOXO6;FOXP1;FOXP2;FOXP3;FOXP4;FOXQ1;FOXR1;FOXR2;FOXS1	FOXA1;FOXA2;FOXA3
ISL1_HUMAN.H11MO.0.A	15	0.05882353	0.7214345	2.206.504	1.310.121	2,92E+05	6,03E+05	HD-LIM factors{3.1.5}	ISL{3.1.5.1}	ISL1;ISL2;LHX1;LHX2;LHX3;LHX4;LHX5;LHX6;LHX8;LHX9;LMX1A;LMX1B	ISL1;ISL2

Motif	Nº of probes	% of probes	lower OR	upper OR	OR	p.value	FDR	TF family	TF subfamily	TF.family.member	TF.subfamily.member
NGN2_HUMAN.H11MO.0.D	60	0.23529412	0.9595911	1.751.115	1.304.458	7,90E+04	2,85E+05	Tal-related factors{1.2.3}	Neurogenin / Atonal-like factors{1.2.3.4}	ATOH1;ATOH7;ATOH8;BHLHA15;BHLHA9;BHLHE22;BHLHE23;FERD3L;FIGLA;HAND1;HAND2;LYL1;MESP1;MESP2;MSC;MSGN1;NEUROD1;NEUROD2;NEUROD4;NEUROD6;NEUROG1;NEUROG2;NEUROG3;NHLH1;NHLH2;OLIG1;OLIG2;OLIG3;PTF1A;SCX;TAL1;TAL2;TCF15;TCF21;TCF23;TWIST1;TWIST2	ATOH1;BHLHA15;BHLHE22;BHLHE23;NEUROD1;NEUROD2;NEUROG2;OLIG1;OLIG2;OLIG3
ATOH1_HUMAN.H11MO.0.B	59	0.23137255	0.9575488	1.753.798	1.304.294	7,72E+04	2,84E+05	Tal-related factors{1.2.3}	Neurogenin / Atonal-like factors{1.2.3.4}	ATOH1;ATOH7;ATOH8;BHLHA15;BHLHA9;BHLHE22;BHLHE23;FERD3L;FIGLA;HAND1;HAND2;LYL1;MESP1;MESP2;MSC;MSGN1;NEUROD1;NEUROD2;NEUROD4;NEUROD6;NEUROG1;NEUROG2;NEUROG3;NHLH1;NHLH2;OLIG1;OLIG2;OLIG3;PTF1A;SCX;TAL1;TAL2;TCF15;TCF21;TCF23;TWIST1;TWIST2	ATOH1;BHLHA15;BHLHE22;BHLHE23;NEUROD1;NEUROD2;NEUROG2;OLIG1;OLIG2;OLIG3
ZN713_HUMAN.H11MO.0.D	42	0.16470588	0.9126306	1.822.401	1.303.410	1,15E+05	3,64E+05	More than 3 adjacent zinc finger factors{2.3.3}	unclassified{2.3.3.0}	BCL6B;BCL6;CTCFL;CTCF;FEZF1;GFI1B;GFI1;GLI1;GLI2;GLI3;GLIS1;GLIS2;GLIS3;MTF1;MYNN;MZF1;OSR2;OVOL1;OVOL2;ZNF146;PLAG1;PLAGL1;PRDM14;PRDM1;PRDM6;SCRT1;SCRT2;SNAI1;SNAI2;YY1;YY2;WT1;ZNF324;ZNF354A;ZBTB14;ZBTB18;ZBTB48;ZBTB49;ZBTB7A;ZBTB7B;ZBTB6;ZFP64;ZFP28;ZFP42;ZFP82;ZFX;ZIC1;ZIC2;ZIC3;ZIC4;ZIM3;ZKSCAN1;ZKSCAN3;ZNF121;ZNF136;ZNF140;ZNF143;ZNF148;ZNF214;ZNF232;ZNF250;ZNF257;ZNF260;ZNF263;ZNF264;ZNF274;ZNF281;ZNF282;ZNF317;ZNF320;ZNF322;ZNF329;ZNF331;ZNF333;ZNF350;ZNF384;ZNF394;ZNF410;ZNF436;ZNF449;ZNF490;ZNF502;ZNF524;ZNF528;ZNF547;ZNF549;ZNF554;ZNF563;ZNF582;ZNF586;ZNF589;ZNF652;ZNF667;ZNF680;ZNF708;ZNF713;ZNF768;ZNF816;ZNF18;ZNF41;ZNF76;ZNF85;ZSCAN16;ZSCAN22;ZSCAN31;ZSCAN4	MYNN;MZF1;OSR2;PRDM14;PRDM6;WT1;ZBTB14;ZBTB48;ZBTB49;ZFP64;ZFP28;ZIM3;ZNF121;ZNF250;ZNF257;ZNF263;ZNF274;ZNF317;ZNF320;ZNF329;ZNF331;ZNF394;ZNF449;ZNF502;ZNF528;ZNF547;ZNF549;ZNF554;ZNF586;ZNF589;ZNF667;ZNF680;ZNF708;ZNF713;ZNF768;ZNF18;ZNF85;ZSCAN16;ZSCAN22
STAT4_HUMAN.H11MO.0.A	28	0.10980392	0.8457553	1.933.875	1.301.951	1,81E+05	4,67E+05	STAT factors{6.2.1}	STAT4{6.2.1.0.4}	STAT1;STAT2;STAT3;STAT4;STAT5A;STAT5B;STAT6	STAT4
PTF1A_HUMAN.H11MO.1.B	80	0.31372549	0.9855922	1.705.020	1.301.075	5,37E+04	2,18E+05	Tal-related factors{1.2.3}	Twist-like factors{1.2.3.2}	ATOH1;ATOH7;ATOH8;BHLHA15;BHLHA9;BHLHE22;BHLHE23;FERD3L;FIGLA;HAND1;HAND2;LYL1;MESP1;MESP2;MSC;MSGN1;NEUROD1;NEUROD2;NEUROD4;NEUROD6;NEUROG1;NEUROG2;NEUROG3;NHLH1;NHLH2;OLIG1;OLIG2;OLIG3;PTF1A;SCX;TAL1;TAL2;TCF15;TCF21;TCF23;TWIST1;TWIST2	FIGLA;HAND1;PTF1A;TWIST1
ELF1_HUMAN.H11MO.0.A	67	0.26274510	0.9682435	1.727.427	1.300.058	6,73E+04	2,60E+05	Ets-related factors{3.5.2}	Elf-1-like factors{3.5.2.3}	EHF;ELF1;ELF2;ELF3;ELF4;ELF5;ELK1;ELK3;ELK4;ERF;ERG;ETS1;ETS2;ETV1;ETV2;ETV3;ETV3L;ETV4;ETV5;ETV6;ETV7;FEV;FLI1;GABPA;SPDEF;SPI1;SPIB;SPIC	ELF1;ELF2
NFIC_HUMAN.H11MO.1.A	34	0.13333333	0.8758594	1.868.251	1.297.218	1,54E+05	4,35E+05	Nuclear factor 1{7.1.2}	NF-1C (NF-IC){7.1.2.0.3}	NFIA;NFIB;NFIC	NFIC
PRD14_HUMAN.H11MO.0.A	19	0.07450980	0.7631570	2.063.090	1.291.054	2,84E+05	5,96E+05	More than 3 adjacent zinc finger factors{2.3.3}	unclassified{2.3.3.0}	BCL6B;BCL6;CTCFL;CTCF;FEZF1;GFI1B;GFI1;GLI1;GLI2;GLI3;GLIS1;GLIS2;GLIS3;MTF1;MYNN;MZF1;OSR2;OVOL1;OVOL2;ZNF146;PLAG1;PLAGL1;PRDM14;PRDM1;PRDM6;SCRT1;SCRT2;SNAI1;SNAI2;YY1;YY2;WT1;ZNF324;ZNF354A;ZBTB14;ZBTB18;ZBTB48;ZBTB49;ZBTB7A;ZBTB7B;ZBTB6;ZFP64;ZFP28;ZFP42;ZFP82;ZFX;ZIC1;ZIC2;ZIC3;ZIC4;ZIM3;ZKSCAN1;ZKSCAN3;ZNF121;ZNF136;ZNF140;ZNF143;ZNF148;ZNF214;ZNF232;ZNF250;ZNF257;ZNF260;ZNF263;ZNF264;ZNF274;ZNF281;ZNF282;ZNF317;ZNF320;ZNF322;ZNF329;ZNF331;ZNF333;ZNF350;ZNF384;ZNF394;ZNF410;ZNF436;ZNF449;ZNF490;ZNF502;ZNF524;ZNF528;ZNF547;ZNF549;ZNF554;ZNF563;ZNF582;ZNF586;ZNF589;ZNF652;ZNF667;ZNF680;ZNF708;ZNF713;ZNF768;ZNF816;ZNF18;ZNF41;ZNF76;ZNF85;ZSCAN16;ZSCAN22;ZSCAN31;ZSCAN4	MYNN;MZF1;OSR2;PRDM14;PRDM6;WT1;ZBTB14;ZBTB48;ZBTB49;ZFP64;ZFP28;ZIM3;ZNF121;ZNF250;ZNF257;ZNF263;ZNF274;ZNF317;ZNF320;ZNF329;ZNF331;ZNF394;ZNF449;ZNF502;ZNF528;ZNF547;ZNF549;ZNF554;ZNF586;ZNF589;ZNF667;ZNF680;ZNF708;ZNF713;ZNF768;ZNF18;ZNF85;ZSCAN16;ZSCAN22
HES7_HUMAN.H11MO.0.D	66	0.25882353	0.9591746	1.716.265	1.289.938	7,83E+04	2,85E+05	Hairy-related factors{1.2.4}	Hairy-like factors{1.2.4.1}	BHLHE40;BHLHE41;HELT;HES1;HES2;HES3;HES4;HES5;HES6;HES7;HEY1;HEY2;HEYL	BHLHE40;BHLHE41;HES1;HES5;HES7;HEY1;HEY2
EMX1_HUMAN.H11MO.0.D	14	0.05490196	0.6934771	2.206.322	1.288.495	3,52E+05	6,62E+05	NK-related factors{3.1.2}	EMX{3.1.2.6}	BARHL1;BARHL2;BARX1;BARX2;BSX;DBX1;DBX2;DLX1;DLX2;DLX3;DLX4;DLX5;DLX6;EMX1;EMX2;EN1;EN2;HHEX;HLX;HMX1;HMX2;HMX3;LBX1;LBX2;MSX1;MSX2;NANOG;NKX1-1;NKX1-2;NKX2-1;NKX2-2;NKX2-3;NKX2-4;NKX2-5;NKX2-6;NKX2-8;NKX3-1;NKX3-2;NKX6-1;NKX6-2;NKX6-3;NOTO;TLX1;TLX2;TLX3;VAX1;VAX2;VENTX	EMX1;EMX2

Motif	Nº of probes	% of probes	lower OR	upper OR	OR	p.value	FDR	TF family	TF subfamily	TF.family.member	TF.subfamily.member
MYOD1_HUMAN.H11MO.1.A	71	0.27843137	0.9644653	1.702.149	1.287.288	7,41E+04	2,79E+05	MyoD / ASC-related factors{1.2.2}	Myogenic transcription factors{1.2.2.1}	ASCL1;ASCL2;ASCL3;ASCL4;ASCL5;MYF5;MYF6;MYOD1;MYOG	MYF6;MYOD1;MYOG
ZN335_HUMAN.H11MO.1.A	31	0.12156863	0.8502522	1.872.579	1.281.830	2,04E+05	5,03E+05	Factors with multiple dispersed zinc fingers{2.3.4}	unclassified{2.3.4.0}	BCL11A;E4F1;MECOM;HIC1;HIC2;HINFP;IKZF1;INSM1;MAZ;PATZ1;PRDM4;REST;RREB1;SALL4;VEZF1;ZBTB17;ZBTB4;HIVEP1;HIVEP2;ZNF134;ZNF219;ZNF335;ZNF341;ZNF382;ZNF418;ZNF423;ZNF467;ZNF770;ZNF784;ZNF8	E4F1;PRDM4;REST;RREB1;ZBTB17;ZBTB4;ZNF335;ZNF341;ZNF467;ZNF770;ZNF784;ZNF8
KAISO_HUMAN.H11MO.2.A	37	0.14509804	0.8779107	1.821.759	1.280.592	1,72E+05	4,62E+05	Other factors with up to three adjacent zinc fingers{2.3.2}	Factors with 2-3 adjacent zinc fingersand a BTB/POZ domain{2.3.2.1}	AEBP2;OSR1;OVOL3;ZBTB22;ZBTB32;ZBTB33;ZBTB34;ZBTB37;ZBTB43;ZBTB46;ZBTB5;ZBTB8B;ZFPM2;ZNF174;ZNF396;ZNF414;ZNF446;ZNF487;ZNF511;ZNF580;ZNF705A;ZNF740;ZSCAN1	ZBTB33
NDF1_HUMAN.H11MO.0.A	51	0.20000000	0.9209907	1.745.384	1.278.174	1,27E+05	3,87E+05	Tal-related factors{1.2.3}	Neurogenin / Atonal-like factors{1.2.3.4}	ATOH1;ATOH7;ATOH8;BHLHA15;BHLHA9;BHLHE22;BHLHE23;FERD3L;FIGLA;HAND1;HAND2;LYL1;MESP1;MESP2;MSC;MSGN1;NEUROD1;NEUROD2;NEUROD4;NEUROD6;NEUROG1;NEUROG2;NEUROG3;NHLH1;NHLH2;OLIG1;OLIG2;OLIG3;PTF1A;SCX;TAL1;TAL2;TCF15;TCF21;TCF23;TWIST1;TWIST2	ATOH1;BHLHA15;BHLHE22;BHLHE23;NEUROD1;NEUROD2;NEUROG2;OLIG1;OLIG2;OLIG3
FOXO6_HUMAN.H11MO.0.D	23	0.09019608	0.7903754	1.957.682	1.272.437	2,74E+05	5,78E+05	Forkhead box (FOX) factors{3.3.1}	FOXO{3.3.1.15}	FOXA1;FOXA2;FOXA3;FOXB1;FOXB2;FOXC1;FOXC2;FOXD1;FOXD2;FOXD3;FOXD4;FOXD4L1;FOXD4L3;FOXD4L4;FOXD4L5;FOXD4L6;FOXE1;FOXE3;FOXF1;FOXF2;FOXG1;FOXH1;FOXI1;FOXI2;FOXI3;FOXJ1;FOXJ2;FOXJ3;FOXK1;FOXK2;FOXL1;FOXL2;FOXM1;FOXN1;FOXN2;FOXN3;FOXN4;FOXO1;FOXO3;FOXO4;FOXO6;FOXP1;FOXP2;FOXP3;FOXP4;FOXQ1;FOXR1;FOXR2;FOXS1	FOXO1;FOXO3;FOXO4;FOXO6
SRBP2_HUMAN.H11MO.0.B	102	0.40000000	0.9769685	1.640.313	1.268.091	6,49E+04	2,54E+05	bHLH-ZIP factors{1.2.6}	SREBP factors{1.2.6.3}	MAX;MITF;MLX;MLXIP;MLXIPL;MNT;MXD1;MXD3;MXD4;MXI1;MYC;MYCL;MYCN;REPIN1;SREBF1;SREBF2;TFAP4;TFE3;TFEB;TFEC;USF1;USF2	SREBF1;SREBF2
OLIG2_HUMAN.H11MO.1.B	46	0.18039216	0.8988620	1.749.687	1.265.908	1,57E+05	4,40E+05	Tal-related factors{1.2.3}	Neurogenin / Atonal-like factors{1.2.3.4}	ATOH1;ATOH7;ATOH8;BHLHA15;BHLHA9;BHLHE22;BHLHE23;FERD3L;FIGLA;HAND1;HAND2;LYL1;MESP1;MESP2;MSC;MSGN1;NEUROD1;NEUROD2;NEUROD4;NEUROD6;NEUROG1;NEUROG2;NEUROG3;NHLH1;NHLH2;OLIG1;OLIG2;OLIG3;PTF1A;SCX;TAL1;TAL2;TCF15;TCF21;TCF23;TWIST1;TWIST2	ATOH1;BHLHA15;BHLHE22;BHLHE23;NEUROD1;NEUROD2;NEUROG2;OLIG1;OLIG2;OLIG3
REL_HUMAN.H11MO.0.B	28	0.10980392	0.8198037	1.874.591	1.262.027	2,70E+05	5,71E+05	NF-kappaB-related factors{6.1.1}	NF-kappaB p65 subunit-like factors{6.1.1.2}	NFKB1;NFKB2;RELB;REL;RELA	RELB;REL;RELA
RFX2_HUMAN.H11MO.1.A	36	0.14117647	0.8596964	1.799.688	1.260.124	2,02E+05	5,00E+05	RFX-related factors{3.3.3}	RFX2{3.3.3.0.2}	RFX1;RFX2;RFX3;RFX4;RFX5;RFX6;RFX7;RFX8	RFX2
SOX4_HUMAN.H11MO.0.B	38	0.14901961	0.8676925	1.785.627	1.259.909	1,81E+05	4,67E+05	SOX-related factors{4.1.1}	Group C{4.1.1.3}	BBX;CIC;HBP1;SOX1;SOX10;SOX11;SOX12;SOX13;SOX14;SOX15;SOX17;SOX18;SOX2;SOX21;SOX3;SOX30;SOX4;SOX5;SOX6;SOX7;SOX8;SOX9;SRY	SOX11;SOX4
ZSC31_HUMAN.H11MO.0.C	80	0.31372549	0.9522826	1.647.387	1.257.114	1,03E+05	3,43E+05	More than 3 adjacent zinc finger factors{2.3.3}	ZNF24-like factors{2.3.3.10}	BCL6;BCL6B;CTCF;CTCFL;FEZF1;FEZF2;GFI1;GFI1B;GLI1;GLI2;GLI3;GLI4;GLIS1;GLIS2;GLIS3;HKR1;MTF1;MYNN;MZF1;OSR2;OVOL1;OVOL2;PLAG1;PLAGL1;PLAGL2;PRDM1;PRDM14;PRDM6;SCRT1;SCRT2;SNAI1;SNAI2;SNAI3;WT1;YY1;YY2;ZBTB12;ZBTB14;ZBTB18;ZBTB20;ZBTB26;ZBTB42;ZBTB45;ZBTB47;ZBTB48;ZBTB49;ZBTB6;ZBTB7A;ZBTB7B;ZBTB7C;ZFP14;ZFP2;ZFP28;ZFP30;ZFP37;ZFP42;ZFP64;ZFP69;ZFP69B;ZFP82;ZFP91;ZFX;ZIC1;ZIC2;ZIC3;ZIC4;ZIC5;ZIK1;ZIM3;ZKSCAN1;ZKSCAN2;ZKSCAN3;ZKSCAN4;ZNF121;ZNF124;ZNF133;ZNF136;ZNF138;ZNF14;ZNF140;ZNF143;ZNF146;ZNF148;ZNF155;ZNF157;ZNF160;ZNF169;ZNF175;ZNF177;ZNF18;ZNF180;ZNF181;ZNF2;ZNF20;ZNF212;ZNF213;ZNF214;ZNF221;ZNF222;ZNF223;ZNF224;ZNF225;ZNF226;ZNF227;ZNF229;ZNF230;ZNF232;ZNF233;ZNF234;ZNF235;ZNF24;ZNF25;ZNF250;ZNF257;ZNF26;ZNF260;ZNF263;ZNF264;ZNF268;ZNF274;ZNF276;ZNF28;ZNF280A;ZNF280B;ZNF280C;ZNF280D;ZNF281;ZNF282;ZNF283;ZNF284;ZNF285;ZNF286A;ZNF286B;ZNF3;ZNF30;ZNF300;ZNF302;ZNF317;ZNF32;ZNF320;ZNF322;ZNF324;ZNF324B;ZNF329;ZNF331;ZNF333;ZNF33A;ZNF33B;ZNF343;ZNF345;ZNF347;ZNF350;ZNF354A;ZNF354B;ZNF362;ZNF366;ZNF383;ZNF384;ZNF394;ZNF397;ZNF398;ZNF404;ZNF41;ZNF410;ZNF419;ZNF420;ZNF431;Z	ZKSCAN1;ZNF232;ZSCAN31

Motif	Nº of probes	% of probes	lower OR	upper OR	OR	p.value	FDR	TF family	TF subfamily	TF.family.member	TF.subfamily.member
										NF432;ZNF436;ZNF439;ZNF44;ZNF440;ZNF442;ZNF443;ZNF446;ZNF449;ZNF45;ZNF460;ZNF468;ZNF479;ZNF484;ZNF490;ZNF500;ZNF502;ZNF524;ZNF525;ZNF528;ZNF543;ZNF544;ZNF546;ZNF547;ZNF548;ZNF549;ZNF554;ZNF555;ZNF557;ZNF558;ZNF559;ZNF561;ZNF562;ZNF563;ZNF564;ZNF566;ZNF567;ZNF568;ZNF57;ZNF570;ZNF571;ZNF572;ZNF577;ZNF581;ZNF582;ZNF583;ZNF585A;ZNF586;ZNF589;ZNF595;ZNF599;ZNF600;ZNF605;ZNF607;ZNF611;ZNF613;ZNF614;ZNF615;ZNF616;ZNF619;ZNF620;ZNF621;ZNF625;ZNF627;ZNF649;ZNF652;ZNF653;ZNF665;ZNF667;ZNF669;ZNF670;ZNF672;ZNF679;ZNF680;ZNF683;ZNF689;ZNF692;ZNF701;ZNF705D;ZNF705E;ZNF705G;ZNF708;ZNF709;ZNF71;ZNF710;ZNF713;ZNF721;ZNF727;ZNF729;ZNF736;ZNF75A;ZNF75D;ZNF76;ZNF763;ZNF764;ZNF765;ZNF768;ZNF77;ZNF771;ZNF773;ZNF774;ZNF776;ZNF777;ZNF780A;ZNF780B;ZNF782;ZNF785;ZNF799;ZNF805;ZNF808;ZNF81;ZNF813;ZNF816;ZNF823;ZNF829;ZNF836;ZNF841;ZNF844;ZNF845;ZNF846;ZNF85;ZNF853;ZNF860;ZNF878;ZNF891;ZNF99;ZSCAN16;ZSCAN2;ZSCAN22;ZSCAN23;ZSCAN29;ZSCAN31;ZSCAN32;ZSCAN4;ZSCAN5A;ZSCAN5B;ZSCAN5C;ZSCAN9;ZXDA;ZXDB;ZXDC	
AHR_HUMAN.H11MO.0.B	94	0.36862745	0.9626337	1.629.749	1.255.475	8,03E+04	2,85E+05	PAS domain factors{1.2.5}	Ahr-like factors{1.2.5.1}	AHR;AHRR;ARNT;ARNT2;ARNTL;ARNTL2;CLOCK;EPAS1;HIF1A;HIF3A;NCOA1;NCOA2;NCOA3;NPAS1;NPAS2;NPAS3;NPAS4;SIM1;SIM2;SOHLH1;SOHLH2;TCFL5	AHR;EPAS1;HIF1A
WT1_HUMAN.H11MO.1.B	99	0.38823529	0.9625909	1.620.749	1.251.461	8,46E+04	2,97E+05	More than 3 adjacent zinc finger factors{2.3.3}	unclassified{2.3.3.0}	BCL6B;BCL6;CTCFL;CTCF;FEZF1;GFI1B;GFI1;GLI1;GLI2;GLI3;GLIS1;GLIS2;GLIS3;MTF1;MYNN;MZF1;OSR2;OVOL1;OVOL2;ZNF146;PLAG1;PLAGL1;PRDM14;PRDM1;PRDM6;SCRT1;SCRT2;SNAI1;SNAI2;YY1;YY2;WT1;ZNF324;ZNF354A;ZBTB14;ZBTB18;ZBTB48;ZBTB49;ZBTB7A;ZBTB7B;ZBTB6;ZFP64;ZFP28;ZFP42;ZFP82;ZFX;ZIC1;ZIC2;ZIC3;ZIC4;ZIM3;ZKSCAN1;ZKSCAN3;ZNF121;ZNF136;ZNF140;ZNF143;ZNF148;ZNF214;ZNF232;ZNF250;ZNF257;ZNF260;ZNF263;ZNF264;ZNF274;ZNF281;ZNF282;ZNF317;ZNF320;ZNF322;ZNF329;ZNF331;ZNF333;ZNF350;ZNF384;ZNF394;ZNF410;ZNF436;ZNF449;ZNF490;ZNF502;ZNF524;ZNF528;ZNF547;ZNF549;ZNF554;ZNF563;ZNF582;ZNF586;ZNF589;ZNF652;ZNF667;ZNF680;ZNF708;ZNF713;ZNF768;ZNF816;ZNF18;ZNF41;ZNF76;ZNF85;ZSCAN16;ZSCAN22;ZSCAN31;ZSCAN4	MYNN;MZF1;OSR2;PRDM14;PRDM6;WT1;ZBTB14;ZBTB48;ZBTB49;ZFP64;ZFP28;ZIM3;ZNF121;ZNF250;ZNF257;ZNF263;ZNF274;ZNF317;ZNF320;ZNF329;ZNF331;ZNF394;ZNF449;ZNF502;ZNF528;ZNF547;ZNF549;ZNF554;ZNF586;ZNF589;ZNF667;ZNF680;ZNF708;ZNF713;ZNF768;ZNF18;ZNF85;ZSCAN16;ZSCAN22
FOXD3_HUMAN.H11MO.0.D	34	0.13333333	0.8443602	1.801.131	1.250.578	2,28E+05	5,38E+05	Forkhead box (FOX) factors{3.3.1}	FOXD{3.3.1.4}	FOXA1;FOXA2;FOXA3;FOXB1;FOXB2;FOXC1;FOXC2;FOXD1;FOXD2;FOXD3;FOXD4;FOXD4L1;FOXD4L3;FOXD4L4;FOXD4L5;FOXD4L6;FOXE1;FOXE3;FOXF1;FOXF2;FOXG1;FOXH1;FOXI1;FOXI2;FOXI3;FOXJ1;FOXJ2;FOXJ3;FOXK1;FOXK2;FOXL1;FOXL2;FOXM1;FOXN1;FOXN2;FOXN3;FOXN4;FOXO1;FOXO3;FOXO4;FOXO6;FOXP1;FOXP2;FOXP3;FOXP4;FOXQ1;FOXR1;FOXR2;FOXS1	FOXD1;FOXD2;FOXD3
NR2E1_HUMAN.H11MO.0.D	16	0.06274510	0.7019939	2.071.898	1.248.899	3,90E+05	6,86E+05	RXR-related receptors (NR2){2.1.3}	Tailless-like receptors (NR2E){2.1.3.3}	HNF4A;HNF4G;NR2C1;NR2C2;NR2E1;NR2E3;NR2F1;NR2F2;NR2F6;RXRA;RXRB;RXRG	NR2E1;NR2E3
IRF1_HUMAN.H11MO.0.A	53	0.20784314	0.9030917	1.695.396	1.246.853	1,59E+05	4,41E+05	Interferon-regulatory factors{3.5.3}	IRF-1{3.5.3.0.1}	IRF1;IRF2;IRF3;IRF4;IRF5;IRF6;IRF7;IRF8;IRF9	IRF1
RFX1_HUMAN.H11MO.1.B	34	0.13333333	0.8416951	1.795.443	1.246.630	2,29E+05	5,38E+05	RFX-related factors{3.3.3}	RFX1 (EF-C){3.3.3.0.1}	RFX1;RFX2;RFX3;RFX4;RFX5;RFX6;RFX7;RFX8	RFX1
TF7L1_HUMAN.H11MO.0.B	19	0.07450980	0.7368588	1.991.945	1.246.540	3,56E+05	6,65E+05	TCF-7-related factors{4.1.3}	TCF-7L1 (TCF-3) [1]{4.1.3.0.2}	LEF1;TAF1;TCF7;TCF7L1;TCF7L2	TCF7L1
MYC_HUMAN.H11MO.0.A	69	0.27058824	0.9311318	1.652.042	1.246.402	1,18E+05	3,71E+05	bHLH-ZIP factors{1.2.6}	Myc / Max factors{1.2.6.5}	MAX;MITF;MLXIPL;MLX;MXI1;MYCN;MYC;SREBF1;SREBF2;TFAP4;TFE3;TFEB;USF1;USF2	MAX;MYCN;MYC
ZN449_HUMAN.H11MO.0.C	52	0.20392157	0.8999345	1.697.315	1.245.649	1,57E+05	4,40E+05	More than 3 adjacent zinc finger factors{2.3.3}	unclassified{2.3.3.0}	BCL6B;BCL6;CTCFL;CTCF;FEZF1;GFI1B;GFI1;GLI1;GLI2;GLI3;GLIS1;GLIS2;GLIS3;MTF1;MYNN;MZF1;OSR2;OVOL1;OVOL2;ZNF146;PLAG1;PLAGL1;PRDM14;PRDM1;PRDM6;SCRT1;SCRT2;SNAI1;SNAI2;YY1;YY2;WT1;ZNF324;ZNF354A;ZBTB14;ZBTB18;ZBTB48;ZBTB49;ZBTB7A;ZBTB7B;ZBTB6;ZFP64;ZFP28;ZFP42;ZFP82;ZFX;ZIC1;ZIC2;ZIC3;ZIC4;ZI	MYNN;MZF1;OSR2;PRDM14;PRDM6;WT1;ZBTB14;ZBTB48;ZBTB49;ZFP64;ZFP28;ZIM3;ZNF121;ZNF250;ZNF257;ZNF263;ZNF274;ZNF317;ZNF320;ZNF329;ZNF331;ZNF394;ZNF449;ZNF502;ZNF528;ZNF547;ZNF549;ZNF554;ZNF586;ZNF589;ZNF667;ZNF680;ZNF708;ZNF713;ZNF768;ZNF18;ZNF85;ZSCAN16;ZSCAN22

Motif	Nº of probes	% of probes	lower OR	upper OR	OR	p.value	FDR	TF family	TF subfamily	TF.family.member	TF.subfamily.member
										M3;ZKSCAN1;ZKSCAN3;ZNF121;ZNF136;ZNF140;ZNF143;ZNF148;ZNF214;ZNF232;ZNF250;ZNF257;ZNF260;ZNF263;ZNF264;ZNF274;ZNF281;ZNF282;ZNF317;ZNF320;ZNF322;ZNF329;ZNF331;ZNF333;ZNF350;ZNF384;ZNF394;ZNF410;ZNF436;ZNF449;ZNF490;ZNF502;ZNF524;ZNF528;ZNF547;ZNF549;ZNF554;ZNF563;ZNF582;ZNF586;ZNF589;ZNF652;ZNF667;ZNF680;ZNF708;ZNF713;ZNF768;ZNF816;ZNF18;ZNF41;ZNF76;ZNF85;ZSCAN16;ZSCAN22;ZSCAN31;ZSCAN4	
ASCL1_HUMAN.H11MO.0.A	83	0.32549020	0.9458562	1.627.316	1.244.844	1,08E+05	3,53E+05	MyoD / ASC-related factors{1.2.2}	Achaete-Scute-like factors{1.2.2.2}	ASCL1;ASCL2;ASCL3;ASCL4;ASCL5;MYF5;MYF6;MYOD1;MYOG	ASCL1;ASCL2
CEBPD_HUMAN.H11MO.0.C	16	0.06274510	0.6995340	2.064.769	1.244.520	3,91E+05	6,86E+05	C/EBP-related{1.1.8}	C/EBP{1.1.8.1}	CEBPA;CEBPB;CEBPD;CEBPE;CEBPG;DBP;DDIT3;HLF;NFIL3;TEF	CEBPA;CEBPB;CEBPD;CEBPE;CEBPG;DDIT3
MGAP_HUMAN.H11MO.0.D	21	0.08235294	0.7534901	1.943.019	1.240.994	3,18E+05	6,30E+05	bHLH-ZIP factors{1.2.6}; TBX6-related factors{6.5.5}	Mad-like factors{1.2.6.7}; MGA (MAD5){6.5.5.0.2}	MGA;TBX6	MGA
MEIS2_HUMAN.H11MO.0.B	24	0.09411765	0.7775821	1.891.139	1.239.107	2,92E+05	6,03E+05	TALE-type homeo domain factors{3.1.4}	MEIS{3.1.4.2}	IRX1;IRX2;IRX3;IRX4;IRX5;IRX6;MEIS1;MEIS2;MEIS3;MKX;PBX1;PBX2;PBX3;PKNOX1;PKNOX2;TGIF1;TGIF2;TGIF2LX;TGIF2LY	MEIS1;MEIS2;MEIS3
ZN770_HUMAN.H11MO.1.C	109	0.42745098	0.9555787	1.595.997	1.236.486	1,06E+05	3,48E+05	Factors with multiple dispersed zinc fingers{2.3.4}	unclassified{2.3.4.0}	BCL11A;E4F1;MECOM;HIC1;HIC2;HINFP;IKZF1;INSM1;MAZ;PATZ1;PRDM4;REST;RREB1;SALL4;VEZF1;ZBTB17;ZBTB4;HIVEP1;HIVEP2;ZNF134;ZNF219;ZNF335;ZNF341;ZNF382;ZNF418;ZNF423;ZNF467;ZNF770;ZNF784;ZNF8	E4F1;PRDM4;REST;RREB1;ZBTB17;ZBTB4;ZNF335;ZNF341;ZNF467;ZNF770;ZNF784;ZNF8
PRDM6_HUMAN.H11MO.0.C	50	0.19607843	0.8857464	1.686.945	1.232.553	2,05E+05	5,05E+05	More than 3 adjacent zinc finger factors{2.3.3}	unclassified{2.3.3.0}	BCL6B;BCL6;CTCFL;CTCF;FEZF1;GFI1B;GFI1;GLI1;GLI2;GLI3;GLIS1;GLIS2;GLIS3;MTF1;MYNN;MZF1;OSR2;OVOL1;OVOL2;ZNF146;PLAG1;PLAGL1;PRDM14;PRDM1;PRDM6;SCRT1;SCRT2;SNAI1;SNAI2;YY1;YY2;WT1;ZNF324;ZNF354A;ZBTB14;ZBTB18;ZBTB48;ZBTB49;ZBTB7A;ZBTB7B;ZBTB6;ZFP64;ZFP28;ZFP42;ZFP82;ZFX;ZIC1;ZIC2;ZIC3;ZIC4;ZIM3;ZKSCAN1;ZKSCAN3;ZNF121;ZNF136;ZNF140;ZNF143;ZNF148;ZNF214;ZNF232;ZNF250;ZNF257;ZNF260;ZNF263;ZNF264;ZNF274;ZNF281;ZNF282;ZNF317;ZNF320;ZNF322;ZNF329;ZNF331;ZNF333;ZNF350;ZNF384;ZNF394;ZNF410;ZNF436;ZNF449;ZNF490;ZNF502;ZNF524;ZNF528;ZNF547;ZNF549;ZNF554;ZNF563;ZNF582;ZNF586;ZNF589;ZNF652;ZNF667;ZNF680;ZNF708;ZNF713;ZNF768;ZNF816;ZNF18;ZNF41;ZNF76;ZNF85;ZSCAN16;ZSCAN22;ZSCAN31;ZSCAN4	MYNN;MZF1;OSR2;PRDM14;PRDM6;WT1;ZBTB14;ZBTB48;ZBTB49;ZFP64;ZFP28;ZIM3;ZNF121;ZNF250;ZNF257;ZNF263;ZNF274;ZNF317;ZNF320;ZNF329;ZNF331;ZNF394;ZNF449;ZNF502;ZNF528;ZNF547;ZNF549;ZNF554;ZNF586;ZNF589;ZNF667;ZNF680;ZNF708;ZNF713;ZNF768;ZNF18;ZNF85;ZSCAN16;ZSCAN22
FOXJ3_HUMAN.H11MO.0.A	38	0.14901961	0.8481625	1.745.358	1.231.517	2,54E+05	5,65E+05	Forkhead box (FOX) factors{3.3.1}	FOXJ{3.3.1.10}	FOXA1;FOXA2;FOXA3;FOXB1;FOXB2;FOXC1;FOXC2;FOXD1;FOXD2;FOXD3;FOXD4;FOXD4L1;FOXD4L3;FOXD4L4;FOXD4L5;FOXD4L6;FOXE1;FOXE3;FOXF1;FOXF2;FOXG1;FOXH1;FOXI1;FOXI2;FOXI3;FOXJ1;FOXJ2;FOXJ3;FOXK1;FOXK2;FOXL1;FOXL2;FOXM1;FOXN1;FOXN2;FOXN3;FOXN4;FOXO1;FOXO3;FOXO4;FOXO6;FOXP1;FOXP2;FOXP3;FOXP4;FOXQ1;FOXR1;FOXR2;FOXS1	FOXJ2;FOXJ3
NFIA_HUMAN.H11MO.0.C	36	0.14117647	0.8378369	1.753.891	1.228.073	2,44E+05	5,59E+05	Nuclear factor 1{7.1.2}	NF-1A (NF-IA){7.1.2.0.1}	NFIA;NFIB;NFIC	NFIA
ZN436_HUMAN.H11MO.0.C	70	0.27450980	0.9177885	1.623.961	1.226.734	1,60E+05	4,41E+05	More than 3 adjacent zinc finger factors{2.3.3}	ZNF180-like factors{2.3.3.58}	BCL6;BCL6B;CTCF;CTCFL;FEZF1;FEZF2;GFI1;GFI1B;GLI1;GLI2;GLI3;GLI4;GLIS1;GLIS2;GLIS3;HKR1;MTF1;MYNN;MZF1;OSR2;OVOL1;OVOL2;PLAG1;PLAGL1;PLAGL2;PRDM1;PRDM14;PRDM6;SCRT1;SCRT2;SNAI1;SNAI2;SNAI3;WT1;YY1;YY2;ZBTB12;ZBTB14;ZBTB18;ZBTB20;ZBTB26;ZBTB42;ZBTB45;ZBTB47;ZBTB48;ZBTB49;ZBTB6;ZBTB7A;ZBTB7B;ZBTB7C;ZFP14;ZFP2;ZFP28;ZFP30;ZFP37;ZFP42;ZFP64;ZFP69;ZFP69B;ZFP82;ZFP91;ZFX;ZIC1;ZIC2;ZIC3;ZIC4;ZIC5;ZIK1;ZIM3;ZKSCAN1;ZKSCAN2;ZKSCAN3;ZKSCAN4;ZNF121;ZNF124;ZNF133;ZNF136;ZNF138;ZNF14;ZNF140;ZNF143;ZNF146;ZNF148;ZNF155;ZNF157;ZNF160;ZNF169;ZNF175;ZNF177;ZNF18;ZNF180;ZNF181;ZNF2;ZNF20;ZNF212;ZNF213;ZNF214;ZNF221;ZNF222;ZNF223;ZNF224;ZNF225;ZNF226;ZNF227;ZNF229;ZNF230;ZNF232;ZNF233;ZNF234;ZNF235;ZNF24;ZNF25;ZNF250;ZNF257;ZNF26;ZNF260;ZNF263;ZNF26	ZNF436

Motif	Nº of probes	% of probes	lower OR	upper OR	OR	p.value	FDR	TF family	TF subfamily	TF.family.member	TF.subfamily.member
										4;ZNF268;ZNF274;ZNF276;ZNF28;ZNF280A;ZNF280B;ZNF280C;ZNF280D;ZNF281;ZNF282;ZNF283;ZNF284;ZNF285;ZNF286A;ZNF286B;ZNF3;ZNF30;ZNF300;ZNF302;ZNF317;ZNF32;ZNF320;ZNF322;ZNF324;ZNF324B;ZNF329;ZNF331;ZNF333;ZNF33A;ZNF33B;ZNF343;ZNF345;ZNF347;ZNF350;ZNF354A;ZNF354B;ZNF362;ZNF366;ZNF383;ZNF384;ZNF394;ZNF397;ZNF398;ZNF404;ZNF41;ZNF410;ZNF419;ZNF420;ZNF431;ZNF432;ZNF436;ZNF439;ZNF44;ZNF440;ZNF442;ZNF443;ZNF446;ZNF449;ZNF45;ZNF460;ZNF468;ZNF479;ZNF484;ZNF490;ZNF500;ZNF502;ZNF524;ZNF525;ZNF528;ZNF543;ZNF544;ZNF546;ZNF547;ZNF548;ZNF549;ZNF554;ZNF555;ZNF557;ZNF558;ZNF559;ZNF561;ZNF562;ZNF563;ZNF564;ZNF566;ZNF567;ZNF568;ZNF57;ZNF570;ZNF571;ZNF572;ZNF577;ZNF581;ZNF582;ZNF583;ZNF585A;ZNF586;ZNF589;ZNF595;ZNF599;ZNF600;ZNF605;ZNF607;ZNF611;ZNF613;ZNF614;ZNF615;ZNF616;ZNF619;ZNF620;ZNF621;ZNF625;ZNF627;ZNF649;ZNF652;ZNF653;ZNF665;ZNF667;ZNF669;ZNF670;ZNF672;ZNF679;ZNF680;ZNF683;ZNF689;ZNF692;ZNF701;ZNF705D;ZNF705E;ZNF705G;ZNF708;ZNF709;ZNF71;ZNF710;ZNF713;ZNF721;ZNF727;ZNF729;ZNF736;ZNF75A;ZNF75D;ZNF76;ZNF763;ZNF764;ZNF765;ZNF768;ZNF77;ZNF771;ZNF773;ZNF774;ZNF776;ZNF777;ZNF780A;ZNF780B;ZNF782;ZNF785;ZNF799;ZNF805;ZNF808;ZNF81;ZNF813;ZNF816;ZNF823;ZNF829;ZNF836;ZNF841;ZNF844;ZNF845;ZNF846;ZNF85;ZNF853;ZNF860;ZNF878;ZNF891;ZNF99;ZSCAN16;ZSCAN2;ZSCAN22;ZSCAN23;ZSCAN29;ZSCAN31;ZSCAN32;ZSCAN4;ZSCAN5A;ZSCAN5B;ZSCAN5C;ZSCAN9;ZXDA;ZXDB;ZXDC	
MYOG_HUMAN.H11MO.0.B	74	0.29019608	0.9227528	1.616.738	1.226.718	1,48E+05	4,26E+05	MyoD / ASC-related factors{1.2.2}	Myogenic transcription factors{1.2.2.1}	ASCL1;ASCL2;ASCL3;ASCL4;ASCL5;MYF5;MYF6;MYOD1;MYOG	MYF6;MYOD1;MYOG
ZN263_HUMAN.H11MO.1.A	91	0.35686275	0.9361187	1.590.889	1.223.505	1,36E+05	4,09E+05	More than 3 adjacent zinc finger factors{2.3.3}	unclassified{2.3.3.0}	BCL6B;BCL6;CTCFL;CTCF;FEZF1;GFI1B;GFI1;GLI1;GLI2;GLI3;GLIS1;GLIS2;GLIS3;MTF1;MYNN;MZF1;OSR2;OVOL1;OVOL2;ZNF146;PLAG1;PLAGL1;PRDM14;PRDM1;PRDM6;SCRT1;SCRT2;SNAI1;SNAI2;YY1;YY2;WT1;ZNF324;ZNF354A;ZBTB14;ZBTB18;ZBTB48;ZBTB49;ZBTB7A;ZBTB7B;ZBTB6;ZFP64;ZFP28;ZFP42;ZFP82;ZFX;ZIC1;ZIC2;ZIC3;ZIC4;ZIM3;ZKSCAN1;ZKSCAN3;ZNF121;ZNF136;ZNF140;ZNF143;ZNF148;ZNF214;ZNF232;ZNF250;ZNF257;ZNF260;ZNF263;ZNF264;ZNF274;ZNF281;ZNF282;ZNF317;ZNF320;ZNF322;ZNF329;ZNF331;ZNF333;ZNF350;ZNF384;ZNF394;ZNF410;ZNF436;ZNF449;ZNF490;ZNF502;ZNF524;ZNF528;ZNF547;ZNF549;ZNF554;ZNF563;ZNF582;ZNF586;ZNF589;ZNF652;ZNF667;ZNF680;ZNF708;ZNF713;ZNF768;ZNF816;ZNF18;ZNF41;ZNF76;ZNF85;ZSCAN16;ZSCAN22;ZSCAN31;ZSCAN4	MYNN;MZF1;OSR2;PRDM14;PRDM6;WT1;ZBTB14;ZBTB48;ZBTB49;ZFP64;ZFP28;ZIM3;ZNF121;ZNF250;ZNF257;ZNF263;ZNF274;ZNF317;ZNF320;ZNF329;ZNF331;ZNF394;ZNF449;ZNF502;ZNF528;ZNF547;ZNF549;ZNF554;ZNF586;ZNF589;ZNF667;ZNF680;ZNF708;ZNF713;ZNF768;ZNF18;ZNF85;ZSCAN16;ZSCAN22
MAX_HUMAN.H11MO.0.A	83	0.32549020	0.9269957	1.594.734	1.219.917	1,44E+05	4,19E+05	bHLH-ZIP factors{1.2.6}	Myc / Max factors{1.2.6.5}	MAX;MITF;MLX;MLXIP;MLXIPL;MNT;MXD1;MXD3;MXD4;MXI1;MYC;MYCL;MYCN;REPIN1;SREBF1;SREBF2;TFAP4;TFE3;TFEB;TFEC;USF1;USF2	MAX;MYCN;MYC
GRHL2_HUMAN.H11MO.0.A	25	0.09803922	0.7703010	1.841.971	1.215.838	3,60E+05	6,67E+05	Grainyhead-related factors{6.7.1}	GRH-like proteins{6.7.1.1}	GRHL1;GRHL2;GRHL3	GRHL1;GRHL2
ZN667_HUMAN.H11MO.0.C	43	0.16862745	0.8543459	1.694.492	1.215.621	2,44E+05	5,59E+05	More than 3 adjacent zinc finger factors{2.3.3}	unclassified{2.3.3.0}	BCL6B;BCL6;CTCFL;CTCF;FEZF1;GFI1B;GFI1;GLI1;GLI2;GLI3;GLIS1;GLIS2;GLIS3;MTF1;MYNN;MZF1;OSR2;OVOL1;OVOL2;ZNF146;PLAG1;PLAGL1;PRDM14;PRDM1;PRDM6;SCRT1;SCRT2;SNAI1;SNAI2;YY1;YY2;WT1;ZNF324;ZNF354A;ZBTB14;ZBTB18;ZBTB48;ZBTB49;ZBTB7A;ZBTB7B;ZBTB6;ZFP64;ZFP28;ZFP42;ZFP82;ZFX;ZIC1;ZIC2;ZIC3;ZIC4;ZIM3;ZKSCAN1;ZKSCAN3;ZNF121;ZNF136;ZNF140;ZNF143;ZNF148;ZNF214;ZNF232;ZNF250;ZNF257;ZNF260;ZNF263;ZNF264;ZNF274;ZNF281;ZNF282;ZNF317;ZNF320;ZNF322;ZNF329;ZNF331;ZNF333;ZNF350;ZNF384;ZNF394;ZNF410;ZNF436;ZNF449;ZNF490;ZNF502;ZNF524;ZNF528;ZNF547;ZNF549;ZNF554;ZNF563;ZNF582;ZNF586;ZNF589;ZNF652;ZNF667;ZNF680;ZNF708;ZNF713;ZNF768;ZNF816;ZNF18;ZNF41;ZNF76;ZNF85;ZSCAN16;ZSCAN22;ZSCAN31;ZSCAN4	MYNN;MZF1;OSR2;PRDM14;PRDM6;WT1;ZBTB14;ZBTB48;ZBTB49;ZFP64;ZFP28;ZIM3;ZNF121;ZNF250;ZNF257;ZNF263;ZNF274;ZNF317;ZNF320;ZNF329;ZNF331;ZNF394;ZNF449;ZNF502;ZNF528;ZNF547;ZNF549;ZNF554;ZNF586;ZNF589;ZNF667;ZNF680;ZNF708;ZNF713;ZNF768;ZNF18;ZNF85;ZSCAN16;ZSCAN22

Motif	Nº of probes	% of probes	lower OR	upper OR	OR	p.value	FDR	TF family	TF subfamily	TF.family.member	TF.subfamily.member
ETV2_HUMAN.H11MO.0.B	67	0.26274510	0.9048325	1.614.312	1.214.920	1,78E+05	4,67E+05	Ets-related factors{3.5.2}	Ets-like factors{3.5.2.1}	EHF;ELF1;ELF2;ELF3;ELF4;ELF5;ELK1;ELK3;ELK4;ERF;ERG;ETS1;ETS2;ETV1;ETV2;ETV3;ETV3L;ETV4;ETV5;ETV6;ETV7;FEV;FLI1;GABPA;SPDEF;SPI1;SPIB;SPIC	ERG;ETS1;ETS2;ETV2;ETV3;FEV;FLI1;GABPA
ZFP82_HUMAN.H11MO.0.C	38	0.14901961	0.8346878	1.717.528	1.211.902	2,59E+05	5,65E+05	More than 3 adjacent zinc finger factors{2.3.3}	ZFP30-like factors{2.3.3.63}	BCL6;BCL6B;CTCF;CTCFL;FEZF1;FEZF2;GFI1;GFI1B;GLI1;GLI2;GLI3;GLI4;GLIS1;GLIS2;GLIS3;HKR1;MTF1;MYNN;MZF1;OSR2;OVOL1;OVOL2;PLAG1;PLAGL1;PLAGL2;PRDM1;PRDM14;PRDM6;SCRT1;SCRT2;SNAI1;SNAI2;SNAI3;WT1;YY1;YY2;ZBTB12;ZBTB14;ZBTB18;ZBTB20;ZBTB26;ZBTB42;ZBTB45;ZBTB47;ZBTB48;ZBTB49;ZBTB6;ZBTB7A;ZBTB7B;ZBTB7C;ZFP14;ZFP2;ZFP28;ZFP30;ZFP37;ZFP42;ZFP64;ZFP69;ZFP69B;ZFP82;ZFP91;ZFX;ZIC1;ZIC2;ZIC3;ZIC4;ZIC5;ZIK1;ZIM3;ZKSCAN1;ZKSCAN2;ZKSCAN3;ZKSCAN4;ZNF121;ZNF124;ZNF133;ZNF136;ZNF138;ZNF14;ZNF140;ZNF143;ZNF146;ZNF148;ZNF155;ZNF157;ZNF160;ZNF169;ZNF175;ZNF177;ZNF18;ZNF180;ZNF181;ZNF2;ZNF20;ZNF212;ZNF213;ZNF214;ZNF221;ZNF222;ZNF223;ZNF224;ZNF225;ZNF226;ZNF227;ZNF229;ZNF230;ZNF232;ZNF233;ZNF234;ZNF235;ZNF24;ZNF25;ZNF250;ZNF257;ZNF26;ZNF260;ZNF263;ZNF264;ZNF268;ZNF274;ZNF276;ZNF28;ZNF280A;ZNF280B;ZNF280C;ZNF280D;ZNF281;ZNF282;ZNF283;ZNF284;ZNF285;ZNF286A;ZNF286B;ZNF3;ZNF30;ZNF300;ZNF302;ZNF317;ZNF32;ZNF320;ZNF322;ZNF324;ZNF324B;ZNF329;ZNF331;ZNF333;ZNF33A;ZNF33B;ZNF343;ZNF345;ZNF347;ZNF350;ZNF354A;ZNF354B;ZNF362;ZNF366;ZNF383;ZNF384;ZNF394;ZNF397;ZNF398;ZNF404;ZNF41;ZNF410;ZNF419;ZNF420;ZNF431;ZNF432;ZNF436;ZNF439;ZNF44;ZNF440;ZNF442;ZNF443;ZNF446;ZNF449;ZNF45;ZNF460;ZNF468;ZNF479;ZNF484;ZNF490;ZNF500;ZNF502;ZNF524;ZNF525;ZNF528;ZNF543;ZNF544;ZNF546;ZNF547;ZNF548;ZNF549;ZNF554;ZNF555;ZNF557;ZNF558;ZNF559;ZNF561;ZNF562;ZNF563;ZNF564;ZNF566;ZNF567;ZNF568;ZNF57;ZNF570;ZNF571;ZNF572;ZNF577;ZNF581;ZNF582;ZNF583;ZNF585A;ZNF586;ZNF589;ZNF595;ZNF599;ZNF600;ZNF605;ZNF607;ZNF611;ZNF613;ZNF614;ZNF615;ZNF616;ZNF619;ZNF620;ZNF621;ZNF625;ZNF627;ZNF649;ZNF652;ZNF653;ZNF665;ZNF667;ZNF669;ZNF670;ZNF672;ZNF679;ZNF680;ZNF683;ZNF689;ZNF692;ZNF701;ZNF705D;ZNF705E;ZNF705G;ZNF708;ZNF709;ZNF71;ZNF710;ZNF713;ZNF721;ZNF727;ZNF729;ZNF736;ZNF75A;ZNF75D;ZNF76;ZNF763;ZNF764;ZNF765;ZNF768;ZNF77;ZNF771;ZNF773;ZNF774;ZNF776;ZNF777;ZNF780A;ZNF780B;ZNF782;ZNF785;ZNF799;ZNF805;ZNF808;ZNF81;ZNF813;ZNF816;ZNF823;ZNF829;ZNF836;ZNF841;ZNF844;ZNF845;ZNF846;ZNF85;ZNF853;ZNF860;ZNF878;ZNF891;ZNF99;ZSCAN16;ZSCAN2;ZSCAN22;ZSCAN23;ZSCAN29;ZSCAN31;ZSCAN32;ZSCAN4;ZSCAN5A;ZSCAN5B;ZSCAN5C;ZSCAN9;ZXDA;ZXDB;ZXDC	ZFP82
DDIT3_HUMAN.H11MO.0.D	19	0.07450980	0.7148365	1.932.262	12.092.770	0.43501784	0.7181986	C/EBP-related{1.1.8}	C/EBP{1.1.8.1}	CEBPA;CEBPB;CEBPD;CEBPE;CEBPG;DBP;DDIT3;HLF;NFIL3;TEF	CEBPA;CEBPB;CEBPD;CEBPE;CEBPG;DDIT3
FOXA2_HUMAN.H11MO.0.A	27	0.10588235	0.7792865	1.807.112	12.090.849	0.32361211	0.6364922	Forkhead box (FOX) factors{3.3.1}	FOXA{3.3.1.1}	FOXA1;FOXA2;FOXA3;FOXB1;FOXB2;FOXC1;FOXC2;FOXD1;FOXD2;FOXD3;FOXD4;FOXD4L1;FOXD4L3;FOXD4L4;FOXD4L5;FOXD4L6;FOXE1;FOXE3;FOXF1;FOXF2;FOXG1;FOXH1;FOXI1;FOXI2;FOXI3;FOXJ1;FOXJ2;FOXJ3;FOXK1;FOXK2;FOXL1;FOXL2;FOXM1;FOXN1;FOXN2;FOXN3;FOXN4;FOXO1;FOXO3;FOXO4;FOXO6;FOXP1;FOXP2;FOXP3;FOXP4;FOXQ1;FOXR1;FOXR2;FOXS1	FOXA1;FOXA2;FOXA3
ZN350_HUMAN.H11MO.1.D	59	0.23137255	0.8872957	1.625.054	12.085.672	0.20926814	0.5093814	More than 3 adjacent zinc finger factors{2.3.3}	ZNF350-like factors{2.3.3.30}	BCL6;BCL6B;CTCF;CTCFL;FEZF1;FEZF2;GFI1;GFI1B;GLI1;GLI2;GLI3;GLI4;GLIS1;GLIS2;GLIS3;HKR1;MTF1;MYNN;MZF1;OSR2;OVOL1;OVOL2;PLAG1;PLAGL1;PLAGL2;PRDM1;PRDM14;PRDM6;SCRT1;SCRT2;SNAI1;SNAI2;SNAI3;WT1;YY1;YY2;ZBTB12;ZBTB14;ZBTB18;ZBTB20;ZBTB26;ZBTB42;ZBTB45;ZBTB47;ZBTB48;ZBTB49;ZBTB6;ZBTB7A;ZBTB7B;ZBTB7C;ZFP14;ZFP2;ZFP28;ZFP30;ZFP37;ZFP42;ZFP64;ZFP69;ZFP69B;ZFP82;ZFP91;ZFX;ZIC1;ZIC2;ZIC3;ZIC4;ZIC5;ZIK1;ZIM3;ZKSCAN1;ZKSCAN2;ZKSCAN3;ZKSCAN4;ZNF121;ZNF124;ZNF133;ZNF136;ZNF138;ZNF14;ZNF140;ZNF14	ZNF350

Motif	Nº of probes	% of probes	lower OR	upper OR	OR	p.value	FDR	TF family	TF subfamily	TF.family.member	TF.subfamily.member
										3;ZNF146;ZNF148;ZNF155;ZNF157;ZNF160;ZNF169;ZNF175;ZNF177;ZNF18;ZNF180;ZNF181;ZNF2;ZNF20;ZNF212;ZNF213;ZNF214;ZNF221;ZNF222;ZNF223;ZNF224;ZNF225;ZNF226;ZNF227;ZNF229;ZNF230;ZNF232;ZNF233;ZNF234;ZNF235;ZNF24;ZNF25;ZNF250;ZNF257;ZNF26;ZNF260;ZNF263;ZNF264;ZNF268;ZNF274;ZNF276;ZNF28;ZNF280A;ZNF280B;ZNF280C;ZNF280D;ZNF281;ZNF282;ZNF283;ZNF284;ZNF285;ZNF286A;ZNF286B;ZNF3;ZNF30;ZNF300;ZNF302;ZNF317;ZNF32;ZNF320;ZNF322;ZNF324;ZNF324B;ZNF329;ZNF331;ZNF333;ZNF33A;ZNF33B;ZNF343;ZNF345;ZNF347;ZNF350;ZNF354A;ZNF354B;ZNF362;ZNF366;ZNF383;ZNF384;ZNF394;ZNF397;ZNF398;ZNF404;ZNF41;ZNF410;ZNF419;ZNF420;ZNF431;ZNF432;ZNF436;ZNF439;ZNF44;ZNF440;ZNF442;ZNF443;ZNF446;ZNF449;ZNF45;ZNF460;ZNF468;ZNF479;ZNF484;ZNF490;ZNF500;ZNF502;ZNF524;ZNF525;ZNF528;ZNF543;ZNF544;ZNF546;ZNF547;ZNF548;ZNF549;ZNF554;ZNF555;ZNF557;ZNF558;ZNF559;ZNF561;ZNF562;ZNF563;ZNF564;ZNF566;ZNF567;ZNF568;ZNF57;ZNF570;ZNF571;ZNF572;ZNF577;ZNF581;ZNF582;ZNF583;ZNF585A;ZNF586;ZNF589;ZNF595;ZNF599;ZNF600;ZNF605;ZNF607;ZNF611;ZNF613;ZNF614;ZNF615;ZNF616;ZNF619;ZNF620;ZNF621;ZNF625;ZNF627;ZNF649;ZNF652;ZNF653;ZNF665;ZNF667;ZNF669;ZNF670;ZNF672;ZNF679;ZNF680;ZNF683;ZNF689;ZNF692;ZNF701;ZNF705D;ZNF705E;ZNF705G;ZNF708;ZNF709;ZNF71;ZNF710;ZNF713;ZNF721;ZNF727;ZNF729;ZNF736;ZNF75A;ZNF75D;ZNF76;ZNF763;ZNF764;ZNF765;ZNF768;ZNF77;ZNF771;ZNF773;ZNF774;ZNF776;ZNF777;ZNF780A;ZNF780B;ZNF782;ZNF785;ZNF799;ZNF805;ZNF808;ZNF81;ZNF813;ZNF816;ZNF823;ZNF829;ZNF836;ZNF841;ZNF844;ZNF845;ZNF846;ZNF85;ZNF853;ZNF860;ZNF878;ZNF891;ZNF99;ZSCAN16;ZSCAN2;ZSCAN22;ZSCAN23;ZSCAN29;ZSCAN31;ZSCAN32;ZSCAN4;ZSCAN5A;ZSCAN5B;ZSCAN5C;ZSCAN9;ZXDA;ZXDB;ZXDC	
SOX11_HUMAN.H11MO.0.D	14	0.05490196	0.6499635	2.067.841	12.076.189	0.45334029	0.7312246	SOX-related factors{4.1.1}	Group C{4.1.1.3}	BBX;CIC;HBP1;SOX1;SOX10;SOX11;SOX12;SOX13;SOX14;SOX15;SOX17;SOX18;SOX2;SOX21;SOX3;SOX30;SOX4;SOX5;SOX6;SOX7;SOX8;SOX9;SRY	SOX11;SOX4
TFAP4_HUMAN.H11MO.0.A	50	0.19607843	0.8677483	1.652.678	12.075.153	0.24021584	0.5578506	bHLH-ZIP factors{1.2.6}	AP-4{1.2.6.4}	MAX;MITF;MLX;MLXIP;MLXIPL;MNT;MXD1;MXD3;MXD4;MXI1;MYC;MYCL;MYCN;REPIN1;SREBF1;SREBF2;TFAP4;TFE3;TFEB;TFEC;USF1;USF2	TFAP4
NR4A2_HUMAN.H11MO.0.C	25	0.09803922	0.7626954	1.823.818	12.038.584	0.36269505	0.6684336	NGFI-B-related receptors (NR4){2.1.4}	NURR1 (NR4A2){2.1.4.0.2}	NR4A1;NR4A2;NR4A3	NR4A2
FOXO4_HUMAN.H11MO.0.C	23	0.09019608	0.7477007	1.851.752	12.036.854	0.40646225	0.7005007	Forkhead box (FOX) factors{3.3.1}	FOXO{3.3.1.15}	FOXA1;FOXA2;FOXA3;FOXB1;FOXB2;FOXC1;FOXC2;FOXD1;FOXD2;FOXD3;FOXD4;FOXD4L1;FOXD4L3;FOXD4L4;FOXD4L5;FOXD4L6;FOXE1;FOXE3;FOXF1;FOXF2;FOXG1;FOXH1;FOXI1;FOXI2;FOXI3;FOXJ1;FOXJ2;FOXJ3;FOXK1;FOXK2;FOXL1;FOXL2;FOXM1;FOXN1;FOXN2;FOXN3;FOXN4;FOXO1;FOXO3;FOXO4;FOXO6;FOXP1;FOXP2;FOXP3;FOXP4;FOXQ1;FOXR1;FOXR2;FOXS1	FOXO1;FOXO3;FOXO4;FOXO6
HAND1_HUMAN.H11MO.0.D	44	0.17254902	0.8481729	1.671.371	12.025.400	0.25203487	0.5648163	Tal-related factors{1.2.3}	Twist-like factors{1.2.3.2}	ATOH1;ATOH7;ATOH8;BHLHA15;BHLHA9;BHLHE22;BHLHE23;FERD3L;FIGLA;HAND1;HAND2;LYL1;MESP1;MESP2;MSC;MSGN1;NEUROD1;NEUROD2;NEUROD4;NEUROD6;NEUROG1;NEUROG2;NEUROG3;NHLH1;NHLH2;OLIG1;OLIG2;OLIG3;PTF1A;SCX;TAL1;TAL2;TCF15;TCF21;TCF23;TWIST1;TWIST2	FIGLA;HAND1;PTF1A;TWIST1
NR1H3_HUMAN.H11MO.1.B	33	0.12941176	0.8068685	1.738.616	12.017.616	0.31655784	0.6296813	Thyroid hormone receptor-related factors (NR1){2.1.2}	LXR (NR1H){2.1.2.7}	NR1D1;NR1D2;NR1H2;NR1H3;NR1H4;NR1I2;NR1I3;PPARA;PPARD;PPARG;RARA;RARB;RARG;RORA;RORB;RORC;THRA;THRB;VDR	NR1H2;NR1H3;NR1H4
BHE23_HUMAN.H11MO.0.D	25	0.09803922	0.7606853	1.819.008	12.006.842	0.36366685	0.6684336	Tal-related factors{1.2.3}	Neurogenin / Atonal-like factors{1.2.3.4}	ATOH1;ATOH7;ATOH8;BHLHA15;BHLHA9;BHLHE22;BHLHE23;FERD3L;FIGLA;HAND1;HAND2;LYL1;MESP1;MESP2;MSC;MSGN1;NEUROD1;NEUROD2;NEUROD4;NEUROD6;NEUROG1;NEUROG2;NEUROG3;NHLH1;NHLH2;OLIG1;OLIG2;OLIG3;PTF1A;SCX;TAL1;TAL2;TCF15;TCF21;TCF23;TWIST1;TWIST2	ATOH1;BHLHA15;BHLHE22;BHLHE23;NEUROD1;NEUROD2;NEUROG2;OLIG1;OLIG2;OLIG3

Motif	Nº of probes	% of probes	lower OR	upper OR	OR	p.value	FDR	TF family	TF subfamily	TF.family.member	TF.subfamily.member
MXI1_HUMAN.H11MO.0.A	115	0.45098039	0.9279034	1.545.111	11.983.695	0.16039100	0.4414777	bHLH-ZIP factors{1.2.6}	Mad-like factors{1.2.6.7}	MAX;MITF;MLX;MLXIP;MLXIPL;MNT;MXD1;MXD3;MXD4;MXI1;MYC;MYCL;MYCN;REPIN1;SREBF1;SREBF2;TFAP4;TFE3;TFEB;TFEC;USF1;USF2	MXI1
OSR2_HUMAN.H11MO.0.C	85	0.33333333	0.9093397	1.559.137	11.944.618	0.19156336	0.4842471	More than 3 adjacent zinc finger factors{2.3.3}	unclassified{2.3.3.0}	BCL6B;BCL6;CTCFL;CTCF;FEZF1;GFI1B;GFI1;GLI1;GLI2;GLI3;GLIS1;GLIS2;GLIS3;MTF1;MYNN;MZF1;OSR2;OVOL1;OVOL2;ZNF146;PLAG1;PLAGL1;PRDM14;PRDM1;PRDM6;SCRT1;SCRT2;SNAI1;SNAI2;YY1;YY2;WT1;ZNF324;ZNF354A;ZBTB14;ZBTB18;ZBTB48;ZBTB49;ZBTB7A;ZBTB7B;ZBTB6;ZFP64;ZFP28;ZFP42;ZFP82;ZFX;ZIC1;ZIC2;ZIC3;ZIC4;ZIM3;ZKSCAN1;ZKSCAN3;ZNF121;ZNF136;ZNF140;ZNF143;ZNF148;ZNF214;ZNF232;ZNF250;ZNF257;ZNF260;ZNF263;ZNF264;ZNF274;ZNF281;ZNF282;ZNF317;ZNF320;ZNF322;ZNF329;ZNF331;ZNF333;ZNF350;ZNF384;ZNF394;ZNF410;ZNF436;ZNF449;ZNF490;ZNF502;ZNF524;ZNF528;ZNF547;ZNF549;ZNF554;ZNF563;ZNF582;ZNF586;ZNF589;ZNF652;ZNF667;ZNF680;ZNF708;ZNF713;ZNF768;ZNF816;ZNF18;ZNF41;ZNF76;ZNF85;ZSCAN16;ZSCAN22;ZSCAN31;ZSCAN4	MYNN;MZF1;OSR2;PRDM14;PRDM6;WT1;ZBTB14;ZBTB48;ZBTB49;ZFP64;ZFP28;ZIM3;ZNF121;ZNF250;ZNF257;ZNF263;ZNF274;ZNF317;ZNF320;ZNF329;ZNF331;ZNF394;ZNF449;ZNF502;ZNF528;ZNF547;ZNF549;ZNF554;ZNF586;ZNF589;ZNF667;ZNF680;ZNF708;ZNF713;ZNF768;ZNF18;ZNF85;ZSCAN16;ZSCAN22
PKNX1_HUMAN.H11MO.0.B	39	0.15294118	0.8248746	1.683.851	11.924.450	0.30759921	0.6224645	TALE-type homeo domain factors{3.1.4}	PKNOX{3.1.4.5}	IRX1;IRX2;IRX3;IRX4;IRX5;IRX6;MEIS1;MEIS2;MEIS3;MKX;PBX1;PBX2;PBX3;PKNOX1;PKNOX2;TGIF1;TGIF2;TGIF2LX;TGIF2LY	PKNOX1
OVOL1_HUMAN.H11MO.0.C	21	0.08235294	0.7240028	1.866.903	11.924.038	0.45941305	0.7344357	More than 3 adjacent zinc finger factors{2.3.3}	OVOL-factors{2.3.3.17}	BCL6;BCL6B;CTCF;CTCFL;FEZF1;FEZF2;GFI1;GFI1B;GLI1;GLI2;GLI3;GLI4;GLIS1;GLIS2;GLIS3;HKR1;MTF1;MYNN;MZF1;OSR2;OVOL1;OVOL2;PLAG1;PLAGL1;PLAGL2;PRDM1;PRDM14;PRDM6;SCRT1;SCRT2;SNAI1;SNAI2;SNAI3;WT1;YY1;YY2;ZBTB12;ZBTB14;ZBTB18;ZBTB20;ZBTB26;ZBTB42;ZBTB45;ZBTB47;ZBTB48;ZBTB49;ZBTB6;ZBTB7A;ZBTB7B;ZBTB7C;ZFP14;ZFP2;ZFP28;ZFP30;ZFP37;ZFP42;ZFP64;ZFP69;ZFP69B;ZFP82;ZFP91;ZFX;ZIC1;ZIC2;ZIC3;ZIC4;ZIC5;ZIK1;ZIM3;ZKSCAN1;ZKSCAN2;ZKSCAN3;ZKSCAN4;ZNF121;ZNF124;ZNF133;ZNF136;ZNF138;ZNF14;ZNF140;ZNF143;ZNF146;ZNF148;ZNF155;ZNF157;ZNF160;ZNF169;ZNF175;ZNF177;ZNF18;ZNF180;ZNF181;ZNF2;ZNF20;ZNF212;ZNF213;ZNF214;ZNF221;ZNF222;ZNF223;ZNF224;ZNF225;ZNF226;ZNF227;ZNF229;ZNF230;ZNF232;ZNF233;ZNF234;ZNF235;ZNF24;ZNF25;ZNF250;ZNF257;ZNF26;ZNF260;ZNF263;ZNF264;ZNF268;ZNF274;ZNF276;ZNF28;ZNF280A;ZNF280B;ZNF280C;ZNF280D;ZNF281;ZNF282;ZNF283;ZNF284;ZNF285;ZNF286A;ZNF286B;ZNF3;ZNF30;ZNF300;ZNF302;ZNF317;ZNF32;ZNF320;ZNF322;ZNF324;ZNF324B;ZNF329;ZNF331;ZNF333;ZNF33A;ZNF33B;ZNF343;ZNF345;ZNF347;ZNF350;ZNF354A;ZNF354B;ZNF362;ZNF366;ZNF383;ZNF384;ZNF394;ZNF397;ZNF398;ZNF404;ZNF41;ZNF410;ZNF419;ZNF420;ZNF431;ZNF432;ZNF436;ZNF439;ZNF44;ZNF440;ZNF442;ZNF443;ZNF446;ZNF449;ZNF45;ZNF460;ZNF468;ZNF479;ZNF484;ZNF490;ZNF500;ZNF502;ZNF524;ZNF525;ZNF528;ZNF543;ZNF544;ZNF546;ZNF547;ZNF548;ZNF549;ZNF554;ZNF555;ZNF557;ZNF558;ZNF559;ZNF561;ZNF562;ZNF563;ZNF564;ZNF566;ZNF567;ZNF568;ZNF57;ZNF570;ZNF571;ZNF572;ZNF577;ZNF581;ZNF582;ZNF583;ZNF585A;ZNF586;ZNF589;ZNF595;ZNF599;ZNF600;ZNF605;ZNF607;ZNF611;ZNF613;ZNF614;ZNF615;ZNF616;ZNF619;ZNF620;ZNF621;ZNF625;ZNF627;ZNF649;ZNF652;ZNF653;ZNF665;ZNF667;ZNF669;ZNF670;ZNF672;ZNF679;ZNF680;ZNF683;ZNF689;ZNF692;ZNF701;ZNF705D;ZNF705E;ZNF705G;ZNF708;ZNF709;ZNF71;ZNF710;ZNF713;ZNF721;ZNF727;ZNF729;ZNF736;ZNF75A;ZNF75D;ZNF76;ZNF763;ZNF764;ZNF765;ZNF768;ZNF77;ZNF771;ZNF773;ZNF774;ZNF776;ZNF777;ZNF780A;ZNF780B;ZNF782;ZNF785;ZNF799;ZNF805;ZNF808;ZNF81;ZNF813;ZNF816;ZNF823;ZNF829;ZNF836;ZNF841;ZNF844;ZNF845;ZNF846;ZNF85;ZNF853;ZNF860;ZNF878;ZNF891;ZNF99;ZSCAN16;ZSCAN2;ZSCAN22;ZSCAN23;ZSCAN29;ZSCAN31;ZSCAN32;ZSCAN4;ZSCAN5A;ZSCAN5B;ZSCAN5C;ZSCAN9;ZXDA;ZXDB;ZXDC	OVOL1;OVOL2

Motif	Nº of probes	% of probes	lower OR	upper OR	OR	p.value	FDR	TF family	TF subfamily	TF.family.member	TF.subfamily.member
SALL4_HUMAN.H11MO.0.B	113	0.44313725	0.9219758	1.536.513	11.913.519	0.17909752	0.4667040	Factors with multiple dispersed zinc fingers {2.3.4}	Sal-like factors{2.3.4.3}	BCL11A;BCL11B;BNC1;BNC2;E4F1;HIC1;HIC2;HINFP;HIVEP1;HIVEP2;HIVEP3;IKZF1;IKZF2;IKZF3;IKZF4;IKZF5;INSM1;INSM2;MAZ;MECOM;PATZ1;PRDM16;PRDM4;REST;RLF;RREB1;SALL1;SALL2;SALL3;SALL4;VEZF1;ZBTB1;ZBTB17;ZBTB2;ZBTB25;ZBTB4;ZFAT;ZNF134;ZNF211;ZNF217;ZNF219;ZNF248;ZNF256;ZNF292;ZNF296;ZNF319;ZNF334;ZNF335;ZNF341;ZNF37A;ZNF382;ZNF417;ZNF418;ZNF423;ZNF467;ZNF510;ZNF512;ZNF512B;ZNF516;ZNF518A;ZNF518B;ZNF521;ZNF526;ZNF532;ZNF536;ZNF552;ZNF574;ZNF587;ZNF587B;ZNF592;ZNF639;ZNF654;ZNF658;ZNF671;ZNF687;ZNF711;ZNF717;ZNF770;ZNF772;ZNF784;ZNF786;ZNF792;ZNF8;ZNF814	SALL4
PBX3_HUMAN.H11MO.0.A	47	0.18431373	0.8478108	1.640.809	11.902.089	0.26697539	0.5714541	TALE-type homeo domain factors{3.1.4}	PBX{3.1.4.4}	IRX2;IRX3;MEIS1;MEIS2;MEIS3;PBX1;PBX2;PBX3;PKNOX1;TGIF2LX;TGIF1;TGIF2	PBX1;PBX2;PBX3
STAT3_HUMAN.H11MO.0.A	28	0.10980392	0.7689814	1.758.240	11.837.025	0.39061546	0.6862478	STAT factors{6.2.1}	STAT3{6.2.1.0.3}	STAT1;STAT2;STAT3;STAT4;STAT5A;STAT5B;STAT6	STAT3
RXRA_HUMAN.H11MO.0.A	85	0.33333333	0.9001753	1.543.535	11.824.472	0.21702035	0.5196357	RXR-related receptors (NR2){2.1.3}	Retinoid X receptors (NR2B){2.1.3.1}	HNF4A;HNF4G;NR2C1;NR2C2;NR2E1;NR2E3;NR2F1;NR2F2;NR2F6;RXRA;RXRB;RXRG	RXRA;RXRB;RXRG
BARH2_HUMAN.H11MO.0.D	15	0.05882353	0.6509364	1.990.487	11.820.196	0.47400529	0.7473580	NK-related factors{3.1.2}	BARHL{3.1.2.1}	BARHL1;BARHL2;BARX1;BARX2;BSX;DBX1;DBX2;DLX1;DLX2;DLX3;DLX4;DLX5;DLX6;EMX1;EMX2;EN1;EN2;HHEX;HLX;HMX1;HMX2;HMX3;LBX1;LBX2;MSX1;MSX2;NANOG;NKX1-1;NKX1-2;NKX2-1;NKX2-2;NKX2-3;NKX2-4;NKX2-5;NKX2-6;NKX2-8;NKX3-1;NKX3-2;NKX6-1;NKX6-2;NKX6-3;NOTO;TLX1;TLX2;TLX3;VAX1;VAX2;VENTX	BARHL1;BARHL2
TEAD1_HUMAN.H11MO.0.A	27	0.10588235	0.7597074	1.761.620	11.786.596	0.38534662	0.6838499	TEF-1-related factors{3.6.1}	TEF-1 (TEAD-1, TCF-13){3.6.1.0.1}	TEAD1;TEAD2;TEAD3;TEAD4	TEAD1
CTCF_HUMAN.H11MO.0.A	100	0.39215686	0.9047398	1.521.735	11.755.902	0.21336543	0.5124758	More than 3 adjacent zinc finger factors{2.3.3}	CTCF-like factors{2.3.3.50}	BCL6;BCL6B;CTCF;CTCFL;FEZF1;FEZF2;GFI1;GFI1B;GLI1;GLI2;GLI3;GLI4;GLIS1;GLIS2;GLIS3;HKR1;MTF1;MYNN;MZF1;OSR2;OVOL1;OVOL2;PLAG1;PLAGL1;PLAGL2;PRDM1;PRDM14;PRDM6;SCRT1;SCRT2;SNAI1;SNAI2;SNAI3;WT1;YY1;YY2;ZBTB12;ZBTB14;ZBTB18;ZBTB20;ZBTB26;ZBTB42;ZBTB45;ZBTB47;ZBTB48;ZBTB49;ZBTB6;ZBTB7A;ZBTB7B;ZBTB7C;ZFP14;ZFP2;ZFP28;ZFP30;ZFP37;ZFP42;ZFP64;ZFP69;ZFP69B;ZFP82;ZFP91;ZFX;ZIC1;ZIC2;ZIC3;ZIC4;ZIC5;ZIK1;ZIM3;ZKSCAN1;ZKSCAN2;ZKSCAN3;ZKSCAN4;ZNF121;ZNF124;ZNF133;ZNF136;ZNF138;ZNF14;ZNF140;ZNF143;ZNF146;ZNF148;ZNF155;ZNF157;ZNF160;ZNF169;ZNF175;ZNF177;ZNF18;ZNF180;ZNF181;ZNF2;ZNF20;ZNF212;ZNF213;ZNF214;ZNF221;ZNF222;ZNF223;ZNF224;ZNF225;ZNF226;ZNF227;ZNF229;ZNF230;ZNF232;ZNF233;ZNF234;ZNF235;ZNF24;ZNF25;ZNF250;ZNF257;ZNF26;ZNF260;ZNF263;ZNF264;ZNF268;ZNF274;ZNF276;ZNF28;ZNF280A;ZNF280B;ZNF280C;ZNF280D;ZNF281;ZNF282;ZNF283;ZNF284;ZNF285;ZNF286A;ZNF286B;ZNF3;ZNF30;ZNF300;ZNF302;ZNF317;ZNF32;ZNF320;ZNF322;ZNF324;ZNF324B;ZNF329;ZNF331;ZNF333;ZNF33A;ZNF33B;ZNF343;ZNF345;ZNF347;ZNF350;ZNF354A;ZNF354B;ZNF362;ZNF366;ZNF383;ZNF384;ZNF394;ZNF397;ZNF398;ZNF404;ZNF41;ZNF410;ZNF419;ZNF420;ZNF431;ZNF432;ZNF436;ZNF439;ZNF44;ZNF440;ZNF442;ZNF443;ZNF446;ZNF449;ZNF45;ZNF460;ZNF468;ZNF479;ZNF484;ZNF490;ZNF500;ZNF502;ZNF524;ZNF525;ZNF528;ZNF543;ZNF544;ZNF546;ZNF547;ZNF548;ZNF549;ZNF554;ZNF555;ZNF557;ZNF558;ZNF559;ZNF561;ZNF562;ZNF563;ZNF564;ZNF566;ZNF567;ZNF568;ZNF57;ZNF570;ZNF571;ZNF572;ZNF577;ZNF581;ZNF582;ZNF583;ZNF585A;ZNF586;ZNF589;ZNF595;ZNF599;ZNF600;ZNF605;ZNF607;ZNF611;ZNF613;ZNF614;ZNF615;ZNF616;ZNF619;ZNF620;ZNF621;ZNF625;ZNF627;ZNF649;ZNF652;ZNF653;ZNF665;ZNF667;ZNF669;ZNF670;ZNF672;ZNF679;ZNF680;ZNF683;ZNF689;ZNF692;ZNF701;ZNF705D;ZNF705E;ZNF705G;ZNF708;ZNF709;ZNF71;ZNF710;ZNF713;Z	CTCFL;CTCF

Motif	Nº of probes	% of probes	lower OR	upper OR	OR	p.value	FDR	TF family	TF subfamily	TF.family.member	TF.subfamily.member
										NF721;ZNF727;ZNF729;ZNF736;ZNF75A;ZNF75D;ZNF76;ZNF763;ZNF764;ZNF765;ZNF768;ZNF77;ZNF771;ZNF773;ZNF774;ZNF776;ZNF777;ZNF780A;ZNF780B;ZNF782;ZNF785;ZNF799;ZNF805;ZNF808;ZNF81;ZNF813;ZNF816;ZNF823;ZNF829;ZNF836;ZNF841;ZNF844;ZNF845;ZNF846;ZNF85;ZNF853;ZNF860;ZNF878;ZNF891;ZNF99;ZSCAN16;ZSCAN2;ZSCAN22;ZSCAN23;ZSCAN29;ZSCAN31;ZSCAN32;ZSCAN4;ZSCAN5A;ZSCAN5B;ZSCAN5C;ZSCAN9;ZXDA;ZXDB;ZXDC	
MYOD1_HUMAN.H11MO.0.A	76	0.29803922	0.8865479	1.546.265	11.755.806	0.25582273	0.5648163	MyoD / ASC-related factors{1.2.2}	Myogenic transcription factors{1.2.2.1}	ASCL1;ASCL2;ASCL3;ASCL4;ASCL5;MYF5;MYF6;MYOD1;MYOG	MYF6;MYOD1;MYOG
NFAC1_HUMAN.H11MO.1.B	23	0.09019608	0.7286315	1.804.475	11.729.644	0.48159255	0.7515619	NFAT-related factors{6.1.3}	NFATc1{6.1.3.0.1}	NFAT5;NFATC1;NFATC2;NFATC3;NFATC4	NFATC1
ZN418_HUMAN.H11MO.0.C	31	0.12156863	0.7780400	1.713.473	11.729.350	0.41357346	0.7038965	Factors with multiple dispersed zinc fingers{2.3.4}	ZNF417-like factors{2.3.4.1}	BCL11A;BCL11B;BNC1;BNC2;E4F1;HIC1;HIC2;HINFP;HIVEP1;HIVEP2;HIVEP3;IKZF1;IKZF2;IKZF3;IKZF4;IKZF5;INSM1;INSM2;MAZ;MECOM;PATZ1;PRDM16;PRDM4;REST;RLF;RREB1;SALL1;SALL2;SALL3;SALL4;VEZF1;ZBTB1;ZBTB17;ZBTB2;ZBTB25;ZBTB4;ZFAT;ZNF134;ZNF211;ZNF217;ZNF219;ZNF248;ZNF256;ZNF292;ZNF296;ZNF319;ZNF334;ZNF335;ZNF341;ZNF37A;ZNF382;ZNF417;ZNF418;ZNF423;ZNF467;ZNF510;ZNF512;ZNF512B;ZNF516;ZNF518A;ZNF518B;ZNF521;ZNF526;ZNF532;ZNF536;ZNF552;ZNF574;ZNF587;ZNF587B;ZNF592;ZNF639;ZNF654;ZNF658;ZNF671;ZNF687;ZNF711;ZNF717;ZNF770;ZNF772;ZNF784;ZNF786;ZNF792;ZNF8;ZNF814	ZNF418
TBP_HUMAN.H11MO.0.A	17	0.06666667	0.6710251	1.918.029	11.719.443	0.49981049	0.7691694	TBP-related factors{8.1.1}	TBP{8.1.1.0.1}	TBP;TBPL1;TBPL2	TBP
HMX3_HUMAN.H11MO.0.D	11	0.04313725	0.5768960	2.135.948	11.717.899	0.61589478	0.8464226	NK-related factors{3.1.2}	NK-5/HMX{3.1.2.18}	BARHL1;BARHL2;BARX1;BARX2;BSX;DBX1;DBX2;DLX1;DLX2;DLX3;DLX4;DLX5;DLX6;EMX1;EMX2;EN1;EN2;HHEX;HLX;HMX1;HMX2;HMX3;LBX1;LBX2;MSX1;MSX2;NANOG;NKX1-1;NKX1-2;NKX2-1;NKX2-2;NKX2-3;NKX2-4;NKX2-5;NKX2-6;NKX2-8;NKX3-1;NKX3-2;NKX6-1;NKX6-2;NKX6-3;NOTO;TLX1;TLX2;TLX3;VAX1;VAX2;VENTX	HMX1;HMX2;HMX3
ZBT18_HUMAN.H11MO.0.C	49	0.19215686	0.8389935	1.606.123	11.708.175	0.31569986	0.6296813	More than 3 adjacent zinc finger factors{2.3.3}	ZNF238-like factors{2.3.3.16}	BCL6;BCL6B;CTCF;CTCFL;FEZF1;FEZF2;GFI1;GFI1B;GLI1;GLI2;GLI3;GLI4;GLIS1;GLIS2;GLIS3;HKR1;MTF1;MYNN;MZF1;OSR2;OVOL1;OVOL2;PLAG1;PLAGL1;PLAGL2;PRDM1;PRDM14;PRDM6;SCRT1;SCRT2;SNAI1;SNAI2;SNAI3;WT1;YY1;YY2;ZBTB12;ZBTB14;ZBTB18;ZBTB20;ZBTB26;ZBTB42;ZBTB45;ZBTB47;ZBTB48;ZBTB49;ZBTB6;ZBTB7A;ZBTB7B;ZBTB7C;ZFP14;ZFP2;ZFP28;ZFP30;ZFP37;ZFP42;ZFP64;ZFP69;ZFP69B;ZFP82;ZFP91;ZFX;ZIC1;ZIC2;ZIC3;ZIC4;ZIC5;ZIK1;ZIM3;ZKSCAN1;ZKSCAN2;ZKSCAN3;ZKSCAN4;ZNF121;ZNF124;ZNF133;ZNF136;ZNF138;ZNF14;ZNF140;ZNF143;ZNF146;ZNF148;ZNF155;ZNF157;ZNF160;ZNF169;ZNF175;ZNF177;ZNF18;ZNF180;ZNF181;ZNF2;ZNF20;ZNF212;ZNF213;ZNF214;ZNF221;ZNF222;ZNF223;ZNF224;ZNF225;ZNF226;ZNF227;ZNF229;ZNF230;ZNF232;ZNF233;ZNF234;ZNF235;ZNF24;ZNF25;ZNF250;ZNF257;ZNF26;ZNF260;ZNF263;ZNF264;ZNF268;ZNF274;ZNF276;ZNF28;ZNF280A;ZNF280B;ZNF280C;ZNF280D;ZNF281;ZNF282;ZNF283;ZNF284;ZNF285;ZNF286A;ZNF286B;ZNF3;ZNF30;ZNF300;ZNF302;ZNF317;ZNF32;ZNF320;ZNF322;ZNF324;ZNF324B;ZNF329;ZNF331;ZNF333;ZNF33A;ZNF33B;ZNF343;ZNF345;ZNF347;ZNF350;ZNF354A;ZNF354B;ZNF362;ZNF366;ZNF383;ZNF384;ZNF394;ZNF397;ZNF398;ZNF404;ZNF41;ZNF410;ZNF419;ZNF420;ZNF431;ZNF432;ZNF436;ZNF439;ZNF44;ZNF440;ZNF442;ZNF443;ZNF446;ZNF449;ZNF45;ZNF460;ZNF468;ZNF479;ZNF484;ZNF490;ZNF500;ZNF502;ZNF524;ZNF525;ZNF528;ZNF543;ZNF544;ZNF546;ZNF547;ZNF548;ZNF549;ZNF554;ZNF555;ZNF557;ZNF558;ZNF559;ZNF561;ZNF562;ZNF563;ZNF564;ZNF566;ZNF567;ZNF568;ZNF57;ZNF570;ZNF571;ZNF572;ZNF577;ZNF5	ZBTB18

Motif	Nº of probes	% of probes	lower OR	upper OR	OR	p.value	FDR	TF family	TF subfamily	TF.family.member	TF.subfamily.member
										81;ZNF582;ZNF583;ZNF585A;ZNF586;ZNF589;ZNF595;ZNF599;ZNF600;ZNF605;ZNF607;ZNF611;ZNF613;ZNF614;ZNF615;ZNF616;ZNF619;ZNF620;ZNF621;ZNF625;ZNF627;ZNF649;ZNF652;ZNF653;ZNF665;ZNF667;ZNF669;ZNF670;ZNF672;ZNF679;ZNF680;ZNF683;ZNF689;ZNF692;ZNF701;ZNF705D;ZNF705E;ZNF705G;ZNF708;ZNF709;ZNF71;ZNF710;ZNF713;ZNF721;ZNF727;ZNF729;ZNF736;ZNF75A;ZNF75D;ZNF76;ZNF763;ZNF764;ZNF765;ZNF768;ZNF77;ZNF771;ZNF773;ZNF774;ZNF776;ZNF777;ZNF780A;ZNF780B;ZNF782;ZNF785;ZNF799;ZNF805;ZNF808;ZNF81;ZNF813;ZNF816;ZNF823;ZNF829;ZNF836;ZNF841;ZNF844;ZNF845;ZNF846;ZNF85;ZNF853;ZNF860;ZNF878;ZNF891;ZNF99;ZSCAN16;ZSCAN2;ZSCAN22;ZSCAN23;ZSCAN29;ZSCAN31;ZSCAN32;ZSCAN4;ZSCAN5A;ZSCAN5B;ZSCAN5C;ZSCAN9;ZXDA;ZXDB;ZXDC	
RFX5_HUMAN.H11MO.0.A	49	0.19215686	0.8388093	1.605.768	11.705.598	0.31577280	0.6296813	RFX-related factors{3.3.3}	RFX5{3.3.3.0.5}	RFX1;RFX2;RFX3;RFX4;RFX5;RFX6;RFX7;RFX8	RFX5
SUH_HUMAN.H11MO.0.A	65	0.25490196	0.8687176	1.559.076	11.702.383	0.29399147	0.6028389	CSL-related factors{6.1.4}	M{6.1.4.1}	RBPJ;RBPJL	RBPJ
ERR2_HUMAN.H11MO.0.A	25	0.09803922	0.7405682	1.770.919	11.689.544	0.43189923	0.7146267	Steroid hormone receptors (NR3){2.1.1}	ER-like receptors (NR3A&B){2.1.1.2}	AR;ESR1;ESR2;ESRRA;ESRRB;ESRRG;NR3C1;NR3C2;PGR	ESRRA;ESRRB;ESRRG;ESR1;ESR2
STAT2_HUMAN.H11MO.0.A	52	0.20392157	0.8431351	1.590.230	11.670.119	0.32736848	0.6422420	STAT factors{6.2.1}	STAT2{6.2.1.0.2}	STAT1;STAT2;STAT3;STAT4;STAT5A;STAT5B;STAT6	STAT2
PLAL1_HUMAN.H11MO.0.D	75	0.29411765	0.8789585	1.536.478	11.669.888	0.25559023	0.5648163	More than 3 adjacent zinc finger factors{2.3.3}	PLAG factors{2.3.3.25}	BCL6;BCL6B;CTCF;CTCFL;FEZF1;FEZF2;GFI1;GFI1B;GLI1;GLI2;GLI3;GLI4;GLIS1;GLIS2;GLIS3;HKR1;MTF1;MYNN;MZF1;OSR2;OVOL1;OVOL2;PLAG1;PLAGL1;PLAGL2;PRDM1;PRDM14;PRDM6;SCRT1;SCRT2;SNAI1;SNAI2;SNAI3;WT1;YY1;YY2;ZBTB12;ZBTB14;ZBTB18;ZBTB20;ZBTB26;ZBTB42;ZBTB45;ZBTB47;ZBTB48;ZBTB49;ZBTB6;ZBTB7A;ZBTB7B;ZBTB7C;ZFP14;ZFP2;ZFP28;ZFP30;ZFP37;ZFP42;ZFP64;ZFP69;ZFP69B;ZFP82;ZFP91;ZFX;ZIC1;ZIC2;ZIC3;ZIC4;ZIC5;ZIK1;ZIM3;ZKSCAN1;ZKSCAN2;ZKSCAN3;ZKSCAN4;ZNF121;ZNF124;ZNF133;ZNF136;ZNF138;ZNF14;ZNF140;ZNF143;ZNF146;ZNF148;ZNF155;ZNF157;ZNF160;ZNF169;ZNF175;ZNF177;ZNF18;ZNF180;ZNF181;ZNF2;ZNF20;ZNF212;ZNF213;ZNF214;ZNF221;ZNF222;ZNF223;ZNF224;ZNF225;ZNF226;ZNF227;ZNF229;ZNF230;ZNF232;ZNF233;ZNF234;ZNF235;ZNF24;ZNF25;ZNF250;ZNF257;ZNF26;ZNF260;ZNF263;ZNF264;ZNF268;ZNF274;ZNF276;ZNF28;ZNF280A;ZNF280B;ZNF280C;ZNF280D;ZNF281;ZNF282;ZNF283;ZNF284;ZNF285;ZNF286A;ZNF286B;ZNF3;ZNF30;ZNF300;ZNF302;ZNF317;ZNF32;ZNF320;ZNF322;ZNF324;ZNF324B;ZNF329;ZNF331;ZNF333;ZNF33A;ZNF33B;ZNF343;ZNF345;ZNF347;ZNF350;ZNF354A;ZNF354B;ZNF362;ZNF366;ZNF383;ZNF384;ZNF394;ZNF397;ZNF398;ZNF404;ZNF41;ZNF410;ZNF419;ZNF420;ZNF431;ZNF432;ZNF436;ZNF439;ZNF44;ZNF440;ZNF442;ZNF443;ZNF446;ZNF449;ZNF45;ZNF460;ZNF468;ZNF479;ZNF484;ZNF490;ZNF500;ZNF502;ZNF524;ZNF525;ZNF528;ZNF543;ZNF544;ZNF546;ZNF547;ZNF548;ZNF549;ZNF554;ZNF555;ZNF557;ZNF558;ZNF559;ZNF561;ZNF562;ZNF563;ZNF564;ZNF566;ZNF567;ZNF568;ZNF57;ZNF570;ZNF571;ZNF572;ZNF577;ZNF581;ZNF582;ZNF583;ZNF585A;ZNF586;ZNF589;ZNF595;ZNF599;ZNF600;ZNF605;ZNF607;ZNF611;ZNF613;ZNF614;ZNF615;ZNF616;ZNF619;ZNF620;ZNF621;ZNF625;ZNF627;ZNF649;ZNF652;ZNF653;ZNF665;ZNF667;ZNF669;ZNF670;ZNF672;ZNF679;ZNF680;ZNF683;ZNF689;ZNF692;ZNF701;ZNF705D;ZNF705E;ZNF705G;ZNF708;ZNF709;ZNF71;ZNF710;ZNF713;ZNF721;ZNF727;ZNF729;ZNF736;ZNF75A;ZNF75D;ZNF76;ZNF763;ZNF764;ZNF765;ZNF768;ZNF77;ZNF771;ZNF773;ZNF774;ZNF776;ZNF777;ZNF780A;ZNF780B;ZNF782;ZNF785;ZNF799;ZNF805;ZNF808;ZNF81;ZNF813;ZNF816;ZNF823;ZNF829;ZNF836;ZNF841;ZNF844;ZNF845;ZNF846;ZNF85;ZNF853;Z	PLAG1;PLAGL1

Motif	Nº of probes	% of probes	lower OR	upper OR	OR	p.value	FDR	TF family	TF subfamily	TF.family.member	TF.subfamily.member
										NF860;ZNF878;ZNF891;ZNF99;ZSCAN16;ZSCAN2;ZSCAN22;ZSCAN23;ZSCAN29;ZSCAN31;ZSCAN32;ZSCAN4;ZSCAN5A;ZSCAN5B;ZSCAN5C;ZSCAN9;ZXDA;ZXDB;ZXDC	
HSF1_HUMAN.H11MO.1.A	23	0.09019608	0.7247375	1.794.824	11.666.936	0.48276389	0.7515619	HSF factors{3.4.1}	HSF1 (HSTF1){3.4.1.0.1}	HSF1;HSF2;HSF4;HSF5;HSFX1;HSFY1;HSFY1; HSFY2	HSF1
NFIB_HUMAN.H11MO.0.D	55	0.21568627	0.8474602	1.576.985	11.643.644	0.33833640	0.6520406	Nuclear factor 1{7.1.2}	NF-1B (NF-IB){7.1.2.0.2}	NFIA;NFIB;NFIC	NFIB
ZN136_HUMAN.H11MO.0.C	16	0.06274510	0.6539558	1.930.031	11.633.804	0.57861992	0.8240120	More than 3 adjacent zinc finger factors{2.3.3}	ZNF763-like factors{2.3.3.33}	BCL6;BCL6B;CTCF;CTCFL;FEZF1;FEZF2;GFI1;GFI1B;GLI1;GLI2;GLI3;GLI4;GLIS1;GLIS2;GLIS3;HKR1;MTF1;MYNN;MZF1;OSR2;OVOL1;OVOL2;PLAG1;PLAGL1;PLAGL2;PRDM1;PRDM14;PRDM6;SCRT1;SCRT2;SNAI1;SNAI2;SNAI3;WT1;YY1;YY2;ZBTB12;ZBTB14;ZBTB18;ZBTB20;ZBTB26;ZBTB42;ZBTB45;ZBTB47;ZBTB48;ZBTB49;ZBTB6;ZBTB7A;ZBTB7B;ZBTB7C;ZFP14;ZFP2;ZFP28;ZFP30;ZFP37;ZFP42;ZFP64;ZFP69;ZFP69B;ZFP82;ZFP91;ZFX;ZIC1;ZIC2;ZIC3;ZIC4;ZIC5;ZIK1;ZIM3;ZKSCAN1;ZKSCAN2;ZKSCAN3;ZKSCAN4;ZNF121;ZNF124;ZNF133;ZNF136;ZNF138;ZNF14;ZNF140;ZNF143;ZNF146;ZNF148;ZNF155;ZNF157;ZNF160;ZNF169;ZNF175;ZNF177;ZNF18;ZNF180;ZNF181;ZNF2;ZNF20;ZNF212;ZNF213;ZNF214;ZNF221;ZNF222;ZNF223;ZNF224;ZNF225;ZNF226;ZNF227;ZNF229;ZNF230;ZNF232;ZNF233;ZNF234;ZNF235;ZNF24;ZNF25;ZNF250;ZNF257;ZNF26;ZNF260;ZNF263;ZNF264;ZNF268;ZNF274;ZNF276;ZNF28;ZNF280A;ZNF280B;ZNF280C;ZNF280D;ZNF281;ZNF282;ZNF283;ZNF284;ZNF285;ZNF286A;ZNF286B;ZNF3;ZNF30;ZNF300;ZNF302;ZNF317;ZNF32;ZNF320;ZNF322;ZNF324;ZNF324B;ZNF329;ZNF331;ZNF333;ZNF33A;ZNF33B;ZNF343;ZNF345;ZNF347;ZNF350;ZNF354A;ZNF354B;ZNF362;ZNF366;ZNF383;ZNF384;ZNF394;ZNF397;ZNF398;ZNF404;ZNF41;ZNF410;ZNF419;ZNF420;ZNF431;ZNF432;ZNF436;ZNF439;ZNF44;ZNF440;ZNF442;ZNF443;ZNF446;ZNF449;ZNF45;ZNF460;ZNF468;ZNF479;ZNF484;ZNF490;ZNF500;ZNF502;ZNF524;ZNF525;ZNF528;ZNF543;ZNF544;ZNF546;ZNF547;ZNF548;ZNF549;ZNF554;ZNF555;ZNF557;ZNF558;ZNF559;ZNF561;ZNF562;ZNF563;ZNF564;ZNF566;ZNF567;ZNF568;ZNF57;ZNF570;ZNF571;ZNF572;ZNF577;ZNF581;ZNF582;ZNF583;ZNF585A;ZNF586;ZNF589;ZNF595;ZNF599;ZNF600;ZNF605;ZNF607;ZNF611;ZNF613;ZNF614;ZNF615;ZNF616;ZNF619;ZNF620;ZNF621;ZNF625;ZNF627;ZNF649;ZNF652;ZNF653;ZNF665;ZNF667;ZNF669;ZNF670;ZNF672;ZNF679;ZNF680;ZNF683;ZNF689;ZNF692;ZNF701;ZNF705D;ZNF705E;ZNF705G;ZNF708;ZNF709;ZNF71;ZNF710;ZNF713;ZNF721;ZNF727;ZNF729;ZNF736;ZNF75A;ZNF75D;ZNF76;ZNF763;ZNF764;ZNF765;ZNF768;ZNF77;ZNF771;ZNF773;ZNF774;ZNF776;ZNF777;ZNF780A;ZNF780B;ZNF782;ZNF785;ZNF799;ZNF805;ZNF808;ZNF81;ZNF813;ZNF816;ZNF823;ZNF829;ZNF836;ZNF841;ZNF844;ZNF845;ZNF846;ZNF85;ZNF853;ZNF860;ZNF878;ZNF891;ZNF99;ZSCAN16;ZSCAN2;ZSCAN22;ZSCAN23;ZSCAN29;ZSCAN31;ZSCAN32;ZSCAN4;ZSCAN5A;ZSCAN5B;ZSCAN5C;ZSCAN9;ZXDA;ZXDB;ZXDC	ZNF136;ZNF490;ZNF563
FOXC1_HUMAN.H11MO.0.C	21	0.08235294	0.7056281	1.819.531	11.621.597	0.46686222	0.7406394	Forkhead box (FOX) factors{3.3.1}	FOXC{3.3.1.3}	FOXA1;FOXA2;FOXA3;FOXB1;FOXB2;FOXC1;FOXC2;FOXD1;FOXD2;FOXD3;FOXD4;FOXD4L1;FOXD4L3;FOXD4L4;FOXD4L5;FOXD4L6;FOXE1;FOXE3;FOXF1;FOXF2;FOXG1;FOXH1;FOXI1;FOXI2;FOXI3;FOXJ1;FOXJ2;FOXJ3;FOXK1;FOXK2;FOXL1;FOXL2;FOXM1;FOXN1;FOXN2;FOXN3;FOXN4;FOXO1;FOXO3;FOXO4;FOXO6;FOXP1;FOXP2;FOXP3;FOXP4;FOXQ1;FOXR1;FOXR2;FOXS1	FOXC1;FOXC2
ZBT7B_HUMAN.H11MO.0.D	64	0.25098039	0.8598224	1.547.968	11.602.306	0.29363267	0.6028389	More than 3 adjacent zinc finger factors{2.3.3}	ZBTB7 factors{2.3.3.8}	BCL6;BCL6B;CTCF;CTCFL;FEZF1;FEZF2;GFI1;GFI1B;GLI1;GLI2;GLI3;GLI4;GLIS1;GLIS2;GLIS3;HKR1;MTF1;MYNN;MZF1;OSR2;OVOL1;OVOL2;PLAG1;PLAGL1;PLAGL2;PRDM1;PRDM14;PRDM6;SCRT1;SCRT2;SNAI1;SNAI2;SNAI3;WT1;YY1;YY2;ZBTB12;ZBTB14;ZBTB18;ZBTB20;ZBTB26;ZBTB42;ZBTB45;ZBTB47;ZBTB48;ZBTB49;ZBTB6;ZBTB7A;ZBTB7B;ZBTB7C;ZFP14;ZFP2;ZFP28;ZFP30;ZFP37;ZFP42;ZFP64;	ZBTB7A;ZBTB7B

Motif	Nº of probes	% of probes	lower OR	upper OR	OR	p.value	FDR	TF family	TF subfamily	TF.family.member	TF.subfamily.member
										ZFP69;ZFP69B;ZFP82;ZFP91;ZFX;ZIC1;ZIC2;ZIC3;ZIC4;ZIC5;ZIK1;ZIM3;ZKSCAN1;ZKSCAN2;ZKSCAN3;ZKSCAN4;ZNF121;ZNF124;ZNF133;ZNF136;ZNF138;ZNF14;ZNF140;ZNF143;ZNF146;ZNF148;ZNF155;ZNF157;ZNF160;ZNF169;ZNF175;ZNF177;ZNF18;ZNF180;ZNF181;ZNF2;ZNF20;ZNF212;ZNF213;ZNF214;ZNF221;ZNF222;ZNF223;ZNF224;ZNF225;ZNF226;ZNF227;ZNF229;ZNF230;ZNF232;ZNF233;ZNF234;ZNF235;ZNF24;ZNF25;ZNF250;ZNF257;ZNF26;ZNF260;ZNF263;ZNF264;ZNF268;ZNF274;ZNF276;ZNF28;ZNF280A;ZNF280B;ZNF280C;ZNF280D;ZNF281;ZNF282;ZNF283;ZNF284;ZNF285;ZNF286A;ZNF286B;ZNF3;ZNF30;ZNF300;ZNF302;ZNF317;ZNF32;ZNF320;ZNF322;ZNF324;ZNF324B;ZNF329;ZNF331;ZNF333;ZNF33A;ZNF33B;ZNF343;ZNF345;ZNF347;ZNF350;ZNF354A;ZNF354B;ZNF362;ZNF366;ZNF383;ZNF384;ZNF394;ZNF397;ZNF398;ZNF404;ZNF41;ZNF410;ZNF419;ZNF420;ZNF431;ZNF432;ZNF436;ZNF439;ZNF44;ZNF440;ZNF442;ZNF443;ZNF446;ZNF449;ZNF45;ZNF460;ZNF468;ZNF479;ZNF484;ZNF490;ZNF500;ZNF502;ZNF524;ZNF525;ZNF528;ZNF543;ZNF544;ZNF546;ZNF547;ZNF548;ZNF549;ZNF554;ZNF555;ZNF557;ZNF558;ZNF559;ZNF561;ZNF562;ZNF563;ZNF564;ZNF566;ZNF567;ZNF568;ZNF57;ZNF570;ZNF571;ZNF572;ZNF577;ZNF581;ZNF582;ZNF583;ZNF585A;ZNF586;ZNF589;ZNF595;ZNF599;ZNF600;ZNF605;ZNF607;ZNF611;ZNF613;ZNF614;ZNF615;ZNF616;ZNF619;ZNF620;ZNF621;ZNF625;ZNF627;ZNF649;ZNF652;ZNF653;ZNF665;ZNF667;ZNF669;ZNF670;ZNF672;ZNF679;ZNF680;ZNF683;ZNF689;ZNF692;ZNF701;ZNF705D;ZNF705E;ZNF705G;ZNF708;ZNF709;ZNF71;ZNF710;ZNF713;ZNF721;ZNF727;ZNF729;ZNF736;ZNF75A;ZNF75D;ZNF76;ZNF763;ZNF764;ZNF765;ZNF768;ZNF77;ZNF771;ZNF773;ZNF774;ZNF776;ZNF777;ZNF780A;ZNF780B;ZNF782;ZNF785;ZNF799;ZNF805;ZNF808;ZNF81;ZNF813;ZNF816;ZNF823;ZNF829;ZNF836;ZNF841;ZNF844;ZNF845;ZNF846;ZNF85;ZNF853;ZNF860;ZNF878;ZNF891;ZNF99;ZSCAN16;ZSCAN2;ZSCAN22;ZSCAN23;ZSCAN29;ZSCAN31;ZSCAN32;ZSCAN4;ZSCAN5A;ZSCAN5B;ZSCAN5C;ZSCAN9;ZXDA;ZXDB;ZXDC	
TF7L2_HUMAN.H11MO.0.A	19	0.07450980	0.6857516	1.853.520	11.600.189	0.52343902	0.7913166	TCF-7-related factors{4.1.3}	TCF-7L2 (TCF-4) [1]{4.1.3.0.3}	LEF1;TAF1;TCF7;TCF7L1;TCF7L2	TCF7L2
DUXA_HUMAN.H11MO.0.D	11	0.04313725	0.5703565	2.111.694	11.584.951	0.61807511	0.8464226	Paired-related HD factors{3.1.3}	DUX{3.1.3.7}	ALX1;ALX3;ALX4;ARGFX;ARX;CRX;DMBX1;DPRX;DRGX;DUX4;DUXA;ESX1;GSC;GSC2;HESX1;ISX;LEUTX;MIXL1;NOBOX;OTP;OTX1;OTX2;PHOX2A;PHOX2B;PITX1;PITX2;PITX3;PROP1;PRRX1;PRRX2;RAX;RAX2;RHOXF1;RHOXF2;SEBOX;SHOX;SHOX2;TPRX1;UNCX;VSX1;VSX2	DUX4;DUXA
ZBT48_HUMAN.H11MO.0.C	39	0.15294118	0.8012108	1.635.436	11.581.621	0.40835721	0.7005007	More than 3 adjacent zinc finger factors{2.3.3}	unclassified{2.3.3.0}	BCL6B;BCL6;CTCFL;CTCF;FEZF1;GFI1B;GFI1;GLI1;GLI2;GLI3;GLIS1;GLIS2;GLIS3;MTF1;MYNN;MZF1;OSR2;OVOL1;OVOL2;ZNF146;PLAG1;PLAGL1;PRDM14;PRDM1;PRDM6;SCRT1;SCRT2;SNAI1;SNAI2;YY1;YY2;WT1;ZNF324;ZNF354A;ZBTB14;ZBTB18;ZBTB48;ZBTB49;ZBTB7A;ZBTB7B;ZBTB6;ZFP64;ZFP28;ZFP42;ZFP82;ZFX;ZIC1;ZIC2;ZIC3;ZIC4;ZIM3;ZKSCAN1;ZKSCAN3;ZNF121;ZNF136;ZNF140;ZNF143;ZNF148;ZNF214;ZNF232;ZNF250;ZNF257;ZNF260;ZNF263;ZNF264;ZNF274;ZNF281;ZNF282;ZNF317;ZNF320;ZNF322;ZNF329;ZNF331;ZNF333;ZNF350;ZNF384;ZNF394;ZNF410;ZNF436;ZNF449;ZNF490;ZNF502;ZNF524;ZNF528;ZNF547;ZNF549;ZNF554;ZNF563;ZNF582;ZNF586;ZNF589;ZNF652;ZNF667;ZNF680;ZNF708;ZNF713;ZNF768;ZNF816;ZNF18;ZNF41;ZNF76;ZNF85;ZSCAN16;ZSCAN22;ZSCAN31;ZSCAN4	MYNN;MZF1;OSR2;PRDM14;PRDM6;WT1;ZBTB14;ZBTB48;ZBTB49;ZFP64;ZFP28;ZIM3;ZNF121;ZNF250;ZNF257;ZNF263;ZNF274;ZNF317;ZNF320;ZNF329;ZNF331;ZNF394;ZNF449;ZNF502;ZNF528;ZNF547;ZNF549;ZNF554;ZNF586;ZNF589;ZNF667;ZNF680;ZNF708;ZNF713;ZNF768;ZNF18;ZNF85;ZSCAN16;ZSCAN22
ARNT2_HUMAN.H11MO.0.D	118	0.46274510	0.8971705	1.492.261	11.578.100	0.25419691	0.5648163	PAS domain factors{1.2.5}	Arnt-like factors{1.2.5.2}	AHR;AHRR;ARNT;ARNT2;ARNTL;ARNTL2;CLOCK;EPAS1;HIF1A;HIF3A;NCOA1;NCOA2;NCOA3;NPAS1;NPAS2;NPAS3;NPAS4;SIM1;SIM2;SOHLH1;SOHLH2;TCFL5	ARNT2;ARNT;ARNTL;CLOCK
PITX1_HUMAN.H11MO.0.D	15	0.05882353	0.6373035	1.948.935	11.572.647	0.56800066	0.8230143	Paired-related HD factors{3.1.3}	PITX{3.1.3.19}	ALX1;ALX3;ALX4;ARGFX;ARX;CRX;DMBX1;DPRX;DRGX;DUX4;DUXA;ESX1;GSC;GSC2;HESX1;ISX;LEUTX;MIXL1;NOBOX;OTP;OTX1;OTX2;PHOX2A;PHOX2B;PITX1;PITX2;	PITX1;PITX2;PITX3

Motif	Nº of probes	% of probes	lower OR	upper OR	OR	p.value	FDR	TF family	TF subfamily	TF.family.member	TF.subfamily.member
										PITX3;PROP1;PRRX1;PRRX2;RAX;RAX2;RHOXF1;RHOXF2;SEBOX;SHOX;SHOX2;TPRX1;UNCX;VSX1;VSX2	
ZN586_HUMAN.H11MO.0.C	39	0.15294118	0.7995364	1.632.062	11.557.895	0.40885254	0.7005007	More than 3 adjacent zinc finger factors{2.3.3}	unclassified{2.3.3.0}	BCL6B;BCL6;CTCFL;CTCF;FEZF1;GFI1B;GFI1;GLI1;GLI2;GLI3;GLIS1;GLIS2;GLIS3;MTF1;MYNN;MZF1;OSR2;OVOL1;OVOL2;ZNF146;PLAG1;PLAGL1;PRDM14;PRDM1;PRDM6;SCRT1;SCRT2;SNAI1;SNAI2;YY1;YY2;WT1;ZNF324;ZNF354A;ZBTB14;ZBTB18;ZBTB48;ZBTB49;ZBTB7A;ZBTB7B;ZBTB6;ZFP64;ZFP28;ZFP42;ZFP82;ZFX;ZIC1;ZIC2;ZIC3;ZIC4;ZIM3;ZKSCAN1;ZKSCAN3;ZNF121;ZNF136;ZNF140;ZNF143;ZNF148;ZNF214;ZNF232;ZNF250;ZNF257;ZNF260;ZNF263;ZNF264;ZNF274;ZNF281;ZNF282;ZNF317;ZNF320;ZNF322;ZNF329;ZNF331;ZNF333;ZNF350;ZNF384;ZNF394;ZNF410;ZNF436;ZNF449;ZNF490;ZNF502;ZNF524;ZNF528;ZNF547;ZNF549;ZNF554;ZNF563;ZNF582;ZNF586;ZNF589;ZNF652;ZNF667;ZNF680;ZNF708;ZNF713;ZNF768;ZNF816;ZNF18;ZNF41;ZNF76;ZNF85;ZSCAN16;ZSCAN22;ZSCAN31;ZSCAN4	MYNN;MZF1;OSR2;PRDM14;PRDM6;WT1;ZBTB14;ZBTB48;ZBTB49;ZFP64;ZFP28;ZIM3;ZNF121;ZNF250;ZNF257;ZNF263;ZNF274;ZNF317;ZNF320;ZNF329;ZNF331;ZNF394;ZNF449;ZNF502;ZNF528;ZNF547;ZNF549;ZNF554;ZNF586;ZNF589;ZNF667;ZNF680;ZNF708;ZNF713;ZNF768;ZNF18;ZNF85;ZSCAN16;ZSCAN22
NFIL3_HUMAN.H11MO.0.D	14	0.05490196	0.6218403	1.978.153	11.553.583	0.55638128	0.8177760	C/EBP-related{1.1.8}	PAR factors{1.1.8.2}	CEBPA;CEBPB;CEBPD;CEBPE;CEBPG;DBP;DDIT3;HLF;NFIL3;TEF	DBP;HLF;NFIL3;TEF
NFAC1_HUMAN.H11MO.0.B	41	0.16078431	0.8049567	1.618.730	11.540.476	0.41893630	0.7063752	NFAT-related factors{6.1.3}	NFATc1{6.1.3.0.1}	NFAT5;NFATC1;NFATC2;NFATC3;NFATC4	NFATC1
NKX21_HUMAN.H11MO.0.A	33	0.12941176	0.7719723	1.663.314	11.497.448	0.43153100	0.7146267	NK-related factors{3.1.2}	NK-2.1{3.1.2.14}	BARHL1;BARHL2;BARX1;BARX2;BSX;DBX1;DBX2;DLX1;DLX2;DLX3;DLX4;DLX5;DLX6;EMX1;EMX2;EN1;EN2;HHEX;HLX;HMX1;HMX2;HMX3;LBX1;LBX2;MSX1;MSX2;NANOG;NKX1-1;NKX1-2;NKX2-1;NKX2-2;NKX2-3;NKX2-4;NKX2-5;NKX2-6;NKX2-8;NKX3-1;NKX3-2;NKX6-1;NKX6-2;NKX6-3;NOTO;TLX1;TLX2;TLX3;VAX1;VAX2;VENTX	NKX2-1
FOXO1_HUMAN.H11MO.0.A	35	0.13725490	0.7799464	1.647.632	11.489.901	0.44263042	0.7245606	Forkhead box (FOX) factors{3.3.1}	FOXO{3.3.1.15}	FOXA1;FOXA2;FOXA3;FOXB1;FOXB2;FOXC1;FOXC2;FOXD1;FOXD2;FOXD3;FOXD4;FOXD4L1;FOXD4L3;FOXD4L4;FOXD4L5;FOXD4L6;FOXE1;FOXE3;FOXF1;FOXF2;FOXG1;FOXH1;FOXI1;FOXI2;FOXI3;FOXJ1;FOXJ2;FOXJ3;FOXK1;FOXK2;FOXL1;FOXL2;FOXM1;FOXN1;FOXN2;FOXN3;FOXN4;FOXO1;FOXO3;FOXO4;FOXO6;FOXP1;FOXP2;FOXP3;FOXP4;FOXQ1;FOXR1;FOXR2;FOXS1	FOXO1;FOXO3;FOXO4;FOXO6
GMEB2_HUMAN.H11MO.0.D	34	0.13333333	0.7732674	1.649.361	11.452.570	0.43831844	0.7221015	GMEB{5.3.3}	GMEB{5.3.3.1}	GMEB1;GMEB2	GMEB2
ANDR_HUMAN.H11MO.0.A	35	0.13725490	0.7765289	1.640.393	11.439.416	0.44394768	0.7251772	Steroid hormone receptors (NR3){2.1.1}	GR-like receptors (NR3C){2.1.1.1}	AR;ESR1;ESR2;ESRRA;ESRRB;ESRRG;NR3C1;NR3C2;PGR	AR;NR3C1;NR3C2;PGR
ZBTB6_HUMAN.H11MO.0.C	70	0.27450980	0.8556615	1.513.963	11.436.428	0.34589445	0.6520406	More than 3 adjacent zinc finger factors{2.3.3}	ZBTB6-like factors{2.3.3.11}	BCL6;BCL6B;CTCF;CTCFL;FEZF1;FEZF2;GFI1;GFI1B;GLI1;GLI2;GLI3;GLI4;GLIS1;GLIS2;GLIS3;HKR1;MTF1;MYNN;MZF1;OSR2;OVOL1;OVOL2;PLAG1;PLAGL1;PLAGL2;PRDM1;PRDM14;PRDM6;SCRT1;SCRT2;SNAI1;SNAI2;SNAI3;WT1;YY1;YY2;ZBTB12;ZBTB14;ZBTB18;ZBTB20;ZBTB26;ZBTB42;ZBTB45;ZBTB47;ZBTB48;ZBTB49;ZBTB6;ZBTB7A;ZBTB7B;ZBTB7C;ZFP14;ZFP2;ZFP28;ZFP30;ZFP37;ZFP42;ZFP64;ZFP69;ZFP69B;ZFP82;ZFP91;ZFX;ZIC1;ZIC2;ZIC3;ZIC4;ZIC5;ZIK1;ZIM3;ZKSCAN1;ZKSCAN2;ZKSCAN3;ZKSCAN4;ZNF121;ZNF124;ZNF133;ZNF136;ZNF138;ZNF14;ZNF140;ZNF143;ZNF146;ZNF148;ZNF155;ZNF157;ZNF160;ZNF169;ZNF175;ZNF177;ZNF18;ZNF180;ZNF181;ZNF2;ZNF20;ZNF212;ZNF213;ZNF214;ZNF221;ZNF222;ZNF223;ZNF224;ZNF225;ZNF226;ZNF227;ZNF229;ZNF230;ZNF232;ZNF233;ZNF234;ZNF235;ZNF24;ZNF25;ZNF250;ZNF257;ZNF26;ZNF260;ZNF263;ZNF264;ZNF268;ZNF274;ZNF276;ZNF28;ZNF280A;ZNF280B;ZNF280C;ZNF280D;ZNF281;ZNF282;ZNF283;ZNF284;ZNF285;ZNF286A;ZNF286B;ZNF3;ZNF30;ZNF300;ZNF302;ZNF317;ZNF32;ZNF320;ZNF322;ZNF324;ZNF324B;ZNF329;ZNF331;ZNF333;ZNF33A;ZNF33B;ZNF343;ZNF345;ZNF347;ZNF350;ZNF354A;ZNF354B;ZNF362;ZNF366;ZNF383;ZNF384;ZNF394;ZNF397;ZNF398;ZNF404;ZNF41;ZNF410;ZNF419;ZNF420;ZNF431;Z	ZBTB6

Motif	Nº of probes	% of probes	lower OR	upper OR	OR	p.value	FDR	TF family	TF subfamily	TF.family.member	TF.subfamily.member
										NF432;ZNF436;ZNF439;ZNF44;ZNF440;ZNF442;ZNF443;ZNF446;ZNF449;ZNF45;ZNF460;ZNF468;ZNF479;ZNF484;ZNF490;ZNF500;ZNF502;ZNF524;ZNF525;ZNF528;ZNF543;ZNF544;ZNF546;ZNF547;ZNF548;ZNF549;ZNF554;ZNF555;ZNF557;ZNF558;ZNF559;ZNF561;ZNF562;ZNF563;ZNF564;ZNF566;ZNF567;ZNF568;ZNF57;ZNF570;ZNF571;ZNF572;ZNF577;ZNF581;ZNF582;ZNF583;ZNF585A;ZNF586;ZNF589;ZNF595;ZNF599;ZNF600;ZNF605;ZNF607;ZNF611;ZNF613;ZNF614;ZNF615;ZNF616;ZNF619;ZNF620;ZNF621;ZNF625;ZNF627;ZNF649;ZNF652;ZNF653;ZNF665;ZNF667;ZNF669;ZNF670;ZNF672;ZNF679;ZNF680;ZNF683;ZNF689;ZNF692;ZNF701;ZNF705D;ZNF705E;ZNF705G;ZNF708;ZNF709;ZNF71;ZNF710;ZNF713;ZNF721;ZNF727;ZNF729;ZNF736;ZNF75A;ZNF75D;ZNF76;ZNF763;ZNF764;ZNF765;ZNF768;ZNF77;ZNF771;ZNF773;ZNF774;ZNF776;ZNF777;ZNF780A;ZNF780B;ZNF782;ZNF785;ZNF799;ZNF805;ZNF808;ZNF81;ZNF813;ZNF816;ZNF823;ZNF829;ZNF836;ZNF841;ZNF844;ZNF845;ZNF846;ZNF85;ZNF853;ZNF860;ZNF878;ZNF891;ZNF99;ZSCAN16;ZSCAN2;ZSCAN22;ZSCAN23;ZSCAN29;ZSCAN31;ZSCAN32;ZSCAN4;ZSCAN5A;ZSCAN5B;ZSCAN5C;ZSCAN9;ZXDA;ZXDB;ZXDC	
P63_HUMAN.H11MO.1.A	41	0.16078431	0.7974621	1.603.607	11.432.572	0.42129175	0.7076600	p53-related factors{6.3.1}	p63{6.3.1.0.2}	TP53;TP63;TP73	TP63
ERR1_HUMAN.H11MO.0.A	31	0.12156863	0.7582746	1.669.877	11.431.081	0.47919701	0.7509368	Steroid hormone receptors (NR3){2.1.1}	ER-like receptors (NR3A&B){2.1.1.2}	AR;ESR1;ESR2;ESRRA;ESRRB;ESRRG;NR3C1;NR3C2;PGR	ESRRA;ESRRB;ESRRG;ESR1;ESR2
ITF2_HUMAN.H11MO.0.C	82	0.32156863	0.8676252	1.495.343	11.429.559	0.33502917	0.6520406	E2A-related factors{1.2.1}	SEF2 (E2-2, TCF-4, ITF-2){1.2.1.0.2}	TCF12;TCF4;TCF3	TCF4
IRF2_HUMAN.H11MO.0.A	43	0.16862745	0.8024570	1.591.569	11.417.593	0.43035530	0.7146267	Interferon-regulatory factors{3.5.3}	IRF-2{3.5.3.0.2}	IRF1;IRF2;IRF3;IRF4;IRF5;IRF6;IRF7;IRF8;IRF9	IRF2
ZN563_HUMAN.H11MO.1.C	85	0.33333333	0.8677881	1.487.948	11.398.943	0.34050342	0.6520406	More than 3 adjacent zinc finger factors{2.3.3}	ZNF763-like factors{2.3.3.33}	BCL6;BCL6B;CTCF;CTCFL;FEZF1;FEZF2;GFI1;GFI1B;GLI1;GLI2;GLI3;GLI4;GLIS1;GLIS2;GLIS3;HKR1;MTF1;MYNN;MZF1;OSR2;OVOL1;OVOL2;PLAG1;PLAGL1;PLAGL2;PRDM1;PRDM14;PRDM6;SCRT1;SCRT2;SNAI1;SNAI2;SNAI3;WT1;YY1;YY2;ZBTB12;ZBTB14;ZBTB18;ZBTB20;ZBTB26;ZBTB42;ZBTB45;ZBTB47;ZBTB48;ZBTB49;ZBTB6;ZBTB7A;ZBTB7B;ZBTB7C;ZFP14;ZFP2;ZFP28;ZFP30;ZFP37;ZFP42;ZFP64;ZFP69;ZFP69B;ZFP82;ZFP91;ZFX;ZIC1;ZIC2;ZIC3;ZIC4;ZIC5;ZIK1;ZIM3;ZKSCAN1;ZKSCAN2;ZKSCAN3;ZKSCAN4;ZNF121;ZNF124;ZNF133;ZNF136;ZNF138;ZNF14;ZNF140;ZNF143;ZNF146;ZNF148;ZNF155;ZNF157;ZNF160;ZNF169;ZNF175;ZNF177;ZNF18;ZNF180;ZNF181;ZNF2;ZNF20;ZNF212;ZNF213;ZNF214;ZNF221;ZNF222;ZNF223;ZNF224;ZNF225;ZNF226;ZNF227;ZNF229;ZNF230;ZNF232;ZNF233;ZNF234;ZNF235;ZNF24;ZNF25;ZNF250;ZNF257;ZNF26;ZNF260;ZNF263;ZNF264;ZNF268;ZNF274;ZNF276;ZNF28;ZNF280A;ZNF280B;ZNF280C;ZNF280D;ZNF281;ZNF282;ZNF283;ZNF284;ZNF285;ZNF286A;ZNF286B;ZNF3;ZNF30;ZNF300;ZNF302;ZNF317;ZNF32;ZNF320;ZNF322;ZNF324;ZNF324B;ZNF329;ZNF331;ZNF333;ZNF33A;ZNF33B;ZNF343;ZNF345;ZNF347;ZNF350;ZNF354A;ZNF354B;ZNF362;ZNF366;ZNF383;ZNF384;ZNF394;ZNF397;ZNF398;ZNF404;ZNF41;ZNF410;ZNF419;ZNF420;ZNF431;ZNF432;ZNF436;ZNF439;ZNF44;ZNF440;ZNF442;ZNF443;ZNF446;ZNF449;ZNF45;ZNF460;ZNF468;ZNF479;ZNF484;ZNF490;ZNF500;ZNF502;ZNF524;ZNF525;ZNF528;ZNF543;ZNF544;ZNF546;ZNF547;ZNF548;ZNF549;ZNF554;ZNF555;ZNF557;ZNF558;ZNF559;ZNF561;ZNF562;ZNF563;ZNF564;ZNF566;ZNF567;ZNF568;ZNF57;ZNF570;ZNF571;ZNF572;ZNF577;ZNF581;ZNF582;ZNF583;ZNF585A;ZNF586;ZNF589;ZNF595;ZNF599;ZNF600;ZNF605;ZNF607;ZNF611;ZNF613;ZNF614;ZNF615;ZNF616;ZNF619;ZNF620;ZNF621;ZNF625;ZNF627;ZNF649;ZNF652;ZNF653;ZNF665;ZNF667;ZNF669;ZNF670;ZNF672;ZNF679;ZNF680;ZNF683;ZNF689;ZNF692;ZNF701;ZNF705D;Z	ZNF136;ZNF490;ZNF563

Motif	Nº of probes	% of probes	lower OR	upper OR	OR	p.value	FDR	TF family	TF subfamily	TF.family.member	TF.subfamily.member
										NF705E;ZNF705G;ZNF708;ZNF709;ZNF71;ZNF710;ZNF713;ZNF721;ZNF727;ZNF729;ZNF736;ZNF75A;ZNF75D;ZNF76;ZNF763;ZNF764;ZNF765;ZNF768;ZNF77;ZNF771;ZNF773;ZNF774;ZNF776;ZNF777;ZNF780A;ZNF780B;ZNF782;ZNF785;ZNF799;ZNF805;ZNF808;ZNF81;ZNF813;ZNF816;ZNF823;ZNF829;ZNF836;ZNF841;ZNF844;ZNF845;ZNF846;ZNF85;ZNF853;ZNF860;ZNF878;ZNF891;ZNF99;ZSCAN16;ZSCAN2;ZSCAN22;ZSCAN23;ZSCAN29;ZSCAN31;ZSCAN32;ZSCAN4;ZSCAN5A;ZSCAN5B;ZSCAN5C;ZSCAN9;ZXDA;ZXDB;ZXDC	
IRF3_HUMAN.H11MO.0.B	68	0.26666667	0.8501525	1.512.379	11.397.056	0.37960574	0.6838499	Interferon-regulatory factors{3.5.3}	IRF-3{3.5.3.0.3}	IRF1;IRF2;IRF3;IRF4;IRF5;IRF6;IRF7;IRF8;IRF9	IRF3
OLIG2_HUMAN.H11MO.0.B	49	0.19215686	0.8156595	1.561.463	11.382.918	0.40746660	0.7005007	Tal-related factors{1.2.3}	Neurogenin / Atonal-like factors{1.2.3.4}	ATOH1;ATOH7;ATOH8;BHLHA15;BHLHA9;BHLHE22;BHLHE23;FERD3L;FIGLA;HAND1;HAND2;LYL1;MESP1;MESP2;MSC;MSGN1;NEUROD1;NEUROD2;NEUROD4;NEUROD6;NEUROG1;NEUROG2;NEUROG3;NHLH1;NHLH2;OLIG1;OLIG2;OLIG3;PTF1A;SCX;TAL1;TAL2;TCF15;TCF21;TCF23;TWIST1;TWIST2	ATOH1;BHLHA15;BHLHE22;BHLHE23;NEUROD1;NEUROD2;NEUROG2;OLIG1;OLIG2;OLIG3
HSF4_HUMAN.H11MO.0.D	23	0.09019608	0.7051656	1.746.314	11.351.754	0.56319397	0.8192878	HSF factors{3.4.1}	HSF4 (HSTF4){3.4.1.0.3}	HSF1;HSF2;HSF4;HSF5;HSFX1;HSFY1;HSFY1; HSFY2	HSF4
STAT1_HUMAN.H11MO.0.A	26	0.10196078	0.7254456	1.707.358	11.349.001	0.51321755	0.7804551	STAT factors{6.2.1}	STAT1{6.2.1.0.1}	STAT1;STAT2;STAT3;STAT4;STAT5A;STAT5B;STAT6	STAT1
MAFA_HUMAN.H11MO.0.D	63	0.24705882	0.8389786	1.515.242	11.340.826	0.40837265	0.7005007	Maf-related factors{1.1.3}	Large Maf factors{1.1.3.1}	MAF;MAFA;MAFB;MAFF;MAFG;MAFK;NRL	MAFA;MAFB;MAF;NRL
HXA7_HUMAN.H11MO.0.D	9	0.03529412	0.5104563	2.183.608	11.304.496	0.71659927	0.9027746	HOX-related factors{3.1.1}	HOX6-7{3.1.1.6}	CDX1;CDX2;CDX4;EVX1;EVX2;GBX1;GBX2;GSX1;GSX2;HDX;HMBOX1;HNF1A;HNF1B;HOXA1;HOXA10;HOXA11;HOXA13;HOXA2;HOXA3;HOXA4;HOXA5;HOXA6;HOXA7;HOXA9;HOXB1;HOXB13;HOXB2;HOXB3;HOXB4;HOXB5;HOXB6;HOXB7;HOXB8;HOXB9;HOXC10;HOXC11;HOXC12;HOXC13;HOXC4;HOXC5;HOXC6;HOXC8;HOXC9;HOXD1;HOXD10;HOXD11;HOXD12;HOXD13;HOXD3;HOXD4;HOXD8;HOXD9;MEOX1;MEOX2;MNX1;PDX1;POU1F1;POU2F1;POU2F2;POU2F3;POU3F1;POU3F2;POU3F3;POU3F4;POU4F1;POU4F2;POU4F3;POU5F1;POU5F2;POU6F1;POU6F2	HOXA7;HOXB6;HOXB7;HOXC6
CEBPG_HUMAN.H11MO.0.B	16	0.06274510	0.6353086	1.875.006	11.302.529	0.58535082	0.8310155	C/EBP-related{1.1.8}	C/EBP{1.1.8.1}	CEBPA;CEBPB;CEBPD;CEBPE;CEBPG;DBP;DDIT3;HLF;NFIL3;TEF	CEBPA;CEBPB;CEBPD;CEBPE;CEBPG;DDIT3
ERR3_HUMAN.H11MO.0.B	18	0.07058824	0.6561578	1.821.414	11.269.598	0.60526747	0.8401621	Steroid hormone receptors (NR3){2.1.1}	ER-like receptors (NR3A&B){2.1.1.2}	AR;ESR1;ESR2;ESRRA;ESRRB;ESRRG;NR3C1;NR3C2;PGR	ESRRA;ESRRB;ESRRG;ESR1;ESR2
RHXF1_HUMAN.H11MO.0.D	15	0.05882353	0.6202358	1.896.593	11.262.741	0.67200327	0.8693197	Paired-related HD factors{3.1.3}	RHOX{3.1.3.23}	ALX1;ALX3;ALX4;ARGFX;ARX;CRX;DMBX1;DPRX;DRGX;DUX4;DUXA;ESX1;GSC;GSC2;HESX1;ISX;LEUTX;MIXL1;NOBOX;OTP;OTX1;OTX2;PHOX2A;PHOX2B;PITX1;PITX2;PITX3;PROP1;PRRX1;PRRX2;RAX;RAX2;RHOXF1;RHOXF2;SEBOX;SHOX;SHOX2;TPRX1;UNCX;VSX1;VSX2	RHOXF1
RXRA_HUMAN.H11MO.1.A	39	0.15294118	0.7768367	1.585.707	11.229.252	0.52484203	0.7918849	RXR-related receptors (NR2){2.1.3}	Retinoid X receptors (NR2B){2.1.3.1}	HNF4A;HNF4G;NR2C1;NR2C2;NR2E1;NR2E3;NR2F1;NR2F2;NR2F6;RXRA;RXRB;RXRG	RXRA;RXRB;RXRG
ESR1_HUMAN.H11MO.1.A	45	0.17647059	0.7936510	1.554.121	11.214.000	0.49419118	0.7635699	Steroid hormone receptors (NR3){2.1.1}	ER-like receptors (NR3A&B){2.1.1.2}	AR;ESR1;ESR2;ESRRA;ESRRB;ESRRG;NR3C1;NR3C2;PGR	ESRRA;ESRRB;ESRRG;ESR1;ESR2
MLXPL_HUMAN.H11MO.0.D	72	0.28235294	0.8396668	1.478.174	11.191.377	0.43192742	0.7146267	bHLH-ZIP factors{1.2.6}	Mondo-like factors{1.2.6.6}	MAX;MITF;MLX;MLXIP;MLXIPL;MNT;MXD1;MXD3;MXD4;MXI1;MYC;MYCL;MYCN;REPIN1;SREBF1;SREBF2;TFAP4;TFE3;TFEB;TFEC;USF1;USF2	MLXIPL;MLX
NR4A1_HUMAN.H11MO.0.A	22	0.08627451	0.6856695	1.731.336	11.159.411	0.63916924	0.8630464	NGFI-B-related receptors (NR4){2.1.4}	NGFI-B (NR4A1){2.1.4.0.1}	NR4A1;NR4A2;NR4A3	NR4A1
RFX2_HUMAN.H11MO.0.A	32	0.12549020	0.7425017	1.616.812	11.123.449	0.55445462	0.8173700	RFX-related factors{3.3.3}	RFX2{3.3.3.0.2}	RFX1;RFX2;RFX3;RFX4;RFX5;RFX6;RFX7;RFX8	RFX2

Motif	Nº of probes	% of probes	lower OR	upper OR	OR	p.value	FDR	TF family	TF subfamily	TF.family.member	TF.subfamily.member
RX_HUMAN.H11MO.0.D	11	0.04313725	0.5475844	2.027.323	11.122.138	0.74421411	0.9136769	Paired-related HD factors{3.1.3}	RAX{3.1.3.22}	ALX1;ALX3;ALX4;ARGFX;ARX;CRX;DMBX1;DPRX;DRGX;DUX4;DUXA;ESX1;GSC;GSC2;HESX1;ISX;LEUTX;MIXL1;NOBOX;OTP;OTX1;OTX2;PHOX2A;PHOX2B;PITX1;PITX2;PITX3;PROP1;PRRX1;PRRX2;RAX;RAX2;RHOXF1;RHOXF2;SEBOX;SHOX;SHOX2;TPRX1;UNCX;VSX1;VSX2	RAX2;RAX
INSM1_HUMAN.H11MO.0.C	49	0.19215686	0.7954198	1.522.719	11.100.419	0.51071021	0.7787856	Factors with multiple dispersed zinc fingers {2.3.4}	Insulinoma-associated proteins{2.3.4.16}	BCL11A;BCL11B;BNC1;BNC2;E4F1;HIC1;HIC2;HINFP;HIVEP1;HIVEP2;HIVEP3;IKZF1;IKZF2;IKZF3;IKZF4;IKZF5;INSM1;INSM2;MAZ;MECOM;PATZ1;PRDM16;PRDM4;REST;RLF;RREB1;SALL1;SALL2;SALL3;SALL4;VEZF1;ZBTB1;ZBTB17;ZBTB2;ZBTB25;ZBTB4;ZFAT;ZNF134;ZNF211;ZNF217;ZNF219;ZNF248;ZNF256;ZNF292;ZNF296;ZNF319;ZNF334;ZNF335;ZNF341;ZNF37A;ZNF382;ZNF417;ZNF418;ZNF423;ZNF467;ZNF510;ZNF512;ZNF512B;ZNF516;ZNF518A;ZNF518B;ZNF521;ZNF526;ZNF532;ZNF536;ZNF552;ZNF574;ZNF587;ZNF587B;ZNF592;ZNF639;ZNF654;ZNF658;ZNF671;ZNF687;ZNF711;ZNF717;ZNF770;ZNF772;ZNF784;ZNF786;ZNF792;ZNF8;ZNF814	INSM1
PRDM1_HUMAN.H11MO.0.A	37	0.14509804	0.7607918	1.578.570	11.096.946	0.57881512	0.8240120	More than 3 adjacent zinc finger factors{2.3.3}	PRDM1-like factors{2.3.3.12}	BCL6;BCL6B;CTCF;CTCFL;FEZF1;FEZF2;GFI1;GFI1B;GLI1;GLI2;GLI3;GLI4;GLIS1;GLIS2;GLIS3;HKR1;MTF1;MYNN;MZF1;OSR2;OVOL1;OVOL2;PLAG1;PLAGL1;PLAGL2;PRDM1;PRDM14;PRDM6;SCRT1;SCRT2;SNAI1;SNAI2;SNAI3;WT1;YY1;YY2;ZBTB12;ZBTB14;ZBTB18;ZBTB20;ZBTB26;ZBTB42;ZBTB45;ZBTB47;ZBTB48;ZBTB49;ZBTB6;ZBTB7A;ZBTB7B;ZBTB7C;ZFP14;ZFP2;ZFP28;ZFP30;ZFP37;ZFP42;ZFP64;ZFP69;ZFP69B;ZFP82;ZFP91;ZFX;ZIC1;ZIC2;ZIC3;ZIC4;ZIC5;ZIK1;ZIM3;ZKSCAN1;ZKSCAN2;ZKSCAN3;ZKSCAN4;ZNF121;ZNF124;ZNF133;ZNF136;ZNF138;ZNF14;ZNF140;ZNF143;ZNF146;ZNF148;ZNF155;ZNF157;ZNF160;ZNF169;ZNF175;ZNF177;ZNF18;ZNF180;ZNF181;ZNF2;ZNF20;ZNF212;ZNF213;ZNF214;ZNF221;ZNF222;ZNF223;ZNF224;ZNF225;ZNF226;ZNF227;ZNF229;ZNF230;ZNF232;ZNF233;ZNF234;ZNF235;ZNF24;ZNF25;ZNF250;ZNF257;ZNF26;ZNF260;ZNF263;ZNF264;ZNF268;ZNF274;ZNF276;ZNF28;ZNF280A;ZNF280B;ZNF280C;ZNF280D;ZNF281;ZNF282;ZNF283;ZNF284;ZNF285;ZNF286A;ZNF286B;ZNF3;ZNF30;ZNF300;ZNF302;ZNF317;ZNF32;ZNF320;ZNF322;ZNF324;ZNF324B;ZNF329;ZNF331;ZNF333;ZNF33A;ZNF33B;ZNF343;ZNF345;ZNF347;ZNF350;ZNF354A;ZNF354B;ZNF362;ZNF366;ZNF383;ZNF384;ZNF394;ZNF397;ZNF398;ZNF404;ZNF41;ZNF410;ZNF419;ZNF420;ZNF431;ZNF432;ZNF436;ZNF439;ZNF44;ZNF440;ZNF442;ZNF443;ZNF446;ZNF449;ZNF45;ZNF460;ZNF468;ZNF479;ZNF484;ZNF490;ZNF500;ZNF502;ZNF524;ZNF525;ZNF528;ZNF543;ZNF544;ZNF546;ZNF547;ZNF548;ZNF549;ZNF554;ZNF555;ZNF557;ZNF558;ZNF559;ZNF561;ZNF562;ZNF563;ZNF564;ZNF566;ZNF567;ZNF568;ZNF57;ZNF570;ZNF571;ZNF572;ZNF577;ZNF581;ZNF582;ZNF583;ZNF585A;ZNF586;ZNF589;ZNF595;ZNF599;ZNF600;ZNF605;ZNF607;ZNF611;ZNF613;ZNF614;ZNF615;ZNF616;ZNF619;ZNF620;ZNF621;ZNF625;ZNF627;ZNF649;ZNF652;ZNF653;ZNF665;ZNF667;ZNF669;ZNF670;ZNF672;ZNF679;ZNF680;ZNF683;ZNF689;ZNF692;ZNF701;ZNF705D;ZNF705E;ZNF705G;ZNF708;ZNF709;ZNF71;ZNF710;ZNF713;ZNF721;ZNF727;ZNF729;ZNF736;ZNF75A;ZNF75D;ZNF76;ZNF763;ZNF764;ZNF765;ZNF768;ZNF77;ZNF771;ZNF773;ZNF774;ZNF776;ZNF777;ZNF780A;ZNF780B;ZNF782;ZNF785;ZNF799;ZNF805;ZNF808;ZNF81;ZNF813;ZNF816;ZNF823;ZNF829;ZNF836;ZNF841;ZNF844;ZNF845;ZNF846;ZNF85;ZNF853;ZNF860;ZNF878;ZNF891;ZNF99;ZSCAN16;ZSCAN2;ZSCAN22;ZSCAN23;ZSCAN29;ZSCAN31;ZSCAN32;ZSCAN4;ZSCAN5A;ZSCAN5B;ZSCAN5C;ZSCAN9;ZXDA;ZXDB;ZXDC	PRDM1
LEF1_HUMAN.H11MO.0.A	28	0.10980392	0.7201811	1.646.625	11.085.841	0.60109009	0.8401621	TCF-7-related factors{4.1.3}	LEF-1 (TCF-1alpha) [1]{4.1.3.0.4}	LEF1;TAF1;TCF7;TCF7L1;TCF7L2	LEF1
Z324A_HUMAN.H11MO.0.C	55	0.21568627	0.8064583	1.500.630	11.080.843	0.48061965	0.7515619	More than 3 adjacent zinc finger factors{2.3.3}	ZNF324 factors{2.3.3.36}	BCL6;BCL6B;CTCF;CTCFL;FEZF1;FEZF2;GFI1;GFI1B;GLI1;GLI2;GLI3;GLI4;GLIS1;GLIS2;GLIS3;HKR1;MTF1;MYNN;MZF1;OSR2;OVOL1;OVOL2;PLAG1;PLAGL1;PLAGL2;PRDM1	ZNF324

Motif	Nº of probes	% of probes	lower OR	upper OR	OR	p.value	FDR	TF family	TF subfamily	TF.family.member	TF.subfamily.member
										;PRDM14;PRDM6;SCRT1;SCRT2;SNAI1;SNAI2;SNAI3;WT1;YY1;YY2;ZBTB12;ZBTB14;ZBTB18;ZBTB20;ZBTB26;ZBTB42;ZBTB45;ZBTB47;ZBTB48;ZBTB49;ZBTB6;ZBTB7A;ZBTB7B;ZBTB7C;ZFP14;ZFP2;ZFP28;ZFP30;ZFP37;ZFP42;ZFP64;ZFP69;ZFP69B;ZFP82;ZFP91;ZFX;ZIC1;ZIC2;ZIC3;ZIC4;ZIC5;ZIK1;ZIM3;ZKSCAN1;ZKSCAN2;ZKSCAN3;ZKSCAN4;ZNF121;ZNF124;ZNF133;ZNF136;ZNF138;ZNF14;ZNF140;ZNF143;ZNF146;ZNF148;ZNF155;ZNF157;ZNF160;ZNF169;ZNF175;ZNF177;ZNF18;ZNF180;ZNF181;ZNF2;ZNF20;ZNF212;ZNF213;ZNF214;ZNF221;ZNF222;ZNF223;ZNF224;ZNF225;ZNF226;ZNF227;ZNF229;ZNF230;ZNF232;ZNF233;ZNF234;ZNF235;ZNF24;ZNF25;ZNF250;ZNF257;ZNF26;ZNF260;ZNF263;ZNF264;ZNF268;ZNF274;ZNF276;ZNF28;ZNF280A;ZNF280B;ZNF280C;ZNF280D;ZNF281;ZNF282;ZNF283;ZNF284;ZNF285;ZNF286A;ZNF286B;ZNF3;ZNF30;ZNF300;ZNF302;ZNF317;ZNF32;ZNF320;ZNF322;ZNF324;ZNF324B;ZNF329;ZNF331;ZNF333;ZNF33A;ZNF33B;ZNF343;ZNF345;ZNF347;ZNF350;ZNF354A;ZNF354B;ZNF362;ZNF366;ZNF383;ZNF384;ZNF394;ZNF397;ZNF398;ZNF404;ZNF41;ZNF410;ZNF419;ZNF420;ZNF431;ZNF432;ZNF436;ZNF439;ZNF44;ZNF440;ZNF442;ZNF443;ZNF446;ZNF449;ZNF45;ZNF460;ZNF468;ZNF479;ZNF484;ZNF490;ZNF500;ZNF502;ZNF524;ZNF525;ZNF528;ZNF543;ZNF544;ZNF546;ZNF547;ZNF548;ZNF549;ZNF554;ZNF555;ZNF557;ZNF558;ZNF559;ZNF561;ZNF562;ZNF563;ZNF564;ZNF566;ZNF567;ZNF568;ZNF57;ZNF570;ZNF571;ZNF572;ZNF577;ZNF581;ZNF582;ZNF583;ZNF585A;ZNF586;ZNF589;ZNF595;ZNF599;ZNF600;ZNF605;ZNF607;ZNF611;ZNF613;ZNF614;ZNF615;ZNF616;ZNF619;ZNF620;ZNF621;ZNF625;ZNF627;ZNF649;ZNF652;ZNF653;ZNF665;ZNF667;ZNF669;ZNF670;ZNF672;ZNF679;ZNF680;ZNF683;ZNF689;ZNF692;ZNF701;ZNF705D;ZNF705E;ZNF705G;ZNF708;ZNF709;ZNF71;ZNF710;ZNF713;ZNF721;ZNF727;ZNF729;ZNF736;ZNF75A;ZNF75D;ZNF76;ZNF763;ZNF764;ZNF765;ZNF768;ZNF77;ZNF771;ZNF773;ZNF774;ZNF776;ZNF777;ZNF780A;ZNF780B;ZNF782;ZNF785;ZNF799;ZNF805;ZNF808;ZNF81;ZNF813;ZNF816;ZNF823;ZNF829;ZNF836;ZNF841;ZNF844;ZNF845;ZNF846;ZNF85;ZNF853;ZNF860;ZNF878;ZNF891;ZNF99;ZSCAN16;ZSCAN2;ZSCAN22;ZSCAN23;ZSCAN29;ZSCAN31;ZSCAN32;ZSCAN4;ZSCAN5A;ZSCAN5B;ZSCAN5C;ZSCAN9;ZXDA;ZXDB;ZXDC	
GATA3_HUMAN.H11MO.0.A	24	0.09411765	0.6953923	1.691.181	11.080.797	0.57693482	0.8240120	GATA-type zinc fingers{2.2.1}	Two zinc-finger GATA factors {2.2.1.1}	GATA1;GATA2;GATA3;GATA4;GATA5;GATA6;GATAD2A;GATAD2B;TRPS1;ZGLP1	GATA1;GATA2;GATA3;GATA4;GATA5;GATA6
SMAD2_HUMAN.H11MO.0.A	56	0.21960784	0.8080130	1.497.235	11.076.835	0.48349507	0.7515619	SMAD factors{7.1.1}	Regulatory Smads (R-Smad){7.1.1.1}	SMAD1;SMAD2;SMAD3;SMAD4	SMAD1;SMAD2;SMAD3
PTF1A_HUMAN.H11MO.0.B	81	0.31764706	0.8396052	1.449.731	11.071.673	0.45050988	0.7307745	Tal-related factors{1.2.3}	Twist-like factors{1.2.3.2}	ATOH1;ATOH7;ATOH8;BHLHA15;BHLHA9;BHLHE22;BHLHE23;FERD3L;FIGLA;HAND1;HAND2;LYL1;MESP1;MESP2;MSC;MSGN1;NEUROD1;NEUROD2;NEUROD4;NEUROD6;NEUROG1;NEUROG2;NEUROG3;NHLH1;NHLH2;OLIG1;OLIG2;OLIG3;PTF1A;SCX;TAL1;TAL2;TCF15;TCF21;TCF23;TWIST1;TWIST2	FIGLA;HAND1;PTF1A;TWIST1
PITX3_HUMAN.H11MO.0.D	12	0.04705882	0.5634480	1.969.745	11.062.992	0.64340030	0.8631094	Paired-related HD factors{3.1.3}	PITX{3.1.3.19}	ALX1;ALX3;ALX4;ARGFX;ARX;CRX;DMBX1;DPRX;DRGX;DUX4;DUXA;ESX1;GSC;GSC2;HESX1;ISX;LEUTX;MIXL1;NOBOX;OTP;OTX1;OTX2;PHOX2A;PHOX2B;PITX1;PITX2;PITX3;PROP1;PRRX1;PRRX2;RAX;RAX2;RHOXF1;RHOXF2;SEBOX;SHOX;SHOX2;TPRX1;UNCX;VSX1;VSX2	PITX1;PITX2;PITX3
ZN134_HUMAN.H11MO.1.C	28	0.10980392	0.7152343	1.635.262	11.009.323	0.60273405	0.8401621	Factors with multiple dispersed zinc fingers {2.3.4}	ZNF134-like factors{2.3.4.24}	BCL11A;BCL11B;BNC1;BNC2;E4F1;HIC1;HIC2;HINFP;HIVEP1;HIVEP2;HIVEP3;IKZF1;IKZF2;IKZF3;IKZF4;IKZF5;INSM1;INSM2;MAZ;MECOM;PATZ1;PRDM16;PRDM4;REST;RLF;RREB1;SALL1;SALL2;SALL3;SALL4;VEZF1;ZBTB1;ZBTB17;ZBTB2;ZBTB25;ZBTB4;ZFAT;ZNF134;ZNF211;ZNF217;ZNF219;ZNF248;ZNF256;ZNF292;ZNF296;ZNF319;ZNF334;ZNF335;ZNF341;ZNF37A;ZNF382;ZNF417;ZNF418;ZNF423;ZNF4	ZNF134

Motif	Nº of probes	% of probes	lower OR	upper OR	OR	p.value	FDR	TF family	TF subfamily	TF.family.member	TF.subfamily.member
										67;ZNF510;ZNF512;ZNF512B;ZNF516;ZNF518A;ZNF518B;ZNF521;ZNF526;ZNF532;ZNF536;ZNF552;ZNF574;ZNF587;ZNF587B;ZNF592;ZNF639;ZNF654;ZNF658;ZNF671;ZNF687;ZNF711;ZNF717;ZNF770;ZNF772;ZNF784;ZNF786;ZNF792;ZNF8;ZNF814	
PPARA_HUMAN.H11MO.1.B	28	0.10980392	0.7151126	1.634.983	11.007.445	0.60277950	0.8401621	Thyroid hormone receptor-related factors (NR1){2.1.2}	PPAR (NR1C){2.1.2.5}	NR1D1;NR1D2;NR1H2;NR1H3;NR1H4;NR1I2;NR1I3;PPARA;PPARD;PPARG;RARA;RARB;RARG;RORA;RORB;RORC;THRA;THRB;VDR	PPARA;PPARD;PPARG
IRF7_HUMAN.H11MO.0.C	26	0.10196078	0.7035080	1.655.683	11.005.538	0.59154848	0.8322699	Interferon-regulatory factors{3.5.3}	IRF-7{3.5.3.0.7}	IRF1;IRF2;IRF3;IRF4;IRF5;IRF6;IRF7;IRF8;IRF9	IRF7
EGR3_HUMAN.H11MO.0.D	50	0.19607843	0.7904462	1.505.341	10.999.387	0.56895800	0.8230143	Three-zinc finger Krüppel-related factors{2.3.1}	EGR factors{2.3.1.3}	EGR1;EGR2;EGR3;EGR4;KLF1;KLF10;KLF11;KLF12;KLF13;KLF14;KLF15;KLF16;KLF17;KLF2;KLF3;KLF4;KLF5;KLF6;KLF7;KLF8;KLF9;SP1;SP2;SP3;SP4;SP5;SP6;SP7;SP8;SP9	EGR1;EGR2;EGR3;EGR4
SRY_HUMAN.H11MO.0.B	27	0.10588235	0.7063559	1.637.831	10.958.554	0.67194320	0.8693197	SOX-related factors{4.1.1}	Group A{4.1.1.1}	BBX;CIC;HBP1;SOX1;SOX10;SOX11;SOX12;SOX13;SOX14;SOX15;SOX17;SOX18;SOX2;SOX21;SOX3;SOX30;SOX4;SOX5;SOX6;SOX7;SOX8;SOX9;SRY	SRY
RFX3_HUMAN.H11MO.0.B	20	0.07843137	0.6564949	1.731.376	10.950.960	0.62924931	0.8537110	RFX-related factors{3.3.3}	RFX3{3.3.3.0.3}	RFX1;RFX2;RFX3;RFX4;RFX5;RFX6;RFX7;RFX8	RFX3
BHE41_HUMAN.H11MO.0.D	71	0.27843137	0.8160055	1.440.117	10.891.243	0.56829210	0.8230143	Hairy-related factors{1.2.4}	Hairy-like factors{1.2.4.1}	BHLHE40;BHLHE41;HELT;HES1;HES2;HES3;HES4;HES5;HES6;HES7;HEY1;HEY2;HEYL	BHLHE40;BHLHE41;HES1;HES5;HES7;HEY1;HEY2
TEAD4_HUMAN.H11MO.0.A	23	0.09019608	0.6765129	1.675.282	10.890.001	0.65070171	0.8634820	TEF-1-related factors{3.6.1}	TEF-3 (TEAD-4, TCF-13L1){3.6.1.0.2}	TEAD1;TEAD2;TEAD3;TEAD4	TEAD4
TAL1_HUMAN.H11MO.1.A	53	0.20784314	0.7885703	1.480.280	10.886.939	0.57926654	0.8240120	Tal-related factors{1.2.3}	Tal / HEN-like factors{1.2.3.1}	ATOH1;ATOH7;ATOH8;BHLHA15;BHLHA9;BHLHE22;BHLHE23;FERD3L;FIGLA;HAND1;HAND2;LYL1;MESP1;MESP2;MSC;MSGN1;NEUROD1;NEUROD2;NEUROD4;NEUROD6;NEUROG1;NEUROG2;NEUROG3;NHLH1;NHLH2;OLIG1;OLIG2;OLIG3;PTF1A;SCX;TAL1;TAL2;TCF15;TCF21;TCF23;TWIST1;TWIST2	NHLH1;LYL1;TAL1
COT2_HUMAN.H11MO.0.A	34	0.13333333	0.7317751	1.560.818	10.838.119	0.63508922	0.8590417	RXR-related receptors (NR2){2.1.3}	COUP-like receptors (NR2F){2.1.3.5}	HNF4A;HNF4G;NR2C1;NR2C2;NR2E1;NR2E3;NR2F1;NR2F2;NR2F6;RXRA;RXRB;RXRG	NR2F1;NR2F2;NR2F6
IRF9_HUMAN.H11MO.0.C	22	0.08627451	0.6652075	1.679.611	10.825.995	0.72851655	0.9074091	Interferon-regulatory factors{3.5.3}	IRF-9 (ISGF-3gamma){3.5.3.0.9}	IRF1;IRF2;IRF3;IRF4;IRF5;IRF6;IRF7;IRF8;IRF9	IRF9
ARNT_HUMAN.H11MO.0.B	77	0.30196078	0.8172756	1.422.366	10.824.742	0.57883753	0.8240120	PAS domain factors{1.2.5}	Arnt-like factors{1.2.5.2}	AHR;AHRR;ARNT;ARNT2;ARNTL;ARNTL2;CLOCK;EPAS1;HIF1A;HIF3A;NCOA1;NCOA2;NCOA3;NPAS1;NPAS2;NPAS3;NPAS4;SIM1;SIM2;SOHLH1;SOHLH2;TCFL5	ARNT2;ARNT;ARNTL;CLOCK
IRF5_HUMAN.H11MO.0.D	36	0.14117647	0.7378252	1.544.388	10.815.046	0.64344589	0.8631094	Interferon-regulatory factors{3.5.3}	IRF-5{3.5.3.0.5}	IRF1;IRF2;IRF3;IRF4;IRF5;IRF6;IRF7;IRF8;IRF9	IRF5
LYL1_HUMAN.H11MO.0.A	63	0.24705882	0.8000851	1.445.020	10.814.937	0.60362063	0.8401621	Tal-related factors{1.2.3}	Tal / HEN-like factors{1.2.3.1}	ATOH1;ATOH7;ATOH8;BHLHA15;BHLHA9;BHLHE22;BHLHE23;FERD3L;FIGLA;HAND1;HAND2;LYL1;MESP1;MESP2;MSC;MSGN1;NEUROD1;NEUROD2;NEUROD4;NEUROD6;NEUROG1;NEUROG2;NEUROG3;NHLH1;NHLH2;OLIG1;OLIG2;OLIG3;PTF1A;SCX;TAL1;TAL2;TCF15;TCF21;TCF23;TWIST1;TWIST2	NHLH1;LYL1;TAL1
FOXB1_HUMAN.H11MO.0.D	21	0.08235294	0.6547967	1.688.271	10.783.309	0.72350763	0.9074091	Forkhead box (FOX) factors{3.3.1}	FOXB{3.3.1.2}	FOXA1;FOXA2;FOXA3;FOXB1;FOXB2;FOXC1;FOXC2;FOXD1;FOXD2;FOXD3;FOXD4;FOXD4L1;FOXD4L3;FOXD4L4;FOXD4L5;FOXD4L6;FOXE1;FOXE3;FOXF1;FOXF2;FOXG1;FOXH1;FOXI1;FOXI2;FOXI3;FOXJ1;FOXJ2;FOXJ3;FOXK1;FOXK2;FOXL1;FOXL2;FOXM1;FOXN1;FOXN2;FOXN3;FOXN4;FOXO1;FOXO3;FOXO4;FOXO6;FOXP1;FOXP2;FOXP3;FOXP4;FOXQ1;FOXR1;FOXR2;FOXS1	FOXB1
HIC2_HUMAN.H11MO.0.D	41	0.16078431	0.7492559	1.506.559	10.741.878	0.66199962	0.8665564	Factors with multiple dispersed zinc fingers{2.3.4}	Hypermethylated in Cancer proteins{2.3.4.17}	BCL11A;BCL11B;BNC1;BNC2;E4F1;HIC1;HIC2;HINFP;HIVEP1;HIVEP2;HIVEP3;IKZF1;IKZF2;IKZF3;IKZF4;IKZF5;INSM1;INSM2;MAZ;MECOM;PATZ1;PRDM16;PRDM4;REST;RLF;RREB1;SALL1;SALL2;SALL3;SALL4;VEZF1;ZBTB1;ZBTB17;ZBTB2;ZBTB25;ZBTB4;ZFAT;ZNF134;ZNF211;ZNF217;ZNF219;ZNF248;ZNF256;ZNF292;ZNF296;ZNF319;ZNF334;ZNF	HIC1;HIC2

Motif	Nº of probes	% of probes	lower OR	upper OR	OR	p.value	FDR	TF family	TF subfamily	TF.family.member	TF.subfamily.member
										335;ZNF341;ZNF37A;ZNF382;ZNF417;ZNF418;ZNF423;ZNF467;ZNF510;ZNF512;ZNF512B;ZNF516;ZNF518A;ZNF518B;ZNF521;ZNF526;ZNF532;ZNF536;ZNF552;ZNF574;ZNF587;ZNF587B;ZNF592;ZNF639;ZNF654;ZNF658;ZNF671;ZNF687;ZNF711;ZNF717;ZNF770;ZNF772;ZNF784;ZNF786;ZNF792;ZNF8;ZNF814	
TEF_HUMAN.H11MO.0.D	18	0.07058824	0.6245938	1.733.730	10.727.715	0.70589711	0.8951426	C/EBP-related{1.1.8}	PAR factors{1.1.8.2}	CEBPA;CEBPB;CEBPD;CEBPE;CEBPG;DBP;DDIT3;HLF;NFIL3;TEF	DBP;HLF;NFIL3;TEF
GRHL1_HUMAN.H11MO.0.D	23	0.09019608	0.6658823	1.648.925	10.719.378	0.73510653	0.9126685	Grainyhead-related factors{6.7.1}	GRH-like proteins{6.7.1.1}	GRHL1;GRHL2;GRHL3	GRHL1;GRHL2
SOX3_HUMAN.H11MO.0.B	27	0.10588235	0.6901126	1.600.126	10.706.810	0.75304933	0.9179869	SOX-related factors{4.1.1}	Group B{4.1.1.2}	BBX;CIC;HBP1;SOX1;SOX10;SOX11;SOX12;SOX13;SOX14;SOX15;SOX17;SOX18;SOX2;SOX21;SOX3;SOX30;SOX4;SOX5;SOX6;SOX7;SOX8;SOX9;SRY	SOX1;SOX21;SOX2;SOX3
USF2_HUMAN.H11MO.0.A	72	0.28235294	0.8028648	1.413.337	10.701.070	0.62108928	0.8490423	bHLH-ZIP factors{1.2.6}	USF factors{1.2.6.2}	MAX;MITF;MLX;MLXIP;MLXIPL;MNT;MXD1;MXD3;MXD4;MXI1;MYC;MYCL;MYCN;REPIN1;SREBF1;SREBF2;TFAP4;TFE3;TFEB;TFEC;USF1;USF2	USF1;USF2
EMX2_HUMAN.H11MO.0.D	12	0.04705882	0.5447160	1.904.087	10.694.715	0.75984661	0.9193105	NK-related factors{3.1.2}	EMX{3.1.2.6}	BARHL1;BARHL2;BARX1;BARX2;BSX;DBX1;DBX2;DLX1;DLX2;DLX3;DLX4;DLX5;DLX6;EMX1;EMX2;EN1;EN2;HHEX;HLX;HMX1;HMX2;HMX3;LBX1;LBX2;MSX1;MSX2;NANOG;NKX1-1;NKX1-2;NKX2-1;NKX2-2;NKX2-3;NKX2-4;NKX2-5;NKX2-6;NKX2-8;NKX3-1;NKX3-2;NKX6-1;NKX6-2;NKX6-3;NOTO;TLX1;TLX2;TLX3;VAX1;VAX2;VENTX	EMX1;EMX2
MYNN_HUMAN.H11MO.0.D	15	0.05882353	0.5888239	1.800.318	10.691.399	0.78297023	0.9272965	More than 3 adjacent zinc finger factors{2.3.3}	unclassified{2.3.3.0}	BCL6B;BCL6;CTCFL;CTCF;FEZF1;GFI1B;GFI1;GLI1;GLI2;GLI3;GLIS1;GLIS2;GLIS3;MTF1;MYNN;MZF1;OSR2;OVOL1;OVOL2;ZNF146;PLAG1;PLAGL1;PRDM14;PRDM1;PRDM6;SCRT1;SCRT2;SNAI1;SNAI2;YY1;YY2;WT1;ZNF324;ZNF354A;ZBTB14;ZBTB18;ZBTB48;ZBTB49;ZBTB7A;ZBTB7B;ZBTB6;ZFP64;ZFP28;ZFP42;ZFP82;ZFX;ZIC1;ZIC2;ZIC3;ZIC4;ZIM3;ZKSCAN1;ZKSCAN3;ZNF121;ZNF136;ZNF140;ZNF143;ZNF148;ZNF214;ZNF232;ZNF250;ZNF257;ZNF260;ZNF263;ZNF264;ZNF274;ZNF281;ZNF282;ZNF317;ZNF320;ZNF322;ZNF329;ZNF331;ZNF333;ZNF350;ZNF384;ZNF394;ZNF410;ZNF436;ZNF449;ZNF490;ZNF502;ZNF524;ZNF528;ZNF547;ZNF549;ZNF554;ZNF563;ZNF582;ZNF586;ZNF589;ZNF652;ZNF667;ZNF680;ZNF708;ZNF713;ZNF768;ZNF816;ZNF18;ZNF41;ZNF76;ZNF85;ZSCAN16;ZSCAN22;ZSCAN31;ZSCAN4	MYNN;MZF1;OSR2;PRDM14;PRDM6;WT1;ZBTB14;ZBTB48;ZBTB49;ZFP64;ZFP28;ZIM3;ZNF121;ZNF250;ZNF257;ZNF263;ZNF274;ZNF317;ZNF320;ZNF329;ZNF331;ZNF394;ZNF449;ZNF502;ZNF528;ZNF547;ZNF549;ZNF554;ZNF586;ZNF589;ZNF667;ZNF680;ZNF708;ZNF713;ZNF768;ZNF18;ZNF85;ZSCAN16;ZSCAN22
MTF1_HUMAN.H11MO.0.C	57	0.22352941	0.7812901	1.441.930	10.686.718	0.64593271	0.8631094	More than 3 adjacent zinc finger factors{2.3.3}	MTF1-like factors{2.3.3.24}	BCL6;BCL6B;CTCF;CTCFL;FEZF1;FEZF2;GFI1;GFI1B;GLI1;GLI2;GLI3;GLI4;GLIS1;GLIS2;GLIS3;HKR1;MTF1;MYNN;MZF1;OSR2;OVOL1;OVOL2;PLAG1;PLAGL1;PLAGL2;PRDM1;PRDM14;PRDM6;SCRT1;SCRT2;SNAI1;SNAI2;SNAI3;WT1;YY1;YY2;ZBTB12;ZBTB14;ZBTB18;ZBTB20;ZBTB26;ZBTB42;ZBTB45;ZBTB47;ZBTB48;ZBTB49;ZBTB6;ZBTB7A;ZBTB7B;ZBTB7C;ZFP14;ZFP2;ZFP28;ZFP30;ZFP37;ZFP42;ZFP64;ZFP69;ZFP69B;ZFP82;ZFP91;ZFX;ZIC1;ZIC2;ZIC3;ZIC4;ZIC5;ZIK1;ZIM3;ZKSCAN1;ZKSCAN2;ZKSCAN3;ZKSCAN4;ZNF121;ZNF124;ZNF133;ZNF136;ZNF138;ZNF14;ZNF140;ZNF143;ZNF146;ZNF148;ZNF155;ZNF157;ZNF160;ZNF169;ZNF175;ZNF177;ZNF18;ZNF180;ZNF181;ZNF2;ZNF20;ZNF212;ZNF213;ZNF214;ZNF221;ZNF222;ZNF223;ZNF224;ZNF225;ZNF226;ZNF227;ZNF229;ZNF230;ZNF232;ZNF233;ZNF234;ZNF235;ZNF24;ZNF25;ZNF250;ZNF257;ZNF26;ZNF260;ZNF263;ZNF264;ZNF268;ZNF274;ZNF276;ZNF28;ZNF280A;ZNF280B;ZNF280C;ZNF280D;ZNF281;ZNF282;ZNF283;ZNF284;ZNF285;ZNF286A;ZNF286B;ZNF3;ZNF30;ZNF300;ZNF302;ZNF317;ZNF32;ZNF320;ZNF322;ZNF324;ZNF324B;ZNF329;ZNF331;ZNF333;ZNF33A;ZNF33B;ZNF343;ZNF345;ZNF347;ZNF350;ZNF354A;ZNF354B;ZNF362;ZNF366;ZNF383;ZNF384;ZNF394;ZNF397;ZNF398;ZNF404;ZNF41;ZNF410;ZNF419;ZNF420;ZNF431;ZNF432;ZNF436;ZNF439;ZNF44;ZNF440;ZNF442;ZNF443;ZNF446;ZNF449;ZNF45;ZNF460;ZNF468;ZNF479;ZNF484;ZNF490;ZNF500;ZNF502;ZNF524;ZNF525;ZNF528;ZNF543;ZNF544;ZNF546;ZNF547;ZNF548;ZNF549;ZNF554;ZNF555;ZNF557;Z	MTF1;ZNF410

Motif	Nº of probes	% of probes	lower OR	upper OR	OR	p.value	FDR	TF family	TF subfamily	TF.family.member	TF.subfamily.member
										NF558;ZNF559;ZNF561;ZNF562;ZNF563;ZNF564;ZNF566;ZNF567;ZNF568;ZNF57;ZNF570;ZNF571;ZNF572;ZNF577;ZNF581;ZNF582;ZNF583;ZNF585A;ZNF586;ZNF589;ZNF595;ZNF599;ZNF600;ZNF605;ZNF607;ZNF611;ZNF613;ZNF614;ZNF615;ZNF616;ZNF619;ZNF620;ZNF621;ZNF625;ZNF627;ZNF649;ZNF652;ZNF653;ZNF665;ZNF667;ZNF669;ZNF670;ZNF672;ZNF679;ZNF680;ZNF683;ZNF689;ZNF692;ZNF701;ZNF705D;ZNF705E;ZNF705G;ZNF708;ZNF709;ZNF71;ZNF710;ZNF713;ZNF721;ZNF727;ZNF729;ZNF736;ZNF75A;ZNF75D;ZNF76;ZNF763;ZNF764;ZNF765;ZNF768;ZNF77;ZNF771;ZNF773;ZNF774;ZNF776;ZNF777;ZNF780A;ZNF780B;ZNF782;ZNF785;ZNF799;ZNF805;ZNF808;ZNF81;ZNF813;ZNF816;ZNF823;ZNF829;ZNF836;ZNF841;ZNF844;ZNF845;ZNF846;ZNF85;ZNF853;ZNF860;ZNF878;ZNF891;ZNF99;ZSCAN16;ZSCAN2;ZSCAN22;ZSCAN23;ZSCAN29;ZSCAN31;ZSCAN32;ZSCAN4;ZSCAN5A;ZSCAN5B;ZSCAN5C;ZSCAN9;ZXDA;ZXDB;ZXDC	
SOX18_HUMAN.H11MO.0.D	17	0.06666667	0.6115130	1.747.659	10.679.938	0.79519191	0.9358929	SOX-related factors{4.1.1}	Group F{4.1.1.6}	BBX;CIC;HBP1;SOX1;SOX10;SOX11;SOX12;SOX13;SOX14;SOX15;SOX17;SOX18;SOX2;SOX21;SOX3;SOX30;SOX4;SOX5;SOX6;SOX7;SOX8;SOX9;SRY	SOX17;SOX18;SOX7
STAT1_HUMAN.H11MO.1.A	53	0.20784314	0.7725948	1.450.331	10.666.524	0.69369052	0.8854891	STAT factors{6.2.1}	STAT1{6.2.1.0.1}	STAT1;STAT2;STAT3;STAT4;STAT5A;STAT5B;STAT6	STAT1
MYCN_HUMAN.H11MO.0.A	78	0.30588235	0.8062248	1.400.224	10.666.143	0.62999976	0.8537110	bHLH-ZIP factors{1.2.6}	Myc / Max factors{1.2.6.5}	MAX;MITF;MLX;MLXIP;MLXIPL;MNT;MXD1;MXD3;MXD4;MXI1;MYC;MYCL;MYCN;REPIN1;SREBF1;SREBF2;TFAP4;TFE3;TFEB;TFEC;USF1;USF2	MAX;MYCN;MYC
FOXJ3_HUMAN.H11MO.1.B	37	0.14509804	0.7309094	1.516.469	10.661.548	0.71551588	0.9027746	Forkhead box (FOX) factors{3.3.1}	FOXJ{3.3.1.10}	FOXA1;FOXA2;FOXA3;FOXB1;FOXB2;FOXC1;FOXC2;FOXD1;FOXD2;FOXD3;FOXD4;FOXD4L1;FOXD4L3;FOXD4L4;FOXD4L5;FOXD4L6;FOXE1;FOXE3;FOXF1;FOXF2;FOXG1;FOXH1;FOXI1;FOXI2;FOXI3;FOXJ1;FOXJ2;FOXJ3;FOXK1;FOXK2;FOXL1;FOXL2;FOXM1;FOXN1;FOXN2;FOXN3;FOXN4;FOXO1;FOXO3;FOXO4;FOXO6;FOXP1;FOXP2;FOXP3;FOXP4;FOXQ1;FOXR1;FOXR2;FOXS1	FOXJ2;FOXJ3
MYBA_HUMAN.H11MO.0.D	33	0.12941176	0.7143855	1.539.146	10.639.849	0.70270088	0.8925575	Myb/SANT domain factors{3.5.1}	Myb-like factors{3.5.1.1}	CDC5L;DMTF1;MYB;MYBL1;MYBL2;SMARCA1;SMARCA5;SNAPC4;TRERF1;ZNF541	CDC5L;MYBL1;MYBL2;MYB
TWST1_HUMAN.H11MO.0.A	19	0.07450980	0.6289492	1.699.849	10.639.210	0.80573759	0.9426763	Tal-related factors{1.2.3}	Twist-like factors{1.2.3.2}	ATOH1;ATOH7;ATOH8;BHLHA15;BHLHA9;BHLHE22;BHLHE23;FERD3L;FIGLA;HAND1;HAND2;LYL1;MESP1;MESP2;MSC;MSGN1;NEUROD1;NEUROD2;NEUROD4;NEUROD6;NEUROG1;NEUROG2;NEUROG3;NHLH1;NHLH2;OLIG1;OLIG2;OLIG3;PTF1A;SCX;TAL1;TAL2;TCF15;TCF21;TCF23;TWIST1;TWIST2	FIGLA;HAND1;PTF1A;TWIST1
NFAT5_HUMAN.H11MO.0.D	45	0.17647059	0.7525655	1.473.595	10.633.317	0.67558759	0.8711016	NFAT-related factors{6.1.3}	NFAT5 (TonEBP){6.1.3.0.5}	NFAT5;NFATC1;NFATC2;NFATC3;NFATC4	NFAT5
ATF4_HUMAN.H11MO.0.A	14	0.05490196	0.5715102	1.817.928	10.618.251	0.77708439	0.9260156	ATF-4-related factors{1.1.6}	ATF-4{1.1.6.0.1}	ATF4;ATF5	ATF4
ESR1_HUMAN.H11MO.0.A	46	0.18039216	0.7522613	1.464.258	10.594.099	0.73958615	0.9126911	Steroid hormone receptors (NR3){2.1.1}	ER-like receptors (NR3A&B){2.1.1.2}	AR;ESR1;ESR2;ESRRA;ESRRB;ESRRG;NR3C1;NR3C2;PGR	ESRRA;ESRRB;ESRRG;ESR1;ESR2
SOX17_HUMAN.H11MO.0.C	17	0.06666667	0.6056214	1.730.809	10.576.950	0.79629795	0.9358929	SOX-related factors{4.1.1}	Group F{4.1.1.6}	BBX;CIC;HBP1;SOX1;SOX10;SOX11;SOX12;SOX13;SOX14;SOX15;SOX17;SOX18;SOX2;SOX21;SOX3;SOX30;SOX4;SOX5;SOX6;SOX7;SOX8;SOX9;SRY	SOX17;SOX18;SOX7
HSFY1_HUMAN.H11MO.0.D	13	0.05098039	0.5543975	1.843.153	10.569.630	0.77040698	0.9225697	HSF factors{3.4.1}	HSFY1 (HSFY2, HSF2L){3.4.1.0.6}	HSF1;HSF2;HSF4;HSFY1; HSFY2	HSFY1; HSFY2
VSX1_HUMAN.H11MO.0.D	13	0.05098039	0.5536551	1.840.677	10.555.452	0.77066394	0.9225697	Paired-related HD factors{3.1.3}	VSX{3.1.3.28}	ALX1;ALX3;ALX4;ARGFX;ARX;CRX;DMBX1;DPRX;DRGX;DUX4;DUXA;ESX1;GSC;GSC2;HESX1;ISX;LEUTX;MIXL1;NOBOX;OTP;OTX1;OTX2;PHOX2A;PHOX2B;PITX1;PITX2;PITX3;PROP1;PRRX1;PRRX2;RAX;RAX2;RHOXF1;RHOXF2;SEBOX;SHOX;SHOX2;TPRX1;UNCX;VSX1;VSX2	VSX1;VSX2
SOX2_HUMAN.H11MO.1.A	20	0.07843137	0.6319973	1.666.729	10.542.691	0.81106764	0.9446120	SOX-related factors{4.1.1}	Group B{4.1.1.2}	BBX;CIC;HBP1;SOX1;SOX10;SOX11;SOX12;SOX13;SOX14;SOX15;SOX17;SOX18;SOX2;SOX21;SOX3;SOX30;SOX4;SOX5;SOX6;SOX7;SOX8;SOX9;SRY	SOX1;SOX21;SOX2;SOX3

Motif	Nº of probes	% of probes	lower OR	upper OR	OR	p.value	FDR	TF family	TF subfamily	TF.family.member	TF.subfamily.member
HXB8_HUMAN.H11MO.0.C	10	0.03921569	0.4974797	1.967.939	10.511.556	0.86783397	0.9683105	HOX-related factors{3.1.1}	HOX8{3.1.1.7}	CDX1;CDX2;CDX4;EVX1;EVX2;GBX1;GBX2;GSX1;GSX2;HDX;HMBOX1;HNF1A;HNF1B;HOXA1;HOXA10;HOXA11;HOXA13;HOXA2;HOXA3;HOXA4;HOXA5;HOXA6;HOXA7;HOXA9;HOXB1;HOXB13;HOXB2;HOXB3;HOXB4;HOXB5;HOXB6;HOXB7;HOXB8;HOXB9;HOXC10;HOXC11;HOXC12;HOXC13;HOXC4;HOXC5;HOXC6;HOXC8;HOXC9;HOXD1;HOXD10;HOXD11;HOXD12;HOXD13;HOXD3;HOXD4;HOXD8;HOXD9;MEOX1;MEOX2;MNX1;PDX1;POU1F1;POU2F1;POU2F2;POU2F3;POU3F1;POU3F2;POU3F3;POU3F4;POU4F1;POU4F2;POU4F3;POU5F1;POU5F2;POU6F1;POU6F2	HOXB8;HOXC8;HOXD8
RARG_HUMAN.H11MO.2.D	46	0.18039216	0.7458721	1.451.804	10.503.966	0.74073465	0.9126911	Thyroid hormone receptor-related factors (NR1){2.1.2}	Retinoic acid receptors (NR1B){2.1.2.1}	NR1D1;NR1D2;NR1H2;NR1H3;NR1H4;NR1I2;NR1I3;PPARA;PPARD;PPARG;RARA;RARB;RARG;RORA;RORB;RORC;THRA;THRB;VDR	RARA;RARB;RARG
PDX1_HUMAN.H11MO.1.A	12	0.04705882	0.5338803	1.866.219	10.482.099	0.87911298	0.9683105	HOX-related factors{3.1.1}	PDX{3.1.1.15}	CDX1;CDX2;CDX4;EVX1;EVX2;GBX1;GBX2;GSX1;GSX2;HDX;HMBOX1;HNF1A;HNF1B;HOXA1;HOXA10;HOXA11;HOXA13;HOXA2;HOXA3;HOXA4;HOXA5;HOXA6;HOXA7;HOXA9;HOXB1;HOXB13;HOXB2;HOXB3;HOXB4;HOXB5;HOXB6;HOXB7;HOXB8;HOXB9;HOXC10;HOXC11;HOXC12;HOXC13;HOXC4;HOXC5;HOXC6;HOXC8;HOXC9;HOXD1;HOXD10;HOXD11;HOXD12;HOXD13;HOXD3;HOXD4;HOXD8;HOXD9;MEOX1;MEOX2;MNX1;PDX1;POU1F1;POU2F1;POU2F2;POU2F3;POU3F1;POU3F2;POU3F3;POU3F4;POU4F1;POU4F2;POU4F3;POU5F1;POU5F2;POU6F1;POU6F2	PDX1
SMAD3_HUMAN.H11MO.0.B	87	0.34117647	0.7952173	1.359.171	10.427.164	0.73978535	0.9126911	SMAD factors{7.1.1}	Regulatory Smads (R-Smad){7.1.1.1}	SMAD1;SMAD2;SMAD3;SMAD4;SMAD5;SMAD9	SMAD1;SMAD2;SMAD3
HXA5_HUMAN.H11MO.0.D	10	0.03921569	0.4921663	1.947.031	10.399.844	0.86865333	0.9683105	HOX-related factors{3.1.1}	HOX5{3.1.1.5}	CDX1;CDX2;CDX4;EVX1;EVX2;GBX1;GBX2;GSX1;GSX2;HDX;HMBOX1;HNF1A;HNF1B;HOXA1;HOXA10;HOXA11;HOXA13;HOXA2;HOXA3;HOXA4;HOXA5;HOXA6;HOXA7;HOXA9;HOXB1;HOXB13;HOXB2;HOXB3;HOXB4;HOXB5;HOXB6;HOXB7;HOXB8;HOXB9;HOXC10;HOXC11;HOXC12;HOXC13;HOXC4;HOXC5;HOXC6;HOXC8;HOXC9;HOXD1;HOXD10;HOXD11;HOXD12;HOXD13;HOXD3;HOXD4;HOXD8;HOXD9;MEOX1;MEOX2;MNX1;PDX1;POU1F1;POU2F1;POU2F2;POU2F3;POU3F1;POU3F2;POU3F3;POU3F4;POU4F1;POU4F2;POU4F3;POU5F1;POU5F2;POU6F1;POU6F2	HOXA5
ELK1_HUMAN.H11MO.0.B	44	0.17254902	0.7313497	1.441.062	10.368.888	0.80141599	0.9390452	Ets-related factors{3.5.2}	Elk-like factors{3.5.2.2}	EHF;ELF1;ELF2;ELF3;ELF4;ELF5;ELK1;ELK3;ELK4;ERF;ERG;ETS1;ETS2;ETV1;ETV2;ETV3;ETV3L;ETV4;ETV5;ETV6;ETV7;FEV;FLI1;GABPA;SPDEF;SPI1;SPIB;SPIC	ELK1;ELK3;ELK4;ETV1;ETV4;ETV5
ZN418_HUMAN.H11MO.1.D	19	0.07450980	0.6127923	1.656.181	10.365.630	0.80903139	0.9443208	Factors with multiple dispersed zinc fingers{2.3.4}	ZNF417-like factors{2.3.4.1}	BCL11A;BCL11B;BNC1;BNC2;E4F1;HIC1;HIC2;HINFP;HIVEP1;HIVEP2;HIVEP3;IKZF1;IKZF2;IKZF3;IKZF4;IKZF5;INSM1;INSM2;MAZ;MECOM;PATZ1;PRDM16;PRDM4;REST;RLF;RREB1;SALL1;SALL2;SALL3;SALL4;VEZF1;ZBTB1;ZBTB17;ZBTB2;ZBTB25;ZBTB4;ZFAT;ZNF134;ZNF211;ZNF217;ZNF219;ZNF248;ZNF256;ZNF292;ZNF296;ZNF319;ZNF334;ZNF335;ZNF341;ZNF37A;ZNF382;ZNF417;ZNF418;ZNF423;ZNF467;ZNF510;ZNF512;ZNF512B;ZNF516;ZNF518A;ZNF518B;ZNF521;ZNF526;ZNF532;ZNF536;ZNF552;ZNF574;ZNF587;ZNF587B;ZNF592;ZNF639;ZNF654;ZNF658;ZNF671;ZNF687;ZNF711;ZNF717;ZNF770;ZNF772;ZNF784;ZNF786;ZNF792;ZNF8;ZNF814	ZNF418
ZFP42_HUMAN.H11MO.0.A	24	0.09411765	0.6502623	1.581.276	10.361.262	0.82765327	0.9547862	More than 3 adjacent zinc finger factors{2.3.3}	YY1-like factors{2.3.3.9}	BCL6;BCL6B;CTCF;CTCFL;FEZF1;FEZF2;GFI1;GFI1B;GLI1;GLI2;GLI3;GLI4;GLIS1;GLIS2;GLIS3;HKR1;MTF1;MYNN;MZF1;OSR2;OVOL1;OVOL2;PLAG1;PLAGL1;PLAGL2;PRDM1;PRDM14;PRDM6;SCRT1;SCRT2;SNAI1;SNAI2;SNAI3;WT1;YY1;YY2;ZBTB12;ZBTB14;ZBTB18;ZBTB20;ZBTB26;ZBTB42;ZBTB45;ZBTB47;ZBTB48;ZBTB49;ZBTB6;ZBTB7A;ZBTB7B;ZBTB7C;ZFP14;ZFP2;ZFP28;ZFP30;ZFP37;ZFP42;ZFP64;ZFP69;ZFP69B;ZFP82;ZFP91;ZFX;ZIC1;ZIC2;ZIC3;ZIC4;ZIC5;ZIK1;ZIM3;ZKSCAN1;ZKSCAN2;ZKSCAN3;ZKSCAN4;ZNF121;ZNF124;ZNF133;ZNF136;ZNF138;ZNF14;ZNF140;ZNF143;ZNF146;ZNF148;ZNF155;ZNF157;ZNF160;ZNF169;ZNF175;ZNF177;ZNF18;ZNF180;ZNF181;ZNF2;ZNF20;ZNF212;ZNF21	YY1;YY2;ZFP42

Motif	Nº of probes	% of probes	lower OR	upper OR	OR	p.value	FDR	TF family	TF subfamily	TF.family.member	TF.subfamily.member
										3;ZNF214;ZNF221;ZNF222;ZNF223;ZNF224;ZNF225;ZNF226;ZNF227;ZNF229;ZNF230;ZNF232;ZNF233;ZNF234;ZNF235;ZNF24;ZNF25;ZNF250;ZNF257;ZNF26;ZNF260;ZNF263;ZNF264;ZNF268;ZNF274;ZNF276;ZNF28;ZNF280A;ZNF280B;ZNF280C;ZNF280D;ZNF281;ZNF282;ZNF283;ZNF284;ZNF285;ZNF286A;ZNF286B;ZNF3;ZNF30;ZNF300;ZNF302;ZNF317;ZNF32;ZNF320;ZNF322;ZNF324;ZNF324B;ZNF329;ZNF331;ZNF333;ZNF33A;ZNF33B;ZNF343;ZNF345;ZNF347;ZNF350;ZNF354A;ZNF354B;ZNF362;ZNF366;ZNF383;ZNF384;ZNF394;ZNF397;ZNF398;ZNF404;ZNF41;ZNF410;ZNF419;ZNF420;ZNF431;ZNF432;ZNF436;ZNF439;ZNF44;ZNF440;ZNF442;ZNF443;ZNF446;ZNF449;ZNF45;ZNF460;ZNF468;ZNF479;ZNF484;ZNF490;ZNF500;ZNF502;ZNF524;ZNF525;ZNF528;ZNF543;ZNF544;ZNF546;ZNF547;ZNF548;ZNF549;ZNF554;ZNF555;ZNF557;ZNF558;ZNF559;ZNF561;ZNF562;ZNF563;ZNF564;ZNF566;ZNF567;ZNF568;ZNF57;ZNF570;ZNF571;ZNF572;ZNF577;ZNF581;ZNF582;ZNF583;ZNF585A;ZNF586;ZNF589;ZNF595;ZNF599;ZNF600;ZNF605;ZNF607;ZNF611;ZNF613;ZNF614;ZNF615;ZNF616;ZNF619;ZNF620;ZNF621;ZNF625;ZNF627;ZNF649;ZNF652;ZNF653;ZNF665;ZNF667;ZNF669;ZNF670;ZNF672;ZNF679;ZNF680;ZNF683;ZNF689;ZNF692;ZNF701;ZNF705D;ZNF705E;ZNF705G;ZNF708;ZNF709;ZNF71;ZNF710;ZNF713;ZNF721;ZNF727;ZNF729;ZNF736;ZNF75A;ZNF75D;ZNF76;ZNF763;ZNF764;ZNF765;ZNF768;ZNF77;ZNF771;ZNF773;ZNF774;ZNF776;ZNF777;ZNF780A;ZNF780B;ZNF782;ZNF785;ZNF799;ZNF805;ZNF808;ZNF81;ZNF813;ZNF816;ZNF823;ZNF829;ZNF836;ZNF841;ZNF844;ZNF845;ZNF846;ZNF85;ZNF853;ZNF860;ZNF878;ZNF891;ZNF99;ZSCAN16;ZSCAN2;ZSCAN22;ZSCAN23;ZSCAN29;ZSCAN31;ZSCAN32;ZSCAN4;ZSCAN5A;ZSCAN5B;ZSCAN5C;ZSCAN9;ZXDA;ZXDB;ZXDC	
ZN547_HUMAN.H11MO.0.C	29	0.11372549	0.6780842	1.529.896	10.361.042	0.84122551	0.9608665	More than 3 adjacent zinc finger factors{2.3.3}	unclassified{2.3.3.0}	BCL6B;BCL6;CTCFL;CTCF;FEZF1;GFI1B;GFI1;GLI1;GLI2;GLI3;GLIS1;GLIS2;GLIS3;MTF1;MYNN;MZF1;OSR2;OVOL1;OVOL2;ZNF146;PLAG1;PLAGL1;PRDM14;PRDM1;PRDM6;SCRT1;SCRT2;SNAI1;SNAI2;YY1;YY2;WT1;ZNF324;ZNF354A;ZBTB14;ZBTB18;ZBTB48;ZBTB49;ZBTB7A;ZBTB7B;ZBTB6;ZFP64;ZFP28;ZFP42;ZFP82;ZFX;ZIC1;ZIC2;ZIC3;ZIC4;ZIM3;ZKSCAN1;ZKSCAN3;ZNF121;ZNF136;ZNF140;ZNF143;ZNF148;ZNF214;ZNF232;ZNF250;ZNF257;ZNF260;ZNF263;ZNF264;ZNF274;ZNF281;ZNF282;ZNF317;ZNF320;ZNF322;ZNF329;ZNF331;ZNF333;ZNF350;ZNF384;ZNF394;ZNF410;ZNF436;ZNF449;ZNF490;ZNF502;ZNF524;ZNF528;ZNF547;ZNF549;ZNF554;ZNF563;ZNF582;ZNF586;ZNF589;ZNF652;ZNF667;ZNF680;ZNF708;ZNF713;ZNF768;ZNF816;ZNF18;ZNF41;ZNF76;ZNF85;ZSCAN16;ZSCAN22;ZSCAN31;ZSCAN4	MYNN;MZF1;OSR2;PRDM14;PRDM6;WT1;ZBTB14;ZBTB48;ZBTB49;ZFP64;ZFP28;ZIM3;ZNF121;ZNF250;ZNF257;ZNF263;ZNF274;ZNF317;ZNF320;ZNF329;ZNF331;ZNF394;ZNF449;ZNF502;ZNF528;ZNF547;ZNF549;ZNF554;ZNF586;ZNF589;ZNF667;ZNF680;ZNF708;ZNF713;ZNF768;ZNF18;ZNF85;ZSCAN16;ZSCAN22
HXA13_HUMAN.H11MO.0.C	13	0.05098039	0.5430165	1.805.389	10.352.181	0.88440192	0.9683105	HOX-related factors{3.1.1}	HOX9-13{3.1.1.8}	CDX1;CDX2;CDX4;EVX1;EVX2;GBX1;GBX2;GSX1;GSX2;HDX;HMBOX1;HNF1A;HNF1B;HOXA1;HOXA10;HOXA11;HOXA13;HOXA2;HOXA3;HOXA4;HOXA5;HOXA6;HOXA7;HOXA9;HOXB1;HOXB13;HOXB2;HOXB3;HOXB4;HOXB5;HOXB6;HOXB7;HOXB8;HOXB9;HOXC10;HOXC11;HOXC12;HOXC13;HOXC4;HOXC5;HOXC6;HOXC8;HOXC9;HOXD1;HOXD10;HOXD11;HOXD12;HOXD13;HOXD3;HOXD4;HOXD8;HOXD9;MEOX1;MEOX2;MNX1;PDX1;POU1F1;POU2F1;POU2F2;POU2F3;POU3F1;POU3F2;POU3F3;POU3F4;POU4F1;POU4F2;POU4F3;POU5F1;POU5F2;POU6F1;POU6F2	HOXA10;HOXA11;HOXA13;HOXA9;HOXB13;HOXC10;HOXC11;HOXC12;HOXC13;HOXC9;HOXD10;HOXD11;HOXD12;HOXD13;HOXD9
HXB7_HUMAN.H11MO.0.C	11	0.04313725	0.5095607	1.886.281	10.349.453	0.87487949	0.9683105	HOX-related factors{3.1.1}	HOX6-7{3.1.1.6}	CDX1;CDX2;CDX4;EVX1;EVX2;GBX1;GBX2;GSX1;GSX2;HDX;HMBOX1;HNF1A;HNF1B;HOXA1;HOXA10;HOXA11;HOXA13;HOXA2;HOXA3;HOXA4;HOXA5;HOXA6;HOXA7;HOXA9;HOXB1;HOXB13;HOXB2;HOXB3;HOXB4;HOXB5;HOXB6;HOXB7;HOXB8;HOXB9;HOXC10;HOXC11;HOXC12;HOXC13;HOXC4;HOXC5;HOXC6;HOXC8;HOXC9;HOXD1;HOXD10;HOXD11;HOXD12;HOXD13;HOXD3;HOXD4;HOXD8;HOXD9;MEOX1;MEOX2;MNX1;PDX1;POU1F1;POU2F1;POU2F2;POU2F3;POU3F1;POU3F2;POU3F3;POU3F4;POU4F1;POU4F2;POU4F3;POU5F1;POU5F2;POU6F1;POU6F2	HOXA7;HOXB6;HOXB7;HOXC6

Motif	Nº of probes	% of probes	lower OR	upper OR	OR	p.value	FDR	TF family	TF subfamily	TF.family.member	TF.subfamily.member
TEAD2_HUMAN.H11MO.0.D	25	0.09803922	0.6553770	1.566.969	10.344.034	0.83089485	0.9550844	TEF-1-related factors{3.6.1}	TEF-4 (TEAD-2){3.6.1.0.3}	TEAD1;TEAD2;TEAD3;TEAD4	TEAD2
HXC6_HUMAN.H11MO.0.D	18	0.07058824	0.6012369	1.668.856	10.326.132	0.90092909	0.9728520	HOX-related factors{3.1.1}	HOX6-7{3.1.1.6}	CDX1;CDX2;CDX4;EVX1;EVX2;GBX1;GBX2;GSX1;GSX2;HDX;HMBOX1;HNF1A;HNF1B;HOXA1;HOXA10;HOXA11;HOXA13;HOXA2;HOXA3;HOXA4;HOXA5;HOXA6;HOXA7;HOXA9;HOXB1;HOXB13;HOXB2;HOXB3;HOXB4;HOXB5;HOXB6;HOXB7;HOXB8;HOXB9;HOXC10;HOXC11;HOXC12;HOXC13;HOXC4;HOXC5;HOXC6;HOXC8;HOXC9;HOXD1;HOXD10;HOXD11;HOXD12;HOXD13;HOXD3;HOXD4;HOXD8;HOXD9;MEOX1;MEOX2;MNX1;PDX1;POU1F1;POU2F1;POU2F2;POU2F3;POU3F1;POU3F2;POU3F3;POU3F4;POU4F1;POU4F2;POU4F3;POU5F1;POU5F2;POU6F1;POU6F2	HOXA7;HOXB6;HOXB7;HOXC6
MAFF_HUMAN.H11MO.1.B	33	0.12941176	0.6906537	1.487.983	10.286.003	0.85040456	0.9680833	Maf-related factors{1.1.3}	Small Maf factors{1.1.3.2}	MAF;MAFA;MAFB;MAFF;MAFG;MAFK;NRL	MAFF;MAFG;MAFK
ZNF41_HUMAN.H11MO.0.C	34	0.13333333	0.6935476	1.479.217	10.271.553	0.85240288	0.9680833	More than 3 adjacent zinc finger factors{2.3.3}	ZFN81-like factors{2.3.3.68}	BCL6;BCL6B;CTCF;CTCFL;FEZF1;FEZF2;GFI1;GFI1B;GLI1;GLI2;GLI3;GLI4;GLIS1;GLIS2;GLIS3;HKR1;MTF1;MYNN;MZF1;OSR2;OVOL1;OVOL2;PLAG1;PLAGL1;PLAGL2;PRDM1;PRDM14;PRDM6;SCRT1;SCRT2;SNAI1;SNAI2;SNAI3;WT1;YY1;YY2;ZBTB12;ZBTB14;ZBTB18;ZBTB20;ZBTB26;ZBTB42;ZBTB45;ZBTB47;ZBTB48;ZBTB49;ZBTB6;ZBTB7A;ZBTB7B;ZBTB7C;ZFP14;ZFP2;ZFP28;ZFP30;ZFP37;ZFP42;ZFP64;ZFP69;ZFP69B;ZFP82;ZFP91;ZFX;ZIC1;ZIC2;ZIC3;ZIC4;ZIC5;ZIK1;ZIM3;ZKSCAN1;ZKSCAN2;ZKSCAN3;ZKSCAN4;ZNF121;ZNF124;ZNF133;ZNF136;ZNF138;ZNF14;ZNF140;ZNF143;ZNF146;ZNF148;ZNF155;ZNF157;ZNF160;ZNF169;ZNF175;ZNF177;ZNF18;ZNF180;ZNF181;ZNF2;ZNF20;ZNF212;ZNF213;ZNF214;ZNF221;ZNF222;ZNF223;ZNF224;ZNF225;ZNF226;ZNF227;ZNF229;ZNF230;ZNF232;ZNF233;ZNF234;ZNF235;ZNF24;ZNF25;ZNF250;ZNF257;ZNF26;ZNF260;ZNF263;ZNF264;ZNF268;ZNF274;ZNF276;ZNF28;ZNF280A;ZNF280B;ZNF280C;ZNF280D;ZNF281;ZNF282;ZNF283;ZNF284;ZNF285;ZNF286A;ZNF286B;ZNF3;ZNF30;ZNF300;ZNF302;ZNF317;ZNF32;ZNF320;ZNF322;ZNF324;ZNF324B;ZNF329;ZNF331;ZNF333;ZNF33A;ZNF33B;ZNF343;ZNF345;ZNF347;ZNF350;ZNF354A;ZNF354B;ZNF362;ZNF366;ZNF383;ZNF384;ZNF394;ZNF397;ZNF398;ZNF404;ZNF41;ZNF410;ZNF419;ZNF420;ZNF431;ZNF432;ZNF436;ZNF439;ZNF44;ZNF440;ZNF442;ZNF443;ZNF446;ZNF449;ZNF45;ZNF460;ZNF468;ZNF479;ZNF484;ZNF490;ZNF500;ZNF502;ZNF524;ZNF525;ZNF528;ZNF543;ZNF544;ZNF546;ZNF547;ZNF548;ZNF549;ZNF554;ZNF555;ZNF557;ZNF558;ZNF559;ZNF561;ZNF562;ZNF563;ZNF564;ZNF566;ZNF567;ZNF568;ZNF57;ZNF570;ZNF571;ZNF572;ZNF577;ZNF581;ZNF582;ZNF583;ZNF585A;ZNF586;ZNF589;ZNF595;ZNF599;ZNF600;ZNF605;ZNF607;ZNF611;ZNF613;ZNF614;ZNF615;ZNF616;ZNF619;ZNF620;ZNF621;ZNF625;ZNF627;ZNF649;ZNF652;ZNF653;ZNF665;ZNF667;ZNF669;ZNF670;ZNF672;ZNF679;ZNF680;ZNF683;ZNF689;ZNF692;ZNF701;ZNF705D;ZNF705E;ZNF705G;ZNF708;ZNF709;ZNF71;ZNF710;ZNF713;ZNF721;ZNF727;ZNF729;ZNF736;ZNF75A;ZNF75D;ZNF76;ZNF763;ZNF764;ZNF765;ZNF768;ZNF77;ZNF771;ZNF773;ZNF774;ZNF776;ZNF777;ZNF780A;ZNF780B;ZNF782;ZNF785;ZNF799;ZNF805;ZNF808;ZNF81;ZNF813;ZNF816;ZNF823;ZNF829;ZNF836;ZNF841;ZNF844;ZNF845;ZNF846;ZNF85;ZNF853;ZNF860;ZNF878;ZNF891;ZNF99;ZSCAN16;ZSCAN2;ZSCAN22;ZSCAN23;ZSCAN29;ZSCAN31;ZSCAN32;ZSCAN4;ZSCAN5A;ZSCAN5B;ZSCAN5C;ZSCAN9;ZXDA;ZXDB;ZXDC	ZNF41
KLF8_HUMAN.H11MO.0.C	62	0.24313725	0.7585032	1.374.379	10.270.935	0.82575171	0.9545046	Three-zinc finger Krüppel-related factors{2.3.1}	Krüppel-like factors{2.3.1.2}	EGR1;EGR2;EGR3;EGR4;KLF1;KLF10;KLF11;KLF12;KLF13;KLF14;KLF15;KLF16;KLF17;KLF2;KLF3;KLF4;KLF5;KLF6;KLF7;KLF8;KLF9;SP1;SP2;SP3;SP4;SP5;SP6;SP7;SP8;SP9	KLF12;KLF13;KLF14;KLF15;KLF16;KLF1;KLF3;KLF4;KLF5;KLF6;KLF8;KLF9
ZBTB4_HUMAN.H11MO.0.D	50	0.19607843	0.7379104	1.405.293	10.268.295	0.87351106	0.9683105	Factors with multiple dispersed zinc fingers {2.3.4}	unclassified{2.3.4.0}	BCL11A;E4F1;MECOM;HIC1;HIC2;HINFP;IKZF1;INSM1;MAZ;PATZ1;PRDM4;REST;RREB1;SALL4;VEZF1;ZBTB17;ZBT	E4F1;PRDM4;REST;RREB1;ZBTB17;ZBTB4;ZNF335;ZNF341;ZNF467;ZNF770;ZNF784;ZNF8

Motif	Nº of probes	% of probes	lower OR	upper OR	OR	p.value	FDR	TF family	TF subfamily	TF.family.member	TF.subfamily.member
										B4;HIVEP1;HIVEP2;ZNF134;ZNF219;ZNF335;ZNF341;ZNF382;ZNF418;ZNF423;ZNF467;ZNF770;ZNF784;ZNF8	
ETS2_HUMAN.H11MO.0.B	65	0.25490196	0.7622800	1.367.952	10.268.031	0.82847209	0.9547862	Ets-related factors{3.5.2}	Ets-like factors{3.5.2.1}	EHF;ELF1;ELF2;ELF3;ELF4;ELF5;ELK1;ELK3;ELK4;ERF;ERG;ETS1;ETS2;ETV1;ETV2;ETV3;ETV3L;ETV4;ETV5;ETV6;ETV7;FEV;FLI1;GABPA;SPDEF;SPI1;SPIB;SPIC	ERG;ETS1;ETS2;ETV2;ETV3;FEV;FLI1;GABPA
ERG_HUMAN.H11MO.0.A	70	0.27450980	0.7682318	1.359.261	10.267.892	0.83244711	0.9550844	Ets-related factors{3.5.2}	Ets-like factors{3.5.2.1}	EHF;ELF1;ELF2;ELF3;ELF4;ELF5;ELK1;ELK3;ELK4;ERF;ERG;ETS1;ETS2;ETV1;ETV2;ETV3;ETV3L;ETV4;ETV5;ETV6;ETV7;FEV;FLI1;GABPA;SPDEF;SPI1;SPIB;SPIC	ERG;ETS1;ETS2;ETV2;ETV3;FEV;FLI1;GABPA
BPTF_HUMAN.H11MO.0.D	34	0.13333333	0.6925096	1.477.006	10.256.169	0.85256621	0.9680833	NA	NA	BPTF	BPTF
RUNX3_HUMAN.H11MO.0.A	27	0.10588235	0.6601517	1.530.564	10.241.520	0.91794999	0.9808967	Runt-related factors{6.4.1}	Runx3 (PEBP2alphaC, CBF-alpha3, AML-2){6.4.1.0.3}	RUNX1;RUNX2;RUNX3	RUNX3
SMCA5_HUMAN.H11MO.0.C	25	0.09803922	0.6483405	1.550.047	10.232.775	0.91511707	0.9808967	Myb/SANT domain factors{3.5.1}	SMARCA-like factors{3.5.1.5}	CDC5L;MYBL1;MYBL2;MYB;SMARCA1;SMARCA5	SMARCA1;SMARCA5
ZN423_HUMAN.H11MO.0.D	41	0.16078431	0.7130813	1.433.873	10.223.399	0.86371052	0.9683105	Factors with multiple dispersed zinc fingers {2.3.4}	ZNF423-like factors{2.3.4.12}	BCL11A;BCL11B;BNC1;BNC2;E4F1;HIC1;HIC2;HINFP;HIVEP1;HIVEP2;HIVEP3;IKZF1;IKZF2;IKZF3;IKZF4;IKZF5;INSM1;INSM2;MAZ;MECOM;PATZ1;PRDM16;PRDM4;REST;RLF;RREB1;SALL1;SALL2;SALL3;SALL4;VEZF1;ZBTB1;ZBTB17;ZBTB2;ZBTB25;ZBTB4;ZFAT;ZNF134;ZNF211;ZNF217;ZNF219;ZNF248;ZNF256;ZNF292;ZNF296;ZNF319;ZNF334;ZNF335;ZNF341;ZNF37A;ZNF382;ZNF417;ZNF418;ZNF423;ZNF467;ZNF510;ZNF512;ZNF512B;ZNF516;ZNF518A;ZNF518B;ZNF521;ZNF526;ZNF532;ZNF536;ZNF552;ZNF574;ZNF587;ZNF587B;ZNF592;ZNF639;ZNF654;ZNF658;ZNF671;ZNF687;ZNF711;ZNF717;ZNF770;ZNF772;ZNF784;ZNF786;ZNF792;ZNF8;ZNF814	ZNF423
FOXP1_HUMAN.H11MO.0.A	29	0.11372549	0.6683245	1.507.895	10.211.991	0.92060019	0.9808967	Forkhead box (FOX) factors{3.3.1}	FOXP{3.3.1.16}	FOXA1;FOXA2;FOXA3;FOXB1;FOXB2;FOXC1;FOXC2;FOXD1;FOXD2;FOXD3;FOXD4;FOXD4L1;FOXD4L3;FOXD4L4;FOXD4L5;FOXD4L6;FOXE1;FOXE3;FOXF1;FOXF2;FOXG1;FOXH1;FOXI1;FOXI2;FOXI3;FOXJ1;FOXJ2;FOXJ3;FOXK1;FOXK2;FOXL1;FOXL2;FOXM1;FOXN1;FOXN2;FOXN3;FOXN4;FOXO1;FOXO3;FOXO4;FOXO6;FOXP1;FOXP2;FOXP3;FOXP4;FOXQ1;FOXR1;FOXR2;FOXS1	FOXP1;FOXP2;FOXP3
FOXK1_HUMAN.H11MO.0.A	28	0.10980392	0.6620562	1.513.622	10.190.987	0.91945037	0.9808967	Forkhead box (FOX) factors{3.3.1}	FOXK{3.3.1.11}	FOXA1;FOXA2;FOXA3;FOXB1;FOXB2;FOXC1;FOXC2;FOXD1;FOXD2;FOXD3;FOXD4;FOXD4L1;FOXD4L3;FOXD4L4;FOXD4L5;FOXD4L6;FOXE1;FOXE3;FOXF1;FOXF2;FOXG1;FOXH1;FOXI1;FOXI2;FOXI3;FOXJ1;FOXJ2;FOXJ3;FOXK1;FOXK2;FOXL1;FOXL2;FOXM1;FOXN1;FOXN2;FOXN3;FOXN4;FOXO1;FOXO3;FOXO4;FOXO6;FOXP1;FOXP2;FOXP3;FOXP4;FOXQ1;FOXR1;FOXR2;FOXS1	FOXK1
TFE2_HUMAN.H11MO.0.A	73	0.28627451	0.7640556	1.341.740	10.170.373	0.88948779	0.9683105	E2A-related factors{1.2.1}	E2A (TCF-3, ITF-1){1.2.1.0.1}	TCF12;TCF4;TCF3	TCF3
SOX9_HUMAN.H11MO.1.B	14	0.05490196	0.5455058	1.735.095	10.134.675	0.89000197	0.9683105	SOX-related factors{4.1.1}	Group E{4.1.1.5}	BBX;CIC;HBP1;SOX1;SOX10;SOX11;SOX12;SOX13;SOX14;SOX15;SOX17;SOX18;SOX2;SOX21;SOX3;SOX30;SOX4;SOX5;SOX6;SOX7;SOX8;SOX9;SRY	SOX10;SOX8;SOX9
BATF3_HUMAN.H11MO.0.B	17	0.06666667	0.5797487	1.656.787	10.124.291	0.89955468	0.9727302	B-ATF-related factors{1.1.4}	B-ATF-3{1.1.4.0.3}	BATF;BATF2;BATF3	BATF3
PEBB_HUMAN.H11MO.0.C	34	0.13333333	0.6831302	1.456.969	10.117.457	0.92624255	0.9819303	NA	NA	CBFB	CBFB
ZN410_HUMAN.H11MO.0.D	11	0.04313725	0.4979420	1.843.138	10.112.909	0.87706968	0.9683105	More than 3 adjacent zinc finger factors{2.3.3}	MTF1-like factors{2.3.3.24}	BCL6;BCL6B;CTCF;CTCFL;FEZF1;FEZF2;GFI1;GFI1B;GLI1;GLI2;GLI3;GLI4;GLIS1;GLIS2;GLIS3;HKR1;MTF1;MYNN;MZF1;OSR2;OVOL1;OVOL2;PLAG1;PLAGL1;PLAGL2;PRDM1;PRDM14;PRDM6;SCRT1;SCRT2;SNAI1;SNAI2;SNAI3;WT1;YY1;YY2;ZBTB12;ZBTB14;ZBTB18;ZBTB20;ZBTB26;ZBTB42;ZBTB45;ZBTB47;ZBTB48;ZBTB49;ZBTB6;ZBTB7A;ZBTB7B;ZBTB7C;ZFP14;ZFP2;ZFP28;ZFP30;ZFP37;ZFP42;ZFP64;ZFP69;ZFP69B;ZFP82;ZFP91;ZFX;ZIC1;ZIC2;ZIC3;ZIC4;ZIC5	MTF1;ZNF410

Motif	Nº of probes	% of probes	lower OR	upper OR	OR	p.value	FDR	TF family	TF subfamily	TF.family.member	TF.subfamily.member
										;ZIK1;ZIM3;ZKSCAN1;ZKSCAN2;ZKSCAN3;ZKSCAN4;ZNF121;ZNF124;ZNF133;ZNF136;ZNF138;ZNF14;ZNF140;ZNF143;ZNF146;ZNF148;ZNF155;ZNF157;ZNF160;ZNF169;ZNF175;ZNF177;ZNF18;ZNF180;ZNF181;ZNF2;ZNF20;ZNF212;ZNF213;ZNF214;ZNF221;ZNF222;ZNF223;ZNF224;ZNF225;ZNF226;ZNF227;ZNF229;ZNF230;ZNF232;ZNF233;ZNF234;ZNF235;ZNF24;ZNF25;ZNF250;ZNF257;ZNF26;ZNF260;ZNF263;ZNF264;ZNF268;ZNF274;ZNF276;ZNF28;ZNF280A;ZNF280B;ZNF280C;ZNF280D;ZNF281;ZNF282;ZNF283;ZNF284;ZNF285;ZNF286A;ZNF286B;ZNF3;ZNF30;ZNF300;ZNF302;ZNF317;ZNF32;ZNF320;ZNF322;ZNF324;ZNF324B;ZNF329;ZNF331;ZNF333;ZNF33A;ZNF33B;ZNF343;ZNF345;ZNF347;ZNF350;ZNF354A;ZNF354B;ZNF362;ZNF366;ZNF383;ZNF384;ZNF394;ZNF397;ZNF398;ZNF404;ZNF41;ZNF410;ZNF419;ZNF420;ZNF431;ZNF432;ZNF436;ZNF439;ZNF44;ZNF440;ZNF442;ZNF443;ZNF446;ZNF449;ZNF45;ZNF460;ZNF468;ZNF479;ZNF484;ZNF490;ZNF500;ZNF502;ZNF524;ZNF525;ZNF528;ZNF543;ZNF544;ZNF546;ZNF547;ZNF548;ZNF549;ZNF554;ZNF555;ZNF557;ZNF558;ZNF559;ZNF561;ZNF562;ZNF563;ZNF564;ZNF566;ZNF567;ZNF568;ZNF57;ZNF570;ZNF571;ZNF572;ZNF577;ZNF581;ZNF582;ZNF583;ZNF585A;ZNF586;ZNF589;ZNF595;ZNF599;ZNF600;ZNF605;ZNF607;ZNF611;ZNF613;ZNF614;ZNF615;ZNF616;ZNF619;ZNF620;ZNF621;ZNF625;ZNF627;ZNF649;ZNF652;ZNF653;ZNF665;ZNF667;ZNF669;ZNF670;ZNF672;ZNF679;ZNF680;ZNF683;ZNF689;ZNF692;ZNF701;ZNF705D;ZNF705E;ZNF705G;ZNF708;ZNF709;ZNF71;ZNF710;ZNF713;ZNF721;ZNF727;ZNF729;ZNF736;ZNF75A;ZNF75D;ZNF76;ZNF763;ZNF764;ZNF765;ZNF768;ZNF77;ZNF771;ZNF773;ZNF774;ZNF776;ZNF777;ZNF780A;ZNF780B;ZNF782;ZNF785;ZNF799;ZNF805;ZNF808;ZNF81;ZNF813;ZNF816;ZNF823;ZNF829;ZNF836;ZNF841;ZNF844;ZNF845;ZNF846;ZNF85;ZNF853;ZNF860;ZNF878;ZNF891;ZNF99;ZSCAN16;ZSCAN2;ZSCAN22;ZSCAN23;ZSCAN29;ZSCAN31;ZSCAN32;ZSCAN4;ZSCAN5A;ZSCAN5B;ZSCAN5C;ZSCAN9;ZXDA;ZXDB;ZXDC	
RUNX1_HUMAN.H11MO.0.A	29	0.11372549	0.6613174	1.492.073	10.104.842	0.92110141	0.9808967	Runt-related factors{6.4.1}	Runx1 (PEBP2alphaB, CBF-alpha2, AML-1){6.4.1.0.2}	RUNX1;RUNX2;RUNX3	RUNX1
FEZF1_HUMAN.H11MO.0.C	21	0.08235294	0.6126942	1.579.651	10.090.179	0.90904496	0.9792516	More than 3 adjacent zinc finger factors{2.3.3}	FEZF factors{2.3.3.20}	BCL6;BCL6B;CTCF;CTCFL;FEZF1;FEZF2;GFI1;GFI1B;GLI1;GLI2;GLI3;GLI4;GLIS1;GLIS2;GLIS3;HKR1;MTF1;MYNN;MZF1;OSR2;OVOL1;OVOL2;PLAG1;PLAGL1;PLAGL2;PRDM1;PRDM14;PRDM6;SCRT1;SCRT2;SNAI1;SNAI2;SNAI3;WT1;YY1;YY2;ZBTB12;ZBTB14;ZBTB18;ZBTB20;ZBTB26;ZBTB42;ZBTB45;ZBTB47;ZBTB48;ZBTB49;ZBTB6;ZBTB7A;ZBTB7B;ZBTB7C;ZFP14;ZFP2;ZFP28;ZFP30;ZFP37;ZFP42;ZFP64;ZFP69;ZFP69B;ZFP82;ZFP91;ZFX;ZIC1;ZIC2;ZIC3;ZIC4;ZIC5;ZIK1;ZIM3;ZKSCAN1;ZKSCAN2;ZKSCAN3;ZKSCAN4;ZNF121;ZNF124;ZNF133;ZNF136;ZNF138;ZNF14;ZNF140;ZNF143;ZNF146;ZNF148;ZNF155;ZNF157;ZNF160;ZNF169;ZNF175;ZNF177;ZNF18;ZNF180;ZNF181;ZNF2;ZNF20;ZNF212;ZNF213;ZNF214;ZNF221;ZNF222;ZNF223;ZNF224;ZNF225;ZNF226;ZNF227;ZNF229;ZNF230;ZNF232;ZNF233;ZNF234;ZNF235;ZNF24;ZNF25;ZNF250;ZNF257;ZNF26;ZNF260;ZNF263;ZNF264;ZNF268;ZNF274;ZNF276;ZNF28;ZNF280A;ZNF280B;ZNF280C;ZNF280D;ZNF281;ZNF282;ZNF283;ZNF284;ZNF285;ZNF286A;ZNF286B;ZNF3;ZNF30;ZNF300;ZNF302;ZNF317;ZNF32;ZNF320;ZNF322;ZNF324;ZNF324B;ZNF329;ZNF331;ZNF333;ZNF33A;ZNF33B;ZNF343;ZNF345;ZNF347;ZNF350;ZNF354A;ZNF354B;ZNF362;ZNF366;ZNF383;ZNF384;ZNF394;ZNF397;ZNF398;ZNF404;ZNF41;ZNF410;ZNF419;ZNF420;ZNF431;ZNF432;ZNF436;ZNF439;ZNF44;ZNF440;ZNF442;ZNF443;ZNF446;ZNF449;ZNF45;ZNF460;ZNF468;ZNF479;ZNF484;ZNF490;ZNF500;ZNF502;ZNF524;ZNF525;ZNF528;ZNF543;ZNF544;ZNF546;ZNF547;ZNF548;ZNF549;ZNF554;ZNF555;ZNF557;ZNF558;ZNF559;ZNF561;ZNF562;ZNF563;ZNF564;ZNF566;ZNF567;ZNF568;ZNF57;ZNF570;ZNF571;ZNF572;ZNF577;ZNF5	FEZF1

Motif	Nº of probes	% of probes	lower OR	upper OR	OR	p.value	FDR	TF family	TF subfamily	TF.family.member	TF.subfamily.member
										81;ZNF582;ZNF583;ZNF585A;ZNF586;ZNF589;ZNF595;ZNF599;ZNF600;ZNF605;ZNF607;ZNF611;ZNF613;ZNF614;ZNF615;ZNF616;ZNF619;ZNF620;ZNF621;ZNF625;ZNF627;ZNF649;ZNF652;ZNF653;ZNF665;ZNF667;ZNF669;ZNF670;ZNF672;ZNF679;ZNF680;ZNF683;ZNF689;ZNF692;ZNF701;ZNF705D;ZNF705E;ZNF705G;ZNF708;ZNF709;ZNF71;ZNF710;ZNF713;ZNF721;ZNF727;ZNF729;ZNF736;ZNF75A;ZNF75D;ZNF76;ZNF763;ZNF764;ZNF765;ZNF768;ZNF77;ZNF771;ZNF773;ZNF774;ZNF776;ZNF777;ZNF780A;ZNF780B;ZNF782;ZNF785;ZNF799;ZNF805;ZNF808;ZNF81;ZNF813;ZNF816;ZNF823;ZNF829;ZNF836;ZNF841;ZNF844;ZNF845;ZNF846;ZNF85;ZNF853;ZNF860;ZNF878;ZNF891;ZNF99;ZSCAN16;ZSCAN2;ZSCAN22;ZSCAN23;ZSCAN29;ZSCAN31;ZSCAN32;ZSCAN4;ZSCAN5A;ZSCAN5B;ZSCAN5C;ZSCAN9;ZXDA;ZXDB;ZXDC	
HXD3_HUMAN.H11MO.0.D	12	0.04705882	0.5135433	1.795.155	10.082.590	0.88230888	0.9683105	HOX-related factors{3.1.1}	HOX3{3.1.1.3}	CDX1;CDX2;CDX4;EVX1;EVX2;GBX1;GBX2;GSX1;GSX2;HDX;HMBOX1;HNF1A;HNF1B;HOXA1;HOXA10;HOXA11;HOXA13;HOXA2;HOXA3;HOXA4;HOXA5;HOXA6;HOXA7;HOXA9;HOXB1;HOXB13;HOXB2;HOXB3;HOXB4;HOXB5;HOXB6;HOXB7;HOXB8;HOXB9;HOXC10;HOXC11;HOXC12;HOXC13;HOXC4;HOXC5;HOXC6;HOXC8;HOXC9;HOXD1;HOXD10;HOXD11;HOXD12;HOXD13;HOXD3;HOXD4;HOXD8;HOXD9;MEOX1;MEOX2;MNX1;PDX1;POU1F1;POU2F1;POU2F2;POU2F3;POU3F1;POU3F2;POU3F3;POU3F4;POU4F1;POU4F2;POU4F3;POU5F1;POU5F2;POU6F1;POU6F2	HOXB3;HOXD3
ZN257_HUMAN.H11MO.0.C	33	0.12941176	0.6767792	1.458.036	10.079.444	0.92550485	0.9819303	More than 3 adjacent zinc finger factors{2.3.3}	unclassified{2.3.3.0}	BCL6B;BCL6;CTCFL;CTCF;FEZF1;GFI1B;GFI1;GLI1;GLI2;GLI3;GLIS1;GLIS2;GLIS3;MTF1;MYNN;MZF1;OSR2;OVOL1;OVOL2;ZNF146;PLAG1;PLAGL1;PRDM14;PRDM1;PRDM6;SCRT1;SCRT2;SNAI1;SNAI2;YY1;YY2;WT1;ZNF324;ZNF354A;ZBTB14;ZBTB18;ZBTB48;ZBTB49;ZBTB7A;ZBTB7B;ZBTB6;ZFP64;ZFP28;ZFP42;ZFP82;ZFX;ZIC1;ZIC2;ZIC3;ZIC4;ZIM3;ZKSCAN1;ZKSCAN3;ZNF121;ZNF136;ZNF140;ZNF143;ZNF148;ZNF214;ZNF232;ZNF250;ZNF257;ZNF260;ZNF263;ZNF264;ZNF274;ZNF281;ZNF282;ZNF317;ZNF320;ZNF322;ZNF329;ZNF331;ZNF333;ZNF350;ZNF384;ZNF394;ZNF410;ZNF436;ZNF449;ZNF490;ZNF502;ZNF524;ZNF528;ZNF547;ZNF549;ZNF554;ZNF563;ZNF582;ZNF586;ZNF589;ZNF652;ZNF667;ZNF680;ZNF708;ZNF713;ZNF768;ZNF816;ZNF18;ZNF41;ZNF76;ZNF85;ZSCAN16;ZSCAN22;ZSCAN31;ZSCAN4	MYNN;MZF1;OSR2;PRDM14;PRDM6;WT1;ZBTB14;ZBTB48;ZBTB49;ZFP64;ZFP28;ZIM3;ZNF121;ZNF250;ZNF257;ZNF263;ZNF274;ZNF317;ZNF320;ZNF329;ZNF331;ZNF394;ZNF449;ZNF502;ZNF528;ZNF547;ZNF549;ZNF554;ZNF586;ZNF589;ZNF667;ZNF680;ZNF708;ZNF713;ZNF768;ZNF18;ZNF85;ZSCAN16;ZSCAN22
DLX4_HUMAN.H11MO.0.D	14	0.05490196	0.5417896	1.723.154	10.064.913	0.89066112	0.9683105	NK-related factors{3.1.2}	DLX{3.1.2.5}	BARHL1;BARHL2;BARX1;BARX2;BSX;DBX1;DBX2;DLX1;DLX2;DLX3;DLX4;DLX5;DLX6;EMX1;EMX2;EN1;EN2;HHEX;HLX;HMX1;HMX2;HMX3;LBX1;LBX2;MSX1;MSX2;NANOG;NKX1-1;NKX1-2;NKX2-1;NKX2-2;NKX2-3;NKX2-4;NKX2-5;NKX2-6;NKX2-8;NKX3-1;NKX3-2;NKX6-1;NKX6-2;NKX6-3;NOTO;TLX1;TLX2;TLX3;VAX1;VAX2;VENTX	DLX1;DLX2;DLX3;DLX4;DLX5;DLX6
VSX2_HUMAN.H11MO.0.D	11	0.04313725	0.4955191	1.834.090	10.063.439	0.87761628	0.9683105	Paired-related HD factors{3.1.3}	VSX{3.1.3.28}	ALX1;ALX3;ALX4;ARGFX;ARX;CRX;DMBX1;DPRX;DRGX;DUX4;DUXA;ESX1;GSC;GSC2;HESX1;ISX;LEUTX;MIXL1;NOBOX;OTP;OTX1;OTX2;PHOX2A;PHOX2B;PITX1;PITX2;PITX3;PROP1;PRRX1;PRRX2;RAX;RAX2;RHOXF1;RHOXF2;SEBOX;SHOX;SHOX2;TPRX1;UNCX;VSX1;VSX2	VSX1;VSX2
CEBPZ_HUMAN.H11MO.0.D	14	0.05490196	0.5400054	1.717.498	10.031.872	100.000.000	10.000.000	NA	NA	CEBPZ	CEBPZ
FLI1_HUMAN.H11MO.1.A	64	0.25098039	0.7422036	1.336.148	10.014.997	100.000.000	10.000.000	Ets-related factors{3.5.2}	Ets-like factors{3.5.2.1}	EHF;ELF1;ELF2;ELF3;ELF4;ELF5;ELK1;ELK3;ELK4;ERF;ERG;ETS1;ETS2;ETV1;ETV2;ETV3;ETV3L;ETV4;ETV5;ETV6;ETV7;FEV;FLI1;GABPA;SPDEF;SPI1;SPIB;SPIC	ERG;ETS1;ETS2;ETV2;ETV3;FEV;FLI1;GABPA
GFI1B_HUMAN.H11MO.0.A	22	0.08627451	0.6151963	1.553.314	10.012.171	100.000.000	10.000.000	More than 3 adjacent zinc finger factors{2.3.3}	GFI1 factors{2.3.3.21}	BCL6;BCL6B;CTCF;CTCFL;FEZF1;FEZF2;GFI1;GFI1B;GLI1;GLI2;GLI3;GLI4;GLIS1;GLIS2;GLIS3;HKR1;MTF1;MYNN;MZF1;OSR2;OVOL1;OVOL2;PLAG1;PLAGL1;PLAGL2;PRDM1;PRDM14;PRDM6;SCRT1;SCRT2;SNAI1;SNAI2;SNAI3;WT1;YY1;YY2;ZBTB12;ZBTB14;ZBTB18;ZBTB20;ZBTB26;ZBTB42;ZBTB45;ZBTB47;ZBTB48;ZBTB49;ZBTB6;ZBTB7A;ZBTB7B;ZBTB7C;ZFP14;ZFP2;ZFP28;ZFP30;ZFP37;ZFP42;ZFP64;ZFP69;ZFP69B;ZFP82;ZFP91;ZFX;ZIC1;ZIC2;ZIC3;ZIC4;ZIC5;ZIK1;ZIM3;ZKSCAN1;ZKSCAN2;ZKSCAN3;ZKSCAN4;ZNF121;ZNF124;ZNF133;ZNF136;ZNF138;ZNF14;ZNF140;ZNF14	GFI1B;GFI1

Motif	Nº of probes	% of probes	lower OR	upper OR	OR	p.value	FDR	TF family	TF subfamily	TF.family.member	TF.subfamily.member
										3;ZNF146;ZNF148;ZNF155;ZNF157;ZNF160;ZNF169;ZNF175;ZNF177;ZNF18;ZNF180;ZNF181;ZNF2;ZNF20;ZNF212;ZNF213;ZNF214;ZNF221;ZNF222;ZNF223;ZNF224;ZNF225;ZNF226;ZNF227;ZNF229;ZNF230;ZNF232;ZNF233;ZNF234;ZNF235;ZNF24;ZNF25;ZNF250;ZNF257;ZNF26;ZNF260;ZNF263;ZNF264;ZNF268;ZNF274;ZNF276;ZNF28;ZNF280A;ZNF280B;ZNF280C;ZNF280D;ZNF281;ZNF282;ZNF283;ZNF284;ZNF285;ZNF286A;ZNF286B;ZNF3;ZNF30;ZNF300;ZNF302;ZNF317;ZNF32;ZNF320;ZNF322;ZNF324;ZNF324B;ZNF329;ZNF331;ZNF333;ZNF33A;ZNF33B;ZNF343;ZNF345;ZNF347;ZNF350;ZNF354A;ZNF354B;ZNF362;ZNF366;ZNF383;ZNF384;ZNF394;ZNF397;ZNF398;ZNF404;ZNF41;ZNF410;ZNF419;ZNF420;ZNF431;ZNF432;ZNF436;ZNF439;ZNF44;ZNF440;ZNF442;ZNF443;ZNF446;ZNF449;ZNF45;ZNF460;ZNF468;ZNF479;ZNF484;ZNF490;ZNF500;ZNF502;ZNF524;ZNF525;ZNF528;ZNF543;ZNF544;ZNF546;ZNF547;ZNF548;ZNF549;ZNF554;ZNF555;ZNF557;ZNF558;ZNF559;ZNF561;ZNF562;ZNF563;ZNF564;ZNF566;ZNF567;ZNF568;ZNF57;ZNF570;ZNF571;ZNF572;ZNF577;ZNF581;ZNF582;ZNF583;ZNF585A;ZNF586;ZNF589;ZNF595;ZNF599;ZNF600;ZNF605;ZNF607;ZNF611;ZNF613;ZNF614;ZNF615;ZNF616;ZNF619;ZNF620;ZNF621;ZNF625;ZNF627;ZNF649;ZNF652;ZNF653;ZNF665;ZNF667;ZNF669;ZNF670;ZNF672;ZNF679;ZNF680;ZNF683;ZNF689;ZNF692;ZNF701;ZNF705D;ZNF705E;ZNF705G;ZNF708;ZNF709;ZNF71;ZNF710;ZNF713;ZNF721;ZNF727;ZNF729;ZNF736;ZNF75A;ZNF75D;ZNF76;ZNF763;ZNF764;ZNF765;ZNF768;ZNF77;ZNF771;ZNF773;ZNF774;ZNF776;ZNF777;ZNF780A;ZNF780B;ZNF782;ZNF785;ZNF799;ZNF805;ZNF808;ZNF81;ZNF813;ZNF816;ZNF823;ZNF829;ZNF836;ZNF841;ZNF844;ZNF845;ZNF846;ZNF85;ZNF853;ZNF860;ZNF878;ZNF891;ZNF99;ZSCAN16;ZSCAN2;ZSCAN22;ZSCAN23;ZSCAN29;ZSCAN31;ZSCAN32;ZSCAN4;ZSCAN5A;ZSCAN5B;ZSCAN5C;ZSCAN9;ZXDA;ZXDB;ZXDC	
CDX2_HUMAN.H11MO.0.A	14	0.05490196	0.5383146	1.712.133	10.000.610	100.000.000	10.000.000	HOX-related factors{3.1.1}	CDX (Caudal type homeobox){3.1.1.9}	CDX1;CDX2;CDX4;EVX1;EVX2;GBX1;GBX2;GSX1;GSX2;HDX;HMBOX1;HNF1A;HNF1B;HOXA1;HOXA10;HOXA11;HOXA13;HOXA2;HOXA3;HOXA4;HOXA5;HOXA6;HOXA7;HOXA9;HOXB1;HOXB13;HOXB2;HOXB3;HOXB4;HOXB5;HOXB6;HOXB7;HOXB8;HOXB9;HOXC10;HOXC11;HOXC12;HOXC13;HOXC4;HOXC5;HOXC6;HOXC8;HOXC9;HOXD1;HOXD10;HOXD11;HOXD12;HOXD13;HOXD3;HOXD4;HOXD8;HOXD9;MEOX1;MEOX2;MNX1;PDX1;POU1F1;POU2F1;POU2F2;POU2F3;POU3F1;POU3F2;POU3F3;POU3F4;POU4F1;POU4F2;POU4F3;POU5F1;POU5F2;POU6F1;POU6F2	CDX1;CDX2
ZIC2_HUMAN.H11MO.0.D	70	0.27450980	0.7479551	1.323.350	0.9996808	100.000.000	10.000.000	More than 3 adjacent zinc finger factors{2.3.3}	GLI-like factors{2.3.3.1}	BCL6;BCL6B;CTCF;CTCFL;FEZF1;FEZF2;GFI1;GFI1B;GLI1;GLI2;GLI3;GLI4;GLIS1;GLIS2;GLIS3;HKR1;MTF1;MYNN;MZF1;OSR2;OVOL1;OVOL2;PLAG1;PLAGL1;PLAGL2;PRDM1;PRDM14;PRDM6;SCRT1;SCRT2;SNAI1;SNAI2;SNAI3;WT1;YY1;YY2;ZBTB12;ZBTB14;ZBTB18;ZBTB20;ZBTB26;ZBTB42;ZBTB45;ZBTB47;ZBTB48;ZBTB49;ZBTB6;ZBTB7A;ZBTB7B;ZBTB7C;ZFP14;ZFP2;ZFP28;ZFP30;ZFP37;ZFP42;ZFP64;ZFP69;ZFP69B;ZFP82;ZFP91;ZFX;ZIC1;ZIC2;ZIC3;ZIC4;ZIC5;ZIK1;ZIM3;ZKSCAN1;ZKSCAN2;ZKSCAN3;ZKSCAN4;ZNF121;ZNF124;ZNF133;ZNF136;ZNF138;ZNF14;ZNF140;ZNF143;ZNF146;ZNF148;ZNF155;ZNF157;ZNF160;ZNF169;ZNF175;ZNF177;ZNF18;ZNF180;ZNF181;ZNF2;ZNF20;ZNF212;ZNF213;ZNF214;ZNF221;ZNF222;ZNF223;ZNF224;ZNF225;ZNF226;ZNF227;ZNF229;ZNF230;ZNF232;ZNF233;ZNF234;ZNF235;ZNF24;ZNF25;ZNF250;ZNF257;ZNF26;ZNF260;ZNF263;ZNF264;ZNF268;ZNF274;ZNF276;ZNF28;ZNF280A;ZNF280B;ZNF280C;ZNF280D;ZNF281;ZNF282;ZNF283;ZNF284;ZNF285;ZNF286A;ZNF286B;ZNF3;ZNF30;ZNF300;ZNF302;ZNF317;ZNF32;ZNF320;ZNF322;ZNF324;ZNF324B;ZNF329;ZNF331;ZNF333;ZNF33A;ZNF33B;ZNF343;ZNF345;ZNF347;ZNF350;ZNF354A;ZNF354B;ZNF362;ZNF366;ZNF383;ZNF384;ZNF394;ZNF397;ZNF398;ZNF404;ZNF41;ZNF410;ZNF419;ZNF420;ZNF431;ZNF432;ZNF436;ZNF439;ZNF44;ZNF440;ZNF442;ZNF443;ZNF446;ZNF449;ZNF45;ZNF460;ZNF468;ZNF479;ZNF484;ZNF49	GLI1;GLI2;GLI3;GLIS1;GLIS2;GLIS3;ZIC1;ZIC2;ZIC3;ZIC4

Motif	Nº of probes	% of probes	lower OR	upper OR	OR	p.value	FDR	TF family	TF subfamily	TF.family.member	TF.subfamily.member
										0;ZNF500;ZNF502;ZNF524;ZNF525;ZNF528;ZNF543;ZNF544;ZNF546;ZNF547;ZNF548;ZNF549;ZNF554;ZNF555;ZNF557;ZNF558;ZNF559;ZNF561;ZNF562;ZNF563;ZNF564;ZNF566;ZNF567;ZNF568;ZNF57;ZNF570;ZNF571;ZNF572;ZNF577;ZNF581;ZNF582;ZNF583;ZNF585A;ZNF586;ZNF589;ZNF595;ZNF599;ZNF600;ZNF605;ZNF607;ZNF611;ZNF613;ZNF614;ZNF615;ZNF616;ZNF619;ZNF620;ZNF621;ZNF625;ZNF627;ZNF649;ZNF652;ZNF653;ZNF665;ZNF667;ZNF669;ZNF670;ZNF672;ZNF679;ZNF680;ZNF683;ZNF689;ZNF692;ZNF701;ZNF705D;ZNF705E;ZNF705G;ZNF708;ZNF709;ZNF71;ZNF710;ZNF713;ZNF721;ZNF727;ZNF729;ZNF736;ZNF75A;ZNF75D;ZNF76;ZNF763;ZNF764;ZNF765;ZNF768;ZNF77;ZNF771;ZNF773;ZNF774;ZNF776;ZNF777;ZNF780A;ZNF780B;ZNF782;ZNF785;ZNF799;ZNF805;ZNF808;ZNF81;ZNF813;ZNF816;ZNF823;ZNF829;ZNF836;ZNF841;ZNF844;ZNF845;ZNF846;ZNF85;ZNF853;ZNF860;ZNF878;ZNF891;ZNF99;ZSCAN16;ZSCAN2;ZSCAN22;ZSCAN23;ZSCAN29;ZSCAN31;ZSCAN32;ZSCAN4;ZSCAN5A;ZSCAN5B;ZSCAN5C;ZSCAN9;ZXDA;ZXDB;ZXDC	
ZN549_HUMAN.H11MO.0.C	22	0.08627451	0.6136322	1.549.228	0.9986707	100.000.000	10.000.000	More than 3 adjacent zinc finger factors{2.3.3}	unclassified{2.3.3.0}	BCL6B;BCL6;CTCFL;CTCF;FEZF1;GFI1B;GFI1;GLI1;GLI2;GLI3;GLIS1;GLIS2;GLIS3;MTF1;MYNN;MZF1;OSR2;OVOL1;OVOL2;ZNF146;PLAG1;PLAGL1;PRDM14;PRDM1;PRDM6;SCRT1;SCRT2;SNAI1;SNAI2;YY1;YY2;WT1;ZNF324;ZNF354A;ZBTB14;ZBTB18;ZBTB48;ZBTB49;ZBTB7A;ZBTB7B;ZBTB6;ZFP64;ZFP28;ZFP42;ZFP82;ZFX;ZIC1;ZIC2;ZIC3;ZIC4;ZIM3;ZKSCAN1;ZKSCAN3;ZNF121;ZNF136;ZNF140;ZNF143;ZNF148;ZNF214;ZNF232;ZNF250;ZNF257;ZNF260;ZNF263;ZNF264;ZNF274;ZNF281;ZNF282;ZNF317;ZNF320;ZNF322;ZNF329;ZNF331;ZNF333;ZNF350;ZNF384;ZNF394;ZNF410;ZNF436;ZNF449;ZNF490;ZNF502;ZNF524;ZNF528;ZNF547;ZNF549;ZNF554;ZNF563;ZNF582;ZNF586;ZNF589;ZNF652;ZNF667;ZNF680;ZNF708;ZNF713;ZNF768;ZNF816;ZNF18;ZNF41;ZNF76;ZNF85;ZSCAN16;ZSCAN22;ZSCAN31;ZSCAN4	MYNN;MZF1;OSR2;PRDM14;PRDM6;WT1;ZBTB14;ZBTB48;ZBTB49;ZFP64;ZFP28;ZIM3;ZNF121;ZNF250;ZNF257;ZNF263;ZNF274;ZNF317;ZNF320;ZNF329;ZNF331;ZNF394;ZNF449;ZNF502;ZNF528;ZNF547;ZNF549;ZNF554;ZNF586;ZNF589;ZNF667;ZNF680;ZNF708;ZNF713;ZNF768;ZNF18;ZNF85;ZSCAN16;ZSCAN22
BARH1_HUMAN.H11MO.0.D	13	0.05098039	0.5224996	1.737.121	0.9960998	100.000.000	10.000.000	NK-related factors{3.1.2}	BARHL{3.1.2.1}	BARHL1;BARHL2;BARX1;BARX2;BSX;DBX1;DBX2;DLX1;DLX2;DLX3;DLX4;DLX5;DLX6;EMX1;EMX2;EN1;EN2;HHEX;HLX;HMX1;HMX2;HMX3;LBX1;LBX2;MSX1;MSX2;NANOG;NKX1-1;NKX1-2;NKX2-1;NKX2-2;NKX2-3;NKX2-4;NKX2-5;NKX2-6;NKX2-8;NKX3-1;NKX3-2;NKX6-1;NKX6-2;NKX6-3;NOTO;TLX1;TLX2;TLX3;VAX1;VAX2;VENTX	BARHL1;BARHL2
PPARG_HUMAN.H11MO.0.A	38	0.14901961	0.6858064	1.411.041	0.9957247	100.000.000	10.000.000	Thyroid hormone receptor-related factors (NR1){2.1.2}	PPAR (NR1C){2.1.2.5}	NR1D1;NR1D2;NR1H2;NR1H3;NR1H4;NR1I2;NR1I3;PPARA;PPARD;PPARG;RARA;RARB;RARG;RORA;RORB;RORC;THRA;THRB;VDR	PPARA;PPARD;PPARG
ZN768_HUMAN.H11MO.0.C	60	0.23529412	0.7313113	1.334.410	0.9941018	100.000.000	10.000.000	More than 3 adjacent zinc finger factors{2.3.3}	unclassified{2.3.3.0}	BCL6B;BCL6;CTCFL;CTCF;FEZF1;GFI1B;GFI1;GLI1;GLI2;GLI3;GLIS1;GLIS2;GLIS3;MTF1;MYNN;MZF1;OSR2;OVOL1;OVOL2;ZNF146;PLAG1;PLAGL1;PRDM14;PRDM1;PRDM6;SCRT1;SCRT2;SNAI1;SNAI2;YY1;YY2;WT1;ZNF324;ZNF354A;ZBTB14;ZBTB18;ZBTB48;ZBTB49;ZBTB7A;ZBTB7B;ZBTB6;ZFP64;ZFP28;ZFP42;ZFP82;ZFX;ZIC1;ZIC2;ZIC3;ZIC4;ZIM3;ZKSCAN1;ZKSCAN3;ZNF121;ZNF136;ZNF140;ZNF143;ZNF148;ZNF214;ZNF232;ZNF250;ZNF257;ZNF260;ZNF263;ZNF264;ZNF274;ZNF281;ZNF282;ZNF317;ZNF320;ZNF322;ZNF329;ZNF331;ZNF333;ZNF350;ZNF384;ZNF394;ZNF410;ZNF436;ZNF449;ZNF490;ZNF502;ZNF524;ZNF528;ZNF547;ZNF549;ZNF554;ZNF563;ZNF582;ZNF586;ZNF589;ZNF652;ZNF667;ZNF680;ZNF708;ZNF713;ZNF768;ZNF816;ZNF18;ZNF41;ZNF76;ZNF85;ZSCAN16;ZSCAN22;ZSCAN31;ZSCAN4	MYNN;MZF1;OSR2;PRDM14;PRDM6;WT1;ZBTB14;ZBTB48;ZBTB49;ZFP64;ZFP28;ZIM3;ZNF121;ZNF250;ZNF257;ZNF263;ZNF274;ZNF317;ZNF320;ZNF329;ZNF331;ZNF394;ZNF449;ZNF502;ZNF528;ZNF547;ZNF549;ZNF554;ZNF586;ZNF589;ZNF667;ZNF680;ZNF708;ZNF713;ZNF768;ZNF18;ZNF85;ZSCAN16;ZSCAN22
RARG_HUMAN.H11MO.1.B	38	0.14901961	0.6845189	1.408.389	0.9938539	100.000.000	10.000.000	Thyroid hormone receptor-related factors (NR1){2.1.2}	Retinoic acid receptors (NR1B){2.1.2.1}	NR1D1;NR1D2;NR1H2;NR1H3;NR1H4;NR1I2;NR1I3;PPARA;PPARD;PPARG;RARA;RARB;RARG;RORA;RORB;RORC;THRA;THRB;VDR	RARA;RARB;RARG
ZN708_HUMAN.H11MO.1.D	47	0.18431373	0.7024126	1.359.332	0.9860596	100.000.000	10.000.000	More than 3 adjacent zinc finger factors{2.3.3}	unclassified{2.3.3.0}	BCL6B;BCL6;CTCFL;CTCF;FEZF1;GFI1B;GFI1;GLI1;GLI2;GLI3;GLIS1;GLIS2;GLIS3;MTF1;MYNN;MZF1;OSR2;OVOL1;OVOL2;ZNF146;PLAG1;PLAGL1;PRDM14;PRDM1;PRDM6;SCRT1;SCRT2;SNAI1;SNAI2;YY1;YY2;WT1;ZNF324;ZNF354A;ZBTB14;ZBTB18;ZBTB48;ZBTB49;ZBTB7A;ZBTB7B;ZBT	MYNN;MZF1;OSR2;PRDM14;PRDM6;WT1;ZBTB14;ZBTB48;ZBTB49;ZFP64;ZFP28;ZIM3;ZNF121;ZNF250;ZNF257;ZNF263;ZNF274;ZNF317;ZNF320;ZNF329;ZNF331;ZNF394;ZNF449;ZNF502;ZNF528;ZNF547;ZNF549

Motif	Nº of probes	% of probes	lower OR	upper OR	OR	p.value	FDR	TF family	TF subfamily	TF.family.member	TF.subfamily.member
										B6;ZFP64;ZFP28;ZFP42;ZFP82;ZFX;ZIC1;ZIC2;ZIC3;ZIC4;ZIM3;ZKSCAN1;ZKSCAN3;ZNF121;ZNF136;ZNF140;ZNF143;ZNF148;ZNF214;ZNF232;ZNF250;ZNF257;ZNF260;ZNF263;ZNF264;ZNF274;ZNF281;ZNF282;ZNF317;ZNF320;ZNF322;ZNF329;ZNF331;ZNF333;ZNF350;ZNF384;ZNF394;ZNF410;ZNF436;ZNF449;ZNF490;ZNF502;ZNF524;ZNF528;ZNF547;ZNF549;ZNF554;ZNF563;ZNF582;ZNF586;ZNF589;ZNF652;ZNF667;ZNF680;ZNF708;ZNF713;ZNF768;ZNF816;ZNF18;ZNF41;ZNF76;ZNF85;ZSCAN16;ZSCAN22;ZSCAN31;ZSCAN4	;ZNF554;ZNF586;ZNF589;ZNF667;ZNF680;ZNF708;ZNF713;ZNF768;ZNF18;ZNF85;ZSCAN16;ZSCAN22
VDR_HUMAN.H11MO.0.A	40	0.15686275	0.6835268	1.384.496	0.9838911	100.000.000	10.000.000	Thyroid hormone receptor-related factors (NR1){2.1.2}	Vitamin D receptor (NR1I){2.1.2.4}	NR1D1;NR1D2;NR1H2;NR1H3;NR1H4;NR1I2;NR1I3;PPARA;PPARD;PPARG;RARA;RARB;RARG;RORA;RORB;RORC;THRA;THRB;VDR	NR1I2;NR1I3;VDR
PPARG_HUMAN.H11MO.1.A	22	0.08627451	0.6033487	1.523.297	0.9819293	100.000.000	10.000.000	Thyroid hormone receptor-related factors (NR1){2.1.2}	PPAR (NR1C){2.1.2.5}	NR1D1;NR1D2;NR1H2;NR1H3;NR1H4;NR1I2;NR1I3;PPARA;PPARD;PPARG;RARA;RARB;RARG;RORA;RORB;RORC;THRA;THRB;VDR	PPARA;PPARD;PPARG
NR1H3_HUMAN.H11MO.0.B	29	0.11372549	0.6420473	1.448.540	0.9809888	100.000.000	10.000.000	Thyroid hormone receptor-related factors (NR1){2.1.2}	LXR (NR1H){2.1.2.7}	NR1D1;NR1D2;NR1H2;NR1H3;NR1H4;NR1I2;NR1I3;PPARA;PPARD;PPARG;RARA;RARB;RARG;RORA;RORB;RORC;THRA;THRB;VDR	NR1H2;NR1H3;NR1H4
HXD10_HUMAN.H11MO.0.D	15	0.05882353	0.5386095	1.646.582	0.9779082	100.000.000	10.000.000	HOX-related factors{3.1.1}	HOX9-13{3.1.1.8}	CDX1;CDX2;CDX4;EVX1;EVX2;GBX1;GBX2;GSX1;GSX2;HDX;HMBOX1;HNF1A;HNF1B;HOXA1;HOXA10;HOXA11;HOXA13;HOXA2;HOXA3;HOXA4;HOXA5;HOXA6;HOXA7;HOXA9;HOXB1;HOXB13;HOXB2;HOXB3;HOXB4;HOXB5;HOXB6;HOXB7;HOXB8;HOXB9;HOXC10;HOXC11;HOXC12;HOXC13;HOXC4;HOXC5;HOXC6;HOXC8;HOXC9;HOXD1;HOXD10;HOXD11;HOXD12;HOXD13;HOXD3;HOXD4;HOXD8;HOXD9;MEOX1;MEOX2;MNX1;PDX1;POU1F1;POU2F1;POU2F2;POU2F3;POU3F1;POU3F2;POU3F3;POU3F4;POU4F1;POU4F2;POU4F3;POU5F1;POU5F2;POU6F1;POU6F2	HOXA10;HOXA11;HOXA13;HOXA9;HOXB13;HOXC10;HOXC11;HOXC12;HOXC13;HOXC9;HOXD10;HOXD11;HOXD12;HOXD13;HOXD9
RARG_HUMAN.H11MO.0.B	44	0.17254902	0.6896794	1.358.858	0.9777456	0.93455519	0.9870439	Thyroid hormone receptor-related factors (NR1){2.1.2}	Retinoic acid receptors (NR1B){2.1.2.1}	NR1D1;NR1D2;NR1H2;NR1H3;NR1H4;NR1I2;NR1I3;PPARA;PPARD;PPARG;RARA;RARB;RARG;RORA;RORB;RORC;THRA;THRB;VDR	RARA;RARB;RARG
FOXF1_HUMAN.H11MO.0.D	28	0.10980392	0.6350968	1.451.995	0.9776026	100.000.000	10.000.000	Forkhead box (FOX) factors{3.3.1}	FOXF{3.3.1.6}	FOXA1;FOXA2;FOXA3;FOXB1;FOXB2;FOXC1;FOXC2;FOXD1;FOXD2;FOXD3;FOXD4;FOXD4L1;FOXD4L3;FOXD4L4;FOXD4L5;FOXD4L6;FOXE1;FOXE3;FOXF1;FOXF2;FOXG1;FOXH1;FOXI1;FOXI2;FOXI3;FOXJ1;FOXJ2;FOXJ3;FOXK1;FOXK2;FOXL1;FOXL2;FOXM1;FOXN1;FOXN2;FOXN3;FOXN4;FOXO1;FOXO3;FOXO4;FOXO6;FOXP1;FOXP2;FOXP3;FOXP4;FOXQ1;FOXR1;FOXR2;FOXS1	FOXF1;FOXF2
ZN322_HUMAN.H11MO.0.B	76	0.29803922	0.7365459	1.284.607	0.9766807	0.89169964	0.9683105	More than 3 adjacent zinc finger factors{2.3.3}	ZNF322-like factors{2.3.3.52}	BCL6;BCL6B;CTCF;CTCFL;FEZF1;FEZF2;GFI1;GFI1B;GLI1;GLI2;GLI3;GLI4;GLIS1;GLIS2;GLIS3;HKR1;MTF1;MYNN;MZF1;OSR2;OVOL1;OVOL2;PLAG1;PLAGL1;PLAGL2;PRDM1;PRDM14;PRDM6;SCRT1;SCRT2;SNAI1;SNAI2;SNAI3;WT1;YY1;YY2;ZBTB12;ZBTB14;ZBTB18;ZBTB20;ZBTB26;ZBTB42;ZBTB45;ZBTB47;ZBTB48;ZBTB49;ZBTB6;ZBTB7A;ZBTB7B;ZBTB7C;ZFP14;ZFP2;ZFP28;ZFP30;ZFP37;ZFP42;ZFP64;ZFP69;ZFP69B;ZFP82;ZFP91;ZFX;ZIC1;ZIC2;ZIC3;ZIC4;ZIC5;ZIK1;ZIM3;ZKSCAN1;ZKSCAN2;ZKSCAN3;ZKSCAN4;ZNF121;ZNF124;ZNF133;ZNF136;ZNF138;ZNF14;ZNF140;ZNF143;ZNF146;ZNF148;ZNF155;ZNF157;ZNF160;ZNF169;ZNF175;ZNF177;ZNF18;ZNF180;ZNF181;ZNF2;ZNF20;ZNF212;ZNF213;ZNF214;ZNF221;ZNF222;ZNF223;ZNF224;ZNF225;ZNF226;ZNF227;ZNF229;ZNF230;ZNF232;ZNF233;ZNF234;ZNF235;ZNF24;ZNF25;ZNF250;ZNF257;ZNF26;ZNF260;ZNF263;ZNF264;ZNF268;ZNF274;ZNF276;ZNF28;ZNF280A;ZNF280B;ZNF280C;ZNF280D;ZNF281;ZNF282;ZNF283;ZNF284;ZNF285;ZNF286A;ZNF286B;ZNF3;ZNF30;ZNF300;ZNF302;ZNF317;ZNF32;ZNF320;ZNF322;ZNF324;ZNF324B;ZNF329;ZNF331;ZNF333;ZNF33A;ZNF33B;ZNF343;ZNF345;ZNF347;ZNF350;ZNF354A;ZNF354B;ZNF362;ZNF366;ZNF383;ZNF384;ZNF394;ZNF397;ZNF398;ZNF404;ZNF41;ZNF410;ZNF419;ZNF420;ZNF431;ZNF432;ZNF436;ZNF439;ZNF44;ZNF440;ZNF442;ZNF443;ZNF	ZNF322

Motif	Nº of probes	% of probes	lower OR	upper OR	OR	p.value	FDR	TF family	TF subfamily	TF.family.member	TF.subfamily.member
										446;ZNF449;ZNF45;ZNF460;ZNF468;ZNF479;ZNF484;ZNF490;ZNF500;ZNF502;ZNF524;ZNF525;ZNF528;ZNF543;ZNF544;ZNF546;ZNF547;ZNF548;ZNF549;ZNF554;ZNF555;ZNF557;ZNF558;ZNF559;ZNF561;ZNF562;ZNF563;ZNF564;ZNF566;ZNF567;ZNF568;ZNF57;ZNF570;ZNF571;ZNF572;ZNF577;ZNF581;ZNF582;ZNF583;ZNF585A;ZNF586;ZNF589;ZNF595;ZNF599;ZNF600;ZNF605;ZNF607;ZNF611;ZNF613;ZNF614;ZNF615;ZNF616;ZNF619;ZNF620;ZNF621;ZNF625;ZNF627;ZNF649;ZNF652;ZNF653;ZNF665;ZNF667;ZNF669;ZNF670;ZNF672;ZNF679;ZNF680;ZNF683;ZNF689;ZNF692;ZNF701;ZNF705D;ZNF705E;ZNF705G;ZNF708;ZNF709;ZNF71;ZNF710;ZNF713;ZNF721;ZNF727;ZNF729;ZNF736;ZNF75A;ZNF75D;ZNF76;ZNF763;ZNF764;ZNF765;ZNF768;ZNF77;ZNF771;ZNF773;ZNF774;ZNF776;ZNF777;ZNF780A;ZNF780B;ZNF782;ZNF785;ZNF799;ZNF805;ZNF808;ZNF81;ZNF813;ZNF816;ZNF823;ZNF829;ZNF836;ZNF841;ZNF844;ZNF845;ZNF846;ZNF85;ZNF853;ZNF860;ZNF878;ZNF891;ZNF99;ZSCAN16;ZSCAN2;ZSCAN22;ZSCAN23;ZSCAN29;ZSCAN31;ZSCAN32;ZSCAN4;ZSCAN5A;ZSCAN5B;ZSCAN5C;ZSCAN9;ZXDA;ZXDB;ZXDC	
MESP1_HUMAN.H11MO.0.D	56	0.21960784	0.7123128	1.319.847	0.9764398	0.94006913	0.9915093	Tal-related factors{1.2.3}	Mesp-like factors{1.2.3.3}	ATOH1;ATOH7;ATOH8;BHLHA15;BHLHA9;BHLHE22;BHLHE23;FERD3L;FIGLA;HAND1;HAND2;LYL1;MESP1;MESP2;MSC;MSGN1;NEUROD1;NEUROD2;NEUROD4;NEUROD6;NEUROG1;NEUROG2;NEUROG3;NHLH1;NHLH2;OLIG1;OLIG2;OLIG3;PTF1A;SCX;TAL1;TAL2;TCF15;TCF21;TCF23;TWIST1;TWIST2	MESP1
HIF1A_HUMAN.H11MO.0.C	60	0.23529412	0.7180884	1.310.263	0.9761027	0.94147495	0.9916355	PAS domain factors{1.2.5}	Ahr-like factors{1.2.5.1}	AHR;AHRR;ARNT;ARNT2;ARNTL;ARNTL2;CLOCK;EPAS1;HIF1A;HIF3A;NCOA1;NCOA2;NCOA3;NPAS1;NPAS2;NPAS3;NPAS4;SIM1;SIM2;SOHLH1;SOHLH2;TCFL5	AHR;EPAS1;HIF1A
HES5_HUMAN.H11MO.0.D	74	0.29019608	0.7324391	1.283.206	0.9736552	0.89084572	0.9683105	Hairy-related factors{1.2.4}	Hairy-like factors{1.2.4.1}	BHLHE40;BHLHE41;HELT;HES1;HES2;HES3;HES4;HES5;HES6;HES7;HEY1;HEY2;HEYL	BHLHE40;BHLHE41;HES1;HES5;HES7;HEY1;HEY2
ZNF85_HUMAN.H11MO.0.C	16	0.06274510	0.5471333	1.614.473	0.9732614	100.000.000	10.000.000	More than 3 adjacent zinc finger factors{2.3.3}	unclassified{2.3.3.0}	BCL6B;BCL6;CTCFL;CTCF;FEZF1;GFI1B;GFI1;GLI1;GLI2;GLI3;GLIS1;GLIS2;GLIS3;MTF1;MYNN;MZF1;OSR2;OVOL1;OVOL2;ZNF146;PLAG1;PLAGL1;PRDM14;PRDM1;PRDM6;SCRT1;SCRT2;SNAI1;SNAI2;YY1;YY2;WT1;ZNF324;ZNF354A;ZBTB14;ZBTB18;ZBTB48;ZBTB49;ZBTB7A;ZBTB7B;ZBTB6;ZFP64;ZFP28;ZFP42;ZFP82;ZFX;ZIC1;ZIC2;ZIC3;ZIC4;ZIM3;ZKSCAN1;ZKSCAN3;ZNF121;ZNF136;ZNF140;ZNF143;ZNF148;ZNF214;ZNF232;ZNF250;ZNF257;ZNF260;ZNF263;ZNF264;ZNF274;ZNF281;ZNF282;ZNF317;ZNF320;ZNF322;ZNF329;ZNF331;ZNF333;ZNF350;ZNF384;ZNF394;ZNF410;ZNF436;ZNF449;ZNF490;ZNF502;ZNF524;ZNF528;ZNF547;ZNF549;ZNF554;ZNF563;ZNF582;ZNF586;ZNF589;ZNF652;ZNF667;ZNF680;ZNF708;ZNF713;ZNF768;ZNF816;ZNF18;ZNF41;ZNF76;ZNF85;ZSCAN16;ZSCAN22;ZSCAN31;ZSCAN4	MYNN;MZF1;OSR2;PRDM14;PRDM6;WT1;ZBTB14;ZBTB48;ZBTB49;ZFP64;ZFP28;ZIM3;ZNF121;ZNF250;ZNF257;ZNF263;ZNF274;ZNF317;ZNF320;ZNF329;ZNF331;ZNF394;ZNF449;ZNF502;ZNF528;ZNF547;ZNF549;ZNF554;ZNF586;ZNF589;ZNF667;ZNF680;ZNF708;ZNF713;ZNF768;ZNF18;ZNF85;ZSCAN16;ZSCAN22
ETS1_HUMAN.H11MO.0.A	60	0.23529412	0.7151771	1.304.947	0.9721417	0.88361613	0.9683105	Ets-related factors{3.5.2}	Ets-like factors{3.5.2.1}	EHF;ELF1;ELF2;ELF3;ELF4;ELF5;ELK1;ELK3;ELK4;ERF;ERG;ETS1;ETS2;ETV1;ETV2;ETV3;ETV3L;ETV4;ETV5;ETV6;ETV7;FEV;FLI1;GABPA;SPDEF;SPI1;SPIB;SPIC	ERG;ETS1;ETS2;ETV2;ETV3;FEV;FLI1;GABPA
GSC2_HUMAN.H11MO.0.D	30	0.11764706	0.6402886	1.426.683	0.9716053	100.000.000	10.000.000	Paired-related HD factors{3.1.3}	GSC{3.1.3.9}	ALX1;ALX3;ALX4;ARGFX;ARX;CRX;DMBX1;DPRX;DRGX;DUX4;DUXA;ESX1;GSC;GSC2;HESX1;ISX;LEUTX;MIXL1;NOBOX;OTP;OTX1;OTX2;PHOX2A;PHOX2B;PITX1;PITX2;PITX3;PROP1;PRRX1;PRRX2;RAX;RAX2;RHOXF1;RHOXF2;SEBOX;SHOX;SHOX2;TPRX1;UNCX;VSX1;VSX2	GSC2;GSC
XBP1_HUMAN.H11MO.0.D	81	0.31764706	0.7360821	1.270.919	0.9706281	0.89347554	0.9688743	XBP-1-related factors{1.1.5}	XBP-1{1.1.5.0.1}	XBP1	XBP1
P73_HUMAN.H11MO.1.A	31	0.12156863	0.6436531	1.417.245	0.9702380	100.000.000	10.000.000	p53-related factors{6.3.1}	p73{6.3.1.0.3}	TP53;TP63;TP73	TP73
ZN582_HUMAN.H11MO.0.C	17	0.06666667	0.5540383	1.583.192	0.9675105	100.000.000	10.000.000	More than 3 adjacent zinc finger factors{2.3.3}	ZNF620-like factors{2.3.3.35}	BCL6;BCL6B;CTCF;CTCFL;FEZF1;FEZF2;GFI1;GFI1B;GLI1;GLI2;GLI3;GLI4;GLIS1;GLIS2;GLIS3;HKR1;MTF1;MYNN;MZF1;OSR2;OVOL1;OVOL2;PLAG1;PLAGL1;PLAGL2;PRDM1;PRDM14;PRDM6;SCRT1;SCRT2;SNAI1;SNAI2;SNAI3;WT1;YY1;YY2;ZBTB12;ZBTB14;ZBTB18;ZBTB20;ZBTB26;ZBTB42;ZBTB45;ZBTB47;ZBTB48;ZBTB49;ZBTB6;ZBTB7A;ZBTB	ZNF582

Motif	Nº of probes	% of probes	lower OR	upper OR	OR	p.value	FDR	TF family	TF subfamily	TF.family.member	TF.subfamily.member
										7B;ZBTB7C;ZFP14;ZFP2;ZFP28;ZFP30;ZFP37;ZFP42;ZFP64;ZFP69;ZFP69B;ZFP82;ZFP91;ZFX;ZIC1;ZIC2;ZIC3;ZIC4;ZIC5;ZIK1;ZIM3;ZKSCAN1;ZKSCAN2;ZKSCAN3;ZKSCAN4;ZNF121;ZNF124;ZNF133;ZNF136;ZNF138;ZNF14;ZNF140;ZNF143;ZNF146;ZNF148;ZNF155;ZNF157;ZNF160;ZNF169;ZNF175;ZNF177;ZNF18;ZNF180;ZNF181;ZNF2;ZNF20;ZNF212;ZNF213;ZNF214;ZNF221;ZNF222;ZNF223;ZNF224;ZNF225;ZNF226;ZNF227;ZNF229;ZNF230;ZNF232;ZNF233;ZNF234;ZNF235;ZNF24;ZNF25;ZNF250;ZNF257;ZNF26;ZNF260;ZNF263;ZNF264;ZNF268;ZNF274;ZNF276;ZNF28;ZNF280A;ZNF280B;ZNF280C;ZNF280D;ZNF281;ZNF282;ZNF283;ZNF284;ZNF285;ZNF286A;ZNF286B;ZNF3;ZNF30;ZNF300;ZNF302;ZNF317;ZNF32;ZNF320;ZNF322;ZNF324;ZNF324B;ZNF329;ZNF331;ZNF333;ZNF33A;ZNF33B;ZNF343;ZNF345;ZNF347;ZNF350;ZNF354A;ZNF354B;ZNF362;ZNF366;ZNF383;ZNF384;ZNF394;ZNF397;ZNF398;ZNF404;ZNF41;ZNF410;ZNF419;ZNF420;ZNF431;ZNF432;ZNF436;ZNF439;ZNF44;ZNF440;ZNF442;ZNF443;ZNF446;ZNF449;ZNF45;ZNF460;ZNF468;ZNF479;ZNF484;ZNF490;ZNF500;ZNF502;ZNF524;ZNF525;ZNF528;ZNF543;ZNF544;ZNF546;ZNF547;ZNF548;ZNF549;ZNF554;ZNF555;ZNF557;ZNF558;ZNF559;ZNF561;ZNF562;ZNF563;ZNF564;ZNF566;ZNF567;ZNF568;ZNF57;ZNF570;ZNF571;ZNF572;ZNF577;ZNF581;ZNF582;ZNF583;ZNF585A;ZNF586;ZNF589;ZNF595;ZNF599;ZNF600;ZNF605;ZNF607;ZNF611;ZNF613;ZNF614;ZNF615;ZNF616;ZNF619;ZNF620;ZNF621;ZNF625;ZNF627;ZNF649;ZNF652;ZNF653;ZNF665;ZNF667;ZNF669;ZNF670;ZNF672;ZNF679;ZNF680;ZNF683;ZNF689;ZNF692;ZNF701;ZNF705D;ZNF705E;ZNF705G;ZNF708;ZNF709;ZNF71;ZNF710;ZNF713;ZNF721;ZNF727;ZNF729;ZNF736;ZNF75A;ZNF75D;ZNF76;ZNF763;ZNF764;ZNF765;ZNF768;ZNF77;ZNF771;ZNF773;ZNF774;ZNF776;ZNF777;ZNF780A;ZNF780B;ZNF782;ZNF785;ZNF799;ZNF805;ZNF808;ZNF81;ZNF813;ZNF816;ZNF823;ZNF829;ZNF836;ZNF841;ZNF844;ZNF845;ZNF846;ZNF85;ZNF853;ZNF860;ZNF878;ZNF891;ZNF99;ZSCAN16;ZSCAN2;ZSCAN22;ZSCAN23;ZSCAN29;ZSCAN31;ZSCAN32;ZSCAN4;ZSCAN5A;ZSCAN5B;ZSCAN5C;ZSCAN9;ZXDA;ZXDB;ZXDC	
NFAC4_HUMAN.H11MO.0.C	16	0.06274510	0.5435004	1.603.767	0.9668120	100.000.000	10.000.000	NFAT-related factors{6.1.3}	NFATc4 (NFAT3){6.1.3.0.4}	NFAT5;NFATC1;NFATC2;NFATC3;NFATC4	NFATC4
ZN320_HUMAN.H11MO.0.C	80	0.31372549	0.7305549	1.263.711	0.9643558	0.84052195	0.9608665	More than 3 adjacent zinc finger factors{2.3.3}	unclassified{2.3.3.0}	BCL6B;BCL6;CTCFL;CTCF;FEZF1;GFI1B;GFI1;GLI1;GLI2;GLI3;GLIS1;GLIS2;GLIS3;MTF1;MYNN;MZF1;OSR2;OVOL1;OVOL2;ZNF146;PLAG1;PLAGL1;PRDM14;PRDM1;PRDM6;SCRT1;SCRT2;SNAI1;SNAI2;YY1;YY2;WT1;ZNF324;ZNF354A;ZBTB14;ZBTB18;ZBTB48;ZBTB49;ZBTB7A;ZBTB7B;ZBTB6;ZFP64;ZFP28;ZFP42;ZFP82;ZFX;ZIC1;ZIC2;ZIC3;ZIC4;ZIM3;ZKSCAN1;ZKSCAN3;ZNF121;ZNF136;ZNF140;ZNF143;ZNF148;ZNF214;ZNF232;ZNF250;ZNF257;ZNF260;ZNF263;ZNF264;ZNF274;ZNF281;ZNF282;ZNF317;ZNF320;ZNF322;ZNF329;ZNF331;ZNF333;ZNF350;ZNF384;ZNF394;ZNF410;ZNF436;ZNF449;ZNF490;ZNF502;ZNF524;ZNF528;ZNF547;ZNF549;ZNF554;ZNF563;ZNF582;ZNF586;ZNF589;ZNF652;ZNF667;ZNF680;ZNF708;ZNF713;ZNF768;ZNF816;ZNF18;ZNF41;ZNF76;ZNF85;ZSCAN16;ZSCAN22;ZSCAN31;ZSCAN4	MYNN;MZF1;OSR2;PRDM14;PRDM6;WT1;ZBTB14;ZBTB48;ZBTB49;ZFP64;ZFP28;ZIM3;ZNF121;ZNF250;ZNF257;ZNF263;ZNF274;ZNF317;ZNF320;ZNF329;ZNF331;ZNF394;ZNF449;ZNF502;ZNF528;ZNF547;ZNF549;ZNF554;ZNF586;ZNF589;ZNF667;ZNF680;ZNF708;ZNF713;ZNF768;ZNF18;ZNF85;ZSCAN16;ZSCAN22
THB_HUMAN.H11MO.0.C	47	0.18431373	0.6857390	1.327.013	0.9626197	0.87318969	0.9683105	Thyroid hormone receptor-related factors (NR1){2.1.2}	Thyroid hormone receptors (NR1A){2.1.2.2}	NR1D1;NR1D2;NR1H2;NR1H3;NR1H4;NR1I2;NR1I3;PPARA;PPARD;PPARG;RARA;RARB;RARG;RORA;RORB;RORC;THRA;THRB;VDR	THRA;THRB
TLX1_HUMAN.H11MO.0.D	26	0.10196078	0.6153273	1.448.030	0.9625880	0.91899429	0.9808967	NK-related factors{3.1.2}	TLX{3.1.2.21}	BARHL1;BARHL2;BARX1;BARX2;BSX;DBX1;DBX2;DLX1;DLX2;DLX3;DLX4;DLX5;DLX6;EMX1;EMX2;EN1;EN2;HHEX;HLX;HMX1;HMX2;HMX3;LBX1;LBX2;MSX1;MSX2;NANOG;NKX1-1;NKX1-2;NKX2-1;NKX2-2;NKX2-3;NKX2-4;NKX2-5;NKX2-6;NKX2-8;NKX3-1;NKX3-2;NKX6-1;NKX6-2;NKX6-3;NOTO;TLX1;TLX2;TLX3;VAX1;VAX2;VENTX	TLX1

Motif	Nº of probes	% of probes	lower OR	upper OR	OR	p.value	FDR	TF family	TF subfamily	TF.family.member	TF.subfamily.member
EHF_HUMAN.H11MO.0.B	35	0.13725490	0.6529772	1.379.256	0.9618991	0.92839262	0.9819303	Ets-related factors{3.5.2}	EHF-like factors{3.5.2.4}	EHF;ELF1;ELF2;ELF3;ELF4;ELF5;ELK1;ELK3;ELK4;ERF;ERG;ETS1;ETS2;ETV1;ETV2;ETV3;ETV3L;ETV4;ETV5;ETV6;ETV7;FEV;FLI1;GABPA;SPDEF;SPI1;SPIB;SPIC	EHF;ELF3;ELF5
ZN331_HUMAN.H11MO.0.C	56	0.21960784	0.7016378	1.300.082	0.9617981	0.88090144	0.9683105	More than 3 adjacent zinc finger factors{2.3.3}	unclassified{2.3.3.0}	BCL6B;BCL6;CTCFL;CTCF;FEZF1;GFI1B;GFI1;GLI1;GLI2;GLI3;GLIS1;GLIS2;GLIS3;MTF1;MYNN;MZF1;OSR2;OVOL1;OVOL2;ZNF146;PLAG1;PLAGL1;PRDM14;PRDM1;PRDM6;SCRT1;SCRT2;SNAI1;SNAI2;YY1;YY2;WT1;ZNF324;ZNF354A;ZBTB14;ZBTB18;ZBTB48;ZBTB49;ZBTB7A;ZBTB7B;ZBTB6;ZFP64;ZFP28;ZFP42;ZFP82;ZFX;ZIC1;ZIC2;ZIC3;ZIC4;ZIM3;ZKSCAN1;ZKSCAN3;ZNF121;ZNF136;ZNF140;ZNF143;ZNF148;ZNF214;ZNF232;ZNF250;ZNF257;ZNF260;ZNF263;ZNF264;ZNF274;ZNF281;ZNF282;ZNF317;ZNF320;ZNF322;ZNF329;ZNF331;ZNF333;ZNF350;ZNF384;ZNF394;ZNF410;ZNF436;ZNF449;ZNF490;ZNF502;ZNF524;ZNF528;ZNF547;ZNF549;ZNF554;ZNF563;ZNF582;ZNF586;ZNF589;ZNF652;ZNF667;ZNF680;ZNF708;ZNF713;ZNF768;ZNF816;ZNF18;ZNF41;ZNF76;ZNF85;ZSCAN16;ZSCAN22;ZSCAN31;ZSCAN4	MYNN;MZF1;OSR2;PRDM14;PRDM6;WT1;ZBTB14;ZBTB48;ZBTB49;ZFP64;ZFP28;ZIM3;ZNF121;ZNF250;ZNF257;ZNF263;ZNF274;ZNF317;ZNF320;ZNF329;ZNF331;ZNF394;ZNF449;ZNF502;ZNF528;ZNF547;ZNF549;ZNF554;ZNF586;ZNF589;ZNF667;ZNF680;ZNF708;ZNF713;ZNF768;ZNF18;ZNF85;ZSCAN16;ZSCAN22
MEF2C_HUMAN.H11MO.0.A	25	0.09803922	0.6080531	1.453.774	0.9596981	0.91763107	0.9808967	Regulators of differentiation{5.1.1}	MEF-2{5.1.1.1}	MEF2A;MEF2B;MEF2C;MEF2D	MEF2A;MEF2B;MEF2C;MEF2D
ZN490_HUMAN.H11MO.0.C	19	0.07450980	0.5672043	1.532.868	0.9594524	100.000.000	10.000.000	More than 3 adjacent zinc finger factors{2.3.3}	ZNF763-like factors{2.3.3.33}	BCL6;BCL6B;CTCF;CTCFL;FEZF1;FEZF2;GFI1;GFI1B;GLI1;GLI2;GLI3;GLI4;GLIS1;GLIS2;GLIS3;HKR1;MTF1;MYNN;MZF1;OSR2;OVOL1;OVOL2;PLAG1;PLAGL1;PLAGL2;PRDM1;PRDM14;PRDM6;SCRT1;SCRT2;SNAI1;SNAI2;SNAI3;WT1;YY1;YY2;ZBTB12;ZBTB14;ZBTB18;ZBTB20;ZBTB26;ZBTB42;ZBTB45;ZBTB47;ZBTB48;ZBTB49;ZBTB6;ZBTB7A;ZBTB7B;ZBTB7C;ZFP14;ZFP2;ZFP28;ZFP30;ZFP37;ZFP42;ZFP64;ZFP69;ZFP69B;ZFP82;ZFP91;ZFX;ZIC1;ZIC2;ZIC3;ZIC4;ZIC5;ZIK1;ZIM3;ZKSCAN1;ZKSCAN2;ZKSCAN3;ZKSCAN4;ZNF121;ZNF124;ZNF133;ZNF136;ZNF138;ZNF14;ZNF140;ZNF143;ZNF146;ZNF148;ZNF155;ZNF157;ZNF160;ZNF169;ZNF175;ZNF177;ZNF18;ZNF180;ZNF181;ZNF2;ZNF20;ZNF212;ZNF213;ZNF214;ZNF221;ZNF222;ZNF223;ZNF224;ZNF225;ZNF226;ZNF227;ZNF229;ZNF230;ZNF232;ZNF233;ZNF234;ZNF235;ZNF24;ZNF25;ZNF250;ZNF257;ZNF26;ZNF260;ZNF263;ZNF264;ZNF268;ZNF274;ZNF276;ZNF28;ZNF280A;ZNF280B;ZNF280C;ZNF280D;ZNF281;ZNF282;ZNF283;ZNF284;ZNF285;ZNF286A;ZNF286B;ZNF3;ZNF30;ZNF300;ZNF302;ZNF317;ZNF32;ZNF320;ZNF322;ZNF324;ZNF324B;ZNF329;ZNF331;ZNF333;ZNF33A;ZNF33B;ZNF343;ZNF345;ZNF347;ZNF350;ZNF354A;ZNF354B;ZNF362;ZNF366;ZNF383;ZNF384;ZNF394;ZNF397;ZNF398;ZNF404;ZNF41;ZNF410;ZNF419;ZNF420;ZNF431;ZNF432;ZNF436;ZNF439;ZNF44;ZNF440;ZNF442;ZNF443;ZNF446;ZNF449;ZNF45;ZNF460;ZNF468;ZNF479;ZNF484;ZNF490;ZNF500;ZNF502;ZNF524;ZNF525;ZNF528;ZNF543;ZNF544;ZNF546;ZNF547;ZNF548;ZNF549;ZNF554;ZNF555;ZNF557;ZNF558;ZNF559;ZNF561;ZNF562;ZNF563;ZNF564;ZNF566;ZNF567;ZNF568;ZNF57;ZNF570;ZNF571;ZNF572;ZNF577;ZNF581;ZNF582;ZNF583;ZNF585A;ZNF586;ZNF589;ZNF595;ZNF599;ZNF600;ZNF605;ZNF607;ZNF611;ZNF613;ZNF614;ZNF615;ZNF616;ZNF619;ZNF620;ZNF621;ZNF625;ZNF627;ZNF649;ZNF652;ZNF653;ZNF665;ZNF667;ZNF669;ZNF670;ZNF672;ZNF679;ZNF680;ZNF683;ZNF689;ZNF692;ZNF701;ZNF705D;ZNF705E;ZNF705G;ZNF708;ZNF709;ZNF71;ZNF710;ZNF713;ZNF721;ZNF727;ZNF729;ZNF736;ZNF75A;ZNF75D;ZNF76;ZNF763;ZNF764;ZNF765;ZNF768;ZNF77;ZNF771;ZNF773;ZNF774;ZNF776;ZNF777;ZNF780A;ZNF780B;ZNF782;ZNF785;ZNF799;ZNF805;ZNF808;ZNF81;ZNF813;ZNF816;ZNF823;ZNF829;ZNF836;ZNF841;ZNF844;ZNF845;ZNF846;ZNF85;ZNF853;ZNF860;ZNF878;ZNF891;ZNF99;ZSCAN16;ZSCAN2;ZSCAN22;ZSCAN23;ZSCAN29;ZSCAN31;ZSCAN32;ZSCAN4;ZSCAN5A;ZSCAN5B;ZSCAN5C;ZSCAN9;ZXDA;ZXDB;ZXDC	ZNF136;ZNF490;ZNF563

Motif	Nº of probes	% of probes	lower OR	upper OR	OR	p.value	FDR	TF family	TF subfamily	TF.family.member	TF.subfamily.member
HTF4_HUMAN.H11MO.0.A	69	0.27058824	0.7166661	1.271.377	0.9592922	0.83393976	0.9553753	E2A-related factors{1.2.1}	HTF-4 (TCF-12, HEB){1.2.1.0.3}	TCF12;TCF4;TCF3	TCF12
ELF3_HUMAN.H11MO.0.A	35	0.13725490	0.6511215	1.375.330	0.9591606	0.92843997	0.9819303	Ets-related factors{3.5.2}	EHF-like factors{3.5.2.4}	EHF;ELF1;ELF2;ELF3;ELF4;ELF5;ELK1;ELK3;ELK4;ERF;ERG;ETS1;ETS2;ETV1;ETV2;ETV3;ETV3L;ETV4;ETV5;ETV6;ETV7;FEV;FLI1;GABPA;SPDEF;SPI1;SPIB;SPIC	EHF;ELF3;ELF5
P53_HUMAN.H11MO.1.A	31	0.12156863	0.6330043	1.393.820	0.9542186	0.92482969	0.9819303	p53-related factors{6.3.1}	p53{6.3.1.0.1}	TP53;TP63;TP73	TP53
SMAD1_HUMAN.H11MO.0.D	58	0.22745098	0.6972248	1.281.646	0.9515876	0.82478824	0.9545046	SMAD factors{7.1.1}	Regulatory Smads (R-Smad){7.1.1.1}	SMAD1;SMAD2;SMAD3;SMAD4;SMAD5;SMAD9	SMAD1;SMAD2;SMAD3
ZKSC3_HUMAN.H11MO.0.D	19	0.07450980	0.5624596	1.520.051	0.9514240	100.000.000	10.000.000	More than 3 adjacent zinc finger factors{2.3.3}	ZKSCAN3-like factors{2.3.3.26}	BCL6;BCL6B;CTCF;CTCFL;FEZF1;FEZF2;GFI1;GFI1B;GLI1;GLI2;GLI3;GLI4;GLIS1;GLIS2;GLIS3;HKR1;MTF1;MYNN;MZF1;OSR2;OVOL1;OVOL2;PLAG1;PLAGL1;PLAGL2;PRDM1;PRDM14;PRDM6;SCRT1;SCRT2;SNAI1;SNAI2;SNAI3;WT1;YY1;YY2;ZBTB12;ZBTB14;ZBTB18;ZBTB20;ZBTB26;ZBTB42;ZBTB45;ZBTB47;ZBTB48;ZBTB49;ZBTB6;ZBTB7A;ZBTB7B;ZBTB7C;ZFP14;ZFP2;ZFP28;ZFP30;ZFP37;ZFP42;ZFP64;ZFP69;ZFP69B;ZFP82;ZFP91;ZFX;ZIC1;ZIC2;ZIC3;ZIC4;ZIC5;ZIK1;ZIM3;ZKSCAN1;ZKSCAN2;ZKSCAN3;ZKSCAN4;ZNF121;ZNF124;ZNF133;ZNF136;ZNF138;ZNF14;ZNF140;ZNF143;ZNF146;ZNF148;ZNF155;ZNF157;ZNF160;ZNF169;ZNF175;ZNF177;ZNF18;ZNF180;ZNF181;ZNF2;ZNF20;ZNF212;ZNF213;ZNF214;ZNF221;ZNF222;ZNF223;ZNF224;ZNF225;ZNF226;ZNF227;ZNF229;ZNF230;ZNF232;ZNF233;ZNF234;ZNF235;ZNF24;ZNF25;ZNF250;ZNF257;ZNF26;ZNF260;ZNF263;ZNF264;ZNF268;ZNF274;ZNF276;ZNF28;ZNF280A;ZNF280B;ZNF280C;ZNF280D;ZNF281;ZNF282;ZNF283;ZNF284;ZNF285;ZNF286A;ZNF286B;ZNF3;ZNF30;ZNF300;ZNF302;ZNF317;ZNF32;ZNF320;ZNF322;ZNF324;ZNF324B;ZNF329;ZNF331;ZNF333;ZNF33A;ZNF33B;ZNF343;ZNF345;ZNF347;ZNF350;ZNF354A;ZNF354B;ZNF362;ZNF366;ZNF383;ZNF384;ZNF394;ZNF397;ZNF398;ZNF404;ZNF41;ZNF410;ZNF419;ZNF420;ZNF431;ZNF432;ZNF436;ZNF439;ZNF44;ZNF440;ZNF442;ZNF443;ZNF446;ZNF449;ZNF45;ZNF460;ZNF468;ZNF479;ZNF484;ZNF490;ZNF500;ZNF502;ZNF524;ZNF525;ZNF528;ZNF543;ZNF544;ZNF546;ZNF547;ZNF548;ZNF549;ZNF554;ZNF555;ZNF557;ZNF558;ZNF559;ZNF561;ZNF562;ZNF563;ZNF564;ZNF566;ZNF567;ZNF568;ZNF57;ZNF570;ZNF571;ZNF572;ZNF577;ZNF581;ZNF582;ZNF583;ZNF585A;ZNF586;ZNF589;ZNF595;ZNF599;ZNF600;ZNF605;ZNF607;ZNF611;ZNF613;ZNF614;ZNF615;ZNF616;ZNF619;ZNF620;ZNF621;ZNF625;ZNF627;ZNF649;ZNF652;ZNF653;ZNF665;ZNF667;ZNF669;ZNF670;ZNF672;ZNF679;ZNF680;ZNF683;ZNF689;ZNF692;ZNF701;ZNF705D;ZNF705E;ZNF705G;ZNF708;ZNF709;ZNF71;ZNF710;ZNF713;ZNF721;ZNF727;ZNF729;ZNF736;ZNF75A;ZNF75D;ZNF76;ZNF763;ZNF764;ZNF765;ZNF768;ZNF77;ZNF771;ZNF773;ZNF774;ZNF776;ZNF777;ZNF780A;ZNF780B;ZNF782;ZNF785;ZNF799;ZNF805;ZNF808;ZNF81;ZNF813;ZNF816;ZNF823;ZNF829;ZNF836;ZNF841;ZNF844;ZNF845;ZNF846;ZNF85;ZNF853;ZNF860;ZNF878;ZNF891;ZNF99;ZSCAN16;ZSCAN2;ZSCAN22;ZSCAN23;ZSCAN29;ZSCAN31;ZSCAN32;ZSCAN4;ZSCAN5A;ZSCAN5B;ZSCAN5C;ZSCAN9;ZXDA;ZXDB;ZXDC	ZKSCAN3
MEIS1_HUMAN.H11MO.1.B	36	0.14117647	0.6484670	1.357.199	0.9504083	0.85966738	0.9683105	TALE-type homeo domain factors{3.1.4}	MEIS{3.1.4.2}	IRX1;IRX2;IRX3;IRX4;IRX5;IRX6;MEIS1;MEIS2;MEIS3;MKX;PBX1;PBX2;PBX3;PKNOX1;PKNOX2;TGIF1;TGIF2;TGIF2LX;TGIF2LY	MEIS1;MEIS2;MEIS3
PRDM4_HUMAN.H11MO.0.D	20	0.07843137	0.5691164	1.500.806	0.9493485	0.90939580	0.9792516	Factors with multiple dispersed zinc fingers{2.3.4}	unclassified{2.3.4.0}	BCL11A;E4F1;MECOM;HIC1;HIC2;HINFP;IKZF1;INSM1;MAZ;PATZ1;PRDM4;REST;RREB1;SALL4;VEZF1;ZBTB17;ZBTB4;HIVEP1;HIVEP2;ZNF134;ZNF219;ZNF335;ZNF341;ZNF382;ZNF418;ZNF423;ZNF467;ZNF770;ZNF784;ZNF8	E4F1;PRDM4;REST;RREB1;ZBTB17;ZBTB4;ZNF335;ZNF341;ZNF467;ZNF770;ZNF784;ZNF8
TYY1_HUMAN.H11MO.0.A	24	0.09411765	0.5946246	1.445.959	0.9474836	0.91625923	0.9808967	More than 3 adjacent zinc finger factors{2.3.3}	YY1-like factors{2.3.3.9}	BCL6;BCL6B;CTCF;CTCFL;FEZF1;FEZF2;GFI1;GFI1B;GLI1;GLI2;GLI3;GLI4;GLIS1;GLIS2;GLIS3;HKR1;MTF1;MYNN;MZF1;OSR2;OVOL1;OVOL2;PLAG1;PLAGL1;PLAGL2;PRDM1	YY1;YY2;ZFP42

Motif	Nº of probes	% of probes	lower OR	upper OR	OR	p.value	FDR	TF family	TF subfamily	TF.family.member	TF.subfamily.member
										;PRDM14;PRDM6;SCRT1;SCRT2;SNAI1;SNAI2;SNAI3;WT1;YY1;YY2;ZBTB12;ZBTB14;ZBTB18;ZBTB20;ZBTB26;ZBTB42;ZBTB45;ZBTB47;ZBTB48;ZBTB49;ZBTB6;ZBTB7A;ZBTB7B;ZBTB7C;ZFP14;ZFP2;ZFP28;ZFP30;ZFP37;ZFP42;ZFP64;ZFP69;ZFP69B;ZFP82;ZFP91;ZFX;ZIC1;ZIC2;ZIC3;ZIC4;ZIC5;ZIK1;ZIM3;ZKSCAN1;ZKSCAN2;ZKSCAN3;ZKSCAN4;ZNF121;ZNF124;ZNF133;ZNF136;ZNF138;ZNF14;ZNF140;ZNF143;ZNF146;ZNF148;ZNF155;ZNF157;ZNF160;ZNF169;ZNF175;ZNF177;ZNF18;ZNF180;ZNF181;ZNF2;ZNF20;ZNF212;ZNF213;ZNF214;ZNF221;ZNF222;ZNF223;ZNF224;ZNF225;ZNF226;ZNF227;ZNF229;ZNF230;ZNF232;ZNF233;ZNF234;ZNF235;ZNF24;ZNF25;ZNF250;ZNF257;ZNF26;ZNF260;ZNF263;ZNF264;ZNF268;ZNF274;ZNF276;ZNF28;ZNF280A;ZNF280B;ZNF280C;ZNF280D;ZNF281;ZNF282;ZNF283;ZNF284;ZNF285;ZNF286A;ZNF286B;ZNF3;ZNF30;ZNF300;ZNF302;ZNF317;ZNF32;ZNF320;ZNF322;ZNF324;ZNF324B;ZNF329;ZNF331;ZNF333;ZNF33A;ZNF33B;ZNF343;ZNF345;ZNF347;ZNF350;ZNF354A;ZNF354B;ZNF362;ZNF366;ZNF383;ZNF384;ZNF394;ZNF397;ZNF398;ZNF404;ZNF41;ZNF410;ZNF419;ZNF420;ZNF431;ZNF432;ZNF436;ZNF439;ZNF44;ZNF440;ZNF442;ZNF443;ZNF446;ZNF449;ZNF45;ZNF460;ZNF468;ZNF479;ZNF484;ZNF490;ZNF500;ZNF502;ZNF524;ZNF525;ZNF528;ZNF543;ZNF544;ZNF546;ZNF547;ZNF548;ZNF549;ZNF554;ZNF555;ZNF557;ZNF558;ZNF559;ZNF561;ZNF562;ZNF563;ZNF564;ZNF566;ZNF567;ZNF568;ZNF57;ZNF570;ZNF571;ZNF572;ZNF577;ZNF581;ZNF582;ZNF583;ZNF585A;ZNF586;ZNF589;ZNF595;ZNF599;ZNF600;ZNF605;ZNF607;ZNF611;ZNF613;ZNF614;ZNF615;ZNF616;ZNF619;ZNF620;ZNF621;ZNF625;ZNF627;ZNF649;ZNF652;ZNF653;ZNF665;ZNF667;ZNF669;ZNF670;ZNF672;ZNF679;ZNF680;ZNF683;ZNF689;ZNF692;ZNF701;ZNF705D;ZNF705E;ZNF705G;ZNF708;ZNF709;ZNF71;ZNF710;ZNF713;ZNF721;ZNF727;ZNF729;ZNF736;ZNF75A;ZNF75D;ZNF76;ZNF763;ZNF764;ZNF765;ZNF768;ZNF77;ZNF771;ZNF773;ZNF774;ZNF776;ZNF777;ZNF780A;ZNF780B;ZNF782;ZNF785;ZNF799;ZNF805;ZNF808;ZNF81;ZNF813;ZNF816;ZNF823;ZNF829;ZNF836;ZNF841;ZNF844;ZNF845;ZNF846;ZNF85;ZNF853;ZNF860;ZNF878;ZNF891;ZNF99;ZSCAN16;ZSCAN2;ZSCAN22;ZSCAN23;ZSCAN29;ZSCAN31;ZSCAN32;ZSCAN4;ZSCAN5A;ZSCAN5B;ZSCAN5C;ZSCAN9;ZXDA;ZXDB;ZXDC	
ZEB1_HUMAN.H11MO.0.A	49	0.19215686	0.6785173	1.298.757	0.9467701	0.81436554	0.9470224	HD-ZF factors{3.1.8}	ZEB{3.1.8.3}	ADNP;ADNP2;HOMEZ;NANOGNB;TSHZ1;TSHZ2;TSHZ3;ZEB1;ZEB2;ZFHX2;ZFHX3;ZFHX4;ZHX1;ZHX2;ZHX3	ZEB1
AP2C_HUMAN.H11MO.0.A	69	0.27058824	0.7060523	1.252.546	0.9450554	0.72787885	0.9074091	AP-2{1.3.1}	AP-2gamma{1.3.1.0.3}	TFAP2A;TFAP2B;TFAP2C;TFAP2D	TFAP2C
SOX1_HUMAN.H11MO.0.D	11	0.04313725	0.4653063	1.722.199	0.9450090	100.000.000	10.000.000	SOX-related factors{4.1.1}	Group B{4.1.1.2}	BBX;CIC;HBP1;SOX1;SOX10;SOX11;SOX12;SOX13;SOX14;SOX15;SOX17;SOX18;SOX2;SOX21;SOX3;SOX30;SOX4;SOX5;SOX6;SOX7;SOX8;SOX9;SRY	SOX1;SOX21;SOX2;SOX3
COT1_HUMAN.H11MO.1.C	55	0.21568627	0.6875661	1.279.327	0.9447289	0.76430352	0.9193105	RXR-related receptors (NR2){2.1.3}	COUP-like receptors (NR2F){2.1.3.5}	HNF4A;HNF4G;NR2C1;NR2C2;NR2E1;NR2E3;NR2F1;NR2F2;NR2F6;RXRA;RXRB;RXRG	NR2F1;NR2F2;NR2F6
ETV4_HUMAN.H11MO.0.B	51	0.20000000	0.6785110	1.285.711	0.9415469	0.75828824	0.9193105	Ets-related factors{3.5.2}	Elk-like factors{3.5.2.2}	EHF;ELF1;ELF2;ELF3;ELF4;ELF5;ELK1;ELK3;ELK4;ERF;ERG;ETS1;ETS2;ETV1;ETV2;ETV3;ETV3L;ETV4;ETV5;ETV6;ETV7;FEV;FLI1;GABPA;SPDEF;SPI1;SPIB;SPIC	ELK1;ELK3;ELK4;ETV1;ETV4;ETV5
LHX4_HUMAN.H11MO.0.D	10	0.03921569	0.4448543	1.759.493	0.9399116	100.000.000	10.000.000	HD-LIM factors{3.1.5}	Lhx-3-like factors{3.1.5.4}	ISL1;ISL2;LHX1;LHX2;LHX3;LHX4;LHX5;LHX6;LHX8;LHX9;LMX1A;LMX1B	LHX3;LHX4
NR2C1_HUMAN.H11MO.0.C	33	0.12941176	0.6289402	1.354.971	0.9366668	0.78487952	0.9281321	RXR-related receptors (NR2){2.1.3}	Testicular receptors (NR2C){2.1.3.4}	HNF4A;HNF4G;NR2C1;NR2C2;NR2E1;NR2E3;NR2F1;NR2F2;NR2F6;RXRA;RXRB;RXRG	NR2C1;NR2C2
COT1_HUMAN.H11MO.0.C	73	0.28627451	0.7036252	1.235.599	0.9365526	0.68164778	0.8759174	RXR-related receptors (NR2){2.1.3}	COUP-like receptors (NR2F){2.1.3.5}	HNF4A;HNF4G;NR2C1;NR2C2;NR2E1;NR2E3;NR2F1;NR2F2;NR2F6;RXRA;RXRB;RXRG	NR2F1;NR2F2;NR2F6

Motif	Nº of probes	% of probes	lower OR	upper OR	OR	p.value	FDR	TF family	TF subfamily	TF.family.member	TF.subfamily.member
CR3L1_HUMAN.H11MO.0.D	94	0.36862745	0.7177664	1.215.079	0.9360937	0.65206781	0.8634820	CREB-related factors{1.1.7}	CREB-3-like factors{1.1.7.2}	ATF1;ATF6;ATF6B;CREB1;CREB3;CREB3L1;CREB3L2;CREB3L3;CREB3L4;CREBL2;CREBZF;CREM	CREB3L1;CREB3L2;CREB3
HEY1_HUMAN.H11MO.0.D	68	0.26666667	0.6980837	1.241.831	0.9358112	0.67564041	0.8711016	Hairy-related factors{1.2.4}	Hairy-like factors{1.2.4.1}	BHLHE40;BHLHE41;HELT;HES1;HES2;HES3;HES4;HES5;HES6;HES7;HEY1;HEY2;HEYL	BHLHE40;BHLHE41;HES1;HES5;HES7;HEY1;HEY2
ARI5B_HUMAN.H11MO.0.C	13	0.05098039	0.4908752	1.631.828	0.9357772	100.000.000	10.000.000	ARID-related factors{3.7.1}	ARID5{3.7.1.5}	ARID2;ARID3A;ARID5A;ARID5B;KDM5B	ARID5B
NR1D1_HUMAN.H11MO.0.B	42	0.16470588	0.6546488	1.307.006	0.9348645	0.74135111	0.9126911	Thyroid hormone receptor-related factors (NR1){2.1.2}	Rev-ErbA (NR1D){2.1.2.3}	NR1D1;NR1D2;NR1H2;NR1H3;NR1H4;NR1I2;NR1I3;PPARA;PPARD;PPARG;RARA;RARB;RARG;RORA;RORB;RORC;THRA;THRB;VDR	NR1D1
ZN652_HUMAN.H11MO.0.D	10	0.03921569	0.4422014	1.748.987	0.9343033	100.000.000	10.000.000	More than 3 adjacent zinc finger factors{2.3.3}	ZNF652-like factors{2.3.3.29}	BCL6;BCL6B;CTCF;CTCFL;FEZF1;FEZF2;GFI1;GFI1B;GLI1;GLI2;GLI3;GLI4;GLIS1;GLIS2;GLIS3;HKR1;MTF1;MYNN;MZF1;OSR2;OVOL1;OVOL2;PLAG1;PLAGL1;PLAGL2;PRDM1;PRDM14;PRDM6;SCRT1;SCRT2;SNAI1;SNAI2;SNAI3;WT1;YY1;YY2;ZBTB12;ZBTB14;ZBTB18;ZBTB20;ZBTB26;ZBTB42;ZBTB45;ZBTB47;ZBTB48;ZBTB49;ZBTB6;ZBTB7A;ZBTB7B;ZBTB7C;ZFP14;ZFP2;ZFP28;ZFP30;ZFP37;ZFP42;ZFP64;ZFP69;ZFP69B;ZFP82;ZFP91;ZFX;ZIC1;ZIC2;ZIC3;ZIC4;ZIC5;ZIK1;ZIM3;ZKSCAN1;ZKSCAN2;ZKSCAN3;ZKSCAN4;ZNF121;ZNF124;ZNF133;ZNF136;ZNF138;ZNF14;ZNF140;ZNF143;ZNF146;ZNF148;ZNF155;ZNF157;ZNF160;ZNF169;ZNF175;ZNF177;ZNF18;ZNF180;ZNF181;ZNF2;ZNF20;ZNF212;ZNF213;ZNF214;ZNF221;ZNF222;ZNF223;ZNF224;ZNF225;ZNF226;ZNF227;ZNF229;ZNF230;ZNF232;ZNF233;ZNF234;ZNF235;ZNF24;ZNF25;ZNF250;ZNF257;ZNF26;ZNF260;ZNF263;ZNF264;ZNF268;ZNF274;ZNF276;ZNF28;ZNF280A;ZNF280B;ZNF280C;ZNF280D;ZNF281;ZNF282;ZNF283;ZNF284;ZNF285;ZNF286A;ZNF286B;ZNF3;ZNF30;ZNF300;ZNF302;ZNF317;ZNF32;ZNF320;ZNF322;ZNF324;ZNF324B;ZNF329;ZNF331;ZNF333;ZNF33A;ZNF33B;ZNF343;ZNF345;ZNF347;ZNF350;ZNF354A;ZNF354B;ZNF362;ZNF366;ZNF383;ZNF384;ZNF394;ZNF397;ZNF398;ZNF404;ZNF41;ZNF410;ZNF419;ZNF420;ZNF431;ZNF432;ZNF436;ZNF439;ZNF44;ZNF440;ZNF442;ZNF443;ZNF446;ZNF449;ZNF45;ZNF460;ZNF468;ZNF479;ZNF484;ZNF490;ZNF500;ZNF502;ZNF524;ZNF525;ZNF528;ZNF543;ZNF544;ZNF546;ZNF547;ZNF548;ZNF549;ZNF554;ZNF555;ZNF557;ZNF558;ZNF559;ZNF561;ZNF562;ZNF563;ZNF564;ZNF566;ZNF567;ZNF568;ZNF57;ZNF570;ZNF571;ZNF572;ZNF577;ZNF581;ZNF582;ZNF583;ZNF585A;ZNF586;ZNF589;ZNF595;ZNF599;ZNF600;ZNF605;ZNF607;ZNF611;ZNF613;ZNF614;ZNF615;ZNF616;ZNF619;ZNF620;ZNF621;ZNF625;ZNF627;ZNF649;ZNF652;ZNF653;ZNF665;ZNF667;ZNF669;ZNF670;ZNF672;ZNF679;ZNF680;ZNF683;ZNF689;ZNF692;ZNF701;ZNF705D;ZNF705E;ZNF705G;ZNF708;ZNF709;ZNF71;ZNF710;ZNF713;ZNF721;ZNF727;ZNF729;ZNF736;ZNF75A;ZNF75D;ZNF76;ZNF763;ZNF764;ZNF765;ZNF768;ZNF77;ZNF771;ZNF773;ZNF774;ZNF776;ZNF777;ZNF780A;ZNF780B;ZNF782;ZNF785;ZNF799;ZNF805;ZNF808;ZNF81;ZNF813;ZNF816;ZNF823;ZNF829;ZNF836;ZNF841;ZNF844;ZNF845;ZNF846;ZNF85;ZNF853;ZNF860;ZNF878;ZNF891;ZNF99;ZSCAN16;ZSCAN2;ZSCAN22;ZSCAN23;ZSCAN29;ZSCAN31;ZSCAN32;ZSCAN4;ZSCAN5A;ZSCAN5B;ZSCAN5C;ZSCAN9;ZXDA;ZXDB;ZXDC	ZNF652
FLI1_HUMAN.H11MO.0.A	87	0.34117647	0.7111986	1.215.514	0.9325321	0.64708693	0.8631557	Ets-related factors{3.5.2}	Ets-like factors{3.5.2.1}	EHF;ELF1;ELF2;ELF3;ELF4;ELF5;ELK1;ELK3;ELK4;ERF;ERG;ETS1;ETS2;ETV1;ETV2;ETV3;ETV3L;ETV4;ETV5;ETV6;ETV7;FEV;FLI1;GABPA;SPDEF;SPI1;SPIB;SPIC	ERG;ETS1;ETS2;ETV2;ETV3;FEV;FLI1;GABPA
THA_HUMAN.H11MO.1.D	31	0.12156863	0.6165393	1.357.615	0.9294118	0.77960013	0.9272965	Thyroid hormone receptor-related factors (NR1){2.1.2}	Thyroid hormone receptors (NR1A){2.1.2.2}	NR1D1;NR1D2;NR1H2;NR1H3;NR1H4;NR1I2;NR1I3;PPARA;PPARD;PPARG;RARA;RARB;RARG;RORA;RORB;RORC;THRA;THRB;VDR	THRA;THRB
TBX20_HUMAN.H11MO.0.D	32	0.12549020	0.6184598	1.346.625	0.9265275	0.78243432	0.9272965	TBX1-related factors{6.5.3}	TBX20{6.5.3.0.5}	TBX1;TBX10;TBX15;TBX18;TBX20;TBX22	TBX20

Motif	Nº of probes	% of probes	lower OR	upper OR	OR	p.value	FDR	TF family	TF subfamily	TF.family.member	TF.subfamily.member
ELF5_HUMAN.H11MO.0.A	52	0.20392157	0.6665531	1.257.005	0.9225646	0.64874719	0.8634820	Ets-related factors{3.5.2}	EHF-like factors{3.5.2.4}	EHF;ELF1;ELF2;ELF3;ELF4;ELF5;ELK1;ELK3;ELK4;ERF;ERG;ETS1;ETS2;ETV1;ETV2;ETV3;ETV3L;ETV4;ETV5;ETV6;ETV7;FEV;FLI1;GABPA;SPDEF;SPI1;SPIB;SPIC	EHF;ELF3;ELF5
EPAS1_HUMAN.H11MO.0.B	66	0.25882353	0.6843701	1.224.454	0.9203243	0.62317956	0.8503919	PAS domain factors{1.2.5}	Ahr-like factors{1.2.5.1}	AHR;AHRR;ARNT;ARNT2;ARNTL;ARNTL2;CLOCK;EPAS1;HIF1A;HIF3A;NCOA1;NCOA2;NCOA3;NPAS1;NPAS2;NPAS3;NPAS4;SIM1;SIM2;SOHLH1;SOHLH2;TCFL5	AHR;EPAS1;HIF1A
SMAD4_HUMAN.H11MO.0.B	43	0.16862745	0.6467989	1.282.654	0.9202648	0.68378555	0.8772024	SMAD factors{7.1.1}	Co-activating Smads (Co-Smad){7.1.1.2}	SMAD1;SMAD2;SMAD3;SMAD4;SMAD5;SMAD9	SMAD4
PBX1_HUMAN.H11MO.0.A	26	0.10196078	0.5882111	1.384.154	0.9201425	0.76387037	0.9193105	TALE-type homeo domain factors{3.1.4}	PBX{3.1.4.4}	IRX2;IRX3;MEIS1;MEIS2;MEIS3;PBX1;PBX2;PBX3;PKNOX1;TGIF2LX;TGIF1;TGIF2	PBX1;PBX2;PBX3
FOXL1_HUMAN.H11MO.0.D	34	0.13333333	0.6211500	1.324.714	0.9198935	0.72057796	0.9063060	Forkhead box (FOX) factors{3.3.1}	FOXL{3.3.1.12}	FOXA1;FOXA2;FOXA3;FOXB1;FOXB2;FOXC1;FOXC2;FOXD1;FOXD2;FOXD3;FOXD4;FOXD4L1;FOXD4L3;FOXD4L4;FOXD4L5;FOXD4L6;FOXE1;FOXE3;FOXF1;FOXF2;FOXG1;FOXH1;FOXI1;FOXI2;FOXI3;FOXJ1;FOXJ2;FOXJ3;FOXK1;FOXK2;FOXL1;FOXL2;FOXM1;FOXN1;FOXN2;FOXN3;FOXN4;FOXO1;FOXO3;FOXO4;FOXO6;FOXP1;FOXP2;FOXP3;FOXP4;FOXQ1;FOXR1;FOXR2;FOXS1	FOXL1
MNX1_HUMAN.H11MO.0.D	19	0.07450980	0.5438424	1.469.527	0.9198395	0.81798438	0.9497981	HOX-related factors{3.1.1}	MNX{3.1.1.13}	CDX1;CDX2;CDX4;EVX1;EVX2;GBX1;GBX2;GSX1;GSX2;HDX;HMBOX1;HNF1A;HNF1B;HOXA1;HOXA10;HOXA11;HOXA13;HOXA2;HOXA3;HOXA4;HOXA5;HOXA6;HOXA7;HOXA9;HOXB1;HOXB13;HOXB2;HOXB3;HOXB4;HOXB5;HOXB6;HOXB7;HOXB8;HOXB9;HOXC10;HOXC11;HOXC12;HOXC13;HOXC4;HOXC5;HOXC6;HOXC8;HOXC9;HOXD1;HOXD10;HOXD11;HOXD12;HOXD13;HOXD3;HOXD4;HOXD8;HOXD9;MEOX1;MEOX2;MNX1;PDX1;POU1F1;POU2F1;POU2F2;POU2F3;POU3F1;POU3F2;POU3F3;POU3F4;POU4F1;POU4F2;POU4F3;POU5F1;POU5F2;POU6F1;POU6F2	MNX1
ZN554_HUMAN.H11MO.1.D	60	0.23529412	0.6759677	1.233.352	0.9188163	0.61296972	0.8439279	More than 3 adjacent zinc finger factors{2.3.3}	unclassified{2.3.3.0}	BCL6B;BCL6;CTCFL;CTCF;FEZF1;GFI1B;GFI1;GLI1;GLI2;GLI3;GLIS1;GLIS2;GLIS3;MTF1;MYNN;MZF1;OSR2;OVOL1;OVOL2;ZNF146;PLAG1;PLAGL1;PRDM14;PRDM1;PRDM6;SCRT1;SCRT2;SNAI1;SNAI2;YY1;YY2;WT1;ZNF324;ZNF354A;ZBTB14;ZBTB18;ZBTB48;ZBTB49;ZBTB7A;ZBTB7B;ZBTB6;ZFP64;ZFP28;ZFP42;ZFP82;ZFX;ZIC1;ZIC2;ZIC3;ZIC4;ZIM3;ZKSCAN1;ZKSCAN3;ZNF121;ZNF136;ZNF140;ZNF143;ZNF148;ZNF214;ZNF232;ZNF250;ZNF257;ZNF260;ZNF263;ZNF264;ZNF274;ZNF281;ZNF282;ZNF317;ZNF320;ZNF322;ZNF329;ZNF331;ZNF333;ZNF350;ZNF384;ZNF394;ZNF410;ZNF436;ZNF449;ZNF490;ZNF502;ZNF524;ZNF528;ZNF547;ZNF549;ZNF554;ZNF563;ZNF582;ZNF586;ZNF589;ZNF652;ZNF667;ZNF680;ZNF708;ZNF713;ZNF768;ZNF816;ZNF18;ZNF41;ZNF76;ZNF85;ZSCAN16;ZSCAN22;ZSCAN31;ZSCAN4	MYNN;MZF1;OSR2;PRDM14;PRDM6;WT1;ZBTB14;ZBTB48;ZBTB49;ZFP64;ZFP28;ZIM3;ZNF121;ZNF250;ZNF257;ZNF263;ZNF274;ZNF317;ZNF320;ZNF329;ZNF331;ZNF394;ZNF449;ZNF502;ZNF528;ZNF547;ZNF549;ZNF554;ZNF586;ZNF589;ZNF667;ZNF680;ZNF708;ZNF713;ZNF768;ZNF18;ZNF85;ZSCAN16;ZSCAN22
ZN680_HUMAN.H11MO.0.C	21	0.08235294	0.5572417	1.436.603	0.9176803	0.82556280	0.9545046	More than 3 adjacent zinc finger factors{2.3.3}	unclassified{2.3.3.0}	BCL6B;BCL6;CTCFL;CTCF;FEZF1;GFI1B;GFI1;GLI1;GLI2;GLI3;GLIS1;GLIS2;GLIS3;MTF1;MYNN;MZF1;OSR2;OVOL1;OVOL2;ZNF146;PLAG1;PLAGL1;PRDM14;PRDM1;PRDM6;SCRT1;SCRT2;SNAI1;SNAI2;YY1;YY2;WT1;ZNF324;ZNF354A;ZBTB14;ZBTB18;ZBTB48;ZBTB49;ZBTB7A;ZBTB7B;ZBTB6;ZFP64;ZFP28;ZFP42;ZFP82;ZFX;ZIC1;ZIC2;ZIC3;ZIC4;ZIM3;ZKSCAN1;ZKSCAN3;ZNF121;ZNF136;ZNF140;ZNF143;ZNF148;ZNF214;ZNF232;ZNF250;ZNF257;ZNF260;ZNF263;ZNF264;ZNF274;ZNF281;ZNF282;ZNF317;ZNF320;ZNF322;ZNF329;ZNF331;ZNF333;ZNF350;ZNF384;ZNF394;ZNF410;ZNF436;ZNF449;ZNF490;ZNF502;ZNF524;ZNF528;ZNF547;ZNF549;ZNF554;ZNF563;ZNF582;ZNF586;ZNF589;ZNF652;ZNF667;ZNF680;ZNF708;ZNF713;ZNF768;ZNF816;ZNF18;ZNF41;ZNF76;ZNF85;ZSCAN16;ZSCAN22;ZSCAN31;ZSCAN4	MYNN;MZF1;OSR2;PRDM14;PRDM6;WT1;ZBTB14;ZBTB48;ZBTB49;ZFP64;ZFP28;ZIM3;ZNF121;ZNF250;ZNF257;ZNF263;ZNF274;ZNF317;ZNF320;ZNF329;ZNF331;ZNF394;ZNF449;ZNF502;ZNF528;ZNF547;ZNF549;ZNF554;ZNF586;ZNF589;ZNF667;ZNF680;ZNF708;ZNF713;ZNF768;ZNF18;ZNF85;ZSCAN16;ZSCAN22
HLTF_HUMAN.H11MO.0.D	23	0.09019608	0.5699017	1.411.101	0.9173524	0.83231134	0.9550844	NA	NA	HLTF	HLTF
STF1_HUMAN.H11MO.0.B	36	0.14117647	0.6249497	1.307.995	0.9159418	0.72679656	0.9074091	FTZ-F1-related receptors (NR5){2.1.5}	FTZ-F1 (SF-1) (NR5A1){2.1.5.0.1}	NR5A1;NR5A2	NR5A1

Motif	Nº of probes	% of probes	lower OR	upper OR	OR	p.value	FDR	TF family	TF subfamily	TF.family.member	TF.subfamily.member
LHX8_HUMAN.H11MO.0.D	9	0.03529412	0.4123873	1.763.360	0.9131384	100.000.000	10.000.000	HD-LIM factors{3.1.5}	Lhx-6-like factors{3.1.5.5}	ISL1;ISL2;LHX1;LHX2;LHX3;LHX4;LHX5;LHX6;LHX8;LHX9;LMX1A;LMX1B	LHX6;LHX8
SOX7_HUMAN.H11MO.0.D	11	0.04313725	0.4469305	1.654.228	0.9076864	0.88275018	0.9683105	SOX-related factors{4.1.1}	Group F{4.1.1.6}	BBX;CIC;HBP1;SOX1;SOX10;SOX11;SOX12;SOX13;SOX14;SOX15;SOX17;SOX18;SOX2;SOX21;SOX3;SOX30;SOX4;SOX5;SOX6;SOX7;SOX8;SOX9;SRY	SOX17;SOX18;SOX7
MEOX2_HUMAN.H11MO.0.D	13	0.05098039	0.4759691	1.582.218	0.9073452	0.89112915	0.9683105	HOX-related factors{3.1.1}	MEOX{3.1.1.14}	CDX1;CDX2;CDX4;EVX1;EVX2;GBX1;GBX2;GSX1;GSX2;HDX;HMBOX1;HNF1A;HNF1B;HOXA1;HOXA10;HOXA11;HOXA13;HOXA2;HOXA3;HOXA4;HOXA5;HOXA6;HOXA7;HOXA9;HOXB1;HOXB13;HOXB2;HOXB3;HOXB4;HOXB5;HOXB6;HOXB7;HOXB8;HOXB9;HOXC10;HOXC11;HOXC12;HOXC13;HOXC4;HOXC5;HOXC6;HOXC8;HOXC9;HOXD1;HOXD10;HOXD11;HOXD12;HOXD13;HOXD3;HOXD4;HOXD8;HOXD9;MEOX1;MEOX2;MNX1;PDX1;POU1F1;POU2F1;POU2F2;POU2F3;POU3F1;POU3F2;POU3F3;POU3F4;POU4F1;POU4F2;POU4F3;POU5F1;POU5F2;POU6F1;POU6F2	MEOX1;MEOX2
FOXG1_HUMAN.H11MO.0.D	40	0.15686275	0.6294603	1.274.922	0.9060715	0.61728793	0.8464226	Forkhead box (FOX) factors{3.3.1}	FOXG{3.3.1.7}	FOXA1;FOXA2;FOXA3;FOXB1;FOXB2;FOXC1;FOXC2;FOXD1;FOXD2;FOXD3;FOXD4;FOXD4L1;FOXD4L3;FOXD4L4;FOXD4L5;FOXD4L6;FOXE1;FOXE3;FOXF1;FOXF2;FOXG1;FOXH1;FOXI1;FOXI2;FOXI3;FOXJ1;FOXJ2;FOXJ3;FOXK1;FOXK2;FOXL1;FOXL2;FOXM1;FOXN1;FOXN2;FOXN3;FOXN4;FOXO1;FOXO3;FOXO4;FOXO6;FOXP1;FOXP2;FOXP3;FOXP4;FOXQ1;FOXR1;FOXR2;FOXS1	FOXG1
DLX6_HUMAN.H11MO.0.D	13	0.05098039	0.4750656	1.579.212	0.9056223	0.89118386	0.9683105	NK-related factors{3.1.2}	DLX{3.1.2.5}	BARHL1;BARHL2;BARX1;BARX2;BSX;DBX1;DBX2;DLX1;DLX2;DLX3;DLX4;DLX5;DLX6;EMX1;EMX2;EN1;EN2;HHEX;HLX;HMX1;HMX2;HMX3;LBX1;LBX2;MSX1;MSX2;NANOG;NKX1-1;NKX1-2;NKX2-1;NKX2-2;NKX2-3;NKX2-4;NKX2-5;NKX2-6;NKX2-8;NKX3-1;NKX3-2;NKX6-1;NKX6-2;NKX6-3;NOTO;TLX1;TLX2;TLX3;VAX1;VAX2;VENTX	DLX1;DLX2;DLX3;DLX4;DLX5;DLX6
HXB13_HUMAN.H11MO.0.A	12	0.04705882	0.4612636	1.612.205	0.9056050	0.88719177	0.9683105	HOX-related factors{3.1.1}	HOX9-13{3.1.1.8}	CDX1;CDX2;CDX4;EVX1;EVX2;GBX1;GBX2;GSX1;GSX2;HDX;HMBOX1;HNF1A;HNF1B;HOXA1;HOXA10;HOXA11;HOXA13;HOXA2;HOXA3;HOXA4;HOXA5;HOXA6;HOXA7;HOXA9;HOXB1;HOXB13;HOXB2;HOXB3;HOXB4;HOXB5;HOXB6;HOXB7;HOXB8;HOXB9;HOXC10;HOXC11;HOXC12;HOXC13;HOXC4;HOXC5;HOXC6;HOXC8;HOXC9;HOXD1;HOXD10;HOXD11;HOXD12;HOXD13;HOXD3;HOXD4;HOXD8;HOXD9;MEOX1;MEOX2;MNX1;PDX1;POU1F1;POU2F1;POU2F2;POU2F3;POU3F1;POU3F2;POU3F3;POU3F4;POU4F1;POU4F2;POU4F3;POU5F1;POU5F2;POU6F1;POU6F2	HOXA10;HOXA11;HOXA13;HOXA9;HOXB13;HOXC10;HOXC11;HOXC12;HOXC13;HOXC9;HOXD10;HOXD11;HOXD12;HOXD13;HOXD9
ZNF76_HUMAN.H11MO.0.C	57	0.22352941	0.6620192	1.221.715	0.9054898	0.55791203	0.8177760	More than 3 adjacent zinc finger factors{2.3.3}	ZNF76-like factors{2.3.3.28}	BCL6;BCL6B;CTCF;CTCFL;FEZF1;FEZF2;GFI1;GFI1B;GLI1;GLI2;GLI3;GLI4;GLIS1;GLIS2;GLIS3;HKR1;MTF1;MYNN;MZF1;OSR2;OVOL1;OVOL2;PLAG1;PLAGL1;PLAGL2;PRDM1;PRDM14;PRDM6;SCRT1;SCRT2;SNAI1;SNAI2;SNAI3;WT1;YY1;YY2;ZBTB12;ZBTB14;ZBTB18;ZBTB20;ZBTB26;ZBTB42;ZBTB45;ZBTB47;ZBTB48;ZBTB49;ZBTB6;ZBTB7A;ZBTB7B;ZBTB7C;ZFP14;ZFP2;ZFP28;ZFP30;ZFP37;ZFP42;ZFP64;ZFP69;ZFP69B;ZFP82;ZFP91;ZFX;ZIC1;ZIC2;ZIC3;ZIC4;ZIC5;ZIK1;ZIM3;ZKSCAN1;ZKSCAN2;ZKSCAN3;ZKSCAN4;ZNF121;ZNF124;ZNF133;ZNF136;ZNF138;ZNF14;ZNF140;ZNF143;ZNF146;ZNF148;ZNF155;ZNF157;ZNF160;ZNF169;ZNF175;ZNF177;ZNF18;ZNF180;ZNF181;ZNF2;ZNF20;ZNF212;ZNF213;ZNF214;ZNF221;ZNF222;ZNF223;ZNF224;ZNF225;ZNF226;ZNF227;ZNF229;ZNF230;ZNF232;ZNF233;ZNF234;ZNF235;ZNF24;ZNF25;ZNF250;ZNF257;ZNF26;ZNF260;ZNF263;ZNF264;ZNF268;ZNF274;ZNF276;ZNF28;ZNF280A;ZNF280B;ZNF280C;ZNF280D;ZNF281;ZNF282;ZNF283;ZNF284;ZNF285;ZNF286A;ZNF286B;ZNF3;ZNF30;ZNF300;ZNF302;ZNF317;ZNF32;ZNF320;ZNF322;ZNF324;ZNF324B;ZNF329;ZNF331;ZNF333;ZNF33A;ZNF33B;ZNF343;ZNF345;ZNF347;ZNF350;ZNF354A;ZNF354B;ZNF362;ZNF366;ZNF383;ZNF384;ZNF394;ZNF397;ZNF398;ZNF404;ZNF41;ZNF410;ZNF419;ZNF420;ZNF431;ZNF432;ZNF436;ZNF439;ZNF44;ZNF440;ZNF442;ZNF443;ZNF446;ZNF449;ZNF45;ZNF460;ZNF468;ZNF479;ZNF484;ZNF490;ZNF500;ZNF502;ZNF524;ZNF525;ZNF528;ZNF543;ZNF544;ZNF546;ZNF547;ZNF548;ZNF549;ZNF554;ZNF555;ZNF557;Z	ZNF143;ZNF76

Motif	Nº of probes	% of probes	lower OR	upper OR	OR	p.value	FDR	TF family	TF subfamily	TF.family.member	TF.subfamily.member
										NF558;ZNF559;ZNF561;ZNF562;ZNF563;ZNF564;ZNF566;ZNF567;ZNF568;ZNF57;ZNF570;ZNF571;ZNF572;ZNF577;ZNF581;ZNF582;ZNF583;ZNF585A;ZNF586;ZNF589;ZNF595;ZNF599;ZNF600;ZNF605;ZNF607;ZNF611;ZNF613;ZNF614;ZNF615;ZNF616;ZNF619;ZNF620;ZNF621;ZNF625;ZNF627;ZNF649;ZNF652;ZNF653;ZNF665;ZNF667;ZNF669;ZNF670;ZNF672;ZNF679;ZNF680;ZNF683;ZNF689;ZNF692;ZNF701;ZNF705D;ZNF705E;ZNF705G;ZNF708;ZNF709;ZNF71;ZNF710;ZNF713;ZNF721;ZNF727;ZNF729;ZNF736;ZNF75A;ZNF75D;ZNF76;ZNF763;ZNF764;ZNF765;ZNF768;ZNF77;ZNF771;ZNF773;ZNF774;ZNF776;ZNF777;ZNF780A;ZNF780B;ZNF782;ZNF785;ZNF799;ZNF805;ZNF808;ZNF81;ZNF813;ZNF816;ZNF823;ZNF829;ZNF836;ZNF841;ZNF844;ZNF845;ZNF846;ZNF85;ZNF853;ZNF860;ZNF878;ZNF891;ZNF99;ZSCAN16;ZSCAN2;ZSCAN22;ZSCAN23;ZSCAN29;ZSCAN31;ZSCAN32;ZSCAN4;ZSCAN5A;ZSCAN5B;ZSCAN5C;ZSCAN9;ZXDA;ZXDB;ZXDC	
ATF1_HUMAN.H11MO.0.B	31	0.12156863	0.6003160	1.321.828	0.9049132	0.71130971	0.8990488	CREB-related factors{1.1.7}	CREB-like factors{1.1.7.1}	ATF1;ATF6;ATF6B;CREB1;CREB3;CREB3L1;CREB3L2;CREB3L3;CREB3L4;CREBL2;CREBZF;CREM	ATF1;CREB1;CREM
CEBPB_HUMAN.H11MO.0.A	10	0.03921569	0.4279366	1.692.571	0.9041466	0.87784344	0.9683105	C/EBP-related{1.1.8}	C/EBP{1.1.8.1}	CEBPA;CEBPB;CEBPD;CEBPE;CEBPG;DBP;DDIT3;HLF;NFIL3;TEF	CEBPA;CEBPB;CEBPD;CEBPE;CEBPG;DDIT3
TCF7_HUMAN.H11MO.0.A	17	0.06666667	0.5173967	1.478.498	0.9035599	0.80959279	0.9443208	TCF-7-related factors{4.1.3}	TCF-7 (TCF-1) [1]{4.1.3.0.1}	LEF1;TAF1;TCF7;TCF7L1;TCF7L2	TCF7
NOBOX_HUMAN.H11MO.0.C	8	0.03137255	0.3849470	1.807.355	0.9022498	100.000.000	10.000.000	Paired-related HD factors{3.1.3}	NOBOX{3.1.3.15}	ALX1;ALX3;ALX4;ARGFX;ARX;CRX;DMBX1;DPRX;DRGX;DUX4;DUXA;ESX1;GSC;GSC2;HESX1;ISX;LEUTX;MIXL1;NOBOX;OTP;OTX1;OTX2;PHOX2A;PHOX2B;PITX1;PITX2;PITX3;PROP1;PRRX1;PRRX2;RAX;RAX2;RHOXF1;RHOXF2;SEBOX;SHOX;SHOX2;TPRX1;UNCX;VSX1;VSX2	NOBOX
THA11_HUMAN.H11MO.0.B	30	0.11764706	0.5945630	1.324.726	0.9021498	0.70778591	0.8960639	THAP-related factors{2.9.1}	THAP11 (HRIHFB2206){2.9.1.0.11}	THAP1;THAP10;THAP11;THAP12;THAP2;THAP3;THAP4;THAP5;THAP6;THAP7;THAP8;THAP9	THAP11
ETV5_HUMAN.H11MO.0.C	61	0.23921569	0.6643757	1.207.946	0.9013387	0.51964260	0.7871207	Ets-related factors{3.5.2}	Elk-like factors{3.5.2.2}	EHF;ELF1;ELF2;ELF3;ELF4;ELF5;ELK1;ELK3;ELK4;ERF;ERG;ETS1;ETS2;ETV1;ETV2;ETV3;ETV3L;ETV4;ETV5;ETV6;ETV7;FEV;FLI1;GABPA;SPDEF;SPI1;SPIB;SPIC	ELK1;ELK3;ELK4;ETV1;ETV4;ETV5
GFI1_HUMAN.H11MO.0.C	19	0.07450980	0.5326112	1.439.213	0.9008195	0.73272704	0.9111815	More than 3 adjacent zinc finger factors{2.3.3}	GFI1 factors{2.3.3.21}	BCL6;BCL6B;CTCF;CTCFL;FEZF1;FEZF2;GFI1;GFI1B;GLI1;GLI2;GLI3;GLI4;GLIS1;GLIS2;GLIS3;HKR1;MTF1;MYNN;MZF1;OSR2;OVOL1;OVOL2;PLAG1;PLAGL1;PLAGL2;PRDM1;PRDM14;PRDM6;SCRT1;SCRT2;SNAI1;SNAI2;SNAI3;WT1;YY1;YY2;ZBTB12;ZBTB14;ZBTB18;ZBTB20;ZBTB26;ZBTB42;ZBTB45;ZBTB47;ZBTB48;ZBTB49;ZBTB6;ZBTB7A;ZBTB7B;ZBTB7C;ZFP14;ZFP2;ZFP28;ZFP30;ZFP37;ZFP42;ZFP64;ZFP69;ZFP69B;ZFP82;ZFP91;ZFX;ZIC1;ZIC2;ZIC3;ZIC4;ZIC5;ZIK1;ZIM3;ZKSCAN1;ZKSCAN2;ZKSCAN3;ZKSCAN4;ZNF121;ZNF124;ZNF133;ZNF136;ZNF138;ZNF14;ZNF140;ZNF143;ZNF146;ZNF148;ZNF155;ZNF157;ZNF160;ZNF169;ZNF175;ZNF177;ZNF18;ZNF180;ZNF181;ZNF2;ZNF20;ZNF212;ZNF213;ZNF214;ZNF221;ZNF222;ZNF223;ZNF224;ZNF225;ZNF226;ZNF227;ZNF229;ZNF230;ZNF232;ZNF233;ZNF234;ZNF235;ZNF24;ZNF25;ZNF250;ZNF257;ZNF26;ZNF260;ZNF263;ZNF264;ZNF268;ZNF274;ZNF276;ZNF28;ZNF280A;ZNF280B;ZNF280C;ZNF280D;ZNF281;ZNF282;ZNF283;ZNF284;ZNF285;ZNF286A;ZNF286B;ZNF3;ZNF30;ZNF300;ZNF302;ZNF317;ZNF32;ZNF320;ZNF322;ZNF324;ZNF324B;ZNF329;ZNF331;ZNF333;ZNF33A;ZNF33B;ZNF343;ZNF345;ZNF347;ZNF350;ZNF354A;ZNF354B;ZNF362;ZNF366;ZNF383;ZNF384;ZNF394;ZNF397;ZNF398;ZNF404;ZNF41;ZNF410;ZNF419;ZNF420;ZNF431;ZNF432;ZNF436;ZNF439;ZNF44;ZNF440;ZNF442;ZNF443;ZNF446;ZNF449;ZNF45;ZNF460;ZNF468;ZNF479;ZNF484;ZNF490;ZNF500;ZNF502;ZNF524;ZNF525;ZNF528;ZNF543;ZNF544;ZNF546;ZNF547;ZNF548;ZNF549;ZNF554;ZNF555;ZNF557;ZNF558;ZNF559;ZNF561;ZNF562;ZNF563;ZNF564;ZNF566;ZNF567;ZNF568;ZNF57;ZNF570;ZNF571;ZNF572;ZNF577;ZNF5	GFI1B;GFI1

Motif	Nº of probes	% of probes	lower OR	upper OR	OR	p.value	FDR	TF family	TF subfamily	TF.family.member	TF.subfamily.member
										81;ZNF582;ZNF583;ZNF585A;ZNF586;ZNF589;ZNF595;ZNF599;ZNF600;ZNF605;ZNF607;ZNF611;ZNF613;ZNF614;ZNF615;ZNF616;ZNF619;ZNF620;ZNF621;ZNF625;ZNF627;ZNF649;ZNF652;ZNF653;ZNF665;ZNF667;ZNF669;ZNF670;ZNF672;ZNF679;ZNF680;ZNF683;ZNF689;ZNF692;ZNF701;ZNF705D;ZNF705E;ZNF705G;ZNF708;ZNF709;ZNF71;ZNF710;ZNF713;ZNF721;ZNF727;ZNF729;ZNF736;ZNF75A;ZNF75D;ZNF76;ZNF763;ZNF764;ZNF765;ZNF768;ZNF77;ZNF771;ZNF773;ZNF774;ZNF776;ZNF777;ZNF780A;ZNF780B;ZNF782;ZNF785;ZNF799;ZNF805;ZNF808;ZNF81;ZNF813;ZNF816;ZNF823;ZNF829;ZNF836;ZNF841;ZNF844;ZNF845;ZNF846;ZNF85;ZNF853;ZNF860;ZNF878;ZNF891;ZNF99;ZSCAN16;ZSCAN2;ZSCAN22;ZSCAN23;ZSCAN29;ZSCAN31;ZSCAN32;ZSCAN4;ZSCAN5A;ZSCAN5B;ZSCAN5C;ZSCAN9;ZXDA;ZXDB;ZXDC	
TBX2_HUMAN.H11MO.0.D	31	0.12156863	0.5967481	1.313.972	0.8995336	0.64501899	0.8631094	TBX2-related factors{6.5.4}	TBX2{6.5.4.0.1}	TBX2;TBX3;TBX4;TBX5	TBX2
HSF1_HUMAN.H11MO.0.A	22	0.08627451	0.5508380	1.390.668	0.8964491	0.74828531	0.9172146	HSF factors{3.4.1}	HSF1 (HSTF1){3.4.1.0.1}	HSF1;HSF2;HSF4;HSF5;HSFX1;HSFY1;HSFY1; HSFY2	HSF1
RARA_HUMAN.H11MO.1.A	55	0.21568627	0.6524009	1.213.904	0.8963630	0.50618251	0.7769854	Thyroid hormone receptor-related factors (NR1){2.1.2}	Retinoic acid receptors (NR1B){2.1.2.1}	NR1D1;NR1D2;NR1H2;NR1H3;NR1H4;NR1I2;NR1I3;PPARA;PPARD;PPARG;RARA;RARB;RARG;RORA;RORB;RORC;THRA;THRB;VDR	RARA;RARB;RARG
ZN384_HUMAN.H11MO.0.C	14	0.05490196	0.4820660	1.533.094	0.8955646	0.79352103	0.9354812	More than 3 adjacent zinc finger factors{2.3.3}	ZNF362-like factors{2.3.3.37}	BCL6;BCL6B;CTCF;CTCFL;FEZF1;FEZF2;GFI1;GFI1B;GLI1;GLI2;GLI3;GLI4;GLIS1;GLIS2;GLIS3;HKR1;MTF1;MYNN;MZF1;OSR2;OVOL1;OVOL2;PLAG1;PLAGL1;PLAGL2;PRDM1;PRDM14;PRDM6;SCRT1;SCRT2;SNAI1;SNAI2;SNAI3;WT1;YY1;YY2;ZBTB12;ZBTB14;ZBTB18;ZBTB20;ZBTB26;ZBTB42;ZBTB45;ZBTB47;ZBTB48;ZBTB49;ZBTB6;ZBTB7A;ZBTB7B;ZBTB7C;ZFP14;ZFP2;ZFP28;ZFP30;ZFP37;ZFP42;ZFP64;ZFP69;ZFP69B;ZFP82;ZFP91;ZFX;ZIC1;ZIC2;ZIC3;ZIC4;ZIC5;ZIK1;ZIM3;ZKSCAN1;ZKSCAN2;ZKSCAN3;ZKSCAN4;ZNF121;ZNF124;ZNF133;ZNF136;ZNF138;ZNF14;ZNF140;ZNF143;ZNF146;ZNF148;ZNF155;ZNF157;ZNF160;ZNF169;ZNF175;ZNF177;ZNF18;ZNF180;ZNF181;ZNF2;ZNF20;ZNF212;ZNF213;ZNF214;ZNF221;ZNF222;ZNF223;ZNF224;ZNF225;ZNF226;ZNF227;ZNF229;ZNF230;ZNF232;ZNF233;ZNF234;ZNF235;ZNF24;ZNF25;ZNF250;ZNF257;ZNF26;ZNF260;ZNF263;ZNF264;ZNF268;ZNF274;ZNF276;ZNF28;ZNF280A;ZNF280B;ZNF280C;ZNF280D;ZNF281;ZNF282;ZNF283;ZNF284;ZNF285;ZNF286A;ZNF286B;ZNF3;ZNF30;ZNF300;ZNF302;ZNF317;ZNF32;ZNF320;ZNF322;ZNF324;ZNF324B;ZNF329;ZNF331;ZNF333;ZNF33A;ZNF33B;ZNF343;ZNF345;ZNF347;ZNF350;ZNF354A;ZNF354B;ZNF362;ZNF366;ZNF383;ZNF384;ZNF394;ZNF397;ZNF398;ZNF404;ZNF41;ZNF410;ZNF419;ZNF420;ZNF431;ZNF432;ZNF436;ZNF439;ZNF44;ZNF440;ZNF442;ZNF443;ZNF446;ZNF449;ZNF45;ZNF460;ZNF468;ZNF479;ZNF484;ZNF490;ZNF500;ZNF502;ZNF524;ZNF525;ZNF528;ZNF543;ZNF544;ZNF546;ZNF547;ZNF548;ZNF549;ZNF554;ZNF555;ZNF557;ZNF558;ZNF559;ZNF561;ZNF562;ZNF563;ZNF564;ZNF566;ZNF567;ZNF568;ZNF57;ZNF570;ZNF571;ZNF572;ZNF577;ZNF581;ZNF582;ZNF583;ZNF585A;ZNF586;ZNF589;ZNF595;ZNF599;ZNF600;ZNF605;ZNF607;ZNF611;ZNF613;ZNF614;ZNF615;ZNF616;ZNF619;ZNF620;ZNF621;ZNF625;ZNF627;ZNF649;ZNF652;ZNF653;ZNF665;ZNF667;ZNF669;ZNF670;ZNF672;ZNF679;ZNF680;ZNF683;ZNF689;ZNF692;ZNF701;ZNF705D;ZNF705E;ZNF705G;ZNF708;ZNF709;ZNF71;ZNF710;ZNF713;ZNF721;ZNF727;ZNF729;ZNF736;ZNF75A;ZNF75D;ZNF76;ZNF763;ZNF764;ZNF765;ZNF768;ZNF77;ZNF771;ZNF773;ZNF774;ZNF776;ZNF777;ZNF780A;ZNF780B;ZNF782;ZNF785;ZNF799;ZNF805;ZNF808;ZNF81;ZNF813;ZNF816;ZNF823;ZNF829;ZNF836;ZNF841;ZNF844;ZNF845;ZNF846;ZNF85;ZNF853;ZNF860;ZNF878;ZNF891;ZNF99;ZSCAN16;ZSCAN2;ZSCAN22	ZNF384

Motif	Nº of probes	% of probes	lower OR	upper OR	OR	p.value	FDR	TF family	TF subfamily	TF.family.member	TF.subfamily.member
										;ZSCAN23;ZSCAN29;ZSCAN31;ZSCAN32;ZSCAN4;ZSCAN5A;ZSCAN5B;ZSCAN5C;ZSCAN9;ZXDA;ZXDB;ZXDC	
ZN502_HUMAN.H11MO.0.C	15	0.05882353	0.4928642	15.067.175	0.8948458	0.79944011	0.9381557	More than 3 adjacent zinc finger factors{2.3.3}	unclassified{2.3.3.0}	BCL6B;BCL6;CTCFL;CTCF;FEZF1;GFI1B;GFI1;GLI1;GLI2;GLI3;GLIS1;GLIS2;GLIS3;MTF1;MYNN;MZF1;OSR2;OVOL1;OVOL2;ZNF146;PLAG1;PLAGL1;PRDM14;PRDM1;PRDM6;SCRT1;SCRT2;SNAI1;SNAI2;YY1;YY2;WT1;ZNF324;ZNF354A;ZBTB14;ZBTB18;ZBTB48;ZBTB49;ZBTB7A;ZBTB7B;ZBTB6;ZFP64;ZFP28;ZFP42;ZFP82;ZFX;ZIC1;ZIC2;ZIC3;ZIC4;ZIM3;ZKSCAN1;ZKSCAN3;ZNF121;ZNF136;ZNF140;ZNF143;ZNF148;ZNF214;ZNF232;ZNF250;ZNF257;ZNF260;ZNF263;ZNF264;ZNF274;ZNF281;ZNF282;ZNF317;ZNF320;ZNF322;ZNF329;ZNF331;ZNF333;ZNF350;ZNF384;ZNF394;ZNF410;ZNF436;ZNF449;ZNF490;ZNF502;ZNF524;ZNF528;ZNF547;ZNF549;ZNF554;ZNF563;ZNF582;ZNF586;ZNF589;ZNF652;ZNF667;ZNF680;ZNF708;ZNF713;ZNF768;ZNF816;ZNF18;ZNF41;ZNF76;ZNF85;ZSCAN16;ZSCAN22;ZSCAN31;ZSCAN4	MYNN;MZF1;OSR2;PRDM14;PRDM6;WT1;ZBTB14;ZBTB48;ZBTB49;ZFP64;ZFP28;ZIM3;ZNF121;ZNF250;ZNF257;ZNF263;ZNF274;ZNF317;ZNF320;ZNF329;ZNF331;ZNF394;ZNF449;ZNF502;ZNF528;ZNF547;ZNF549;ZNF554;ZNF586;ZNF589;ZNF667;ZNF680;ZNF708;ZNF713;ZNF768;ZNF18;ZNF85;ZSCAN16;ZSCAN22
OTX2_HUMAN.H11MO.0.A	11	0.04313725	0.4397950	16.277.037	0.8931413	0.88304850	0.9683105	Paired-related HD factors{3.1.3}	OTX{3.1.3.17}	ALX1;ALX3;ALX4;ARGFX;ARX;CRX;DMBX1;DPRX;DRGX;DUX4;DUXA;ESX1;GSC;GSC2;HESX1;ISX;LEUTX;MIXL1;NOBOX;OTP;OTX1;OTX2;PHOX2A;PHOX2B;PITX1;PITX2;PITX3;PROP1;PRRX1;PRRX2;RAX;RAX2;RHOXF1;RHOXF2;SEBOX;SHOX;SHOX2;TPRX1;UNCX;VSX1;VSX2	CRX;OTX1;OTX2
MZF1_HUMAN.H11MO.0.B	32	0.12549020	0.5952101	12.959.340	0.8916457	0.58723735	0.8310155	More than 3 adjacent zinc finger factors{2.3.3}	unclassified{2.3.3.0}	BCL6B;BCL6;CTCFL;CTCF;FEZF1;GFI1B;GFI1;GLI1;GLI2;GLI3;GLIS1;GLIS2;GLIS3;MTF1;MYNN;MZF1;OSR2;OVOL1;OVOL2;ZNF146;PLAG1;PLAGL1;PRDM14;PRDM1;PRDM6;SCRT1;SCRT2;SNAI1;SNAI2;YY1;YY2;WT1;ZNF324;ZNF354A;ZBTB14;ZBTB18;ZBTB48;ZBTB49;ZBTB7A;ZBTB7B;ZBTB6;ZFP64;ZFP28;ZFP42;ZFP82;ZFX;ZIC1;ZIC2;ZIC3;ZIC4;ZIM3;ZKSCAN1;ZKSCAN3;ZNF121;ZNF136;ZNF140;ZNF143;ZNF148;ZNF214;ZNF232;ZNF250;ZNF257;ZNF260;ZNF263;ZNF264;ZNF274;ZNF281;ZNF282;ZNF317;ZNF320;ZNF322;ZNF329;ZNF331;ZNF333;ZNF350;ZNF384;ZNF394;ZNF410;ZNF436;ZNF449;ZNF490;ZNF502;ZNF524;ZNF528;ZNF547;ZNF549;ZNF554;ZNF563;ZNF582;ZNF586;ZNF589;ZNF652;ZNF667;ZNF680;ZNF708;ZNF713;ZNF768;ZNF816;ZNF18;ZNF41;ZNF76;ZNF85;ZSCAN16;ZSCAN22;ZSCAN31;ZSCAN4	MYNN;MZF1;OSR2;PRDM14;PRDM6;WT1;ZBTB14;ZBTB48;ZBTB49;ZFP64;ZFP28;ZIM3;ZNF121;ZNF250;ZNF257;ZNF263;ZNF274;ZNF317;ZNF320;ZNF329;ZNF331;ZNF394;ZNF449;ZNF502;ZNF528;ZNF547;ZNF549;ZNF554;ZNF586;ZNF589;ZNF667;ZNF680;ZNF708;ZNF713;ZNF768;ZNF18;ZNF85;ZSCAN16;ZSCAN22
SNAI2_HUMAN.H11MO.0.A	50	0.19607843	0.6403828	12.195.467	0.8911259	0.49303174	0.7633082	More than 3 adjacent zinc finger factors{2.3.3}	Snail-like factors{2.3.3.2}	BCL6;BCL6B;CTCF;CTCFL;FEZF1;FEZF2;GFI1;GFI1B;GLI1;GLI2;GLI3;GLI4;GLIS1;GLIS2;GLIS3;HKR1;MTF1;MYNN;MZF1;OSR2;OVOL1;OVOL2;PLAG1;PLAGL1;PLAGL2;PRDM1;PRDM14;PRDM6;SCRT1;SCRT2;SNAI1;SNAI2;SNAI3;WT1;YY1;YY2;ZBTB12;ZBTB14;ZBTB18;ZBTB20;ZBTB26;ZBTB42;ZBTB45;ZBTB47;ZBTB48;ZBTB49;ZBTB6;ZBTB7A;ZBTB7B;ZBTB7C;ZFP14;ZFP2;ZFP28;ZFP30;ZFP37;ZFP42;ZFP64;ZFP69;ZFP69B;ZFP82;ZFP91;ZFX;ZIC1;ZIC2;ZIC3;ZIC4;ZIC5;ZIK1;ZIM3;ZKSCAN1;ZKSCAN2;ZKSCAN3;ZKSCAN4;ZNF121;ZNF124;ZNF133;ZNF136;ZNF138;ZNF14;ZNF140;ZNF143;ZNF146;ZNF148;ZNF155;ZNF157;ZNF160;ZNF169;ZNF175;ZNF177;ZNF18;ZNF180;ZNF181;ZNF2;ZNF20;ZNF212;ZNF213;ZNF214;ZNF221;ZNF222;ZNF223;ZNF224;ZNF225;ZNF226;ZNF227;ZNF229;ZNF230;ZNF232;ZNF233;ZNF234;ZNF235;ZNF24;ZNF25;ZNF250;ZNF257;ZNF26;ZNF260;ZNF263;ZNF264;ZNF268;ZNF274;ZNF276;ZNF28;ZNF280A;ZNF280B;ZNF280C;ZNF280D;ZNF281;ZNF282;ZNF283;ZNF284;ZNF285;ZNF286A;ZNF286B;ZNF3;ZNF30;ZNF300;ZNF302;ZNF317;ZNF32;ZNF320;ZNF322;ZNF324;ZNF324B;ZNF329;ZNF331;ZNF333;ZNF33A;ZNF33B;ZNF343;ZNF345;ZNF347;ZNF350;ZNF354A;ZNF354B;ZNF362;ZNF366;ZNF383;ZNF384;ZNF394;ZNF397;ZNF398;ZNF404;ZNF41;ZNF410;ZNF419;ZNF420;ZNF431;ZNF432;ZNF436;ZNF439;ZNF44;ZNF440;ZNF442;ZNF443;ZNF446;ZNF449;ZNF45;ZNF460;ZNF468;ZNF479;ZNF484;ZNF490;ZNF500;ZNF502;ZNF524;ZNF525;ZNF528;ZNF543;ZNF544;ZNF546;ZNF547;ZNF548;ZNF549;ZNF554;ZNF555;ZNF557;ZNF558;ZNF559;ZNF561;ZNF562;ZNF563;ZNF564;ZNF566;ZNF567;ZNF568;ZNF57;ZNF570;ZNF571;ZNF572;ZNF577;ZNF581;ZNF582;ZNF583;ZNF585A;ZNF586;ZNF589;ZNF595;ZNF599;ZNF600;ZNF605;ZNF607;ZNF611;ZNF613;ZNF614;ZNF61	SCRT1;SCRT2;SNAI1;SNAI2

Motif	Nº of probes	% of probes	lower OR	upper OR	OR	p.value	FDR	TF family	TF subfamily	TF.family.member	TF.subfamily.member
										5;ZNF616;ZNF619;ZNF620;ZNF621;ZNF625;ZNF627;ZNF649;ZNF652;ZNF653;ZNF665;ZNF667;ZNF669;ZNF670;ZNF672;ZNF679;ZNF680;ZNF683;ZNF689;ZNF692;ZNF701;ZNF705D;ZNF705E;ZNF705G;ZNF708;ZNF709;ZNF71;ZNF710;ZNF713;ZNF721;ZNF727;ZNF729;ZNF736;ZNF75A;ZNF75D;ZNF76;ZNF763;ZNF764;ZNF765;ZNF768;ZNF77;ZNF771;ZNF773;ZNF774;ZNF776;ZNF777;ZNF780A;ZNF780B;ZNF782;ZNF785;ZNF799;ZNF805;ZNF808;ZNF81;ZNF813;ZNF816;ZNF823;ZNF829;ZNF836;ZNF841;ZNF844;ZNF845;ZNF846;ZNF85;ZNF853;ZNF860;ZNF878;ZNF891;ZNF99;ZSCAN16;ZSCAN2;ZSCAN22;ZSCAN23;ZSCAN29;ZSCAN31;ZSCAN32;ZSCAN4;ZSCAN5A;ZSCAN5B;ZSCAN5C;ZSCAN9;ZXDA;ZXDB;ZXDC	
HXB3_HUMAN.H11MO.0.D	12	0.04705882	0.4531379	15.837.309	0.8896204	0.88778566	0.9683105	HOX-related factors{3.1.1}	HOX3{3.1.1.3}	CDX1;CDX2;CDX4;EVX1;EVX2;GBX1;GBX2;GSX1;GSX2;HDX;HMBOX1;HNF1A;HNF1B;HOXA1;HOXA10;HOXA11;HOXA13;HOXA2;HOXA3;HOXA4;HOXA5;HOXA6;HOXA7;HOXA9;HOXB1;HOXB13;HOXB2;HOXB3;HOXB4;HOXB5;HOXB6;HOXB7;HOXB8;HOXB9;HOXC10;HOXC11;HOXC12;HOXC13;HOXC4;HOXC5;HOXC6;HOXC8;HOXC9;HOXD1;HOXD10;HOXD11;HOXD12;HOXD13;HOXD3;HOXD4;HOXD8;HOXD9;MEOX1;MEOX2;MNX1;PDX1;POU1F1;POU2F1;POU2F2;POU2F3;POU3F1;POU3F2;POU3F3;POU3F4;POU4F1;POU4F2;POU4F3;POU5F1;POU5F2;POU6F1;POU6F2	HOXB3;HOXD3
BCL6_HUMAN.H11MO.0.A	21	0.08235294	0.5396691	13.911.866	0.8886726	0.66509232	0.8680770	More than 3 adjacent zinc finger factors{2.3.3}	BCL6 factors{2.3.3.22}	BCL6;BCL6B;CTCF;CTCFL;FEZF1;FEZF2;GFI1;GFI1B;GLI1;GLI2;GLI3;GLI4;GLIS1;GLIS2;GLIS3;HKR1;MTF1;MYNN;MZF1;OSR2;OVOL1;OVOL2;PLAG1;PLAGL1;PLAGL2;PRDM1;PRDM14;PRDM6;SCRT1;SCRT2;SNAI1;SNAI2;SNAI3;WT1;YY1;YY2;ZBTB12;ZBTB14;ZBTB18;ZBTB20;ZBTB26;ZBTB42;ZBTB45;ZBTB47;ZBTB48;ZBTB49;ZBTB6;ZBTB7A;ZBTB7B;ZBTB7C;ZFP14;ZFP2;ZFP28;ZFP30;ZFP37;ZFP42;ZFP64;ZFP69;ZFP69B;ZFP82;ZFP91;ZFX;ZIC1;ZIC2;ZIC3;ZIC4;ZIC5;ZIK1;ZIM3;ZKSCAN1;ZKSCAN2;ZKSCAN3;ZKSCAN4;ZNF121;ZNF124;ZNF133;ZNF136;ZNF138;ZNF14;ZNF140;ZNF143;ZNF146;ZNF148;ZNF155;ZNF157;ZNF160;ZNF169;ZNF175;ZNF177;ZNF18;ZNF180;ZNF181;ZNF2;ZNF20;ZNF212;ZNF213;ZNF214;ZNF221;ZNF222;ZNF223;ZNF224;ZNF225;ZNF226;ZNF227;ZNF229;ZNF230;ZNF232;ZNF233;ZNF234;ZNF235;ZNF24;ZNF25;ZNF250;ZNF257;ZNF26;ZNF260;ZNF263;ZNF264;ZNF268;ZNF274;ZNF276;ZNF28;ZNF280A;ZNF280B;ZNF280C;ZNF280D;ZNF281;ZNF282;ZNF283;ZNF284;ZNF285;ZNF286A;ZNF286B;ZNF3;ZNF30;ZNF300;ZNF302;ZNF317;ZNF32;ZNF320;ZNF322;ZNF324;ZNF324B;ZNF329;ZNF331;ZNF333;ZNF33A;ZNF33B;ZNF343;ZNF345;ZNF347;ZNF350;ZNF354A;ZNF354B;ZNF362;ZNF366;ZNF383;ZNF384;ZNF394;ZNF397;ZNF398;ZNF404;ZNF41;ZNF410;ZNF419;ZNF420;ZNF431;ZNF432;ZNF436;ZNF439;ZNF44;ZNF440;ZNF442;ZNF443;ZNF446;ZNF449;ZNF45;ZNF460;ZNF468;ZNF479;ZNF484;ZNF490;ZNF500;ZNF502;ZNF524;ZNF525;ZNF528;ZNF543;ZNF544;ZNF546;ZNF547;ZNF548;ZNF549;ZNF554;ZNF555;ZNF557;ZNF558;ZNF559;ZNF561;ZNF562;ZNF563;ZNF564;ZNF566;ZNF567;ZNF568;ZNF57;ZNF570;ZNF571;ZNF572;ZNF577;ZNF581;ZNF582;ZNF583;ZNF585A;ZNF586;ZNF589;ZNF595;ZNF599;ZNF600;ZNF605;ZNF607;ZNF611;ZNF613;ZNF614;ZNF615;ZNF616;ZNF619;ZNF620;ZNF621;ZNF625;ZNF627;ZNF649;ZNF652;ZNF653;ZNF665;ZNF667;ZNF669;ZNF670;ZNF672;ZNF679;ZNF680;ZNF683;ZNF689;ZNF692;ZNF701;ZNF705D;ZNF705E;ZNF705G;ZNF708;ZNF709;ZNF71;ZNF710;ZNF713;ZNF721;ZNF727;ZNF729;ZNF736;ZNF75A;ZNF75D;ZNF76;ZNF763;ZNF764;ZNF765;ZNF768;ZNF77;ZNF771;ZNF773;ZNF774;ZNF776;ZNF777;ZNF780A;ZNF780B;ZNF782;ZNF785;ZNF799;ZNF805;ZNF808;ZNF81;ZNF813;ZNF816;ZNF823;ZNF829;ZNF836;ZNF841;ZNF844;ZNF845;ZNF846;ZNF85;ZNF853;ZNF860;ZNF878;ZNF891;ZNF99;ZSCAN16;ZSCAN2;ZSCAN22;ZSCAN23;ZSCAN29;ZSCAN31;ZSCAN32;ZSCAN4;ZSCAN5A;ZSCAN5B;ZSCAN5C;ZSCAN9;ZXDA;ZXDB;ZXDC	BCL6B;BCL6

Motif	Nº of probes	% of probes	lower OR	upper OR	OR	p.value	FDR	TF family	TF subfamily	TF.family.member	TF.subfamily.member
ZNF85_HUMAN.H11MO.1.C	13	0.05098039	0.4658727	15.485.708	0.8880895	0.78715858	0.9294016	More than 3 adjacent zinc finger factors{2.3.3}	unclassified{2.3.3.0}	BCL6B;BCL6;CTCFL;CTCF;FEZF1;GFI1B;GFI1;GLI1;GLI2;GLI3;GLIS1;GLIS2;GLIS3;MTF1;MYNN;MZF1;OSR2;OVOL1;OVOL2;ZNF146;PLAG1;PLAGL1;PRDM14;PRDM1;PRDM6;SCRT1;SCRT2;SNAI1;SNAI2;YY1;YY2;WT1;ZNF324;ZNF354A;ZBTB14;ZBTB18;ZBTB48;ZBTB49;ZBTB7A;ZBTB7B;ZBTB6;ZFP64;ZFP28;ZFP42;ZFP82;ZFX;ZIC1;ZIC2;ZIC3;ZIC4;ZIM3;ZKSCAN1;ZKSCAN3;ZNF121;ZNF136;ZNF140;ZNF143;ZNF148;ZNF214;ZNF232;ZNF250;ZNF257;ZNF260;ZNF263;ZNF264;ZNF274;ZNF281;ZNF282;ZNF317;ZNF320;ZNF322;ZNF329;ZNF331;ZNF333;ZNF350;ZNF384;ZNF394;ZNF410;ZNF436;ZNF449;ZNF490;ZNF502;ZNF524;ZNF528;ZNF547;ZNF549;ZNF554;ZNF563;ZNF582;ZNF586;ZNF589;ZNF652;ZNF667;ZNF680;ZNF708;ZNF713;ZNF768;ZNF816;ZNF18;ZNF41;ZNF76;ZNF85;ZSCAN16;ZSCAN22;ZSCAN31;ZSCAN4	MYNN;MZF1;OSR2;PRDM14;PRDM6;WT1;ZBTB14;ZBTB48;ZBTB49;ZFP64;ZFP28;ZIM3;ZNF121;ZNF250;ZNF257;ZNF263;ZNF274;ZNF317;ZNF320;ZNF329;ZNF331;ZNF394;ZNF449;ZNF502;ZNF528;ZNF547;ZNF549;ZNF554;ZNF586;ZNF589;ZNF667;ZNF680;ZNF708;ZNF713;ZNF768;ZNF18;ZNF85;ZSCAN16;ZSCAN22
DPRX_HUMAN.H11MO.0.D	10	0.03921569	0.4191637	16.578.256	0.8855982	0.87815442	0.9683105	Paired-related HD factors{3.1.3}	DPRX{3.1.3.5}	ALX1;ALX3;ALX4;ARGFX;ARX;CRX;DMBX1;DPRX;DRGX;DUX4;DUXA;ESX1;GSC;GSC2;HESX1;ISX;LEUTX;MIXL1;NOBOX;OTP;OTX1;OTX2;PHOX2A;PHOX2B;PITX1;PITX2;PITX3;PROP1;PRRX1;PRRX2;RAX;RAX2;RHOXF1;RHOXF2;SEBOX;SHOX;SHOX2;TPRX1;UNCX;VSX1;VSX2	DPRX
SRF_HUMAN.H11MO.0.A	10	0.03921569	0.4180913	16.535.781	0.8833307	0.87823159	0.9683105	Responders to external signals (SRF/RLM1){5.1.2}	SRF{5.1.2.0.1}	SRF	SRF
MEF2D_HUMAN.H11MO.0.A	22	0.08627451	0.5427004	13.701.389	0.8832172	0.67100287	0.8693197	Regulators of differentiation{5.1.1}	MEF-2{5.1.1.1}	MEF2A;MEF2B;MEF2C;MEF2D	MEF2A;MEF2B;MEF2C;MEF2D
IRF4_HUMAN.H11MO.0.A	32	0.12549020	0.5886915	12.817.500	0.8819346	0.58742341	0.8310155	Interferon-regulatory factors{3.5.3}	IRF-4 (LSIRF, NF-EM5, MUM1, Pip){3.5.3.0.4}	IRF1;IRF2;IRF3;IRF4;IRF5;IRF6;IRF7;IRF8;IRF9	IRF4
ZN528_HUMAN.H11MO.0.C	27	0.10588235	0.5676323	13.159.523	0.8805641	0.62733722	0.8537110	More than 3 adjacent zinc finger factors{2.3.3}	unclassified{2.3.3.0}	BCL6B;BCL6;CTCFL;CTCF;FEZF1;GFI1B;GFI1;GLI1;GLI2;GLI3;GLIS1;GLIS2;GLIS3;MTF1;MYNN;MZF1;OSR2;OVOL1;OVOL2;ZNF146;PLAG1;PLAGL1;PRDM14;PRDM1;PRDM6;SCRT1;SCRT2;SNAI1;SNAI2;YY1;YY2;WT1;ZNF324;ZNF354A;ZBTB14;ZBTB18;ZBTB48;ZBTB49;ZBTB7A;ZBTB7B;ZBTB6;ZFP64;ZFP28;ZFP42;ZFP82;ZFX;ZIC1;ZIC2;ZIC3;ZIC4;ZIM3;ZKSCAN1;ZKSCAN3;ZNF121;ZNF136;ZNF140;ZNF143;ZNF148;ZNF214;ZNF232;ZNF250;ZNF257;ZNF260;ZNF263;ZNF264;ZNF274;ZNF281;ZNF282;ZNF317;ZNF320;ZNF322;ZNF329;ZNF331;ZNF333;ZNF350;ZNF384;ZNF394;ZNF410;ZNF436;ZNF449;ZNF490;ZNF502;ZNF524;ZNF528;ZNF547;ZNF549;ZNF554;ZNF563;ZNF582;ZNF586;ZNF589;ZNF652;ZNF667;ZNF680;ZNF708;ZNF713;ZNF768;ZNF816;ZNF18;ZNF41;ZNF76;ZNF85;ZSCAN16;ZSCAN22;ZSCAN31;ZSCAN4	MYNN;MZF1;OSR2;PRDM14;PRDM6;WT1;ZBTB14;ZBTB48;ZBTB49;ZFP64;ZFP28;ZIM3;ZNF121;ZNF250;ZNF257;ZNF263;ZNF274;ZNF317;ZNF320;ZNF329;ZNF331;ZNF394;ZNF449;ZNF502;ZNF528;ZNF547;ZNF549;ZNF554;ZNF586;ZNF589;ZNF667;ZNF680;ZNF708;ZNF713;ZNF768;ZNF18;ZNF85;ZSCAN16;ZSCAN22
LHX3_HUMAN.H11MO.0.C	11	0.04313725	0.4320483	15.990.482	0.8774274	0.77218806	0.9225697	HD-LIM factors{3.1.5}	Lhx-3-like factors{3.1.5.4}	ISL1;ISL2;LHX1;LHX2;LHX3;LHX4;LHX5;LHX6;LHX8;LHX9;LMX1A;LMX1B	LHX3;LHX4
TGIF2_HUMAN.H11MO.0.D	23	0.09019608	0.5450214	13.494.290	0.8773008	0.60484301	0.8401621	TALE-type homeo domain factors{3.1.4}	TGIF{3.1.4.6}	IRX1;IRX2;IRX3;IRX4;IRX5;IRX6;MEIS1;MEIS2;MEIS3;MKX;PBX1;PBX2;PBX3;PKNOX1;PKNOX2;TGIF1;TGIF2;TGIF2LX;TGIF2LY	TGIF2LX;TGIF1;TGIF2
ONEC2_HUMAN.H11MO.0.D	18	0.07058824	0.5102853	14.161.252	0.8763350	0.72779180	0.9074091	HD-CUT factors{3.1.9}	ONECUT{3.1.9.1}	CUX1;CUX2;ONECUT1;ONECUT2;ONECUT3;SATB1;SATB2	ONECUT1;ONECUT2;ONECUT3
STA5A_HUMAN.H11MO.0.A	18	0.07058824	0.5102316	14.159.766	0.8762428	0.72780146	0.9074091	STAT factors{6.2.1}	STAT5A{6.2.1.0.5}	STAT1;STAT2;STAT3;STAT4;STAT5A;STAT5B;STAT6	STAT5A
NR1H4_HUMAN.H11MO.1.B	21	0.08235294	0.5316490	13.705.738	0.8755067	0.66541309	0.8680770	Thyroid hormone receptor-related factors (NR1){2.1.2}	LXR (NR1H){2.1.2.7}	NR1D1;NR1D2;NR1H2;NR1H3;NR1H4;NR1I2;NR1I3;PPARA;PPARD;PPARG;RARA;RARB;RARG;RORA;RORB;RORC;THRA;THRB;VDR	NR1H2;NR1H3;NR1H4
SNAI1_HUMAN.H11MO.0.C	51	0.20000000	0.6305615	11.948.327	0.8750408	0.45068385	0.7307745	More than 3 adjacent zinc finger factors{2.3.3}	Snail-like factors{2.3.3.2}	BCL6;BCL6B;CTCF;CTCFL;FEZF1;FEZF2;GFI1;GFI1B;GLI1;GLI2;GLI3;GLI4;GLIS1;GLIS2;GLIS3;HKR1;MTF1;MYNN;MZF1;OSR2;OVOL1;OVOL2;PLAG1;PLAGL1;PLAGL2;PRDM1;PRDM14;PRDM6;SCRT1;SCRT2;SNAI1;SNAI2;SNAI3;WT1;YY1;YY2;ZBTB12;ZBTB14;ZBTB18;ZBTB20;ZBTB26;ZBTB	SCRT1;SCRT2;SNAI1;SNAI2

Motif	Nº of probes	% of probes	lower OR	upper OR	OR	p.value	FDR	TF family	TF subfamily	TF.family.member	TF.subfamily.member
										42;ZBTB45;ZBTB47;ZBTB48;ZBTB49;ZBTB6;ZBTB7A;ZBTB7B;ZBTB7C;ZFP14;ZFP2;ZFP28;ZFP30;ZFP37;ZFP42;ZFP64;ZFP69;ZFP69B;ZFP82;ZFP91;ZFX;ZIC1;ZIC2;ZIC3;ZIC4;ZIC5;ZIK1;ZIM3;ZKSCAN1;ZKSCAN2;ZKSCAN3;ZKSCAN4;ZNF121;ZNF124;ZNF133;ZNF136;ZNF138;ZNF14;ZNF140;ZNF143;ZNF146;ZNF148;ZNF155;ZNF157;ZNF160;ZNF169;ZNF175;ZNF177;ZNF18;ZNF180;ZNF181;ZNF2;ZNF20;ZNF212;ZNF213;ZNF214;ZNF221;ZNF222;ZNF223;ZNF224;ZNF225;ZNF226;ZNF227;ZNF229;ZNF230;ZNF232;ZNF233;ZNF234;ZNF235;ZNF24;ZNF25;ZNF250;ZNF257;ZNF26;ZNF260;ZNF263;ZNF264;ZNF268;ZNF274;ZNF276;ZNF28;ZNF280A;ZNF280B;ZNF280C;ZNF280D;ZNF281;ZNF282;ZNF283;ZNF284;ZNF285;ZNF286A;ZNF286B;ZNF3;ZNF30;ZNF300;ZNF302;ZNF317;ZNF32;ZNF320;ZNF322;ZNF324;ZNF324B;ZNF329;ZNF331;ZNF333;ZNF33A;ZNF33B;ZNF343;ZNF345;ZNF347;ZNF350;ZNF354A;ZNF354B;ZNF362;ZNF366;ZNF383;ZNF384;ZNF394;ZNF397;ZNF398;ZNF404;ZNF41;ZNF410;ZNF419;ZNF420;ZNF431;ZNF432;ZNF436;ZNF439;ZNF44;ZNF440;ZNF442;ZNF443;ZNF446;ZNF449;ZNF45;ZNF460;ZNF468;ZNF479;ZNF484;ZNF490;ZNF500;ZNF502;ZNF524;ZNF525;ZNF528;ZNF543;ZNF544;ZNF546;ZNF547;ZNF548;ZNF549;ZNF554;ZNF555;ZNF557;ZNF558;ZNF559;ZNF561;ZNF562;ZNF563;ZNF564;ZNF566;ZNF567;ZNF568;ZNF57;ZNF570;ZNF571;ZNF572;ZNF577;ZNF581;ZNF582;ZNF583;ZNF585A;ZNF586;ZNF589;ZNF595;ZNF599;ZNF600;ZNF605;ZNF607;ZNF611;ZNF613;ZNF614;ZNF615;ZNF616;ZNF619;ZNF620;ZNF621;ZNF625;ZNF627;ZNF649;ZNF652;ZNF653;ZNF665;ZNF667;ZNF669;ZNF670;ZNF672;ZNF679;ZNF680;ZNF683;ZNF689;ZNF692;ZNF701;ZNF705D;ZNF705E;ZNF705G;ZNF708;ZNF709;ZNF71;ZNF710;ZNF713;ZNF721;ZNF727;ZNF729;ZNF736;ZNF75A;ZNF75D;ZNF76;ZNF763;ZNF764;ZNF765;ZNF768;ZNF77;ZNF771;ZNF773;ZNF774;ZNF776;ZNF777;ZNF780A;ZNF780B;ZNF782;ZNF785;ZNF799;ZNF805;ZNF808;ZNF81;ZNF813;ZNF816;ZNF823;ZNF829;ZNF836;ZNF841;ZNF844;ZNF845;ZNF846;ZNF85;ZNF853;ZNF860;ZNF878;ZNF891;ZNF99;ZSCAN16;ZSCAN2;ZSCAN22;ZSCAN23;ZSCAN29;ZSCAN31;ZSCAN32;ZSCAN4;ZSCAN5A;ZSCAN5B;ZSCAN5C;ZSCAN9;ZXDA;ZXDB;ZXDC	
SRBP1_HUMAN.H11MO.0.A	41	0.16078431	0.6080798	12.226.014	0.8717363	0.46321522	0.7363689	bHLH-ZIP factors{1.2.6}	SREBP factors{1.2.6.3}	MAX;MITF;MLX;MLXIP;MLXIPL;MNT;MXD1;MXD3;MXD4;MXI1;MYC;MYCL;MYCN;REPIN1;SREBF1;SREBF2;TFAP4;TFE3;TFEB;TFEC;USF1;USF2	SREBF1;SREBF2
MAF_HUMAN.H11MO.0.A	23	0.09019608	0.5412105	13.400.304	0.8711745	0.60484435	0.8401621	Maf-related factors{1.1.3}	Large Maf factors{1.1.3.1}	MAF;MAFA;MAFB;MAFF;MAFG;MAFK;NRL	MAFA;MAFB;MAF;NRL
HXB6_HUMAN.H11MO.0.D	9	0.03529412	0.3931177	16.808.238	0.8704151	0.87276583	0.9683105	HOX-related factors{3.1.1}	HOX6-7{3.1.1.6}	CDX1;CDX2;CDX4;EVX1;EVX2;GBX1;GBX2;GSX1;GSX2;HDX;HMBOX1;HNF1A;HNF1B;HOXA1;HOXA10;HOXA11;HOXA13;HOXA2;HOXA3;HOXA4;HOXA5;HOXA6;HOXA7;HOXA9;HOXB1;HOXB13;HOXB2;HOXB3;HOXB4;HOXB5;HOXB6;HOXB7;HOXB8;HOXB9;HOXC10;HOXC11;HOXC12;HOXC13;HOXC4;HOXC5;HOXC6;HOXC8;HOXC9;HOXD1;HOXD10;HOXD11;HOXD12;HOXD13;HOXD3;HOXD4;HOXD8;HOXD9;MEOX1;MEOX2;MNX1;PDX1;POU1F1;POU2F1;POU2F2;POU2F3;POU3F1;POU3F2;POU3F3;POU3F4;POU4F1;POU4F2;POU4F3;POU5F1;POU5F2;POU6F1;POU6F2	HOXA7;HOXB6;HOXB7;HOXC6
TFCP2_HUMAN.H11MO.0.D	51	0.20000000	0.6271359	11.883.473	0.8702918	0.40791090	0.7005007	CP2-related factors{6.7.2}	CP2 (LSF, SEF){6.7.2.0.1}	TFCP2;UBP1	TFCP2
HME1_HUMAN.H11MO.0.D	10	0.03921569	0.4105088	16.235.414	0.8672957	0.76329665	0.9193105	NK-related factors{3.1.2}	EN (Engrailed-like factors){3.1.2.7}	BARHL1;BARHL2;BARX1;BARX2;BSX;DBX1;DBX2;DLX1;DLX2;DLX3;DLX4;DLX5;DLX6;EMX1;EMX2;EN1;EN2;HHEX;HLX;HMX1;HMX2;HMX3;LBX1;LBX2;MSX1;MSX2;NANOG;NKX1-1;NKX1-2;NKX2-1;NKX2-2;NKX2-3;NKX2-4;NKX2-5;NKX2-6;NKX2-8;NKX3-1;NKX3-2;NKX6-1;NKX6-2;NKX6-3;NOTO;TLX1;TLX2;TLX3;VAX1;VAX2;VENTX	EN1;EN2
MYBB_HUMAN.H11MO.0.D	11	0.04313725	0.4265912	15.788.273	0.8663386	0.77231439	0.9225697	Myb/SANT domain factors{3.5.1}	Myb-like factors{3.5.1.1}	CDC5L;DMTF1;MYB;MYBL1;MYBL2;SMARCA1;SMARCA5;SNAPC4;TRERF1;ZNF541	CDC5L;MYBL1;MYBL2;MYB

Motif	Nº of probes	% of probes	lower OR	upper OR	OR	p.value	FDR	TF family	TF subfamily	TF.family.member	TF.subfamily.member
ZIM3_HUMAN.H11MO.0.C	24	0.09411765	0.5429504	13.201.933	0.8650903	0.61177693	0.8438072	More than 3 adjacent zinc finger factors{2.3.3}	unclassified{2.3.3.0}	BCL6B;BCL6;CTCFL;CTCF;FEZF1;GFI1B;GFI1;GLI1;GLI2;GLI3;GLIS1;GLIS2;GLIS3;MTF1;MYNN;MZF1;OSR2;OVOL1;OVOL2;ZNF146;PLAG1;PLAGL1;PRDM14;PRDM1;PRDM6;SCRT1;SCRT2;SNAI1;SNAI2;YY1;YY2;WT1;ZNF324;ZNF354A;ZBTB14;ZBTB18;ZBTB48;ZBTB49;ZBTB7A;ZBTB7B;ZBTB6;ZFP64;ZFP28;ZFP42;ZFP82;ZFX;ZIC1;ZIC2;ZIC3;ZIC4;ZIM3;ZKSCAN1;ZKSCAN3;ZNF121;ZNF136;ZNF140;ZNF143;ZNF148;ZNF214;ZNF232;ZNF250;ZNF257;ZNF260;ZNF263;ZNF264;ZNF274;ZNF281;ZNF282;ZNF317;ZNF320;ZNF322;ZNF329;ZNF331;ZNF333;ZNF350;ZNF384;ZNF394;ZNF410;ZNF436;ZNF449;ZNF490;ZNF502;ZNF524;ZNF528;ZNF547;ZNF549;ZNF554;ZNF563;ZNF582;ZNF586;ZNF589;ZNF652;ZNF667;ZNF680;ZNF708;ZNF713;ZNF768;ZNF816;ZNF18;ZNF41;ZNF76;ZNF85;ZSCAN16;ZSCAN22;ZSCAN31;ZSCAN4	MYNN;MZF1;OSR2;PRDM14;PRDM6;WT1;ZBTB14;ZBTB48;ZBTB49;ZFP64;ZFP28;ZIM3;ZNF121;ZNF250;ZNF257;ZNF263;ZNF274;ZNF317;ZNF320;ZNF329;ZNF331;ZNF394;ZNF449;ZNF502;ZNF528;ZNF547;ZNF549;ZNF554;ZNF586;ZNF589;ZNF667;ZNF680;ZNF708;ZNF713;ZNF768;ZNF18;ZNF85;ZSCAN16;ZSCAN22
NR5A2_HUMAN.H11MO.0.B	24	0.09411765	0.5429065	13.200.868	0.8650205	0.61178755	0.8438072	FTZ-F1-related receptors (NR5){2.1.5}	LRH-1 (NR5A2){2.1.5.0.2}	NR5A1;NR5A2	NR5A2
FUBP1_HUMAN.H11MO.0.D	28	0.10980392	0.5607859	12.819.659	0.8631486	0.50791266	0.7769854	NA	NA	FUBP1	FUBP1
ZN554_HUMAN.H11MO.0.C	50	0.19607843	0.6200978	11.808.923	0.8628902	0.40524987	0.7005007	More than 3 adjacent zinc finger factors{2.3.3}	unclassified{2.3.3.0}	BCL6B;BCL6;CTCFL;CTCF;FEZF1;GFI1B;GFI1;GLI1;GLI2;GLI3;GLIS1;GLIS2;GLIS3;MTF1;MYNN;MZF1;OSR2;OVOL1;OVOL2;ZNF146;PLAG1;PLAGL1;PRDM14;PRDM1;PRDM6;SCRT1;SCRT2;SNAI1;SNAI2;YY1;YY2;WT1;ZNF324;ZNF354A;ZBTB14;ZBTB18;ZBTB48;ZBTB49;ZBTB7A;ZBTB7B;ZBTB6;ZFP64;ZFP28;ZFP42;ZFP82;ZFX;ZIC1;ZIC2;ZIC3;ZIC4;ZIM3;ZKSCAN1;ZKSCAN3;ZNF121;ZNF136;ZNF140;ZNF143;ZNF148;ZNF214;ZNF232;ZNF250;ZNF257;ZNF260;ZNF263;ZNF264;ZNF274;ZNF281;ZNF282;ZNF317;ZNF320;ZNF322;ZNF329;ZNF331;ZNF333;ZNF350;ZNF384;ZNF394;ZNF410;ZNF436;ZNF449;ZNF490;ZNF502;ZNF524;ZNF528;ZNF547;ZNF549;ZNF554;ZNF563;ZNF582;ZNF586;ZNF589;ZNF652;ZNF667;ZNF680;ZNF708;ZNF713;ZNF768;ZNF816;ZNF18;ZNF41;ZNF76;ZNF85;ZSCAN16;ZSCAN22;ZSCAN31;ZSCAN4	MYNN;MZF1;OSR2;PRDM14;PRDM6;WT1;ZBTB14;ZBTB48;ZBTB49;ZFP64;ZFP28;ZIM3;ZNF121;ZNF250;ZNF257;ZNF263;ZNF274;ZNF317;ZNF320;ZNF329;ZNF331;ZNF394;ZNF449;ZNF502;ZNF528;ZNF547;ZNF549;ZNF554;ZNF586;ZNF589;ZNF667;ZNF680;ZNF708;ZNF713;ZNF768;ZNF18;ZNF85;ZSCAN16;ZSCAN22
SPI1_HUMAN.H11MO.0.A	38	0.14901961	0.5937780	12.216.038	0.8620748	0.45129400	0.7307745	Ets-related factors{3.5.2}	Spi-like factors{3.5.2.5}	EHF;ELF1;ELF2;ELF3;ELF4;ELF5;ELK1;ELK3;ELK4;ERF;ERG;ETS1;ETS2;ETV1;ETV2;ETV3;ETV3L;ETV4;ETV5;ETV6;ETV7;FEV;FLI1;GABPA;SPDEF;SPI1;SPIB;SPIC	SPI1;SPIB;SPIC
CLOCK_HUMAN.H11MO.0.C	45	0.17647059	0.6100217	11.944.276	0.8618808	0.38880794	0.6862478	PAS domain factors{1.2.5}	Arnt-like factors{1.2.5.2}	AHR;AHRR;ARNT;ARNT2;ARNTL;ARNTL2;CLOCK;EPAS1;HIF1A;HIF3A;NCOA1;NCOA2;NCOA3;NPAS1;NPAS2;NPAS3;NPAS4;SIM1;SIM2;SOHLH1;SOHLH2;TCFL5	ARNT2;ARNT;ARNTL;CLOCK
MAFB_HUMAN.H11MO.0.B	23	0.09019608	0.5345290	13.234.196	0.8603671	0.60587563	0.8401621	Maf-related factors{1.1.3}	Large Maf factors{1.1.3.1}	MAF;MAFA;MAFB;MAFF;MAFG;MAFK;NRL	MAFA;MAFB;MAF;NRL
PO3F1_HUMAN.H11MO.0.C	12	0.04705882	0.4375741	15.292.972	0.8590752	0.78119210	0.9272965	POU domain factors{3.1.10}	POU3 (Oct-6-like factors){3.1.10.3}	CDX1;CDX2;CDX4;EVX1;EVX2;GBX1;GBX2;GSX1;GSX2;HDX;HMBOX1;HNF1A;HNF1B;HOXA1;HOXA10;HOXA11;HOXA13;HOXA2;HOXA3;HOXA4;HOXA5;HOXA6;HOXA7;HOXA9;HOXB1;HOXB13;HOXB2;HOXB3;HOXB4;HOXB5;HOXB6;HOXB7;HOXB8;HOXB9;HOXC10;HOXC11;HOXC12;HOXC13;HOXC4;HOXC5;HOXC6;HOXC8;HOXC9;HOXD1;HOXD10;HOXD11;HOXD12;HOXD13;HOXD3;HOXD4;HOXD8;HOXD9;MEOX1;MEOX2;MNX1;PDX1;POU1F1;POU2F1;POU2F2;POU2F3;POU3F1;POU3F2;POU3F3;POU3F4;POU4F1;POU4F2;POU4F3;POU5F1;POU5F1B;POU5F2;POU6F1;POU6F2	POU3F1;POU3F2;POU3F3;POU3F4
MEF2A_HUMAN.H11MO.0.A	23	0.09019608	0.5332366	13.202.275	0.8582915	0.53790665	0.8052933	Regulators of differentiation{5.1.1}	MEF-2{5.1.1.1}	MEF2A;MEF2B;MEF2C;MEF2D	MEF2A;MEF2B;MEF2C;MEF2D
ANDR_HUMAN.H11MO.1.A	26	0.10196078	0.5484914	12.906.700	0.8579739	0.55759429	0.8177760	Steroid hormone receptors (NR3){2.1.1}	GR-like receptors (NR3C){2.1.1.1}	AR;ESR1;ESR2;ESRRA;ESRRB;ESRRG;NR3C1;NR3C2;PGR	AR;NR3C1;NR3C2;PGR
SPDEF_HUMAN.H11MO.0.D	23	0.09019608	0.5328812	13.193.492	0.8577205	0.53789428	0.8052933	Ets-related factors{3.5.2}	SPDEF-like factors{3.5.2.7}	EHF;ELF1;ELF2;ELF3;ELF4;ELF5;ELK1;ELK3;ELK4;ERF;ERG;ETS1;ETS2;ETV1;ETV2;ETV3;ETV3L;ETV4;ETV5;ETV6;ETV7;FEV;FLI1;GABPA;SPDEF;SPI1;SPIB;SPIC	SPDEF

Motif	Nº of probes	% of probes	lower OR	upper OR	OR	p.value	FDR	TF family	TF subfamily	TF.family.member	TF.subfamily.member
NFYC_HUMAN.H11MO.0.A	10	0.03921569	0.4052635	16.027.580	0.8562003	0.76341662	0.9193105	Heteromeric CCAAT-binding factors{4.2.1}	NF-YC{4.2.1.0.3}	NFYA;NFYB;NFYC	NFYC
HMX1_HUMAN.H11MO.0.D	13	0.05098039	0.4489978	14.925.394	0.8559476	0.69004410	0.8837608	NK-related factors{3.1.2}	NK-5/HMX{3.1.2.18}	BARHL1;BARHL2;BARX1;BARX2;BSX;DBX1;DBX2;DLX1;DLX2;DLX3;DLX4;DLX5;DLX6;EMX1;EMX2;EN1;EN2;HHEX;HLX;HMX1;HMX2;HMX3;LBX1;LBX2;MSX1;MSX2;NANOG;NKX1-1;NKX1-2;NKX2-1;NKX2-2;NKX2-3;NKX2-4;NKX2-5;NKX2-6;NKX2-8;NKX3-1;NKX3-2;NKX6-1;NKX6-2;NKX6-3;NOTO;TLX1;TLX2;TLX3;VAX1;VAX2;VENTX	HMX1;HMX2;HMX3
IRF8_HUMAN.H11MO.0.B	33	0.12941176	0.5734596	12.353.907	0.8540397	0.42834709	0.7146267	Interferon-regulatory factors{3.5.3}	IRF-8 (ICSBP1){3.5.3.0.8}	IRF1;IRF2;IRF3;IRF4;IRF5;IRF6;IRF7;IRF8;IRF9	IRF8
OTX1_HUMAN.H11MO.0.D	11	0.04313725	0.4203772	15.557.502	0.8537108	0.77299618	0.9225697	Paired-related HD factors{3.1.3}	OTX{3.1.3.17}	ALX1;ALX3;ALX4;ARGFX;ARX;CRX;DMBX1;DPRX;DRGX;DUX4;DUXA;ESX1;GSC;GSC2;HESX1;ISX;LEUTX;MIXL1;NOBOX;OTP;OTX1;OTX2;PHOX2A;PHOX2B;PITX1;PITX2;PITX3;PROP1;PRRX1;PRRX2;RAX;RAX2;RHOXF1;RHOXF2;SEBOX;SHOX;SHOX2;TPRX1;UNCX;VSX1;VSX2	CRX;OTX1;OTX2
ESX1_HUMAN.H11MO.0.D	10	0.03921569	0.4036865	15.965.084	0.8528639	0.76353646	0.9193105	Paired-related HD factors{3.1.3}	ESX{3.1.3.8}	ALX1;ALX3;ALX4;ARGFX;ARX;CRX;DMBX1;DPRX;DRGX;DUX4;DUXA;ESX1;GSC;GSC2;HESX1;ISX;LEUTX;MIXL1;NOBOX;OTP;OTX1;OTX2;PHOX2A;PHOX2B;PITX1;PITX2;PITX3;PROP1;PRRX1;PRRX2;RAX;RAX2;RHOXF1;RHOXF2;SEBOX;SHOX;SHOX2;TPRX1;UNCX;VSX1;VSX2	ESX1
NR2E3_HUMAN.H11MO.0.C	14	0.05490196	0.4586248	14.584.387	0.8519575	0.69953870	0.8910393	RXR-related receptors (NR2){2.1.3}	Tailless-like receptors (NR2E){2.1.3.3}	HNF4A;HNF4G;NR2C1;NR2C2;NR2E1;NR2E3;NR2F1;NR2F2;NR2F6;RXRA;RXRB;RXRG	NR2E1;NR2E3
MAFG_HUMAN.H11MO.1.A	29	0.11372549	0.5569453	12.564.913	0.8509934	0.45792005	0.7344357	Maf-related factors{1.1.3}	Small Maf factors{1.1.3.2}	MAF;MAFA;MAFB;MAFF;MAFG;MAFK;NRL	MAFF;MAFG;MAFK
ZN282_HUMAN.H11MO.0.D	19	0.07450980	0.5030063	13.591.966	0.8507650	0.57706041	0.8240120	More than 3 adjacent zinc finger factors{2.3.3}	ZNF282-like factors{2.3.3.38}	BCL6;BCL6B;CTCF;CTCFL;FEZF1;FEZF2;GFI1;GFI1B;GLI1;GLI2;GLI3;GLI4;GLIS1;GLIS2;GLIS3;HKR1;MTF1;MYNN;MZF1;OSR2;OVOL1;OVOL2;PLAG1;PLAGL1;PLAGL2;PRDM1;PRDM14;PRDM6;SCRT1;SCRT2;SNAI1;SNAI2;SNAI3;WT1;YY1;YY2;ZBTB12;ZBTB14;ZBTB18;ZBTB20;ZBTB26;ZBTB42;ZBTB45;ZBTB47;ZBTB48;ZBTB49;ZBTB6;ZBTB7A;ZBTB7B;ZBTB7C;ZFP14;ZFP2;ZFP28;ZFP30;ZFP37;ZFP42;ZFP64;ZFP69;ZFP69B;ZFP82;ZFP91;ZFX;ZIC1;ZIC2;ZIC3;ZIC4;ZIC5;ZIK1;ZIM3;ZKSCAN1;ZKSCAN2;ZKSCAN3;ZKSCAN4;ZNF121;ZNF124;ZNF133;ZNF136;ZNF138;ZNF14;ZNF140;ZNF143;ZNF146;ZNF148;ZNF155;ZNF157;ZNF160;ZNF169;ZNF175;ZNF177;ZNF18;ZNF180;ZNF181;ZNF2;ZNF20;ZNF212;ZNF213;ZNF214;ZNF221;ZNF222;ZNF223;ZNF224;ZNF225;ZNF226;ZNF227;ZNF229;ZNF230;ZNF232;ZNF233;ZNF234;ZNF235;ZNF24;ZNF25;ZNF250;ZNF257;ZNF26;ZNF260;ZNF263;ZNF264;ZNF268;ZNF274;ZNF276;ZNF28;ZNF280A;ZNF280B;ZNF280C;ZNF280D;ZNF281;ZNF282;ZNF283;ZNF284;ZNF285;ZNF286A;ZNF286B;ZNF3;ZNF30;ZNF300;ZNF302;ZNF317;ZNF32;ZNF320;ZNF322;ZNF324;ZNF324B;ZNF329;ZNF331;ZNF333;ZNF33A;ZNF33B;ZNF343;ZNF345;ZNF347;ZNF350;ZNF354A;ZNF354B;ZNF362;ZNF366;ZNF383;ZNF384;ZNF394;ZNF397;ZNF398;ZNF404;ZNF41;ZNF410;ZNF419;ZNF420;ZNF431;ZNF432;ZNF436;ZNF439;ZNF44;ZNF440;ZNF442;ZNF443;ZNF446;ZNF449;ZNF45;ZNF460;ZNF468;ZNF479;ZNF484;ZNF490;ZNF500;ZNF502;ZNF524;ZNF525;ZNF528;ZNF543;ZNF544;ZNF546;ZNF547;ZNF548;ZNF549;ZNF554;ZNF555;ZNF557;ZNF558;ZNF559;ZNF561;ZNF562;ZNF563;ZNF564;ZNF566;ZNF567;ZNF568;ZNF57;ZNF570;ZNF571;ZNF572;ZNF577;ZNF581;ZNF582;ZNF583;ZNF585A;ZNF586;ZNF589;ZNF595;ZNF599;ZNF600;ZNF605;ZNF607;ZNF611;ZNF613;ZNF614;ZNF615;ZNF616;ZNF619;ZNF620;ZNF621;ZNF625;ZNF627;ZNF649;ZNF652;ZNF653;ZNF665;ZNF667;ZNF669;ZNF670;ZNF672;ZNF679;ZNF680;ZNF683;ZNF689;ZNF692;ZNF701;ZNF705D;ZNF705E;ZNF705G;ZNF708;ZNF709;ZNF71;ZNF710;ZNF713;ZNF721;ZNF727;ZNF729;ZNF736;ZNF75A;ZNF75D;ZNF76;ZNF763;ZNF764;ZNF765;ZNF768;ZNF77;ZNF771;ZNF773;ZNF7	ZNF282

Motif	Nº of probes	% of probes	lower OR	upper OR	OR	p.value	FDR	TF family	TF subfamily	TF.family.member	TF.subfamily.member
										74;ZNF776;ZNF777;ZNF780A;ZNF780B;ZNF782;ZNF785;ZNF799;ZNF805;ZNF808;ZNF81;ZNF813;ZNF816;ZNF823;ZNF829;ZNF836;ZNF841;ZNF844;ZNF845;ZNF846;ZNF85;ZNF853;ZNF860;ZNF878;ZNF891;ZNF99;ZSCAN16;ZSCAN2;ZSCAN22;ZSCAN23;ZSCAN29;ZSCAN31;ZSCAN32;ZSCAN4;ZSCAN5A;ZSCAN5B;ZSCAN5C;ZSCAN9;ZXDA;ZXDB;ZXDC	
PPARD_HUMAN.H11MO.0.D	24	0.09411765	0.5328918	12.956.819	0.8490269	0.54545211	0.8104030	Thyroid hormone receptor-related factors (NR1){2.1.2}	PPAR (NR1C){2.1.2.5}	NR1D1;NR1D2;NR1H2;NR1H3;NR1H4;NR1I2;NR1I3;PPARA;PPARD;PPARG;RARA;RARB;RARG;RORA;RORB;RORC;THRA;THRB;VDR	PPARA;PPARD;PPARG
BC11A_HUMAN.H11MO.0.A	44	0.17254902	0.5965461	11.753.304	0.8457250	0.34512564	0.6520406	Factors with multiple dispersed zinc fingers{2.3.4}	BCL11{2.3.4.15}	BCL11A;BCL11B;BNC1;BNC2;E4F1;HIC1;HIC2;HINFP;HIVEP1;HIVEP2;HIVEP3;IKZF1;IKZF2;IKZF3;IKZF4;IKZF5;INSM1;INSM2;MAZ;MECOM;PATZ1;PRDM16;PRDM4;REST;RLF;RREB1;SALL1;SALL2;SALL3;SALL4;VEZF1;ZBTB1;ZBTB17;ZBTB2;ZBTB25;ZBTB4;ZFAT;ZNF134;ZNF211;ZNF217;ZNF219;ZNF248;ZNF256;ZNF292;ZNF296;ZNF319;ZNF334;ZNF335;ZNF341;ZNF37A;ZNF382;ZNF417;ZNF418;ZNF423;ZNF467;ZNF510;ZNF512;ZNF512B;ZNF516;ZNF518A;ZNF518B;ZNF521;ZNF526;ZNF532;ZNF536;ZNF552;ZNF574;ZNF587;ZNF587B;ZNF592;ZNF639;ZNF654;ZNF658;ZNF671;ZNF687;ZNF711;ZNF717;ZNF770;ZNF772;ZNF784;ZNF786;ZNF792;ZNF8;ZNF814	BCL11A
ZN317_HUMAN.H11MO.0.C	14	0.05490196	0.4549798	14.468.365	0.8451828	0.70034993	0.8910393	More than 3 adjacent zinc finger factors{2.3.3}	unclassified{2.3.3.0}	BCL6B;BCL6;CTCFL;CTCF;FEZF1;GFI1B;GFI1;GLI1;GLI2;GLI3;GLIS1;GLIS2;GLIS3;MTF1;MYNN;MZF1;OSR2;OVOL1;OVOL2;ZNF146;PLAG1;PLAGL1;PRDM14;PRDM1;PRDM6;SCRT1;SCRT2;SNAI1;SNAI2;YY1;YY2;WT1;ZNF324;ZNF354A;ZBTB14;ZBTB18;ZBTB48;ZBTB49;ZBTB7A;ZBTB7B;ZBTB6;ZFP64;ZFP28;ZFP42;ZFP82;ZFX;ZIC1;ZIC2;ZIC3;ZIC4;ZIM3;ZKSCAN1;ZKSCAN3;ZNF121;ZNF136;ZNF140;ZNF143;ZNF148;ZNF214;ZNF232;ZNF250;ZNF257;ZNF260;ZNF263;ZNF264;ZNF274;ZNF281;ZNF282;ZNF317;ZNF320;ZNF322;ZNF329;ZNF331;ZNF333;ZNF350;ZNF384;ZNF394;ZNF410;ZNF436;ZNF449;ZNF490;ZNF502;ZNF524;ZNF528;ZNF547;ZNF549;ZNF554;ZNF563;ZNF582;ZNF586;ZNF589;ZNF652;ZNF667;ZNF680;ZNF708;ZNF713;ZNF768;ZNF816;ZNF18;ZNF41;ZNF76;ZNF85;ZSCAN16;ZSCAN22;ZSCAN31;ZSCAN4	MYNN;MZF1;OSR2;PRDM14;PRDM6;WT1;ZBTB14;ZBTB48;ZBTB49;ZFP64;ZFP28;ZIM3;ZNF121;ZNF250;ZNF257;ZNF263;ZNF274;ZNF317;ZNF320;ZNF329;ZNF331;ZNF394;ZNF449;ZNF502;ZNF528;ZNF547;ZNF549;ZNF554;ZNF586;ZNF589;ZNF667;ZNF680;ZNF708;ZNF713;ZNF768;ZNF18;ZNF85;ZSCAN16;ZSCAN22
AP2A_HUMAN.H11MO.0.A	43	0.16862745	0.5934622	11.768.569	0.8443527	0.34146408	0.6520406	AP-2{1.3.1}	AP-2alpha{1.3.1.0.1}	TFAP2A;TFAP2B;TFAP2C;TFAP2D	TFAP2A
RARA_HUMAN.H11MO.2.A	28	0.10980392	0.5485216	12.539.444	0.8443170	0.45211339	0.7307745	Thyroid hormone receptor-related factors (NR1){2.1.2}	Retinoic acid receptors (NR1B){2.1.2.1}	NR1D1;NR1D2;NR1H2;NR1H3;NR1H4;NR1I2;NR1I3;PPARA;PPARD;PPARG;RARA;RARB;RARG;RORA;RORB;RORC;THRA;THRB;VDR	RARA;RARB;RARG
PO5F1_HUMAN.H11MO.1.A	16	0.06274510	0.4741451	13.989.846	0.8433929	0.63004094	0.8537110	POU domain factors{3.1.10}	POU5 (Oct-3/4-like factors){3.1.10.5}	CDX1;CDX2;CDX4;EVX1;EVX2;GBX1;GBX2;GSX1;GSX2;HDX;HMBOX1;HNF1A;HNF1B;HOXA1;HOXA10;HOXA11;HOXA13;HOXA2;HOXA3;HOXA4;HOXA5;HOXA6;HOXA7;HOXA9;HOXB1;HOXB13;HOXB2;HOXB3;HOXB4;HOXB5;HOXB6;HOXB7;HOXB8;HOXB9;HOXC10;HOXC11;HOXC12;HOXC13;HOXC4;HOXC5;HOXC6;HOXC8;HOXC9;HOXD1;HOXD10;HOXD11;HOXD12;HOXD13;HOXD3;HOXD4;HOXD8;HOXD9;MEOX1;MEOX2;MNX1;PDX1;POU1F1;POU2F1;POU2F2;POU2F3;POU3F1;POU3F2;POU3F3;POU3F4;POU4F1;POU4F2;POU4F3;POU5F1;POU5F1B;POU5F2;POU6F1;POU6F2	POU5F1B;POU5F1
SOX13_HUMAN.H11MO.0.D	11	0.04313725	0.4150636	15.360.787	0.8429113	0.66966766	0.8693197	SOX-related factors{4.1.1}	Group D{4.1.1.4}	BBX;CIC;HBP1;SOX1;SOX10;SOX11;SOX12;SOX13;SOX14;SOX15;SOX17;SOX18;SOX2;SOX21;SOX3;SOX30;SOX4;SOX5;SOX6;SOX7;SOX8;SOX9;SRY	SOX13;SOX5
TBX3_HUMAN.H11MO.0.C	42	0.16470588	0.5900430	11.779.988	0.8426062	0.33770784	0.6520406	TBX2-related factors{6.5.4}	TBX3{6.5.4.0.2}	TBX2;TBX3;TBX4;TBX5	TBX3
GATA1_HUMAN.H11MO.1.A	14	0.05490196	0.4534673	14.419.863	0.8423717	0.61073564	0.8438072	GATA-type zinc fingers{2.2.1}	Two zinc-finger GATA factors{2.2.1.1}	GATA1;GATA2;GATA3;GATA4;GATA5;GATA6;GATAD2A;GATAD2B;TRPS1;ZGLP1	GATA1;GATA2;GATA3;GATA4;GATA5;GATA6

Motif	Nº of probes	% of probes	lower OR	upper OR	OR	p.value	FDR	TF family	TF subfamily	TF.family.member	TF.subfamily.member
PAX7_HUMAN.H11MO.0.D	7	0.02745098	0.3347961	17.623.444	0.8419303	0.85868638	0.9683105	Paired plus homeo domain{3.2.1}	PAX-3/7{3.2.1.1}	PAX3;PAX4;PAX6;PAX7	PAX3;PAX7
OZF_HUMAN.H11MO.0.C	13	0.05098039	0.4401438	14.630.144	0.8390210	0.69133030	0.8839397	More than 3 adjacent zinc finger factors{2.3.3}	ZNF146-like factors{2.3.3.55}	BCL6;BCL6B;CTCF;CTCFL;FEZF1;FEZF2;GFI1;GFI1B;GLI1;GLI2;GLI3;GLI4;GLIS1;GLIS2;GLIS3;HKR1;MTF1;MYNN;MZF1;OSR2;OVOL1;OVOL2;PLAG1;PLAGL1;PLAGL2;PRDM1;PRDM14;PRDM6;SCRT1;SCRT2;SNAI1;SNAI2;SNAI3;WT1;YY1;YY2;ZBTB12;ZBTB14;ZBTB18;ZBTB20;ZBTB26;ZBTB42;ZBTB45;ZBTB47;ZBTB48;ZBTB49;ZBTB6;ZBTB7A;ZBTB7B;ZBTB7C;ZFP14;ZFP2;ZFP28;ZFP30;ZFP37;ZFP42;ZFP64;ZFP69;ZFP69B;ZFP82;ZFP91;ZFX;ZIC1;ZIC2;ZIC3;ZIC4;ZIC5;ZIK1;ZIM3;ZKSCAN1;ZKSCAN2;ZKSCAN3;ZKSCAN4;ZNF121;ZNF124;ZNF133;ZNF136;ZNF138;ZNF14;ZNF140;ZNF143;ZNF146;ZNF148;ZNF155;ZNF157;ZNF160;ZNF169;ZNF175;ZNF177;ZNF18;ZNF180;ZNF181;ZNF2;ZNF20;ZNF212;ZNF213;ZNF214;ZNF221;ZNF222;ZNF223;ZNF224;ZNF225;ZNF226;ZNF227;ZNF229;ZNF230;ZNF232;ZNF233;ZNF234;ZNF235;ZNF24;ZNF25;ZNF250;ZNF257;ZNF26;ZNF260;ZNF263;ZNF264;ZNF268;ZNF274;ZNF276;ZNF28;ZNF280A;ZNF280B;ZNF280C;ZNF280D;ZNF281;ZNF282;ZNF283;ZNF284;ZNF285;ZNF286A;ZNF286B;ZNF3;ZNF30;ZNF300;ZNF302;ZNF317;ZNF32;ZNF320;ZNF322;ZNF324;ZNF324B;ZNF329;ZNF331;ZNF333;ZNF33A;ZNF33B;ZNF343;ZNF345;ZNF347;ZNF350;ZNF354A;ZNF354B;ZNF362;ZNF366;ZNF383;ZNF384;ZNF394;ZNF397;ZNF398;ZNF404;ZNF41;ZNF410;ZNF419;ZNF420;ZNF431;ZNF432;ZNF436;ZNF439;ZNF44;ZNF440;ZNF442;ZNF443;ZNF446;ZNF449;ZNF45;ZNF460;ZNF468;ZNF479;ZNF484;ZNF490;ZNF500;ZNF502;ZNF524;ZNF525;ZNF528;ZNF543;ZNF544;ZNF546;ZNF547;ZNF548;ZNF549;ZNF554;ZNF555;ZNF557;ZNF558;ZNF559;ZNF561;ZNF562;ZNF563;ZNF564;ZNF566;ZNF567;ZNF568;ZNF57;ZNF570;ZNF571;ZNF572;ZNF577;ZNF581;ZNF582;ZNF583;ZNF585A;ZNF586;ZNF589;ZNF595;ZNF599;ZNF600;ZNF605;ZNF607;ZNF611;ZNF613;ZNF614;ZNF615;ZNF616;ZNF619;ZNF620;ZNF621;ZNF625;ZNF627;ZNF649;ZNF652;ZNF653;ZNF665;ZNF667;ZNF669;ZNF670;ZNF672;ZNF679;ZNF680;ZNF683;ZNF689;ZNF692;ZNF701;ZNF705D;ZNF705E;ZNF705G;ZNF708;ZNF709;ZNF71;ZNF710;ZNF713;ZNF721;ZNF727;ZNF729;ZNF736;ZNF75A;ZNF75D;ZNF76;ZNF763;ZNF764;ZNF765;ZNF768;ZNF77;ZNF771;ZNF773;ZNF774;ZNF776;ZNF777;ZNF780A;ZNF780B;ZNF782;ZNF785;ZNF799;ZNF805;ZNF808;ZNF81;ZNF813;ZNF816;ZNF823;ZNF829;ZNF836;ZNF841;ZNF844;ZNF845;ZNF846;ZNF85;ZNF853;ZNF860;ZNF878;ZNF891;ZNF99;ZSCAN16;ZSCAN2;ZSCAN22;ZSCAN23;ZSCAN29;ZSCAN31;ZSCAN32;ZSCAN4;ZSCAN5A;ZSCAN5B;ZSCAN5C;ZSCAN9;ZXDA;ZXDB;ZXDC	ZNF146;ZNF260
HIC1_HUMAN.H11MO.0.C	29	0.11372549	0.5482304	12.368.198	0.8376656	0.40651297	0.7005007	Factors with multiple dispersed zinc fingers {2.3.4}	Hypermethylated in Cancer proteins{2.3.4.17}	BCL11A;BCL11B;BNC1;BNC2;E4F1;HIC1;HIC2;HINFP;HIVEP1;HIVEP2;HIVEP3;IKZF1;IKZF2;IKZF3;IKZF4;IKZF5;INSM1;INSM2;MAZ;MECOM;PATZ1;PRDM16;PRDM4;REST;RLF;RREB1;SALL1;SALL2;SALL3;SALL4;VEZF1;ZBTB1;ZBTB17;ZBTB2;ZBTB25;ZBTB4;ZFAT;ZNF134;ZNF211;ZNF217;ZNF219;ZNF248;ZNF256;ZNF292;ZNF296;ZNF319;ZNF334;ZNF335;ZNF341;ZNF37A;ZNF382;ZNF417;ZNF418;ZNF423;ZNF467;ZNF510;ZNF512;ZNF512B;ZNF516;ZNF518A;ZNF518B;ZNF521;ZNF526;ZNF532;ZNF536;ZNF552;ZNF574;ZNF587;ZNF587B;ZNF592;ZNF639;ZNF654;ZNF658;ZNF671;ZNF687;ZNF711;ZNF717;ZNF770;ZNF772;ZNF784;ZNF786;ZNF792;ZNF8;ZNF814	HIC1;HIC2
VAX2_HUMAN.H11MO.0.D	9	0.03529412	0.3780023	16.161.956	0.8369707	0.75359261	0.9179869	NK-related factors{3.1.2}	VAX{3.1.2.22}	BARHL1;BARHL2;BARX1;BARX2;BSX;DBX1;DBX2;DLX1;DLX2;DLX3;DLX4;DLX5;DLX6;EMX1;EMX2;EN1;EN2;HHEX;HLX;HMX1;HMX2;HMX3;LBX1;LBX2;MSX1;MSX2;NANOG;NKX1-1;NKX1-2;NKX2-1;NKX2-2;NKX2-3;NKX2-4;NKX2-5;NKX2-6;NKX2-8;NKX3-1;NKX3-2;NKX6-1;NKX6-2;NKX6-3;NOTO;TLX1;TLX2;TLX3;VAX1;VAX2;VENTX	VAX1;VAX2

Motif	Nº of probes	% of probes	lower OR	upper OR	OR	p.value	FDR	TF family	TF subfamily	TF.family.member	TF.subfamily.member
PRGR_HUMAN.H11MO.1.A	33	0.12941176	0.5616108	12.098.644	0.8363857	0.38119089	0.6838499	Steroid hormone receptors (NR3){2.1.1}	GR-like receptors (NR3C){2.1.1.1}	AR;ESR1;ESR2;ESRRA;ESRRB;ESRRG;NR3C1;NR3C2;PGR	AR;NR3C1;NR3C2;PGR
ZNF8_HUMAN.H11MO.0.C	12	0.04705882	0.4260002	14.887.580	0.8363031	0.68098635	0.8759174	Factors with multiple dispersed zinc fingers {2.3.4}	unclassified{2.3.4.0}	BCL11A;E4F1;MECOM;HIC1;HIC2;HINFP;IKZF1;INSM1;MAZ;PATZ1;PRDM4;REST;RREB1;SALL4;VEZF1;ZBTB17;ZBTB4;HIVEP1;HIVEP2;ZNF134;ZNF219;ZNF335;ZNF341;ZNF382;ZNF418;ZNF423;ZNF467;ZNF770;ZNF784;ZNF8	E4F1;PRDM4;REST;RREB1;ZBTB17;ZBTB4;ZNF335;ZNF341;ZNF467;ZNF770;ZNF784;ZNF8
RUNX2_HUMAN.H11MO.0.A	30	0.11764706	0.5508686	12.273.424	0.8358618	0.41242226	0.7034902	Runt-related factors{6.4.1}	Runx2 (PEBP2alphaA, CBF-alpha1){6.4.1.0.1}	RUNX1;RUNX2;RUNX3	RUNX2
ETV6_HUMAN.H11MO.0.D	34	0.13333333	0.5621551	11.988.633	0.8325223	0.34297310	0.6520406	Ets-related factors{3.5.2}	ETV6-like factors{3.5.2.6}	EHF;ELF1;ELF2;ELF3;ELF4;ELF5;ELK1;ELK3;ELK4;ERF;ERG;ETS1;ETS2;ETV1;ETV2;ETV3;ETV3L;ETV4;ETV5;ETV6;ETV7;FEV;FLI1;GABPA;SPDEF;SPI1;SPIB;SPIC	ETV6;ETV7
NKX28_HUMAN.H11MO.0.C	8	0.03137255	0.3548672	16.656.798	0.8315981	0.74159531	0.9126911	NK-related factors{3.1.2}	NK-2.2{3.1.2.15}	BARHL1;BARHL2;BARX1;BARX2;BSX;DBX1;DBX2;DLX1;DLX2;DLX3;DLX4;DLX5;DLX6;EMX1;EMX2;EN1;EN2;HHEX;HLX;HMX1;HMX2;HMX3;LBX1;LBX2;MSX1;MSX2;NANOG;NKX1-1;NKX1-2;NKX2-1;NKX2-2;NKX2-3;NKX2-4;NKX2-5;NKX2-6;NKX2-8;NKX3-1;NKX3-2;NKX6-1;NKX6-2;NKX6-3;NOTO;TLX1;TLX2;TLX3;VAX1;VAX2;VENTX	NKX2-2;NKX2-8
BHE40_HUMAN.H11MO.0.A	50	0.19607843	0.5967266	11.362.925	0.8303332	0.26176863	0.5664041	Hairy-related factors{1.2.4}	Hairy-like factors{1.2.4.1}	BHLHE40;BHLHE41;HELT;HES1;HES2;HES3;HES4;HES5;HES6;HES7;HEY1;HEY2;HEYL	BHLHE40;BHLHE41;HES1;HES5;HES7;HEY1;HEY2
LBX2_HUMAN.H11MO.0.D	7	0.02745098	0.3298225	17.362.654	0.8294761	0.85937937	0.9683105	NK-related factors{3.1.2}	LBX{3.1.2.10}	BARHL1;BARHL2;BARX1;BARX2;BSX;DBX1;DBX2;DLX1;DLX2;DLX3;DLX4;DLX5;DLX6;EMX1;EMX2;EN1;EN2;HHEX;HLX;HMX1;HMX2;HMX3;LBX1;LBX2;MSX1;MSX2;NANOG;NKX1-1;NKX1-2;NKX2-1;NKX2-2;NKX2-3;NKX2-4;NKX2-5;NKX2-6;NKX2-8;NKX3-1;NKX3-2;NKX6-1;NKX6-2;NKX6-3;NOTO;TLX1;TLX2;TLX3;VAX1;VAX2;VENTX	LBX2
RARB_HUMAN.H11MO.0.D	36	0.14117647	0.5653760	11.832.096	0.8286361	0.35300523	0.6616975	Thyroid hormone receptor-related factors (NR1){2.1.2}	Retinoic acid receptors (NR1B){2.1.2.1}	NR1D1;NR1D2;NR1H2;NR1H3;NR1H4;NR1I2;NR1I3;PPARA;PPARD;PPARG;RARA;RARB;RARG;RORA;RORB;RORC;THRA;THRB;VDR	RARA;RARB;RARG
BRAC_HUMAN.H11MO.0.A	18	0.07058824	0.4824055	13.387.349	0.8284339	0.49860395	0.7688473	Brachyury-related factors{6.5.1}	T (Brachyury){6.5.1.0.1}	T;TBX19	T
HMX2_HUMAN.H11MO.0.D	9	0.03529412	0.3738302	15.983.254	0.8277283	0.75424706	0.9179869	NK-related factors{3.1.2}	NK-5/HMX{3.1.2.18}	BARHL1;BARHL2;BARX1;BARX2;BSX;DBX1;DBX2;DLX1;DLX2;DLX3;DLX4;DLX5;DLX6;EMX1;EMX2;EN1;EN2;HHEX;HLX;HMX1;HMX2;HMX3;LBX1;LBX2;MSX1;MSX2;NANOG;NKX1-1;NKX1-2;NKX2-1;NKX2-2;NKX2-3;NKX2-4;NKX2-5;NKX2-6;NKX2-8;NKX3-1;NKX3-2;NKX6-1;NKX6-2;NKX6-3;NOTO;TLX1;TLX2;TLX3;VAX1;VAX2;VENTX	HMX1;HMX2;HMX3
ZN589_HUMAN.H11MO.0.D	27	0.10588235	0.5333591	12.364.203	0.8273736	0.39427436	0.6908762	More than 3 adjacent zinc finger factors{2.3.3}	unclassified{2.3.3.0}	BCL6B;BCL6;CTCFL;CTCF;FEZF1;GFI1B;GFI1;GLI1;GLI2;GLI3;GLIS1;GLIS2;GLIS3;MTF1;MYNN;MZF1;OSR2;OVOL1;OVOL2;ZNF146;PLAG1;PLAGL1;PRDM14;PRDM1;PRDM6;SCRT1;SCRT2;SNAI1;SNAI2;YY1;YY2;WT1;ZNF324;ZNF354A;ZBTB14;ZBTB18;ZBTB48;ZBTB49;ZBTB7A;ZBTB7B;ZBTB6;ZFP64;ZFP28;ZFP42;ZFP82;ZFX;ZIC1;ZIC2;ZIC3;ZIC4;ZIM3;ZKSCAN1;ZKSCAN3;ZNF121;ZNF136;ZNF140;ZNF143;ZNF148;ZNF214;ZNF232;ZNF250;ZNF257;ZNF260;ZNF263;ZNF264;ZNF274;ZNF281;ZNF282;ZNF317;ZNF320;ZNF322;ZNF329;ZNF331;ZNF333;ZNF350;ZNF384;ZNF394;ZNF410;ZNF436;ZNF449;ZNF490;ZNF502;ZNF524;ZNF528;ZNF547;ZNF549;ZNF554;ZNF563;ZNF582;ZNF586;ZNF589;ZNF652;ZNF667;ZNF680;ZNF708;ZNF713;ZNF768;ZNF816;ZNF18;ZNF41;ZNF76;ZNF85;ZSCAN16;ZSCAN22;ZSCAN31;ZSCAN4	MYNN;MZF1;OSR2;PRDM14;PRDM6;WT1;ZBTB14;ZBTB48;ZBTB49;ZFP64;ZFP28;ZIM3;ZNF121;ZNF250;ZNF257;ZNF263;ZNF274;ZNF317;ZNF320;ZNF329;ZNF331;ZNF394;ZNF449;ZNF502;ZNF528;ZNF547;ZNF549;ZNF554;ZNF586;ZNF589;ZNF667;ZNF680;ZNF708;ZNF713;ZNF768;ZNF18;ZNF85;ZSCAN16;ZSCAN22
BARX2_HUMAN.H11MO.0.D	9	0.03529412	0.3733326	15.961.940	0.8266260	0.75434592	0.9179869	NK-related factors{3.1.2}	BARX{3.1.2.2}	BARHL1;BARHL2;BARX1;BARX2;BSX;DBX1;DBX2;DLX1;DLX2;DLX3;DLX4;DLX5;DLX6;EMX1;EMX2;EN1;EN2;HHEX;HLX;HMX1;HMX2;HMX3;LBX1;LBX2;MSX1;MSX2;NANOG;NKX1-1;NKX1-2;NKX2-1;NKX2-2;NKX2-3;NKX2-4;NKX2-5;NKX2-6;NKX2-8;NKX3-1;NKX3-2;NKX6-1;NKX6-2;NKX6-3;NOTO;TLX1;TLX2;TLX3;VAX1;VAX2;VENTX	BARX1;BARX2
PO6F1_HUMAN.H11MO.0.D	10	0.03921569	0.3901451	15.427.866	0.8241999	0.65774114	0.8634820	POU domain factors{3.1.10}	POU6 (Brn-5-like factors){3.1.10.6}	CDX1;CDX2;CDX4;EVX1;EVX2;GBX1;GBX2;GSX1;GSX2;HDX;HMBOX1;HNF1A;HNF1B;HOXA1;HOXA10;HOXA11;HOXA13;HOXA2;HOXA3;HOXA4;HOXA5;HOXA6;HOXA7;H	POU6F1;POU6F2

Motif	Nº of probes	% of probes	lower OR	upper OR	OR	p.value	FDR	TF family	TF subfamily	TF.family.member	TF.subfamily.member
										OXA9;HOXB1;HOXB13;HOXB2;HOXB3;HOXB4;HOXB5;HOXB6;HOXB7;HOXB8;HOXB9;HOXC10;HOXC11;HOXC12;HOXC13;HOXC4;HOXC5;HOXC6;HOXC8;HOXC9;HOXD1;HOXD10;HOXD11;HOXD12;HOXD13;HOXD3;HOXD4;HOXD8;HOXD9;MEOX1;MEOX2;MNX1;PDX1;POU1F1;POU2F1;POU2F2;POU2F3;POU3F1;POU3F2;POU3F3;POU3F4;POU4F1;POU4F2;POU4F3;POU5F1;POU5F1B;POU5F2;POU6F1;POU6F2	
PPARA_HUMAN.H11MO.0.B	22	0.08627451	0.5062820	12.780.720	0.8238803	0.46914424	0.7412094	Thyroid hormone receptor-related factors (NR1){2.1.2}	PPAR (NR1C){2.1.2.5}	NR1D1;NR1D2;NR1H2;NR1H3;NR1H4;NR1I2;NR1I3;PPARA;PPARD;PPARG;RARA;RARB;RARG;RORA;RORB;RORC;THRA;THRB;VDR	PPARA;PPARD;PPARG
HXB2_HUMAN.H11MO.0.D	15	0.05882353	0.4534661	13.861.924	0.8232859	0.54088251	0.8081791	HOX-related factors{3.1.1}	HOX2{3.1.1.2}	CDX1;CDX2;CDX4;EVX1;EVX2;GBX1;GBX2;GSX1;GSX2;HDX;HMBOX1;HNF1A;HNF1B;HOXA1;HOXA10;HOXA11;HOXA13;HOXA2;HOXA3;HOXA4;HOXA5;HOXA6;HOXA7;HOXA9;HOXB1;HOXB13;HOXB2;HOXB3;HOXB4;HOXB5;HOXB6;HOXB7;HOXB8;HOXB9;HOXC10;HOXC11;HOXC12;HOXC13;HOXC4;HOXC5;HOXC6;HOXC8;HOXC9;HOXD1;HOXD10;HOXD11;HOXD12;HOXD13;HOXD3;HOXD4;HOXD8;HOXD9;MEOX1;MEOX2;MNX1;PDX1;POU1F1;POU2F1;POU2F2;POU2F3;POU3F1;POU3F2;POU3F3;POU3F4;POU4F1;POU4F2;POU4F3;POU5F1;POU5F2;POU6F1;POU6F2	HOXA2;HOXB2
ID4_HUMAN.H11MO.0.D	43	0.16862745	0.5781689	11.465.413	0.8226059	0.27053277	0.5714541	HLH domain only{1.2.8}	Id4{1.2.8.0.4}	ID4	ID4
IRX3_HUMAN.H11MO.0.D	12	0.04705882	0.4188109	14.636.088	0.8221807	0.58867999	0.8312679	TALE-type homeo domain factors{3.1.4}	IRX (Iroquois){3.1.4.1}	IRX1;IRX2;IRX3;IRX4;IRX5;IRX6;MEIS1;MEIS2;MEIS3;MKX;PBX1;PBX2;PBX3;PKNOX1;PKNOX2;TGIF1;TGIF2;TGIF2LX;TGIF2LY	IRX2;IRX3
NFYA_HUMAN.H11MO.0.A	9	0.03529412	0.3710040	15.862.184	0.8214673	0.75486862	0.9179869	Heteromeric CCAAT-binding factors{4.2.1}	NF-YA (CP1A, CBF-B){4.2.1.0.1}	NFYA;NFYB;NFYC	NFYA
RORA_HUMAN.H11MO.0.C	29	0.11372549	0.5375052	12.126.122	0.8212616	0.35920317	0.6666074	Thyroid hormone receptor-related factors (NR1){2.1.2}	ROR (NR1F){2.1.2.6}	NR1D1;NR1D2;NR1H2;NR1H3;NR1H4;NR1I2;NR1I3;PPARA;PPARD;PPARG;RARA;RARB;RARG;RORA;RORB;RORC;THRA;THRB;VDR	RORA;RORC
E4F1_HUMAN.H11MO.0.D	32	0.12549020	0.5476587	11.924.354	0.8204246	0.33265255	0.6509521	Factors with multiple dispersed zinc fingers{2.3.4}	unclassified{2.3.4.0}	BCL11A;E4F1;MECOM;HIC1;HIC2;HINFP;IKZF1;INSM1;MAZ;PATZ1;PRDM4;REST;RREB1;SALL4;VEZF1;ZBTB17;ZBTB4;HIVEP1;HIVEP2;ZNF134;ZNF219;ZNF335;ZNF341;ZNF382;ZNF418;ZNF423;ZNF467;ZNF770;ZNF784;ZNF8	E4F1;PRDM4;REST;RREB1;ZBTB17;ZBTB4;ZNF335;ZNF341;ZNF467;ZNF770;ZNF784;ZNF8
RARA_HUMAN.H11MO.0.A	41	0.16078431	0.5721727	11.503.987	0.8202665	0.26319680	0.5668289	Thyroid hormone receptor-related factors (NR1){2.1.2}	Retinoic acid receptors (NR1B){2.1.2.1}	NR1D1;NR1D2;NR1H2;NR1H3;NR1H4;NR1I2;NR1I3;PPARA;PPARD;PPARG;RARA;RARB;RARG;RORA;RORB;RORC;THRA;THRB;VDR	RARA;RARB;RARG
ZNF41_HUMAN.H11MO.1.C	21	0.08235294	0.4980119	12.838.537	0.8201058	0.46104655	0.7344357	More than 3 adjacent zinc finger factors{2.3.3}	ZFN81-like factors{2.3.3.68}	BCL6;BCL6B;CTCF;CTCFL;FEZF1;FEZF2;GFI1;GFI1B;GLI1;GLI2;GLI3;GLI4;GLIS1;GLIS2;GLIS3;HKR1;MTF1;MYNN;MZF1;OSR2;OVOL1;OVOL2;PLAG1;PLAGL1;PLAGL2;PRDM1;PRDM14;PRDM6;SCRT1;SCRT2;SNAI1;SNAI2;SNAI3;WT1;YY1;YY2;ZBTB12;ZBTB14;ZBTB18;ZBTB20;ZBTB26;ZBTB42;ZBTB45;ZBTB47;ZBTB48;ZBTB49;ZBTB6;ZBTB7A;ZBTB7B;ZBTB7C;ZFP14;ZFP2;ZFP28;ZFP30;ZFP37;ZFP42;ZFP64;ZFP69;ZFP69B;ZFP82;ZFP91;ZFX;ZIC1;ZIC2;ZIC3;ZIC4;ZIC5;ZIK1;ZIM3;ZKSCAN1;ZKSCAN2;ZKSCAN3;ZKSCAN4;ZNF121;ZNF124;ZNF133;ZNF136;ZNF138;ZNF14;ZNF140;ZNF143;ZNF146;ZNF148;ZNF155;ZNF157;ZNF160;ZNF169;ZNF175;ZNF177;ZNF18;ZNF180;ZNF181;ZNF2;ZNF20;ZNF212;ZNF213;ZNF214;ZNF221;ZNF222;ZNF223;ZNF224;ZNF225;ZNF226;ZNF227;ZNF229;ZNF230;ZNF232;ZNF233;ZNF234;ZNF235;ZNF24;ZNF25;ZNF250;ZNF257;ZNF26;ZNF260;ZNF263;ZNF264;ZNF268;ZNF274;ZNF276;ZNF28;ZNF280A;ZNF280B;ZNF280C;ZNF280D;ZNF281;ZNF282;ZNF283;ZNF284;ZNF285;ZNF286A;ZNF286B;ZNF3;ZNF30;ZNF300;ZNF302;ZNF317;ZNF32;ZNF320;ZNF322;ZNF324;ZNF324B;ZNF329;ZNF331;ZNF333;ZNF33A;ZNF33B;ZNF343;ZNF345;ZNF347;ZNF350;ZNF354A;ZNF354B;ZNF362;ZNF366;ZNF383;ZNF384;ZNF394;ZNF397	ZNF41

Motif	Nº of probes	% of probes	lower OR	upper OR	OR	p.value	FDR	TF family	TF subfamily	TF.family.member	TF.subfamily.member
										;ZNF398;ZNF404;ZNF41;ZNF410;ZNF419;ZNF420;ZNF431;ZNF432;ZNF436;ZNF439;ZNF44;ZNF440;ZNF442;ZNF443;ZNF446;ZNF449;ZNF45;ZNF460;ZNF468;ZNF479;ZNF484;ZNF490;ZNF500;ZNF502;ZNF524;ZNF525;ZNF528;ZNF543;ZNF544;ZNF546;ZNF547;ZNF548;ZNF549;ZNF554;ZNF555;ZNF557;ZNF558;ZNF559;ZNF561;ZNF562;ZNF563;ZNF564;ZNF566;ZNF567;ZNF568;ZNF57;ZNF570;ZNF571;ZNF572;ZNF577;ZNF581;ZNF582;ZNF583;ZNF585A;ZNF586;ZNF589;ZNF595;ZNF599;ZNF600;ZNF605;ZNF607;ZNF611;ZNF613;ZNF614;ZNF615;ZNF616;ZNF619;ZNF620;ZNF621;ZNF625;ZNF627;ZNF649;ZNF652;ZNF653;ZNF665;ZNF667;ZNF669;ZNF670;ZNF672;ZNF679;ZNF680;ZNF683;ZNF689;ZNF692;ZNF701;ZNF705D;ZNF705E;ZNF705G;ZNF708;ZNF709;ZNF71;ZNF710;ZNF713;ZNF721;ZNF727;ZNF729;ZNF736;ZNF75A;ZNF75D;ZNF76;ZNF763;ZNF764;ZNF765;ZNF768;ZNF77;ZNF771;ZNF773;ZNF774;ZNF776;ZNF777;ZNF780A;ZNF780B;ZNF782;ZNF785;ZNF799;ZNF805;ZNF808;ZNF81;ZNF813;ZNF816;ZNF823;ZNF829;ZNF836;ZNF841;ZNF844;ZNF845;ZNF846;ZNF85;ZNF853;ZNF860;ZNF878;ZNF891;ZNF99;ZSCAN16;ZSCAN2;ZSCAN22;ZSCAN23;ZSCAN29;ZSCAN31;ZSCAN32;ZSCAN4;ZSCAN5A;ZSCAN5B;ZSCAN5C;ZSCAN9;ZXDA;ZXDB;ZXDC	
DLX2_HUMAN.H11MO.0.D	10	0.03921569	0.3879566	15.343.529	0.8196938	0.65789508	0.8634820	NK-related factors{3.1.2}	DLX{3.1.2.5}	BARHL1;BARHL2;BARX1;BARX2;BSX;DBX1;DBX2;DLX1;DLX2;DLX3;DLX4;DLX5;DLX6;EMX1;EMX2;EN1;EN2;HHEX;HLX;HMX1;HMX2;HMX3;LBX1;LBX2;MSX1;MSX2;NANOG;NKX1-1;NKX1-2;NKX2-1;NKX2-2;NKX2-3;NKX2-4;NKX2-5;NKX2-6;NKX2-8;NKX3-1;NKX3-2;NKX6-1;NKX6-2;NKX6-3;NOTO;TLX1;TLX2;TLX3;VAX1;VAX2;VENTX	DLX1;DLX2;DLX3;DLX4;DLX5;DLX6
HXC13_HUMAN.H11MO.0.D	11	0.04313725	0.4033628	14.927.219	0.8191202	0.67095782	0.8693197	HOX-related factors{3.1.1}	HOX9-13{3.1.1.8}	CDX1;CDX2;CDX4;EVX1;EVX2;GBX1;GBX2;GSX1;GSX2;HDX;HMBOX1;HNF1A;HNF1B;HOXA1;HOXA10;HOXA11;HOXA13;HOXA2;HOXA3;HOXA4;HOXA5;HOXA6;HOXA7;HOXA9;HOXB1;HOXB13;HOXB2;HOXB3;HOXB4;HOXB5;HOXB6;HOXB7;HOXB8;HOXB9;HOXC10;HOXC11;HOXC12;HOXC13;HOXC4;HOXC5;HOXC6;HOXC8;HOXC9;HOXD1;HOXD10;HOXD11;HOXD12;HOXD13;HOXD3;HOXD4;HOXD8;HOXD9;MEOX1;MEOX2;MNX1;PDX1;POU1F1;POU2F1;POU2F2;POU2F3;POU3F1;POU3F2;POU3F3;POU3F4;POU4F1;POU4F2;POU4F3;POU5F1;POU5F2;POU6F1;POU6F2	HOXA10;HOXA11;HOXA13;HOXA9;HOXB13;HOXC10;HOXC11;HOXC12;HOXC13;HOXC9;HOXD10;HOXD11;HOXD12;HOXD13;HOXD9
USF1_HUMAN.H11MO.0.A	36	0.14117647	0.5583404	11.685.322	0.8183229	0.31298245	0.6296813	bHLH-ZIP factors{1.2.6}	USF factors{1.2.6.2}	MAX;MITF;MLX;MLXIP;MLXIPL;MNT;MXD1;MXD3;MXD4;MXI1;MYC;MYCL;MYCN;REPIN1;SREBF1;SREBF2;TFAP4;TFE3;TFEB;TFEC;USF1;USF2	USF1;USF2
BACH1_HUMAN.H11MO.0.A	52	0.20392157	0.5907371	11.139.765	0.8176096	0.21131228	0.5123326	Jun-related factors{1.1.1}	NF-E2-like factors{1.1.1.2}	ATF2;ATF7;BACH1;BACH2;CREB5;JUN;JUNB;JUND;NFE2;NFE2L1;NFE2L2;NFE2L3	BACH1;BACH2;NFE2L1;NFE2L2;NFE2
DUX4_HUMAN.H11MO.0.A	8	0.03137255	0.3488963	16.376.084	0.8175920	0.74222736	0.9126911	Paired-related HD factors{3.1.3}	DUX{3.1.3.7}	ALX1;ALX3;ALX4;ARGFX;ARX;CRX;DMBX1;DPRX;DRGX;DUX4;DUXA;ESX1;GSC;GSC2;HESX1;ISX;LEUTX;MIXL1;NOBOX;OTP;OTX1;OTX2;PHOX2A;PHOX2B;PITX1;PITX2;PITX3;PROP1;PRRX1;PRRX2;RAX;RAX2;RHOXF1;RHOXF2;SEBOX;SHOX;SHOX2;TPRX1;UNCX;VSX1;VSX2	DUX4;DUXA
PAX4_HUMAN.H11MO.0.D	10	0.03921569	0.3850879	15.228.977	0.8135739	0.65828573	0.8634820	Paired plus homeo domain{3.2.1}	PAX-4/6{3.2.1.2}	PAX3;PAX4;PAX6;PAX7	PAX4;PAX6
LHX2_HUMAN.H11MO.0.A	10	0.03921569	0.3846310	15.210.901	0.8126083	0.65836679	0.8634820	HD-LIM factors{3.1.5}	Lhx-2-like factors{3.1.5.3}	ISL1;ISL2;LHX1;LHX2;LHX3;LHX4;LHX5;LHX6;LHX8;LHX9;LMX1A;LMX1B	LHX2;LHX9
GCM1_HUMAN.H11MO.0.D	22	0.08627451	0.4991986	12.601.448	0.8123005	0.41121531	0.7029867	GCM factors{7.2.1}	GCMa (GCM1){7.2.1.0.1}	GCM1;GCM2	GCM1
ZN329_HUMAN.H11MO.0.C	28	0.10980392	0.5274448	12.057.363	0.8118384	0.35359194	0.6616975	More than 3 adjacent zinc finger factors{2.3.3}	unclassified{2.3.3.0}	BCL6B;BCL6;CTCFL;CTCF;FEZF1;GFI1B;GFI1;GLI1;GLI2;GLI3;GLIS1;GLIS2;GLIS3;MTF1;MYNN;MZF1;OSR2;OVOL1;OVOL2;ZNF146;PLAG1;PLAGL1;PRDM14;PRDM1;PRDM6;SCRT1;SCRT2;SNAI1;SNAI2;YY1;YY2;WT1;ZNF324;ZNF354A;ZBTB14;ZBTB18;ZBTB48;ZBTB49;ZBTB7A;ZBTB7B;ZBTB6;ZFP64;ZFP28;ZFP42;ZFP82;ZFX;ZIC1;ZIC2;ZIC3;ZIC4;ZIM3;ZKSCAN1;ZKSCAN3;ZNF121;ZNF136;ZNF140;ZNF143;Z	MYNN;MZF1;OSR2;PRDM14;PRDM6;WT1;ZBTB14;ZBTB48;ZB

Motif	Nº of probes	% of probes	lower OR	upper OR	OR	p.value	FDR	TF family	TF subfamily	TF.family.member	TF.subfamily.member
										NF148;ZNF214;ZNF232;ZNF250;ZNF257;ZNF260;ZNF263;ZNF264;ZNF274;ZNF281;ZNF282;ZNF317;ZNF320;ZNF322;ZNF329;ZNF331;ZNF333;ZNF350;ZNF384;ZNF394;ZNF410;ZNF436;ZNF449;ZNF490;ZNF502;ZNF524;ZNF528;ZNF547;ZNF549;ZNF554;ZNF563;ZNF582;ZNF586;ZNF589;ZNF652;ZNF667;ZNF680;ZNF708;ZNF713;ZNF768;ZNF816;ZNF18;ZNF41;ZNF76;ZNF85;ZSCAN16;ZSCAN22;ZSCAN31;ZSCAN4	
SHOX2_HUMAN.H11MO.0.D	10	0.03921569	0.3839802	15.185.149	0.8112327	0.658491530	0.86348197	Paired-related HD factors{3.1.3}	SHOX{3.1.3.25}	ALX1;ALX3;ALX4;ARGFX;ARX;CRX;DMBX1;DPRX;DRGX;DUX4;DUXA;ESX1;GSC;GSC2;HESX1;ISX;LEUTX;MIXL1;NOBOX;OTP;OTX1;OTX2;PHOX2A;PHOX2B;PITX1;PITX2;PITX3;PROP1;PRRX1;PRRX2;RAX;RAX2;RHOXF1;RHOXF2;SEBOX;SHOX;SHOX2;TPRX1;UNCX;VSX1;VSX2	SHOX2;SHOX
NR1I3_HUMAN.H11MO.1.D	10	0.03921569	0.3837853	15.177.439	0.8108209	0.658530995	0.86348197	Thyroid hormone receptor-related factors (NR1){2.1.2}	Vitamin D receptor (NR1I){2.1.2.4}	NR1D1;NR1D2;NR1H2;NR1H3;NR1H4;NR1I2;NR1I3;PPARA;PPARD;PPARG;RARA;RARB;RARG;RORA;RORB;RORC;THRA;THRB;VDR	NR1I2;NR1I3;VDR
NR1I2_HUMAN.H11MO.1.D	10	0.03921569	0.3837853	15.177.439	0.8108209	0.658530995	0.86348197	Thyroid hormone receptor-related factors (NR1){2.1.2}	Vitamin D receptor (NR1I){2.1.2.4}	NR1D1;NR1D2;NR1H2;NR1H3;NR1H4;NR1I2;NR1I3;PPARA;PPARD;PPARG;RARA;RARB;RARG;RORA;RORB;RORC;THRA;THRB;VDR	NR1I2;NR1I3;VDR
ATF6A_HUMAN.H11MO.0.B	61	0.23921569	0.5944541	10.808.114	0.8064750	0.162603734	0.44456553	CREB-related factors{1.1.7}	ATF-6 factors{1.1.7.3}	ATF1;ATF6;ATF6B;CREB1;CREB3;CREB3L1;CREB3L2;CREB3L3;CREB3L4;CREBL2;CREBZF;CREM	ATF6
HXD4_HUMAN.H11MO.0.D	6	0.02352941	0.2923397	17.782.858	0.8047814	0.850564768	0.96808328	HOX-related factors{3.1.1}	HOX4{3.1.1.4}	CDX1;CDX2;CDX4;EVX1;EVX2;GBX1;GBX2;GSX1;GSX2;HDX;HMBOX1;HNF1A;HNF1B;HOXA1;HOXA10;HOXA11;HOXA13;HOXA2;HOXA3;HOXA4;HOXA5;HOXA6;HOXA7;HOXA9;HOXB1;HOXB13;HOXB2;HOXB3;HOXB4;HOXB5;HOXB6;HOXB7;HOXB8;HOXB9;HOXC10;HOXC11;HOXC12;HOXC13;HOXC4;HOXC5;HOXC6;HOXC8;HOXC9;HOXD1;HOXD10;HOXD11;HOXD12;HOXD13;HOXD3;HOXD4;HOXD8;HOXD9;MEOX1;MEOX2;MNX1;PDX1;POU1F1;POU2F1;POU2F2;POU2F3;POU3F1;POU3F2;POU3F3;POU3F4;POU4F1;POU4F2;POU4F3;POU5F1;POU5F2;POU6F1;POU6F2	HOXB4;HOXD4
NR2F6_HUMAN.H11MO.0.D	17	0.06666667	0.4603583	13.153.910	0.8039093	0.490737140	0.76128438	RXR-related receptors (NR2){2.1.3}	COUP-like receptors (NR2F){2.1.3.5}	HNF4A;HNF4G;NR2C1;NR2C2;NR2E1;NR2E3;NR2F1;NR2F2;NR2F6;RXRA;RXRB;RXRG	NR2F1;NR2F2;NR2F6
TFEB_HUMAN.H11MO.0.C	33	0.12941176	0.5377716	11.584.703	0.8008715	0.262002260	0.56640407	bHLH-ZIP factors{1.2.6}	TFE3-like factors{1.2.6.1}	MAX;MITF;MLX;MLXIP;MLXIPL;MNT;MXD1;MXD3;MXD4;MXI1;MYC;MYCL;MYCN;REPIN1;SREBF1;SREBF2;TFAP4;TFE3;TFEB;TFEC;USF1;USF2	MITF;TFE3;TFEB
GATA4_HUMAN.H11MO.0.A	12	0.04705882	0.4076161	14.243.877	0.8001820	0.590413387	0.83219145	GATA-type zinc fingers{2.2.1}	Two zinc-finger GATA factors{2.2.1.1}	GATA1;GATA2;GATA3;GATA4;GATA5;GATA6;GATAD2A;GATAD2B;TRPS1;ZGLP1	GATA1;GATA2;GATA3;GATA4;GATA5;GATA6
NR6A1_HUMAN.H11MO.0.B	14	0.05490196	0.4302182	13.680.413	0.7991713	0.531427611	0.79869530	GCNF-related receptors (NR6){2.1.6}	GCNF (NR6A1){2.1.6.0.1}	NR6A1	NR6A1
ISL2_HUMAN.H11MO.0.D	9	0.03529412	0.3606275	15.418.262	0.7984806	0.644827429	0.86310939	HD-LIM factors{3.1.5}	ISL{3.1.5.1}	ISL1;ISL2;LHX1;LHX2;LHX3;LHX4;LHX5;LHX6;LHX8;LHX9;LMX1A;LMX1B	ISL1;ISL2
MLX_HUMAN.H11MO.0.D	108	0.42352941	0.6165931	10.305.076	0.7981846	0.078992568	0.28545111	bHLH-ZIP factors{1.2.6}	Mondo-like factors{1.2.6.6}	MAX;MITF;MLX;MLXIP;MLXIPL;MNT;MXD1;MXD3;MXD4;MXI1;MYC;MYCL;MYCN;REPIN1;SREBF1;SREBF2;TFAP4;TFE3;TFEB;TFEC;USF1;USF2	MLXIPL;MLX
NFAC2_HUMAN.H11MO.0.B	13	0.05098039	0.4182707	13.902.411	0.7973024	0.518598032	0.78708481	NFAT-related factors{6.1.3}	NFATc2 (NFATp, NFAT1){6.1.3.0.2}	NFAT5;NFATC1;NFATC2;NFATC3;NFATC4	NFATC2
OLIG3_HUMAN.H11MO.0.D	9	0.03529412	0.3594354	15.367.268	0.7958397	0.645064851	0.86310939	Tal-related factors{1.2.3}	Neurogenin / Atonal-like factors{1.2.3.4}	ATOH1;ATOH7;ATOH8;BHLHA15;BHLHA9;BHLHE22;BHLHE23;FERD3L;FIGLA;HAND1;HAND2;LYL1;MESP1;MESP2;MSC;MSGN1;NEUROD1;NEUROD2;NEUROD4;NEUROD6;NEUROG1;NEUROG2;NEUROG3;NHLH1;NHLH2;OLIG1;OLIG2;OLIG3;PTF1A;SCX;TAL1;TAL2;TCF15;TCF21;TCF23;TWIST1;TWIST2	ATOH1;BHLHA15;BHLHE22;BHLHE23;NEUROD1;NEUROD2;NEUROG2;OLIG1;OLIG2;OLIG3
HSF2_HUMAN.H11MO.0.A	15	0.05882353	0.4376345	13.376.790	0.7944910	0.468744770	0.74120944	HSF factors{3.4.1}	HSF2 (HSTF2){3.4.1.0.2}	HSF1;HSF2;HSF4;HSF5;HSFX1;HSFY1;HSFY1; HSFY2	HSF2

Motif	Nº of probes	% of probes	lower OR	upper OR	OR	p.value	FDR	TF family	TF subfamily	TF.family.member	TF.subfamily.member
ZN816_HUMAN.H11MO.1.C	30	0.11764706	0.5225276	11.641.164	0.7928147	0.246767296	0.55958113	More than 3 adjacent zinc finger factors{2.3.3}	ZNF816A-like factors{2.3.3.73}	BCL6;BCL6B;CTCF;CTCFL;FEZF1;FEZF2;GFI1;GFI1B;GLI1;GLI2;GLI3;GLI4;GLIS1;GLIS2;GLIS3;HKR1;MTF1;MYNN;MZF1;OSR2;OVOL1;OVOL2;PLAG1;PLAGL1;PLAGL2;PRDM1;PRDM14;PRDM6;SCRT1;SCRT2;SNAI1;SNAI2;SNAI3;WT1;YY1;YY2;ZBTB12;ZBTB14;ZBTB18;ZBTB20;ZBTB26;ZBTB42;ZBTB45;ZBTB47;ZBTB48;ZBTB49;ZBTB6;ZBTB7A;ZBTB7B;ZBTB7C;ZFP14;ZFP2;ZFP28;ZFP30;ZFP37;ZFP42;ZFP64;ZFP69;ZFP69B;ZFP82;ZFP91;ZFX;ZIC1;ZIC2;ZIC3;ZIC4;ZIC5;ZIK1;ZIM3;ZKSCAN1;ZKSCAN2;ZKSCAN3;ZKSCAN4;ZNF121;ZNF124;ZNF133;ZNF136;ZNF138;ZNF14;ZNF140;ZNF143;ZNF146;ZNF148;ZNF155;ZNF157;ZNF160;ZNF169;ZNF175;ZNF177;ZNF18;ZNF180;ZNF181;ZNF2;ZNF20;ZNF212;ZNF213;ZNF214;ZNF221;ZNF222;ZNF223;ZNF224;ZNF225;ZNF226;ZNF227;ZNF229;ZNF230;ZNF232;ZNF233;ZNF234;ZNF235;ZNF24;ZNF25;ZNF250;ZNF257;ZNF26;ZNF260;ZNF263;ZNF264;ZNF268;ZNF274;ZNF276;ZNF28;ZNF280A;ZNF280B;ZNF280C;ZNF280D;ZNF281;ZNF282;ZNF283;ZNF284;ZNF285;ZNF286A;ZNF286B;ZNF3;ZNF30;ZNF300;ZNF302;ZNF317;ZNF32;ZNF320;ZNF322;ZNF324;ZNF324B;ZNF329;ZNF331;ZNF333;ZNF33A;ZNF33B;ZNF343;ZNF345;ZNF347;ZNF350;ZNF354A;ZNF354B;ZNF362;ZNF366;ZNF383;ZNF384;ZNF394;ZNF397;ZNF398;ZNF404;ZNF41;ZNF410;ZNF419;ZNF420;ZNF431;ZNF432;ZNF436;ZNF439;ZNF44;ZNF440;ZNF442;ZNF443;ZNF446;ZNF449;ZNF45;ZNF460;ZNF468;ZNF479;ZNF484;ZNF490;ZNF500;ZNF502;ZNF524;ZNF525;ZNF528;ZNF543;ZNF544;ZNF546;ZNF547;ZNF548;ZNF549;ZNF554;ZNF555;ZNF557;ZNF558;ZNF559;ZNF561;ZNF562;ZNF563;ZNF564;ZNF566;ZNF567;ZNF568;ZNF57;ZNF570;ZNF571;ZNF572;ZNF577;ZNF581;ZNF582;ZNF583;ZNF585A;ZNF586;ZNF589;ZNF595;ZNF599;ZNF600;ZNF605;ZNF607;ZNF611;ZNF613;ZNF614;ZNF615;ZNF616;ZNF619;ZNF620;ZNF621;ZNF625;ZNF627;ZNF649;ZNF652;ZNF653;ZNF665;ZNF667;ZNF669;ZNF670;ZNF672;ZNF679;ZNF680;ZNF683;ZNF689;ZNF692;ZNF701;ZNF705D;ZNF705E;ZNF705G;ZNF708;ZNF709;ZNF71;ZNF710;ZNF713;ZNF721;ZNF727;ZNF729;ZNF736;ZNF75A;ZNF75D;ZNF76;ZNF763;ZNF764;ZNF765;ZNF768;ZNF77;ZNF771;ZNF773;ZNF774;ZNF776;ZNF777;ZNF780A;ZNF780B;ZNF782;ZNF785;ZNF799;ZNF805;ZNF808;ZNF81;ZNF813;ZNF816;ZNF823;ZNF829;ZNF836;ZNF841;ZNF844;ZNF845;ZNF846;ZNF85;ZNF853;ZNF860;ZNF878;ZNF891;ZNF99;ZSCAN16;ZSCAN2;ZSCAN22;ZSCAN23;ZSCAN29;ZSCAN31;ZSCAN32;ZSCAN4;ZSCAN5A;ZSCAN5B;ZSCAN5C;ZSCAN9;ZXDA;ZXDB;ZXDC	ZNF816
MAF_HUMAN.H11MO.1.B	19	0.07450980	0.4679033	12.642.281	0.7913358	0.385829729	0.68384993	Maf-related factors{1.1.3}	Large Maf factors{1.1.3.1}	MAF;MAFA;MAFB;MAFF;MAFG;MAFK;NRL	MAFA;MAFB;MAF;NRL
SPIB_HUMAN.H11MO.0.A	32	0.12549020	0.5282295	11.500.511	0.7912857	0.257575758	0.56481632	Ets-related factors{3.5.2}	Spi-like factors{3.5.2.5}	EHF;ELF1;ELF2;ELF3;ELF4;ELF5;ELK1;ELK3;ELK4;ERF;ERG;ETS1;ETS2;ETV1;ETV2;ETV3;ETV3L;ETV4;ETV5;ETV6;ETV7;FEV;FLI1;GABPA;SPDEF;SPI1;SPIB;SPIC	SPI1;SPIB;SPIC
MXI1_HUMAN.H11MO.1.A	60	0.23529412	0.5819715	10.618.146	0.7910633	0.124355725	0.38660590	bHLH-ZIP factors{1.2.6}	Mad-like factors{1.2.6.7}	MAX;MITF;MLX;MLXIP;MLXIPL;MNT;MXD1;MXD3;MXD4;MXI1;MYC;MYCL;MYCN;REPIN1;SREBF1;SREBF2;TFAP4;TFE3;TFEB;TFEC;USF1;USF2	MXI1
HXA1_HUMAN.H11MO.0.C	11	0.04313725	0.3895007	14.415.035	0.7910259	0.577203448	0.82401200	HOX-related factors{3.1.1}	HOX1{3.1.1.1}	CDX1;CDX2;CDX4;EVX1;EVX2;GBX1;GBX2;GSX1;GSX2;HDX;HMBOX1;HNF1A;HNF1B;HOXA1;HOXA10;HOXA11;HOXA13;HOXA2;HOXA3;HOXA4;HOXA5;HOXA6;HOXA7;HOXA9;HOXB1;HOXB13;HOXB2;HOXB3;HOXB4;HOXB5;HOXB6;HOXB7;HOXB8;HOXB9;HOXC10;HOXC11;HOXC12;HOXC13;HOXC4;HOXC5;HOXC6;HOXC8;HOXC9;HOXD1;HOXD10;HOXD11;HOXD12;HOXD13;HOXD3;HOXD4;HOXD8;HOXD9;MEOX1;MEOX2;MNX1;PDX1;POU1F1;POU2F1;POU2F2;POU2F3;POU3F1;POU3F2;POU3F3;POU3F4;POU4F1;POU4F2;POU4F3;POU5F1;POU5F2;POU6F1;POU6F2	HOXA1;HOXB1
ATF2_HUMAN.H11MO.0.B	22	0.08627451	0.4860217	12.268.703	0.7909520	0.359674040	0.66660742	Jun-related factors{1.1.1}	ATF-2-like factors{1.1.1.3}	ATF2;ATF7;BACH1;BACH2;CREB5;JUN;JUNB;JUND;NFE2;NFE2L1;NFE2L2;NFE2L3	ATF2;ATF7;CREB5

Motif	Nº of probes	% of probes	lower OR	upper OR	OR	p.value	FDR	TF family	TF subfamily	TF.family.member	TF.subfamily.member
ZSCA4_HUMAN.H11MO.0.D	34	0.13333333	0.5340580	11.388.525	0.7908981	0.234104427	0.54695307	More than 3 adjacent zinc finger factors{2.3.3}	ZSCAN5-like zinc finger factors{2.3.3.5}	BCL6;BCL6B;CTCF;CTCFL;FEZF1;FEZF2;GFI1;GFI1B;GLI1;GLI2;GLI3;GLI4;GLIS1;GLIS2;GLIS3;HKR1;MTF1;MYNN;MZF1;OSR2;OVOL1;OVOL2;PLAG1;PLAGL1;PLAGL2;PRDM1;PRDM14;PRDM6;SCRT1;SCRT2;SNAI1;SNAI2;SNAI3;WT1;YY1;YY2;ZBTB12;ZBTB14;ZBTB18;ZBTB20;ZBTB26;ZBTB42;ZBTB45;ZBTB47;ZBTB48;ZBTB49;ZBTB6;ZBTB7A;ZBTB7B;ZBTB7C;ZFP14;ZFP2;ZFP28;ZFP30;ZFP37;ZFP42;ZFP64;ZFP69;ZFP69B;ZFP82;ZFP91;ZFX;ZIC1;ZIC2;ZIC3;ZIC4;ZIC5;ZIK1;ZIM3;ZKSCAN1;ZKSCAN2;ZKSCAN3;ZKSCAN4;ZNF121;ZNF124;ZNF133;ZNF136;ZNF138;ZNF14;ZNF140;ZNF143;ZNF146;ZNF148;ZNF155;ZNF157;ZNF160;ZNF169;ZNF175;ZNF177;ZNF18;ZNF180;ZNF181;ZNF2;ZNF20;ZNF212;ZNF213;ZNF214;ZNF221;ZNF222;ZNF223;ZNF224;ZNF225;ZNF226;ZNF227;ZNF229;ZNF230;ZNF232;ZNF233;ZNF234;ZNF235;ZNF24;ZNF25;ZNF250;ZNF257;ZNF26;ZNF260;ZNF263;ZNF264;ZNF268;ZNF274;ZNF276;ZNF28;ZNF280A;ZNF280B;ZNF280C;ZNF280D;ZNF281;ZNF282;ZNF283;ZNF284;ZNF285;ZNF286A;ZNF286B;ZNF3;ZNF30;ZNF300;ZNF302;ZNF317;ZNF32;ZNF320;ZNF322;ZNF324;ZNF324B;ZNF329;ZNF331;ZNF333;ZNF33A;ZNF33B;ZNF343;ZNF345;ZNF347;ZNF350;ZNF354A;ZNF354B;ZNF362;ZNF366;ZNF383;ZNF384;ZNF394;ZNF397;ZNF398;ZNF404;ZNF41;ZNF410;ZNF419;ZNF420;ZNF431;ZNF432;ZNF436;ZNF439;ZNF44;ZNF440;ZNF442;ZNF443;ZNF446;ZNF449;ZNF45;ZNF460;ZNF468;ZNF479;ZNF484;ZNF490;ZNF500;ZNF502;ZNF524;ZNF525;ZNF528;ZNF543;ZNF544;ZNF546;ZNF547;ZNF548;ZNF549;ZNF554;ZNF555;ZNF557;ZNF558;ZNF559;ZNF561;ZNF562;ZNF563;ZNF564;ZNF566;ZNF567;ZNF568;ZNF57;ZNF570;ZNF571;ZNF572;ZNF577;ZNF581;ZNF582;ZNF583;ZNF585A;ZNF586;ZNF589;ZNF595;ZNF599;ZNF600;ZNF605;ZNF607;ZNF611;ZNF613;ZNF614;ZNF615;ZNF616;ZNF619;ZNF620;ZNF621;ZNF625;ZNF627;ZNF649;ZNF652;ZNF653;ZNF665;ZNF667;ZNF669;ZNF670;ZNF672;ZNF679;ZNF680;ZNF683;ZNF689;ZNF692;ZNF701;ZNF705D;ZNF705E;ZNF705G;ZNF708;ZNF709;ZNF71;ZNF710;ZNF713;ZNF721;ZNF727;ZNF729;ZNF736;ZNF75A;ZNF75D;ZNF76;ZNF763;ZNF764;ZNF765;ZNF768;ZNF77;ZNF771;ZNF773;ZNF774;ZNF776;ZNF777;ZNF780A;ZNF780B;ZNF782;ZNF785;ZNF799;ZNF805;ZNF808;ZNF81;ZNF813;ZNF816;ZNF823;ZNF829;ZNF836;ZNF841;ZNF844;ZNF845;ZNF846;ZNF85;ZNF853;ZNF860;ZNF878;ZNF891;ZNF99;ZSCAN16;ZSCAN2;ZSCAN22;ZSCAN23;ZSCAN29;ZSCAN31;ZSCAN32;ZSCAN4;ZSCAN5A;ZSCAN5B;ZSCAN5C;ZSCAN9;ZXDA;ZXDB;ZXDC	ZSCAN4
ZIC3_HUMAN.H11MO.0.B	63	0.24705882	0.5849140	10.562.748	0.7906041	0.113231716	0.36075063	More than 3 adjacent zinc finger factors{2.3.3}	GLI-like factors{2.3.3.1}	BCL6;BCL6B;CTCF;CTCFL;FEZF1;FEZF2;GFI1;GFI1B;GLI1;GLI2;GLI3;GLI4;GLIS1;GLIS2;GLIS3;HKR1;MTF1;MYNN;MZF1;OSR2;OVOL1;OVOL2;PLAG1;PLAGL1;PLAGL2;PRDM1;PRDM14;PRDM6;SCRT1;SCRT2;SNAI1;SNAI2;SNAI3;WT1;YY1;YY2;ZBTB12;ZBTB14;ZBTB18;ZBTB20;ZBTB26;ZBTB42;ZBTB45;ZBTB47;ZBTB48;ZBTB49;ZBTB6;ZBTB7A;ZBTB7B;ZBTB7C;ZFP14;ZFP2;ZFP28;ZFP30;ZFP37;ZFP42;ZFP64;ZFP69;ZFP69B;ZFP82;ZFP91;ZFX;ZIC1;ZIC2;ZIC3;ZIC4;ZIC5;ZIK1;ZIM3;ZKSCAN1;ZKSCAN2;ZKSCAN3;ZKSCAN4;ZNF121;ZNF124;ZNF133;ZNF136;ZNF138;ZNF14;ZNF140;ZNF143;ZNF146;ZNF148;ZNF155;ZNF157;ZNF160;ZNF169;ZNF175;ZNF177;ZNF18;ZNF180;ZNF181;ZNF2;ZNF20;ZNF212;ZNF213;ZNF214;ZNF221;ZNF222;ZNF223;ZNF224;ZNF225;ZNF226;ZNF227;ZNF229;ZNF230;ZNF232;ZNF233;ZNF234;ZNF235;ZNF24;ZNF25;ZNF250;ZNF257;ZNF26;ZNF260;ZNF263;ZNF264;ZNF268;ZNF274;ZNF276;ZNF28;ZNF280A;ZNF280B;ZNF280C;ZNF280D;ZNF281;ZNF282;ZNF283;ZNF284;ZNF285;ZNF286A;ZNF286B;ZNF3;ZNF30;ZNF300;ZNF302;ZNF317;ZNF32;ZNF320;ZNF322;ZNF324;ZNF324B;ZNF329;ZNF331;ZNF333;ZNF33A;ZNF33B;ZNF343;ZNF345;ZNF347;ZNF350;ZNF354A;ZNF354B;ZNF362;ZNF366;ZNF383;ZNF384;ZNF394;ZNF397;ZNF398;ZNF404;ZNF41;ZNF410;ZNF419;ZNF420;ZNF431;ZNF432;ZNF436;ZNF439;ZNF44;ZNF440;ZNF442;ZNF443;ZNF446;ZNF449;ZNF45;ZNF460;ZNF468;ZNF479;ZNF484;ZNF49	GLI1;GLI2;GLI3;GLIS1;GLIS2;GLIS3;ZIC1;ZIC2;ZIC3;ZIC4

Motif	Nº of probes	% of probes	lower OR	upper OR	OR	p.value	FDR	TF family	TF subfamily	TF.family.member	TF.subfamily.member
										0;ZNF500;ZNF502;ZNF524;ZNF525;ZNF528;ZNF543;ZNF544;ZNF546;ZNF547;ZNF548;ZNF549;ZNF554;ZNF555;ZNF557;ZNF558;ZNF559;ZNF561;ZNF562;ZNF563;ZNF564;ZNF566;ZNF567;ZNF568;ZNF57;ZNF570;ZNF571;ZNF572;ZNF577;ZNF581;ZNF582;ZNF583;ZNF585A;ZNF586;ZNF589;ZNF595;ZNF599;ZNF600;ZNF605;ZNF607;ZNF611;ZNF613;ZNF614;ZNF615;ZNF616;ZNF619;ZNF620;ZNF621;ZNF625;ZNF627;ZNF649;ZNF652;ZNF653;ZNF665;ZNF667;ZNF669;ZNF670;ZNF672;ZNF679;ZNF680;ZNF683;ZNF689;ZNF692;ZNF701;ZNF705D;ZNF705E;ZNF705G;ZNF708;ZNF709;ZNF71;ZNF710;ZNF713;ZNF721;ZNF727;ZNF729;ZNF736;ZNF75A;ZNF75D;ZNF76;ZNF763;ZNF764;ZNF765;ZNF768;ZNF77;ZNF771;ZNF773;ZNF774;ZNF776;ZNF777;ZNF780A;ZNF780B;ZNF782;ZNF785;ZNF799;ZNF805;ZNF808;ZNF81;ZNF813;ZNF816;ZNF823;ZNF829;ZNF836;ZNF841;ZNF844;ZNF845;ZNF846;ZNF85;ZNF853;ZNF860;ZNF878;ZNF891;ZNF99;ZSCAN16;ZSCAN2;ZSCAN22;ZSCAN23;ZSCAN29;ZSCAN31;ZSCAN32;ZSCAN4;ZSCAN5A;ZSCAN5B;ZSCAN5C;ZSCAN9;ZXDA;ZXDB;ZXDC	
SMCA1_HUMAN.H11MO.0.C	18	0.07058824	0.4589413	12.735.049	0.7880686	0.376208096	0.68384993	Myb/SANT domain factors{3.5.1}	SMARCA-like factors{3.5.1.5}	CDC5L;MYBL1;MYBL2;MYB;SMARCA1;SMARCA5	SMARCA1;SMARCA5
ZN143_HUMAN.H11MO.0.A	30	0.11764706	0.5189715	11.562.801	0.7874810	0.246648582	0.55958113	More than 3 adjacent zinc finger factors{2.3.3}	ZNF76-like factors{2.3.3.28}	BCL6;BCL6B;CTCF;CTCFL;FEZF1;FEZF2;GFI1;GFI1B;GLI1;GLI2;GLI3;GLI4;GLIS1;GLIS2;GLIS3;HKR1;MTF1;MYNN;MZF1;OSR2;OVOL1;OVOL2;PLAG1;PLAGL1;PLAGL2;PRDM1;PRDM14;PRDM6;SCRT1;SCRT2;SNAI1;SNAI2;SNAI3;WT1;YY1;YY2;ZBTB12;ZBTB14;ZBTB18;ZBTB20;ZBTB26;ZBTB42;ZBTB45;ZBTB47;ZBTB48;ZBTB49;ZBTB6;ZBTB7A;ZBTB7B;ZBTB7C;ZFP14;ZFP2;ZFP28;ZFP30;ZFP37;ZFP42;ZFP64;ZFP69;ZFP69B;ZFP82;ZFP91;ZFX;ZIC1;ZIC2;ZIC3;ZIC4;ZIC5;ZIK1;ZIM3;ZKSCAN1;ZKSCAN2;ZKSCAN3;ZKSCAN4;ZNF121;ZNF124;ZNF133;ZNF136;ZNF138;ZNF14;ZNF140;ZNF143;ZNF146;ZNF148;ZNF155;ZNF157;ZNF160;ZNF169;ZNF175;ZNF177;ZNF18;ZNF180;ZNF181;ZNF2;ZNF20;ZNF212;ZNF213;ZNF214;ZNF221;ZNF222;ZNF223;ZNF224;ZNF225;ZNF226;ZNF227;ZNF229;ZNF230;ZNF232;ZNF233;ZNF234;ZNF235;ZNF24;ZNF25;ZNF250;ZNF257;ZNF26;ZNF260;ZNF263;ZNF264;ZNF268;ZNF274;ZNF276;ZNF28;ZNF280A;ZNF280B;ZNF280C;ZNF280D;ZNF281;ZNF282;ZNF283;ZNF284;ZNF285;ZNF286A;ZNF286B;ZNF3;ZNF30;ZNF300;ZNF302;ZNF317;ZNF32;ZNF320;ZNF322;ZNF324;ZNF324B;ZNF329;ZNF331;ZNF333;ZNF33A;ZNF33B;ZNF343;ZNF345;ZNF347;ZNF350;ZNF354A;ZNF354B;ZNF362;ZNF366;ZNF383;ZNF384;ZNF394;ZNF397;ZNF398;ZNF404;ZNF41;ZNF410;ZNF419;ZNF420;ZNF431;ZNF432;ZNF436;ZNF439;ZNF44;ZNF440;ZNF442;ZNF443;ZNF446;ZNF449;ZNF45;ZNF460;ZNF468;ZNF479;ZNF484;ZNF490;ZNF500;ZNF502;ZNF524;ZNF525;ZNF528;ZNF543;ZNF544;ZNF546;ZNF547;ZNF548;ZNF549;ZNF554;ZNF555;ZNF557;ZNF558;ZNF559;ZNF561;ZNF562;ZNF563;ZNF564;ZNF566;ZNF567;ZNF568;ZNF57;ZNF570;ZNF571;ZNF572;ZNF577;ZNF581;ZNF582;ZNF583;ZNF585A;ZNF586;ZNF589;ZNF595;ZNF599;ZNF600;ZNF605;ZNF607;ZNF611;ZNF613;ZNF614;ZNF615;ZNF616;ZNF619;ZNF620;ZNF621;ZNF625;ZNF627;ZNF649;ZNF652;ZNF653;ZNF665;ZNF667;ZNF669;ZNF670;ZNF672;ZNF679;ZNF680;ZNF683;ZNF689;ZNF692;ZNF701;ZNF705D;ZNF705E;ZNF705G;ZNF708;ZNF709;ZNF71;ZNF710;ZNF713;ZNF721;ZNF727;ZNF729;ZNF736;ZNF75A;ZNF75D;ZNF76;ZNF763;ZNF764;ZNF765;ZNF768;ZNF77;ZNF771;ZNF773;ZNF774;ZNF776;ZNF777;ZNF780A;ZNF780B;ZNF782;ZNF785;ZNF799;ZNF805;ZNF808;ZNF81;ZNF813;ZNF816;ZNF823;ZNF829;ZNF836;ZNF841;ZNF844;ZNF845;ZNF846;ZNF85;ZNF853;ZNF860;ZNF878;ZNF891;ZNF99;ZSCAN16;ZSCAN2;ZSCAN22;ZSCAN23;ZSCAN29;ZSCAN31;ZSCAN32;ZSCAN4;ZSCAN5A;ZSCAN5B;ZSCAN5C;ZSCAN9;ZXDA;ZXDB;ZXDC	ZNF143;ZNF76

Motif	Nº of probes	% of probes	lower OR	upper OR	OR	p.value	FDR	TF family	TF subfamily	TF.family.member	TF.subfamily.member
HNF1A_HUMAN.H11MO.0.C	11	0.04313725	0.3872218	14.330.367	0.7863820	0.577939965	0.82401200	POU domain factors{3.1.10}	HNF1-like factors{3.1.10.7}	CDX1;CDX2;CDX4;EVX1;EVX2;GBX1;GBX2;GSX1;GSX2;HDX;HMBOX1;HNF1A;HNF1B;HOXA1;HOXA10;HOXA11;HOXA13;HOXA2;HOXA3;HOXA4;HOXA5;HOXA6;HOXA7;HOXA9;HOXB1;HOXB13;HOXB2;HOXB3;HOXB4;HOXB5;HOXB6;HOXB7;HOXB8;HOXB9;HOXC10;HOXC11;HOXC12;HOXC13;HOXC4;HOXC5;HOXC6;HOXC8;HOXC9;HOXD1;HOXD10;HOXD11;HOXD12;HOXD13;HOXD3;HOXD4;HOXD8;HOXD9;MEOX1;MEOX2;MNX1;PDX1;POU1F1;POU2F1;POU2F2;POU2F3;POU3F1;POU3F2;POU3F3;POU3F4;POU4F1;POU4F2;POU4F3;POU5F1;POU5F1B;POU5F2;POU6F1;POU6F2	HMBOX1;HNF1A;HNF1B
FOXD2_HUMAN.H11MO.0.D	17	0.06666667	0.4501863	12.863.059	0.7861347	0.425782680	0.71210075	Forkhead box (FOX) factors{3.3.1}	FOXD{3.3.1.4}	FOXA1;FOXA2;FOXA3;FOXB1;FOXB2;FOXC1;FOXC2;FOXD1;FOXD2;FOXD3;FOXD4;FOXD4L1;FOXD4L3;FOXD4L4;FOXD4L5;FOXD4L6;FOXE1;FOXE3;FOXF1;FOXF2;FOXG1;FOXH1;FOXI1;FOXI2;FOXI3;FOXJ1;FOXJ2;FOXJ3;FOXK1;FOXK2;FOXL1;FOXL2;FOXM1;FOXN1;FOXN2;FOXN3;FOXN4;FOXO1;FOXO3;FOXO4;FOXO6;FOXP1;FOXP2;FOXP3;FOXP4;FOXQ1;FOXR1;FOXR2;FOXS1	FOXD1;FOXD2;FOXD3
FOXI1_HUMAN.H11MO.0.B	10	0.03921569	0.3718173	14.703.800	0.7855252	0.562184746	0.81928784	Forkhead box (FOX) factors{3.3.1}	FOXI{3.3.1.9}	FOXA1;FOXA2;FOXA3;FOXB1;FOXB2;FOXC1;FOXC2;FOXD1;FOXD2;FOXD3;FOXD4;FOXD4L1;FOXD4L3;FOXD4L4;FOXD4L5;FOXD4L6;FOXE1;FOXE3;FOXF1;FOXF2;FOXG1;FOXH1;FOXI1;FOXI2;FOXI3;FOXJ1;FOXJ2;FOXJ3;FOXK1;FOXK2;FOXL1;FOXL2;FOXM1;FOXN1;FOXN2;FOXN3;FOXN4;FOXO1;FOXO3;FOXO4;FOXO6;FOXP1;FOXP2;FOXP3;FOXP4;FOXQ1;FOXR1;FOXR2;FOXS1	FOXI1
ANDR_HUMAN.H11MO.2.A	23	0.09019608	0.4873545	12.065.440	0.7844633	0.319741963	0.63062840	Steroid hormone receptors (NR3){2.1.1}	GR-like receptors (NR3C){2.1.1.1}	AR;ESR1;ESR2;ESRRA;ESRRB;ESRRG;NR3C1;NR3C2;PGR	AR;NR3C1;NR3C2;PGR
ELK3_HUMAN.H11MO.0.D	37	0.14509804	0.5375961	11.152.701	0.7841095	0.189572142	0.48237664	Ets-related factors{3.5.2}	Elk-like factors{3.5.2.2}	EHF;ELF1;ELF2;ELF3;ELF4;ELF5;ELK1;ELK3;ELK4;ERF;ERG;ETS1;ETS2;ETV1;ETV2;ETV3;ETV3L;ETV4;ETV5;ETV6;ETV7;FEV;FLI1;GABPA;SPDEF;SPI1;SPIB;SPIC	ELK1;ELK3;ELK4;ETV1;ETV4;ETV5
GCR_HUMAN.H11MO.1.A	23	0.09019608	0.4870524	12.057.963	0.7839768	0.319812845	0.63062840	Steroid hormone receptors (NR3){2.1.1}	GR-like receptors (NR3C){2.1.1.1}	AR;ESR1;ESR2;ESRRA;ESRRB;ESRRG;NR3C1;NR3C2;PGR	AR;NR3C1;NR3C2;PGR
COT2_HUMAN.H11MO.1.A	38	0.14901961	0.5398092	11.105.376	0.7837159	0.193690833	0.48643528	RXR-related receptors (NR2){2.1.3}	COUP-like receptors (NR2F){2.1.3.5}	HNF4A;HNF4G;NR2C1;NR2C2;NR2E1;NR2E3;NR2F1;NR2F2;NR2F6;RXRA;RXRB;RXRG	NR2F1;NR2F2;NR2F6
PO4F3_HUMAN.H11MO.0.D	14	0.05490196	0.4200956	13.358.226	0.7803482	0.457626421	0.73443572	POU domain factors{3.1.10}	POU4 (Brn-3-like factors){3.1.10.4}	CDX1;CDX2;CDX4;EVX1;EVX2;GBX1;GBX2;GSX1;GSX2;HDX;HMBOX1;HNF1A;HNF1B;HOXA1;HOXA10;HOXA11;HOXA13;HOXA2;HOXA3;HOXA4;HOXA5;HOXA6;HOXA7;HOXA9;HOXB1;HOXB13;HOXB2;HOXB3;HOXB4;HOXB5;HOXB6;HOXB7;HOXB8;HOXB9;HOXC10;HOXC11;HOXC12;HOXC13;HOXC4;HOXC5;HOXC6;HOXC8;HOXC9;HOXD1;HOXD10;HOXD11;HOXD12;HOXD13;HOXD3;HOXD4;HOXD8;HOXD9;MEOX1;MEOX2;MNX1;PDX1;POU1F1;POU2F1;POU2F2;POU2F3;POU3F1;POU3F2;POU3F3;POU3F4;POU4F1;POU4F2;POU4F3;POU5F1;POU5F1B;POU5F2;POU6F1;POU6F2	POU4F1;POU4F2;POU4F3
VENTX_HUMAN.H11MO.0.D	10	0.03921569	0.3687850	14.583.779	0.7791162	0.562789871	0.81928784	NK-related factors{3.1.2}	VENTX{3.1.2.23}	BARHL1;BARHL2;BARX1;BARX2;BSX;DBX1;DBX2;DLX1;DLX2;DLX3;DLX4;DLX5;DLX6;EMX1;EMX2;EN1;EN2;HHEX;HLX;HMX1;HMX2;HMX3;LBX1;LBX2;MSX1;MSX2;NANOG;NKX1-1;NKX1-2;NKX2-1;NKX2-2;NKX2-3;NKX2-4;NKX2-5;NKX2-6;NKX2-8;NKX3-1;NKX3-2;NKX6-1;NKX6-2;NKX6-3;NOTO;TLX1;TLX2;TLX3;VAX1;VAX2;VENTX	VENTX
VAX1_HUMAN.H11MO.0.D	10	0.03921569	0.3684844	14.571.879	0.7784807	0.562867450	0.81928784	NK-related factors{3.1.2}	VAX{3.1.2.22}	BARHL1;BARHL2;BARX1;BARX2;BSX;DBX1;DBX2;DLX1;DLX2;DLX3;DLX4;DLX5;DLX6;EMX1;EMX2;EN1;EN2;HHEX;HLX;HMX1;HMX2;HMX3;LBX1;LBX2;MSX1;MSX2;NANOG;NKX1-1;NKX1-2;NKX2-1;NKX2-2;NKX2-3;NKX2-4;NKX2-5;NKX2-6;NKX2-8;NKX3-1;NKX3-2;NKX6-1;NKX6-2;NKX6-3;NOTO;TLX1;TLX2;TLX3;VAX1;VAX2;VENTX	VAX1;VAX2
MEF2B_HUMAN.H11MO.0.A	20	0.07843137	0.4650412	12.261.597	0.7756911	0.343342564	0.65204063	Regulators of differentiation{5.1.1}	MEF-2{5.1.1.1}	MEF2A;MEF2B;MEF2C;MEF2D	MEF2A;MEF2B;MEF2C;MEF2D

Motif	Nº of probes	% of probes	lower OR	upper OR	OR	p.value	FDR	TF family	TF subfamily	TF.family.member	TF.subfamily.member
ISX_HUMAN.H11MO.0.D	9	0.03529412	0.3502261	14.973.254	0.7754387	0.546138106	0.81040299	Paired-related HD factors{3.1.3}	ISX{3.1.3.12}	ALX1;ALX3;ALX4;ARGFX;ARX;CRX;DMBX1;DPRX;DRGX;DUX4;DUXA;ESX1;GSC;GSC2;HESX1;ISX;LEUTX;MIXL1;NOBOX;OTP;OTX1;OTX2;PHOX2A;PHOX2B;PITX1;PITX2;PITX3;PROP1;PRRX1;PRRX2;RAX;RAX2;RHOXF1;RHOXF2;SEBOX;SHOX;SHOX2;TPRX1;UNCX;VSX1;VSX2	ISX
BRAC_HUMAN.H11MO.1.B	20	0.07843137	0.4648399	12.256.298	0.7753550	0.343411195	0.65204063	Brachyury-related factors{6.5.1}	T (Brachyury){6.5.1.0.1}	T;TBX19	T
MAFK_HUMAN.H11MO.1.A	29	0.11372549	0.5069243	11.435.189	0.7745212	0.209376984	0.50938142	Maf-related factors{1.1.3}	Small Maf factors{1.1.3.2}	MAF;MAFA;MAFB;MAFF;MAFG;MAFK;NRL	MAFF;MAFG;MAFK
ONEC3_HUMAN.H11MO.0.D	12	0.04705882	0.3945431	13.786.264	0.7744624	0.507249940	0.77698545	HD-CUT factors{3.1.9}	ONECUT{3.1.9.1}	CUX1;CUX2;ONECUT1;ONECUT2;ONECUT3;SATB1;SATB2	ONECUT1;ONECUT2;ONECUT3
GCR_HUMAN.H11MO.0.A	21	0.08235294	0.4698817	12.112.089	0.7737793	0.303783464	0.61756191	Steroid hormone receptors (NR3){2.1.1}	GR-like receptors (NR3C){2.1.1.1}	AR;ESR1;ESR2;ESRRA;ESRRB;ESRRG;NR3C1;NR3C2;PGR	AR;NR3C1;NR3C2;PGR
MIXL1_HUMAN.H11MO.0.D	9	0.03529412	0.3481272	14.882.382	0.7707344	0.546310749	0.81040299	Paired-related HD factors{3.1.3}	MIX{3.1.3.14}	ALX1;ALX3;ALX4;ARGFX;ARX;CRX;DMBX1;DPRX;DRGX;DUX4;DUXA;ESX1;GSC;GSC2;HESX1;ISX;LEUTX;MIXL1;NOBOX;OTP;OTX1;OTX2;PHOX2A;PHOX2B;PITX1;PITX2;PITX3;PROP1;PRRX1;PRRX2;RAX;RAX2;RHOXF1;RHOXF2;SEBOX;SHOX;SHOX2;TPRX1;UNCX;VSX1;VSX2	MIXL1
IKZF1_HUMAN.H11MO.0.C	29	0.11372549	0.5044345	11.379.049	0.7707116	0.209360094	0.50938142	Factors with multiple dispersed zinc fingers{2.3.4}	Ikaros{2.3.4.4}	BCL11A;BCL11B;BNC1;BNC2;E4F1;HIC1;HIC2;HINFP;HIVEP1;HIVEP2;HIVEP3;IKZF1;IKZF2;IKZF3;IKZF4;IKZF5;INSM1;INSM2;MAZ;MECOM;PATZ1;PRDM16;PRDM4;REST;RLF;RREB1;SALL1;SALL2;SALL3;SALL4;VEZF1;ZBTB1;ZBTB17;ZBTB2;ZBTB25;ZBTB4;ZFAT;ZNF134;ZNF211;ZNF217;ZNF219;ZNF248;ZNF256;ZNF292;ZNF296;ZNF319;ZNF334;ZNF335;ZNF341;ZNF37A;ZNF382;ZNF417;ZNF418;ZNF423;ZNF467;ZNF510;ZNF512;ZNF512B;ZNF516;ZNF518A;ZNF518B;ZNF521;ZNF526;ZNF532;ZNF536;ZNF552;ZNF574;ZNF587;ZNF587B;ZNF592;ZNF639;ZNF654;ZNF658;ZNF671;ZNF687;ZNF711;ZNF717;ZNF770;ZNF772;ZNF784;ZNF786;ZNF792;ZNF8;ZNF814	IKZF1
GLI3_HUMAN.H11MO.0.B	29	0.11372549	0.5035791	11.359.720	0.7694004	0.209434383	0.50938142	More than 3 adjacent zinc finger factors{2.3.3}	GLI-like factors{2.3.3.1}	BCL6;BCL6B;CTCF;CTCFL;FEZF1;FEZF2;GFI1;GFI1B;GLI1;GLI2;GLI3;GLI4;GLIS1;GLIS2;GLIS3;HKR1;MTF1;MYNN;MZF1;OSR2;OVOL1;OVOL2;PLAG1;PLAGL1;PLAGL2;PRDM1;PRDM14;PRDM6;SCRT1;SCRT2;SNAI1;SNAI2;SNAI3;WT1;YY1;YY2;ZBTB12;ZBTB14;ZBTB18;ZBTB20;ZBTB26;ZBTB42;ZBTB45;ZBTB47;ZBTB48;ZBTB49;ZBTB6;ZBTB7A;ZBTB7B;ZBTB7C;ZFP14;ZFP2;ZFP28;ZFP30;ZFP37;ZFP42;ZFP64;ZFP69;ZFP69B;ZFP82;ZFP91;ZFX;ZIC1;ZIC2;ZIC3;ZIC4;ZIC5;ZIK1;ZIM3;ZKSCAN1;ZKSCAN2;ZKSCAN3;ZKSCAN4;ZNF121;ZNF124;ZNF133;ZNF136;ZNF138;ZNF14;ZNF140;ZNF143;ZNF146;ZNF148;ZNF155;ZNF157;ZNF160;ZNF169;ZNF175;ZNF177;ZNF18;ZNF180;ZNF181;ZNF2;ZNF20;ZNF212;ZNF213;ZNF214;ZNF221;ZNF222;ZNF223;ZNF224;ZNF225;ZNF226;ZNF227;ZNF229;ZNF230;ZNF232;ZNF233;ZNF234;ZNF235;ZNF24;ZNF25;ZNF250;ZNF257;ZNF26;ZNF260;ZNF263;ZNF264;ZNF268;ZNF274;ZNF276;ZNF28;ZNF280A;ZNF280B;ZNF280C;ZNF280D;ZNF281;ZNF282;ZNF283;ZNF284;ZNF285;ZNF286A;ZNF286B;ZNF3;ZNF30;ZNF300;ZNF302;ZNF317;ZNF32;ZNF320;ZNF322;ZNF324;ZNF324B;ZNF329;ZNF331;ZNF333;ZNF33A;ZNF33B;ZNF343;ZNF345;ZNF347;ZNF350;ZNF354A;ZNF354B;ZNF362;ZNF366;ZNF383;ZNF384;ZNF394;ZNF397;ZNF398;ZNF404;ZNF41;ZNF410;ZNF419;ZNF420;ZNF431;ZNF432;ZNF436;ZNF439;ZNF44;ZNF440;ZNF442;ZNF443;ZNF446;ZNF449;ZNF45;ZNF460;ZNF468;ZNF479;ZNF484;ZNF490;ZNF500;ZNF502;ZNF524;ZNF525;ZNF528;ZNF543;ZNF544;ZNF546;ZNF547;ZNF548;ZNF549;ZNF554;ZNF555;ZNF557;ZNF558;ZNF559;ZNF561;ZNF562;ZNF563;ZNF564;ZNF566;ZNF567;ZNF568;ZNF57;ZNF570;ZNF571;ZNF572;ZNF577;ZNF581;ZNF582;ZNF583;ZNF585A;ZNF586;ZNF589;ZNF595;ZNF599;ZNF600;ZNF605;ZNF607;ZNF611;ZNF613;ZNF614;ZNF61	GLI1;GLI2;GLI3;GLIS1;GLIS2;GLIS3;ZIC1;ZIC2;ZIC3;ZIC4

Motif	Nº of probes	% of probes	lower OR	upper OR	OR	p.value	FDR	TF family	TF subfamily	TF.family.member	TF.subfamily.member
										5;ZNF616;ZNF619;ZNF620;ZNF621;ZNF625;ZNF627;ZNF649;ZNF652;ZNF653;ZNF665;ZNF667;ZNF669;ZNF670;ZNF672;ZNF679;ZNF680;ZNF683;ZNF689;ZNF692;ZNF701;ZNF705D;ZNF705E;ZNF705G;ZNF708;ZNF709;ZNF71;ZNF710;ZNF713;ZNF721;ZNF727;ZNF729;ZNF736;ZNF75A;ZNF75D;ZNF76;ZNF763;ZNF764;ZNF765;ZNF768;ZNF77;ZNF771;ZNF773;ZNF774;ZNF776;ZNF777;ZNF780A;ZNF780B;ZNF782;ZNF785;ZNF799;ZNF805;ZNF808;ZNF81;ZNF813;ZNF816;ZNF823;ZNF829;ZNF836;ZNF841;ZNF844;ZNF845;ZNF846;ZNF85;ZNF853;ZNF860;ZNF878;ZNF891;ZNF99;ZSCAN16;ZSCAN2;ZSCAN22;ZSCAN23;ZSCAN29;ZSCAN31;ZSCAN32;ZSCAN4;ZSCAN5A;ZSCAN5B;ZSCAN5C;ZSCAN9;ZXDA;ZXDB;ZXDC	
Z354A_HUMAN.H11MO.0.C	21	0.08235294	0.4670539	12.039.194	0.7691196	0.304375518	0.61756191	More than 3 adjacent zinc finger factors{2.3.3}	ZNF354A-like factors{2.3.3.64}	BCL6;BCL6B;CTCF;CTCFL;FEZF1;FEZF2;GFI1;GFI1B;GLI1;GLI2;GLI3;GLI4;GLIS1;GLIS2;GLIS3;HKR1;MTF1;MYNN;MZF1;OSR2;OVOL1;OVOL2;PLAG1;PLAGL1;PLAGL2;PRDM1;PRDM14;PRDM6;SCRT1;SCRT2;SNAI1;SNAI2;SNAI3;WT1;YY1;YY2;ZBTB12;ZBTB14;ZBTB18;ZBTB20;ZBTB26;ZBTB42;ZBTB45;ZBTB47;ZBTB48;ZBTB49;ZBTB6;ZBTB7A;ZBTB7B;ZBTB7C;ZFP14;ZFP2;ZFP28;ZFP30;ZFP37;ZFP42;ZFP64;ZFP69;ZFP69B;ZFP82;ZFP91;ZFX;ZIC1;ZIC2;ZIC3;ZIC4;ZIC5;ZIK1;ZIM3;ZKSCAN1;ZKSCAN2;ZKSCAN3;ZKSCAN4;ZNF121;ZNF124;ZNF133;ZNF136;ZNF138;ZNF14;ZNF140;ZNF143;ZNF146;ZNF148;ZNF155;ZNF157;ZNF160;ZNF169;ZNF175;ZNF177;ZNF18;ZNF180;ZNF181;ZNF2;ZNF20;ZNF212;ZNF213;ZNF214;ZNF221;ZNF222;ZNF223;ZNF224;ZNF225;ZNF226;ZNF227;ZNF229;ZNF230;ZNF232;ZNF233;ZNF234;ZNF235;ZNF24;ZNF25;ZNF250;ZNF257;ZNF26;ZNF260;ZNF263;ZNF264;ZNF268;ZNF274;ZNF276;ZNF28;ZNF280A;ZNF280B;ZNF280C;ZNF280D;ZNF281;ZNF282;ZNF283;ZNF284;ZNF285;ZNF286A;ZNF286B;ZNF3;ZNF30;ZNF300;ZNF302;ZNF317;ZNF32;ZNF320;ZNF322;ZNF324;ZNF324B;ZNF329;ZNF331;ZNF333;ZNF33A;ZNF33B;ZNF343;ZNF345;ZNF347;ZNF350;ZNF354A;ZNF354B;ZNF362;ZNF366;ZNF383;ZNF384;ZNF394;ZNF397;ZNF398;ZNF404;ZNF41;ZNF410;ZNF419;ZNF420;ZNF431;ZNF432;ZNF436;ZNF439;ZNF44;ZNF440;ZNF442;ZNF443;ZNF446;ZNF449;ZNF45;ZNF460;ZNF468;ZNF479;ZNF484;ZNF490;ZNF500;ZNF502;ZNF524;ZNF525;ZNF528;ZNF543;ZNF544;ZNF546;ZNF547;ZNF548;ZNF549;ZNF554;ZNF555;ZNF557;ZNF558;ZNF559;ZNF561;ZNF562;ZNF563;ZNF564;ZNF566;ZNF567;ZNF568;ZNF57;ZNF570;ZNF571;ZNF572;ZNF577;ZNF581;ZNF582;ZNF583;ZNF585A;ZNF586;ZNF589;ZNF595;ZNF599;ZNF600;ZNF605;ZNF607;ZNF611;ZNF613;ZNF614;ZNF615;ZNF616;ZNF619;ZNF620;ZNF621;ZNF625;ZNF627;ZNF649;ZNF652;ZNF653;ZNF665;ZNF667;ZNF669;ZNF670;ZNF672;ZNF679;ZNF680;ZNF683;ZNF689;ZNF692;ZNF701;ZNF705D;ZNF705E;ZNF705G;ZNF708;ZNF709;ZNF71;ZNF710;ZNF713;ZNF721;ZNF727;ZNF729;ZNF736;ZNF75A;ZNF75D;ZNF76;ZNF763;ZNF764;ZNF765;ZNF768;ZNF77;ZNF771;ZNF773;ZNF774;ZNF776;ZNF777;ZNF780A;ZNF780B;ZNF782;ZNF785;ZNF799;ZNF805;ZNF808;ZNF81;ZNF813;ZNF816;ZNF823;ZNF829;ZNF836;ZNF841;ZNF844;ZNF845;ZNF846;ZNF85;ZNF853;ZNF860;ZNF878;ZNF891;ZNF99;ZSCAN16;ZSCAN2;ZSCAN22;ZSCAN23;ZSCAN29;ZSCAN31;ZSCAN32;ZSCAN4;ZSCAN5A;ZSCAN5B;ZSCAN5C;ZSCAN9;ZXDA;ZXDB;ZXDC	ZNF354A
FOXQ1_HUMAN.H11MO.0.C	15	0.05882353	0.4226401	12.918.552	0.7672531	0.404200419	0.70050069	Forkhead box (FOX) factors{3.3.1}	FOXQ{3.3.1.17}	FOXA1;FOXA2;FOXA3;FOXB1;FOXB2;FOXC1;FOXC2;FOXD1;FOXD2;FOXD3;FOXD4;FOXD4L1;FOXD4L3;FOXD4L4;FOXD4L5;FOXD4L6;FOXE1;FOXE3;FOXF1;FOXF2;FOXG1;FOXH1;FOXI1;FOXI2;FOXI3;FOXJ1;FOXJ2;FOXJ3;FOXK1;FOXK2;FOXL1;FOXL2;FOXM1;FOXN1;FOXN2;FOXN3;FOXN4;FOXO1;FOXO3;FOXO4;FOXO6;FOXP1;FOXP2;FOXP3;FOXP4;FOXQ1;FOXR1;FOXR2;FOXS1	FOXQ1
TWST1_HUMAN.H11MO.1.A	24	0.09411765	0.4808729	11.690.823	0.7661161	0.245599130	0.55958113	Tal-related factors{1.2.3}	Twist-like factors{1.2.3.2}	ATOH1;ATOH7;ATOH8;BHLHA15;BHLHA9;BHLHE22;BHLHE23;FERD3L;FIGLA;HAND1;HAND2;LYL1;MESP1;MESP2;MSC;MSGN1;NEUROD1;NEUROD2;NEUROD4;NEUROD6;NEUROG1;NEUROG2;NEUROG3;NHLH1;NHLH2;OLIG1;OL	FIGLA;HAND1;PTF1A;TWIST1

Motif	Nº of probes	% of probes	lower OR	upper OR	OR	p.value	FDR	TF family	TF subfamily	TF.family.member	TF.subfamily.member
										IG2;OLIG3;PTF1A;SCX;TAL1;TAL2;TCF15;TCF21;TCF23;TWIST1;TWIST2	
ETV3_HUMAN.H11MO.0.D	13	0.05098039	0.3992150	13.267.824	0.7609263	0.446625621	0.72800920	Ets-related factors{3.5.2}	Ets-like factors{3.5.2.1}	EHF;ELF1;ELF2;ELF3;ELF4;ELF5;ELK1;ELK3;ELK4;ERF;ERG;ETS1;ETS2;ETV1;ETV2;ETV3;ETV3L;ETV4;ETV5;ETV6;ETV7;FEV;FLI1;GABPA;SPDEF;SPI1;SPIB;SPIC	ERG;ETS1;ETS2;ETV2;ETV3;FEV;FLI1;GABPA
PHX2A_HUMAN.H11MO.0.D	9	0.03529412	0.3434707	14.683.003	0.7604134	0.547280492	0.81040299	Paired-related HD factors{3.1.3}	PHOX{3.1.3.18}	ALX1;ALX3;ALX4;ARGFX;ARX;CRX;DMBX1;DPRX;DRGX;DUX4;DUXA;ESX1;GSC;GSC2;HESX1;ISX;LEUTX;MIXL1;NOBOX;OTP;OTX1;OTX2;PHOX2A;PHOX2B;PITX1;PITX2;PITX3;PROP1;PRRX1;PRRX2;RAX;RAX2;RHOXF1;RHOXF2;SEBOX;SHOX;SHOX2;TPRX1;UNCX;VSX1;VSX2	PHOX2A;PHOX2B
CPEB1_HUMAN.H11MO.0.D	34	0.13333333	0.5126388	10.931.335	0.7591245	0.153981305	0.43490953	NA	NA	CPEB1	CPEB1
CREB3_HUMAN.H11MO.0.D	33	0.12941176	0.5093429	10.971.495	0.7585014	0.149884450	0.43119743	CREB-related factors{1.1.7}	CREB-3-like factors{1.1.7.2}	ATF1;ATF6;ATF6B;CREB1;CREB3;CREB3L1;CREB3L2;CREB3L3;CREB3L4;CREBL2;CREBZF;CREM	CREB3L1;CREB3L2;CREB3
LMX1B_HUMAN.H11MO.0.D	15	0.05882353	0.4178020	12.771.206	0.7584988	0.344610579	0.65204063	HD-LIM factors{3.1.5}	Lmx{3.1.5.6}	ISL1;ISL2;LHX1;LHX2;LHX3;LHX4;LHX5;LHX6;LHX8;LHX9;LMX1A;LMX1B	LMX1A;LMX1B
SOX21_HUMAN.H11MO.0.D	9	0.03529412	0.3423851	14.636.524	0.7580075	0.547626407	0.81040299	SOX-related factors{4.1.1}	Group B{4.1.1.2}	BBX;CIC;HBP1;SOX1;SOX10;SOX11;SOX12;SOX13;SOX14;SOX15;SOX17;SOX18;SOX2;SOX21;SOX3;SOX30;SOX4;SOX5;SOX6;SOX7;SOX8;SOX9;SRY	SOX1;SOX21;SOX2;SOX3
GLI2_HUMAN.H11MO.0.D	32	0.12549020	0.5037702	10.967.669	0.7546485	0.145894560	0.42287483	More than 3 adjacent zinc finger factors{2.3.3}	GLI-like factors{2.3.3.1}	BCL6;BCL6B;CTCF;CTCFL;FEZF1;FEZF2;GFI1;GFI1B;GLI1;GLI2;GLI3;GLI4;GLIS1;GLIS2;GLIS3;HKR1;MTF1;MYNN;MZF1;OSR2;OVOL1;OVOL2;PLAG1;PLAGL1;PLAGL2;PRDM1;PRDM14;PRDM6;SCRT1;SCRT2;SNAI1;SNAI2;SNAI3;WT1;YY1;YY2;ZBTB12;ZBTB14;ZBTB18;ZBTB20;ZBTB26;ZBTB42;ZBTB45;ZBTB47;ZBTB48;ZBTB49;ZBTB6;ZBTB7A;ZBTB7B;ZBTB7C;ZFP14;ZFP2;ZFP28;ZFP30;ZFP37;ZFP42;ZFP64;ZFP69;ZFP69B;ZFP82;ZFP91;ZFX;ZIC1;ZIC2;ZIC3;ZIC4;ZIC5;ZIK1;ZIM3;ZKSCAN1;ZKSCAN2;ZKSCAN3;ZKSCAN4;ZNF121;ZNF124;ZNF133;ZNF136;ZNF138;ZNF14;ZNF140;ZNF143;ZNF146;ZNF148;ZNF155;ZNF157;ZNF160;ZNF169;ZNF175;ZNF177;ZNF18;ZNF180;ZNF181;ZNF2;ZNF20;ZNF212;ZNF213;ZNF214;ZNF221;ZNF222;ZNF223;ZNF224;ZNF225;ZNF226;ZNF227;ZNF229;ZNF230;ZNF232;ZNF233;ZNF234;ZNF235;ZNF24;ZNF25;ZNF250;ZNF257;ZNF26;ZNF260;ZNF263;ZNF264;ZNF268;ZNF274;ZNF276;ZNF28;ZNF280A;ZNF280B;ZNF280C;ZNF280D;ZNF281;ZNF282;ZNF283;ZNF284;ZNF285;ZNF286A;ZNF286B;ZNF3;ZNF30;ZNF300;ZNF302;ZNF317;ZNF32;ZNF320;ZNF322;ZNF324;ZNF324B;ZNF329;ZNF331;ZNF333;ZNF33A;ZNF33B;ZNF343;ZNF345;ZNF347;ZNF350;ZNF354A;ZNF354B;ZNF362;ZNF366;ZNF383;ZNF384;ZNF394;ZNF397;ZNF398;ZNF404;ZNF41;ZNF410;ZNF419;ZNF420;ZNF431;ZNF432;ZNF436;ZNF439;ZNF44;ZNF440;ZNF442;ZNF443;ZNF446;ZNF449;ZNF45;ZNF460;ZNF468;ZNF479;ZNF484;ZNF490;ZNF500;ZNF502;ZNF524;ZNF525;ZNF528;ZNF543;ZNF544;ZNF546;ZNF547;ZNF548;ZNF549;ZNF554;ZNF555;ZNF557;ZNF558;ZNF559;ZNF561;ZNF562;ZNF563;ZNF564;ZNF566;ZNF567;ZNF568;ZNF57;ZNF570;ZNF571;ZNF572;ZNF577;ZNF581;ZNF582;ZNF583;ZNF585A;ZNF586;ZNF589;ZNF595;ZNF599;ZNF600;ZNF605;ZNF607;ZNF611;ZNF613;ZNF614;ZNF615;ZNF616;ZNF619;ZNF620;ZNF621;ZNF625;ZNF627;ZNF649;ZNF652;ZNF653;ZNF665;ZNF667;ZNF669;ZNF670;ZNF672;ZNF679;ZNF680;ZNF683;ZNF689;ZNF692;ZNF701;ZNF705D;ZNF705E;ZNF705G;ZNF708;ZNF709;ZNF71;ZNF710;ZNF713;ZNF721;ZNF727;ZNF729;ZNF736;ZNF75A;ZNF75D;ZNF76;ZNF763;ZNF764;ZNF765;ZNF768;ZNF77;ZNF771;ZNF773;ZNF774;ZNF776;ZNF777;ZNF780A;ZNF780B;ZNF782;ZNF785;ZNF799;ZNF805;ZNF808;ZNF81;ZNF813;ZNF816;ZNF823;ZNF829;ZNF836;ZNF841;ZNF844;ZNF845;ZNF846;ZNF85;ZNF853;ZNF860;ZNF878;ZNF891;ZNF99;ZSCAN16;ZSCAN2;ZSCAN22;ZSCAN23;ZSCAN29;ZSCAN31;ZSCAN32;ZSCAN4;ZSCAN5A;ZSCAN5B;ZSCAN5C;ZSCAN9;ZXDA;ZXDB;ZXDC	GLI1;GLI2;GLI3;GLIS1;GLIS2;GLIS3;ZIC1;ZIC2;ZIC3;ZIC4

Motif	Nº of probes	% of probes	lower OR	upper OR	OR	p.value	FDR	TF family	TF subfamily	TF.family.member	TF.subfamily.member
PAX8_HUMAN.H11MO.0.D	17	0.06666667	0.4310440	12.315.320	0.7526649	0.314783627	0.62968135	Paired domain only{3.2.2}	PAX-2-like factors (partial homeobox){3.2.2.2}	PAX1;PAX2;PAX5;PAX8;PAX9	PAX2;PAX5;PAX8
ZN121_HUMAN.H11MO.0.C	47	0.18431373	0.5359647	10.370.687	0.7523589	0.086753788	0.29994247	More than 3 adjacent zinc finger factors{2.3.3}	unclassified{2.3.3.0}	BCL6B;BCL6;CTCFL;CTCF;FEZF1;GFI1B;GFI1;GLI1;GLI2;GLI3;GLIS1;GLIS2;GLIS3;MTF1;MYNN;MZF1;OSR2;OVOL1;OVOL2;ZNF146;PLAG1;PLAGL1;PRDM14;PRDM1;PRDM6;SCRT1;SCRT2;SNAI1;SNAI2;YY1;YY2;WT1;ZNF324;ZNF354A;ZBTB14;ZBTB18;ZBTB48;ZBTB49;ZBTB7A;ZBTB7B;ZBTB6;ZFP64;ZFP28;ZFP42;ZFP82;ZFX;ZIC1;ZIC2;ZIC3;ZIC4;ZIM3;ZKSCAN1;ZKSCAN3;ZNF121;ZNF136;ZNF140;ZNF143;ZNF148;ZNF214;ZNF232;ZNF250;ZNF257;ZNF260;ZNF263;ZNF264;ZNF274;ZNF281;ZNF282;ZNF317;ZNF320;ZNF322;ZNF329;ZNF331;ZNF333;ZNF350;ZNF384;ZNF394;ZNF410;ZNF436;ZNF449;ZNF490;ZNF502;ZNF524;ZNF528;ZNF547;ZNF549;ZNF554;ZNF563;ZNF582;ZNF586;ZNF589;ZNF652;ZNF667;ZNF680;ZNF708;ZNF713;ZNF768;ZNF816;ZNF18;ZNF41;ZNF76;ZNF85;ZSCAN16;ZSCAN22;ZSCAN31;ZSCAN4	MYNN;MZF1;OSR2;PRDM14;PRDM6;WT1;ZBTB14;ZBTB48;ZBTB49;ZFP64;ZFP28;ZIM3;ZNF121;ZNF250;ZNF257;ZNF263;ZNF274;ZNF317;ZNF320;ZNF329;ZNF331;ZNF394;ZNF449;ZNF502;ZNF528;ZNF547;ZNF549;ZNF554;ZNF586;ZNF589;ZNF667;ZNF680;ZNF708;ZNF713;ZNF768;ZNF18;ZNF85;ZSCAN16;ZSCAN22
NKX62_HUMAN.H11MO.0.D	10	0.03921569	0.3558442	14.070.062	0.7517019	0.476823489	0.75026716	NK-related factors{3.1.2}	NK-6{3.1.2.19}	BARHL1;BARHL2;BARX1;BARX2;BSX;DBX1;DBX2;DLX1;DLX2;DLX3;DLX4;DLX5;DLX6;EMX1;EMX2;EN1;EN2;HHEX;HLX;HMX1;HMX2;HMX3;LBX1;LBX2;MSX1;MSX2;NANOG;NKX1-1;NKX1-2;NKX2-1;NKX2-2;NKX2-3;NKX2-4;NKX2-5;NKX2-6;NKX2-8;NKX3-1;NKX3-2;NKX6-1;NKX6-2;NKX6-3;NOTO;TLX1;TLX2;TLX3;VAX1;VAX2;VENTX	NKX6-1;NKX6-2
ZN394_HUMAN.H11MO.1.D	15	0.05882353	0.4139835	12.653.892	0.7515617	0.345103871	0.65204063	More than 3 adjacent zinc finger factors{2.3.3}	unclassified{2.3.3.0}	BCL6B;BCL6;CTCFL;CTCF;FEZF1;GFI1B;GFI1;GLI1;GLI2;GLI3;GLIS1;GLIS2;GLIS3;MTF1;MYNN;MZF1;OSR2;OVOL1;OVOL2;ZNF146;PLAG1;PLAGL1;PRDM14;PRDM1;PRDM6;SCRT1;SCRT2;SNAI1;SNAI2;YY1;YY2;WT1;ZNF324;ZNF354A;ZBTB14;ZBTB18;ZBTB48;ZBTB49;ZBTB7A;ZBTB7B;ZBTB6;ZFP64;ZFP28;ZFP42;ZFP82;ZFX;ZIC1;ZIC2;ZIC3;ZIC4;ZIM3;ZKSCAN1;ZKSCAN3;ZNF121;ZNF136;ZNF140;ZNF143;ZNF148;ZNF214;ZNF232;ZNF250;ZNF257;ZNF260;ZNF263;ZNF264;ZNF274;ZNF281;ZNF282;ZNF317;ZNF320;ZNF322;ZNF329;ZNF331;ZNF333;ZNF350;ZNF384;ZNF394;ZNF410;ZNF436;ZNF449;ZNF490;ZNF502;ZNF524;ZNF528;ZNF547;ZNF549;ZNF554;ZNF563;ZNF582;ZNF586;ZNF589;ZNF652;ZNF667;ZNF680;ZNF708;ZNF713;ZNF768;ZNF816;ZNF18;ZNF41;ZNF76;ZNF85;ZSCAN16;ZSCAN22;ZSCAN31;ZSCAN4	MYNN;MZF1;OSR2;PRDM14;PRDM6;WT1;ZBTB14;ZBTB48;ZBTB49;ZFP64;ZFP28;ZIM3;ZNF121;ZNF250;ZNF257;ZNF263;ZNF274;ZNF317;ZNF320;ZNF329;ZNF331;ZNF394;ZNF449;ZNF502;ZNF528;ZNF547;ZNF549;ZNF554;ZNF586;ZNF589;ZNF667;ZNF680;ZNF708;ZNF713;ZNF768;ZNF18;ZNF85;ZSCAN16;ZSCAN22
ATF3_HUMAN.H11MO.0.A	31	0.12156863	0.4984653	10.974.609	0.7513661	0.141873086	0.41596671	Fos-related factors{1.1.2}	ATF-3-like factors{1.1.2.2}	ATF3;FOS;FOSB;FOSL1;FOSL2;JDP2	ATF3;JDP2
HNF1B_HUMAN.H11MO.1.A	9	0.03529412	0.3392263	14.501.281	0.7510070	0.548898121	0.81072883	POU domain factors{3.1.10}	HNF1-like factors{3.1.10.7}	CDX1;CDX2;CDX4;EVX1;EVX2;GBX1;GBX2;GSX1;GSX2;HDX;HMBOX1;HNF1A;HNF1B;HOXA1;HOXA10;HOXA11;HOXA13;HOXA2;HOXA3;HOXA4;HOXA5;HOXA6;HOXA7;HOXA9;HOXB1;HOXB13;HOXB2;HOXB3;HOXB4;HOXB5;HOXB6;HOXB7;HOXB8;HOXB9;HOXC10;HOXC11;HOXC12;HOXC13;HOXC4;HOXC5;HOXC6;HOXC8;HOXC9;HOXD1;HOXD10;HOXD11;HOXD12;HOXD13;HOXD3;HOXD4;HOXD8;HOXD9;MEOX1;MEOX2;MNX1;PDX1;POU1F1;POU2F1;POU2F2;POU2F3;POU3F1;POU3F2;POU3F3;POU3F4;POU4F1;POU4F2;POU4F3;POU5F1;POU5F1B;POU5F2;POU6F1;POU6F2	HMBOX1;HNF1A;HNF1B
ZNF18_HUMAN.H11MO.0.C	28	0.10980392	0.4875686	11.145.095	0.7504534	0.176146540	0.46670403	More than 3 adjacent zinc finger factors{2.3.3}	unclassified{2.3.3.0}	BCL6B;BCL6;CTCFL;CTCF;FEZF1;GFI1B;GFI1;GLI1;GLI2;GLI3;GLIS1;GLIS2;GLIS3;MTF1;MYNN;MZF1;OSR2;OVOL1;OVOL2;ZNF146;PLAG1;PLAGL1;PRDM14;PRDM1;PRDM6;SCRT1;SCRT2;SNAI1;SNAI2;YY1;YY2;WT1;ZNF324;ZNF354A;ZBTB14;ZBTB18;ZBTB48;ZBTB49;ZBTB7A;ZBTB7B;ZBTB6;ZFP64;ZFP28;ZFP42;ZFP82;ZFX;ZIC1;ZIC2;ZIC3;ZIC4;ZIM3;ZKSCAN1;ZKSCAN3;ZNF121;ZNF136;ZNF140;ZNF143;ZNF148;ZNF214;ZNF232;ZNF250;ZNF257;ZNF260;ZNF263;ZNF264;ZNF274;ZNF281;ZNF282;ZNF317;ZNF320;ZNF322;ZNF329;ZNF331;ZNF333;ZNF350;ZNF384;ZNF394;ZNF410;ZNF436;ZNF449;ZNF490;ZNF502;ZNF524;ZNF528;ZNF547;ZNF549;ZNF554;ZNF563;ZNF582;ZNF586;ZNF589;ZNF652;ZNF667;ZNF680;ZNF708;ZNF713;ZNF768;ZNF816;ZNF18;ZNF41;ZNF76;ZNF85;ZSCAN16;ZSCAN22;ZSCAN31;ZSCAN4	MYNN;MZF1;OSR2;PRDM14;PRDM6;WT1;ZBTB14;ZBTB48;ZBTB49;ZFP64;ZFP28;ZIM3;ZNF121;ZNF250;ZNF257;ZNF263;ZNF274;ZNF317;ZNF320;ZNF329;ZNF331;ZNF394;ZNF449;ZNF502;ZNF528;ZNF547;ZNF549;ZNF554;ZNF586;ZNF589;ZNF667;ZNF680;ZNF708;ZNF713;ZNF768;ZNF18;ZNF85;ZSCAN16;ZSCAN22

Motif	Nº of probes	% of probes	lower OR	upper OR	OR	p.value	FDR	TF family	TF subfamily	TF.family.member	TF.subfamily.member
HMGA2_HUMAN.H11MO.0.D	8	0.03137255	0.3198785	15.014.132	0.7496114	0.528632269	0.79604586	HMGA factors{8.2.1}	HMGA2 (HMGI-C){8.2.1.0.2}	HMGA1;HMGA2	HMGA2
DRGX_HUMAN.H11MO.0.D	9	0.03529412	0.3372289	14.415.768	0.7465807	0.459064676	0.73443572	Paired-related HD factors{3.1.3}	DRGX{3.1.3.6}	ALX1;ALX3;ALX4;ARGFX;ARX;CRX;DMBX1;DPRX;DRGX;DUX4;DUXA;ESX1;GSC;GSC2;HESX1;ISX;LEUTX;MIXL1;NOBOX;OTP;OTX1;OTX2;PHOX2A;PHOX2B;PITX1;PITX2;PITX3;PROP1;PRRX1;PRRX2;RAX;RAX2;RHOXF1;RHOXF2;SEBOX;SHOX;SHOX2;TPRX1;UNCX;VSX1;VSX2	DRGX
HXC11_HUMAN.H11MO.0.D	11	0.04313725	0.3669060	13.577.805	0.7450820	0.416597788	0.70568537	HOX-related factors{3.1.1}	HOX9-13{3.1.1.8}	CDX1;CDX2;CDX4;EVX1;EVX2;GBX1;GBX2;GSX1;GSX2;HDX;HMBOX1;HNF1A;HNF1B;HOXA1;HOXA10;HOXA11;HOXA13;HOXA2;HOXA3;HOXA4;HOXA5;HOXA6;HOXA7;HOXA9;HOXB1;HOXB13;HOXB2;HOXB3;HOXB4;HOXB5;HOXB6;HOXB7;HOXB8;HOXB9;HOXC10;HOXC11;HOXC12;HOXC13;HOXC4;HOXC5;HOXC6;HOXC8;HOXC9;HOXD1;HOXD10;HOXD11;HOXD12;HOXD13;HOXD3;HOXD4;HOXD8;HOXD9;MEOX1;MEOX2;MNX1;PDX1;POU1F1;POU2F1;POU2F2;POU2F3;POU3F1;POU3F2;POU3F3;POU3F4;POU4F1;POU4F2;POU4F3;POU5F1;POU5F2;POU6F1;POU6F2	HOXA10;HOXA11;HOXA13;HOXA9;HOXB13;HOXC10;HOXC11;HOXC12;HOXC13;HOXC9;HOXD10;HOXD11;HOXD12;HOXD13;HOXD9
ZFHX3_HUMAN.H11MO.0.D	11	0.04313725	0.3666955	13.569.994	0.7446544	0.416635855	0.70568537	HD-ZF factors{3.1.8}	ZFHX{3.1.8.4}	ADNP;ADNP2;HOMEZ;NANOGNB;TSHZ1;TSHZ2;TSHZ3;ZEB1;ZEB2;ZFHX2;ZFHX3;ZFHX4;ZHX1;ZHX2;ZHX3	ZFHX3
ZN350_HUMAN.H11MO.0.C	13	0.05098039	0.3898872	12.958.569	0.7431943	0.379703919	0.68384993	More than 3 adjacent zinc finger factors{2.3.3}	ZNF350-like factors{2.3.3.30}	BCL6;BCL6B;CTCF;CTCFL;FEZF1;FEZF2;GFI1;GFI1B;GLI1;GLI2;GLI3;GLI4;GLIS1;GLIS2;GLIS3;HKR1;MTF1;MYNN;MZF1;OSR2;OVOL1;OVOL2;PLAG1;PLAGL1;PLAGL2;PRDM1;PRDM14;PRDM6;SCRT1;SCRT2;SNAI1;SNAI2;SNAI3;WT1;YY1;YY2;ZBTB12;ZBTB14;ZBTB18;ZBTB20;ZBTB26;ZBTB42;ZBTB45;ZBTB47;ZBTB48;ZBTB49;ZBTB6;ZBTB7A;ZBTB7B;ZBTB7C;ZFP14;ZFP2;ZFP28;ZFP30;ZFP37;ZFP42;ZFP64;ZFP69;ZFP69B;ZFP82;ZFP91;ZFX;ZIC1;ZIC2;ZIC3;ZIC4;ZIC5;ZIK1;ZIM3;ZKSCAN1;ZKSCAN2;ZKSCAN3;ZKSCAN4;ZNF121;ZNF124;ZNF133;ZNF136;ZNF138;ZNF14;ZNF140;ZNF143;ZNF146;ZNF148;ZNF155;ZNF157;ZNF160;ZNF169;ZNF175;ZNF177;ZNF18;ZNF180;ZNF181;ZNF2;ZNF20;ZNF212;ZNF213;ZNF214;ZNF221;ZNF222;ZNF223;ZNF224;ZNF225;ZNF226;ZNF227;ZNF229;ZNF230;ZNF232;ZNF233;ZNF234;ZNF235;ZNF24;ZNF25;ZNF250;ZNF257;ZNF26;ZNF260;ZNF263;ZNF264;ZNF268;ZNF274;ZNF276;ZNF28;ZNF280A;ZNF280B;ZNF280C;ZNF280D;ZNF281;ZNF282;ZNF283;ZNF284;ZNF285;ZNF286A;ZNF286B;ZNF3;ZNF30;ZNF300;ZNF302;ZNF317;ZNF32;ZNF320;ZNF322;ZNF324;ZNF324B;ZNF329;ZNF331;ZNF333;ZNF33A;ZNF33B;ZNF343;ZNF345;ZNF347;ZNF350;ZNF354A;ZNF354B;ZNF362;ZNF366;ZNF383;ZNF384;ZNF394;ZNF397;ZNF398;ZNF404;ZNF41;ZNF410;ZNF419;ZNF420;ZNF431;ZNF432;ZNF436;ZNF439;ZNF44;ZNF440;ZNF442;ZNF443;ZNF446;ZNF449;ZNF45;ZNF460;ZNF468;ZNF479;ZNF484;ZNF490;ZNF500;ZNF502;ZNF524;ZNF525;ZNF528;ZNF543;ZNF544;ZNF546;ZNF547;ZNF548;ZNF549;ZNF554;ZNF555;ZNF557;ZNF558;ZNF559;ZNF561;ZNF562;ZNF563;ZNF564;ZNF566;ZNF567;ZNF568;ZNF57;ZNF570;ZNF571;ZNF572;ZNF577;ZNF581;ZNF582;ZNF583;ZNF585A;ZNF586;ZNF589;ZNF595;ZNF599;ZNF600;ZNF605;ZNF607;ZNF611;ZNF613;ZNF614;ZNF615;ZNF616;ZNF619;ZNF620;ZNF621;ZNF625;ZNF627;ZNF649;ZNF652;ZNF653;ZNF665;ZNF667;ZNF669;ZNF670;ZNF672;ZNF679;ZNF680;ZNF683;ZNF689;ZNF692;ZNF701;ZNF705D;ZNF705E;ZNF705G;ZNF708;ZNF709;ZNF71;ZNF710;ZNF713;ZNF721;ZNF727;ZNF729;ZNF736;ZNF75A;ZNF75D;ZNF76;ZNF763;ZNF764;ZNF765;ZNF768;ZNF77;ZNF771;ZNF773;ZNF774;ZNF776;ZNF777;ZNF780A;ZNF780B;ZNF782;ZNF785;ZNF799;ZNF805;ZNF808;ZNF81;ZNF813;ZNF816;ZNF823;ZNF829;ZNF836;ZNF841;ZNF844;ZNF845;ZNF846;ZNF85;ZNF853;ZNF860;ZNF878;ZNF891;ZNF99;ZSCAN16;ZSCAN2;ZSCAN22;ZSCAN23;ZSCAN29;ZSCAN31;ZSCAN32;ZSCAN4;ZSCAN5A;ZSCAN5B;ZSCAN5C;ZSCAN9;ZXDA;ZXDB;ZXDC	ZNF350

Motif	Nº of probes	% of probes	lower OR	upper OR	OR	p.value	FDR	TF family	TF subfamily	TF.family.member	TF.subfamily.member
ZN394_HUMAN.H11MO.0.C	25	0.09803922	0.4706119	11.249.723	0.7427138	0.187548416	0.47880738	More than 3 adjacent zinc finger factors{2.3.3}	unclassified{2.3.3.0}	BCL6B;BCL6;CTCFL;CTCF;FEZF1;GFI1B;GFI1;GLI1;GLI2;GLI3;GLIS1;GLIS2;GLIS3;MTF1;MYNN;MZF1;OSR2;OVOL1;OVOL2;ZNF146;PLAG1;PLAGL1;PRDM14;PRDM1;PRDM6;SCRT1;SCRT2;SNAI1;SNAI2;YY1;YY2;WT1;ZNF324;ZNF354A;ZBTB14;ZBTB18;ZBTB48;ZBTB49;ZBTB7A;ZBTB7B;ZBTB6;ZFP64;ZFP28;ZFP42;ZFP82;ZFX;ZIC1;ZIC2;ZIC3;ZIC4;ZIM3;ZKSCAN1;ZKSCAN3;ZNF121;ZNF136;ZNF140;ZNF143;ZNF148;ZNF214;ZNF232;ZNF250;ZNF257;ZNF260;ZNF263;ZNF264;ZNF274;ZNF281;ZNF282;ZNF317;ZNF320;ZNF322;ZNF329;ZNF331;ZNF333;ZNF350;ZNF384;ZNF394;ZNF410;ZNF436;ZNF449;ZNF490;ZNF502;ZNF524;ZNF528;ZNF547;ZNF549;ZNF554;ZNF563;ZNF582;ZNF586;ZNF589;ZNF652;ZNF667;ZNF680;ZNF708;ZNF713;ZNF768;ZNF816;ZNF18;ZNF41;ZNF76;ZNF85;ZSCAN16;ZSCAN22;ZSCAN31;ZSCAN4	MYNN;MZF1;OSR2;PRDM14;PRDM6;WT1;ZBTB14;ZBTB48;ZBTB49;ZFP64;ZFP28;ZIM3;ZNF121;ZNF250;ZNF257;ZNF263;ZNF274;ZNF317;ZNF320;ZNF329;ZNF331;ZNF394;ZNF449;ZNF502;ZNF528;ZNF547;ZNF549;ZNF554;ZNF586;ZNF589;ZNF667;ZNF680;ZNF708;ZNF713;ZNF768;ZNF18;ZNF85;ZSCAN16;ZSCAN22
HXA2_HUMAN.H11MO.0.D	10	0.03921569	0.3506324	13.866.182	0.7408032	0.478711799	0.75093678	HOX-related factors{3.1.1}	HOX2{3.1.1.2}	CDX1;CDX2;CDX4;EVX1;EVX2;GBX1;GBX2;GSX1;GSX2;HDX;HMBOX1;HNF1A;HNF1B;HOXA1;HOXA10;HOXA11;HOXA13;HOXA2;HOXA3;HOXA4;HOXA5;HOXA6;HOXA7;HOXA9;HOXB1;HOXB13;HOXB2;HOXB3;HOXB4;HOXB5;HOXB6;HOXB7;HOXB8;HOXB9;HOXC10;HOXC11;HOXC12;HOXC13;HOXC4;HOXC5;HOXC6;HOXC8;HOXC9;HOXD1;HOXD10;HOXD11;HOXD12;HOXD13;HOXD3;HOXD4;HOXD8;HOXD9;MEOX1;MEOX2;MNX1;PDX1;POU1F1;POU2F1;POU2F2;POU2F3;POU3F1;POU3F2;POU3F3;POU3F4;POU4F1;POU4F2;POU4F3;POU5F1;POU5F2;POU6F1;POU6F2	HOXA2;HOXB2
GATA2_HUMAN.H11MO.0.A	13	0.05098039	0.3876590	12.884.593	0.7389264	0.380634132	0.68384993	GATA-type zinc fingers{2.2.1}	Two zinc-finger GATA factors{2.2.1.1}	GATA1;GATA2;GATA3;GATA4;GATA5;GATA6;GATAD2A;GATAD2B;TRPS1;ZGLP1	GATA1;GATA2;GATA3;GATA4;GATA5;GATA6
BATF_HUMAN.H11MO.1.A	11	0.04313725	0.3638244	13.463.490	0.7388222	0.417370336	0.70568537	B-ATF-related factors{1.1.4}	B-ATF{1.1.4.0.1}	BATF;BATF2;BATF3	BATF
DLX1_HUMAN.H11MO.0.D	12	0.04705882	0.3757677	13.130.806	0.7376569	0.364956981	0.66843361	NK-related factors{3.1.2}	DLX{3.1.2.5}	BARHL1;BARHL2;BARX1;BARX2;BSX;DBX1;DBX2;DLX1;DLX2;DLX3;DLX4;DLX5;DLX6;EMX1;EMX2;EN1;EN2;HHEX;HLX;HMX1;HMX2;HMX3;LBX1;LBX2;MSX1;MSX2;NANOG;NKX1-1;NKX1-2;NKX2-1;NKX2-2;NKX2-3;NKX2-4;NKX2-5;NKX2-6;NKX2-8;NKX3-1;NKX3-2;NKX6-1;NKX6-2;NKX6-3;NOTO;TLX1;TLX2;TLX3;VAX1;VAX2;VENTX	DLX1;DLX2;DLX3;DLX4;DLX5;DLX6
TF2LX_HUMAN.H11MO.0.D	15	0.05882353	0.4060614	12.411.171	0.7371629	0.292936979	0.60283889	TALE-type homeo domain factors{3.1.4}	TGIF{3.1.4.6}	IRX1;IRX2;IRX3;IRX4;IRX5;IRX6;MEIS1;MEIS2;MEIS3;MKX;PBX1;PBX2;PBX3;PKNOX1;PKNOX2;TGIF1;TGIF2;TGIF2LX;TGIF2LY	TGIF2LX;TGIF1;TGIF2
ZBED1_HUMAN.H11MO.0.D	17	0.06666667	0.4203989	12.010.635	0.7340740	0.268752547	0.57145415	BED zinc finger factors{2.3.5}	ZBED1 (ALTE, DREF, TRAMP){2.3.5.0.1}	ZBED1;ZBED2;ZBED3;ZBED4;ZBED5;ZBED6	ZBED1
STAT6_HUMAN.H11MO.0.B	17	0.06666667	0.4203585	12.009.488	0.7340038	0.268764900	0.57145415	STAT factors{6.2.1}	STAT6{6.2.1.0.7}	STAT1;STAT2;STAT3;STAT4;STAT5A;STAT5B;STAT6	STAT6
TBX5_HUMAN.H11MO.0.D	21	0.08235294	0.4457320	11.488.938	0.7339836	0.191048113	0.48424706	TBX2-related factors{6.5.4}	TBX5{6.5.4.0.4}	TBX2;TBX3;TBX4;TBX5	TBX5
PITX2_HUMAN.H11MO.0.D	16	0.06274510	0.4125797	12.171.579	0.7338185	0.257917146	0.56481632	Paired-related HD factors{3.1.3}	PITX{3.1.3.19}	ALX1;ALX3;ALX4;ARGFX;ARX;CRX;DMBX1;DPRX;DRGX;DUX4;DUXA;ESX1;GSC;GSC2;HESX1;ISX;LEUTX;MIXL1;NOBOX;OTP;OTX1;OTX2;PHOX2A;PHOX2B;PITX1;PITX2;PITX3;PROP1;PRRX1;PRRX2;RAX;RAX2;RHOXF1;RHOXF2;SEBOX;SHOX;SHOX2;TPRX1;UNCX;VSX1;VSX2	PITX1;PITX2;PITX3
ZN140_HUMAN.H11MO.0.C	15	0.05882353	0.4032987	12.327.124	0.7321376	0.293392868	0.60283889	More than 3 adjacent zinc finger factors{2.3.3}	ZNF302-like factors{2.3.3.44}	BCL6;BCL6B;CTCF;CTCFL;FEZF1;FEZF2;GFI1;GFI1B;GLI1;GLI2;GLI3;GLI4;GLIS1;GLIS2;GLIS3;HKR1;MTF1;MYNN;MZF1;OSR2;OVOL1;OVOL2;PLAG1;PLAGL1;PLAGL2;PRDM1;PRDM14;PRDM6;SCRT1;SCRT2;SNAI1;SNAI2;SNAI3;WT1;YY1;YY2;ZBTB12;ZBTB14;ZBTB18;ZBTB20;ZBTB26;ZBTB42;ZBTB45;ZBTB47;ZBTB48;ZBTB49;ZBTB6;ZBTB7A;ZBTB7B;ZBTB7C;ZFP14;ZFP2;ZFP28;ZFP30;ZFP37;ZFP42;ZFP64;ZFP69;ZFP69B;ZFP82;ZFP91;ZFX;ZIC1;ZIC2;ZIC3;ZIC4;ZIC5;ZIK1;ZIM3;ZKSCAN1;ZKSCAN2;ZKSCAN3;ZKSCAN4;ZNF121;ZNF124;ZNF133;ZNF136;ZNF138;ZNF14;ZNF140;ZNF143;ZNF146;ZNF148;ZNF155;ZNF157;ZNF160;ZNF169;ZNF175;	ZNF140

Motif	Nº of probes	% of probes	lower OR	upper OR	OR	p.value	FDR	TF family	TF subfamily	TF.family.member	TF.subfamily.member
										ZNF177;ZNF18;ZNF180;ZNF181;ZNF2;ZNF20;ZNF212;ZNF213;ZNF214;ZNF221;ZNF222;ZNF223;ZNF224;ZNF225;ZNF226;ZNF227;ZNF229;ZNF230;ZNF232;ZNF233;ZNF234;ZNF235;ZNF24;ZNF25;ZNF250;ZNF257;ZNF26;ZNF260;ZNF263;ZNF264;ZNF268;ZNF274;ZNF276;ZNF28;ZNF280A;ZNF280B;ZNF280C;ZNF280D;ZNF281;ZNF282;ZNF283;ZNF284;ZNF285;ZNF286A;ZNF286B;ZNF3;ZNF30;ZNF300;ZNF302;ZNF317;ZNF32;ZNF320;ZNF322;ZNF324;ZNF324B;ZNF329;ZNF331;ZNF333;ZNF33A;ZNF33B;ZNF343;ZNF345;ZNF347;ZNF350;ZNF354A;ZNF354B;ZNF362;ZNF366;ZNF383;ZNF384;ZNF394;ZNF397;ZNF398;ZNF404;ZNF41;ZNF410;ZNF419;ZNF420;ZNF431;ZNF432;ZNF436;ZNF439;ZNF44;ZNF440;ZNF442;ZNF443;ZNF446;ZNF449;ZNF45;ZNF460;ZNF468;ZNF479;ZNF484;ZNF490;ZNF500;ZNF502;ZNF524;ZNF525;ZNF528;ZNF543;ZNF544;ZNF546;ZNF547;ZNF548;ZNF549;ZNF554;ZNF555;ZNF557;ZNF558;ZNF559;ZNF561;ZNF562;ZNF563;ZNF564;ZNF566;ZNF567;ZNF568;ZNF57;ZNF570;ZNF571;ZNF572;ZNF577;ZNF581;ZNF582;ZNF583;ZNF585A;ZNF586;ZNF589;ZNF595;ZNF599;ZNF600;ZNF605;ZNF607;ZNF611;ZNF613;ZNF614;ZNF615;ZNF616;ZNF619;ZNF620;ZNF621;ZNF625;ZNF627;ZNF649;ZNF652;ZNF653;ZNF665;ZNF667;ZNF669;ZNF670;ZNF672;ZNF679;ZNF680;ZNF683;ZNF689;ZNF692;ZNF701;ZNF705D;ZNF705E;ZNF705G;ZNF708;ZNF709;ZNF71;ZNF710;ZNF713;ZNF721;ZNF727;ZNF729;ZNF736;ZNF75A;ZNF75D;ZNF76;ZNF763;ZNF764;ZNF765;ZNF768;ZNF77;ZNF771;ZNF773;ZNF774;ZNF776;ZNF777;ZNF780A;ZNF780B;ZNF782;ZNF785;ZNF799;ZNF805;ZNF808;ZNF81;ZNF813;ZNF816;ZNF823;ZNF829;ZNF836;ZNF841;ZNF844;ZNF845;ZNF846;ZNF85;ZNF853;ZNF860;ZNF878;ZNF891;ZNF99;ZSCAN16;ZSCAN2;ZSCAN22;ZSCAN23;ZSCAN29;ZSCAN31;ZSCAN32;ZSCAN4;ZSCAN5A;ZSCAN5B;ZSCAN5C;ZSCAN9;ZXDA;ZXDB;ZXDC	
STA5B_HUMAN.H11MO.0.A	15	0.05882353	0.4028703	12.313.920	0.7313580	0.293501392	0.60283889	STAT factors{6.2.1}	STAT5B{6.2.1.0.6}	STAT1;STAT2;STAT3;STAT4;STAT5A;STAT5B;STAT6	STAT5B
NFYB_HUMAN.H11MO.0.A	9	0.03529412	0.3296757	14.095.084	0.7299817	0.460236356	0.73443572	Heteromeric CCAAT-binding factors{4.2.1}	NF-YB (CP1B, CBF-A){4.2.1.0.2}	NFYA;NFYB;NFYC	NFYB
CR3L2_HUMAN.H11MO.0.D	46	0.18039216	0.5177218	10.075.817	0.7290540	0.053332769	0.21757209	CREB-related factors{1.1.7}	CREB-3-like factors{1.1.7.2}	ATF1;ATF6;ATF6B;CREB1;CREB3;CREB3L1;CREB3L2;CREB3L3;CREB3L4;CREBL2;CREBZF;CREM	CREB3L1;CREB3L2;CREB3
BMAL1_HUMAN.H11MO.0.A	42	0.16470588	0.5104999	10.191.692	0.7290231	0.065727078	0.25593726	PAS domain factors{1.2.5}	Arnt-like factors{1.2.5.2}	AHR;AHRR;ARNT;ARNT2;ARNTL;ARNTL2;CLOCK;EPAS1;HIF1A;HIF3A;NCOA1;NCOA2;NCOA3;NPAS1;NPAS2;NPAS3;NPAS4;SIM1;SIM2;SOHLH1;SOHLH2;TCFL5	ARNT2;ARNT;ARNTL;CLOCK
FIGLA_HUMAN.H11MO.0.D	26	0.10196078	0.4651021	10.943.093	0.7275215	0.141453260	0.41596671	Tal-related factors{1.2.3}	Twist-like factors{1.2.3.2}	ATOH1;ATOH7;ATOH8;BHLHA15;BHLHA9;BHLHE22;BHLHE23;FERD3L;FIGLA;HAND1;HAND2;LYL1;MESP1;MESP2;MSC;MSGN1;NEUROD1;NEUROD2;NEUROD4;NEUROD6;NEUROG1;NEUROG2;NEUROG3;NHLH1;NHLH2;OLIG1;OLIG2;OLIG3;PTF1A;SCX;TAL1;TAL2;TCF15;TCF21;TCF23;TWIST1;TWIST2	FIGLA;HAND1;PTF1A;TWIST1
GSC_HUMAN.H11MO.0.D	8	0.03137255	0.3102966	14.562.990	0.7270981	0.440113820	0.72321602	Paired-related HD factors{3.1.3}	GSC{3.1.3.9}	ALX1;ALX3;ALX4;ARGFX;ARX;CRX;DMBX1;DPRX;DRGX;DUX4;DUXA;ESX1;GSC;GSC2;HESX1;ISX;LEUTX;MIXL1;NOBOX;OTP;OTX1;OTX2;PHOX2A;PHOX2B;PITX1;PITX2;PITX3;PROP1;PRRX1;PRRX2;RAX;RAX2;RHOXF1;RHOXF2;SEBOX;SHOX;SHOX2;TPRX1;UNCX;VSX1;VSX2	GSC2;GSC
GLI1_HUMAN.H11MO.0.D	50	0.19607843	0.5199673	0.9901160	0.7235191	0.042935437	0.19134810	More than 3 adjacent zinc finger factors{2.3.3}	GLI-like factors{2.3.3.1}	BCL6;BCL6B;CTCF;CTCFL;FEZF1;FEZF2;GFI1;GFI1B;GLI1;GLI2;GLI3;GLI4;GLIS1;GLIS2;GLIS3;HKR1;MTF1;MYNN;MZF1;OSR2;OVOL1;OVOL2;PLAG1;PLAGL1;PLAGL2;PRDM1;PRDM14;PRDM6;SCRT1;SCRT2;SNAI1;SNAI2;SNAI3;WT1;YY1;YY2;ZBTB12;ZBTB14;ZBTB18;ZBTB20;ZBTB26;ZBTB42;ZBTB45;ZBTB47;ZBTB48;ZBTB49;ZBTB6;ZBTB7A;ZBTB7B;ZBTB7C;ZFP14;ZFP2;ZFP28;ZFP30;ZFP37;ZFP42;ZFP64;ZFP69;ZFP69B;ZFP82;ZFP91;ZFX;ZIC1;ZIC2;ZIC3;ZIC4;ZIC5;ZIK1;ZIM3;ZKSCAN1;ZKSCAN2;ZKSCAN3;ZKSCAN4;ZNF	GLI1;GLI2;GLI3;GLIS1;GLIS2;GLIS3;ZIC1;ZIC2;ZIC3;ZIC4

Motif	Nº of probes	% of probes	lower OR	upper OR	OR	p.value	FDR	TF family	TF subfamily	TF.family.member	TF.subfamily.member
										121;ZNF124;ZNF133;ZNF136;ZNF138;ZNF14;ZNF140;ZNF143;ZNF146;ZNF148;ZNF155;ZNF157;ZNF160;ZNF169;ZNF175;ZNF177;ZNF18;ZNF180;ZNF181;ZNF2;ZNF20;ZNF212;ZNF213;ZNF214;ZNF221;ZNF222;ZNF223;ZNF224;ZNF225;ZNF226;ZNF227;ZNF229;ZNF230;ZNF232;ZNF233;ZNF234;ZNF235;ZNF24;ZNF25;ZNF250;ZNF257;ZNF26;ZNF260;ZNF263;ZNF264;ZNF268;ZNF274;ZNF276;ZNF28;ZNF280A;ZNF280B;ZNF280C;ZNF280D;ZNF281;ZNF282;ZNF283;ZNF284;ZNF285;ZNF286A;ZNF286B;ZNF3;ZNF30;ZNF300;ZNF302;ZNF317;ZNF32;ZNF320;ZNF322;ZNF324;ZNF324B;ZNF329;ZNF331;ZNF333;ZNF33A;ZNF33B;ZNF343;ZNF345;ZNF347;ZNF350;ZNF354A;ZNF354B;ZNF362;ZNF366;ZNF383;ZNF384;ZNF394;ZNF397;ZNF398;ZNF404;ZNF41;ZNF410;ZNF419;ZNF420;ZNF431;ZNF432;ZNF436;ZNF439;ZNF44;ZNF440;ZNF442;ZNF443;ZNF446;ZNF449;ZNF45;ZNF460;ZNF468;ZNF479;ZNF484;ZNF490;ZNF500;ZNF502;ZNF524;ZNF525;ZNF528;ZNF543;ZNF544;ZNF546;ZNF547;ZNF548;ZNF549;ZNF554;ZNF555;ZNF557;ZNF558;ZNF559;ZNF561;ZNF562;ZNF563;ZNF564;ZNF566;ZNF567;ZNF568;ZNF57;ZNF570;ZNF571;ZNF572;ZNF577;ZNF581;ZNF582;ZNF583;ZNF585A;ZNF586;ZNF589;ZNF595;ZNF599;ZNF600;ZNF605;ZNF607;ZNF611;ZNF613;ZNF614;ZNF615;ZNF616;ZNF619;ZNF620;ZNF621;ZNF625;ZNF627;ZNF649;ZNF652;ZNF653;ZNF665;ZNF667;ZNF669;ZNF670;ZNF672;ZNF679;ZNF680;ZNF683;ZNF689;ZNF692;ZNF701;ZNF705D;ZNF705E;ZNF705G;ZNF708;ZNF709;ZNF71;ZNF710;ZNF713;ZNF721;ZNF727;ZNF729;ZNF736;ZNF75A;ZNF75D;ZNF76;ZNF763;ZNF764;ZNF765;ZNF768;ZNF77;ZNF771;ZNF773;ZNF774;ZNF776;ZNF777;ZNF780A;ZNF780B;ZNF782;ZNF785;ZNF799;ZNF805;ZNF808;ZNF81;ZNF813;ZNF816;ZNF823;ZNF829;ZNF836;ZNF841;ZNF844;ZNF845;ZNF846;ZNF85;ZNF853;ZNF860;ZNF878;ZNF891;ZNF99;ZSCAN16;ZSCAN2;ZSCAN22;ZSCAN23;ZSCAN29;ZSCAN31;ZSCAN32;ZSCAN4;ZSCAN5A;ZSCAN5B;ZSCAN5C;ZSCAN9;ZXDA;ZXDB;ZXDC	
P53_HUMAN.H11MO.0.A	18	0.07058824	0.4208860	11.678.922	0.7227917	0.200239536	0.49862236	p53-related factors{6.3.1}	p53{6.3.1.0.1}	TP53;TP63;TP73	TP53
TBX4_HUMAN.H11MO.0.D	15	0.05882353	0.39553703	12.090.636	0.7181102	2,48E+05	0.5596881501	TBX2-related factors{6.5.4}	TBX4{6.5.4.0.3}	TBX2;TBX3;TBX4;TBX5	TBX4
NF2L2_HUMAN.H11MO.0.A	17	0.06666667	0.40973611	11.705.694	0.7154922	2,28E+05	0.5380286101	Jun-related factors{1.1.1}	NF-E2-like factors{1.1.1.2}	ATF2;ATF7;BACH1;BACH2;CREB5;JUN;JUNB;JUND;NFE2;NFE2L1;NFE2L2;NFE2L3	BACH1;BACH2;NFE2L1;NFE2L2;NFE2
GBX1_HUMAN.H11MO.0.D	9	0.03529412	0.32297200	13.805.586	0.7150242	3,83E+05	0.6838499330	HOX-related factors{3.1.1}	GBX (Gastrulation brain homeobox){3.1.1.11}	CDX1;CDX2;CDX4;EVX1;EVX2;GBX1;GBX2;GSX1;GSX2;HDX;HMBOX1;HNF1A;HNF1B;HOXA1;HOXA10;HOXA11;HOXA13;HOXA2;HOXA3;HOXA4;HOXA5;HOXA6;HOXA7;HOXA9;HOXB1;HOXB13;HOXB2;HOXB3;HOXB4;HOXB5;HOXB6;HOXB7;HOXB8;HOXB9;HOXC10;HOXC11;HOXC12;HOXC13;HOXC4;HOXC5;HOXC6;HOXC8;HOXC9;HOXD1;HOXD10;HOXD11;HOXD12;HOXD13;HOXD3;HOXD4;HOXD8;HOXD9;MEOX1;MEOX2;MNX1;PDX1;POU1F1;POU2F1;POU2F2;POU2F3;POU3F1;POU3F2;POU3F3;POU3F4;POU4F1;POU4F2;POU4F3;POU5F1;POU5F2;POU6F1;POU6F2	GBX1;GBX2
ZKSC1_HUMAN.H11MO.0.B	27	0.10588235	0.46054773	10.675.882	0.7144192	1,06E+05	0.3476407958	More than 3 adjacent zinc finger factors{2.3.3}	ZNF24-like factors{2.3.3.10}	BCL6;BCL6B;CTCF;CTCFL;FEZF1;FEZF2;GFI1;GFI1B;GLI1;GLI2;GLI3;GLI4;GLIS1;GLIS2;GLIS3;HKR1;MTF1;MYNN;MZF1;OSR2;OVOL1;OVOL2;PLAG1;PLAGL1;PLAGL2;PRDM1;PRDM14;PRDM6;SCRT1;SCRT2;SNAI1;SNAI2;SNAI3;WT1;YY1;YY2;ZBTB12;ZBTB14;ZBTB18;ZBTB20;ZBTB26;ZBTB42;ZBTB45;ZBTB47;ZBTB48;ZBTB49;ZBTB6;ZBTB7A;ZBTB7B;ZBTB7C;ZFP14;ZFP2;ZFP28;ZFP30;ZFP37;ZFP42;ZFP64;ZFP69;ZFP69B;ZFP82;ZFP91;ZFX;ZIC1;ZIC2;ZIC3;ZIC4;ZIC5;ZIK1;ZIM3;ZKSCAN1;ZKSCAN2;ZKSCAN3;ZKSCAN4;ZNF121;ZNF124;ZNF133;ZNF136;ZNF138;ZNF14;ZNF140;ZNF143;ZNF146;ZNF148;ZNF155;ZNF157;ZNF160;ZNF169;ZNF175;ZNF177;ZNF18;ZNF180;ZNF181;ZNF2;ZNF20;ZNF212;ZNF213;ZNF214;ZNF221;ZNF222;ZNF223;ZNF224;ZNF225;ZNF226;	ZKSCAN1;ZNF232;ZSCAN31

Motif	Nº of probes	% of probes	lower OR	upper OR	OR	p.value	FDR	TF family	TF subfamily	TF.family.member	TF.subfamily.member
										ZNF227;ZNF229;ZNF230;ZNF232;ZNF233;ZNF234;ZNF235;ZNF24;ZNF25;ZNF250;ZNF257;ZNF26;ZNF260;ZNF263;ZNF264;ZNF268;ZNF274;ZNF276;ZNF28;ZNF280A;ZNF280B;ZNF280C;ZNF280D;ZNF281;ZNF282;ZNF283;ZNF284;ZNF285;ZNF286A;ZNF286B;ZNF3;ZNF30;ZNF300;ZNF302;ZNF317;ZNF32;ZNF320;ZNF322;ZNF324;ZNF324B;ZNF329;ZNF331;ZNF333;ZNF33A;ZNF33B;ZNF343;ZNF345;ZNF347;ZNF350;ZNF354A;ZNF354B;ZNF362;ZNF366;ZNF383;ZNF384;ZNF394;ZNF397;ZNF398;ZNF404;ZNF41;ZNF410;ZNF419;ZNF420;ZNF431;ZNF432;ZNF436;ZNF439;ZNF44;ZNF440;ZNF442;ZNF443;ZNF446;ZNF449;ZNF45;ZNF460;ZNF468;ZNF479;ZNF484;ZNF490;ZNF500;ZNF502;ZNF524;ZNF525;ZNF528;ZNF543;ZNF544;ZNF546;ZNF547;ZNF548;ZNF549;ZNF554;ZNF555;ZNF557;ZNF558;ZNF559;ZNF561;ZNF562;ZNF563;ZNF564;ZNF566;ZNF567;ZNF568;ZNF57;ZNF570;ZNF571;ZNF572;ZNF577;ZNF581;ZNF582;ZNF583;ZNF585A;ZNF586;ZNF589;ZNF595;ZNF599;ZNF600;ZNF605;ZNF607;ZNF611;ZNF613;ZNF614;ZNF615;ZNF616;ZNF619;ZNF620;ZNF621;ZNF625;ZNF627;ZNF649;ZNF652;ZNF653;ZNF665;ZNF667;ZNF669;ZNF670;ZNF672;ZNF679;ZNF680;ZNF683;ZNF689;ZNF692;ZNF701;ZNF705D;ZNF705E;ZNF705G;ZNF708;ZNF709;ZNF71;ZNF710;ZNF713;ZNF721;ZNF727;ZNF729;ZNF736;ZNF75A;ZNF75D;ZNF76;ZNF763;ZNF764;ZNF765;ZNF768;ZNF77;ZNF771;ZNF773;ZNF774;ZNF776;ZNF777;ZNF780A;ZNF780B;ZNF782;ZNF785;ZNF799;ZNF805;ZNF808;ZNF81;ZNF813;ZNF816;ZNF823;ZNF829;ZNF836;ZNF841;ZNF844;ZNF845;ZNF846;ZNF85;ZNF853;ZNF860;ZNF878;ZNF891;ZNF99;ZSCAN16;ZSCAN2;ZSCAN22;ZSCAN23;ZSCAN29;ZSCAN31;ZSCAN32;ZSCAN4;ZSCAN5A;ZSCAN5B;ZSCAN5C;ZSCAN9;ZXDA;ZXDB;ZXDC	
CREM_HUMAN.H11MO.0.C	23	0.09019608	0.44314794	10.970.752	0.7132756	1,26E+05	0.3867481384	CREB-related factors{1.1.7}	CREB-like factors{1.1.7.1}	ATF1;ATF6;ATF6B;CREB1;CREB3;CREB3L1;CREB3L2;CREB3L3;CREB3L4;CREBL2;CREBZF;CREM	ATF1;CREB1;CREM
TAL1_HUMAN.H11MO.0.A	14	0.05490196	0.38362463	12.197.580	0.7125727	2,83E+05	0.5950593214	Tal-related factors{1.2.3}	Tal / HEN-like factors{1.2.3.1}	ATOH1;ATOH7;ATOH8;BHLHA15;BHLHA9;BHLHE22;BHLHE23;FERD3L;FIGLA;HAND1;HAND2;LYL1;MESP1;MESP2;MSC;MSGN1;NEUROD1;NEUROD2;NEUROD4;NEUROD6;NEUROG1;NEUROG2;NEUROG3;NHLH1;NHLH2;OLIG1;OLIG2;OLIG3;PTF1A;SCX;TAL1;TAL2;TCF15;TCF21;TCF23;TWIST1;TWIST2	NHLH1;LYL1;TAL1
TBX21_HUMAN.H11MO.0.A	42	0.16470588	0.49898922	0.9961415	0.7125598	4,75E+04	0.2036721520	TBrain-related factors{6.5.2}	TBX21 (T-bet){6.5.2.0.3}	EOMES;TBR1;TBX21	TBX21
SHOX_HUMAN.H11MO.0.D	9	0.03529412	0.32178910	13.756.140	0.7124102	3,83E+05	0.6838499330	Paired-related HD factors{3.1.3}	SHOX{3.1.3.25}	ALX1;ALX3;ALX4;ARGFX;ARX;CRX;DMBX1;DPRX;DRGX;DUX4;DUXA;ESX1;GSC;GSC2;HESX1;ISX;LEUTX;MIXL1;NOBOX;OTP;OTX1;OTX2;PHOX2A;PHOX2B;PITX1;PITX2;PITX3;PROP1;PRRX1;PRRX2;RAX;RAX2;RHOXF1;RHOXF2;SEBOX;SHOX;SHOX2;TPRX1;UNCX;VSX1;VSX2	SHOX2;SHOX
ZFP28_HUMAN.H11MO.0.C	30	0.11764706	0.46932997	10.456.057	0.7121039	8,51E+04	0.2968616474	More than 3 adjacent zinc finger factors{2.3.3}	unclassified{2.3.3.0}	BCL6B;BCL6;CTCFL;CTCF;FEZF1;GFI1B;GFI1;GLI1;GLI2;GLI3;GLIS1;GLIS2;GLIS3;MTF1;MYNN;MZF1;OSR2;OVOL1;OVOL2;ZNF146;PLAG1;PLAGL1;PRDM14;PRDM1;PRDM6;SCRT1;SCRT2;SNAI1;SNAI2;YY1;YY2;WT1;ZNF324;ZNF354A;ZBTB14;ZBTB18;ZBTB48;ZBTB49;ZBTB7A;ZBTB7B;ZBTB6;ZFP64;ZFP28;ZFP42;ZFP82;ZFX;ZIC1;ZIC2;ZIC3;ZIC4;ZIM3;ZKSCAN1;ZKSCAN3;ZNF121;ZNF136;ZNF140;ZNF143;ZNF148;ZNF214;ZNF232;ZNF250;ZNF257;ZNF260;ZNF263;ZNF264;ZNF274;ZNF281;ZNF282;ZNF317;ZNF320;ZNF322;ZNF329;ZNF331;ZNF333;ZNF350;ZNF384;ZNF394;ZNF410;ZNF436;ZNF449;ZNF490;ZNF502;ZNF524;ZNF528;ZNF547;ZNF549;ZNF554;ZNF563;ZNF582;ZNF586;ZNF589;ZNF652;ZNF667;ZNF680;ZNF708;ZNF713;ZNF768;ZNF816;ZNF18;ZNF41;ZNF76;ZNF85;ZSCAN16;ZSCAN22;ZSCAN31;ZSCAN4	MYNN;MZF1;OSR2;PRDM14;PRDM6;WT1;ZBTB14;ZBTB48;ZBTB49;ZFP64;ZFP28;ZIM3;ZNF121;ZNF250;ZNF257;ZNF263;ZNF274;ZNF317;ZNF320;ZNF329;ZNF331;ZNF394;ZNF449;ZNF502;ZNF528;ZNF547;ZNF549;ZNF554;ZNF586;ZNF589;ZNF667;ZNF680;ZNF708;ZNF713;ZNF768;ZNF18;ZNF85;ZSCAN16;ZSCAN22
LHX6_HUMAN.H11MO.0.D	8	0.03137255	0.30344755	14.241.958	0.7110787	4,41E+05	0.7232160222	HD-LIM factors{3.1.5}	Lhx-6-like factors{3.1.5.5}	ISL1;ISL2;LHX1;LHX2;LHX3;LHX4;LHX5;LHX6;LHX8;LHX9;LMX1A;LMX1B	LHX6;LHX8

Motif	Nº of probes	% of probes	lower OR	upper OR	OR	p.value	FDR	TF family	TF subfamily	TF.family.member	TF.subfamily.member
NRL_HUMAN.H11MO.0.D	22	0.08627451	0.43556347	10.994.021	0.7087611	1,43E+05	0.4185244156	Maf-related factors{1.1.3}	Large Maf factors{1.1.3.1}	MAF;MAFA;MAFB;MAFF;MAFG;MAFK;NRL	MAFA;MAFB;MAF;NRL
PROP1_HUMAN.H11MO.0.D	9	0.03529412	0.31934313	13.651.494	0.7070029	3,83E+05	0.6838499330	Paired-related HD factors{3.1.3}	PROP{3.1.3.20}	ALX1;ALX3;ALX4;ARGFX;ARX;CRX;DMBX1;DPRX;DRGX;DUX4;DUXA;ESX1;GSC;GSC2;HESX1;ISX;LEUTX;MIXL1;NOBOX;OTP;OTX1;OTX2;PHOX2A;PHOX2B;PITX1;PITX2;PITX3;PROP1;PRRX1;PRRX2;RAX;RAX2;RHOXF1;RHOXF2;SEBOX;SHOX;SHOX2;TPRX1;UNCX;VSX1;VSX2	PROP1
HLF_HUMAN.H11MO.0.C	9	0.03529412	0.31929036	13.649.237	0.7068862	3,83E+05	0.6838499330	C/EBP-related{1.1.8}	PAR factors{1.1.8.2}	CEBPA;CEBPB;CEBPD;CEBPE;CEBPG;DBP;DDIT3;HLF;NFIL3;TEF	DBP;HLF;NFIL3;TEF
HXC8_HUMAN.H11MO.0.D	7	0.02745098	0.28087402	14.781.286	0.7062565	5,11E+05	0.7787856458	HOX-related factors{3.1.1}	HOX8{3.1.1.7}	CDX1;CDX2;CDX4;EVX1;EVX2;GBX1;GBX2;GSX1;GSX2;HDX;HMBOX1;HNF1A;HNF1B;HOXA1;HOXA10;HOXA11;HOXA13;HOXA2;HOXA3;HOXA4;HOXA5;HOXA6;HOXA7;HOXA9;HOXB1;HOXB13;HOXB2;HOXB3;HOXB4;HOXB5;HOXB6;HOXB7;HOXB8;HOXB9;HOXC10;HOXC11;HOXC12;HOXC13;HOXC4;HOXC5;HOXC6;HOXC8;HOXC9;HOXD1;HOXD10;HOXD11;HOXD12;HOXD13;HOXD3;HOXD4;HOXD8;HOXD9;MEOX1;MEOX2;MNX1;PDX1;POU1F1;POU2F1;POU2F2;POU2F3;POU3F1;POU3F2;POU3F3;POU3F4;POU4F1;POU4F2;POU4F3;POU5F1;POU5F2;POU6F1;POU6F2	HOXB8;HOXC8;HOXD8
ZSC16_HUMAN.H11MO.0.D	13	0.05098039	0.37048052	12.313.050	0.7061944	2,70E+05	0.5714541470	More than 3 adjacent zinc finger factors{2.3.3}	unclassified{2.3.3.0}	BCL6B;BCL6;CTCFL;CTCF;FEZF1;GFI1B;GFI1;GLI1;GLI2;GLI3;GLIS1;GLIS2;GLIS3;MTF1;MYNN;MZF1;OSR2;OVOL1;OVOL2;ZNF146;PLAG1;PLAGL1;PRDM14;PRDM1;PRDM6;SCRT1;SCRT2;SNAI1;SNAI2;YY1;YY2;WT1;ZNF324;ZNF354A;ZBTB14;ZBTB18;ZBTB48;ZBTB49;ZBTB7A;ZBTB7B;ZBTB6;ZFP64;ZFP28;ZFP42;ZFP82;ZFX;ZIC1;ZIC2;ZIC3;ZIC4;ZIM3;ZKSCAN1;ZKSCAN3;ZNF121;ZNF136;ZNF140;ZNF143;ZNF148;ZNF214;ZNF232;ZNF250;ZNF257;ZNF260;ZNF263;ZNF264;ZNF274;ZNF281;ZNF282;ZNF317;ZNF320;ZNF322;ZNF329;ZNF331;ZNF333;ZNF350;ZNF384;ZNF394;ZNF410;ZNF436;ZNF449;ZNF490;ZNF502;ZNF524;ZNF528;ZNF547;ZNF549;ZNF554;ZNF563;ZNF582;ZNF586;ZNF589;ZNF652;ZNF667;ZNF680;ZNF708;ZNF713;ZNF768;ZNF816;ZNF18;ZNF41;ZNF76;ZNF85;ZSCAN16;ZSCAN22;ZSCAN31;ZSCAN4	MYNN;MZF1;OSR2;PRDM14;PRDM6;WT1;ZBTB14;ZBTB48;ZBTB49;ZFP64;ZFP28;ZIM3;ZNF121;ZNF250;ZNF257;ZNF263;ZNF274;ZNF317;ZNF320;ZNF329;ZNF331;ZNF394;ZNF449;ZNF502;ZNF528;ZNF547;ZNF549;ZNF554;ZNF586;ZNF589;ZNF667;ZNF680;ZNF708;ZNF713;ZNF768;ZNF18;ZNF85;ZSCAN16;ZSCAN22
TBX19_HUMAN.H11MO.0.D	10	0.03921569	0.33369072	13.195.107	0.7049621	3,34E+05	0.6520406288	Brachyury-related factors{6.5.1}	TBX19{6.5.1.0.2}	T;TBX19	TBX19
SCRT2_HUMAN.H11MO.0.D	13	0.05098039	0.36961111	12.284.091	0.7045377	2,70E+05	0.5714541470	More than 3 adjacent zinc finger factors{2.3.3}	Snail-like factors{2.3.3.2}	BCL6;BCL6B;CTCF;CTCFL;FEZF1;FEZF2;GFI1;GFI1B;GLI1;GLI2;GLI3;GLI4;GLIS1;GLIS2;GLIS3;HKR1;MTF1;MYNN;MZF1;OSR2;OVOL1;OVOL2;PLAG1;PLAGL1;PLAGL2;PRDM1;PRDM14;PRDM6;SCRT1;SCRT2;SNAI1;SNAI2;SNAI3;WT1;YY1;YY2;ZBTB12;ZBTB14;ZBTB18;ZBTB20;ZBTB26;ZBTB42;ZBTB45;ZBTB47;ZBTB48;ZBTB49;ZBTB6;ZBTB7A;ZBTB7B;ZBTB7C;ZFP14;ZFP2;ZFP28;ZFP30;ZFP37;ZFP42;ZFP64;ZFP69;ZFP69B;ZFP82;ZFP91;ZFX;ZIC1;ZIC2;ZIC3;ZIC4;ZIC5;ZIK1;ZIM3;ZKSCAN1;ZKSCAN2;ZKSCAN3;ZKSCAN4;ZNF121;ZNF124;ZNF133;ZNF136;ZNF138;ZNF14;ZNF140;ZNF143;ZNF146;ZNF148;ZNF155;ZNF157;ZNF160;ZNF169;ZNF175;ZNF177;ZNF18;ZNF180;ZNF181;ZNF2;ZNF20;ZNF212;ZNF213;ZNF214;ZNF221;ZNF222;ZNF223;ZNF224;ZNF225;ZNF226;ZNF227;ZNF229;ZNF230;ZNF232;ZNF233;ZNF234;ZNF235;ZNF24;ZNF25;ZNF250;ZNF257;ZNF26;ZNF260;ZNF263;ZNF264;ZNF268;ZNF274;ZNF276;ZNF28;ZNF280A;ZNF280B;ZNF280C;ZNF280D;ZNF281;ZNF282;ZNF283;ZNF284;ZNF285;ZNF286A;ZNF286B;ZNF3;ZNF30;ZNF300;ZNF302;ZNF317;ZNF32;ZNF320;ZNF322;ZNF324;ZNF324B;ZNF329;ZNF331;ZNF333;ZNF33A;ZNF33B;ZNF343;ZNF345;ZNF347;ZNF350;ZNF354A;ZNF354B;ZNF362;ZNF366;ZNF383;ZNF384;ZNF394;ZNF397;ZNF398;ZNF404;ZNF41;ZNF410;ZNF419;ZNF420;ZNF431;ZNF432;ZNF436;ZNF439;ZNF44;ZNF440;ZNF442;ZNF443;ZNF446;ZNF449;ZNF45;ZNF460;ZNF468;ZNF479;ZNF484;ZNF490;ZNF500;ZNF502;ZNF524;ZNF525;ZNF528;ZNF543;ZNF544;ZNF546;ZNF547;ZNF548;ZNF549;ZNF554;ZNF555;ZNF557;ZNF558;ZNF559;ZNF561;ZNF562;ZNF563;ZNF564;ZNF566;ZN	SCRT1;SCRT2;SNAI1;SNAI2

Motif	Nº of probes	% of probes	lower OR	upper OR	OR	p.value	FDR	TF family	TF subfamily	TF.family.member	TF.subfamily.member
										F567;ZNF568;ZNF57;ZNF570;ZNF571;ZNF572;ZNF577;ZNF581;ZNF582;ZNF583;ZNF585A;ZNF586;ZNF589;ZNF595;ZNF599;ZNF600;ZNF605;ZNF607;ZNF611;ZNF613;ZNF614;ZNF615;ZNF616;ZNF619;ZNF620;ZNF621;ZNF625;ZNF627;ZNF649;ZNF652;ZNF653;ZNF665;ZNF667;ZNF669;ZNF670;ZNF672;ZNF679;ZNF680;ZNF683;ZNF689;ZNF692;ZNF701;ZNF705D;ZNF705E;ZNF705G;ZNF708;ZNF709;ZNF71;ZNF710;ZNF713;ZNF721;ZNF727;ZNF729;ZNF736;ZNF75A;ZNF75D;ZNF76;ZNF763;ZNF764;ZNF765;ZNF768;ZNF77;ZNF771;ZNF773;ZNF774;ZNF776;ZNF777;ZNF780A;ZNF780B;ZNF782;ZNF785;ZNF799;ZNF805;ZNF808;ZNF81;ZNF813;ZNF816;ZNF823;ZNF829;ZNF836;ZNF841;ZNF844;ZNF845;ZNF846;ZNF85;ZNF853;ZNF860;ZNF878;ZNF891;ZNF99;ZSCAN16;ZSCAN2;ZSCAN22;ZSCAN23;ZSCAN29;ZSCAN31;ZSCAN32;ZSCAN4;ZSCAN5A;ZSCAN5B;ZSCAN5C;ZSCAN9;ZXDA;ZXDB;ZXDC	
UNC4_HUMAN.H11MO.0.D	9	0.03529412	0.31707037	13.555.078	0.7020183	3,84E+05	0.6838499330	Paired-related HD factors{3.1.3}	UNCX{3.1.3.27}	ALX1;ALX3;ALX4;ARGFX;ARX;CRX;DMBX1;DPRX;DRGX;DUX4;DUXA;ESX1;GSC;GSC2;HESX1;ISX;LEUTX;MIXL1;NOBOX;OTP;OTX1;OTX2;PHOX2A;PHOX2B;PITX1;PITX2;PITX3;PROP1;PRRX1;PRRX2;RAX;RAX2;RHOXF1;RHOXF2;SEBOX;SHOX;SHOX2;TPRX1;UNCX;VSX1;VSX2	UNCX
JDP2_HUMAN.H11MO.0.D	16	0.06274510	0.39315215	11.598.841	0.6993344	1,83E+05	0.4683207170	Fos-related factors{1.1.2}	ATF-3-like factors{1.1.2.2}	ATF3;FOS;FOSB;FOSL1;FOSL2;JDP2	ATF3;JDP2
PAX1_HUMAN.H11MO.0.D	17	0.06666667	0.39960029	11.415.664	0.6977561	1,61E+05	0.4414776601	Paired domain only{3.2.2}	PAX-1/9 (no homeo remnant){3.2.2.1}	PAX1;PAX2;PAX5;PAX8;PAX9	PAX1
TYY2_HUMAN.H11MO.0.D	21	0.08235294	0.42312666	10.906.230	0.6967445	1,15E+05	0.3640206229	More than 3 adjacent zinc finger factors{2.3.3}	YY1-like factors{2.3.3.9}	BCL6;BCL6B;CTCF;CTCFL;FEZF1;FEZF2;GFI1;GFI1B;GLI1;GLI2;GLI3;GLI4;GLIS1;GLIS2;GLIS3;HKR1;MTF1;MYNN;MZF1;OSR2;OVOL1;OVOL2;PLAG1;PLAGL1;PLAGL2;PRDM1;PRDM14;PRDM6;SCRT1;SCRT2;SNAI1;SNAI2;SNAI3;WT1;YY1;YY2;ZBTB12;ZBTB14;ZBTB18;ZBTB20;ZBTB26;ZBTB42;ZBTB45;ZBTB47;ZBTB48;ZBTB49;ZBTB6;ZBTB7A;ZBTB7B;ZBTB7C;ZFP14;ZFP2;ZFP28;ZFP30;ZFP37;ZFP42;ZFP64;ZFP69;ZFP69B;ZFP82;ZFP91;ZFX;ZIC1;ZIC2;ZIC3;ZIC4;ZIC5;ZIK1;ZIM3;ZKSCAN1;ZKSCAN2;ZKSCAN3;ZKSCAN4;ZNF121;ZNF124;ZNF133;ZNF136;ZNF138;ZNF14;ZNF140;ZNF143;ZNF146;ZNF148;ZNF155;ZNF157;ZNF160;ZNF169;ZNF175;ZNF177;ZNF18;ZNF180;ZNF181;ZNF2;ZNF20;ZNF212;ZNF213;ZNF214;ZNF221;ZNF222;ZNF223;ZNF224;ZNF225;ZNF226;ZNF227;ZNF229;ZNF230;ZNF232;ZNF233;ZNF234;ZNF235;ZNF24;ZNF25;ZNF250;ZNF257;ZNF26;ZNF260;ZNF263;ZNF264;ZNF268;ZNF274;ZNF276;ZNF28;ZNF280A;ZNF280B;ZNF280C;ZNF280D;ZNF281;ZNF282;ZNF283;ZNF284;ZNF285;ZNF286A;ZNF286B;ZNF3;ZNF30;ZNF300;ZNF302;ZNF317;ZNF32;ZNF320;ZNF322;ZNF324;ZNF324B;ZNF329;ZNF331;ZNF333;ZNF33A;ZNF33B;ZNF343;ZNF345;ZNF347;ZNF350;ZNF354A;ZNF354B;ZNF362;ZNF366;ZNF383;ZNF384;ZNF394;ZNF397;ZNF398;ZNF404;ZNF41;ZNF410;ZNF419;ZNF420;ZNF431;ZNF432;ZNF436;ZNF439;ZNF44;ZNF440;ZNF442;ZNF443;ZNF446;ZNF449;ZNF45;ZNF460;ZNF468;ZNF479;ZNF484;ZNF490;ZNF500;ZNF502;ZNF524;ZNF525;ZNF528;ZNF543;ZNF544;ZNF546;ZNF547;ZNF548;ZNF549;ZNF554;ZNF555;ZNF557;ZNF558;ZNF559;ZNF561;ZNF562;ZNF563;ZNF564;ZNF566;ZNF567;ZNF568;ZNF57;ZNF570;ZNF571;ZNF572;ZNF577;ZNF581;ZNF582;ZNF583;ZNF585A;ZNF586;ZNF589;ZNF595;ZNF599;ZNF600;ZNF605;ZNF607;ZNF611;ZNF613;ZNF614;ZNF615;ZNF616;ZNF619;ZNF620;ZNF621;ZNF625;ZNF627;ZNF649;ZNF652;ZNF653;ZNF665;ZNF667;ZNF669;ZNF670;ZNF672;ZNF679;ZNF680;ZNF683;ZNF689;ZNF692;ZNF701;ZNF705D;ZNF705E;ZNF705G;ZNF708;ZNF709;ZNF71;ZNF710;ZNF713;ZNF721;ZNF727;ZNF729;ZNF736;ZNF75A;ZNF75D;ZNF76;ZNF763;ZNF764;ZNF765;ZNF768;ZNF77;ZNF771;ZNF773;ZNF774;ZNF776;ZNF777;ZNF780A;ZNF780B;ZNF782;ZNF785;ZNF799;ZNF805;ZNF808;ZNF81;ZNF813;ZNF816;ZNF823;ZNF829;ZNF836;ZNF841;ZNF844;ZNF845;ZNF846;ZNF85;ZNF853;Z	YY1;YY2;ZFP42

Motif	Nº of probes	% of probes	lower OR	upper OR	OR	p.value	FDR	TF family	TF subfamily	TF.family.member	TF.subfamily.member
										NF860;ZNF878;ZNF891;ZNF99;ZSCAN16;ZSCAN2;ZSCAN22;ZSCAN23;ZSCAN29;ZSCAN31;ZSCAN32;ZSCAN4;ZSCAN5A;ZSCAN5B;ZSCAN5C;ZSCAN9;ZXDA;ZXDB;ZXDC	
PIT1_HUMAN.H11MO.0.C	11	0.04313725	0.34281063	12.684.952	0.6961253	2,93E+05	0.6028388854	POU domain factors{3.1.10}	POU1 (Pit-1-like factors){3.1.10.1}	CDX1;CDX2;CDX4;EVX1;EVX2;GBX1;GBX2;GSX1;GSX2;HDX;HMBOX1;HNF1A;HNF1B;HOXA1;HOXA10;HOXA11;HOXA13;HOXA2;HOXA3;HOXA4;HOXA5;HOXA6;HOXA7;HOXA9;HOXB1;HOXB13;HOXB2;HOXB3;HOXB4;HOXB5;HOXB6;HOXB7;HOXB8;HOXB9;HOXC10;HOXC11;HOXC12;HOXC13;HOXC4;HOXC5;HOXC6;HOXC8;HOXC9;HOXD1;HOXD10;HOXD11;HOXD12;HOXD13;HOXD3;HOXD4;HOXD8;HOXD9;MEOX1;MEOX2;MNX1;PDX1;POU1F1;POU2F1;POU2F2;POU2F3;POU3F1;POU3F2;POU3F3;POU3F4;POU4F1;POU4F2;POU4F3;POU5F1;POU5F1B;POU5F2;POU6F1;POU6F2	POU1F1
GATA1_HUMAN.H11MO.0.A	12	0.04705882	0.35376147	12.361.329	0.6944464	2,56E+05	0.5648163191	GATA-type zinc fingers{2.2.1}	Two zinc-finger GATA factors{2.2.1.1}	GATA1;GATA2;GATA3;GATA4;GATA5;GATA6;GATAD2A;GATAD2B;TRPS1;ZGLP1	GATA1;GATA2;GATA3;GATA4;GATA5;GATA6
PO4F2_HUMAN.H11MO.0.D	9	0.03529412	0.31317236	13.387.876	0.6933699	3,86E+05	0.6838499330	POU domain factors{3.1.10}	POU4 (Brn-3-like factors){3.1.10.4}	CDX1;CDX2;CDX4;EVX1;EVX2;GBX1;GBX2;GSX1;GSX2;HDX;HMBOX1;HNF1A;HNF1B;HOXA1;HOXA10;HOXA11;HOXA13;HOXA2;HOXA3;HOXA4;HOXA5;HOXA6;HOXA7;HOXA9;HOXB1;HOXB13;HOXB2;HOXB3;HOXB4;HOXB5;HOXB6;HOXB7;HOXB8;HOXB9;HOXC10;HOXC11;HOXC12;HOXC13;HOXC4;HOXC5;HOXC6;HOXC8;HOXC9;HOXD1;HOXD10;HOXD11;HOXD12;HOXD13;HOXD3;HOXD4;HOXD8;HOXD9;MEOX1;MEOX2;MNX1;PDX1;POU1F1;POU2F1;POU2F2;POU2F3;POU3F1;POU3F2;POU3F3;POU3F4;POU4F1;POU4F2;POU4F3;POU5F1;POU5F1B;POU5F2;POU6F1;POU6F2	POU4F1;POU4F2;POU4F3
HXD11_HUMAN.H11MO.0.D	13	0.05098039	0.36352494	12.081.367	0.6928991	2,25E+05	0.5368495959	HOX-related factors{3.1.1}	HOX9-13{3.1.1.8}	CDX1;CDX2;CDX4;EVX1;EVX2;GBX1;GBX2;GSX1;GSX2;HDX;HMBOX1;HNF1A;HNF1B;HOXA1;HOXA10;HOXA11;HOXA13;HOXA2;HOXA3;HOXA4;HOXA5;HOXA6;HOXA7;HOXA9;HOXB1;HOXB13;HOXB2;HOXB3;HOXB4;HOXB5;HOXB6;HOXB7;HOXB8;HOXB9;HOXC10;HOXC11;HOXC12;HOXC13;HOXC4;HOXC5;HOXC6;HOXC8;HOXC9;HOXD1;HOXD10;HOXD11;HOXD12;HOXD13;HOXD3;HOXD4;HOXD8;HOXD9;MEOX1;MEOX2;MNX1;PDX1;POU1F1;POU2F1;POU2F2;POU2F3;POU3F1;POU3F2;POU3F3;POU3F4;POU4F1;POU4F2;POU4F3;POU5F1;POU5F2;POU6F1;POU6F2	HOXA10;HOXA11;HOXA13;HOXA9;HOXB13;HOXC10;HOXC11;HOXC12;HOXC13;HOXC9;HOXD10;HOXD11;HOXD12;HOXD13;HOXD9
HXC9_HUMAN.H11MO.0.C	9	0.03529412	0.31221155	13.346.698	0.6912394	3,17E+05	0.6296813490	HOX-related factors{3.1.1}	HOX9-13{3.1.1.8}	CDX1;CDX2;CDX4;EVX1;EVX2;GBX1;GBX2;GSX1;GSX2;HDX;HMBOX1;HNF1A;HNF1B;HOXA1;HOXA10;HOXA11;HOXA13;HOXA2;HOXA3;HOXA4;HOXA5;HOXA6;HOXA7;HOXA9;HOXB1;HOXB13;HOXB2;HOXB3;HOXB4;HOXB5;HOXB6;HOXB7;HOXB8;HOXB9;HOXC10;HOXC11;HOXC12;HOXC13;HOXC4;HOXC5;HOXC6;HOXC8;HOXC9;HOXD1;HOXD10;HOXD11;HOXD12;HOXD13;HOXD3;HOXD4;HOXD8;HOXD9;MEOX1;MEOX2;MNX1;PDX1;POU1F1;POU2F1;POU2F2;POU2F3;POU3F1;POU3F2;POU3F3;POU3F4;POU4F1;POU4F2;POU4F3;POU5F1;POU5F2;POU6F1;POU6F2	HOXA10;HOXA11;HOXA13;HOXA9;HOXB13;HOXC10;HOXC11;HOXC12;HOXC13;HOXC9;HOXD10;HOXD11;HOXD12;HOXD13;HOXD9
NKX23_HUMAN.H11MO.0.D	10	0.03921569	0.32590291	12.886.774	0.6884978	3,37E+05	0.6520406288	NK-related factors{3.1.2}	NK-4{3.1.2.17}	BARHL1;BARHL2;BARX1;BARX2;BSX;DBX1;DBX2;DLX1;DLX2;DLX3;DLX4;DLX5;DLX6;EMX1;EMX2;EN1;EN2;HHEX;HLX;HMX1;HMX2;HMX3;LBX1;LBX2;MSX1;MSX2;NANOG;NKX1-1;NKX1-2;NKX2-1;NKX2-2;NKX2-3;NKX2-4;NKX2-5;NKX2-6;NKX2-8;NKX3-1;NKX3-2;NKX6-1;NKX6-2;NKX6-3;NOTO;TLX1;TLX2;TLX3;VAX1;VAX2;VENTX	NKX2-3;NKX2-5
ALX4_HUMAN.H11MO.0.D	9	0.03529412	0.31040594	13.269.342	0.6872365	3,17E+05	0.6296813490	Paired-related HD factors{3.1.3}	ALX{3.1.3.1}	ALX1;ALX3;ALX4;ARGFX;ARX;CRX;DMBX1;DPRX;DRGX;DUX4;DUXA;ESX1;GSC;GSC2;HESX1;ISX;LEUTX;MIXL1;NOBOX;OTP;OTX1;OTX2;PHOX2A;PHOX2B;PITX1;PITX2;PITX3;PROP1;PRRX1;PRRX2;RAX;RAX2;RHOXF1;RHOXF2;SEBOX;SHOX;SHOX2;TPRX1;UNCX;VSX1;VSX2	ALX1;ALX3;ALX4
NFAC3_HUMAN.H11MO.0.B	12	0.04705882	0.34920138	12.201.305	0.6854710	2,58E+05	0.5648163191	NFAT-related factors{6.1.3}	NFATc3 (NFATx){6.1.3.0.3}	NFAT5;NFATC1;NFATC2;NFATC3;NFATC4	NFATC3

Motif	Nº of probes	% of probes	lower OR	upper OR	OR	p.value	FDR	TF family	TF subfamily	TF.family.member	TF.subfamily.member
AIRE_HUMAN.H11MO.0.C	7	0.02745098	0.27228021	14.328.364	0.6846283	4,20E+05	0.7063751650	AIRE{5.3.1}	AIRE{5.3.1.0.1}	AIRE	AIRE
ZN382_HUMAN.H11MO.0.C	15	0.05882353	0.37657928	11.510.274	0.6836945	1,74E+05	0.4652048204	Factors with multiple dispersed zinc fingers{2.3.4}	ZNF37A-like factors{2.3.4.25}	BCL11A;BCL11B;BNC1;BNC2;E4F1;HIC1;HIC2;HINFP;HIVEP1;HIVEP2;HIVEP3;IKZF1;IKZF2;IKZF3;IKZF4;IKZF5;INSM1;INSM2;MAZ;MECOM;PATZ1;PRDM16;PRDM4;REST;RLF;RREB1;SALL1;SALL2;SALL3;SALL4;VEZF1;ZBTB1;ZBTB17;ZBTB2;ZBTB25;ZBTB4;ZFAT;ZNF134;ZNF211;ZNF217;ZNF219;ZNF248;ZNF256;ZNF292;ZNF296;ZNF319;ZNF334;ZNF335;ZNF341;ZNF37A;ZNF382;ZNF417;ZNF418;ZNF423;ZNF467;ZNF510;ZNF512;ZNF512B;ZNF516;ZNF518A;ZNF518B;ZNF521;ZNF526;ZNF532;ZNF536;ZNF552;ZNF574;ZNF587;ZNF587B;ZNF592;ZNF639;ZNF654;ZNF658;ZNF671;ZNF687;ZNF711;ZNF717;ZNF770;ZNF772;ZNF784;ZNF786;ZNF792;ZNF8;ZNF814	ZNF382
GATA2_HUMAN.H11MO.1.A	12	0.04705882	0.34757440	12.144.268	0.6822759	2,58E+05	0.5648163191	GATA-type zinc fingers{2.2.1}	Two zinc-finger GATA factors{2.2.1.1}	GATA1;GATA2;GATA3;GATA4;GATA5;GATA6;GATAD2A;GATAD2B;TRPS1;ZGLP1	GATA1;GATA2;GATA3;GATA4;GATA5;GATA6
NR1I2_HUMAN.H11MO.0.C	12	0.04705882	0.34549720	12.072.270	0.6781967	2,13E+05	0.5124758438	Thyroid hormone receptor-related factors (NR1){2.1.2}	Vitamin D receptor (NR1I){2.1.2.4}	NR1D1;NR1D2;NR1H2;NR1H3;NR1H4;NR1I2;NR1I3;PPARA;PPARD;PPARG;RARA;RARB;RARG;RORA;RORB;RORC;THRA;THRB;VDR	NR1I2;NR1I3;VDR
ZN333_HUMAN.H11MO.0.D	8	0.03137255	0.28936610	13.580.947	0.6780849	3,65E+05	0.6684336053	More than 3 adjacent zinc finger factors{2.3.3}	ZNF177-like factors{2.3.3.40}	BCL6;BCL6B;CTCF;CTCFL;FEZF1;FEZF2;GFI1;GFI1B;GLI1;GLI2;GLI3;GLI4;GLIS1;GLIS2;GLIS3;HKR1;MTF1;MYNN;MZF1;OSR2;OVOL1;OVOL2;PLAG1;PLAGL1;PLAGL2;PRDM1;PRDM14;PRDM6;SCRT1;SCRT2;SNAI1;SNAI2;SNAI3;WT1;YY1;YY2;ZBTB12;ZBTB14;ZBTB18;ZBTB20;ZBTB26;ZBTB42;ZBTB45;ZBTB47;ZBTB48;ZBTB49;ZBTB6;ZBTB7A;ZBTB7B;ZBTB7C;ZFP14;ZFP2;ZFP28;ZFP30;ZFP37;ZFP42;ZFP64;ZFP69;ZFP69B;ZFP82;ZFP91;ZFX;ZIC1;ZIC2;ZIC3;ZIC4;ZIC5;ZIK1;ZIM3;ZKSCAN1;ZKSCAN2;ZKSCAN3;ZKSCAN4;ZNF121;ZNF124;ZNF133;ZNF136;ZNF138;ZNF14;ZNF140;ZNF143;ZNF146;ZNF148;ZNF155;ZNF157;ZNF160;ZNF169;ZNF175;ZNF177;ZNF18;ZNF180;ZNF181;ZNF2;ZNF20;ZNF212;ZNF213;ZNF214;ZNF221;ZNF222;ZNF223;ZNF224;ZNF225;ZNF226;ZNF227;ZNF229;ZNF230;ZNF232;ZNF233;ZNF234;ZNF235;ZNF24;ZNF25;ZNF250;ZNF257;ZNF26;ZNF260;ZNF263;ZNF264;ZNF268;ZNF274;ZNF276;ZNF28;ZNF280A;ZNF280B;ZNF280C;ZNF280D;ZNF281;ZNF282;ZNF283;ZNF284;ZNF285;ZNF286A;ZNF286B;ZNF3;ZNF30;ZNF300;ZNF302;ZNF317;ZNF32;ZNF320;ZNF322;ZNF324;ZNF324B;ZNF329;ZNF331;ZNF333;ZNF33A;ZNF33B;ZNF343;ZNF345;ZNF347;ZNF350;ZNF354A;ZNF354B;ZNF362;ZNF366;ZNF383;ZNF384;ZNF394;ZNF397;ZNF398;ZNF404;ZNF41;ZNF410;ZNF419;ZNF420;ZNF431;ZNF432;ZNF436;ZNF439;ZNF44;ZNF440;ZNF442;ZNF443;ZNF446;ZNF449;ZNF45;ZNF460;ZNF468;ZNF479;ZNF484;ZNF490;ZNF500;ZNF502;ZNF524;ZNF525;ZNF528;ZNF543;ZNF544;ZNF546;ZNF547;ZNF548;ZNF549;ZNF554;ZNF555;ZNF557;ZNF558;ZNF559;ZNF561;ZNF562;ZNF563;ZNF564;ZNF566;ZNF567;ZNF568;ZNF57;ZNF570;ZNF571;ZNF572;ZNF577;ZNF581;ZNF582;ZNF583;ZNF585A;ZNF586;ZNF589;ZNF595;ZNF599;ZNF600;ZNF605;ZNF607;ZNF611;ZNF613;ZNF614;ZNF615;ZNF616;ZNF619;ZNF620;ZNF621;ZNF625;ZNF627;ZNF649;ZNF652;ZNF653;ZNF665;ZNF667;ZNF669;ZNF670;ZNF672;ZNF679;ZNF680;ZNF683;ZNF689;ZNF692;ZNF701;ZNF705D;ZNF705E;ZNF705G;ZNF708;ZNF709;ZNF71;ZNF710;ZNF713;ZNF721;ZNF727;ZNF729;ZNF736;ZNF75A;ZNF75D;ZNF76;ZNF763;ZNF764;ZNF765;ZNF768;ZNF77;ZNF771;ZNF773;ZNF774;ZNF776;ZNF777;ZNF780A;ZNF780B;ZNF782;ZNF785;ZNF799;ZNF805;ZNF808;ZNF81;ZNF813;ZNF816;ZNF823;ZNF829;ZNF836;ZNF841;ZNF844;ZNF845;ZNF846;ZNF85;ZNF853;ZNF860;ZNF878;ZNF891;ZNF99;ZSCAN16;ZSCAN2;ZSCAN22;ZSCAN23;ZSCAN29;ZSCAN31;ZSCAN32;ZSCAN4;ZSCAN5A;ZSCAN5B;ZSCAN5C;ZSCAN9;ZXDA;ZXDB;ZXDC	ZNF333

Motif	Nº of probes	% of probes	lower OR	upper OR	OR	p.value	FDR	TF family	TF subfamily	TF.family.member	TF.subfamily.member
PHX2B_HUMAN.H11MO.0.D	8	0.03137255	0.28895447	13.561.665	0.6771234	3,65E+05	0.6684336053	Paired-related HD factors{3.1.3}	PHOX{3.1.3.18}	ALX1;ALX3;ALX4;ARGFX;ARX;CRX;DMBX1;DPRX;DRGX;DUX4;DUXA;ESX1;GSC;GSC2;HESX1;ISX;LEUTX;MIXL1;NOBOX;OTP;OTX1;OTX2;PHOX2A;PHOX2B;PITX1;PITX2;PITX3;PROP1;PRRX1;PRRX2;RAX;RAX2;RHOXF1;RHOXF2;SEBOX;SHOX;SHOX2;TPRX1;UNCX;VSX1;VSX2	PHOX2A;PHOX2B
TGIF1_HUMAN.H11MO.0.A	16	0.06274510	0.37895397	11.179.034	0.6740497	1,54E+05	0.4349095331	TALE-type homeo domain factors{3.1.4}	TGIF{3.1.4.6}	IRX1;IRX2;IRX3;IRX4;IRX5;IRX6;MEIS1;MEIS2;MEIS3;MKX;PBX1;PBX2;PBX3;PKNOX1;PKNOX2;TGIF1;TGIF2;TGIF2LX;TGIF2LY	TGIF2LX;TGIF1;TGIF2
HXB4_HUMAN.H11MO.0.B	7	0.02745098	0.26639068	14.017.649	0.6697910	4,23E+05	0.7083743276	HOX-related factors{3.1.1}	HOX4{3.1.1.4}	CDX1;CDX2;CDX4;EVX1;EVX2;GBX1;GBX2;GSX1;GSX2;HDX;HMBOX1;HNF1A;HNF1B;HOXA1;HOXA10;HOXA11;HOXA13;HOXA2;HOXA3;HOXA4;HOXA5;HOXA6;HOXA7;HOXA9;HOXB1;HOXB13;HOXB2;HOXB3;HOXB4;HOXB5;HOXB6;HOXB7;HOXB8;HOXB9;HOXC10;HOXC11;HOXC12;HOXC13;HOXC4;HOXC5;HOXC6;HOXC8;HOXC9;HOXD1;HOXD10;HOXD11;HOXD12;HOXD13;HOXD3;HOXD4;HOXD8;HOXD9;MEOX1;MEOX2;MNX1;PDX1;POU1F1;POU2F1;POU2F2;POU2F3;POU3F1;POU3F2;POU3F3;POU3F4;POU4F1;POU4F2;POU4F3;POU5F1;POU5F2;POU6F1;POU6F2	HOXB4;HOXD4
COE1_HUMAN.H11MO.0.A	31	0.12156863	0.44354726	0.9765271	0.6686017	3,71E+04	0.1755779144	Early B-Cell Factor-related factors{6.1.5}	EBF1 (COE1){6.1.5.0.1}	EBF1;EBF2;EBF3;EBF4	EBF1
RXRG_HUMAN.H11MO.0.B	12	0.04705882	0.33761662	11.796.525	0.6627208	1,76E+05	0.4667040255	RXR-related receptors (NR2){2.1.3}	Retinoid X receptors (NR2B){2.1.3.1}	HNF4A;HNF4G;NR2C1;NR2C2;NR2E1;NR2E3;NR2F1;NR2F2;NR2F6;RXRA;RXRB;RXRG	RXRA;RXRB;RXRG
NR1H2_HUMAN.H11MO.0.D	10	0.03921569	0.31363244	12.399.782	0.6624903	2,28E+05	0.5380286101	Thyroid hormone receptor-related factors (NR1){2.1.2}	LXR (NR1H){2.1.2.7}	NR1D1;NR1D2;NR1H2;NR1H3;NR1H4;NR1I2;NR1I3;PPARA;PPARD;PPARG;RARA;RARB;RARG;RORA;RORB;RORC;THRA;THRB;VDR	NR1H2;NR1H3;NR1H4
IRX2_HUMAN.H11MO.0.D	26	0.10196078	0.42261129	0.9942582	0.6610228	5,05E+04	0.2126316484	TALE-type homeo domain factors{3.1.4}	IRX (Iroquois){3.1.4.1}	IRX1;IRX2;IRX3;IRX4;IRX5;IRX6;MEIS1;MEIS2;MEIS3;MKX;PBX1;PBX2;PBX3;PKNOX1;PKNOX2;TGIF1;TGIF2;TGIF2LX;TGIF2LY	IRX2;IRX3
CREB1_HUMAN.H11MO.0.A	19	0.07450980	0.38851600	10.496.692	0.6570892	8,65E+04	0.2999424695	CREB-related factors{1.1.7}	CREB-like factors{1.1.7.1}	ATF1;ATF6;ATF6B;CREB1;CREB3;CREB3L1;CREB3L2;CREB3L3;CREB3L4;CREBL2;CREBZF;CREM	ATF1;CREB1;CREM
ZN274_HUMAN.H11MO.0.A	15	0.05882353	0.36116539	11.038.367	0.6556839	1,19E+05	0.3738663298	More than 3 adjacent zinc finger factors{2.3.3}	unclassified{2.3.3.0}	BCL6;BCL6B;CTCF;CTCFL;FEZF1;FEZF2;GFI1;GFI1B;GLI1;GLI2;GLI3;GLI4;GLIS1;GLIS2;GLIS3;HKR1;MTF1;MYNN;MZF1;OSR2;OVOL1;OVOL2;PLAG1;PLAGL1;PLAGL2;PRDM1;PRDM14;PRDM6;SCRT1;SCRT2;SNAI1;SNAI2;SNAI3;WT1;YY1;YY2;ZBTB12;ZBTB14;ZBTB18;ZBTB20;ZBTB26;ZBTB42;ZBTB45;ZBTB47;ZBTB48;ZBTB49;ZBTB6;ZBTB7A;ZBTB7B;ZBTB7C;ZFP14;ZFP2;ZFP28;ZFP30;ZFP37;ZFP42;ZFP64;ZFP69;ZFP69B;ZFP82;ZFP91;ZFX;ZIC1;ZIC2;ZIC3;ZIC4;ZIC5;ZIK1;ZIM3;ZKSCAN1;ZKSCAN2;ZKSCAN3;ZKSCAN4;ZNF121;ZNF124;ZNF133;ZNF136;ZNF138;ZNF14;ZNF140;ZNF143;ZNF146;ZNF148;ZNF155;ZNF157;ZNF160;ZNF169;ZNF175;ZNF177;ZNF18;ZNF180;ZNF181;ZNF2;ZNF20;ZNF212;ZNF213;ZNF214;ZNF221;ZNF222;ZNF223;ZNF224;ZNF225;ZNF226;ZNF227;ZNF229;ZNF230;ZNF232;ZNF233;ZNF234;ZNF235;ZNF24;ZNF25;ZNF250;ZNF257;ZNF26;ZNF260;ZNF263;ZNF264;ZNF268;ZNF274;ZNF276;ZNF28;ZNF280A;ZNF280B;ZNF280C;ZNF280D;ZNF281;ZNF282;ZNF283;ZNF284;ZNF285;ZNF286A;ZNF286B;ZNF3;ZNF30;ZNF300;ZNF302;ZNF317;ZNF32;ZNF320;ZNF322;ZNF324;ZNF324B;ZNF329;ZNF331;ZNF333;ZNF33A;ZNF33B;ZNF343;ZNF345;ZNF347;ZNF350;ZNF354A;ZNF354B;ZNF362;ZNF366;ZNF383;ZNF384;ZNF394;ZNF397;ZNF398;ZNF404;ZNF41;ZNF410;ZNF419;ZNF420;ZNF431;ZNF432;ZNF436;ZNF439;ZNF44;ZNF440;ZNF442;ZNF443;ZNF446;ZNF449;ZNF45;ZNF460;ZNF468;ZNF479;ZNF484;ZNF490;ZNF500;ZNF502;ZNF524;ZNF525;ZNF528;ZNF543;ZNF544;ZNF546;ZNF547;ZNF548;ZNF549;ZNF554;ZNF555;ZNF557;ZNF558;ZNF559;ZNF561;ZNF562;ZNF563;ZNF564;ZNF566;ZNF567;ZNF568;ZNF57;ZNF570;ZNF571;ZNF572;ZNF577;ZNF581;ZNF582;ZNF583;ZNF585A;ZNF586;ZNF589;ZNF595;ZNF599;ZNF600;ZNF605;ZNF607;ZNF611;ZNF613;ZNF614;ZNF615;ZNF616;ZNF619;ZNF620;ZNF621;ZNF625;ZNF627;ZNF649;	MYNN;MZF1;OSR2;PRDM14;PRDM6;WT1;ZBTB14;ZBTB48;ZBTB49;ZFP64;ZFP28;ZIM3;ZNF121;ZNF250;ZNF257;ZNF263;ZNF274;ZNF317;ZNF320;ZNF329;ZNF331;ZNF394;ZNF449;ZNF502;ZNF528;ZNF547;ZNF549;ZNF554;ZNF586;ZNF589;ZNF667;ZNF680;ZNF708;ZNF713;ZNF768;ZNF18;ZNF85;ZSCAN16;ZSCAN22

Motif	Nº of probes	% of probes	lower OR	upper OR	OR	p.value	FDR	TF family	TF subfamily	TF.family.member	TF.subfamily.member
										ZNF652;ZNF653;ZNF665;ZNF667;ZNF669;ZNF670;ZNF672;ZNF679;ZNF680;ZNF683;ZNF689;ZNF692;ZNF701;ZNF705D;ZNF705E;ZNF705G;ZNF708;ZNF709;ZNF71;ZNF710;ZNF713;ZNF721;ZNF727;ZNF729;ZNF736;ZNF75A;ZNF75D;ZNF76;ZNF763;ZNF764;ZNF765;ZNF768;ZNF77;ZNF771;ZNF773;ZNF774;ZNF776;ZNF777;ZNF780A;ZNF780B;ZNF782;ZNF785;ZNF799;ZNF805;ZNF808;ZNF81;ZNF813;ZNF816;ZNF823;ZNF829;ZNF836;ZNF841;ZNF844;ZNF845;ZNF846;ZNF85;ZNF853;ZNF860;ZNF878;ZNF891;ZNF99;ZSCAN16;ZSCAN2;ZSCAN22;ZSCAN23;ZSCAN29;ZSCAN31;ZSCAN32;ZSCAN4;ZSCAN5A;ZSCAN5B;ZSCAN5C;ZSCAN9;ZXDA;ZXDB;ZXDC	
ARX_HUMAN.H11MO.0.D	7	0.02745098	0.25983542	13.674.401	0.6533911	3,43E+05	0.6520406288	Paired-related HD factors{3.1.3}	ARX{3.1.3.3}	ALX1;ALX3;ALX4;ARGFX;ARX;CRX;DMBX1;DPRX;DRGX;DUX4;DUXA;ESX1;GSC;GSC2;HESX1;ISX;LEUTX;MIXL1;NOBOX;OTP;OTX1;OTX2;PHOX2A;PHOX2B;PITX1;PITX2;PITX3;PROP1;PRRX1;PRRX2;RAX;RAX2;RHOXF1;RHOXF2;SEBOX;SHOX;SHOX2;TPRX1;UNCX;VSX1;VSX2	ARX
HXC12_HUMAN.H11MO.0.D	11	0.04313725	0.32168962	11.902.755	0.6532137	2,00E+05	0.4986223562	HOX-related factors{3.1.1}	HOX9-13{3.1.1.8}	CDX1;CDX2;CDX4;EVX1;EVX2;GBX1;GBX2;GSX1;GSX2;HDX;HMBOX1;HNF1A;HNF1B;HOXA1;HOXA10;HOXA11;HOXA13;HOXA2;HOXA3;HOXA4;HOXA5;HOXA6;HOXA7;HOXA9;HOXB1;HOXB13;HOXB2;HOXB3;HOXB4;HOXB5;HOXB6;HOXB7;HOXB8;HOXB9;HOXC10;HOXC11;HOXC12;HOXC13;HOXC4;HOXC5;HOXC6;HOXC8;HOXC9;HOXD1;HOXD10;HOXD11;HOXD12;HOXD13;HOXD3;HOXD4;HOXD8;HOXD9;MEOX1;MEOX2;MNX1;PDX1;POU1F1;POU2F1;POU2F2;POU2F3;POU3F1;POU3F2;POU3F3;POU3F4;POU4F1;POU4F2;POU4F3;POU5F1;POU5F2;POU6F1;POU6F2	HOXA10;HOXA11;HOXA13;HOXA9;HOXB13;HOXC10;HOXC11;HOXC12;HOXC13;HOXC9;HOXD10;HOXD11;HOXD12;HOXD13;HOXD9
PAX2_HUMAN.H11MO.0.D	10	0.03921569	0.30873036	12.206.373	0.6521629	2,29E+05	0.5380286101	Paired domain only{3.2.2}	PAX-2-like factors (partial homeobox){3.2.2.2}	PAX1;PAX2;PAX5;PAX8;PAX9	PAX2;PAX5;PAX8
HME2_HUMAN.H11MO.0.D	8	0.03137255	0.27829485	13.060.631	0.6521134	2,99E+05	0.6099942992	NK-related factors{3.1.2}	EN (Engrailed-like factors){3.1.2.7}	BARHL1;BARHL2;BARX1;BARX2;BSX;DBX1;DBX2;DLX1;DLX2;DLX3;DLX4;DLX5;DLX6;EMX1;EMX2;EN1;EN2;HHEX;HLX;HMX1;HMX2;HMX3;LBX1;LBX2;MSX1;MSX2;NANOG;NKX1-1;NKX1-2;NKX2-1;NKX2-2;NKX2-3;NKX2-4;NKX2-5;NKX2-6;NKX2-8;NKX3-1;NKX3-2;NKX6-1;NKX6-2;NKX6-3;NOTO;TLX1;TLX2;TLX3;VAX1;VAX2;VENTX	EN1;EN2
PAX3_HUMAN.H11MO.0.D	6	0.02352941	0.23685588	14.403.764	0.6519692	3,96E+05	0.6915823960	Paired plus homeo domain{3.2.1}	PAX-3/7{3.2.1.1}	PAX3;PAX4;PAX6;PAX7	PAX3;PAX7
ZN214_HUMAN.H11MO.0.C	9	0.03529412	0.29244371	12.500.386	0.6474222	2,62E+05	0.5664040702	More than 3 adjacent zinc finger factors{2.3.3}	ZNF214-like factors{2.3.3.56}	BCL6;BCL6B;CTCF;CTCFL;FEZF1;FEZF2;GFI1;GFI1B;GLI1;GLI2;GLI3;GLI4;GLIS1;GLIS2;GLIS3;HKR1;MTF1;MYNN;MZF1;OSR2;OVOL1;OVOL2;PLAG1;PLAGL1;PLAGL2;PRDM1;PRDM14;PRDM6;SCRT1;SCRT2;SNAI1;SNAI2;SNAI3;WT1;YY1;YY2;ZBTB12;ZBTB14;ZBTB18;ZBTB20;ZBTB26;ZBTB42;ZBTB45;ZBTB47;ZBTB48;ZBTB49;ZBTB6;ZBTB7A;ZBTB7B;ZBTB7C;ZFP14;ZFP2;ZFP28;ZFP30;ZFP37;ZFP42;ZFP64;ZFP69;ZFP69B;ZFP82;ZFP91;ZFX;ZIC1;ZIC2;ZIC3;ZIC4;ZIC5;ZIK1;ZIM3;ZKSCAN1;ZKSCAN2;ZKSCAN3;ZKSCAN4;ZNF121;ZNF124;ZNF133;ZNF136;ZNF138;ZNF14;ZNF140;ZNF143;ZNF146;ZNF148;ZNF155;ZNF157;ZNF160;ZNF169;ZNF175;ZNF177;ZNF18;ZNF180;ZNF181;ZNF2;ZNF20;ZNF212;ZNF213;ZNF214;ZNF221;ZNF222;ZNF223;ZNF224;ZNF225;ZNF226;ZNF227;ZNF229;ZNF230;ZNF232;ZNF233;ZNF234;ZNF235;ZNF24;ZNF25;ZNF250;ZNF257;ZNF26;ZNF260;ZNF263;ZNF264;ZNF268;ZNF274;ZNF276;ZNF28;ZNF280A;ZNF280B;ZNF280C;ZNF280D;ZNF281;ZNF282;ZNF283;ZNF284;ZNF285;ZNF286A;ZNF286B;ZNF3;ZNF30;ZNF300;ZNF302;ZNF317;ZNF32;ZNF320;ZNF322;ZNF324;ZNF324B;ZNF329;ZNF331;ZNF333;ZNF33A;ZNF33B;ZNF343;ZNF345;ZNF347;ZNF350;ZNF354A;ZNF354B;ZNF362;ZNF366;ZNF383;ZNF384;ZNF394;ZNF397;ZNF398;ZNF404;ZNF41;ZNF410;ZNF419;ZNF420;ZNF431;ZNF432;ZNF436;ZNF439;ZNF44;ZNF440;ZNF442;ZNF443;ZNF446;ZNF449;ZNF45;ZNF460;ZNF468;ZNF479;ZNF484;ZNF490;ZNF500;ZNF502;ZNF524;ZNF525;ZNF528;ZNF543;ZNF544;ZNF546;ZNF547;ZNF548;ZNF549;ZNF554;ZNF555;ZNF557;ZNF558;ZNF559;ZNF561;ZNF562;ZNF563;ZNF564;ZNF566;ZN	ZNF214

Motif	Nº of probes	% of probes	lower OR	upper OR	OR	p.value	FDR	TF family	TF subfamily	TF.family.member	TF.subfamily.member
										F567;ZNF568;ZNF57;ZNF570;ZNF571;ZNF572;ZNF577;ZNF581;ZNF582;ZNF583;ZNF585A;ZNF586;ZNF589;ZNF595;ZNF599;ZNF600;ZNF605;ZNF607;ZNF611;ZNF613;ZNF614;ZNF615;ZNF616;ZNF619;ZNF620;ZNF621;ZNF625;ZNF627;ZNF649;ZNF652;ZNF653;ZNF665;ZNF667;ZNF669;ZNF670;ZNF672;ZNF679;ZNF680;ZNF683;ZNF689;ZNF692;ZNF701;ZNF705D;ZNF705E;ZNF705G;ZNF708;ZNF709;ZNF71;ZNF710;ZNF713;ZNF721;ZNF727;ZNF729;ZNF736;ZNF75A;ZNF75D;ZNF76;ZNF763;ZNF764;ZNF765;ZNF768;ZNF77;ZNF771;ZNF773;ZNF774;ZNF776;ZNF777;ZNF780A;ZNF780B;ZNF782;ZNF785;ZNF799;ZNF805;ZNF808;ZNF81;ZNF813;ZNF816;ZNF823;ZNF829;ZNF836;ZNF841;ZNF844;ZNF845;ZNF846;ZNF85;ZNF853;ZNF860;ZNF878;ZNF891;ZNF99;ZSCAN16;ZSCAN2;ZSCAN22;ZSCAN23;ZSCAN29;ZSCAN31;ZSCAN32;ZSCAN4;ZSCAN5A;ZSCAN5B;ZSCAN5C;ZSCAN9;ZXDA;ZXDB;ZXDC	
RXRB_HUMAN.H11MO.0.C	25	0.09803922	0.40729214	0.9734824	0.6427311	3,24E+04	0.1664173379	RXR-related receptors (NR2){2.1.3}	Retinoid X receptors (NR2B){2.1.3.1}	HNF4A;HNF4G;NR2C1;NR2C2;NR2E1;NR2E3;NR2F1;NR2F2;NR2F6;RXRA;RXRB;RXRG	RXRA;RXRB;RXRG
CRX_HUMAN.H11MO.0.B	8	0.03137255	0.27360787	12.839.934	0.6411222	2,43E+05	0.5585674387	Paired-related HD factors{3.1.3}	OTX{3.1.3.17}	ALX1;ALX3;ALX4;ARGFX;ARX;CRX;DMBX1;DPRX;DRGX;DUX4;DUXA;ESX1;GSC;GSC2;HESX1;ISX;LEUTX;MIXL1;NOBOX;OTP;OTX1;OTX2;PHOX2A;PHOX2B;PITX1;PITX2;PITX3;PROP1;PRRX1;PRRX2;RAX;RAX2;RHOXF1;RHOXF2;SEBOX;SHOX;SHOX2;TPRX1;UNCX;VSX1;VSX2	CRX;OTX1;OTX2
DBP_HUMAN.H11MO.0.B	8	0.03137255	0.27273457	12.798.933	0.6390736	2,43E+05	0.5585674387	C/EBP-related{1.1.8}	PAR factors{1.1.8.2}	CEBPA;CEBPB;CEBPD;CEBPE;CEBPG;DBP;DDIT3;HLF;NFIL3;TEF	DBP;HLF;NFIL3;TEF
VDR_HUMAN.H11MO.1.A	21	0.08235294	0.38809099	10.002.840	0.6390456	4,52E+04	0.1948391141	Thyroid hormone receptor-related factors (NR1){2.1.2}	Vitamin D receptor (NR1I){2.1.2.4}	NR1D1;NR1D2;NR1H2;NR1H3;NR1H4;NR1I2;NR1I3;PPARA;PPARD;PPARG;RARA;RARB;RARG;RORA;RORB;RORC;THRA;THRB;VDR	NR1I2;NR1I3;VDR
THB_HUMAN.H11MO.1.D	26	0.10196078	0.40808783	0.9601428	0.6383295	2,83E+04	0.1501614358	Thyroid hormone receptor-related factors (NR1){2.1.2}	Thyroid hormone receptors (NR1A){2.1.2.2}	NR1D1;NR1D2;NR1H2;NR1H3;NR1H4;NR1I2;NR1I3;PPARA;PPARD;PPARG;RARA;RARB;RARG;RORA;RORB;RORC;THRA;THRB;VDR	THRA;THRB
MAFG_HUMAN.H11MO.0.A	13	0.05098039	0.33449543	11.115.986	0.6375448	1,27E+05	0.3867481384	Maf-related factors{1.1.3}	Small Maf factors{1.1.3.2}	MAF;MAFA;MAFB;MAFF;MAFG;MAFK;NRL	MAFF;MAFG;MAFK
ZN264_HUMAN.H11MO.0.C	9	0.03529412	0.28721626	12.277.099	0.6358691	2,13E+05	0.5124758438	More than 3 adjacent zinc finger factors{2.3.3}	ZNF460-like factors{2.3.3.54}	BCL6;BCL6B;CTCF;CTCFL;FEZF1;FEZF2;GFI1;GFI1B;GLI1;GLI2;GLI3;GLI4;GLIS1;GLIS2;GLIS3;HKR1;MTF1;MYNN;MZF1;OSR2;OVOL1;OVOL2;PLAG1;PLAGL1;PLAGL2;PRDM1;PRDM14;PRDM6;SCRT1;SCRT2;SNAI1;SNAI2;SNAI3;WT1;YY1;YY2;ZBTB12;ZBTB14;ZBTB18;ZBTB20;ZBTB26;ZBTB42;ZBTB45;ZBTB47;ZBTB48;ZBTB49;ZBTB6;ZBTB7A;ZBTB7B;ZBTB7C;ZFP14;ZFP2;ZFP28;ZFP30;ZFP37;ZFP42;ZFP64;ZFP69;ZFP69B;ZFP82;ZFP91;ZFX;ZIC1;ZIC2;ZIC3;ZIC4;ZIC5;ZIK1;ZIM3;ZKSCAN1;ZKSCAN2;ZKSCAN3;ZKSCAN4;ZNF121;ZNF124;ZNF133;ZNF136;ZNF138;ZNF14;ZNF140;ZNF143;ZNF146;ZNF148;ZNF155;ZNF157;ZNF160;ZNF169;ZNF175;ZNF177;ZNF18;ZNF180;ZNF181;ZNF2;ZNF20;ZNF212;ZNF213;ZNF214;ZNF221;ZNF222;ZNF223;ZNF224;ZNF225;ZNF226;ZNF227;ZNF229;ZNF230;ZNF232;ZNF233;ZNF234;ZNF235;ZNF24;ZNF25;ZNF250;ZNF257;ZNF26;ZNF260;ZNF263;ZNF264;ZNF268;ZNF274;ZNF276;ZNF28;ZNF280A;ZNF280B;ZNF280C;ZNF280D;ZNF281;ZNF282;ZNF283;ZNF284;ZNF285;ZNF286A;ZNF286B;ZNF3;ZNF30;ZNF300;ZNF302;ZNF317;ZNF32;ZNF320;ZNF322;ZNF324;ZNF324B;ZNF329;ZNF331;ZNF333;ZNF33A;ZNF33B;ZNF343;ZNF345;ZNF347;ZNF350;ZNF354A;ZNF354B;ZNF362;ZNF366;ZNF383;ZNF384;ZNF394;ZNF397;ZNF398;ZNF404;ZNF41;ZNF410;ZNF419;ZNF420;ZNF431;ZNF432;ZNF436;ZNF439;ZNF44;ZNF440;ZNF442;ZNF443;ZNF446;ZNF449;ZNF45;ZNF460;ZNF468;ZNF479;ZNF484;ZNF490;ZNF500;ZNF502;ZNF524;ZNF525;ZNF528;ZNF543;ZNF544;ZNF546;ZNF547;ZNF548;ZNF549;ZNF554;ZNF555;ZNF557;ZNF558;ZNF559;ZNF561;ZNF562;ZNF563;ZNF564;ZNF566;ZNF567;ZNF568;ZNF57;ZNF570;ZNF571;ZNF572;ZNF577;ZNF5	ZNF264

Motif	Nº of probes	% of probes	lower OR	upper OR	OR	p.value	FDR	TF family	TF subfamily	TF.family.member	TF.subfamily.member
										81;ZNF582;ZNF583;ZNF585A;ZNF586;ZNF589;ZNF595;ZNF599;ZNF600;ZNF605;ZNF607;ZNF611;ZNF613;ZNF614;ZNF615;ZNF616;ZNF619;ZNF620;ZNF621;ZNF625;ZNF627;ZNF649;ZNF652;ZNF653;ZNF665;ZNF667;ZNF669;ZNF670;ZNF672;ZNF679;ZNF680;ZNF683;ZNF689;ZNF692;ZNF701;ZNF705D;ZNF705E;ZNF705G;ZNF708;ZNF709;ZNF71;ZNF710;ZNF713;ZNF721;ZNF727;ZNF729;ZNF736;ZNF75A;ZNF75D;ZNF76;ZNF763;ZNF764;ZNF765;ZNF768;ZNF77;ZNF771;ZNF773;ZNF774;ZNF776;ZNF777;ZNF780A;ZNF780B;ZNF782;ZNF785;ZNF799;ZNF805;ZNF808;ZNF81;ZNF813;ZNF816;ZNF823;ZNF829;ZNF836;ZNF841;ZNF844;ZNF845;ZNF846;ZNF85;ZNF853;ZNF860;ZNF878;ZNF891;ZNF99;ZSCAN16;ZSCAN2;ZSCAN22;ZSCAN23;ZSCAN29;ZSCAN31;ZSCAN32;ZSCAN4;ZSCAN5A;ZSCAN5B;ZSCAN5C;ZSCAN9;ZXDA;ZXDB;ZXDC	
NFE2_HUMAN.H11MO.0.A	22	0.08627451	0.39042849	0.9854651	0.6353314	3,96E+04	0.1828900448	Jun-related factors{1.1.1}	NF-E2-like factors{1.1.1.2}	ATF2;ATF7;BACH1;BACH2;CREB5;JUN;JUNB;JUND;NFE2;NFE2L1;NFE2L2;NFE2L3	BACH1;BACH2;NFE2L1;NFE2L2;NFE2
MEIS1_HUMAN.H11MO.0.A	8	0.03137255	0.27073293	12.704.893	0.6343771	2,43E+05	0.5585674387	TALE-type homeo domain factors{3.1.4}	MEIS{3.1.4.2}	IRX1;IRX2;IRX3;IRX4;IRX5;IRX6;MEIS1;MEIS2;MEIS3;MKX;PBX1;PBX2;PBX3;PKNOX1;PKNOX2;TGIF1;TGIF2;TGIF2LX;TGIF2LY	MEIS1;MEIS2;MEIS3
HNF4A_HUMAN.H11MO.0.A	14	0.05490196	0.34083148	10.836.053	0.6331064	1,12E+05	0.3603587159	RXR-related receptors (NR2){2.1.3}	HNF-4 (NR2A){2.1.3.2}	HNF4A;HNF4G;NR2C1;NR2C2;NR2E1;NR2E3;NR2F1;NR2F2;NR2F6;RXRA;RXRB;RXRG	HNF4A;HNF4G
MYB_HUMAN.H11MO.0.A	14	0.05490196	0.33940138	10.790.565	0.6304491	1,13E+05	0.3603587159	Myb/SANT domain factors{3.5.1}	Myb-like factors{3.5.1.1}	CDC5L;DMTF1;MYB;MYBL1;MYBL2;SMARCA1;SMARCA5;SNAPC4;TRERF1;ZNF541	CDC5L;MYBL1;MYBL2;MYB
THA_HUMAN.H11MO.0.C	26	0.10196078	0.40287992	0.9478844	0.6301915	2,32E+04	0.1304521137	Thyroid hormone receptor-related factors (NR1){2.1.2}	Thyroid hormone receptors (NR1A){2.1.2.2}	NR1D1;NR1D2;NR1H2;NR1H3;NR1H4;NR1I2;NR1I3;PPARA;PPARD;PPARG;RARA;RARB;RARG;RORA;RORB;RORC;THRA;THRB;VDR	THRA;THRB
ZN708_HUMAN.H11MO.0.C	25	0.09803922	0.39931262	0.9544604	0.6301504	2,66E+04	0.1440260117	More than 3 adjacent zinc finger factors{2.3.3}	unclassified{2.3.3.0}	BCL6B;BCL6;CTCFL;CTCF;FEZF1;GFI1B;GFI1;GLI1;GLI2;GLI3;GLIS1;GLIS2;GLIS3;MTF1;MYNN;MZF1;OSR2;OVOL1;OVOL2;ZNF146;PLAG1;PLAGL1;PRDM14;PRDM1;PRDM6;SCRT1;SCRT2;SNAI1;SNAI2;YY1;YY2;WT1;ZNF324;ZNF354A;ZBTB14;ZBTB18;ZBTB48;ZBTB49;ZBTB7A;ZBTB7B;ZBTB6;ZFP64;ZFP28;ZFP42;ZFP82;ZFX;ZIC1;ZIC2;ZIC3;ZIC4;ZIM3;ZKSCAN1;ZKSCAN3;ZNF121;ZNF136;ZNF140;ZNF143;ZNF148;ZNF214;ZNF232;ZNF250;ZNF257;ZNF260;ZNF263;ZNF264;ZNF274;ZNF281;ZNF282;ZNF317;ZNF320;ZNF322;ZNF329;ZNF331;ZNF333;ZNF350;ZNF384;ZNF394;ZNF410;ZNF436;ZNF449;ZNF490;ZNF502;ZNF524;ZNF528;ZNF547;ZNF549;ZNF554;ZNF563;ZNF582;ZNF586;ZNF589;ZNF652;ZNF667;ZNF680;ZNF708;ZNF713;ZNF768;ZNF816;ZNF18;ZNF41;ZNF76;ZNF85;ZSCAN16;ZSCAN22;ZSCAN31;ZSCAN4	MYNN;MZF1;OSR2;PRDM14;PRDM6;WT1;ZBTB14;ZBTB48;ZBTB49;ZFP64;ZFP28;ZIM3;ZNF121;ZNF250;ZNF257;ZNF263;ZNF274;ZNF317;ZNF320;ZNF329;ZNF331;ZNF394;ZNF449;ZNF502;ZNF528;ZNF547;ZNF549;ZNF554;ZNF586;ZNF589;ZNF667;ZNF680;ZNF708;ZNF713;ZNF768;ZNF18;ZNF85;ZSCAN16;ZSCAN22
PAX6_HUMAN.H11MO.0.C	11	0.04313725	0.31008154	11.473.180	0.6296461	1,65E+05	0.4482388799	Paired plus homeo domain{3.2.1}	PAX-4/6{3.2.1.2}	PAX3;PAX4;PAX6;PAX7	PAX4;PAX6
ESR2_HUMAN.H11MO.1.A	13	0.05098039	0.32680750	10.860.362	0.6229460	1,04E+05	0.3444492807	Steroid hormone receptors (NR3){2.1.1}	ER-like receptors (NR3A&B){2.1.1.2}	AR;ESR1;ESR2;ESRRA;ESRRB;ESRRG;NR3C1;NR3C2;PGR	ESRRA;ESRRB;ESRRG;ESR1;ESR2
HBP1_HUMAN.H11MO.0.D	5	0.01960784	0.19997851	14.713.061	0.6216149	3,67E+05	0.6704894827	SOX-related factors{4.1.1}	Further Sox-related factors{4.1.1.9}	BBX;CIC;HBP1;SOX1;SOX10;SOX11;SOX12;SOX13;SOX14;SOX15;SOX17;SOX18;SOX2;SOX21;SOX3;SOX30;SOX4;SOX5;SOX6;SOX7;SOX8;SOX9;SRY	HBP1
PO4F1_HUMAN.H11MO.0.D	11	0.04313725	0.30582931	11.314.631	0.6209470	1,34E+05	0.4038412812	POU domain factors{3.1.10}	POU4 (Brn-3-like factors){3.1.10.4}	CDX1;CDX2;CDX4;EVX1;EVX2;GBX1;GBX2;GSX1;GSX2;HDX;HMBOX1;HNF1A;HNF1B;HOXA1;HOXA10;HOXA11;HOXA13;HOXA2;HOXA3;HOXA4;HOXA5;HOXA6;HOXA7;HOXA9;HOXB1;HOXB13;HOXB2;HOXB3;HOXB4;HOXB5;HOXB6;HOXB7;HOXB8;HOXB9;HOXC10;HOXC11;HOXC12;HOXC13;HOXC4;HOXC5;HOXC6;HOXC8;HOXC9;HOXD1;HOXD10;HOXD11;HOXD12;HOXD13;HOXD3;HOXD4;HOXD8;HOXD9;MEOX1;MEOX2;MNX1;PDX1;POU1F1;POU2F1;POU2F2;POU2F3;POU3F1;POU3F2;POU3F3;POU3F4;POU4F1;POU4F2;POU4F3;POU5F1;POU5F1B;POU5F2;POU6F1;POU6F2	POU4F1;POU4F2;POU4F3

Motif	Nº of probes	% of probes	lower OR	upper OR	OR	p.value	FDR	TF family	TF subfamily	TF.family.member	TF.subfamily.member
BATF_HUMAN.H11MO.0.A	9	0.03529412	0.27810590	11.887.201	0.6156864	1,72E+05	0.4632076119	B-ATF-related factors{1.1.4}	B-ATF{1.1.4.0.1}	BATF;BATF2;BATF3	BATF
BRCA1_HUMAN.H11MO.0.D	11	0.04313725	0.30296314	11.208.829	0.6151467	1,34E+05	0.4038863746	NA	NA	BRCA1	BRCA1
DLX5_HUMAN.H11MO.0.D	8	0.03137255	0.25885135	12.147.368	0.6065470	1,97E+05	0.4920727943	NK-related factors{3.1.2}	DLX{3.1.2.5}	BARHL1;BARHL2;BARX1;BARX2;BSX;DBX1;DBX2;DLX1;DLX2;DLX3;DLX4;DLX5;DLX6;EMX1;EMX2;EN1;EN2;HHEX;HLX;HMX1;HMX2;HMX3;LBX1;LBX2;MSX1;MSX2;NANOG;NKX1-1;NKX1-2;NKX2-1;NKX2-2;NKX2-3;NKX2-4;NKX2-5;NKX2-6;NKX2-8;NKX3-1;NKX3-2;NKX6-1;NKX6-2;NKX6-3;NOTO;TLX1;TLX2;TLX3;VAX1;VAX2;VENTX	DLX1;DLX2;DLX3;DLX4;DLX5;DLX6
MCR_HUMAN.H11MO.0.D	12	0.04705882	0.30880751	10.789.158	0.6061422	9,55E+04	0.3243872980	Steroid hormone receptors (NR3){2.1.1}	GR-like receptors (NR3C){2.1.1.1}	AR;ESR1;ESR2;ESRRA;ESRRB;ESRRG;NR3C1;NR3C2;PGR	AR;NR3C1;NR3C2;PGR
TFE3_HUMAN.H11MO.0.B	22	0.08627451	0.36783980	0.9285034	0.5986065	1,74E+04	0.1031636406	bHLH-ZIP factors{1.2.6}	TFE3-like factors{1.2.6.1}	MAX;MITF;MLX;MLXIP;MLXIPL;MNT;MXD1;MXD3;MXD4;MXI1;MYC;MYCL;MYCN;REPIN1;SREBF1;SREBF2;TFAP4;TFE3;TFEB;TFEC;USF1;USF2	MITF;TFE3;TFEB
CDC5L_HUMAN.H11MO.0.D	9	0.03529412	0.27033288	11.554.517	0.5984623	1,39E+05	0.4135427206	Myb/SANT domain factors{3.5.1}	Myb-like factors{3.5.1.1}	CDC5L;DMTF1;MYB;MYBL1;MYBL2;SMARCA1;SMARCA5;SNAPC4;TRERF1;ZNF541	CDC5L;MYBL1;MYBL2;MYB
RFX5_HUMAN.H11MO.1.A	17	0.06666667	0.34241524	0.9781750	0.5978967	4,14E+04	0.1867821692	RFX-related factors{3.3.3}	RFX5{3.3.3.0.5}	RFX1;RFX2;RFX3;RFX4;RFX5;RFX6;RFX7;RFX8	RFX5
MEIS3_HUMAN.H11MO.0.D	13	0.05098039	0.31361434	10.421.529	0.5977859	6,81E+04	0.2598919152	TALE-type homeo domain factors{3.1.4}	MEIS{3.1.4.2}	IRX1;IRX2;IRX3;IRX4;IRX5;IRX6;MEIS1;MEIS2;MEIS3;MKX;PBX1;PBX2;PBX3;PKNOX1;PKNOX2;TGIF1;TGIF2;TGIF2LX;TGIF2LY	MEIS1;MEIS2;MEIS3
BCL6B_HUMAN.H11MO.0.D	13	0.05098039	0.31313510	10.405.621	0.5968719	6,81E+04	0.2598919152	More than 3 adjacent zinc finger factors{2.3.3}	BCL6 factors{2.3.3.22}	BCL6;BCL6B;CTCF;CTCFL;FEZF1;FEZF2;GFI1;GFI1B;GLI1;GLI2;GLI3;GLI4;GLIS1;GLIS2;GLIS3;HKR1;MTF1;MYNN;MZF1;OSR2;OVOL1;OVOL2;PLAG1;PLAGL1;PLAGL2;PRDM1;PRDM14;PRDM6;SCRT1;SCRT2;SNAI1;SNAI2;SNAI3;WT1;YY1;YY2;ZBTB12;ZBTB14;ZBTB18;ZBTB20;ZBTB26;ZBTB42;ZBTB45;ZBTB47;ZBTB48;ZBTB49;ZBTB6;ZBTB7A;ZBTB7B;ZBTB7C;ZFP14;ZFP2;ZFP28;ZFP30;ZFP37;ZFP42;ZFP64;ZFP69;ZFP69B;ZFP82;ZFP91;ZFX;ZIC1;ZIC2;ZIC3;ZIC4;ZIC5;ZIK1;ZIM3;ZKSCAN1;ZKSCAN2;ZKSCAN3;ZKSCAN4;ZNF121;ZNF124;ZNF133;ZNF136;ZNF138;ZNF14;ZNF140;ZNF143;ZNF146;ZNF148;ZNF155;ZNF157;ZNF160;ZNF169;ZNF175;ZNF177;ZNF18;ZNF180;ZNF181;ZNF2;ZNF20;ZNF212;ZNF213;ZNF214;ZNF221;ZNF222;ZNF223;ZNF224;ZNF225;ZNF226;ZNF227;ZNF229;ZNF230;ZNF232;ZNF233;ZNF234;ZNF235;ZNF24;ZNF25;ZNF250;ZNF257;ZNF26;ZNF260;ZNF263;ZNF264;ZNF268;ZNF274;ZNF276;ZNF28;ZNF280A;ZNF280B;ZNF280C;ZNF280D;ZNF281;ZNF282;ZNF283;ZNF284;ZNF285;ZNF286A;ZNF286B;ZNF3;ZNF30;ZNF300;ZNF302;ZNF317;ZNF32;ZNF320;ZNF322;ZNF324;ZNF324B;ZNF329;ZNF331;ZNF333;ZNF33A;ZNF33B;ZNF343;ZNF345;ZNF347;ZNF350;ZNF354A;ZNF354B;ZNF362;ZNF366;ZNF383;ZNF384;ZNF394;ZNF397;ZNF398;ZNF404;ZNF41;ZNF410;ZNF419;ZNF420;ZNF431;ZNF432;ZNF436;ZNF439;ZNF44;ZNF440;ZNF442;ZNF443;ZNF446;ZNF449;ZNF45;ZNF460;ZNF468;ZNF479;ZNF484;ZNF490;ZNF500;ZNF502;ZNF524;ZNF525;ZNF528;ZNF543;ZNF544;ZNF546;ZNF547;ZNF548;ZNF549;ZNF554;ZNF555;ZNF557;ZNF558;ZNF559;ZNF561;ZNF562;ZNF563;ZNF564;ZNF566;ZNF567;ZNF568;ZNF57;ZNF570;ZNF571;ZNF572;ZNF577;ZNF581;ZNF582;ZNF583;ZNF585A;ZNF586;ZNF589;ZNF595;ZNF599;ZNF600;ZNF605;ZNF607;ZNF611;ZNF613;ZNF614;ZNF615;ZNF616;ZNF619;ZNF620;ZNF621;ZNF625;ZNF627;ZNF649;ZNF652;ZNF653;ZNF665;ZNF667;ZNF669;ZNF670;ZNF672;ZNF679;ZNF680;ZNF683;ZNF689;ZNF692;ZNF701;ZNF705D;ZNF705E;ZNF705G;ZNF708;ZNF709;ZNF71;ZNF710;ZNF713;ZNF721;ZNF727;ZNF729;ZNF736;ZNF75A;ZNF75D;ZNF76;ZNF763;ZNF764;ZNF765;ZNF768;ZNF77;ZNF771;ZNF773;ZNF7	BCL6B;BCL6

Motif	Nº of probes	% of probes	lower OR	upper OR	OR	p.value	FDR	TF family	TF subfamily	TF.family.member	TF.subfamily.member
										74;ZNF776;ZNF777;ZNF780A;ZNF780B;ZNF782;ZNF785;ZNF799;ZNF805;ZNF808;ZNF81;ZNF813;ZNF816;ZNF823;ZNF829;ZNF836;ZNF841;ZNF844;ZNF845;ZNF846;ZNF85;ZNF853;ZNF860;ZNF878;ZNF891;ZNF99;ZSCAN16;ZSCAN2;ZSCAN22;ZSCAN23;ZSCAN29;ZSCAN31;ZSCAN32;ZSCAN4;ZSCAN5A;ZSCAN5B;ZSCAN5C;ZSCAN9;ZXDA;ZXDB;ZXDC	
MAFK_HUMAN.H11MO.0.A	11	0.04313725	0.29390339	10.872.808	0.5967091	1,09E+05	0.3543937687	Maf-related factors{1.1.3}	Small Maf factors{1.1.3.2}	MAF;MAFA;MAFB;MAFF;MAFG;MAFK;NRL	MAFF;MAFG;MAFK
MAFF_HUMAN.H11MO.0.B	14	0.05490196	0.32078154	10.198.224	0.5958490	6,02E+04	0.2404985012	Maf-related factors{1.1.3}	Small Maf factors{1.1.3.2}	MAF;MAFA;MAFB;MAFF;MAFG;MAFK;NRL	MAFF;MAFG;MAFK
P73_HUMAN.H11MO.0.A	17	0.06666667	0.34095052	0.9739882	0.5953398	4,17E+04	0.1867821692	p53-related factors{6.3.1}	p73{6.3.1.0.3}	TP53;TP63;TP73	TP73
PO5F1_HUMAN.H11MO.0.A	10	0.03921569	0.27487214	10.867.006	0.5806244	9,87E+04	0.3323756794	POU domain factors{3.1.10}	POU5 (Oct-3/4-like factors){3.1.10.5}	CDX1;CDX2;CDX4;EVX1;EVX2;GBX1;GBX2;GSX1;GSX2;HDX;HMBOX1;HNF1A;HNF1B;HOXA1;HOXA10;HOXA11;HOXA13;HOXA2;HOXA3;HOXA4;HOXA5;HOXA6;HOXA7;HOXA9;HOXB1;HOXB13;HOXB2;HOXB3;HOXB4;HOXB5;HOXB6;HOXB8;HOXB9;HOXC10;HOXC11;HOXC12;HOXC13;HOXC4;HOXC5;HOXC6;HOXC8;HOXC9;HOXD1;HOXD10;HOXD11;HOXD12;HOXD13;HOXD3;HOXD4;HOXD8;HOXD9;MEOX1;MEOX2;MNX1;PDX1;POU1F1;POU2F1;POU2F2;POU2F3;POU3F1;POU3F2;POU3F3;POU3F4;POU4F1;POU4F2;POU4F3;POU5F1;POU5F1B;POU5F2;POU6F1;POU6F2	POU5F1B;POU5F1
GBX2_HUMAN.H11MO.0.D	7	0.02745098	0.22717014	11.951.930	0.5711334	1,80E+05	0.4667040255	HOX-related factors{3.1.1}	GBX (Gastrulation brain homeobox){3.1.1.11}	CDX1;CDX2;CDX4;EVX1;EVX2;GBX1;GBX2;GSX1;GSX2;HDX;HMBOX1;HNF1A;HNF1B;HOXA1;HOXA10;HOXA11;HOXA13;HOXA2;HOXA3;HOXA4;HOXA5;HOXA6;HOXA7;HOXA9;HOXB1;HOXB13;HOXB2;HOXB3;HOXB4;HOXB5;HOXB6;HOXB7;HOXB8;HOXB9;HOXC10;HOXC11;HOXC12;HOXC13;HOXC4;HOXC5;HOXC6;HOXC8;HOXC9;HOXD1;HOXD10;HOXD11;HOXD12;HOXD13;HOXD3;HOXD4;HOXD8;HOXD9;MEOX1;MEOX2;MNX1;PDX1;POU1F1;POU2F1;POU2F2;POU2F3;POU3F1;POU3F2;POU3F3;POU3F4;POU4F1;POU4F2;POU4F3;POU5F1;POU5F2;POU6F1;POU6F2	GBX1;GBX2
CEBPA_HUMAN.H11MO.0.A	7	0.02745098	0.22596250	11.888.517	0.5681022	1,81E+05	0.4667040255	C/EBP-related{1.1.8}	C/EBP{1.1.8.1}	CEBPA;CEBPB;CEBPD;CEBPE;CEBPG;DBP;DDIT3;HLF;NFIL3;TEF	CEBPA;CEBPB;CEBPD;CEBPE;CEBPG;DDIT3
CUX1_HUMAN.H11MO.0.C	7	0.02745098	0.22565286	11.872.258	0.5673250	1,81E+05	0.4667040255	HD-CUT factors{3.1.9}	CUX{3.1.9.2}	CUX1;CUX2;ONECUT1;ONECUT2;ONECUT3;SATB1;SATB2	CUX1;CUX2
SOX5_HUMAN.H11MO.0.C	7	0.02745098	0.22561421	11.870.229	0.5672280	1,81E+05	0.4667040255	SOX-related factors{4.1.1}	Group D{4.1.1.4}	BBX;CIC;HBP1;SOX1;SOX10;SOX11;SOX12;SOX13;SOX14;SOX15;SOX17;SOX18;SOX2;SOX21;SOX3;SOX30;SOX4;SOX5;SOX6;SOX7;SOX8;SOX9;SRY	SOX13;SOX5
ALX3_HUMAN.H11MO.0.D	7	0.02745098	0.22335632	11.751.667	0.5615594	1,42E+05	0.4159667122	Paired-related HD factors{3.1.3}	ALX{3.1.3.1}	ALX1;ALX3;ALX4;ARGFX;ARX;CRX;DMBX1;DPRX;DRGX;DUX4;DUXA;ESX1;GSC;GSC2;HESX1;ISX;LEUTX;MIXL1;NOBOX;OTP;OTX1;OTX2;PHOX2A;PHOX2B;PITX1;PITX2;PITX3;PROP1;PRRX1;PRRX2;RAX;RAX2;RHOXF1;RHOXF2;SEBOX;SHOX;SHOX2;TPRX1;UNCX;VSX1;VSX2	ALX1;ALX3;ALX4
HXD13_HUMAN.H11MO.0.D	10	0.03921569	0.26506403	10.478.460	0.5598593	7,94E+04	0.2854511109	HOX-related factors{3.1.1}	HOX9-13{3.1.1.8}	CDX1;CDX2;CDX4;EVX1;EVX2;GBX1;GBX2;GSX1;GSX2;HDX;HMBOX1;HNF1A;HNF1B;HOXA1;HOXA10;HOXA11;HOXA13;HOXA2;HOXA3;HOXA4;HOXA5;HOXA6;HOXA7;HOXA9;HOXB1;HOXB13;HOXB2;HOXB3;HOXB4;HOXB5;HOXB6;HOXB7;HOXB8;HOXB9;HOXC10;HOXC11;HOXC12;HOXC13;HOXC4;HOXC5;HOXC6;HOXC8;HOXC9;HOXD1;HOXD10;HOXD11;HOXD12;HOXD13;HOXD3;HOXD4;HOXD8;HOXD9;MEOX1;MEOX2;MNX1;PDX1;POU1F1;POU2F1;POU2F2;POU2F3;POU3F1;POU3F2;POU3F3;POU3F4;POU4F1;POU4F2;POU4F3;POU5F1;POU5F2;POU6F1;POU6F2	HOXA10;HOXA11;HOXA13;HOXA9;HOXB13;HOXC10;HOXC11;HOXC12;HOXC13;HOXC9;HOXD10;HOXD11;HOXD12;HOXD13;HOXD9
ALX1_HUMAN.H11MO.0.B	7	0.02745098	0.22226244	11.694.218	0.5588125	1,42E+05	0.4159667122	Paired-related HD factors{3.1.3}	ALX{3.1.3.1}	ALX1;ALX3;ALX4;ARGFX;ARX;CRX;DMBX1;DPRX;DRGX;DUX4;DUXA;ESX1;GSC;GSC2;HESX1;ISX;LEUTX;MIXL1;NOBOX;OTP;OTX1;OTX2;PHOX2A;PHOX2B;PITX1;PITX2;	ALX1;ALX3;ALX4

Motif	Nº of probes	% of probes	lower OR	upper OR	OR	p.value	FDR	TF family	TF subfamily	TF.family.member	TF.subfamily.member
										PITX3;PROP1;PRRX1;PRRX2;RAX;RAX2;RHOXF1;RHOXF2;SEBOX;SHOX;SHOX2;TPRX1;UNCX;VSX1;VSX2	
NR1I3_HUMAN.H11MO.0.C	11	0.04313725	0.27469356	10.162.537	0.5577367	5,58E+04	0.2252006824	Thyroid hormone receptor-related factors (NR1){2.1.2}	Vitamin D receptor (NR1I){2.1.2.4}	NR1D1;NR1D2;NR1H2;NR1H3;NR1H4;NR1I2;NR1I3;PPARA;PPARD;PPARG;RARA;RARB;RARG;RORA;RORB;RORC;THRA;THRB;VDR	NR1I2;NR1I3;VDR
EOMES_HUMAN.H11MO.0.D	13	0.05098039	0.29200453	0.9702960	0.5565714	3,50E+04	0.1724127549	TBrain-related factors{6.5.2}	TBR-2 (EOMES){6.5.2.0.2}	EOMES;TBR1;TBX21	EOMES
MITF_HUMAN.H11MO.0.A	25	0.09803922	0.35213494	0.8416333	0.5557028	3,80E+03	0.0374406390	bHLH-ZIP factors{1.2.6}	TFE3-like factors{1.2.6.1}	MAX;MITF;MLX;MLXIP;MLXIPL;MNT;MXD1;MXD3;MXD4;MXI1;MYC;MYCL;MYCN;REPIN1;SREBF1;SREBF2;TFAP4;TFE3;TFEB;TFEC;USF1;USF2	MITF;TFE3;TFEB
JUND_HUMAN.H11MO.0.A	15	0.05882353	0.30150712	0.9213911	0.5473399	2,22E+04	0.1267136742	Jun-related factors{1.1.1}	Jun factors{1.1.1.1}	ATF2;ATF7;BACH1;BACH2;CREB5;JUN;JUNB;JUND;NFE2;NFE2L1;NFE2L2;NFE2L3	JUNB;JUND;JUN
P63_HUMAN.H11MO.0.A	19	0.07450980	0.32039999	0.8656203	0.5419068	8,53E+03	0.0625511107	p53-related factors{6.3.1}	p63{6.3.1.0.2}	TP53;TP63;TP73	TP63
TBR1_HUMAN.H11MO.0.D	11	0.04313725	0.26668349	0.9865586	0.5415031	4,44E+04	0.1938611433	TBrain-related factors{6.5.2}	TBR-1{6.5.2.0.1}	EOMES;TBR1;TBX21	TBR1
OLIG1_HUMAN.H11MO.0.D	7	0.02745098	0.21373861	11.244.855	0.5373752	1,12E+05	0.3603587159	Tal-related factors{1.2.3}	Neurogenin / Atonal-like factors{1.2.3.4}	ATOH1;ATOH7;ATOH8;BHLHA15;BHLHA9;BHLHE22;BHLHE23;FERD3L;FIGLA;HAND1;HAND2;LYL1;MESP1;MESP2;MSC;MSGN1;NEUROD1;NEUROD2;NEUROD4;NEUROD6;NEUROG1;NEUROG2;NEUROG3;NHLH1;NHLH2;OLIG1;OLIG2;OLIG3;PTF1A;SCX;TAL1;TAL2;TCF15;TCF21;TCF23;TWIST1;TWIST2	ATOH1;BHLHA15;BHLHE22;BHLHE23;NEUROD1;NEUROD2;NEUROG2;OLIG1;OLIG2;OLIG3
SCRT1_HUMAN.H11MO.0.D	10	0.03921569	0.25428387	10.052.909	0.5371313	4,96E+04	0.2101699987	More than 3 adjacent zinc finger factors{2.3.3}	Snail-like factors{2.3.3.2}	BCL6;BCL6B;CTCF;CTCFL;FEZF1;FEZF2;GFI1;GFI1B;GLI1;GLI2;GLI3;GLI4;GLIS1;GLIS2;GLIS3;HKR1;MTF1;MYNN;MZF1;OSR2;OVOL1;OVOL2;PLAG1;PLAGL1;PLAGL2;PRDM1;PRDM14;PRDM6;SCRT1;SCRT2;SNAI1;SNAI2;SNAI3;WT1;YY1;YY2;ZBTB12;ZBTB14;ZBTB18;ZBTB20;ZBTB26;ZBTB42;ZBTB45;ZBTB47;ZBTB48;ZBTB49;ZBTB6;ZBTB7A;ZBTB7B;ZBTB7C;ZFP14;ZFP2;ZFP28;ZFP30;ZFP37;ZFP42;ZFP64;ZFP69;ZFP69B;ZFP82;ZFP91;ZFX;ZIC1;ZIC2;ZIC3;ZIC4;ZIC5;ZIK1;ZIM3;ZKSCAN1;ZKSCAN2;ZKSCAN3;ZKSCAN4;ZNF121;ZNF124;ZNF133;ZNF136;ZNF138;ZNF14;ZNF140;ZNF143;ZNF146;ZNF148;ZNF155;ZNF157;ZNF160;ZNF169;ZNF175;ZNF177;ZNF18;ZNF180;ZNF181;ZNF2;ZNF20;ZNF212;ZNF213;ZNF214;ZNF221;ZNF222;ZNF223;ZNF224;ZNF225;ZNF226;ZNF227;ZNF229;ZNF230;ZNF232;ZNF233;ZNF234;ZNF235;ZNF24;ZNF25;ZNF250;ZNF257;ZNF26;ZNF260;ZNF263;ZNF264;ZNF268;ZNF274;ZNF276;ZNF28;ZNF280A;ZNF280B;ZNF280C;ZNF280D;ZNF281;ZNF282;ZNF283;ZNF284;ZNF285;ZNF286A;ZNF286B;ZNF3;ZNF30;ZNF300;ZNF302;ZNF317;ZNF32;ZNF320;ZNF322;ZNF324;ZNF324B;ZNF329;ZNF331;ZNF333;ZNF33A;ZNF33B;ZNF343;ZNF345;ZNF347;ZNF350;ZNF354A;ZNF354B;ZNF362;ZNF366;ZNF383;ZNF384;ZNF394;ZNF397;ZNF398;ZNF404;ZNF41;ZNF410;ZNF419;ZNF420;ZNF431;ZNF432;ZNF436;ZNF439;ZNF44;ZNF440;ZNF442;ZNF443;ZNF446;ZNF449;ZNF45;ZNF460;ZNF468;ZNF479;ZNF484;ZNF490;ZNF500;ZNF502;ZNF524;ZNF525;ZNF528;ZNF543;ZNF544;ZNF546;ZNF547;ZNF548;ZNF549;ZNF554;ZNF555;ZNF557;ZNF558;ZNF559;ZNF561;ZNF562;ZNF563;ZNF564;ZNF566;ZNF567;ZNF568;ZNF57;ZNF570;ZNF571;ZNF572;ZNF577;ZNF581;ZNF582;ZNF583;ZNF585A;ZNF586;ZNF589;ZNF595;ZNF599;ZNF600;ZNF605;ZNF607;ZNF611;ZNF613;ZNF614;ZNF615;ZNF616;ZNF619;ZNF620;ZNF621;ZNF625;ZNF627;ZNF649;ZNF652;ZNF653;ZNF665;ZNF667;ZNF669;ZNF670;ZNF672;ZNF679;ZNF680;ZNF683;ZNF689;ZNF692;ZNF701;ZNF705D;ZNF705E;ZNF705G;ZNF708;ZNF709;ZNF71;ZNF710;ZNF713;ZNF721;ZNF727;ZNF729;ZNF736;ZNF75A;ZNF75D;ZNF76;ZNF763;ZNF764;ZNF765;ZNF768;ZNF77;ZNF771;ZNF773;ZNF774;ZNF776;ZNF777;ZNF780A;ZNF780B;ZNF782;ZNF785;ZNF	SCRT1;SCRT2;SNAI1;SNAI2

Motif	Nº of probes	% of probes	lower OR	upper OR	OR	p.value	FDR	TF family	TF subfamily	TF.family.member	TF.subfamily.member
										799;ZNF805;ZNF808;ZNF81;ZNF813;ZNF816;ZNF823;ZNF829;ZNF836;ZNF841;ZNF844;ZNF845;ZNF846;ZNF85;ZNF853;ZNF860;ZNF878;ZNF891;ZNF99;ZSCAN16;ZSCAN2;ZSCAN22;ZSCAN23;ZSCAN29;ZSCAN31;ZSCAN32;ZSCAN4;ZSCAN5A;ZSCAN5B;ZSCAN5C;ZSCAN9;ZXDA;ZXDB;ZXDC	
LHX9_HUMAN.H11MO.0.D	7	0.02745098	0.21211505	11.159.327	0.5332915	1,12E+05	0.3603587159	HD-LIM factors{3.1.5}	Lhx-2-like factors{3.1.5.3}	ISL1;ISL2;LHX1;LHX2;LHX3;LHX4;LHX5;LHX6;LHX8;LHX9;LMX1A;LMX1B	LHX2;LHX9
ZBT49_HUMAN.H11MO.0.D	5	0.01960784	0.16944119	12.461.935	0.5265694	1,80E+05	0.4667040255	More than 3 adjacent zinc finger factors{2.3.3}	unclassified{2.3.3.0}	BCL6B;BCL6;CTCFL;CTCF;FEZF1;GFI1B;GFI1;GLI1;GLI2;GLI3;GLIS1;GLIS2;GLIS3;MTF1;MYNN;MZF1;OSR2;OVOL1;OVOL2;ZNF146;PLAG1;PLAGL1;PRDM14;PRDM1;PRDM6;SCRT1;SCRT2;SNAI1;SNAI2;YY1;YY2;WT1;ZNF324;ZNF354A;ZBTB14;ZBTB18;ZBTB48;ZBTB49;ZBTB7A;ZBTB7B;ZBTB6;ZFP64;ZFP28;ZFP42;ZFP82;ZFX;ZIC1;ZIC2;ZIC3;ZIC4;ZIM3;ZKSCAN1;ZKSCAN3;ZNF121;ZNF136;ZNF140;ZNF143;ZNF148;ZNF214;ZNF232;ZNF250;ZNF257;ZNF260;ZNF263;ZNF264;ZNF274;ZNF281;ZNF282;ZNF317;ZNF320;ZNF322;ZNF329;ZNF331;ZNF333;ZNF350;ZNF384;ZNF394;ZNF410;ZNF436;ZNF449;ZNF490;ZNF502;ZNF524;ZNF528;ZNF547;ZNF549;ZNF554;ZNF563;ZNF582;ZNF586;ZNF589;ZNF652;ZNF667;ZNF680;ZNF708;ZNF713;ZNF768;ZNF816;ZNF18;ZNF41;ZNF76;ZNF85;ZSCAN16;ZSCAN22;ZSCAN31;ZSCAN4	MYNN;MZF1;OSR2;PRDM14;PRDM6;WT1;ZBTB14;ZBTB48;ZBTB49;ZFP64;ZFP28;ZIM3;ZNF121;ZNF250;ZNF257;ZNF263;ZNF274;ZNF317;ZNF320;ZNF329;ZNF331;ZNF394;ZNF449;ZNF502;ZNF528;ZNF547;ZNF549;ZNF554;ZNF586;ZNF589;ZNF667;ZNF680;ZNF708;ZNF713;ZNF768;ZNF18;ZNF85;ZSCAN16;ZSCAN22
PRGR_HUMAN.H11MO.0.A	12	0.04705882	0.26588300	0.9287748	0.5218685	2,48E+04	0.1372529551	Steroid hormone receptors (NR3){2.1.1}	GR-like receptors (NR3C){2.1.1.1}	AR;ESR1;ESR2;ESRRA;ESRRB;ESRRG;NR3C1;NR3C2;PGR	AR;NR3C1;NR3C2;PGR
PO3F2_HUMAN.H11MO.0.A	8	0.03137255	0.22219907	10.425.778	0.5206187	8,02E+04	0.2854511109	POU domain factors{3.1.10}	POU3 (Oct-6-like factors){3.1.10.3}	CDX1;CDX2;CDX4;EVX1;EVX2;GBX1;GBX2;GSX1;GSX2;HDX;HMBOX1;HNF1A;HNF1B;HOXA1;HOXA10;HOXA11;HOXA13;HOXA2;HOXA3;HOXA4;HOXA5;HOXA6;HOXA7;HOXA9;HOXB1;HOXB13;HOXB2;HOXB3;HOXB4;HOXB5;HOXB6;HOXB7;HOXB8;HOXB9;HOXC10;HOXC11;HOXC12;HOXC13;HOXC4;HOXC5;HOXC6;HOXC8;HOXC9;HOXD1;HOXD10;HOXD11;HOXD12;HOXD13;HOXD3;HOXD4;HOXD8;HOXD9;MEOX1;MEOX2;MNX1;PDX1;POU1F1;POU2F1;POU2F2;POU2F3;POU3F1;POU3F2;POU3F3;POU3F4;POU4F1;POU4F2;POU4F3;POU5F1;POU5F1B;POU5F2;POU6F1;POU6F2	POU3F1;POU3F2;POU3F3;POU3F4
BACH2_HUMAN.H11MO.0.A	16	0.06274510	0.29264110	0.8632063	0.5205035	7,55E+03	0.0589805058	Jun-related factors{1.1.1}	NF-E2-like factors{1.1.1.2}	ATF2;ATF7;BACH1;BACH2;CREB5;JUN;JUNB;JUND;NFE2;NFE2L1;NFE2L2;NFE2L3	BACH1;BACH2;NFE2L1;NFE2L2;NFE2
GSX1_HUMAN.H11MO.0.D	7	0.02745098	0.20689889	10.884.611	0.5201498	8,79E+04	0.3016918260	HOX-related factors{3.1.1}	GSX{3.1.1.12}	CDX1;CDX2;CDX4;EVX1;EVX2;GBX1;GBX2;GSX1;GSX2;HDX;HMBOX1;HNF1A;HNF1B;HOXA1;HOXA10;HOXA11;HOXA13;HOXA2;HOXA3;HOXA4;HOXA5;HOXA6;HOXA7;HOXA9;HOXB1;HOXB13;HOXB2;HOXB3;HOXB4;HOXB5;HOXB6;HOXB7;HOXB8;HOXB9;HOXC10;HOXC11;HOXC12;HOXC13;HOXC4;HOXC5;HOXC6;HOXC8;HOXC9;HOXD1;HOXD10;HOXD11;HOXD12;HOXD13;HOXD3;HOXD4;HOXD8;HOXD9;MEOX1;MEOX2;MNX1;PDX1;POU1F1;POU2F1;POU2F2;POU2F3;POU3F1;POU3F2;POU3F3;POU3F4;POU4F1;POU4F2;POU4F3;POU5F1;POU5F2;POU6F1;POU6F2	GSX1;GSX2
HXD8_HUMAN.H11MO.0.D	7	0.02745098	0.20569466	10.821.125	0.5171226	8,80E+04	0.3016918260	HOX-related factors{3.1.1}	HOX8{3.1.1.7}	CDX1;CDX2;CDX4;EVX1;EVX2;GBX1;GBX2;GSX1;GSX2;HDX;HMBOX1;HNF1A;HNF1B;HOXA1;HOXA10;HOXA11;HOXA13;HOXA2;HOXA3;HOXA4;HOXA5;HOXA6;HOXA7;HOXA9;HOXB1;HOXB13;HOXB2;HOXB3;HOXB4;HOXB5;HOXB6;HOXB7;HOXB8;HOXB9;HOXC10;HOXC11;HOXC12;HOXC13;HOXC4;HOXC5;HOXC6;HOXC8;HOXC9;HOXD1;HOXD10;HOXD11;HOXD12;HOXD13;HOXD3;HOXD4;HOXD8;HOXD9;MEOX1;MEOX2;MNX1;PDX1;POU1F1;POU2F1;POU2F2;POU2F3;POU3F1;POU3F2;POU3F3;POU3F4;POU4F1;POU4F2;POU4F3;POU5F1;POU5F2;POU6F1;POU6F2	HOXB8;HOXC8;HOXD8
RAX2_HUMAN.H11MO.0.D	6	0.02352941	0.18621904	11.320.524	0.5124745	1,27E+05	0.3867481384	Paired-related HD factors{3.1.3}	RAX{3.1.3.22}	ALX1;ALX3;ALX4;ARGFX;ARX;CRX;DMBX1;DPRX;DRGX;DUX4;DUXA;ESX1;GSC;GSC2;HESX1;ISX;LEUTX;MIXL1;NOBOX;OTP;OTX1;OTX2;PHOX2A;PHOX2B;PITX1;PITX2;PITX3;PROP1;PRRX1;PRRX2;RAX;RAX2;RHOXF1;RHOXF2;SEBOX;SHOX;SHOX2;TPRX1;UNCX;VSX1;VSX2	RAX2;RAX

Motif	Nº of probes	% of probes	lower OR	upper OR	OR	p.value	FDR	TF family	TF subfamily	TF.family.member	TF.subfamily.member
BSH_HUMAN.H11MO.0.D	6	0.02352941	0.18473682	11.231.476	0.5084454	1,28E+05	0.3883266665	NK-related factors{3.1.2}	BSX{3.1.2.3}	BARHL1;BARHL2;BARX1;BARX2;BSX;DBX1;DBX2;DLX1;DLX2;DLX3;DLX4;DLX5;DLX6;EMX1;EMX2;EN1;EN2;HHEX;HLX;HMX1;HMX2;HMX3;LBX1;LBX2;MSX1;MSX2;NANOG;NKX1-1;NKX1-2;NKX2-1;NKX2-2;NKX2-3;NKX2-4;NKX2-5;NKX2-6;NKX2-8;NKX3-1;NKX3-2;NKX6-1;NKX6-2;NKX6-3;NOTO;TLX1;TLX2;TLX3;VAX1;VAX2;VENTX	BSX
GATA5_HUMAN.H11MO.0.D	6	0.02352941	0.18391449	11.181.469	0.5061830	9,77E+04	0.3304320339	GATA-type zinc fingers{2.2.1}	Two zinc-finger GATA factors{2.2.1.1}	GATA1;GATA2;GATA3;GATA4;GATA5;GATA6;GATAD2A;GATAD2B;TRPS1;ZGLP1	GATA1;GATA2;GATA3;GATA4;GATA5;GATA6
PO2F2_HUMAN.H11MO.0.A	9	0.03529412	0.22853387	0.9766709	0.5058914	4,40E+04	0.1937915279	POU domain factors{3.1.10}	POU2 (Oct-1/2-like factors){3.1.10.2}	CDX1;CDX2;CDX4;EVX1;EVX2;GBX1;GBX2;GSX1;GSX2;HDX;HMBOX1;HNF1A;HNF1B;HOXA1;HOXA10;HOXA11;HOXA13;HOXA2;HOXA3;HOXA4;HOXA5;HOXA6;HOXA7;HOXA9;HOXB1;HOXB13;HOXB2;HOXB3;HOXB4;HOXB5;HOXB6;HOXB7;HOXB8;HOXB9;HOXC10;HOXC11;HOXC12;HOXC13;HOXC4;HOXC5;HOXC6;HOXC8;HOXC9;HOXD1;HOXD10;HOXD11;HOXD12;HOXD13;HOXD3;HOXD4;HOXD8;HOXD9;MEOX1;MEOX2;MNX1;PDX1;POU1F1;POU2F1;POU2F2;POU2F3;POU3F1;POU3F2;POU3F3;POU3F4;POU4F1;POU4F2;POU4F3;POU5F1;POU5F1B;POU5F2;POU6F1;POU6F2	POU2F1;POU2F2;POU2F3
JUN_HUMAN.H11MO.0.A	13	0.05098039	0.26408980	0.8775793	0.5033899	1,06E+04	0.0721403255	Jun-related factors{1.1.1}	Jun factors{1.1.1.1}	ATF2;ATF7;BACH1;BACH2;CREB5;JUN;JUNB;JUND;NFE2;NFE2L1;NFE2L2;NFE2L3	JUNB;JUND;JUN
NANOG_HUMAN.H11MO.0.A	9	0.03529412	0.22450696	0.9594412	0.4969708	3,40E+04	0.1714868564	NK-related factors{3.1.2}	NANOG{3.1.2.12}	BARHL1;BARHL2;BARX1;BARX2;BSX;DBX1;DBX2;DLX1;DLX2;DLX3;DLX4;DLX5;DLX6;EMX1;EMX2;EN1;EN2;HHEX;HLX;HMX1;HMX2;HMX3;LBX1;LBX2;MSX1;MSX2;NANOG;NKX1-1;NKX1-2;NKX2-1;NKX2-2;NKX2-3;NKX2-4;NKX2-5;NKX2-6;NKX2-8;NKX3-1;NKX3-2;NKX6-1;NKX6-2;NKX6-3;NOTO;TLX1;TLX2;TLX3;VAX1;VAX2;VENTX	NANOG
ARI3A_HUMAN.H11MO.0.D	14	0.05490196	0.26571440	0.8445711	0.4934801	7,51E+03	0.0589805058	ARID-related factors{3.7.1}	ARID3{3.7.1.3}	ARID2;ARID3A;ARID5A;ARID5B;KDM5B	ARID3A
HXD9_HUMAN.H11MO.0.D	3	0.01176471	0.10095470	14.590.253	0.4931040	2,98E+05	0.6092985589	HOX-related factors{3.1.1}	HOX9-13{3.1.1.8}	CDX1;CDX2;CDX4;EVX1;EVX2;GBX1;GBX2;GSX1;GSX2;HDX;HMBOX1;HNF1A;HNF1B;HOXA1;HOXA10;HOXA11;HOXA13;HOXA2;HOXA3;HOXA4;HOXA5;HOXA6;HOXA7;HOXA9;HOXB1;HOXB13;HOXB2;HOXB3;HOXB4;HOXB5;HOXB6;HOXB7;HOXB8;HOXB9;HOXC10;HOXC11;HOXC12;HOXC13;HOXC4;HOXC5;HOXC6;HOXC8;HOXC9;HOXD1;HOXD10;HOXD11;HOXD12;HOXD13;HOXD3;HOXD4;HOXD8;HOXD9;MEOX1;MEOX2;MNX1;PDX1;POU1F1;POU2F1;POU2F2;POU2F3;POU3F1;POU3F2;POU3F3;POU3F4;POU4F1;POU4F2;POU4F3;POU5F1;POU5F2;POU6F1;POU6F2	HOXA10;HOXA11;HOXA13;HOXA9;HOXB13;HOXC10;HOXC11;HOXC12;HOXC13;HOXC9;HOXD10;HOXD11;HOXD12;HOXD13;HOXD9
HNF1B_HUMAN.H11MO.0.A	7	0.02745098	0.19411299	10.211.586	0.4880083	5,32E+04	0.2175720934	POU domain factors{3.1.10}	HNF1-like factors{3.1.10.7}	CDX1;CDX2;CDX4;EVX1;EVX2;GBX1;GBX2;GSX1;GSX2;HDX;HMBOX1;HNF1A;HNF1B;HOXA1;HOXA10;HOXA11;HOXA13;HOXA2;HOXA3;HOXA4;HOXA5;HOXA6;HOXA7;HOXA9;HOXB1;HOXB13;HOXB2;HOXB3;HOXB4;HOXB5;HOXB6;HOXB7;HOXB8;HOXB9;HOXC10;HOXC11;HOXC12;HOXC13;HOXC4;HOXC5;HOXC6;HOXC8;HOXC9;HOXD1;HOXD10;HOXD11;HOXD12;HOXD13;HOXD3;HOXD4;HOXD8;HOXD9;MEOX1;MEOX2;MNX1;PDX1;POU1F1;POU2F1;POU2F2;POU2F3;POU3F1;POU3F2;POU3F3;POU3F4;POU4F1;POU4F2;POU4F3;POU5F1;POU5F1B;POU5F2;POU6F1;POU6F2	HMBOX1;HNF1A;HNF1B
FOSL1_HUMAN.H11MO.0.A	13	0.05098039	0.25480137	0.8466794	0.4856807	8,39E+03	0.0625511107	Fos-related factors{1.1.2}	Fos factors{1.1.2.1}	ATF3;FOS;FOSB;FOSL1;FOSL2;JDP2	FOSB;FOSL1;FOSL2;FOS
GATA6_HUMAN.H11MO.0.A	7	0.02745098	0.19244475	10.123.794	0.4838261	5,33E+04	0.2175720934	GATA-type zinc fingers{2.2.1}	Two zinc-finger GATA factors{2.2.1.1}	GATA1;GATA2;GATA3;GATA4;GATA5;GATA6;GATAD2A;GATAD2B;TRPS1;ZGLP1	GATA1;GATA2;GATA3;GATA4;GATA5;GATA6
DLX3_HUMAN.H11MO.0.C	6	0.02352941	0.17526217	10.654.682	0.4823520	7,59E+04	0.2830672629	NK-related factors{3.1.2}	DLX{3.1.2.5}	BARHL1;BARHL2;BARX1;BARX2;BSX;DBX1;DBX2;DLX1;DLX2;DLX3;DLX4;DLX5;DLX6;EMX1;EMX2;EN1;EN2;HHEX;HLX;HMX1;HMX2;HMX3;LBX1;LBX2;MSX1;MSX2;NANOG;NKX1-1;NKX1-2;NKX2-1;NKX2-2;NKX2-3;NKX2-	DLX1;DLX2;DLX3;DLX4;DLX5;DLX6

Motif	Nº of probes	% of probes	lower OR	upper OR	OR	p.value	FDR	TF family	TF subfamily	TF.family.member	TF.subfamily.member
										4;NKX2-5;NKX2-6;NKX2-8;NKX3-1;NKX3-2;NKX6-1;NKX6-2;NKX6-3;NOTO;TLX1;TLX2;TLX3;VAX1;VAX2;VENTX	
PO6F2_HUMAN.H11MO.0.D	6	0.02352941	0.17360754	10.553.689	0.4777909	7,63E+04	0.2830672629	POU domain factors{3.1.10}	POU6 (Brn-5-like factors){3.1.10.6}	CDX1;CDX2;CDX4;EVX1;EVX2;GBX1;GBX2;GSX1;GSX2;HDX;HMBOX1;HNF1A;HNF1B;HOXA1;HOXA10;HOXA11;HOXA13;HOXA2;HOXA3;HOXA4;HOXA5;HOXA6;HOXA7;HOXA9;HOXB1;HOXB13;HOXB2;HOXB3;HOXB4;HOXB5;HOXB6;HOXB7;HOXB8;HOXB9;HOXC10;HOXC11;HOXC12;HOXC13;HOXC4;HOXC5;HOXC6;HOXC8;HOXC9;HOXD1;HOXD10;HOXD11;HOXD12;HOXD13;HOXD3;HOXD4;HOXD8;HOXD9;MEOX1;MEOX2;MNX1;PDX1;POU1F1;POU2F1;POU2F2;POU2F3;POU3F1;POU3F2;POU3F3;POU3F4;POU4F1;POU4F2;POU4F3;POU5F1;POU5F1B;POU5F2;POU6F1;POU6F2	POU6F1;POU6F2
ZN232_HUMAN.H11MO.0.D	6	0.02352941	0.17340286	10.541.207	0.4772265	7,64E+04	0.2830672629	More than 3 adjacent zinc finger factors{2.3.3}	ZNF24-like factors{2.3.3.10}	BCL6;BCL6B;CTCF;CTCFL;FEZF1;FEZF2;GFI1;GFI1B;GLI1;GLI2;GLI3;GLI4;GLIS1;GLIS2;GLIS3;HKR1;MTF1;MYNN;MZF1;OSR2;OVOL1;OVOL2;PLAG1;PLAGL1;PLAGL2;PRDM1;PRDM14;PRDM6;SCRT1;SCRT2;SNAI1;SNAI2;SNAI3;WT1;YY1;YY2;ZBTB12;ZBTB14;ZBTB18;ZBTB20;ZBTB26;ZBTB42;ZBTB45;ZBTB47;ZBTB48;ZBTB49;ZBTB6;ZBTB7A;ZBTB7B;ZBTB7C;ZFP14;ZFP2;ZFP28;ZFP30;ZFP37;ZFP42;ZFP64;ZFP69;ZFP69B;ZFP82;ZFP91;ZFX;ZIC1;ZIC2;ZIC3;ZIC4;ZIC5;ZIK1;ZIM3;ZKSCAN1;ZKSCAN2;ZKSCAN3;ZKSCAN4;ZNF121;ZNF124;ZNF133;ZNF136;ZNF138;ZNF14;ZNF140;ZNF143;ZNF146;ZNF148;ZNF155;ZNF157;ZNF160;ZNF169;ZNF175;ZNF177;ZNF18;ZNF180;ZNF181;ZNF2;ZNF20;ZNF212;ZNF213;ZNF214;ZNF221;ZNF222;ZNF223;ZNF224;ZNF225;ZNF226;ZNF227;ZNF229;ZNF230;ZNF232;ZNF233;ZNF234;ZNF235;ZNF24;ZNF25;ZNF250;ZNF257;ZNF26;ZNF260;ZNF263;ZNF264;ZNF268;ZNF274;ZNF276;ZNF28;ZNF280A;ZNF280B;ZNF280C;ZNF280D;ZNF281;ZNF282;ZNF283;ZNF284;ZNF285;ZNF286A;ZNF286B;ZNF3;ZNF30;ZNF300;ZNF302;ZNF317;ZNF32;ZNF320;ZNF322;ZNF324;ZNF324B;ZNF329;ZNF331;ZNF333;ZNF33A;ZNF33B;ZNF343;ZNF345;ZNF347;ZNF350;ZNF354A;ZNF354B;ZNF362;ZNF366;ZNF383;ZNF384;ZNF394;ZNF397;ZNF398;ZNF404;ZNF41;ZNF410;ZNF419;ZNF420;ZNF431;ZNF432;ZNF436;ZNF439;ZNF44;ZNF440;ZNF442;ZNF443;ZNF446;ZNF449;ZNF45;ZNF460;ZNF468;ZNF479;ZNF484;ZNF490;ZNF500;ZNF502;ZNF524;ZNF525;ZNF528;ZNF543;ZNF544;ZNF546;ZNF547;ZNF548;ZNF549;ZNF554;ZNF555;ZNF557;ZNF558;ZNF559;ZNF561;ZNF562;ZNF563;ZNF564;ZNF566;ZNF567;ZNF568;ZNF57;ZNF570;ZNF571;ZNF572;ZNF577;ZNF581;ZNF582;ZNF583;ZNF585A;ZNF586;ZNF589;ZNF595;ZNF599;ZNF600;ZNF605;ZNF607;ZNF611;ZNF613;ZNF614;ZNF615;ZNF616;ZNF619;ZNF620;ZNF621;ZNF625;ZNF627;ZNF649;ZNF652;ZNF653;ZNF665;ZNF667;ZNF669;ZNF670;ZNF672;ZNF679;ZNF680;ZNF683;ZNF689;ZNF692;ZNF701;ZNF705D;ZNF705E;ZNF705G;ZNF708;ZNF709;ZNF71;ZNF710;ZNF713;ZNF721;ZNF727;ZNF729;ZNF736;ZNF75A;ZNF75D;ZNF76;ZNF763;ZNF764;ZNF765;ZNF768;ZNF77;ZNF771;ZNF773;ZNF774;ZNF776;ZNF777;ZNF780A;ZNF780B;ZNF782;ZNF785;ZNF799;ZNF805;ZNF808;ZNF81;ZNF813;ZNF816;ZNF823;ZNF829;ZNF836;ZNF841;ZNF844;ZNF845;ZNF846;ZNF85;ZNF853;ZNF860;ZNF878;ZNF891;ZNF99;ZSCAN16;ZSCAN2;ZSCAN22;ZSCAN23;ZSCAN29;ZSCAN31;ZSCAN32;ZSCAN4;ZSCAN5A;ZSCAN5B;ZSCAN5C;ZSCAN9;ZXDA;ZXDB;ZXDC	ZKSCAN1;ZNF232;ZSCAN31
HNF4G_HUMAN.H11MO.0.B	12	0.04705882	0.24009724	0.8386313	0.4712179	7,10E+03	0.0573233912	RXR-related receptors (NR2){2.1.3}	HNF-4 (NR2A){2.1.3.2}	HNF4A;HNF4G;NR2C1;NR2C2;NR2E1;NR2E3;NR2F1;NR2F2;NR2F6;RXRA;RXRB;RXRG	HNF4A;HNF4G
PO3F3_HUMAN.H11MO.0.D	12	0.04705882	0.23931470	0.8359249	0.4696968	7,14E+03	0.0573233912	POU domain factors{3.1.10}	POU3 (Oct-6-like factors){3.1.10.3}	CDX1;CDX2;CDX4;EVX1;EVX2;GBX1;GBX2;GSX1;GSX2;HDX;HMBOX1;HNF1A;HNF1B;HOXA1;HOXA10;HOXA11;HOXA13;HOXA2;HOXA3;HOXA4;HOXA5;HOXA6;HOXA7;HOXA9;HOXB1;HOXB13;HOXB2;HOXB3;HOXB4;HOXB5;HOXB6;HOXB7;HOXB8;HOXB9;HOXC10;HOXC11;HOXC12;HOXC13;HOXC4;HOXC5;HOXC6;HOXC8;HOXC9;HOXD1;HO	POU3F1;POU3F2;POU3F3;POU3F4

Motif	Nº of probes	% of probes	lower OR	upper OR	OR	p.value	FDR	TF family	TF subfamily	TF.family.member	TF.subfamily.member
										XD10;HOXD11;HOXD12;HOXD13;HOXD3;HOXD4;HOXD8;HOXD9;MEOX1;MEOX2;MNX1;PDX1;POU1F1;POU2F1;POU2F2;POU2F3;POU3F1;POU3F2;POU3F3;POU3F4;POU4F1;POU4F2;POU4F3;POU5F1;POU5F1B;POU5F2;POU6F1;POU6F2	
FOSB_HUMAN.H11MO.0.A	12	0.04705882	0.23605800	0.8245951	0.4633300	5,45E+03	0.0488772307	Fos-related factors{1.1.2}	Fos factors{1.1.2.1}	ATF3;FOS;FOSB;FOSL1;FOSL2;JDP2	FOSB;FOSL1;FOSL2;FOS
EVI1_HUMAN.H11MO.0.B	7	0.02745098	0.18410566	0.9683190	0.4627801	4,17E+04	0.1867821692	Factors with multiple dispersed zinc fingers {2.3.4}	Evi-1-like factors{2.3.4.14}	BCL11A;BCL11B;BNC1;BNC2;E4F1;HIC1;HIC2;HINFP;HIVEP1;HIVEP2;HIVEP3;IKZF1;IKZF2;IKZF3;IKZF4;IKZF5;INSM1;INSM2;MAZ;MECOM;PATZ1;PRDM16;PRDM4;REST;RLF;RREB1;SALL1;SALL2;SALL3;SALL4;VEZF1;ZBTB1;ZBTB17;ZBTB2;ZBTB25;ZBTB4;ZFAT;ZNF134;ZNF211;ZNF217;ZNF219;ZNF248;ZNF256;ZNF292;ZNF296;ZNF319;ZNF334;ZNF335;ZNF341;ZNF37A;ZNF382;ZNF417;ZNF418;ZNF423;ZNF467;ZNF510;ZNF512;ZNF512B;ZNF516;ZNF518A;ZNF518B;ZNF521;ZNF526;ZNF532;ZNF536;ZNF552;ZNF574;ZNF587;ZNF587B;ZNF592;ZNF639;ZNF654;ZNF658;ZNF671;ZNF687;ZNF711;ZNF717;ZNF770;ZNF772;ZNF784;ZNF786;ZNF792;ZNF8;ZNF814	MECOM
GSX2_HUMAN.H11MO.0.D	7	0.02745098	0.18399307	0.9680429	0.4626482	4,17E+04	0.1867821692	HOX-related factors{3.1.1}	GSX{3.1.1.12}	CDX1;CDX2;CDX4;EVX1;EVX2;GBX1;GBX2;GSX1;GSX2;HDX;HMBOX1;HNF1A;HNF1B;HOXA1;HOXA10;HOXA11;HOXA13;HOXA2;HOXA3;HOXA4;HOXA5;HOXA6;HOXA7;HOXA9;HOXB1;HOXB13;HOXB2;HOXB3;HOXB4;HOXB5;HOXB6;HOXB7;HOXB8;HOXB9;HOXC10;HOXC11;HOXC12;HOXC13;HOXC4;HOXC5;HOXC6;HOXC8;HOXC9;HOXD1;HOXD10;HOXD11;HOXD12;HOXD13;HOXD3;HOXD4;HOXD8;HOXD9;MEOX1;MEOX2;MNX1;PDX1;POU1F1;POU2F1;POU2F2;POU2F3;POU3F1;POU3F2;POU3F3;POU3F4;POU4F1;POU4F2;POU4F3;POU5F1;POU5F2;POU6F1;POU6F2	GSX1;GSX2
HXD12_HUMAN.H11MO.0.D	8	0.03137255	0.19733226	0.9258965	0.4623828	2,24E+04	0.1267136742	HOX-related factors{3.1.1}	HOX9-13{3.1.1.8}	CDX1;CDX2;CDX4;EVX1;EVX2;GBX1;GBX2;GSX1;GSX2;HDX;HMBOX1;HNF1A;HNF1B;HOXA1;HOXA10;HOXA11;HOXA13;HOXA2;HOXA3;HOXA4;HOXA5;HOXA6;HOXA7;HOXA9;HOXB1;HOXB13;HOXB2;HOXB3;HOXB4;HOXB5;HOXB6;HOXB7;HOXB8;HOXB9;HOXC10;HOXC11;HOXC12;HOXC13;HOXC4;HOXC5;HOXC6;HOXC8;HOXC9;HOXD1;HOXD10;HOXD11;HOXD12;HOXD13;HOXD3;HOXD4;HOXD8;HOXD9;MEOX1;MEOX2;MNX1;PDX1;POU1F1;POU2F1;POU2F2;POU2F3;POU3F1;POU3F2;POU3F3;POU3F4;POU4F1;POU4F2;POU4F3;POU5F1;POU5F2;POU6F1;POU6F2	HOXA10;HOXA11;HOXA13;HOXA9;HOXB13;HOXC10;HOXC11;HOXC12;HOXC13;HOXC9;HOXD10;HOXD11;HOXD12;HOXD13;HOXD9
FOSL2_HUMAN.H11MO.0.A	12	0.04705882	0.23433192	0.8185632	0.4599411	5,51E+03	0.0488772307	Fos-related factors{1.1.2}	Fos factors{1.1.2.1}	ATF3;FOS;FOSB;FOSL1;FOSL2;JDP2	FOSB;FOSL1;FOSL2;FOS
FOXH1_HUMAN.H11MO.0.A	9	0.03529412	0.20542603	0.8778883	0.4547752	1,60E+04	0.0965085819	Forkhead box (FOX) factors{3.3.1}	FOXH{3.3.1.8}	FOXA1;FOXA2;FOXA3;FOXB1;FOXB2;FOXC1;FOXC2;FOXD1;FOXD2;FOXD3;FOXD4;FOXD4L1;FOXD4L3;FOXD4L4;FOXD4L5;FOXD4L6;FOXE1;FOXE3;FOXF1;FOXF2;FOXG1;FOXH1;FOXI1;FOXI2;FOXI3;FOXJ1;FOXJ2;FOXJ3;FOXK1;FOXK2;FOXL1;FOXL2;FOXM1;FOXN1;FOXN2;FOXN3;FOXN4;FOXO1;FOXO3;FOXO4;FOXO6;FOXP1;FOXP2;FOXP3;FOXP4;FOXQ1;FOXR1;FOXR2;FOXS1	FOXH1
FOS_HUMAN.H11MO.0.A	12	0.04705882	0.23163345	0.8091320	0.4546427	4,18E+03	0.0403138639	Fos-related factors{1.1.2}	Fos factors{1.1.2.1}	ATF3;FOS;FOSB;FOSL1;FOSL2;JDP2	FOSB;FOSL1;FOSL2;FOS
PO2F3_HUMAN.H11MO.0.D	6	0.02352941	0.16384787	0.9960995	0.4509738	4,48E+04	0.1940528864	POU domain factors{3.1.10}	POU2 (Oct-1/2-like factors){3.1.10.2}	CDX1;CDX2;CDX4;EVX1;EVX2;GBX1;GBX2;GSX1;GSX2;HDX;HMBOX1;HNF1A;HNF1B;HOXA1;HOXA10;HOXA11;HOXA13;HOXA2;HOXA3;HOXA4;HOXA5;HOXA6;HOXA7;HOXA9;HOXB1;HOXB13;HOXB2;HOXB3;HOXB4;HOXB5;HOXB6;HOXB7;HOXB8;HOXB9;HOXC10;HOXC11;HOXC12;HOXC13;HOXC4;HOXC5;HOXC6;HOXC8;HOXC9;HOXD1;HOXD10;HOXD11;HOXD12;HOXD13;HOXD3;HOXD4;HOXD8;HOXD9;MEOX1;MEOX2;MNX1;PDX1;POU1F1;POU2F1;POU2F2;POU2F3;POU3F1;POU3F2;POU3F3;POU3F4;POU4F1;POU4F2;POU4F3;POU5F1;POU5F1B;POU5F2;POU6F1;POU6F2	POU2F1;POU2F2;POU2F3

Motif	Nº of probes	% of probes	lower OR	upper OR	OR	p.value	FDR	TF family	TF subfamily	TF.family.member	TF.subfamily.member
LMX1A_HUMAN.H11MO.0.D	10	0.03921569	0.21031568	0.8314074	0.4442845	6,51E+03	0.0545580619	HD-LIM factors{3.1.5}	Lmx{3.1.5.6}	ISL1;ISL2;LHX1;LHX2;LHX3;LHX4;LHX5;LHX6;LHX8;LHX9;LMX1A;LMX1B	LMX1A;LMX1B
PRRX1_HUMAN.H11MO.0.D	5	0.01960784	0.14201586	10.443.073	0.4413020	6,36E+04	0.2499855315	Paired-related HD factors{3.1.3}	PRRX{3.1.3.21}	ALX1;ALX3;ALX4;ARGFX;ARX;CRX;DMBX1;DPRX;DRGX;DUX4;DUXA;ESX1;GSC;GSC2;HESX1;ISX;LEUTX;MIXL1;NOBOX;OTP;OTX1;OTX2;PHOX2A;PHOX2B;PITX1;PITX2;PITX3;PROP1;PRRX1;PRRX2;RAX;RAX2;RHOXF1;RHOXF2;SEBOX;SHOX;SHOX2;TPRX1;UNCX;VSX1;VSX2	PRRX1;PRRX2
ATF2_HUMAN.H11MO.2.C	12	0.04705882	0.22462956	0.7846455	0.4408879	3,27E+03	0.0327080111	Jun-related factors{1.1.1}	ATF-2-like factors{1.1.1.3}	ATF2;ATF7;BACH1;BACH2;CREB5;JUN;JUNB;JUND;NFE2;NFE2L1;NFE2L2;NFE2L3	ATF2;ATF7;CREB5
HNF6_HUMAN.H11MO.0.B	4	0.01568627	0.11897936	11.421.685	0.4402780	1,20E+05	0.3759318182	HD-CUT factors{3.1.9}	ONECUT{3.1.9.1}	CUX1;CUX2;ONECUT1;ONECUT2;ONECUT3;SATB1;SATB2	ONECUT1;ONECUT2;ONECUT3
MSX1_HUMAN.H11MO.0.D	6	0.02352941	0.15826674	0.9620815	0.4355790	3,42E+04	0.1714868564	NK-related factors{3.1.2}	MSX{3.1.2.11}	BARHL1;BARHL2;BARX1;BARX2;BSX;DBX1;DBX2;DLX1;DLX2;DLX3;DLX4;DLX5;DLX6;EMX1;EMX2;EN1;EN2;HHEX;HLX;HMX1;HMX2;HMX3;LBX1;LBX2;MSX1;MSX2;NANOG;NKX1-1;NKX1-2;NKX2-1;NKX2-2;NKX2-3;NKX2-4;NKX2-5;NKX2-6;NKX2-8;NKX3-1;NKX3-2;NKX6-1;NKX6-2;NKX6-3;NOTO;TLX1;TLX2;TLX3;VAX1;VAX2;VENTX	MSX1;MSX2
RORG_HUMAN.H11MO.0.C	9	0.03529412	0.19578957	0.8366800	0.4334266	9,40E+03	0.0665145065	Thyroid hormone receptor-related factors (NR1){2.1.2}	ROR (NR1F){2.1.2.6}	NR1D1;NR1D2;NR1H2;NR1H3;NR1H4;NR1I2;NR1I3;PPARA;PPARD;PPARG;RARA;RARB;RARG;RORA;RORB;RORC;THRA;THRB;VDR	RORA;RORC
HXA9_HUMAN.H11MO.0.B	6	0.02352941	0.15653710	0.9515699	0.4308214	3,44E+04	0.1714868564	HOX-related factors{3.1.1}	HOX9-13{3.1.1.8}	CDX1;CDX2;CDX4;EVX1;EVX2;GBX1;GBX2;GSX1;GSX2;HDX;HMBOX1;HNF1A;HNF1B;HOXA1;HOXA10;HOXA11;HOXA13;HOXA2;HOXA3;HOXA4;HOXA5;HOXA6;HOXA7;HOXA9;HOXB1;HOXB13;HOXB2;HOXB3;HOXB4;HOXB5;HOXB6;HOXB7;HOXB8;HOXB9;HOXC10;HOXC11;HOXC12;HOXC13;HOXC4;HOXC5;HOXC6;HOXC8;HOXC9;HOXD1;HOXD10;HOXD11;HOXD12;HOXD13;HOXD3;HOXD4;HOXD8;HOXD9;MEOX1;MEOX2;MNX1;PDX1;POU1F1;POU2F1;POU2F2;POU2F3;POU3F1;POU3F2;POU3F3;POU3F4;POU4F1;POU4F2;POU4F3;POU5F1;POU5F2;POU6F1;POU6F2	HOXA10;HOXA11;HOXA13;HOXA9;HOXB13;HOXC10;HOXC11;HOXC12;HOXC13;HOXC9;HOXD10;HOXD11;HOXD12;HOXD13;HOXD9
HMBX1_HUMAN.H11MO.0.D	5	0.01960784	0.13693970	10.070.897	0.4255894	4,83E+04	0.2056256305	POU domain factors{3.1.10}	HNF1-like factors{3.1.10.7}	CDX1;CDX2;CDX4;EVX1;EVX2;GBX1;GBX2;GSX1;GSX2;HDX;HMBOX1;HNF1A;HNF1B;HOXA1;HOXA10;HOXA11;HOXA13;HOXA2;HOXA3;HOXA4;HOXA5;HOXA6;HOXA7;HOXA9;HOXB1;HOXB13;HOXB2;HOXB3;HOXB4;HOXB5;HOXB6;HOXB7;HOXB8;HOXB9;HOXC10;HOXC11;HOXC12;HOXC13;HOXC4;HOXC5;HOXC6;HOXC8;HOXC9;HOXD1;HOXD10;HOXD11;HOXD12;HOXD13;HOXD3;HOXD4;HOXD8;HOXD9;MEOX1;MEOX2;MNX1;PDX1;POU1F1;POU2F1;POU2F2;POU2F3;POU3F1;POU3F2;POU3F3;POU3F4;POU4F1;POU4F2;POU4F3;POU5F1;POU5F1B;POU5F2;POU6F1;POU6F2	HMBOX1;HNF1A;HNF1B
DMBX1_HUMAN.H11MO.0.D	6	0.02352941	0.15418318	0.9372719	0.4243497	3,51E+04	0.1724127549	Paired-related HD factors{3.1.3}	DMBX{3.1.3.4}	ALX1;ALX3;ALX4;ARGFX;ARX;CRX;DMBX1;DPRX;DRGX;DUX4;DUXA;ESX1;GSC;GSC2;HESX1;ISX;LEUTX;MIXL1;NOBOX;OTP;OTX1;OTX2;PHOX2A;PHOX2B;PITX1;PITX2;PITX3;PROP1;PRRX1;PRRX2;RAX;RAX2;RHOXF1;RHOXF2;SEBOX;SHOX;SHOX2;TPRX1;UNCX;VSX1;VSX2	DMBX1
ATF2_HUMAN.H11MO.1.B	7	0.02745098	0.16449190	0.8652513	0.4135313	1,41E+04	0.0862047325	Jun-related factors{1.1.1}	ATF-2-like factors{1.1.1.3}	ATF2;ATF7;BACH1;BACH2;CREB5;JUN;JUNB;JUND;NFE2;NFE2L1;NFE2L2;NFE2L3	ATF2;ATF7;CREB5
CEBPE_HUMAN.H11MO.0.A	5	0.01960784	0.13217048	0.9720013	0.4107686	3,65E+04	0.1745771031	C/EBP-related{1.1.8}	C/EBP{1.1.8.1}	CEBPA;CEBPB;CEBPD;CEBPE;CEBPG;DBP;DDIT3;HLF;NFIL3;TEF	CEBPA;CEBPB;CEBPD;CEBPE;CEBPG;DDIT3
PO2F1_HUMAN.H11MO.0.C	7	0.02745098	0.16208434	0.8525740	0.4074763	1,06E+04	0.0721403255	POU domain factors{3.1.10}	POU2 (Oct-1/2-like factors){3.1.10.2}	CDX1;CDX2;CDX4;EVX1;EVX2;GBX1;GBX2;GSX1;GSX2;HDX;HMBOX1;HNF1A;HNF1B;HOXA1;HOXA10;HOXA11;HOXA13;HOXA2;HOXA3;HOXA4;HOXA5;HOXA6;HOXA7;HOXA9;HOXB1;HOXB13;HOXB2;HOXB3;HOXB4;HOXB5;HOXB6;HOXB7;HOXB8;HOXB9;HOXC10;HOXC11;HOXC12;HOXC13;HOXC4;HOXC5;HOXC6;HOXC8;HOXC9;HOXD1;HOXD10;HOXD11;HOXD12;HOXD13;HOXD3;HOXD4;HOXD8;HOXD9;MEOX1;MEOX2;MNX1;PDX1;POU1F1;POU2F1;PO	POU2F1;POU2F2;POU2F3

Motif	Nº of probes	% of probes	lower OR	upper OR	OR	p.value	FDR	TF family	TF subfamily	TF.family.member	TF.subfamily.member
										U2F2;POU2F3;POU3F1;POU3F2;POU3F3;POU3F4;POU4F1;POU4F2;POU4F3;POU5F1;POU5F1B;POU5F2;POU6F1;POU6F2	
ZN250_HUMAN.H11MO.0.C	7	0.02745098	0.16118063	0.8478147	0.4052032	1,06E+04	0.0721403255	More than 3 adjacent zinc finger factors{2.3.3}	unclassified{2.3.3.0}	BCL6B;BCL6;CTCFL;CTCF;FEZF1;GFI1B;GFI1;GLI1;GLI2;GLI3;GLIS1;GLIS2;GLIS3;MTF1;MYNN;MZF1;OSR2;OVOL1;OVOL2;ZNF146;PLAG1;PLAGL1;PRDM14;PRDM1;PRDM6;SCRT1;SCRT2;SNAI1;SNAI2;YY1;YY2;WT1;ZNF324;ZNF354A;ZBTB14;ZBTB18;ZBTB48;ZBTB49;ZBTB7A;ZBTB7B;ZBTB6;ZFP64;ZFP28;ZFP42;ZFP82;ZFX;ZIC1;ZIC2;ZIC3;ZIC4;ZIM3;ZKSCAN1;ZKSCAN3;ZNF121;ZNF136;ZNF140;ZNF143;ZNF148;ZNF214;ZNF232;ZNF250;ZNF257;ZNF260;ZNF263;ZNF264;ZNF274;ZNF281;ZNF282;ZNF317;ZNF320;ZNF322;ZNF329;ZNF331;ZNF333;ZNF350;ZNF384;ZNF394;ZNF410;ZNF436;ZNF449;ZNF490;ZNF502;ZNF524;ZNF528;ZNF547;ZNF549;ZNF554;ZNF563;ZNF582;ZNF586;ZNF589;ZNF652;ZNF667;ZNF680;ZNF708;ZNF713;ZNF768;ZNF816;ZNF18;ZNF41;ZNF76;ZNF85;ZSCAN16;ZSCAN22;ZSCAN31;ZSCAN4	MYNN;MZF1;OSR2;PRDM14;PRDM6;WT1;ZBTB14;ZBTB48;ZBTB49;ZFP64;ZFP28;ZIM3;ZNF121;ZNF250;ZNF257;ZNF263;ZNF274;ZNF317;ZNF320;ZNF329;ZNF331;ZNF394;ZNF449;ZNF502;ZNF528;ZNF547;ZNF549;ZNF554;ZNF586;ZNF589;ZNF667;ZNF680;ZNF708;ZNF713;ZNF768;ZNF18;ZNF85;ZSCAN16;ZSCAN22
PO3F4_HUMAN.H11MO.0.D	7	0.02745098	0.15978240	0.8404322	0.4016860	1,07E+04	0.0721403255	POU domain factors{3.1.10}	POU3 (Oct-6-like factors){3.1.10.3}	CDX1;CDX2;CDX4;EVX1;EVX2;GBX1;GBX2;GSX1;GSX2;HDX;HMBOX1;HNF1A;HNF1B;HOXA1;HOXA10;HOXA11;HOXA13;HOXA2;HOXA3;HOXA4;HOXA5;HOXA6;HOXA7;HOXA9;HOXB1;HOXB13;HOXB2;HOXB3;HOXB4;HOXB5;HOXB6;HOXB7;HOXB8;HOXB9;HOXC10;HOXC11;HOXC12;HOXC13;HOXC4;HOXC5;HOXC6;HOXC8;HOXC9;HOXD1;HOXD10;HOXD11;HOXD12;HOXD13;HOXD3;HOXD4;HOXD8;HOXD9;MEOX1;MEOX2;MNX1;PDX1;POU1F1;POU2F1;POU2F2;POU2F3;POU3F1;POU3F2;POU3F3;POU3F4;POU4F1;POU4F2;POU4F3;POU5F1;POU5F1B;POU5F2;POU6F1;POU6F2	POU3F1;POU3F2;POU3F3;POU3F4
DMRT1_HUMAN.H11MO.0.D	6	0.02352941	0.14309129	0.8698660	0.3938408	1,48E+04	0.0900207328	DMRT{2.5.1}	DMRT1{2.5.1.0.1}	DMRT1;DMRT2;DMRT3;DMRTA1;DMRTA2;DMRTB1;DMRTC2	DMRT1
NR1D1_HUMAN.H11MO.1.D	5	0.01960784	0.12032793	0.8848332	0.3739486	2,09E+04	0.1220397538	Thyroid hormone receptor-related factors (NR1){2.1.2}	Rev-ErbA (NR1D){2.1.2.3}	NR1D1;NR1D2;NR1H2;NR1H3;NR1H4;NR1I2;NR1I3;PPARA;PPARD;PPARG;RARA;RARB;RARG;RORA;RORB;RORC;THRA;THRB;VDR	NR1D1
NANOG_HUMAN.H11MO.1.B	6	0.02352941	0.13569644	0.8247942	0.3734498	8,31E+03	0.0625511107	NK-related factors{3.1.2}	NANOG{3.1.2.12}	BARHL1;BARHL2;BARX1;BARX2;BSX;DBX1;DBX2;DLX1;DLX2;DLX3;DLX4;DLX5;DLX6;EMX1;EMX2;EN1;EN2;HHEX;HLX;HMX1;HMX2;HMX3;LBX1;LBX2;MSX1;MSX2;NANOG;NKX1-1;NKX1-2;NKX2-1;NKX2-2;NKX2-3;NKX2-4;NKX2-5;NKX2-6;NKX2-8;NKX3-1;NKX3-2;NKX6-1;NKX6-2;NKX6-3;NOTO;TLX1;TLX2;TLX3;VAX1;VAX2;VENTX	NANOG
CUX2_HUMAN.H11MO.0.D	4	0.01568627	0.10080180	0.9676576	0.3730412	3,84E+04	0.1805496224	HD-CUT factors{3.1.9}	CUX{3.1.9.2}	CUX1;CUX2;ONECUT1;ONECUT2;ONECUT3;SATB1;SATB2	CUX1;CUX2
NKX32_HUMAN.H11MO.0.C	5	0.01960784	0.11986715	0.8814415	0.3725159	2,11E+04	0.1220397538	NK-related factors{3.1.2}	NK-3{3.1.2.16}	BARHL1;BARHL2;BARX1;BARX2;BSX;DBX1;DBX2;DLX1;DLX2;DLX3;DLX4;DLX5;DLX6;EMX1;EMX2;EN1;EN2;HHEX;HLX;HMX1;HMX2;HMX3;LBX1;LBX2;MSX1;MSX2;NANOG;NKX1-1;NKX1-2;NKX2-1;NKX2-2;NKX2-3;NKX2-4;NKX2-5;NKX2-6;NKX2-8;NKX3-1;NKX3-2;NKX6-1;NKX6-2;NKX6-3;NOTO;TLX1;TLX2;TLX3;VAX1;VAX2;VENTX	NKX3-1;NKX3-2
ATF7_HUMAN.H11MO.0.D	10	0.03921569	0.17306140	0.6840001	0.3655290	5,05E+02	0.0056378899	Jun-related factors{1.1.1}	ATF-2-like factors{1.1.1.3}	ATF2;ATF7;BACH1;BACH2;CREB5;JUN;JUNB;JUND;NFE2;NFE2L1;NFE2L2;NFE2L3	ATF2;ATF7;CREB5
HOMEZ_HUMAN.H11MO.0.D	6	0.02352941	0.13096641	0.7961129	0.3604677	6,19E+03	0.0530527548	HD-ZF factors{3.1.8}	ZHX{3.1.8.5}	ADNP;ADNP2;HOMEZ;NANOGNB;TSHZ1;TSHZ2;TSHZ3;ZEB1;ZEB2;ZFHX2;ZFHX3;ZFHX4;ZHX1;ZHX2;ZHX3	HOMEZ
CDX1_HUMAN.H11MO.0.C	5	0.01960784	0.11451880	0.8420652	0.3558834	1,15E+04	0.0751577421	HOX-related factors{3.1.1}	CDX (Caudal type homeobox){3.1.1.9}	CDX1;CDX2;CDX4;EVX1;EVX2;GBX1;GBX2;GSX1;GSX2;HDX;HMBOX1;HNF1A;HNF1B;HOXA1;HOXA10;HOXA11;HOXA13;HOXA2;HOXA3;HOXA4;HOXA5;HOXA6;HOXA7;HOXA9;HOXB1;HOXB13;HOXB2;HOXB3;HOXB4;HOXB5;HOXB6;HOXB7;HOXB8;HOXB9;HOXC10;HOXC11;HOXC12;HOXC13;HOXC4;HOXC5;HOXC6;HOXC8;HOXC9;HOXD1;HOXD10;HOXD11;HOXD12;HOXD13;HOXD3;HOXD4;HOXD8;	CDX1;CDX2

Motif	Nº of probes	% of probes	lower OR	upper OR	OR	p.value	FDR	TF family	TF subfamily	TF.family.member	TF.subfamily.member
										HOXD9;MEOX1;MEOX2;MNX1;PDX1;POU1F1;POU2F1;POU2F2;POU2F3;POU3F1;POU3F2;POU3F3;POU3F4;POU4F1;POU4F2;POU4F3;POU5F1;POU5F2;POU6F1;POU6F2	
JUNB_HUMAN.H11MO.0.A	10	0.03921569	0.16803118	0.6641359	0.3549125	2,72E+02	0.0032479474	Jun-related factors{1.1.1}	Jun factors{1.1.1.1}	ATF2;ATF7;BACH1;BACH2;CREB5;JUN;JUNB;JUND;NFE2;NFE2L1;NFE2L2;NFE2L3	JUNB;JUND;JUN
P5F1B_HUMAN.H11MO.0.D	6	0.02352941	0.12546264	0.7624963	0.3452655	4,71E+03	0.0431918038	POU domain factors{3.1.10}	POU5 (Oct-3/4-like factors){3.1.10.5}	HMBOX1;HNF1A;HNF1B;POU5F1B;POU1F1;POU2F1;POU2F2;POU2F3;POU3F1;POU3F2;POU3F3;POU3F4;POU4F1;POU4F2;POU4F3;POU5F1;POU6F1;POU6F2	POU5F1B;POU5F1
NKX61_HUMAN.H11MO.0.B	5	0.01960784	0.10806356	0.7945151	0.3357985	8,80E+03	0.0633789603	NK-related factors{3.1.2}	NK-6{3.1.2.19}	BARHL1;BARHL2;BARX1;BARX2;BSX;DBX1;DBX2;DLX1;DLX2;DLX3;DLX4;DLX5;DLX6;EMX1;EMX2;EN1;EN2;HHEX;HLX;HMX1;HMX2;HMX3;LBX1;LBX2;MSX1;MSX2;NANOG;NKX1-1;NKX1-2;NKX2-1;NKX2-2;NKX2-3;NKX2-4;NKX2-5;NKX2-6;NKX2-8;NKX3-1;NKX3-2;NKX6-1;NKX6-2;NKX6-3;NOTO;TLX1;TLX2;TLX3;VAX1;VAX2;VENTX	NKX6-1;NKX6-2
HXA11_HUMAN.H11MO.0.D	5	0.01960784	0.10556104	0.7760554	0.3280066	6,39E+03	0.0541002459	HOX-related factors{3.1.1}	HOX9-13{3.1.1.8}	CDX1;CDX2;CDX4;EVX1;EVX2;GBX1;GBX2;GSX1;GSX2;HDX;HMBOX1;HNF1A;HNF1B;HOXA1;HOXA10;HOXA11;HOXA13;HOXA2;HOXA3;HOXA4;HOXA5;HOXA6;HOXA7;HOXA9;HOXB1;HOXB13;HOXB2;HOXB3;HOXB4;HOXB5;HOXB6;HOXB7;HOXB8;HOXB9;HOXC10;HOXC11;HOXC12;HOXC13;HOXC4;HOXC5;HOXC6;HOXC8;HOXC9;HOXD1;HOXD10;HOXD11;HOXD12;HOXD13;HOXD3;HOXD4;HOXD8;HOXD9;MEOX1;MEOX2;MNX1;PDX1;POU1F1;POU2F1;POU2F2;POU2F3;POU3F1;POU3F2;POU3F3;POU3F4;POU4F1;POU4F2;POU4F3;POU5F1;POU5F2;POU6F1;POU6F2	HOXA10;HOXA11;HOXA13;HOXA9;HOXB13;HOXC10;HOXC11;HOXC12;HOXC13;HOXC9;HOXD10;HOXD11;HOXD12;HOXD13;HOXD9
NKX31_HUMAN.H11MO.0.C	5	0.01960784	0.10424094	0.7663661	0.3238945	4,61E+03	0.0427925615	NK-related factors{3.1.2}	NK-3{3.1.2.16}	BARHL1;BARHL2;BARX1;BARX2;BSX;DBX1;DBX2;DLX1;DLX2;DLX3;DLX4;DLX5;DLX6;EMX1;EMX2;EN1;EN2;HHEX;HLX;HMX1;HMX2;HMX3;LBX1;LBX2;MSX1;MSX2;NANOG;NKX1-1;NKX1-2;NKX2-1;NKX2-2;NKX2-3;NKX2-4;NKX2-5;NKX2-6;NKX2-8;NKX3-1;NKX3-2;NKX6-1;NKX6-2;NKX6-3;NOTO;TLX1;TLX2;TLX3;VAX1;VAX2;VENTX	NKX3-1;NKX3-2
BHE22_HUMAN.H11MO.0.D	4	0.01568627	0.08652877	0.8302447	0.3200968	1,15E+04	0.0751577421	Tal-related factors{1.2.3}	Neurogenin / Atonal-like factors{1.2.3.4}	ATOH1;ATOH7;ATOH8;BHLHA15;BHLHA9;BHLHE22;BHLHE23;FERD3L;FIGLA;HAND1;HAND2;LYL1;MESP1;MESP2;MSC;MSGN1;NEUROD1;NEUROD2;NEUROD4;NEUROD6;NEUROG1;NEUROG2;NEUROG3;NHLH1;NHLH2;OLIG1;OLIG2;OLIG3;PTF1A;SCX;TAL1;TAL2;TCF15;TCF21;TCF23;TWIST1;TWIST2	ATOH1;BHLHA15;BHLHE22;BHLHE23;NEUROD1;NEUROD2;NEUROG2;OLIG1;OLIG2;OLIG3
HXB1_HUMAN.H11MO.0.D	4	0.01568627	0.08322924	0.7986403	0.3079168	8,45E+03	0.0625511107	HOX-related factors{3.1.1}	HOX1{3.1.1.1}	CDX1;CDX2;CDX4;EVX1;EVX2;GBX1;GBX2;GSX1;GSX2;HDX;HMBOX1;HNF1A;HNF1B;HOXA1;HOXA10;HOXA11;HOXA13;HOXA2;HOXA3;HOXA4;HOXA5;HOXA6;HOXA7;HOXA9;HOXB1;HOXB13;HOXB2;HOXB3;HOXB4;HOXB5;HOXB6;HOXB7;HOXB8;HOXB9;HOXC10;HOXC11;HOXC12;HOXC13;HOXC4;HOXC5;HOXC6;HOXC8;HOXC9;HOXD1;HOXD10;HOXD11;HOXD12;HOXD13;HOXD3;HOXD4;HOXD8;HOXD9;MEOX1;MEOX2;MNX1;PDX1;POU1F1;POU2F1;POU2F2;POU2F3;POU3F1;POU3F2;POU3F3;POU3F4;POU4F1;POU4F2;POU4F3;POU5F1;POU5F2;POU6F1;POU6F2	HOXA1;HOXB1
PDX1_HUMAN.H11MO.0.A	3	0.01176471	0.06243902	0.9017849	0.3048805	2,96E+04	0.1553384787	HOX-related factors{3.1.1}	PDX{3.1.1.15}	CDX1;CDX2;CDX4;EVX1;EVX2;GBX1;GBX2;GSX1;GSX2;HDX;HMBOX1;HNF1A;HNF1B;HOXA1;HOXA10;HOXA11;HOXA13;HOXA2;HOXA3;HOXA4;HOXA5;HOXA6;HOXA7;HOXA9;HOXB1;HOXB13;HOXB2;HOXB3;HOXB4;HOXB5;HOXB6;HOXB7;HOXB8;HOXB9;HOXC10;HOXC11;HOXC12;HOXC13;HOXC4;HOXC5;HOXC6;HOXC8;HOXC9;HOXD1;HOXD10;HOXD11;HOXD12;HOXD13;HOXD3;HOXD4;HOXD8;HOXD9;MEOX1;MEOX2;MNX1;PDX1;POU1F1;POU2F1;POU2F2;POU2F3;POU3F1;POU3F2;POU3F3;POU3F4;POU4F1;POU4F2;POU4F3;POU5F1;POU5F2;POU6F1;POU6F2	PDX1
CREB5_HUMAN.H11MO.0.D	6	0.02352941	0.10623978	0.6455404	0.2923124	5,21E+02	0.0057432079	Jun-related factors{1.1.1}	ATF-2-like factors{1.1.1.3}	ATF2;ATF7;BACH1;BACH2;CREB5;JUN;JUNB;JUND;NFE2;NFE2L1;NFE2L2;NFE2L3	ATF2;ATF7;CREB5
HXC10_HUMAN.H11MO.0.D	8	0.03137255	0.11427175	0.5359713	0.2677028	1,06E+01	0.0001595175	HOX-related factors{3.1.1}	HOX9-13{3.1.1.8}	CDX1;CDX2;CDX4;EVX1;EVX2;GBX1;GBX2;GSX1;GSX2;HDX;HMBOX1;HNF1A;HNF1B;HOXA1;HOXA10;HOXA11;HOXA13;HOXA2;HOXA3;HOXA4;HOXA5;HOXA6;HOXA7;HOXA9;HOXB1;HOXB13;HOXB2;HOXB3;HOXB4;HOXB5;HOXB6;HOXB7;HOXB8;HOXB9;HOXC10;HOXC11;HOXC12;H	HOXA10;HOXA11;HOXA13;HOXA9;HOXB13;HOXC10;HOXC11;HOXC12;HOXC13;HOXC9;HOXD10;HOXD11;HOXD12;HOXD13;HOXD9

Motif	Nº of probes	% of probes	lower OR	upper OR	OR	p.value	FDR	TF family	TF subfamily	TF.family.member	TF.subfamily.member
										OXC13;HOXC4;HOXC5;HOXC6;HOXC8;HOXC9;HOXD1;HOXD10;HOXD11;HOXD12;HOXD13;HOXD3;HOXD4;HOXD8;HOXD9;MEOX1;MEOX2;MNX1;PDX1;POU1F1;POU2F1;POU2F2;POU2F3;POU3F1;POU3F2;POU3F3;POU3F4;POU4F1;POU4F2;POU4F3;POU5F1;POU5F2;POU6F1;POU6F2	
ZN260_HUMAN.H11MO.0.C	4	0.01568627	0.06960024	0.6680950	0.2576113	1,76E+03	0.0178635106	More than 3 adjacent zinc finger factors{2.3.3}	ZNF146-like factors{2.3.3.55}	BCL6;BCL6B;CTCF;CTCFL;FEZF1;FEZF2;GFI1;GFI1B;GLI1;GLI2;GLI3;GLI4;GLIS1;GLIS2;GLIS3;HKR1;MTF1;MYNN;MZF1;OSR2;OVOL1;OVOL2;PLAG1;PLAGL1;PLAGL2;PRDM1;PRDM14;PRDM6;SCRT1;SCRT2;SNAI1;SNAI2;SNAI3;WT1;YY1;YY2;ZBTB12;ZBTB14;ZBTB18;ZBTB20;ZBTB26;ZBTB42;ZBTB45;ZBTB47;ZBTB48;ZBTB49;ZBTB6;ZBTB7A;ZBTB7B;ZBTB7C;ZFP14;ZFP2;ZFP28;ZFP30;ZFP37;ZFP42;ZFP64;ZFP69;ZFP69B;ZFP82;ZFP91;ZFX;ZIC1;ZIC2;ZIC3;ZIC4;ZIC5;ZIK1;ZIM3;ZKSCAN1;ZKSCAN2;ZKSCAN3;ZKSCAN4;ZNF121;ZNF124;ZNF133;ZNF136;ZNF138;ZNF14;ZNF140;ZNF143;ZNF146;ZNF148;ZNF155;ZNF157;ZNF160;ZNF169;ZNF175;ZNF177;ZNF18;ZNF180;ZNF181;ZNF2;ZNF20;ZNF212;ZNF213;ZNF214;ZNF221;ZNF222;ZNF223;ZNF224;ZNF225;ZNF226;ZNF227;ZNF229;ZNF230;ZNF232;ZNF233;ZNF234;ZNF235;ZNF24;ZNF25;ZNF250;ZNF257;ZNF26;ZNF260;ZNF263;ZNF264;ZNF268;ZNF274;ZNF276;ZNF28;ZNF280A;ZNF280B;ZNF280C;ZNF280D;ZNF281;ZNF282;ZNF283;ZNF284;ZNF285;ZNF286A;ZNF286B;ZNF3;ZNF30;ZNF300;ZNF302;ZNF317;ZNF32;ZNF320;ZNF322;ZNF324;ZNF324B;ZNF329;ZNF331;ZNF333;ZNF33A;ZNF33B;ZNF343;ZNF345;ZNF347;ZNF350;ZNF354A;ZNF354B;ZNF362;ZNF366;ZNF383;ZNF384;ZNF394;ZNF397;ZNF398;ZNF404;ZNF41;ZNF410;ZNF419;ZNF420;ZNF431;ZNF432;ZNF436;ZNF439;ZNF44;ZNF440;ZNF442;ZNF443;ZNF446;ZNF449;ZNF45;ZNF460;ZNF468;ZNF479;ZNF484;ZNF490;ZNF500;ZNF502;ZNF524;ZNF525;ZNF528;ZNF543;ZNF544;ZNF546;ZNF547;ZNF548;ZNF549;ZNF554;ZNF555;ZNF557;ZNF558;ZNF559;ZNF561;ZNF562;ZNF563;ZNF564;ZNF566;ZNF567;ZNF568;ZNF57;ZNF570;ZNF571;ZNF572;ZNF577;ZNF581;ZNF582;ZNF583;ZNF585A;ZNF586;ZNF589;ZNF595;ZNF599;ZNF600;ZNF605;ZNF607;ZNF611;ZNF613;ZNF614;ZNF615;ZNF616;ZNF619;ZNF620;ZNF621;ZNF625;ZNF627;ZNF649;ZNF652;ZNF653;ZNF665;ZNF667;ZNF669;ZNF670;ZNF672;ZNF679;ZNF680;ZNF683;ZNF689;ZNF692;ZNF701;ZNF705D;ZNF705E;ZNF705G;ZNF708;ZNF709;ZNF71;ZNF710;ZNF713;ZNF721;ZNF727;ZNF729;ZNF736;ZNF75A;ZNF75D;ZNF76;ZNF763;ZNF764;ZNF765;ZNF768;ZNF77;ZNF771;ZNF773;ZNF774;ZNF776;ZNF777;ZNF780A;ZNF780B;ZNF782;ZNF785;ZNF799;ZNF805;ZNF808;ZNF81;ZNF813;ZNF816;ZNF823;ZNF829;ZNF836;ZNF841;ZNF844;ZNF845;ZNF846;ZNF85;ZNF853;ZNF860;ZNF878;ZNF891;ZNF99;ZSCAN16;ZSCAN2;ZSCAN22;ZSCAN23;ZSCAN29;ZSCAN31;ZSCAN32;ZSCAN4;ZSCAN5A;ZSCAN5B;ZSCAN5C;ZSCAN9;ZXDA;ZXDB;ZXDC	ZNF146;ZNF260
HXA10_HUMAN.H11MO.0.C	4	0.01568627	0.06814284	0.6541013	0.2522093	1,24E+03	0.0129083449	HOX-related factors{3.1.1}	HOX9-13{3.1.1.8}	CDX1;CDX2;CDX4;EVX1;EVX2;GBX1;GBX2;GSX1;GSX2;HDX;HMBOX1;HNF1A;HNF1B;HOXA1;HOXA10;HOXA11;HOXA13;HOXA2;HOXA3;HOXA4;HOXA5;HOXA6;HOXA7;HOXA9;HOXB1;HOXB13;HOXB2;HOXB3;HOXB4;HOXB5;HOXB6;HOXB7;HOXB8;HOXB9;HOXC10;HOXC11;HOXC12;HOXC13;HOXC4;HOXC5;HOXC6;HOXC8;HOXC9;HOXD1;HOXD10;HOXD11;HOXD12;HOXD13;HOXD3;HOXD4;HOXD8;HOXD9;MEOX1;MEOX2;MNX1;PDX1;POU1F1;POU2F1;POU2F2;POU2F3;POU3F1;POU3F2;POU3F3;POU3F4;POU4F1;POU4F2;POU4F3;POU5F1;POU5F2;POU6F1;POU6F2	HOXA10;HOXA11;HOXA13;HOXA9;HOXB13;HOXC10;HOXC11;HOXC12;HOXC13;HOXC9;HOXD10;HOXD11;HOXD12;HOXD13;HOXD9
UBIP1_HUMAN.H11MO.0.D	0	0.00000000	0.00000000	Inf	0.0000000	1,00E+06	10.000.000.000	CP2-related factors{6.7.2}	UBP-1 (LBP-1, SEF){6.7.2.0.3}	TFCP2;UBP1	UBP1
SOX15_HUMAN.H11MO.0.D	0	0.00000000	0.00000000	Inf	0.0000000	1,00E+06	10.000.000.000	SOX-related factors{4.1.1}	Group G{4.1.1.7}	BBX;CIC;HBP1;SOX1;SOX10;SOX11;SOX12;SOX13;SOX14;SOX15;SOX17;SOX18;SOX2;SOX21;SOX3;SOX30;SOX4;SOX5;SOX6;SOX7;SOX8;SOX9;SRY	SOX15

Motif	Nº of probes	% of probes	lower OR	upper OR	OR	p.value	FDR	TF family	TF subfamily	TF.family.member	TF.subfamily.member
PRRX2_HUMAN.H11MO.0.C	0	0.00000000	0.00000000	Inf	0.0000000	1,00E+06	10.000.000.000	Paired-related HD factors{3.1.3}	PRRX{3.1.3.21}	ALX1;ALX3;ALX4;ARGFX;ARX;CRX;DMBX1;DPRX;DRGX;DUX4;DUXA;ESX1;GSC;GSC2;HESX1;ISX;LEUTX;MIXL1;NOBOX;OTP;OTX1;OTX2;PHOX2A;PHOX2B;PITX1;PITX2;PITX3;PROP1;PRRX1;PRRX2;RAX;RAX2;RHOXF1;RHOXF2;SEBOX;SHOX;SHOX2;TPRX1;UNCX;VSX1;VSX2	PRRX1;PRRX2
NKX61_HUMAN.H11MO.1.B	0	0.00000000	0.00000000	Inf	0.0000000	1,00E+06	10.000.000.000	NK-related factors{3.1.2}	NK-6{3.1.2.19}	BARHL1;BARHL2;BARX1;BARX2;BSX;DBX1;DBX2;DLX1;DLX2;DLX3;DLX4;DLX5;DLX6;EMX1;EMX2;EN1;EN2;HHEX;HLX;HMX1;HMX2;HMX3;LBX1;LBX2;MSX1;MSX2;NANOG;NKX1-1;NKX1-2;NKX2-1;NKX2-2;NKX2-3;NKX2-4;NKX2-5;NKX2-6;NKX2-8;NKX3-1;NKX3-2;NKX6-1;NKX6-2;NKX6-3;NOTO;TLX1;TLX2;TLX3;VAX1;VAX2;VENTX	NKX6-1;NKX6-2
NFIA_HUMAN.H11MO.1.D	0	0.00000000	0.00000000	Inf	0.0000000	1,00E+06	10.000.000.000	Nuclear factor 1{7.1.2}	NF-1A (NF-IA){7.1.2.0.1}	NFIA;NFIB;NFIC	NFIA
NF2L1_HUMAN.H11MO.0.C	0	0.00000000	0.00000000	Inf	0.0000000	1,00E+06	10.000.000.000	Jun-related factors{1.1.1}	NF-E2-like factors{1.1.1.2}	ATF2;ATF7;BACH1;BACH2;CREB5;JUN;JUNB;JUND;NFE2;NFE2L1;NFE2L2;NFE2L3	BACH1;BACH2;NFE2L1;NFE2L2;NFE2
MSX2_HUMAN.H11MO.0.D	0	0.00000000	0.00000000	Inf	0.0000000	1,00E+06	10.000.000.000	NK-related factors{3.1.2}	MSX{3.1.2.11}	BARHL1;BARHL2;BARX1;BARX2;BSX;DBX1;DBX2;DLX1;DLX2;DLX3;DLX4;DLX5;DLX6;EMX1;EMX2;EN1;EN2;HHEX;HLX;HMX1;HMX2;HMX3;LBX1;LBX2;MSX1;MSX2;NANOG;NKX1-1;NKX1-2;NKX2-1;NKX2-2;NKX2-3;NKX2-4;NKX2-5;NKX2-6;NKX2-8;NKX3-1;NKX3-2;NKX6-1;NKX6-2;NKX6-3;NOTO;TLX1;TLX2;TLX3;VAX1;VAX2;VENTX	MSX1;MSX2
HMGA1_HUMAN.H11MO.0.D	0	0.00000000	0.00000000	Inf	0.0000000	1,00E+06	10.000.000.000	HMGA factors{8.2.1}	HMGA1 (HMGI(Y)){8.2.1.0.1}	HMGA1;HMGA2	HMGA1
ETV7_HUMAN.H11MO.0.D	0	0.00000000	0.00000000	Inf	0.0000000	1,00E+06	10.000.000.000	Ets-related factors{3.5.2}	ETV6-like factors{3.5.2.6}	EHF;ELF1;ELF2;ELF3;ELF4;ELF5;ELK1;ELK3;ELK4;ERF;ERG;ETS1;ETS2;ETV1;ETV2;ETV3;ETV3L;ETV4;ETV5;ETV6;ETV7;FEV;FLI1;GABPA;SPDEF;SPI1;SPIB;SPIC	ETV6;ETV7

Table S3B - Transcription factors binding motifs and TF lists associated with DNA hypermethylation in SCCOT

Probe	GeneID	Symbol	Distance	Sides	Raw.p	Pe
cg03345454	ENSG00000155760	FZD7	0	L1	3,96E+03	3,59E+09
cg22582862	ENSG00000213988	ZNF90	25896	R2	8,31E+03	3,59E+09
cg25508181	ENSG00000251076	AC104126.1	592471	R10	1,72E+04	3,59E+09
cg22582862	ENSG00000197124	ZNF682	-12590	L2	2,67E+04	3,59E+09
cg05577810	ENSG00000066032	CTNNA2	191845	R6	3,60E+04	3,59E+09
cg04599356	ENSG00000181449	SOX2	-4418	L3	4,05E+04	0.000466334254044553
cg11254700	ENSG00000197928	ZNF677	177246	R8	4,55E+04	7,17E+09
cg24981400	ENSG00000144218	AFF3	114353	R1	6,24E+04	0.000107615597087205
cg08092966	ENSG00000104613	INTS10	665308	R9	7,10E+04	0.000251103059870144
cg02037307	ENSG00000249082	C5orf66-AS1	10957	R3	8,99E+04	0.000286974925565879
cg17920195	ENSG00000138709	LARP1B	-5296	L1	9,00E+04	0.000107615597087205
cg16362786	ENSG00000160216	AGPAT3	107376	R6	9,01E+04	3,59E+09
cg11598935	ENSG00000226308	AL122058.1	4232	R1	9,25E+04	0.000430462388348818
cg08206318	ENSG00000249082	C5orf66-AS1	10882	R3	1,14E+05	0.000322846791261614
cg04783764	ENSG00000163053	SLC16A14	54273	R1	1,23E+05	0.000215231194174409
cg15007548	ENSG00000163331	DAPL1	-230982	L8	1,39E+05	0.000394590522653083
cg23894086	ENSG00000233760	AC004947.1	-531226	L8	1,45E+05	7,17E+09
cg26271591	ENSG00000213963	AC019080.1	22278	R2	1,50E+05	3,59E+09
cg22582862	ENSG00000256229	ZNF486	115116	R9	1,75E+05	0.000143487462782939
cg26509691	ENSG00000249082	C5orf66-AS1	11003	R3	1,82E+05	0.000430462388348818
cg20701183	ENSG00000143013	LMO4	0	L1	1,83E+05	0.000394590522653083
cg15201536	ENSG00000197124	ZNF682	-12525	L2	1,88E+05	3,59E+09
cg02423318	ENSG00000229886	AC068533.2	-12593	L1	1,92E+05	0.000143487462782939
cg02037307	ENSG00000277619	AC008406.3	6647	R2	2,17E+05	7,17E+09
cg12622597	ENSG00000249082	C5orf66-AS1	11466	R3	2,39E+05	0.000430462388348818
cg22207139	ENSG00000157470	FAM81A	197154	R6	2,39E+05	7,17E+09
cg26161708	ENSG00000125851	PCSK2	-130224	L8	2,39E+04	0.000107615597087205
cg11254700	ENSG00000170954	ZNF415	49744	R2	2,68E+02	7,17E+09
cg08062273	ENSG00000163820	FYCO1	318269	R10	2,69E+04	3,59E+09
cg08206318	ENSG00000277619	AC008406.3	6572	R2	2,71E+04	0.000107615597087205
cg12473285	ENSG00000161649	CD300LG	91539	R5	2,95E+04	0.000143487462782939
cg04570362	ENSG00000162444	RBP7	185860	R10	3,38E+04	0.000107615597087205
cg19416570	ENSG00000176293	ZNF135	-117999	L6	3,63E+05	0.000502206119740288
cg08933939	ENSG00000112238	PRDM13	0	L1	3,75E+05	3,59E+09
cg05935800	ENSG00000143819	EPHX1	0	L1	3,94E+05	0.000932668508089106
cg07019303	ENSG00000111261	MANSC1	619228	R8	3,94E+05	0.000789181045306166
cg02037307	ENSG00000224186	C5orf66	5406	R1	4,58E+05	0.000215231194174409
cg08263071	ENSG00000135333	EPHA7	0	L1	5,94E+05	0.000107615597087205
cg11171221	ENSG00000121898	CPXM2	562877	R9	6,68E+05	0.000789181045306166
cg26509691	ENSG00000277619	AC008406.3	6693	R2	7,05E+05	0.000358718656957348
cg22369786	ENSG00000136158	SPRY2	0	L1	7,21E+05	3,59E+09
cg13474848	ENSG00000226197	AL583785.1	-863280	L8	8,41E+05	0.000860924776697636
cg16260349	ENSG00000133665	DYDC2	-93767	L3	9,13E+05	0.000251103059870144
cg16163847	ENSG00000231856	AL162377.1	212616	R4	9,31E+05	3,59E+09
cg08206318	ENSG00000224186	C5orf66	5331	R1	9,34E+05	0.000394590522653083
cg25556035	ENSG00000008441	NFIX	0	L1	9,71E+05	3,59E+09
cg03535099	ENSG00000167363	FN3K	-138197	L4	1,04E+06	0.000358718656957348
cg26271591	ENSG00000116044	NFE2L2	0	L1	1,05E+06	3,59E+09
cg16638385	ENSG00000161649	CD300LG	91761	R5	1,06E+05	0.000179359328478674
cg08153621	ENSG00000170954	ZNF415	49689	R2	1,08E+06	7,17E+09
**Probe**	**GeneID**	**Symbol**	**Distance**	**Sides**	**Raw.p**	**Pe**
cg03431524	ENSG00000106351	AGFG2	0	L1	1,17E+06	0.000107615597087205
cg16629695	ENSG00000130287	NCAN	-129033	L8	1,33E+06	0.000179359328478674
cg11254700	ENSG00000197497	ZNF665	101078	R4	1,44E+06	0.000143487462782939
cg15201536	ENSG00000256229	ZNF486	115181	R9	1,45E+06	0.000681565448218962
cg23394510	ENSG00000139438	FAM222A	-107280	L7	1,51E+06	0.000286974925565879
cg00691123	ENSG00000132170	PPARG	695866	R15	1,56E+06	0.000538077985436022
cg08823240	ENSG00000006062	MAP3K14	0	L1	1,63E+06	7,17E+09
cg15374435	ENSG00000136158	SPRY2	0	L1	1,63E+06	3,59E+09
cg03345454	ENSG00000273209	AC069148.1	-3167	L2	1,66E+06	0.000322846791261614
cg02037307	ENSG00000069011	PITX1	0	L1	1,75E+06	0.000143487462782939
cg01012280	ENSG00000167562	ZNF701	-13970	L4	1,81E+06	3,59E+09
cg24576358	ENSG00000132570	PCBD2	-6472	L2	1,88E+06	0.000215231194174409
cg12622597	ENSG00000277619	AC008406.3	7156	R2	1,91E+06	0.000466334254044553
cg01012280	ENSG00000182986	ZNF320	262643	R8	2,02E+06	3,59E+09
cg15534561	ENSG00000081818	PCDHB4	80864	R7	2,16E+06	0.000286974925565879
cg10157208	ENSG00000008441	NFIX	0	L1	2,17E+06	7,17E+09
cg04223420	ENSG00000277619	AC008406.3	6332	R2	2,36E+06	0.000466334254044553
cg11254700	ENSG00000242779	ZNF702P	-20234	L2	2,50E+06	0.000179359328478674
cg16471612	ENSG00000066583	ISOC1	1135746	R7	2,51E+06	0.000860924776697636
cg05903330	ENSG00000100307	CBX7	0	L1	2,51E+06	0.000860924776697636
cg25018049	ENSG00000159176	CSRP1	0	L1	2,51E+06	0.000143487462782939
cg02074274	ENSG00000160216	AGPAT3	106771	R6	2,70E+06	0.000179359328478674
cg07134316	ENSG00000166866	MYO1A	-43130	L3	2,87E+06	0.000143487462782939
cg26509691	ENSG00000224186	C5orf66	5452	R1	3,11E+06	0.000573949851131757
cg25959472	ENSG00000156427	FGF18	-499784	L4	3,14E+06	0.000789181045306166
cg13122377	ENSG00000268379	AC025588.1	2912	L1	3,23E+06	3,59E+09
cg08969304	ENSG00000186766	FOXI2	-800148	L9	3,36E+06	0.000394590522653083
cg04319659	ENSG00000185345	PRKN	580110	R9	3,58E+06	0.000609821716827492
cg08805241	ENSG00000121057	AKAP1	51547	R2	3,60E+06	0.000143487462782939
cg01461840	ENSG00000186766	FOXI2	-545637	L8	3,70E+05	0.000394590522653083
cg01012280	ENSG00000198482	ZNF808	-36680	L6	4,00E+06	7,17E+09
cg06552182	ENSG00000167562	ZNF701	-14569	L4	4,77E+06	7,17E+09
cg01012280	ENSG00000123870	ZNF137P	-3799	L3	4,86E+06	3,59E+09
cg15201536	ENSG00000213988	ZNF90	25961	R2	4,89E+06	0.000179359328478674
cg25973895	ENSG00000197124	ZNF682	-12712	L2	4,91E+06	0.000286974925565879
cg06552182	ENSG00000258405	ZNF578	-84861	L8	5,50E+06	3,59E+09
cg21651328	ENSG00000268555	AC123912.4	95866	R5	5,68E+06	0.000753309179610432
cg01527159	ENSG00000268081	AC123912.1	79814	R4	5,74E+06	3,59E+09
cg08955941	ENSG00000081818	PCDHB4	80880	R7	5,87E+05	0.000753309179610432
cg03781505	ENSG00000141040	ZNF287	139907	R9	5,89E+05	0.000215231194174409
cg01577475	ENSG00000225398	PGM5P4	265931	R9	6,03E+06	0.000825052911001901
cg20984972	ENSG00000141905	NFIC	0	L1	6,33E+06	0.000681565448218962
cg11676902	ENSG00000112238	PRDM13	0	L1	6,45E+06	3,59E+09
cg00558749	ENSG00000178821	TMEM52	135077	R4	7,01E+06	7,17E+09

cg13366501	ENSG00000259673	IQCH-AS1	-393968	L9	7,26E+06	0.000358718656957348
cg26271591	ENSG00000222043	AC079305.1	3129	R1	7,28E+06	3,59E+09
cg07187971	ENSG00000081913	PHLPP1	0	L1	7,28E+06	0.000789181045306166
cg10993086	ENSG00000143315	PIGM	75391	R5	7,80E+06	7,17E+09
cg00636508	ENSG00000157353	FUK	-110252	L6	8,07E+06	7,17E+09
cg00753287	ENSG00000224023	EDRF1-DT	639656	R7	8,12E+06	0.000358718656957348
cg22758493	ENSG00000226266	AC009961.1	-870594	L9	8,23E+06	0.000609821716827492

Probe	GeneID	Symbol	Distance	Sides	Raw.p	Pe
cg02576468	ENSG00000160460	SPTBN4	0	L1	8,30E+06	0.000394590522653083
cg26546884	ENSG00000112699	GMDS	0	L1	8,36E+06	0.000538077985436022
cg11654900	ENSG00000123080	CDKN2C	-4378	L1	8,37E+06	3,59E+09
cg06946708	ENSG00000065154	OAT	424444	R3	8,97E+06	0.000143487462782939
cg25973895	ENSG00000213988	ZNF90	25774	R2	9,17E+06	0.000286974925565879
cg13077545	ENSG00000161649	CD300LG	91641	R5	9,92E+06	0.000681565448218962
cg17129821	ENSG00000119147	C2orf40	693317	R5	1,11E+07	0.000358718656957348
cg22207139	ENSG00000140297	GCNT3	419336	R13	1,14E+07	0.000466334254044553
cg08376828	ENSG00000186766	FOXI2	-800076	L9	1,15E+07	0.000896796642393371
cg13677144	ENSG00000268379	AC025588.1	2714	L1	1,19E+07	3,59E+09
cg09489306	ENSG00000267058	AC006213.3	192041	R8	1,37E+07	7,17E+09
cg13056369	ENSG00000253764	AC019257.1	13033	R1	1,38E+07	0.000179359328478674
cg16206520	ENSG00000176714	CCDC121	-150471	L8	1,40E+07	0.000609821716827492
cg08206318	ENSG00000069011	PITX1	0	L1	1,45E+07	0.000645693582523227
cg07413467	ENSG00000188175	HEPACAM2	579812	R6	1,74E+07	7,17E+09
cg15261712	ENSG00000127980	PEX1	-80413	L5	1,78E+07	0.000179359328478674
cg25439496	ENSG00000112837	TBX18	-10310	L1	1,84E+07	7,17E+09
cg06552182	ENSG00000198482	ZNF808	-37279	L6	1,84E+07	0.000143487462782939
cg04908380	ENSG00000162368	CMPK1	-71350	L5	1,90E+07	7,17E+09
cg03998264	ENSG00000270175	AC023509.3	-37634	L5	1,95E+07	0.000502206119740288
cg26646427	ENSG00000075240	GRAMD4	201037	R3	2,03E+07	3,59E+09
cg01841306	ENSG00000184811	TRARG1	273977	R7	2,05E+06	0.000107615597087205
cg06552182	ENSG00000123870	ZNF137P	-4398	L3	2,26E+07	0.000107615597087205
cg22758104	ENSG00000262558	AC129507.3	154061	R5	2,48E+07	3,59E+09
cg27553162	ENSG00000149599	DUSP15	-124429	L6	2,60E+06	0.000466334254044553
cg04646674	ENSG00000008441	NFIX	0	L1	2,66E+07	0.000681565448218962
cg26216323	ENSG00000156675	RAB11FIP1	0	L1	2,66E+07	0.000753309179610432
cg15534561	ENSG00000255408	PCDHA3	-28585	L2	2,67E+07	0.000394590522653083
cg12081325	ENSG00000117425	PTCH2	-104353	L4	2,82E+07	0.000681565448218962
cg22582862	ENSG00000184635	ZNF93	-88631	L5	2,84E+07	0.000143487462782939
cg03998264	ENSG00000135390	ATP5MC2	94153	R3	2,84E+07	0.000538077985436022
cg12363903	ENSG00000069011	PITX1	-155765	L6	3,04E+07	0.000968540373784841
cg19788754	ENSG00000141556	TBCD	0	L1	3,04E+07	0.000215231194174409
cg15201536	ENSG00000184635	ZNF93	-88566	L5	3,35E+07	0.000179359328478674
cg11254700	ENSG00000269001	AC092070.2	138976	R6	3,38E+07	0.000645693582523227
cg26727435	ENSG00000198355	PIM3	-50935	L3	3,47E+07	3,59E+09
cg07413467	ENSG00000127980	PEX1	-80251	L5	3,83E+06	0.000358718656957348
cg12914733	ENSG00000117983	MUC5B	267736	R5	3,91E+07	0.000286974925565879
cg16309595	ENSG00000017427	IGF1	-483922	L5	4,35E+07	0.000538077985436022
cg08318283	ENSG00000138622	HCN4	996	L1	4,60E+07	7,17E+09
cg01316378	ENSG00000183346	CABCOCO1	-103054	L3	4,81E+07	0.000466334254044553
cg11669516	ENSG00000115457	IGFBP2	0	L1	4,82E+07	0.000394590522653083
cg03711485	ENSG00000228778	AL513542.1	-7811	L3	4,89E+07	0.000430462388348818
cg25336900	ENSG00000188338	SLC38A3	39846	R3	4,95E+07	0.000860924776697636

cg16988611	ENSG00000151224	MAT1A	-175531	L7	4,97E+07	0.000860924776697636
cg15649702	ENSG00000135373	EHF	465544	R9	5,14E+07	0.000645693582523227
cg09887059	ENSG00000186815	TPCN1	-180273	L7	5,31E+07	0.000645693582523227
cg19460095	ENSG00000135373	EHF	0	L1	5,49E+07	0.000645693582523227
cg10156366	ENSG00000066032	CTNNA2	191974	R6	5,56E+07	0.000753309179610432
cg23983887	ENSG00000272482	AC254633.1	169247	R6	5,56E+07	0.000430462388348818
cg06552182	ENSG00000268970	AC022150.2	-2856	L2	5,58E+07	0.000179359328478674

Probe	GeneID	Symbol	Distance	Sides	Raw.p	Pe
cg16260349	ENSG00000151224	MAT1A	-172182	L7	6,59E+07	0.000932668508089106
cg21156912	ENSG00000141556	TBCD	0	L1	6,66E+06	0.000322846791261614
cg08669447	ENSG00000267058	AC006213.3	192205	R8	6,83E+07	0.000215231194174409
cg18347010	ENSG00000136451	VEZF1	160374	R5	7,11E+07	0.000502206119740288
cg13337047	ENSG00000268379	AC025588.1	2756	L1	7,56E+07	3,59E+09
cg03535099	ENSG00000141556	TBCD	0	L1	7,58E+07	0.000358718656957348
cg17329534	ENSG00000160685	ZBTB7B	0	L1	7,58E+07	0.000107615597087205
cg08769507	ENSG00000188573	FBLL1	363450	R5	7,68E+07	0.000107615597087205
cg07019303	ENSG00000070018	LRP6	409197	R3	7,82E+07	0.000860924776697636
cg19396867	ENSG00000275395	FCGBP	39099	R3	8,08E+07	0.000322846791261614
cg26271591	ENSG00000271996	AC019080.4	45143	R4	8,23E+07	0.000143487462782939
cg11809014	ENSG00000212900	KRTAP3-2	88362	R5	8,36E+07	0.000251103059870144
cg26469608	ENSG00000186815	TPCN1	-177304	L7	8,61E+07	0.000860924776697636
cg14290616	ENSG00000100346	CACNA1I	-335245	L8	9,11E+07	0.000394590522653083
cg24913868	ENSG00000228778	AL513542.1	-7844	L3	9,70E+07	0.000825052911001901
cg06552182	ENSG00000182986	ZNF320	262044	R8	9,78E+07	3,59E+09
cg06936564	ENSG00000275395	FCGBP	39034	R3	1,18E+08	0.000430462388348818
cg01012280	ENSG00000268970	AC022150.2	-2257	L2	1,19E+08	0.000286974925565879
cg22686132	ENSG00000232040	ZBED9	-141967	L4	1,21E+07	3,59E+09
cg19416570	ENSG00000243642	RN7SL526P	-134021	L7	1,22E+08	0.000717437313914697
cg00811065	ENSG00000230487	PSMG3-AS1	322548	R10	1,26E+08	0.000717437313914697
cg04987053	ENSG00000103199	ZNF500	-220452	L10	1,43E+08	0.000215231194174409
cg18845189	ENSG00000268379	AC025588.1	2887	L1	1,45E+08	7,17E+09
cg00744431	ENSG00000171811	CFAP46	395347	R8	1,64E+08	0.000717437313914697
cg25284397	ENSG00000188175	HEPACAM2	579742	R6	1,84E+08	0.000538077985436022
cg03535099	ENSG00000141542	RAB40B	-190695	L9	2,07E+08	0.000430462388348818
cg20655070	ENSG00000275395	FCGBP	38950	R3	2,07E+08	0.000717437313914697
cg10277651	ENSG00000125878	TCF15	-32318	L2	2,12E+08	0.000789181045306166
cg07791011	ENSG00000163618	CADPS	20097	R1	2,18E+08	0.000573949851131757
cg13315923	ENSG00000154310	TNIK	-743496	L10	2,20E+08	0.000825052911001901
cg12405048	ENSG00000111275	ALDH2	130603	R4	2,20E+08	0.000502206119740288
cg05903330	ENSG00000280216	AL022326.2	233477	R9	2,23E+08	0.000753309179610432
cg11758945	ENSG00000181790	ADGRB1	0	L1	2,33E+08	0.000896796642393371
cg06528584	ENSG00000275395	FCGBP	39062	R3	2,34E+08	0.000753309179610432
cg13005999	ENSG00000258986	TMEM179	12958	R1	2,52E+07	0.000860924776697636
cg06688763	ENSG00000188175	HEPACAM2	579691	R6	2,53E+08	0.000609821716827492
cg05230392	ENSG00000180891	CUEDC1	0	L1	2,65E+08	0.000394590522653083
cg12126859	ENSG00000198010	DLGAP2	352313	R7	2,67E+08	0.000609821716827492
cg07637741	ENSG00000164674	SYTL3	0	L1	3,18E+08	0.000717437313914697
cg26271591	ENSG00000271825	AC019080.3	39370	R3	3,40E+08	0.000394590522653083
cg03529261	ENSG00000180712	LINC02363	52266	R2	3,64E+08	0.000430462388348818
cg15261712	ENSG00000188175	HEPACAM2	579650	R6	3,82E+08	0.000609821716827492
cg02162886	ENSG00000258952	SALRNA1	-4837	L2	4,26E+08	0.000789181045306166
cg16163847	ENSG00000102796	DHRS12	176771	R3	4,29E+08	0.000251103059870144

cg21069176	ENSG00000174738	NR1D2	-325547	L4	4,56E+08	0.000430462388348818
cg08472795	ENSG00000214100	PLAC9P1	1613182	R9	4,59E+08	0.000358718656957348
cg11500797	ENSG00000006128	TAC1	709271	R6	4,68E+08	0.000896796642393371
cg06111140	ENSG00000106018	VIPR2	245108	R3	4,81E+08	0.000358718656957348
cg21069176	ENSG00000237838	AC092422.1	381752	R7	4,84E+08	0.000753309179610432
cg23251200	ENSG00000154478	GPR26	-381089	L7	5,06E+08	0.000430462388348818
cg14503441	ENSG00000207563	MIR23B	89092	R2	5,56E+08	0.000358718656957348

**Probe**	**GeneID**	**Symbol**	**Distance**	**Sides**	**Raw.p**	**Pe**
cg04157263	ENSG00000141750	STAC2	-370792	L9	6,25E+08	0.000322846791261614
cg04282694	ENSG00000124785	NRN1	1818	L1	6,30E+08	0.000107615597087205
cg06001519	ENSG00000262619	LINC00621	-10329	L3	6,61E+08	0.000358718656957348
cg03529261	ENSG00000270426	AC099343.2	183434	R6	7,14E+08	0.000573949851131757
cg02216731	ENSG00000261659	Z92544.2	-59381	L10	7,27E+08	0.000322846791261614
cg05230392	ENSG00000264364	DYNLL2	184256	R10	7,74E+08	0.000107615597087205
cg16387532	ENSG00000155324	GRAMD2B	948001	R6	7,96E+08	0.000358718656957348
cg04173586	ENSG00000099875	MKNK2	-116252	L5	8,20E+08	0.000789181045306166
cg26546884	ENSG00000272279	AL512329.2	-238858	L4	8,71E+08	0.000717437313914697
cg02849693	ENSG00000142408	CACNG8	63837	R2	9,18E+08	0.000322846791261614
cg10451262	ENSG00000165156	ZHX1	0	L1	9,20E+08	0.000179359328478674
cg03564793	ENSG00000118407	FILIP1	206692	R4	1,03E+09	0.000502206119740288
cg24839529	ENSG00000151726	ACSL1	8354	L1	1,09E+09	0.000968540373784841
cg23422268	ENSG00000075891	PAX2	78643	R1	1,12E+09	0.000753309179610432
cg26216323	ENSG00000104221	BRF2	-14770	L3	1,23E+09	0.000860924776697636
cg00636508	ENSG00000189091	SF3B3	-12856	L2	1,23E+09	0.000896796642393371
cg11342645	ENSG00000174697	LEP	-82057	L4	1,36E+09	0.000717437313914697
cg12082089	ENSG00000147457	CHMP7	262068	R13	1,41E+09	0.000825052911001901
cg17345994	ENSG00000198561	CTNND1	0	L1	1,45E+09	0.000717437313914697
cg19343518	ENSG00000271551	AL355297.4	-409122	L6	1,52E+09	0.000358718656957348
cg06587521	ENSG00000257541	LINC02403	-800642	L8	1,67E+09	0.000107615597087205
cg21277243	ENSG00000124785	NRN1	1899	L1	1,72E+09	0.000286974925565879
cg26156256	ENSG00000235933	LINC01779	336012	R7	1,72E+09	0.000573949851131757
cg00991875	ENSG00000231863	AL139393.1	251734	R8	1,79E+09	0.000107615597087205
cg00849713	ENSG00000112539	C6orf118	-544650	L6	1,91E+09	0.000896796642393371
cg26551092	ENSG00000271551	AL355297.4	-405875	L6	1,92E+09	0.000466334254044553
cg13258774	ENSG00000244310	AC093904.3	-198977	L4	1,98E+09	0.000896796642393371
cg15534561	ENSG00000250120	PCDHA10	-28585	L4	2,03E+09	0.000717437313914697
cg02029908	ENSG00000120129	DUSP1	0	L1	2,15E+09	0.000968540373784841
cg16629695	ENSG00000129933	MAU2	-22512	L2	2,15E+09	0.000107615597087205
cg22758104	ENSG00000262920	AC129507.4	120716	R2	2,33E+09	0.000609821716827492
cg26426898	ENSG00000101282	RSPO4	230000	R4	2,40E+09	0.000573949851131757
cg22191696	ENSG00000141540	TTYH2	260859	R4	2,53E+09	0.000179359328478674
cg00862376	ENSG00000170653	ATF7	-323516	L9	2,60E+08	0.000789181045306166
cg09489306	ENSG00000176222	ZNF404	172600	R6	2,66E+08	0.000430462388348818
cg12118843	ENSG00000232040	ZBED9	-57927	L3	2,80E+09	0.000645693582523227
cg00580340	ENSG00000225028	AC096541.1	385497	R8	2,94E+09	0.000860924776697636
cg24312063	ENSG00000269345	VN1R85P	-86515	L5	3,07E+09	0.000896796642393371
cg06776173	ENSG00000159658	EFCAB14	208049	R10	3,15E+09	0.000215231194174409
cg22762492	ENSG00000170549	IRX1	4773	L1	3,49E+09	0.000286974925565879
cg02483029	ENSG00000221869	CEBPD	352200	R6	4,01E+09	0.000717437313914697
cg06676778	ENSG00000272411	AC116312.1	140560	R2	4,23E+09	7,17E+09
cg01937780	ENSG00000157107	FCHO2	-210589	L6	4,35E+09	0.000717437313914697
cg10512292	ENSG00000118971	CCND2	4659	R1	4,35E+09	0.000215231194174409

cg25585712	ENSG00000109132	PHOX2B	-118225	L6	4,35E+09	0.000466334254044553
cg07018389	ENSG00000213386	AC022217.1	-5416	L2	4,44E+09	0.000107615597087205
cg12929487	ENSG00000109132	PHOX2B	-118187	L6	4,61E+09	0.000466334254044553
cg25439496	ENSG00000228290	TBX18-AS1	-65330	L2	4,96E+09	0.000179359328478674
cg13507084	ENSG00000164967	RPP25L	55440	R2	5,67E+09	3,59E+09
cg14742809	ENSG00000251279	SMIM15-AS1	-103160	L3	5,71E+09	0.000825052911001901
cg06884352	ENSG00000272384	AC016405.3	983286	R9	6,00E+09	0.000753309179610432

**Probe**	**GeneID**	**Symbol**	**Distance**	**Sides**	**Raw.p**	**Pe**
cg07019303	ENSG00000121335	PRB2	-205784	L8	6,03E+09	0.000466334254044553
cg03690956	ENSG00000277879	AL391988.1	126301	R7	6,09E+09	0.000825052911001901
cg10157208	ENSG00000267417	AC138474.1	12025	R1	6,45E+09	0.000681565448218962
cg20023898	ENSG00000265408	AC009084.1	-51309	L4	7,02E+08	0.000215231194174409
cg09970120	ENSG00000260426	AC008060.4	24771	R1	7,21E+09	0.000538077985436022
cg22191696	ENSG00000246731	AC100786.1	257268	R3	7,27E+09	0.000107615597087205
cg03714522	ENSG00000164900	GBX1	22772	R1	7,28E+08	0.000286974925565879
cg14024242	ENSG00000281769	LINC01230	2754	L1	7,90E+09	0.000286974925565879
cg04982922	ENSG00000271755	AL031118.1	143457	R6	8,04E+09	0.000466334254044553
cg20372666	ENSG00000156515	HK1	0	L1	8,58E+09	0.000538077985436022
cg08472795	ENSG00000229536	LINC02572	1515445	R8	9,36E+09	0.000968540373784841
cg20157281	ENSG00000230495	AL132765.1	283448	R7	0.000104884762171704	7,17E+09
cg15138125	ENSG00000229914	AL138899.2	216528	R5	0.000108412821813999	0.000502206119740288
cg00991875	ENSG00000233342	AL391361.3	247023	R7	0.000111796738218858	0.000860924776697636
cg13591204	ENSG00000165275	TRMT10B	-285212	L9	0.000116262907203187	0.000538077985436022
cg27448110	ENSG00000225365	AC078942.1	845538	R6	0.000119229971576302	0.000358718656957348
cg06338710	ENSG00000189195	BTBD8	-295904	L9	0.000122220836481462	0.000896796642393371
cg25874119	ENSG00000154358	OBSCN	-67328	L8	0.000135186130326223	0.000286974925565879
cg13591204	ENSG00000107338	SHB	0	L1	0.000138573624070567	7,17E+09
cg07522913	ENSG00000253552	HOXA-AS2	0	R1	0.000141853111941352	0.000645693582523227
cg22538959	ENSG00000148826	NKX6-2	-2511	L1	0.000157234674661372	0.000358718656957348
cg14189141	ENSG00000281769	LINC01230	3018	L1	0.000160900626807808	0.000322846791261614
cg11186405	ENSG00000229520	LINC00404	32201	R2	0.000161007866099575	0.000179359328478674
cg03345454	ENSG00000222035	KIAA2012-AS1	76919	R2	0.000165946928618071	0.000789181045306166
cg21609706	ENSG00000236676	AC117944.1	427142	R8	0.000167420939764025	7,17E+09
cg08743751	ENSG00000249364	AC112206.2	212733	R3	0.000174335117472589	0.000645693582523227
cg24002887	ENSG00000234338	AC073349.2	-110482	L7	0.000186985014795876	0.000466334254044553
cg26727435	ENSG00000272836	AL022328.1	190575	R9	0.000210269074198529	0.000538077985436022
cg22582862	ENSG00000268070	AC006539.2	-17763	L3	0.000301731359115497	0.000789181045306166
cg07019303	ENSG00000255790	AC078950.1	-220147	L10	0.00035613978880898	0.000107615597087205
cg13175739	ENSG00000257016	SLC25A39P2	-85079	L5	0.000357557618409309	3,59E+09
cg12405048	ENSG00000257595	LINC02356	-232971	L7	0.000384570753618009	0.000825052911001901
cg00980592	ENSG00000199627	RNU6-1010P	157613	R5	0.000395300026046387	0.000932668508089106
cg04178787	ENSG00000230993	RPL12P15	-1002237	L9	0.000419782164538477	0.000430462388348818
cg11437784	ENSG00000229520	LINC00404	49584	R3	0.000527905598832765	0.000753309179610432
cg26509691	ENSG00000249647	C5orf66-AS2	208406	R6	0.000548925007972197	0.000573949851131757
cg07453440	ENSG00000267079	AP001269.2	-33810	L2	0.000551331821398804	0.000358718656957348
cg03345454	ENSG00000182329	KIAA2012	36582	R1	0.000580928404797321	0.000860924776697636
cg19014435	ENSG00000256906	LINC02419	-32021	L2	0.000607517003571918	0.000968540373784841
cg08206318	ENSG00000249647	C5orf66-AS2	208285	R6	0.000640166058976897	0.000609821716827492
cg15142819	ENSG00000236324	AL035634.1	-110355	L6	0.000704429822256429	3,59E+09
cg06543221	ENSG00000260026	LINC02189	-144052	L3	0.000724159554009953	0.000753309179610432
cg03012170	ENSG00000266743	AC103808.6	256891	R3	0.000729559162728327	0.000609821716827492

cg10746936	ENSG00000258805	ADIPOR1P2	-139281	L5	0.000844830168986729	0.000215231194174409
cg06494592	ENSG00000242229	RPS3AP14	-194397	L10	0.000962601929659732	0.000681565448218962
cg20248093	ENSG00000275516	AC100791.3	203964	R10	0.0010082263067875	0.000681565448218962
cg02029908	ENSG00000253295	AC022217.2	-6042	L3	0.00101032282283124	0.000860924776697636
cg02304751	ENSG00000250293	CRYZP2	-745392	L10	0.00144038617722156	0.000251103059870144
cg06946708	ENSG00000231138	AC009987.1	-195107	L4	0.00145642715096599	0.000251103059870144
cg07839742	ENSG00000230852	AL161621.1	-26140	L2	0.00157583533405814	0.000753309179610432
**Probe**	**GeneID**	**Symbol**	**Distance**	**Sides**	**Raw.p**	**Pe**
cg00153106	ENSG00000254200	RPL7AP33	-18134	L2	0.00160396021839113	0.000215231194174409
cg25396728	ENSG00000256263	DDX11L8	-125832	L5	0.00185230565778747	0.000717437313914697
cg11205552	ENSG00000239453	SIDT1-AS1	311903	R7	0.00192927359981009	0.000609821716827492
cg13974773	ENSG00000237154	MCFD2P1	-87441	L3	0.00195182513568028	0.000860924776697636
cg11186405	ENSG00000224243	SOX1-OT	33225	R3	0.00207323657759764	0.000860924776697636
cg23217622	ENSG00000260460	AL365181.1	74206	R4	0.00208101189733867	0.000753309179610432
cg15690696	ENSG00000250493	AP004147.1	520922	R4	0.0023302734640985	0.000502206119740288
cg13507084	ENSG00000137100	DCTN3	58499	R3	0.0024881958933943	0.000573949851131757
cg02552189	ENSG00000278083	AC013476.2	-80177	L2	0.00286473619995781	0.000645693582523227
cg13687996	ENSG00000236106	AC010729.1	53096	R4	0.00286473619995781	0.000502206119740288
cg20053465	ENSG00000224190	LINC02667	371214	R1	0.00287807644167594	0.000466334254044553
cg15457934	ENSG00000242894	RN7SL634P	-172564	L6	0.00288609686280858	0.000322846791261614
cg00929523	ENSG00000236957	AL139010.1	-30013	L2	0.00385070225088299	0.000753309179610432
cg14314029	ENSG00000225795	AC006463.1	359989	R2	0.00563894131693046	0.000143487462782939
cg00469207	ENSG00000231139	AC104843.2	-334428	L8	0.00608315139651585	0.000394590522653083
cg16263224	ENSG00000258931	AC100836.1	-30471	L2	0.0108886890915923	3,59E+09
cg09164580	ENSG00000272249	AP003117.2	987183	R10	0.0109214228431017	0.000358718656957348
cg15311814	ENSG00000200446	RNU6-1085P	673554	R9	0.0109214228431017	0.000430462388348818
cg17820365	ENSG00000272249	AP003117.2	987205	R10	0.0109214228431017	0.000358718656957348
cg16589299	ENSG00000232985	AL355537.1	-1000459	L9	0.0109378034886543	0.000251103059870144
cg02091109	ENSG00000251605	AC104108.1	662877	R7	0.0109509146054654	0.000215231194174409
cg01926877	ENSG00000259403	AC020704.1	862354	R12	0.0210992520232155	0.000286974925565879
cg04987053	ENSG00000261811	LINC02164	227315	R10	0.0408295922183942	0.000215231194174409
cg06033721	ENSG00000228504	LINC01760	437250	R12	0.0408295922183942	3,59E+09
cg13683550	ENSG00000207158	RNU6-929P	-40774	L1	0.0408295922183942	0.000609821716827492
cg23329272	ENSG00000248717	LINC02222	25840	R2	0.0408295922183942	3,59E+09
cg23819016	ENSG00000271026	KATNBL1P1	244529	R9	0.0408295922183942	0.000286974925565879
